# Supplementary figures and images for: Non-cell-autonomous control of mouse gastruloid development by the ultra-conserved lncRNA T-UCstem1 (part 1 of 6)
Source: EMBO J. 2025 Oct 31;44(24):7620–48. doi: 10.1038/s44318-025-00558-2 (PMC12706062; doi:10.1038/s44318-025-00558-2)

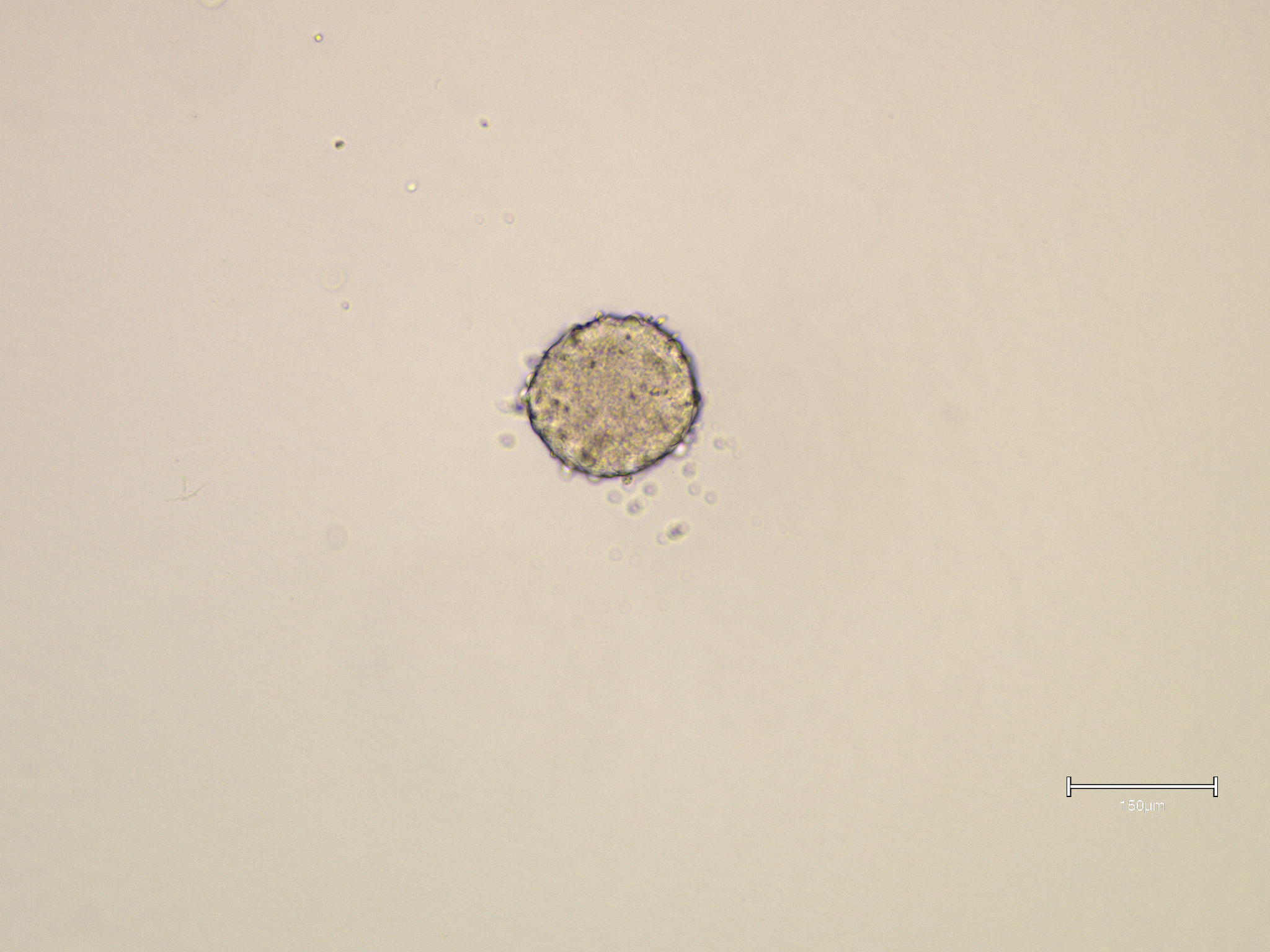

Supplement: Supplementary file 3 — Source data Fig. 1 [file 44318_2025_558_MOESM3_ESM.zip › Figure 1/panel 1B/KD-1_48h.tiff]

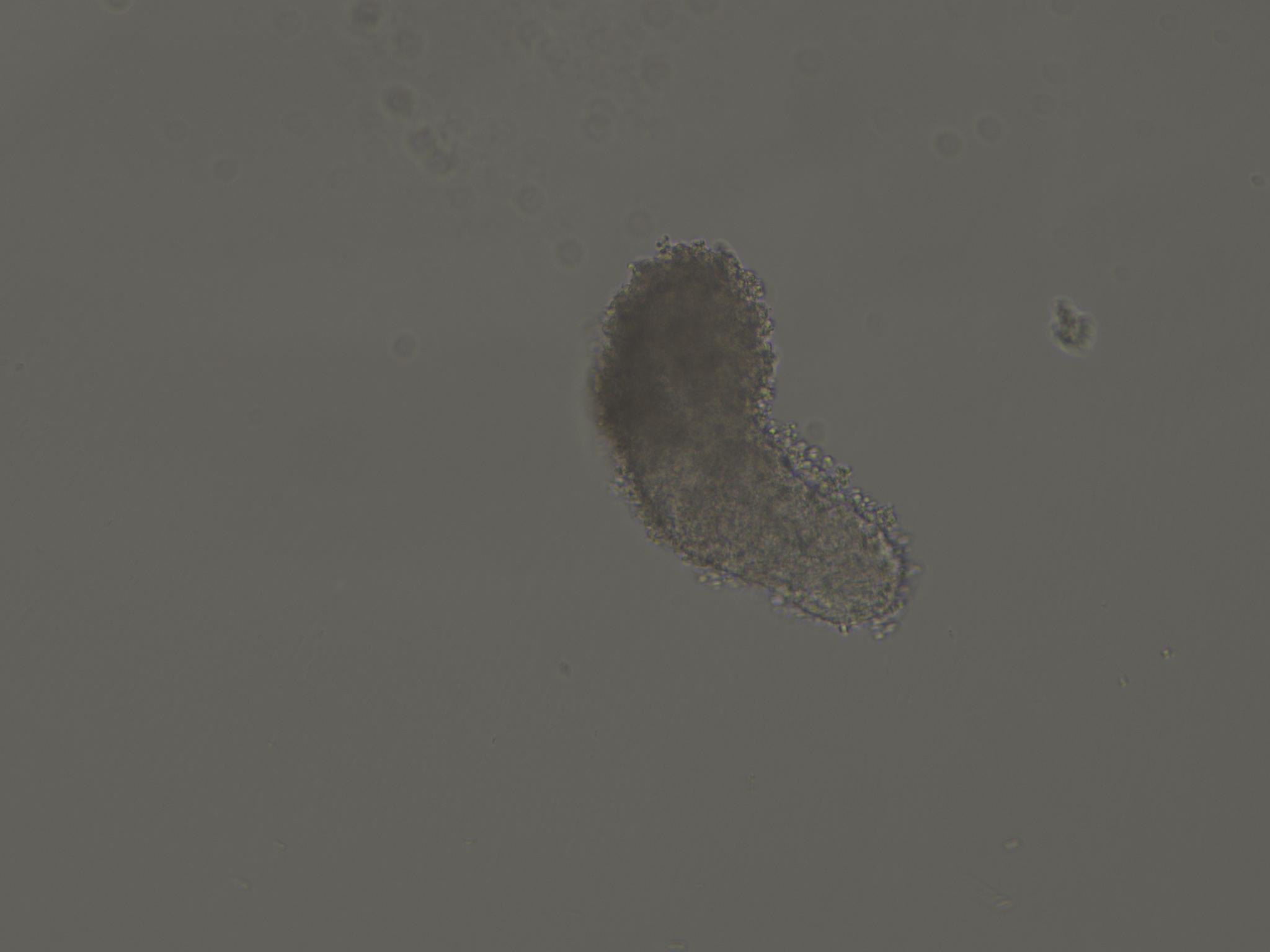

Supplement: Supplementary file 3 — Source data Fig. 1 [file 44318_2025_558_MOESM3_ESM.zip › Figure 1/panel 1B/NT_120h.tiff]

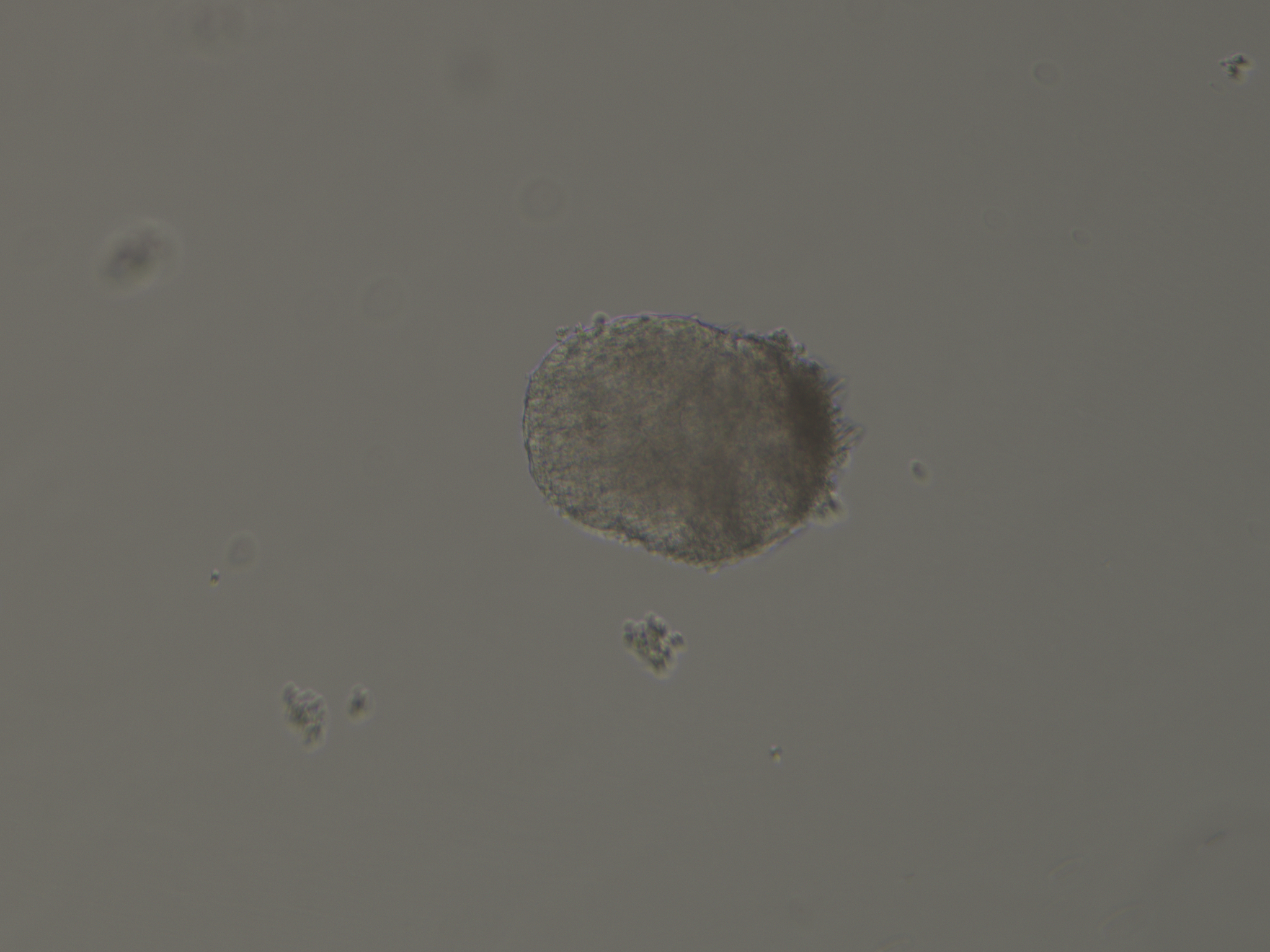

Supplement: Supplementary file 3 — Source data Fig. 1 [file 44318_2025_558_MOESM3_ESM.zip › Figure 1/panel 1B/KD-1_120h.tiff]

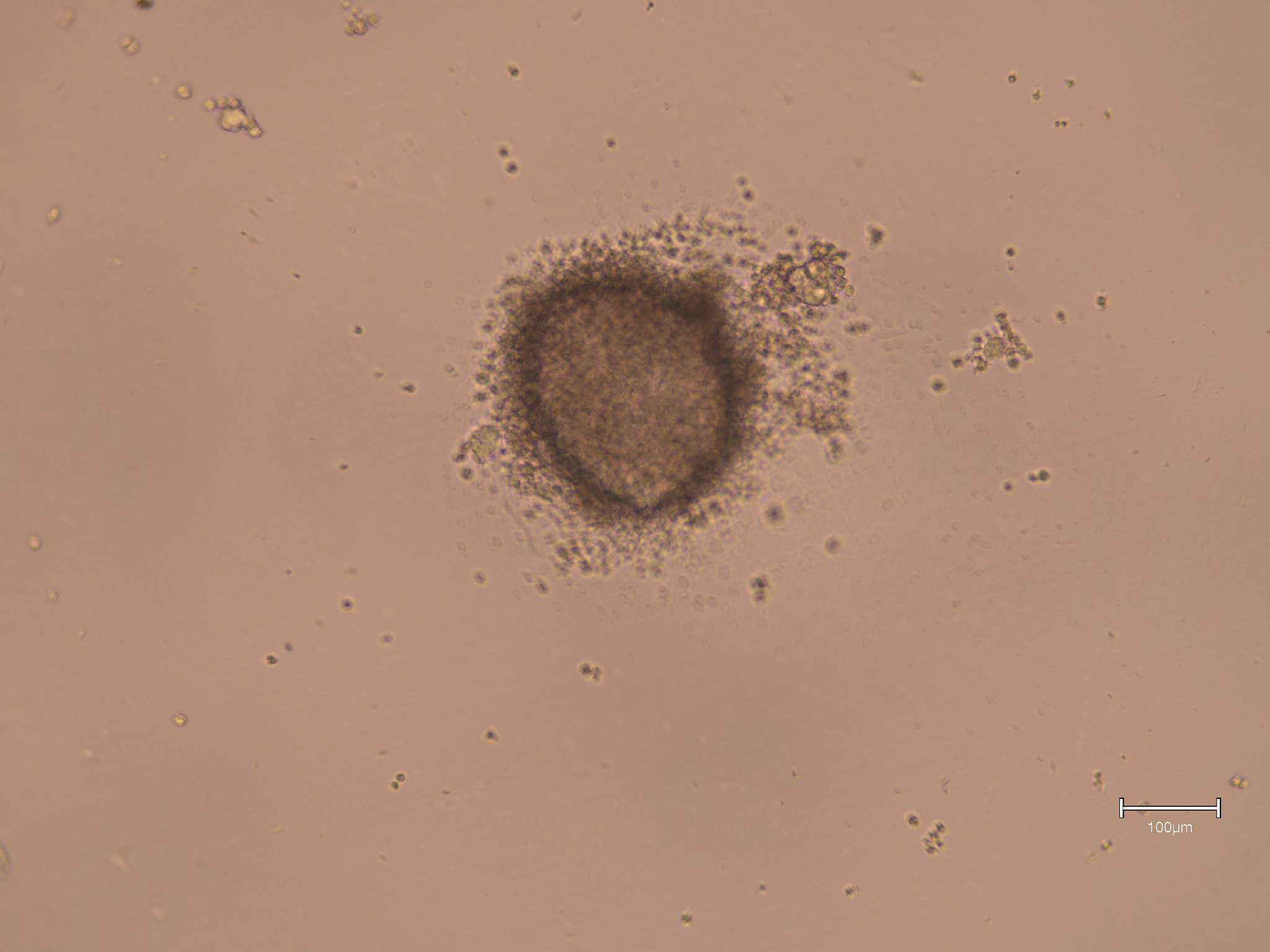

Supplement: Supplementary file 3 — Source data Fig. 1 [file 44318_2025_558_MOESM3_ESM.zip › Figure 1/panel 1B/KD-1_72h.tiff]

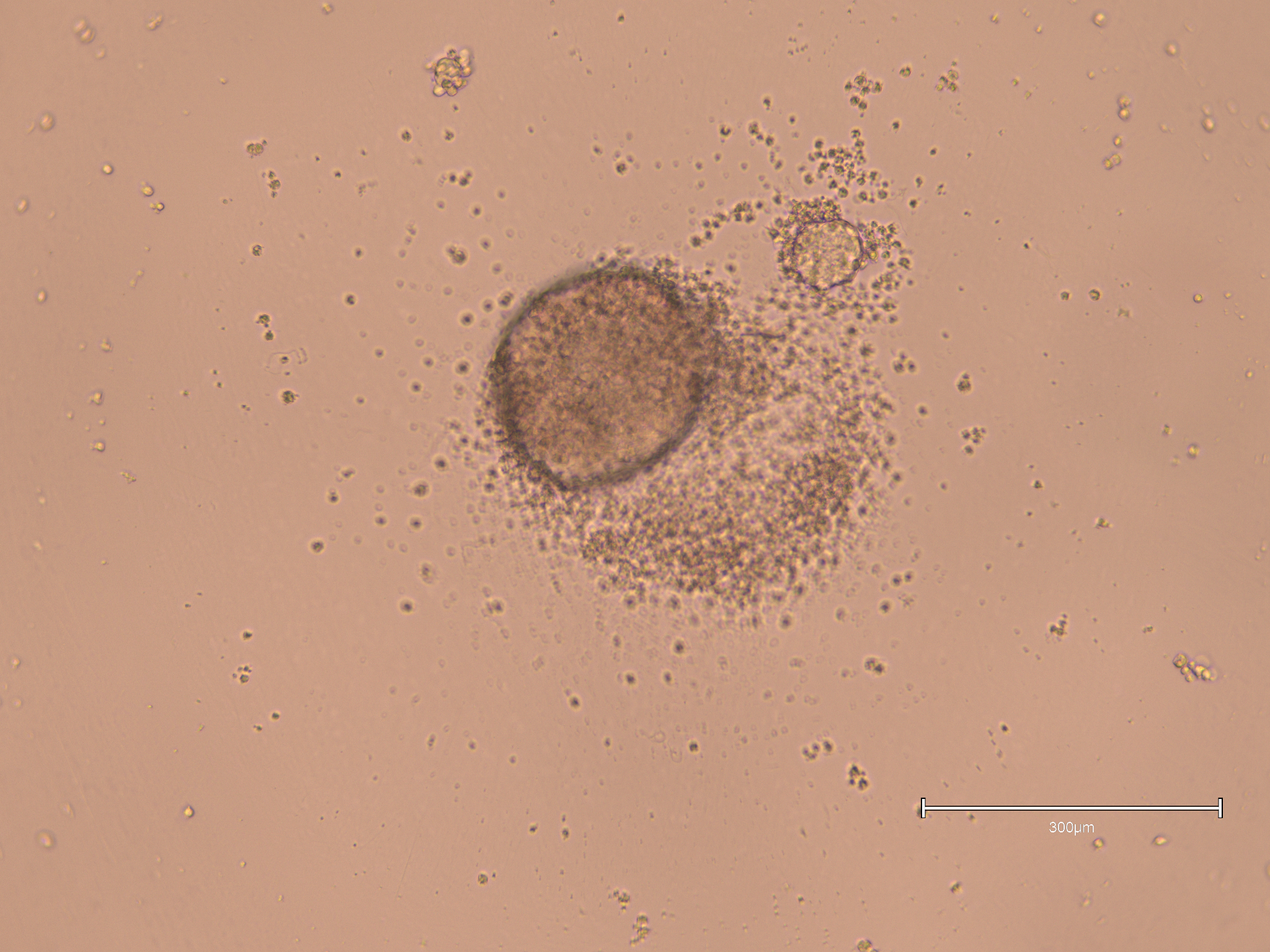

Supplement: Supplementary file 3 — Source data Fig. 1 [file 44318_2025_558_MOESM3_ESM.zip › Figure 1/panel 1B/KD-1_96h.tiff]

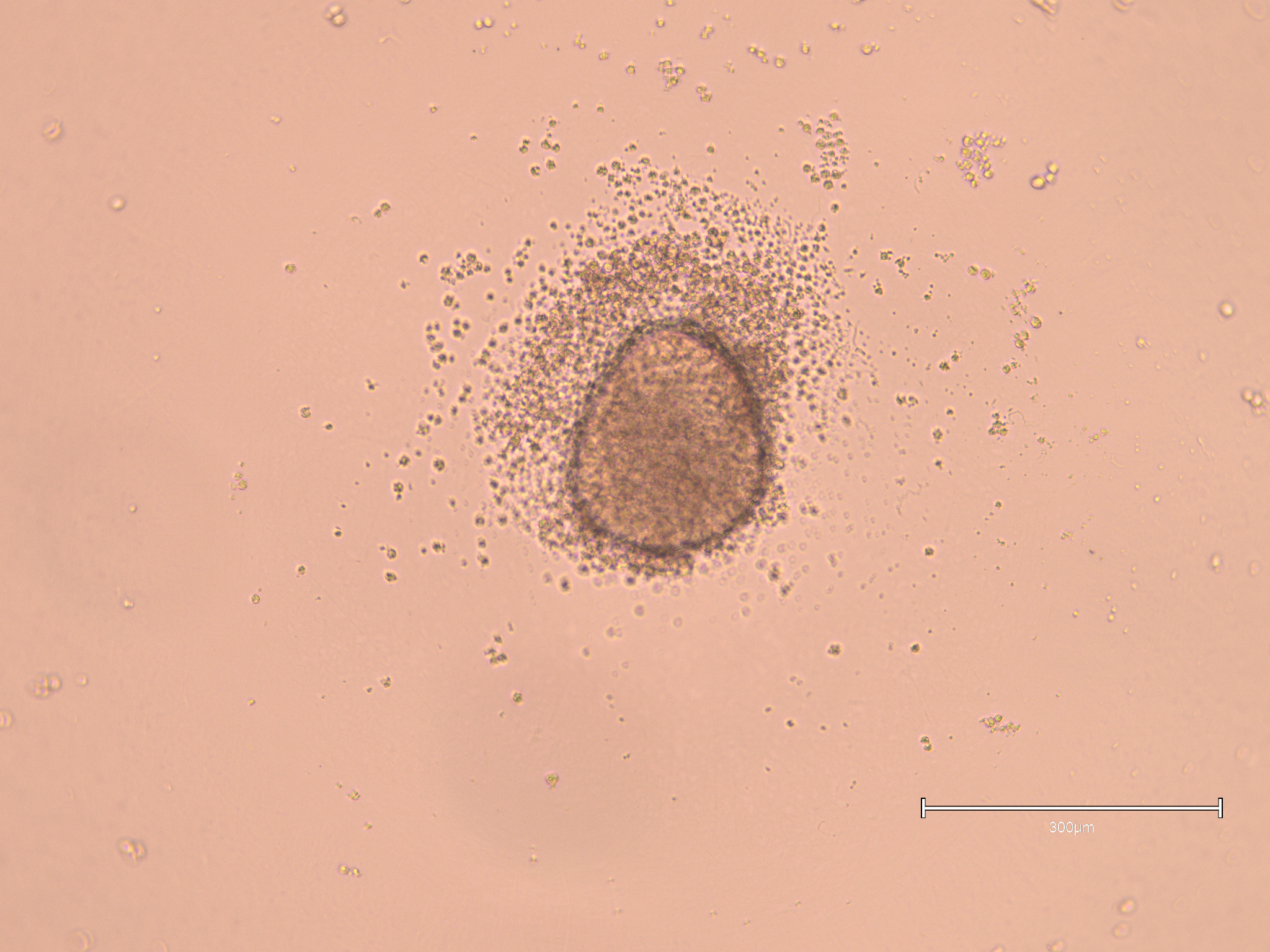

Supplement: Supplementary file 3 — Source data Fig. 1 [file 44318_2025_558_MOESM3_ESM.zip › Figure 1/panel 1B/NT_96h.tiff]

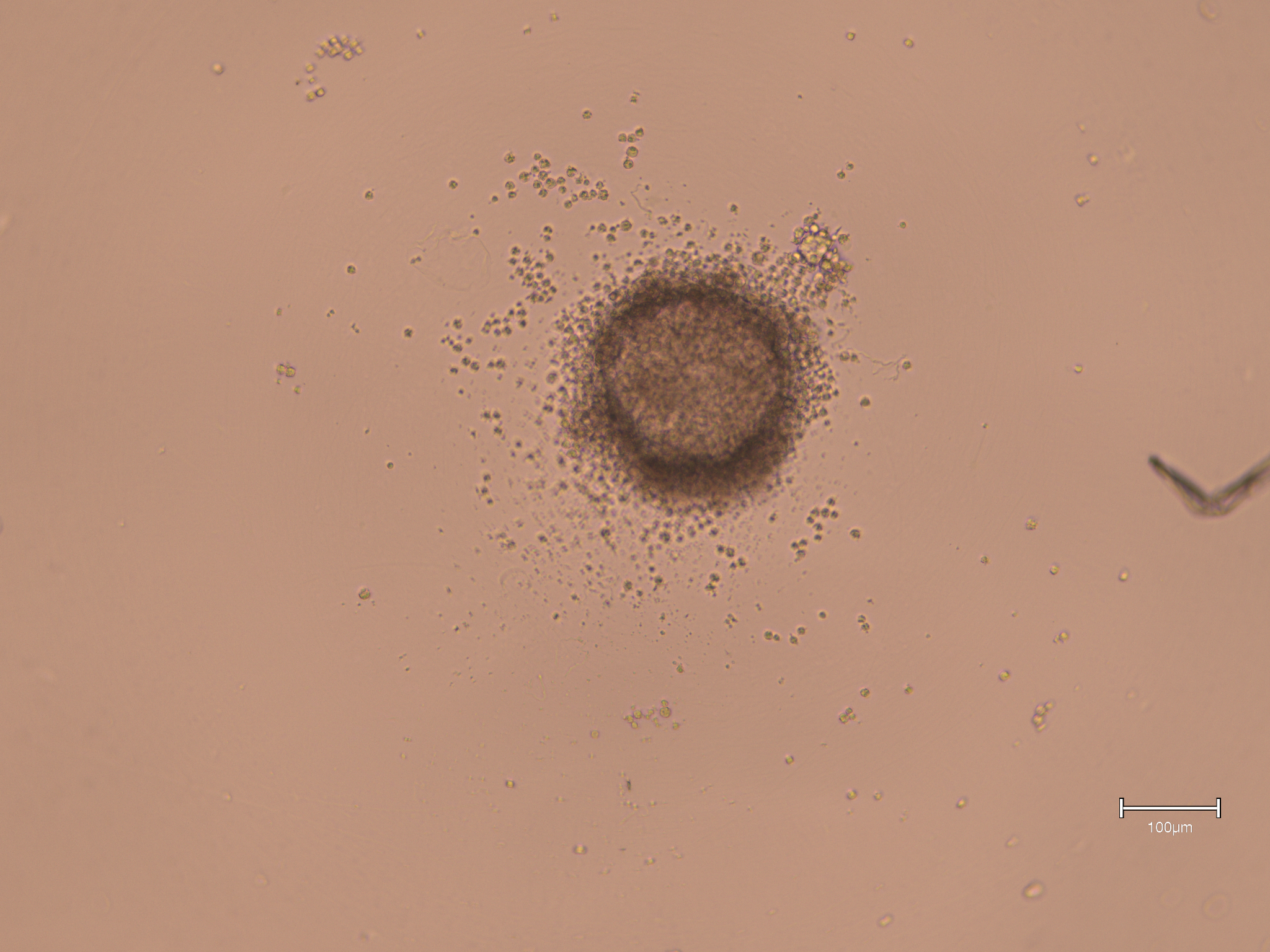

Supplement: Supplementary file 3 — Source data Fig. 1 [file 44318_2025_558_MOESM3_ESM.zip › Figure 1/panel 1B/NT_72h.tiff]

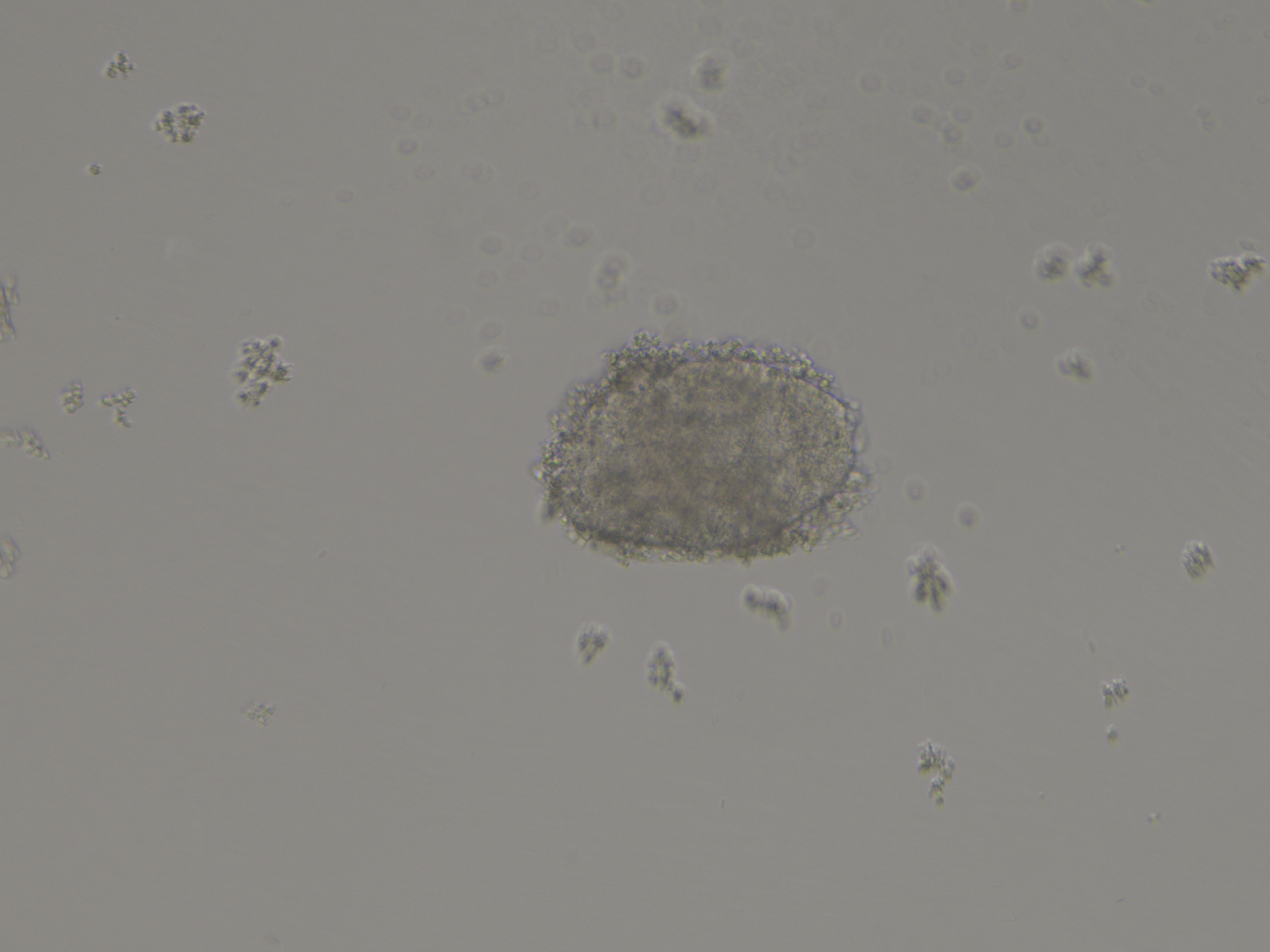

Supplement: Supplementary file 3 — Source data Fig. 1 [file 44318_2025_558_MOESM3_ESM.zip › Figure 1/panel 1B/KD-2_120h.tiff]

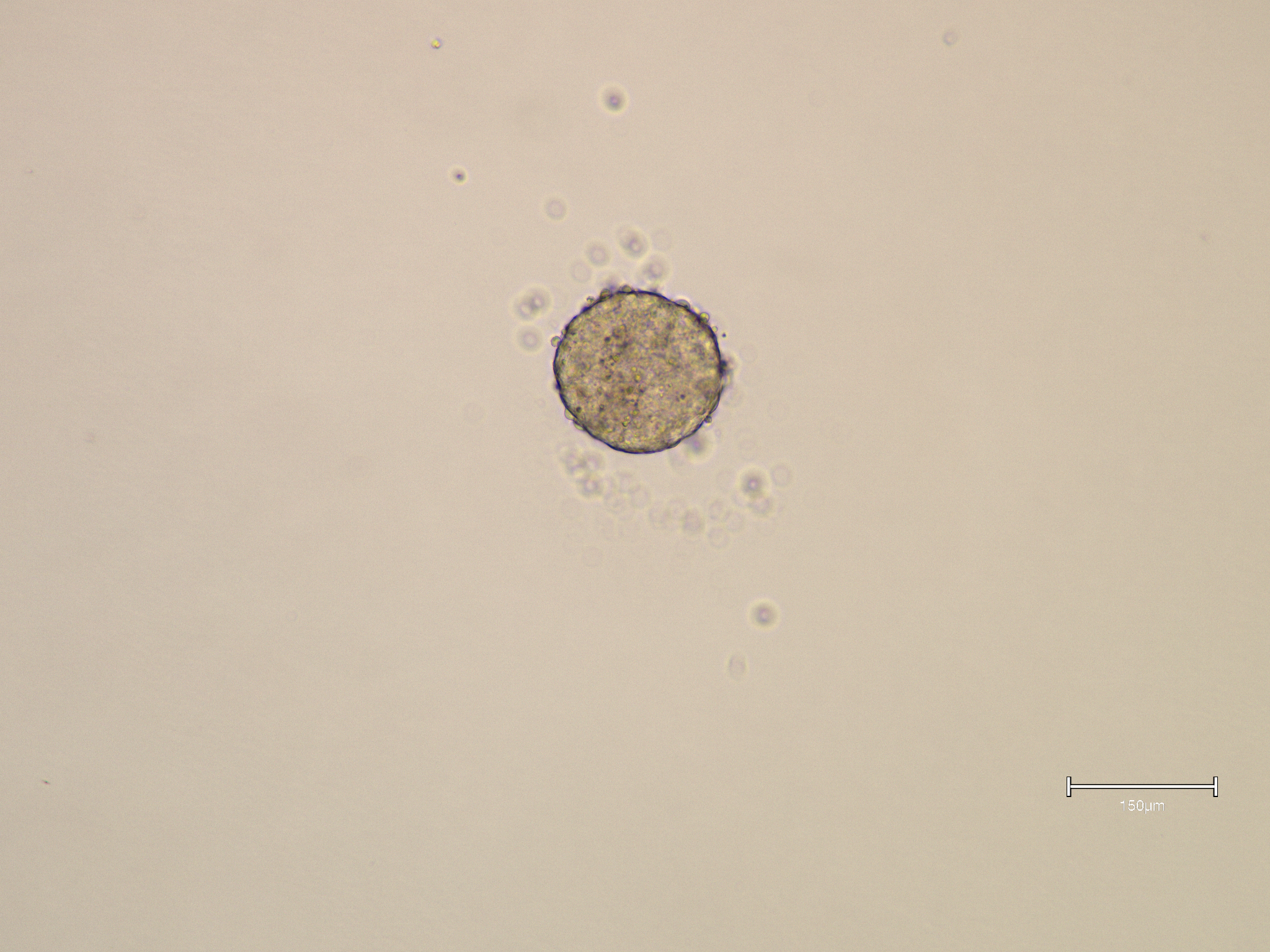

Supplement: Supplementary file 3 — Source data Fig. 1 [file 44318_2025_558_MOESM3_ESM.zip › Figure 1/panel 1B/KD-2_48h.tiff]

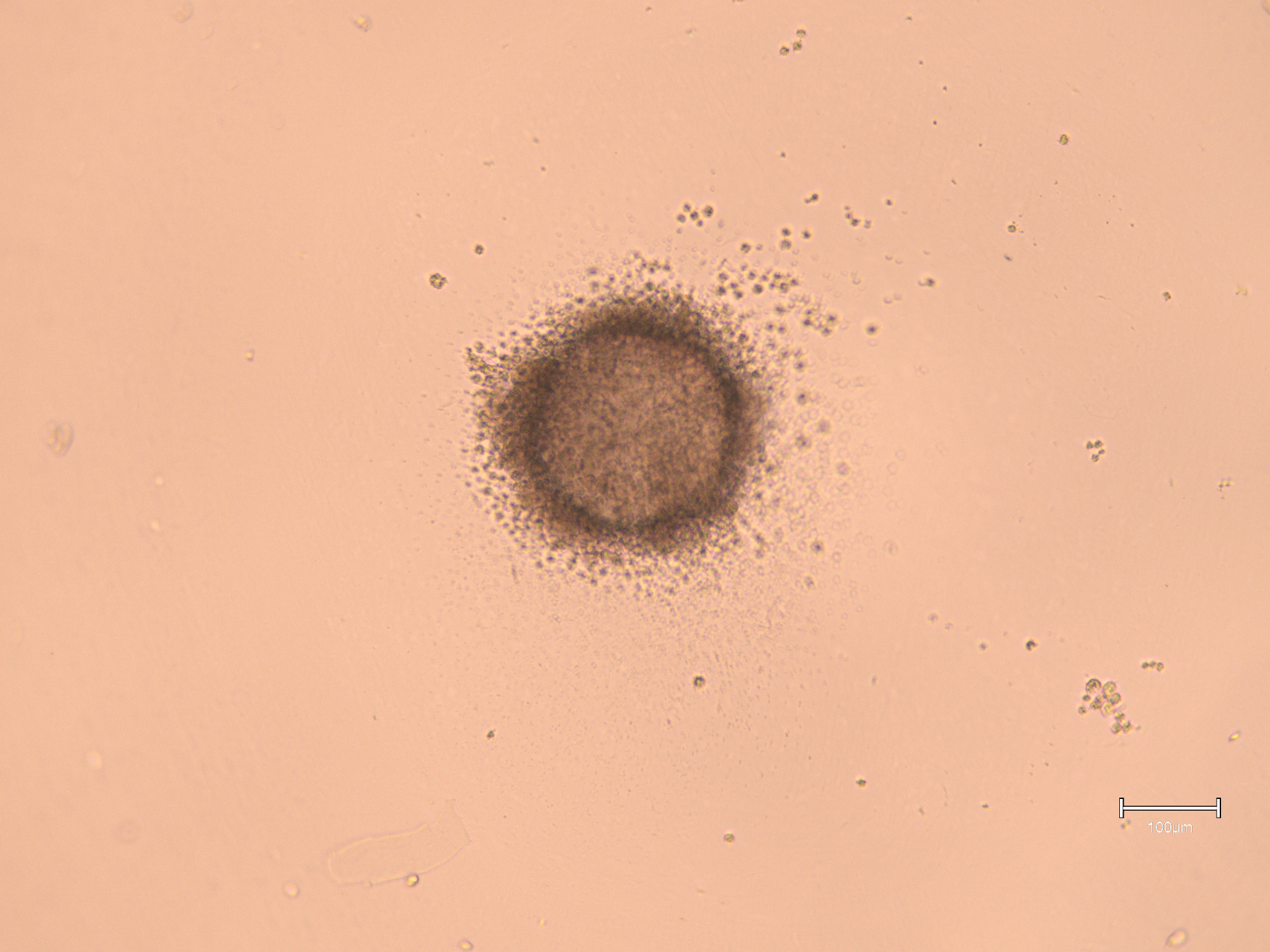

Supplement: Supplementary file 3 — Source data Fig. 1 [file 44318_2025_558_MOESM3_ESM.zip › Figure 1/panel 1B/KD-2_72h.tiff]

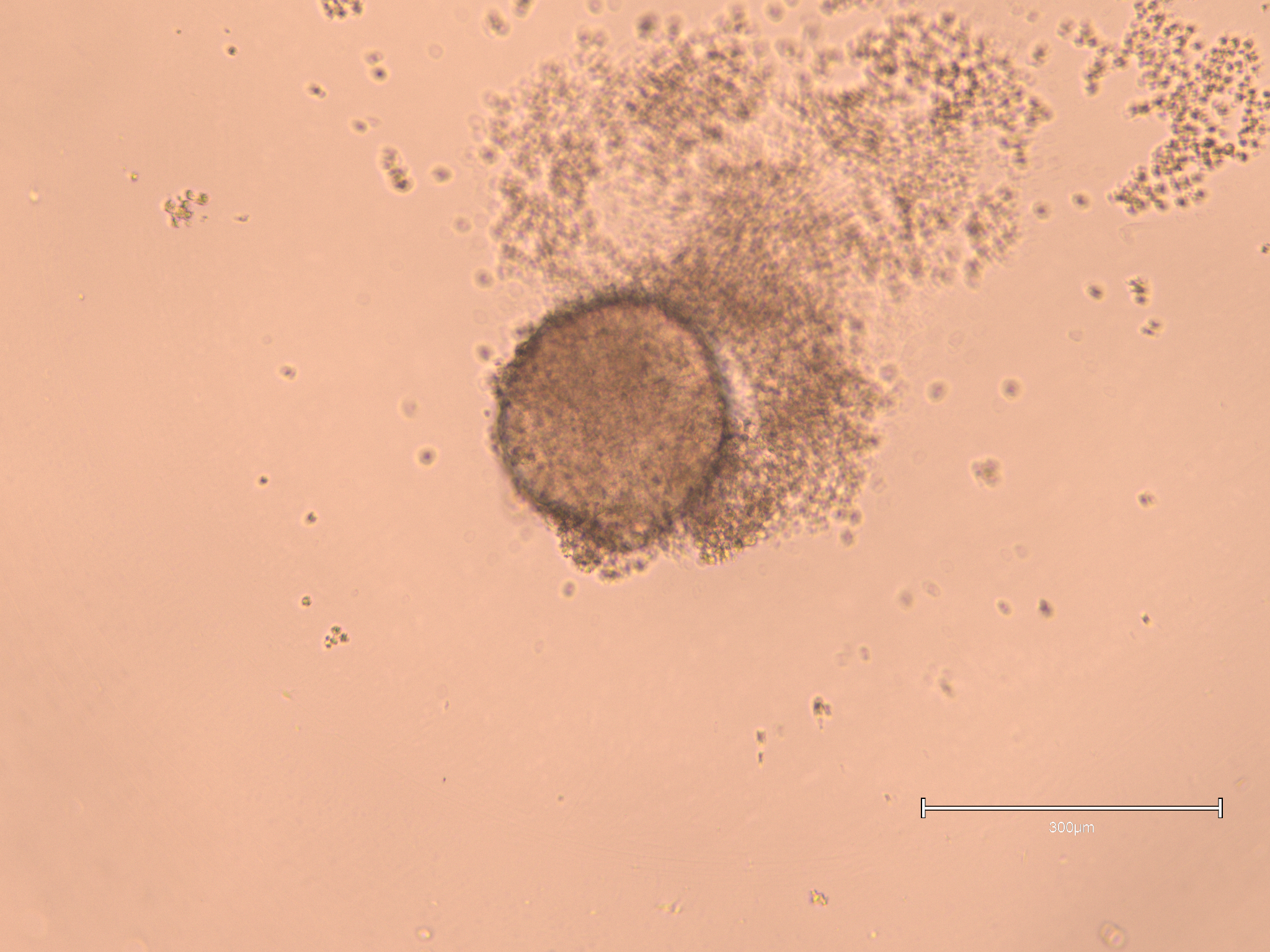

Supplement: Supplementary file 3 — Source data Fig. 1 [file 44318_2025_558_MOESM3_ESM.zip › Figure 1/panel 1B/KD-2_96h.tiff]

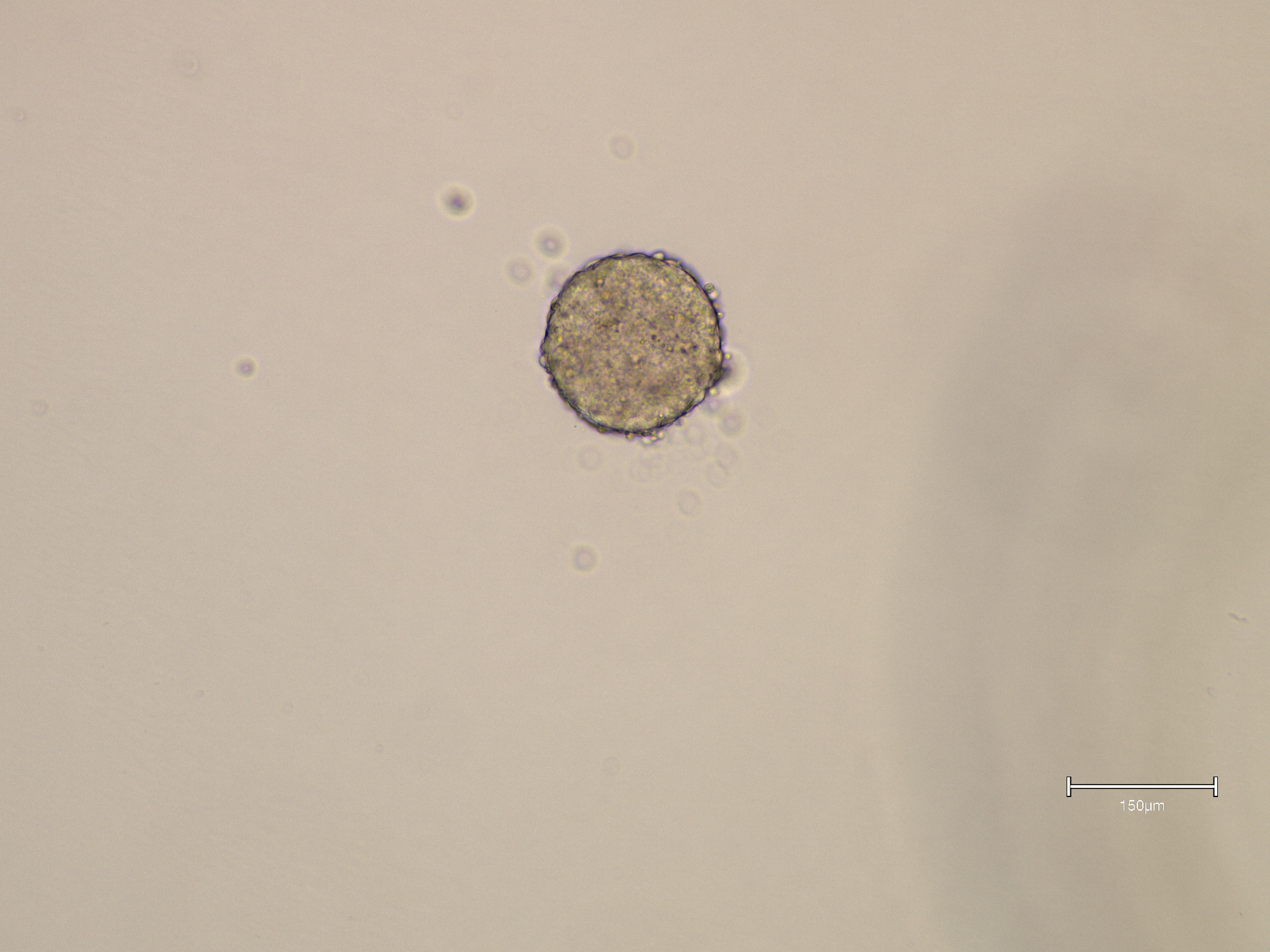

Supplement: Supplementary file 3 — Source data Fig. 1 [file 44318_2025_558_MOESM3_ESM.zip › Figure 1/panel 1B/NT_48h.tiff]

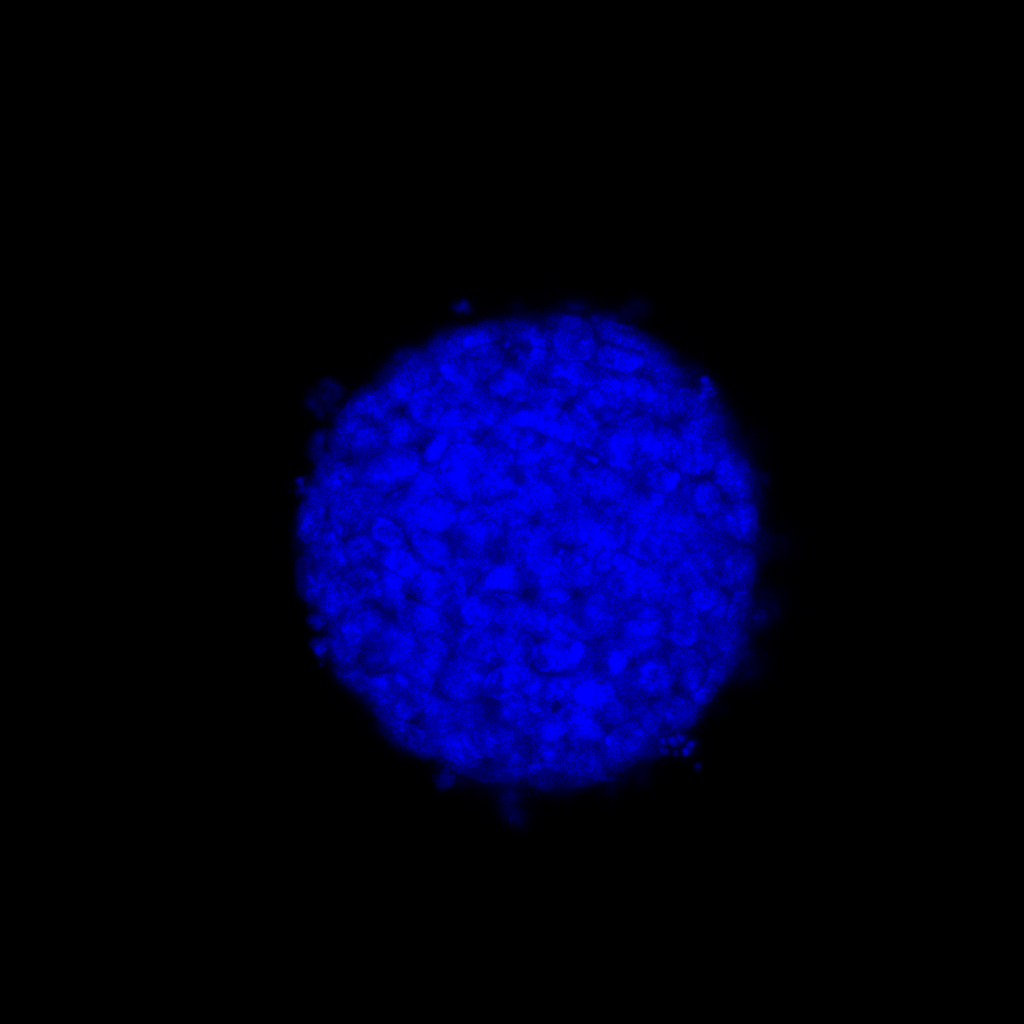

Supplement: Supplementary file 3 — Source data Fig. 1 [file 44318_2025_558_MOESM3_ESM.zip › Figure 1/panel 1D/KD-1_Bra/seq9645_seq9645_RGB_DAPI.tif]

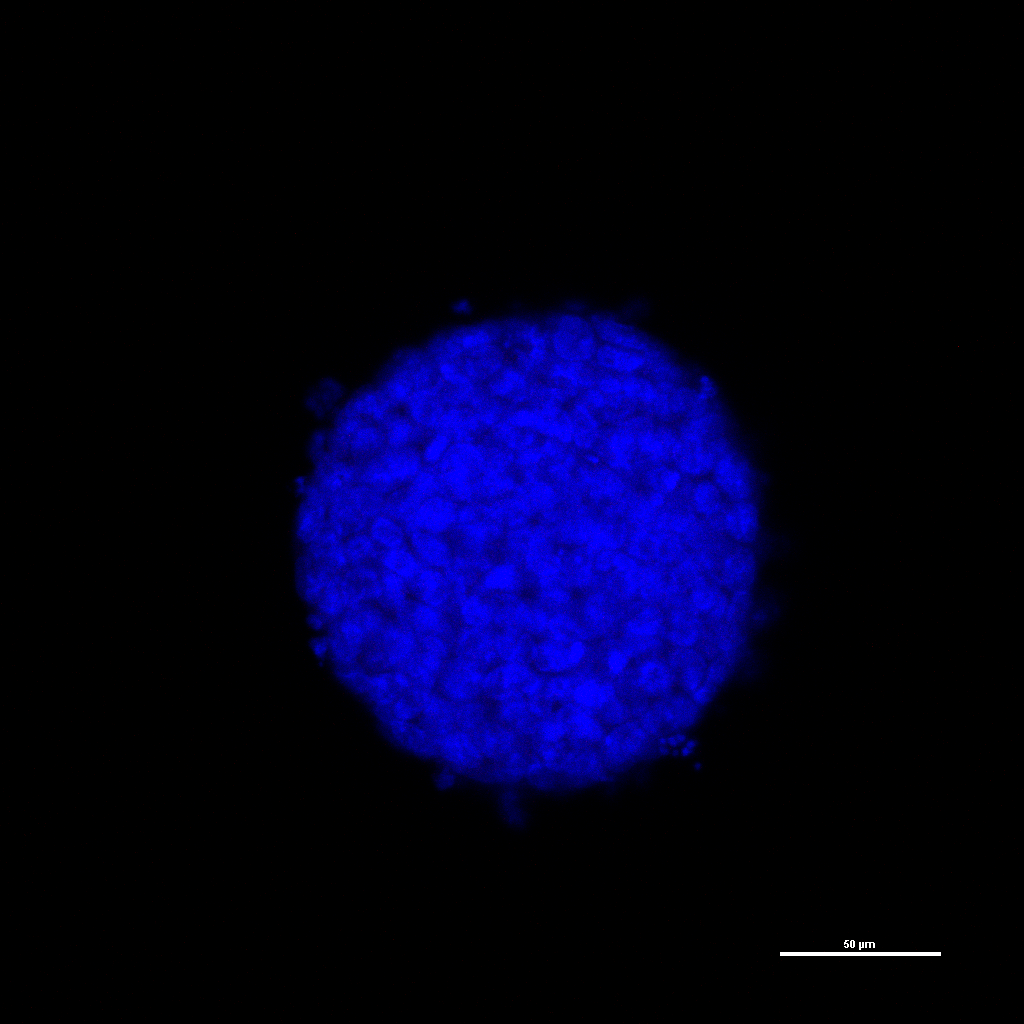

Supplement: Supplementary file 3 — Source data Fig. 1 [file 44318_2025_558_MOESM3_ESM.zip › Figure 1/panel 1D/KD-1_Bra/seq9645_seq9645_RGB.tif]

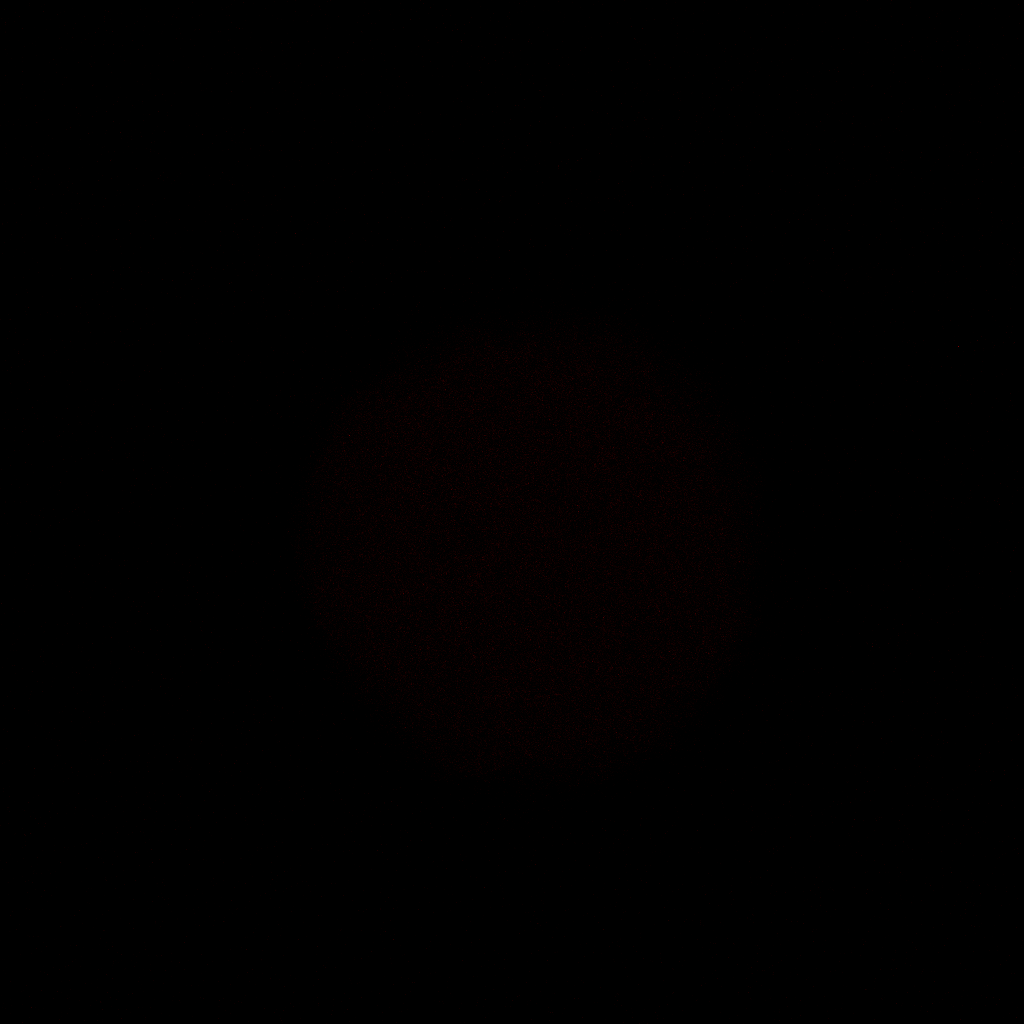

Supplement: Supplementary file 3 — Source data Fig. 1 [file 44318_2025_558_MOESM3_ESM.zip › Figure 1/panel 1D/KD-1_Bra/seq9645_seq9645_RGB_Texas Red.tif]

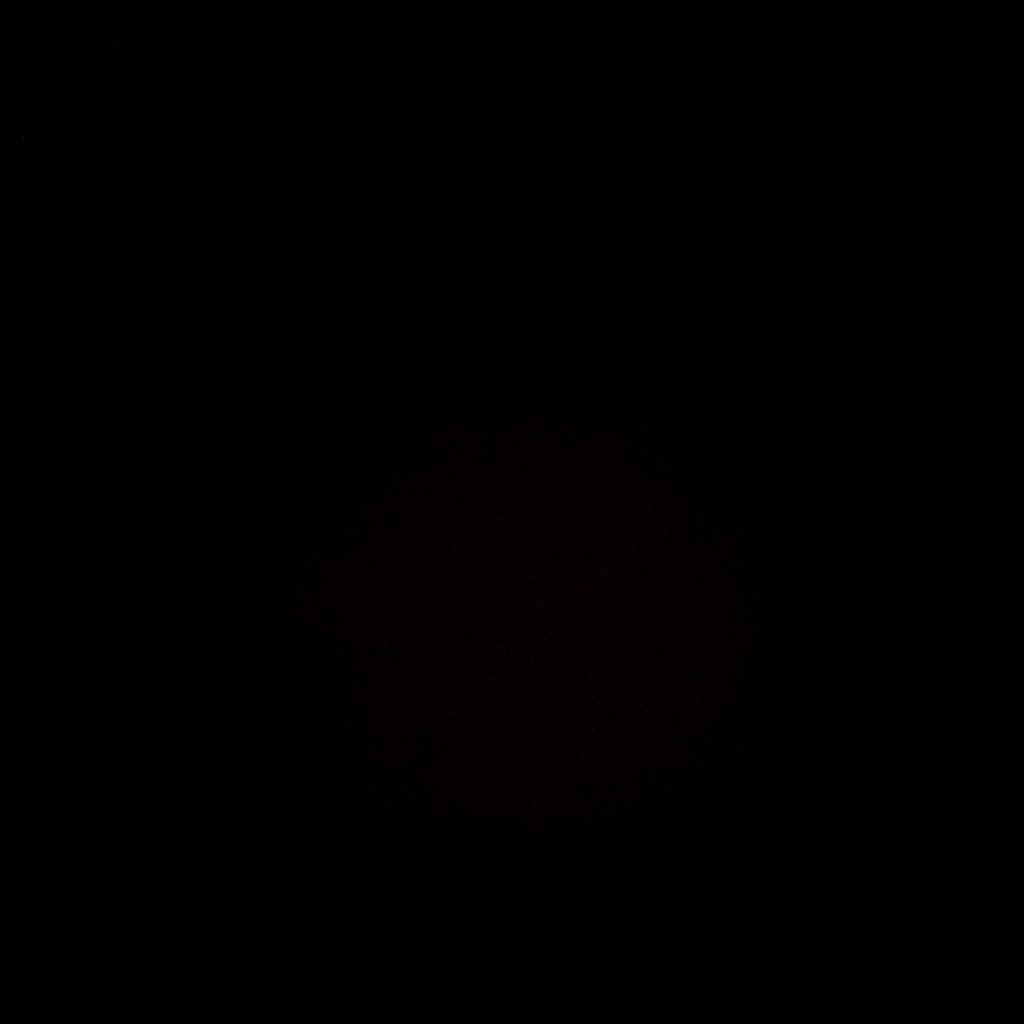

Supplement: Supplementary file 3 — Source data Fig. 1 [file 44318_2025_558_MOESM3_ESM.zip › Figure 1/panel 1D/KD-2_Bra/seq9643_seq9643_RGB_Texas Red.tif]

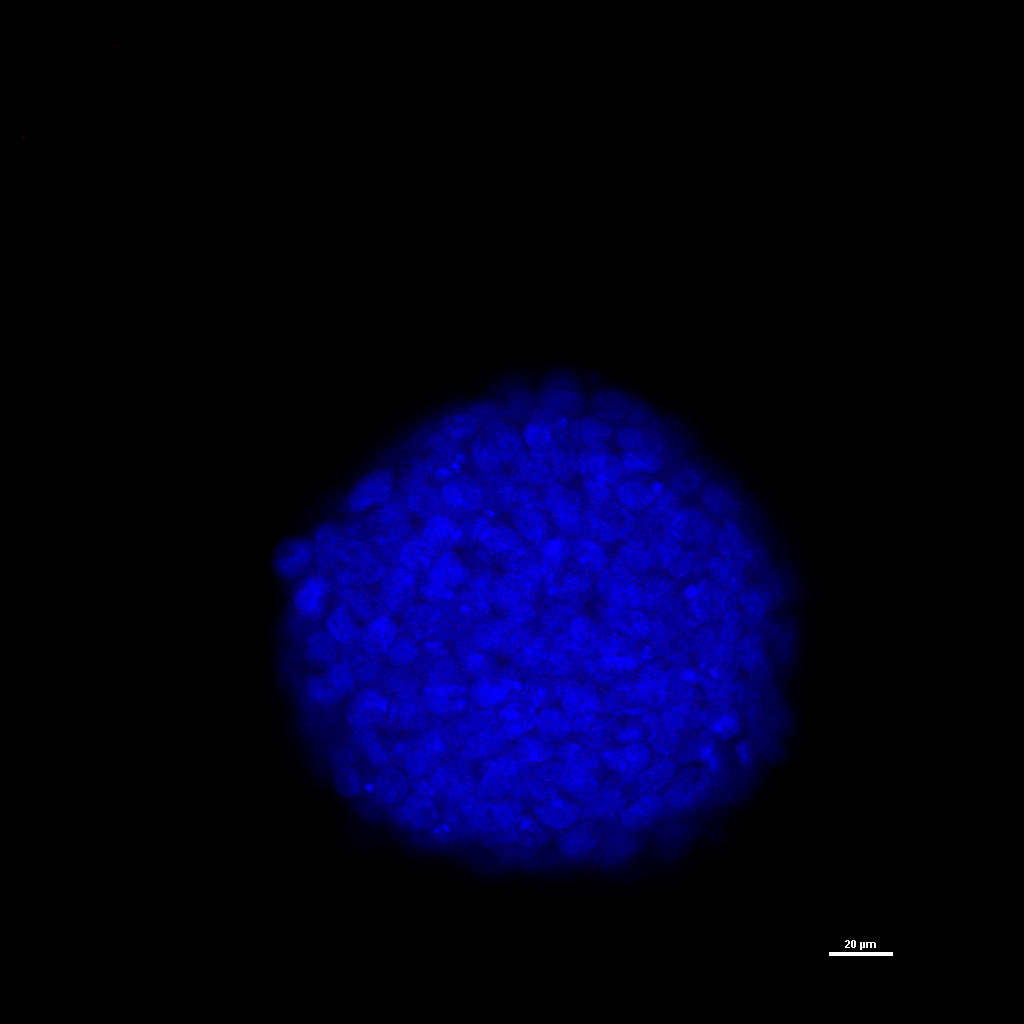

Supplement: Supplementary file 3 — Source data Fig. 1 [file 44318_2025_558_MOESM3_ESM.zip › Figure 1/panel 1D/KD-2_Bra/seq9643_seq9643_RGB.tif]

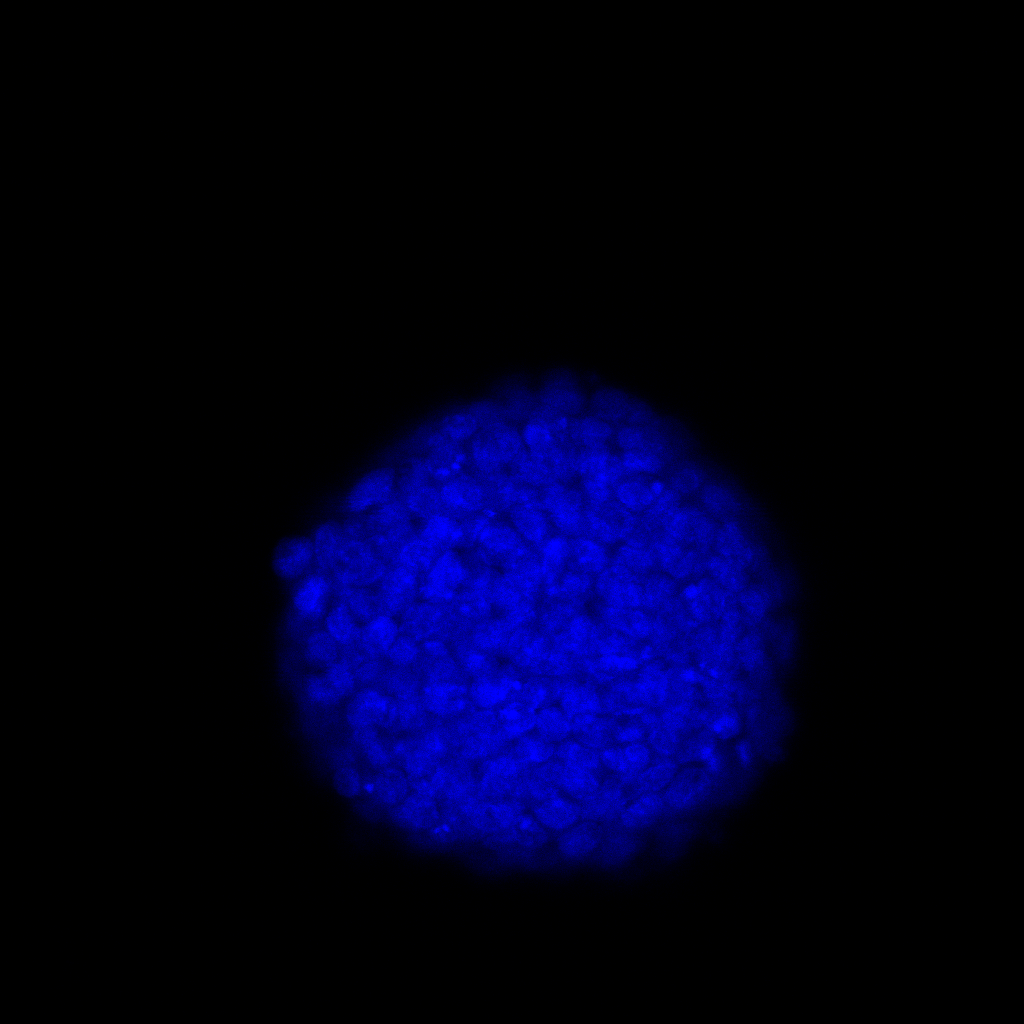

Supplement: Supplementary file 3 — Source data Fig. 1 [file 44318_2025_558_MOESM3_ESM.zip › Figure 1/panel 1D/KD-2_Bra/seq9643_seq9643_RGB_DAPI.tif]

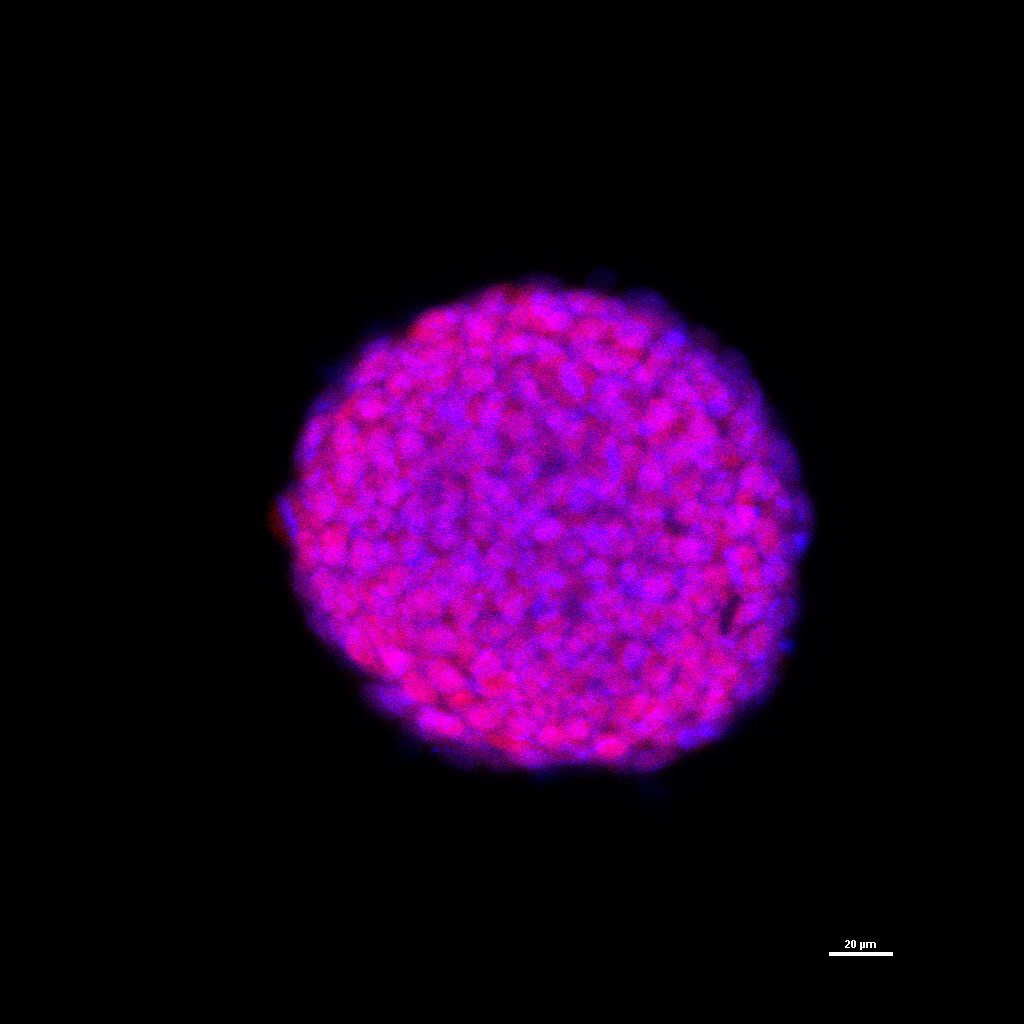

Supplement: Supplementary file 3 — Source data Fig. 1 [file 44318_2025_558_MOESM3_ESM.zip › Figure 1/panel 1D/KD-2_Oct4/seq9638_seq9638_RGB.tif]

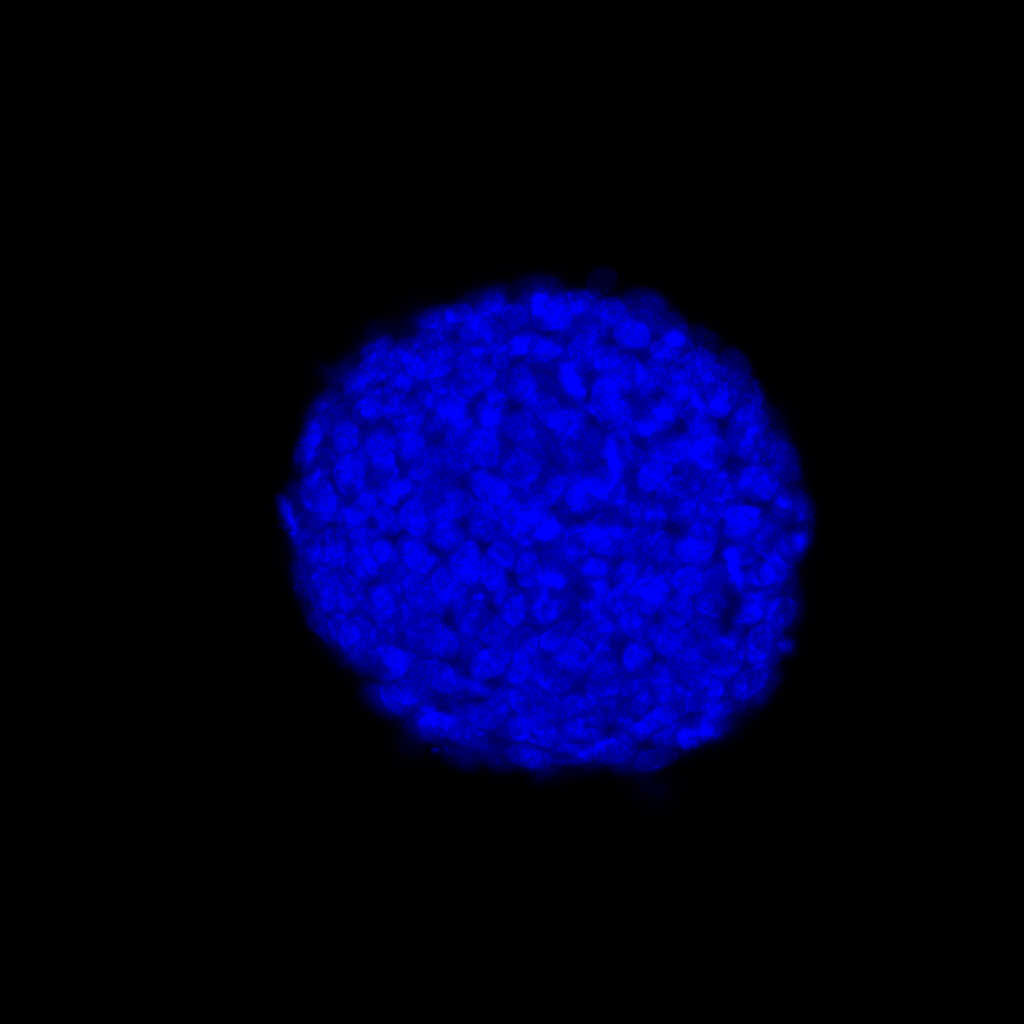

Supplement: Supplementary file 3 — Source data Fig. 1 [file 44318_2025_558_MOESM3_ESM.zip › Figure 1/panel 1D/KD-2_Oct4/seq9638_seq9638_RGB_DAPI.tif]

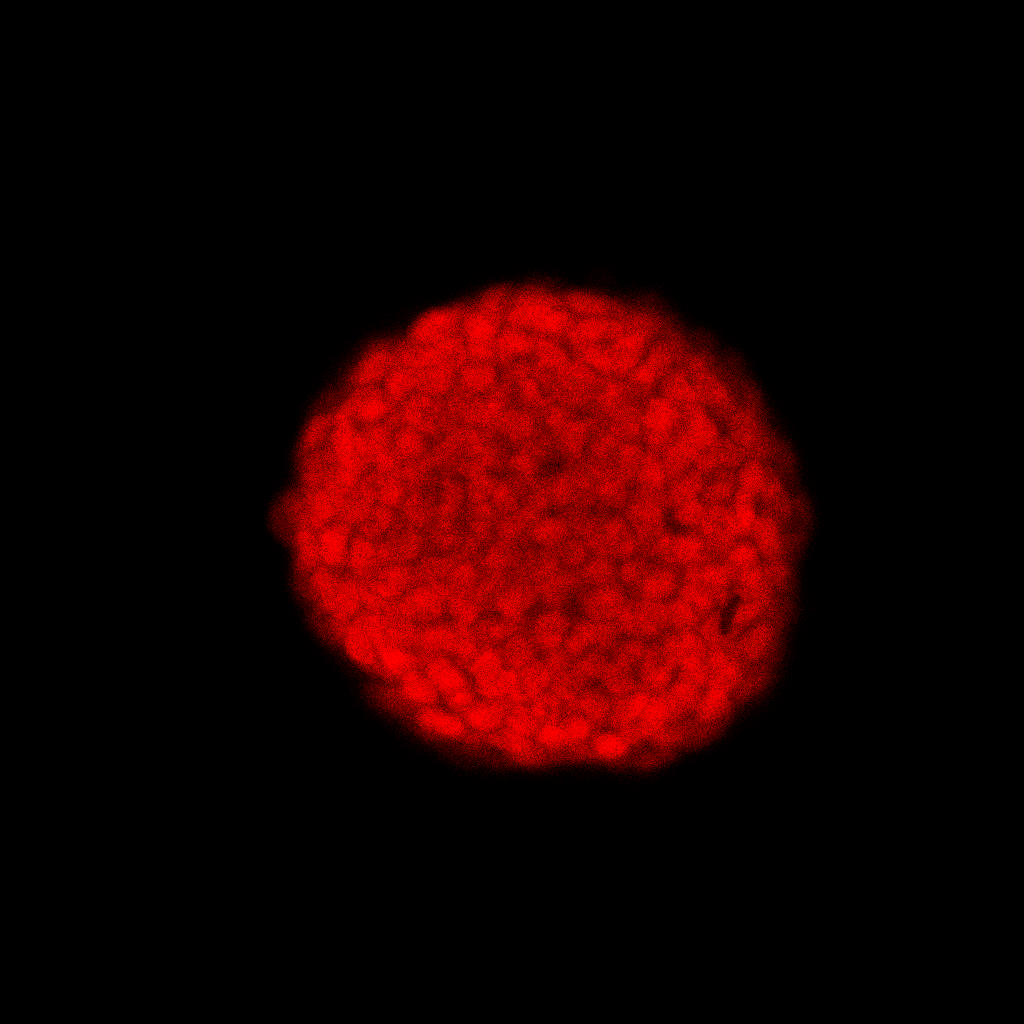

Supplement: Supplementary file 3 — Source data Fig. 1 [file 44318_2025_558_MOESM3_ESM.zip › Figure 1/panel 1D/KD-2_Oct4/seq9638_seq9638_RGB_Texas Red.tif]

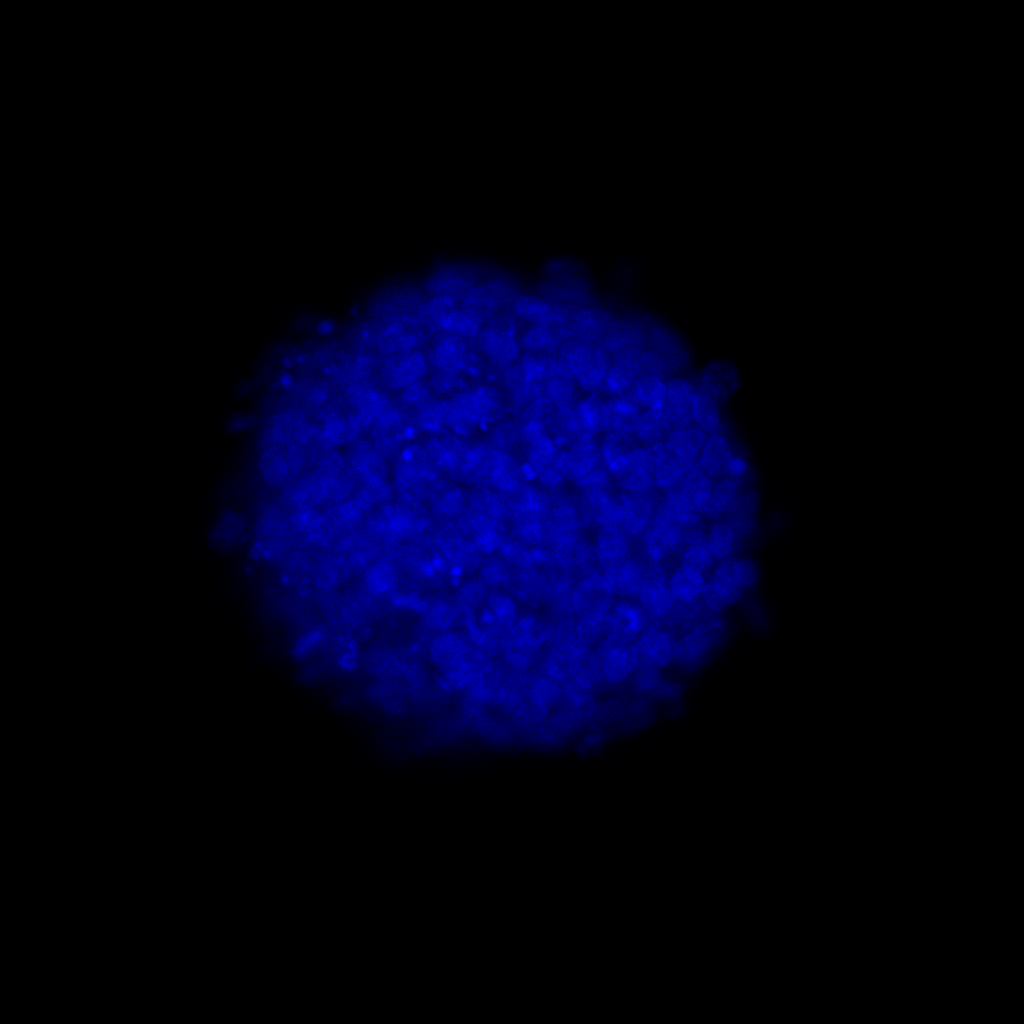

Supplement: Supplementary file 3 — Source data Fig. 1 [file 44318_2025_558_MOESM3_ESM.zip › Figure 1/panel 1D/NT_Bra/seq9649_seq9649_RGB_DAPI.tif]

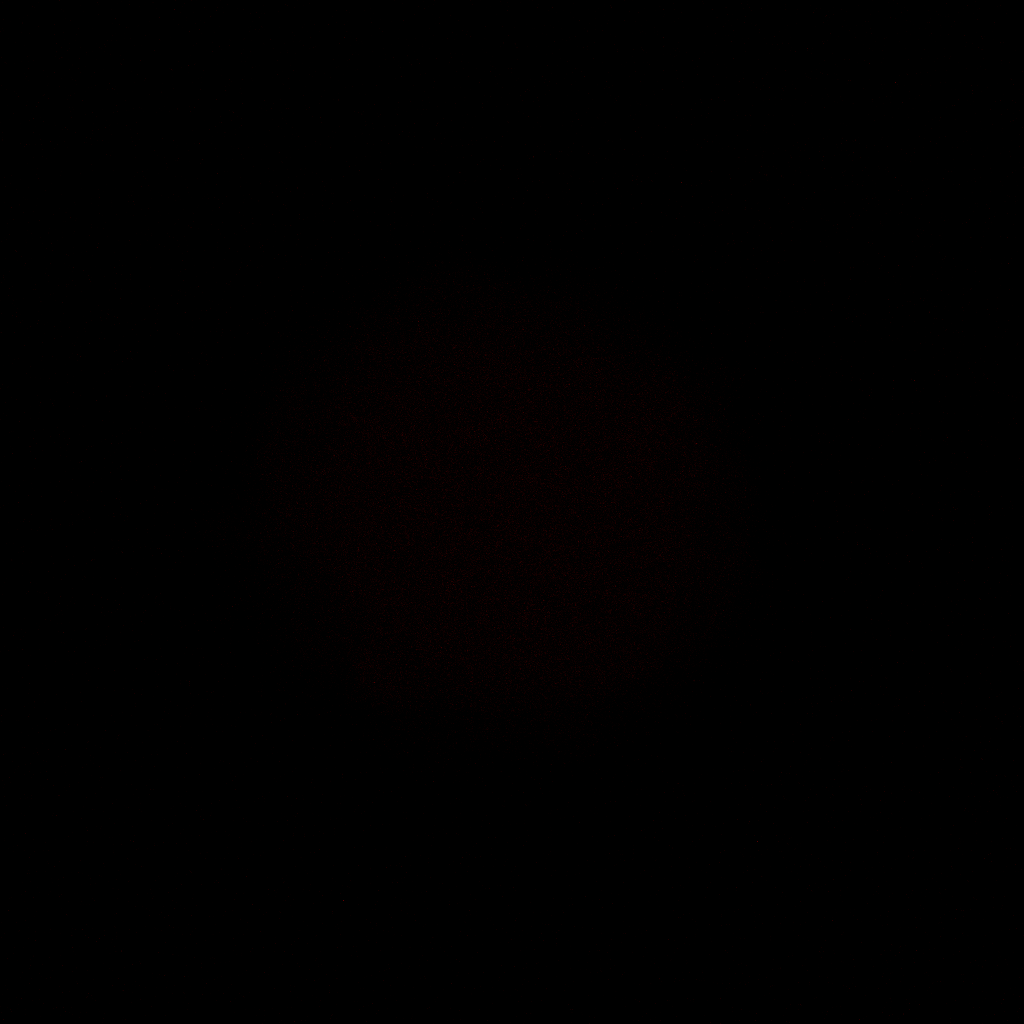

Supplement: Supplementary file 3 — Source data Fig. 1 [file 44318_2025_558_MOESM3_ESM.zip › Figure 1/panel 1D/NT_Bra/seq9649_seq9649_RGB_Texas Red.tif]

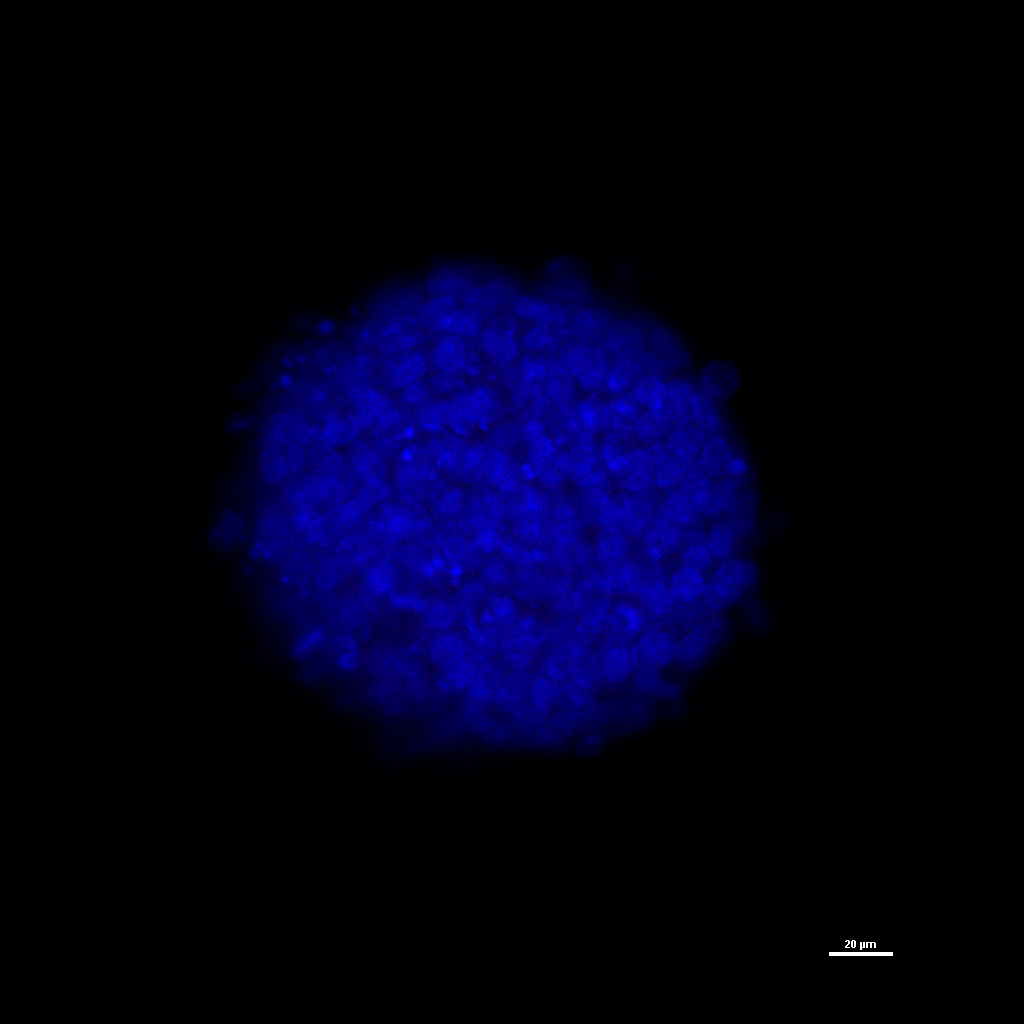

Supplement: Supplementary file 3 — Source data Fig. 1 [file 44318_2025_558_MOESM3_ESM.zip › Figure 1/panel 1D/NT_Bra/seq9649_seq9649_RGB.tif]

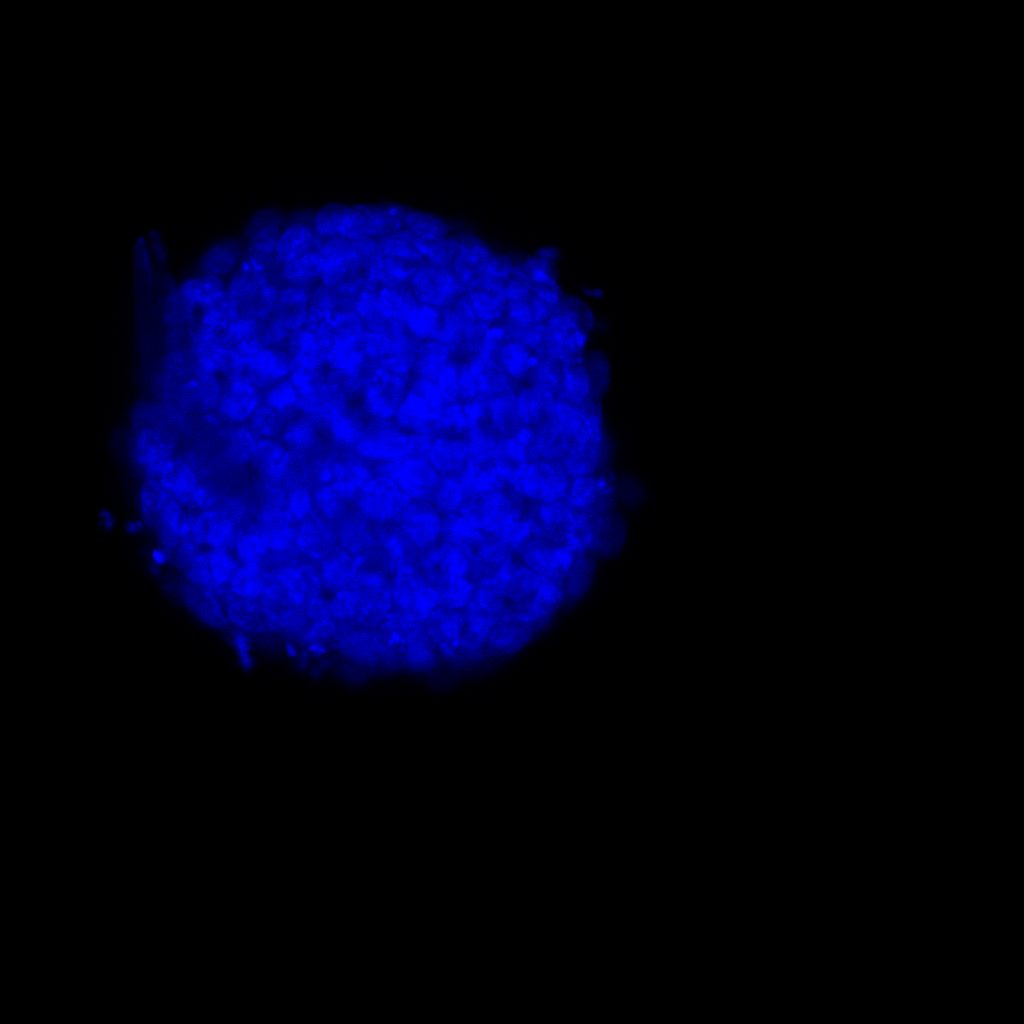

Supplement: Supplementary file 3 — Source data Fig. 1 [file 44318_2025_558_MOESM3_ESM.zip › Figure 1/panel 1D/NT_Oct4/seq9633_seq9633_RGB_DAPI.tif]

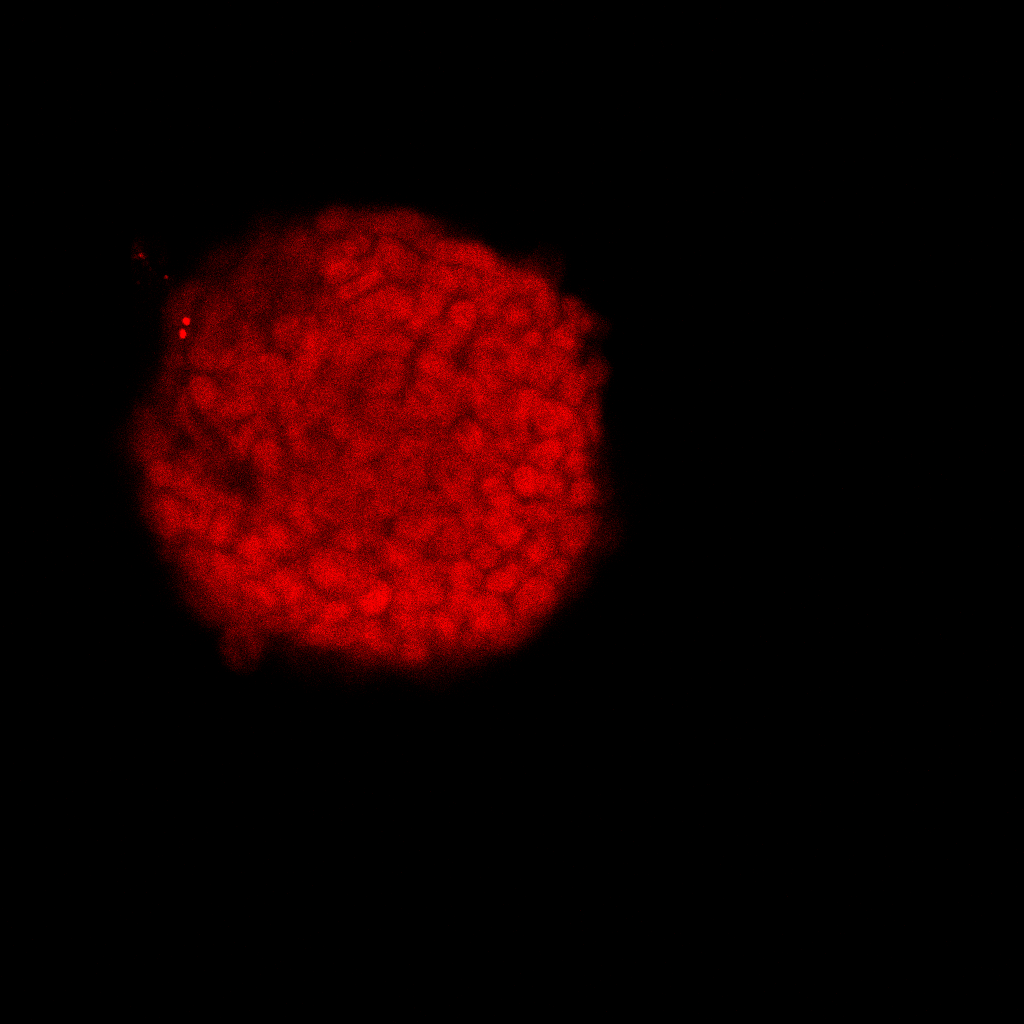

Supplement: Supplementary file 3 — Source data Fig. 1 [file 44318_2025_558_MOESM3_ESM.zip › Figure 1/panel 1D/NT_Oct4/seq9633_seq9633_RGB_Texas Red.tif]

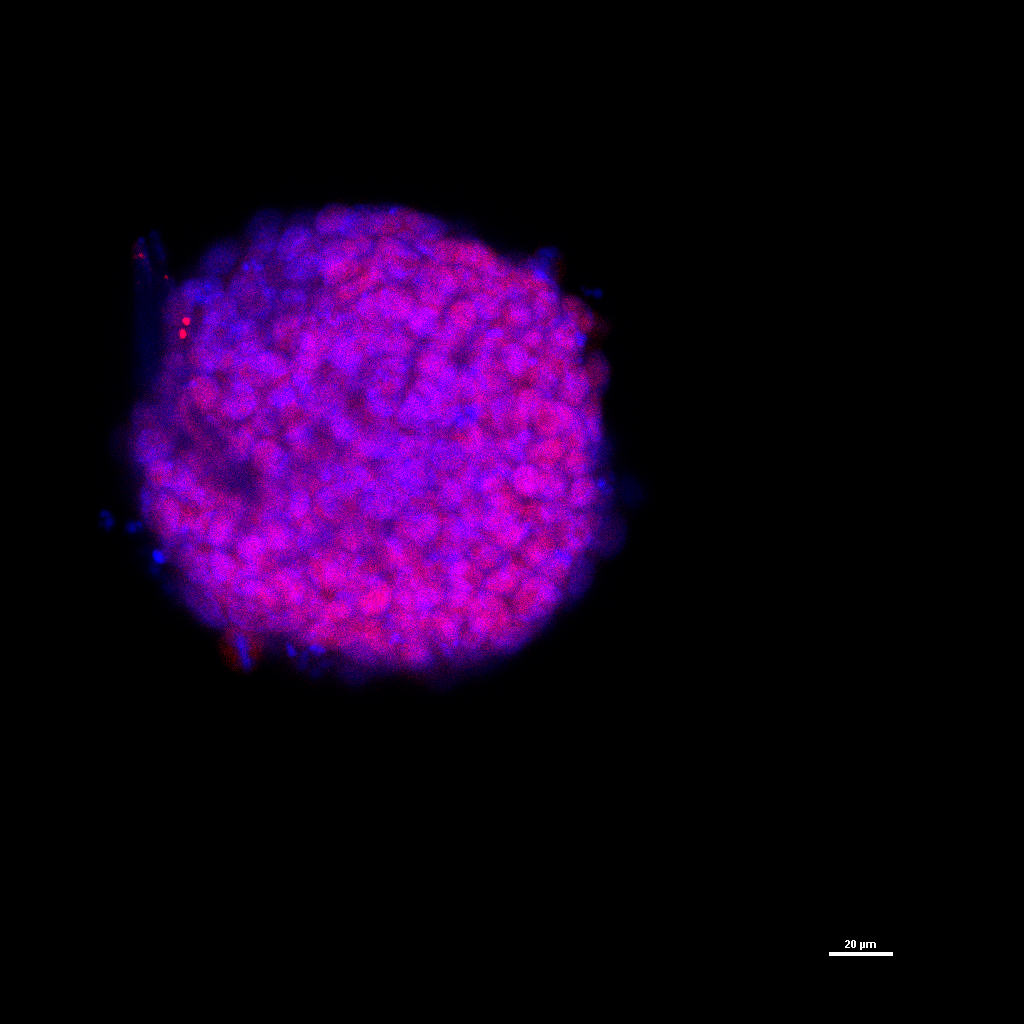

Supplement: Supplementary file 3 — Source data Fig. 1 [file 44318_2025_558_MOESM3_ESM.zip › Figure 1/panel 1D/NT_Oct4/seq9633_seq9633_RGB.tif]

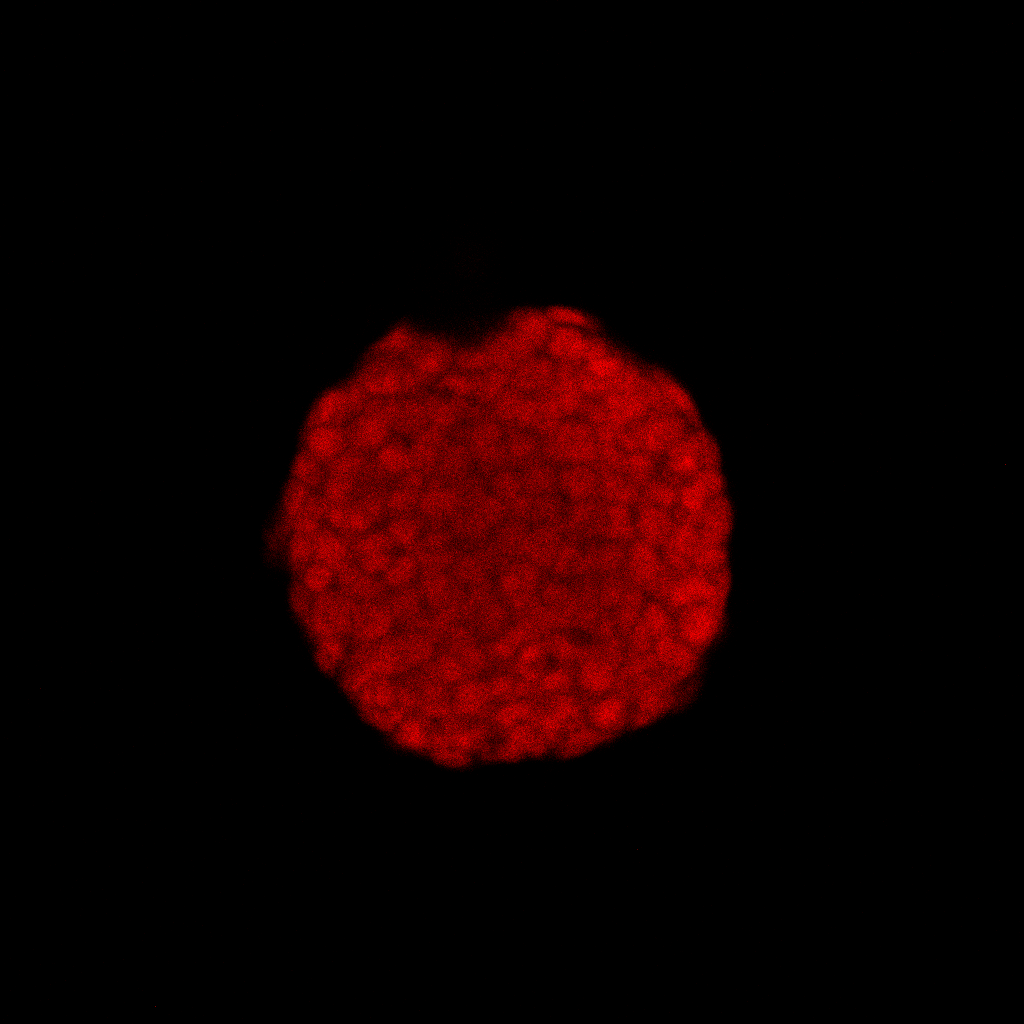

Supplement: Supplementary file 3 — Source data Fig. 1 [file 44318_2025_558_MOESM3_ESM.zip › Figure 1/panel 1D/KD-1_Oct4/seq9635_seq9635_RGB_Texas Red.tif]

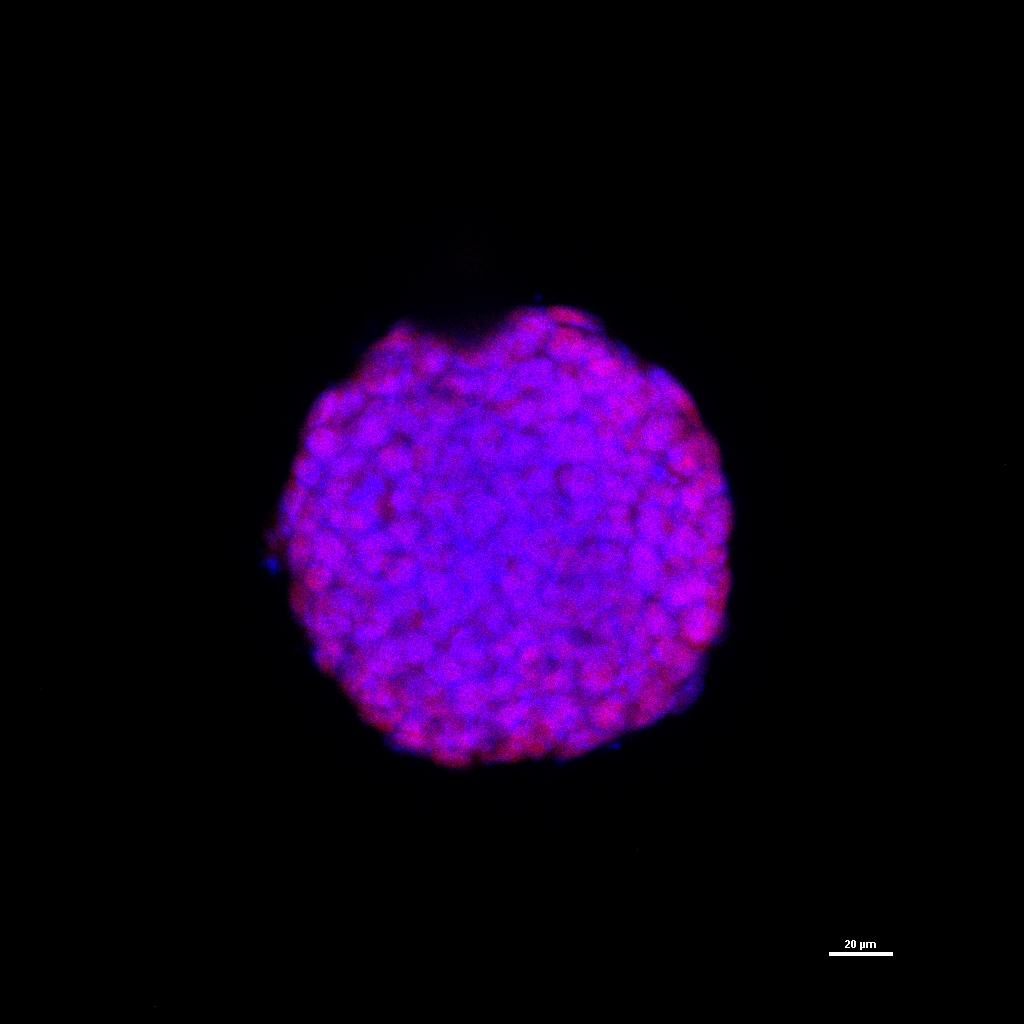

Supplement: Supplementary file 3 — Source data Fig. 1 [file 44318_2025_558_MOESM3_ESM.zip › Figure 1/panel 1D/KD-1_Oct4/seq9635_seq9635_RGB.tif]

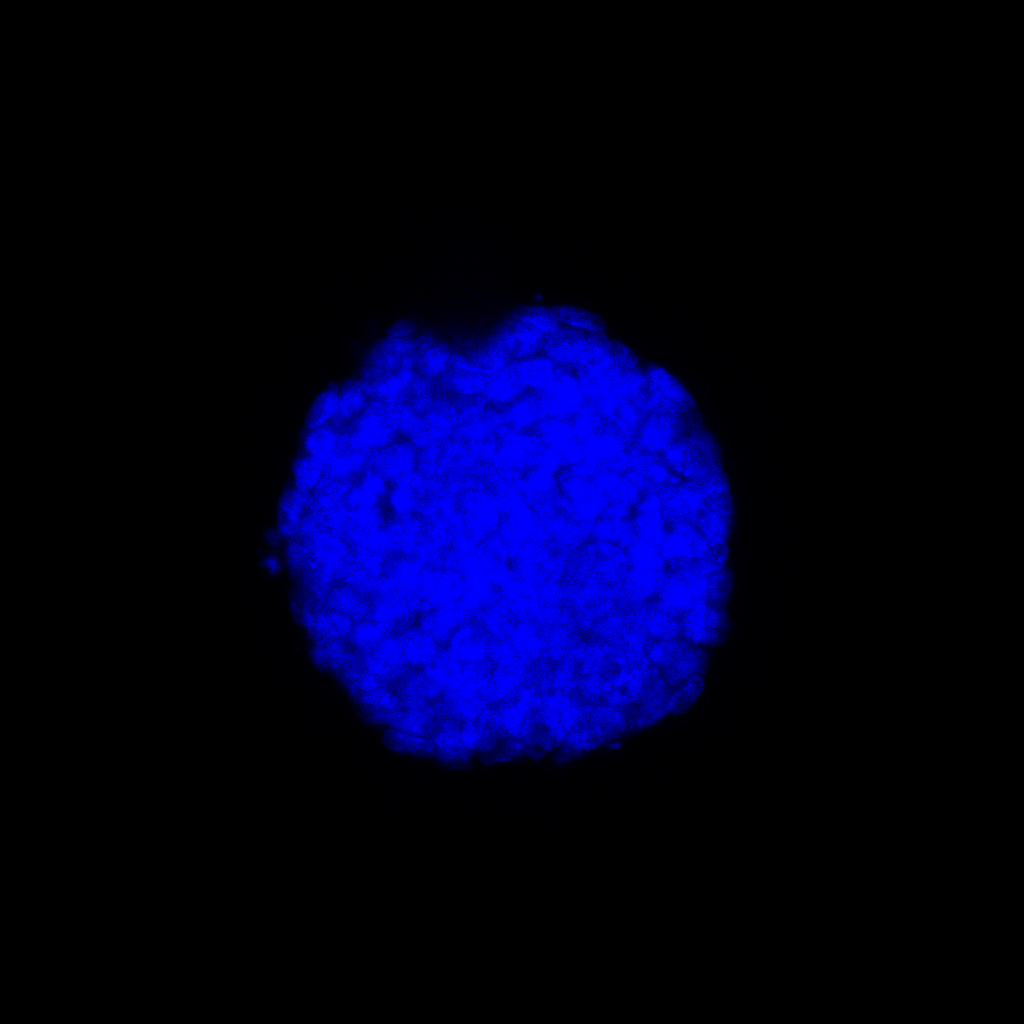

Supplement: Supplementary file 3 — Source data Fig. 1 [file 44318_2025_558_MOESM3_ESM.zip › Figure 1/panel 1D/KD-1_Oct4/seq9635_seq9635_RGB_DAPI.tif]

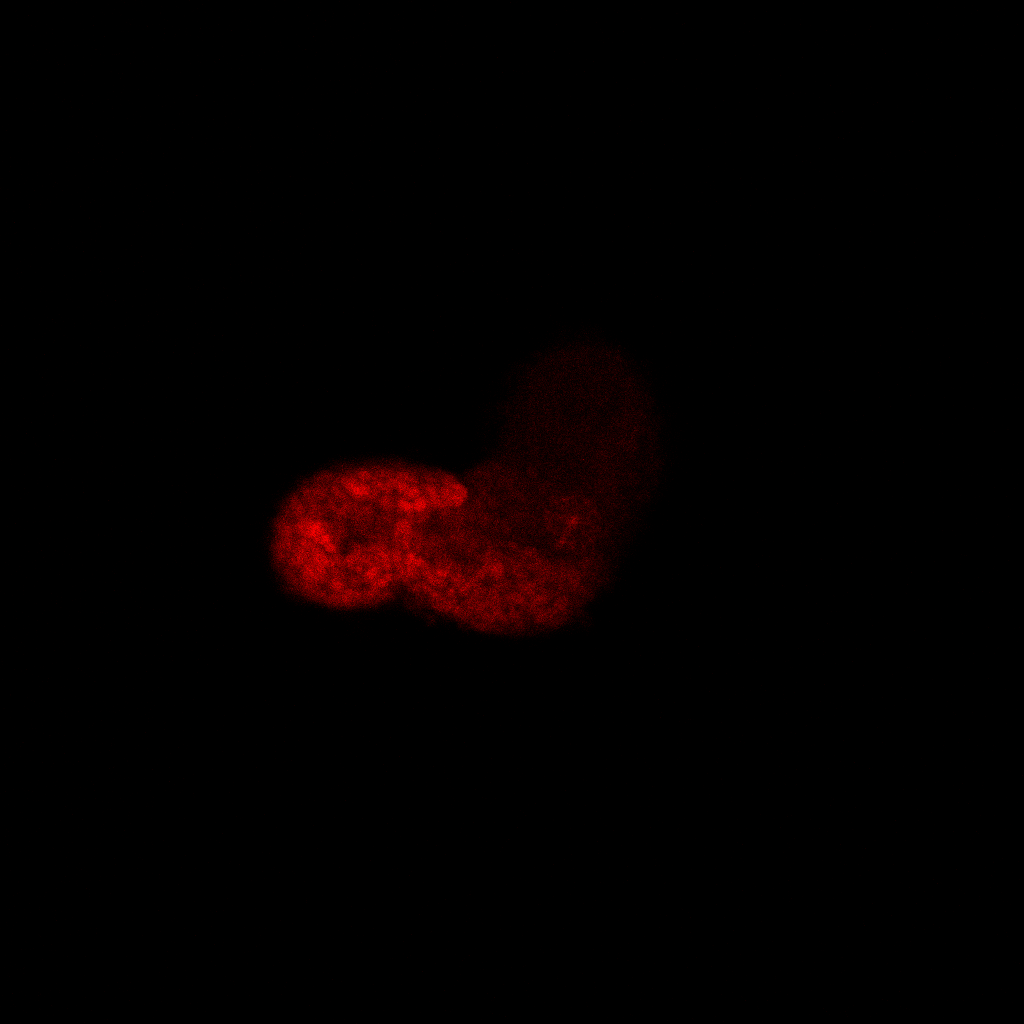

Supplement: Supplementary file 3 — Source data Fig. 1 [file 44318_2025_558_MOESM3_ESM.zip › Figure 1/panel 1F/NT_Cdx2/seq8790_seq8790_RGB_Texas Red.tif]

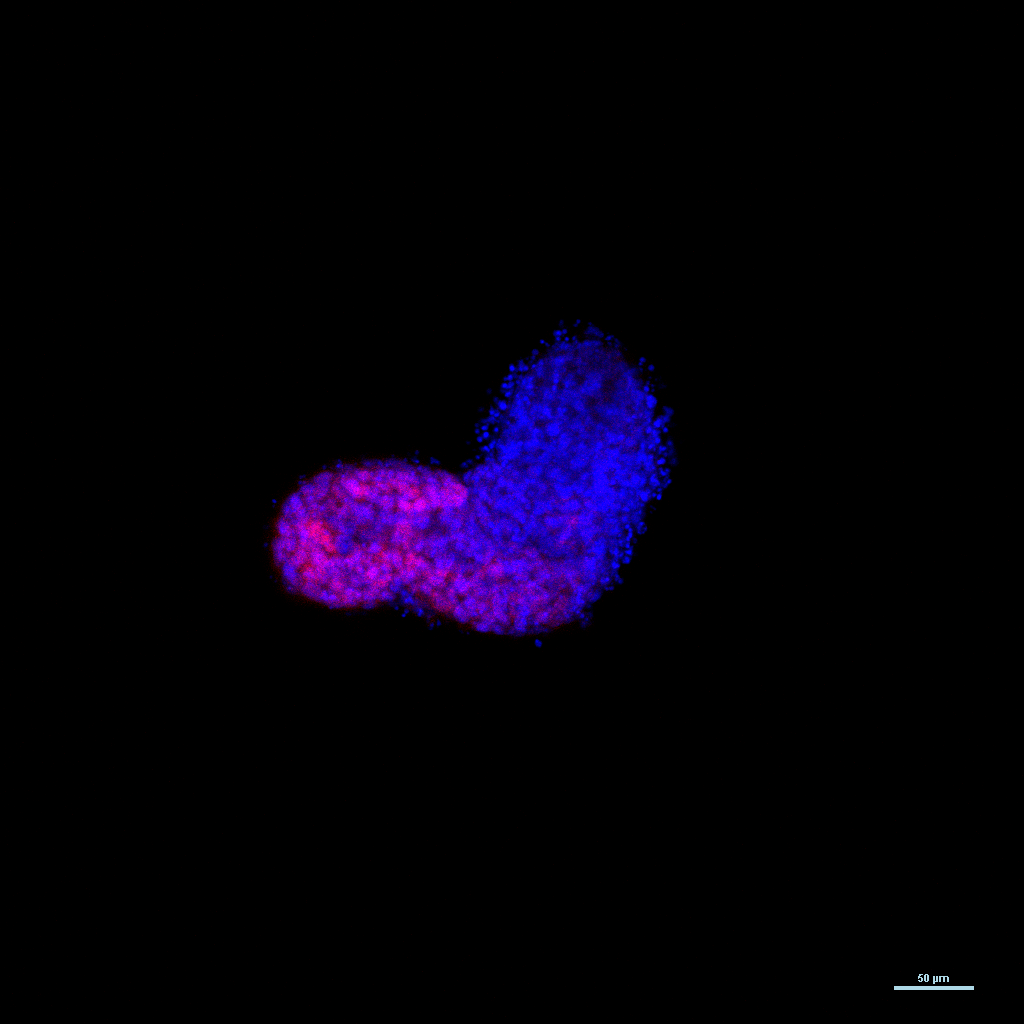

Supplement: Supplementary file 3 — Source data Fig. 1 [file 44318_2025_558_MOESM3_ESM.zip › Figure 1/panel 1F/NT_Cdx2/seq8790_seq8790_RGB.tif]

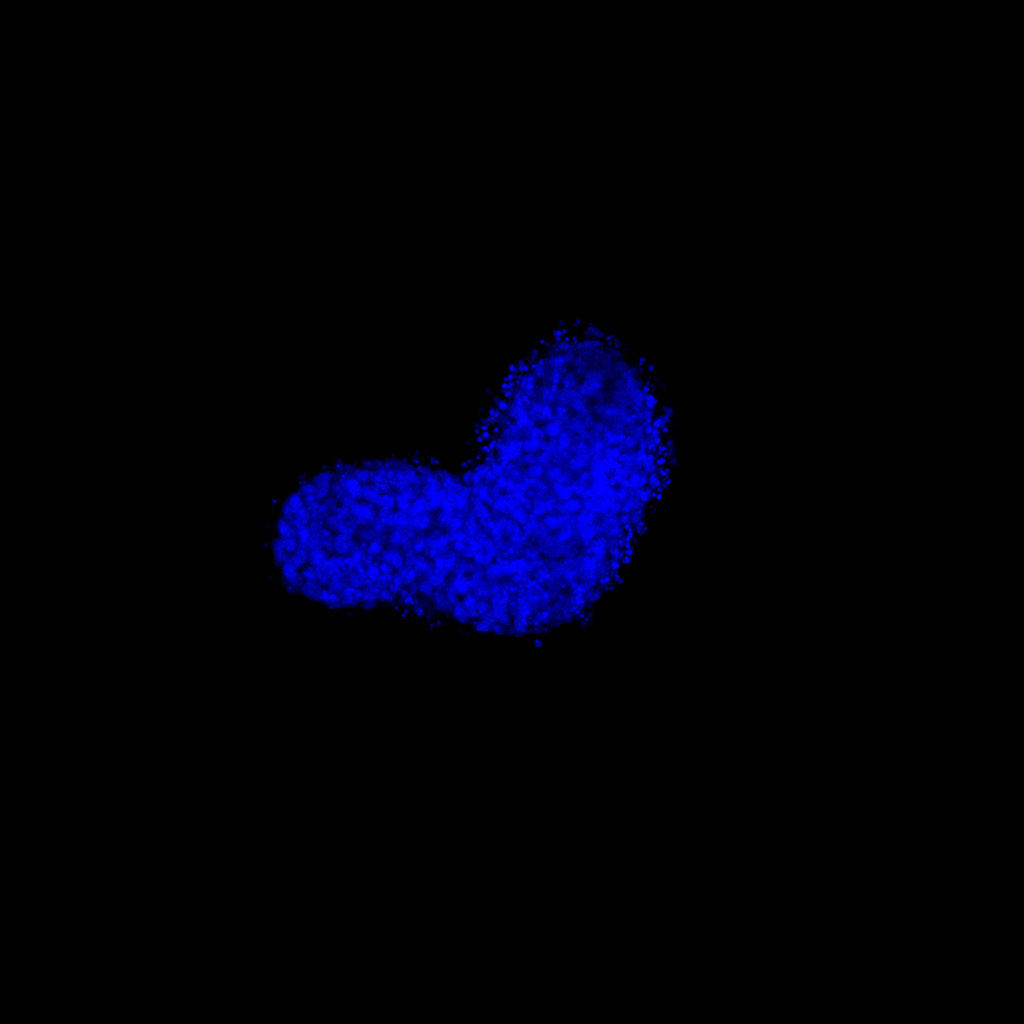

Supplement: Supplementary file 3 — Source data Fig. 1 [file 44318_2025_558_MOESM3_ESM.zip › Figure 1/panel 1F/NT_Cdx2/seq8790_seq8790_RGB_DAPI.tif]

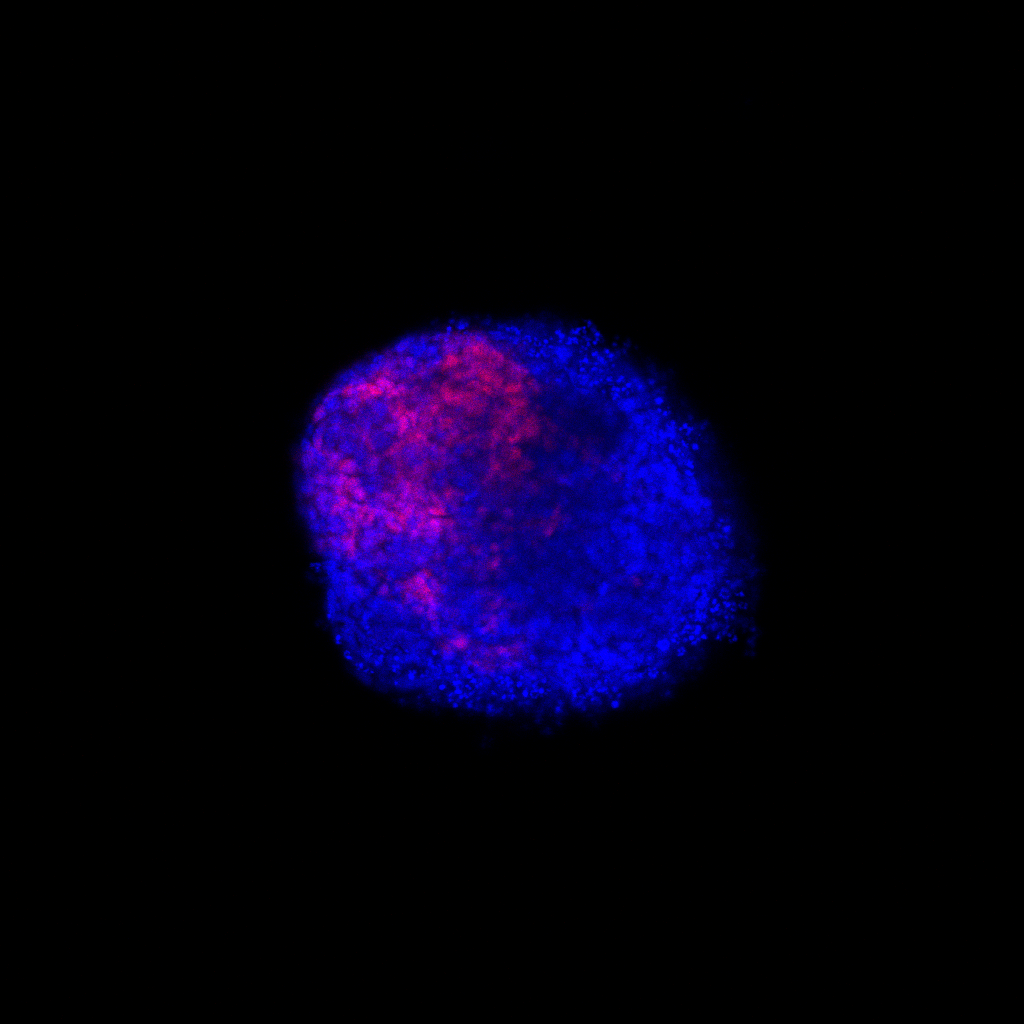

Supplement: Supplementary file 3 — Source data Fig. 1 [file 44318_2025_558_MOESM3_ESM.zip › Figure 1/panel 1F/KD-1_Bra/seq8686.tif]

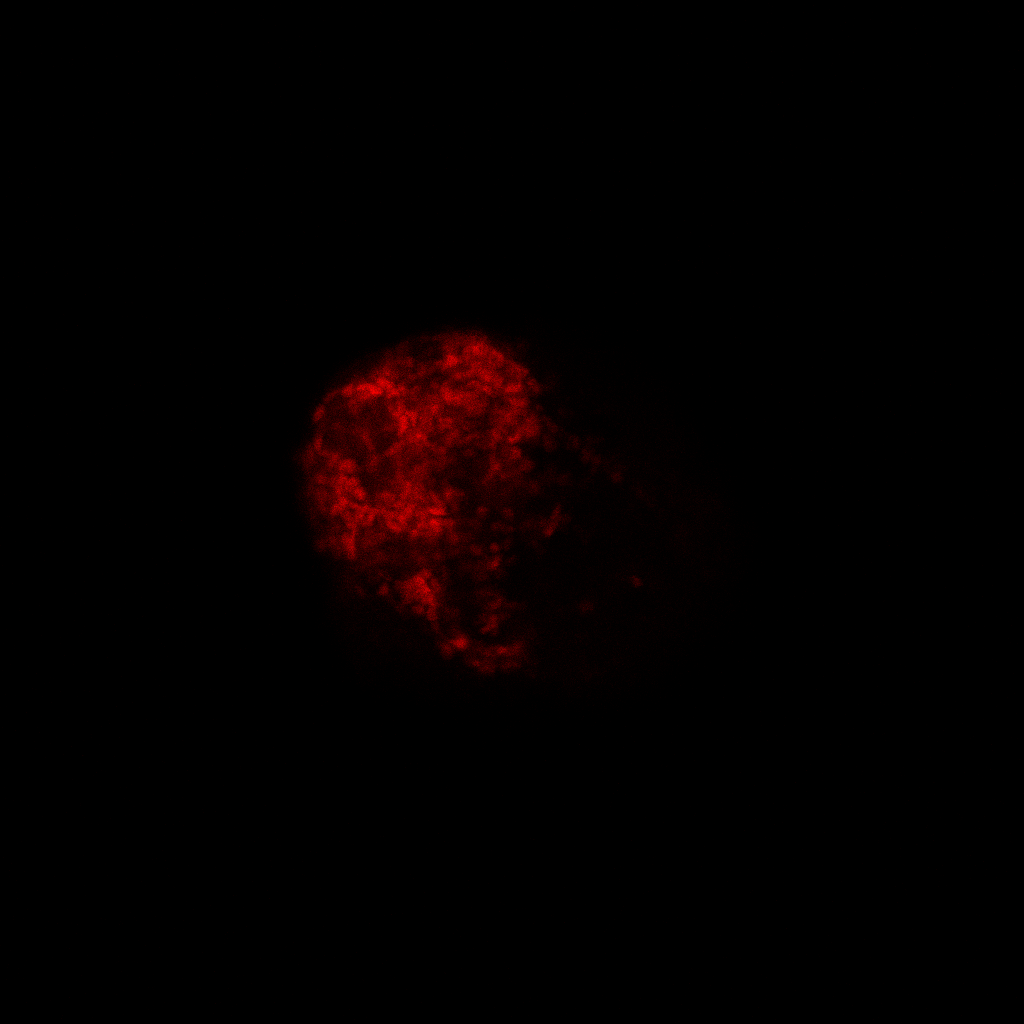

Supplement: Supplementary file 3 — Source data Fig. 1 [file 44318_2025_558_MOESM3_ESM.zip › Figure 1/panel 1F/KD-1_Bra/seq8686c2.tif]

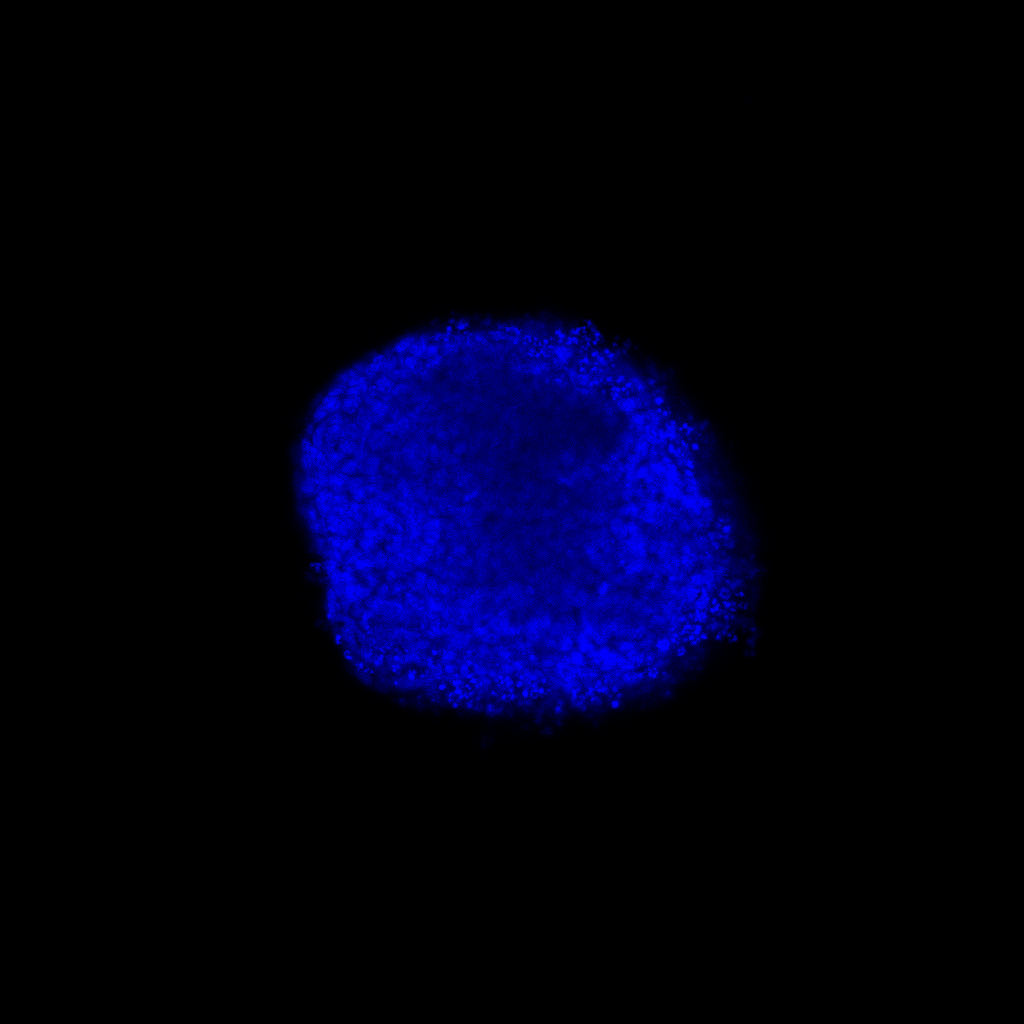

Supplement: Supplementary file 3 — Source data Fig. 1 [file 44318_2025_558_MOESM3_ESM.zip › Figure 1/panel 1F/KD-1_Bra/seq8686c1.tif]

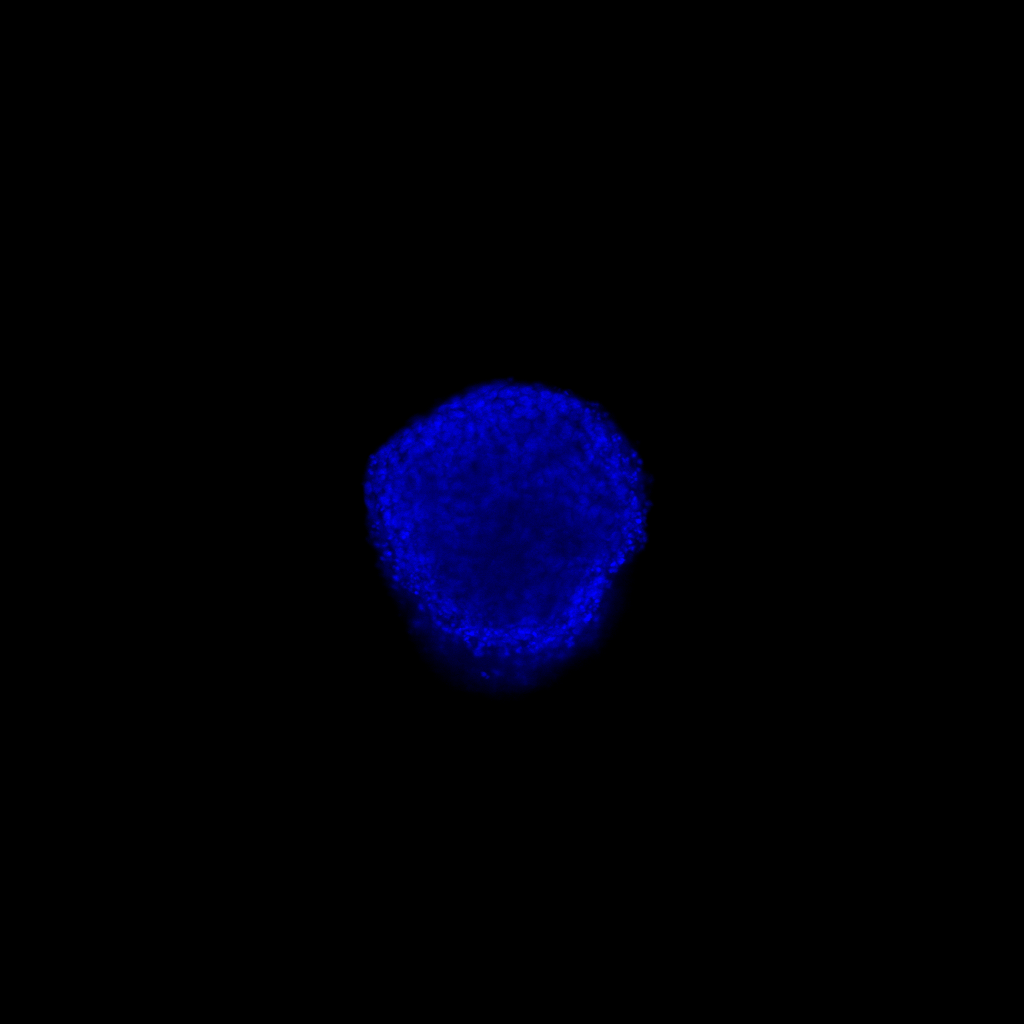

Supplement: Supplementary file 3 — Source data Fig. 1 [file 44318_2025_558_MOESM3_ESM.zip › Figure 1/panel 1F/KD-1_Sox17/image0046_image0046_RGB_DAPI.tif]

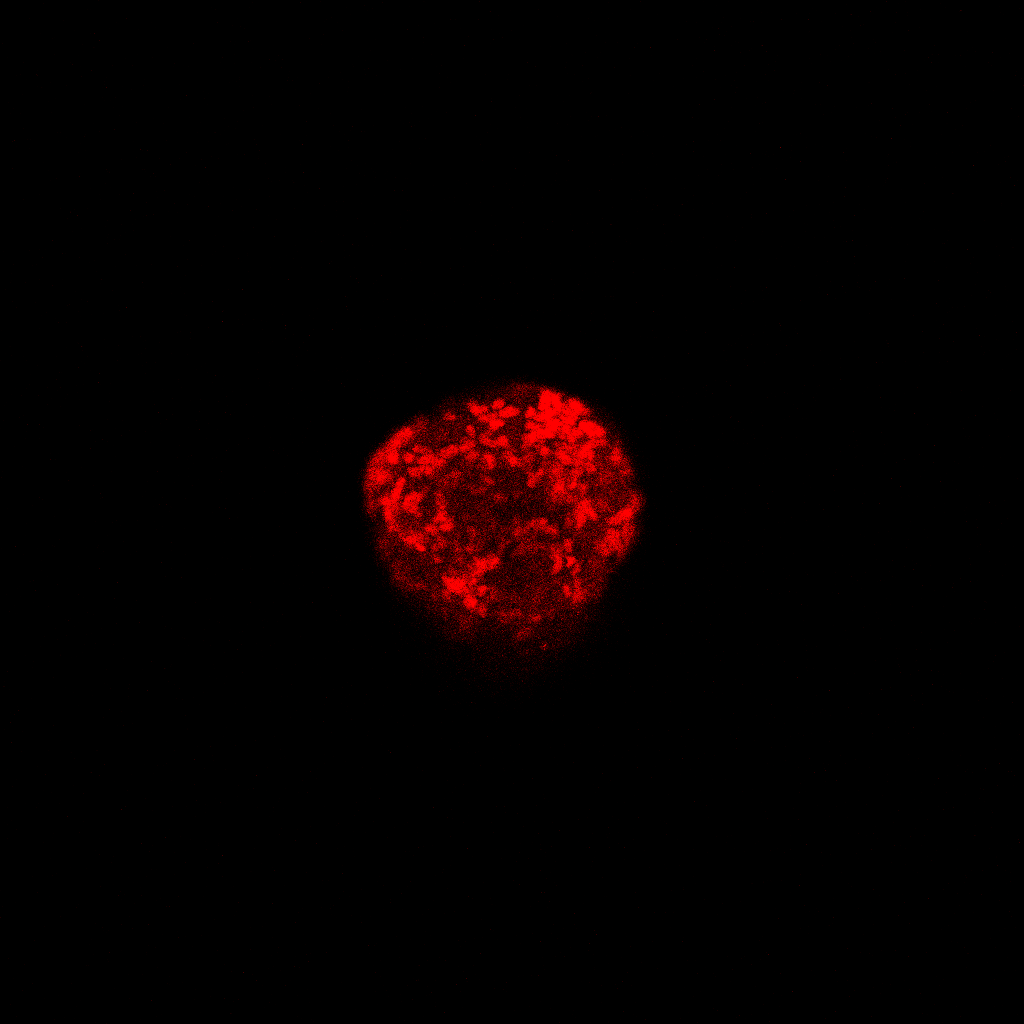

Supplement: Supplementary file 3 — Source data Fig. 1 [file 44318_2025_558_MOESM3_ESM.zip › Figure 1/panel 1F/KD-1_Sox17/image0046_image0046_RGB_Cy3 dyeΓÇôlabeled IgG antibody_pH 7.2.tif]

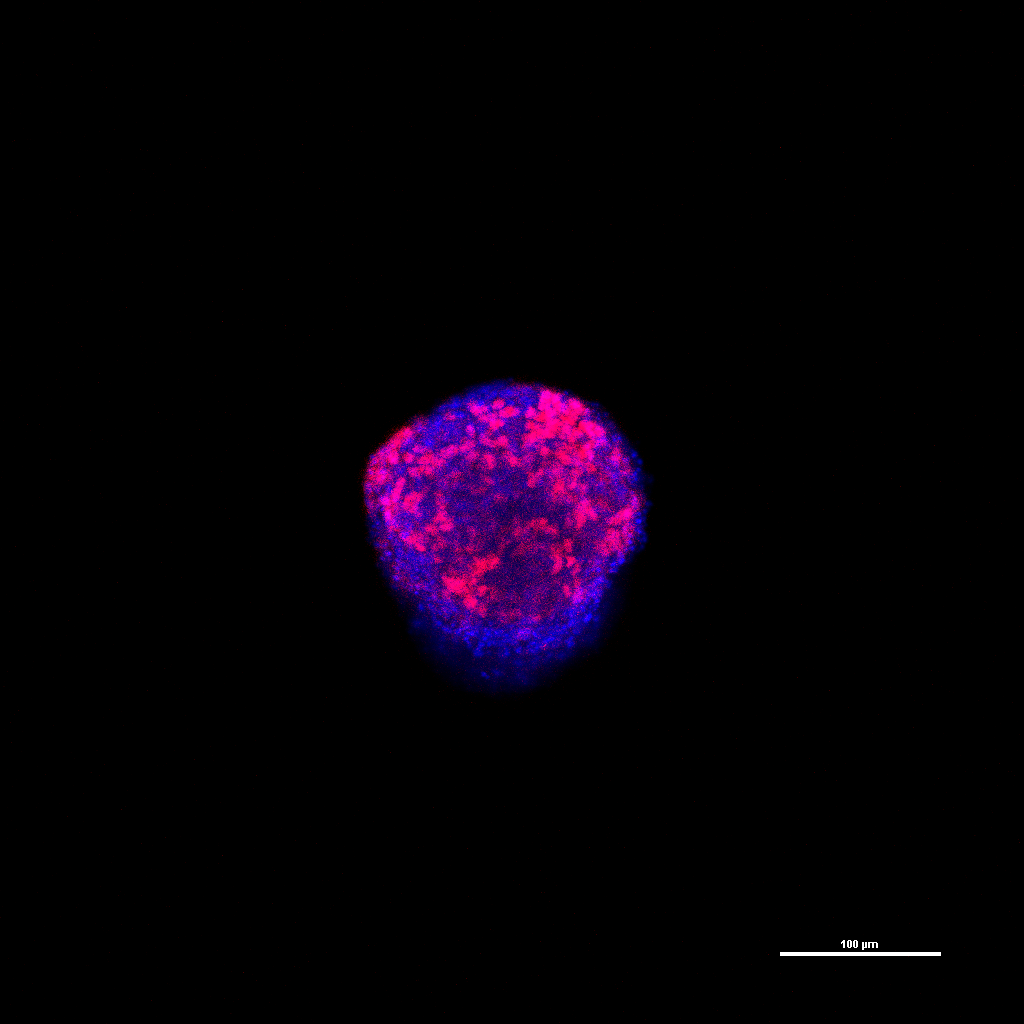

Supplement: Supplementary file 3 — Source data Fig. 1 [file 44318_2025_558_MOESM3_ESM.zip › Figure 1/panel 1F/KD-1_Sox17/image0046_image0046_RGB.tif]

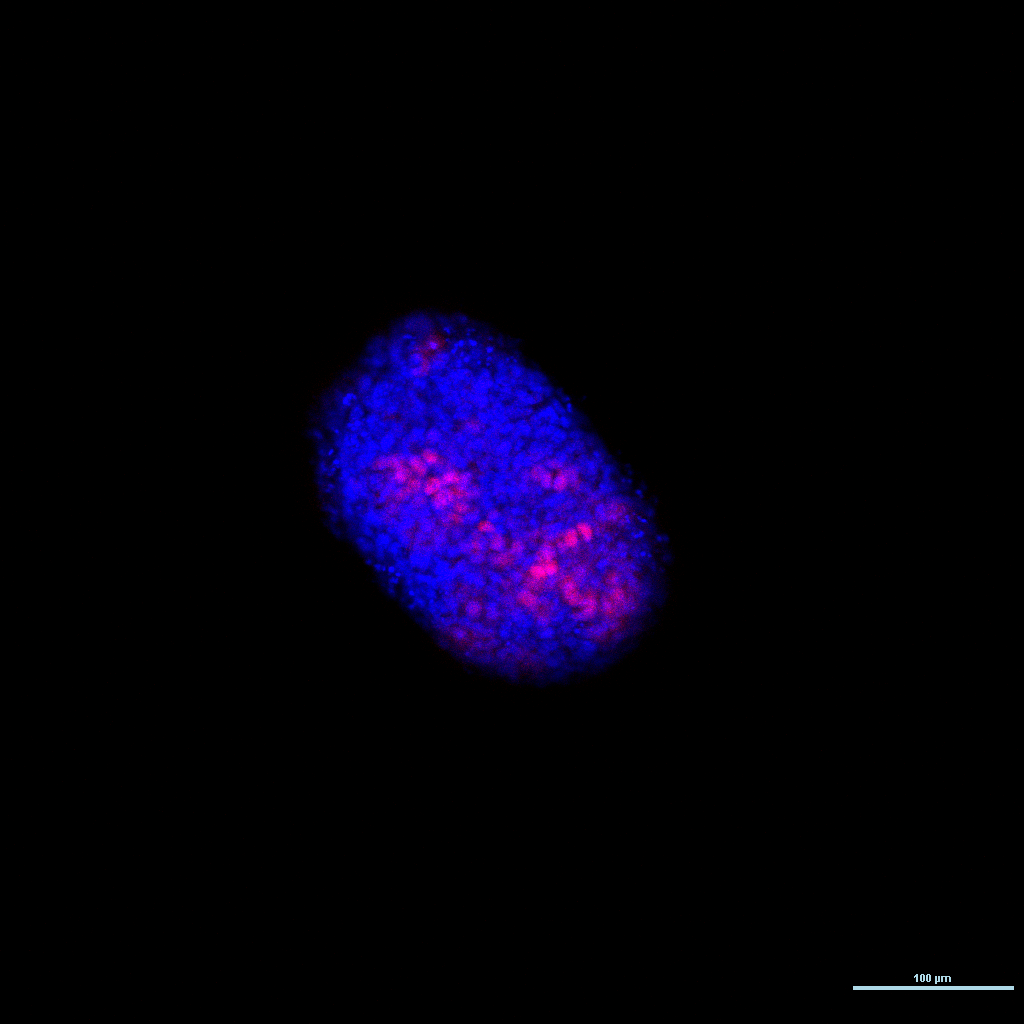

Supplement: Supplementary file 3 — Source data Fig. 1 [file 44318_2025_558_MOESM3_ESM.zip › Figure 1/panel 1F/KD-1_Nanog/seq8801_seq8801_RGB.tif]

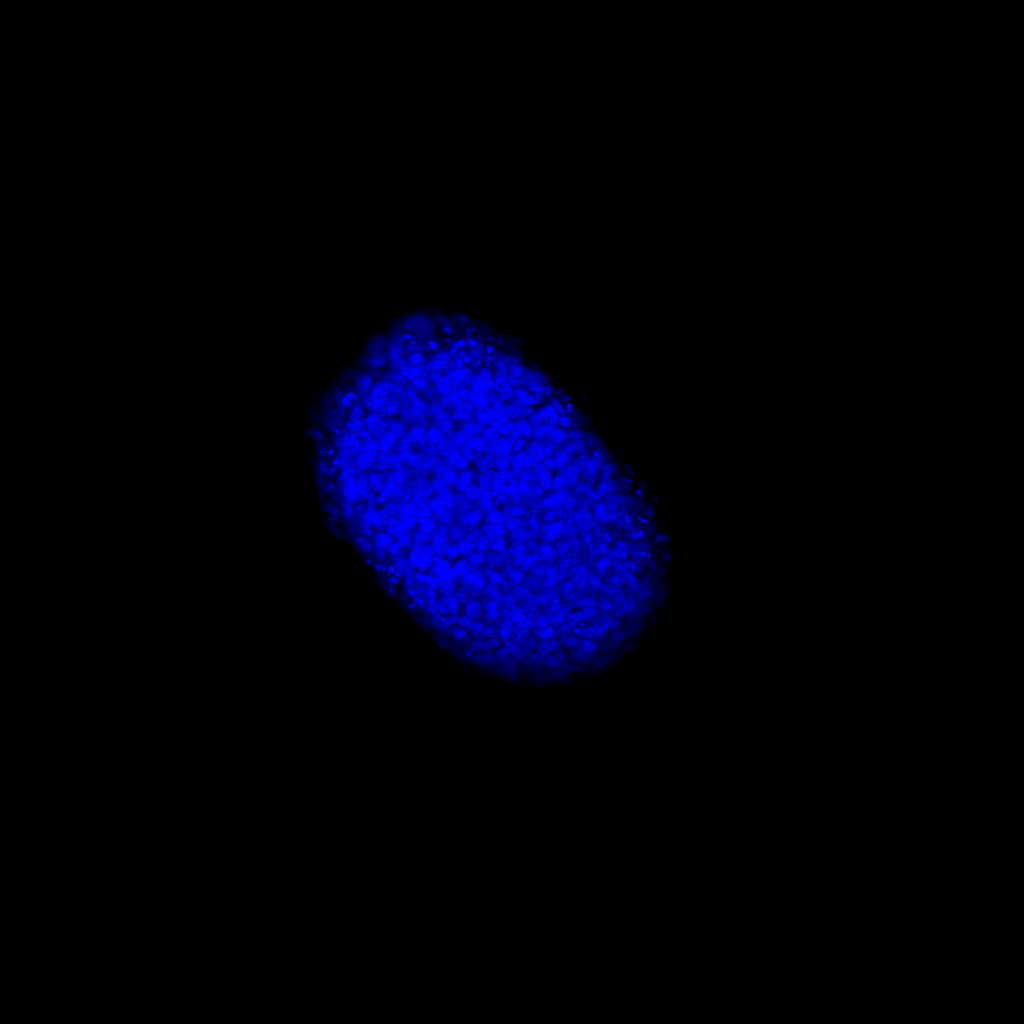

Supplement: Supplementary file 3 — Source data Fig. 1 [file 44318_2025_558_MOESM3_ESM.zip › Figure 1/panel 1F/KD-1_Nanog/seq8801_seq8801_RGB_DAPI.tif]

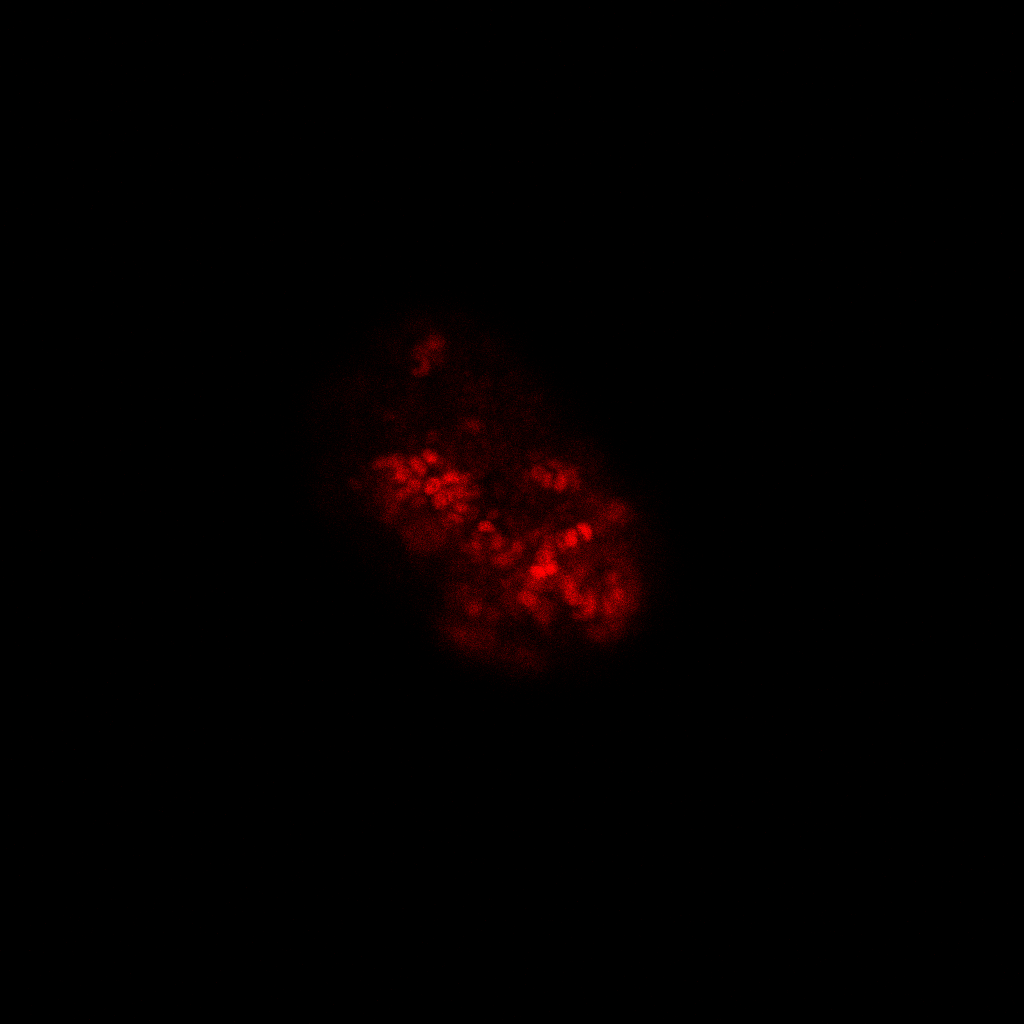

Supplement: Supplementary file 3 — Source data Fig. 1 [file 44318_2025_558_MOESM3_ESM.zip › Figure 1/panel 1F/KD-1_Nanog/seq8801_seq8801_RGB_Texas Red.tif]

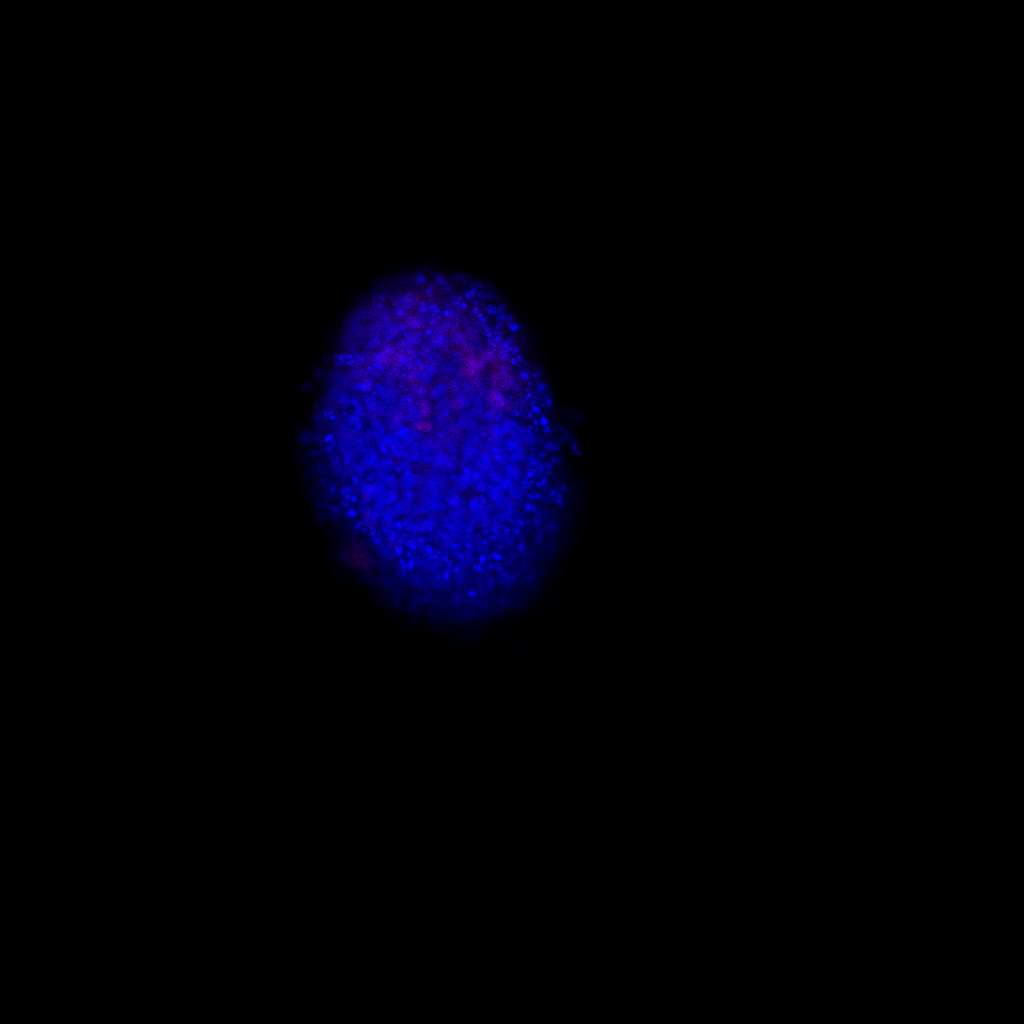

Supplement: Supplementary file 3 — Source data Fig. 1 [file 44318_2025_558_MOESM3_ESM.zip › Figure 1/panel 1F/KD-2_Bra/seq8690.tif]

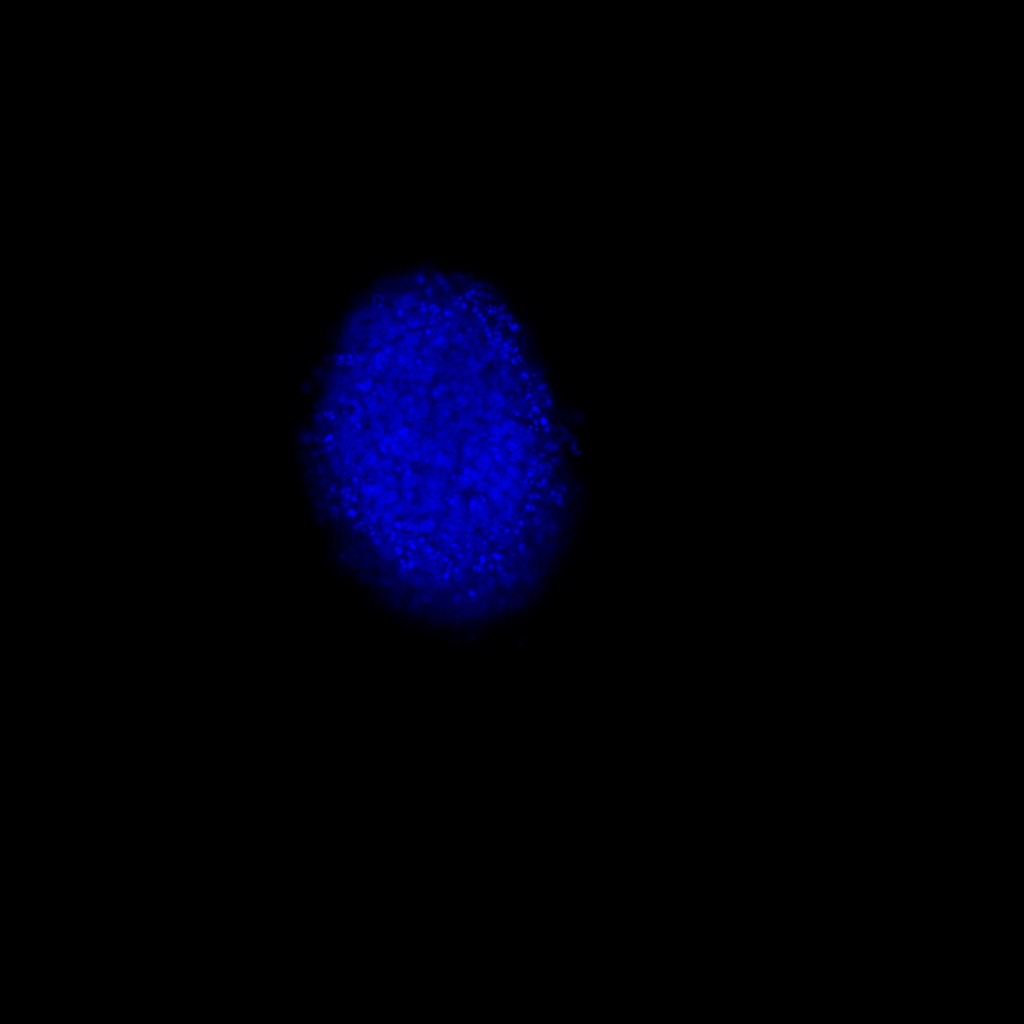

Supplement: Supplementary file 3 — Source data Fig. 1 [file 44318_2025_558_MOESM3_ESM.zip › Figure 1/panel 1F/KD-2_Bra/seq8690c1.tif]

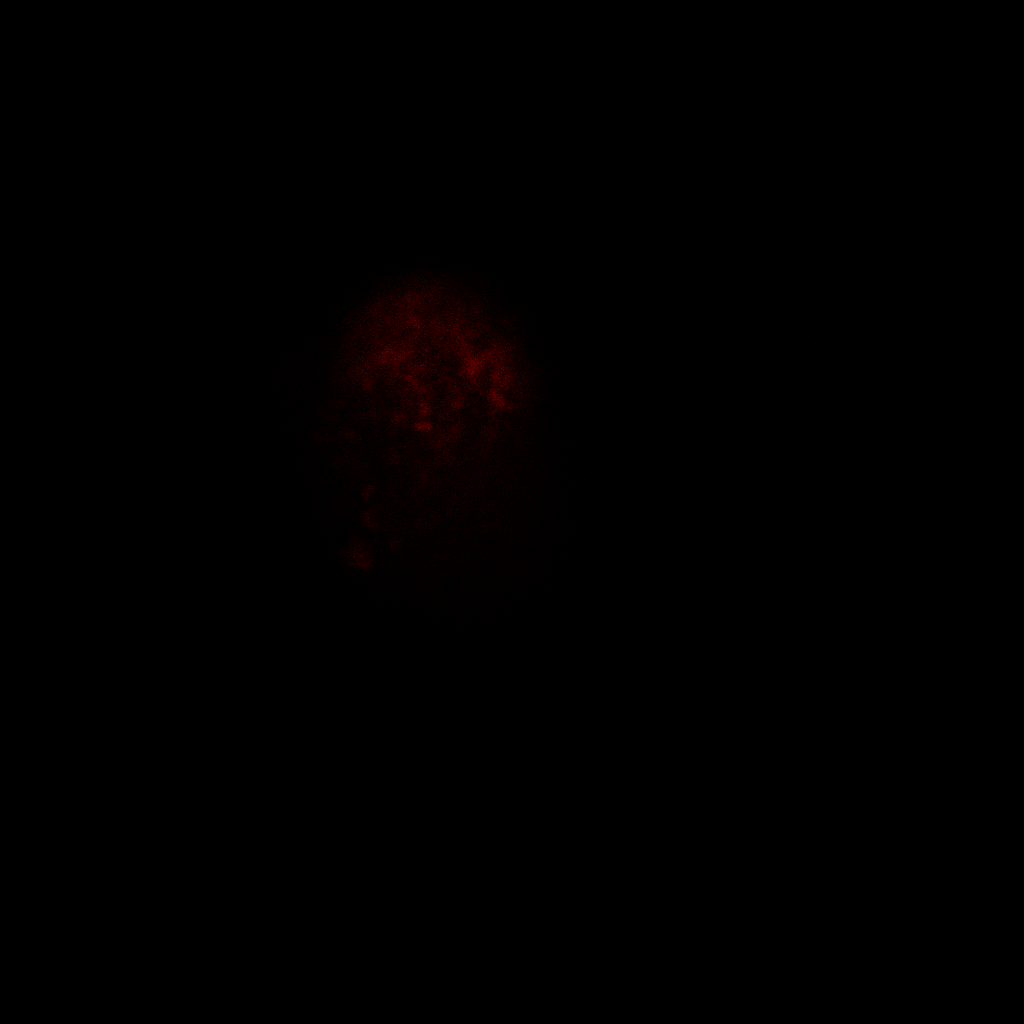

Supplement: Supplementary file 3 — Source data Fig. 1 [file 44318_2025_558_MOESM3_ESM.zip › Figure 1/panel 1F/KD-2_Bra/seq8690c2.tif]

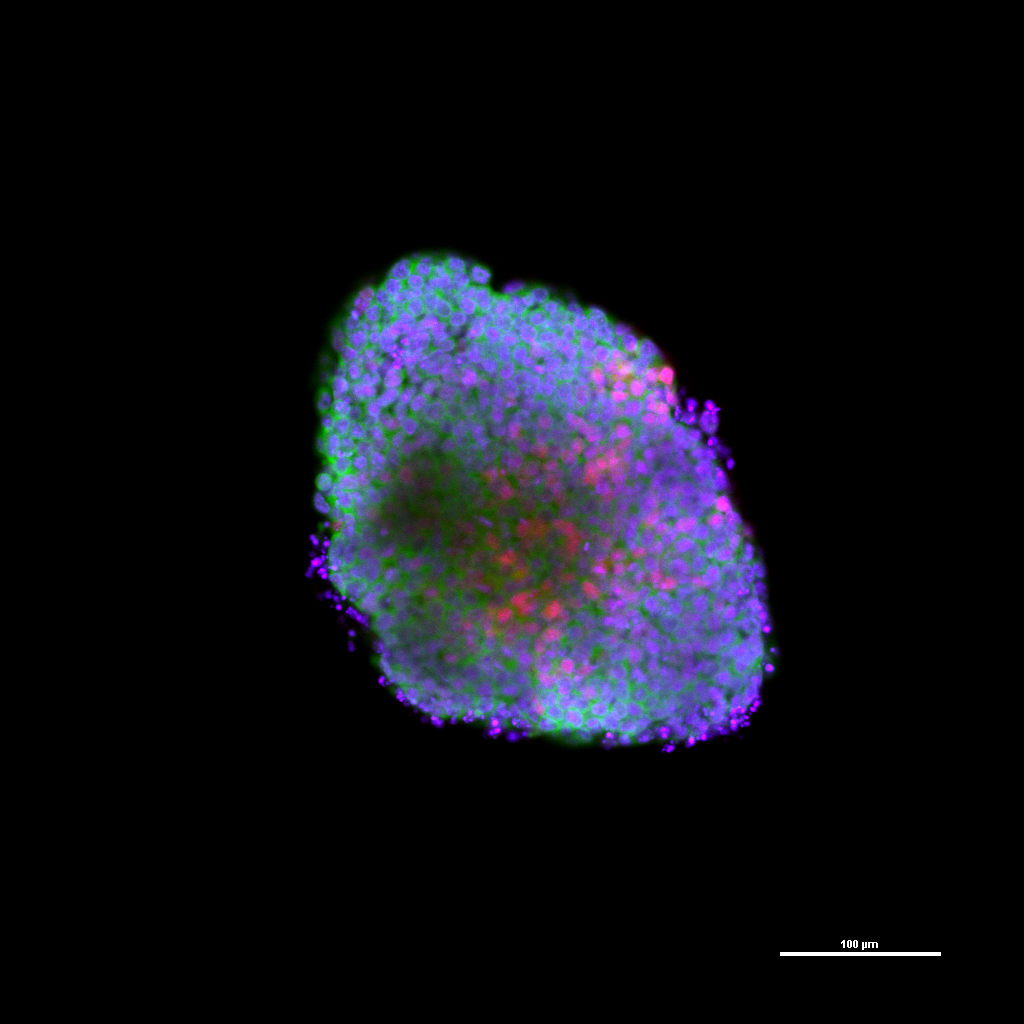

Supplement: Supplementary file 3 — Source data Fig. 1 [file 44318_2025_558_MOESM3_ESM.zip › Figure 1/panel 1F/KD-2_Bra:e-cadh/seq11061_seq11061_RGB.tif]

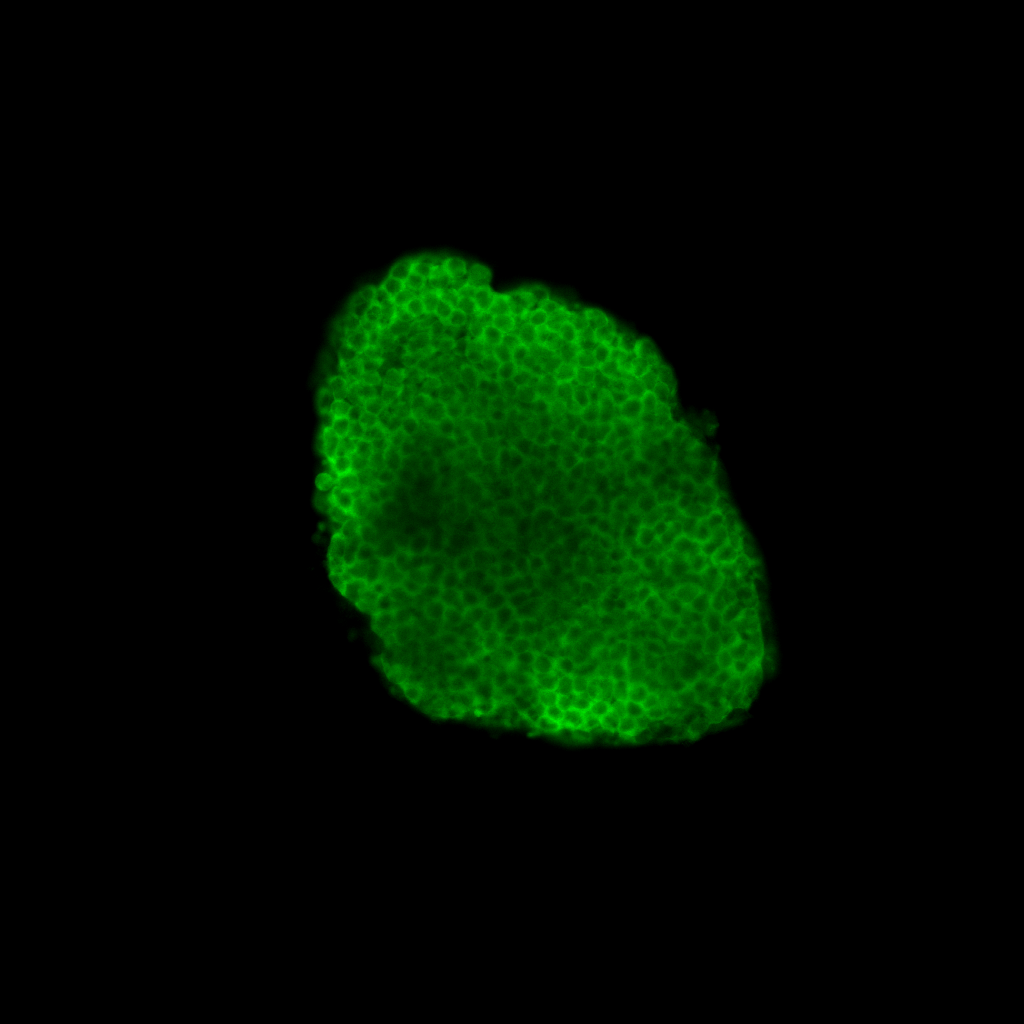

Supplement: Supplementary file 3 — Source data Fig. 1 [file 44318_2025_558_MOESM3_ESM.zip › Figure 1/panel 1F/KD-2_Bra:e-cadh/seq11061_seq11061_RGB_FITC.tif]

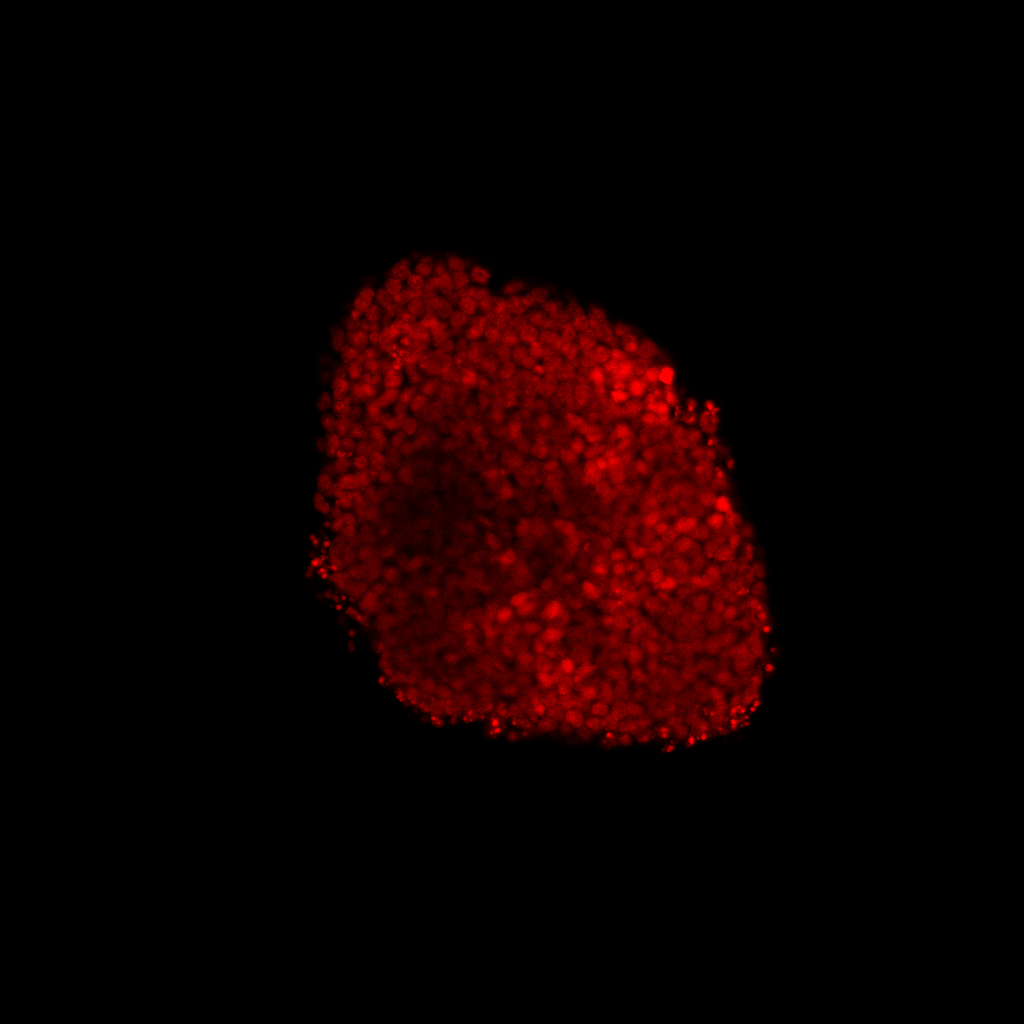

Supplement: Supplementary file 3 — Source data Fig. 1 [file 44318_2025_558_MOESM3_ESM.zip › Figure 1/panel 1F/KD-2_Bra:e-cadh/seq11061_seq11061_RGB_TRITC.tif]

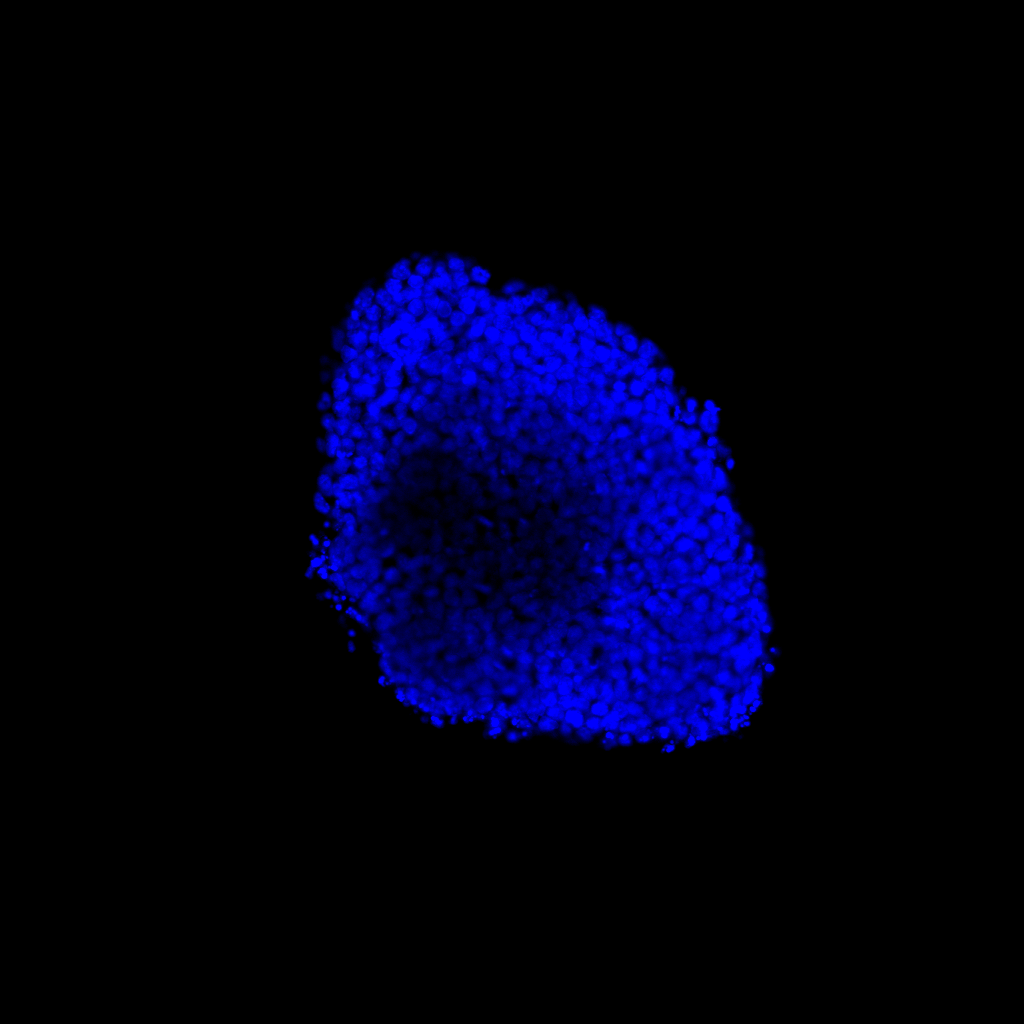

Supplement: Supplementary file 3 — Source data Fig. 1 [file 44318_2025_558_MOESM3_ESM.zip › Figure 1/panel 1F/KD-2_Bra:e-cadh/seq11061_seq11061_RGB_DAPI.tif]

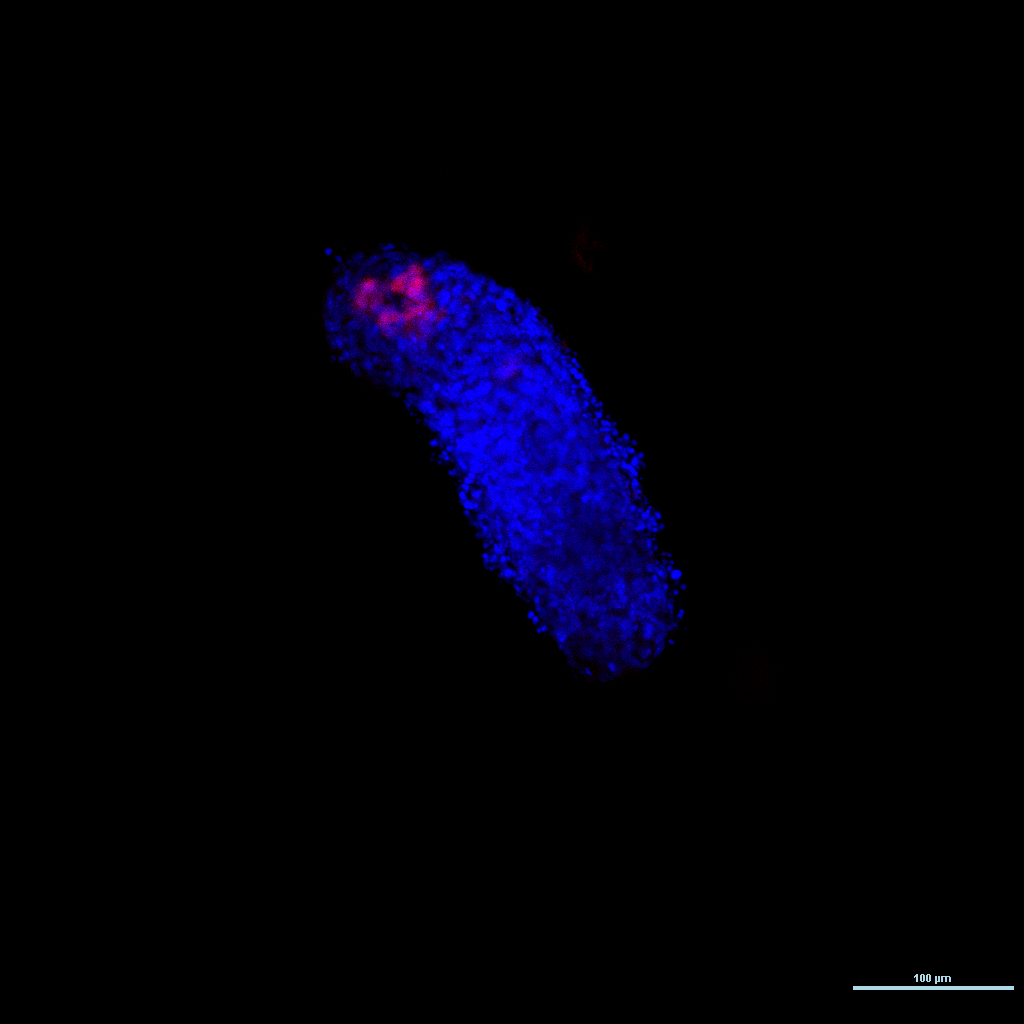

Supplement: Supplementary file 3 — Source data Fig. 1 [file 44318_2025_558_MOESM3_ESM.zip › Figure 1/panel 1F/NT_Nanog/seq8799_seq8799_RGB.tif]

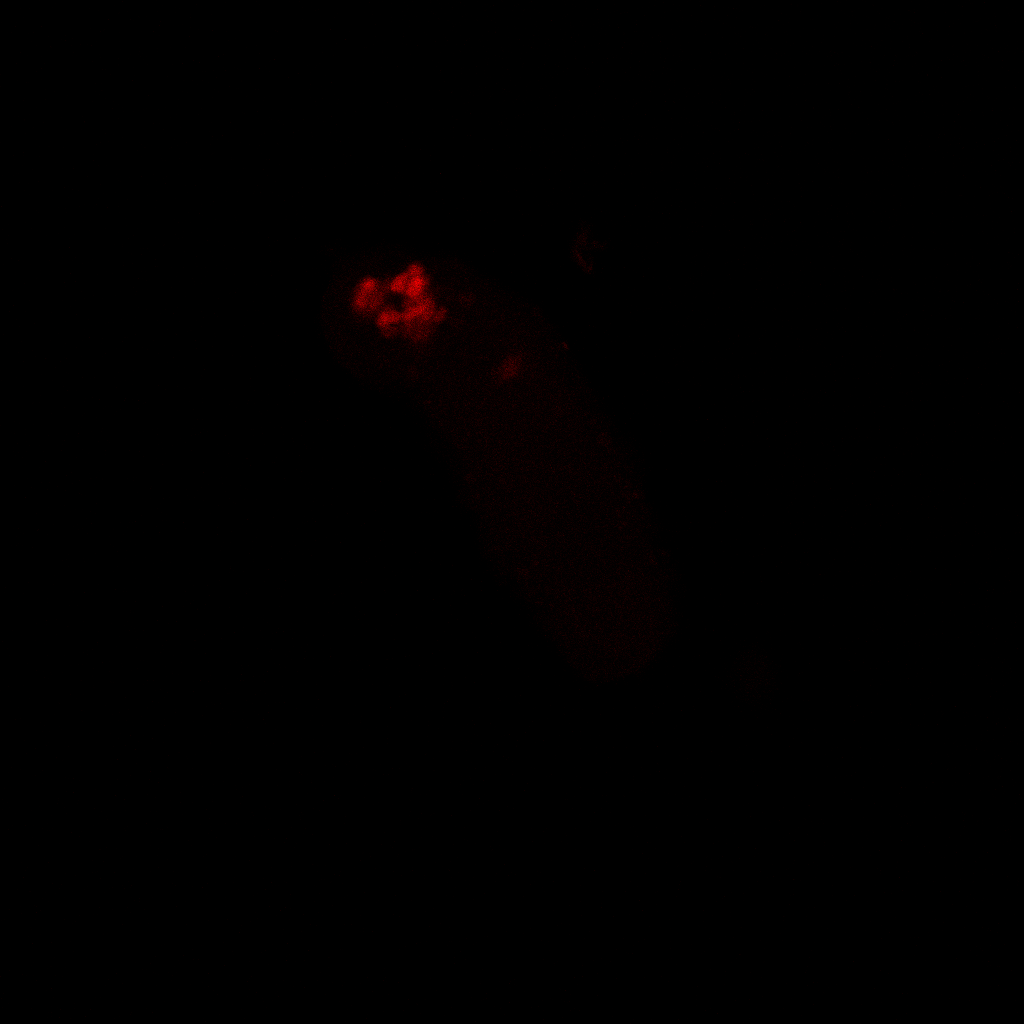

Supplement: Supplementary file 3 — Source data Fig. 1 [file 44318_2025_558_MOESM3_ESM.zip › Figure 1/panel 1F/NT_Nanog/seq8799_seq8799_RGB_Texas Red.tif]

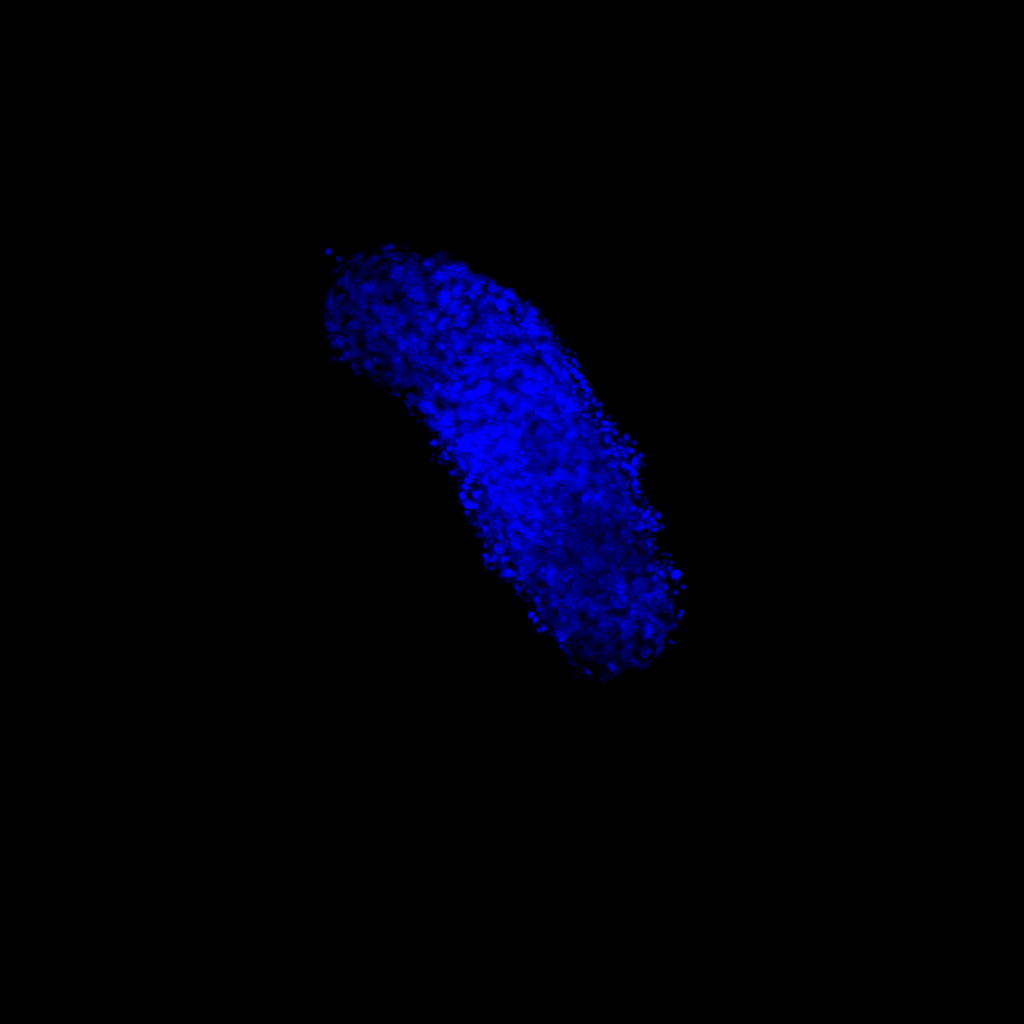

Supplement: Supplementary file 3 — Source data Fig. 1 [file 44318_2025_558_MOESM3_ESM.zip › Figure 1/panel 1F/NT_Nanog/seq8799_seq8799_RGB_DAPI.tif]

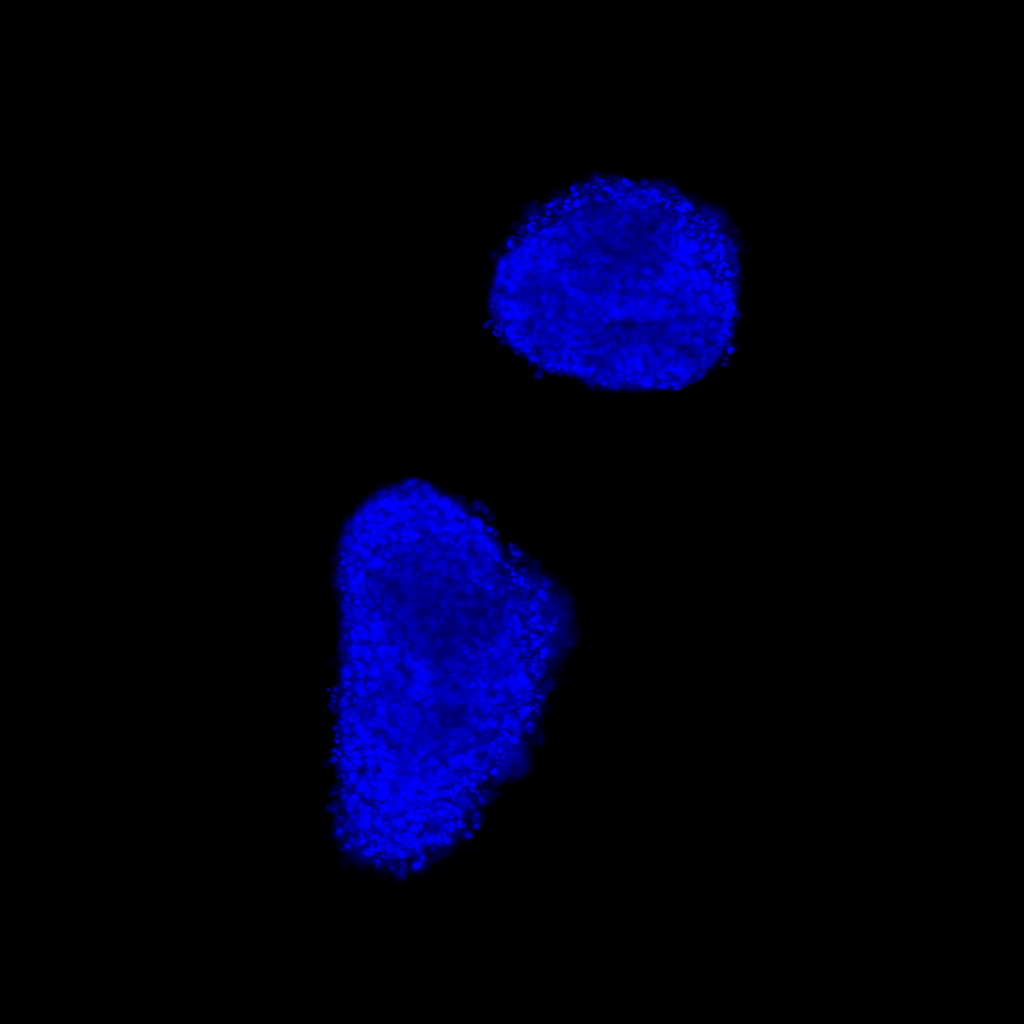

Supplement: Supplementary file 3 — Source data Fig. 1 [file 44318_2025_558_MOESM3_ESM.zip › Figure 1/panel 1F/KD-2_Sox2/image0036_image0036_RGB_DAPI.tif]

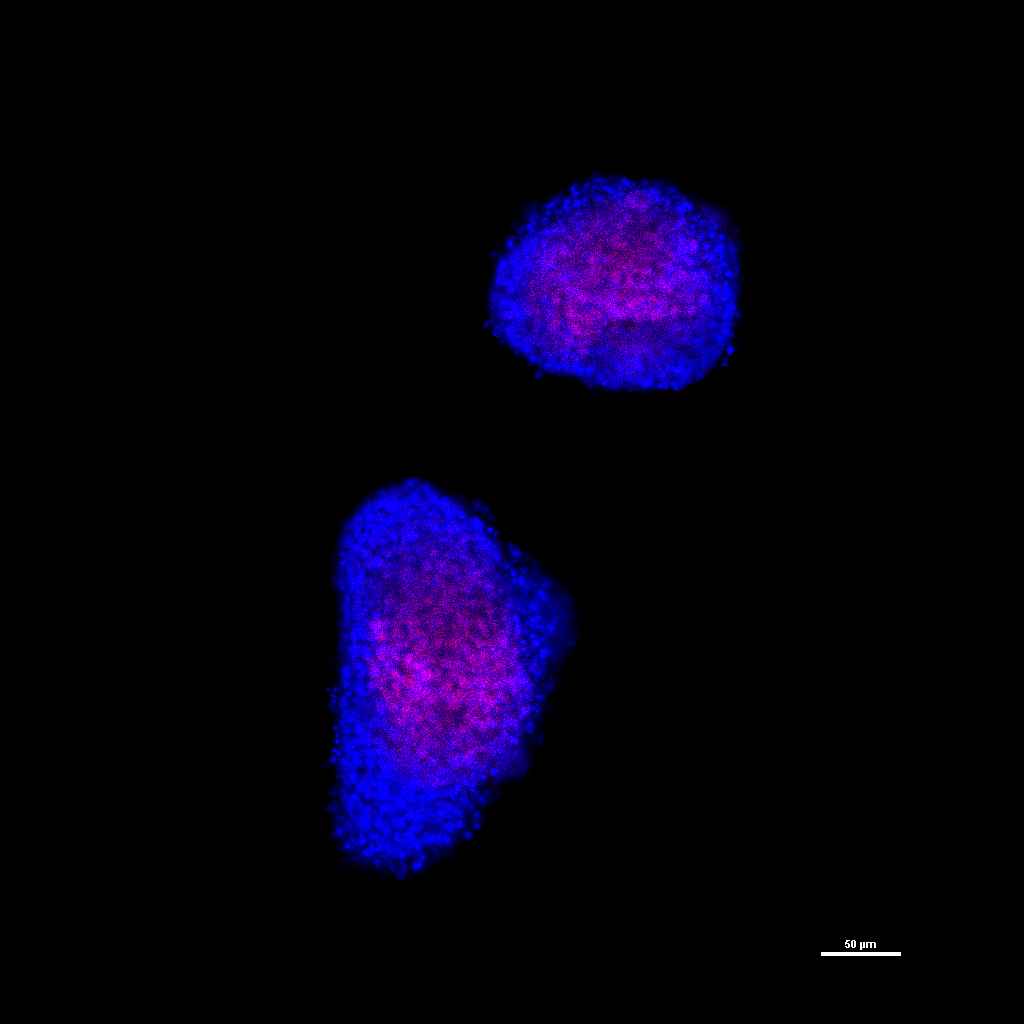

Supplement: Supplementary file 3 — Source data Fig. 1 [file 44318_2025_558_MOESM3_ESM.zip › Figure 1/panel 1F/KD-2_Sox2/image0036_image0036_RGB.tif]

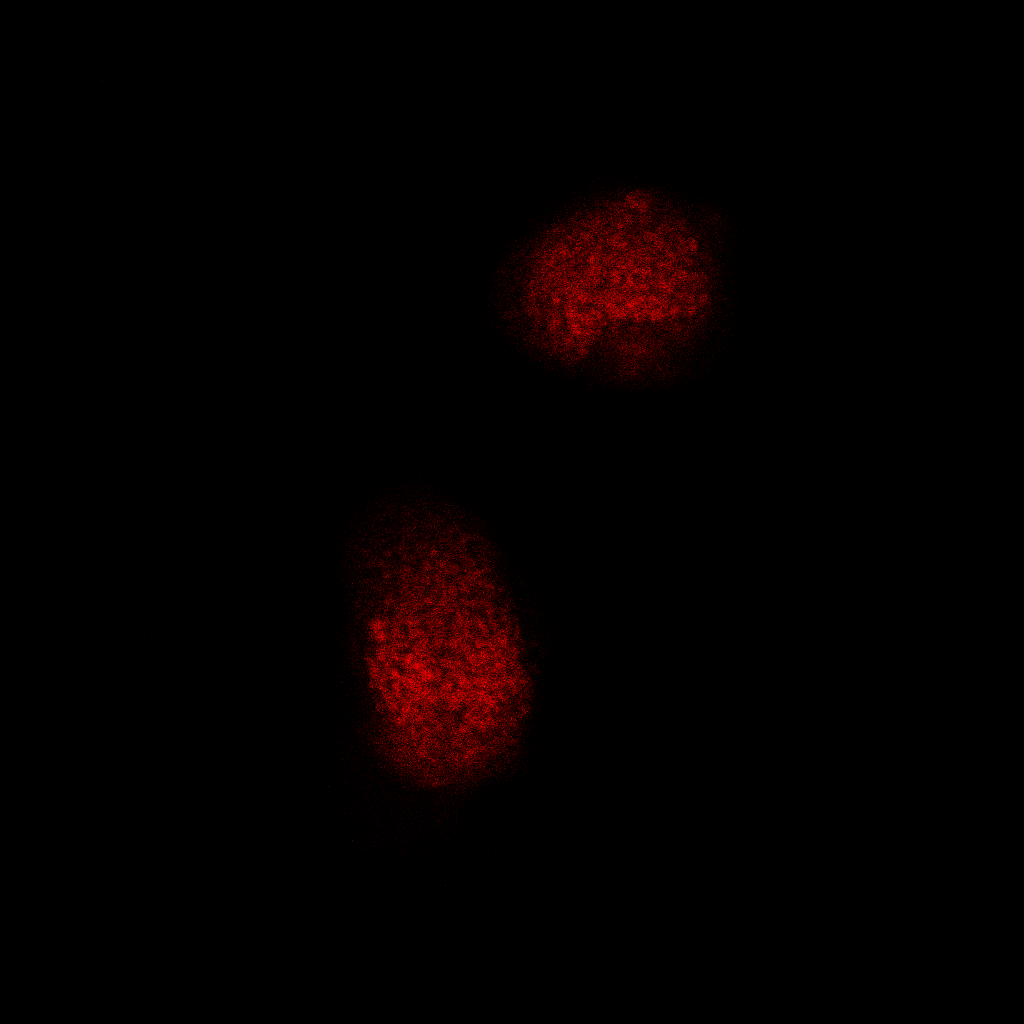

Supplement: Supplementary file 3 — Source data Fig. 1 [file 44318_2025_558_MOESM3_ESM.zip › Figure 1/panel 1F/KD-2_Sox2/image0036_image0036_RGB_Cy3 dyeΓÇôlabeled IgG antibody_pH 7.2.tif]

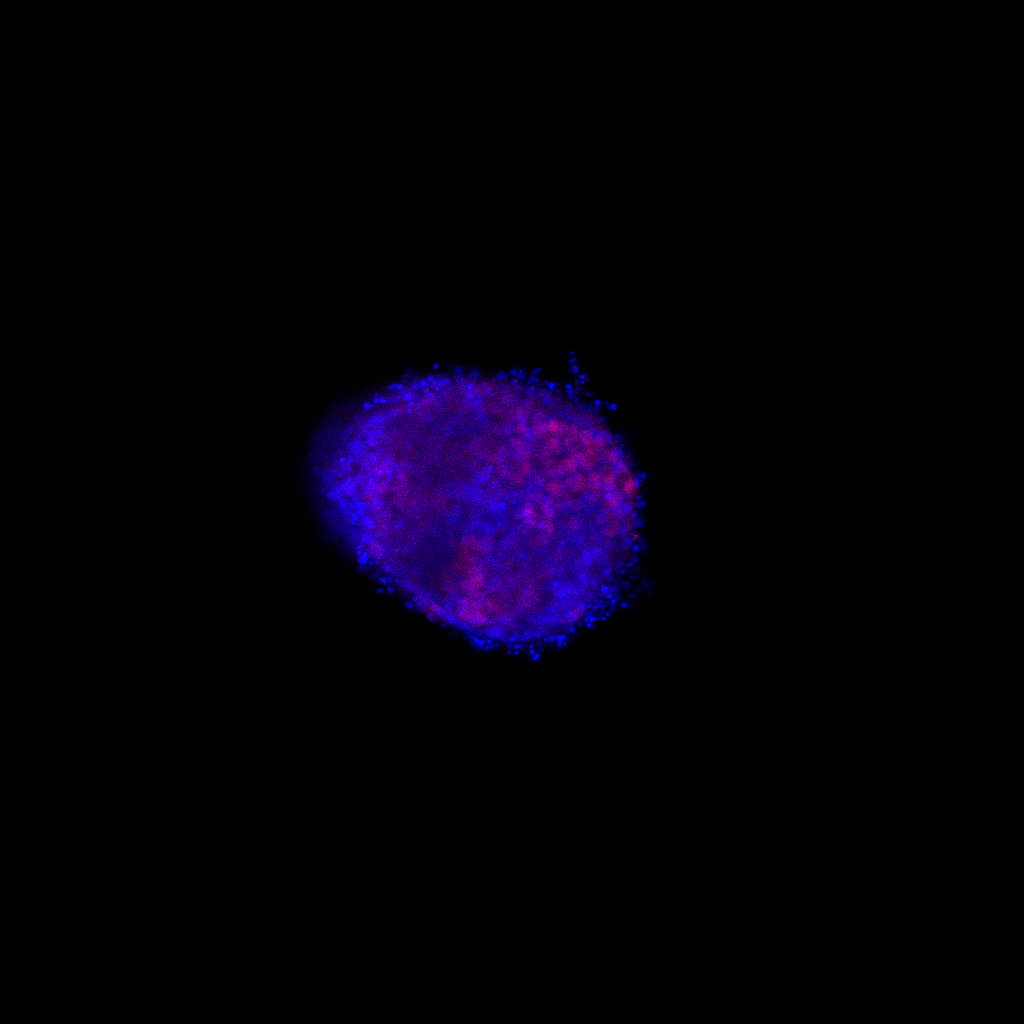

Supplement: Supplementary file 3 — Source data Fig. 1 [file 44318_2025_558_MOESM3_ESM.zip › Figure 1/panel 1F/KD-2_Oct4/seq8696.tif]

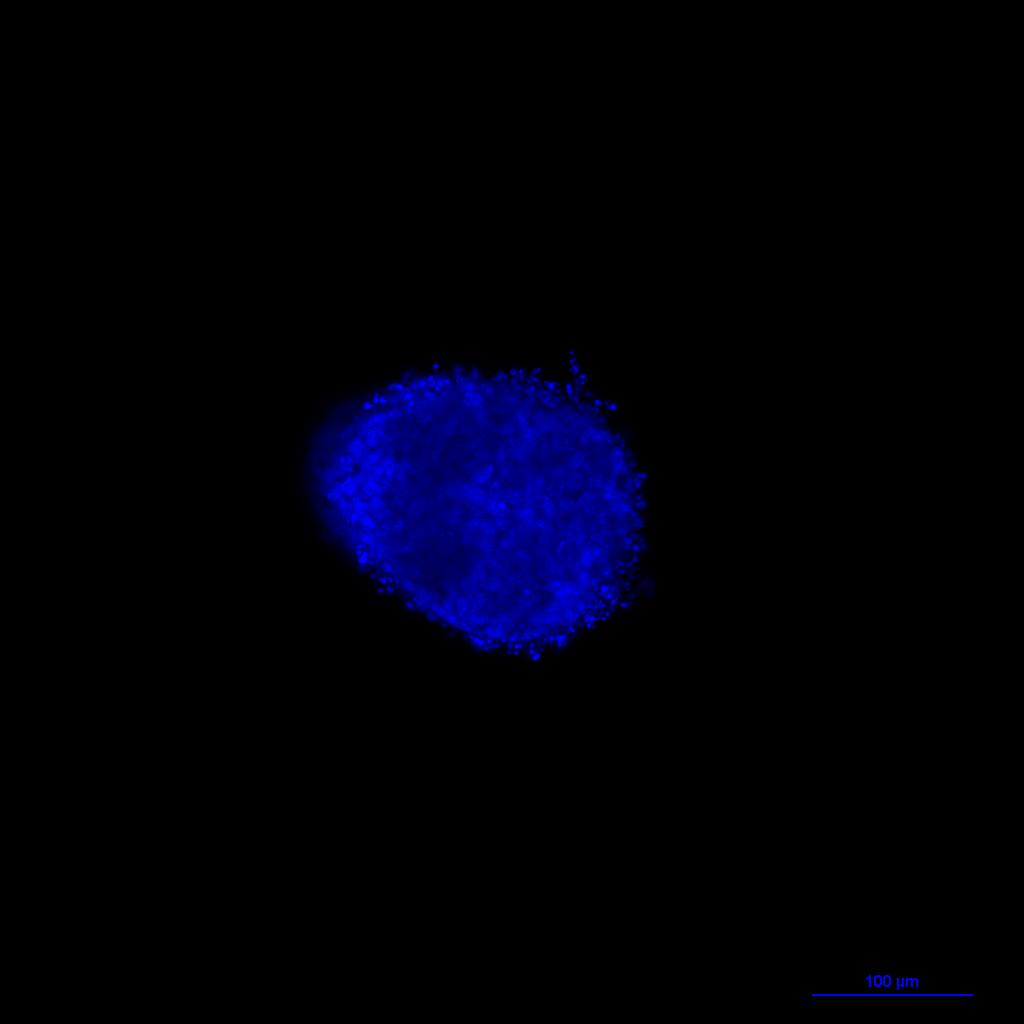

Supplement: Supplementary file 3 — Source data Fig. 1 [file 44318_2025_558_MOESM3_ESM.zip › Figure 1/panel 1F/KD-2_Oct4/seq8696c1.tif]

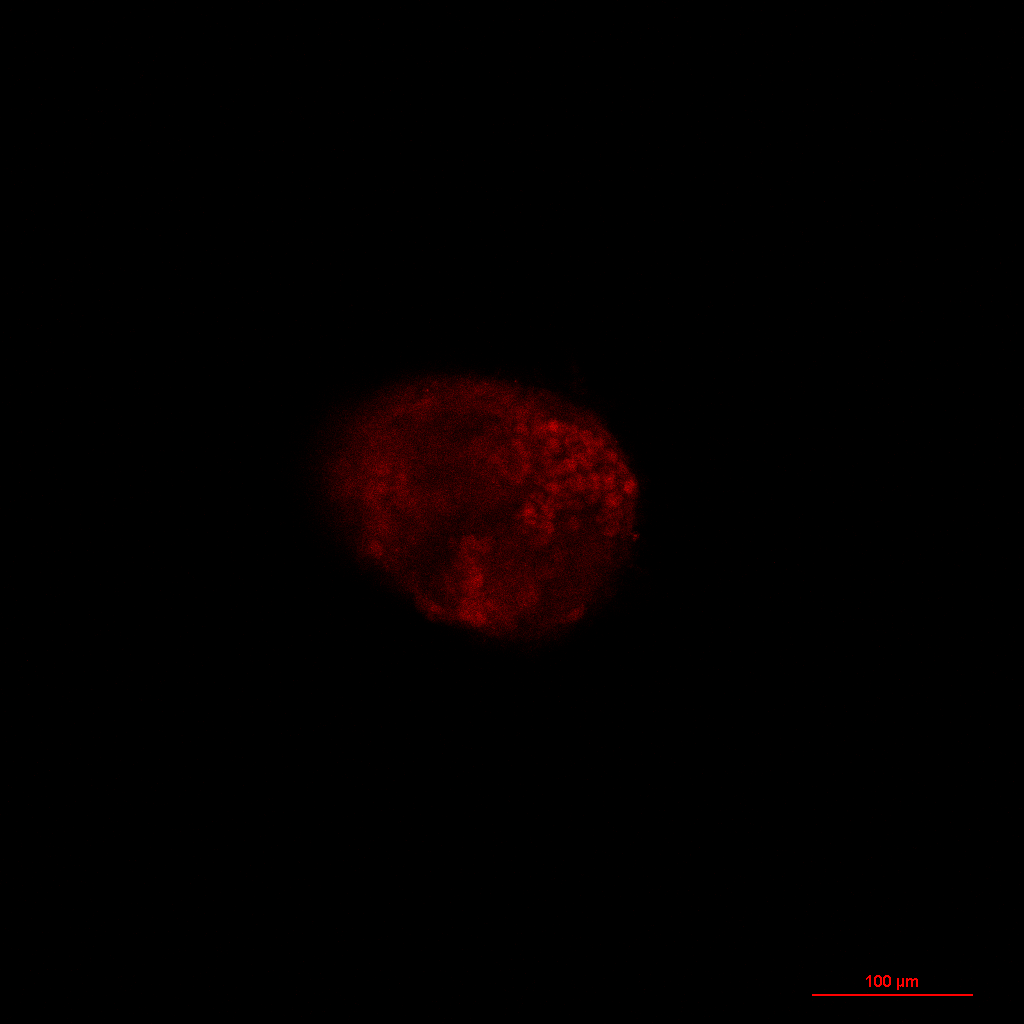

Supplement: Supplementary file 3 — Source data Fig. 1 [file 44318_2025_558_MOESM3_ESM.zip › Figure 1/panel 1F/KD-2_Oct4/seq8696c3.tif]

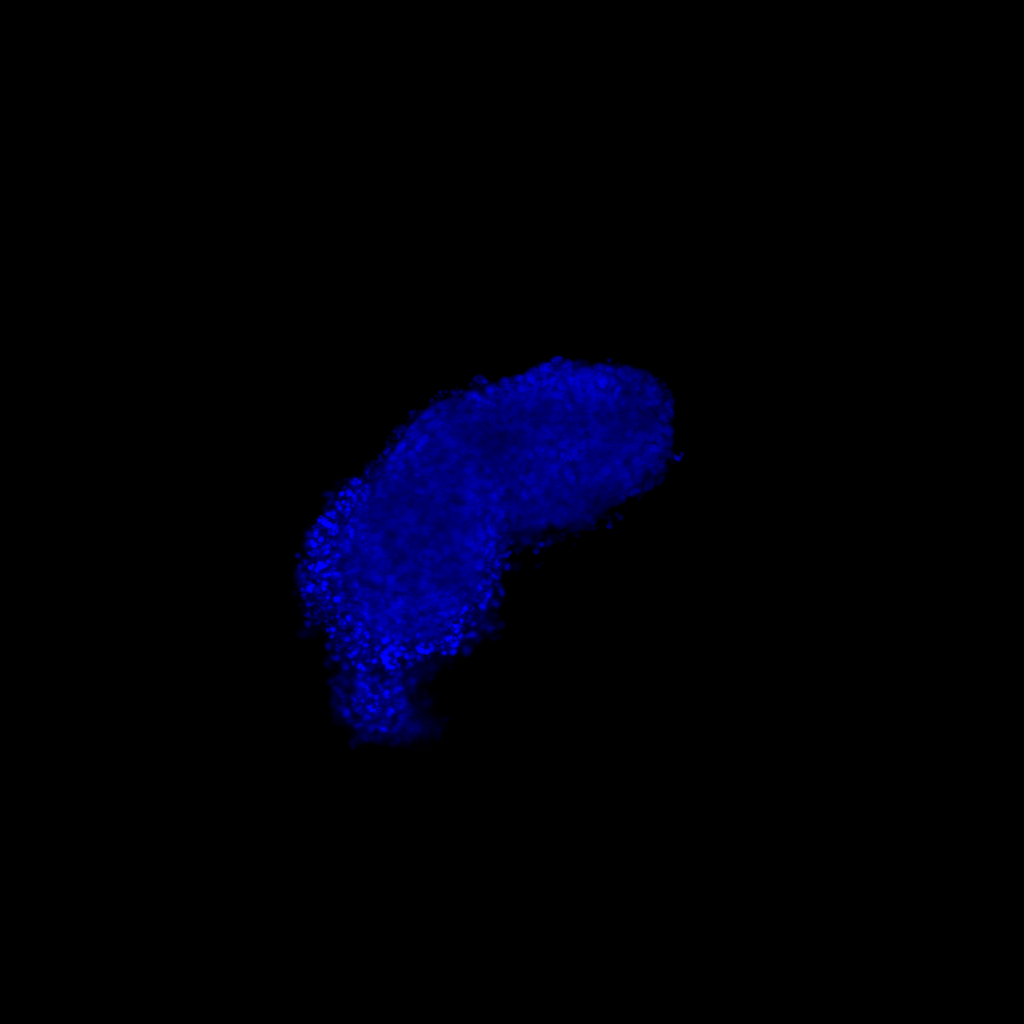

Supplement: Supplementary file 3 — Source data Fig. 1 [file 44318_2025_558_MOESM3_ESM.zip › Figure 1/panel 1F/NT_Sox17/image0040_image0040_RGB_DAPI.tif]

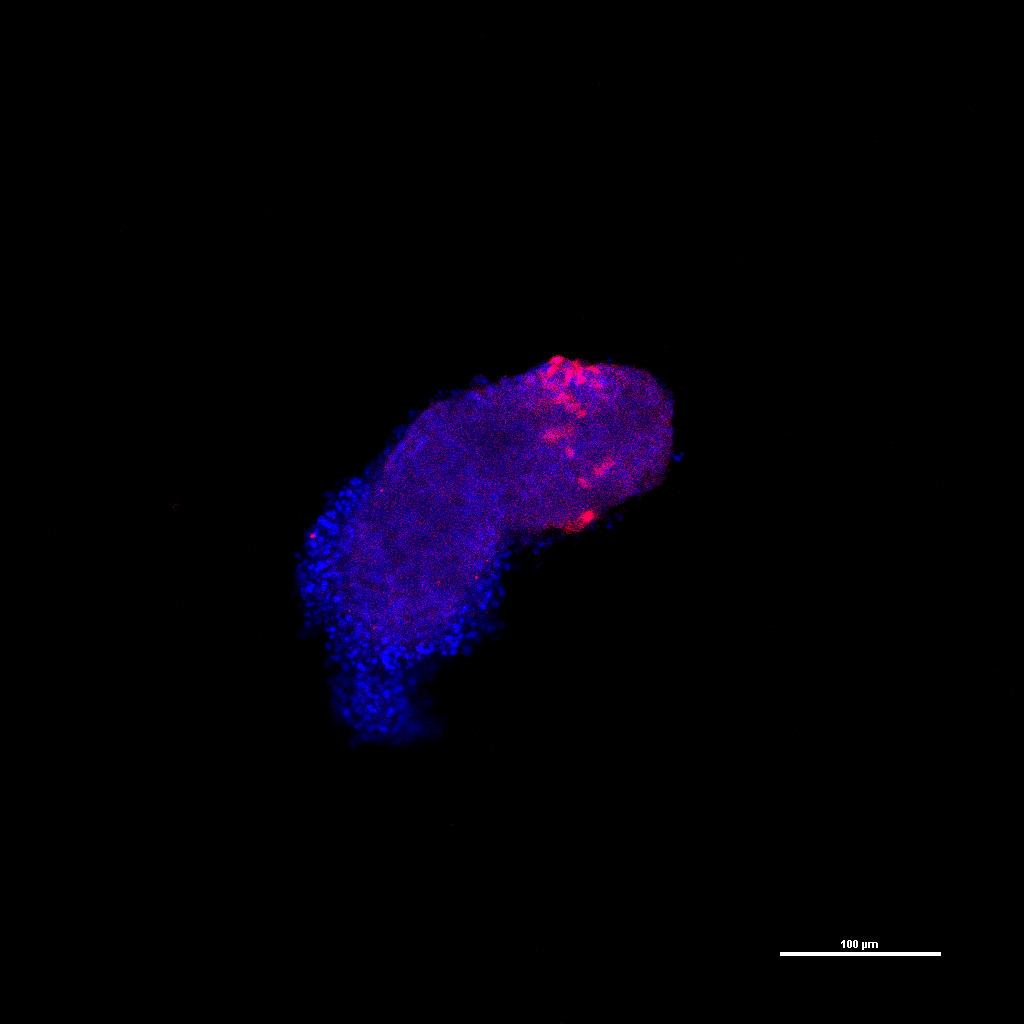

Supplement: Supplementary file 3 — Source data Fig. 1 [file 44318_2025_558_MOESM3_ESM.zip › Figure 1/panel 1F/NT_Sox17/image0040_image0040_RGB.tif]

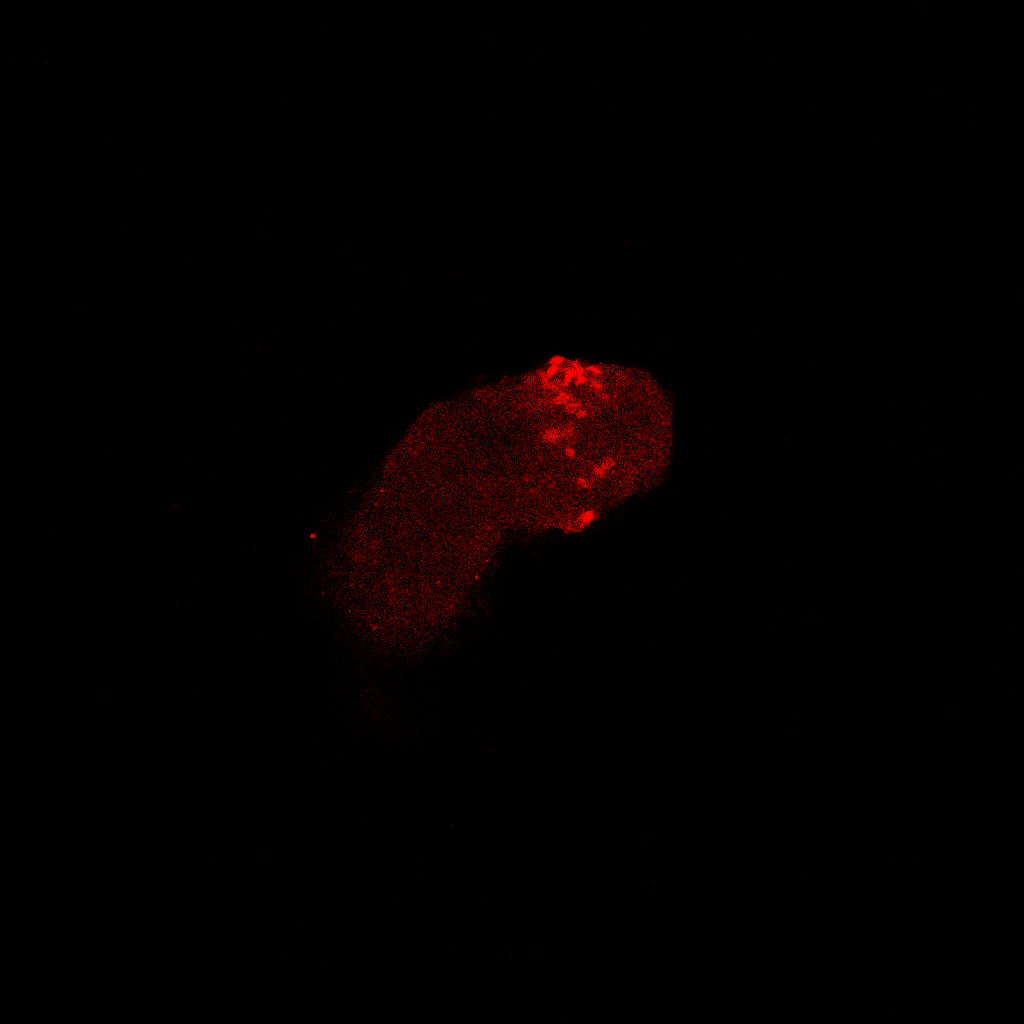

Supplement: Supplementary file 3 — Source data Fig. 1 [file 44318_2025_558_MOESM3_ESM.zip › Figure 1/panel 1F/NT_Sox17/image0040_image0040_RGB_Cy3 dyeΓÇôlabeled IgG antibody_pH 7.2.tif]

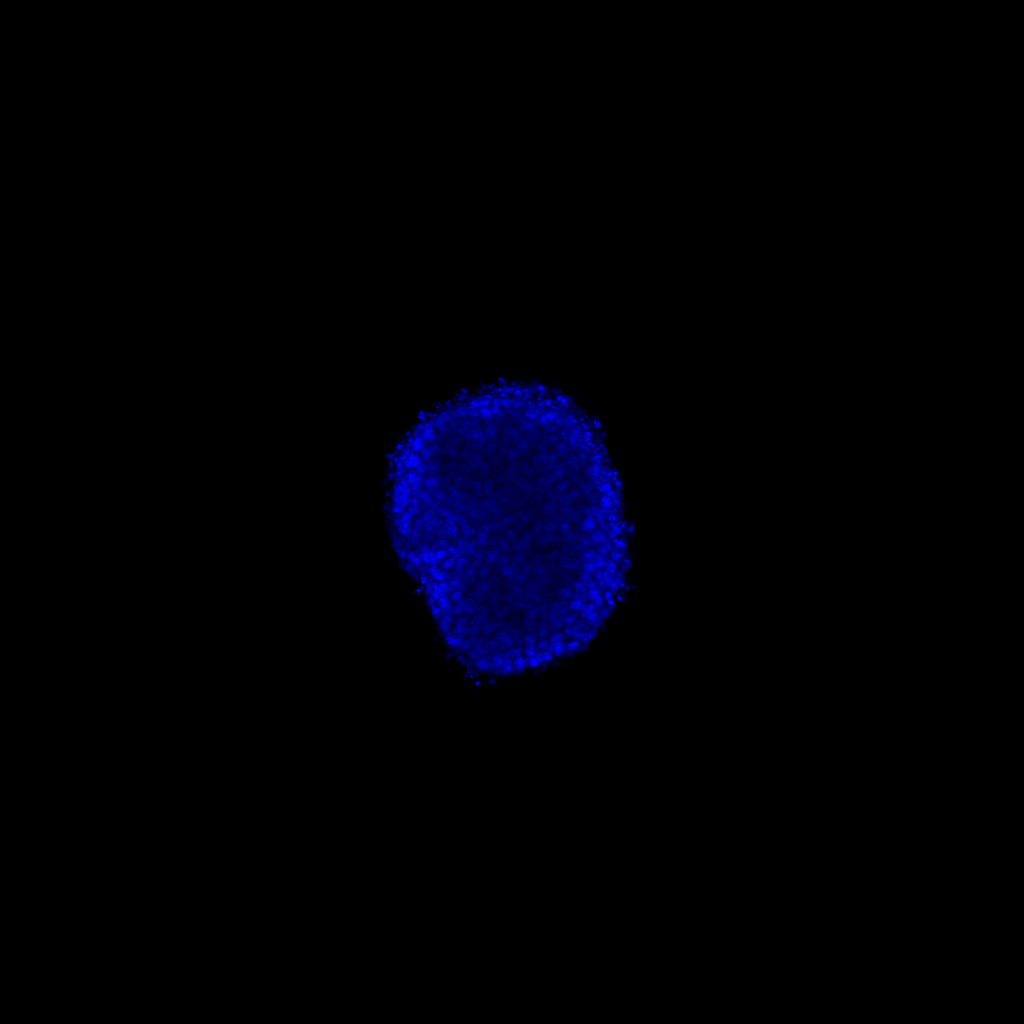

Supplement: Supplementary file 3 — Source data Fig. 1 [file 44318_2025_558_MOESM3_ESM.zip › Figure 1/panel 1F/KD-2_Sox17/image0130_image0130_RGB_DAPI.tif]

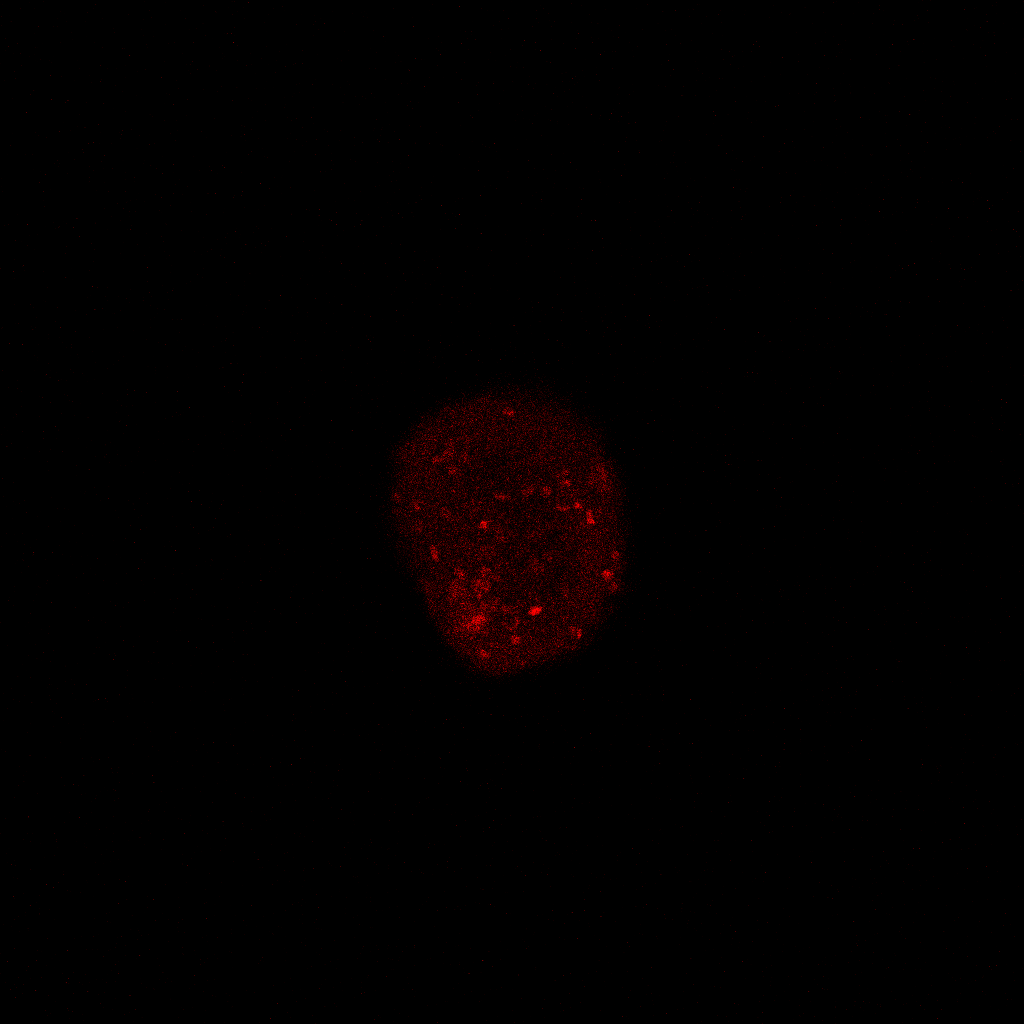

Supplement: Supplementary file 3 — Source data Fig. 1 [file 44318_2025_558_MOESM3_ESM.zip › Figure 1/panel 1F/KD-2_Sox17/image0130_image0130_RGB_Texas Red.tif]

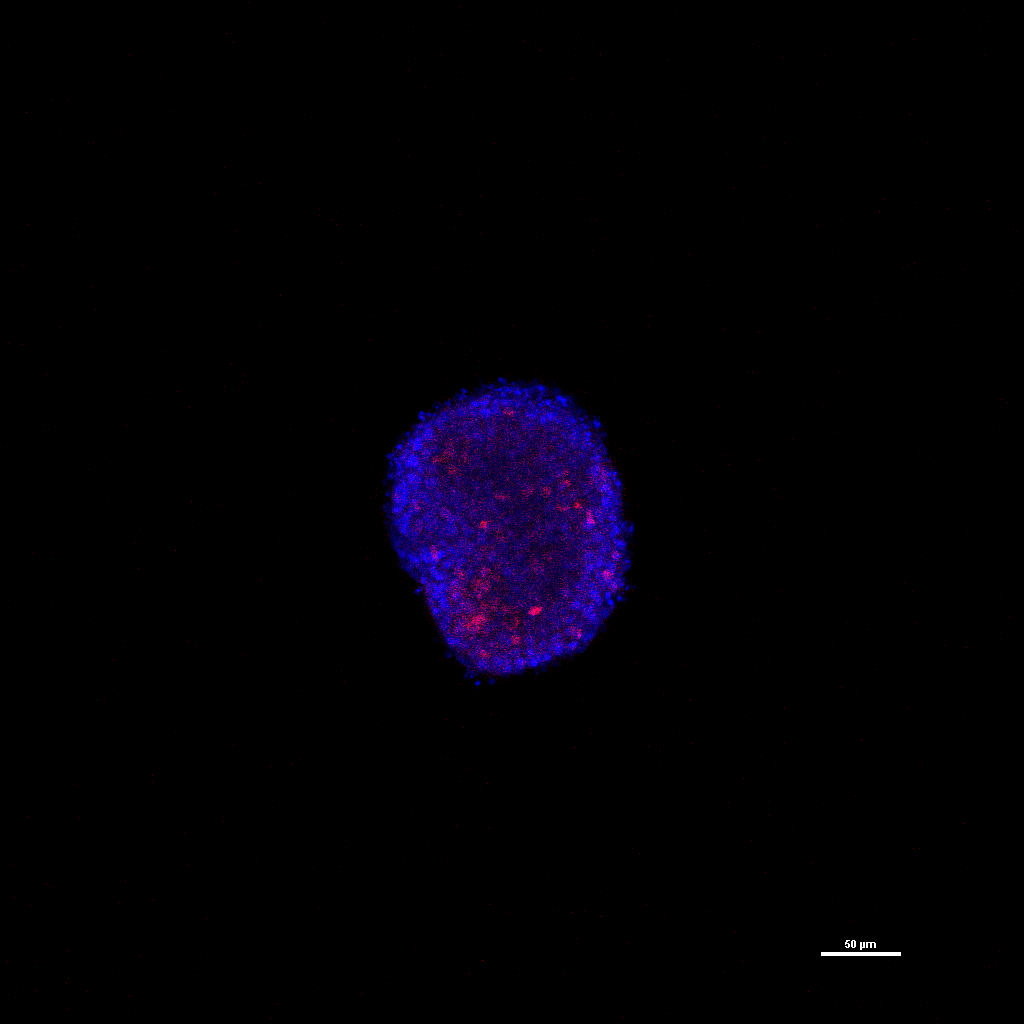

Supplement: Supplementary file 3 — Source data Fig. 1 [file 44318_2025_558_MOESM3_ESM.zip › Figure 1/panel 1F/KD-2_Sox17/image0130_image0130_RGB.tif]

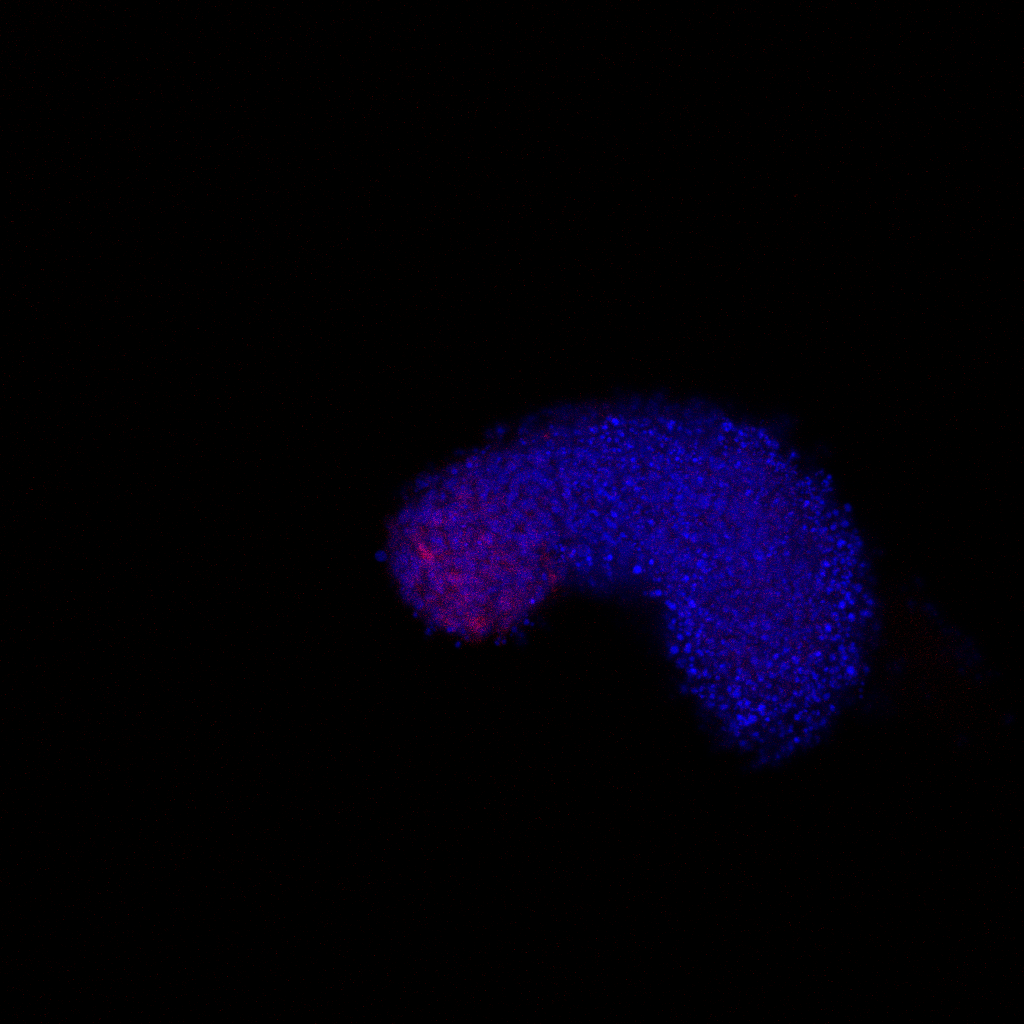

Supplement: Supplementary file 3 — Source data Fig. 1 [file 44318_2025_558_MOESM3_ESM.zip › Figure 1/panel 1F/NT_Bra/seq8683.tif]

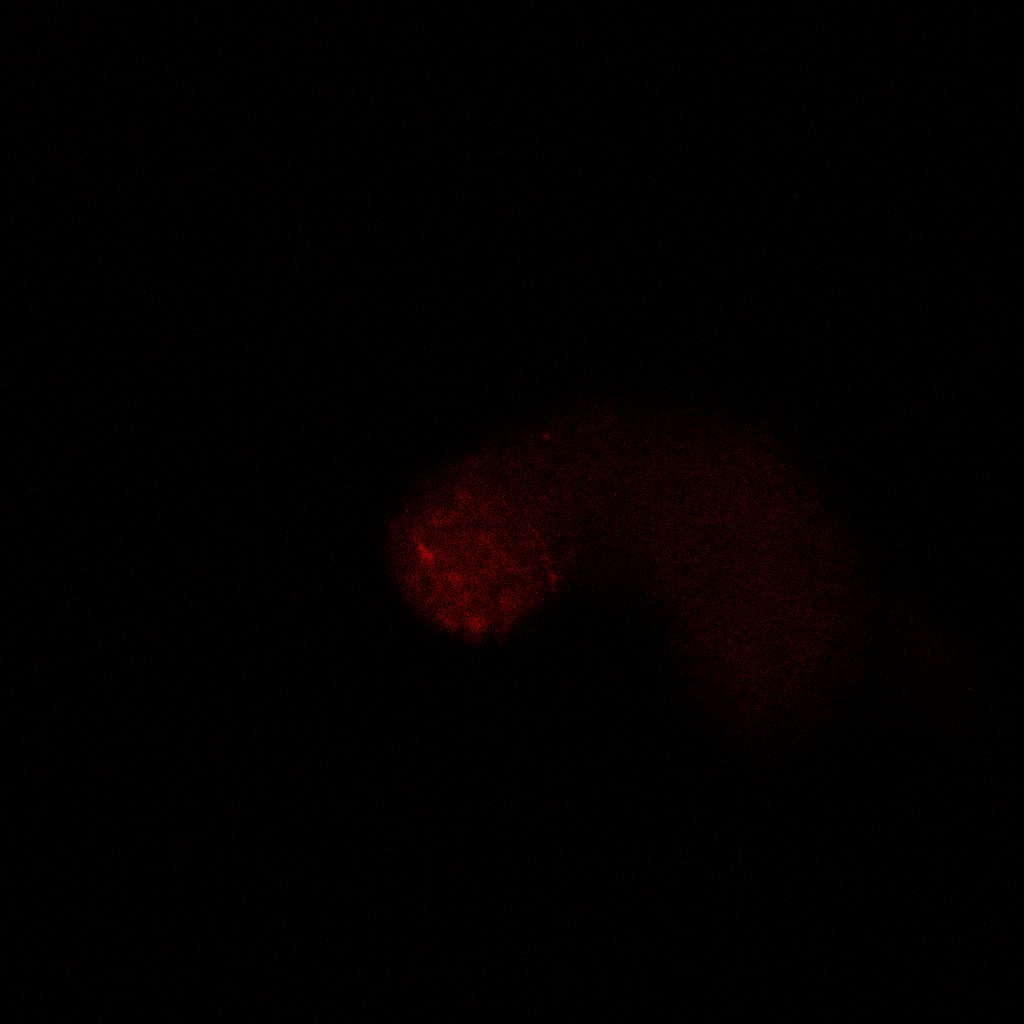

Supplement: Supplementary file 3 — Source data Fig. 1 [file 44318_2025_558_MOESM3_ESM.zip › Figure 1/panel 1F/NT_Bra/seq8683c2.tif]

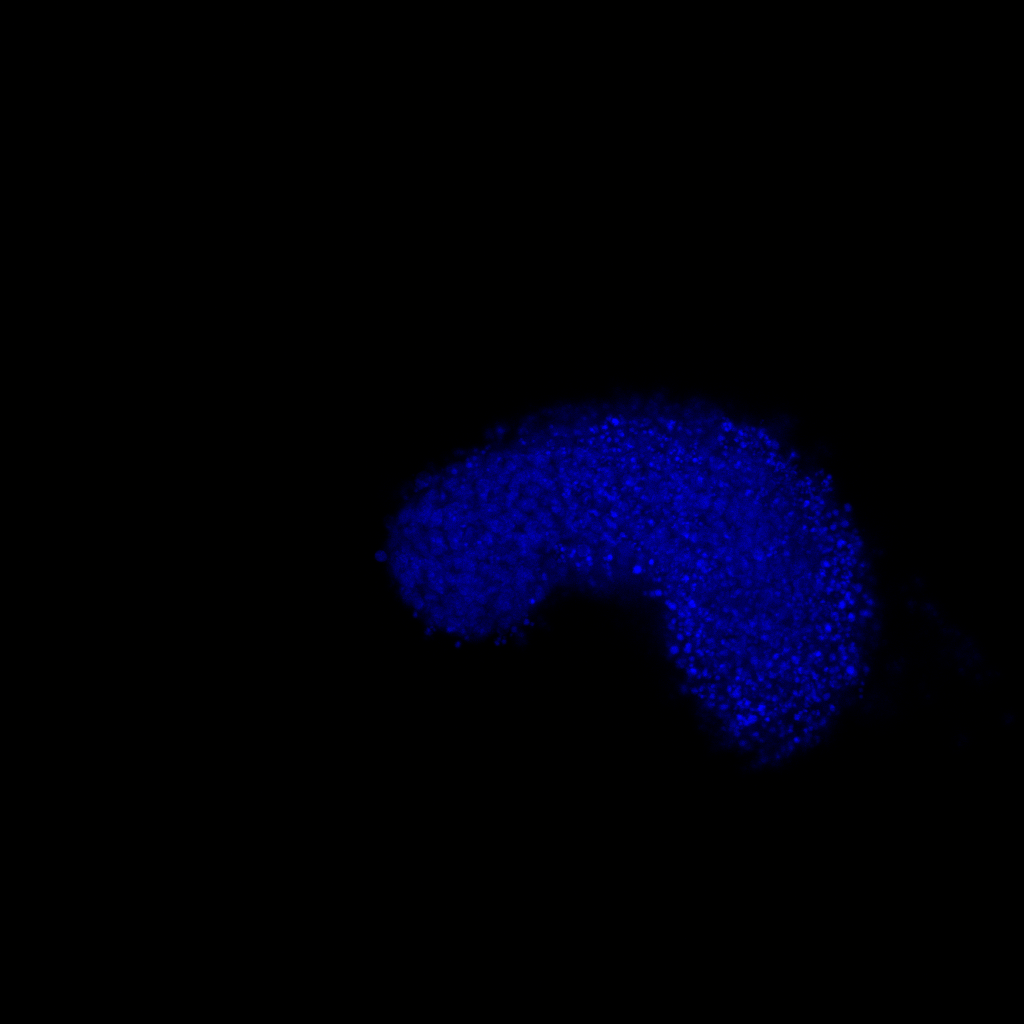

Supplement: Supplementary file 3 — Source data Fig. 1 [file 44318_2025_558_MOESM3_ESM.zip › Figure 1/panel 1F/NT_Bra/seq8683c1.tif]

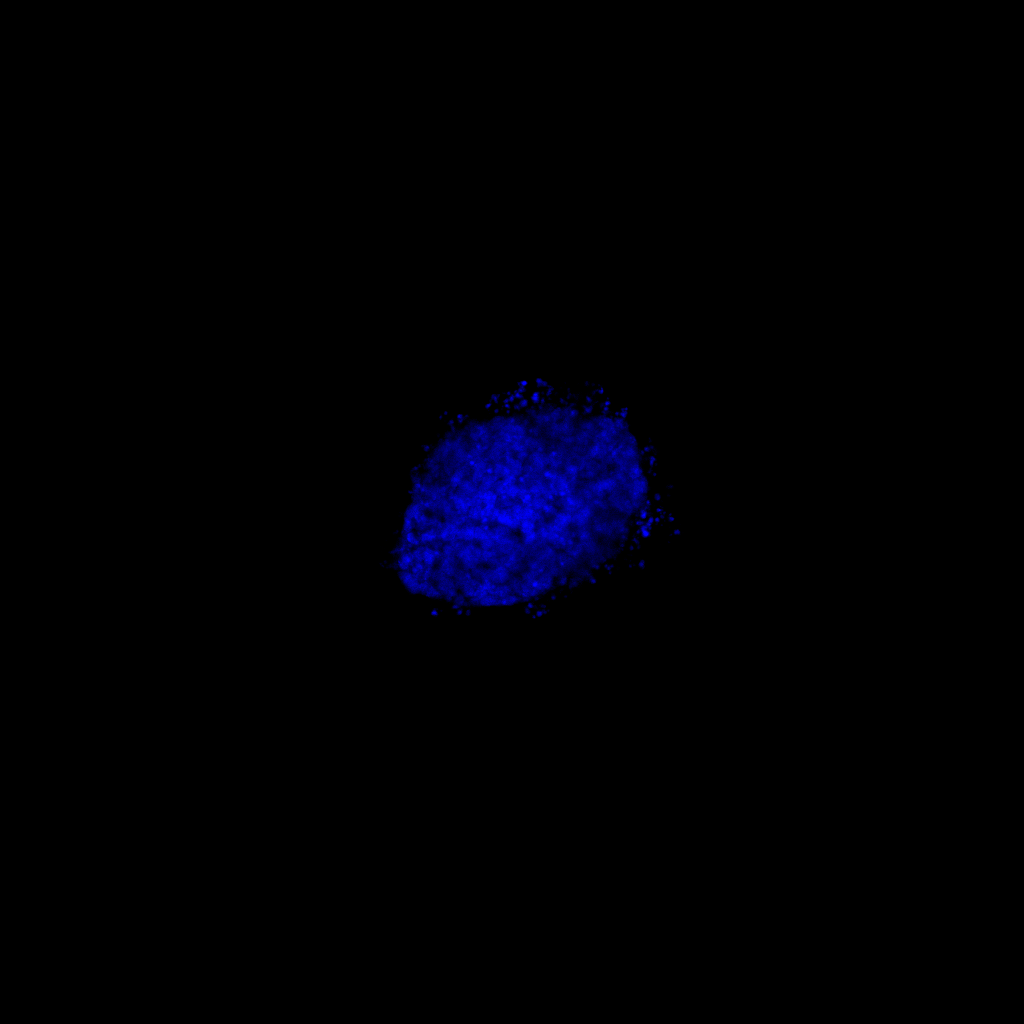

Supplement: Supplementary file 3 — Source data Fig. 1 [file 44318_2025_558_MOESM3_ESM.zip › Figure 1/panel 1F/KD-2_Nanog/seq8803_seq8803_RGB_DAPI.tif]

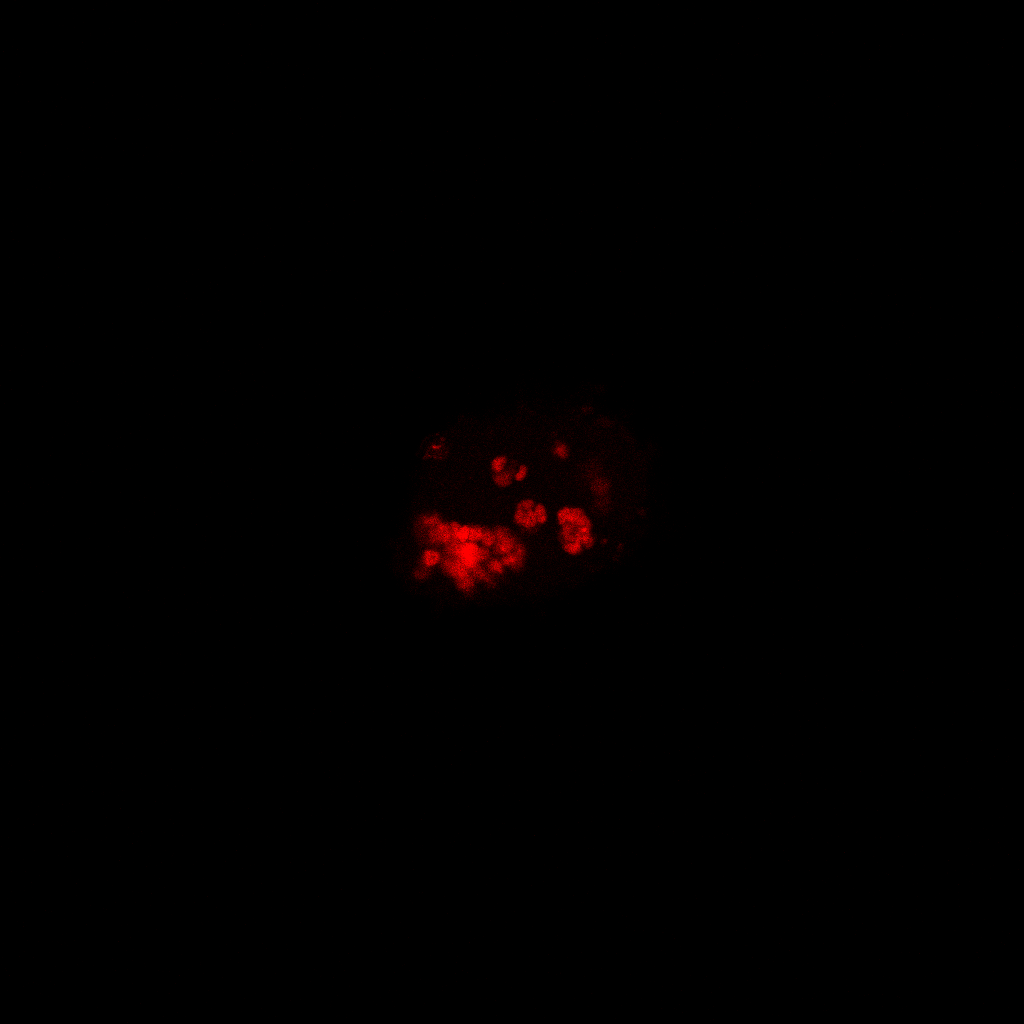

Supplement: Supplementary file 3 — Source data Fig. 1 [file 44318_2025_558_MOESM3_ESM.zip › Figure 1/panel 1F/KD-2_Nanog/seq8803_seq8803_RGB_Texas Red.tif]

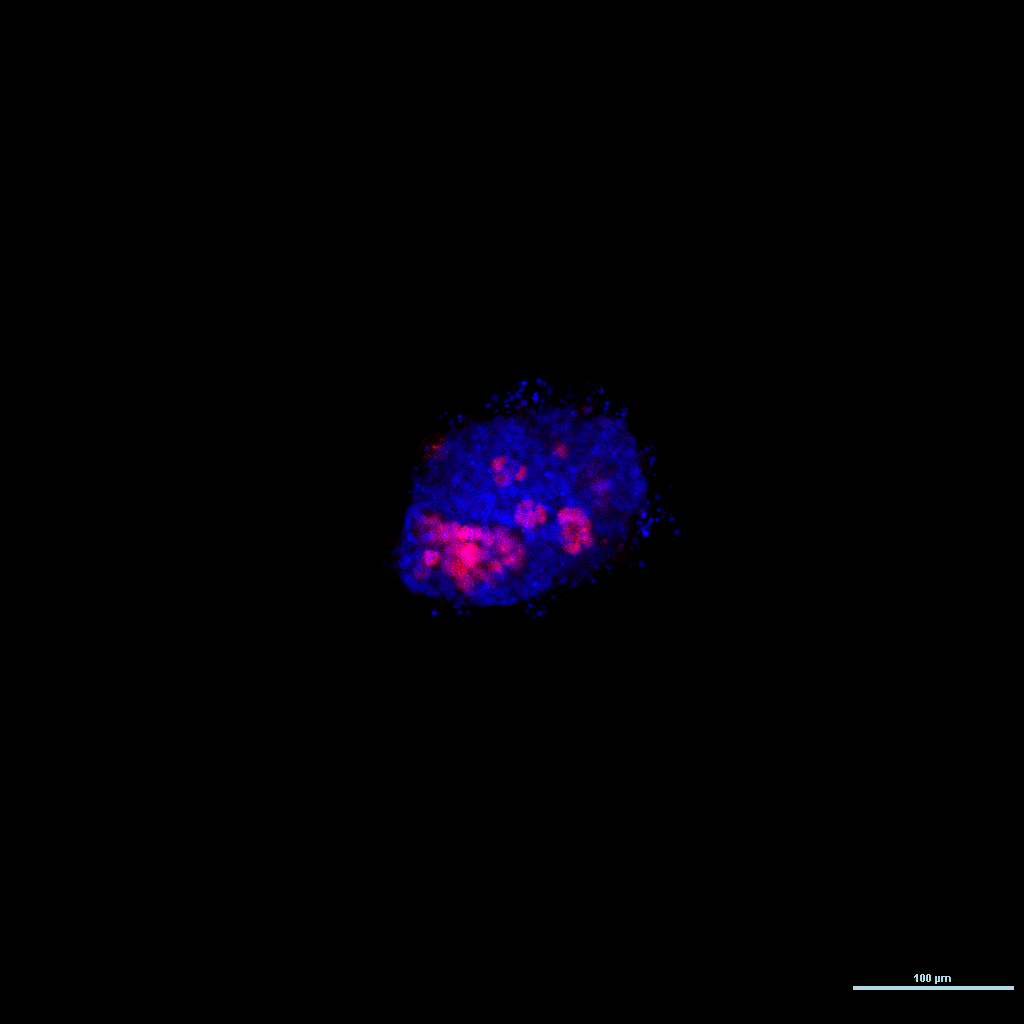

Supplement: Supplementary file 3 — Source data Fig. 1 [file 44318_2025_558_MOESM3_ESM.zip › Figure 1/panel 1F/KD-2_Nanog/seq8803_seq8803_RGB.tif]

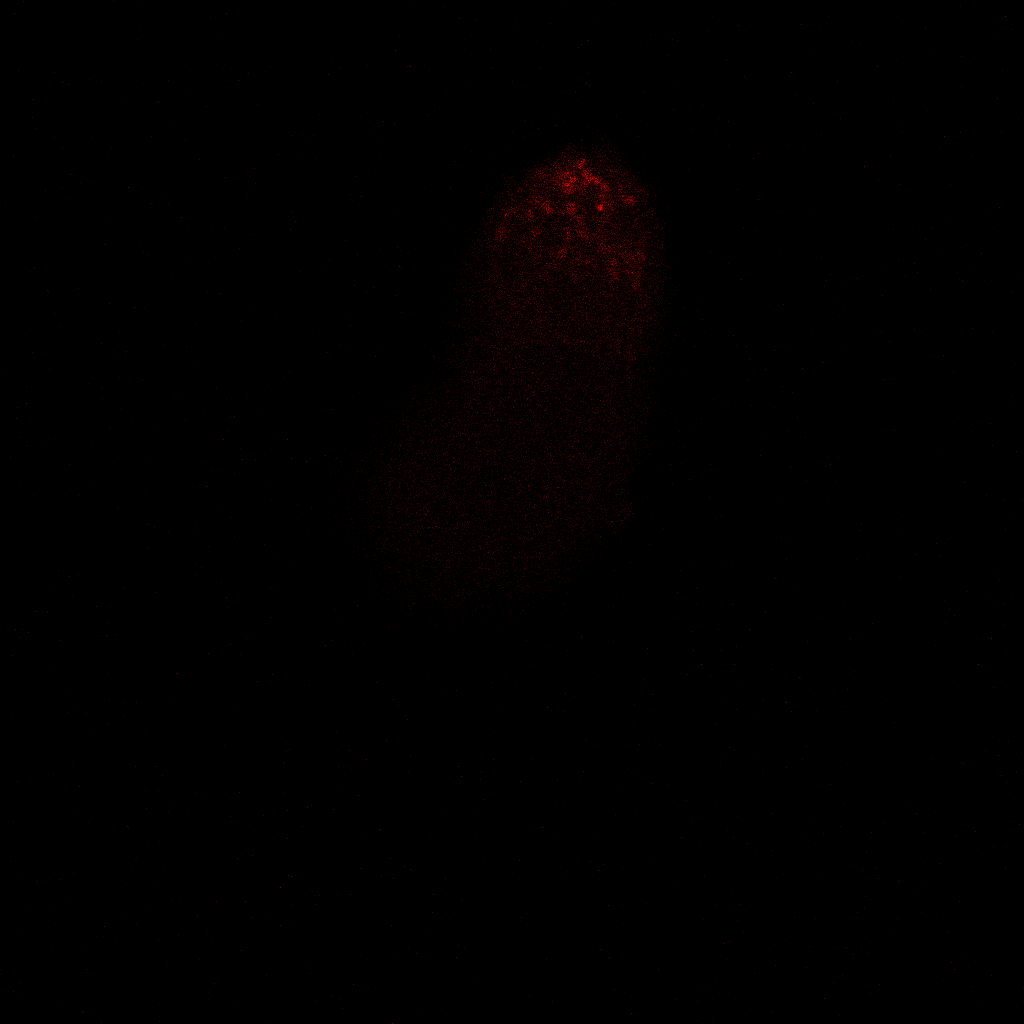

Supplement: Supplementary file 3 — Source data Fig. 1 [file 44318_2025_558_MOESM3_ESM.zip › Figure 1/panel 1F/NT_Sox2/image0142_image0142_RGB_Texas Red.tif]

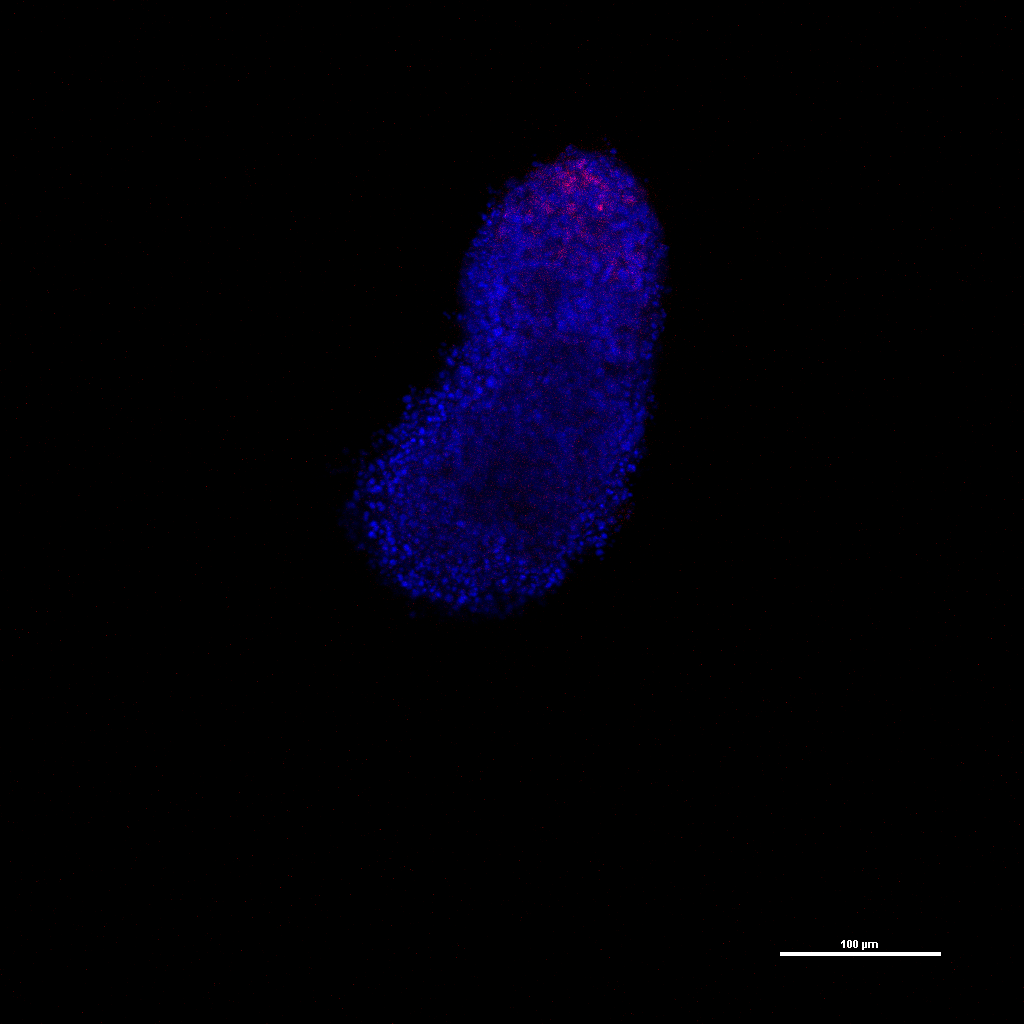

Supplement: Supplementary file 3 — Source data Fig. 1 [file 44318_2025_558_MOESM3_ESM.zip › Figure 1/panel 1F/NT_Sox2/image0142_image0142_RGB.tif]

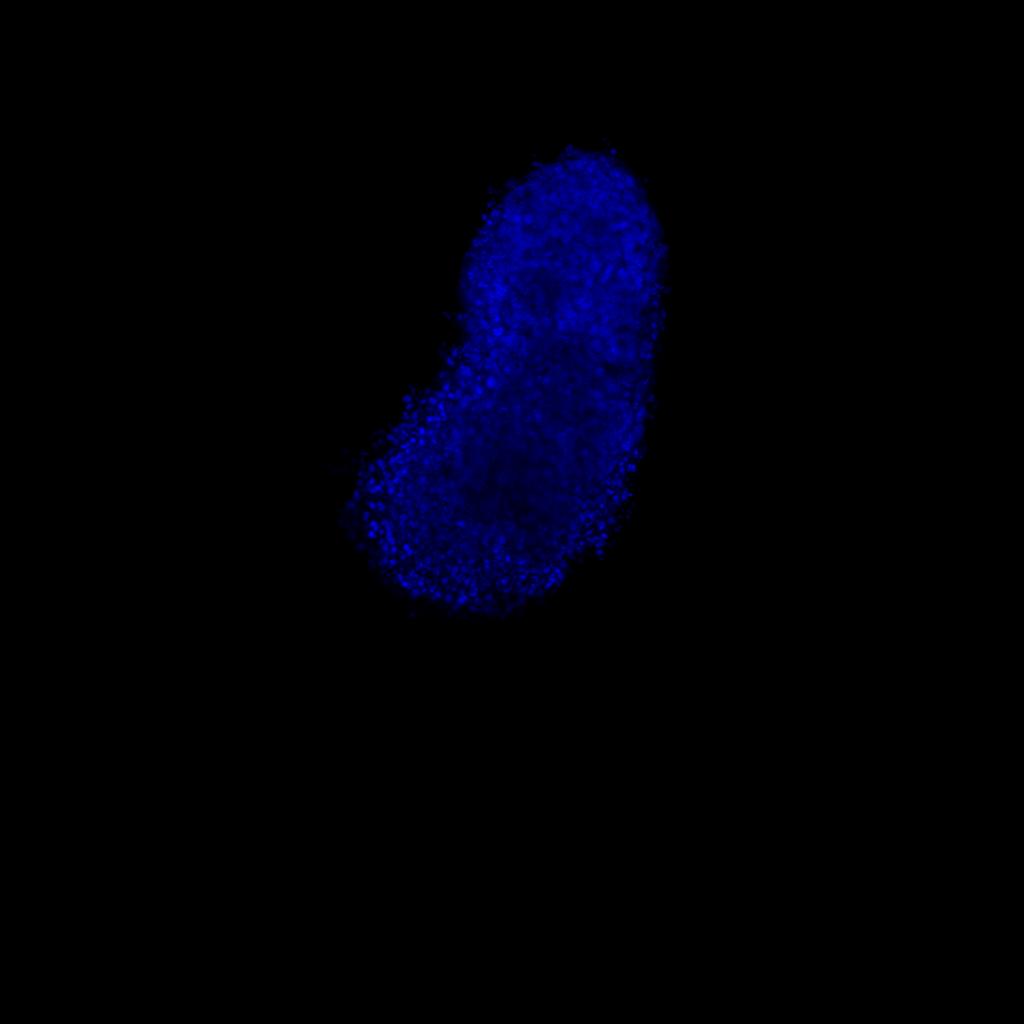

Supplement: Supplementary file 3 — Source data Fig. 1 [file 44318_2025_558_MOESM3_ESM.zip › Figure 1/panel 1F/NT_Sox2/image0142_image0142_RGB_DAPI.tif]

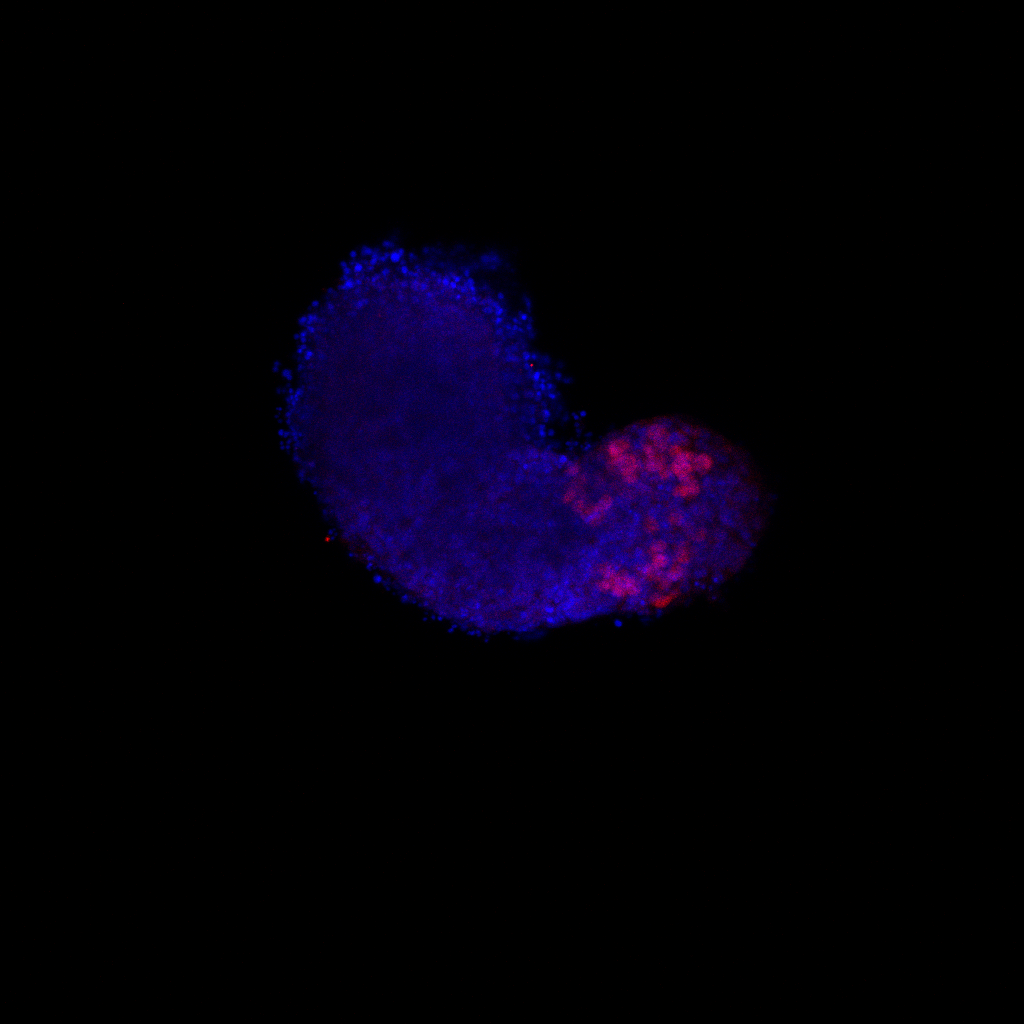

Supplement: Supplementary file 3 — Source data Fig. 1 [file 44318_2025_558_MOESM3_ESM.zip › Figure 1/panel 1F/NT_Oct4/seq8695.tif]

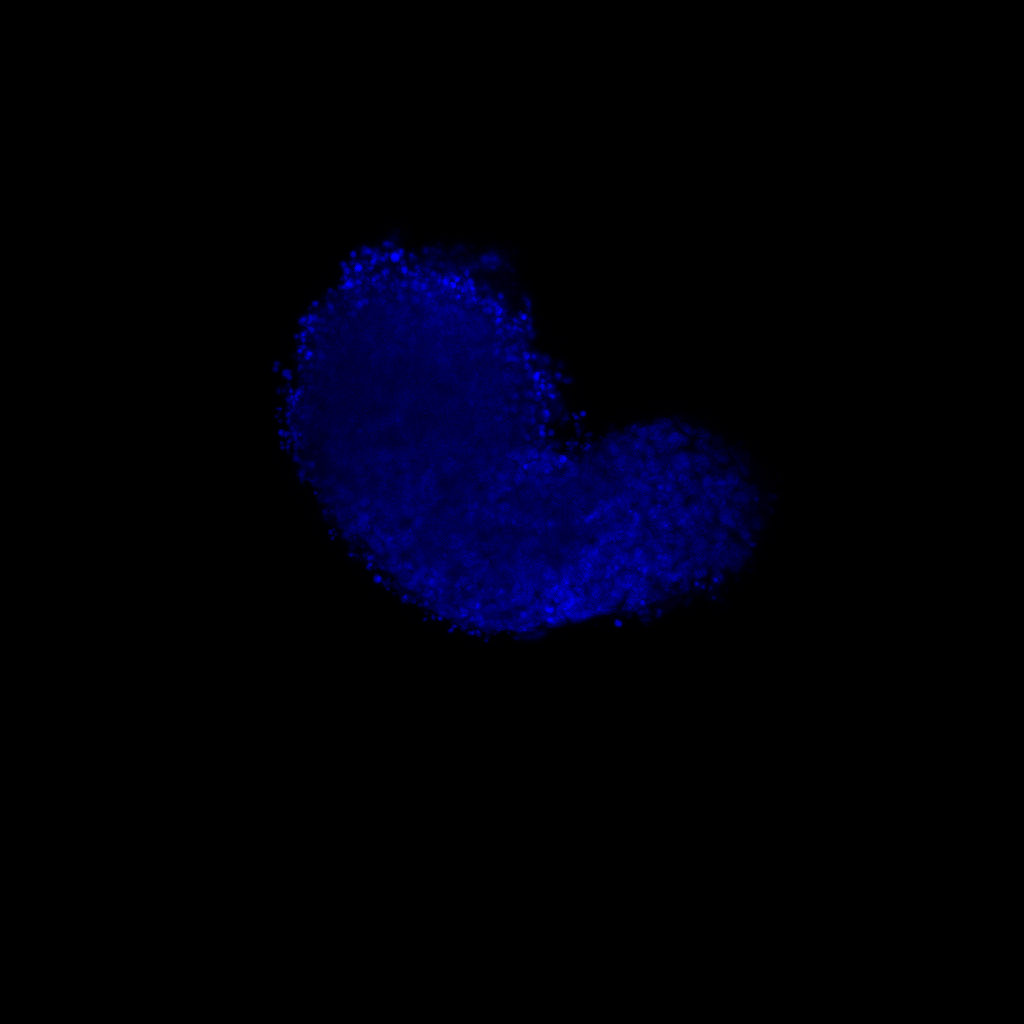

Supplement: Supplementary file 3 — Source data Fig. 1 [file 44318_2025_558_MOESM3_ESM.zip › Figure 1/panel 1F/NT_Oct4/seq8695c1.tif]

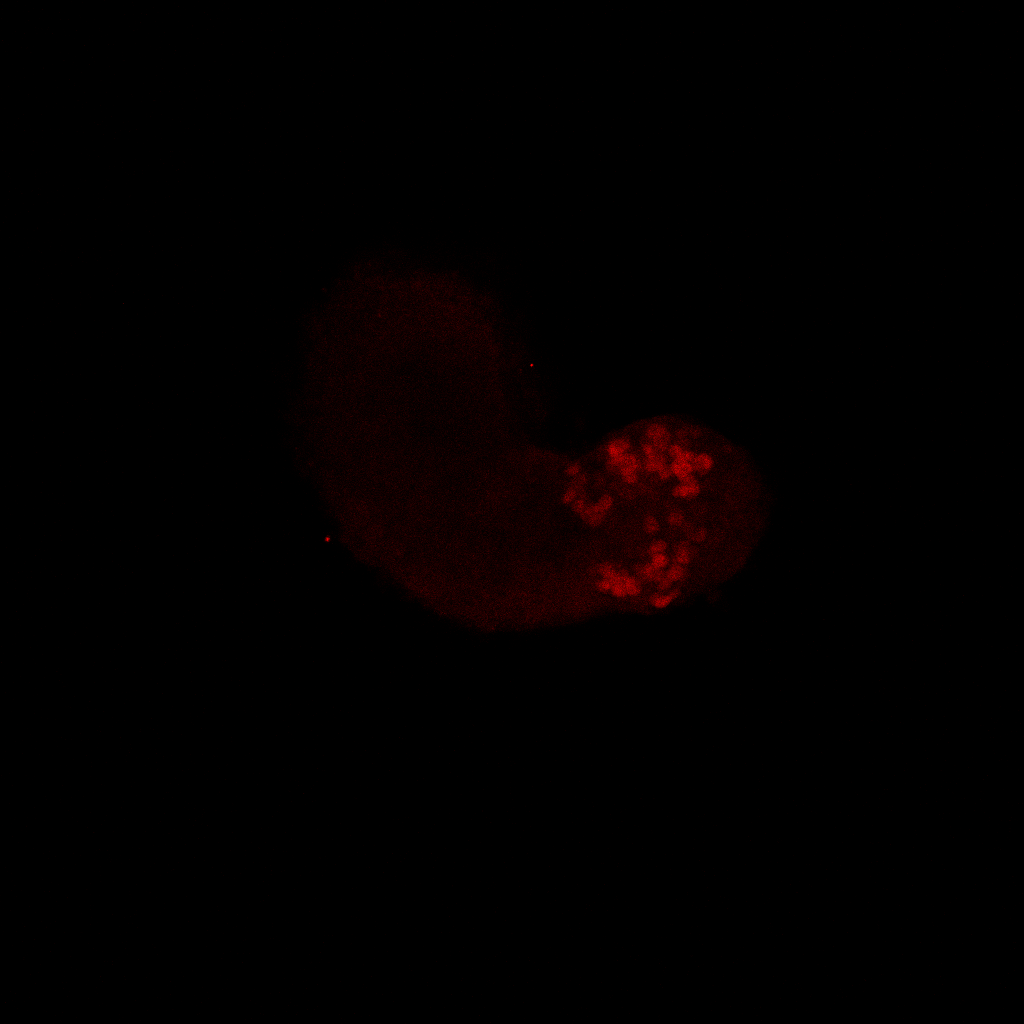

Supplement: Supplementary file 3 — Source data Fig. 1 [file 44318_2025_558_MOESM3_ESM.zip › Figure 1/panel 1F/NT_Oct4/seq8695c2.tif]

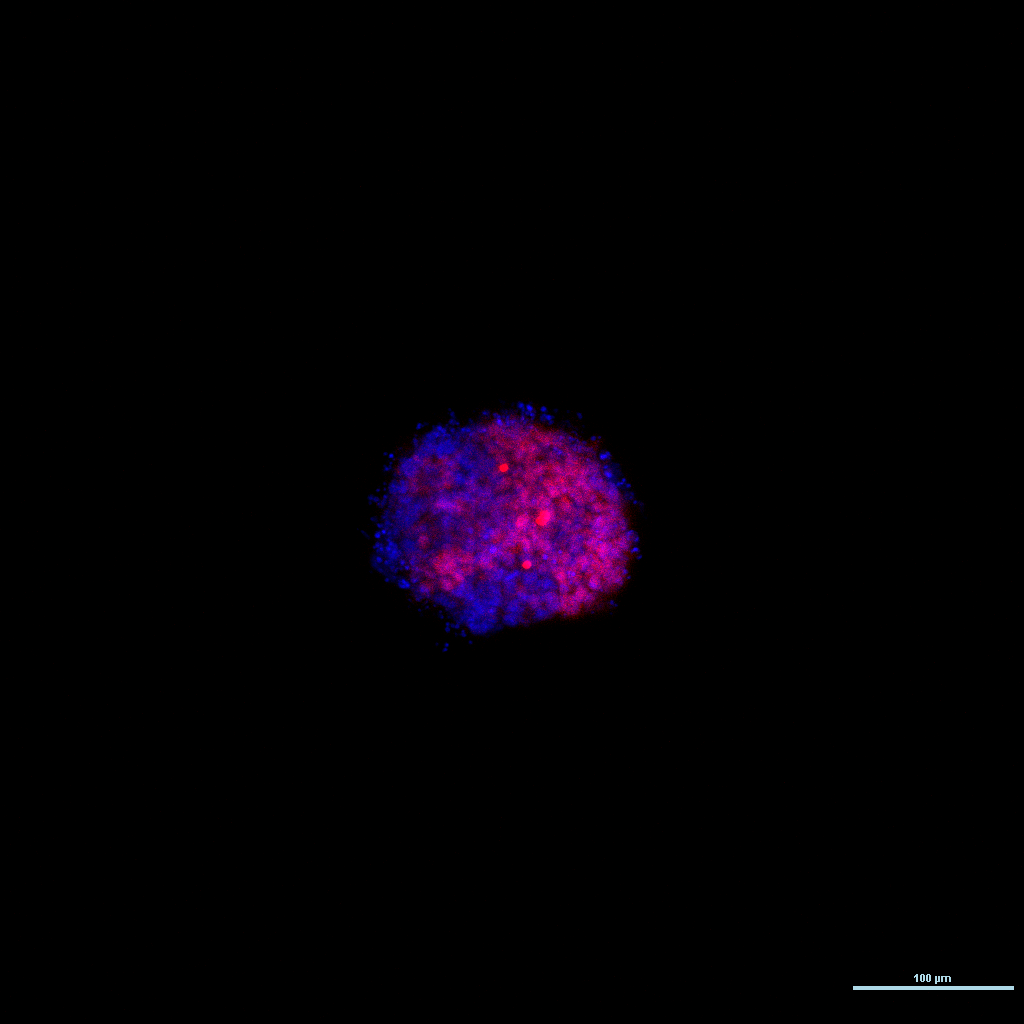

Supplement: Supplementary file 3 — Source data Fig. 1 [file 44318_2025_558_MOESM3_ESM.zip › Figure 1/panel 1F/KD-2_Cdx2/seq8794_seq8794_RGB.tif]

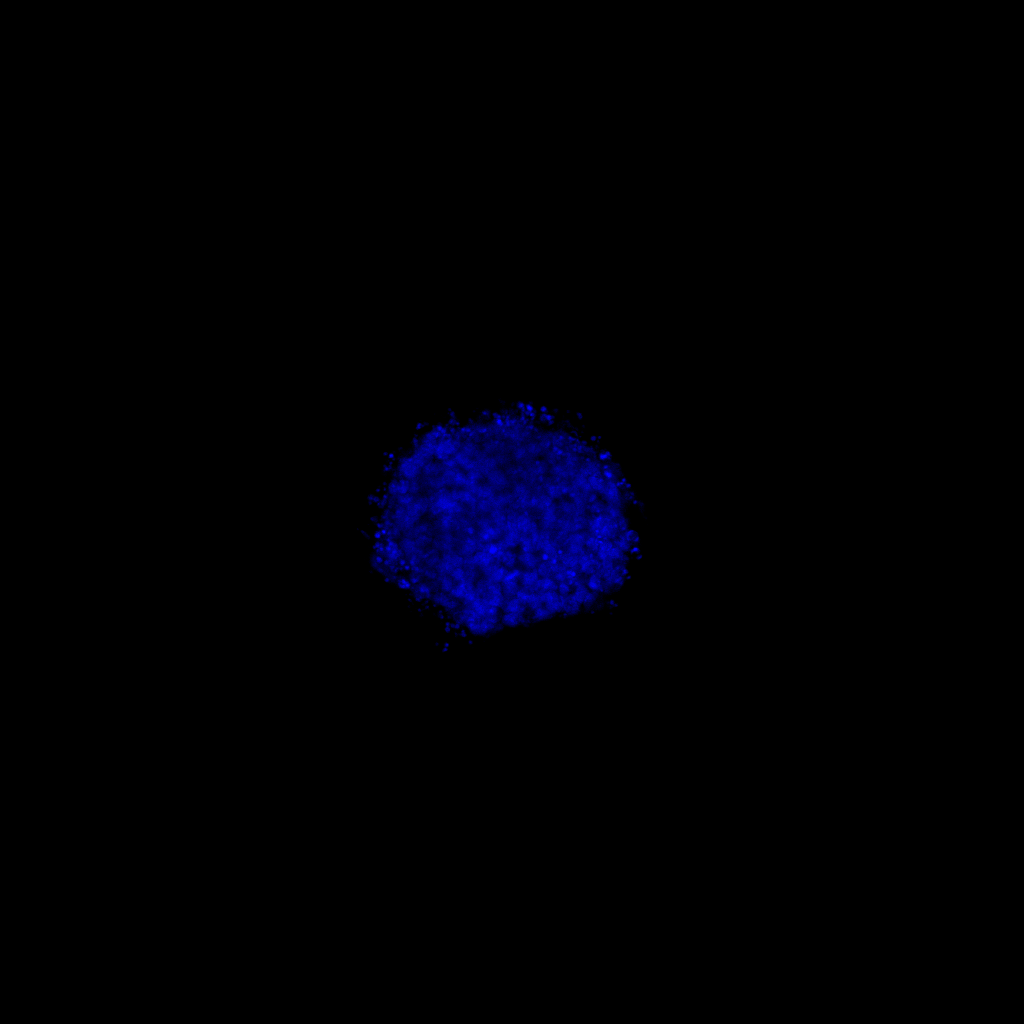

Supplement: Supplementary file 3 — Source data Fig. 1 [file 44318_2025_558_MOESM3_ESM.zip › Figure 1/panel 1F/KD-2_Cdx2/seq8794_seq8794_RGB_DAPI.tif]

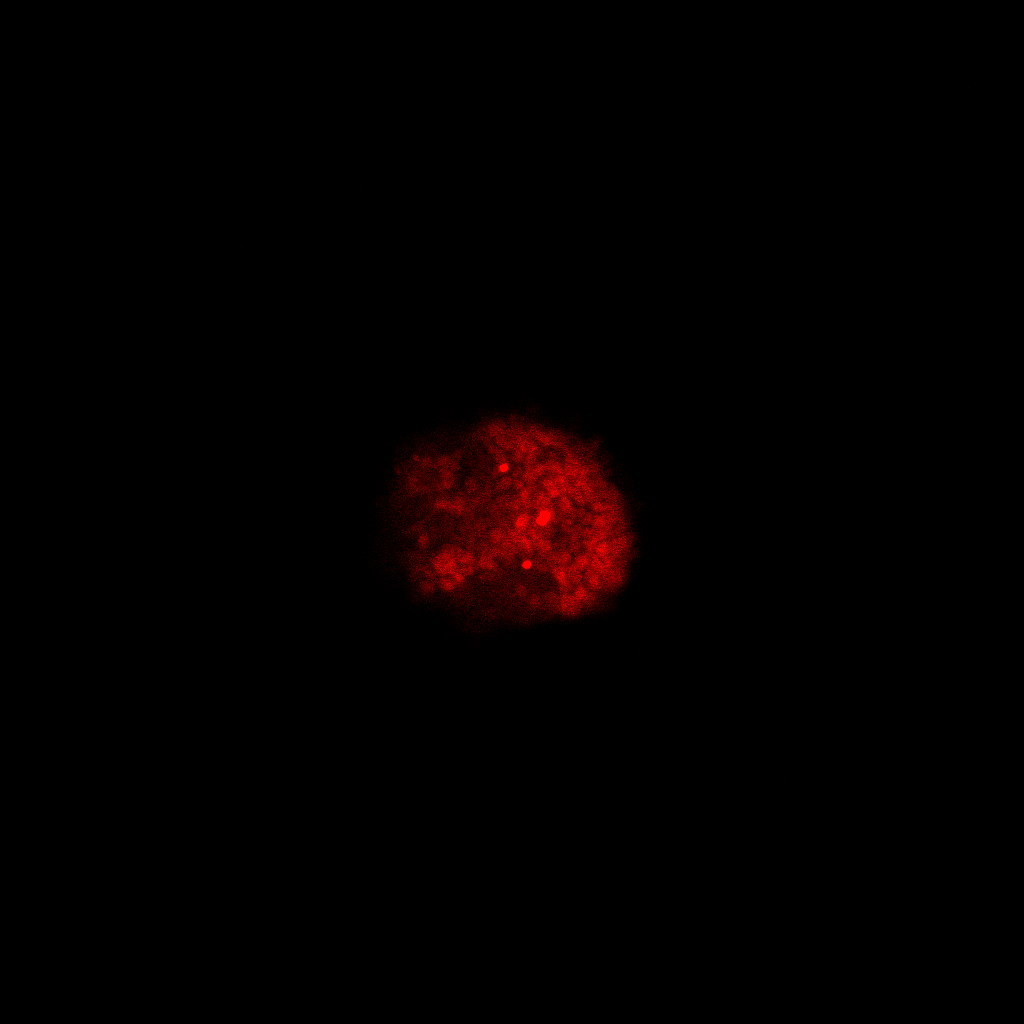

Supplement: Supplementary file 3 — Source data Fig. 1 [file 44318_2025_558_MOESM3_ESM.zip › Figure 1/panel 1F/KD-2_Cdx2/seq8794_seq8794_RGB_Texas Red.tif]

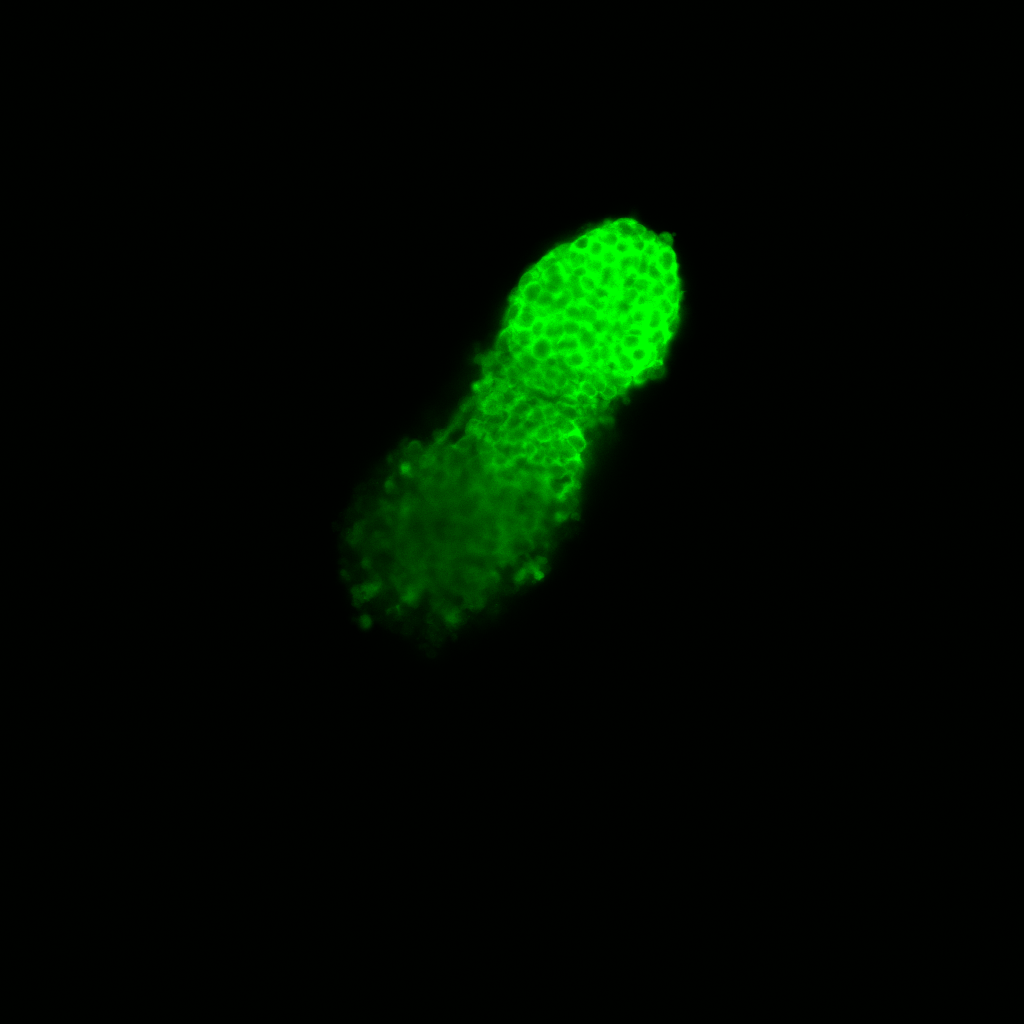

Supplement: Supplementary file 3 — Source data Fig. 1 [file 44318_2025_558_MOESM3_ESM.zip › Figure 1/panel 1F/NT_Bra:e-cadh/seq11148_seq11148_RGB_FITC.tif]

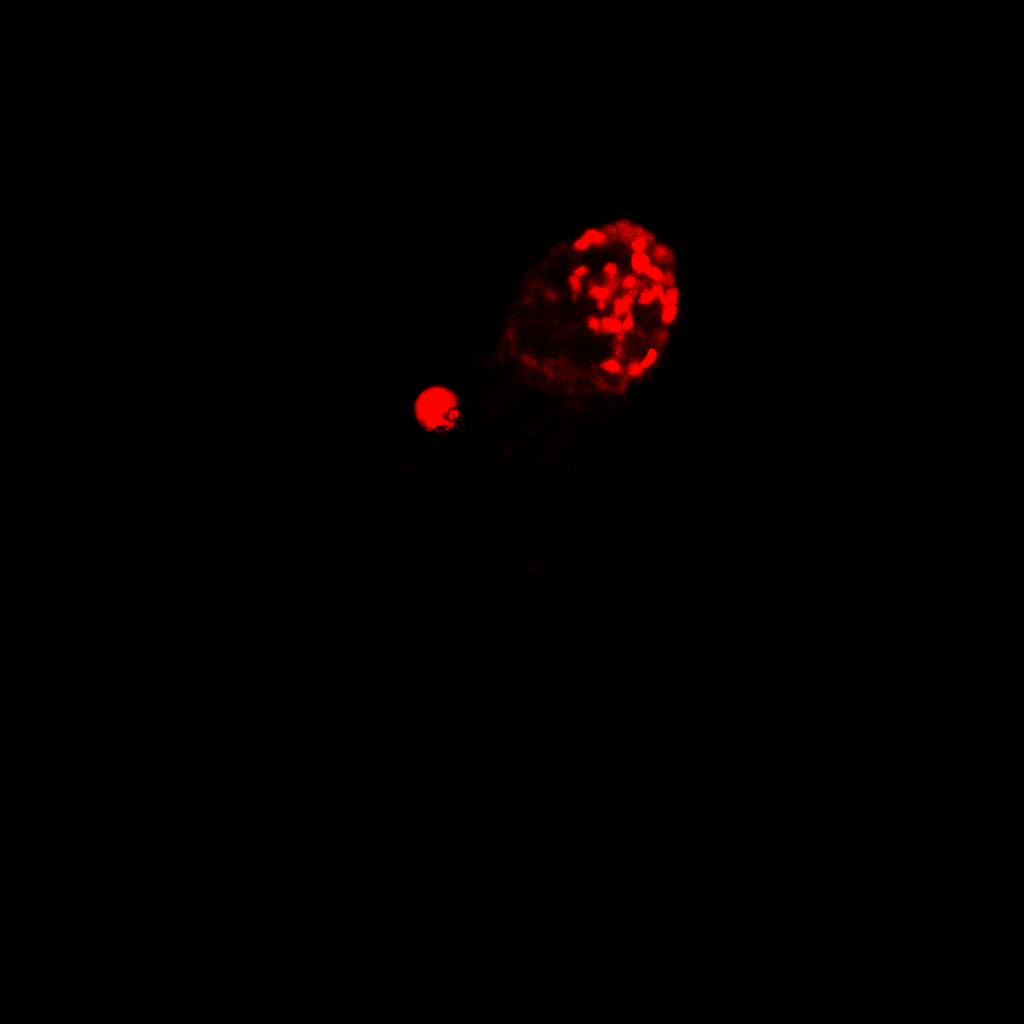

Supplement: Supplementary file 3 — Source data Fig. 1 [file 44318_2025_558_MOESM3_ESM.zip › Figure 1/panel 1F/NT_Bra:e-cadh/seq11148_seq11148_RGB_TRITC.tif]

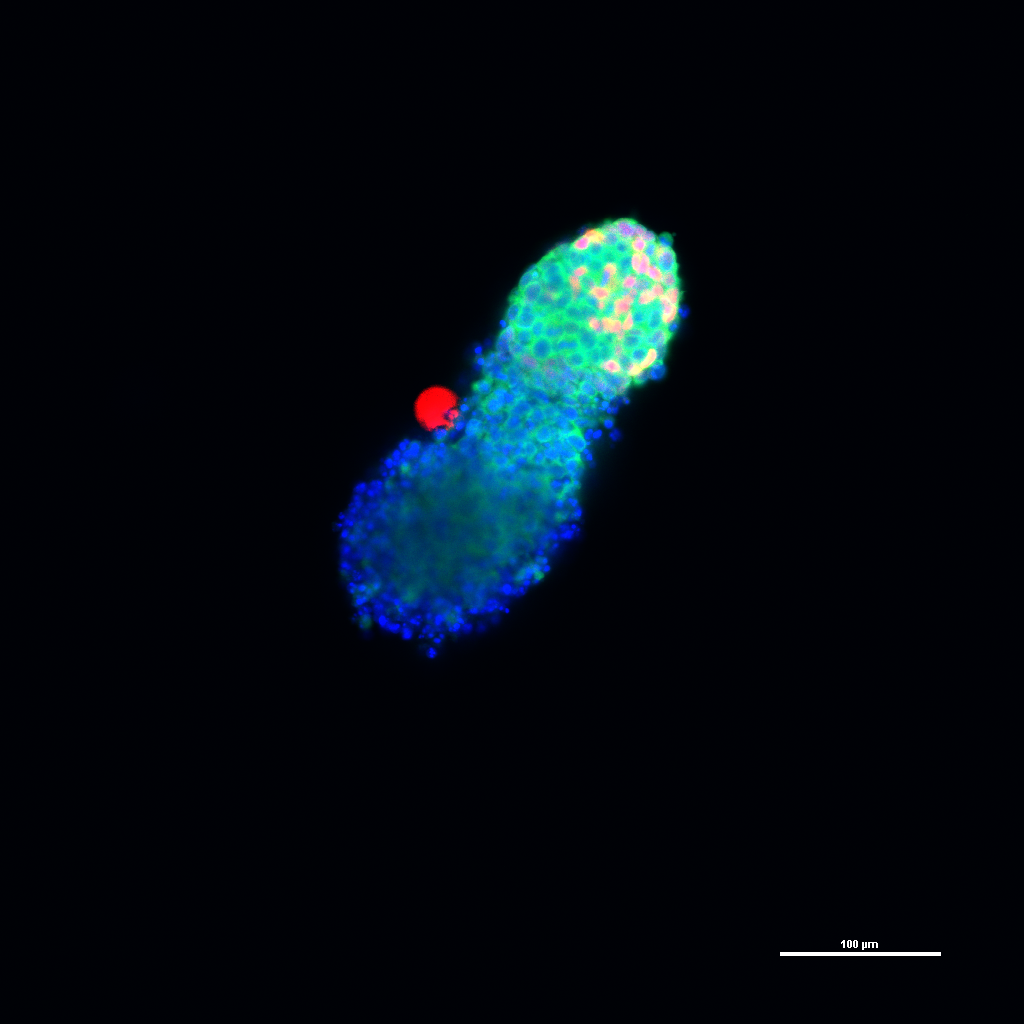

Supplement: Supplementary file 3 — Source data Fig. 1 [file 44318_2025_558_MOESM3_ESM.zip › Figure 1/panel 1F/NT_Bra:e-cadh/seq11148_seq11148_RGB.tif]

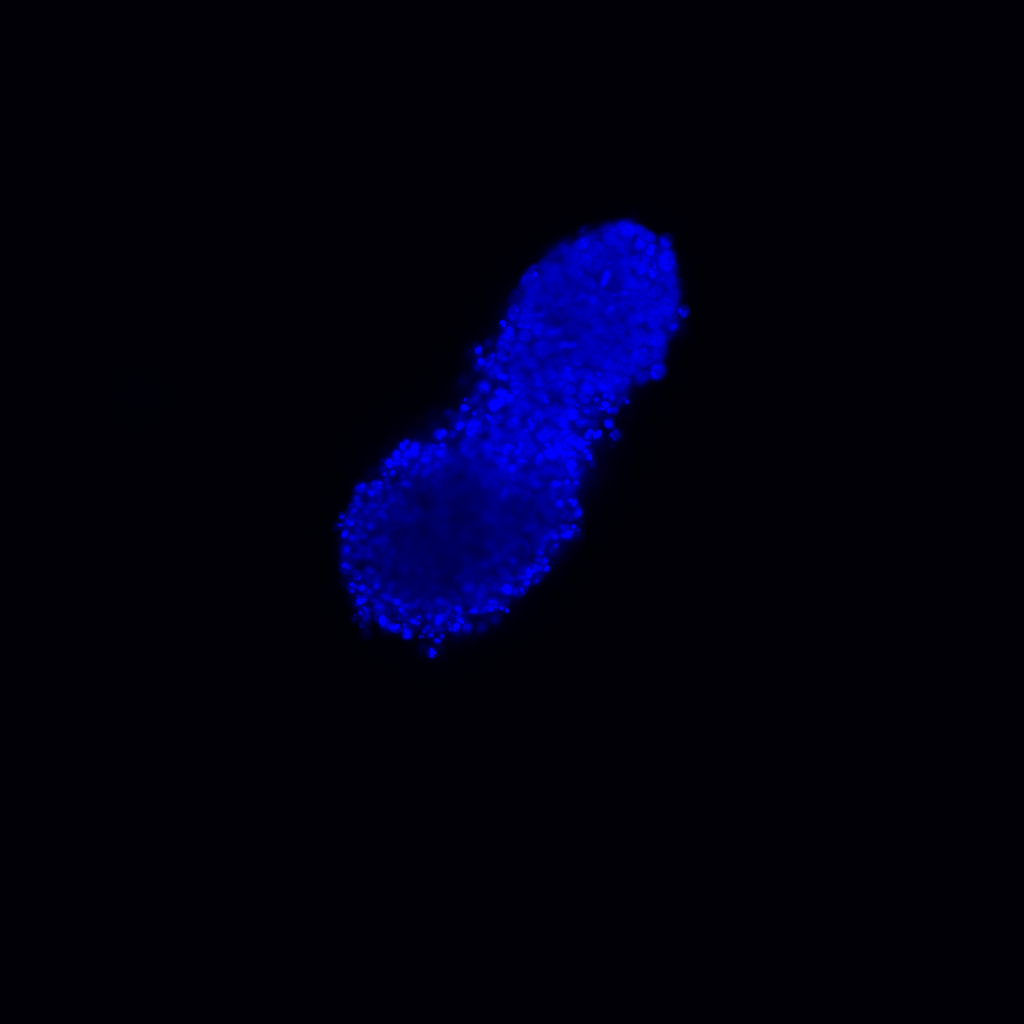

Supplement: Supplementary file 3 — Source data Fig. 1 [file 44318_2025_558_MOESM3_ESM.zip › Figure 1/panel 1F/NT_Bra:e-cadh/seq11148_seq11148_RGB_DAPI.tif]

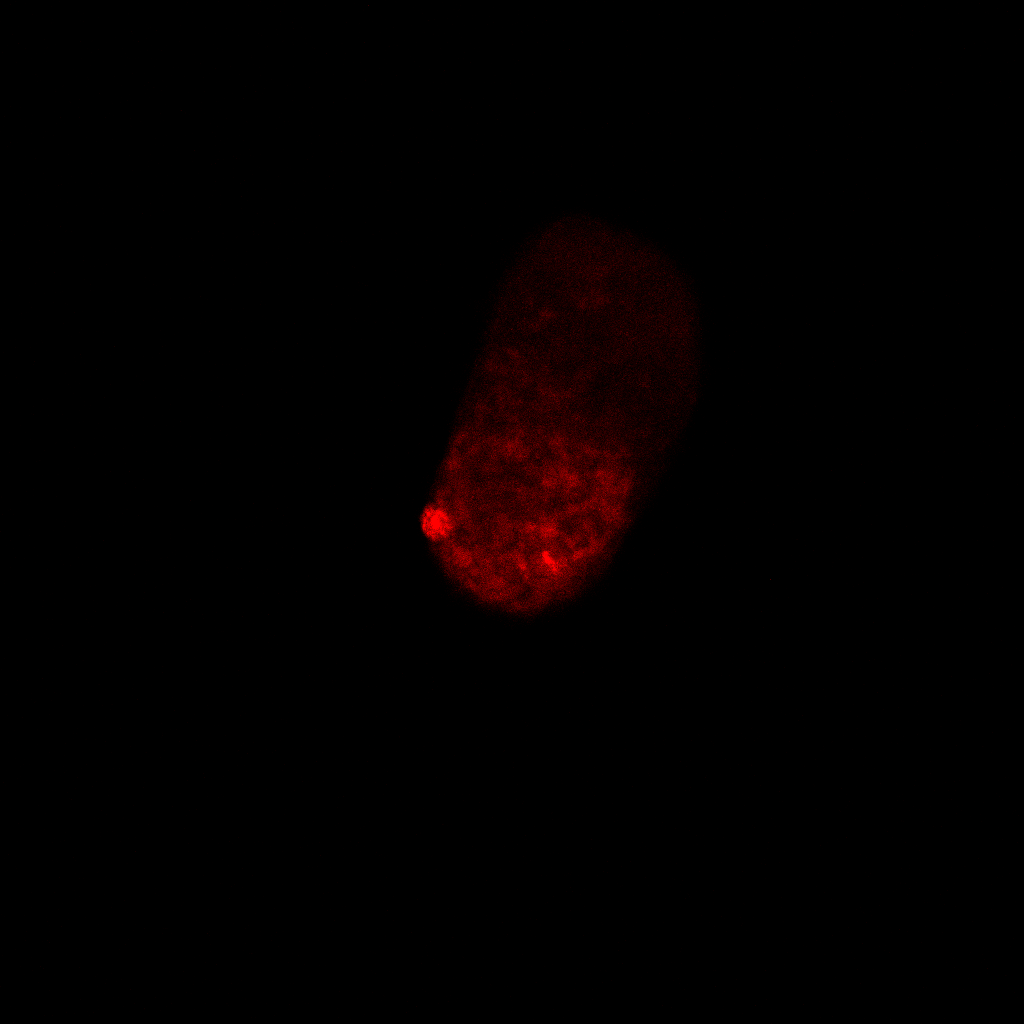

Supplement: Supplementary file 3 — Source data Fig. 1 [file 44318_2025_558_MOESM3_ESM.zip › Figure 1/panel 1F/KD-1_Cdx2/seq8792_seq8792_RGB_Texas Red.tif]

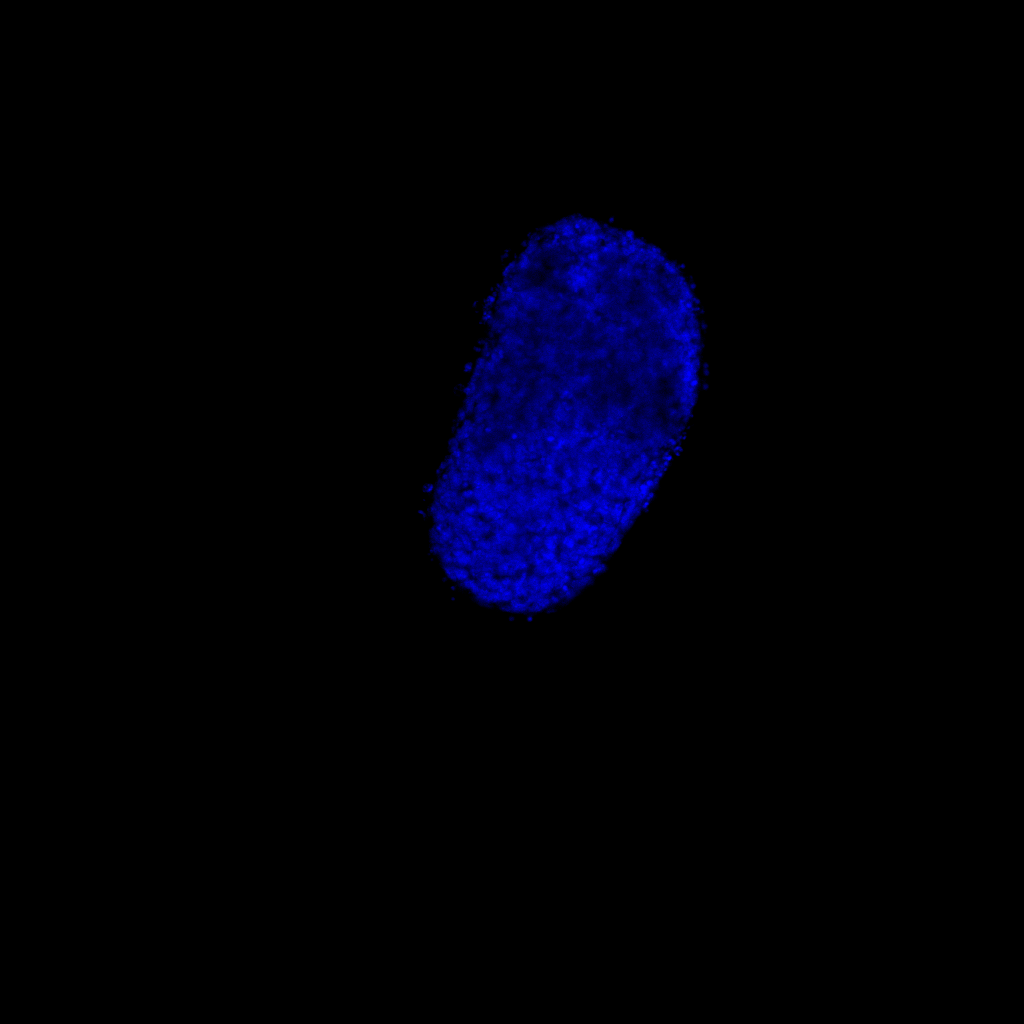

Supplement: Supplementary file 3 — Source data Fig. 1 [file 44318_2025_558_MOESM3_ESM.zip › Figure 1/panel 1F/KD-1_Cdx2/seq8792_seq8792_RGB_DAPI.tif]

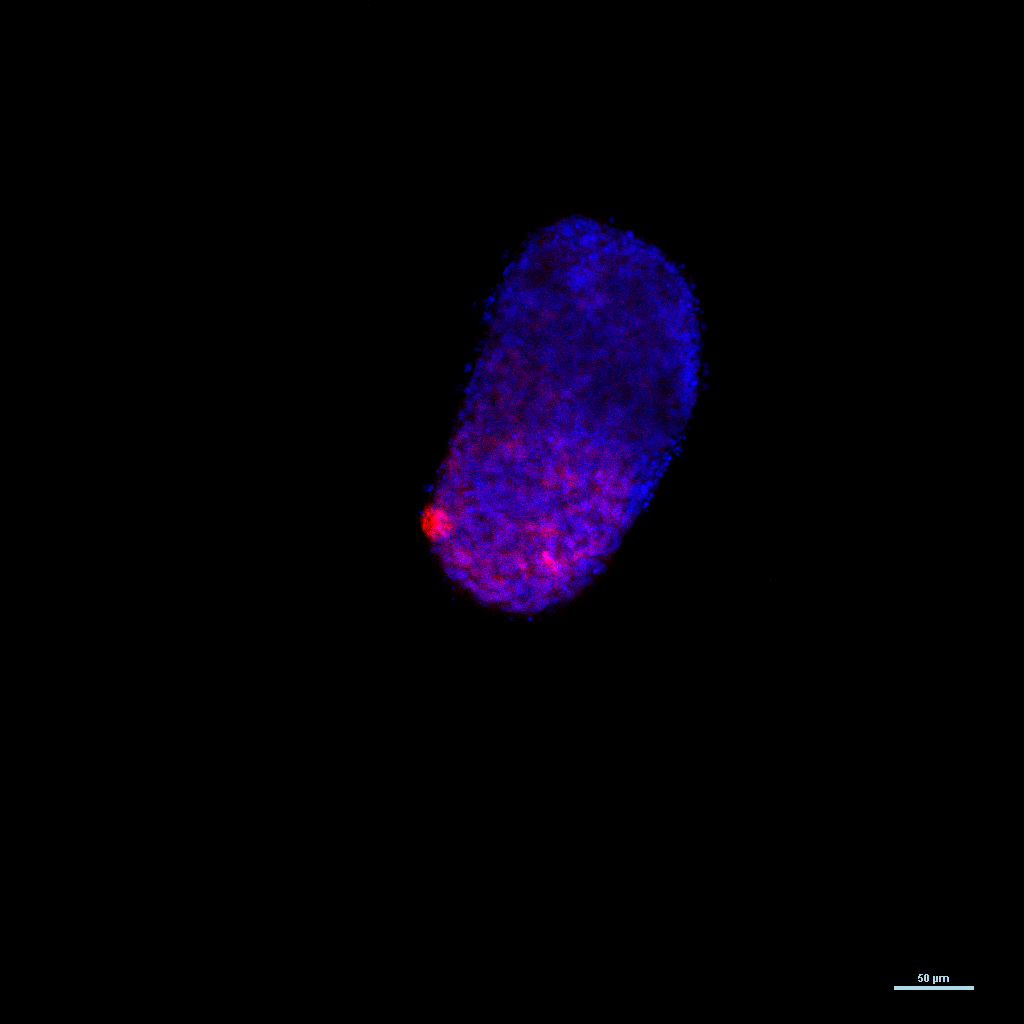

Supplement: Supplementary file 3 — Source data Fig. 1 [file 44318_2025_558_MOESM3_ESM.zip › Figure 1/panel 1F/KD-1_Cdx2/seq8792_seq8792_RGB.tif]

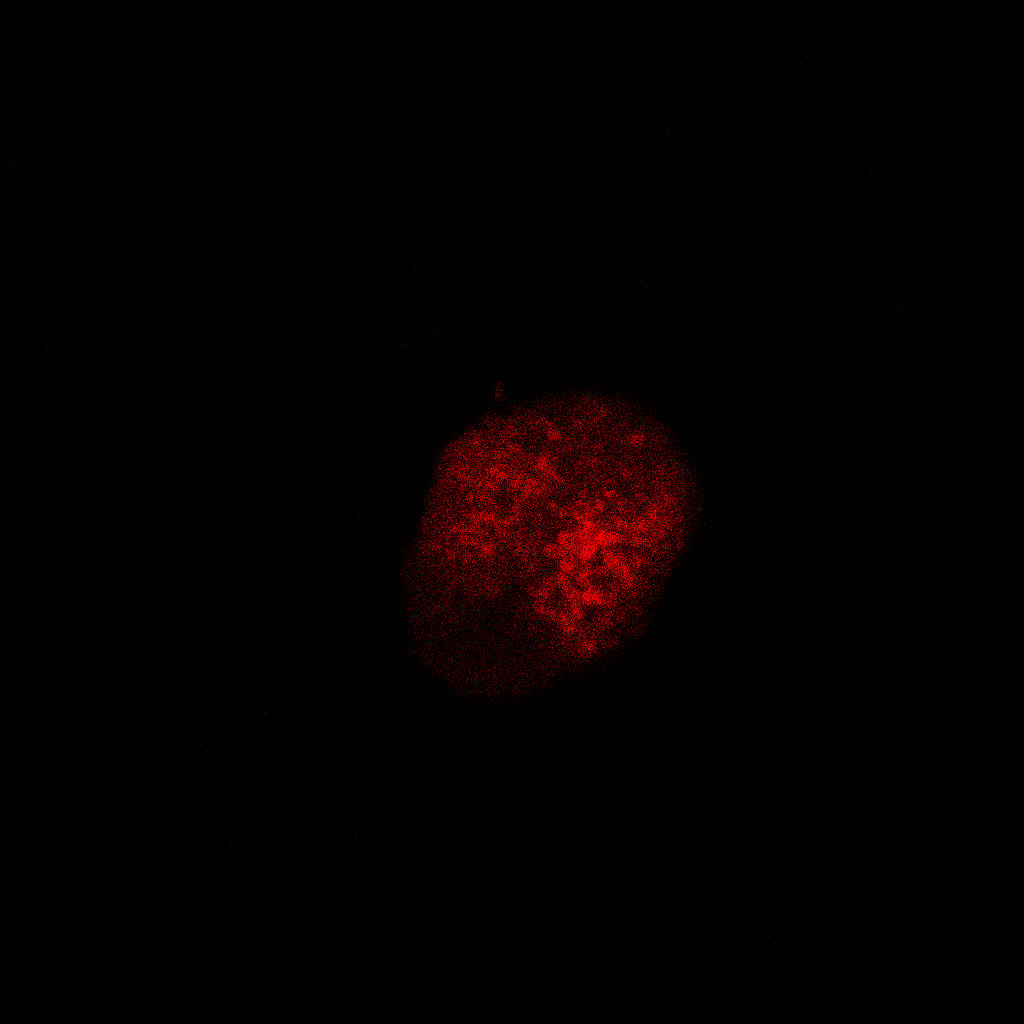

Supplement: Supplementary file 3 — Source data Fig. 1 [file 44318_2025_558_MOESM3_ESM.zip › Figure 1/panel 1F/KD-1_Sox2/image0035_image0035_RGB_Cy3 dyeΓÇôlabeled IgG antibody_pH 7.2.tif]

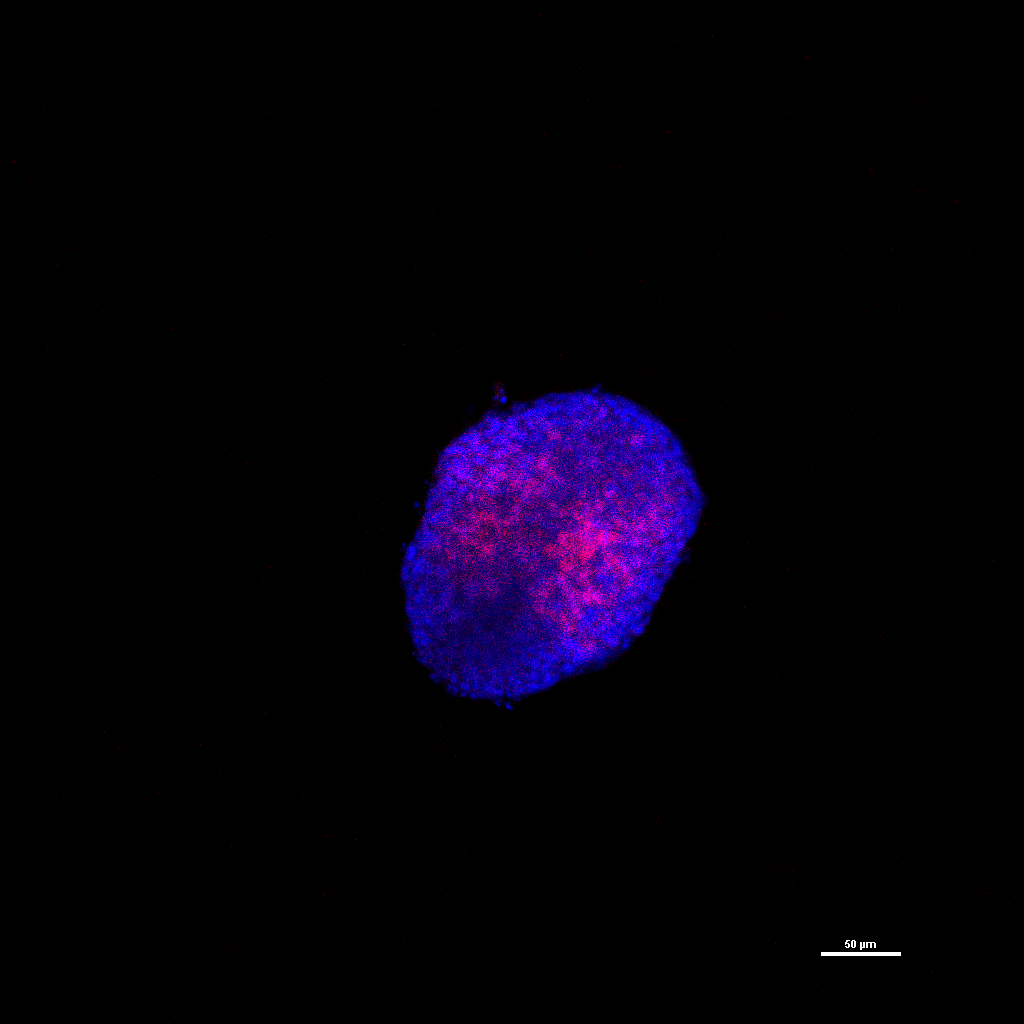

Supplement: Supplementary file 3 — Source data Fig. 1 [file 44318_2025_558_MOESM3_ESM.zip › Figure 1/panel 1F/KD-1_Sox2/image0035_image0035_RGB.tif]

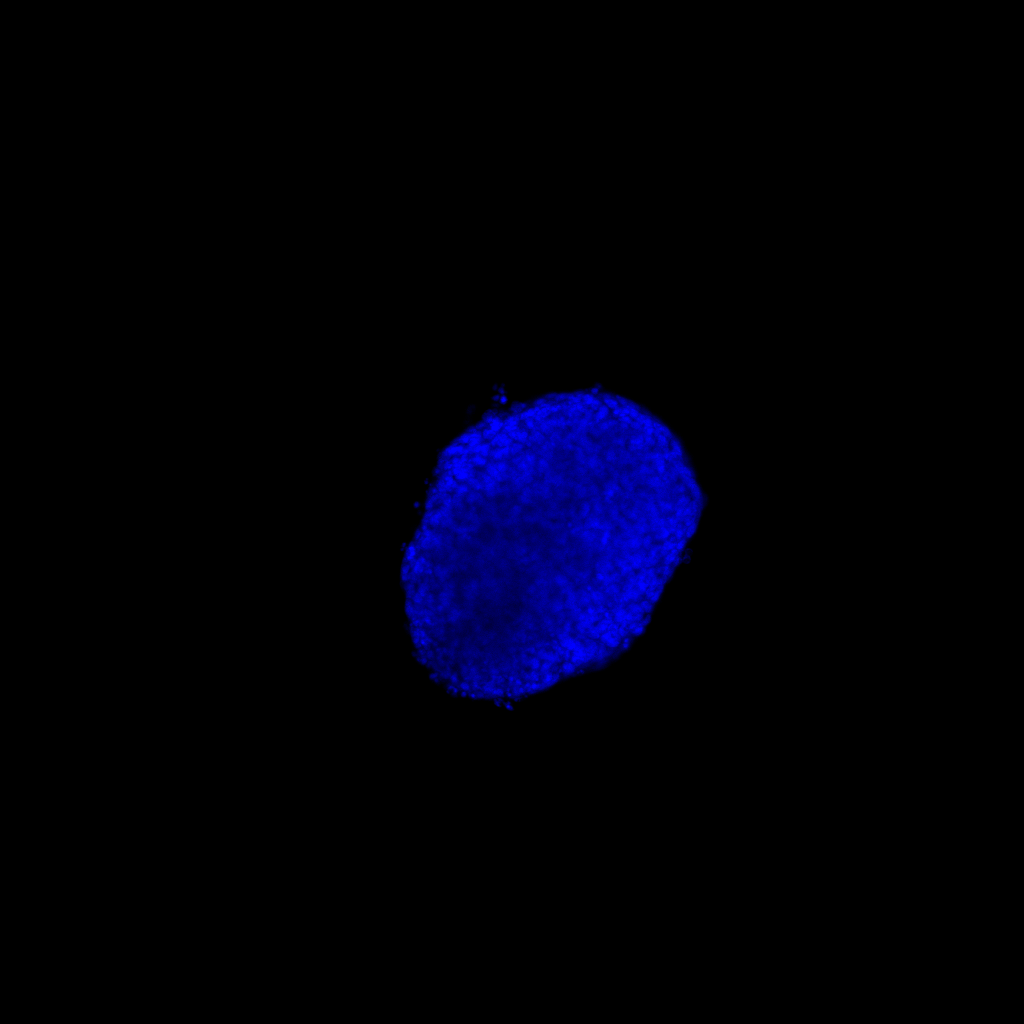

Supplement: Supplementary file 3 — Source data Fig. 1 [file 44318_2025_558_MOESM3_ESM.zip › Figure 1/panel 1F/KD-1_Sox2/image0035_image0035_RGB_DAPI.tif]

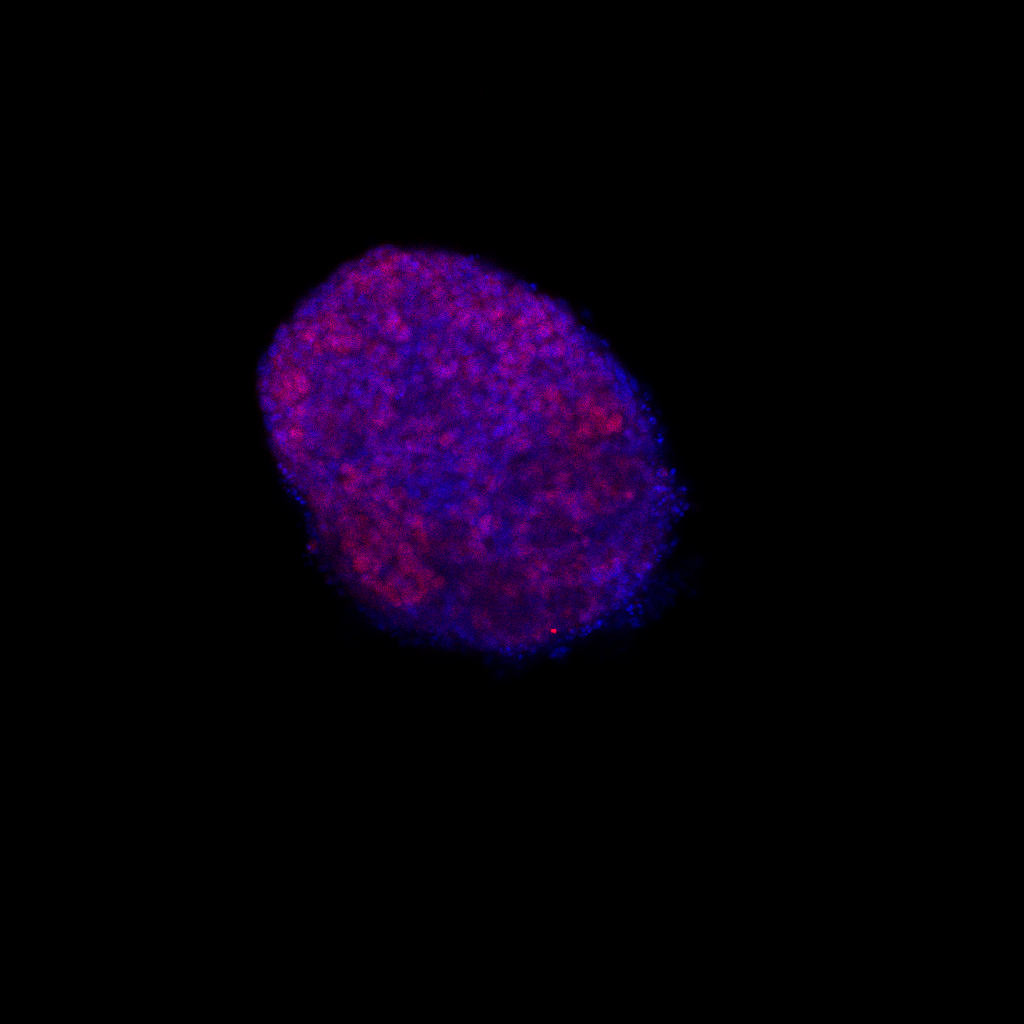

Supplement: Supplementary file 3 — Source data Fig. 1 [file 44318_2025_558_MOESM3_ESM.zip › Figure 1/panel 1F/KD-1_Oct4/seq8691.tif]

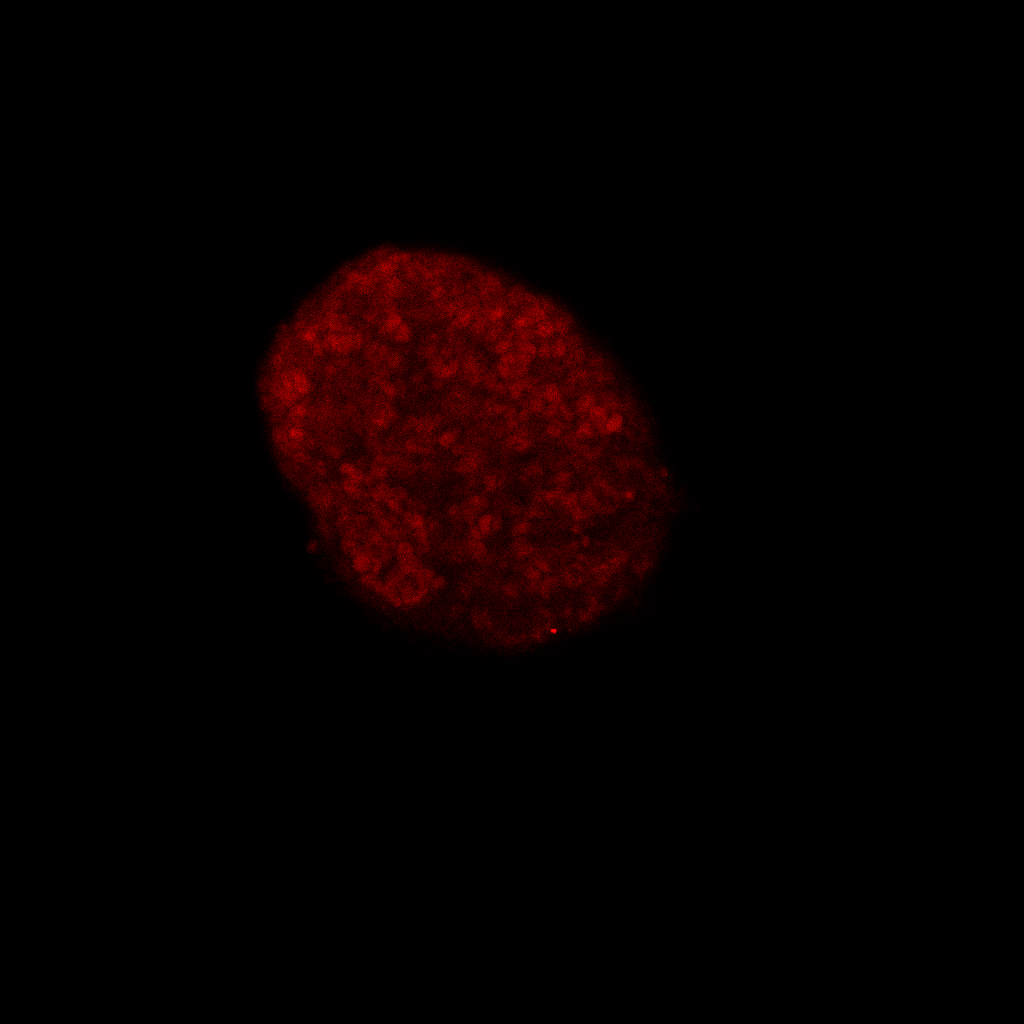

Supplement: Supplementary file 3 — Source data Fig. 1 [file 44318_2025_558_MOESM3_ESM.zip › Figure 1/panel 1F/KD-1_Oct4/seq8691c2.tif]

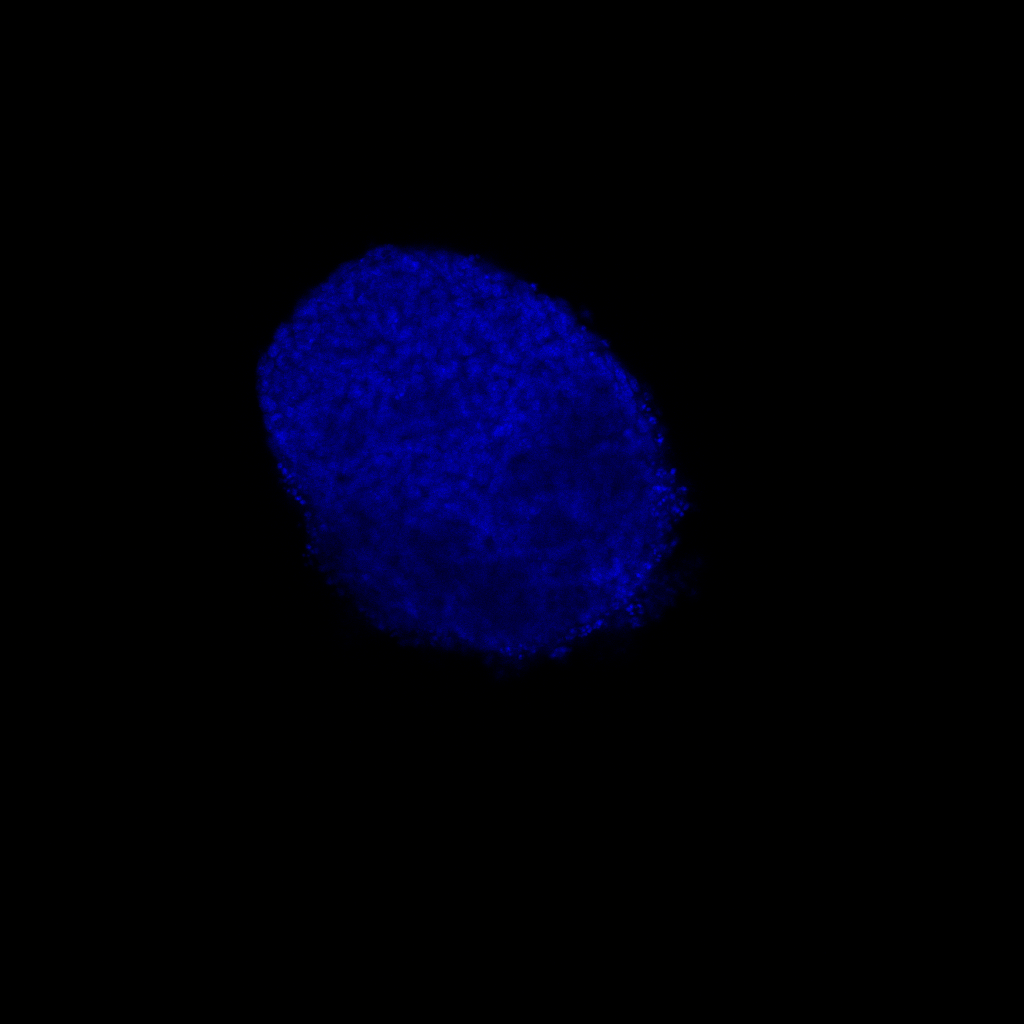

Supplement: Supplementary file 3 — Source data Fig. 1 [file 44318_2025_558_MOESM3_ESM.zip › Figure 1/panel 1F/KD-1_Oct4/seq8691c1.tif]

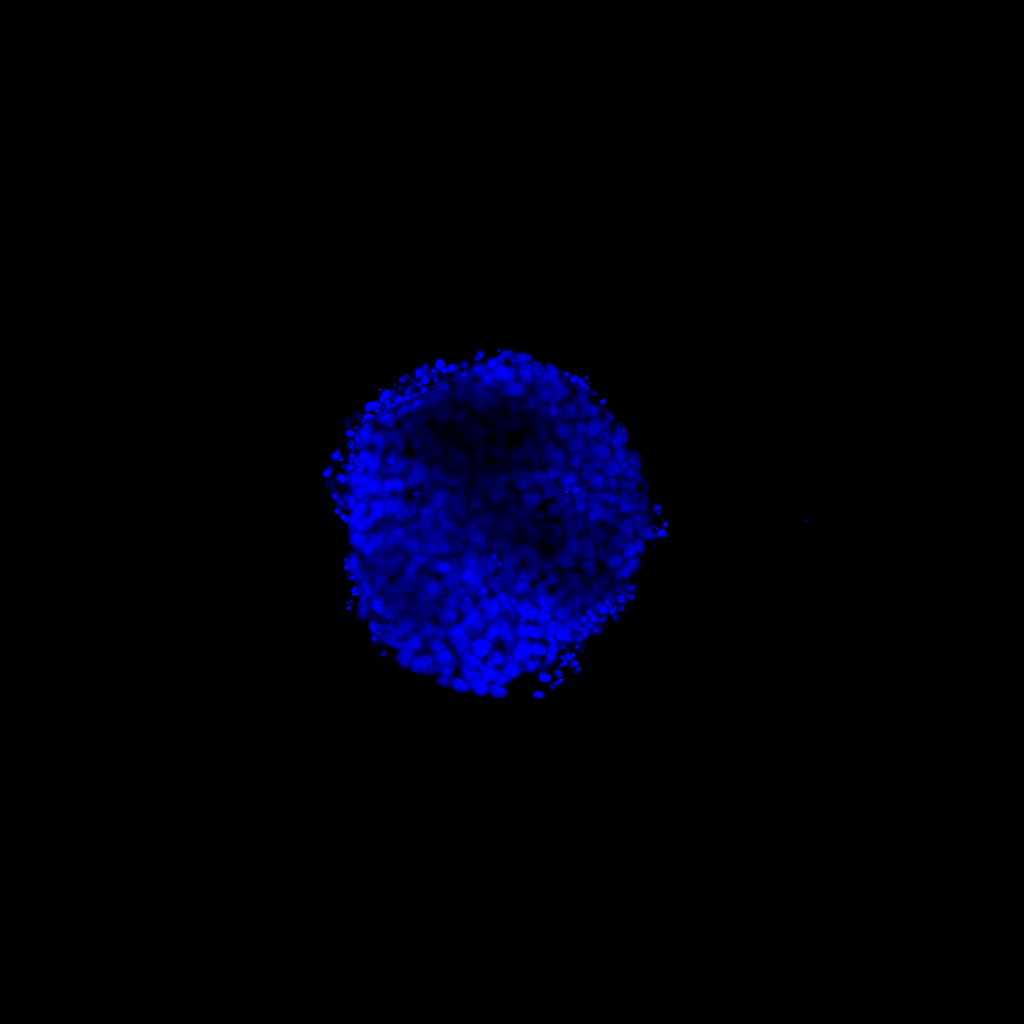

Supplement: Supplementary file 3 — Source data Fig. 1 [file 44318_2025_558_MOESM3_ESM.zip › Figure 1/panel 1F/KD-1_Bra:e-cadh/seq11055_seq11055_RGB_DAPI.tif]

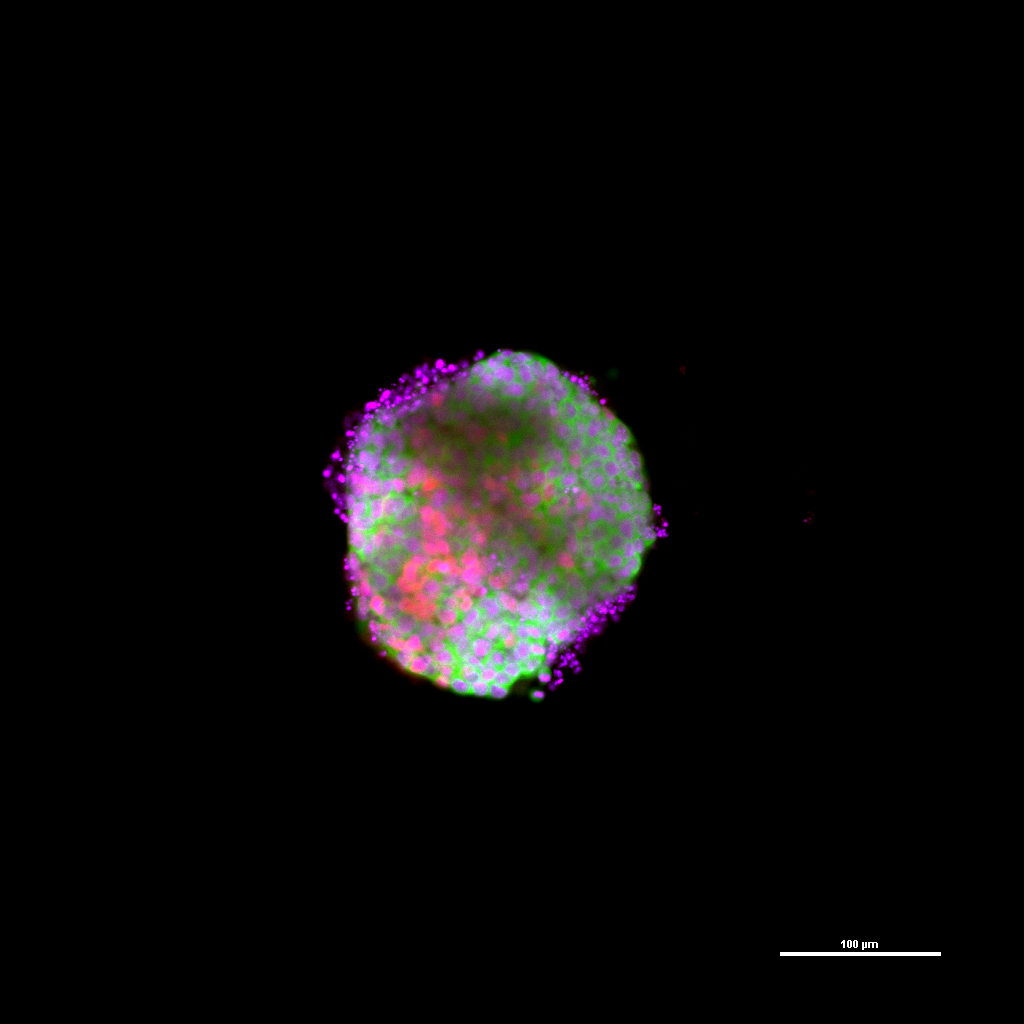

Supplement: Supplementary file 3 — Source data Fig. 1 [file 44318_2025_558_MOESM3_ESM.zip › Figure 1/panel 1F/KD-1_Bra:e-cadh/seq11055_seq11055_RGB.tif]

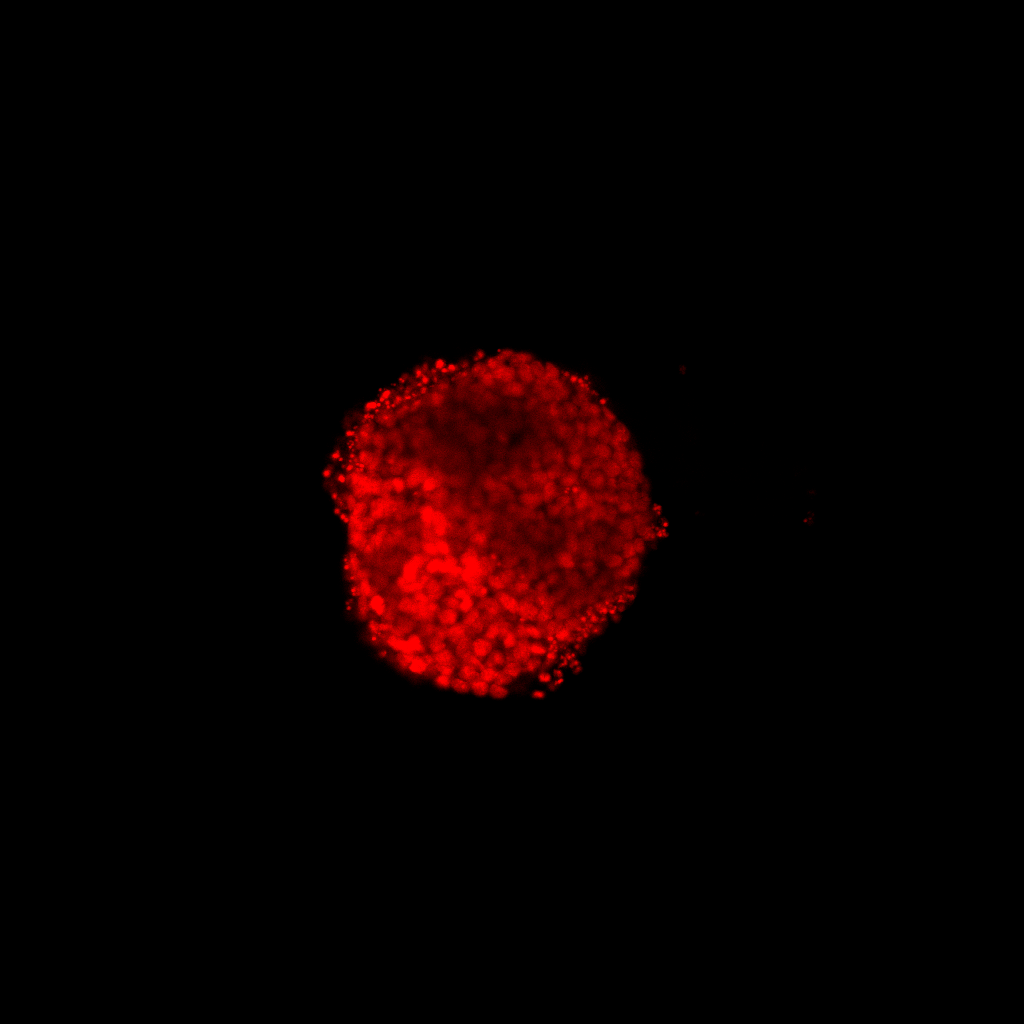

Supplement: Supplementary file 3 — Source data Fig. 1 [file 44318_2025_558_MOESM3_ESM.zip › Figure 1/panel 1F/KD-1_Bra:e-cadh/seq11055_seq11055_RGB_TRITC.tif]

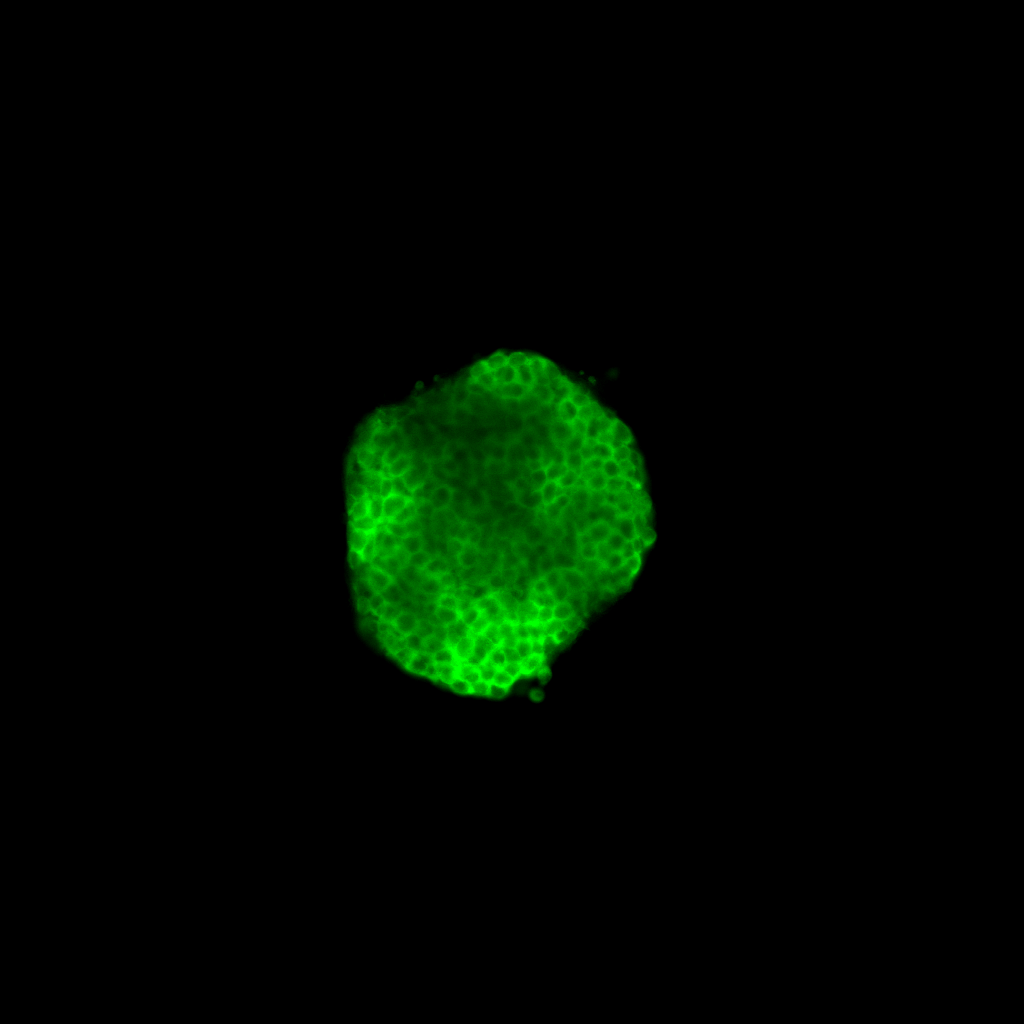

Supplement: Supplementary file 3 — Source data Fig. 1 [file 44318_2025_558_MOESM3_ESM.zip › Figure 1/panel 1F/KD-1_Bra:e-cadh/seq11055_seq11055_RGB_FITC.tif]

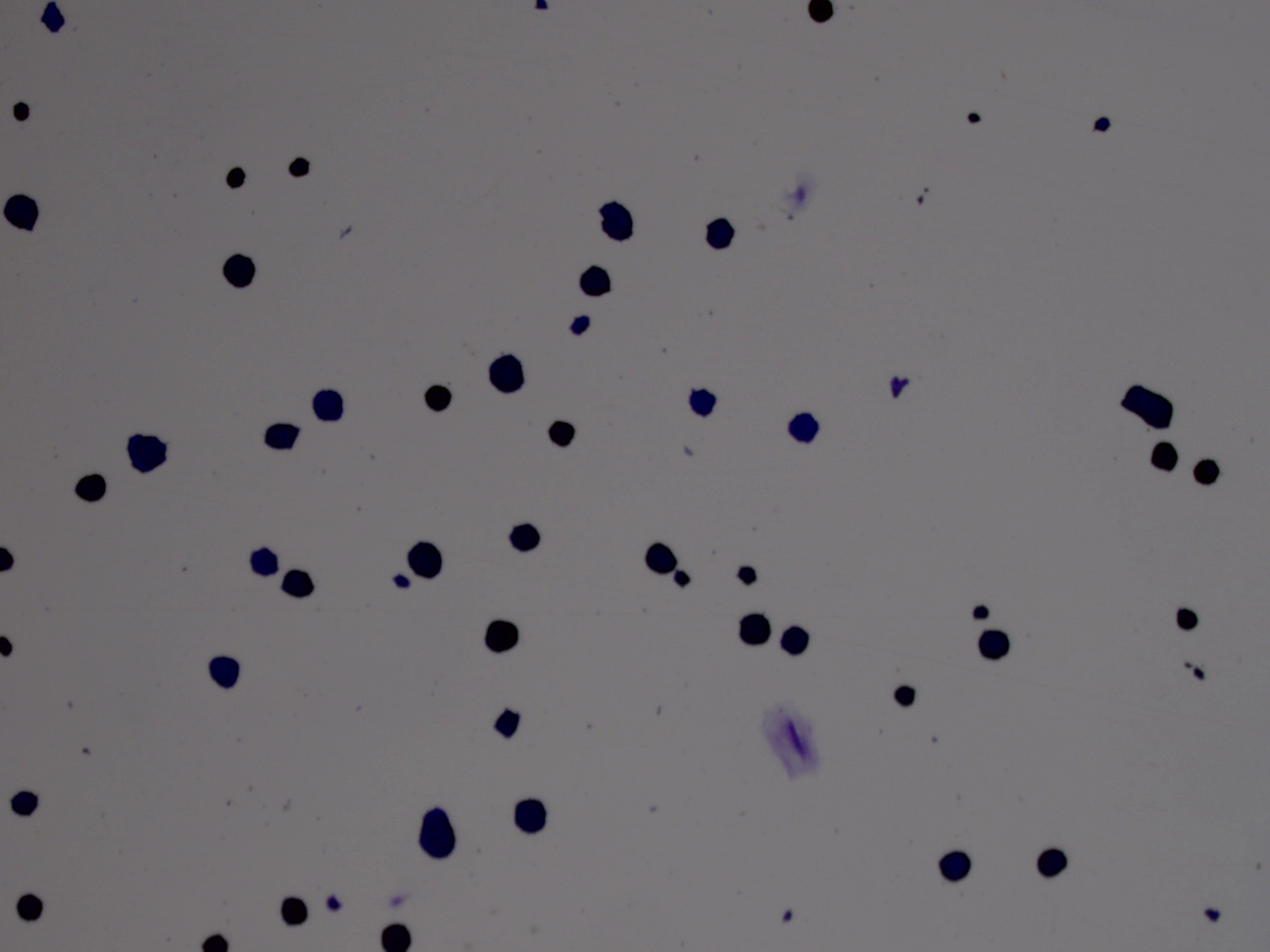

Supplement: Supplementary file 4 — Source data Fig. 2 [file 44318_2025_558_MOESM4_ESM.zip › Figure 2/panel 2B/KD-2.tiff]

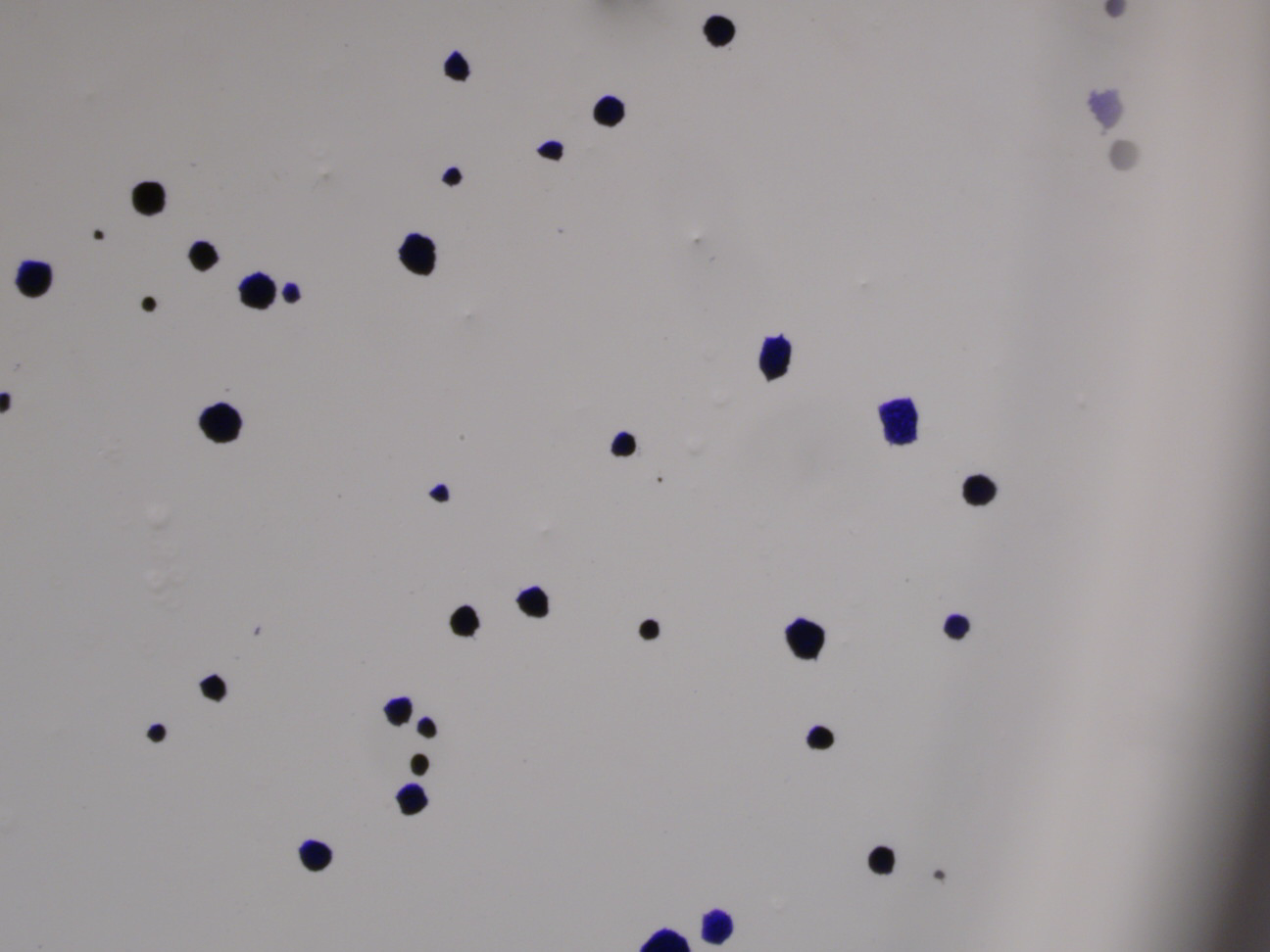

Supplement: Supplementary file 4 — Source data Fig. 2 [file 44318_2025_558_MOESM4_ESM.zip › Figure 2/panel 2B/NT.tiff]

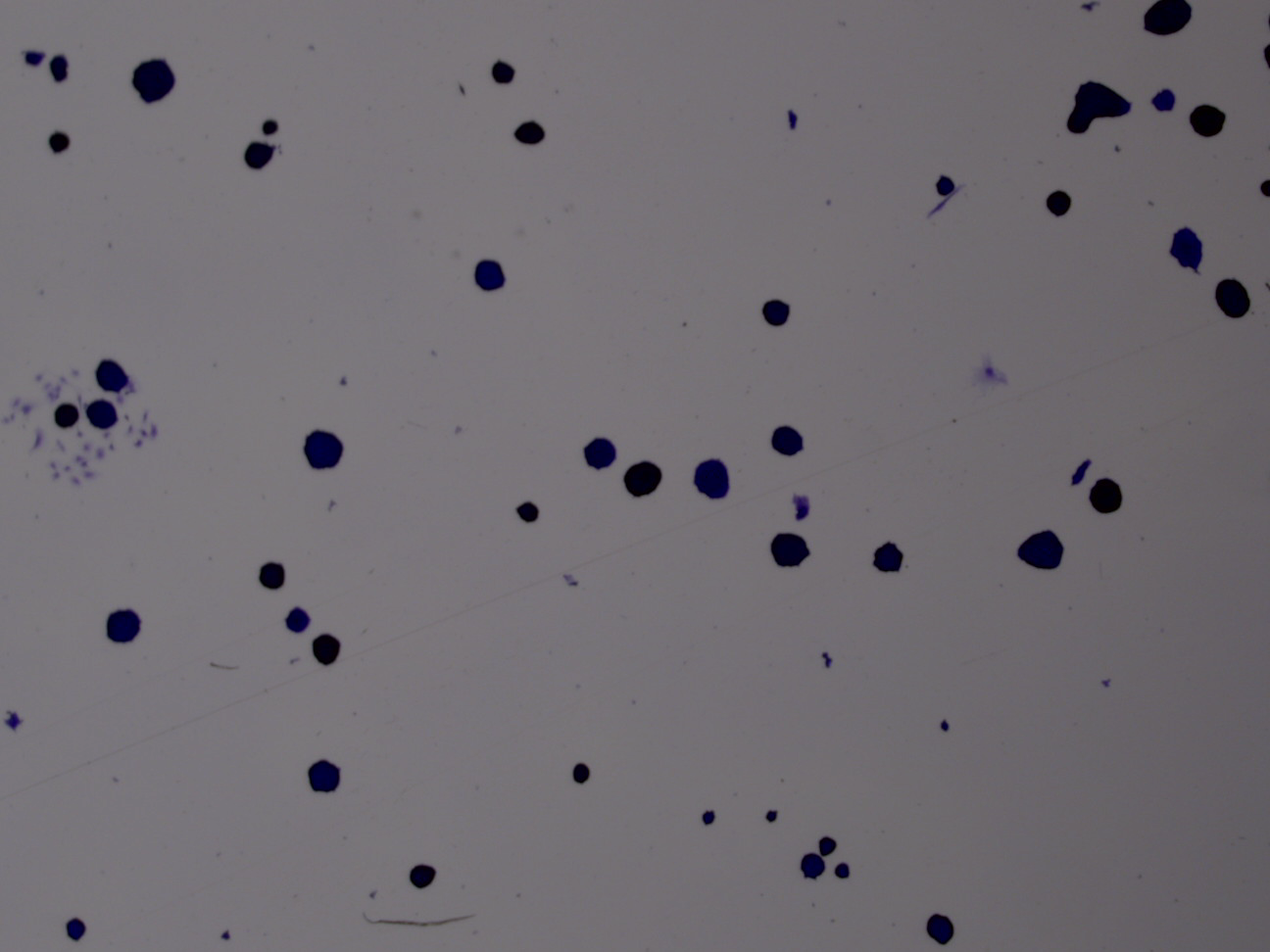

Supplement: Supplementary file 4 — Source data Fig. 2 [file 44318_2025_558_MOESM4_ESM.zip › Figure 2/panel 2B/KD-1.tiff]

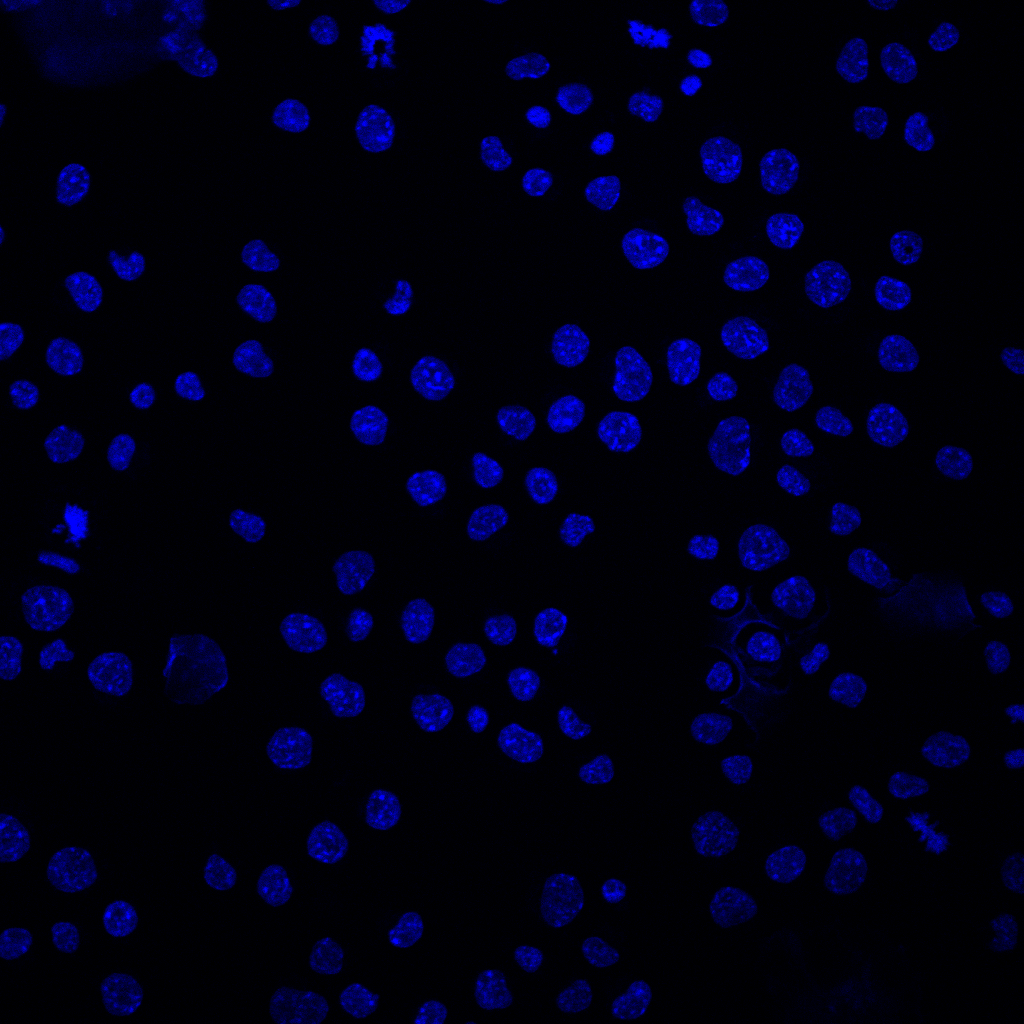

Supplement: Supplementary file 4 — Source data Fig. 2 [file 44318_2025_558_MOESM4_ESM.zip › Figure 2/panel 2C/NT_Cdx2/seq9707_seq9707_RGB_DAPI.tif]
