# Supplementary figures and images for: Non-cell-autonomous control of mouse gastruloid development by the ultra-conserved lncRNA T-UCstem1 (part 2 of 6)
Source: EMBO J. 2025 Oct 31;44(24):7620–48. doi: 10.1038/s44318-025-00558-2 (PMC12706062; doi:10.1038/s44318-025-00558-2)

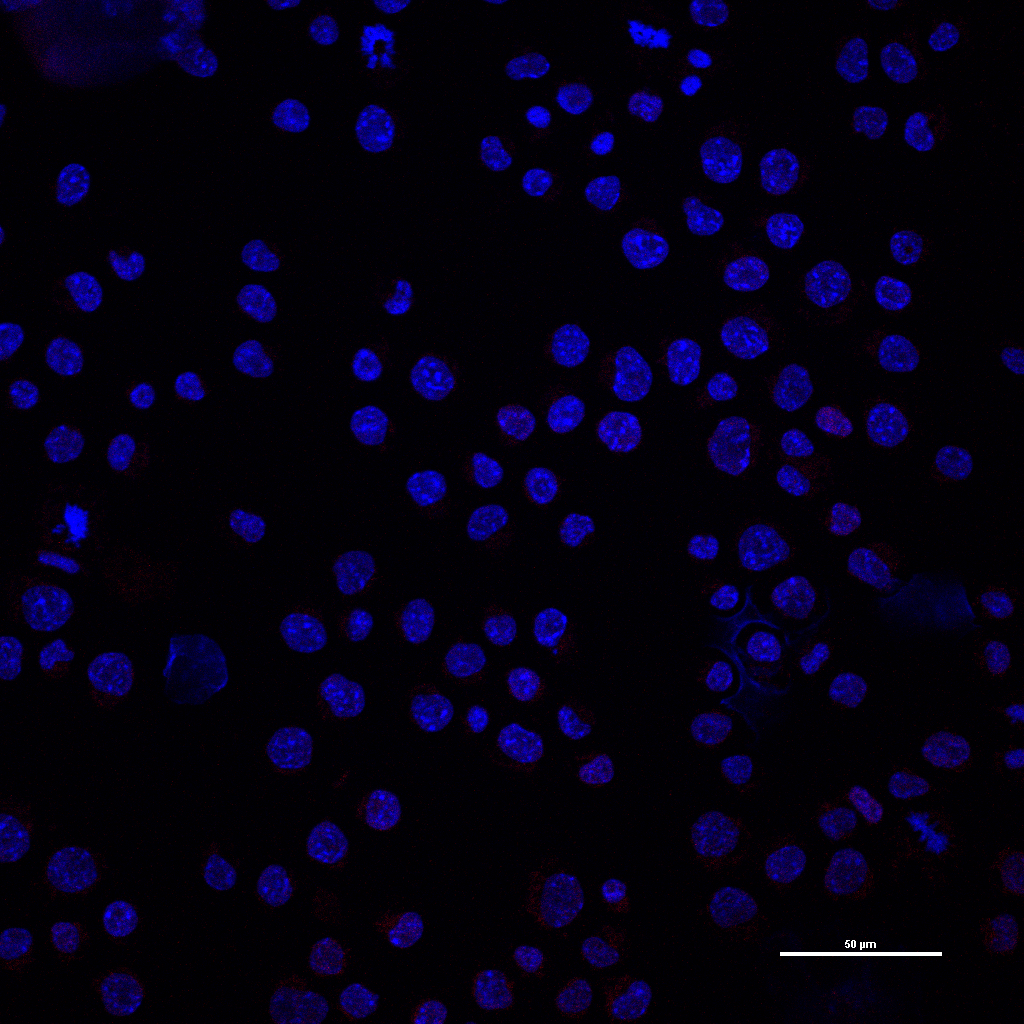

Supplement: Supplementary file 4 — Source data Fig. 2 [file 44318_2025_558_MOESM4_ESM.zip › Figure 2/panel 2C/NT_Cdx2/seq9707_seq9707_RGB.tif]

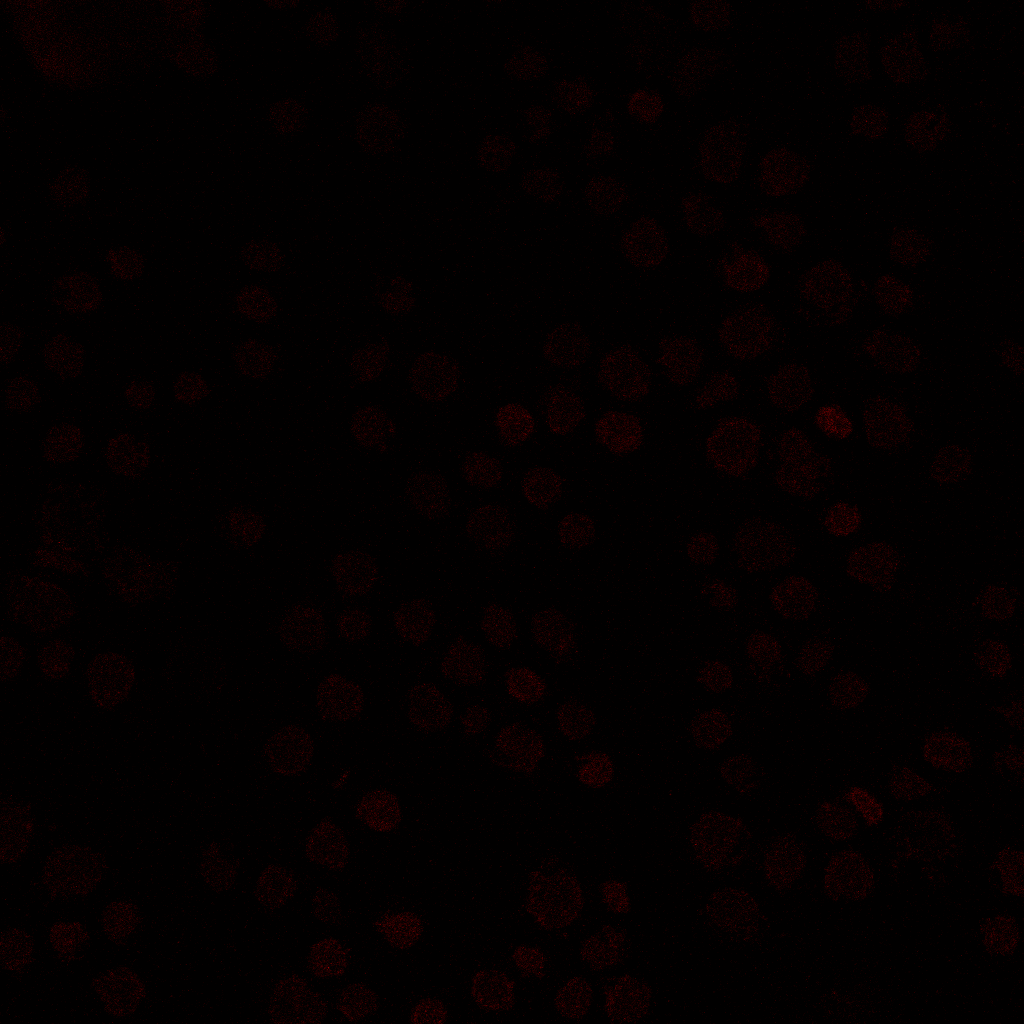

Supplement: Supplementary file 4 — Source data Fig. 2 [file 44318_2025_558_MOESM4_ESM.zip › Figure 2/panel 2C/NT_Cdx2/seq9707_seq9707_RGB_Texas Red.tif]

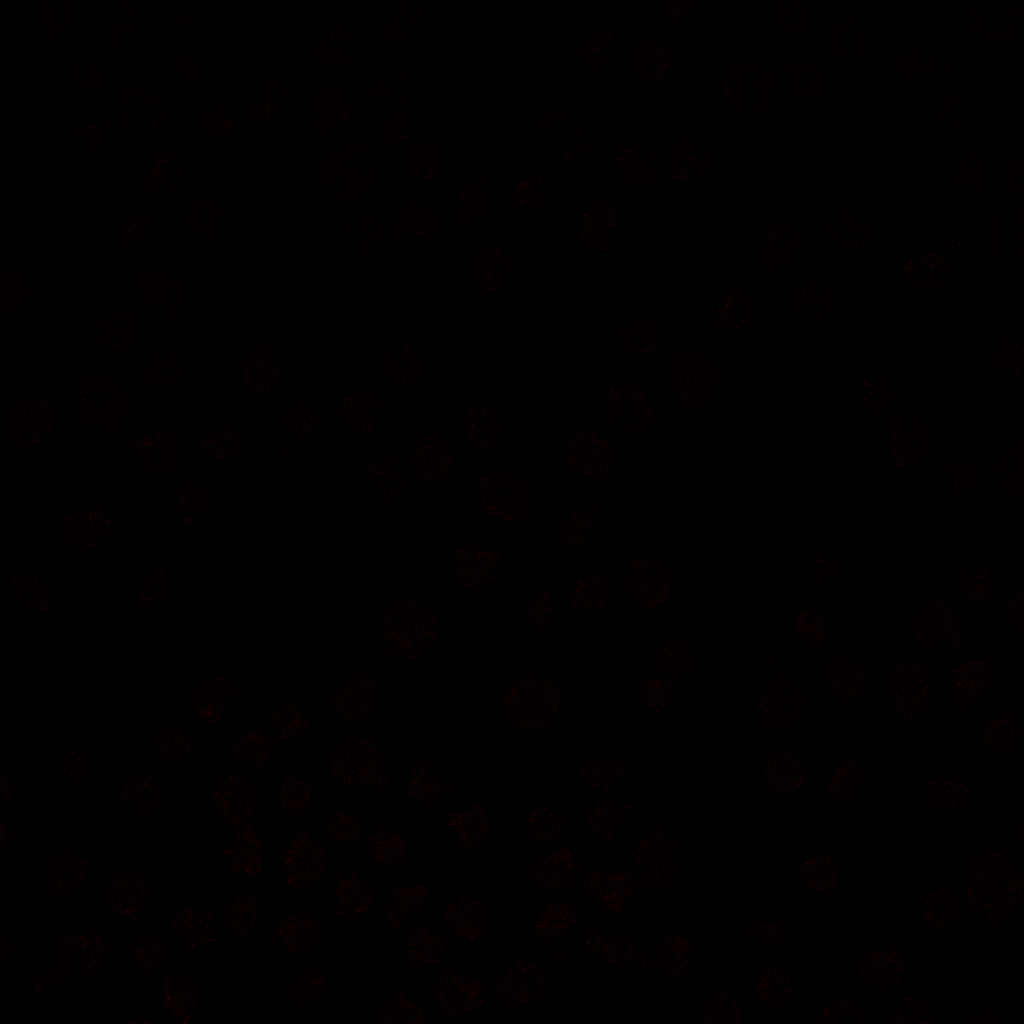

Supplement: Supplementary file 4 — Source data Fig. 2 [file 44318_2025_558_MOESM4_ESM.zip › Figure 2/panel 2C/KD-1_Bra/seq9703_seq9703_RGB_Texas Red.tif]

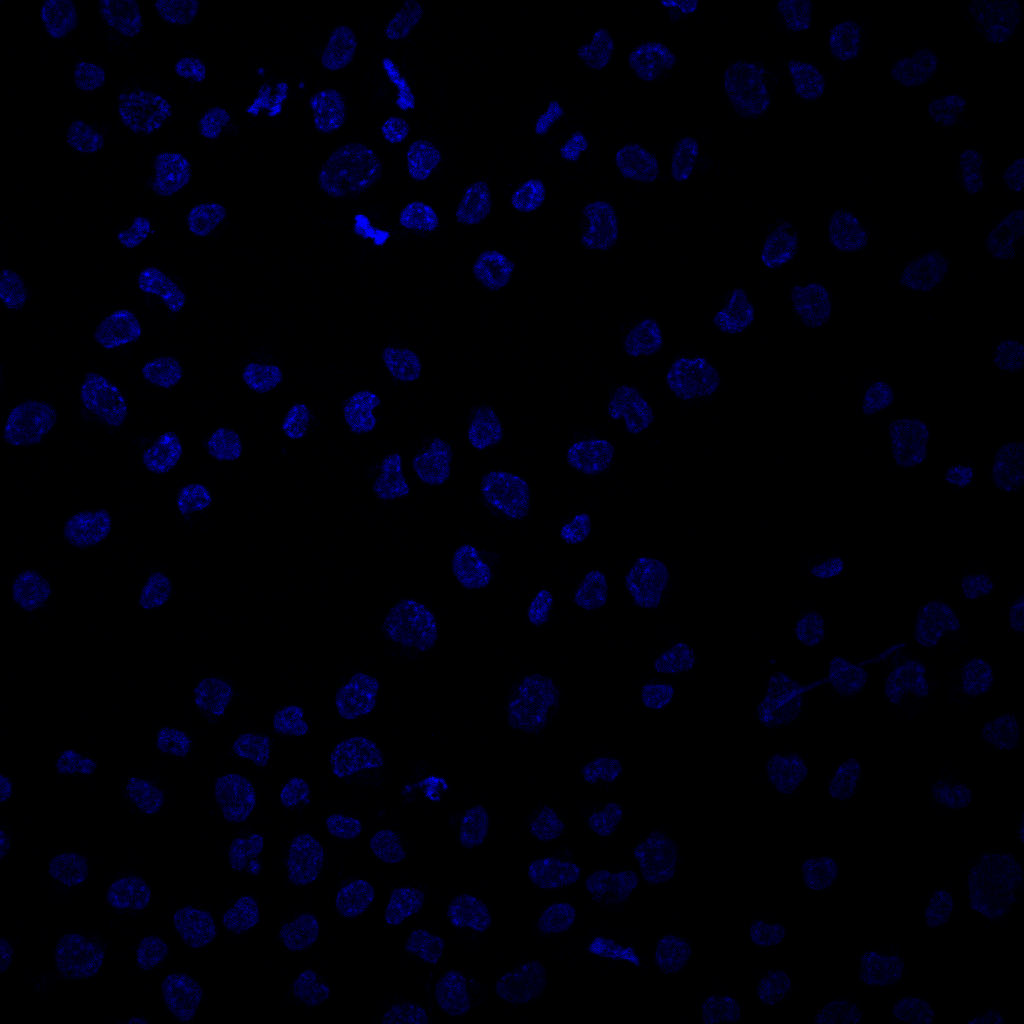

Supplement: Supplementary file 4 — Source data Fig. 2 [file 44318_2025_558_MOESM4_ESM.zip › Figure 2/panel 2C/KD-1_Bra/seq9703_seq9703_RGB_DAPI.tif]

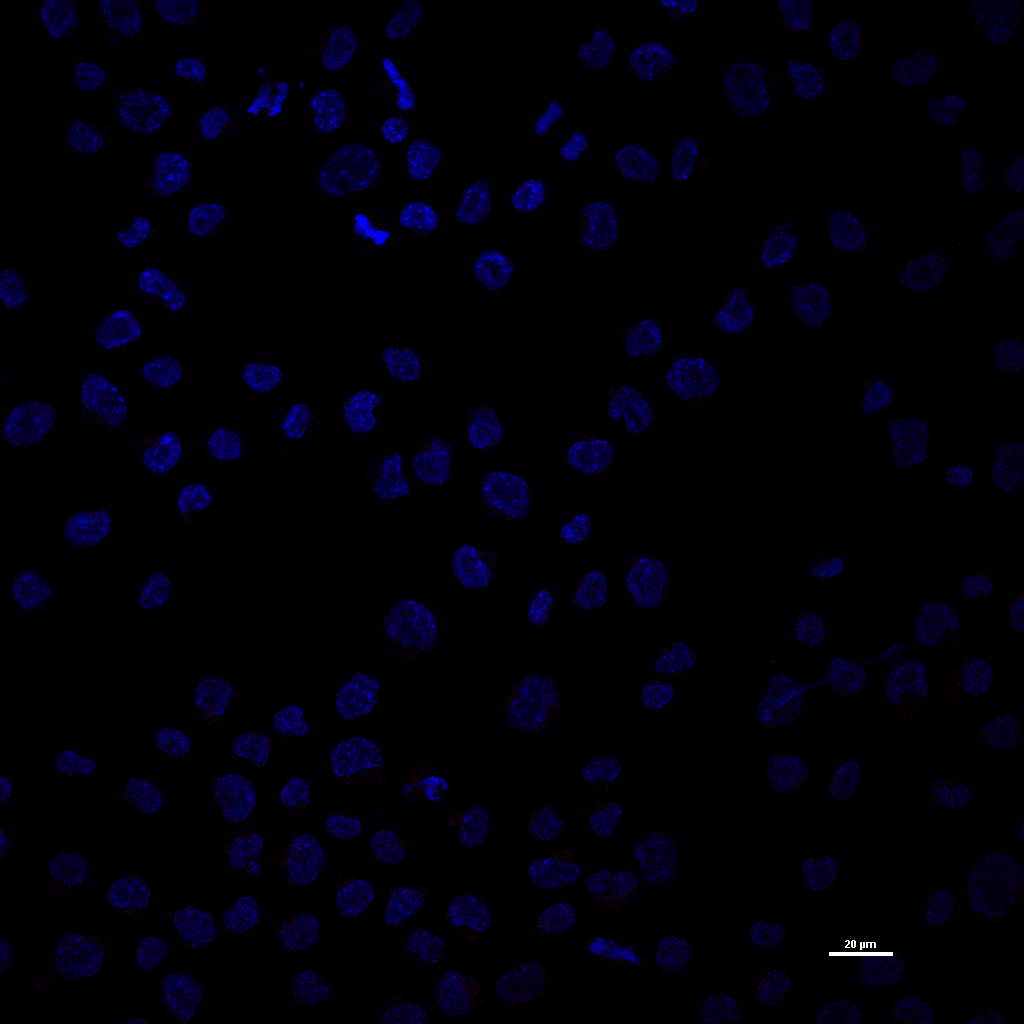

Supplement: Supplementary file 4 — Source data Fig. 2 [file 44318_2025_558_MOESM4_ESM.zip › Figure 2/panel 2C/KD-1_Bra/seq9703_seq9703_RGB.tif]

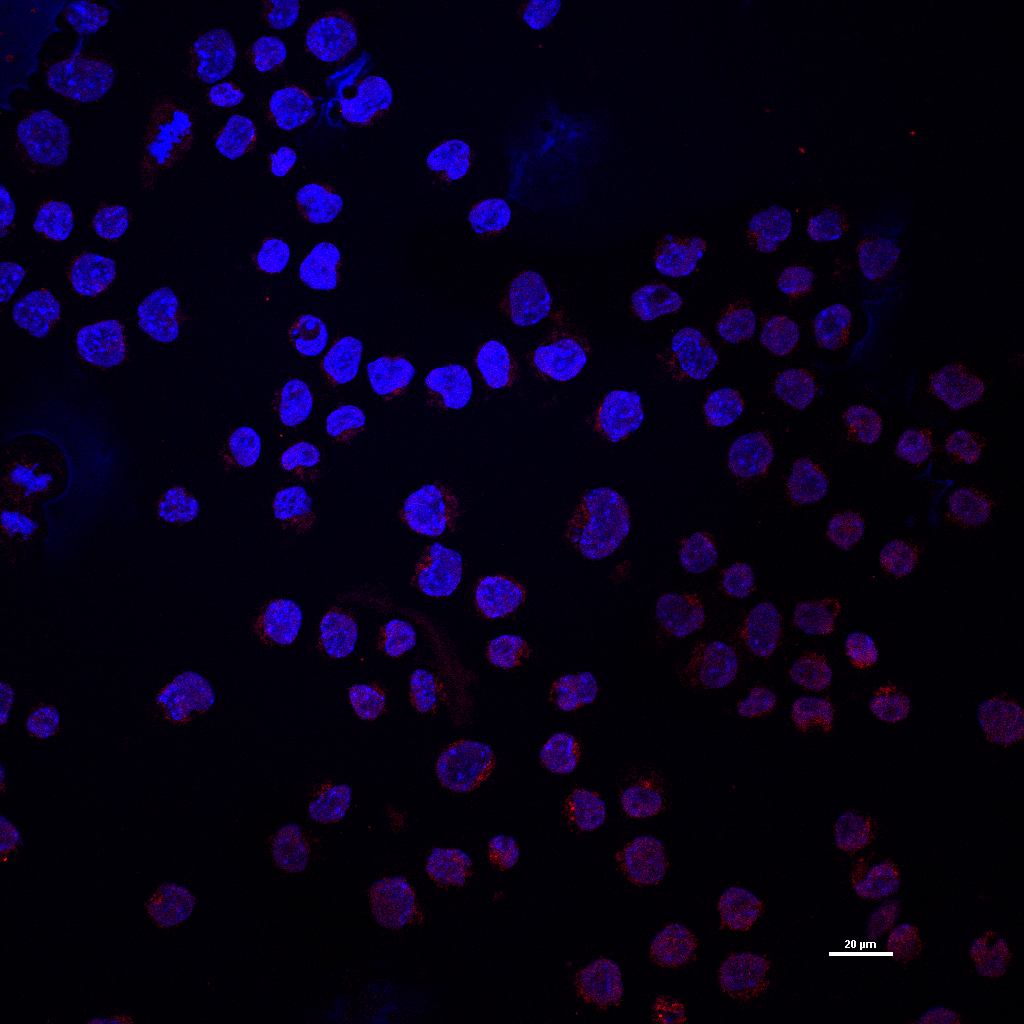

Supplement: Supplementary file 4 — Source data Fig. 2 [file 44318_2025_558_MOESM4_ESM.zip › Figure 2/panel 2C/KD-1_Sox17/seq9700_seq9700_RGB.tif]

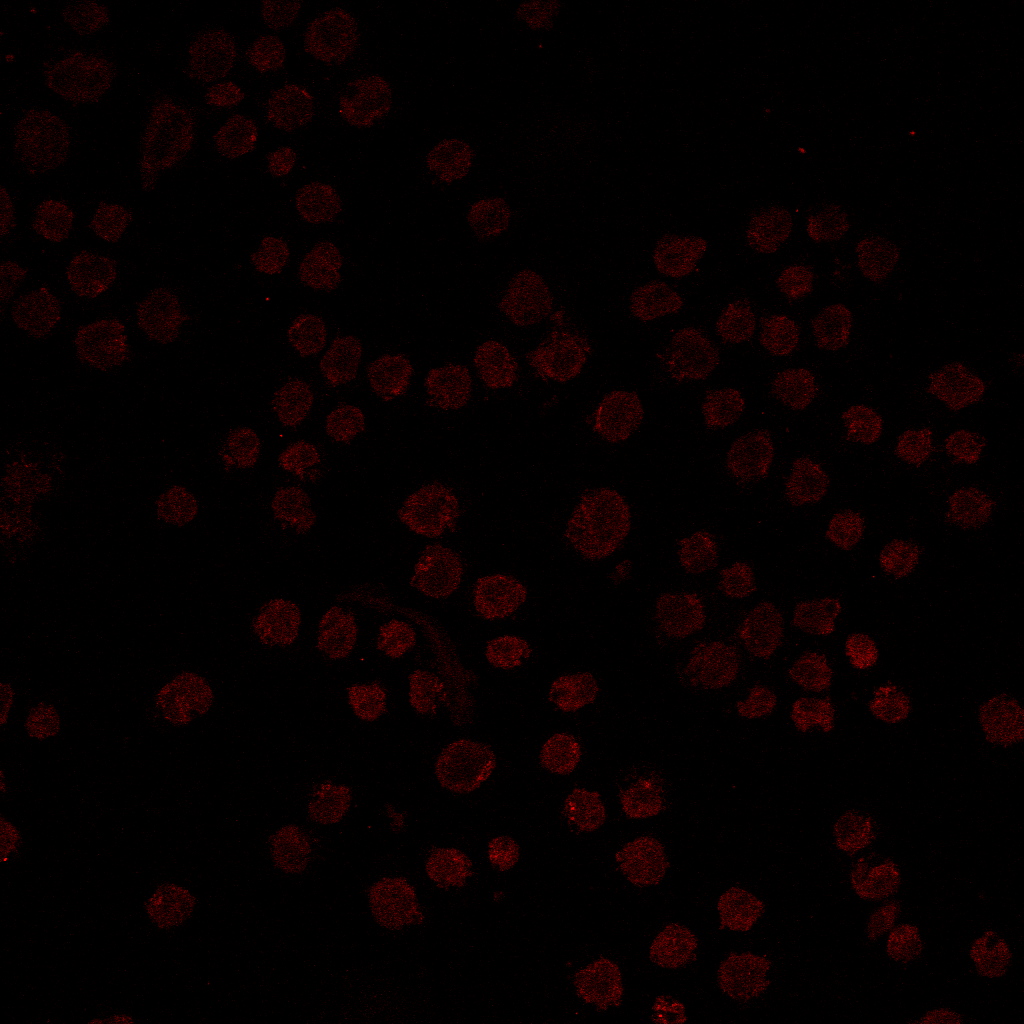

Supplement: Supplementary file 4 — Source data Fig. 2 [file 44318_2025_558_MOESM4_ESM.zip › Figure 2/panel 2C/KD-1_Sox17/seq9700_seq9700_RGB_Texas Red.tif]

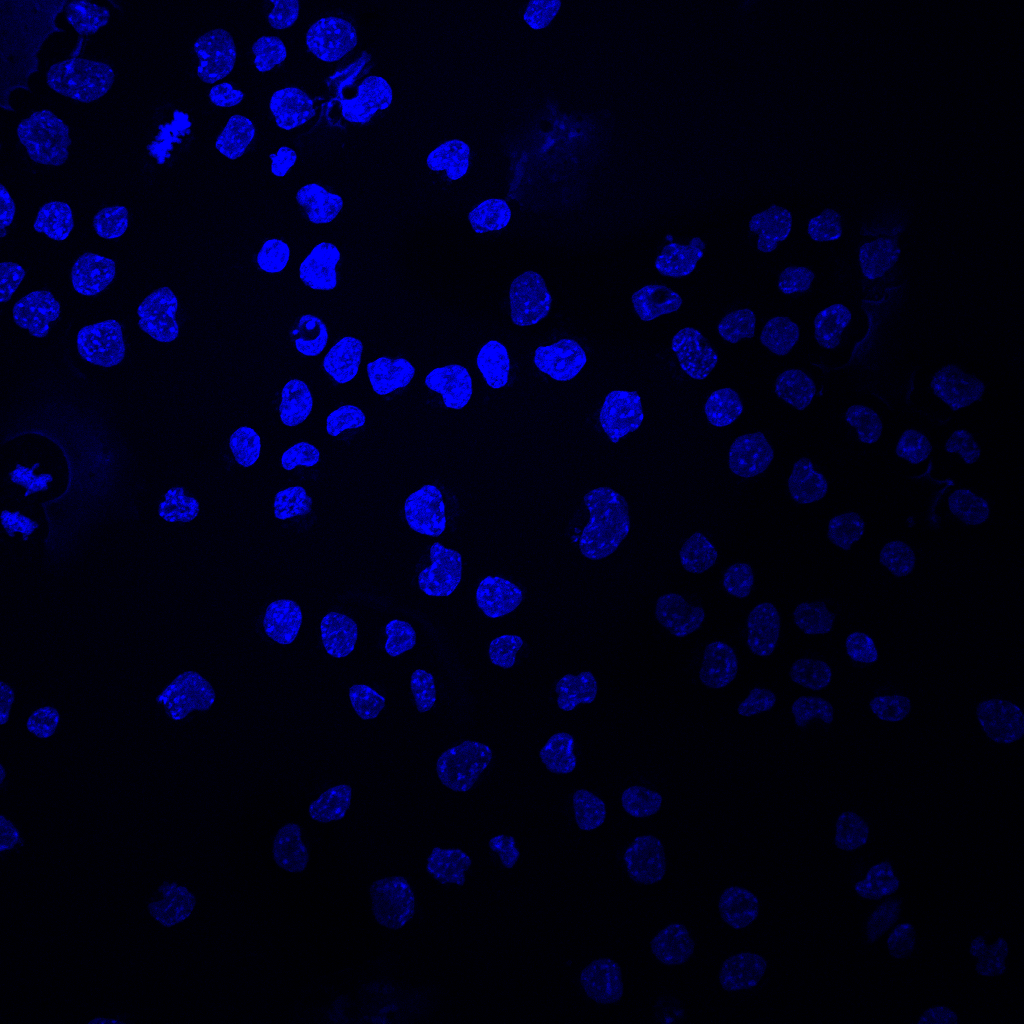

Supplement: Supplementary file 4 — Source data Fig. 2 [file 44318_2025_558_MOESM4_ESM.zip › Figure 2/panel 2C/KD-1_Sox17/seq9700_seq9700_RGB_DAPI.tif]

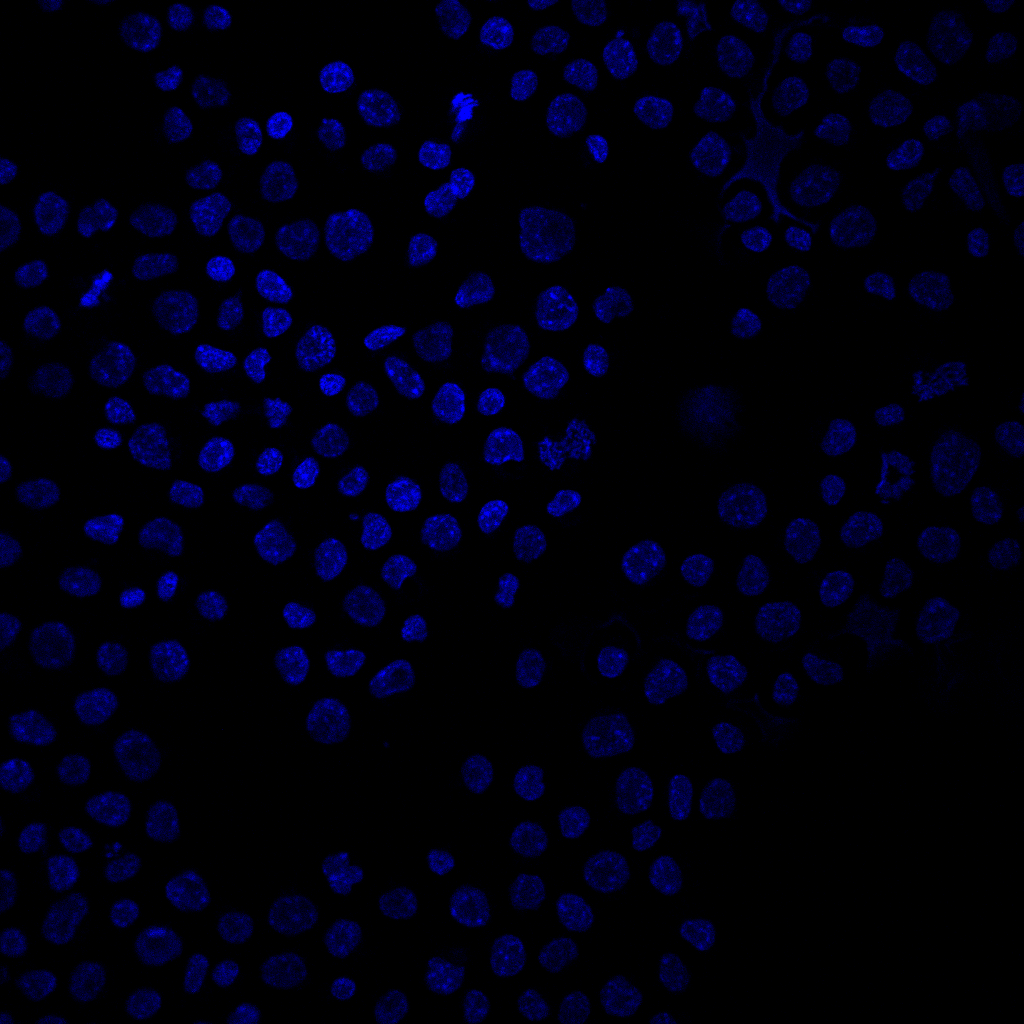

Supplement: Supplementary file 4 — Source data Fig. 2 [file 44318_2025_558_MOESM4_ESM.zip › Figure 2/panel 2C/NT_Nestin/seq9702_seq9702_RGB_DAPI.tif]

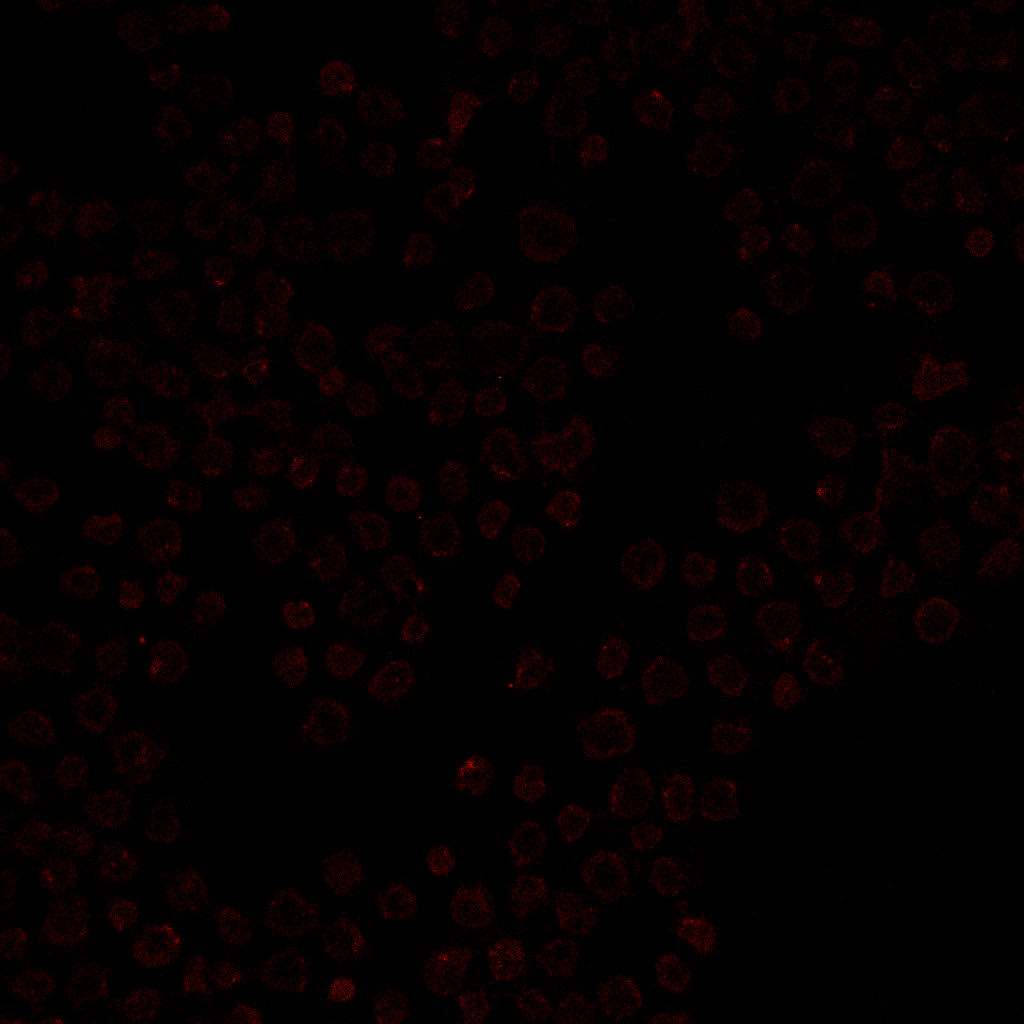

Supplement: Supplementary file 4 — Source data Fig. 2 [file 44318_2025_558_MOESM4_ESM.zip › Figure 2/panel 2C/NT_Nestin/seq9702_seq9702_RGB_Texas Red.tif]

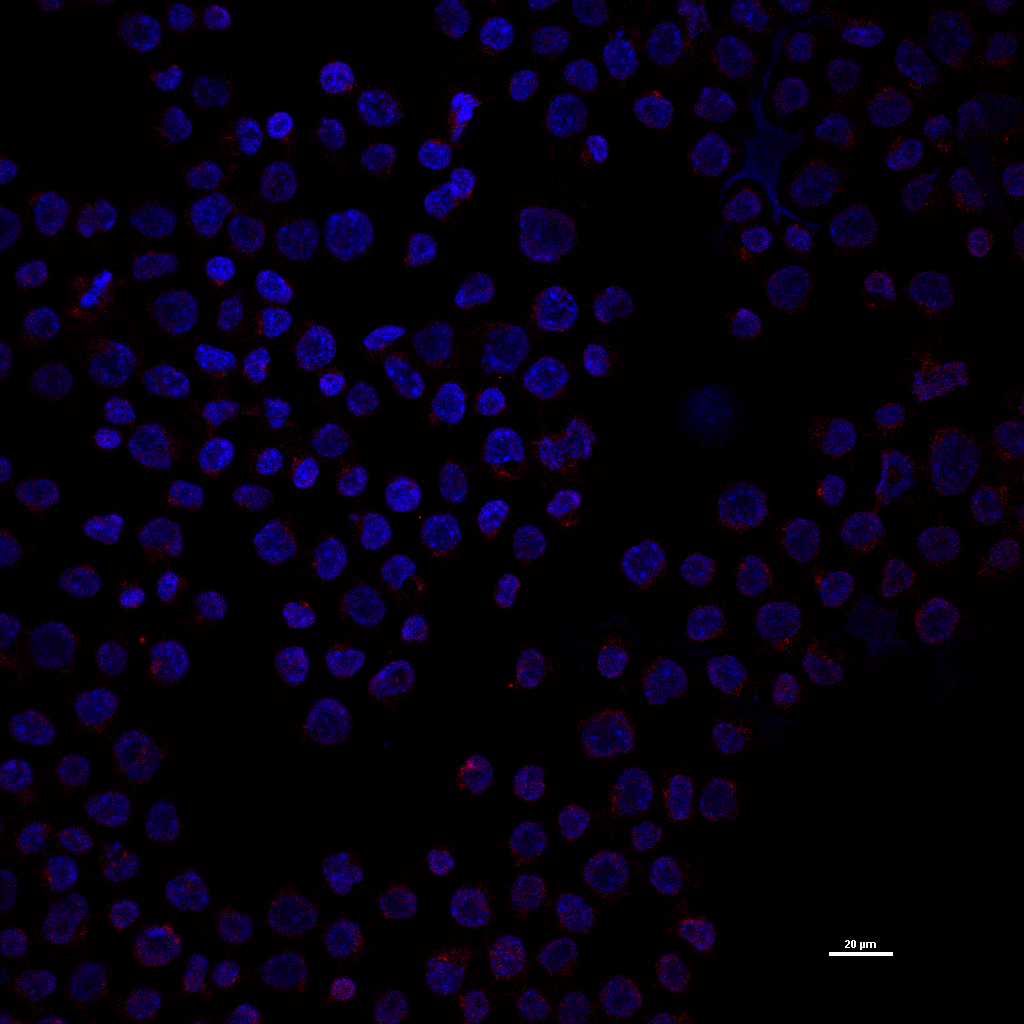

Supplement: Supplementary file 4 — Source data Fig. 2 [file 44318_2025_558_MOESM4_ESM.zip › Figure 2/panel 2C/NT_Nestin/seq9702_seq9702_RGB.tif]

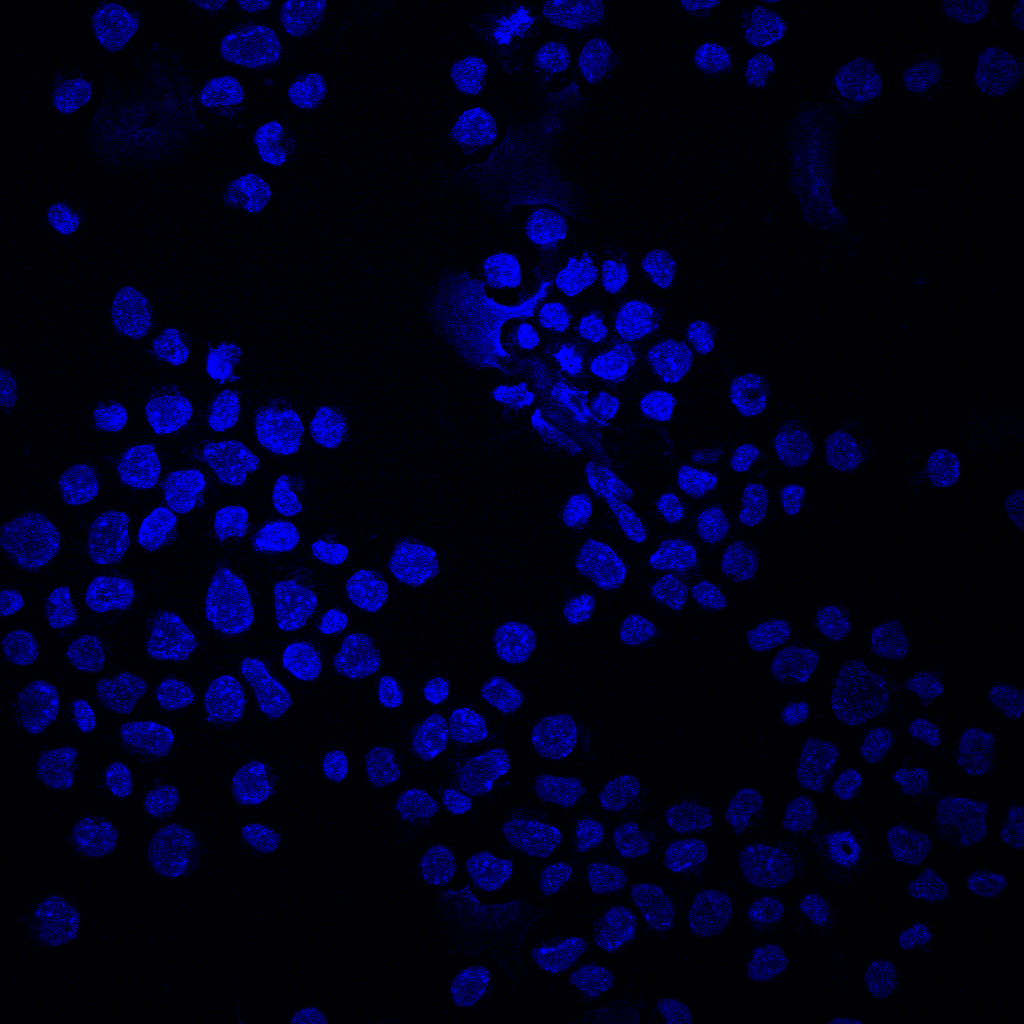

Supplement: Supplementary file 4 — Source data Fig. 2 [file 44318_2025_558_MOESM4_ESM.zip › Figure 2/panel 2C/KD-1_Nanog/seq9702_seq9702_RGB_DAPI.tif]

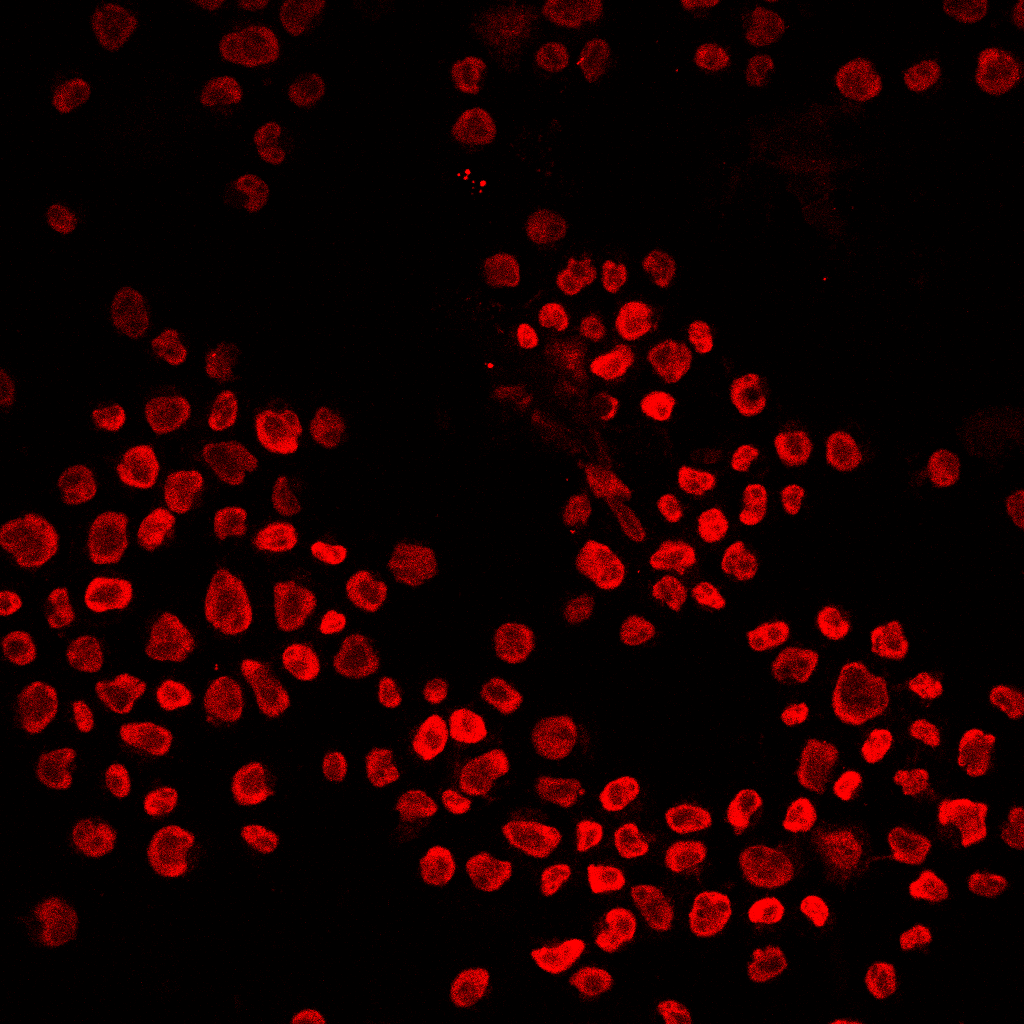

Supplement: Supplementary file 4 — Source data Fig. 2 [file 44318_2025_558_MOESM4_ESM.zip › Figure 2/panel 2C/KD-1_Nanog/seq9702_seq9702_RGB_Texas Red.tif]

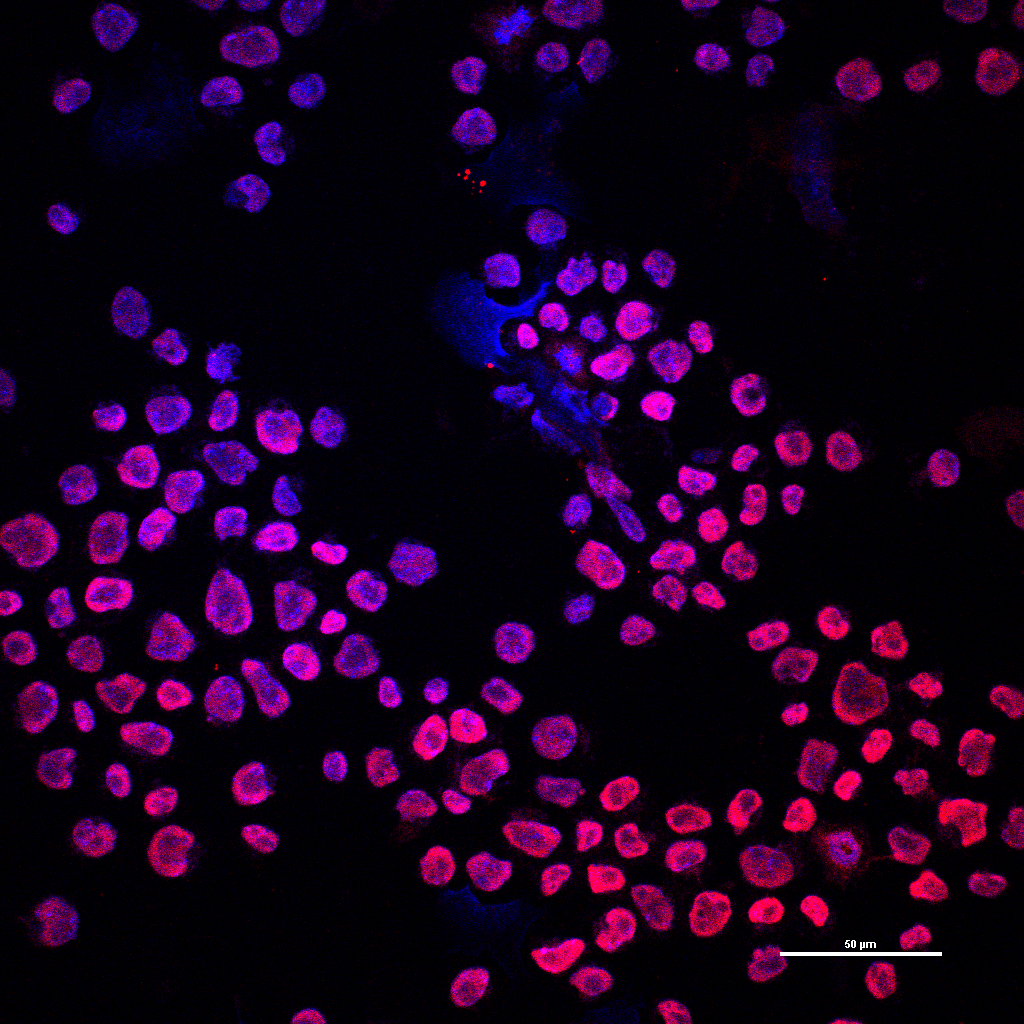

Supplement: Supplementary file 4 — Source data Fig. 2 [file 44318_2025_558_MOESM4_ESM.zip › Figure 2/panel 2C/KD-1_Nanog/seq9702_seq9702_RGB.tif]

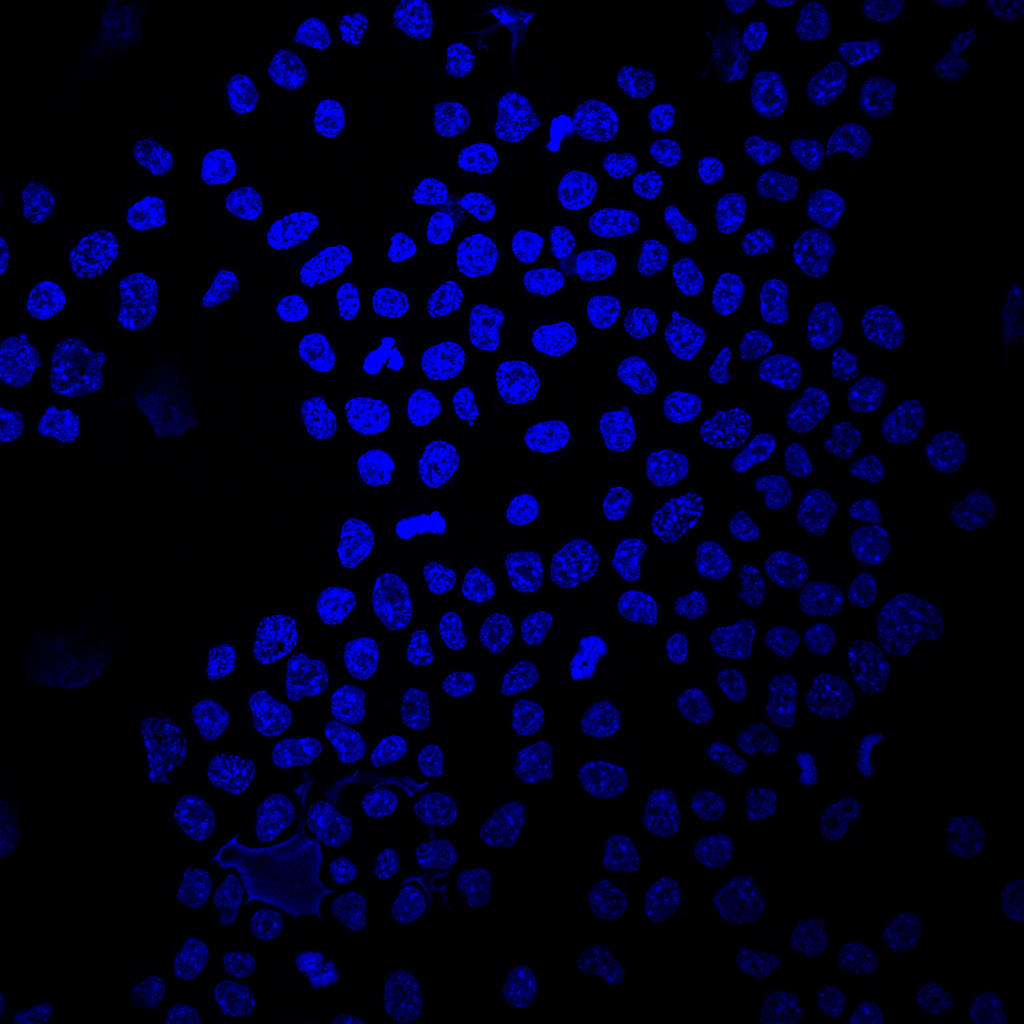

Supplement: Supplementary file 4 — Source data Fig. 2 [file 44318_2025_558_MOESM4_ESM.zip › Figure 2/panel 2C/KD-2_Bra/seq9705_seq9705_RGB_DAPI.tif]

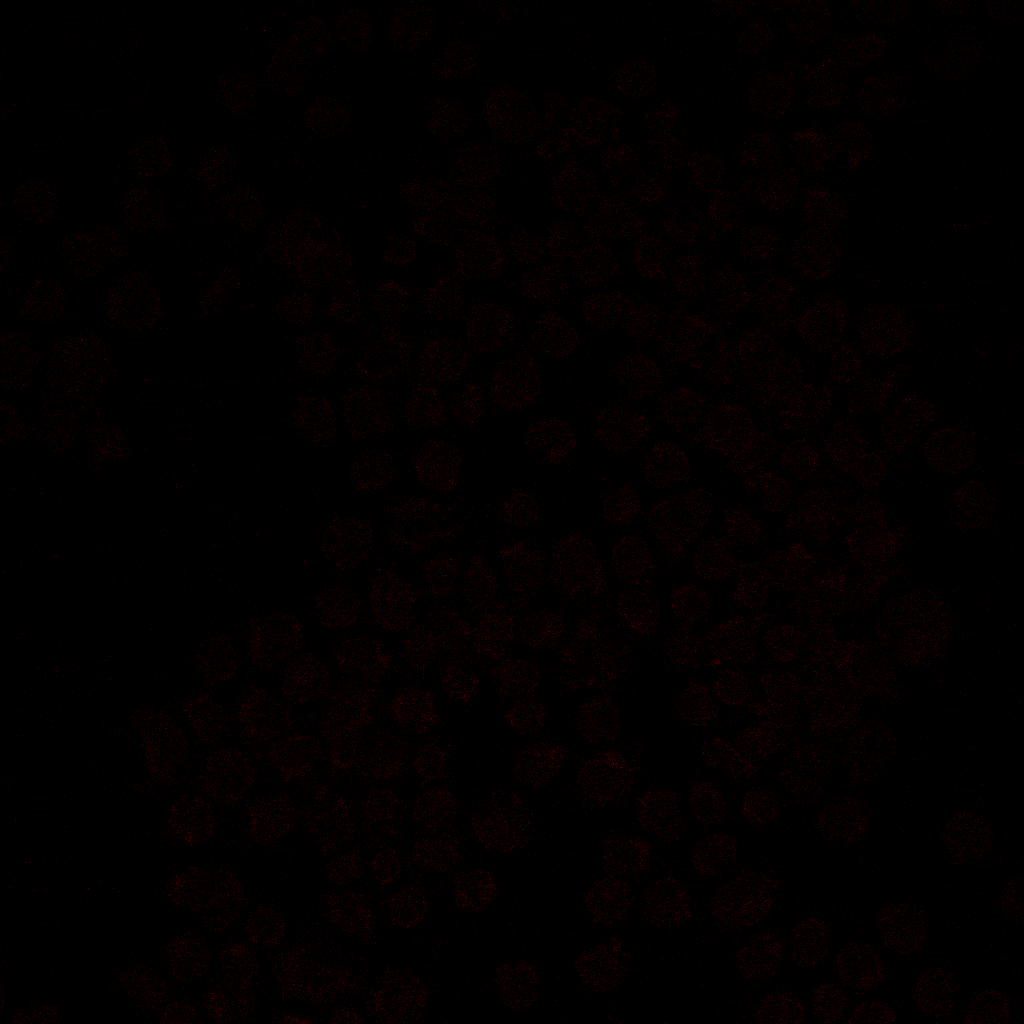

Supplement: Supplementary file 4 — Source data Fig. 2 [file 44318_2025_558_MOESM4_ESM.zip › Figure 2/panel 2C/KD-2_Bra/seq9705_seq9705_RGB_Texas Red.tif]

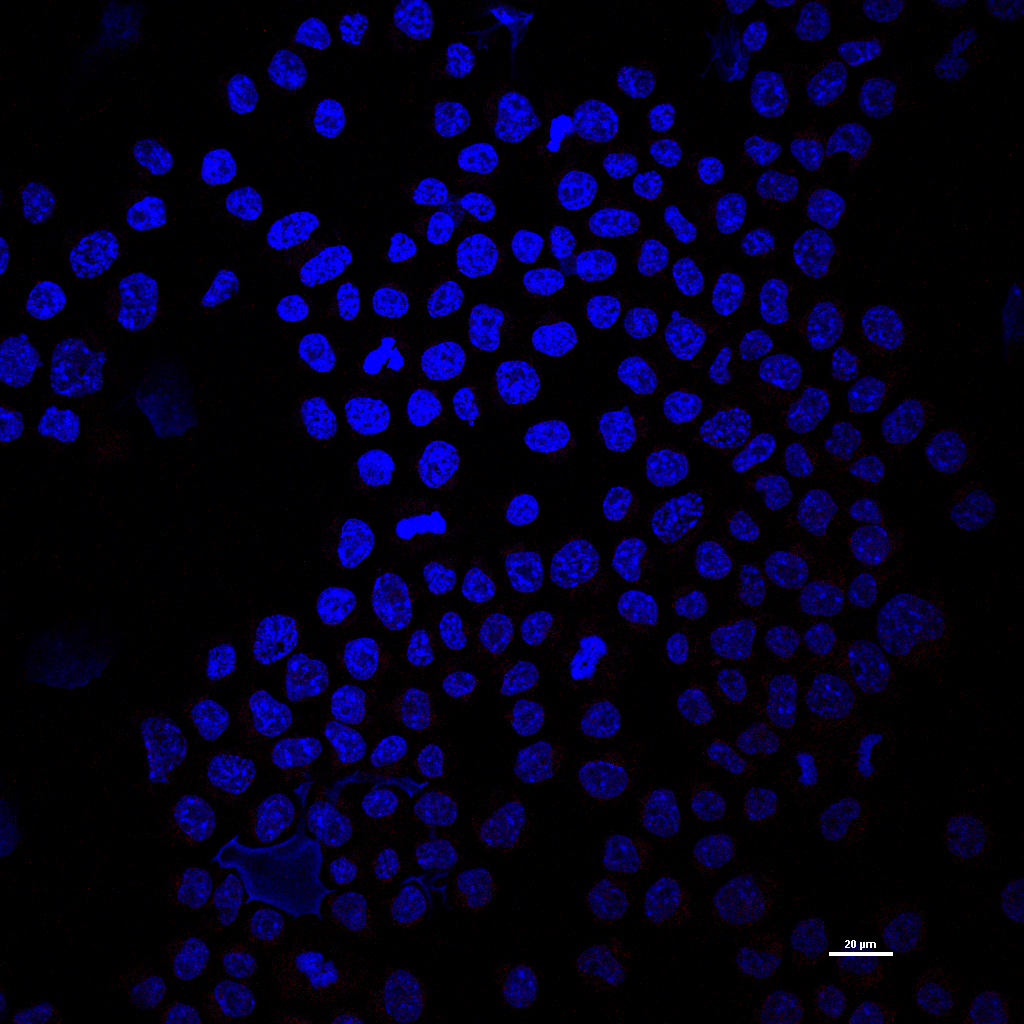

Supplement: Supplementary file 4 — Source data Fig. 2 [file 44318_2025_558_MOESM4_ESM.zip › Figure 2/panel 2C/KD-2_Bra/seq9705_seq9705_RGB.tif]

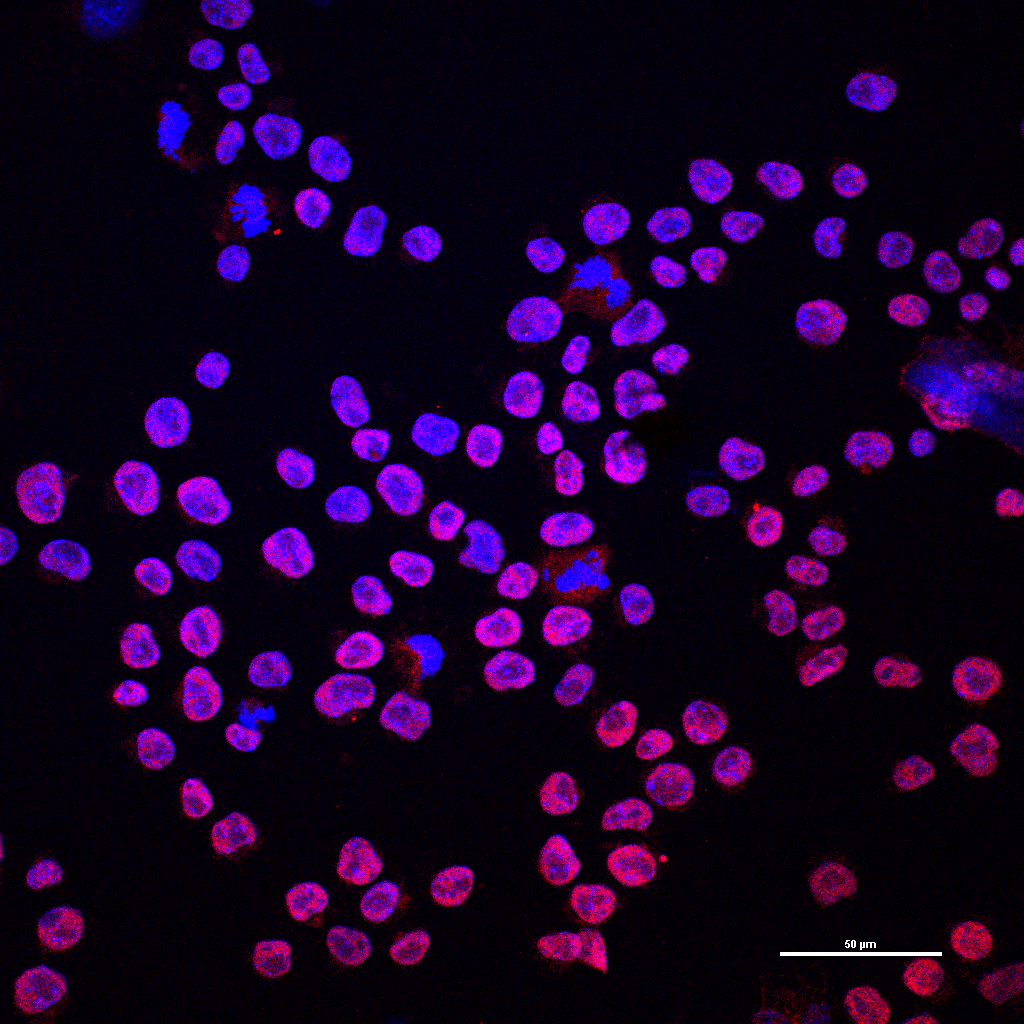

Supplement: Supplementary file 4 — Source data Fig. 2 [file 44318_2025_558_MOESM4_ESM.zip › Figure 2/panel 2C/NT_Nanog/seq9700_seq9700_RGB.tif]

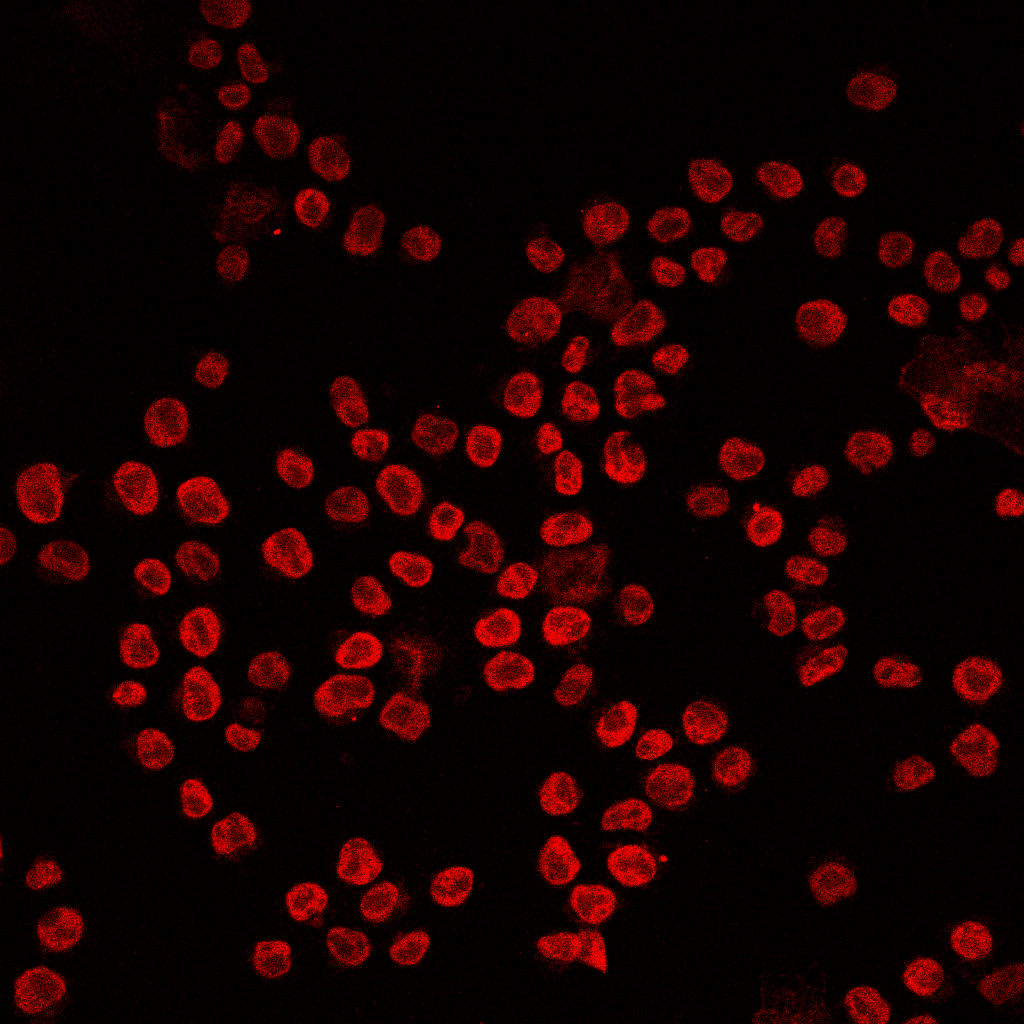

Supplement: Supplementary file 4 — Source data Fig. 2 [file 44318_2025_558_MOESM4_ESM.zip › Figure 2/panel 2C/NT_Nanog/seq9700_seq9700_RGB_Texas Red.tif]

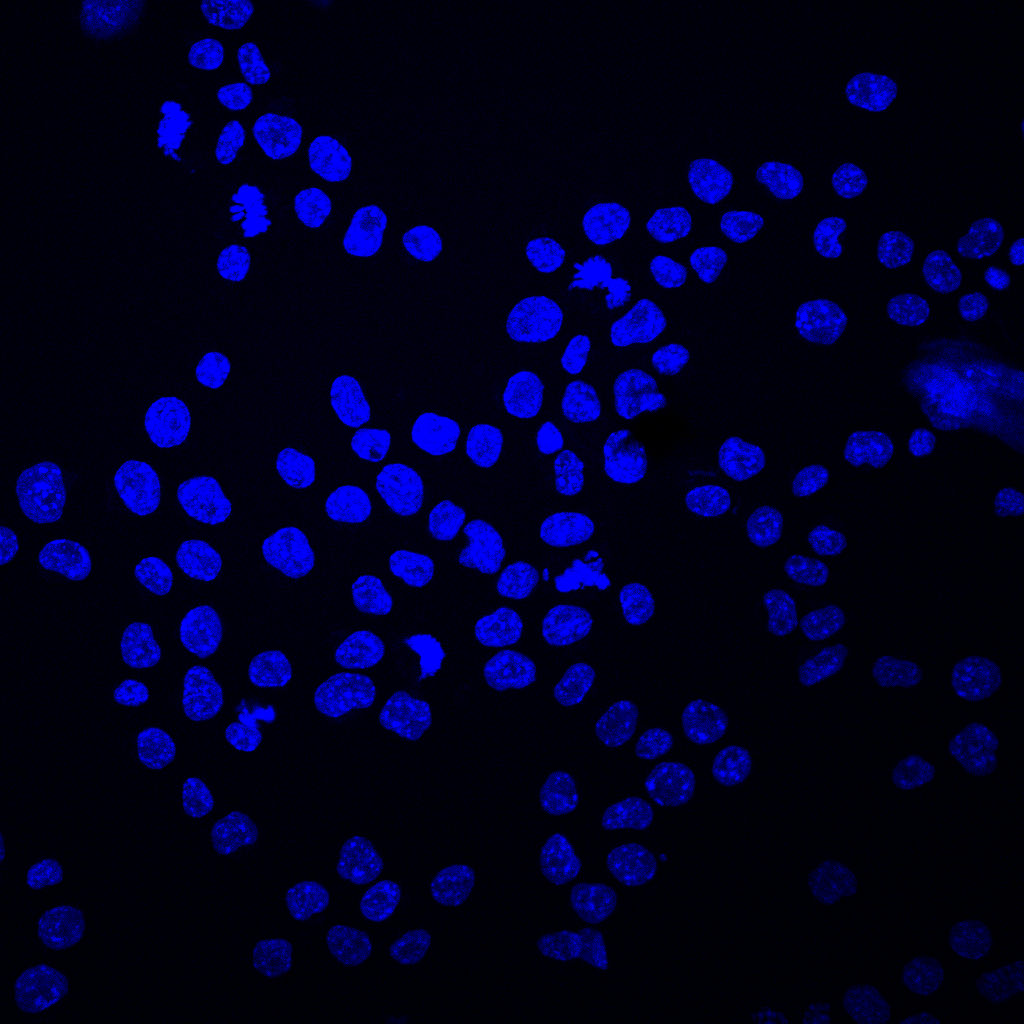

Supplement: Supplementary file 4 — Source data Fig. 2 [file 44318_2025_558_MOESM4_ESM.zip › Figure 2/panel 2C/NT_Nanog/seq9700_seq9700_RGB_DAPI.tif]

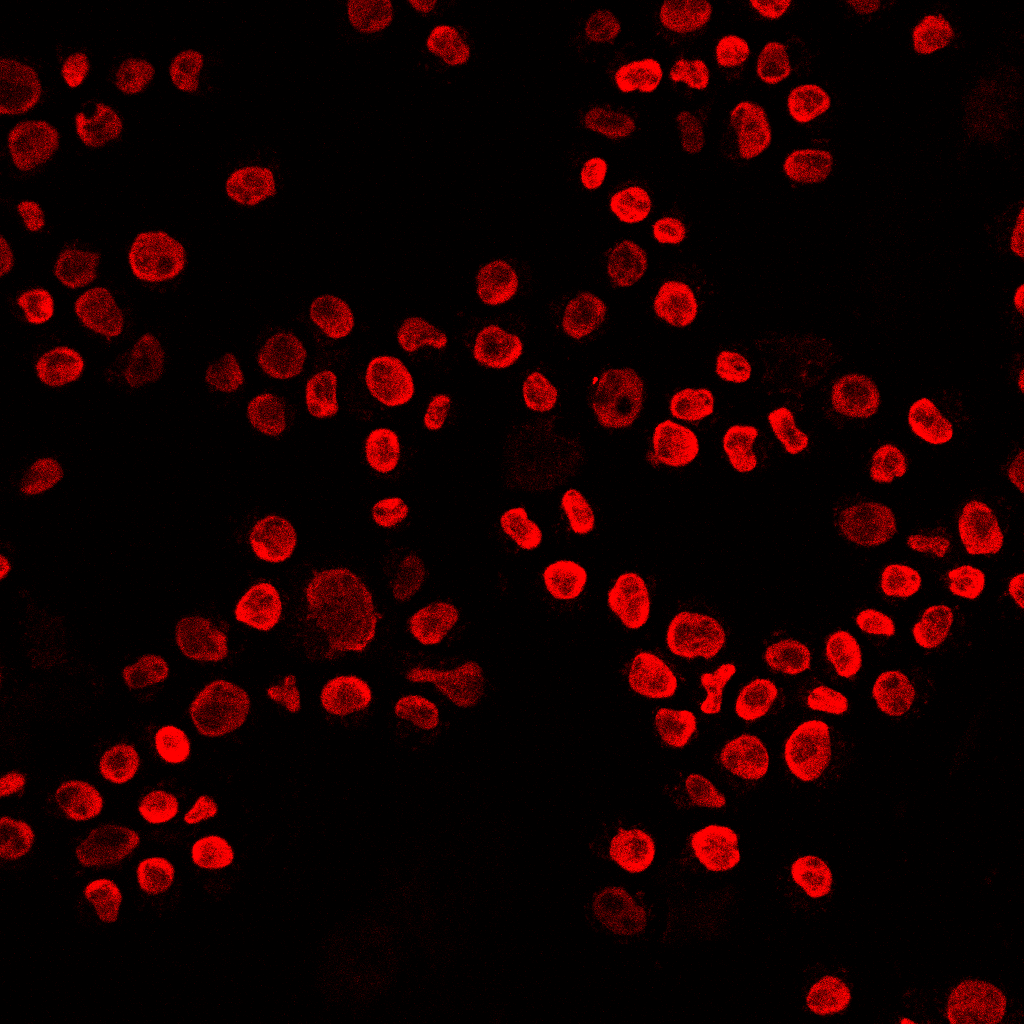

Supplement: Supplementary file 4 — Source data Fig. 2 [file 44318_2025_558_MOESM4_ESM.zip › Figure 2/panel 2C/KD-2_Oct4/seq9703_seq9703_RGB_Texas Red.tif]

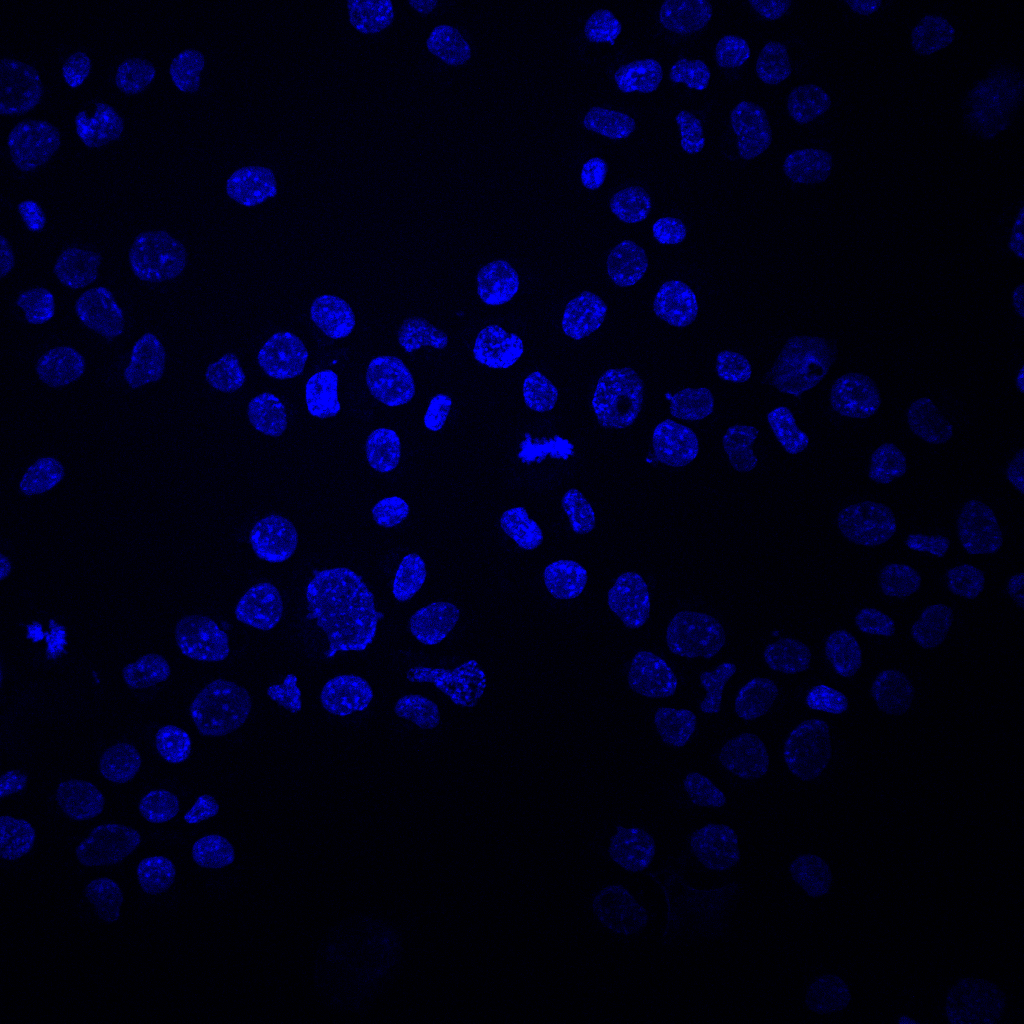

Supplement: Supplementary file 4 — Source data Fig. 2 [file 44318_2025_558_MOESM4_ESM.zip › Figure 2/panel 2C/KD-2_Oct4/seq9703_seq9703_RGB_DAPI.tif]

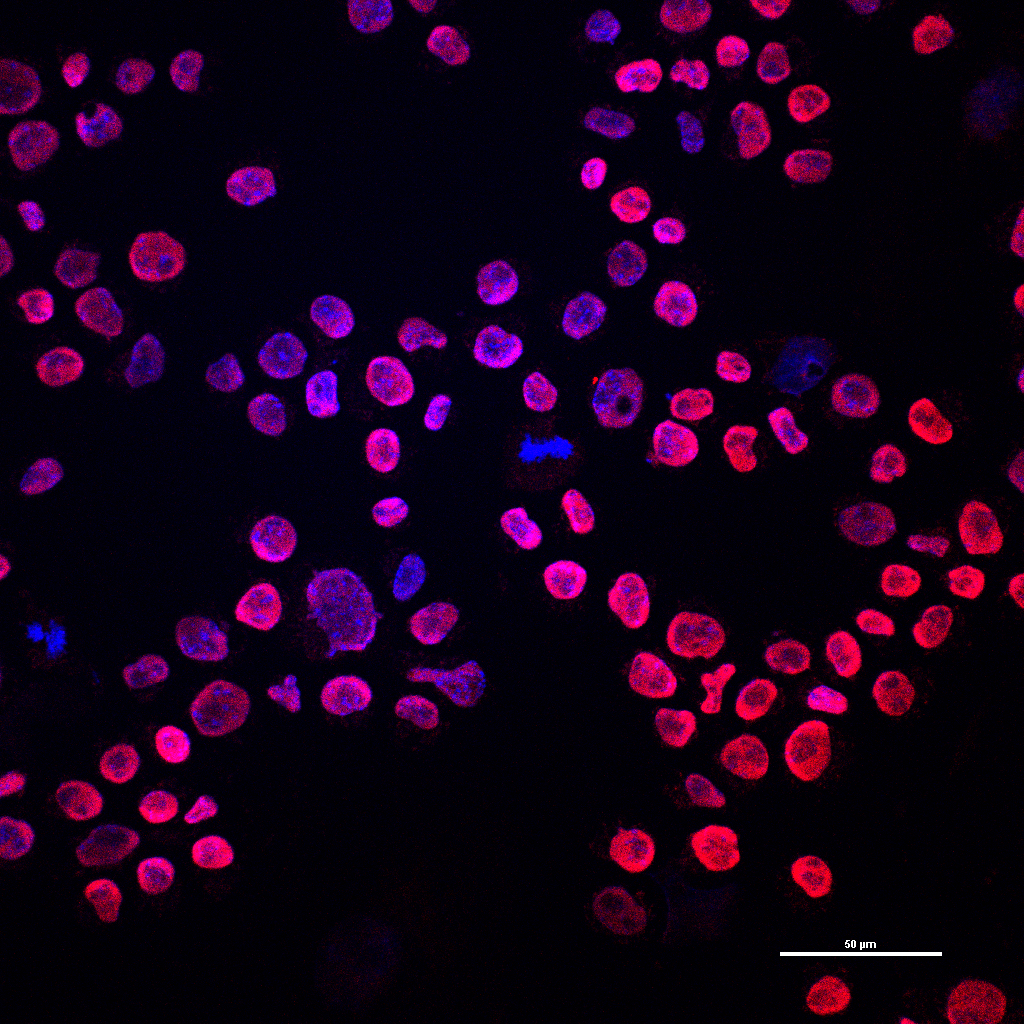

Supplement: Supplementary file 4 — Source data Fig. 2 [file 44318_2025_558_MOESM4_ESM.zip › Figure 2/panel 2C/KD-2_Oct4/seq9703_seq9703_RGB.tif]

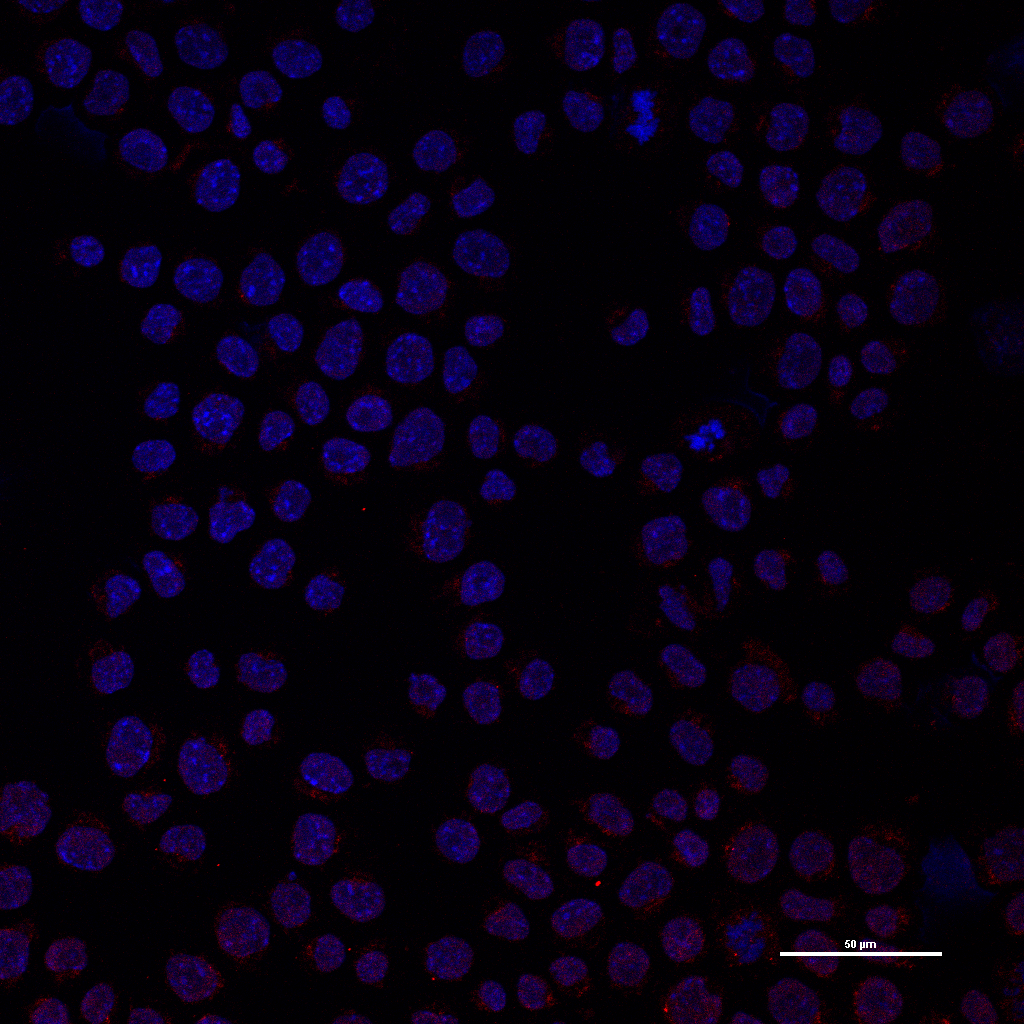

Supplement: Supplementary file 4 — Source data Fig. 2 [file 44318_2025_558_MOESM4_ESM.zip › Figure 2/panel 2C/NT_Sox17/seq9708_seq9708_RGB.tif]

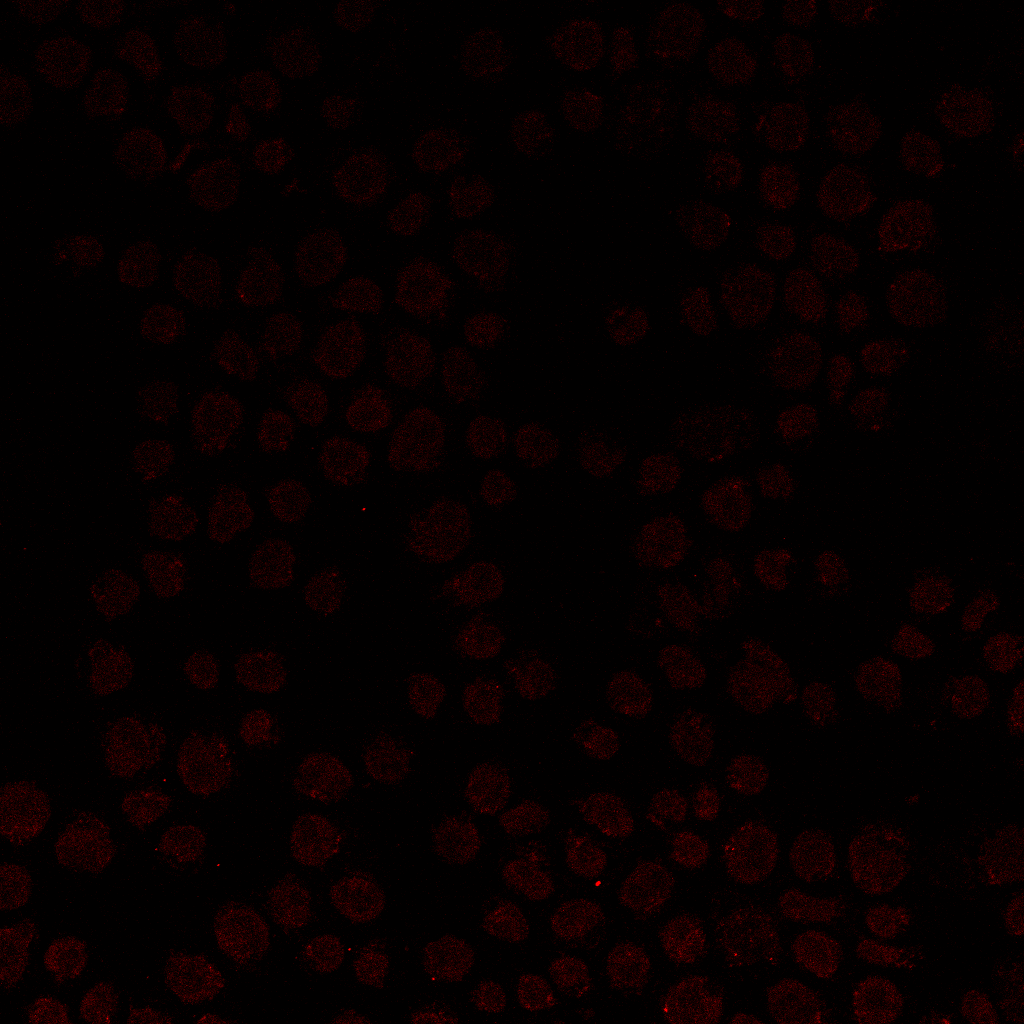

Supplement: Supplementary file 4 — Source data Fig. 2 [file 44318_2025_558_MOESM4_ESM.zip › Figure 2/panel 2C/NT_Sox17/seq9708_seq9708_RGB_Texas Red.tif]

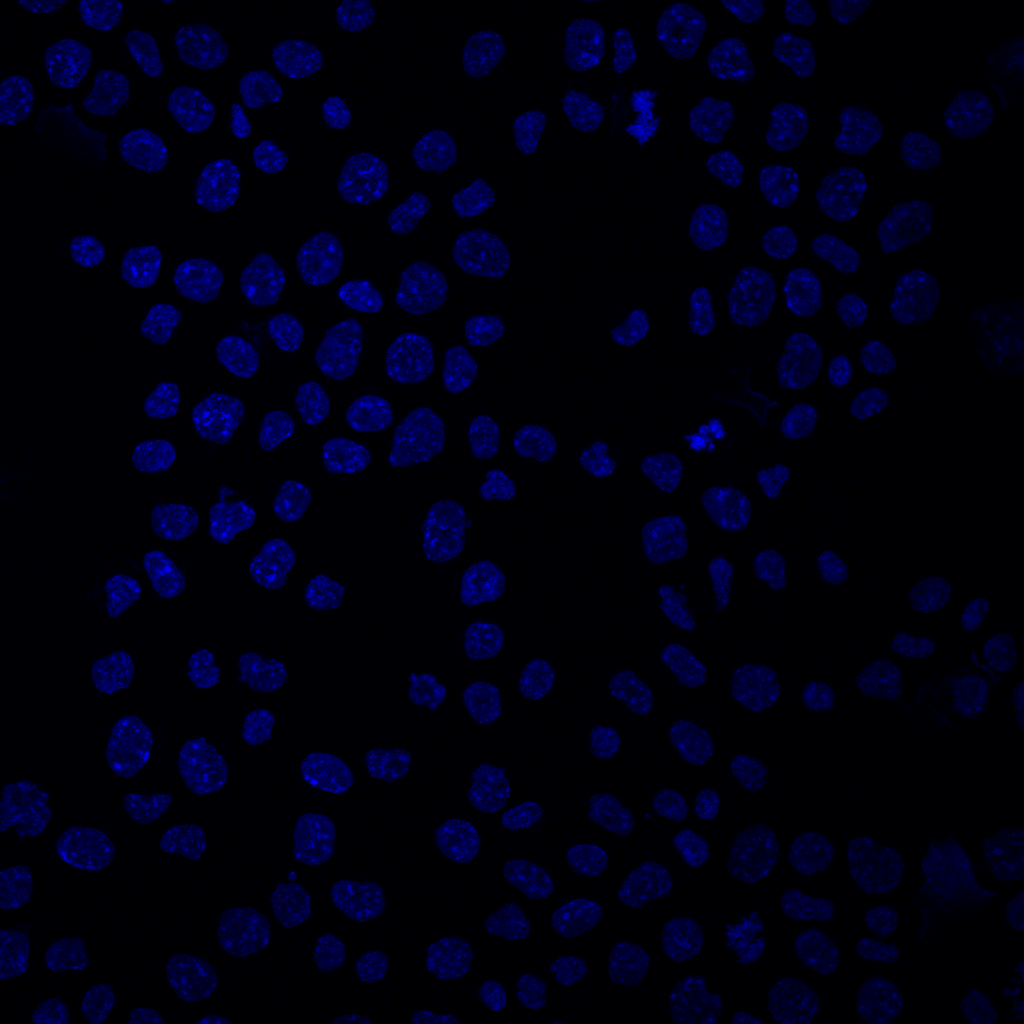

Supplement: Supplementary file 4 — Source data Fig. 2 [file 44318_2025_558_MOESM4_ESM.zip › Figure 2/panel 2C/NT_Sox17/seq9708_seq9708_RGB_DAPI.tif]

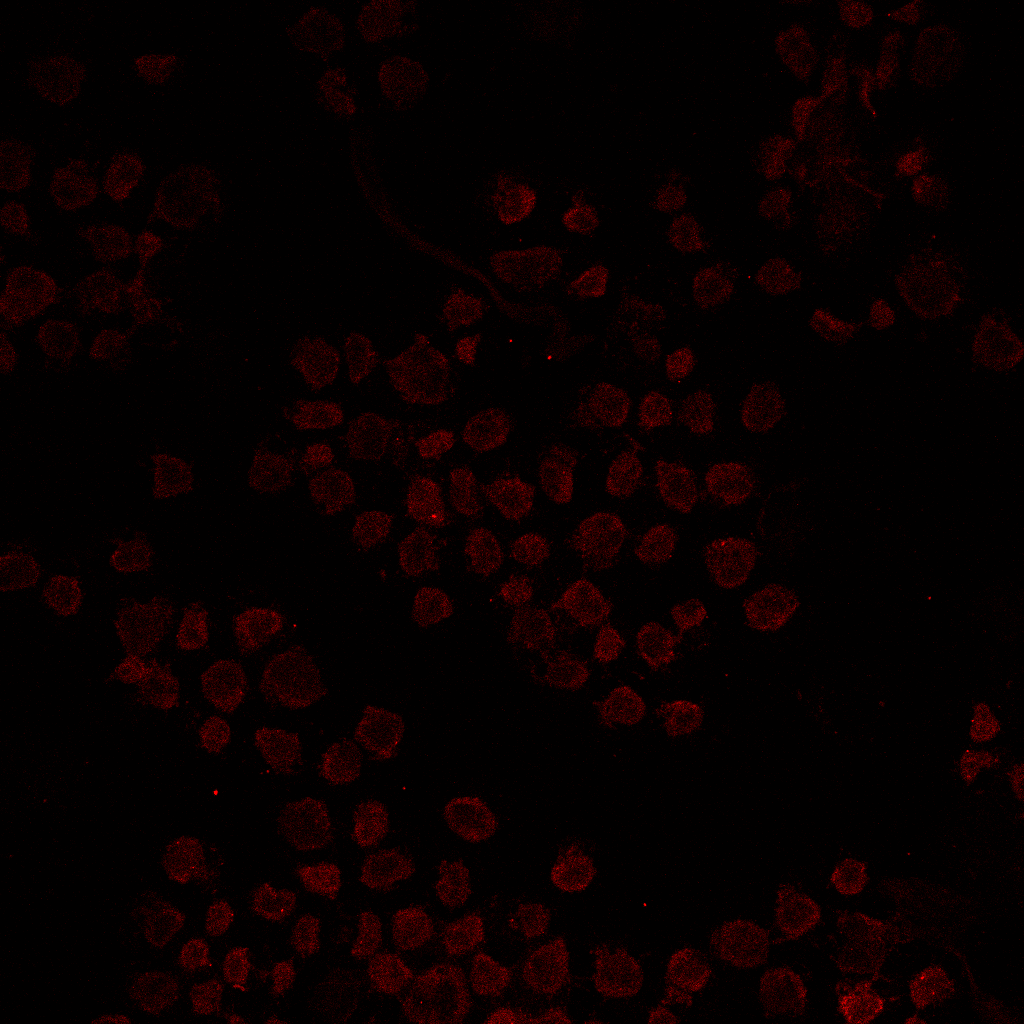

Supplement: Supplementary file 4 — Source data Fig. 2 [file 44318_2025_558_MOESM4_ESM.zip › Figure 2/panel 2C/KD-2_Sox17/seq9706_seq9706_RGB_Texas Red.tif]

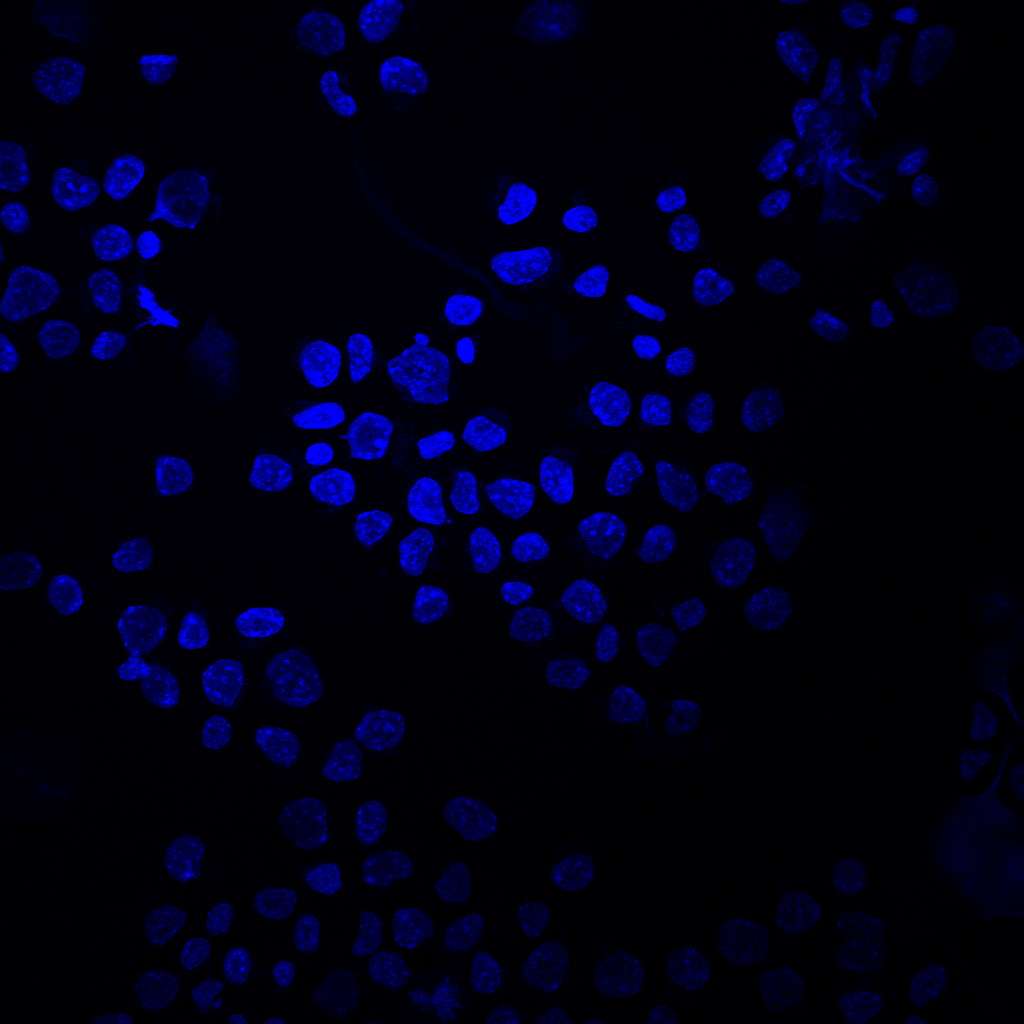

Supplement: Supplementary file 4 — Source data Fig. 2 [file 44318_2025_558_MOESM4_ESM.zip › Figure 2/panel 2C/KD-2_Sox17/seq9706_seq9706_RGB_DAPI.tif]

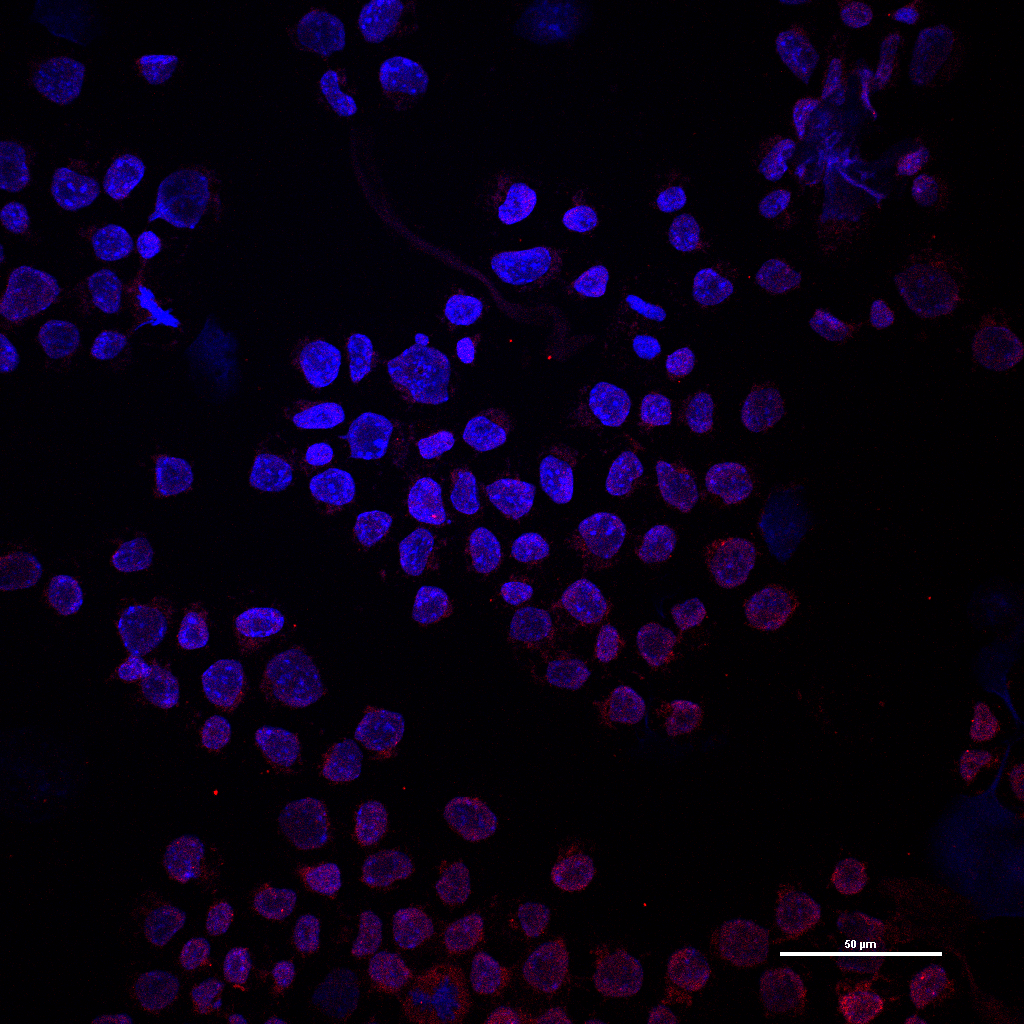

Supplement: Supplementary file 4 — Source data Fig. 2 [file 44318_2025_558_MOESM4_ESM.zip › Figure 2/panel 2C/KD-2_Sox17/seq9706_seq9706_RGB.tif]

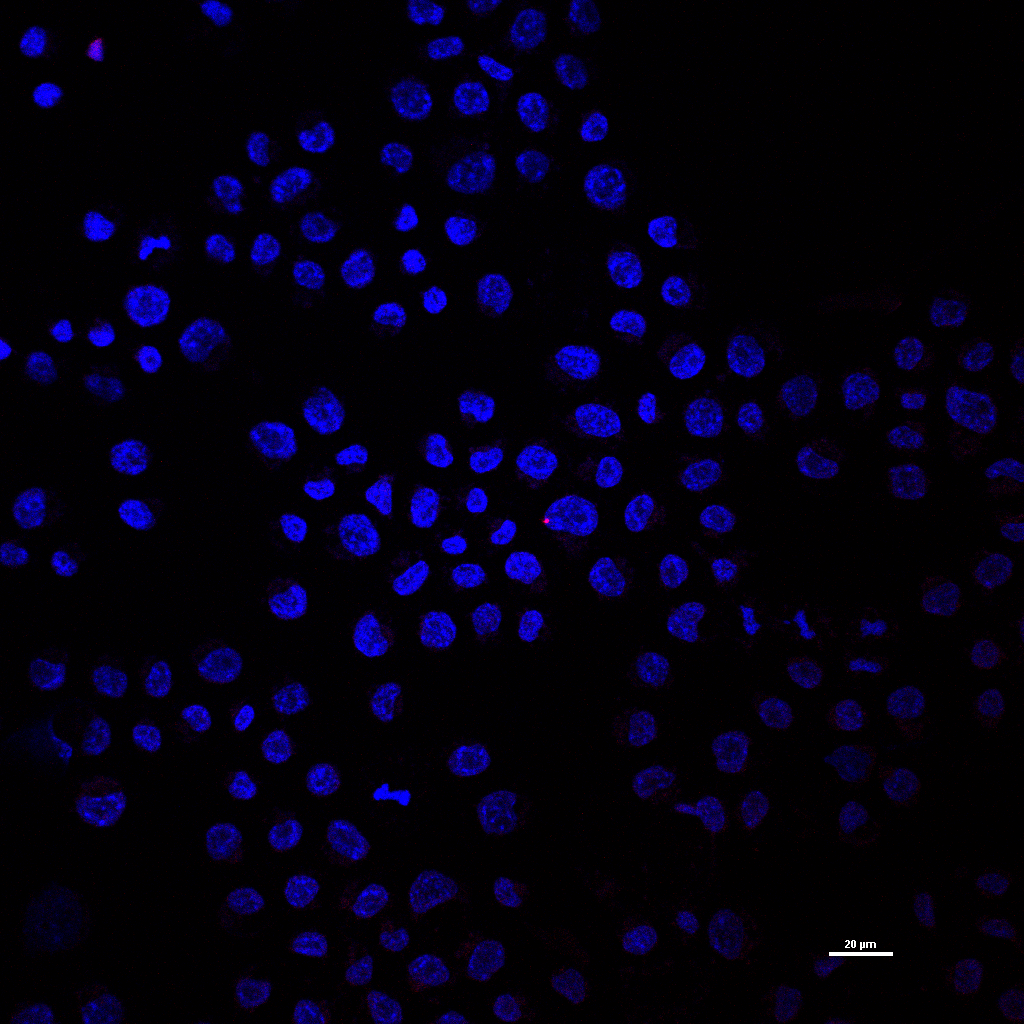

Supplement: Supplementary file 4 — Source data Fig. 2 [file 44318_2025_558_MOESM4_ESM.zip › Figure 2/panel 2C/NT_Bra/seq9701_seq9701_RGB.tif]

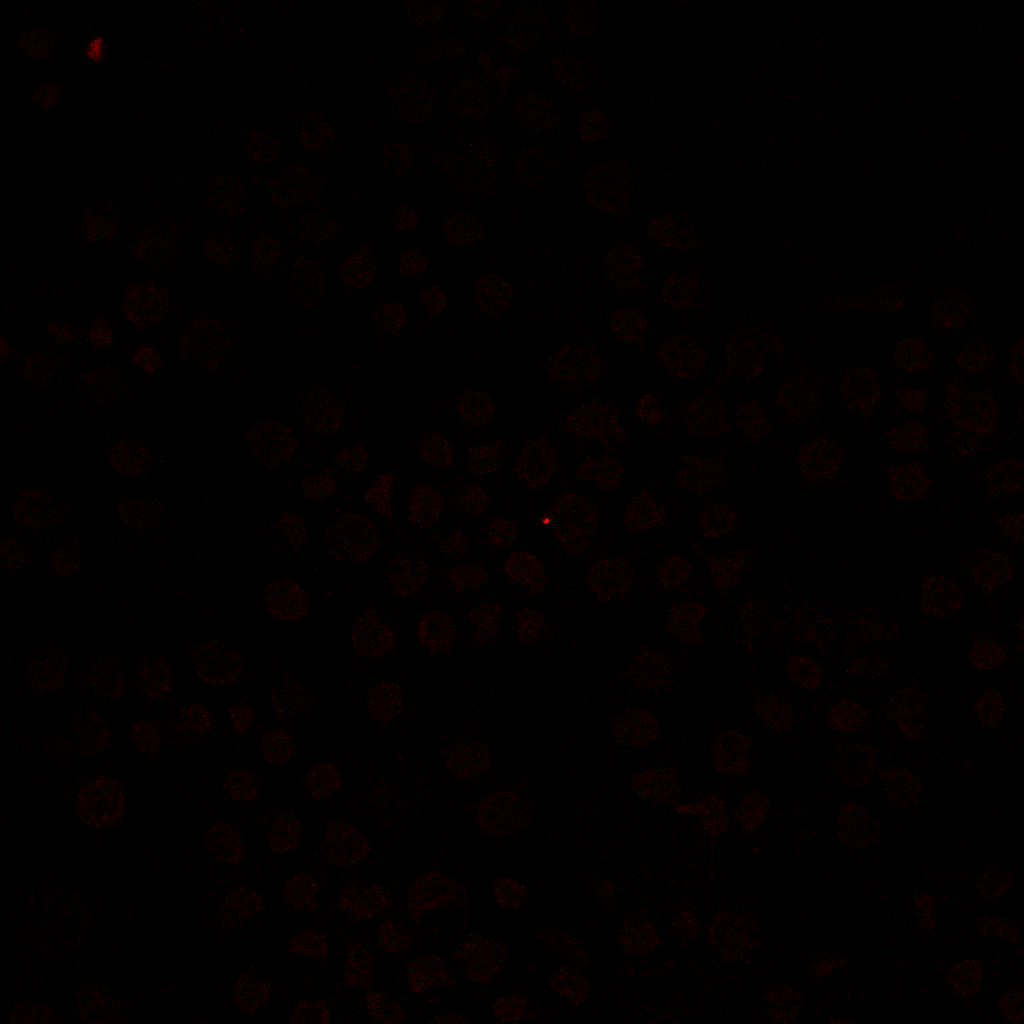

Supplement: Supplementary file 4 — Source data Fig. 2 [file 44318_2025_558_MOESM4_ESM.zip › Figure 2/panel 2C/NT_Bra/seq9701_seq9701_RGB_Texas Red.tif]

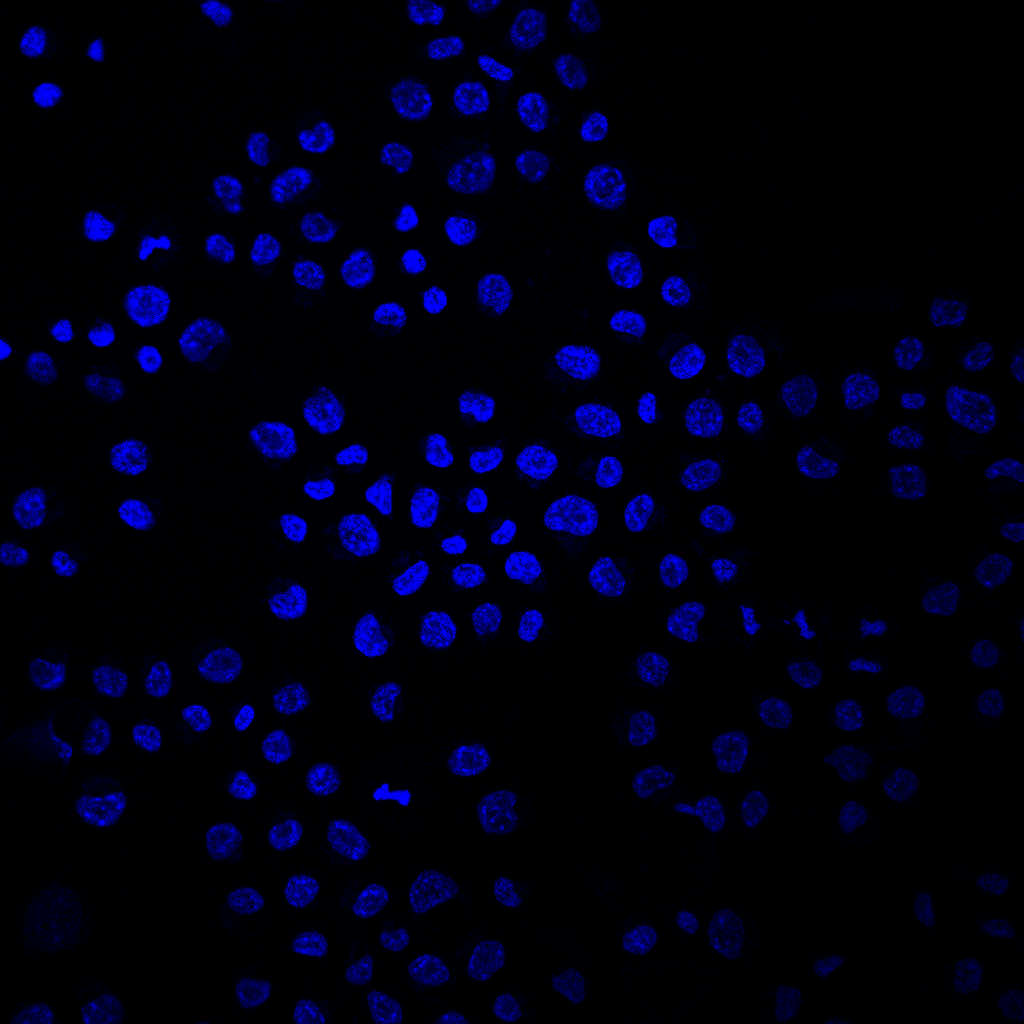

Supplement: Supplementary file 4 — Source data Fig. 2 [file 44318_2025_558_MOESM4_ESM.zip › Figure 2/panel 2C/NT_Bra/seq9701_seq9701_RGB_DAPI.tif]

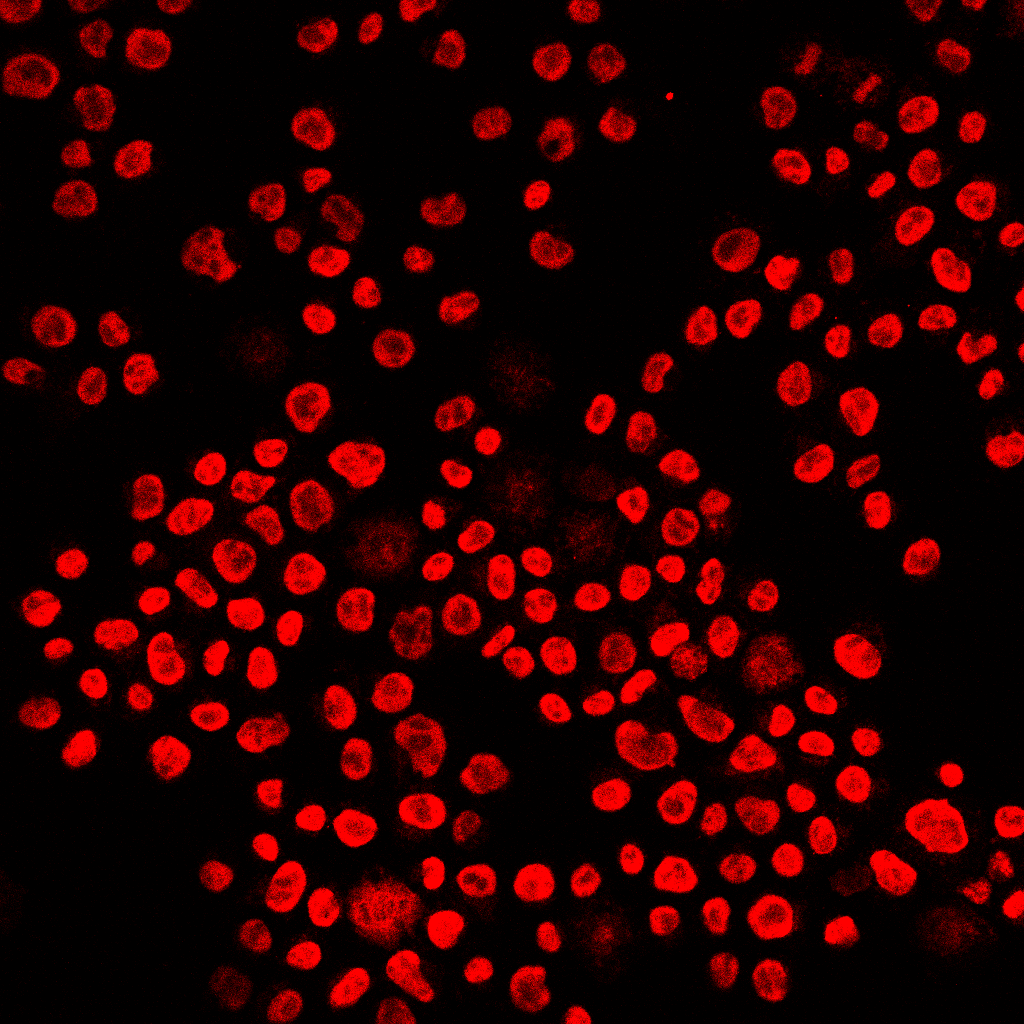

Supplement: Supplementary file 4 — Source data Fig. 2 [file 44318_2025_558_MOESM4_ESM.zip › Figure 2/panel 2C/KD-2_Nanog/seq9704_seq9704_RGB_Texas Red.tif]

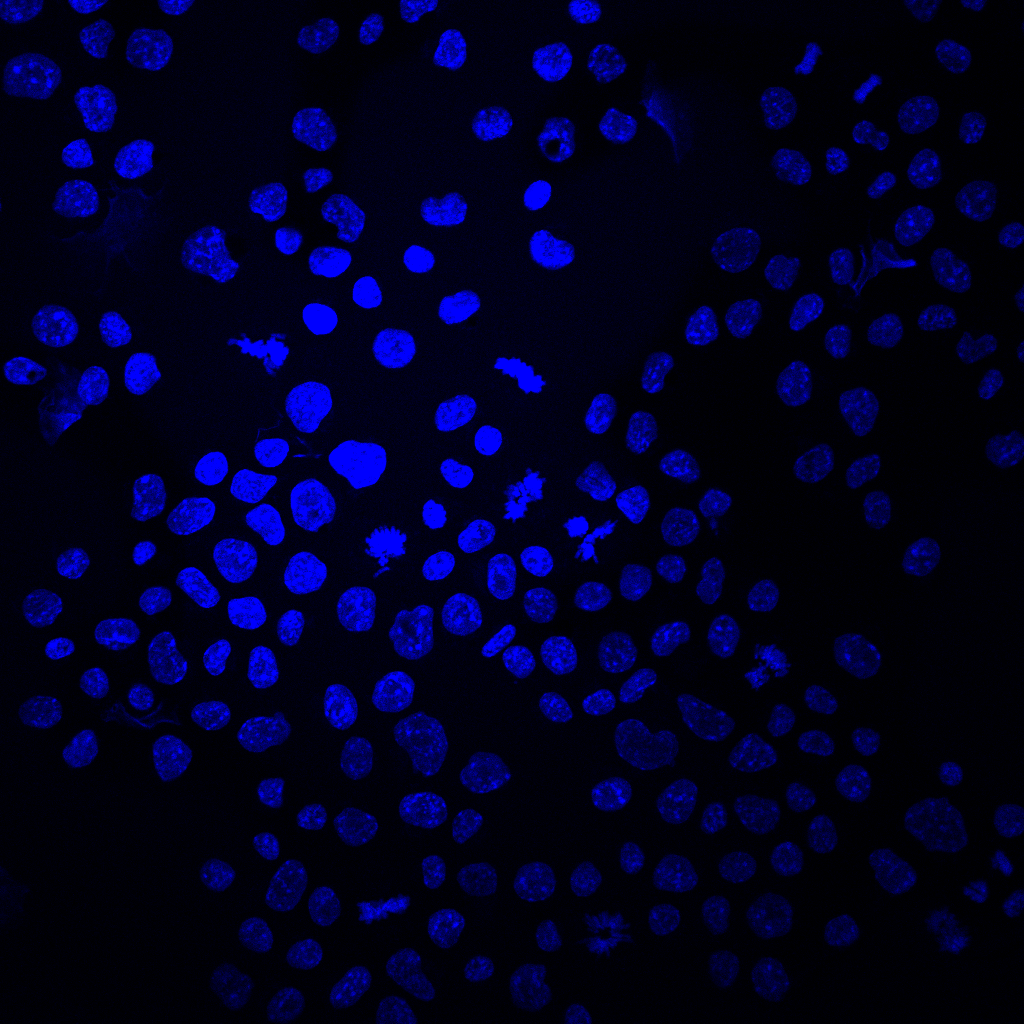

Supplement: Supplementary file 4 — Source data Fig. 2 [file 44318_2025_558_MOESM4_ESM.zip › Figure 2/panel 2C/KD-2_Nanog/seq9704_seq9704_RGB_DAPI.tif]

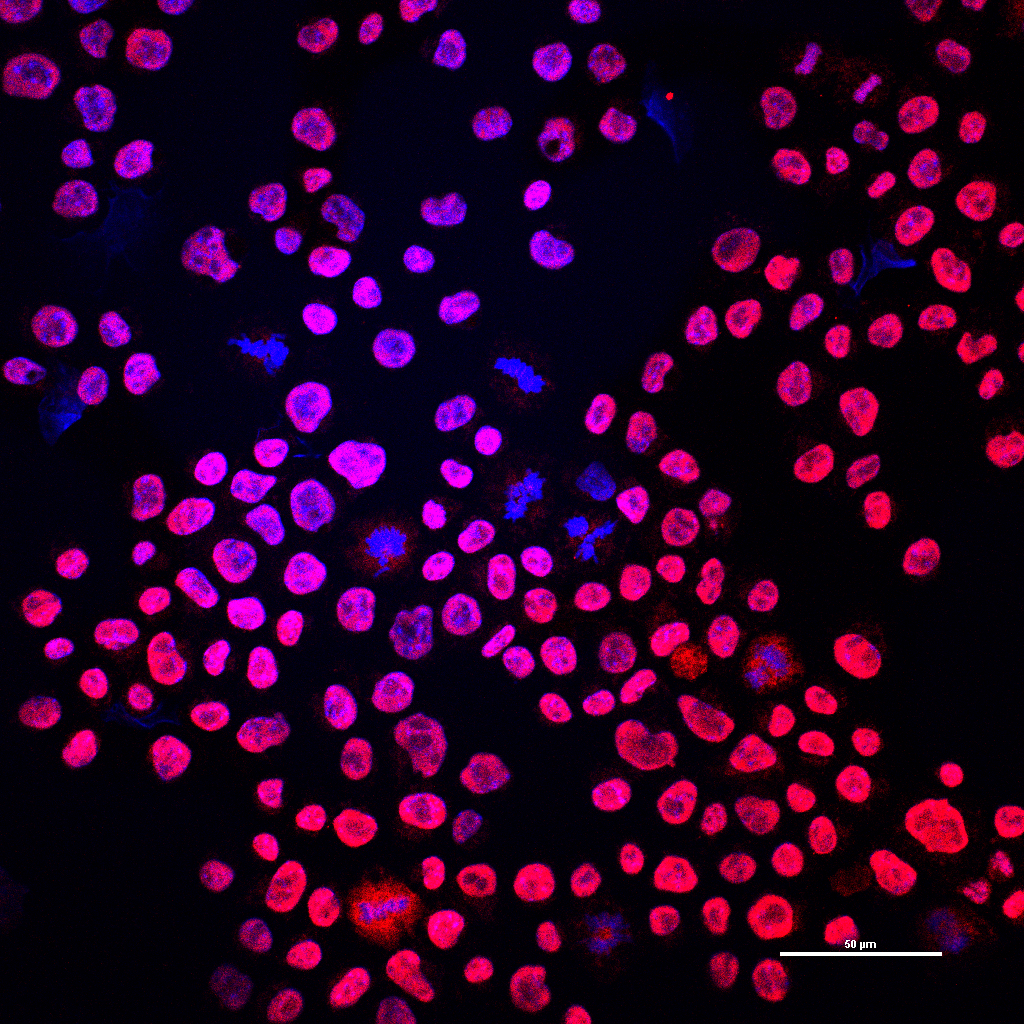

Supplement: Supplementary file 4 — Source data Fig. 2 [file 44318_2025_558_MOESM4_ESM.zip › Figure 2/panel 2C/KD-2_Nanog/seq9704_seq9704_RGB.tif]

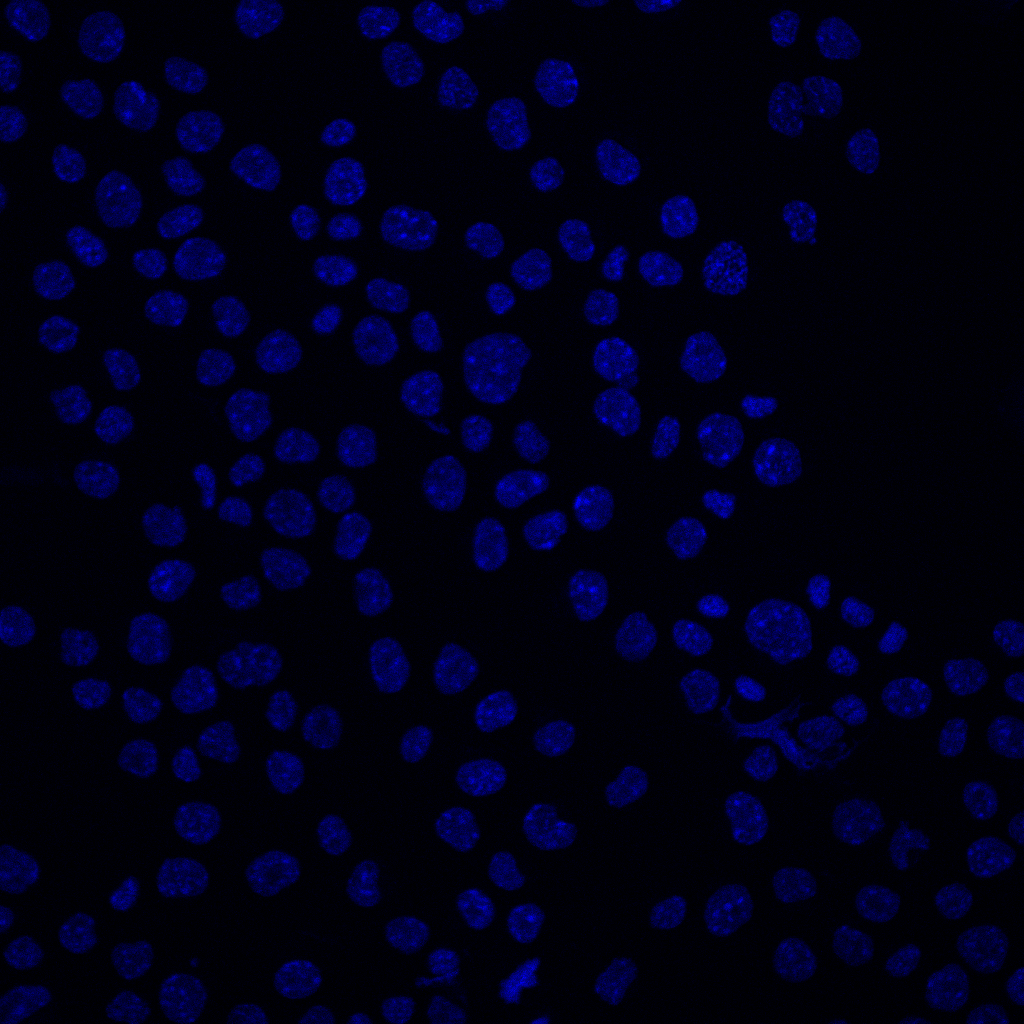

Supplement: Supplementary file 4 — Source data Fig. 2 [file 44318_2025_558_MOESM4_ESM.zip › Figure 2/panel 2C/NT_Oct4/seq9699_seq9699_RGB_DAPI.tif]

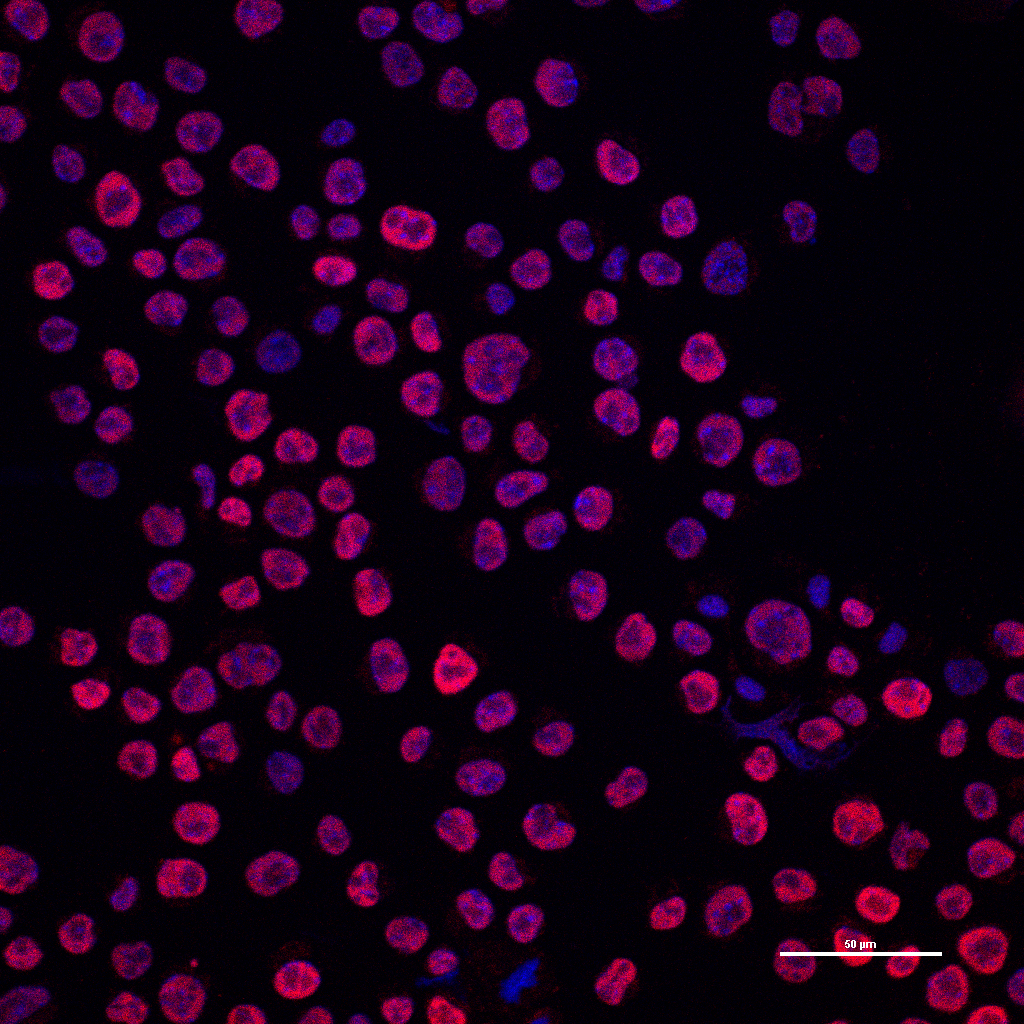

Supplement: Supplementary file 4 — Source data Fig. 2 [file 44318_2025_558_MOESM4_ESM.zip › Figure 2/panel 2C/NT_Oct4/seq9699_seq9699_RGB.tif]

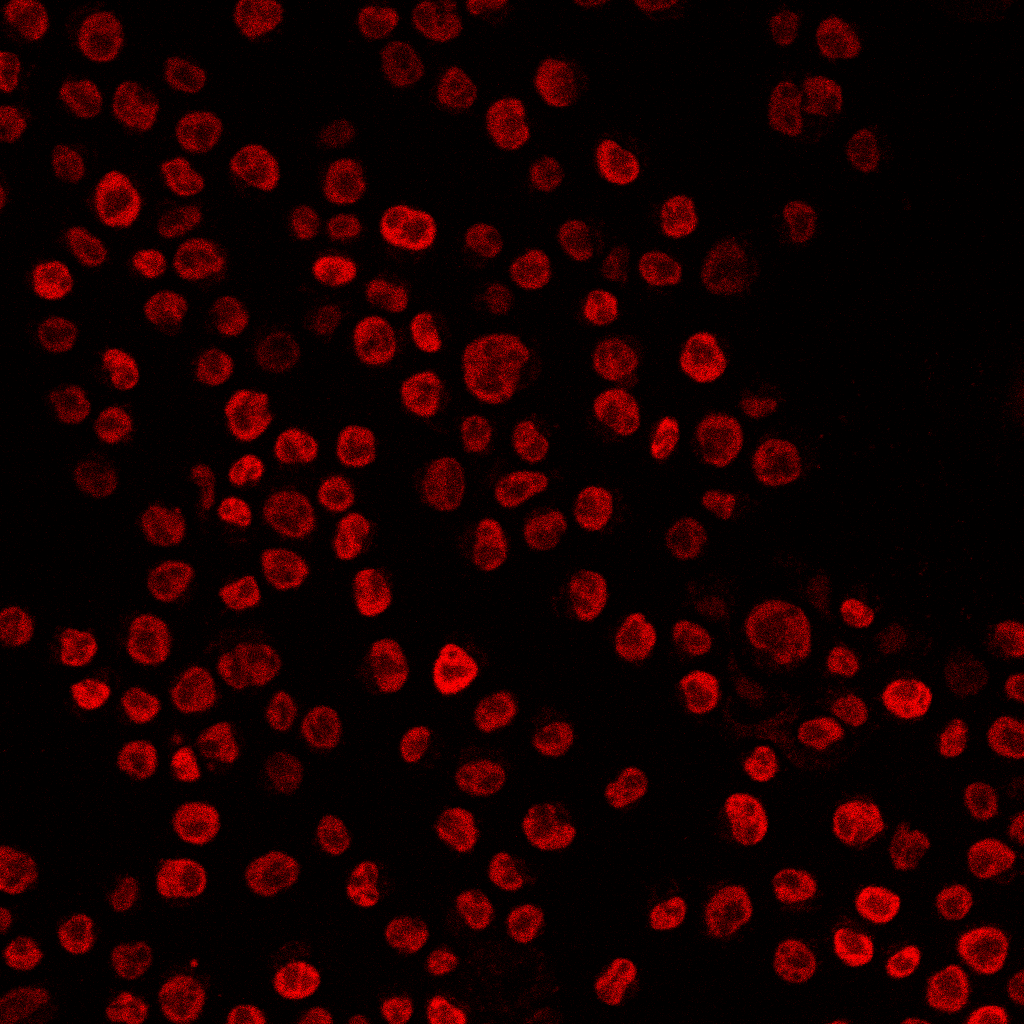

Supplement: Supplementary file 4 — Source data Fig. 2 [file 44318_2025_558_MOESM4_ESM.zip › Figure 2/panel 2C/NT_Oct4/seq9699_seq9699_RGB_Texas Red.tif]

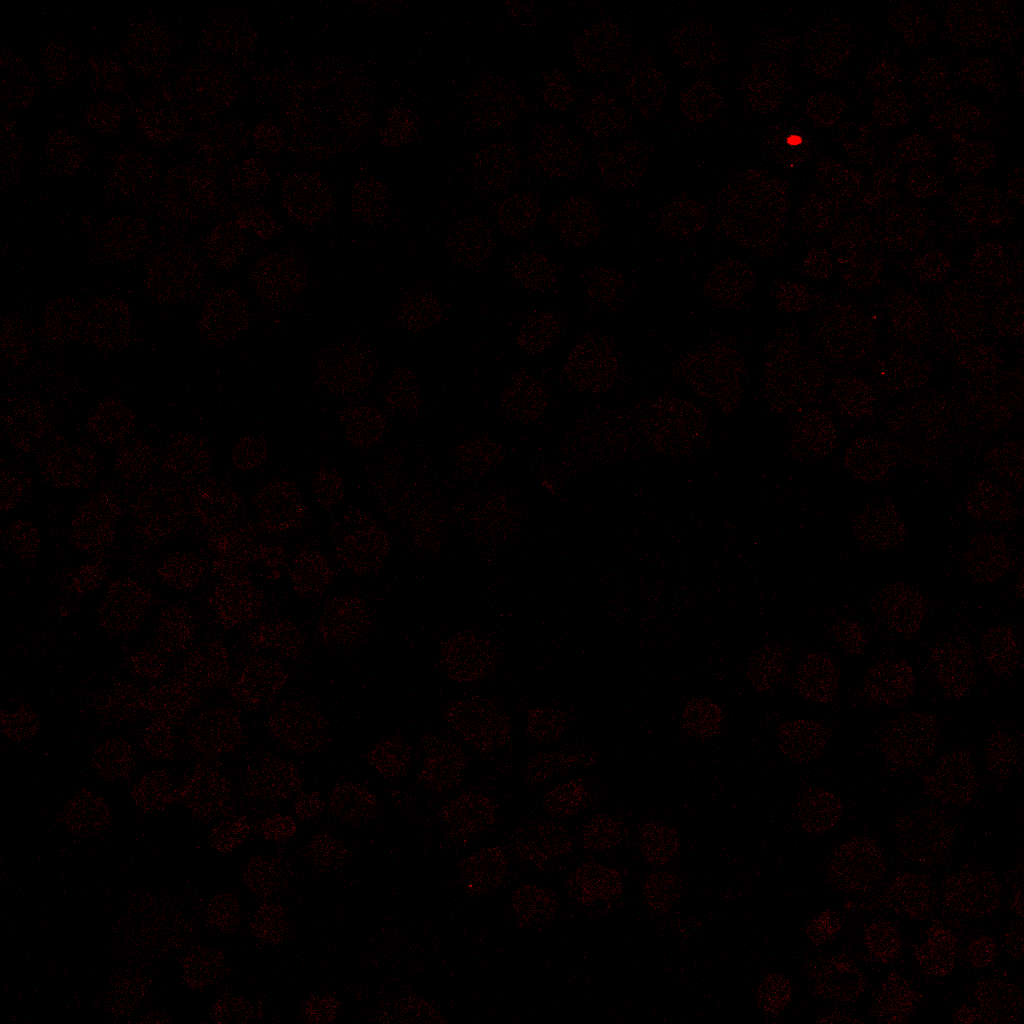

Supplement: Supplementary file 4 — Source data Fig. 2 [file 44318_2025_558_MOESM4_ESM.zip › Figure 2/panel 2C/KD-2_Nestin/seq9706_seq9706_RGB_Texas Red.tif]

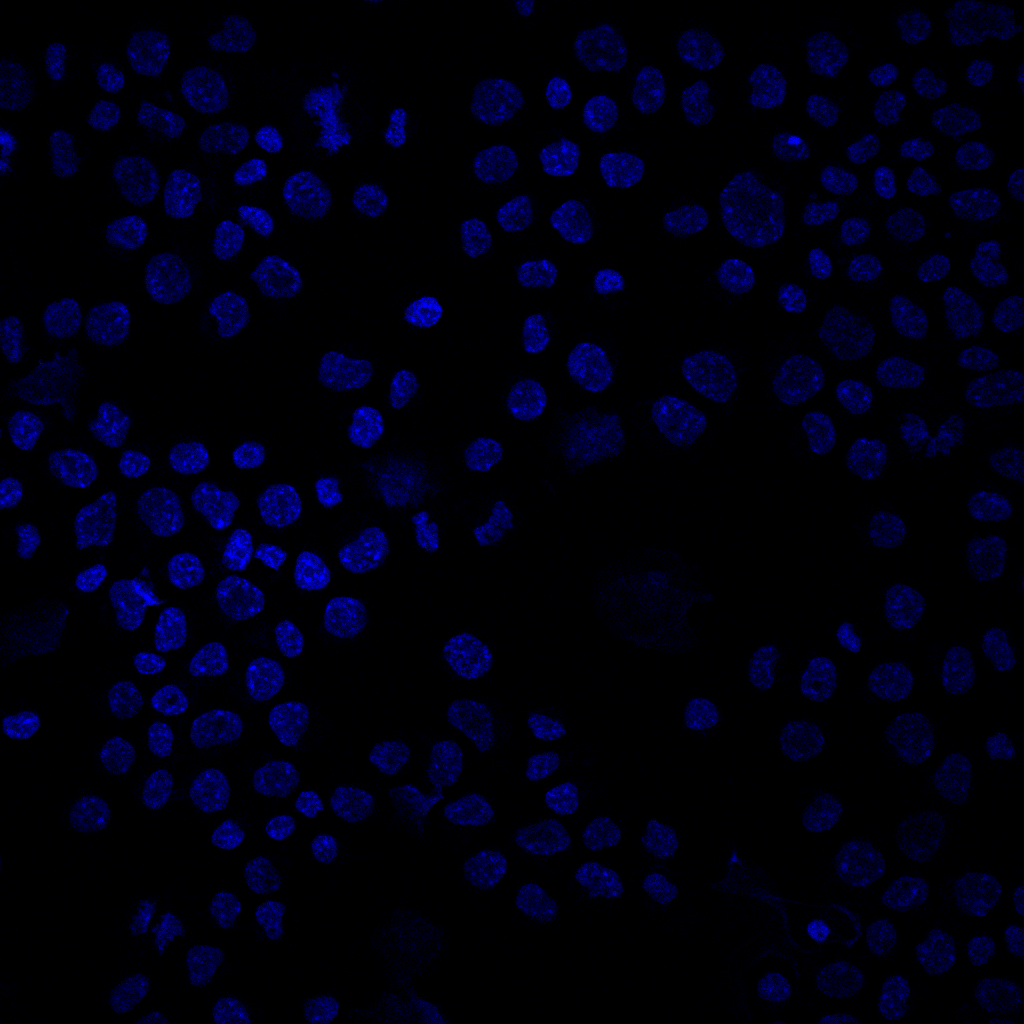

Supplement: Supplementary file 4 — Source data Fig. 2 [file 44318_2025_558_MOESM4_ESM.zip › Figure 2/panel 2C/KD-2_Nestin/seq9706_seq9706_RGB_DAPI.tif]

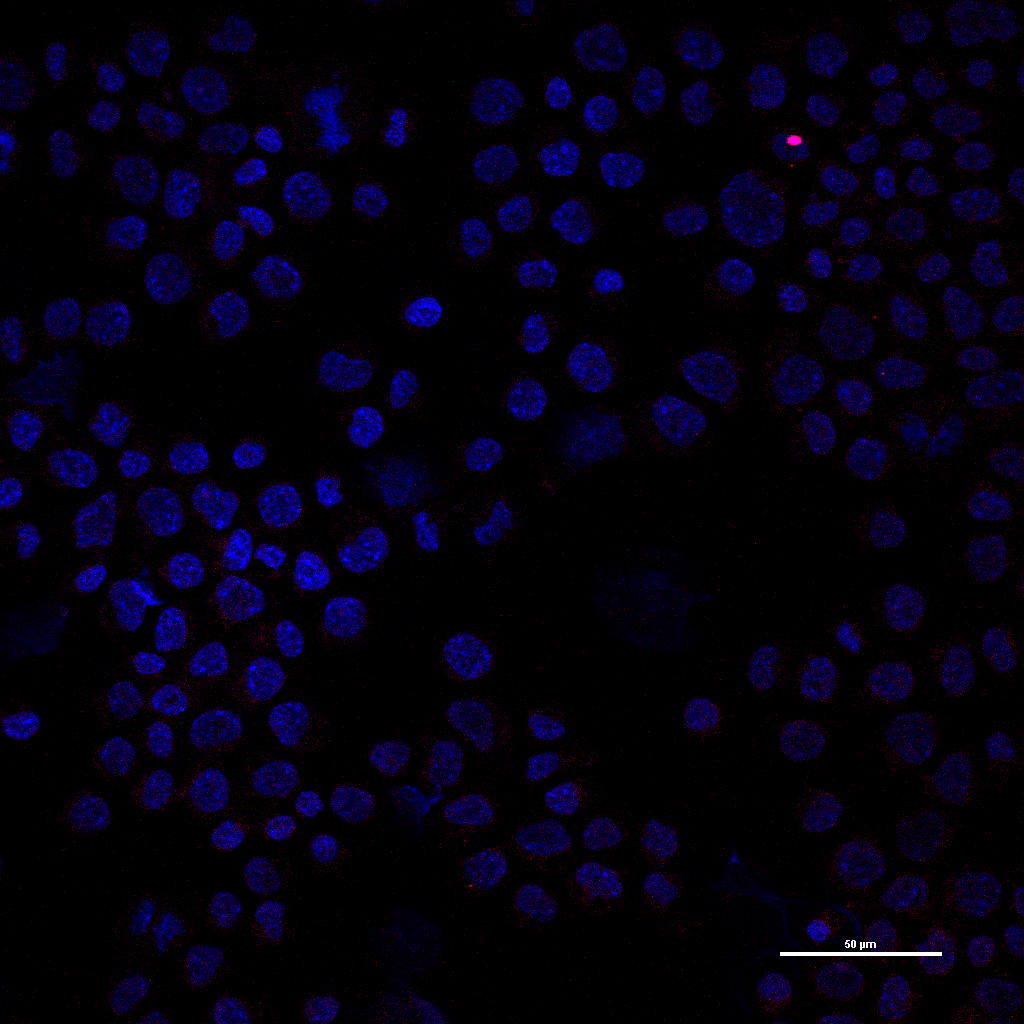

Supplement: Supplementary file 4 — Source data Fig. 2 [file 44318_2025_558_MOESM4_ESM.zip › Figure 2/panel 2C/KD-2_Nestin/seq9706_seq9706_RGB.tif]

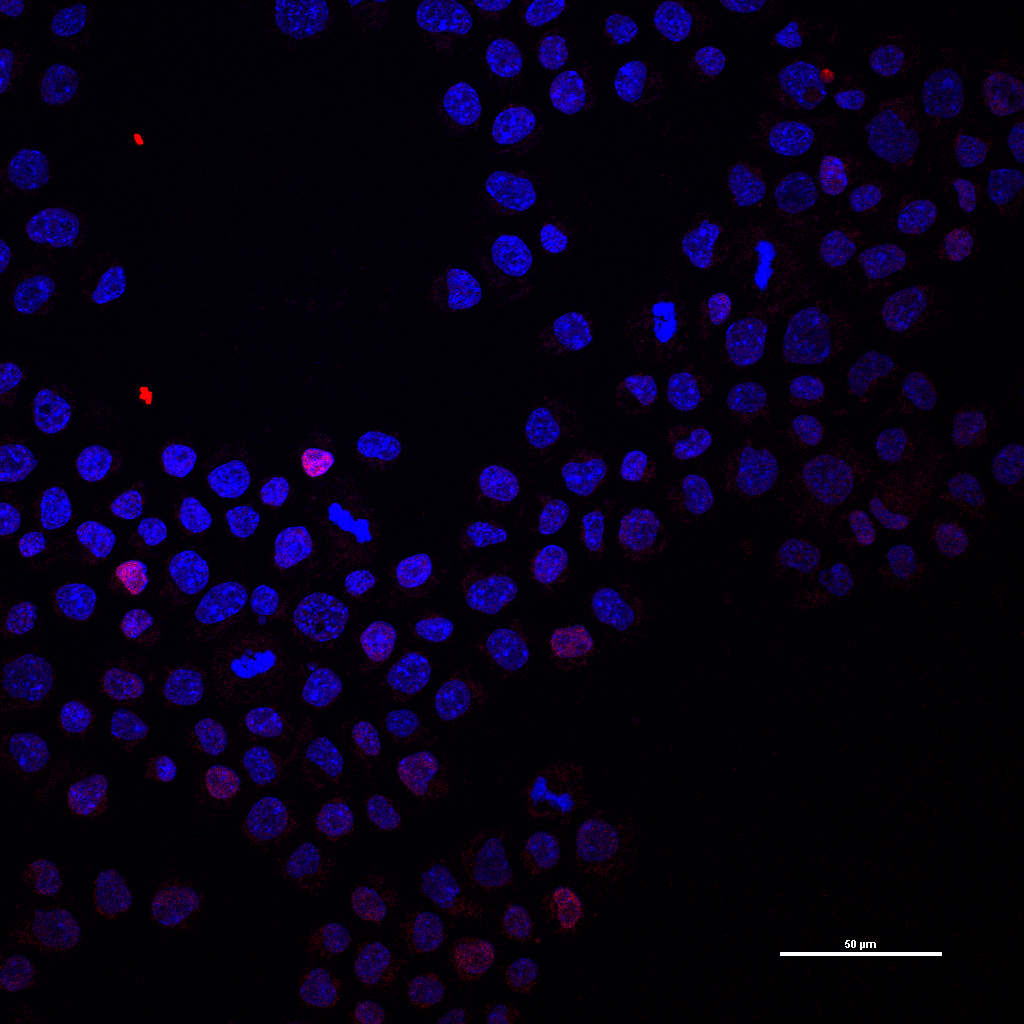

Supplement: Supplementary file 4 — Source data Fig. 2 [file 44318_2025_558_MOESM4_ESM.zip › Figure 2/panel 2C/KD-2_Cdx2/seq9705_seq9705_RGB.tif]

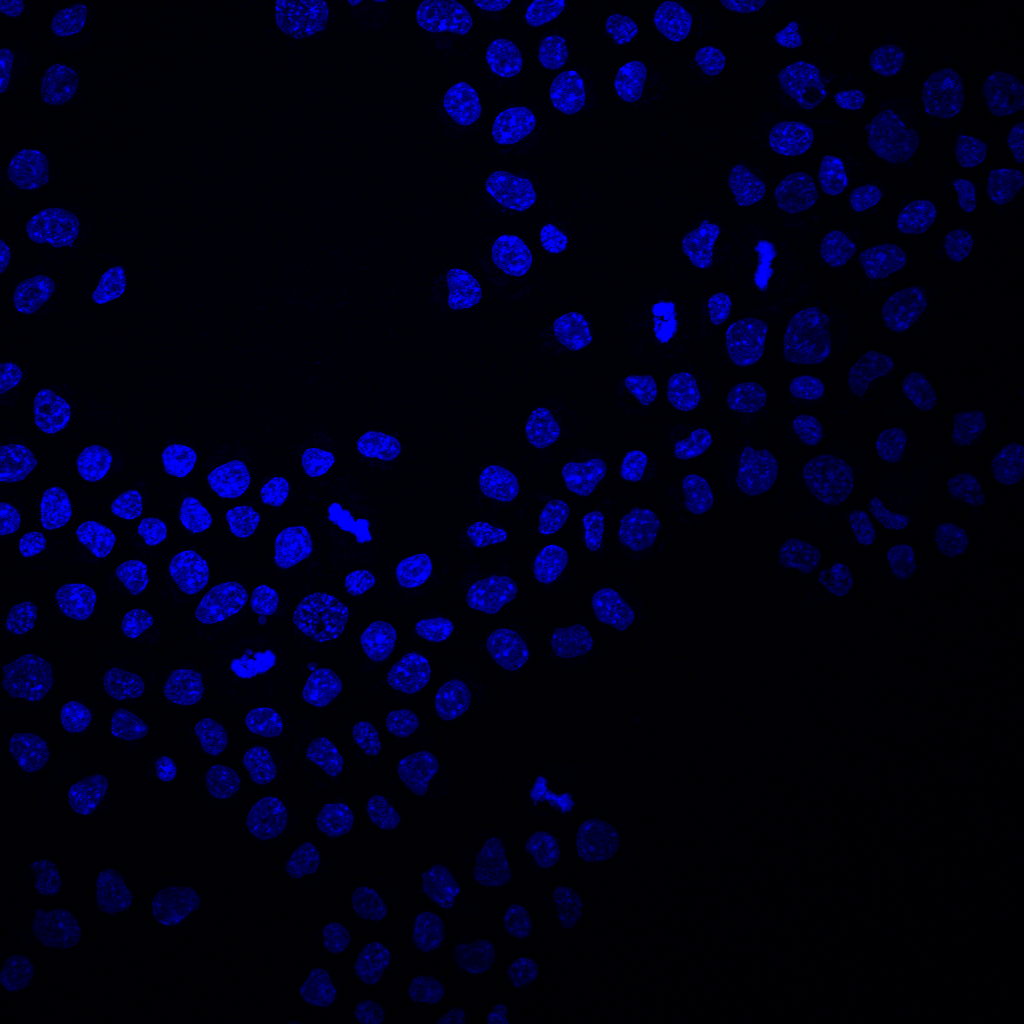

Supplement: Supplementary file 4 — Source data Fig. 2 [file 44318_2025_558_MOESM4_ESM.zip › Figure 2/panel 2C/KD-2_Cdx2/seq9705_seq9705_RGB_DAPI.tif]

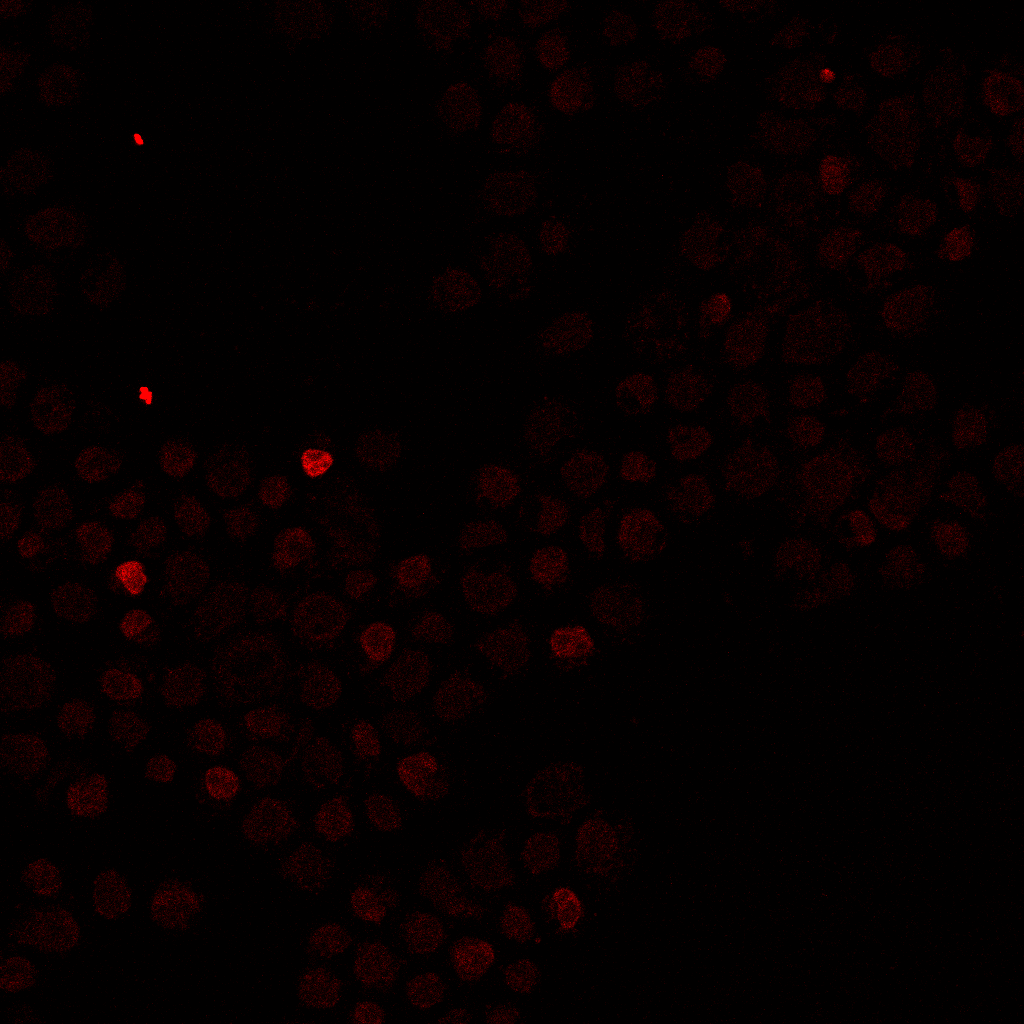

Supplement: Supplementary file 4 — Source data Fig. 2 [file 44318_2025_558_MOESM4_ESM.zip › Figure 2/panel 2C/KD-2_Cdx2/seq9705_seq9705_RGB_Texas Red.tif]

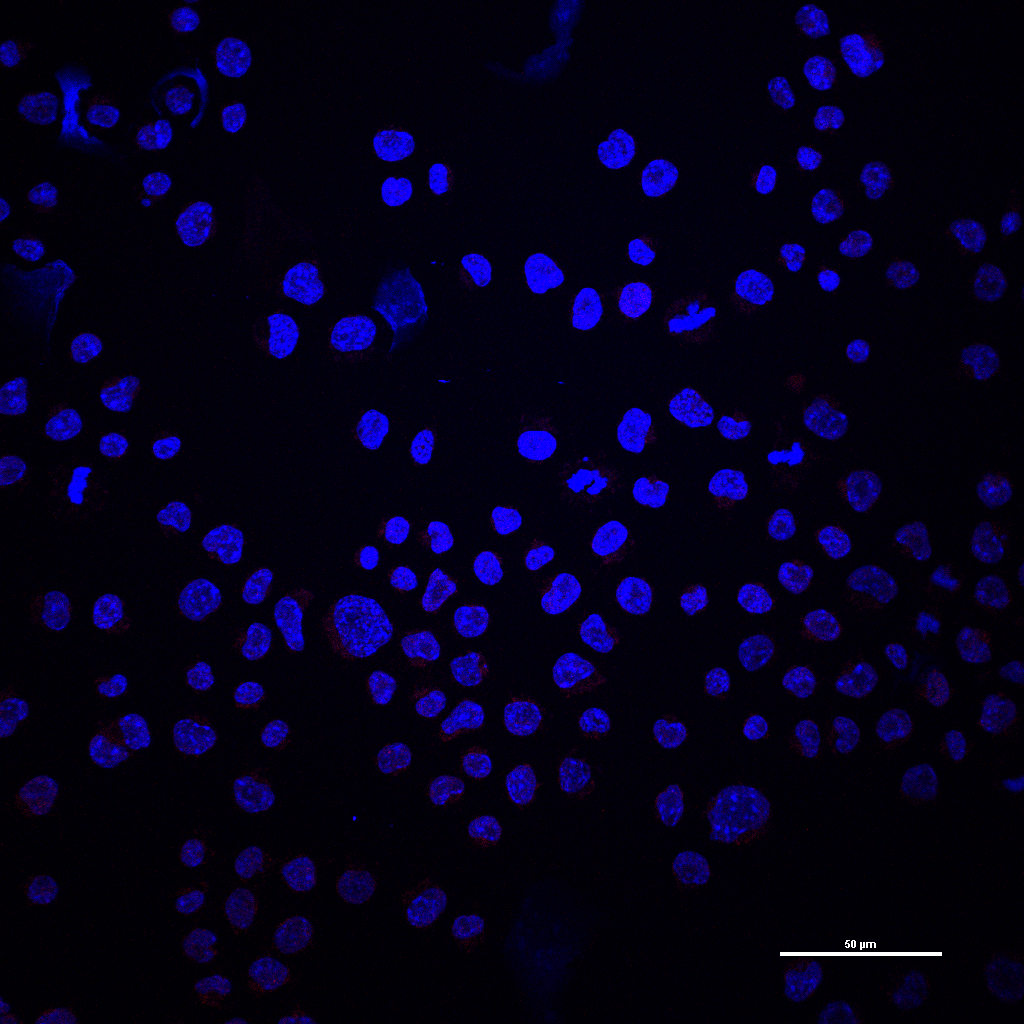

Supplement: Supplementary file 4 — Source data Fig. 2 [file 44318_2025_558_MOESM4_ESM.zip › Figure 2/panel 2C/KD-1_Cdx2/seq9709_seq9709_RGB.tif]

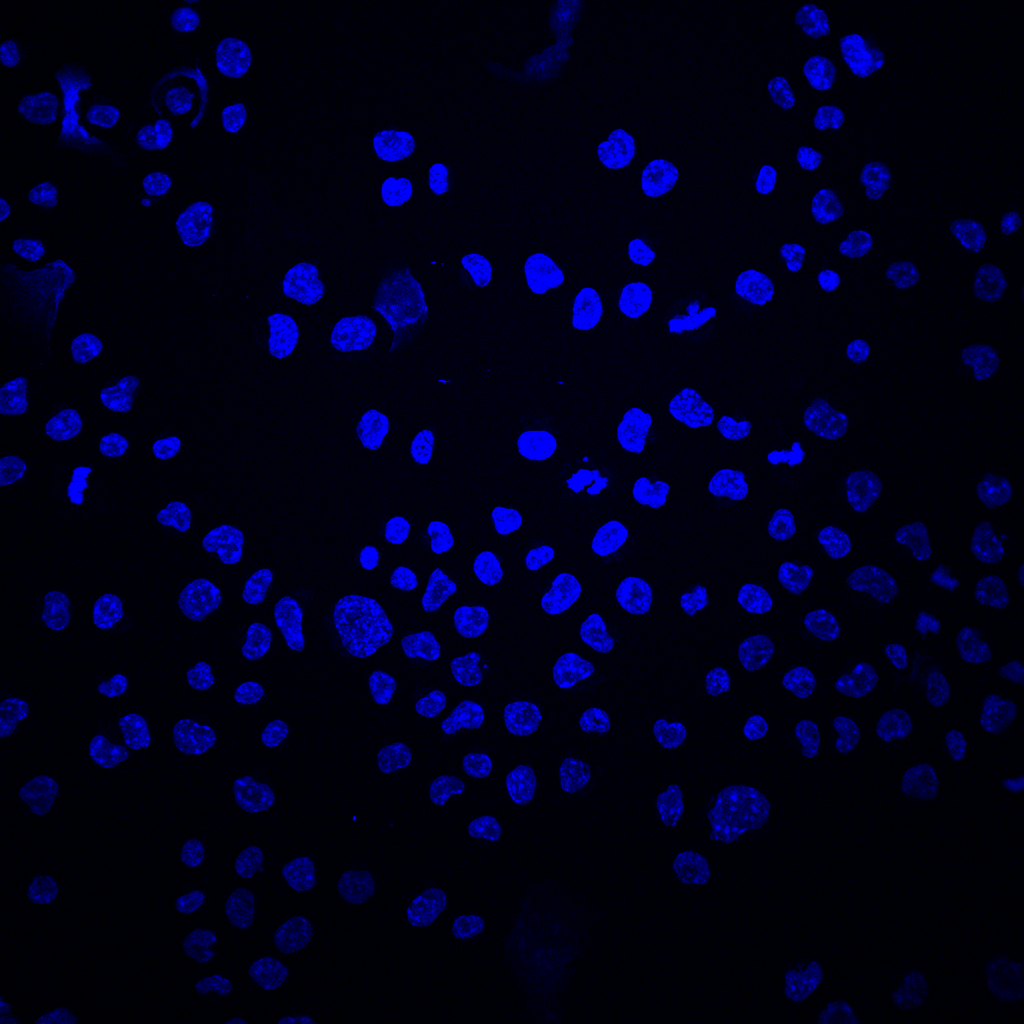

Supplement: Supplementary file 4 — Source data Fig. 2 [file 44318_2025_558_MOESM4_ESM.zip › Figure 2/panel 2C/KD-1_Cdx2/seq9709_seq9709_RGB_DAPI.tif]

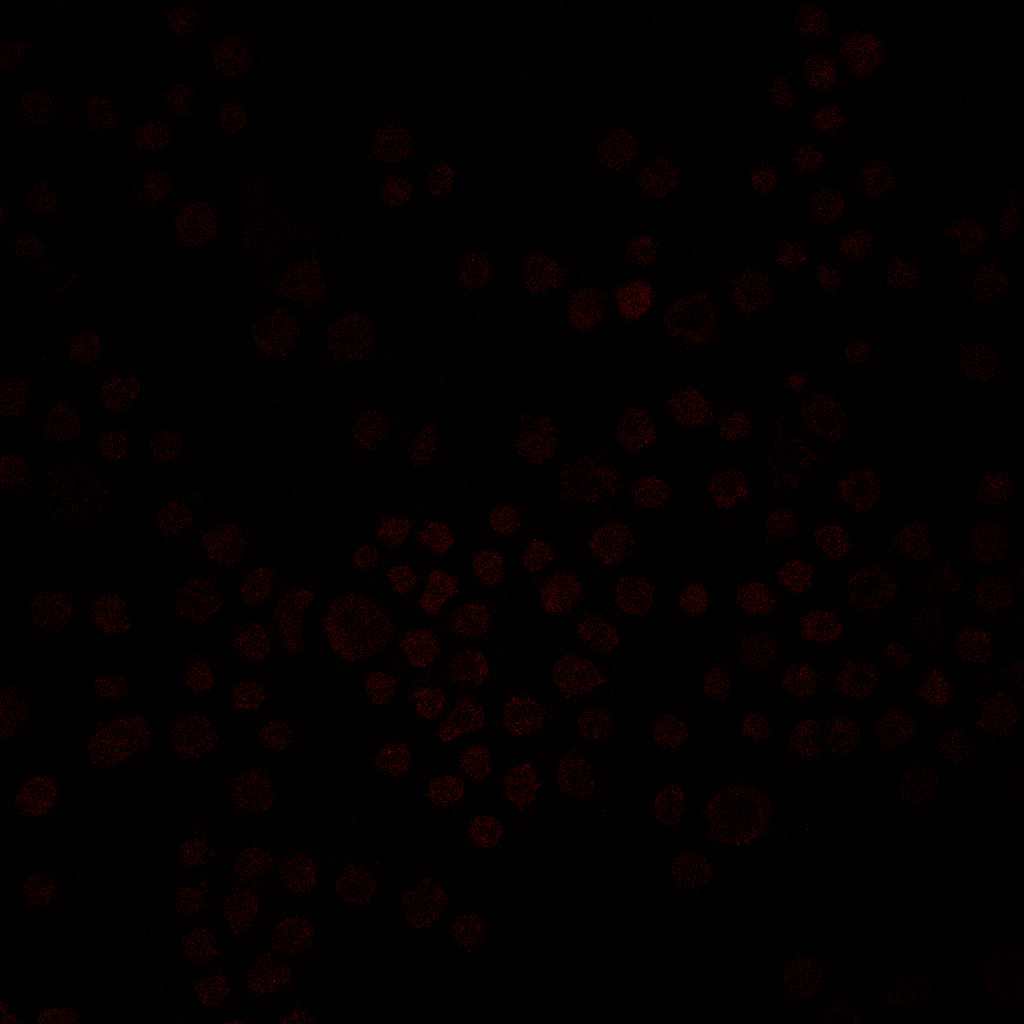

Supplement: Supplementary file 4 — Source data Fig. 2 [file 44318_2025_558_MOESM4_ESM.zip › Figure 2/panel 2C/KD-1_Cdx2/seq9709_seq9709_RGB_Texas Red.tif]

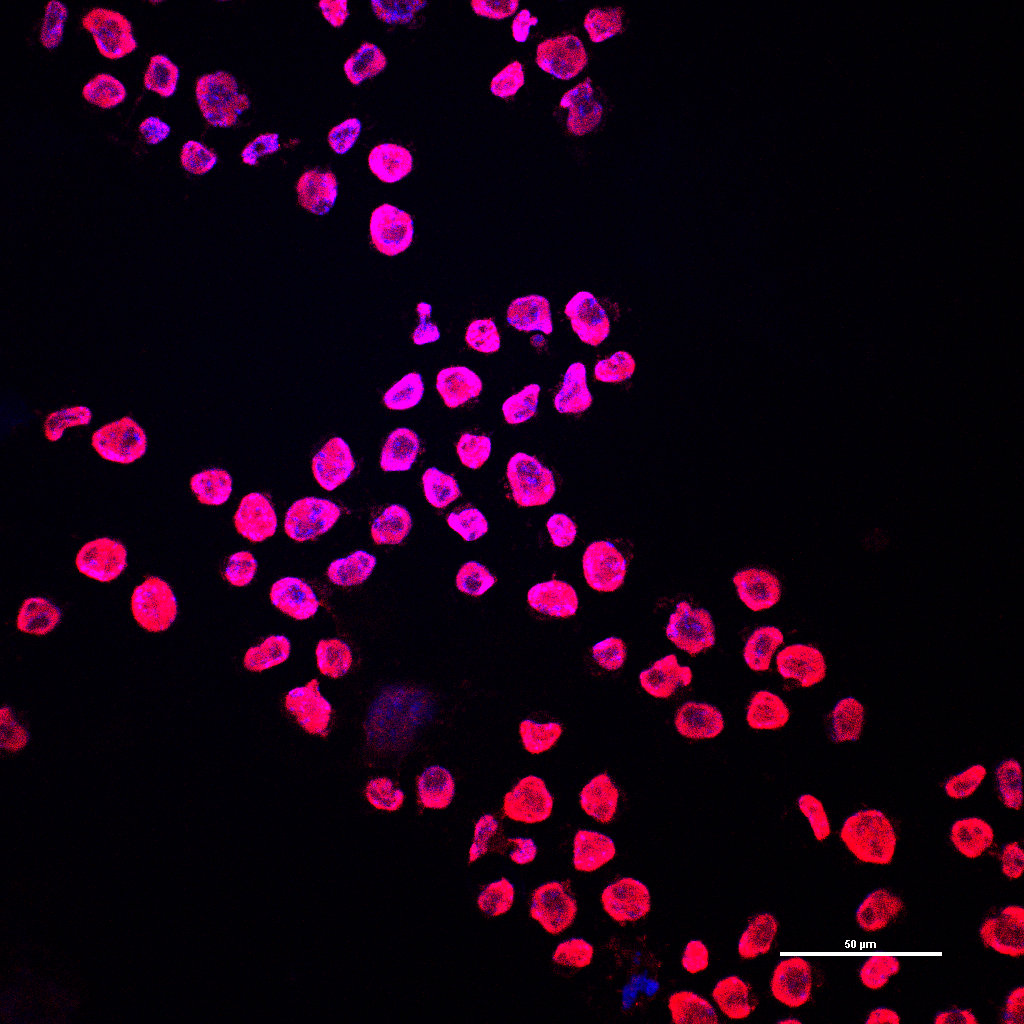

Supplement: Supplementary file 4 — Source data Fig. 2 [file 44318_2025_558_MOESM4_ESM.zip › Figure 2/panel 2C/KD-1_Oct4/seq9701_seq9701_RGB.tif]

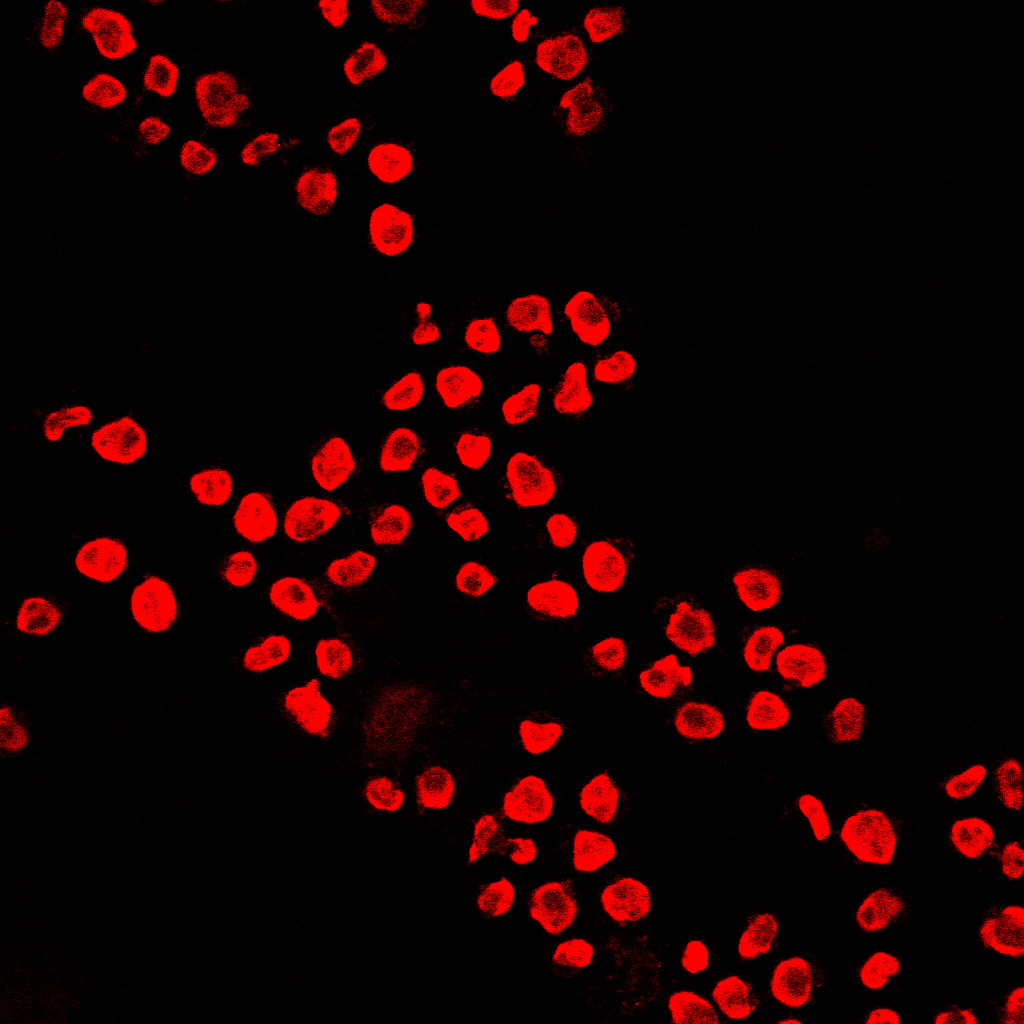

Supplement: Supplementary file 4 — Source data Fig. 2 [file 44318_2025_558_MOESM4_ESM.zip › Figure 2/panel 2C/KD-1_Oct4/seq9701_seq9701_RGB_Texas Red.tif]

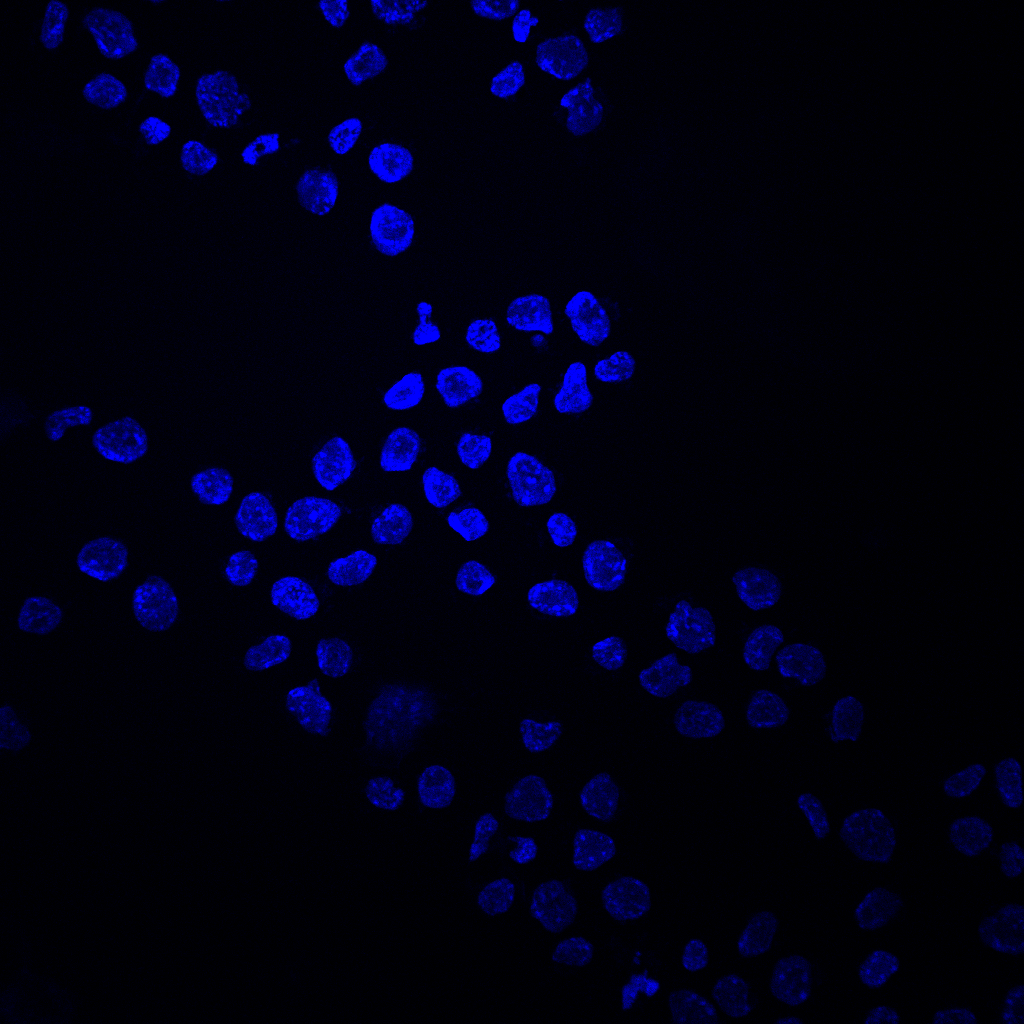

Supplement: Supplementary file 4 — Source data Fig. 2 [file 44318_2025_558_MOESM4_ESM.zip › Figure 2/panel 2C/KD-1_Oct4/seq9701_seq9701_RGB_DAPI.tif]

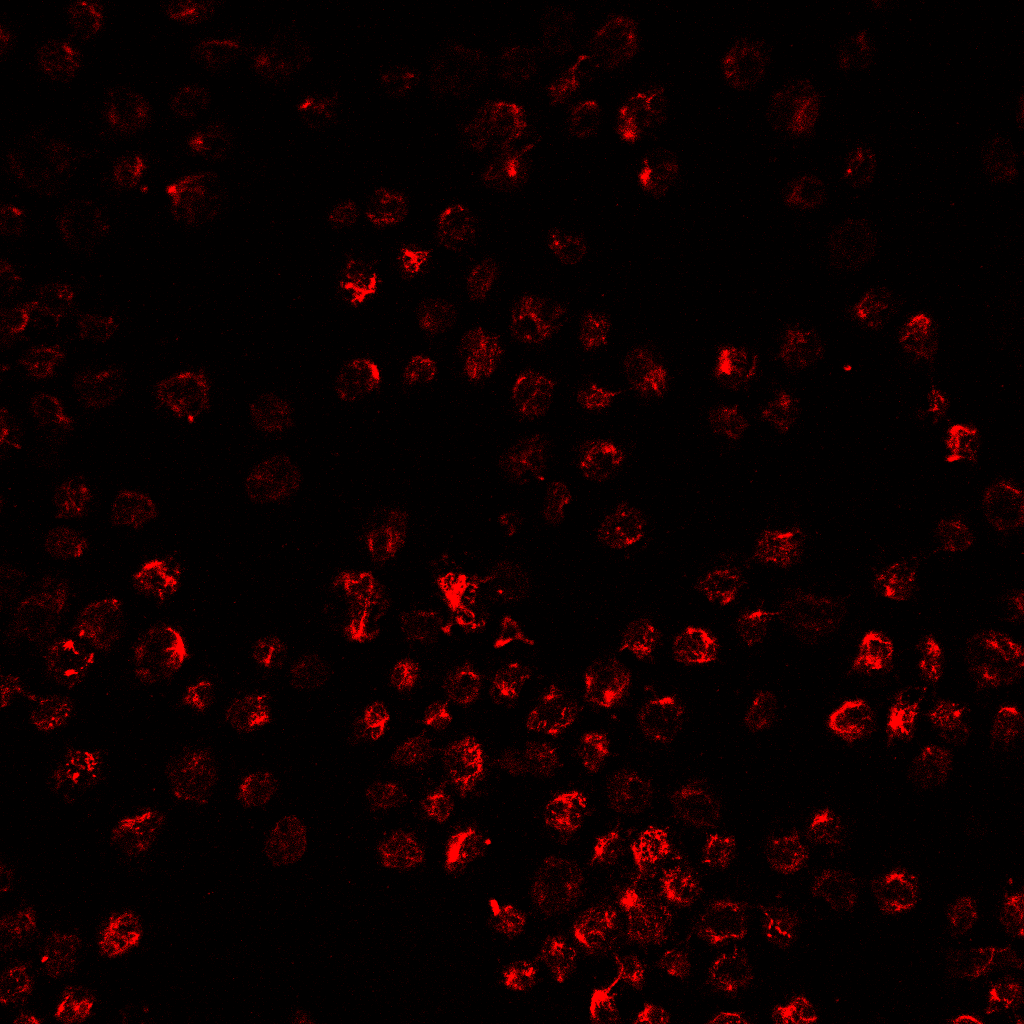

Supplement: Supplementary file 4 — Source data Fig. 2 [file 44318_2025_558_MOESM4_ESM.zip › Figure 2/panel 2C/KD-1_Nestin/seq9704_seq9704_RGB_Texas Red.tif]

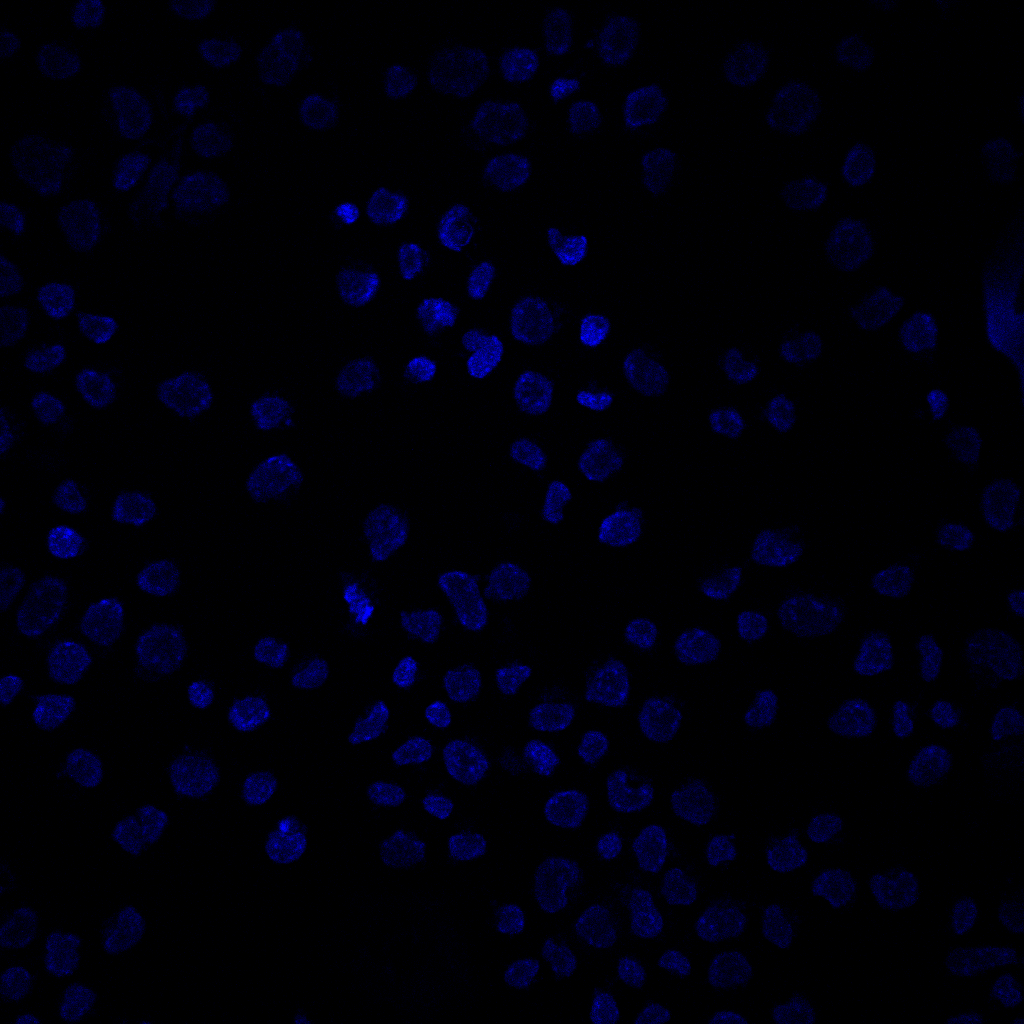

Supplement: Supplementary file 4 — Source data Fig. 2 [file 44318_2025_558_MOESM4_ESM.zip › Figure 2/panel 2C/KD-1_Nestin/seq9704_seq9704_RGB_DAPI.tif]

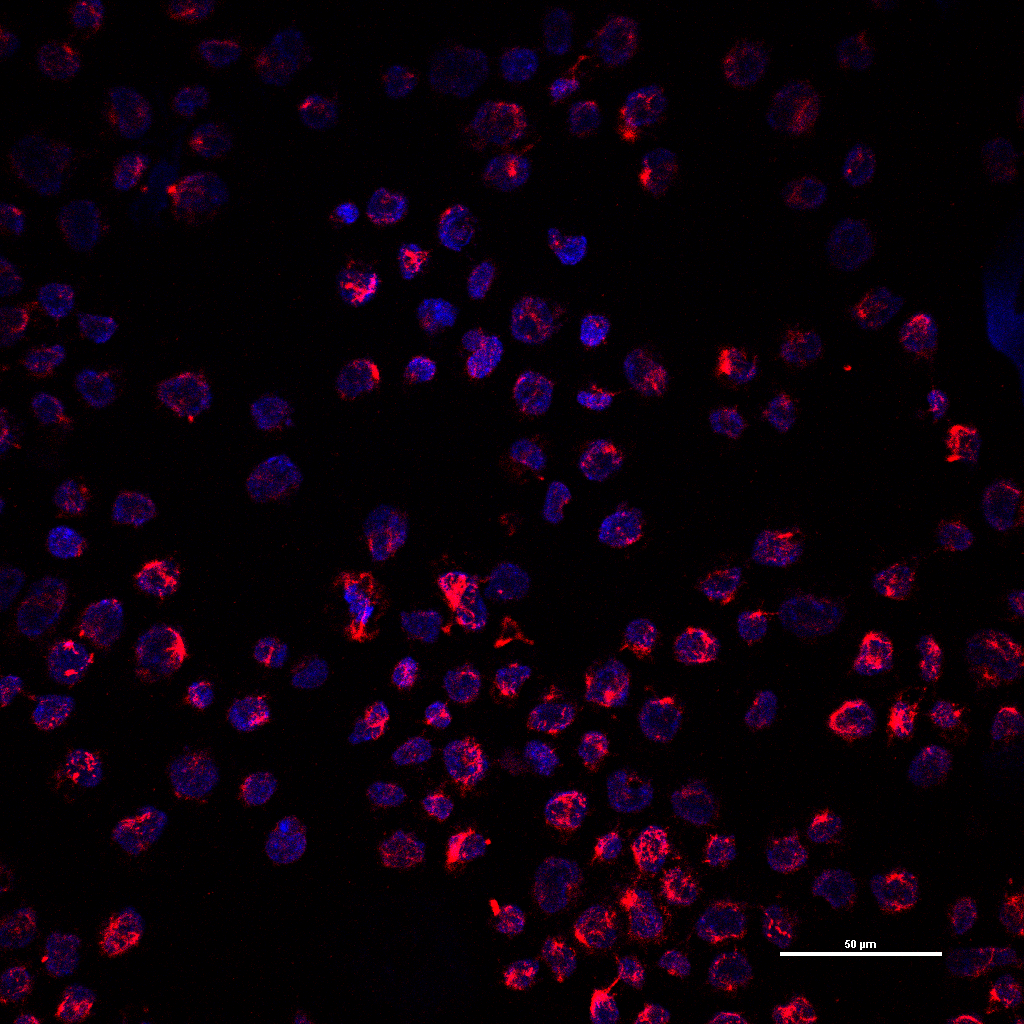

Supplement: Supplementary file 4 — Source data Fig. 2 [file 44318_2025_558_MOESM4_ESM.zip › Figure 2/panel 2C/KD-1_Nestin/seq9704_seq9704_RGB.tif]

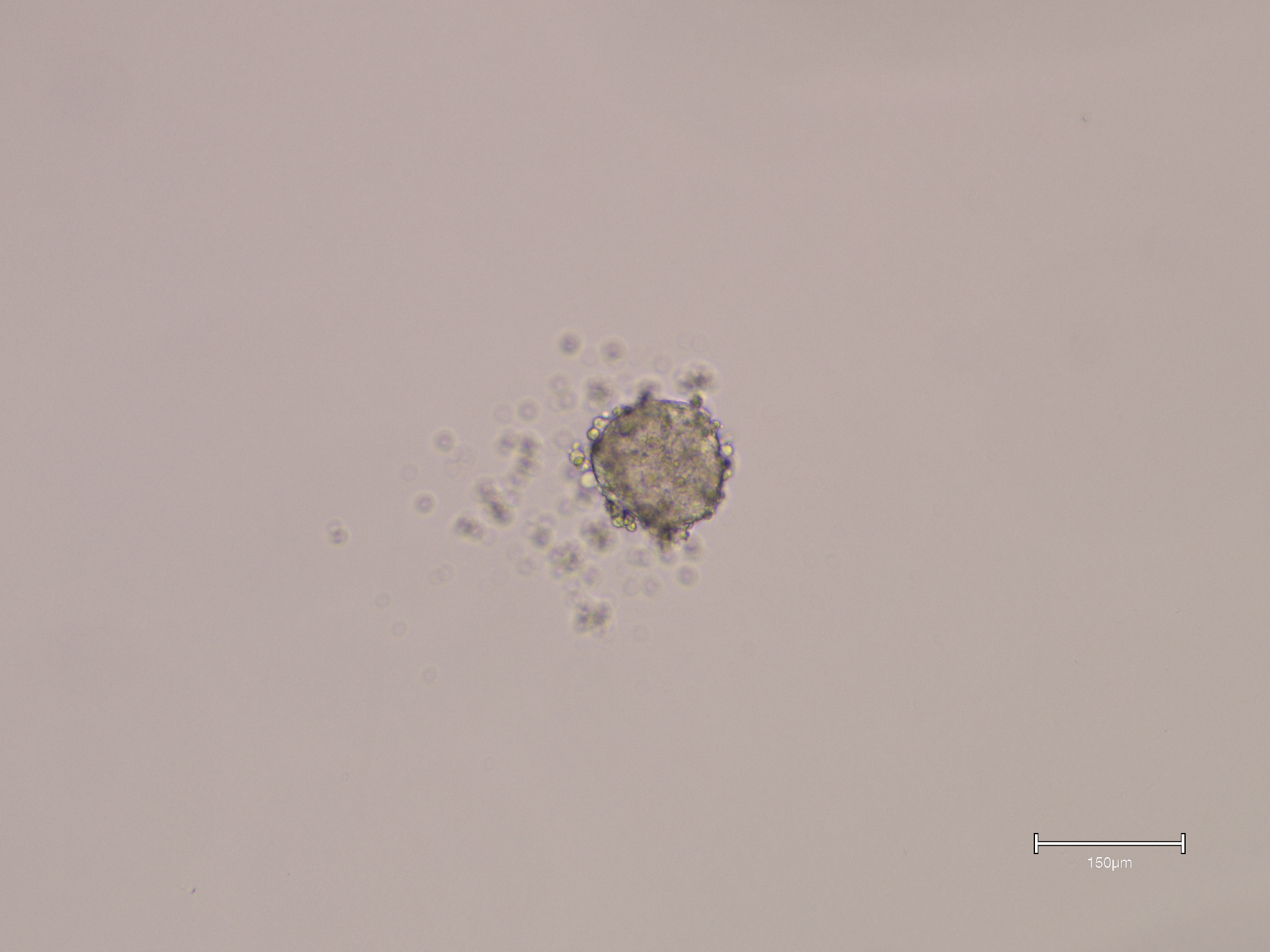

Supplement: Supplementary file 5 — Source data Fig. 3 [file 44318_2025_558_MOESM5_ESM.zip › Figure 3/panel 3A/KD-1_150cell.tiff]

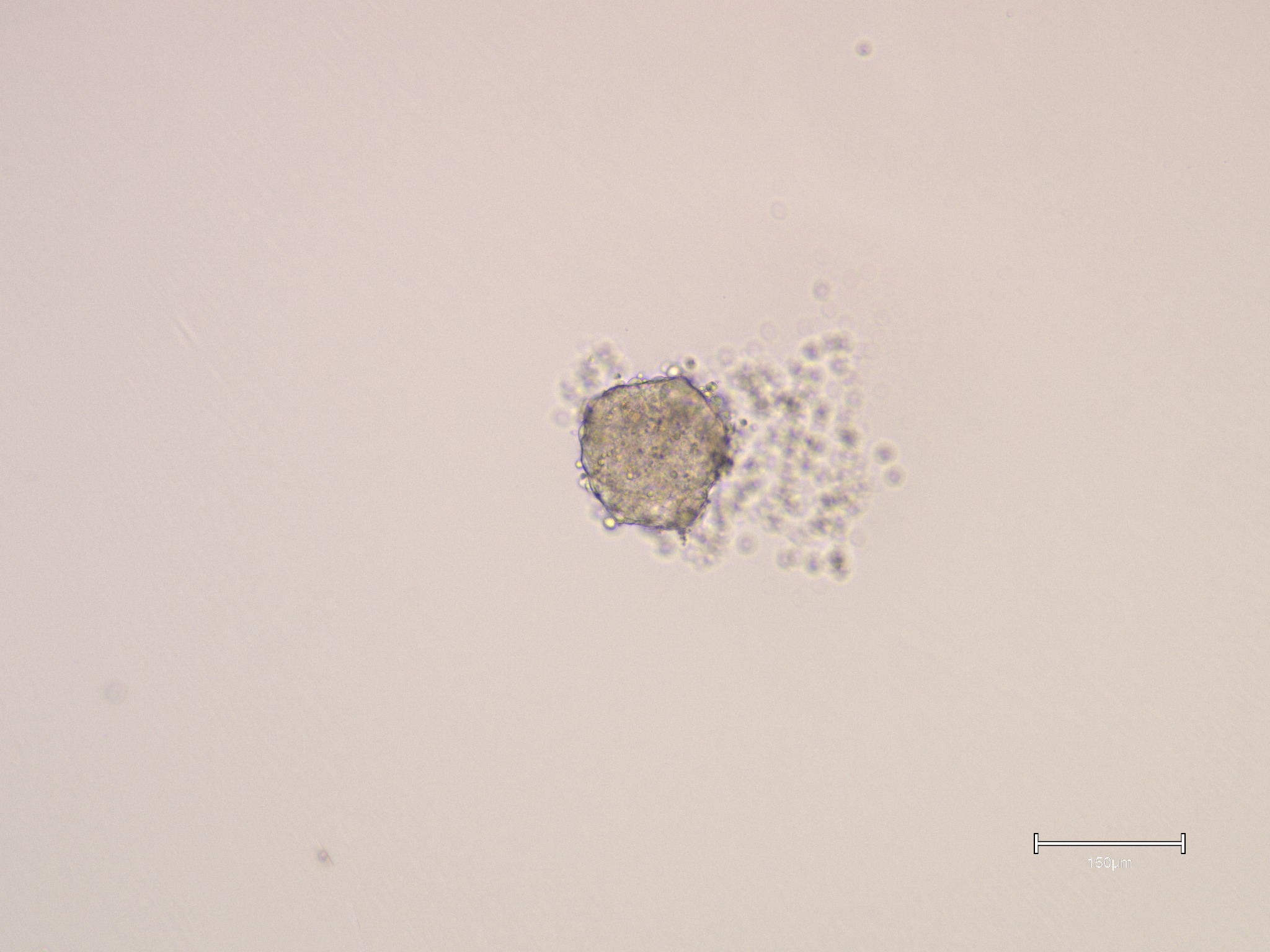

Supplement: Supplementary file 5 — Source data Fig. 3 [file 44318_2025_558_MOESM5_ESM.zip › Figure 3/panel 3A/KD-1_200cell.tiff]

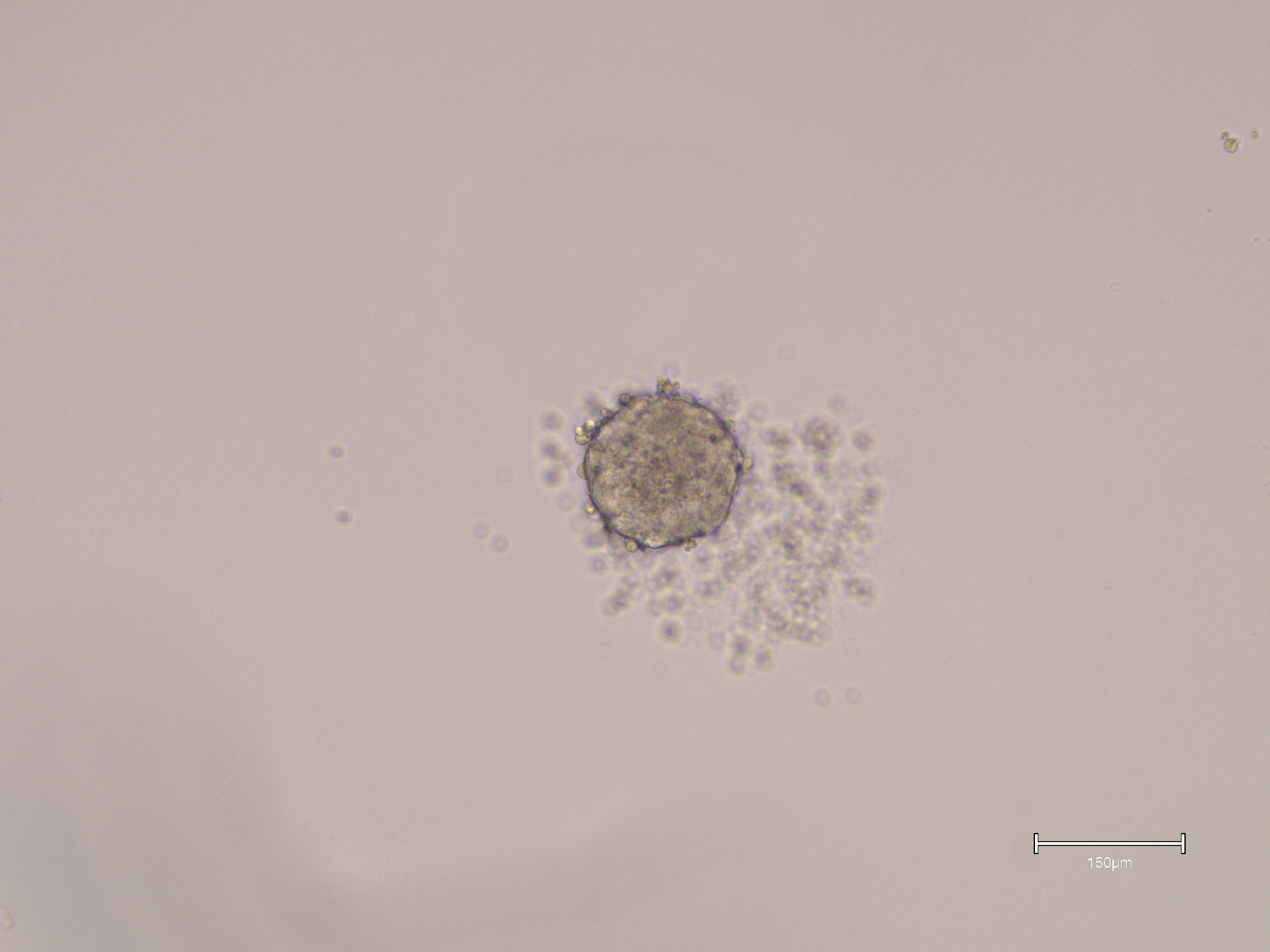

Supplement: Supplementary file 5 — Source data Fig. 3 [file 44318_2025_558_MOESM5_ESM.zip › Figure 3/panel 3A/KD-2_250cell.tiff]

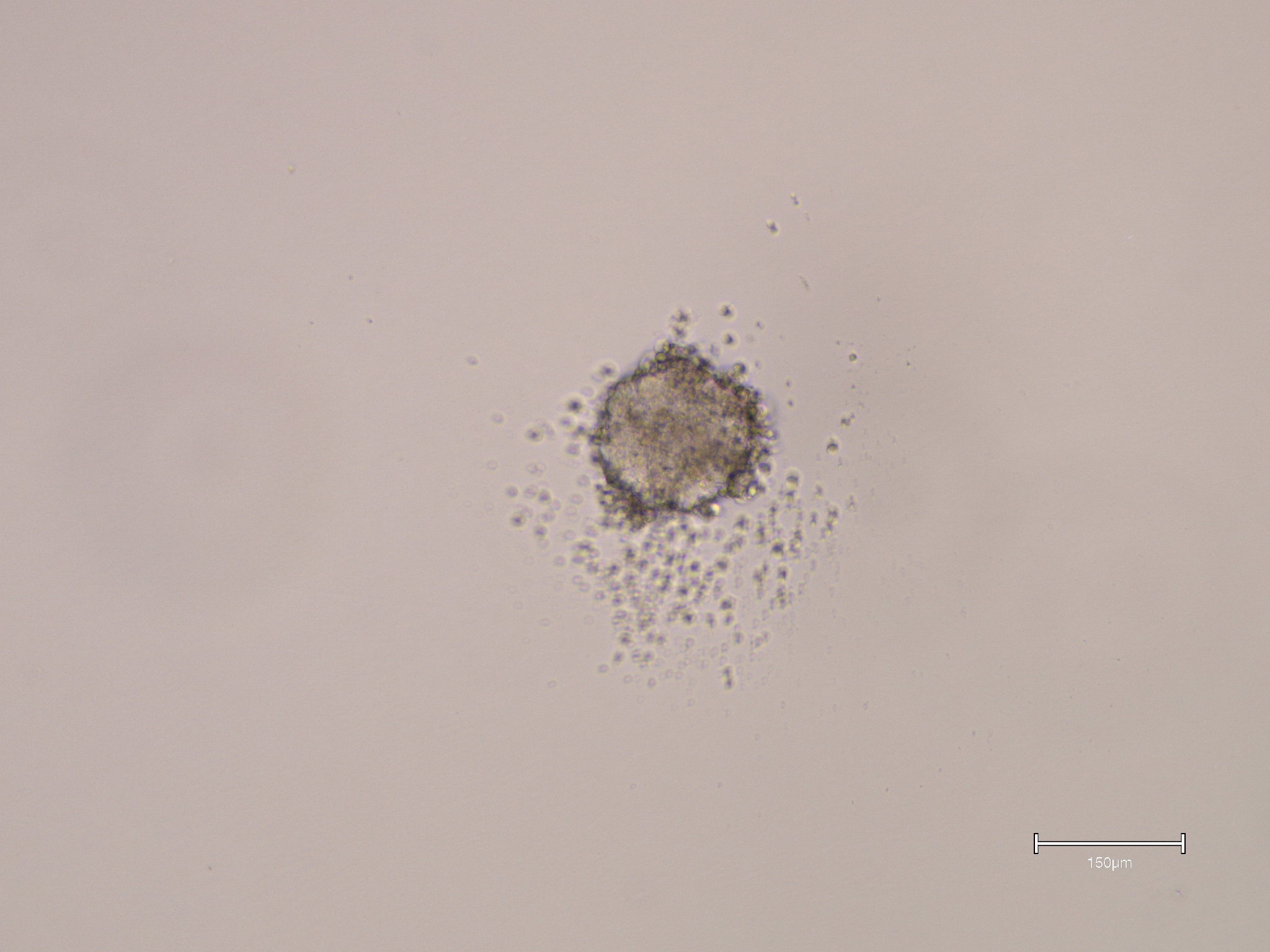

Supplement: Supplementary file 5 — Source data Fig. 3 [file 44318_2025_558_MOESM5_ESM.zip › Figure 3/panel 3A/NT_250cell.tiff]

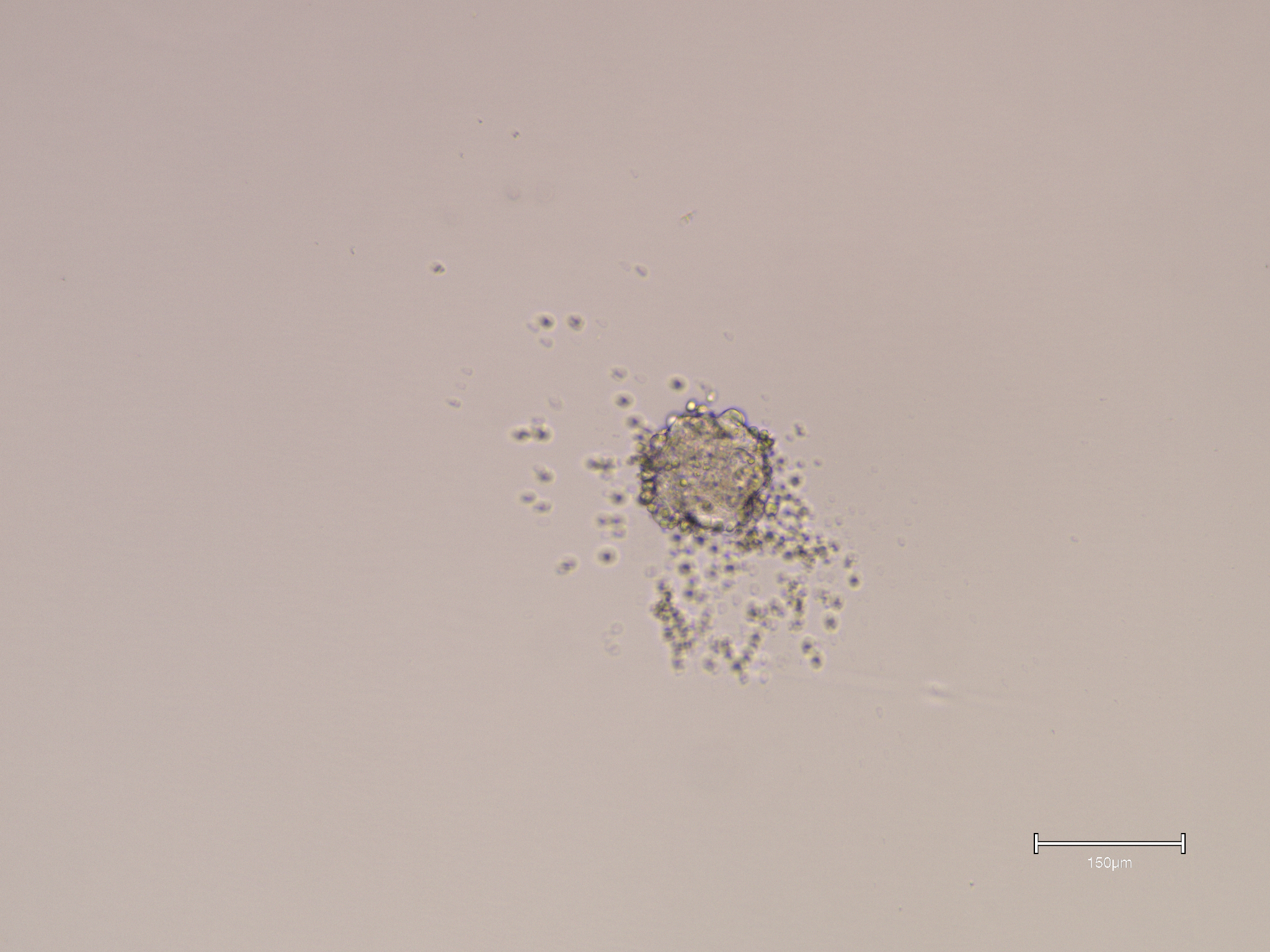

Supplement: Supplementary file 5 — Source data Fig. 3 [file 44318_2025_558_MOESM5_ESM.zip › Figure 3/panel 3A/NT_100cell.tiff]

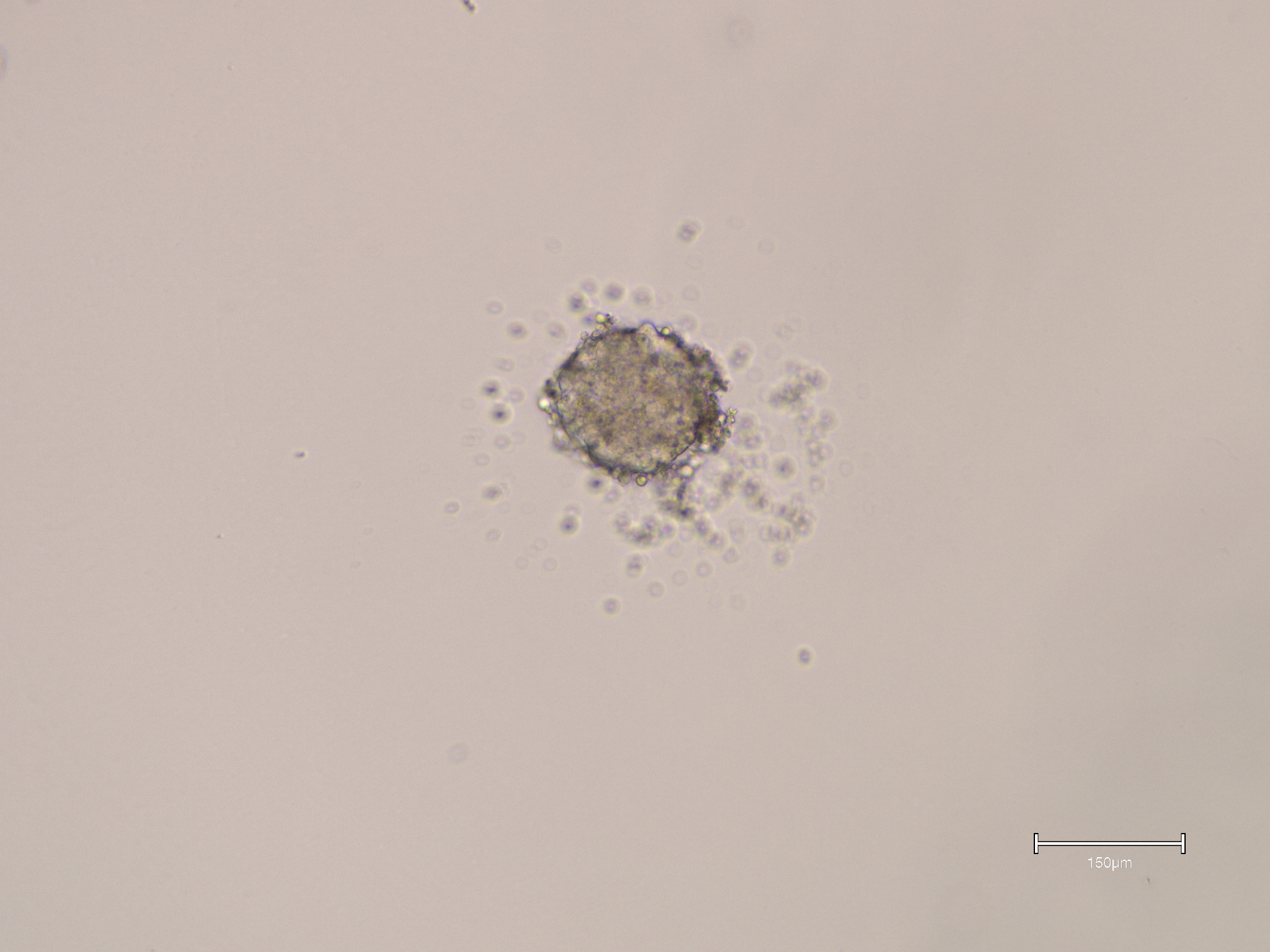

Supplement: Supplementary file 5 — Source data Fig. 3 [file 44318_2025_558_MOESM5_ESM.zip › Figure 3/panel 3A/NT_200cell.tiff]

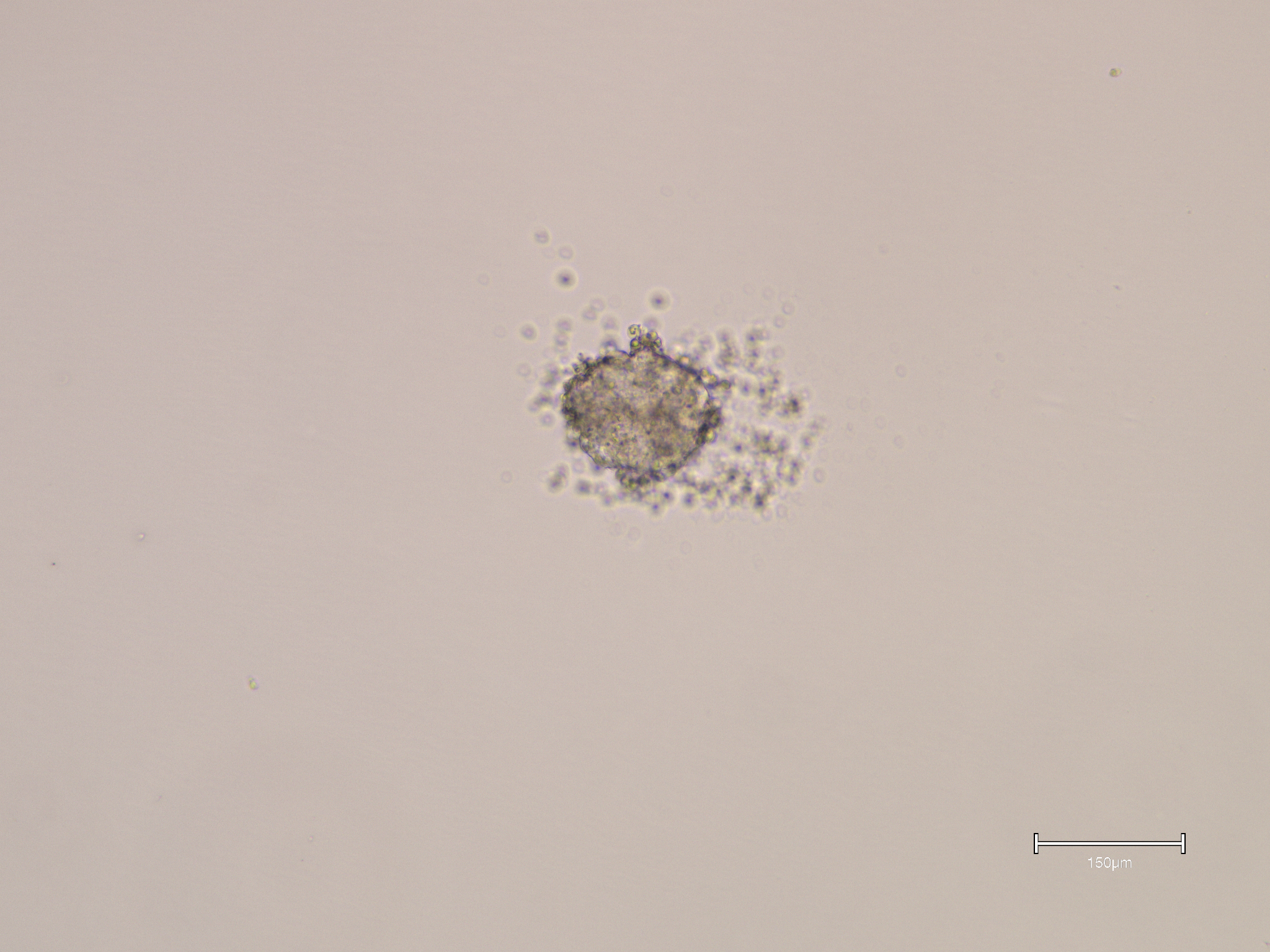

Supplement: Supplementary file 5 — Source data Fig. 3 [file 44318_2025_558_MOESM5_ESM.zip › Figure 3/panel 3A/NT_150cell.tiff]

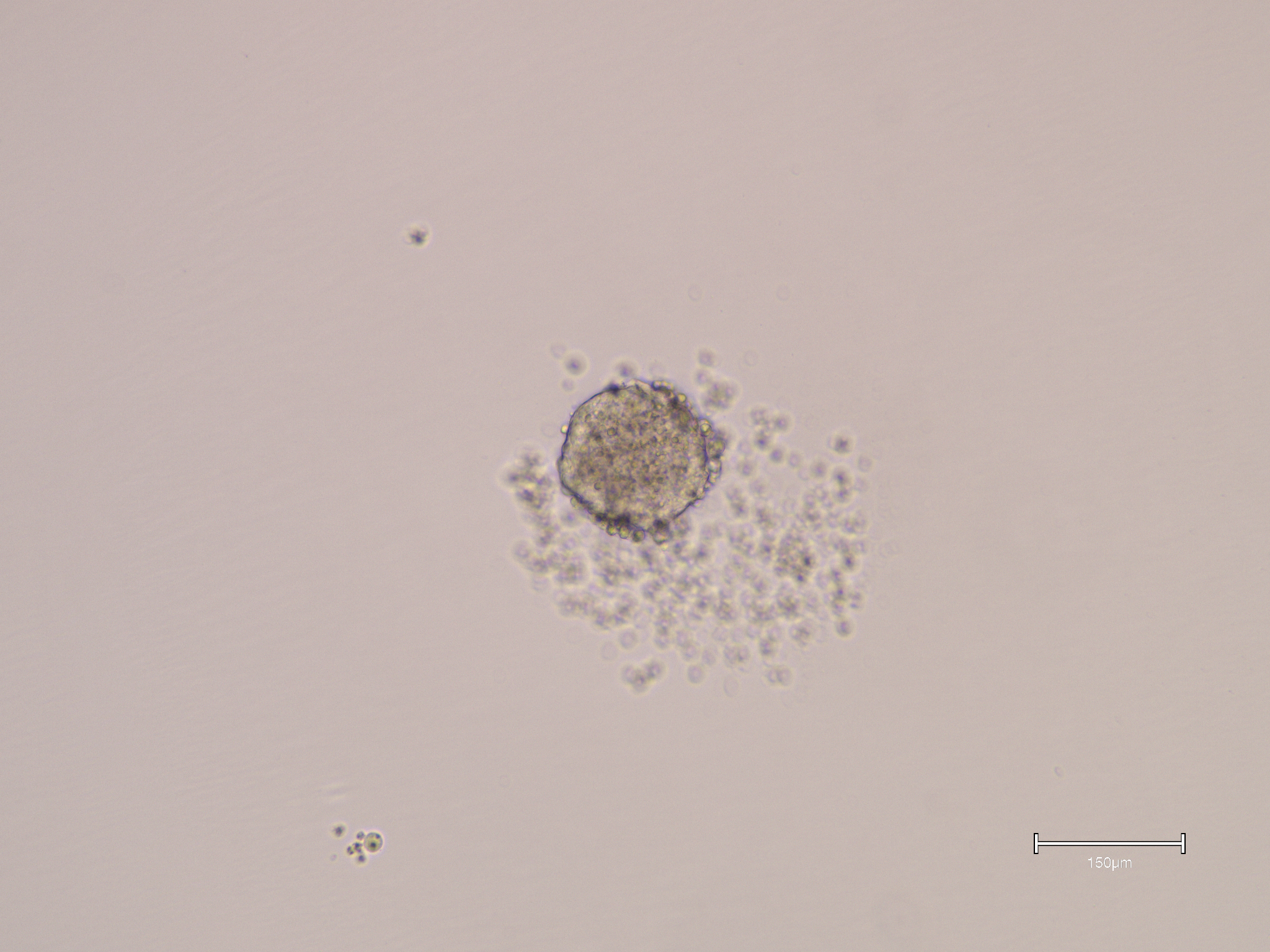

Supplement: Supplementary file 5 — Source data Fig. 3 [file 44318_2025_558_MOESM5_ESM.zip › Figure 3/panel 3A/KD-2_200cell.tiff]

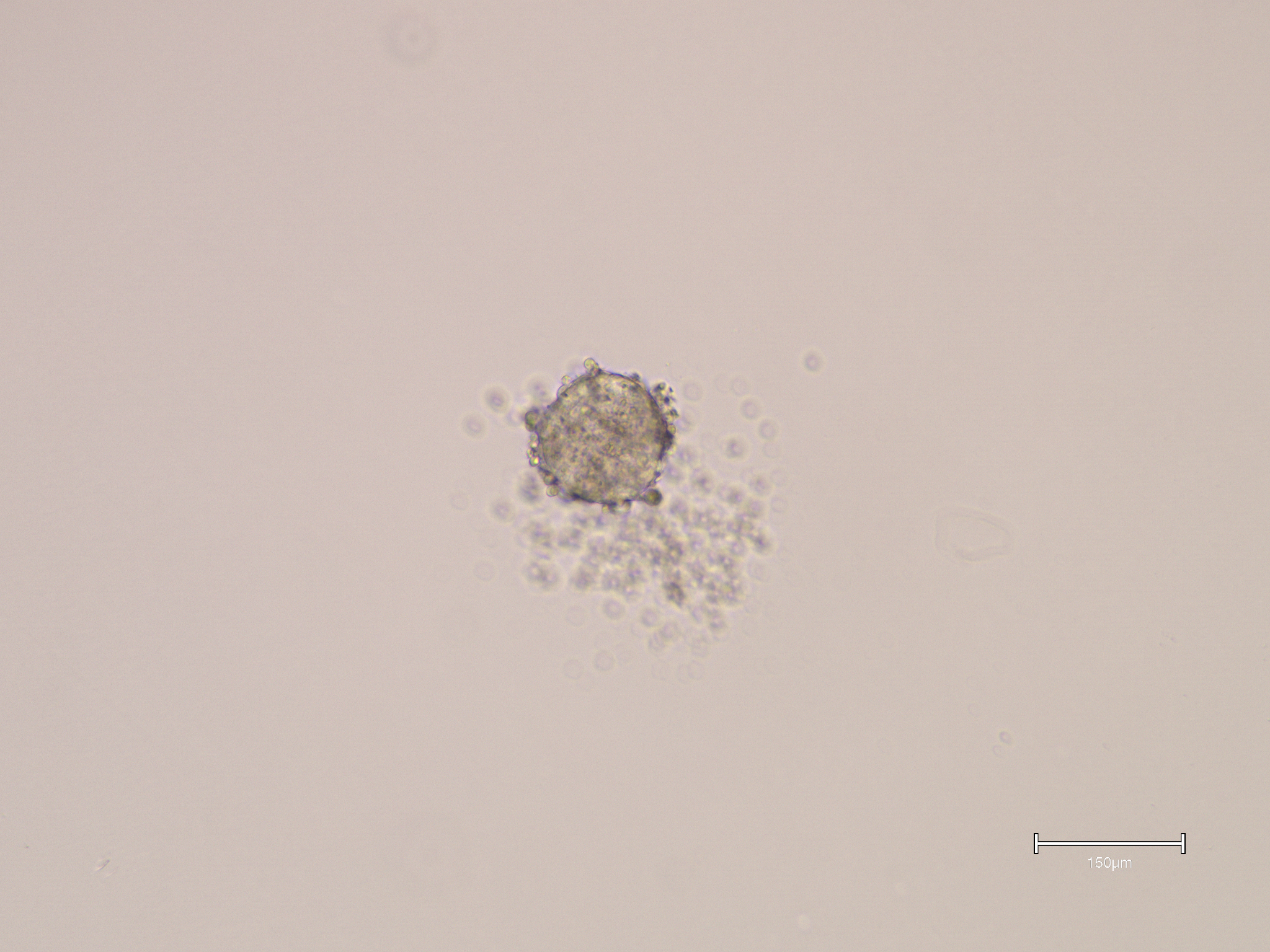

Supplement: Supplementary file 5 — Source data Fig. 3 [file 44318_2025_558_MOESM5_ESM.zip › Figure 3/panel 3A/KD-2_150cell.tiff]

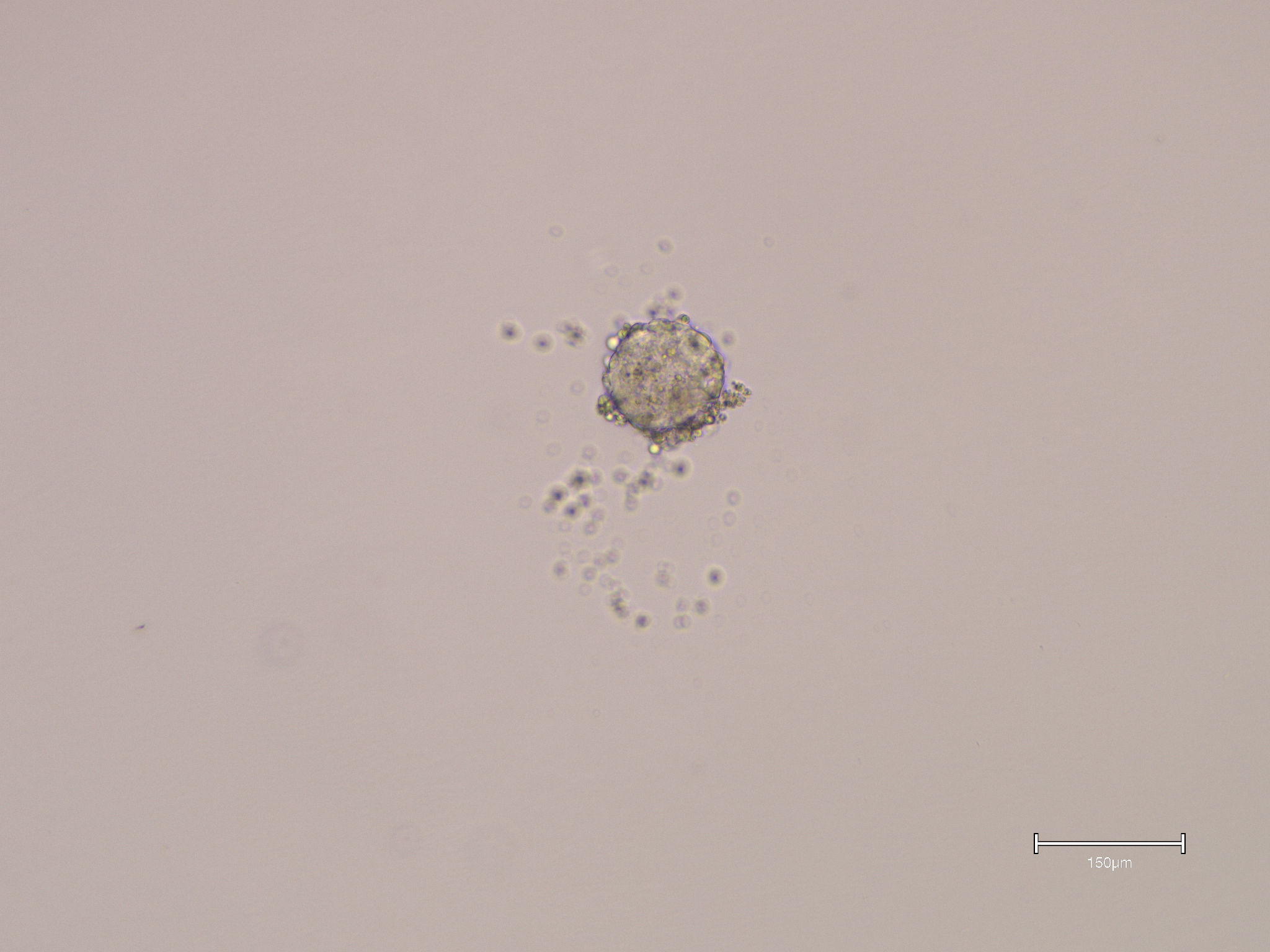

Supplement: Supplementary file 5 — Source data Fig. 3 [file 44318_2025_558_MOESM5_ESM.zip › Figure 3/panel 3A/KD-1_100cell.tiff]

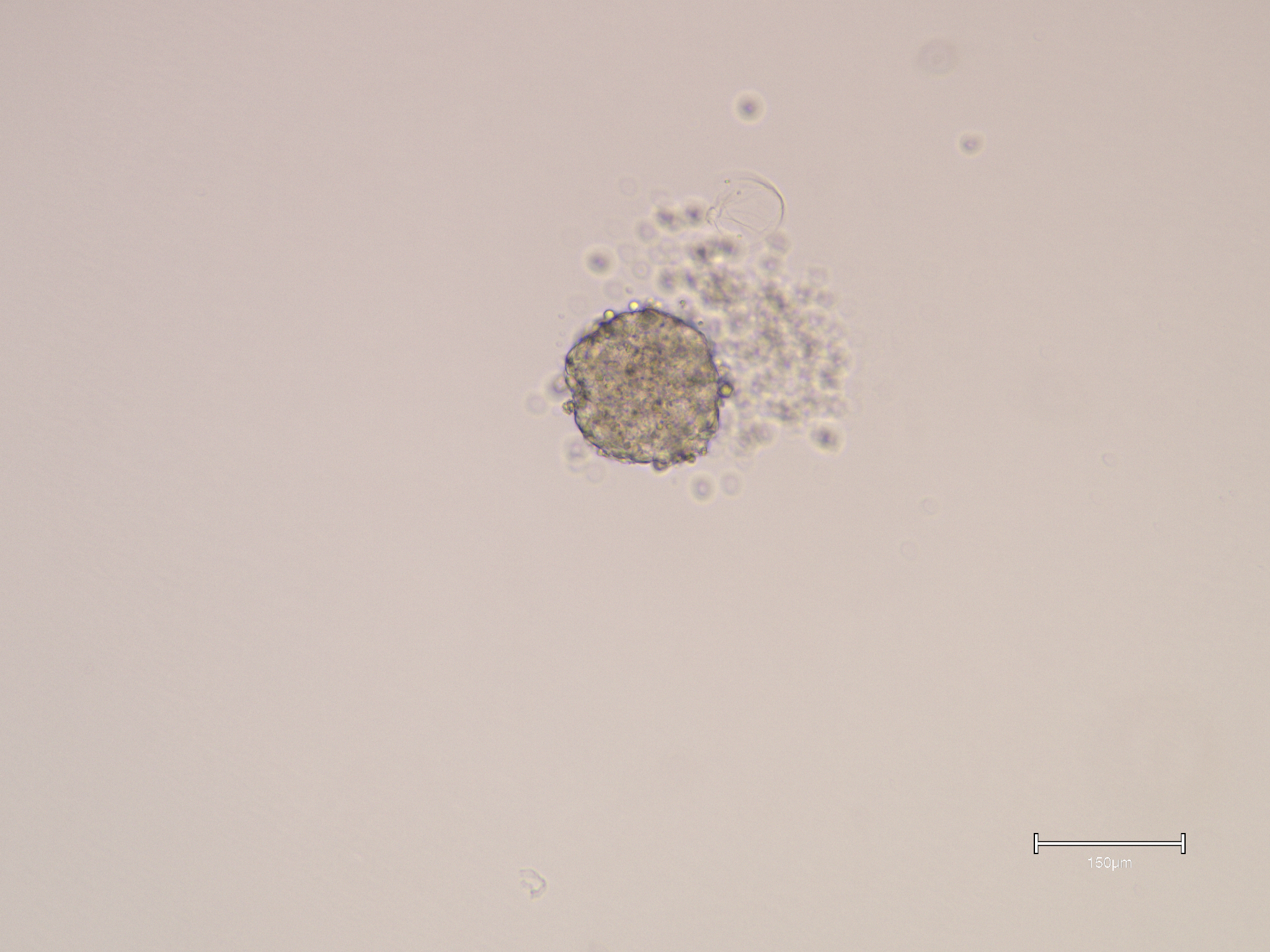

Supplement: Supplementary file 5 — Source data Fig. 3 [file 44318_2025_558_MOESM5_ESM.zip › Figure 3/panel 3A/KD-1_250cell.tiff]

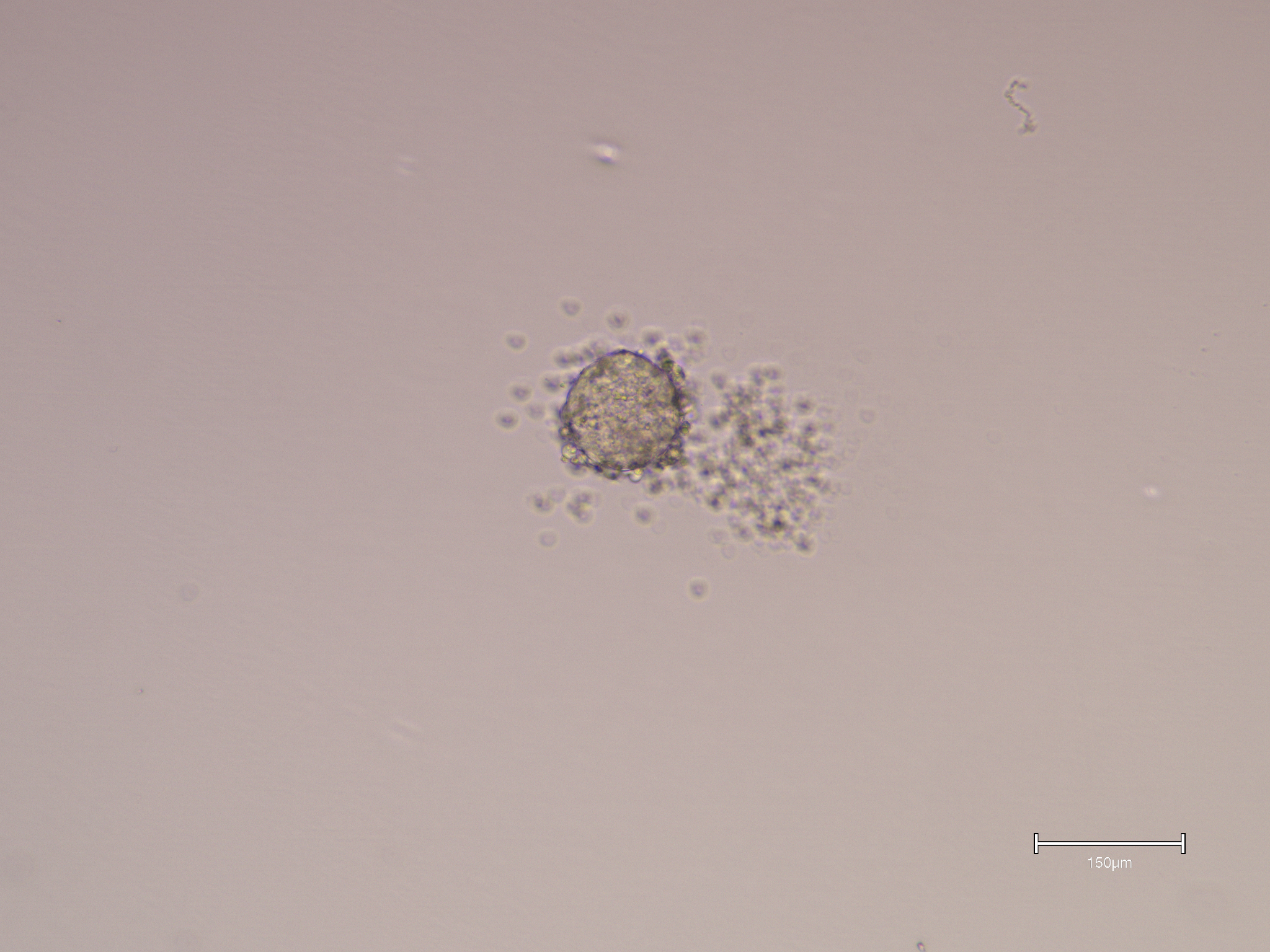

Supplement: Supplementary file 5 — Source data Fig. 3 [file 44318_2025_558_MOESM5_ESM.zip › Figure 3/panel 3A/KD-2_100cell.tiff]

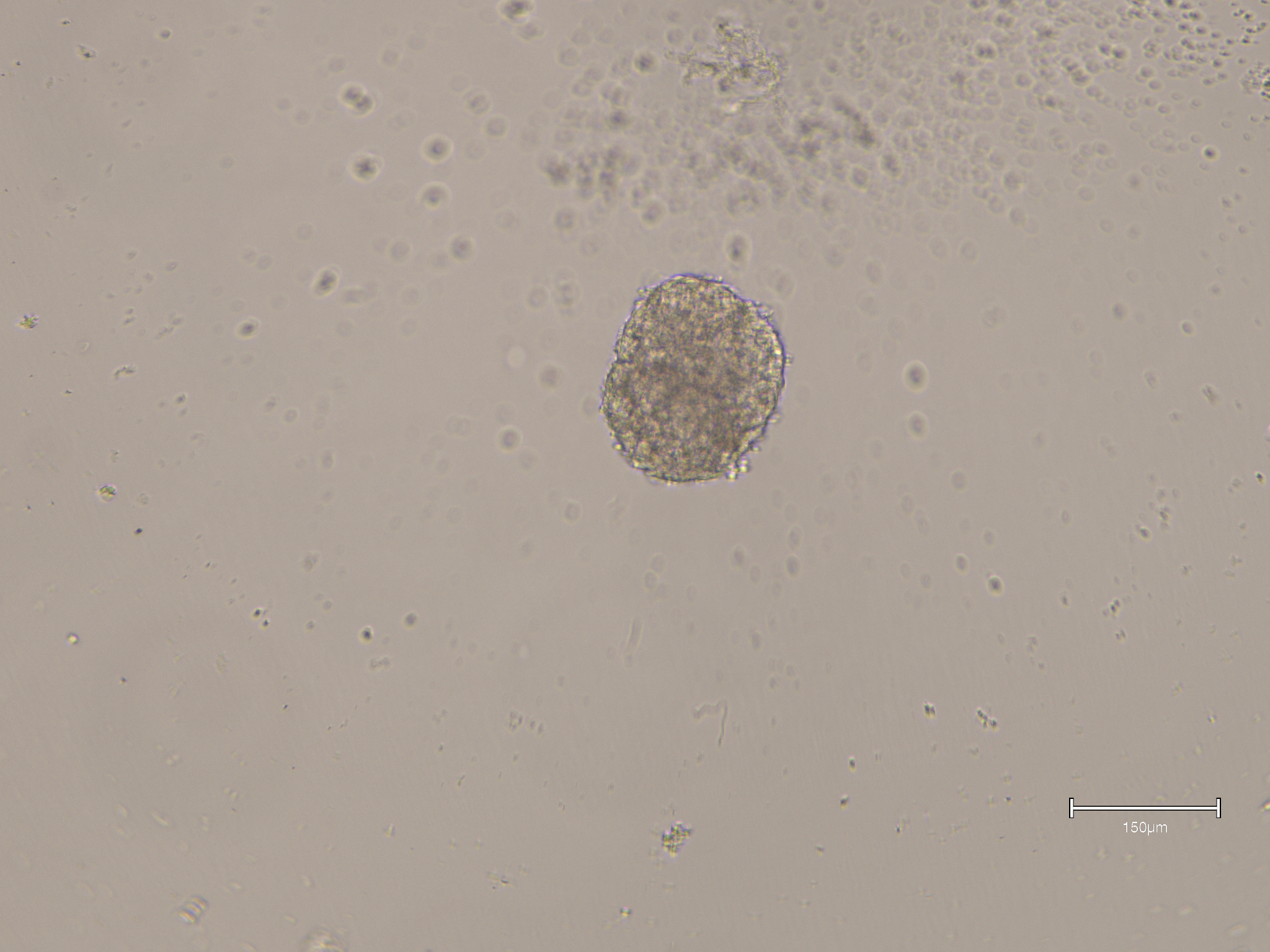

Supplement: Supplementary file 5 — Source data Fig. 3 [file 44318_2025_558_MOESM5_ESM.zip › Figure 3/panel 3B/KD-1_150cell.tiff]

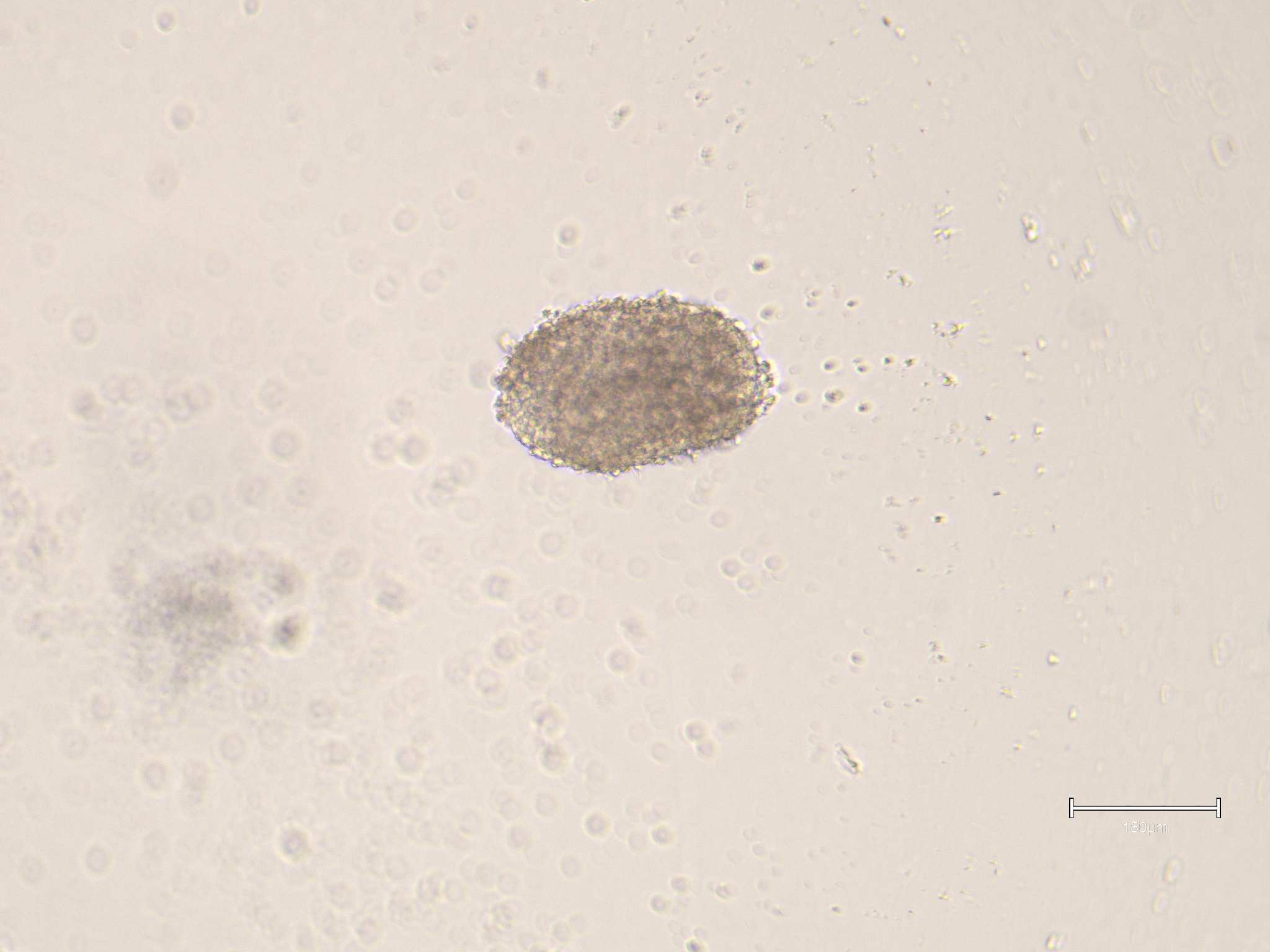

Supplement: Supplementary file 5 — Source data Fig. 3 [file 44318_2025_558_MOESM5_ESM.zip › Figure 3/panel 3B/KD-1_200cell.tiff]

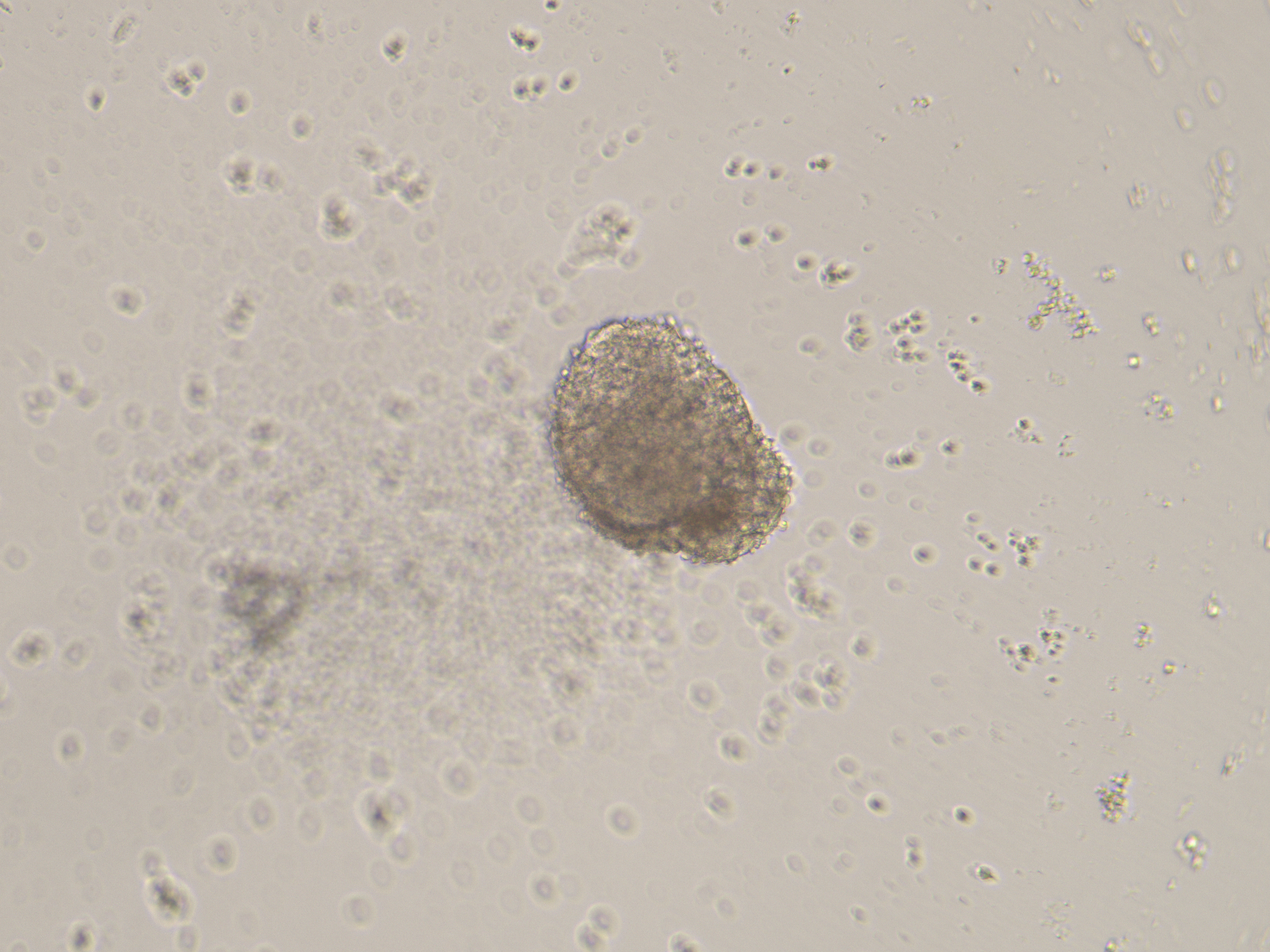

Supplement: Supplementary file 5 — Source data Fig. 3 [file 44318_2025_558_MOESM5_ESM.zip › Figure 3/panel 3B/KD-2_250cell.tiff]

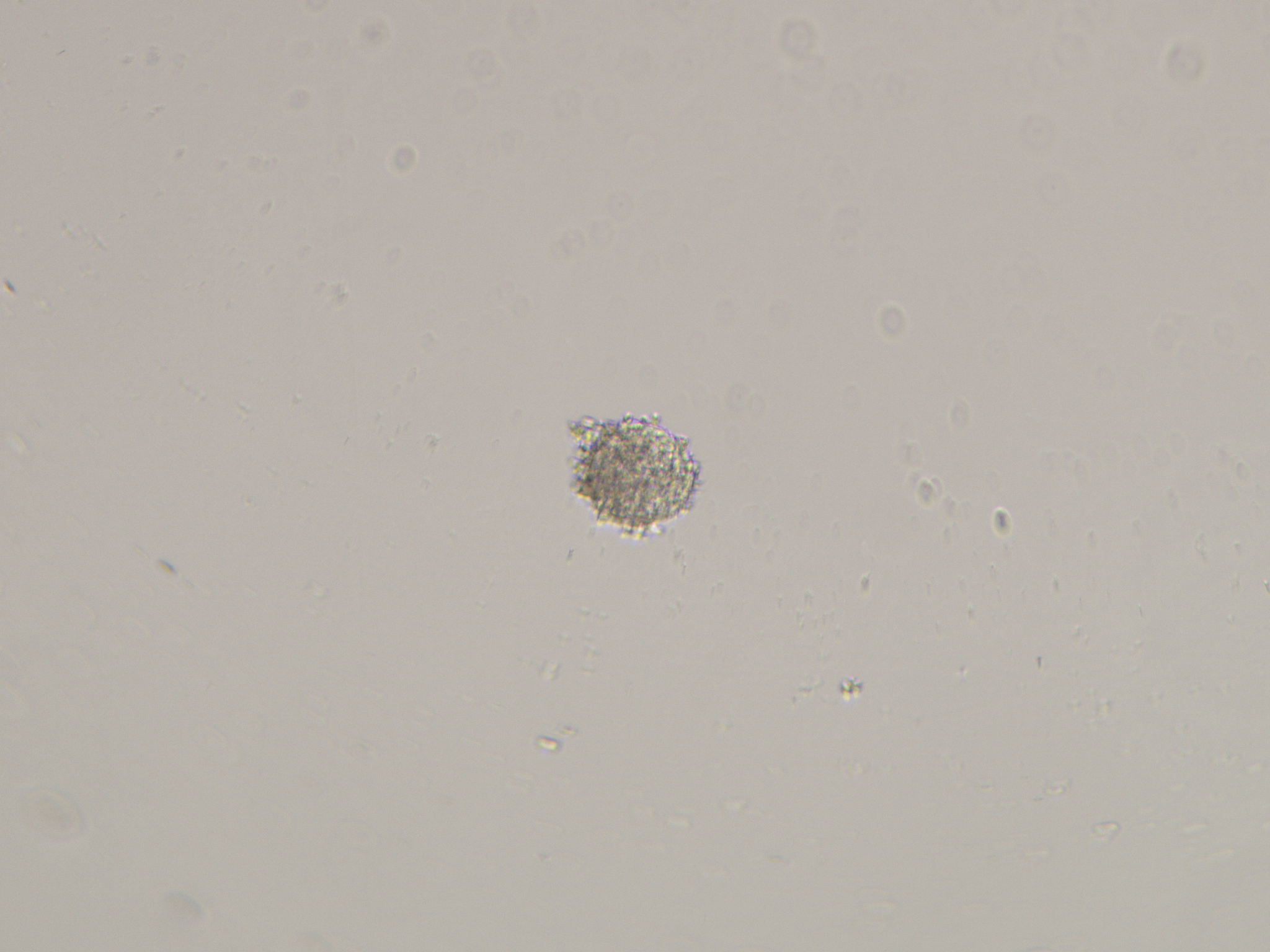

Supplement: Supplementary file 5 — Source data Fig. 3 [file 44318_2025_558_MOESM5_ESM.zip › Figure 3/panel 3B/KD-2_100cell.tiff]

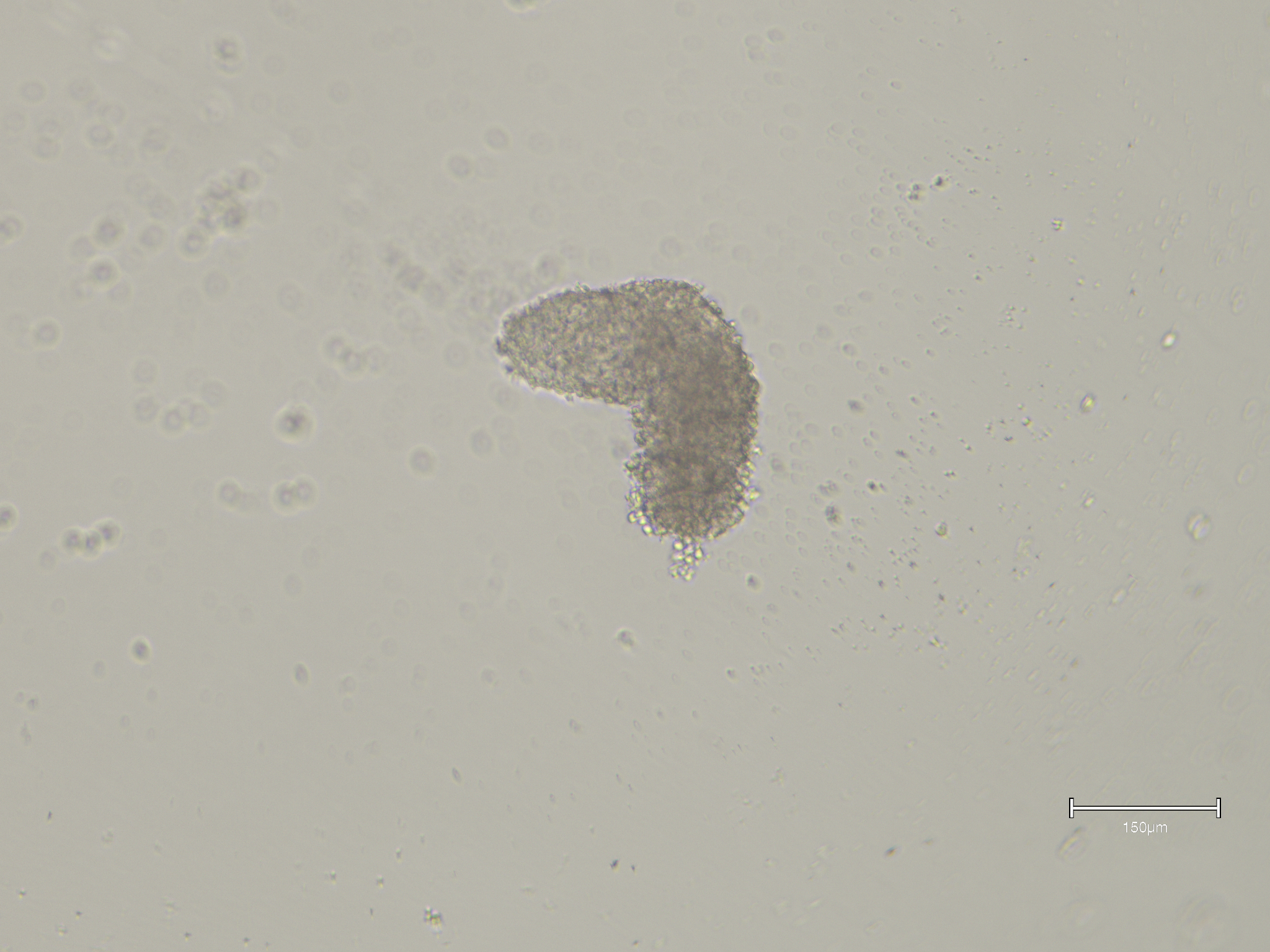

Supplement: Supplementary file 5 — Source data Fig. 3 [file 44318_2025_558_MOESM5_ESM.zip › Figure 3/panel 3B/NT_250cell.tiff]

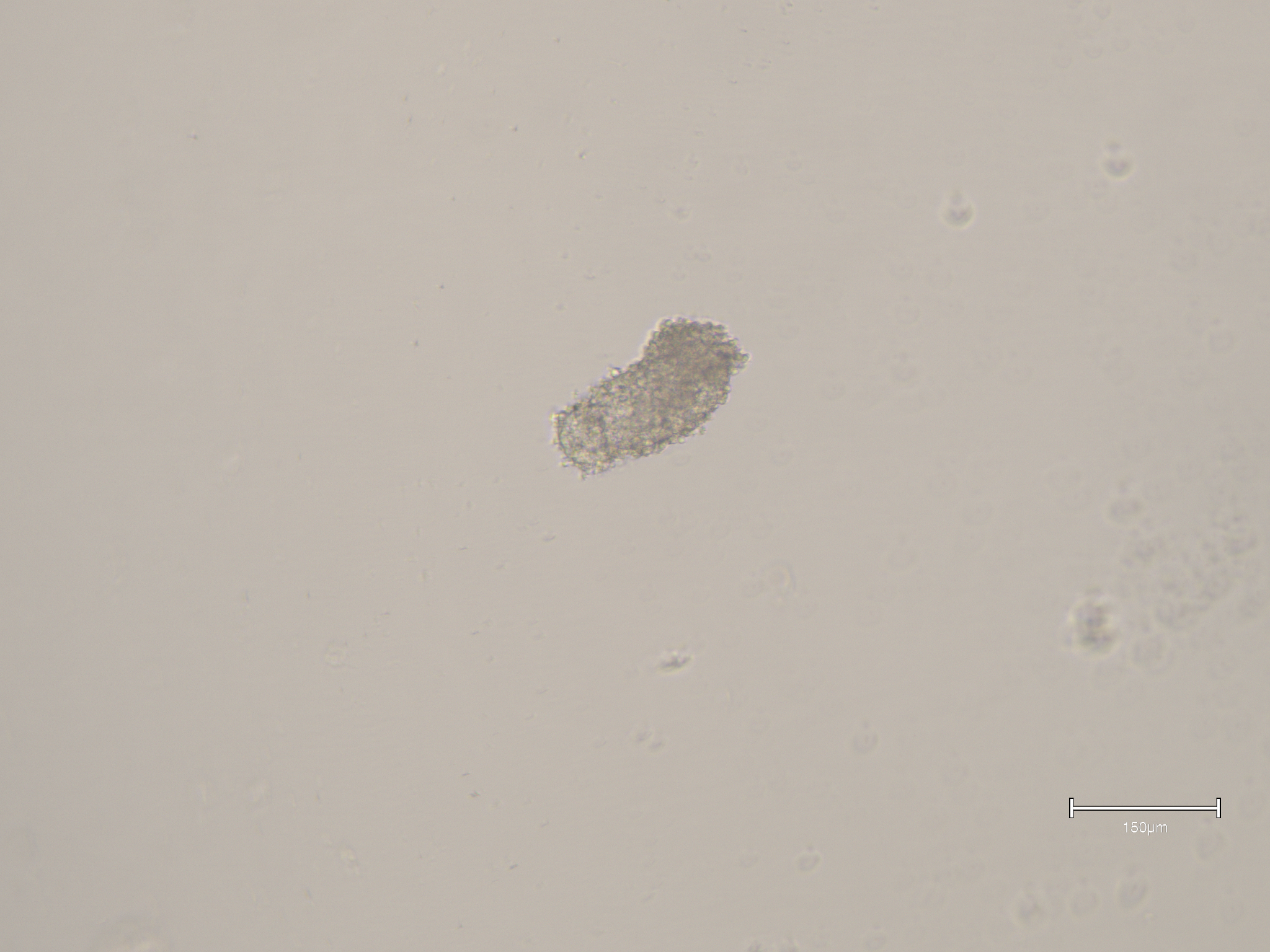

Supplement: Supplementary file 5 — Source data Fig. 3 [file 44318_2025_558_MOESM5_ESM.zip › Figure 3/panel 3B/NT_100cell.tiff]

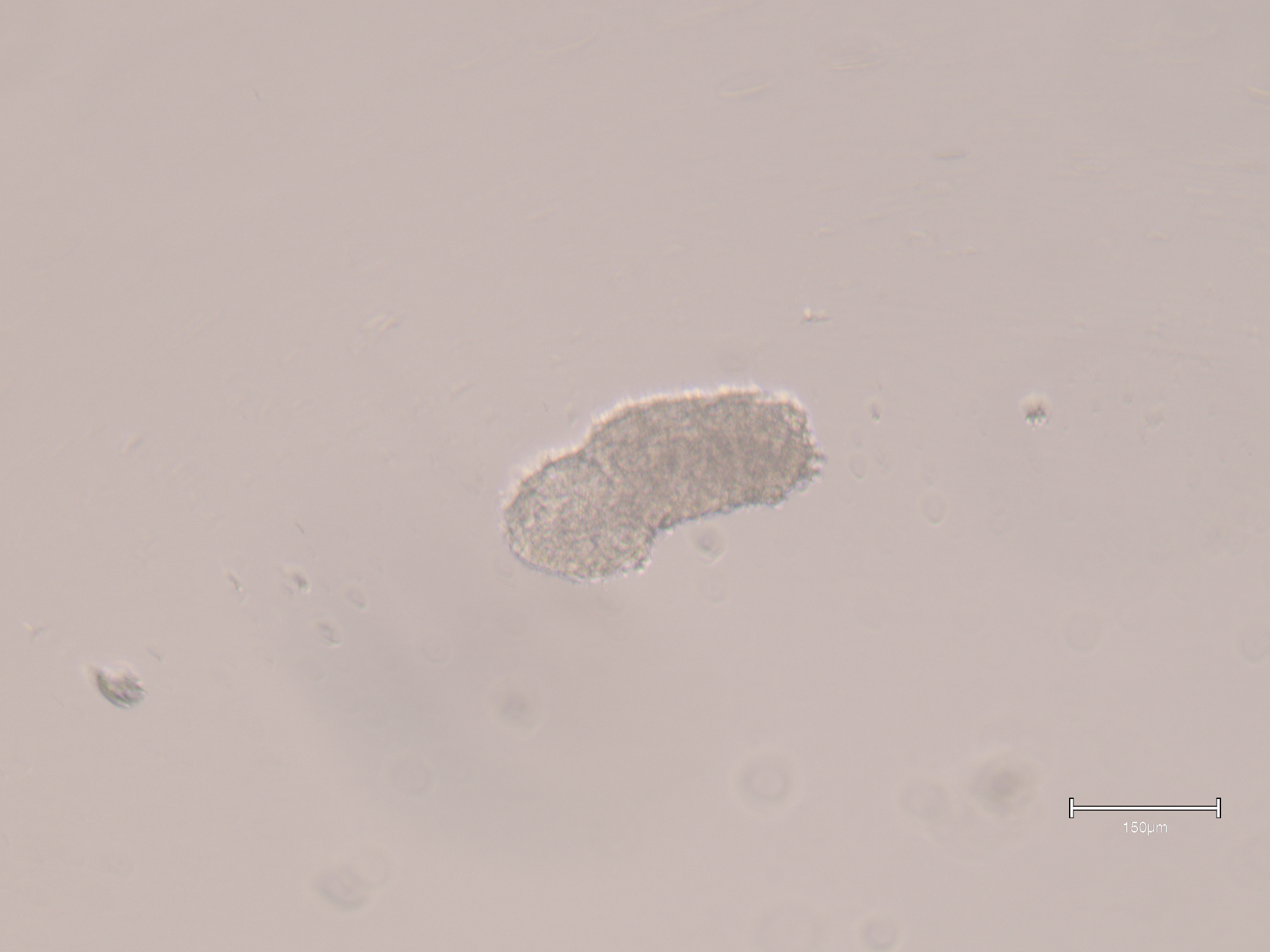

Supplement: Supplementary file 5 — Source data Fig. 3 [file 44318_2025_558_MOESM5_ESM.zip › Figure 3/panel 3B/NT_200cell.tiff]

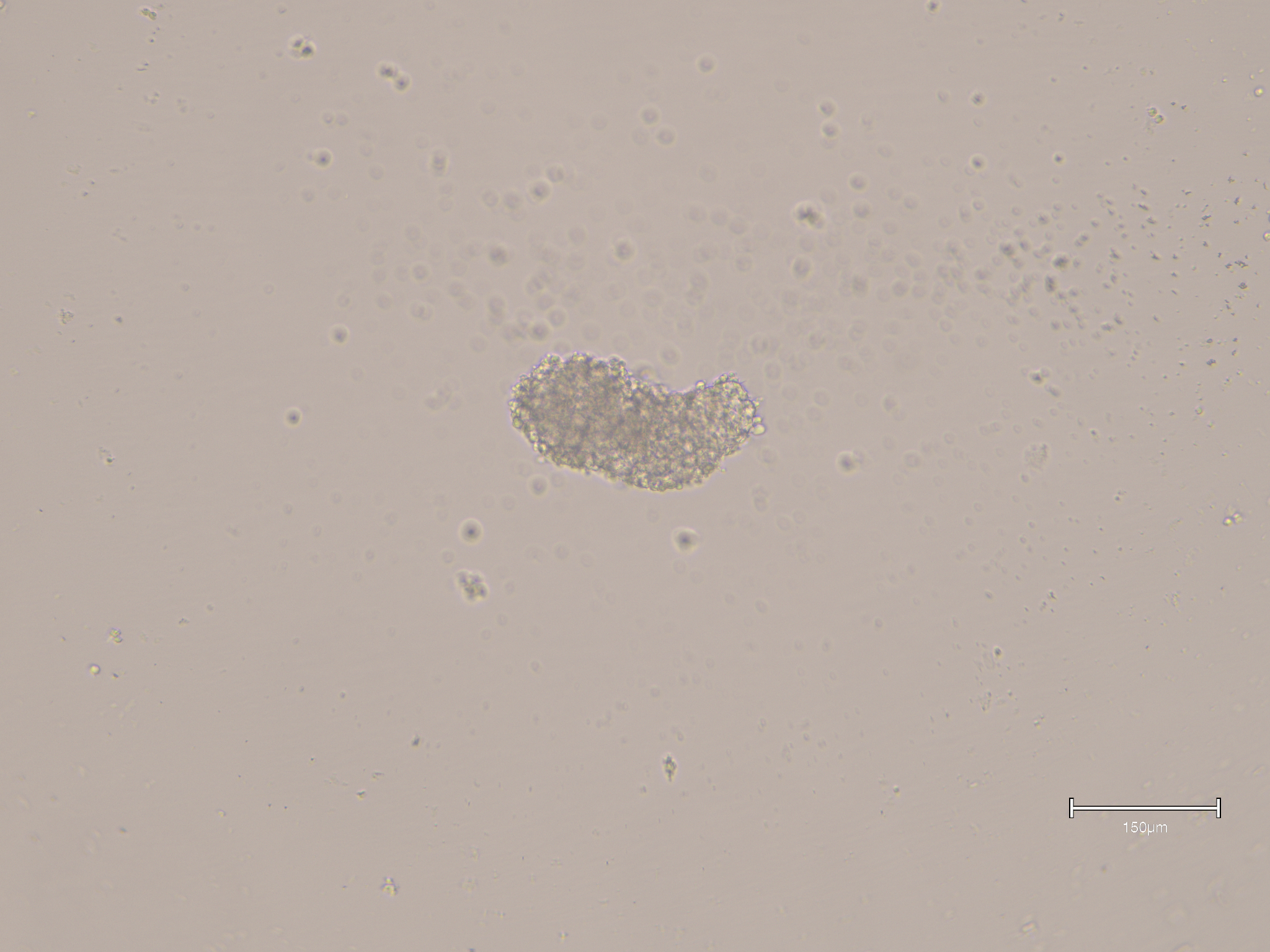

Supplement: Supplementary file 5 — Source data Fig. 3 [file 44318_2025_558_MOESM5_ESM.zip › Figure 3/panel 3B/NT_150cell.tiff]

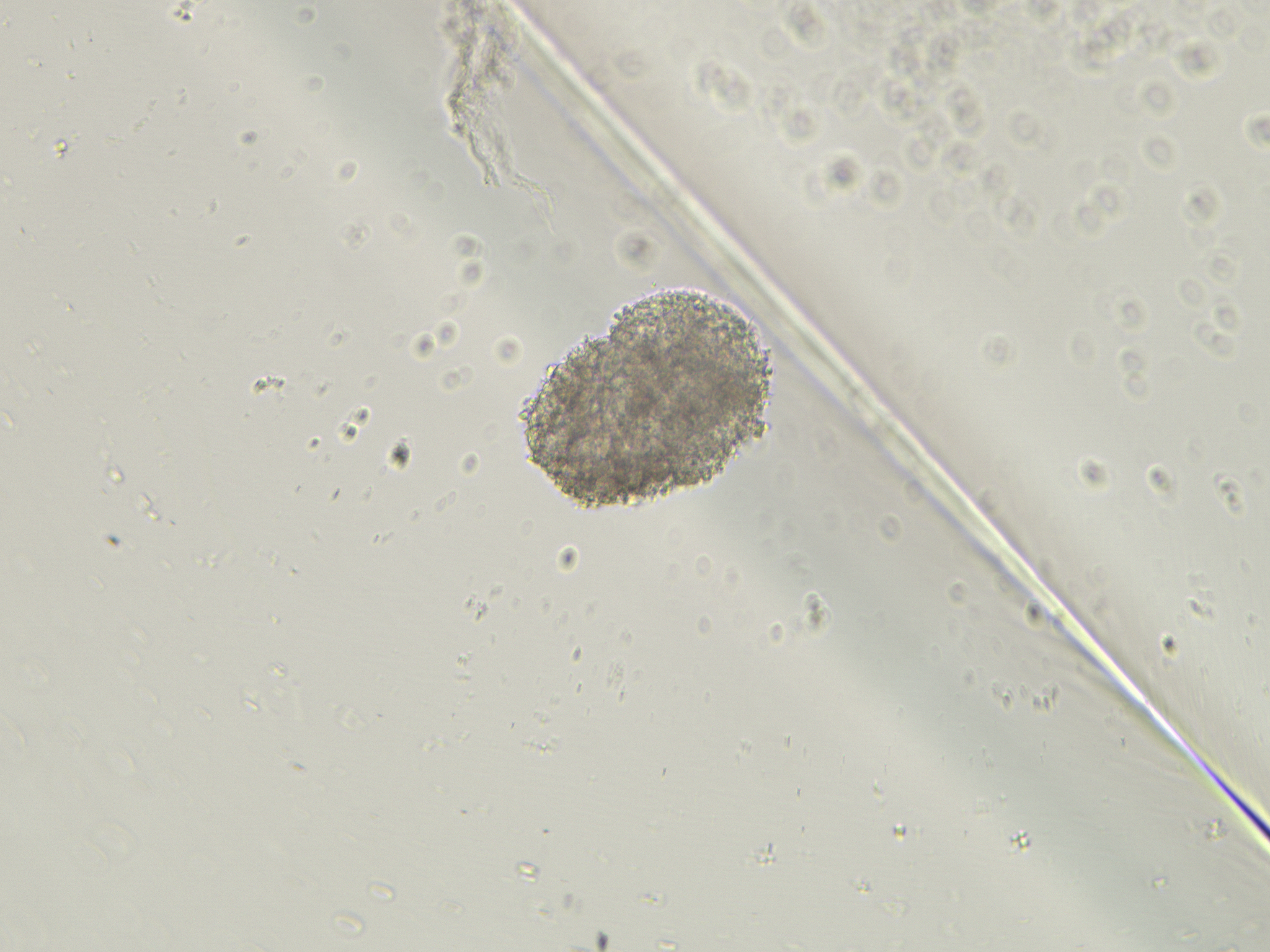

Supplement: Supplementary file 5 — Source data Fig. 3 [file 44318_2025_558_MOESM5_ESM.zip › Figure 3/panel 3B/KD-2_200cell.tiff]

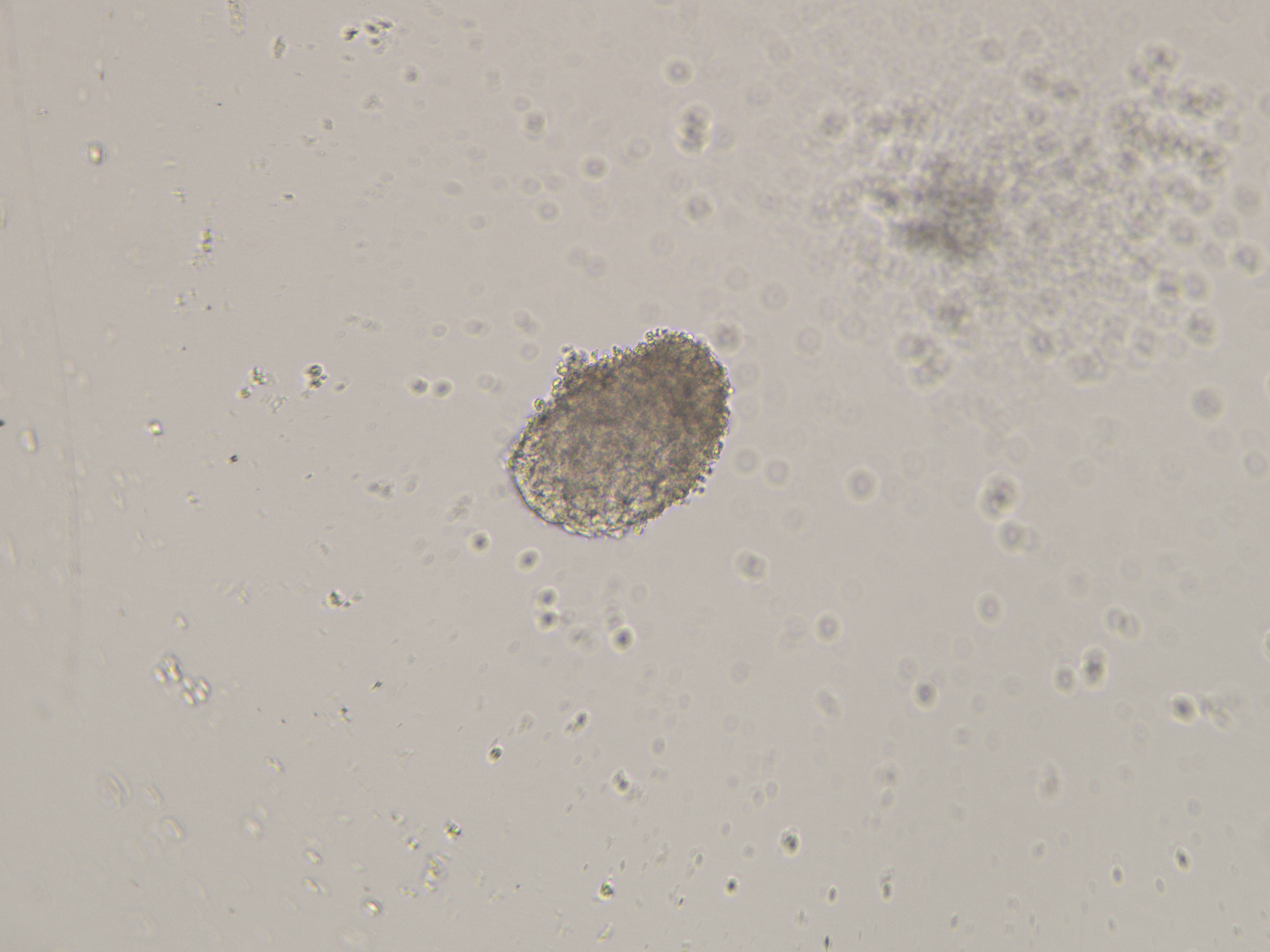

Supplement: Supplementary file 5 — Source data Fig. 3 [file 44318_2025_558_MOESM5_ESM.zip › Figure 3/panel 3B/KD-2_150cell.tiff]

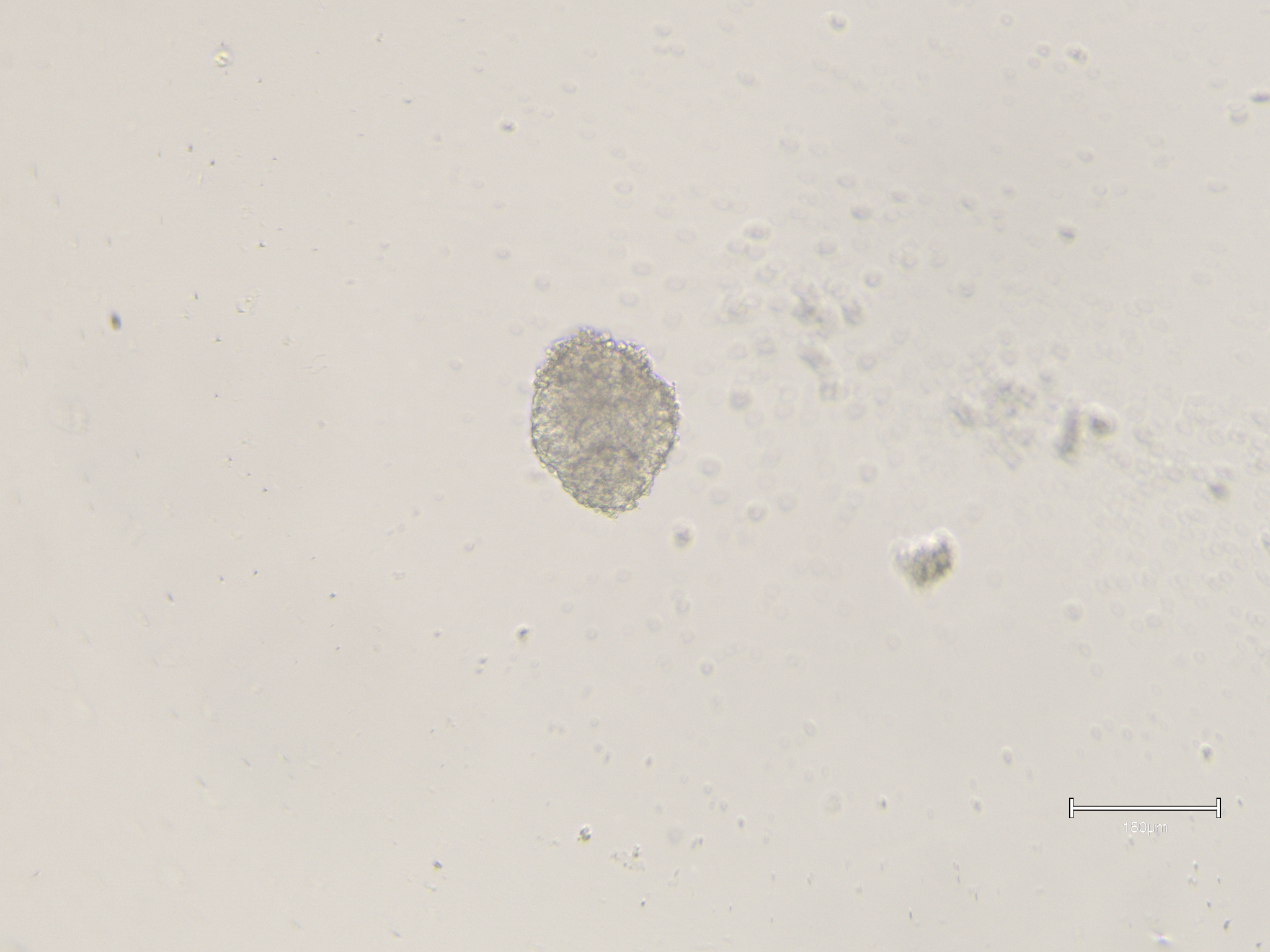

Supplement: Supplementary file 5 — Source data Fig. 3 [file 44318_2025_558_MOESM5_ESM.zip › Figure 3/panel 3B/KD-1_100cell.tiff]

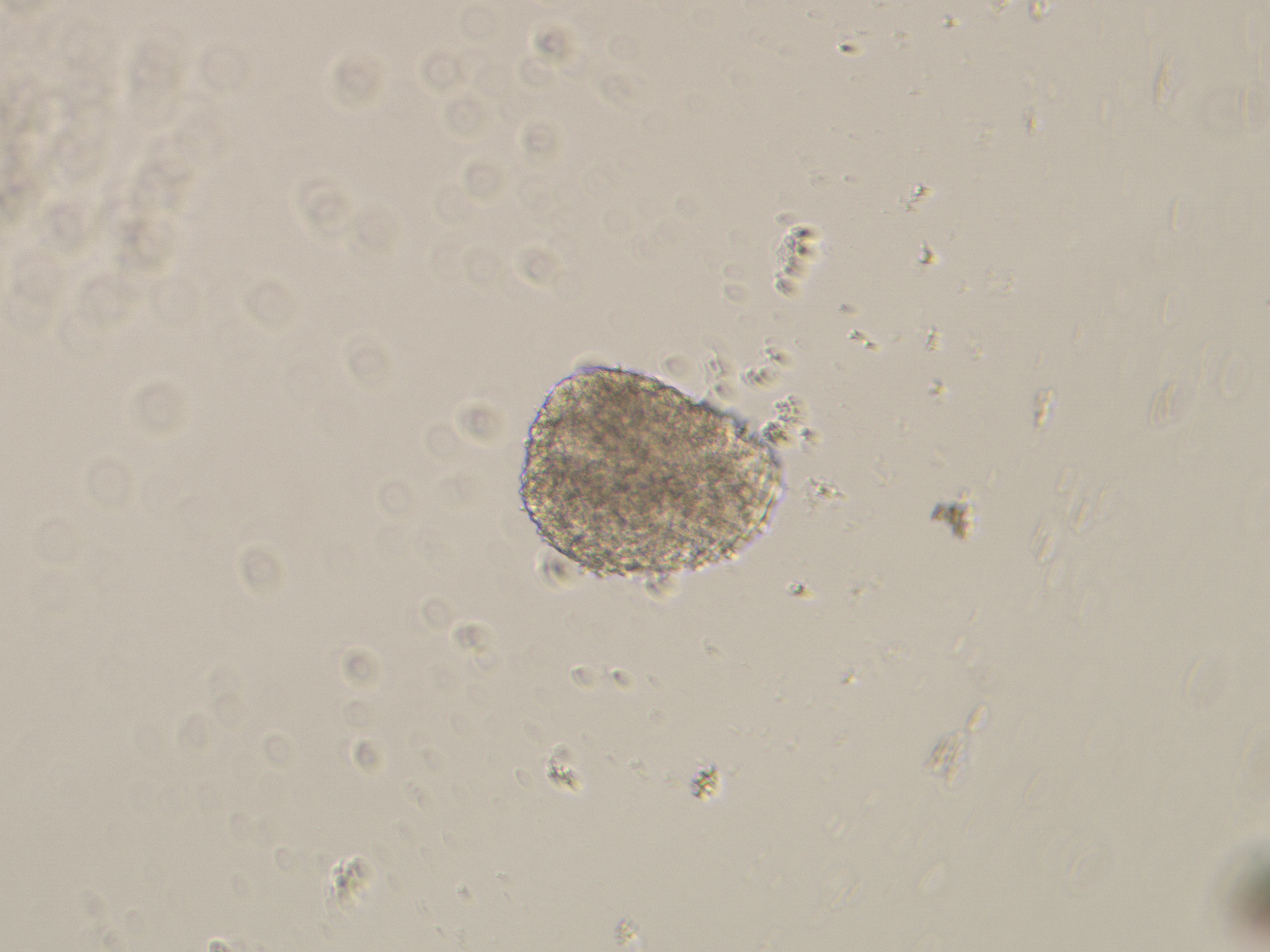

Supplement: Supplementary file 5 — Source data Fig. 3 [file 44318_2025_558_MOESM5_ESM.zip › Figure 3/panel 3B/KD-1_250cell.tiff]

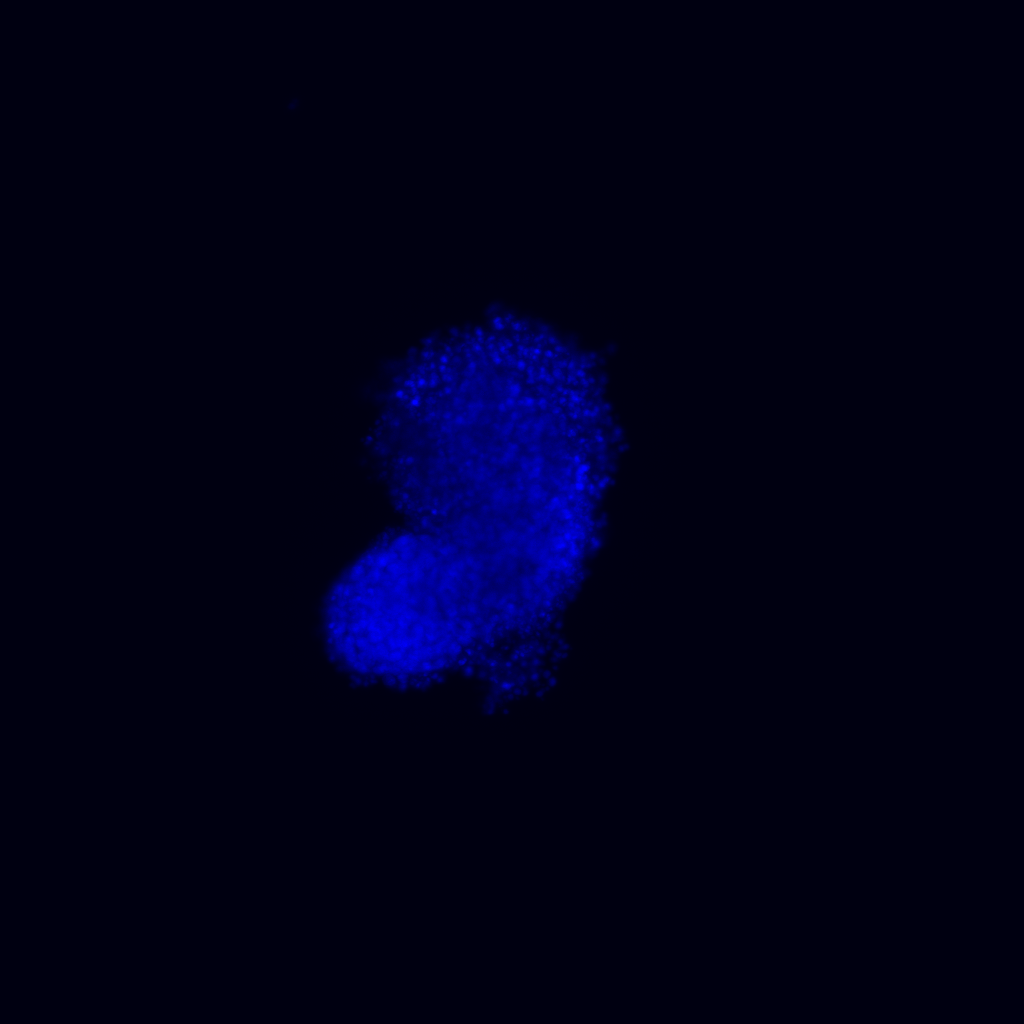

Supplement: Supplementary file 5 — Source data Fig. 3 [file 44318_2025_558_MOESM5_ESM.zip › Figure 3/panel 3C/NT_Cdx2/seq11117_seq11117_RGB_DAPI.tif]

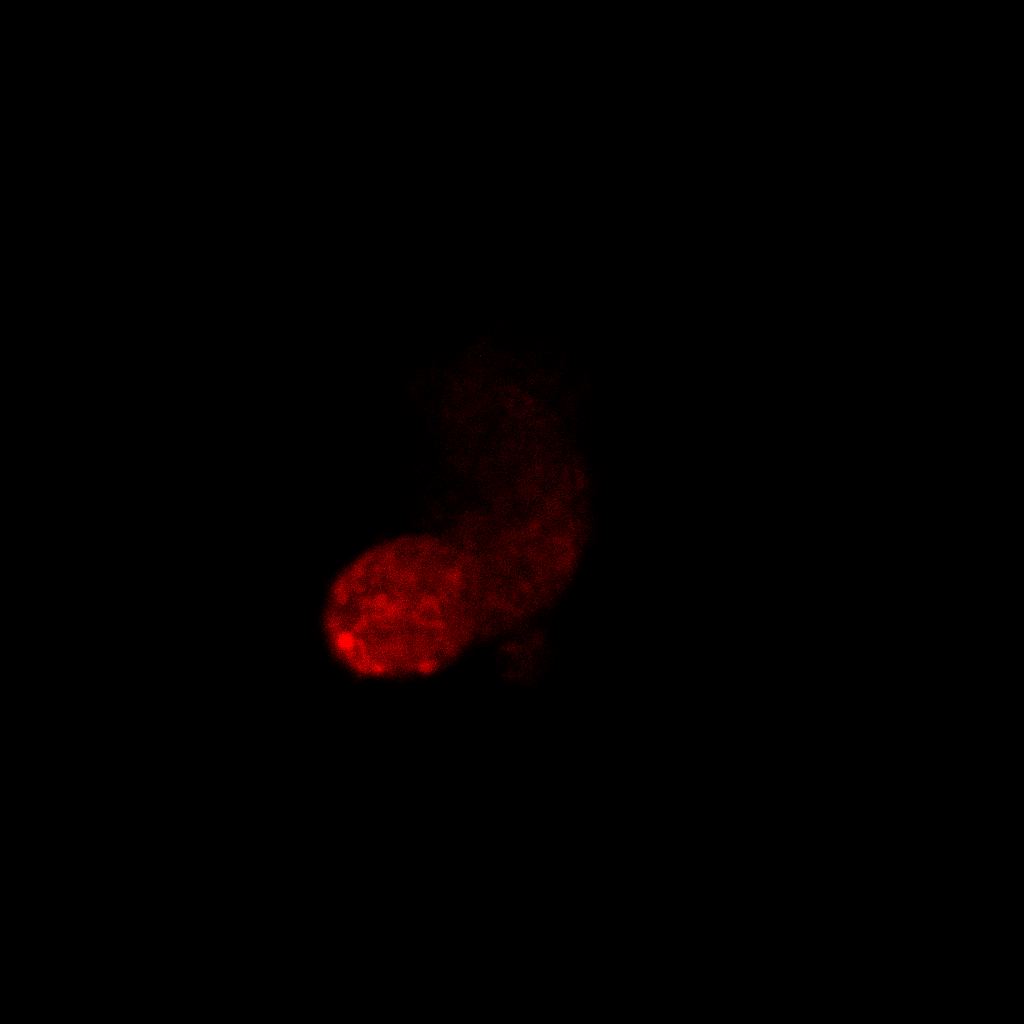

Supplement: Supplementary file 5 — Source data Fig. 3 [file 44318_2025_558_MOESM5_ESM.zip › Figure 3/panel 3C/NT_Cdx2/seq11117_seq11117_RGB_TRITC.tif]

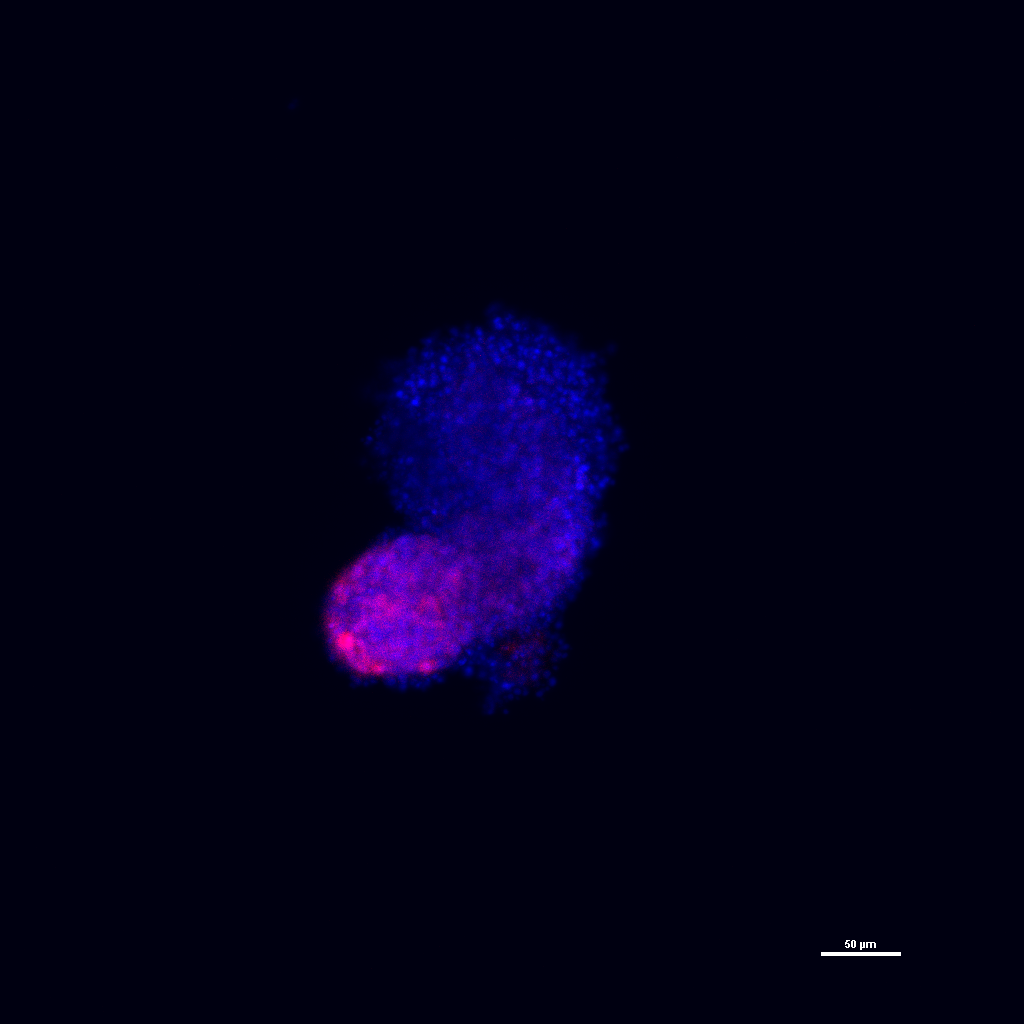

Supplement: Supplementary file 5 — Source data Fig. 3 [file 44318_2025_558_MOESM5_ESM.zip › Figure 3/panel 3C/NT_Cdx2/seq11117_seq11117_RGB.tif]

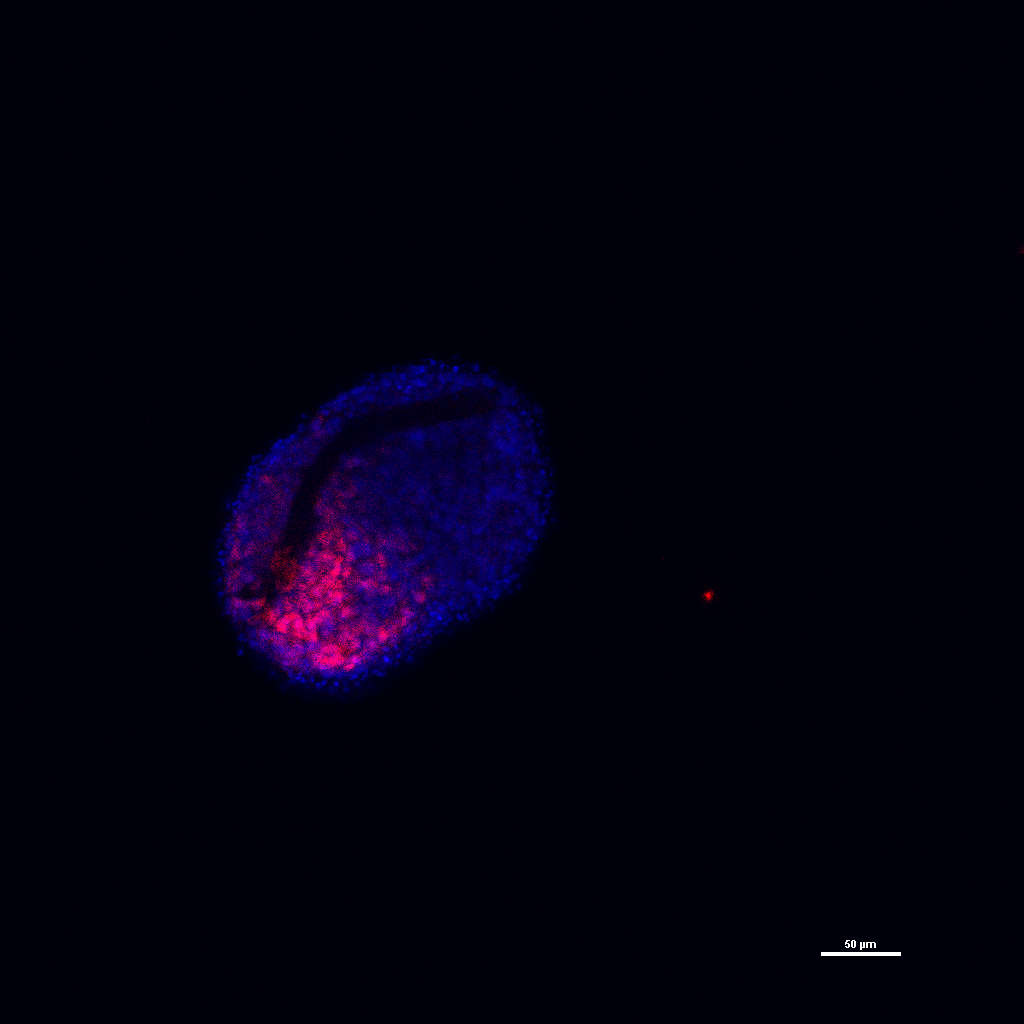

Supplement: Supplementary file 5 — Source data Fig. 3 [file 44318_2025_558_MOESM5_ESM.zip › Figure 3/panel 3C/KD-1_Bra/seq8961_seq8961_RGB.tif]

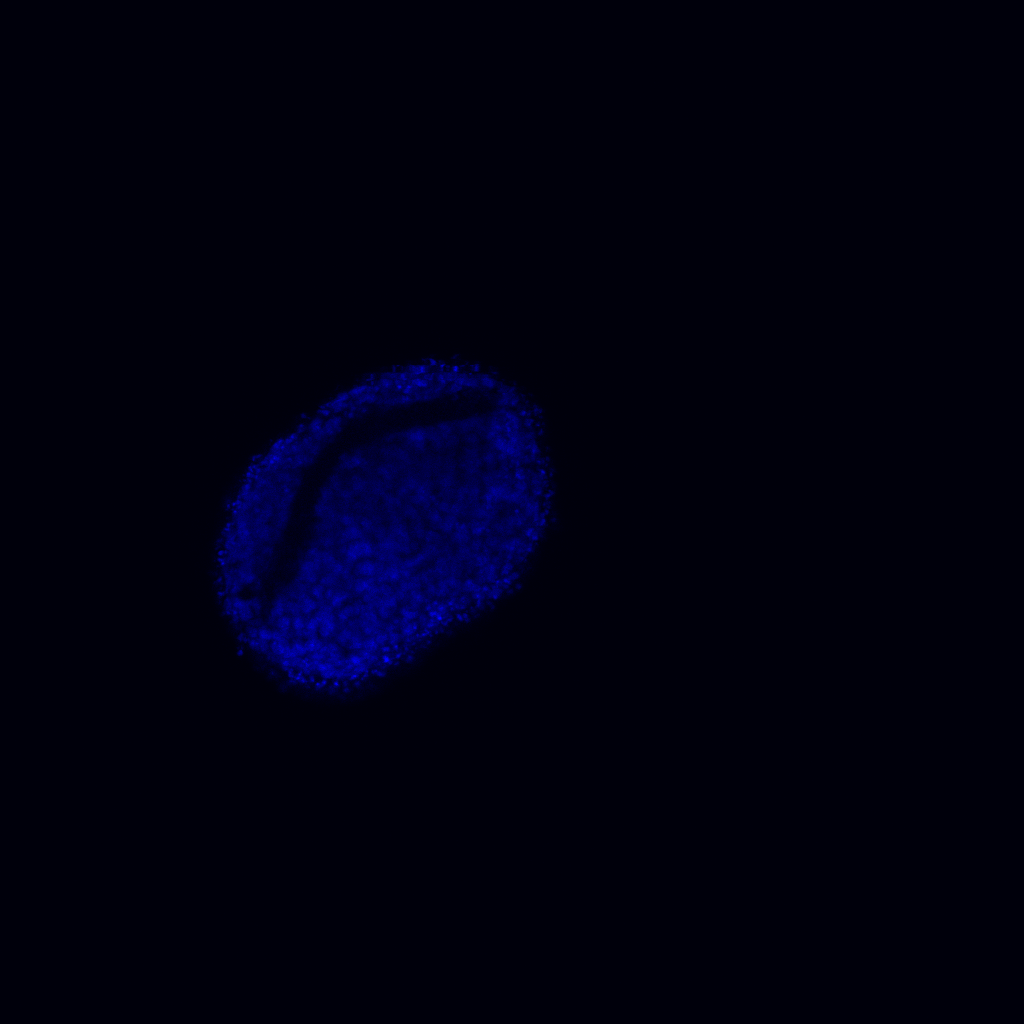

Supplement: Supplementary file 5 — Source data Fig. 3 [file 44318_2025_558_MOESM5_ESM.zip › Figure 3/panel 3C/KD-1_Bra/seq8961_seq8961_RGB_DAPI.tif]

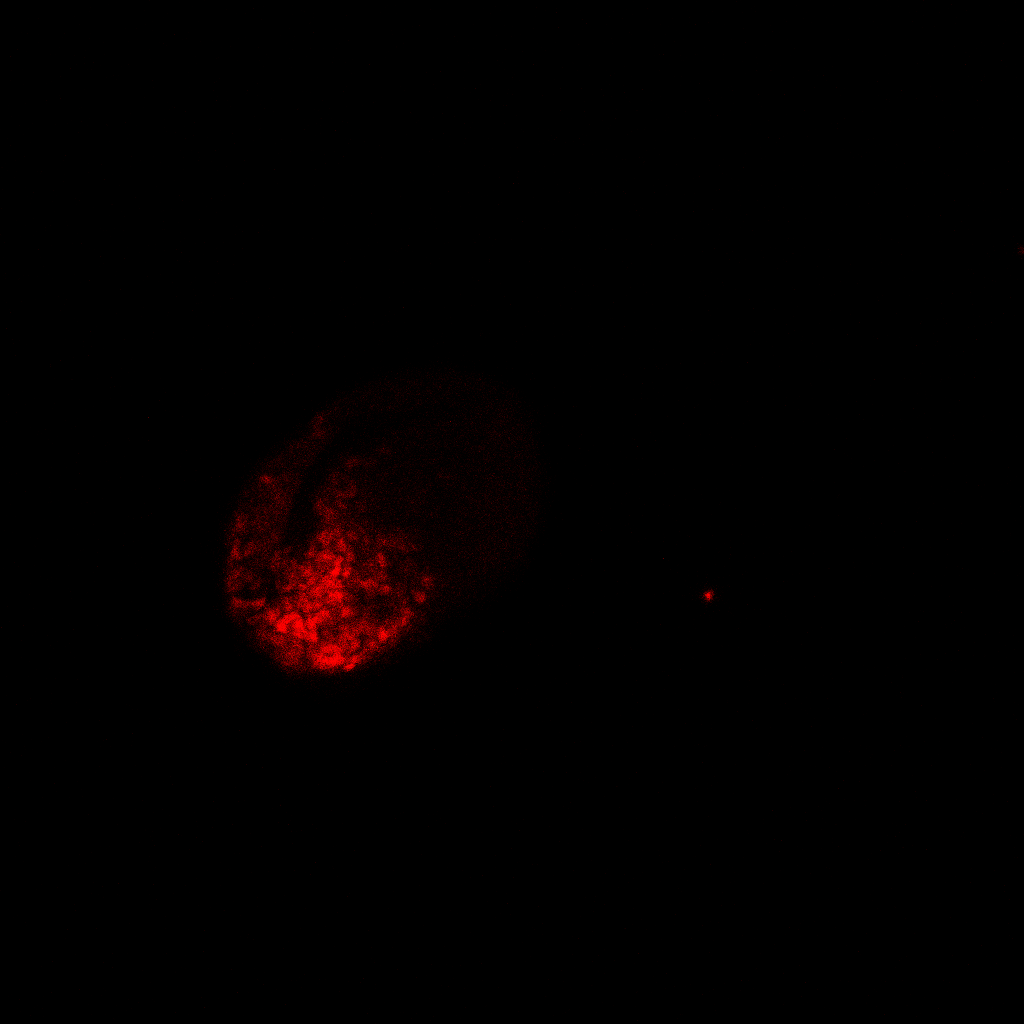

Supplement: Supplementary file 5 — Source data Fig. 3 [file 44318_2025_558_MOESM5_ESM.zip › Figure 3/panel 3C/KD-1_Bra/seq8961_seq8961_RGB_Texas Red.tif]

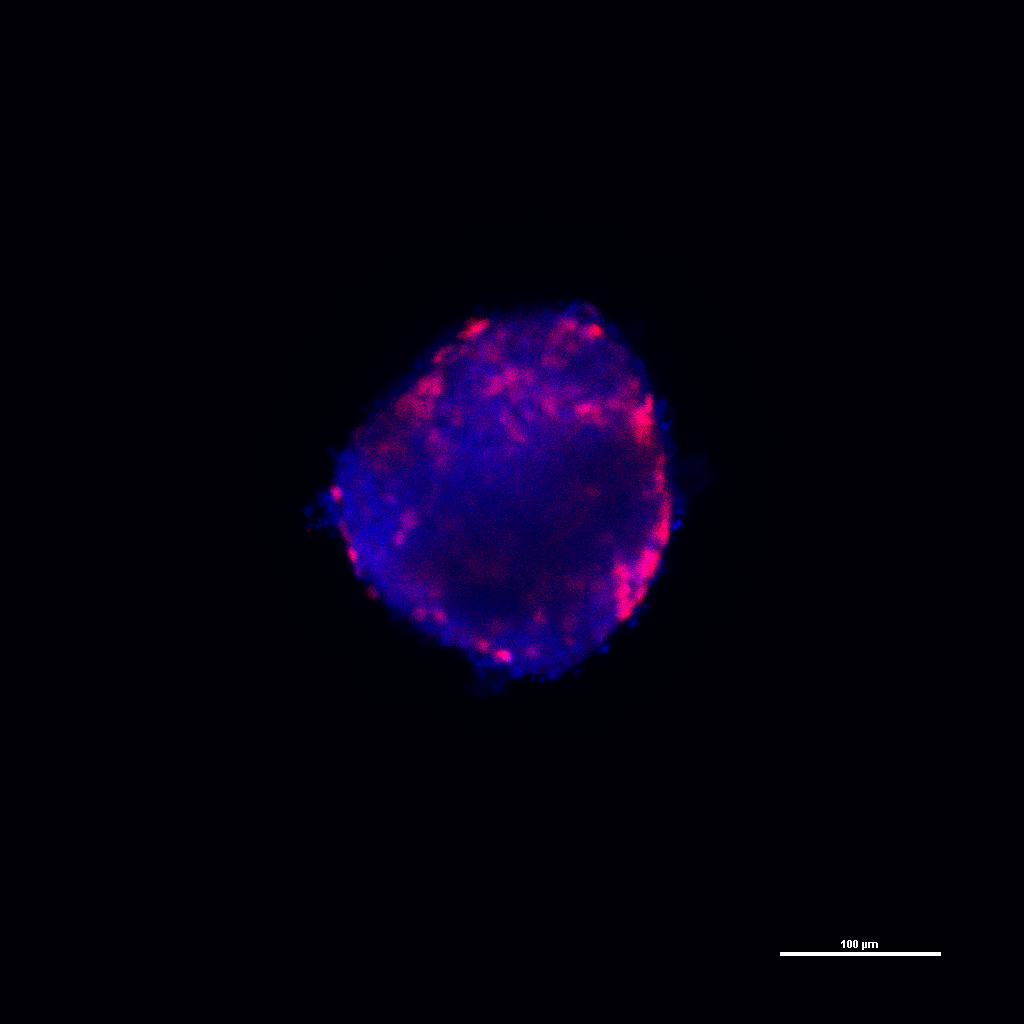

Supplement: Supplementary file 5 — Source data Fig. 3 [file 44318_2025_558_MOESM5_ESM.zip › Figure 3/panel 3C/KD-1_Sox17/seq11137_seq11137_RGB.tif]

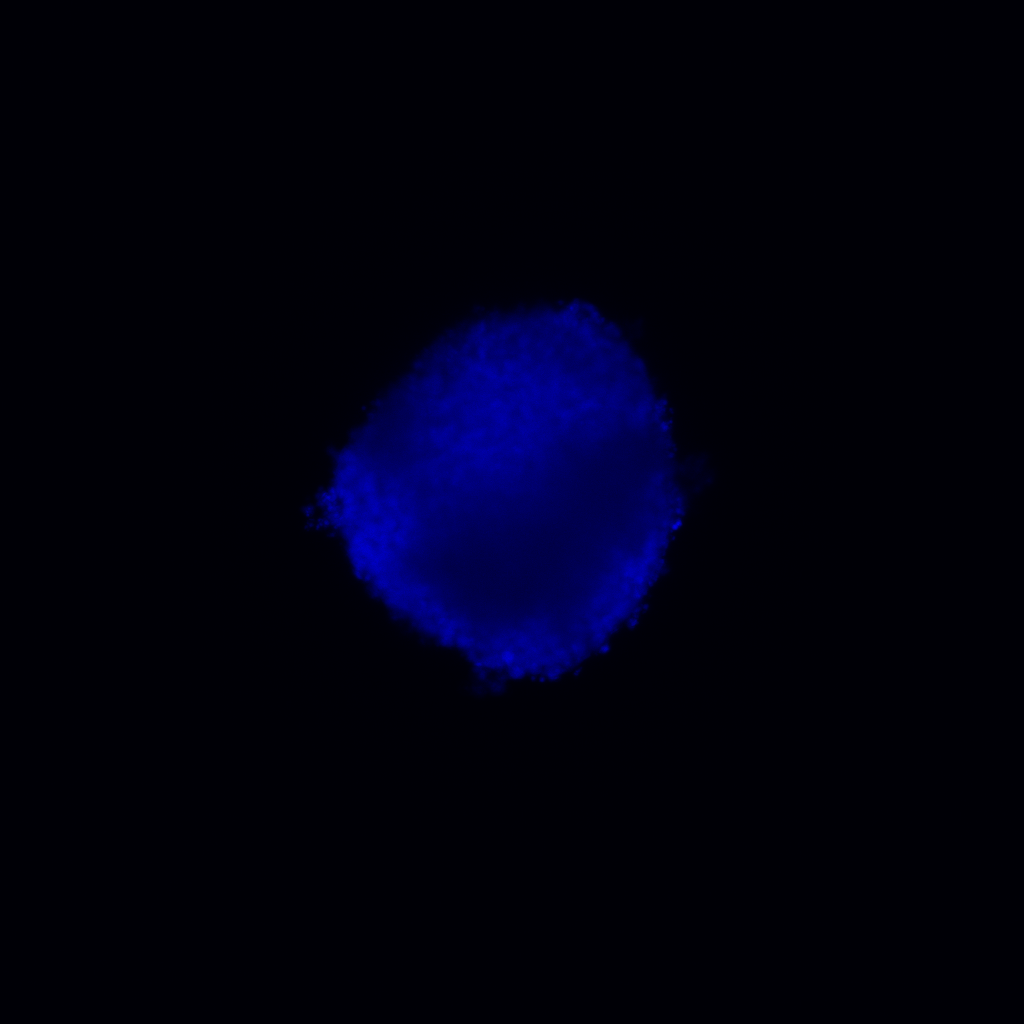

Supplement: Supplementary file 5 — Source data Fig. 3 [file 44318_2025_558_MOESM5_ESM.zip › Figure 3/panel 3C/KD-1_Sox17/seq11137_seq11137_RGB_DAPI.tif]

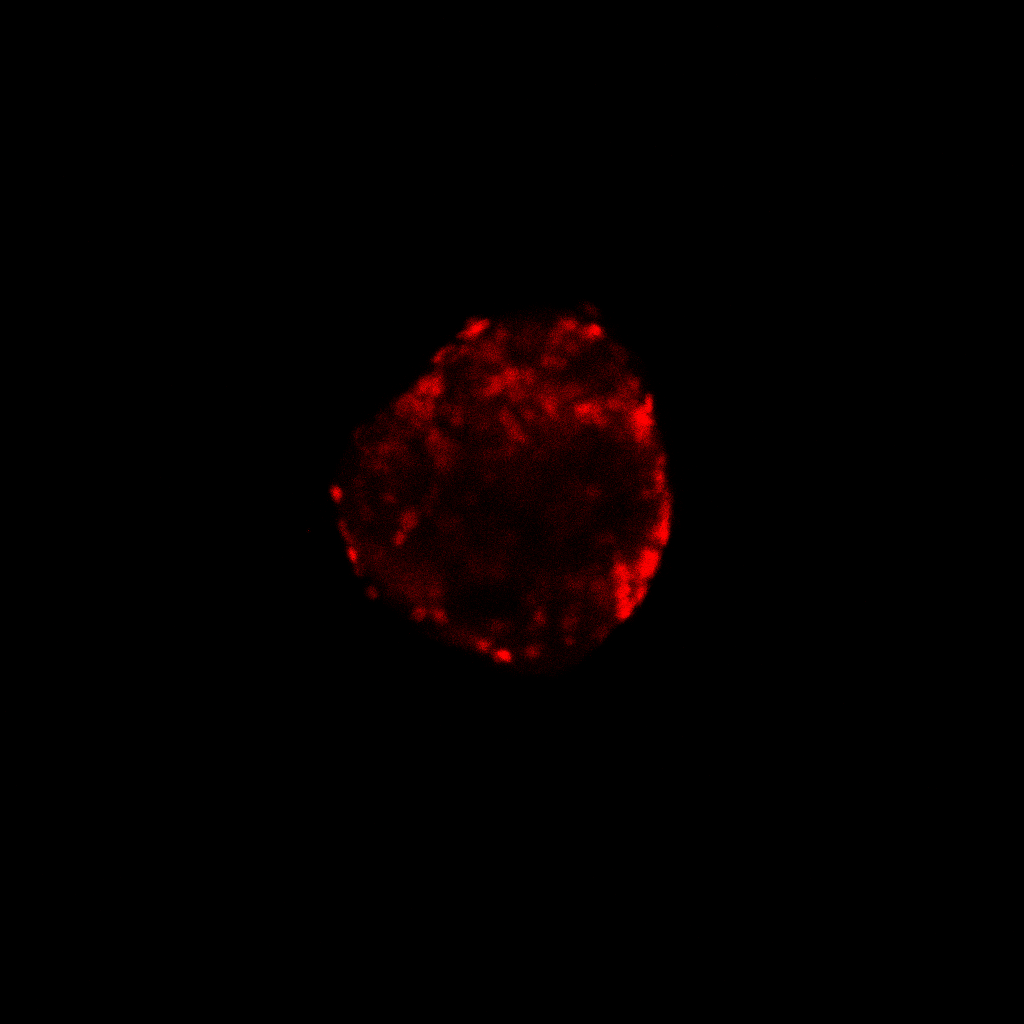

Supplement: Supplementary file 5 — Source data Fig. 3 [file 44318_2025_558_MOESM5_ESM.zip › Figure 3/panel 3C/KD-1_Sox17/seq11137_seq11137_RGB_TRITC.tif]

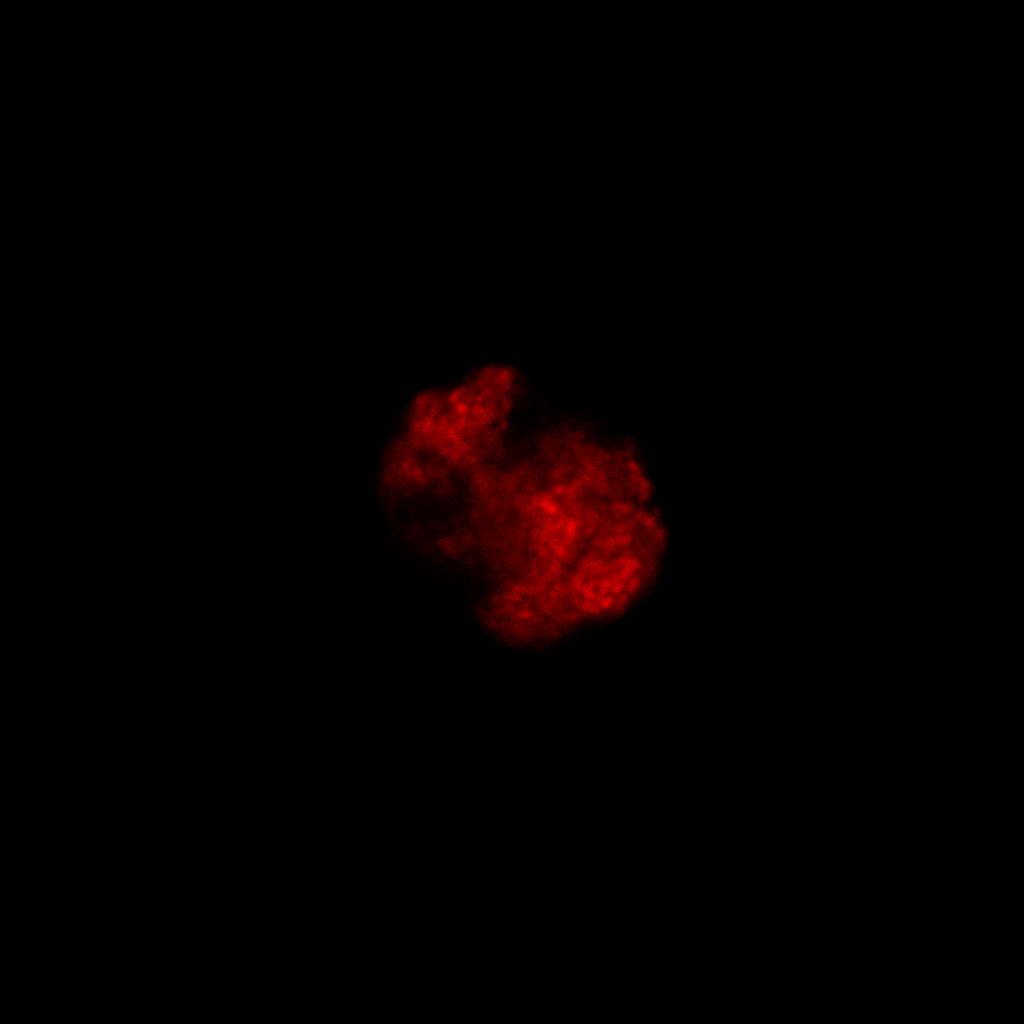

Supplement: Supplementary file 5 — Source data Fig. 3 [file 44318_2025_558_MOESM5_ESM.zip › Figure 3/panel 3C/KD-1_Nanog/seq11132_seq11132_RGB_TRITC.tif]

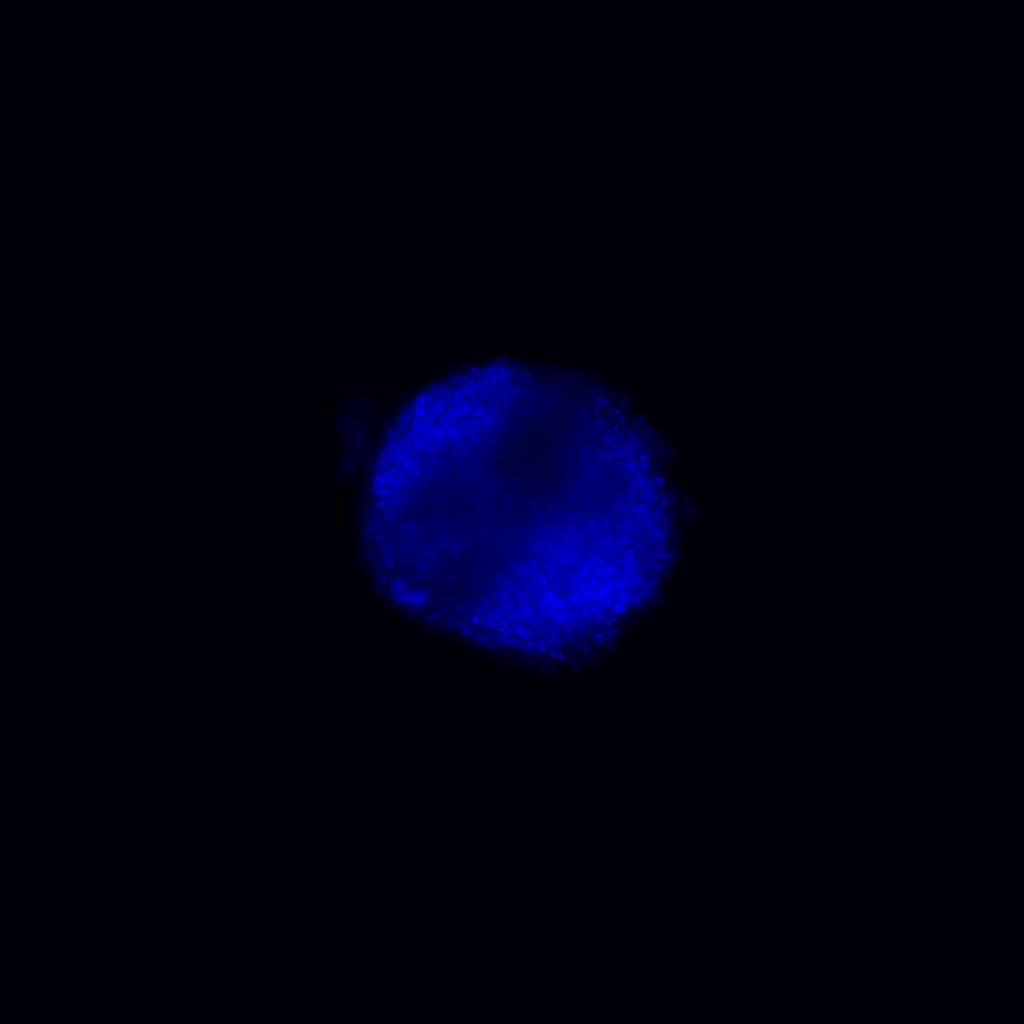

Supplement: Supplementary file 5 — Source data Fig. 3 [file 44318_2025_558_MOESM5_ESM.zip › Figure 3/panel 3C/KD-1_Nanog/seq11132_seq11132_RGB_DAPI.tif]

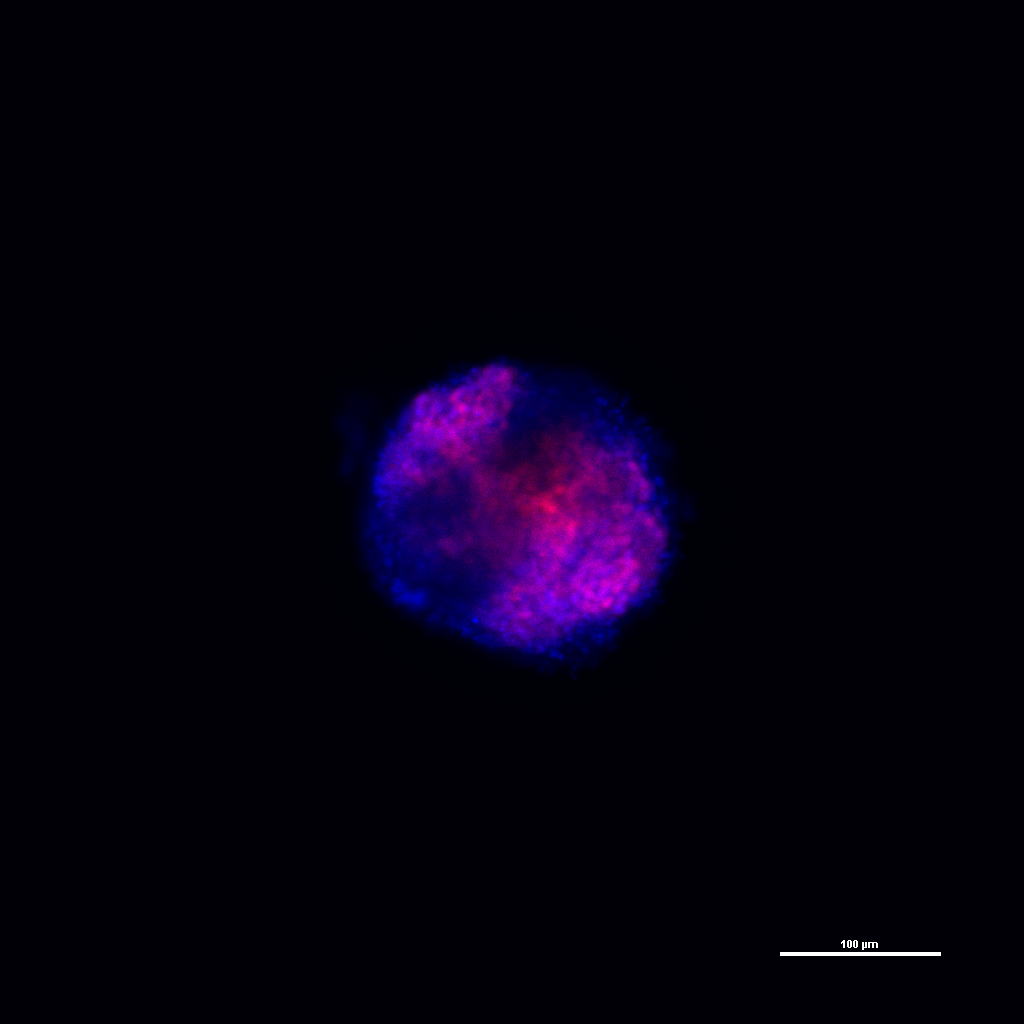

Supplement: Supplementary file 5 — Source data Fig. 3 [file 44318_2025_558_MOESM5_ESM.zip › Figure 3/panel 3C/KD-1_Nanog/seq11132_seq11132_RGB.tif]

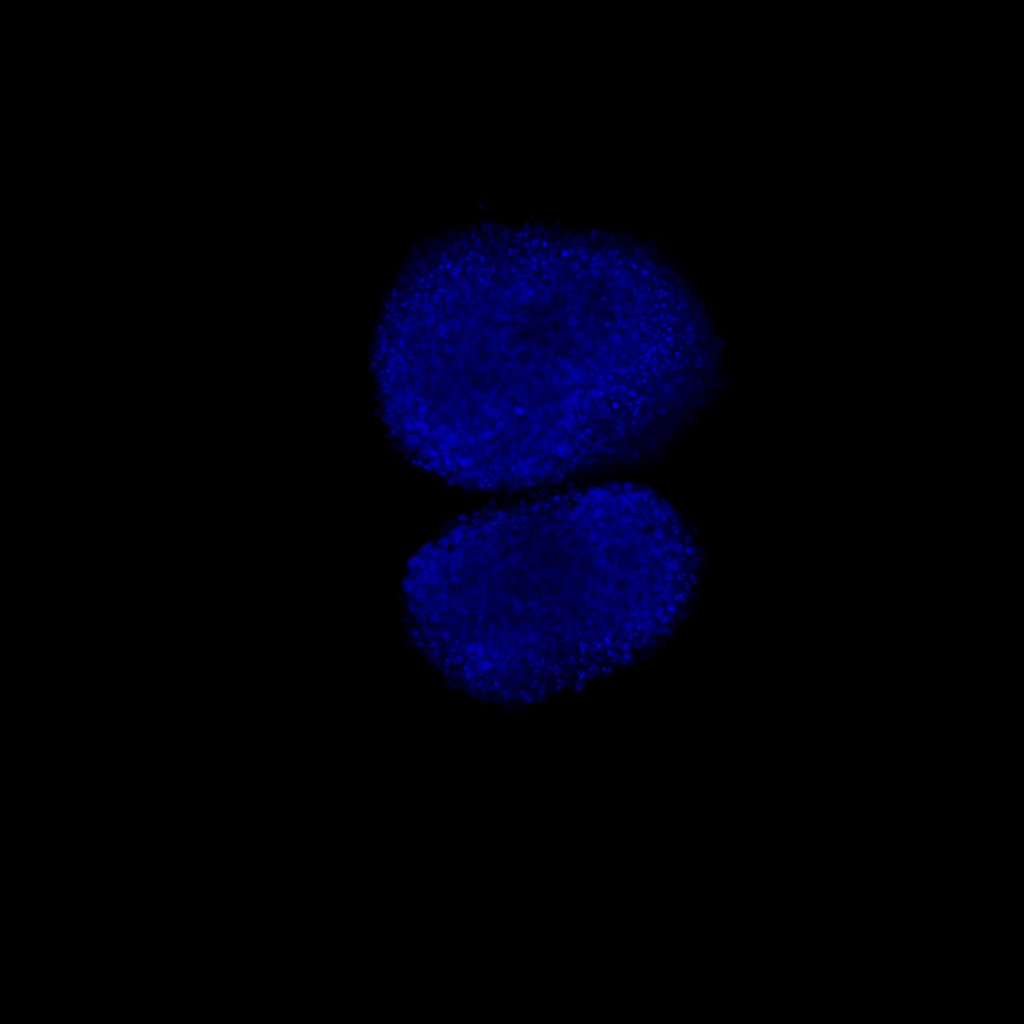

Supplement: Supplementary file 5 — Source data Fig. 3 [file 44318_2025_558_MOESM5_ESM.zip › Figure 3/panel 3C/KD-2_Bra/image0146_image0146_RGB_DAPI.tif]

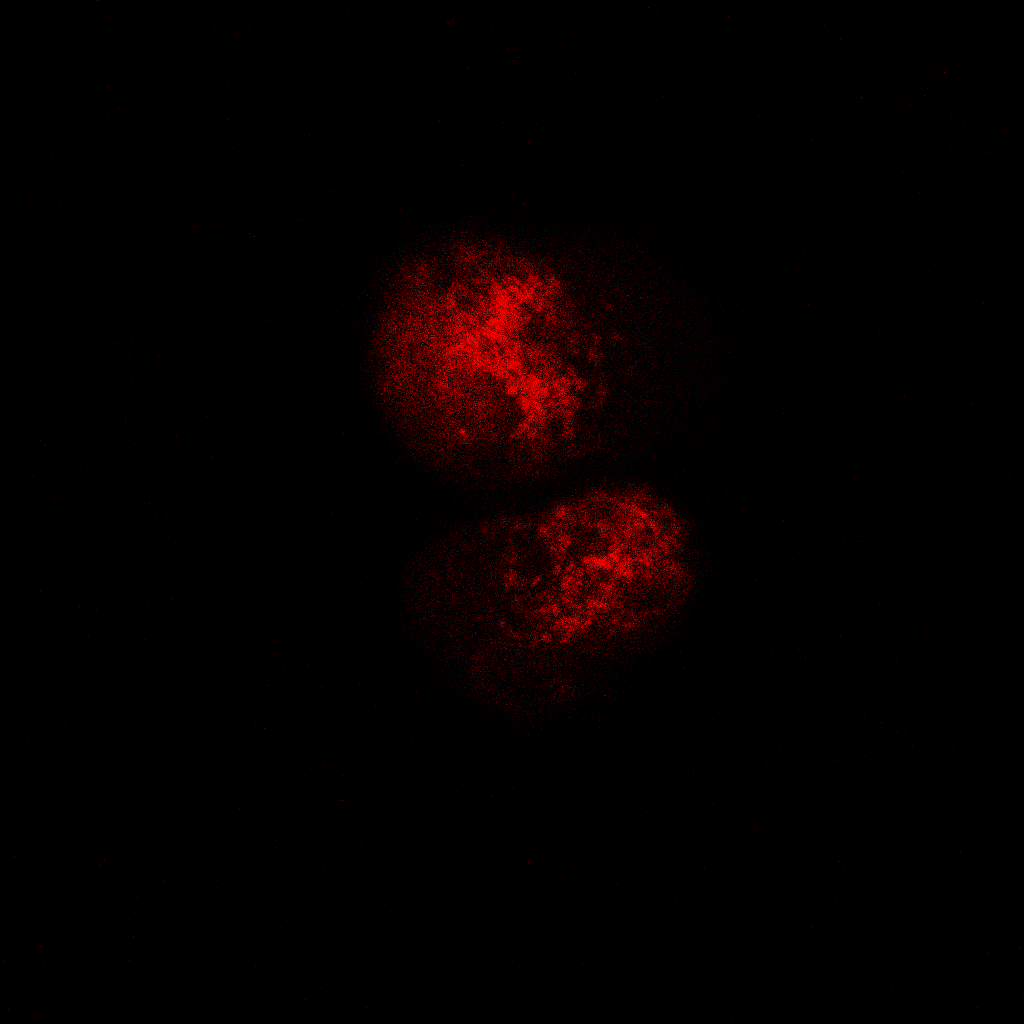

Supplement: Supplementary file 5 — Source data Fig. 3 [file 44318_2025_558_MOESM5_ESM.zip › Figure 3/panel 3C/KD-2_Bra/image0146_image0146_RGB_Texas Red.tif]

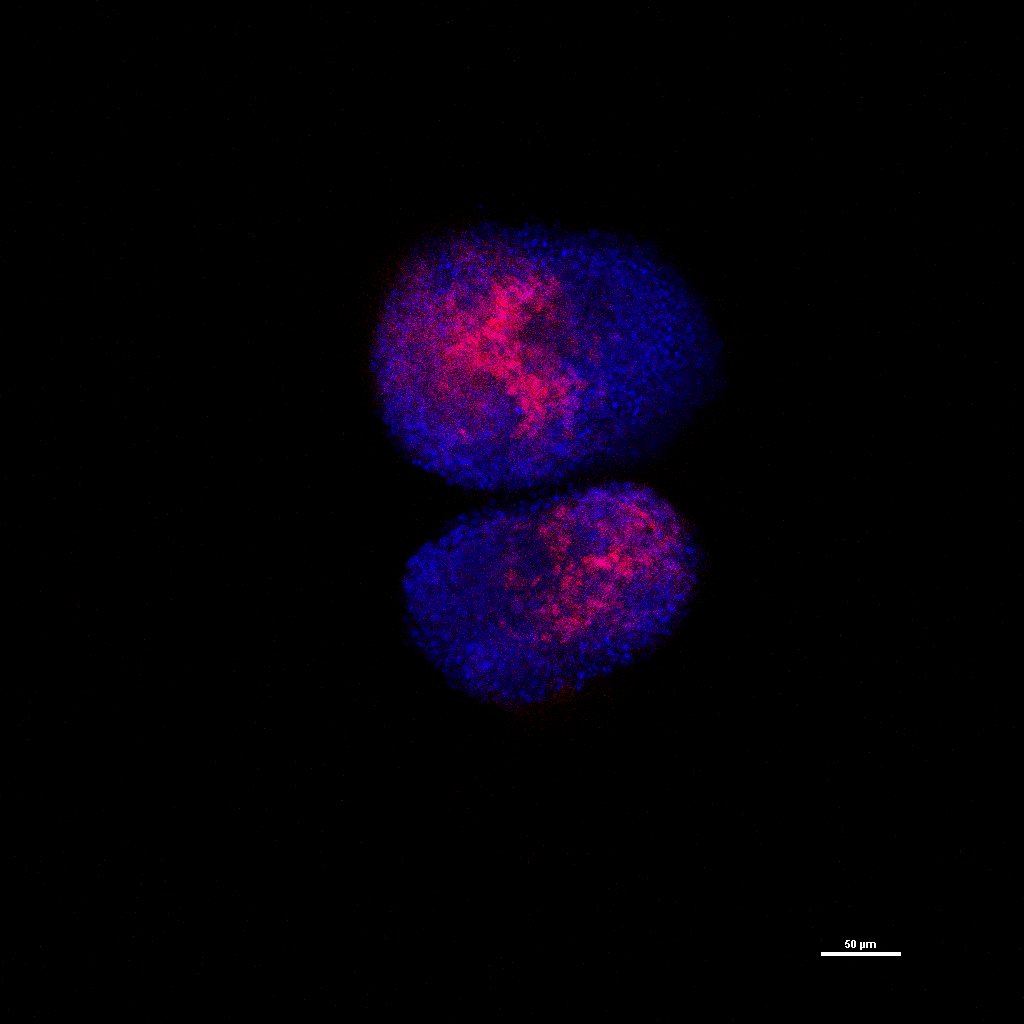

Supplement: Supplementary file 5 — Source data Fig. 3 [file 44318_2025_558_MOESM5_ESM.zip › Figure 3/panel 3C/KD-2_Bra/image0146_image0146_RGB.tif]

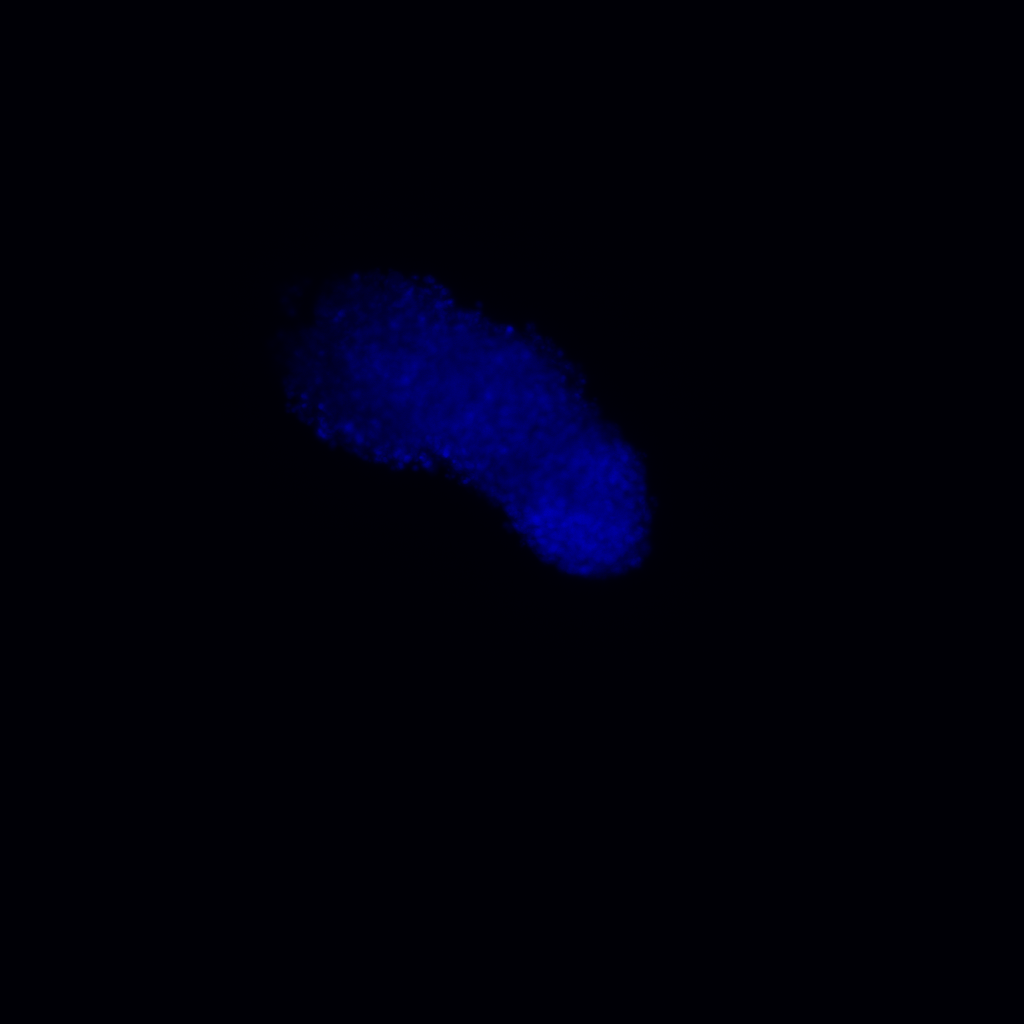

Supplement: Supplementary file 5 — Source data Fig. 3 [file 44318_2025_558_MOESM5_ESM.zip › Figure 3/panel 3C/NT_Nanog/seq11128_seq11128_RGB_DAPI.tif]

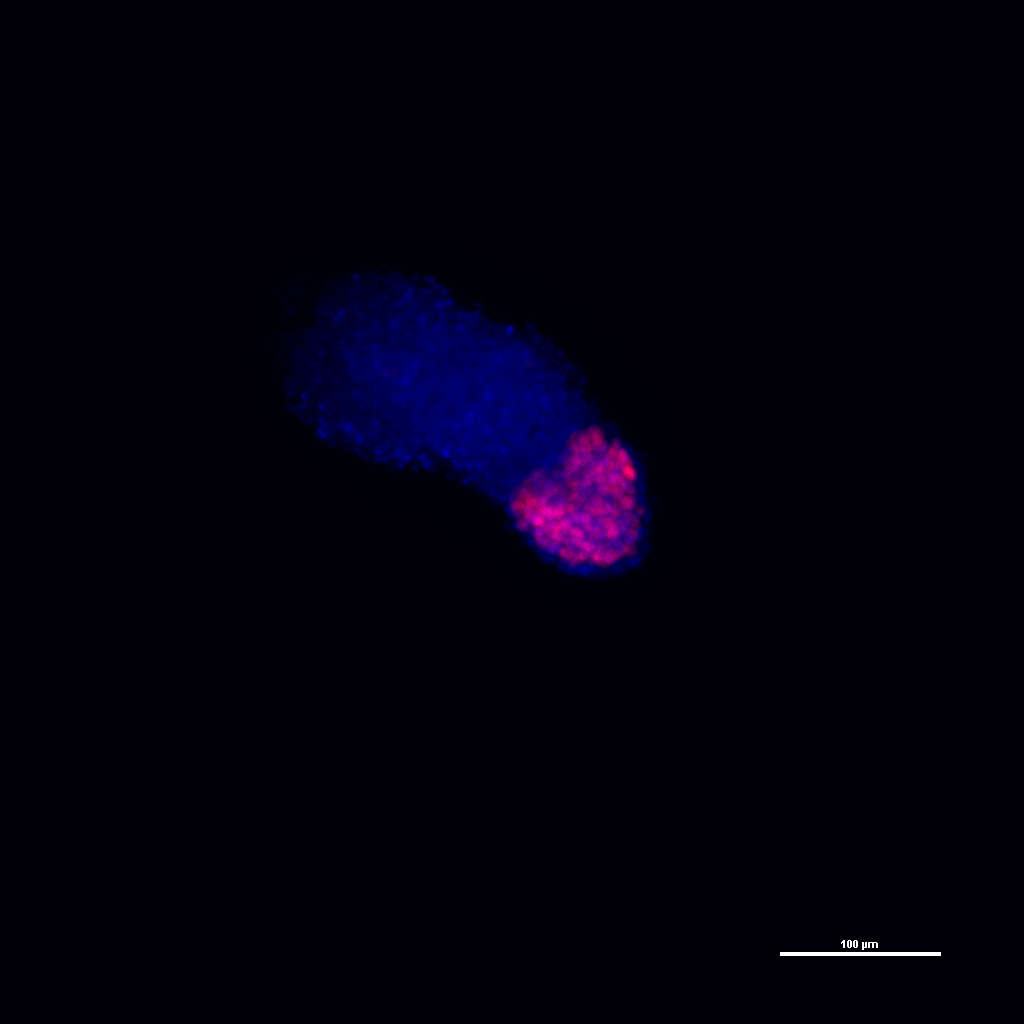

Supplement: Supplementary file 5 — Source data Fig. 3 [file 44318_2025_558_MOESM5_ESM.zip › Figure 3/panel 3C/NT_Nanog/seq11128_seq11128_RGB.tif]

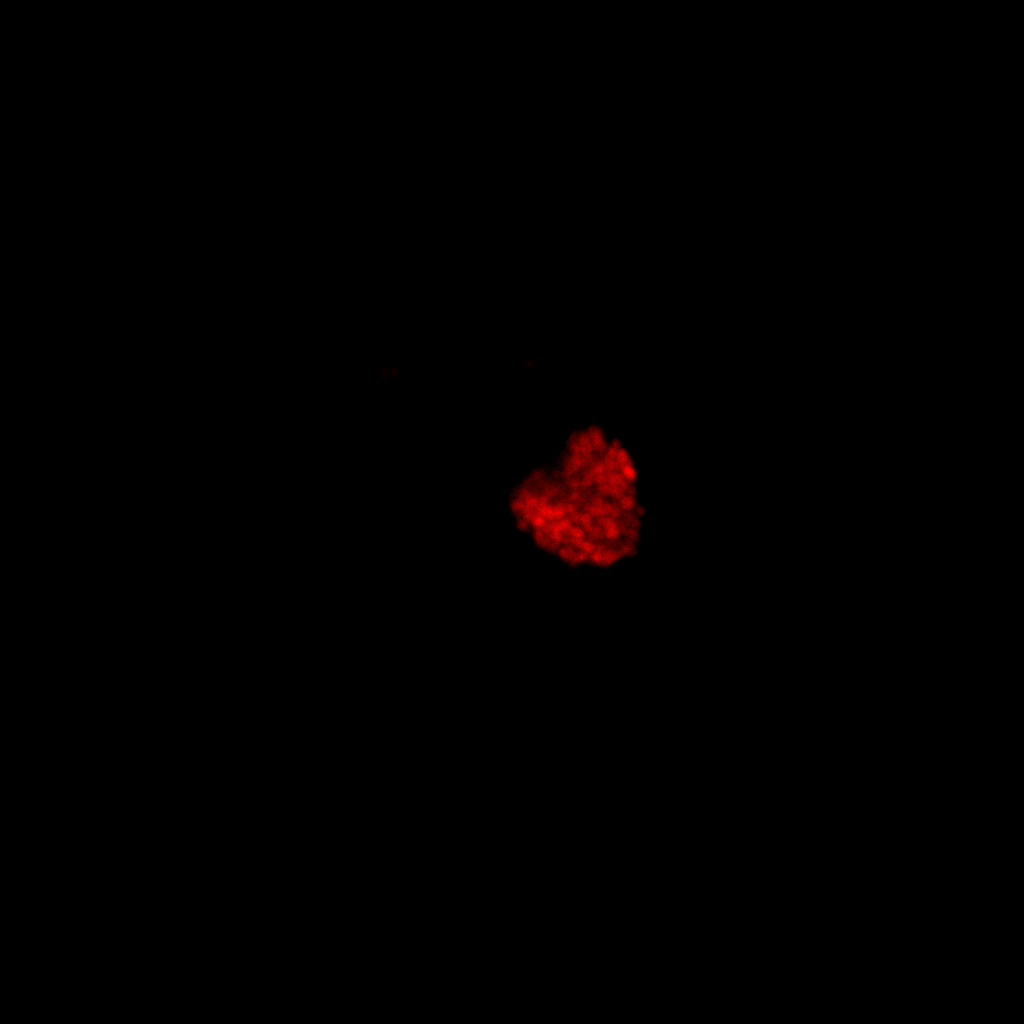

Supplement: Supplementary file 5 — Source data Fig. 3 [file 44318_2025_558_MOESM5_ESM.zip › Figure 3/panel 3C/NT_Nanog/seq11128_seq11128_RGB_TRITC.tif]

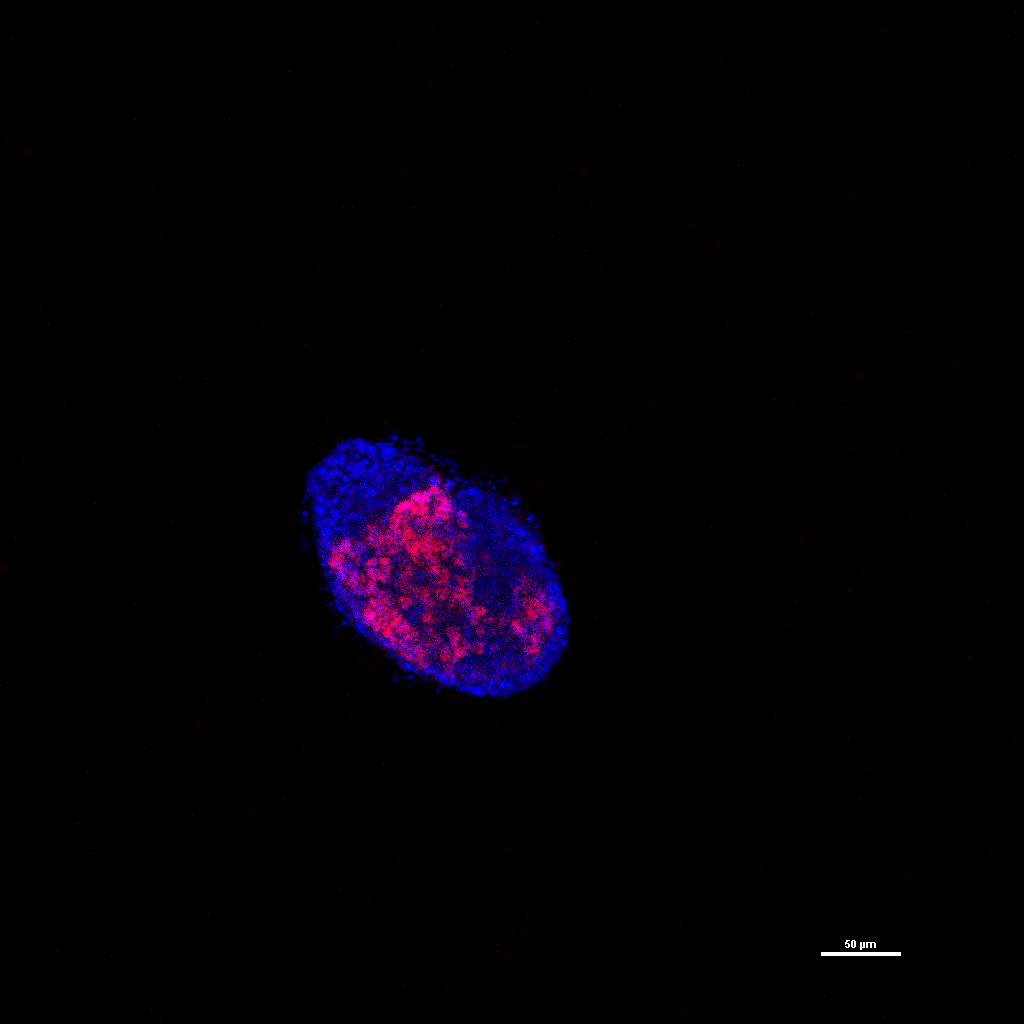

Supplement: Supplementary file 5 — Source data Fig. 3 [file 44318_2025_558_MOESM5_ESM.zip › Figure 3/panel 3C/KD-2_Sox2/image0139_image0139_RGB.tif]

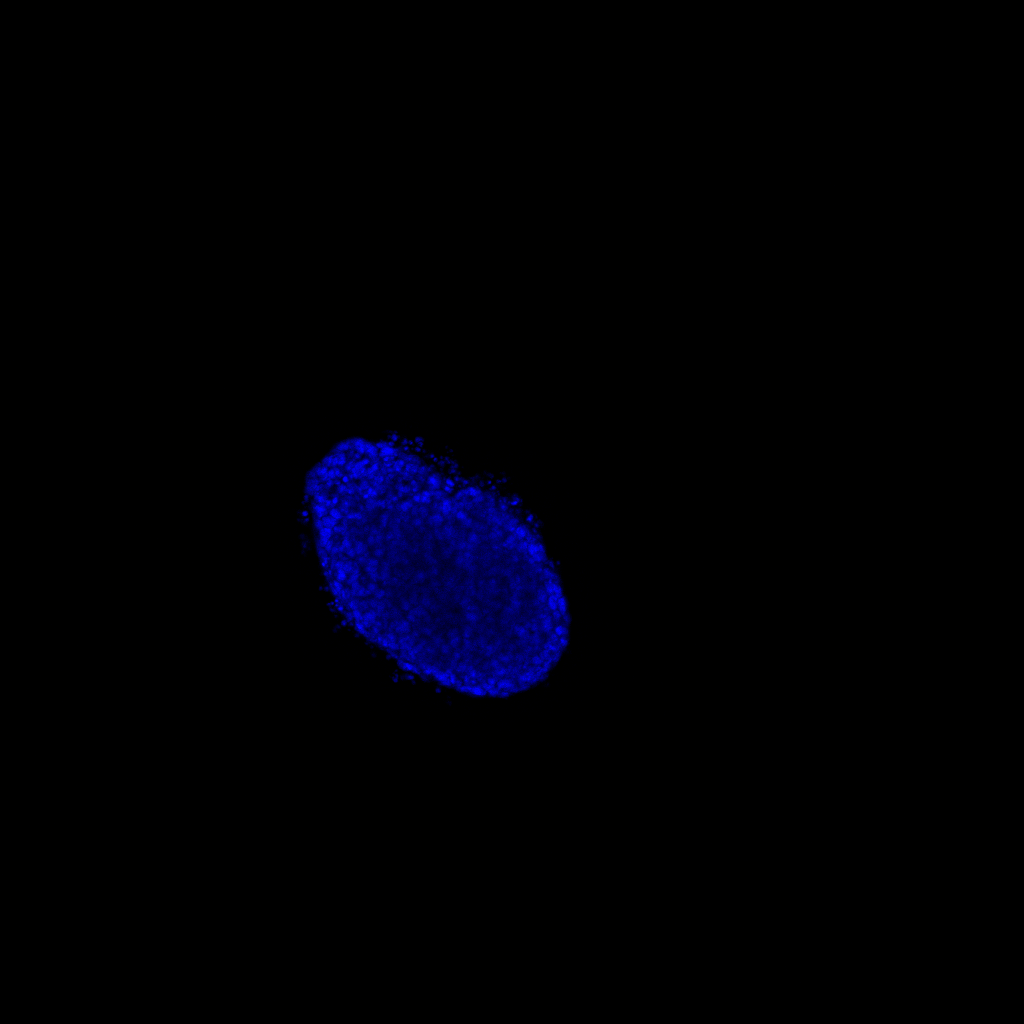

Supplement: Supplementary file 5 — Source data Fig. 3 [file 44318_2025_558_MOESM5_ESM.zip › Figure 3/panel 3C/KD-2_Sox2/image0139_image0139_RGB_DAPI.tif]

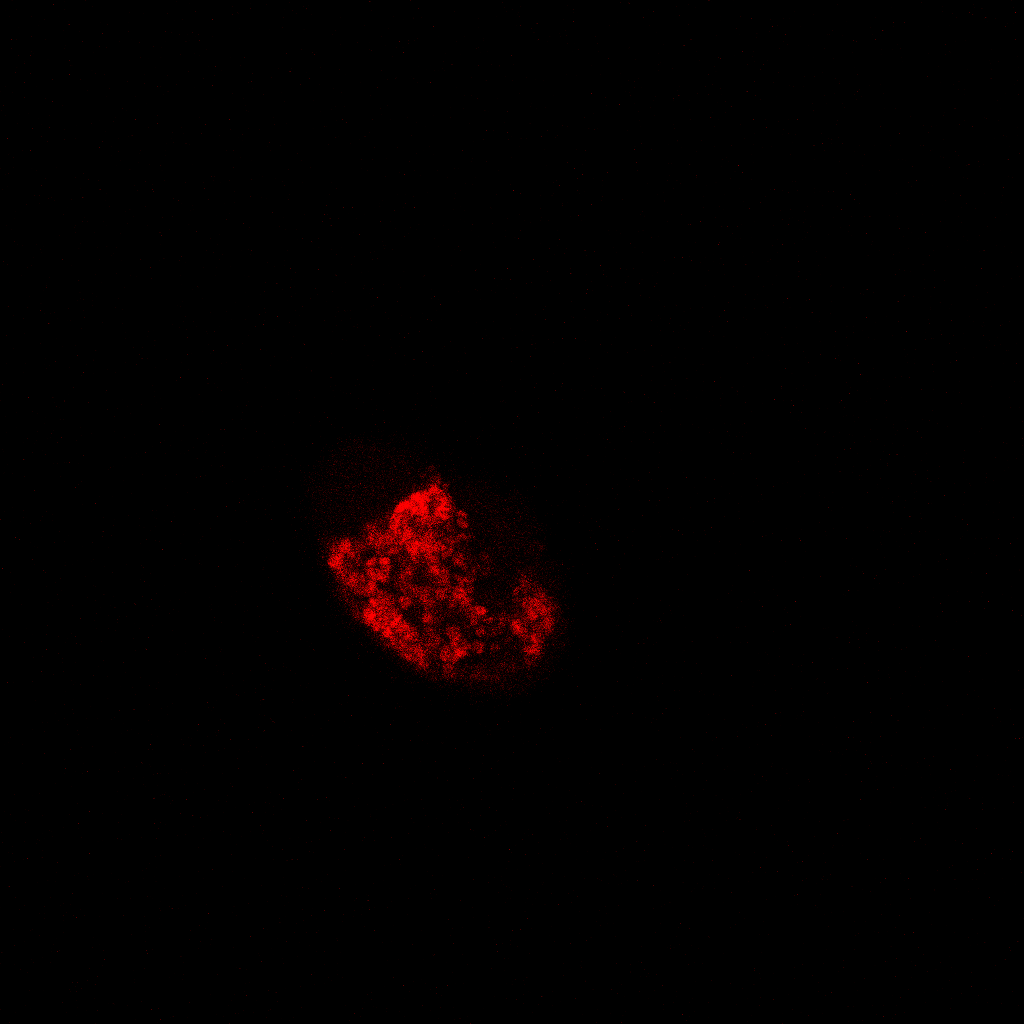

Supplement: Supplementary file 5 — Source data Fig. 3 [file 44318_2025_558_MOESM5_ESM.zip › Figure 3/panel 3C/KD-2_Sox2/image0139_image0139_RGB_Texas Red.tif]

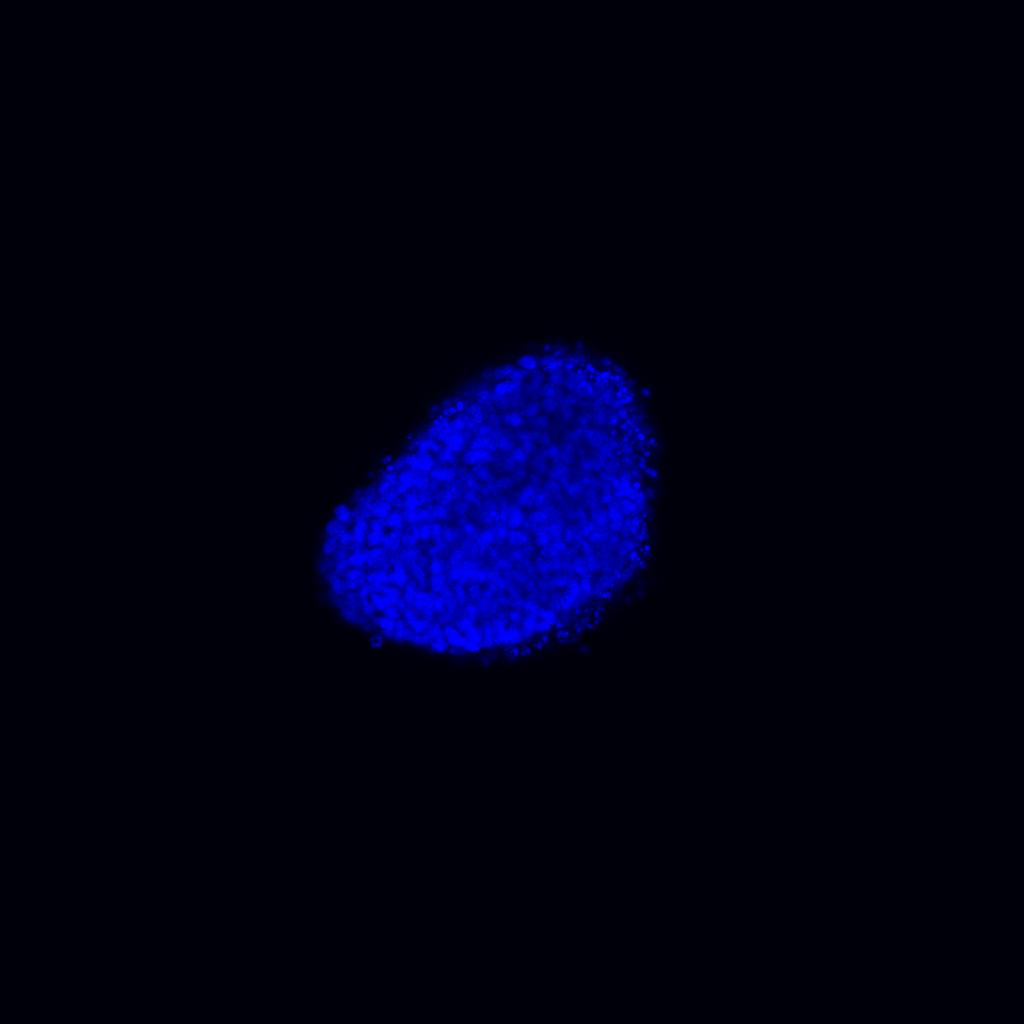

Supplement: Supplementary file 5 — Source data Fig. 3 [file 44318_2025_558_MOESM5_ESM.zip › Figure 3/panel 3C/KD-2_Oct4/seq8979_seq8979_RGB_DAPI.tif]

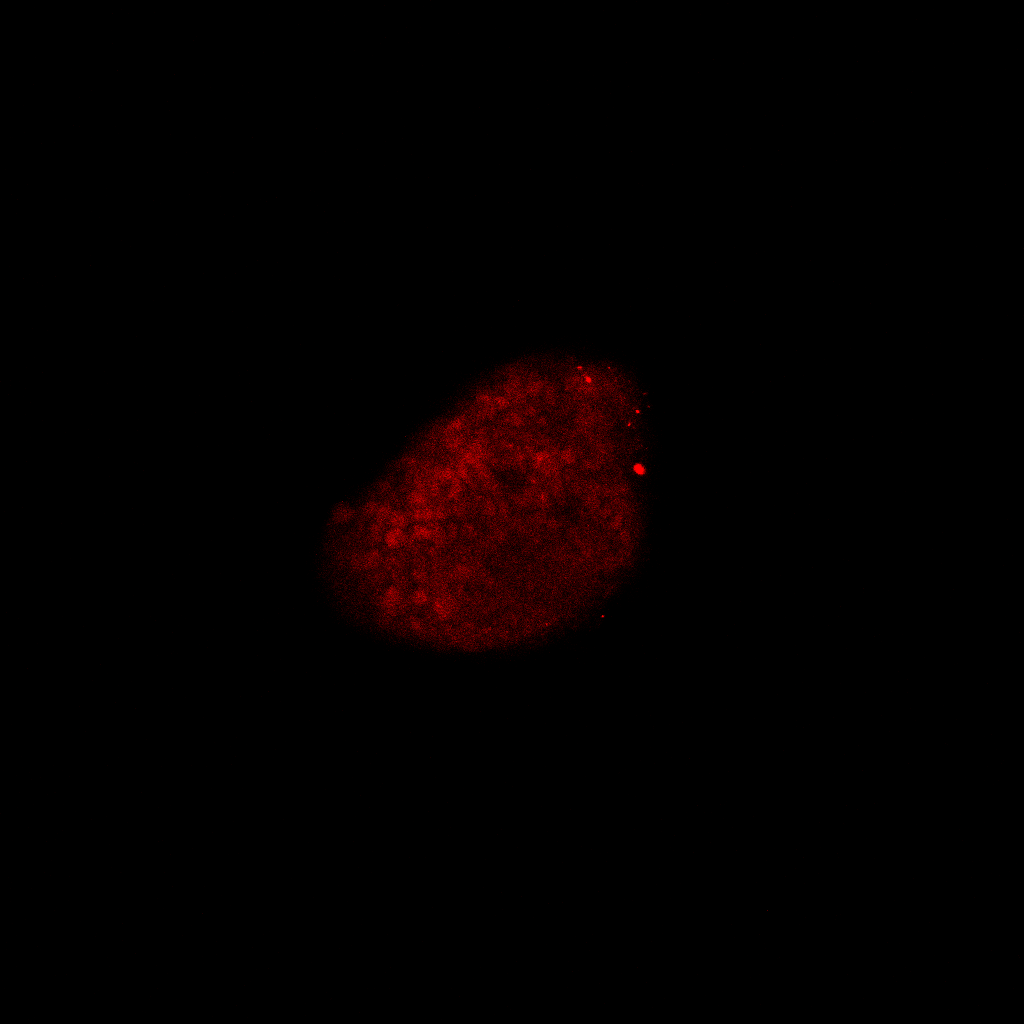

Supplement: Supplementary file 5 — Source data Fig. 3 [file 44318_2025_558_MOESM5_ESM.zip › Figure 3/panel 3C/KD-2_Oct4/seq8979_seq8979_RGB_Texas Red.tif]
