# Supplementary figures and images for: Non-cell-autonomous control of mouse gastruloid development by the ultra-conserved lncRNA T-UCstem1 (part 3 of 6)
Source: EMBO J. 2025 Oct 31;44(24):7620–48. doi: 10.1038/s44318-025-00558-2 (PMC12706062; doi:10.1038/s44318-025-00558-2)

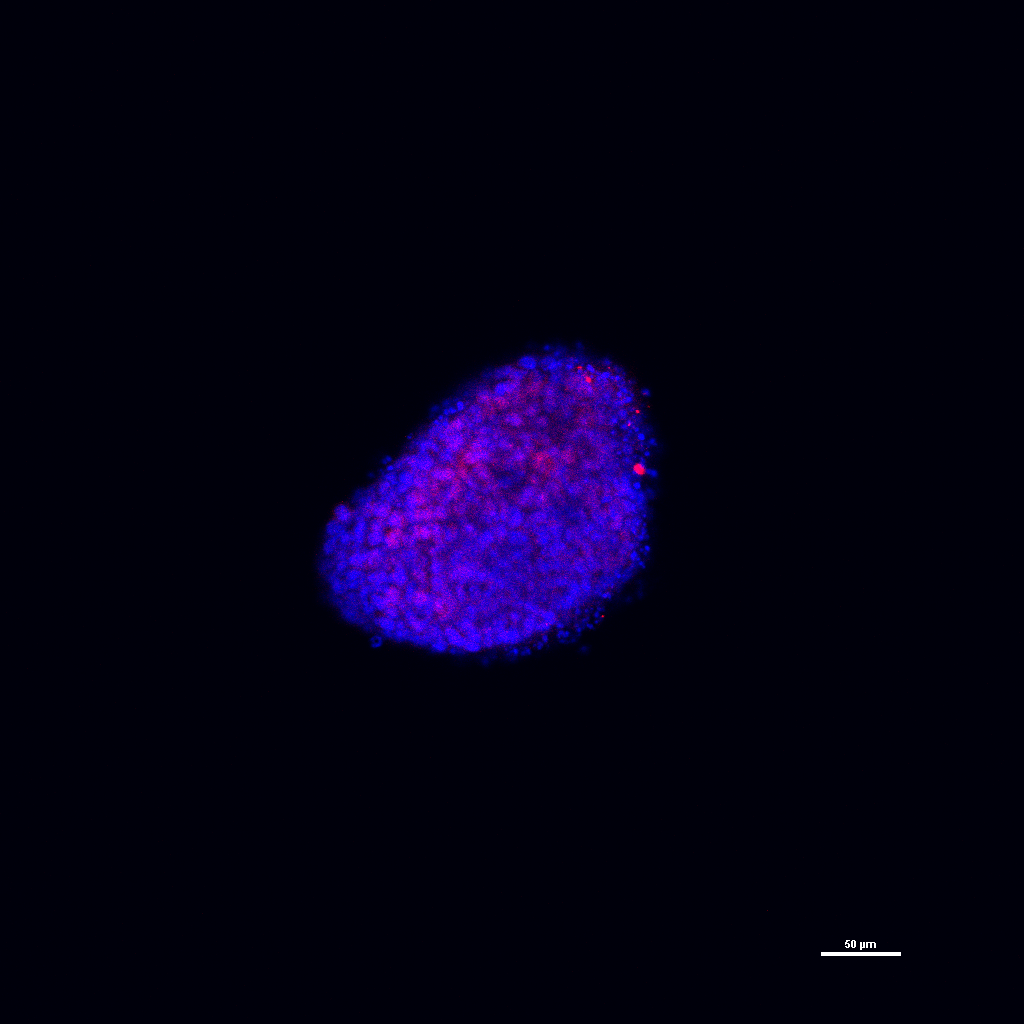

Supplement: Supplementary file 5 — Source data Fig. 3 [file 44318_2025_558_MOESM5_ESM.zip › Figure 3/panel 3C/KD-2_Oct4/seq8979_seq8979_RGB.tif]

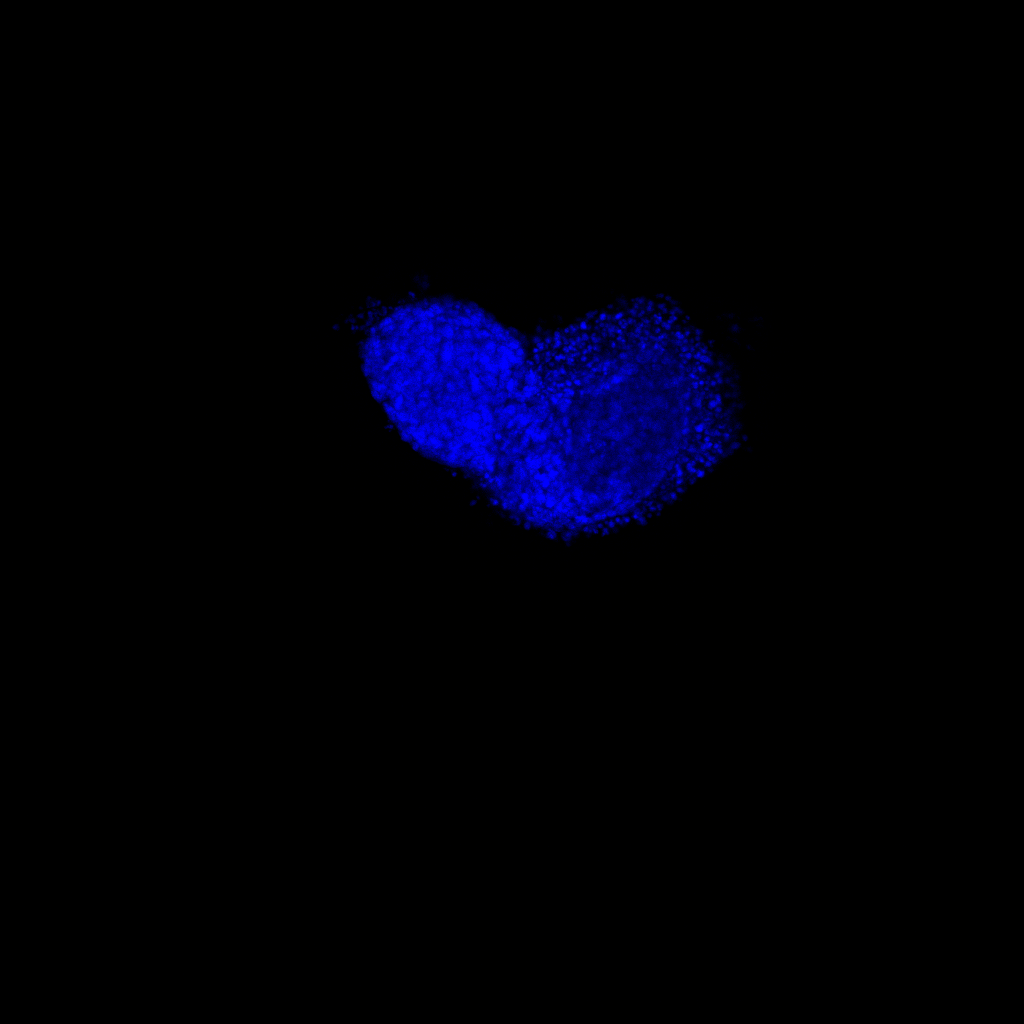

Supplement: Supplementary file 5 — Source data Fig. 3 [file 44318_2025_558_MOESM5_ESM.zip › Figure 3/panel 3C/NT_Sox17/image0128_image0128_RGB_DAPI.tif]

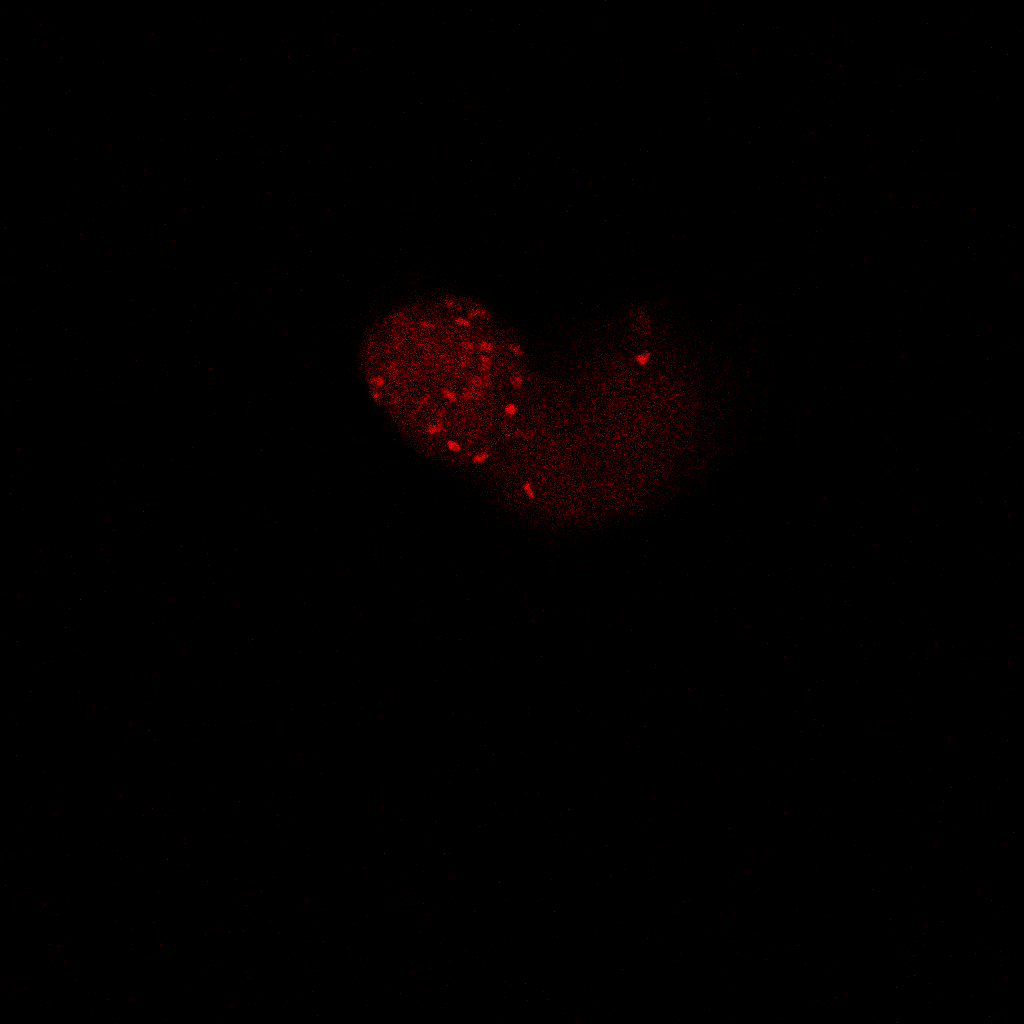

Supplement: Supplementary file 5 — Source data Fig. 3 [file 44318_2025_558_MOESM5_ESM.zip › Figure 3/panel 3C/NT_Sox17/image0128_image0128_RGB_Texas Red.tif]

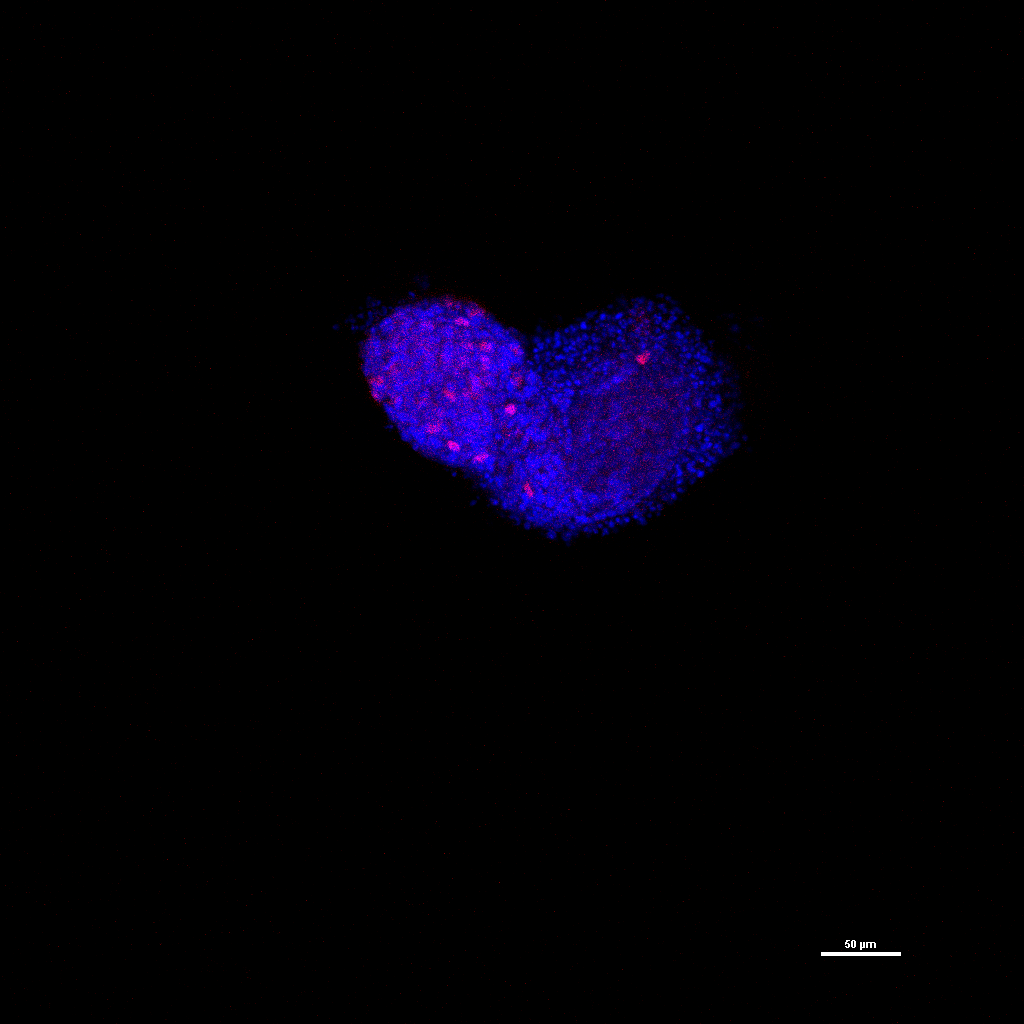

Supplement: Supplementary file 5 — Source data Fig. 3 [file 44318_2025_558_MOESM5_ESM.zip › Figure 3/panel 3C/NT_Sox17/image0128_image0128_RGB.tif]

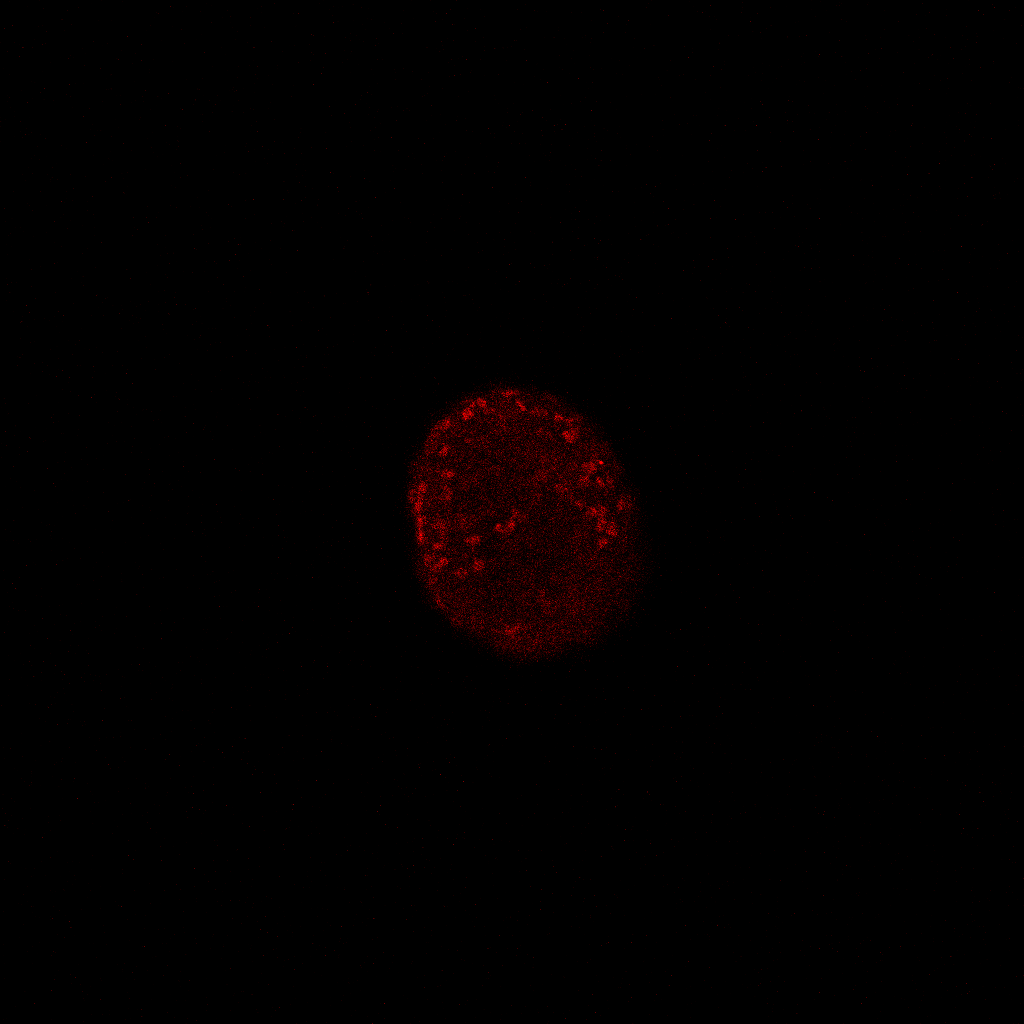

Supplement: Supplementary file 5 — Source data Fig. 3 [file 44318_2025_558_MOESM5_ESM.zip › Figure 3/panel 3C/KD-2_Sox17/image0132_image0132_RGB_Texas Red.tif]

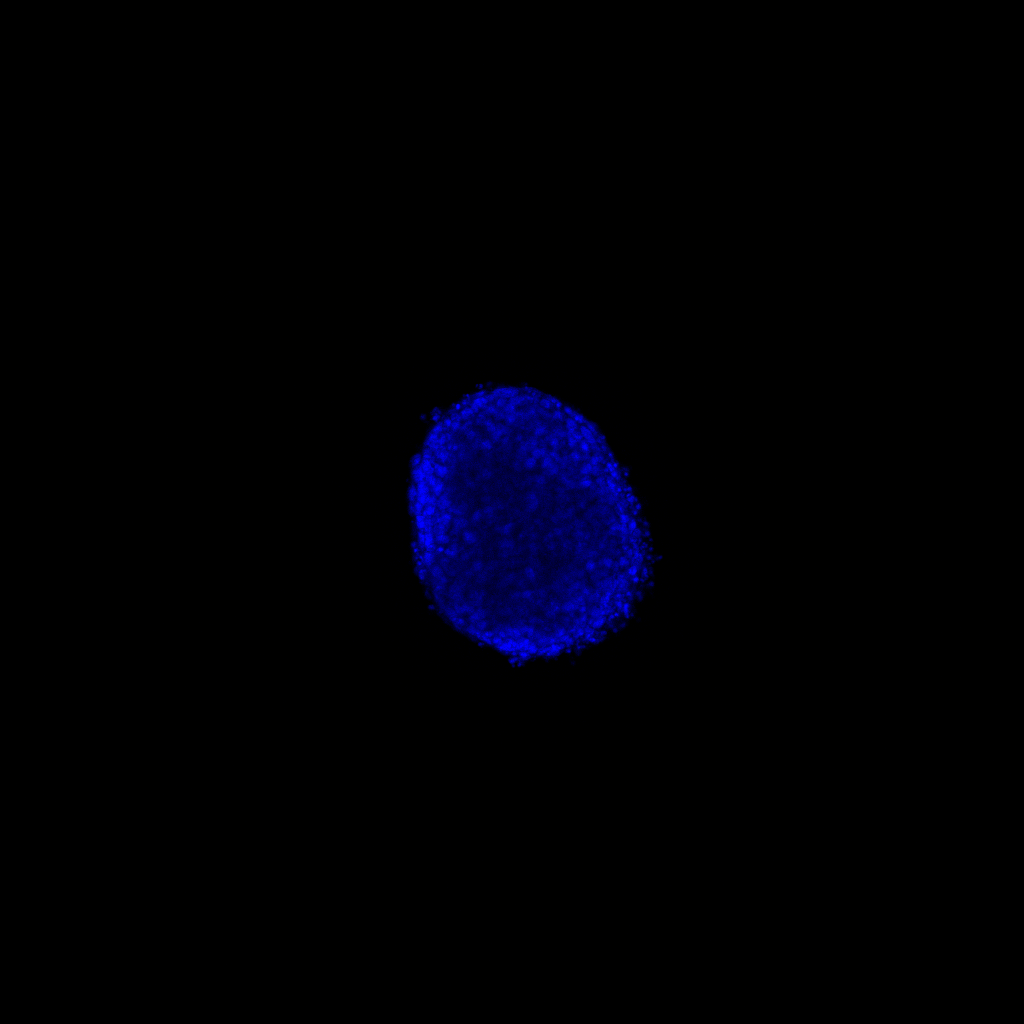

Supplement: Supplementary file 5 — Source data Fig. 3 [file 44318_2025_558_MOESM5_ESM.zip › Figure 3/panel 3C/KD-2_Sox17/image0132_image0132_RGB_DAPI.tif]

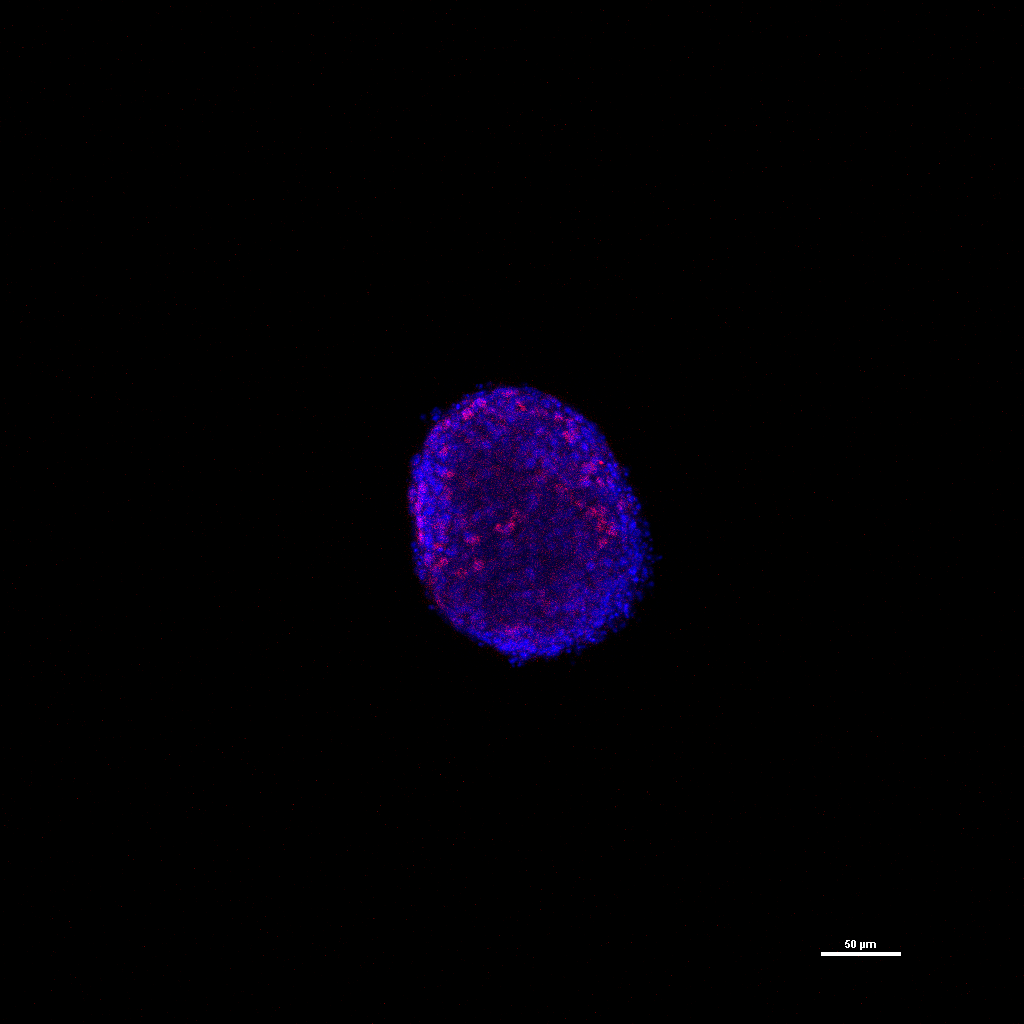

Supplement: Supplementary file 5 — Source data Fig. 3 [file 44318_2025_558_MOESM5_ESM.zip › Figure 3/panel 3C/KD-2_Sox17/image0132_image0132_RGB.tif]

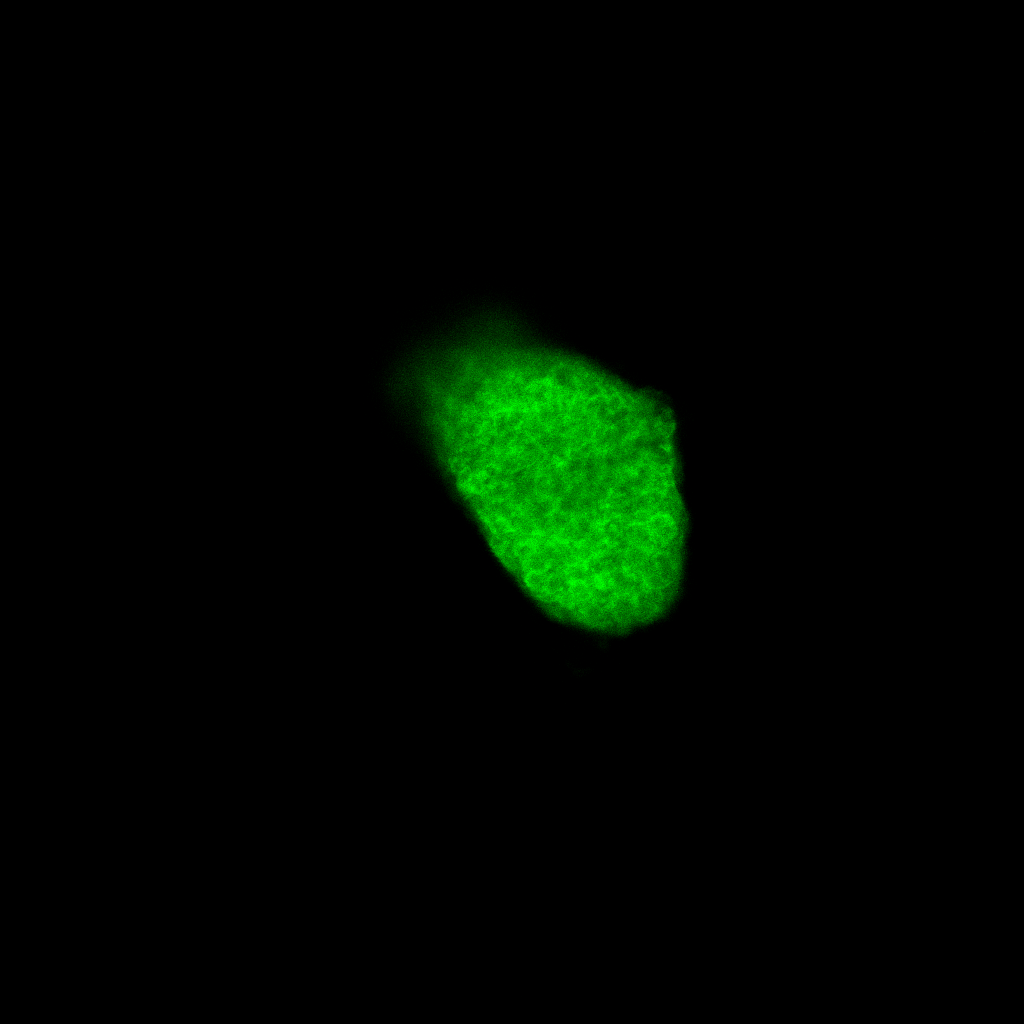

Supplement: Supplementary file 5 — Source data Fig. 3 [file 44318_2025_558_MOESM5_ESM.zip › Figure 3/panel 3C/KD-2_e-cadh/seq9125_seq9125_RGB_FITC.tif]

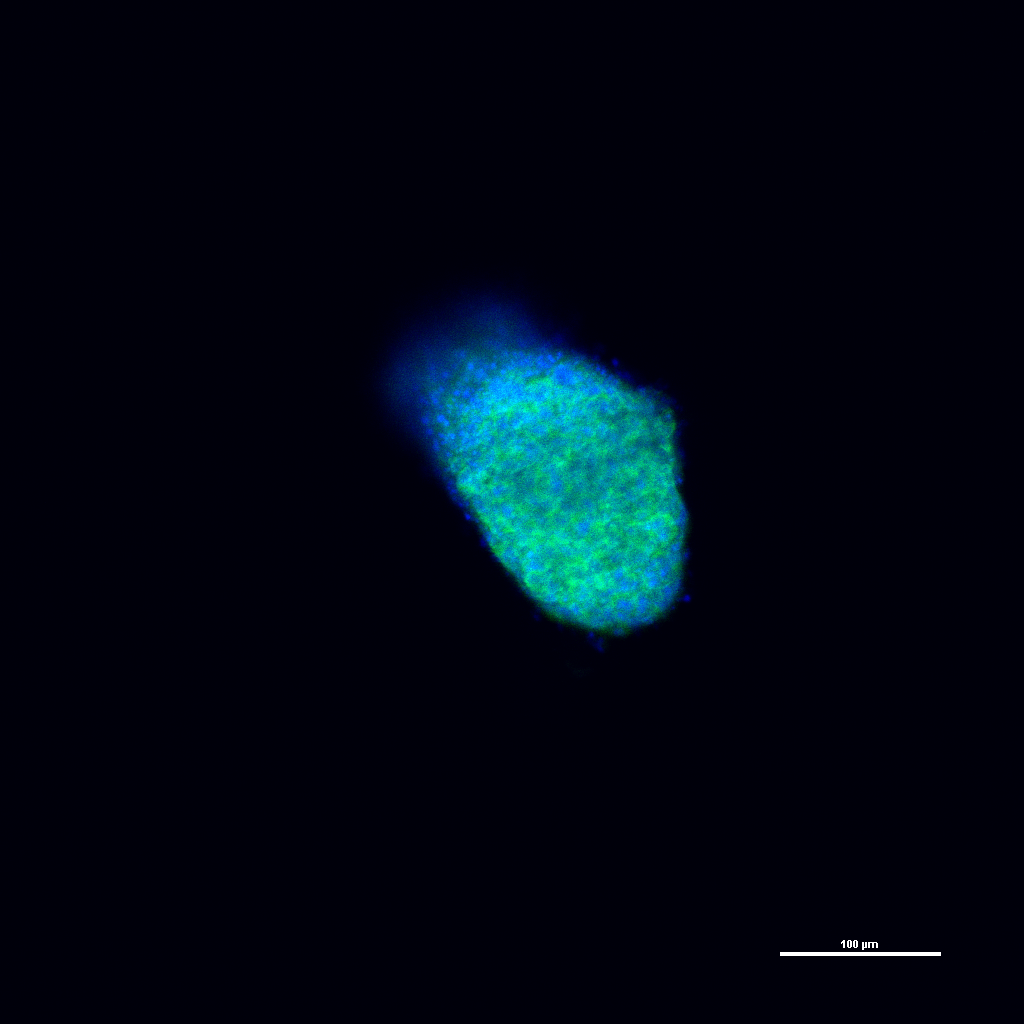

Supplement: Supplementary file 5 — Source data Fig. 3 [file 44318_2025_558_MOESM5_ESM.zip › Figure 3/panel 3C/KD-2_e-cadh/seq9125_seq9125_RGB.tif]

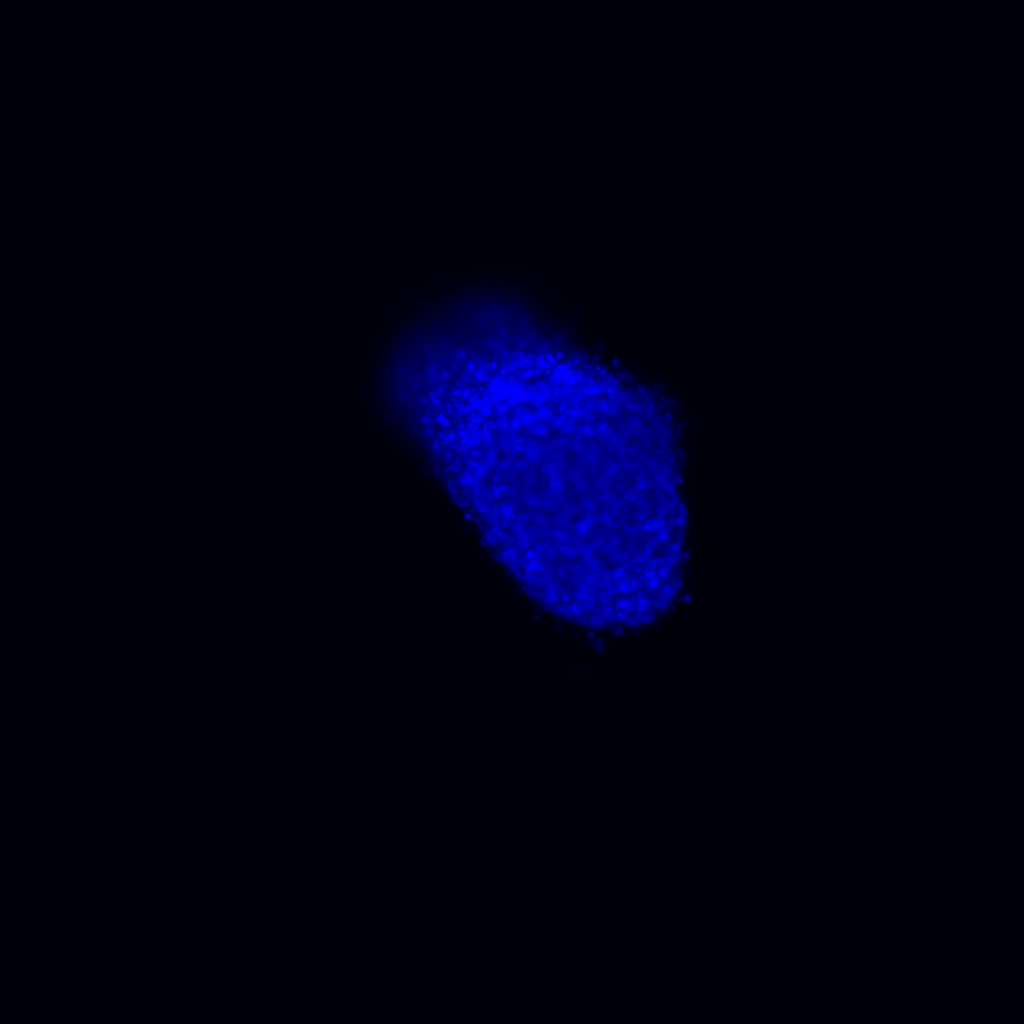

Supplement: Supplementary file 5 — Source data Fig. 3 [file 44318_2025_558_MOESM5_ESM.zip › Figure 3/panel 3C/KD-2_e-cadh/seq9125_seq9125_RGB_DAPI.tif]

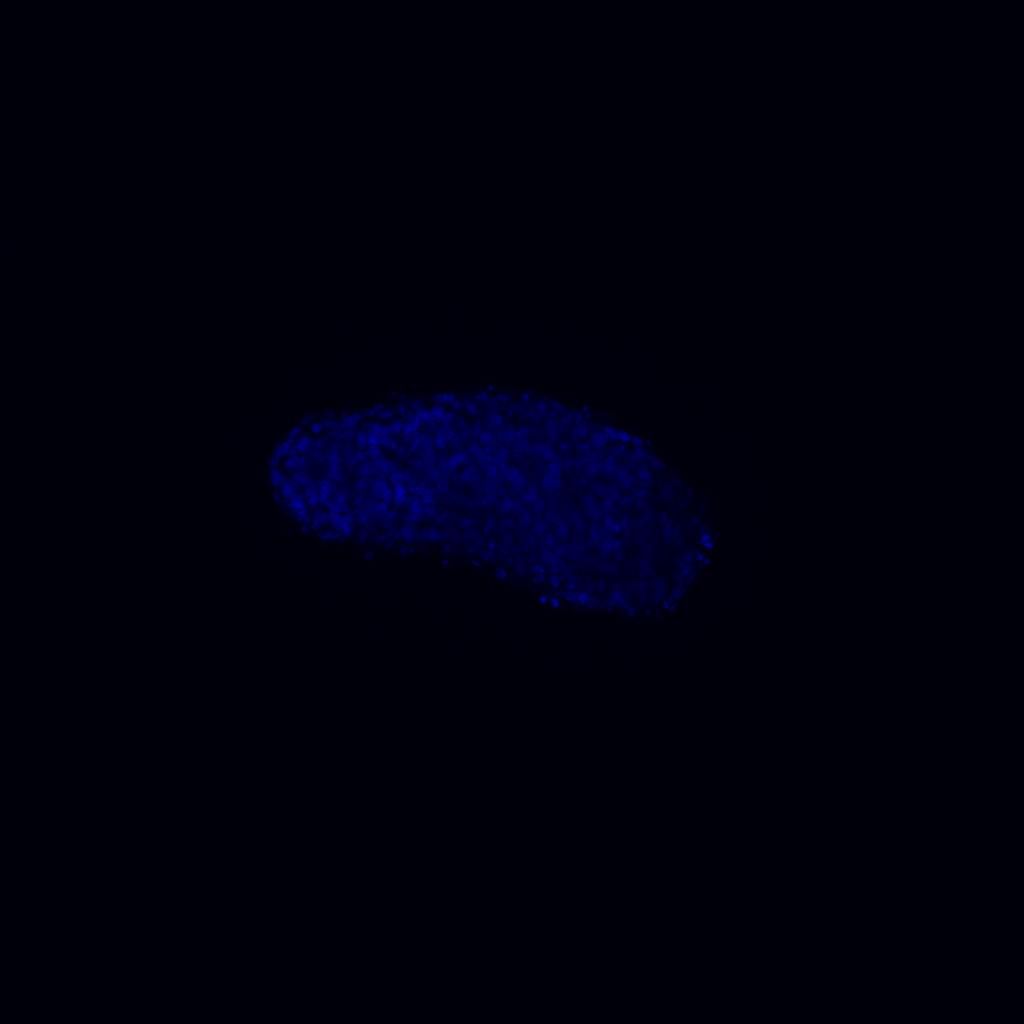

Supplement: Supplementary file 5 — Source data Fig. 3 [file 44318_2025_558_MOESM5_ESM.zip › Figure 3/panel 3C/NT-Bra/seq8958_seq8958_RGB_DAPI.tif]

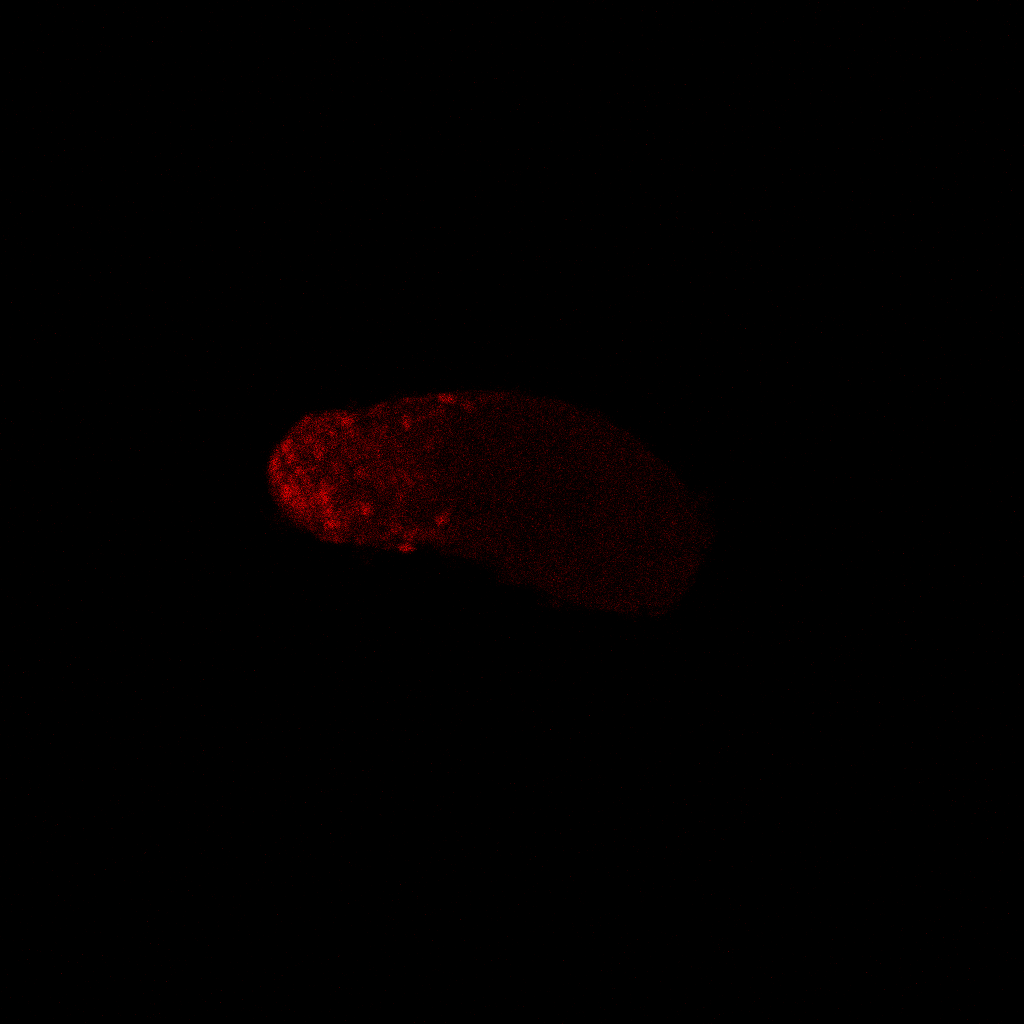

Supplement: Supplementary file 5 — Source data Fig. 3 [file 44318_2025_558_MOESM5_ESM.zip › Figure 3/panel 3C/NT-Bra/seq8958_seq8958_RGB_Texas Red.tif]

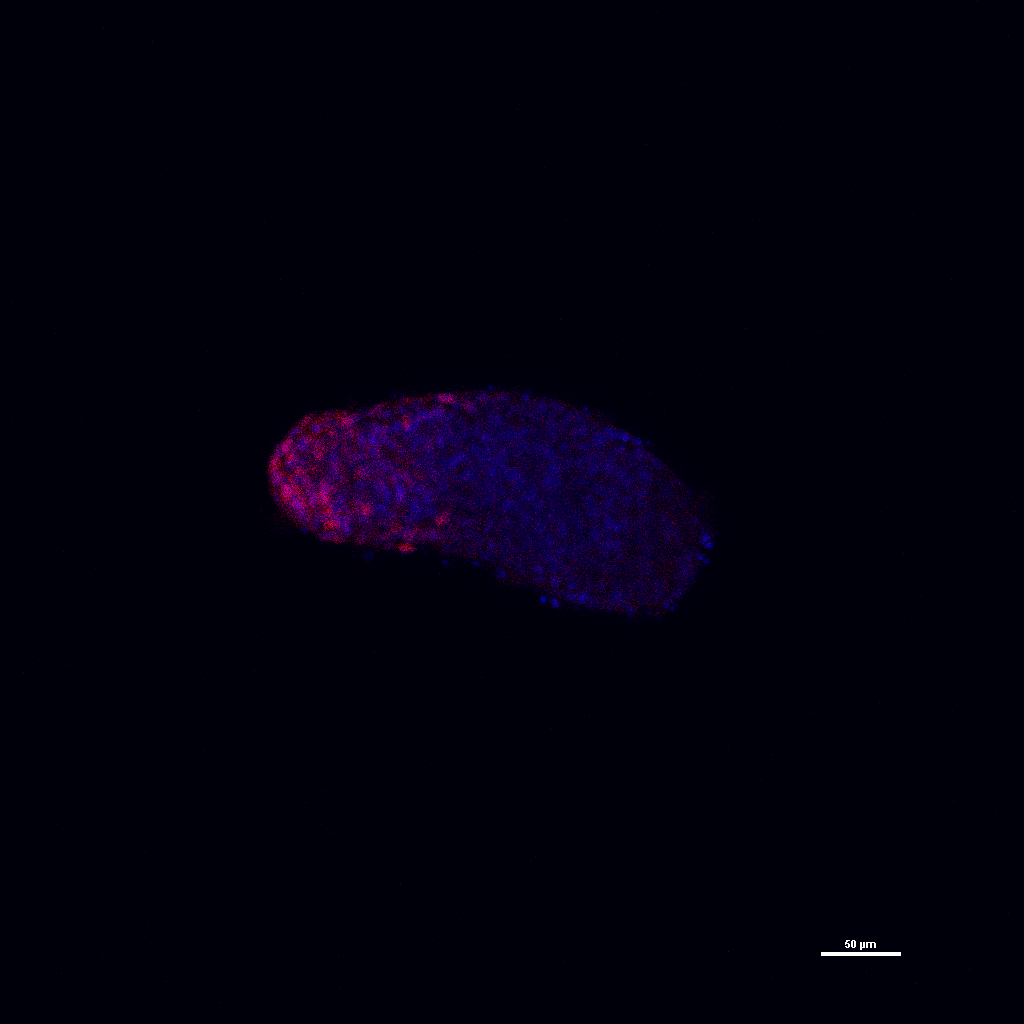

Supplement: Supplementary file 5 — Source data Fig. 3 [file 44318_2025_558_MOESM5_ESM.zip › Figure 3/panel 3C/NT-Bra/seq8958_seq8958_RGB.tif]

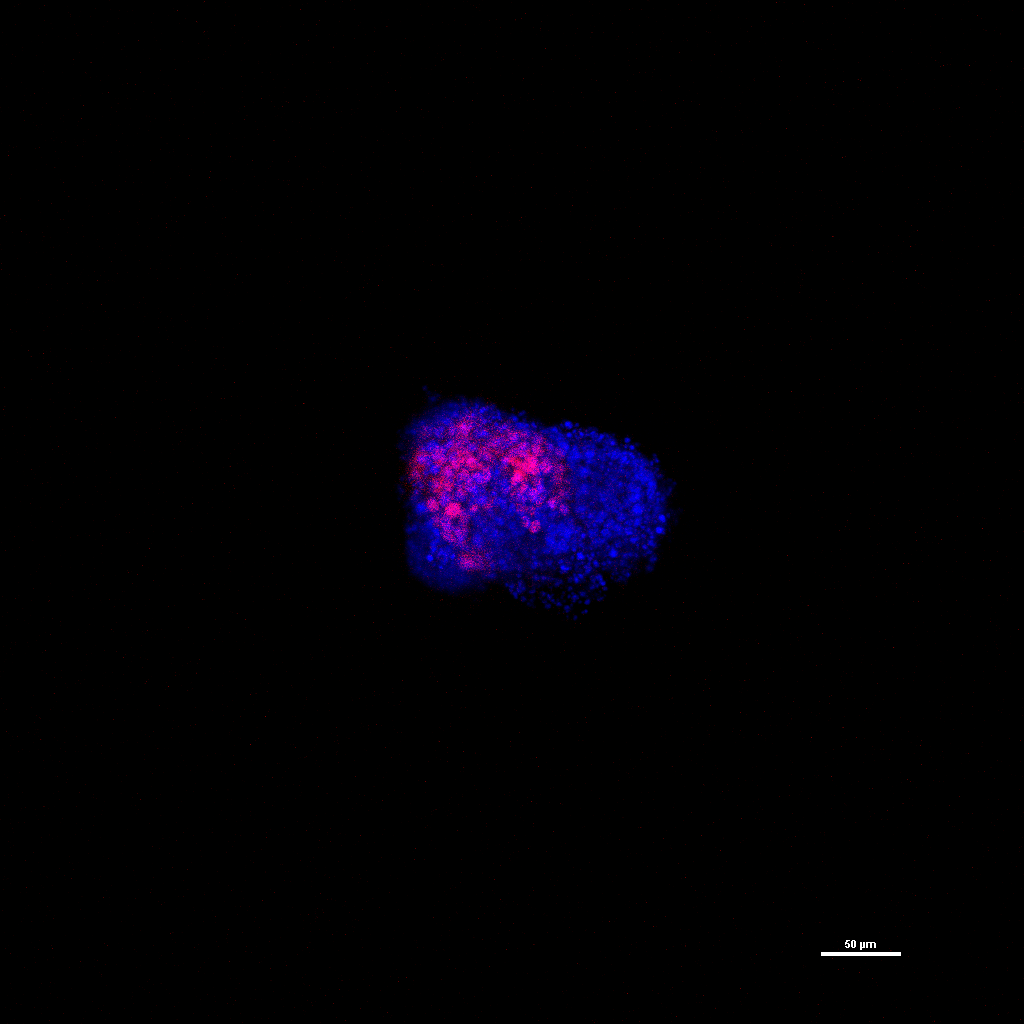

Supplement: Supplementary file 5 — Source data Fig. 3 [file 44318_2025_558_MOESM5_ESM.zip › Figure 3/panel 3C/KD-2_Nanog/image0140_image0140_RGB.tif]

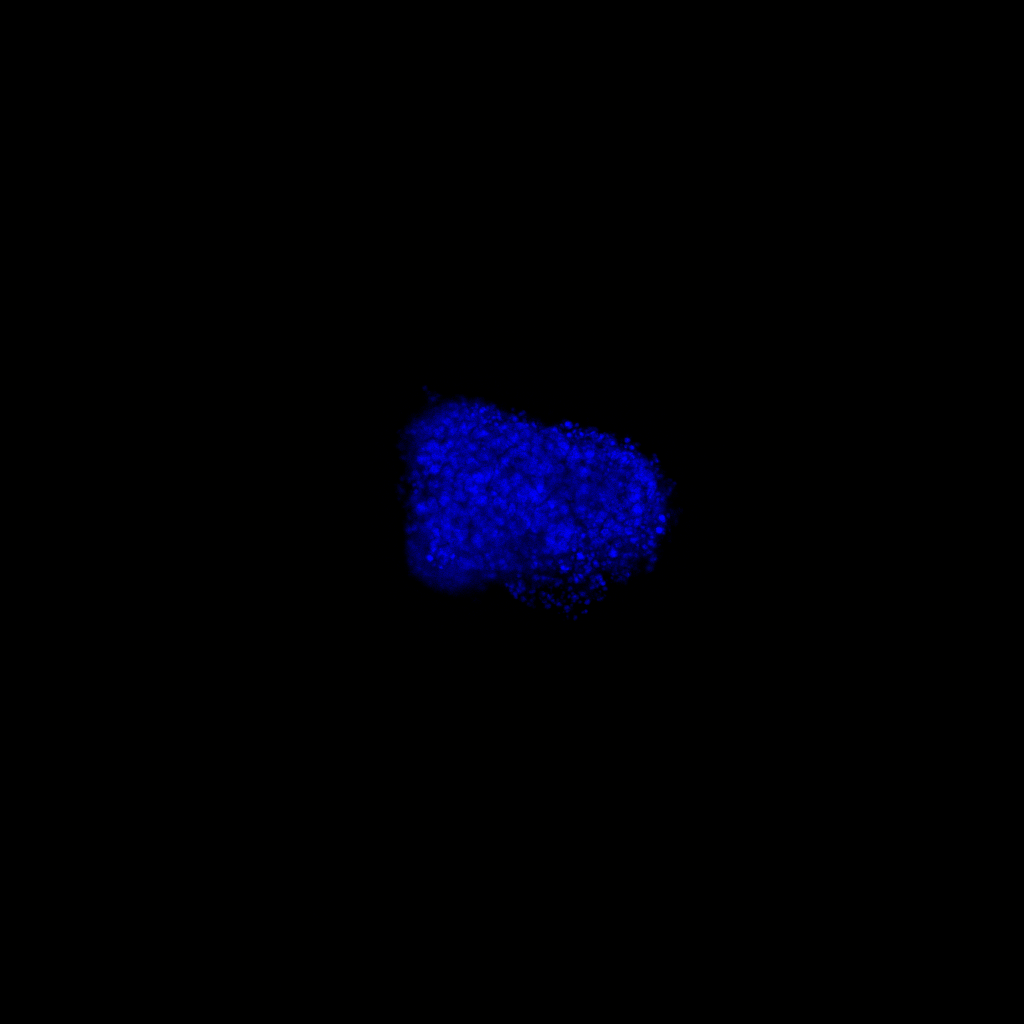

Supplement: Supplementary file 5 — Source data Fig. 3 [file 44318_2025_558_MOESM5_ESM.zip › Figure 3/panel 3C/KD-2_Nanog/image0140_image0140_RGB_DAPI.tif]

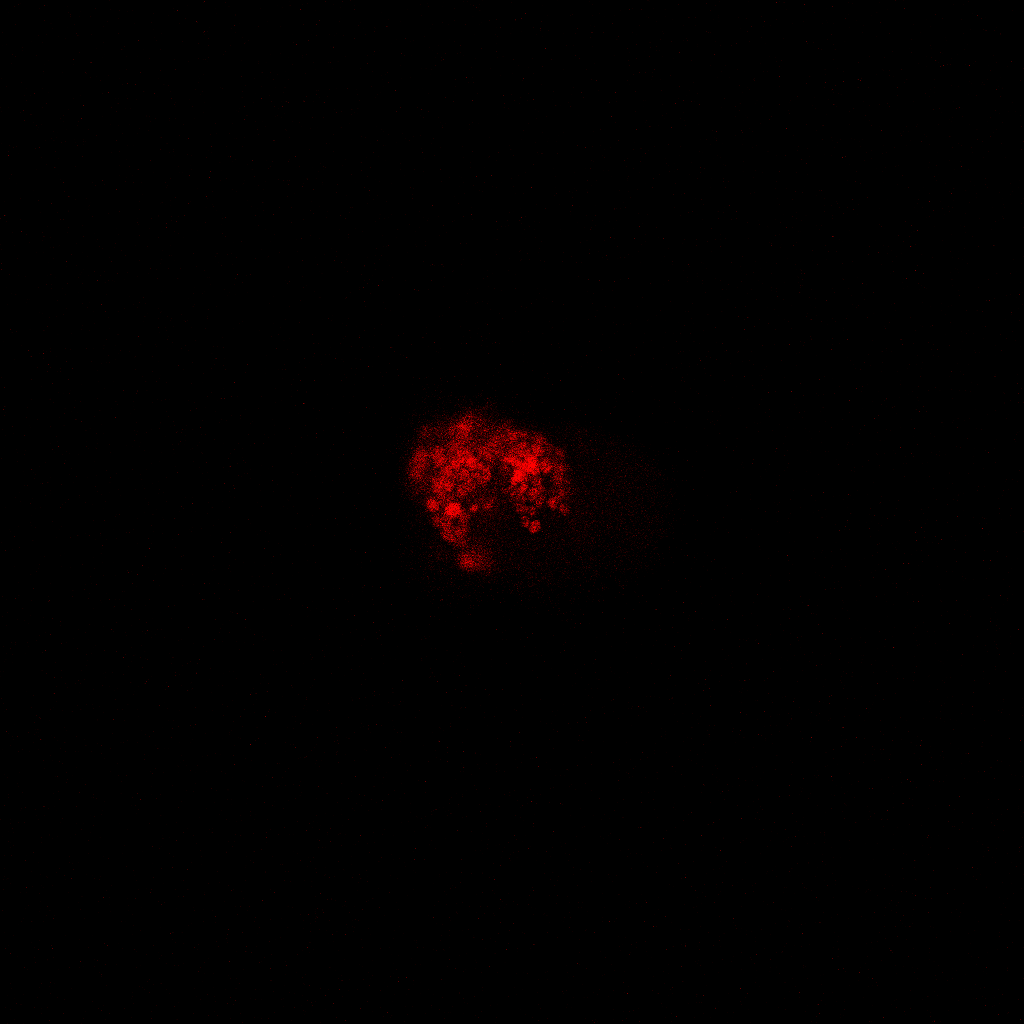

Supplement: Supplementary file 5 — Source data Fig. 3 [file 44318_2025_558_MOESM5_ESM.zip › Figure 3/panel 3C/KD-2_Nanog/image0140_image0140_RGB_Texas Red.tif]

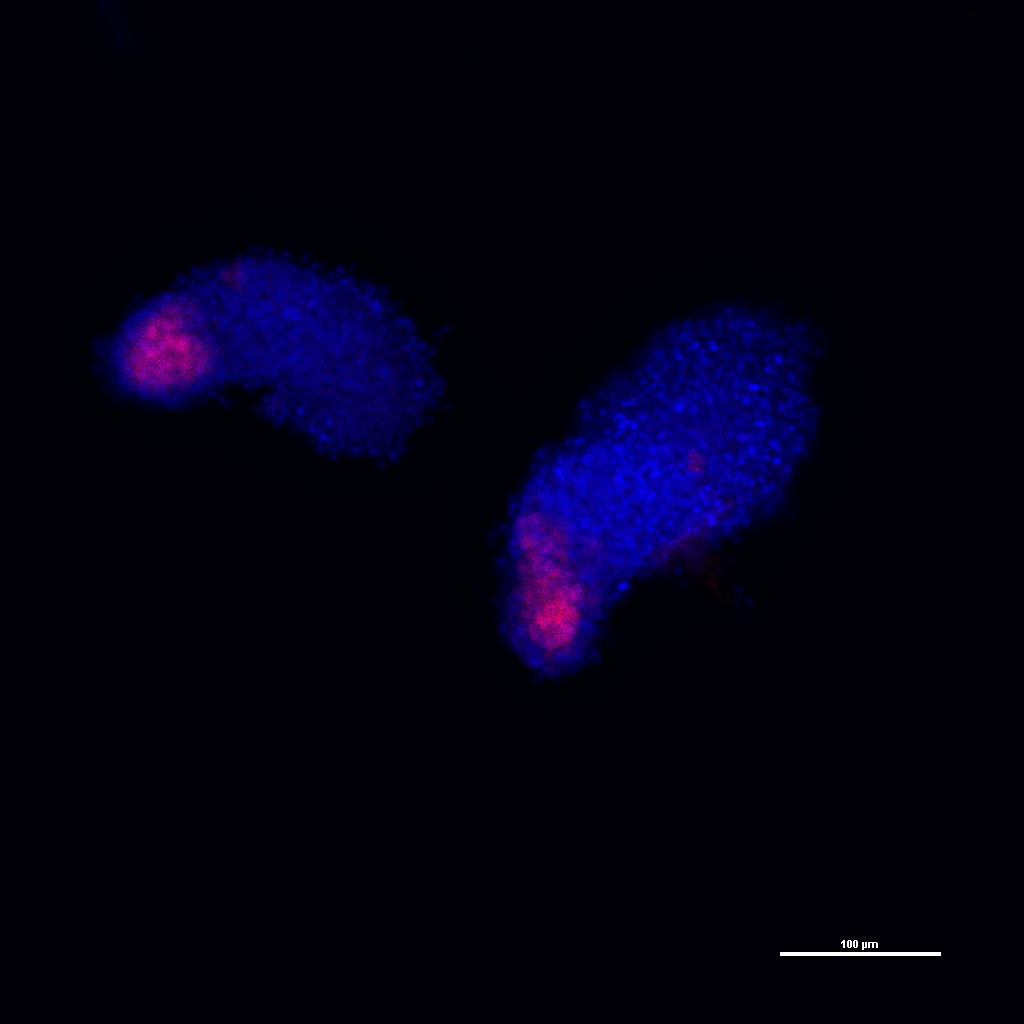

Supplement: Supplementary file 5 — Source data Fig. 3 [file 44318_2025_558_MOESM5_ESM.zip › Figure 3/panel 3C/NT_Sox2/seq11141_seq11141_RGB.tif]

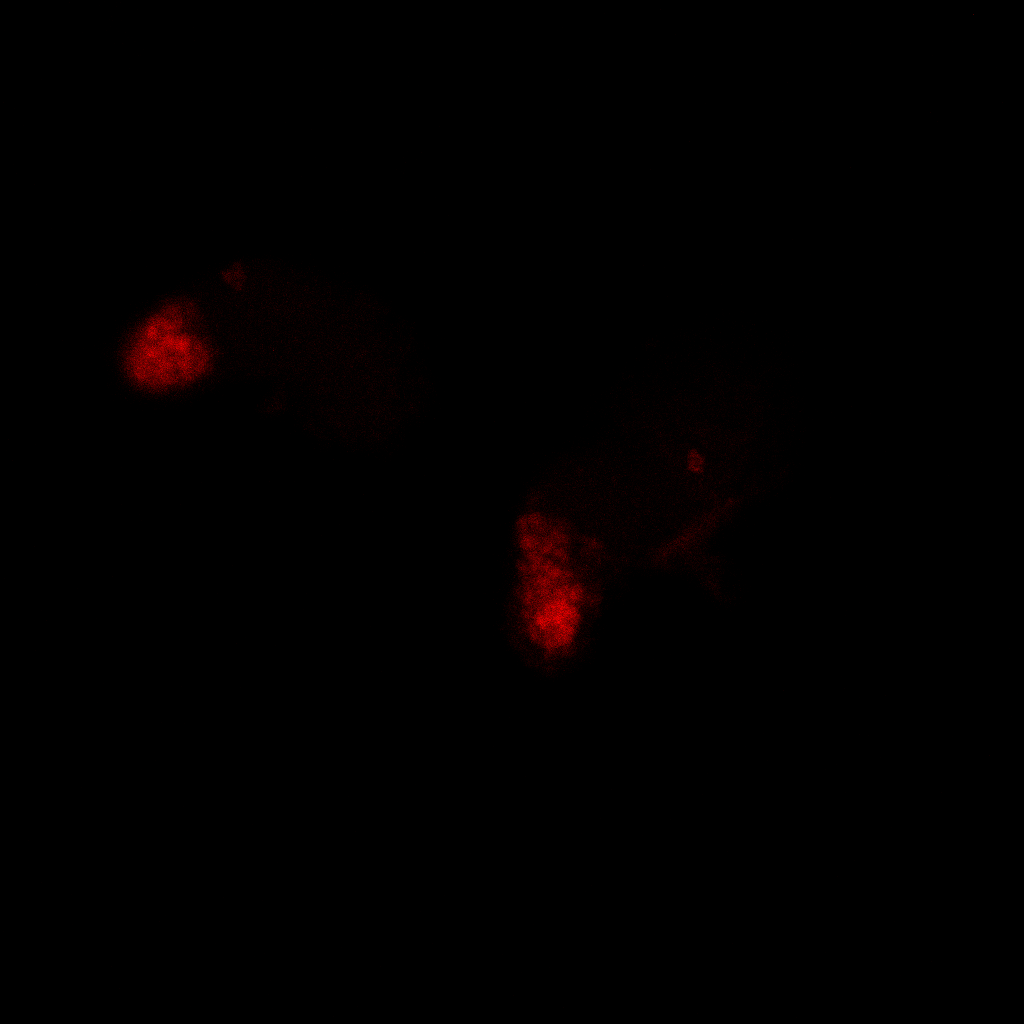

Supplement: Supplementary file 5 — Source data Fig. 3 [file 44318_2025_558_MOESM5_ESM.zip › Figure 3/panel 3C/NT_Sox2/seq11141_seq11141_RGB_TRITC.tif]

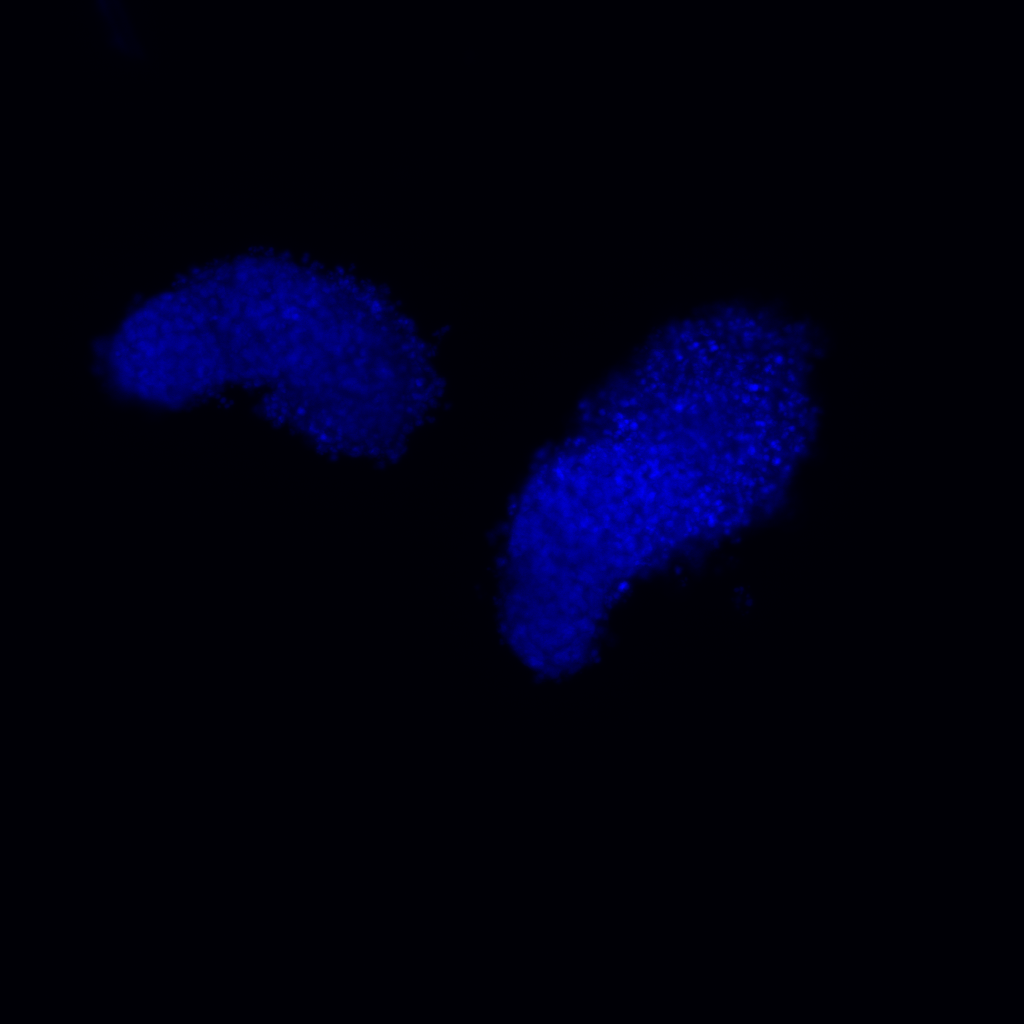

Supplement: Supplementary file 5 — Source data Fig. 3 [file 44318_2025_558_MOESM5_ESM.zip › Figure 3/panel 3C/NT_Sox2/seq11141_seq11141_RGB_DAPI.tif]

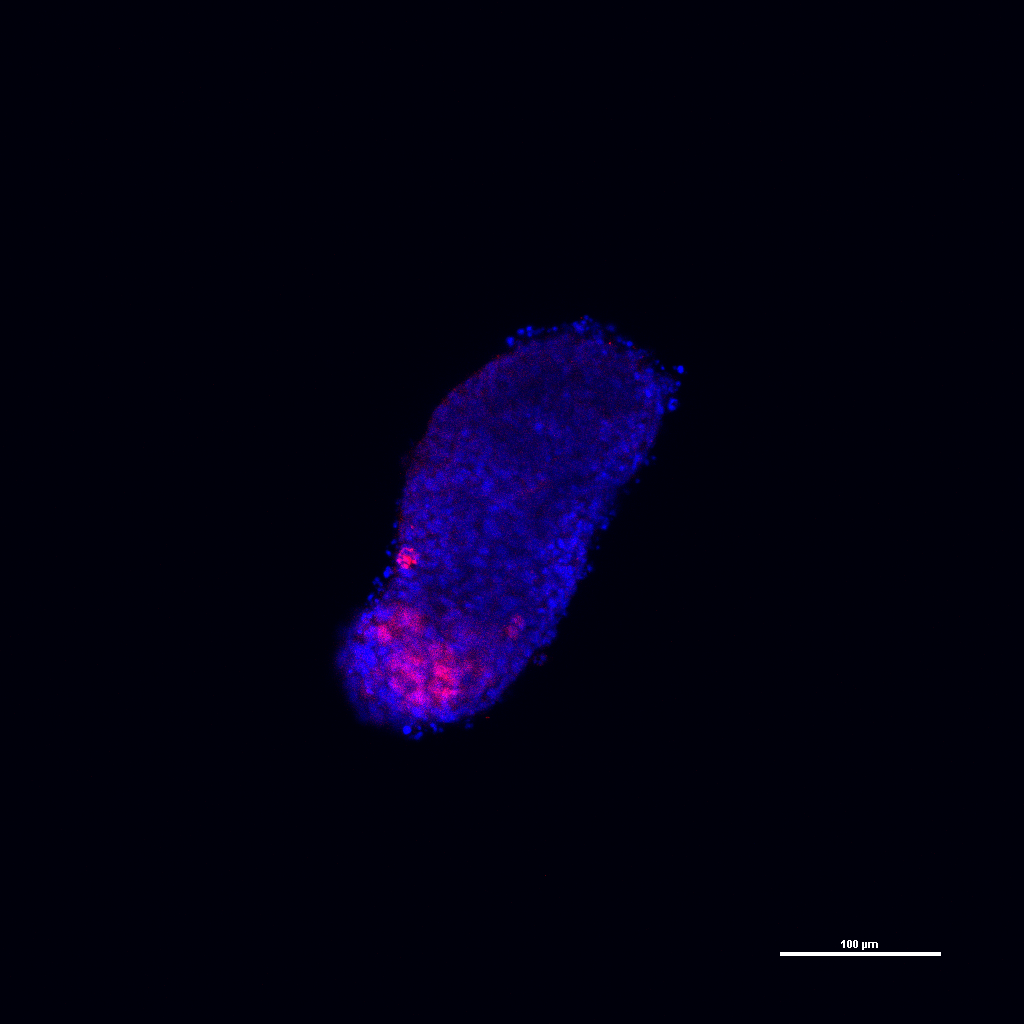

Supplement: Supplementary file 5 — Source data Fig. 3 [file 44318_2025_558_MOESM5_ESM.zip › Figure 3/panel 3C/NT_Oct4/seq8972_seq8972_RGB.tif]

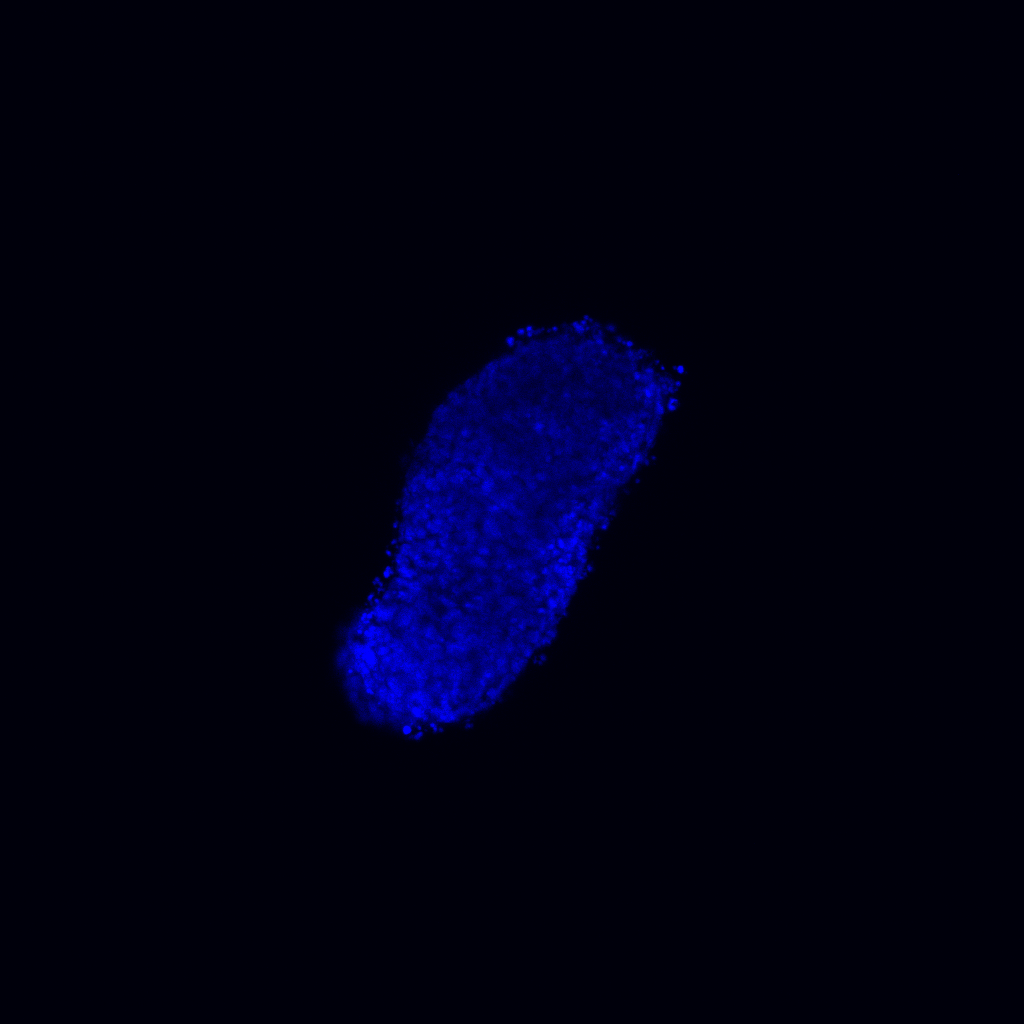

Supplement: Supplementary file 5 — Source data Fig. 3 [file 44318_2025_558_MOESM5_ESM.zip › Figure 3/panel 3C/NT_Oct4/seq8972_seq8972_RGB_DAPI.tif]

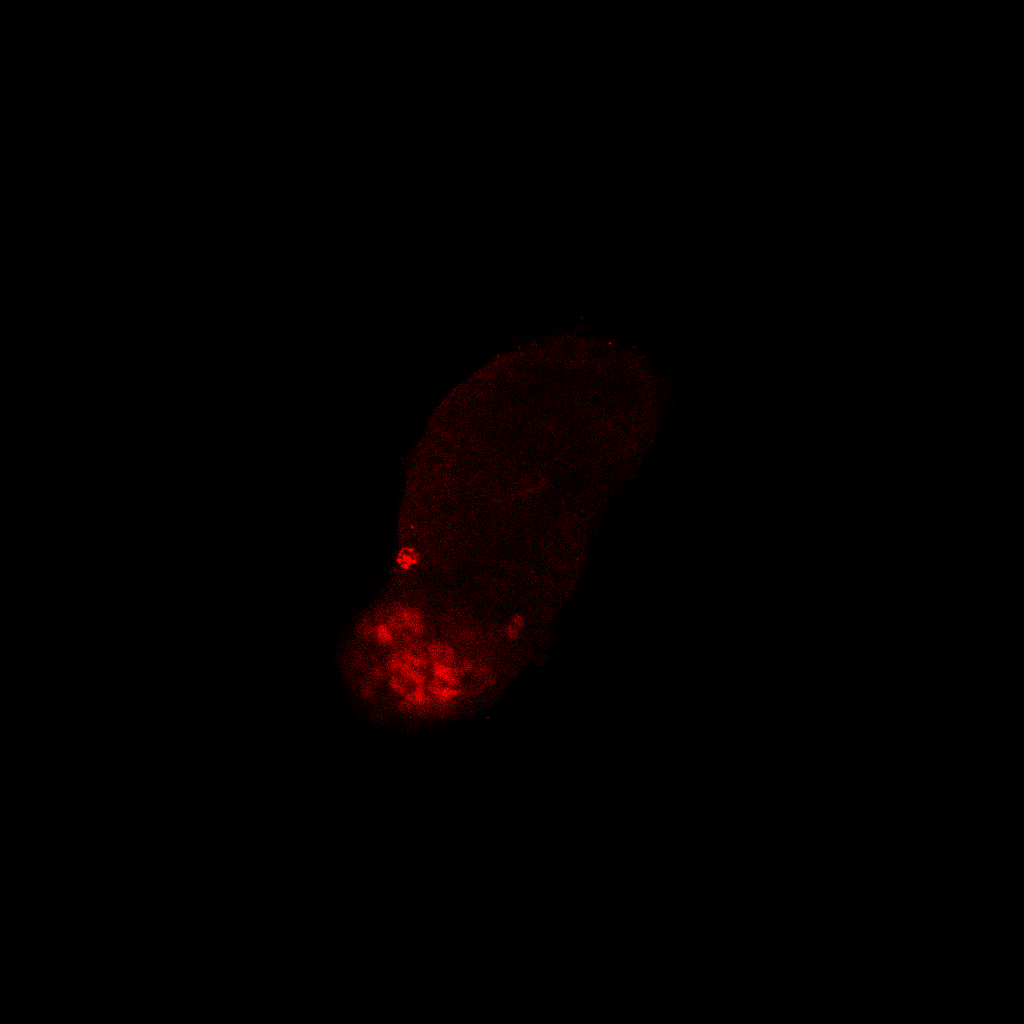

Supplement: Supplementary file 5 — Source data Fig. 3 [file 44318_2025_558_MOESM5_ESM.zip › Figure 3/panel 3C/NT_Oct4/seq8972_seq8972_RGB_Texas Red.tif]

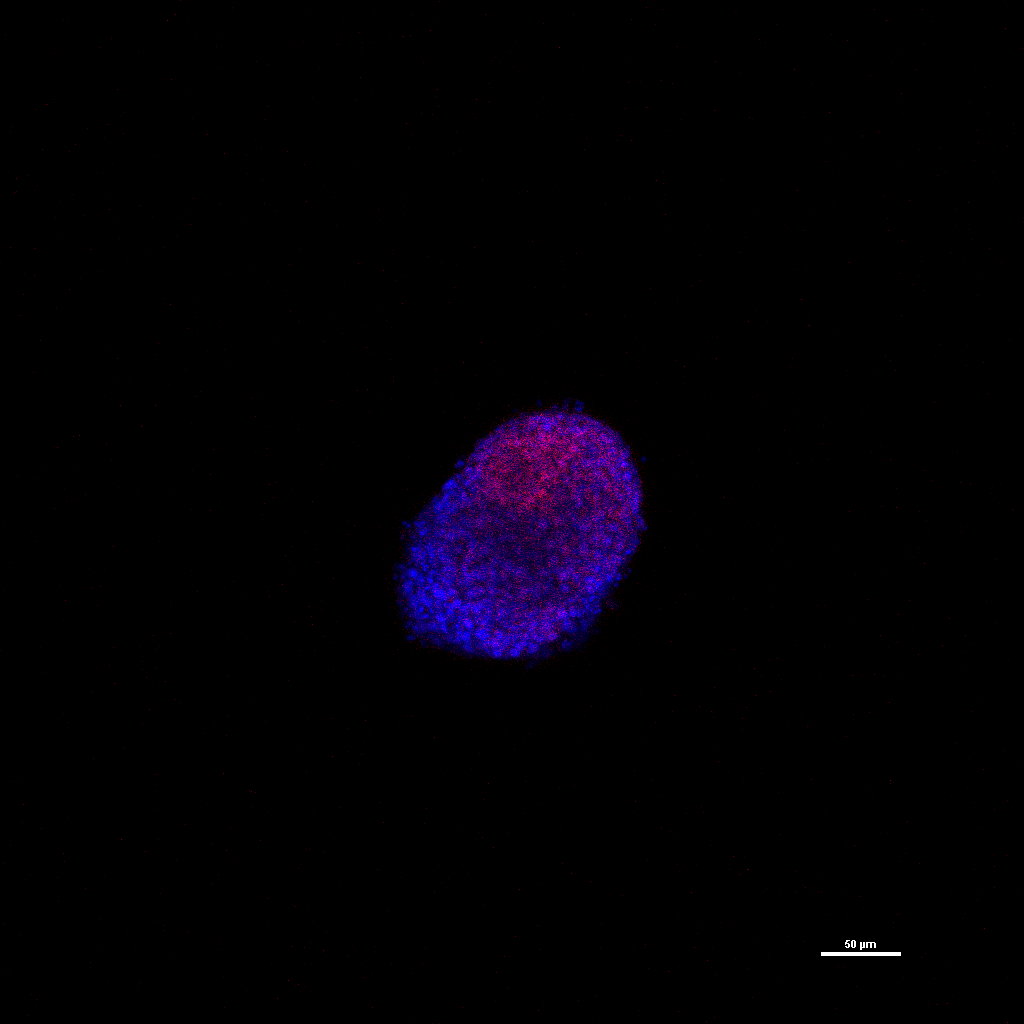

Supplement: Supplementary file 5 — Source data Fig. 3 [file 44318_2025_558_MOESM5_ESM.zip › Figure 3/panel 3C/KD-2_Cdx2/image0137_image0137_RGB.tif]

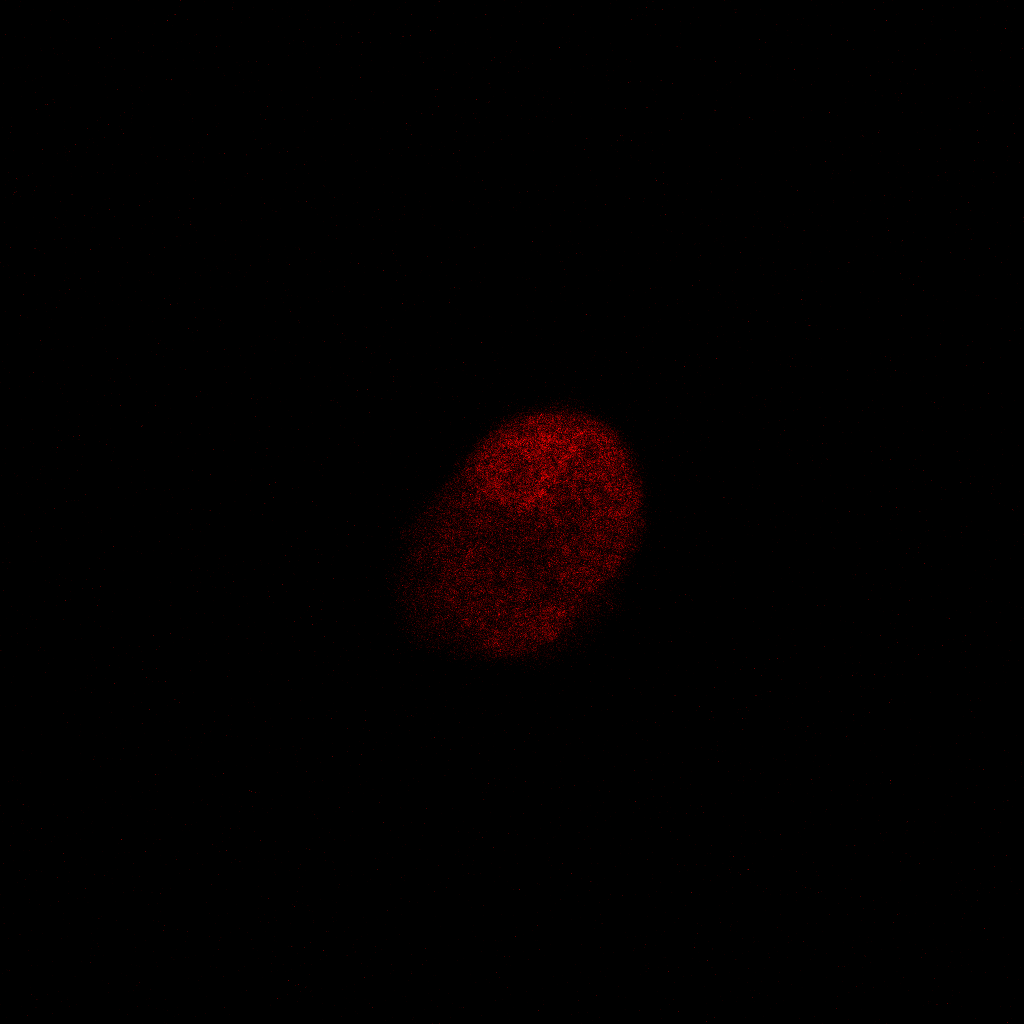

Supplement: Supplementary file 5 — Source data Fig. 3 [file 44318_2025_558_MOESM5_ESM.zip › Figure 3/panel 3C/KD-2_Cdx2/image0137_image0137_RGB_Texas Red.tif]

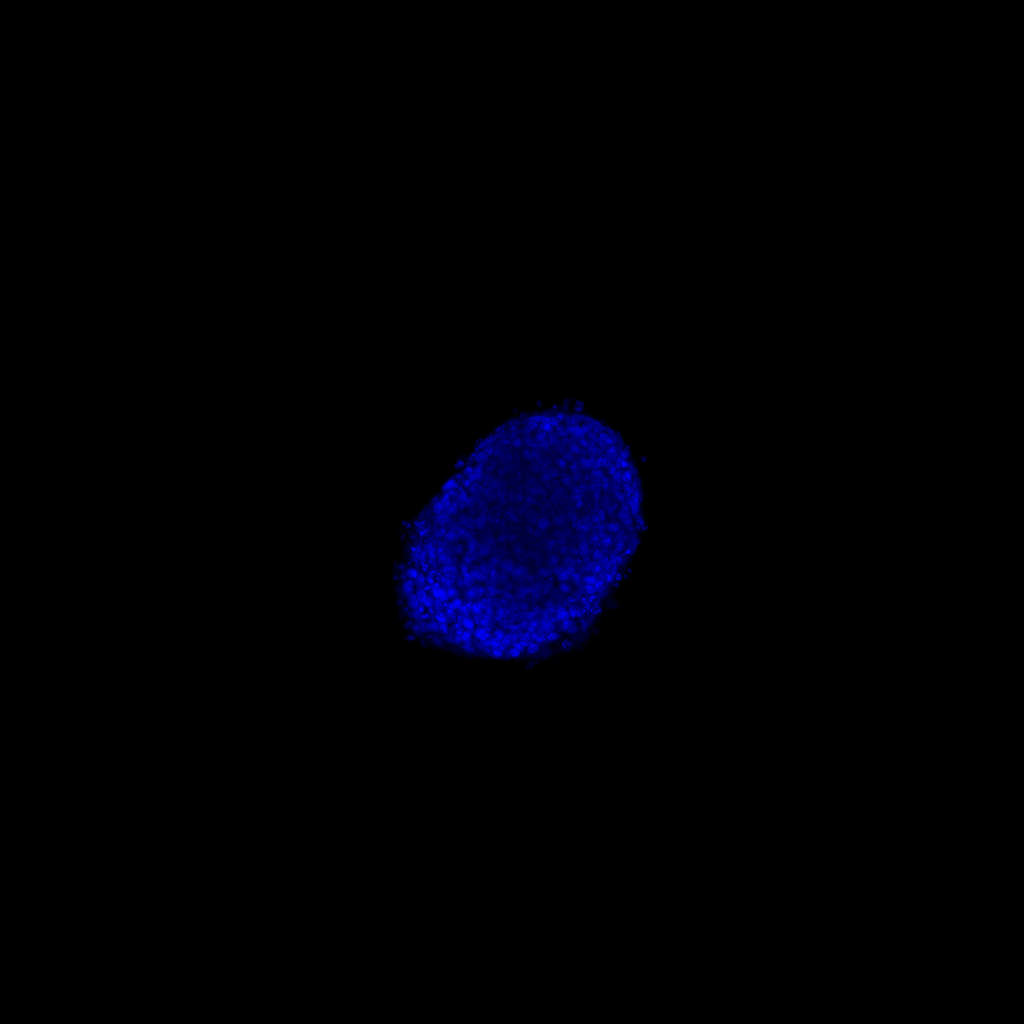

Supplement: Supplementary file 5 — Source data Fig. 3 [file 44318_2025_558_MOESM5_ESM.zip › Figure 3/panel 3C/KD-2_Cdx2/image0137_image0137_RGB_DAPI.tif]

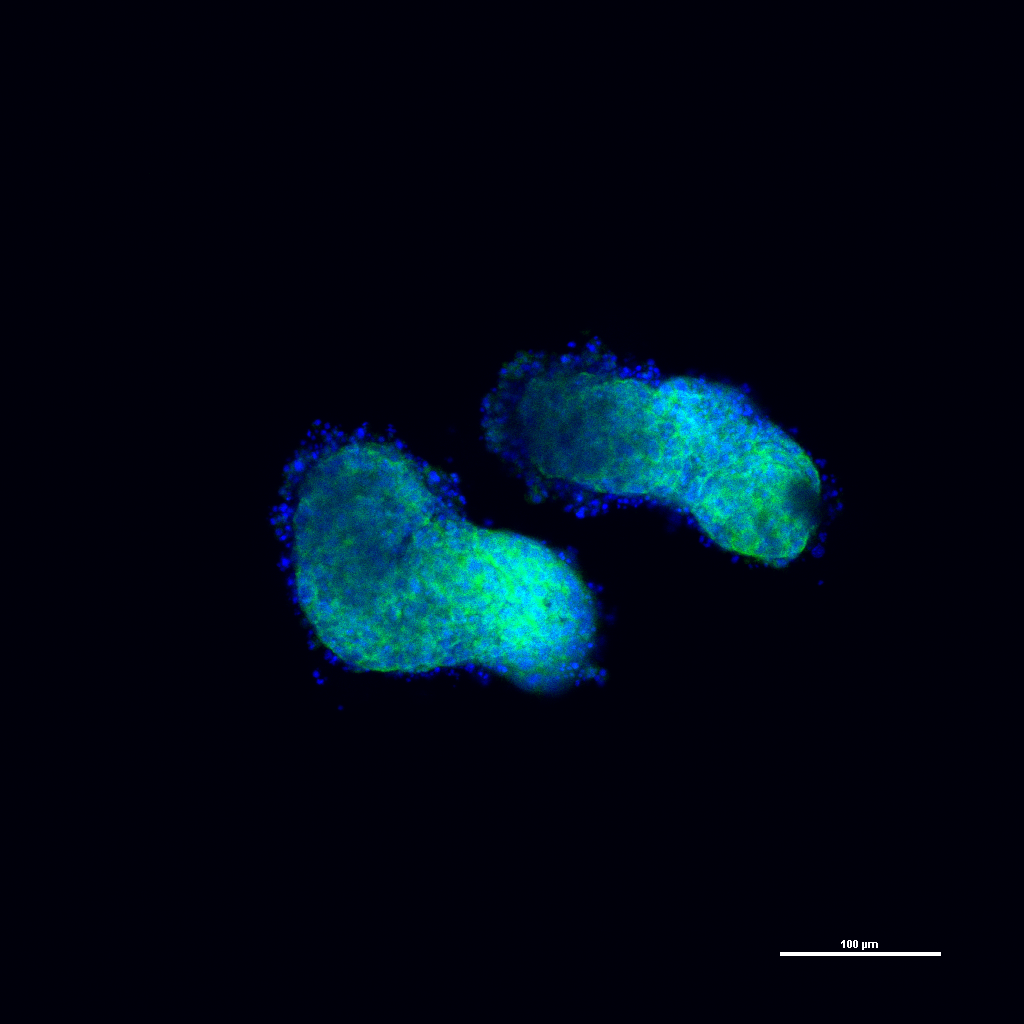

Supplement: Supplementary file 5 — Source data Fig. 3 [file 44318_2025_558_MOESM5_ESM.zip › Figure 3/panel 3C/NT-e-cadh/seq9124_seq9124_RGB.tif]

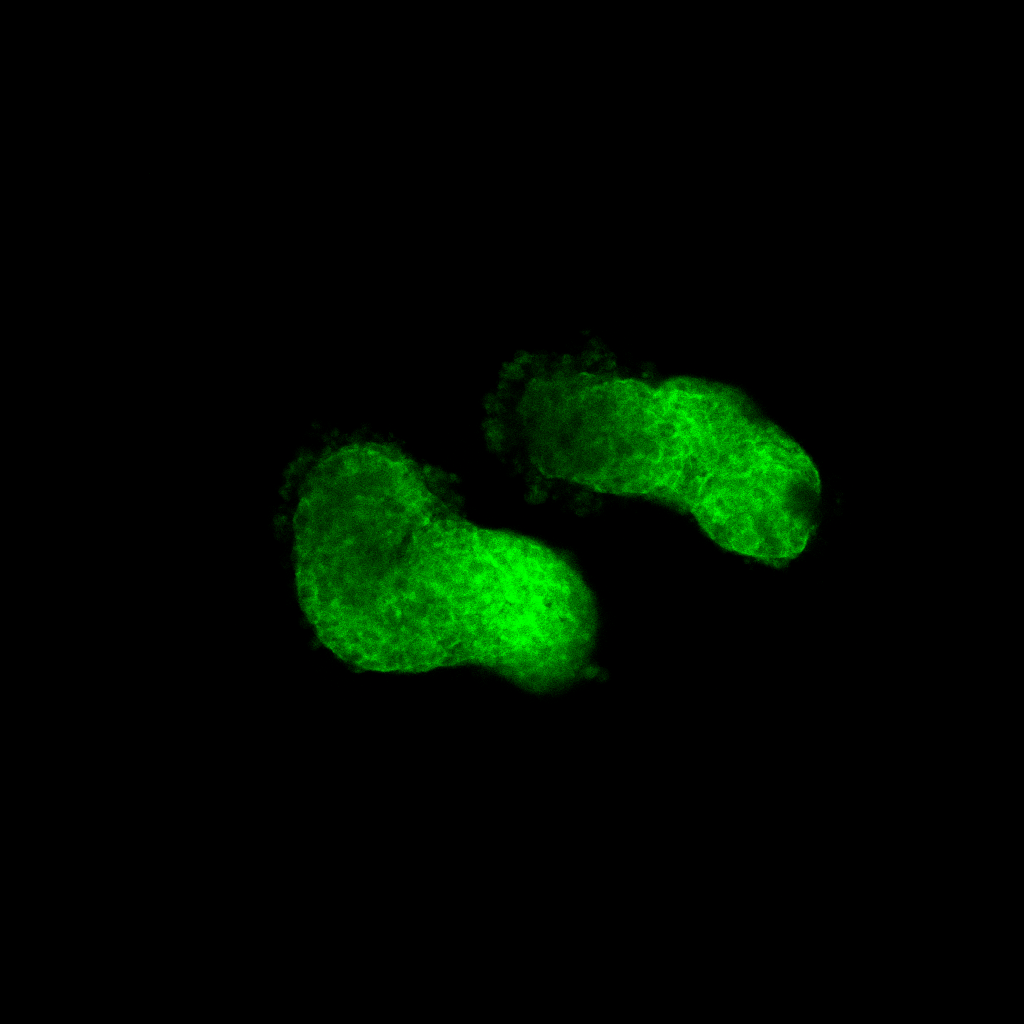

Supplement: Supplementary file 5 — Source data Fig. 3 [file 44318_2025_558_MOESM5_ESM.zip › Figure 3/panel 3C/NT-e-cadh/seq9124_seq9124_RGB_FITC.tif]

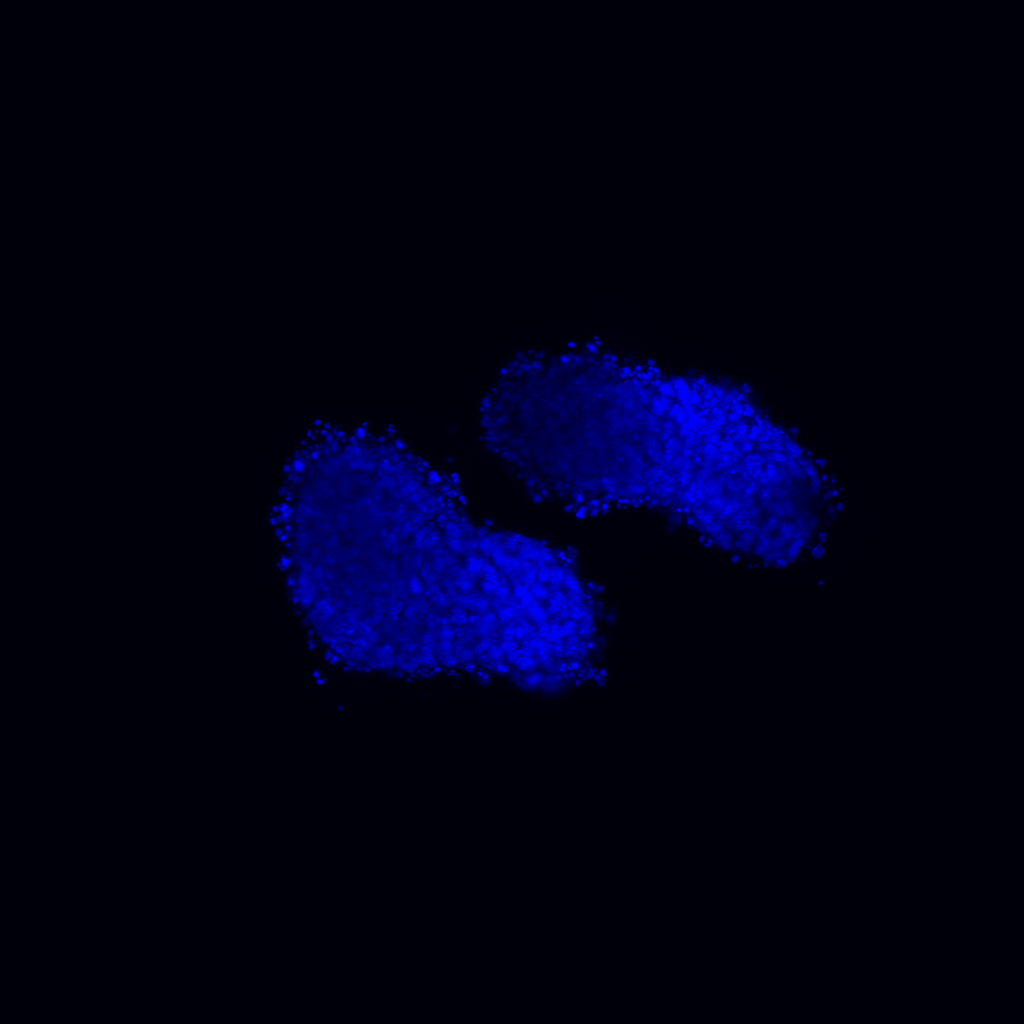

Supplement: Supplementary file 5 — Source data Fig. 3 [file 44318_2025_558_MOESM5_ESM.zip › Figure 3/panel 3C/NT-e-cadh/seq9124_seq9124_RGB_DAPI.tif]

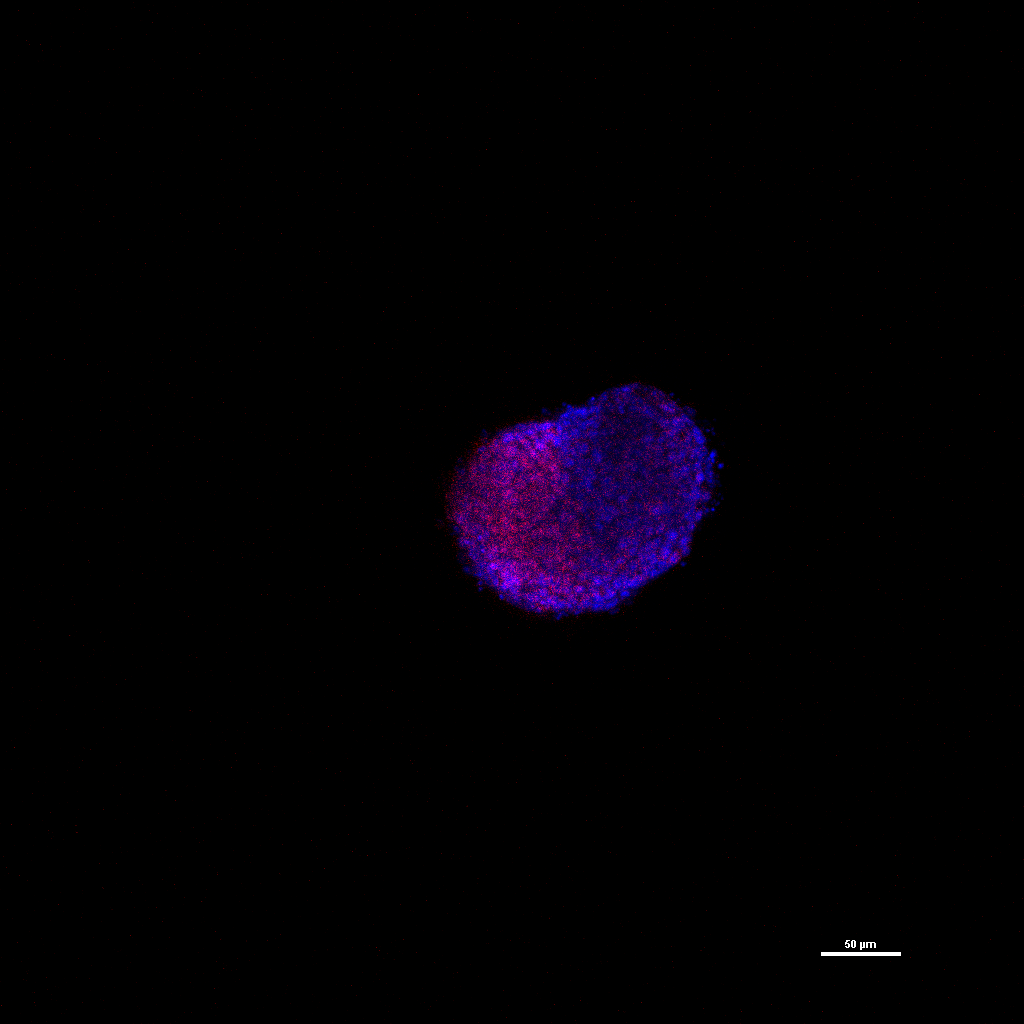

Supplement: Supplementary file 5 — Source data Fig. 3 [file 44318_2025_558_MOESM5_ESM.zip › Figure 3/panel 3C/KD-1_Cdx2/image0138_image0138_RGB.tif]

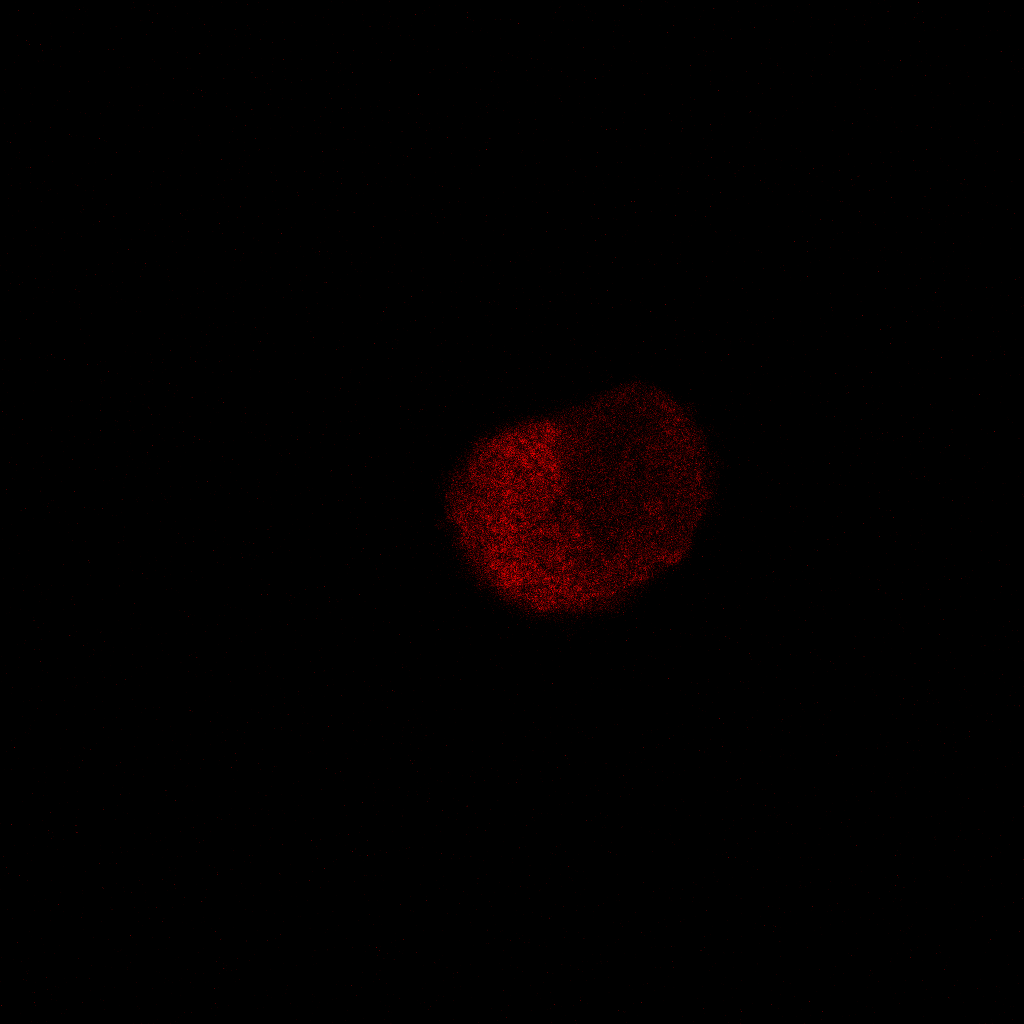

Supplement: Supplementary file 5 — Source data Fig. 3 [file 44318_2025_558_MOESM5_ESM.zip › Figure 3/panel 3C/KD-1_Cdx2/image0138_image0138_RGB_Texas Red.tif]

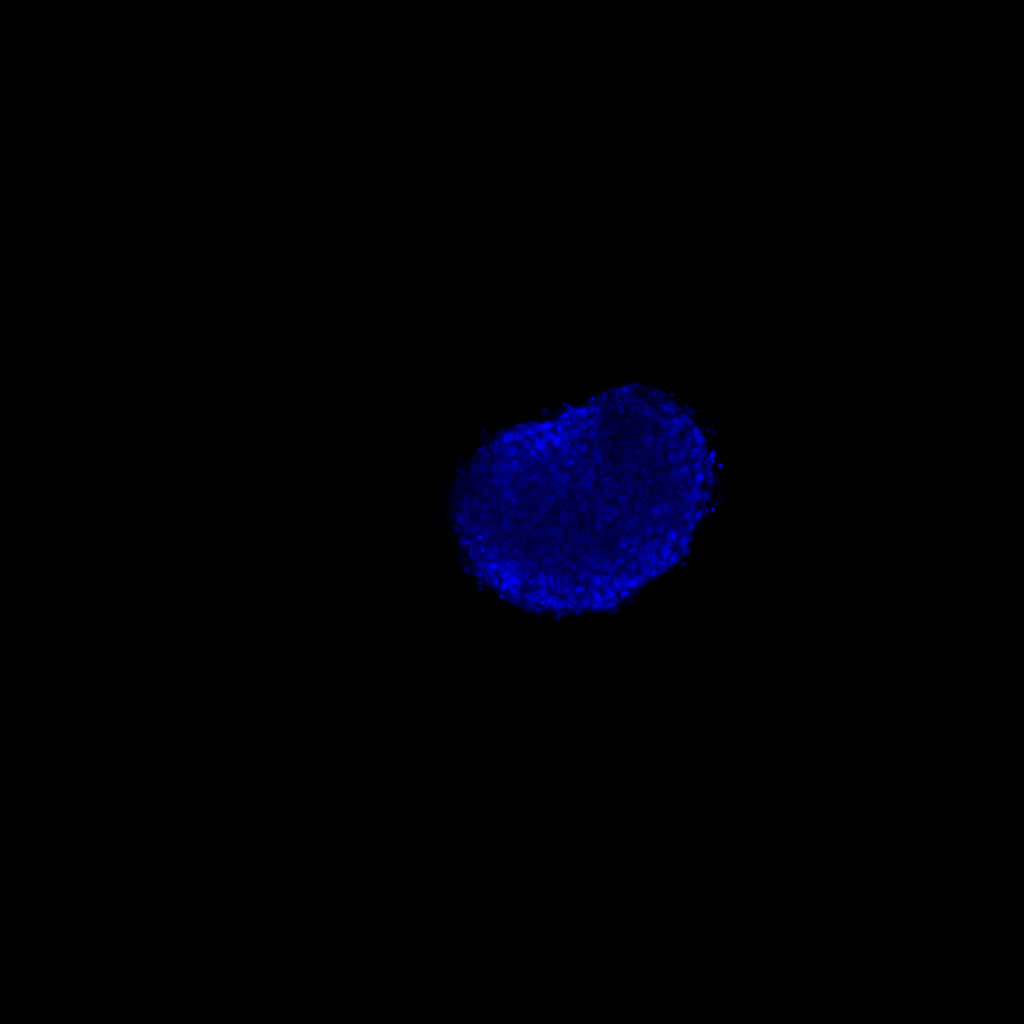

Supplement: Supplementary file 5 — Source data Fig. 3 [file 44318_2025_558_MOESM5_ESM.zip › Figure 3/panel 3C/KD-1_Cdx2/image0138_image0138_RGB_DAPI.tif]

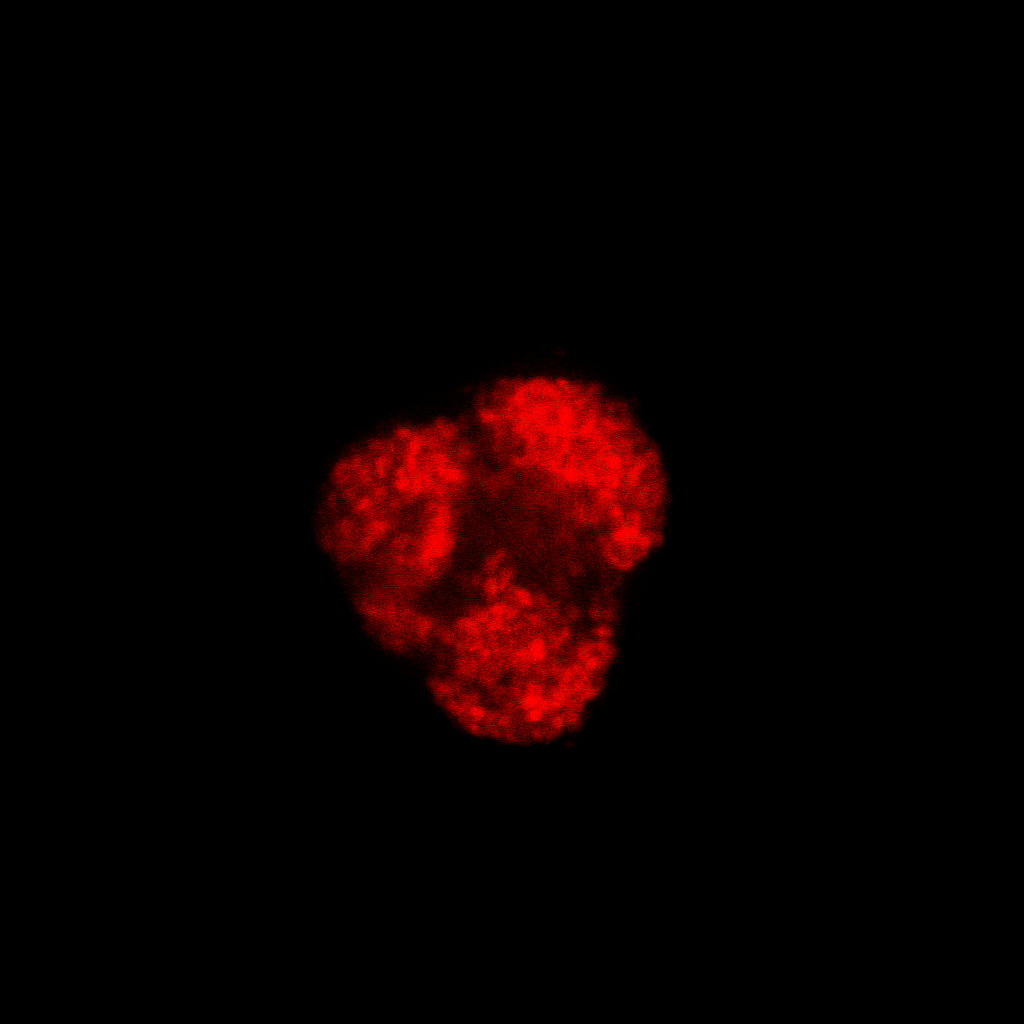

Supplement: Supplementary file 5 — Source data Fig. 3 [file 44318_2025_558_MOESM5_ESM.zip › Figure 3/panel 3C/KD-1_Sox2/seq11145_seq11145_RGB_TRITC.tif]

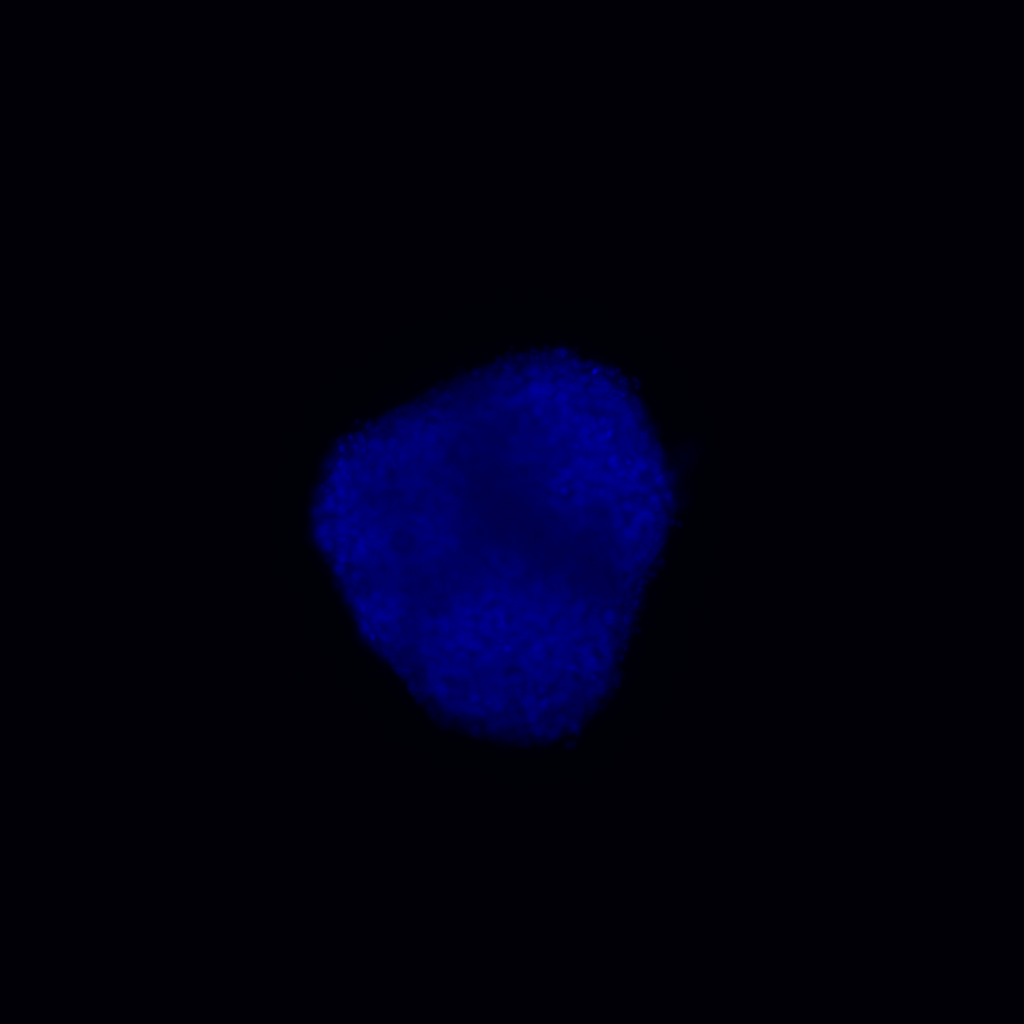

Supplement: Supplementary file 5 — Source data Fig. 3 [file 44318_2025_558_MOESM5_ESM.zip › Figure 3/panel 3C/KD-1_Sox2/seq11145_seq11145_RGB_DAPI.tif]

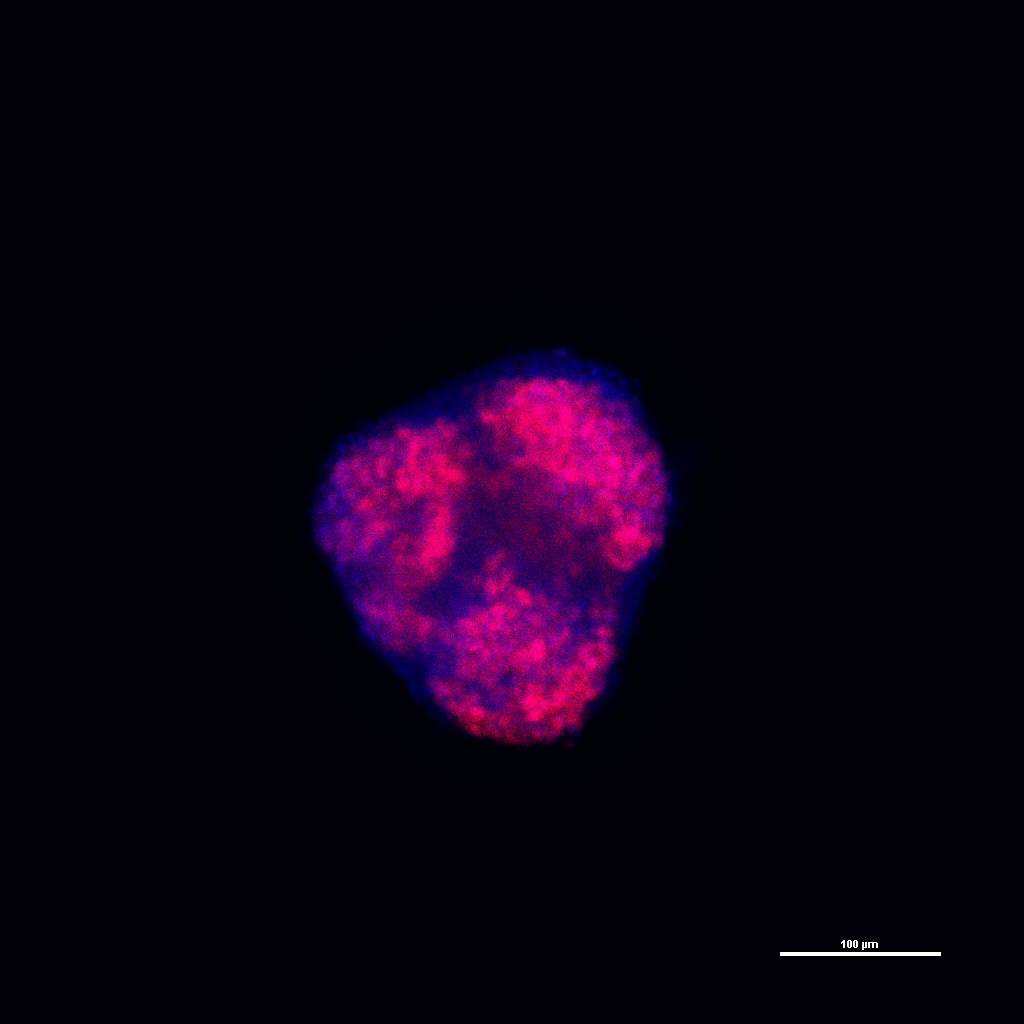

Supplement: Supplementary file 5 — Source data Fig. 3 [file 44318_2025_558_MOESM5_ESM.zip › Figure 3/panel 3C/KD-1_Sox2/seq11145_seq11145_RGB.tif]

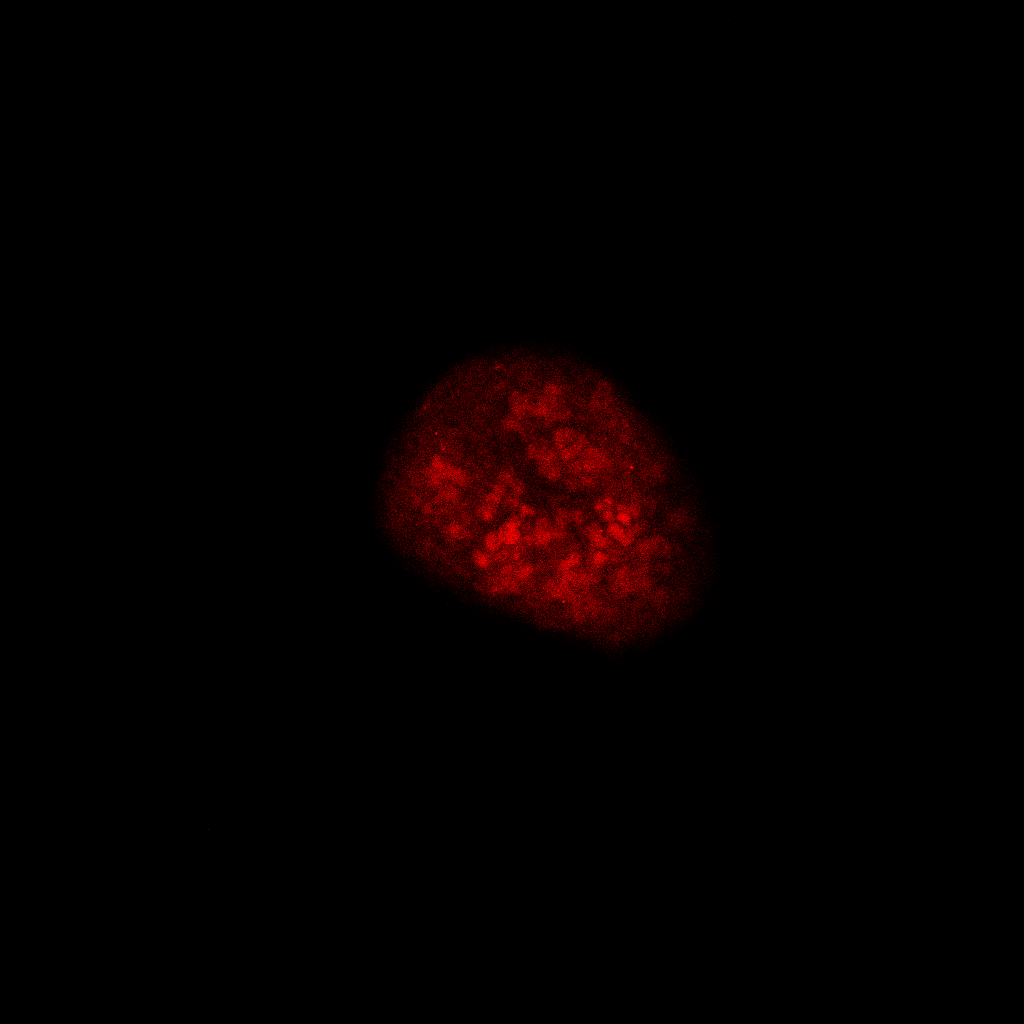

Supplement: Supplementary file 5 — Source data Fig. 3 [file 44318_2025_558_MOESM5_ESM.zip › Figure 3/panel 3C/KD-1_Oct4/seq8976_seq8976_RGB_Texas Red.tif]

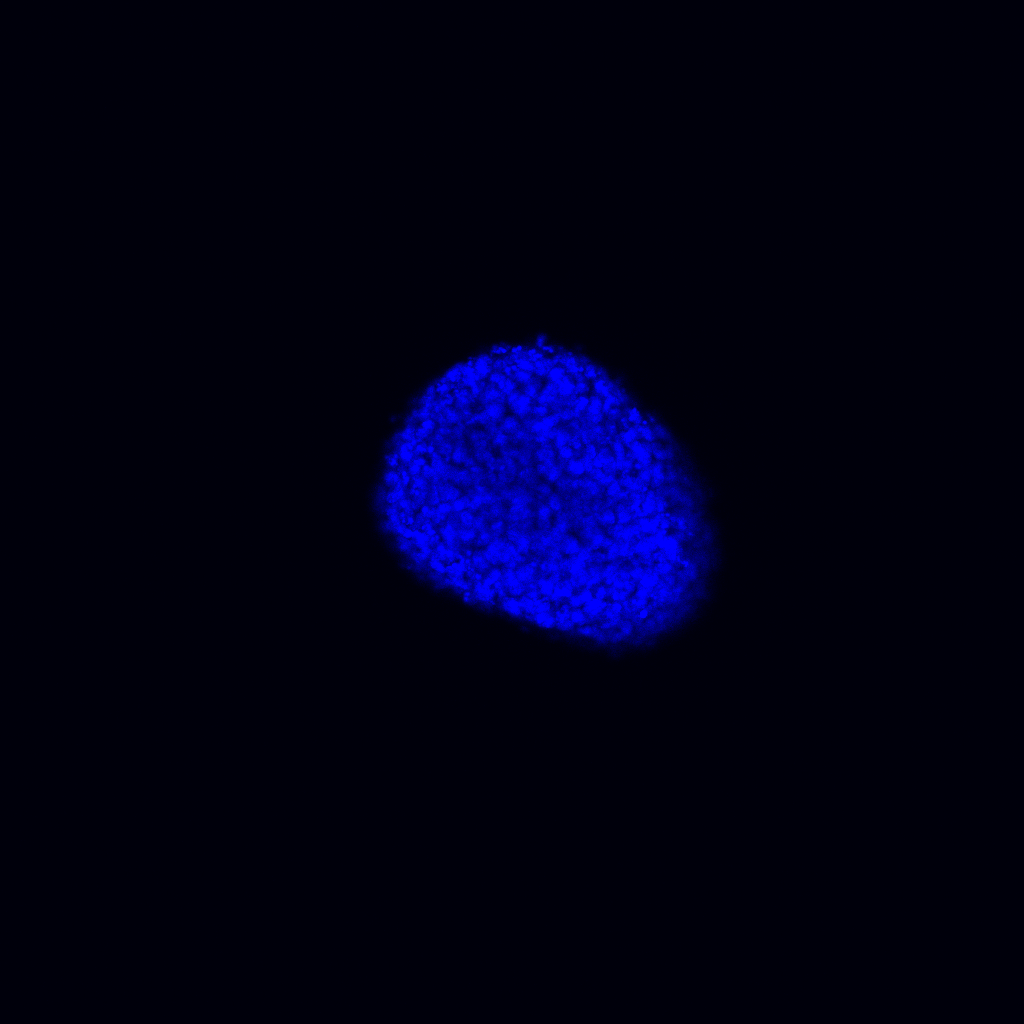

Supplement: Supplementary file 5 — Source data Fig. 3 [file 44318_2025_558_MOESM5_ESM.zip › Figure 3/panel 3C/KD-1_Oct4/seq8976_seq8976_RGB_DAPI.tif]

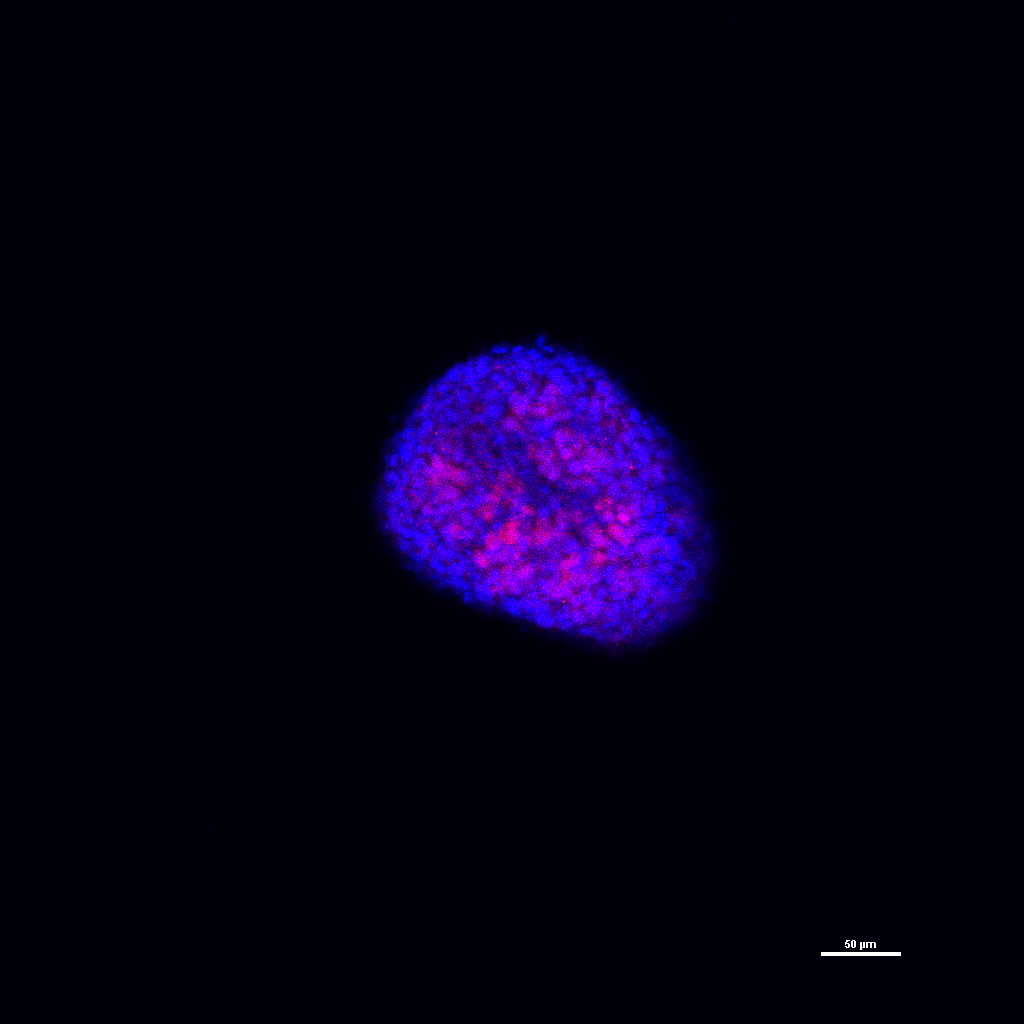

Supplement: Supplementary file 5 — Source data Fig. 3 [file 44318_2025_558_MOESM5_ESM.zip › Figure 3/panel 3C/KD-1_Oct4/seq8976_seq8976_RGB.tif]

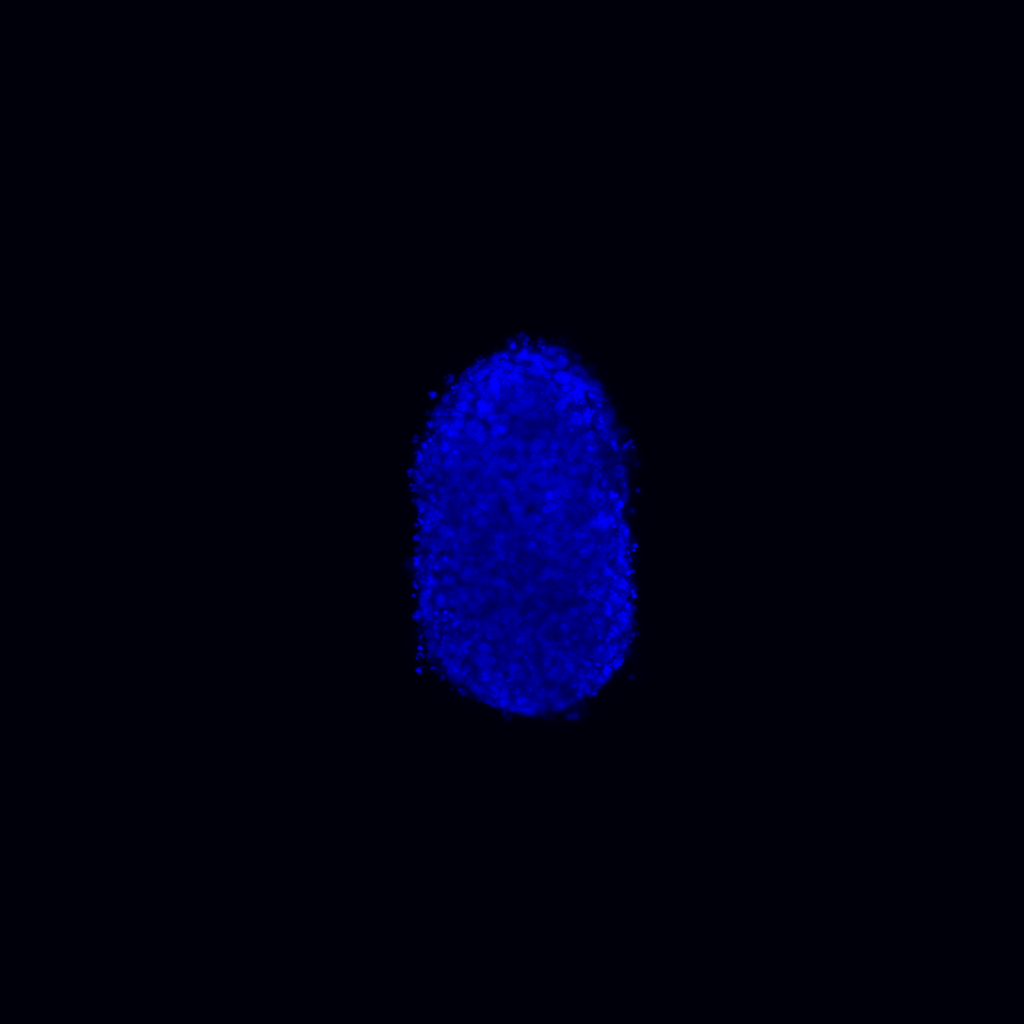

Supplement: Supplementary file 5 — Source data Fig. 3 [file 44318_2025_558_MOESM5_ESM.zip › Figure 3/panel 3C/KD-1_e-cadh/seq9121_seq9121_RGB_DAPI.tif]

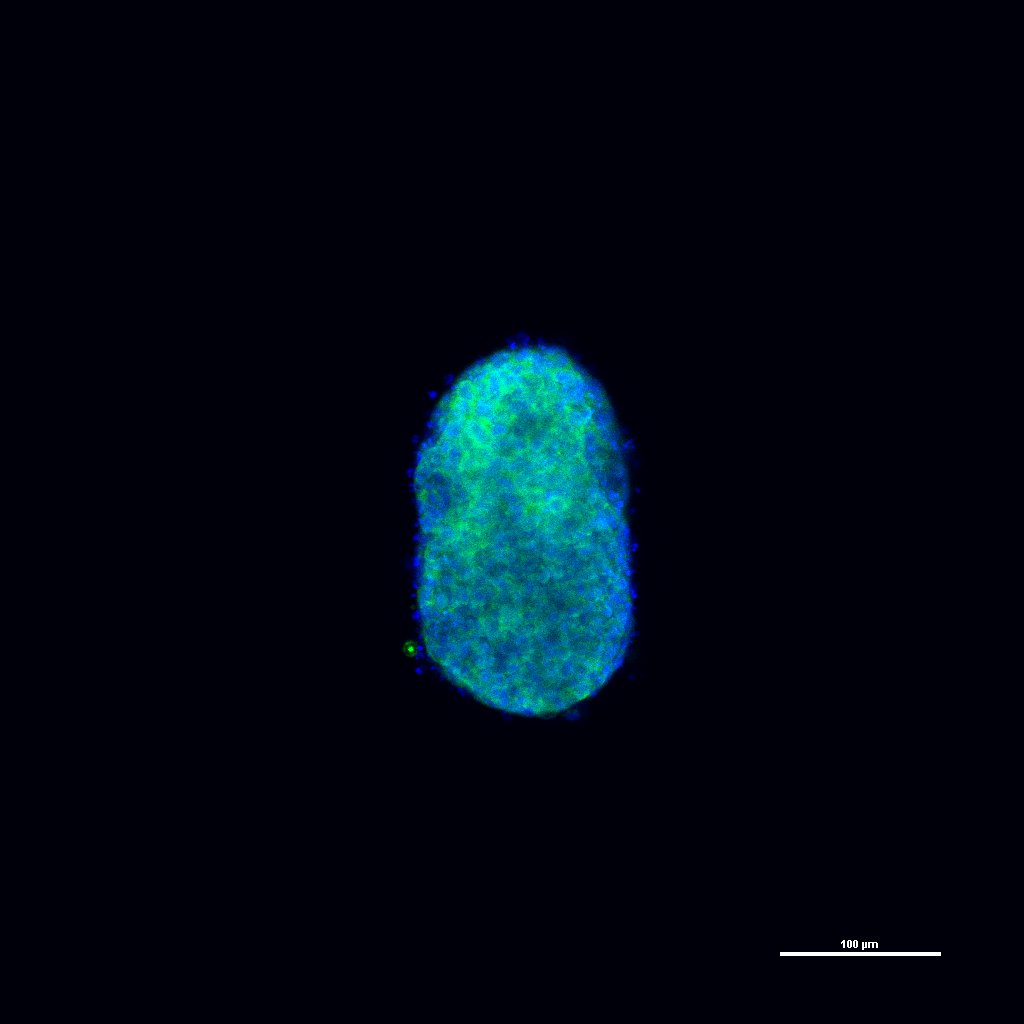

Supplement: Supplementary file 5 — Source data Fig. 3 [file 44318_2025_558_MOESM5_ESM.zip › Figure 3/panel 3C/KD-1_e-cadh/seq9121_seq9121_RGB.tif]

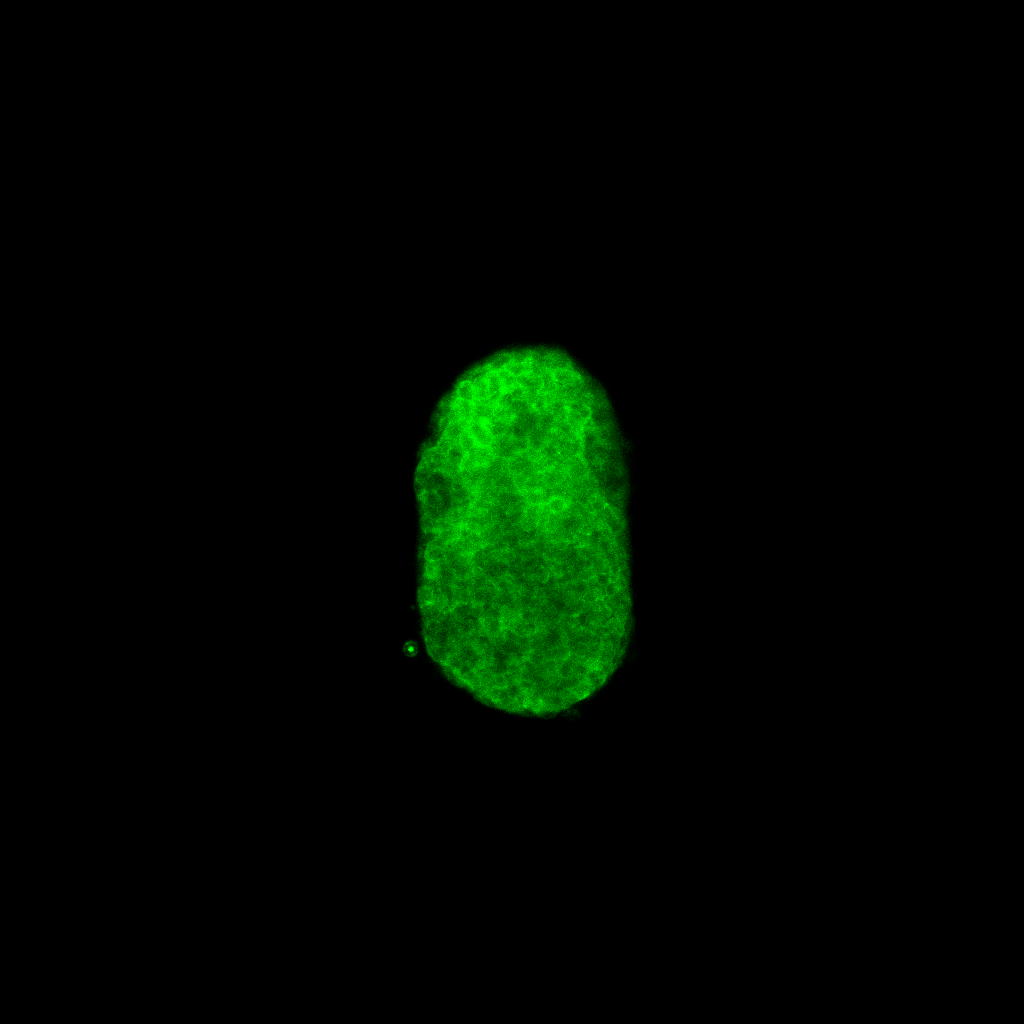

Supplement: Supplementary file 5 — Source data Fig. 3 [file 44318_2025_558_MOESM5_ESM.zip › Figure 3/panel 3C/KD-1_e-cadh/seq9121_seq9121_RGB_FITC.tif]

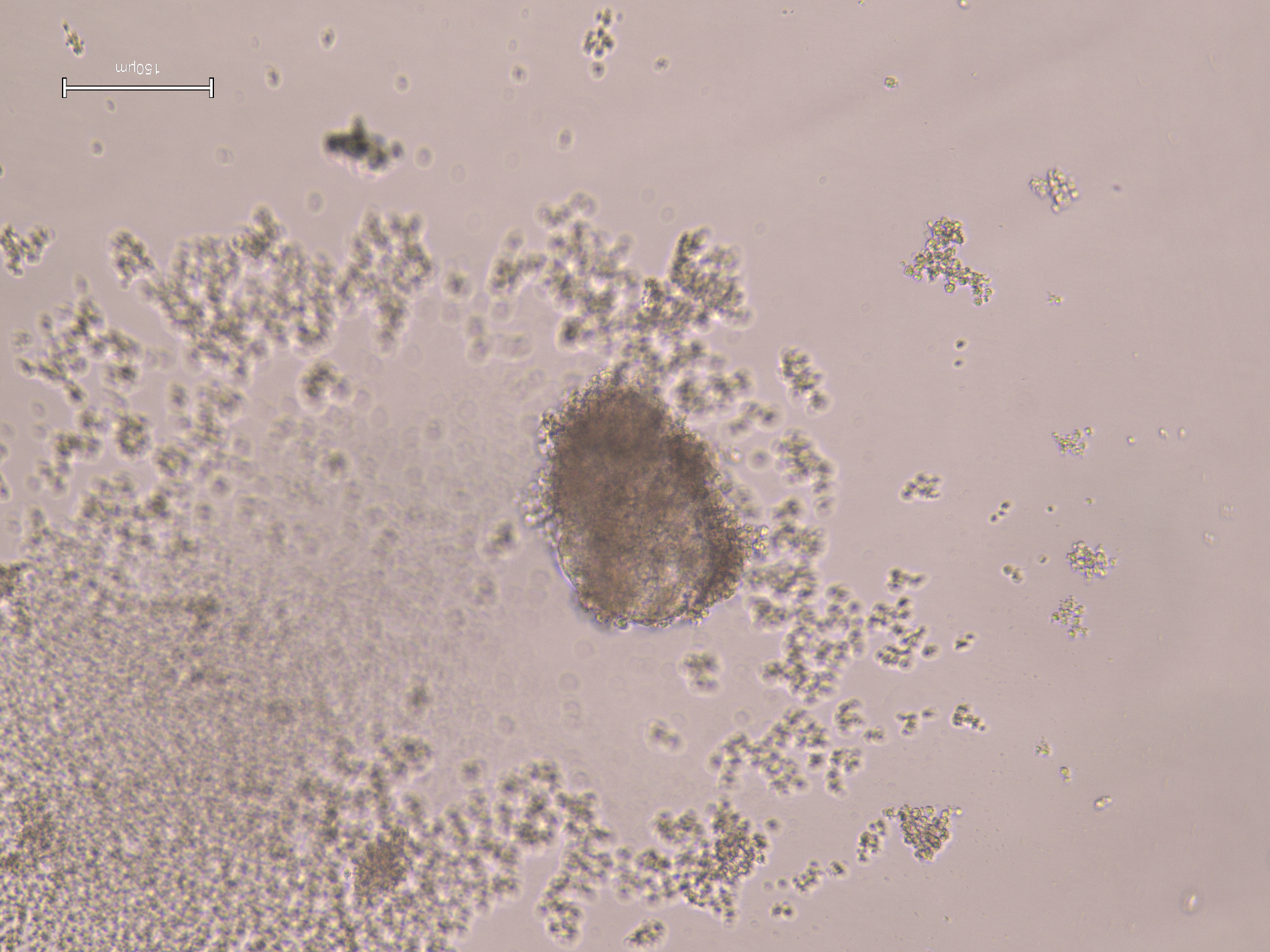

Supplement: Supplementary file 6 — Source data Fig. 4 [file 44318_2025_558_MOESM6_ESM.zip › Figure 4/panel 4A/KD-1_6uM.tiff]

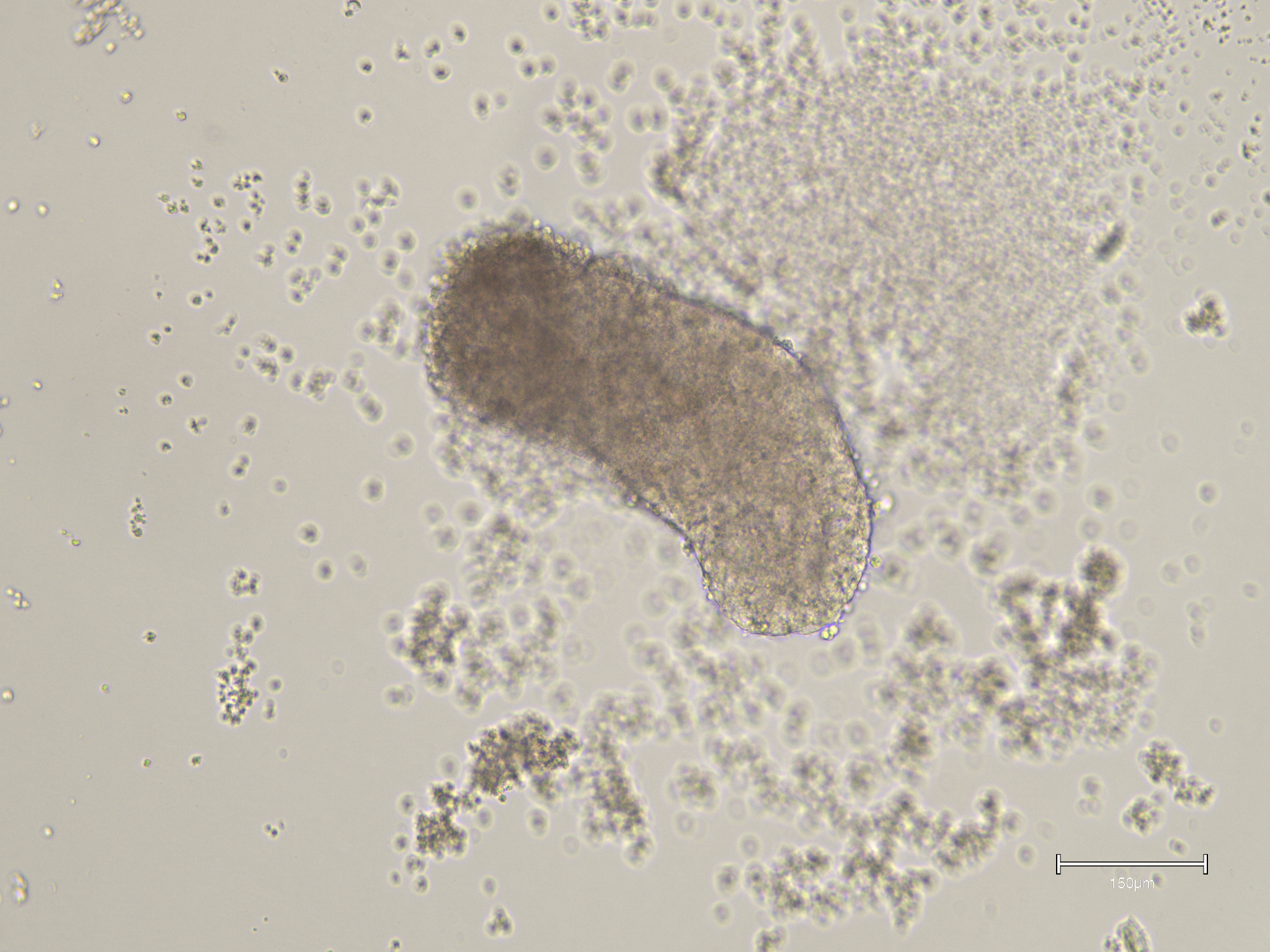

Supplement: Supplementary file 6 — Source data Fig. 4 [file 44318_2025_558_MOESM6_ESM.zip › Figure 4/panel 4A/NT_3uM.tiff]

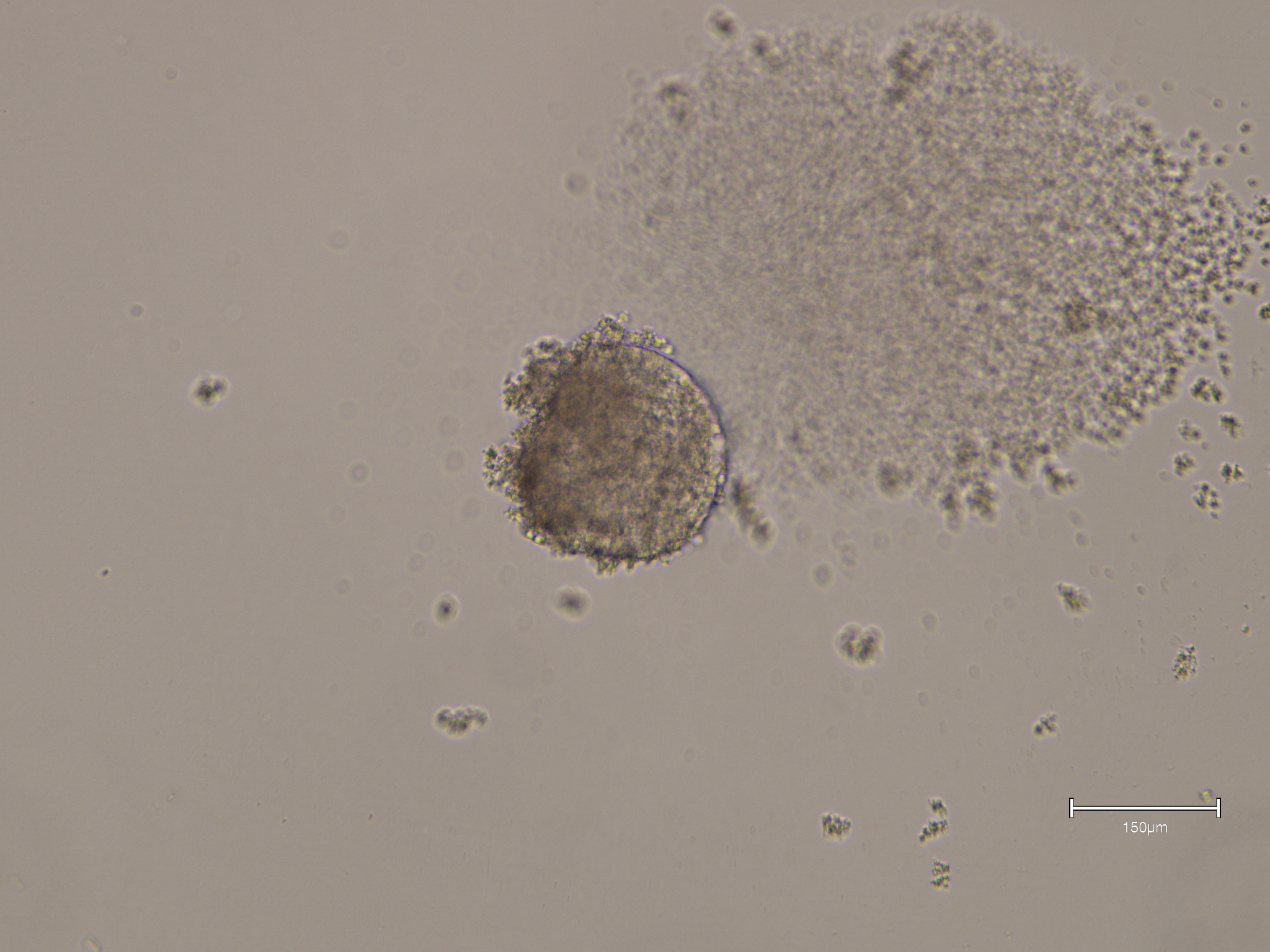

Supplement: Supplementary file 6 — Source data Fig. 4 [file 44318_2025_558_MOESM6_ESM.zip › Figure 4/panel 4A/KD-2_5uM.tiff]

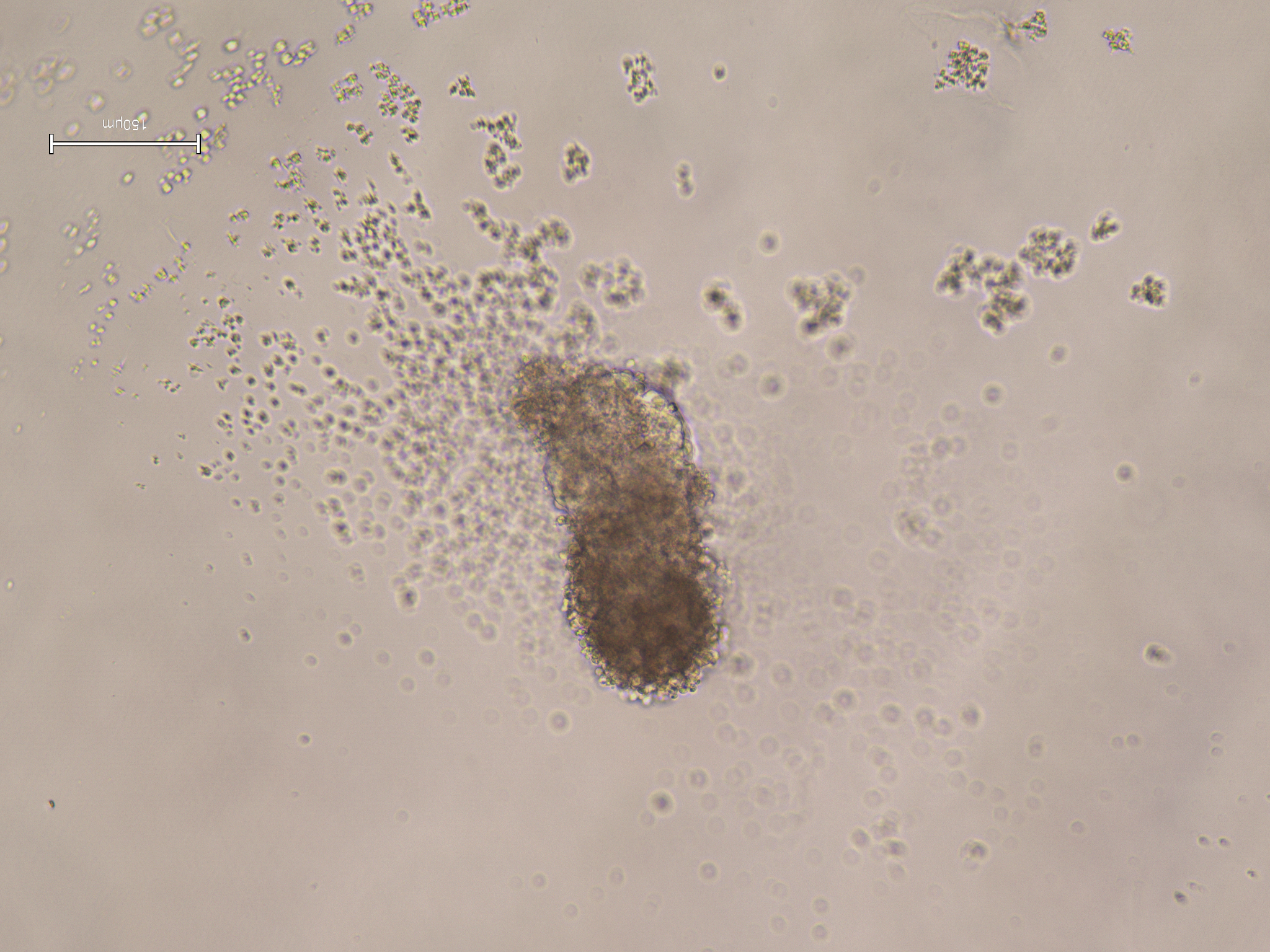

Supplement: Supplementary file 6 — Source data Fig. 4 [file 44318_2025_558_MOESM6_ESM.zip › Figure 4/panel 4A/KD-1_4uM.tiff]

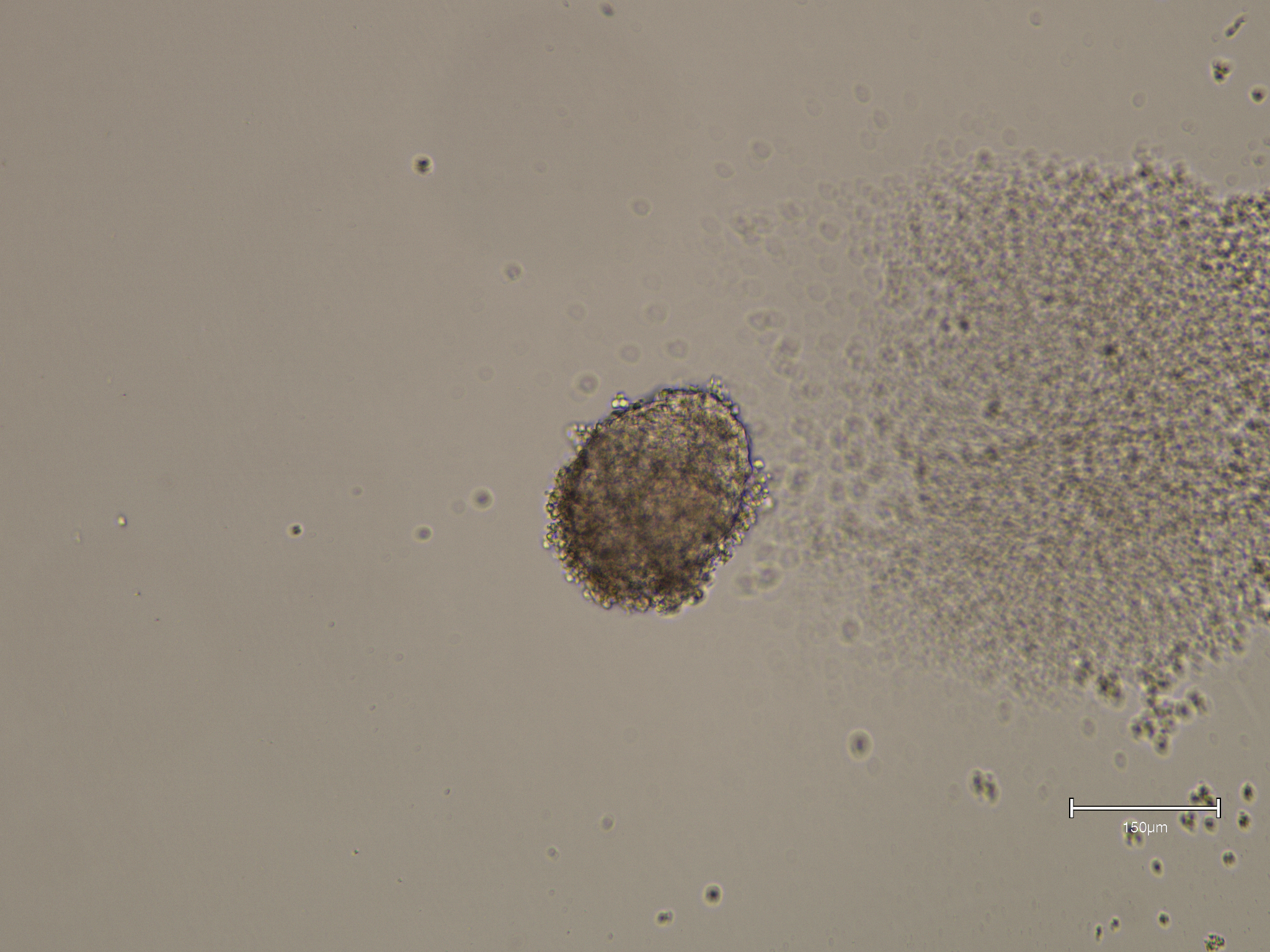

Supplement: Supplementary file 6 — Source data Fig. 4 [file 44318_2025_558_MOESM6_ESM.zip › Figure 4/panel 4A/KD-2_3uM.tiff]

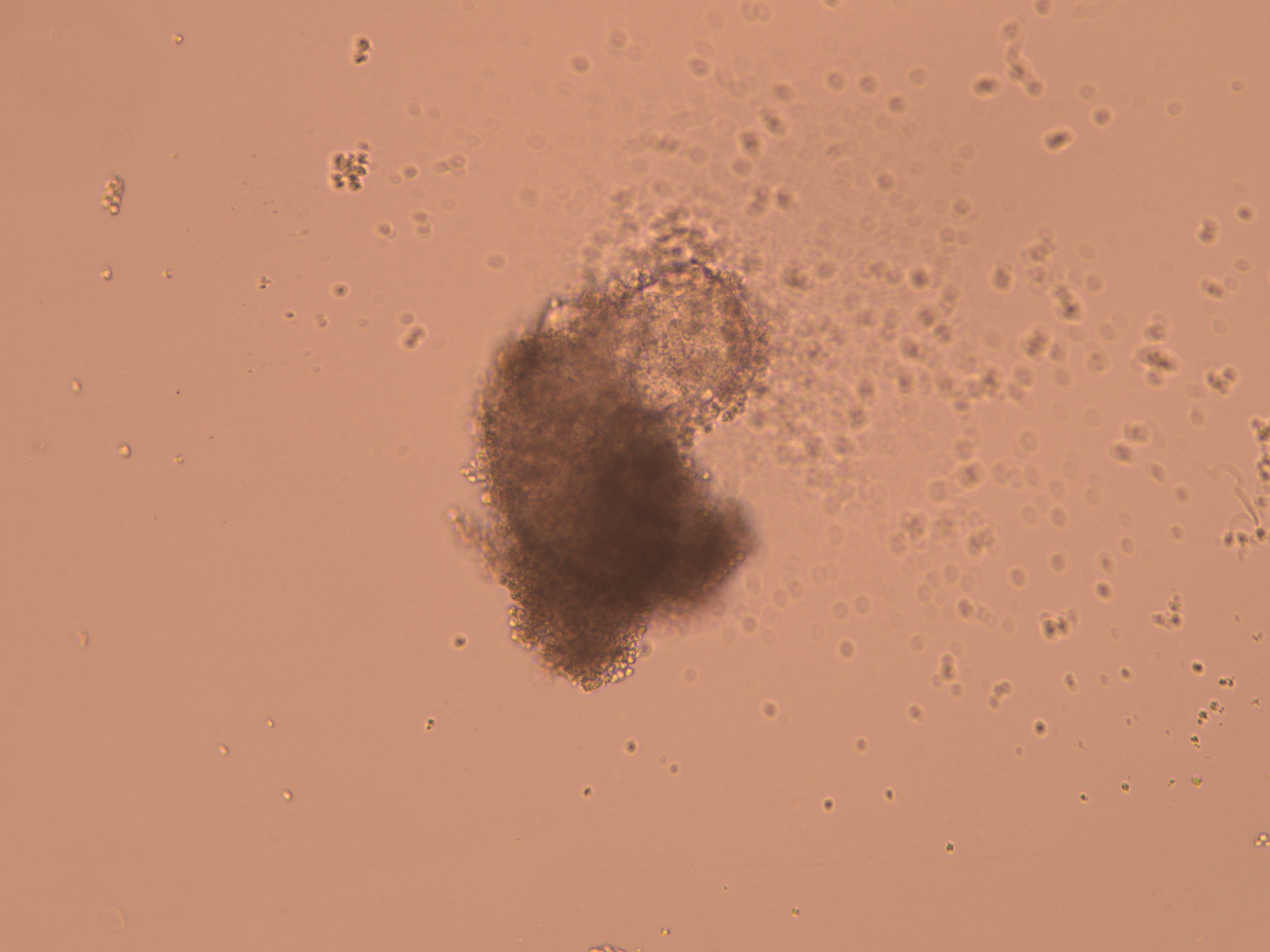

Supplement: Supplementary file 6 — Source data Fig. 4 [file 44318_2025_558_MOESM6_ESM.zip › Figure 4/panel 4A/NT_5uM.tiff]

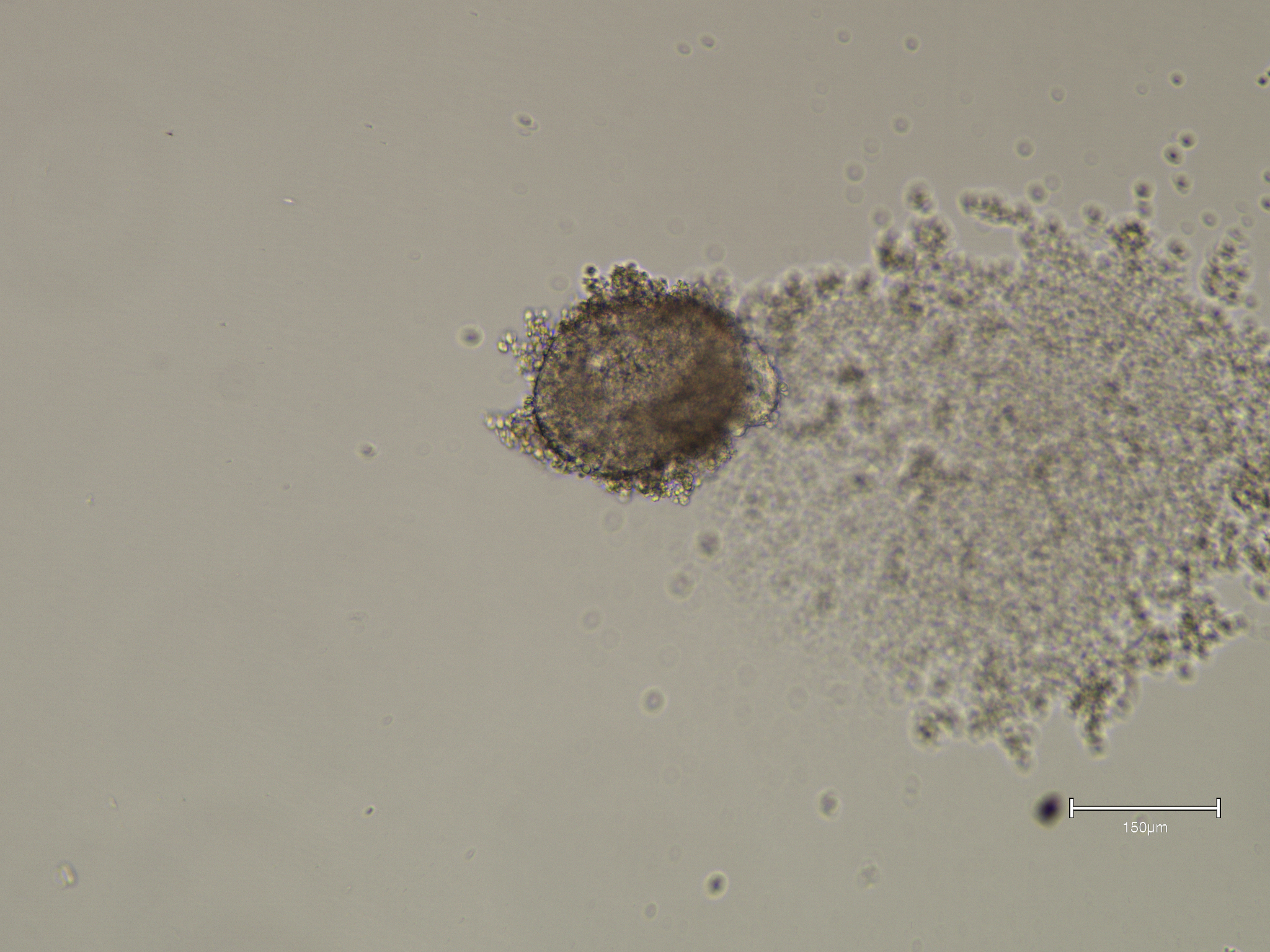

Supplement: Supplementary file 6 — Source data Fig. 4 [file 44318_2025_558_MOESM6_ESM.zip › Figure 4/panel 4A/KD-2_4uM.tiff]

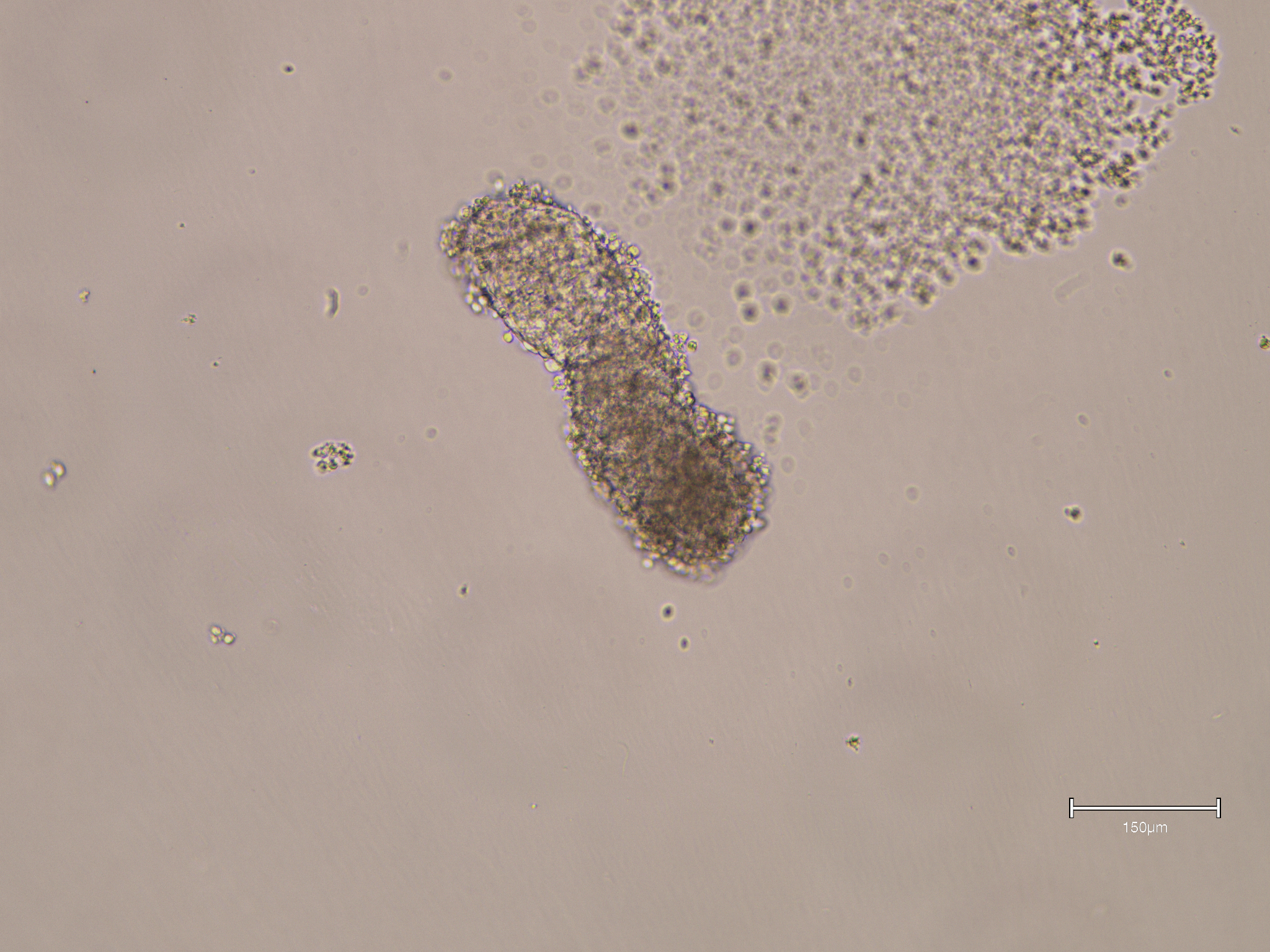

Supplement: Supplementary file 6 — Source data Fig. 4 [file 44318_2025_558_MOESM6_ESM.zip › Figure 4/panel 4A/KD-1_5uM.tiff]

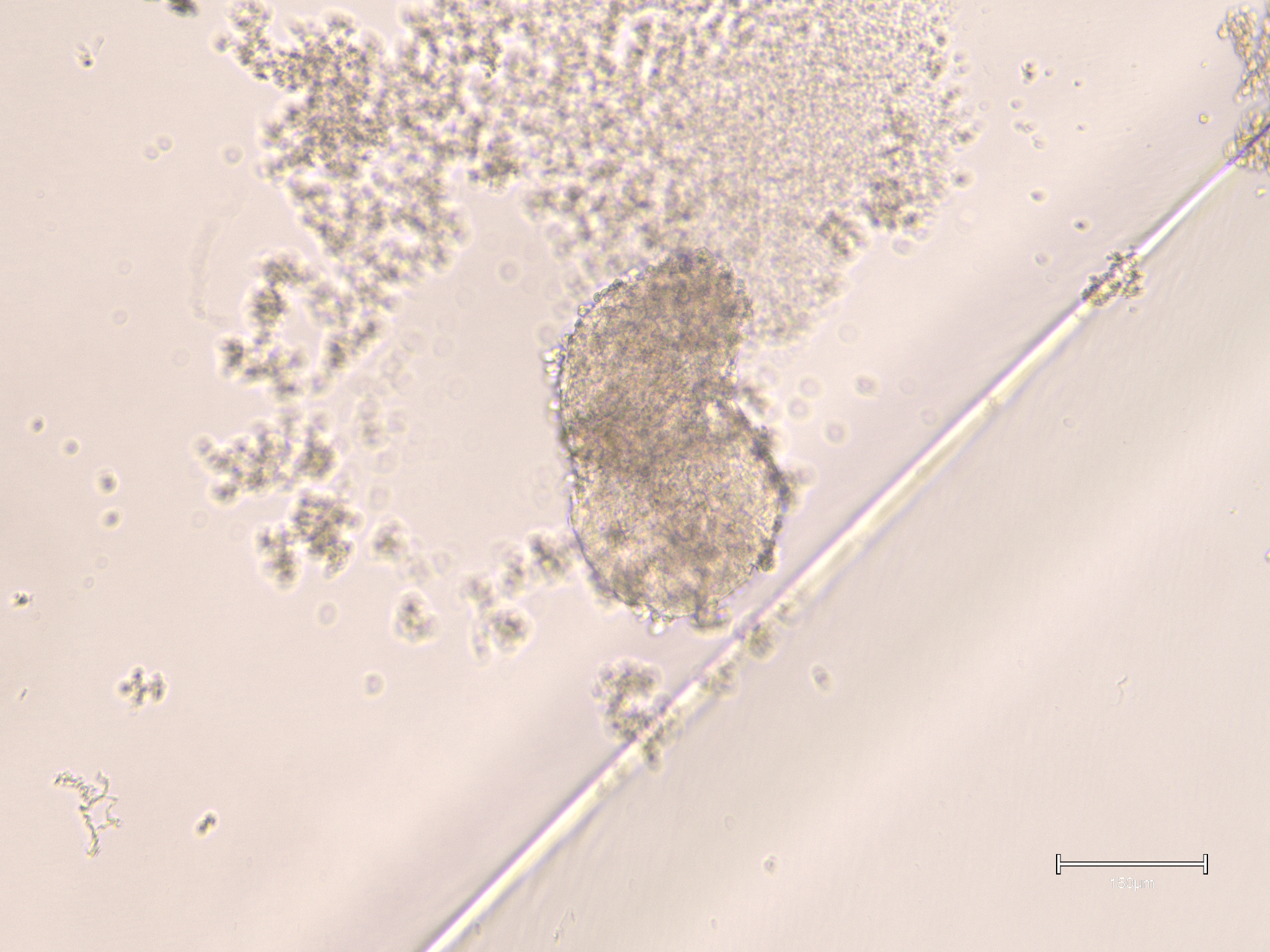

Supplement: Supplementary file 6 — Source data Fig. 4 [file 44318_2025_558_MOESM6_ESM.zip › Figure 4/panel 4A/KD-2_6uM.tiff]

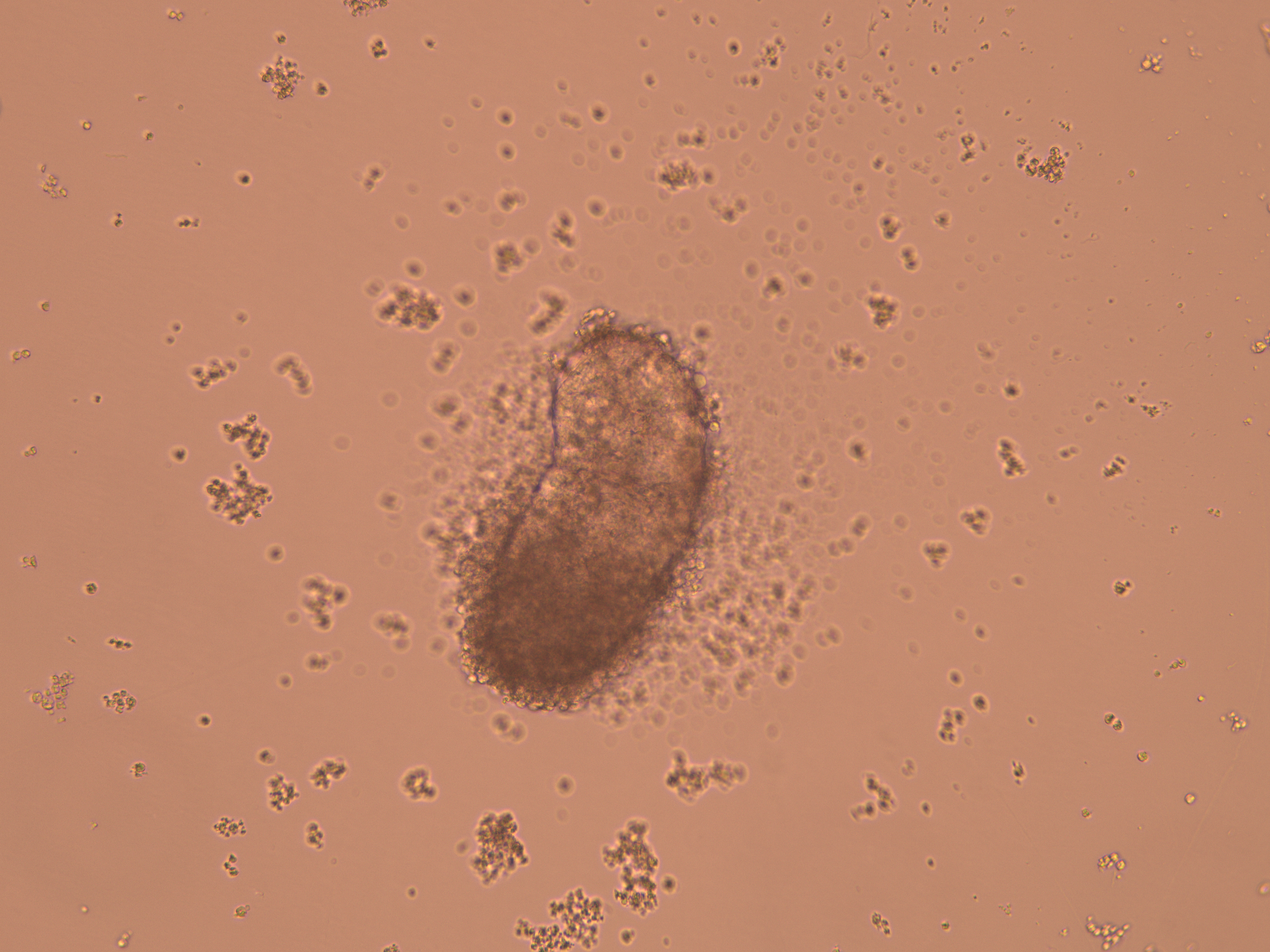

Supplement: Supplementary file 6 — Source data Fig. 4 [file 44318_2025_558_MOESM6_ESM.zip › Figure 4/panel 4A/NT_4uM.tiff]

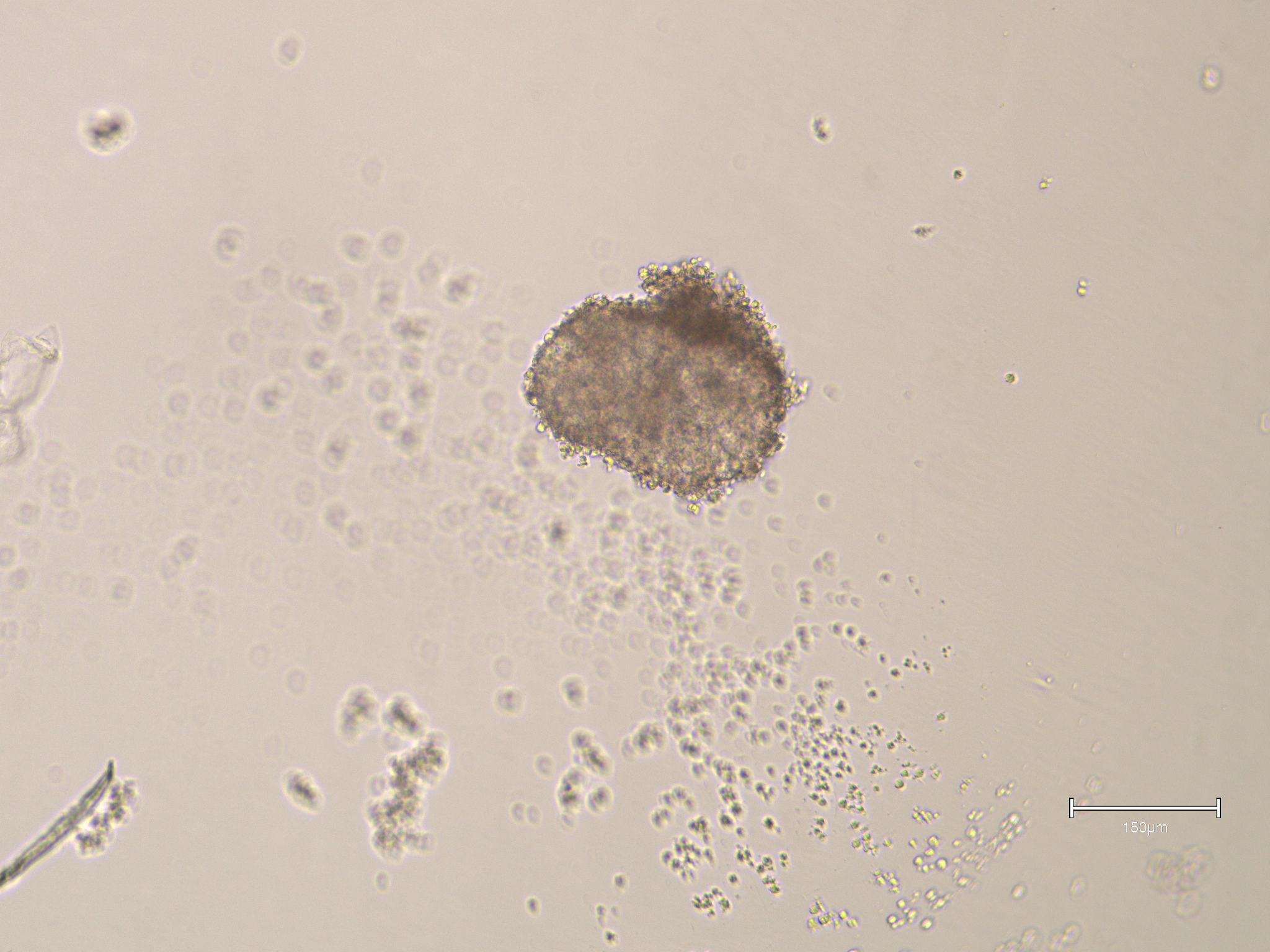

Supplement: Supplementary file 6 — Source data Fig. 4 [file 44318_2025_558_MOESM6_ESM.zip › Figure 4/panel 4A/KD-1_3uM.tiff]

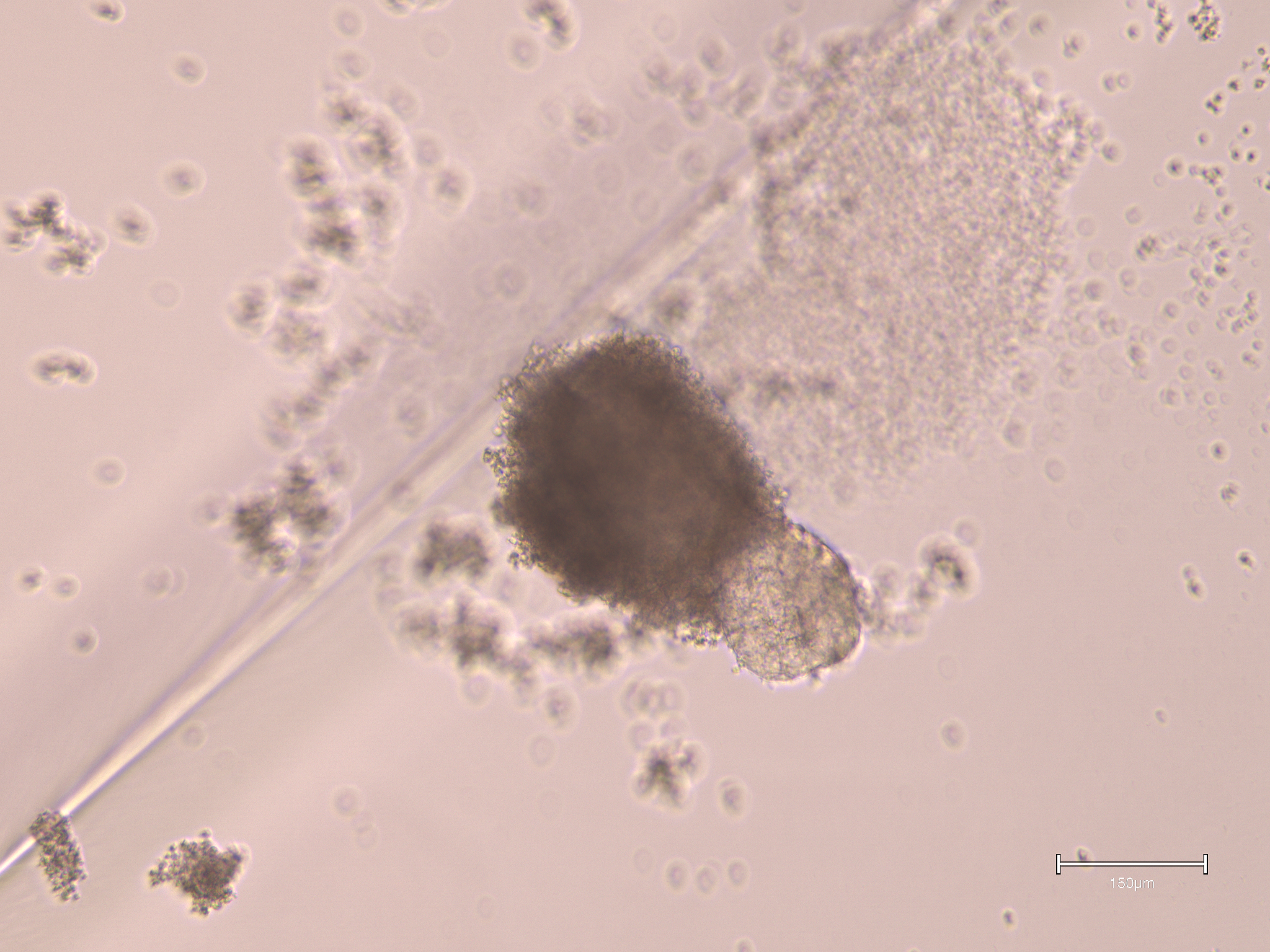

Supplement: Supplementary file 6 — Source data Fig. 4 [file 44318_2025_558_MOESM6_ESM.zip › Figure 4/panel 4A/NT_6uM.tiff]

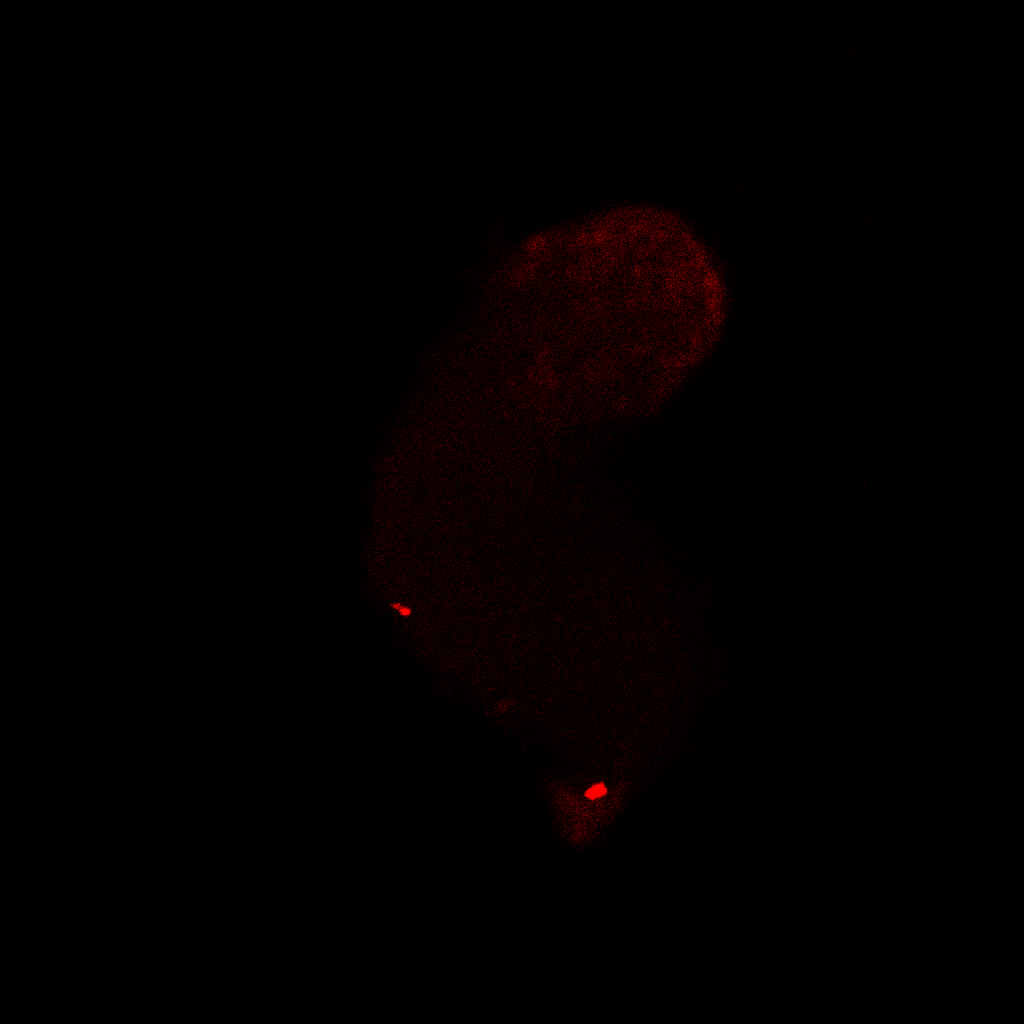

Supplement: Supplementary file 6 — Source data Fig. 4 [file 44318_2025_558_MOESM6_ESM.zip › Figure 4/panel 4B/NT_Cdx2_3uM/seq9877_seq9877_RGB_TRITC.tif]

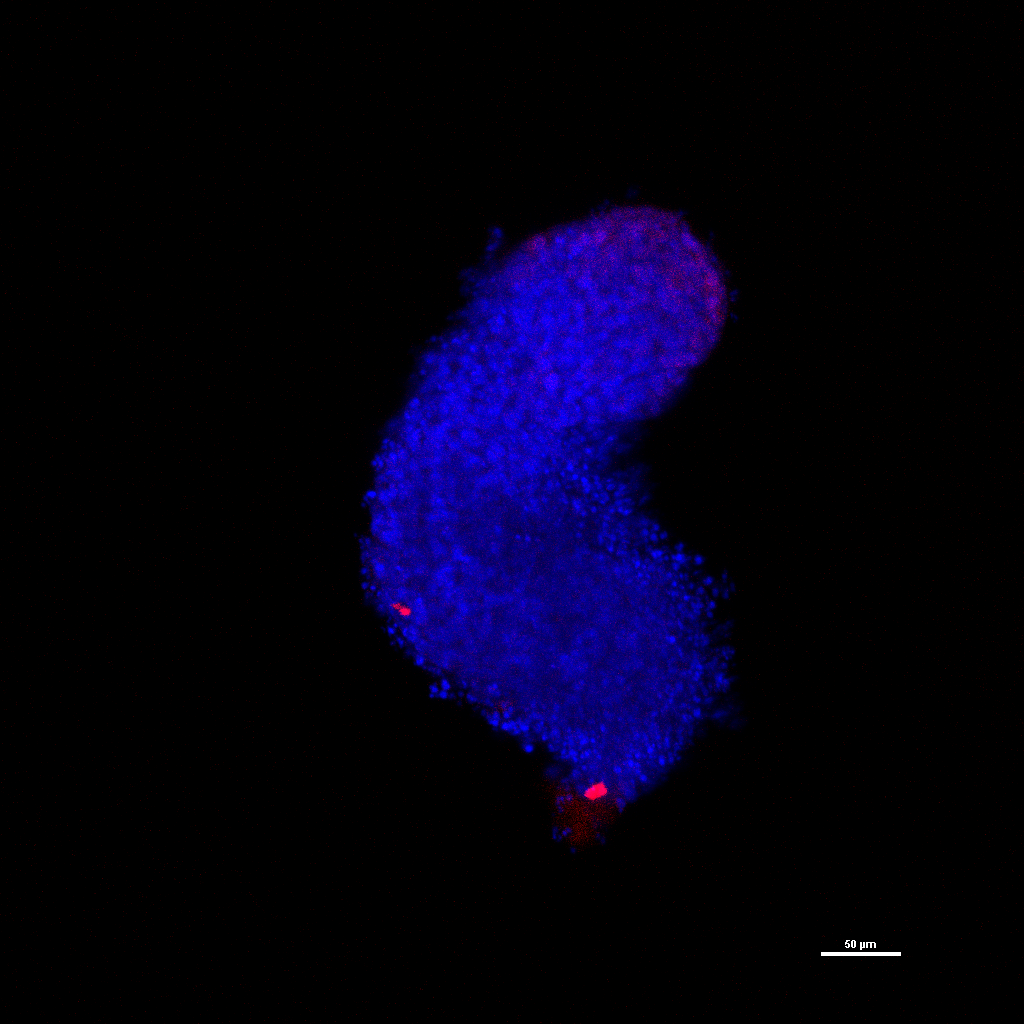

Supplement: Supplementary file 6 — Source data Fig. 4 [file 44318_2025_558_MOESM6_ESM.zip › Figure 4/panel 4B/NT_Cdx2_3uM/seq9877_seq9877_RGB.tif]

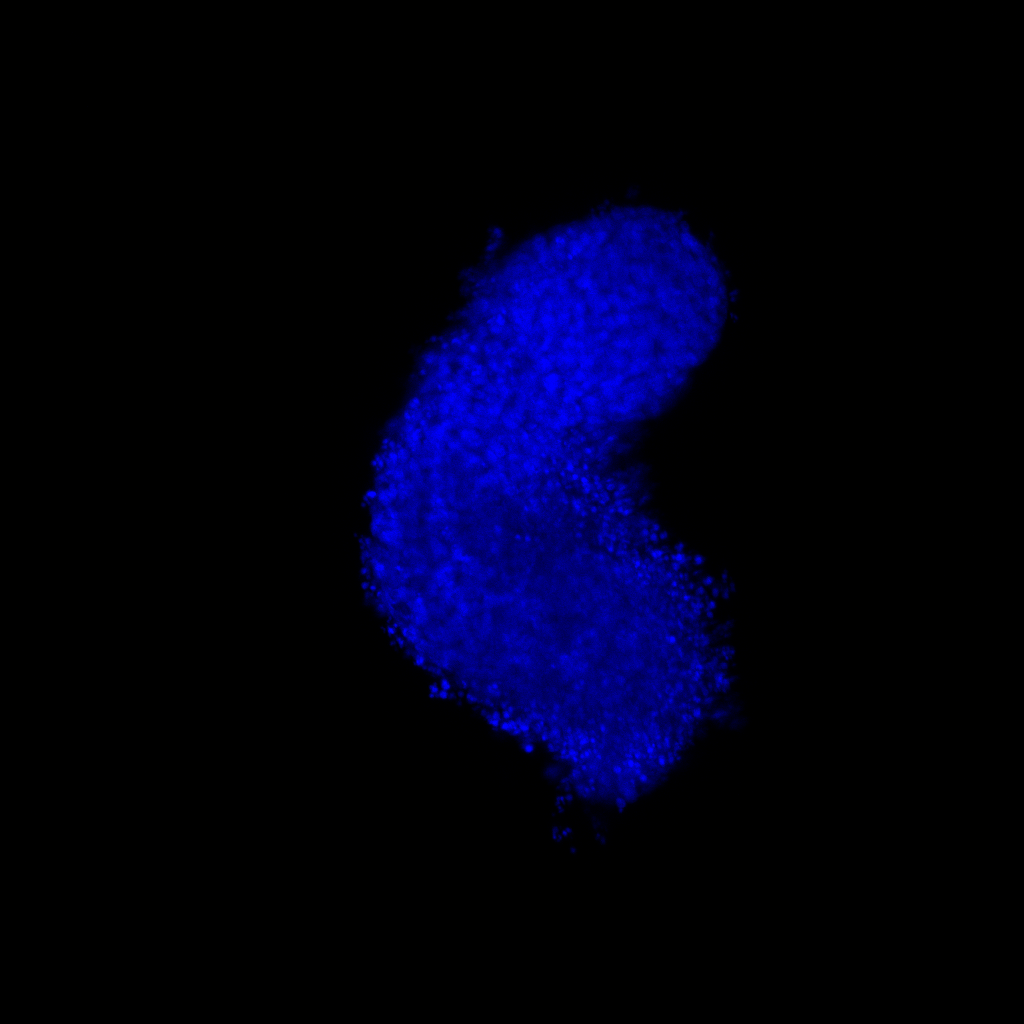

Supplement: Supplementary file 6 — Source data Fig. 4 [file 44318_2025_558_MOESM6_ESM.zip › Figure 4/panel 4B/NT_Cdx2_3uM/seq9877_seq9877_RGB_DAPI.tif]

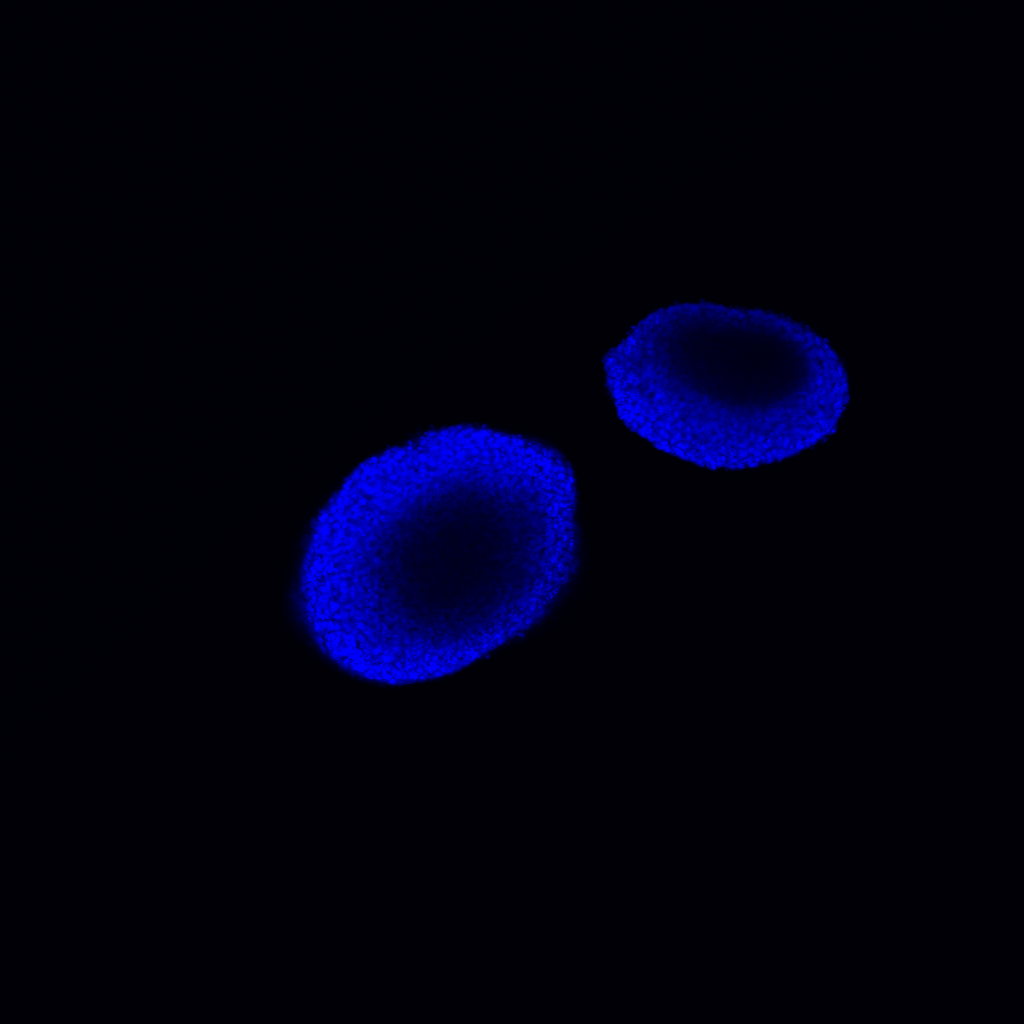

Supplement: Supplementary file 6 — Source data Fig. 4 [file 44318_2025_558_MOESM6_ESM.zip › Figure 4/panel 4B/KD-2_Bra_5uM/fila51877_RGB_DAPI.tif]

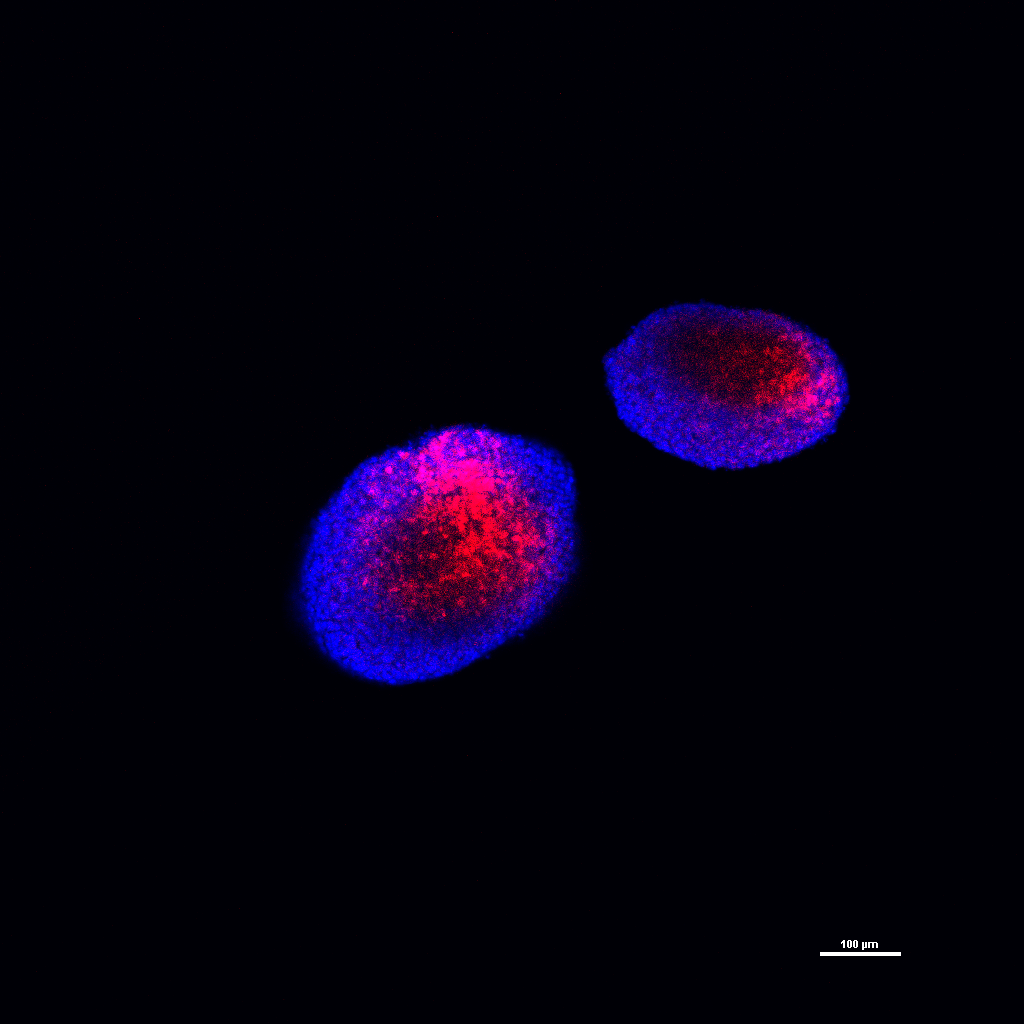

Supplement: Supplementary file 6 — Source data Fig. 4 [file 44318_2025_558_MOESM6_ESM.zip › Figure 4/panel 4B/KD-2_Bra_5uM/fila51877_RGB.tif]

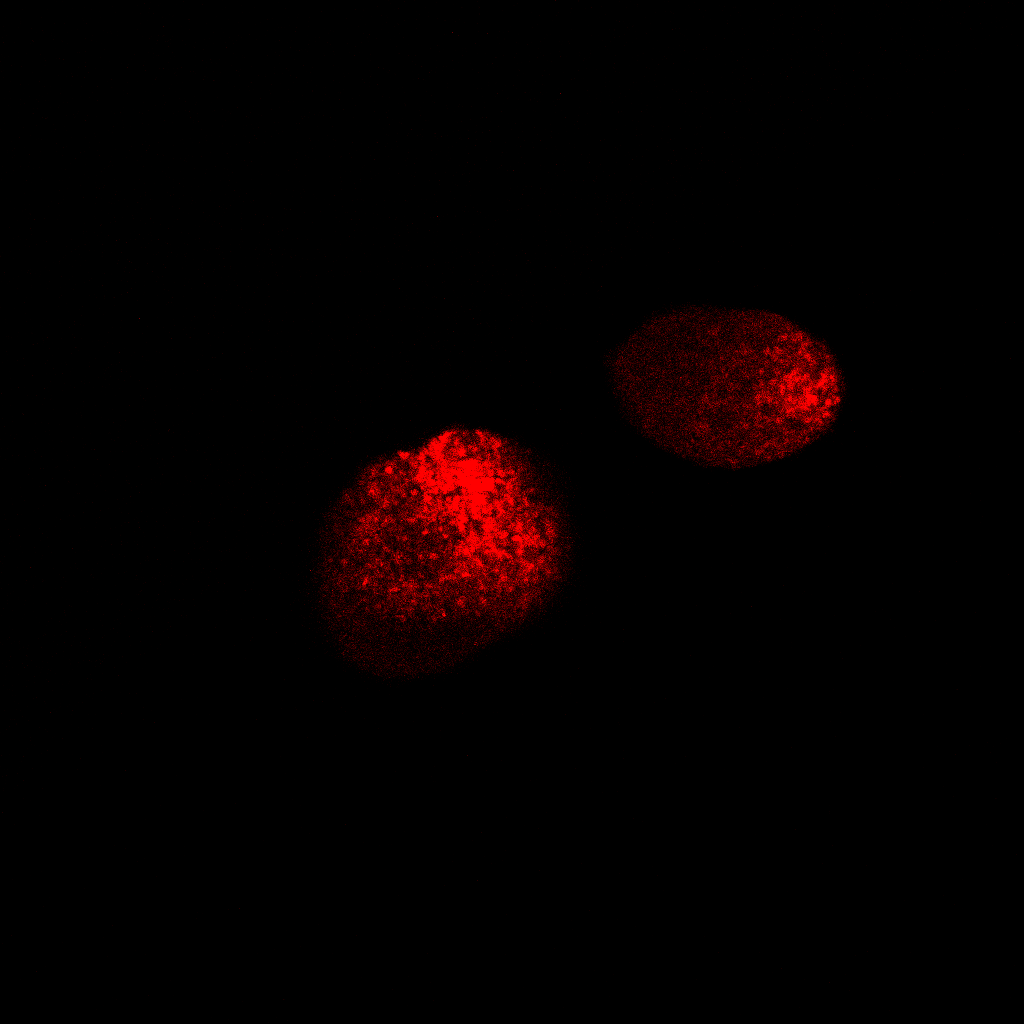

Supplement: Supplementary file 6 — Source data Fig. 4 [file 44318_2025_558_MOESM6_ESM.zip › Figure 4/panel 4B/KD-2_Bra_5uM/fila51877_RGB_Alexa Fluor 594 cadaverine_H2O.tif]

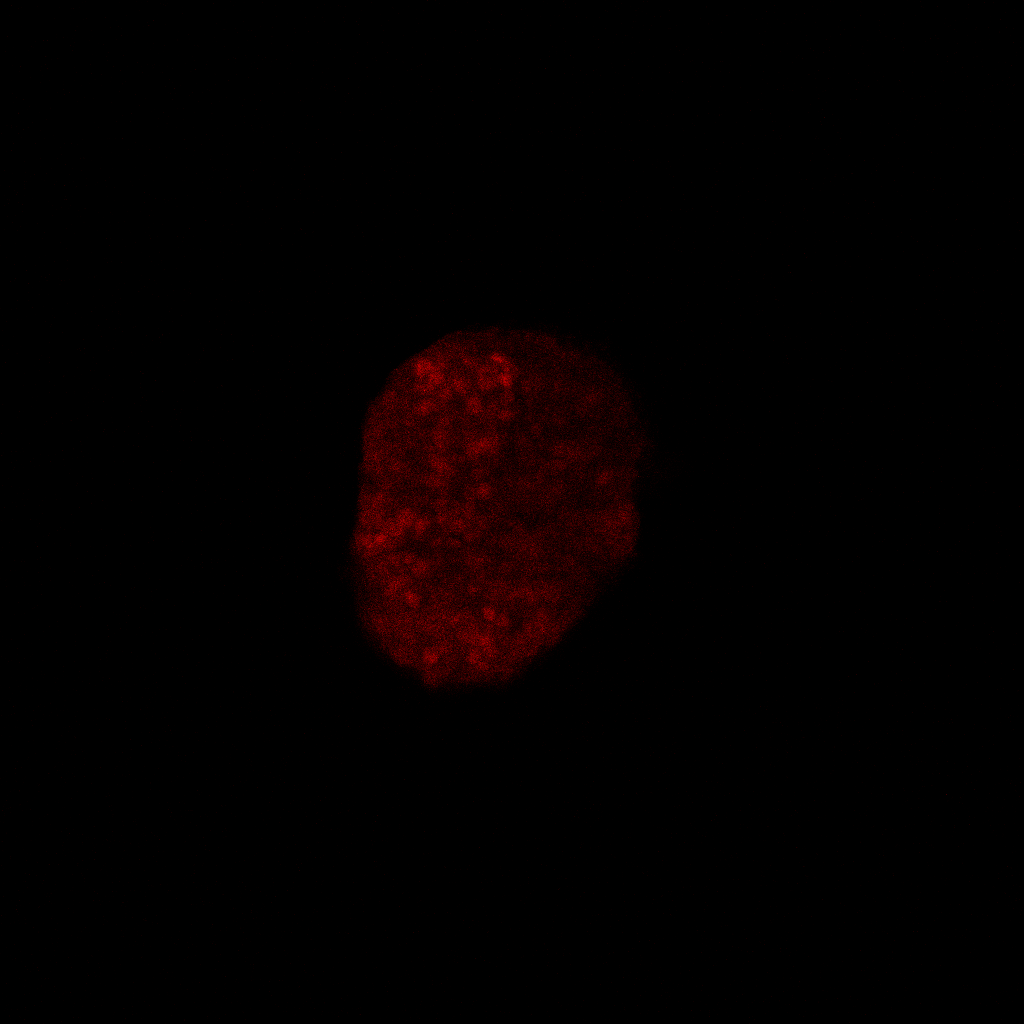

Supplement: Supplementary file 6 — Source data Fig. 4 [file 44318_2025_558_MOESM6_ESM.zip › Figure 4/panel 4B/KD-2_Oct4_3uM/seq9865_seq9865_RGB_TRITC.tif]

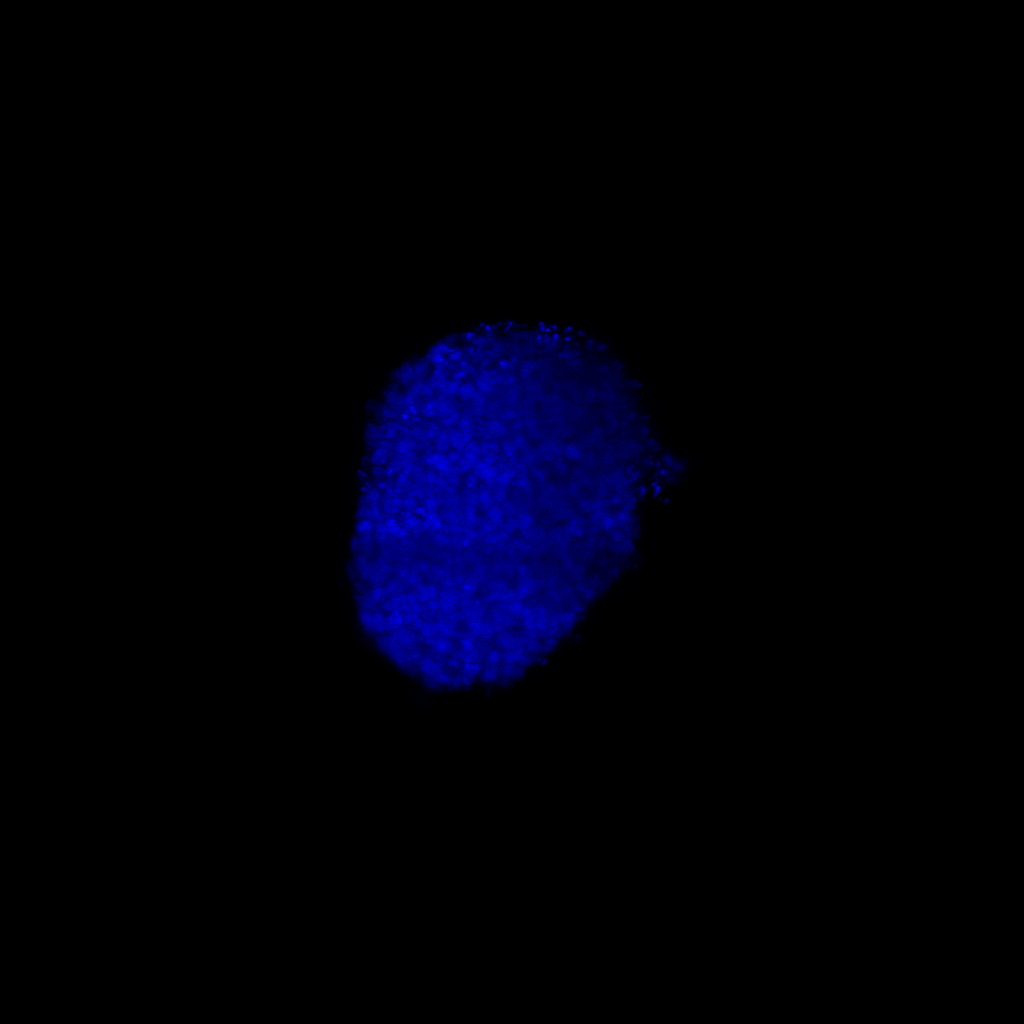

Supplement: Supplementary file 6 — Source data Fig. 4 [file 44318_2025_558_MOESM6_ESM.zip › Figure 4/panel 4B/KD-2_Oct4_3uM/seq9865_seq9865_RGB_DAPI.tif]

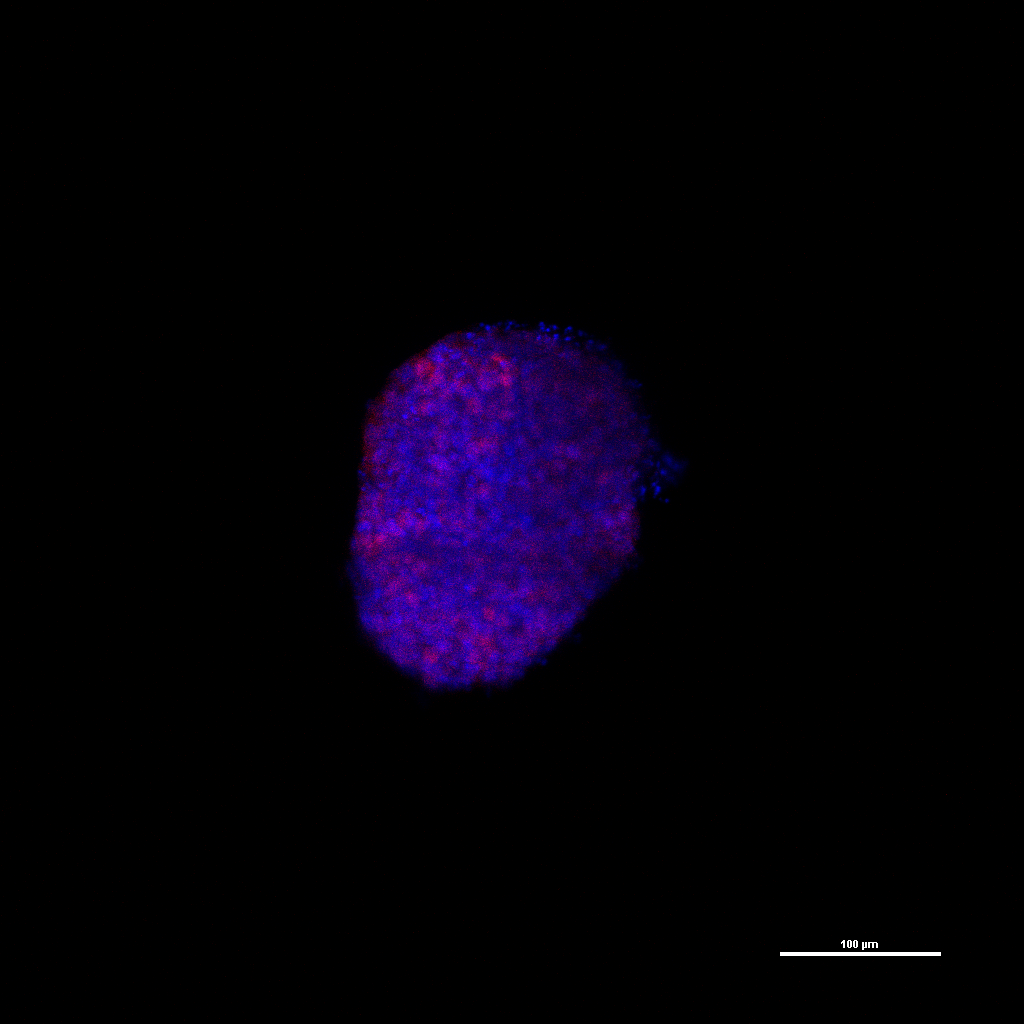

Supplement: Supplementary file 6 — Source data Fig. 4 [file 44318_2025_558_MOESM6_ESM.zip › Figure 4/panel 4B/KD-2_Oct4_3uM/seq9865_seq9865_RGB.tif]

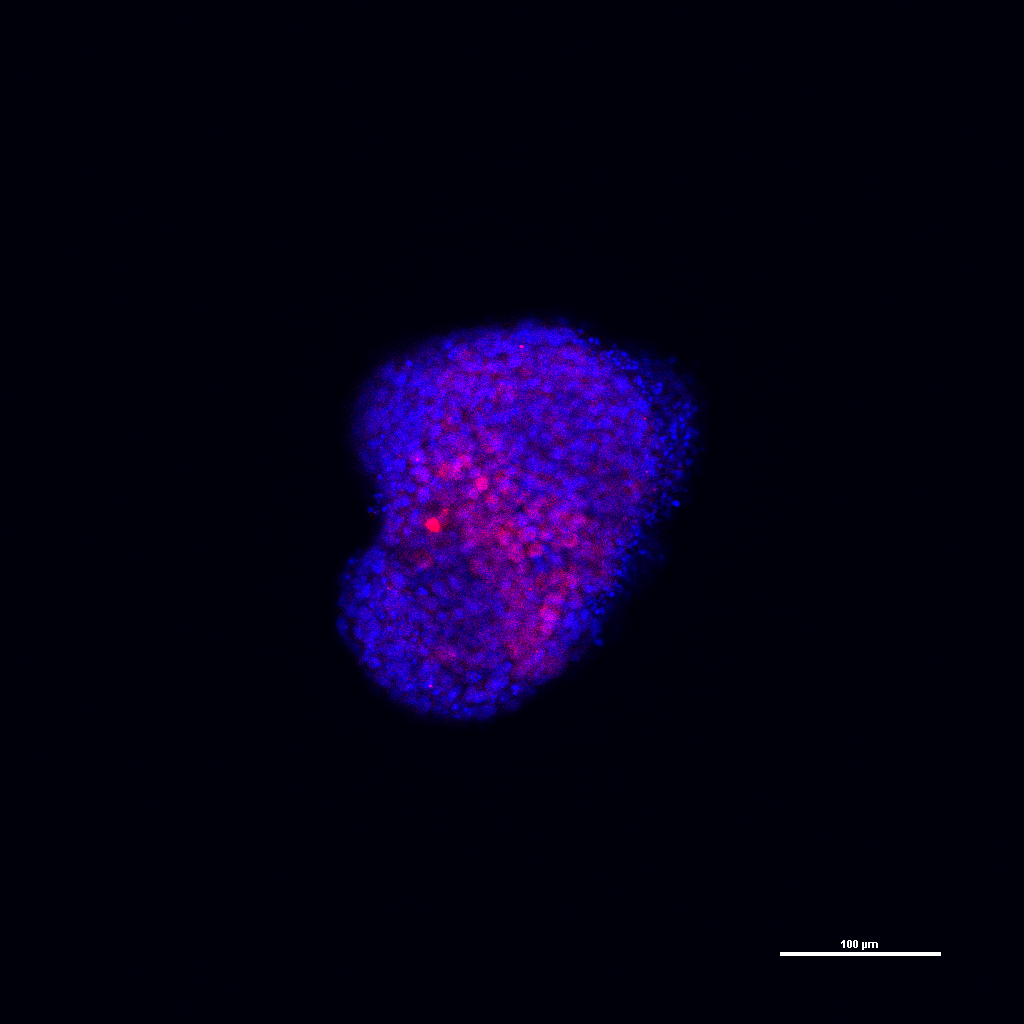

Supplement: Supplementary file 6 — Source data Fig. 4 [file 44318_2025_558_MOESM6_ESM.zip › Figure 4/panel 4B/KD-1_Bra_6uM/seq8980_seq8980_RGB.tif]

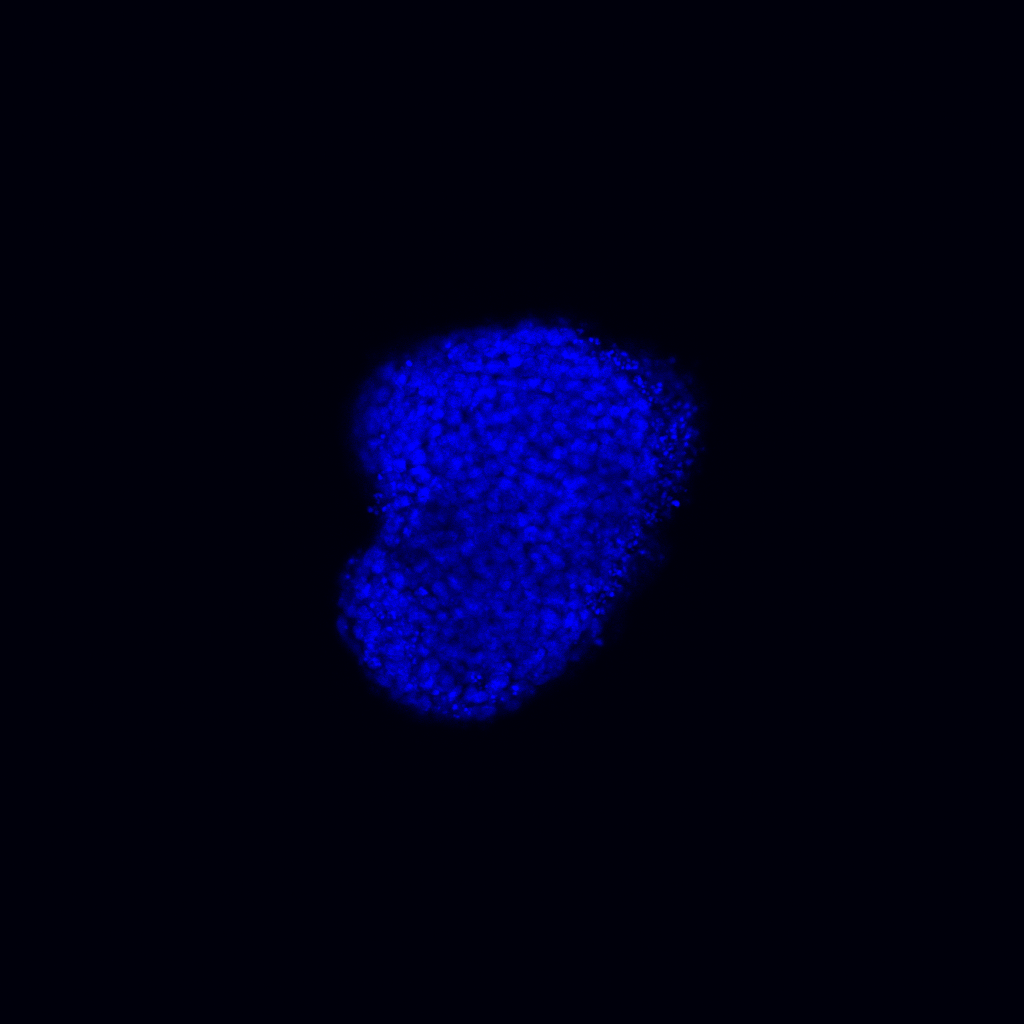

Supplement: Supplementary file 6 — Source data Fig. 4 [file 44318_2025_558_MOESM6_ESM.zip › Figure 4/panel 4B/KD-1_Bra_6uM/seq8980_seq8980_RGB_DAPI.tif]

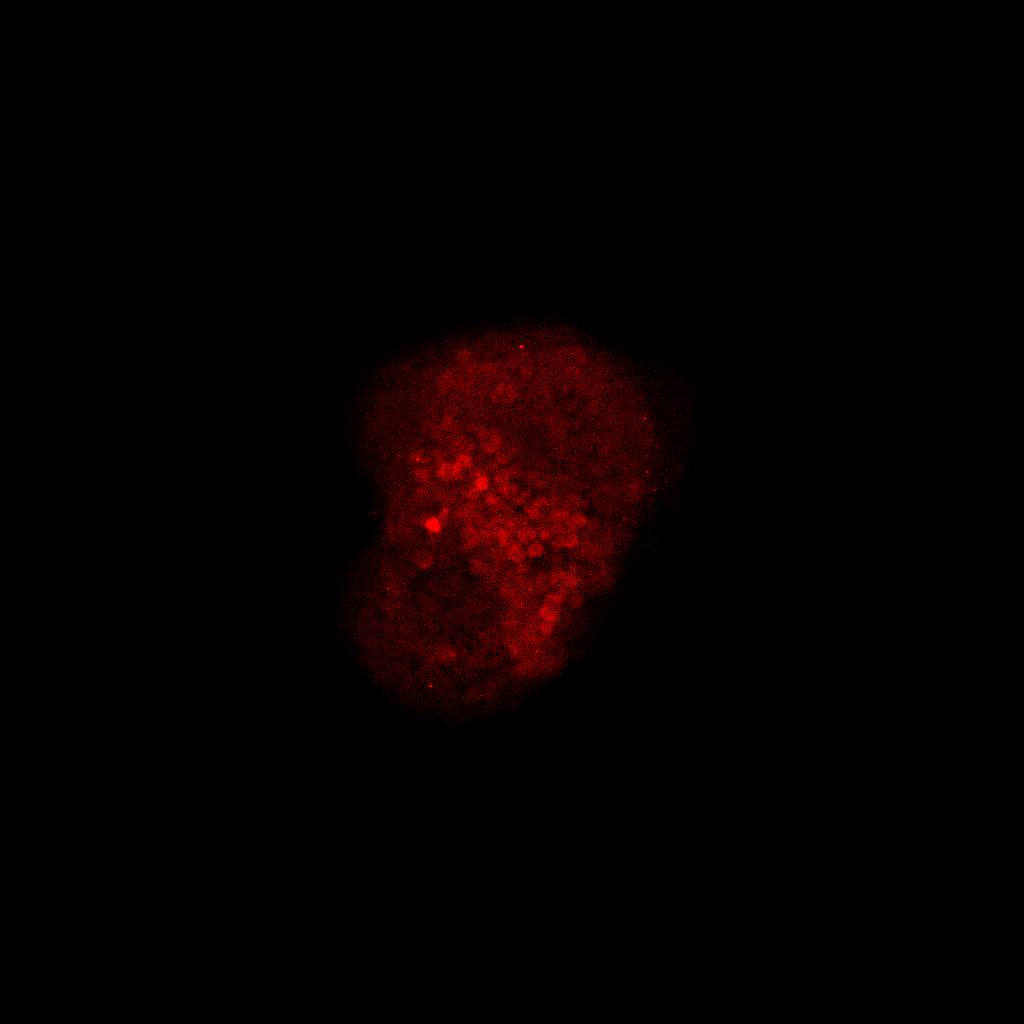

Supplement: Supplementary file 6 — Source data Fig. 4 [file 44318_2025_558_MOESM6_ESM.zip › Figure 4/panel 4B/KD-1_Bra_6uM/seq8980_seq8980_RGB_Texas Red.tif]

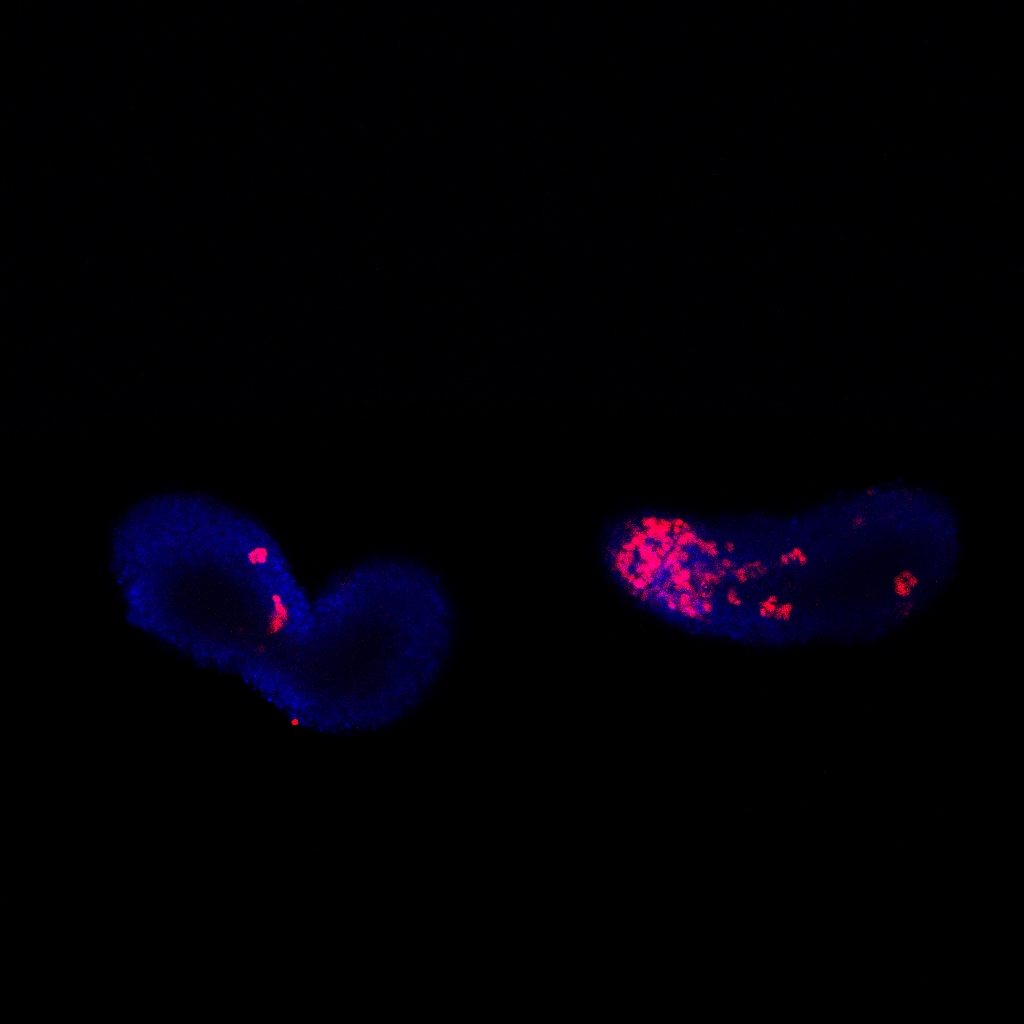

Supplement: Supplementary file 6 — Source data Fig. 4 [file 44318_2025_558_MOESM6_ESM.zip › Figure 4/panel 4B/KD-1_Nanog_5uM/fila51855-1.tif]

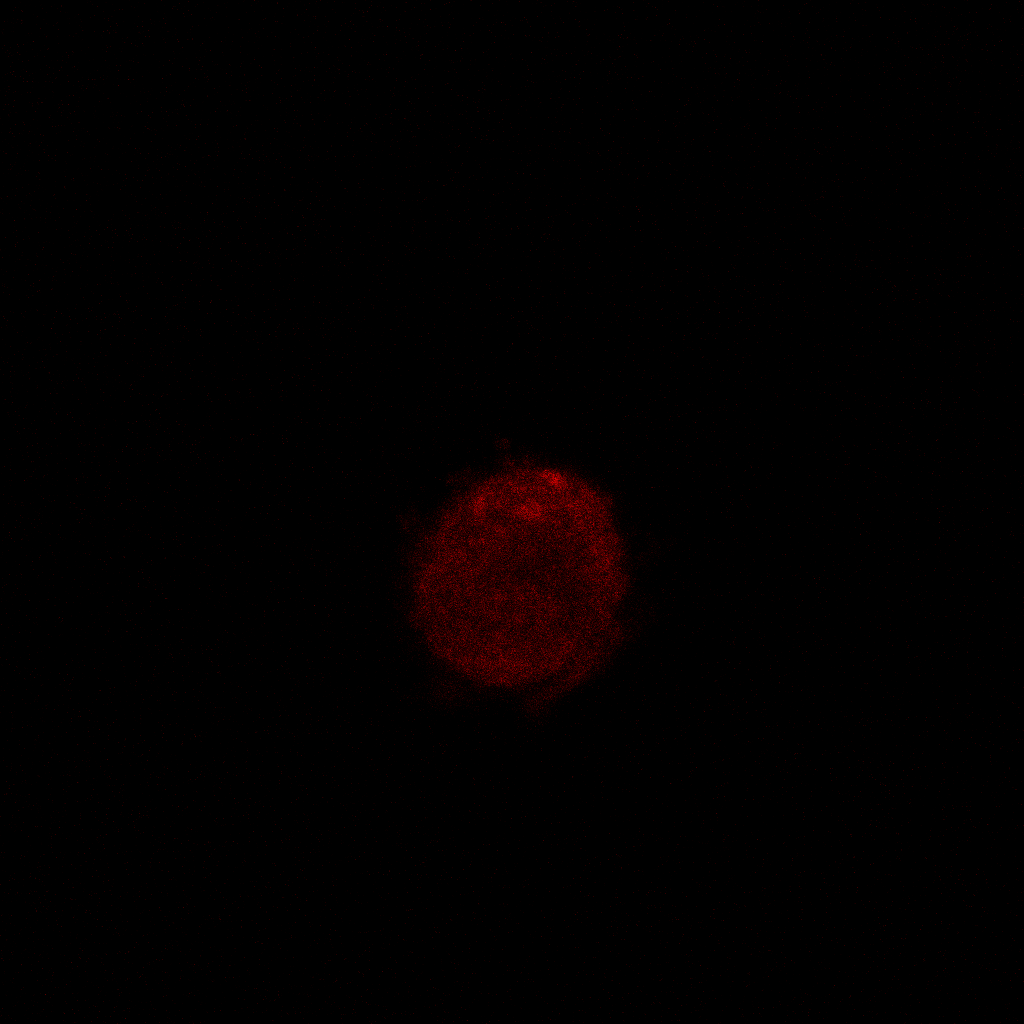

Supplement: Supplementary file 6 — Source data Fig. 4 [file 44318_2025_558_MOESM6_ESM.zip › Figure 4/panel 4B/KD-1_Cdx2_6uM/seq9893_seq9893_RGB_TRITC.tif]

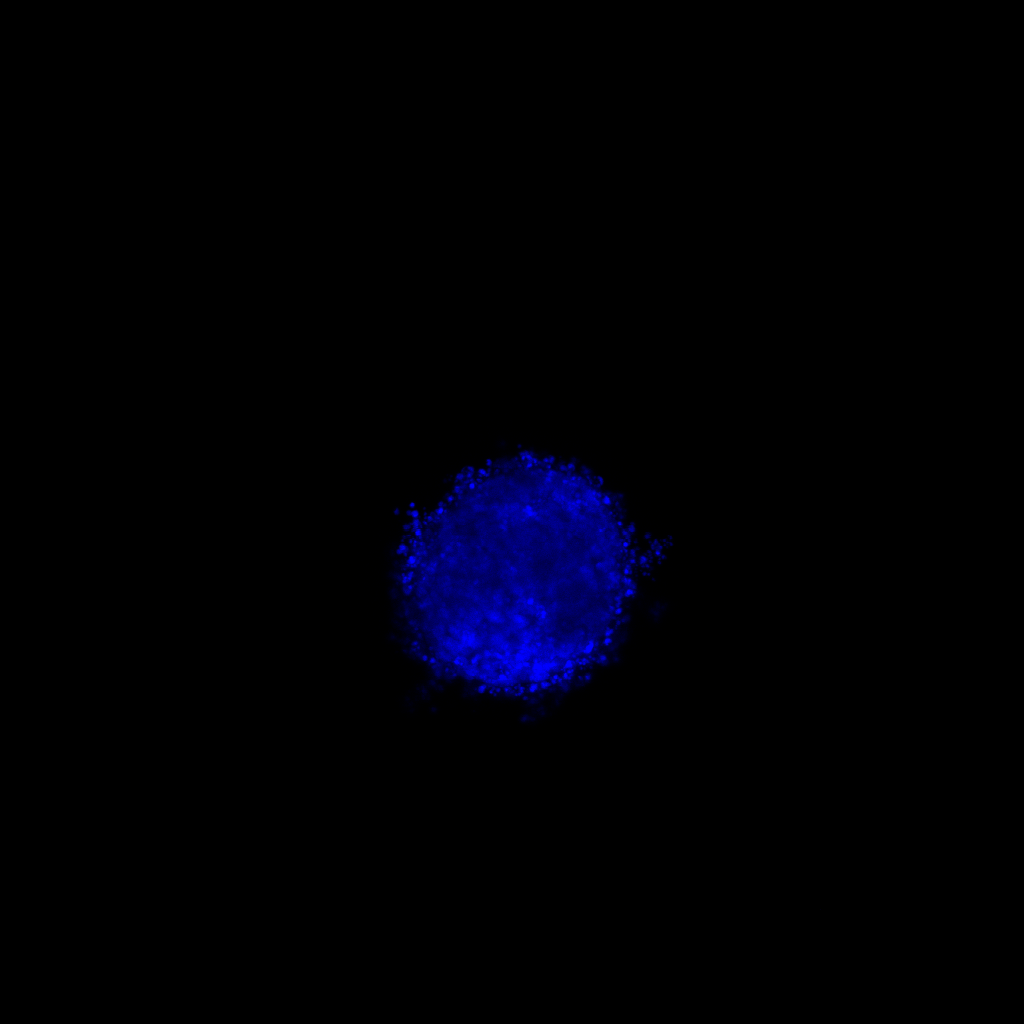

Supplement: Supplementary file 6 — Source data Fig. 4 [file 44318_2025_558_MOESM6_ESM.zip › Figure 4/panel 4B/KD-1_Cdx2_6uM/seq9893_seq9893_RGB_DAPI.tif]

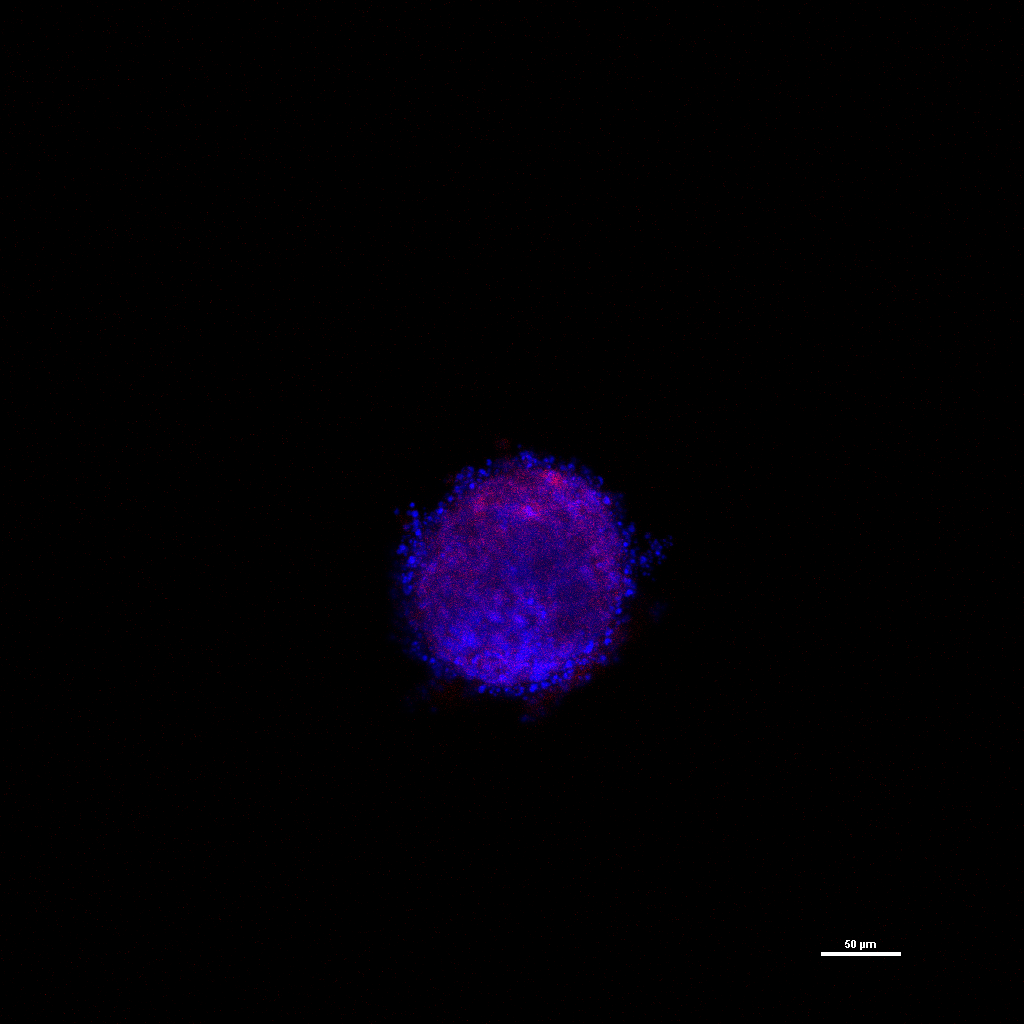

Supplement: Supplementary file 6 — Source data Fig. 4 [file 44318_2025_558_MOESM6_ESM.zip › Figure 4/panel 4B/KD-1_Cdx2_6uM/seq9893_seq9893_RGB.tif]

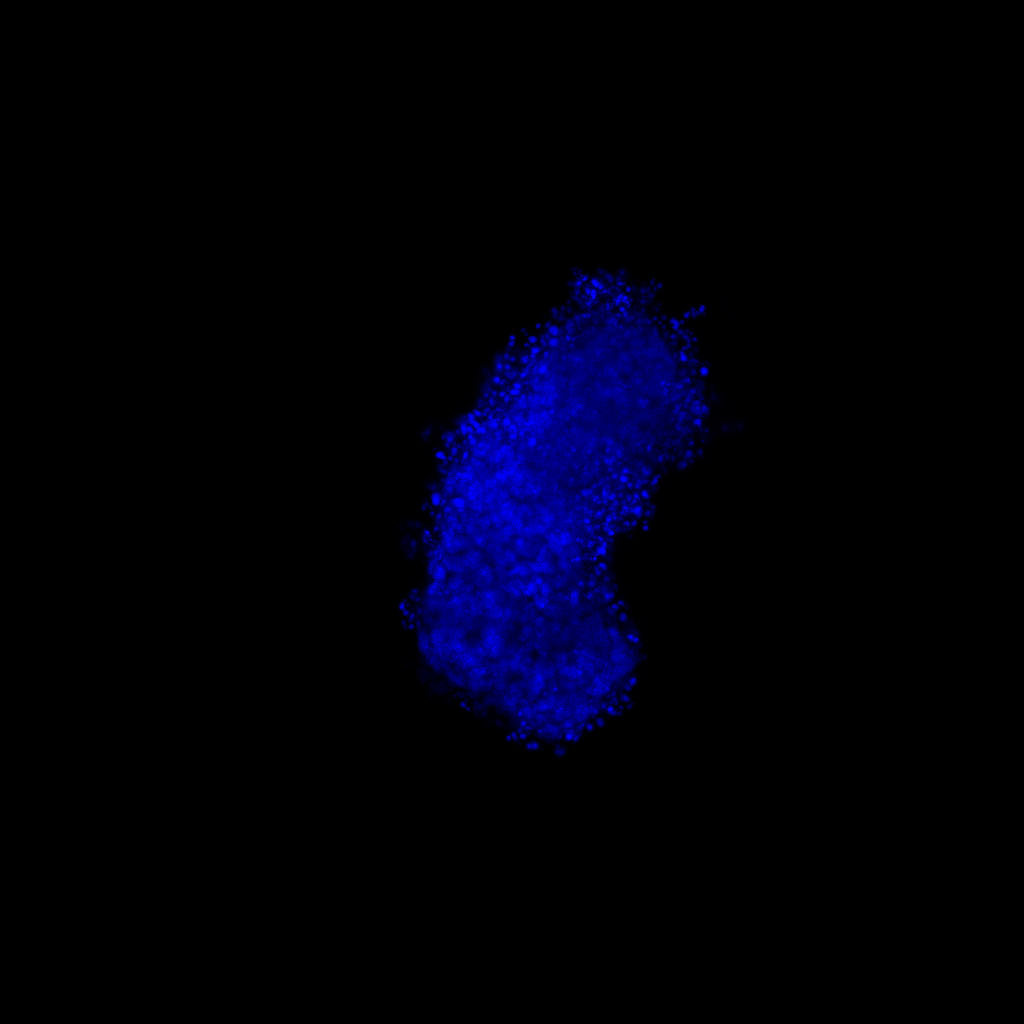

Supplement: Supplementary file 6 — Source data Fig. 4 [file 44318_2025_558_MOESM6_ESM.zip › Figure 4/panel 4B/KD-1_Oct4_5uM/seq9860_seq9860_RGB_DAPI.tif]

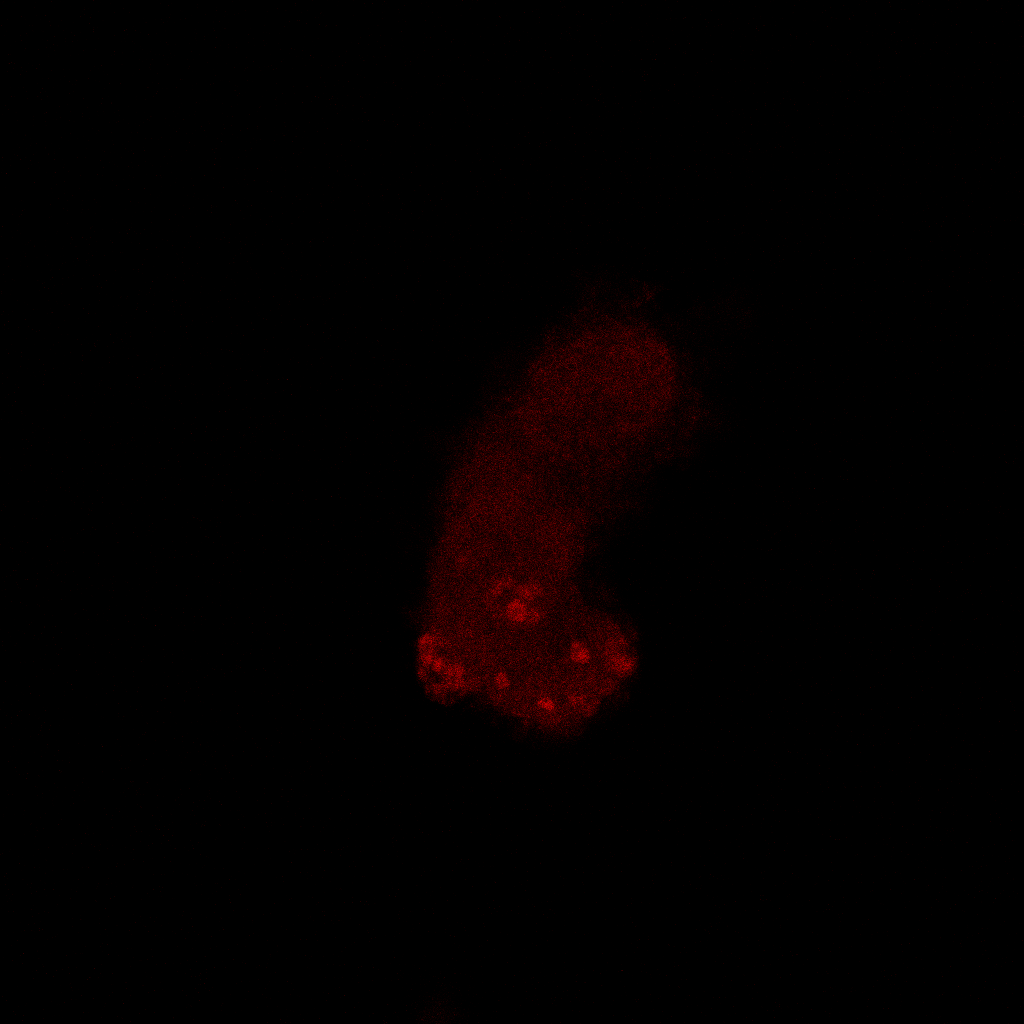

Supplement: Supplementary file 6 — Source data Fig. 4 [file 44318_2025_558_MOESM6_ESM.zip › Figure 4/panel 4B/KD-1_Oct4_5uM/seq9860_seq9860_RGB_TRITC.tif]

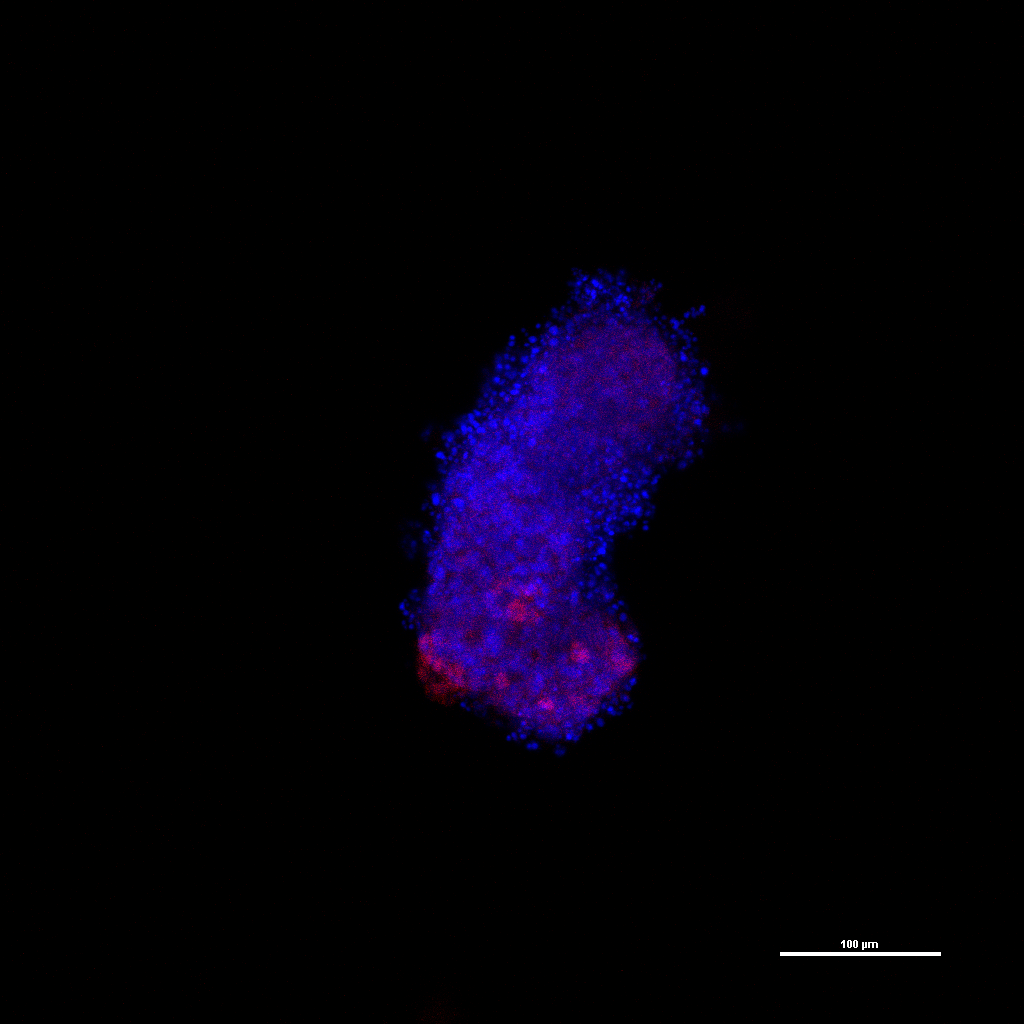

Supplement: Supplementary file 6 — Source data Fig. 4 [file 44318_2025_558_MOESM6_ESM.zip › Figure 4/panel 4B/KD-1_Oct4_5uM/seq9860_seq9860_RGB.tif]

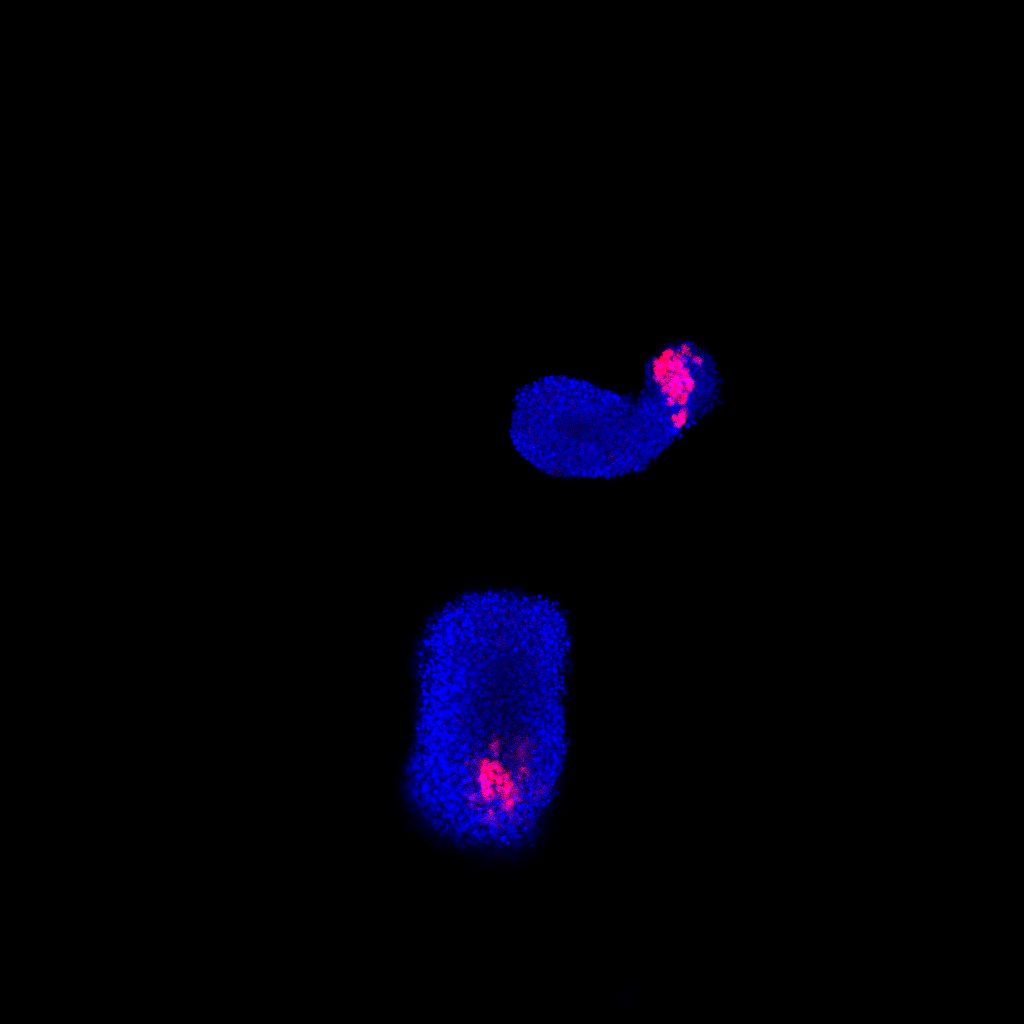

Supplement: Supplementary file 6 — Source data Fig. 4 [file 44318_2025_558_MOESM6_ESM.zip › Figure 4/panel 4B/NT_Nanog_6uM/fila51851-1.tif]

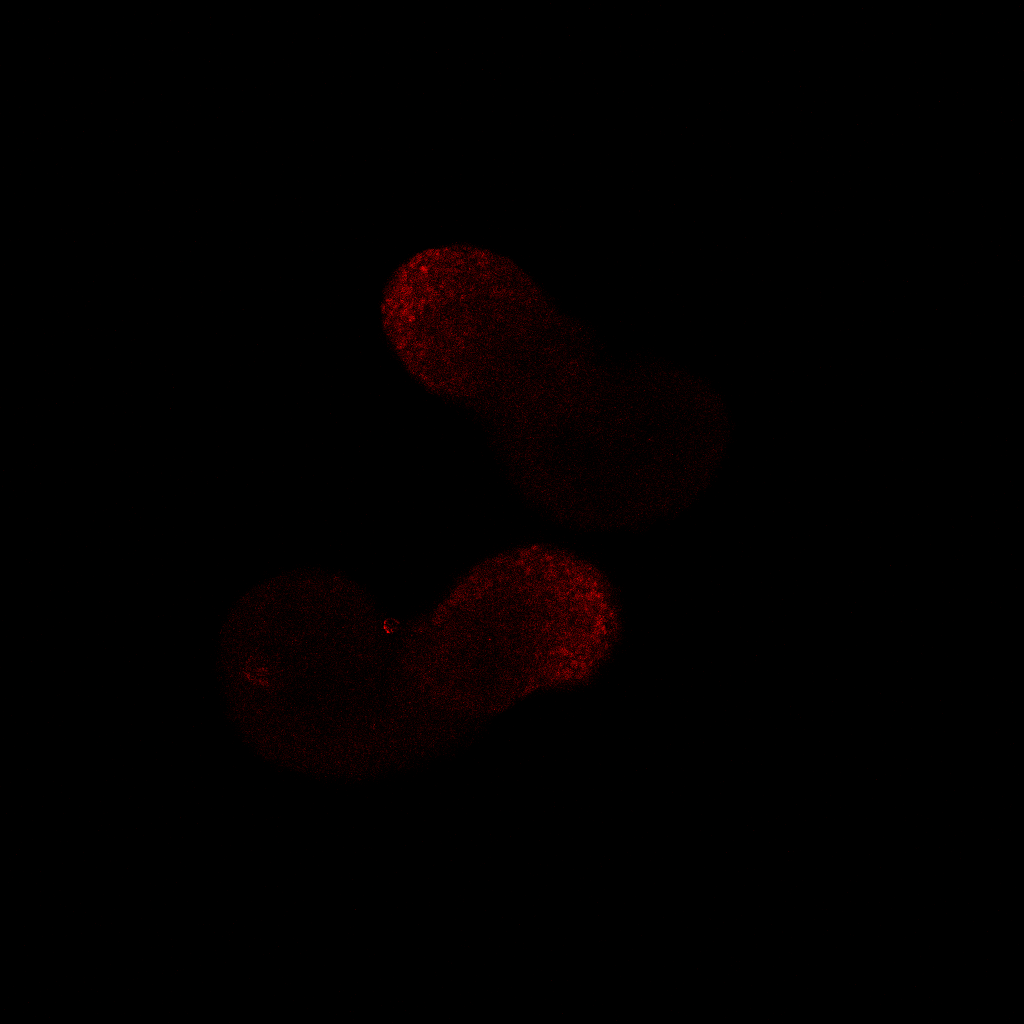

Supplement: Supplementary file 6 — Source data Fig. 4 [file 44318_2025_558_MOESM6_ESM.zip › Figure 4/panel 4B/KD-2_Bra_6uM/fila51875_RGB_Alexa Fluor 594 cadaverine_H2O.tif]

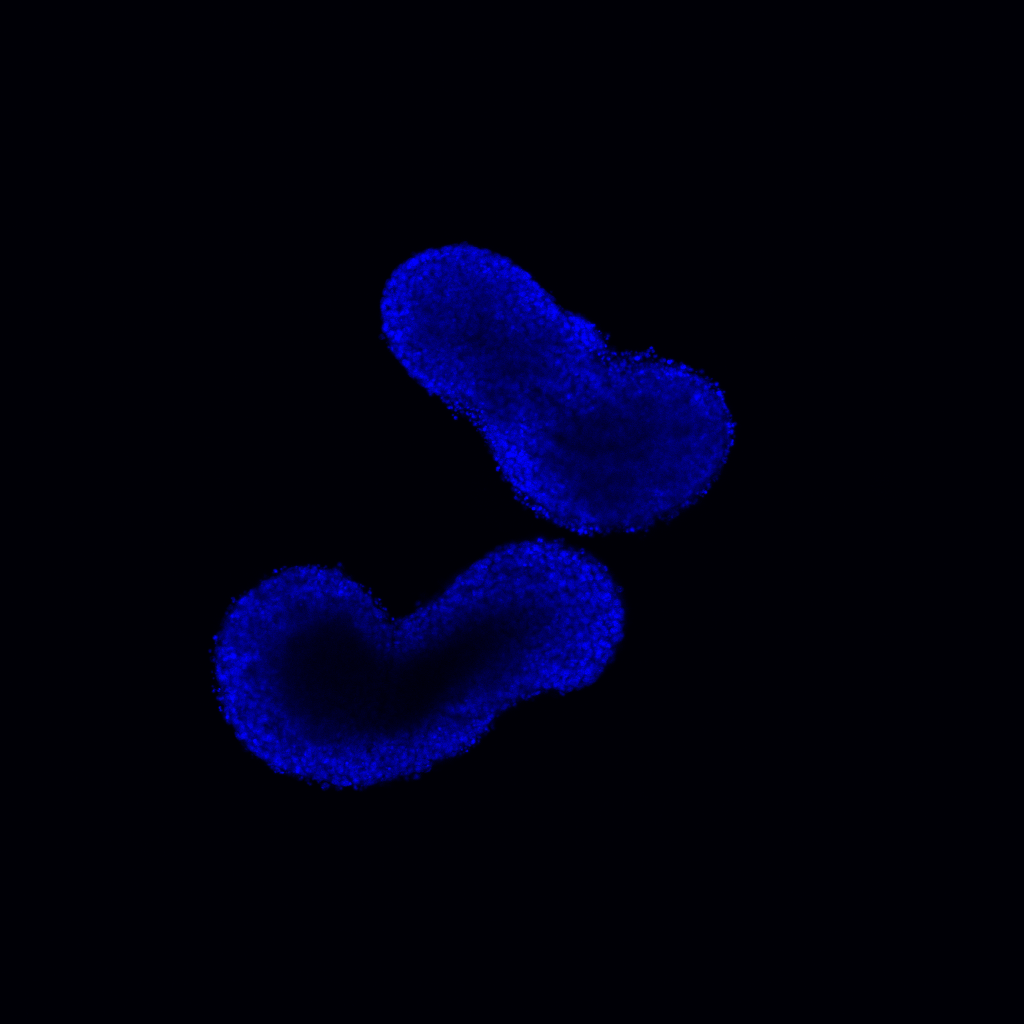

Supplement: Supplementary file 6 — Source data Fig. 4 [file 44318_2025_558_MOESM6_ESM.zip › Figure 4/panel 4B/KD-2_Bra_6uM/fila51875_RGB_DAPI.tif]

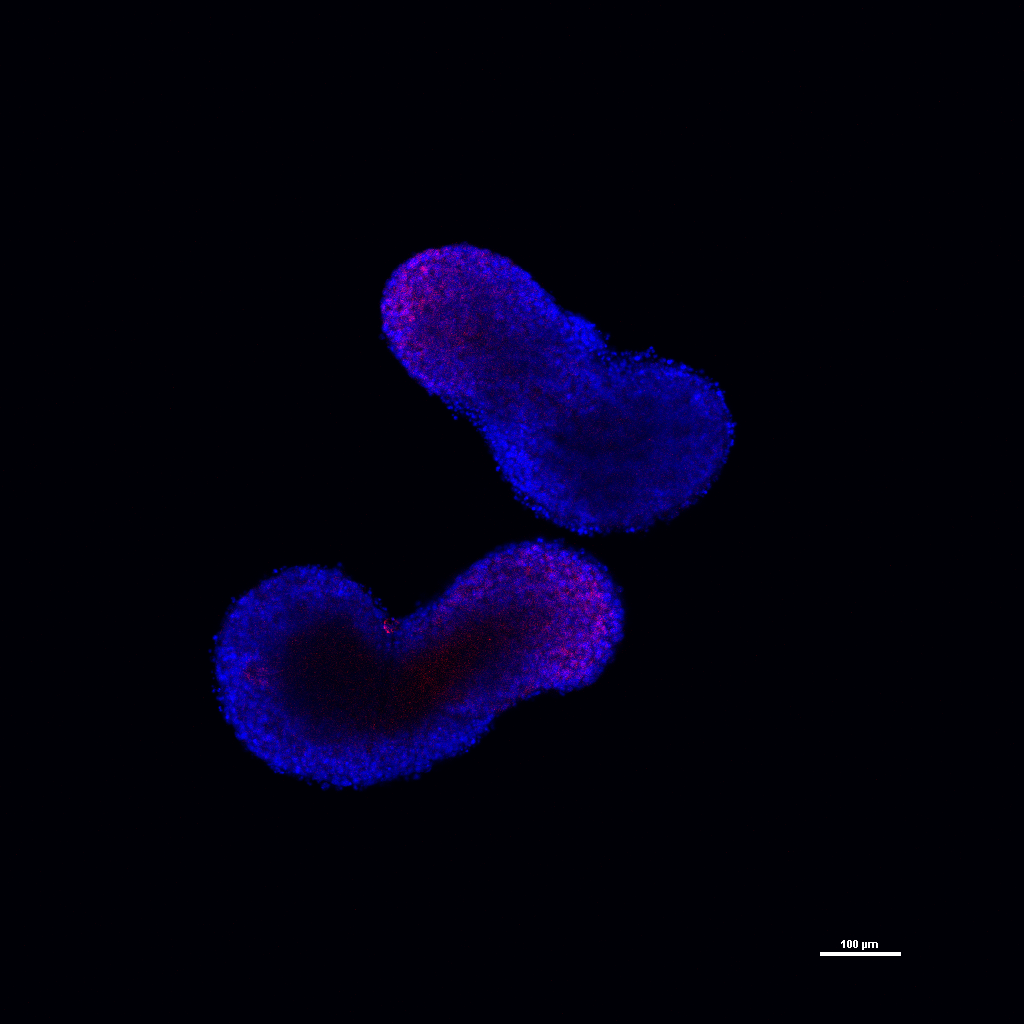

Supplement: Supplementary file 6 — Source data Fig. 4 [file 44318_2025_558_MOESM6_ESM.zip › Figure 4/panel 4B/KD-2_Bra_6uM/fila51875_RGB.tif]

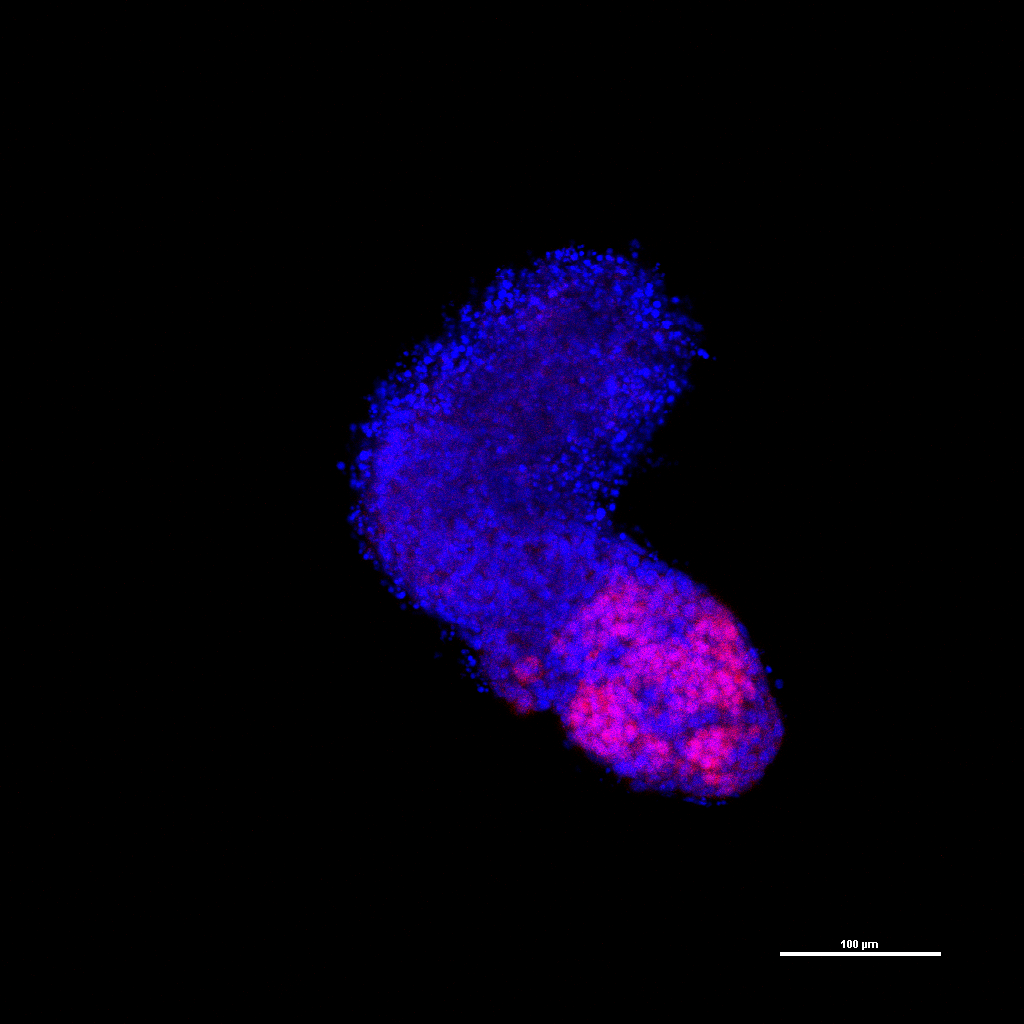

Supplement: Supplementary file 6 — Source data Fig. 4 [file 44318_2025_558_MOESM6_ESM.zip › Figure 4/panel 4B/NT_Oct4_3uM/seq9847_seq9847_RGB.tif]

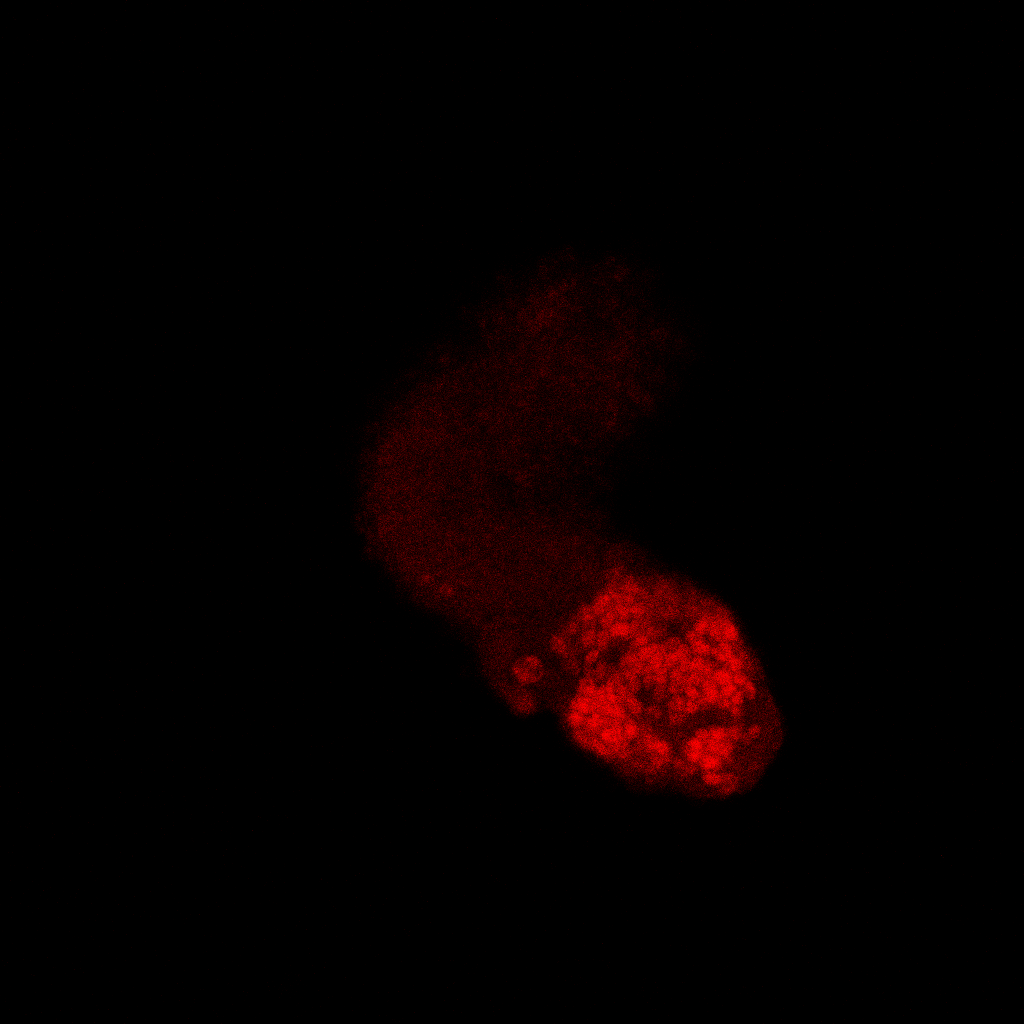

Supplement: Supplementary file 6 — Source data Fig. 4 [file 44318_2025_558_MOESM6_ESM.zip › Figure 4/panel 4B/NT_Oct4_3uM/seq9847_seq9847_RGB_TRITC.tif]

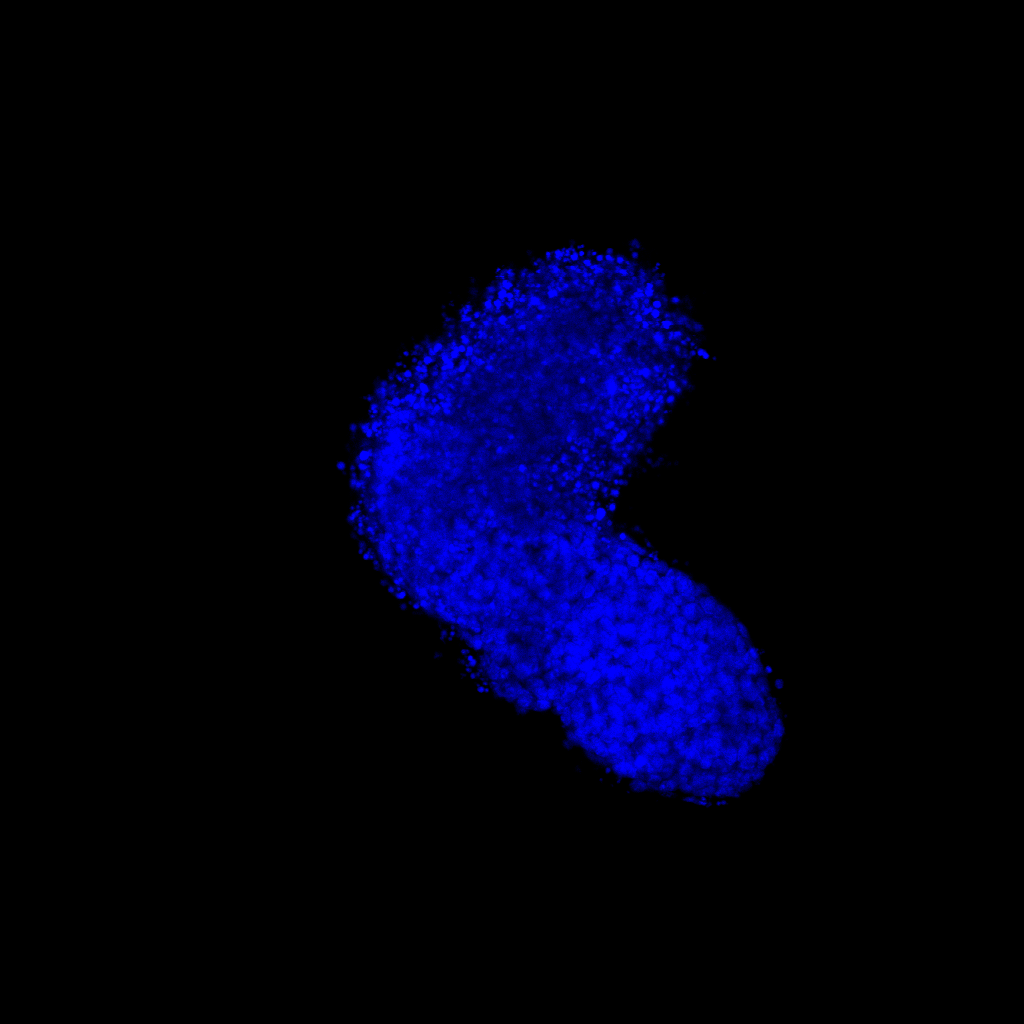

Supplement: Supplementary file 6 — Source data Fig. 4 [file 44318_2025_558_MOESM6_ESM.zip › Figure 4/panel 4B/NT_Oct4_3uM/seq9847_seq9847_RGB_DAPI.tif]

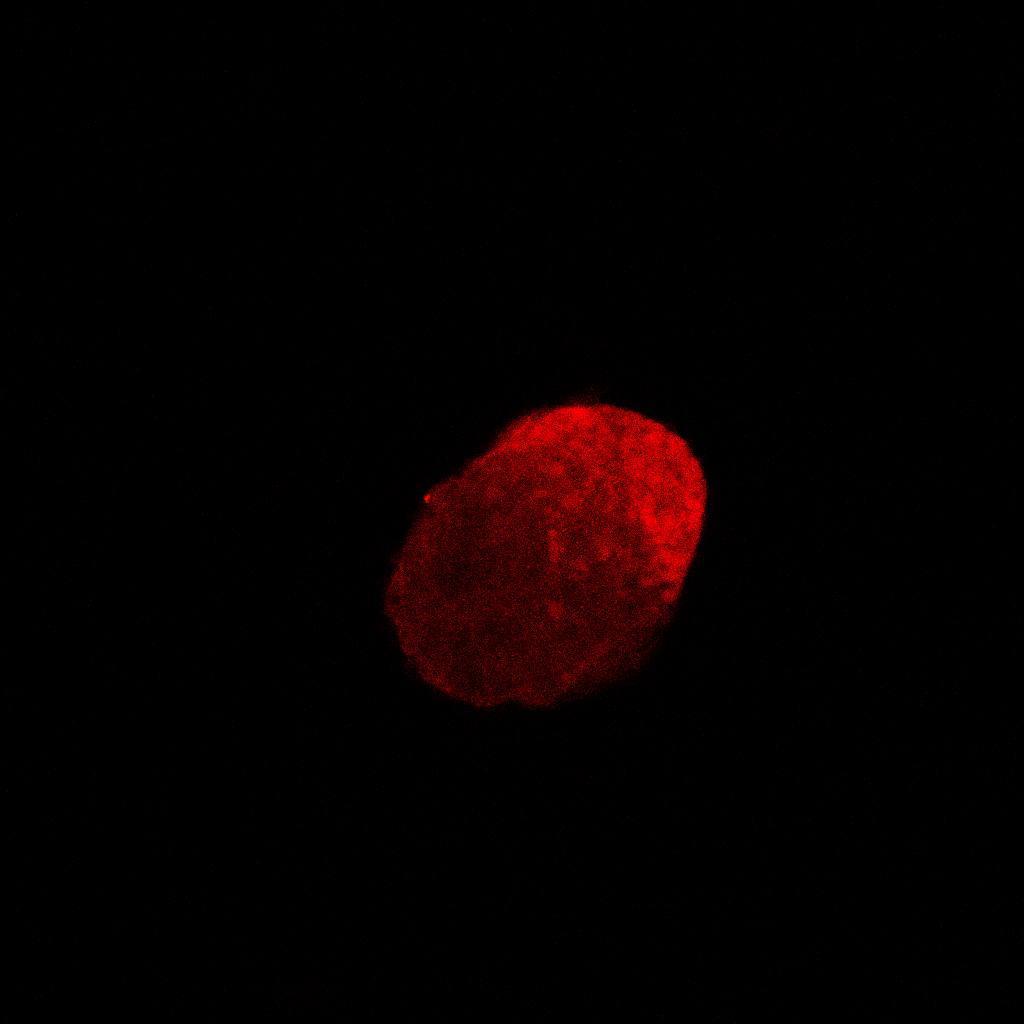

Supplement: Supplementary file 6 — Source data Fig. 4 [file 44318_2025_558_MOESM6_ESM.zip › Figure 4/panel 4B/KD-2_Cdx2_3uM/seq9900_seq9900_RGB_TRITC.tif]

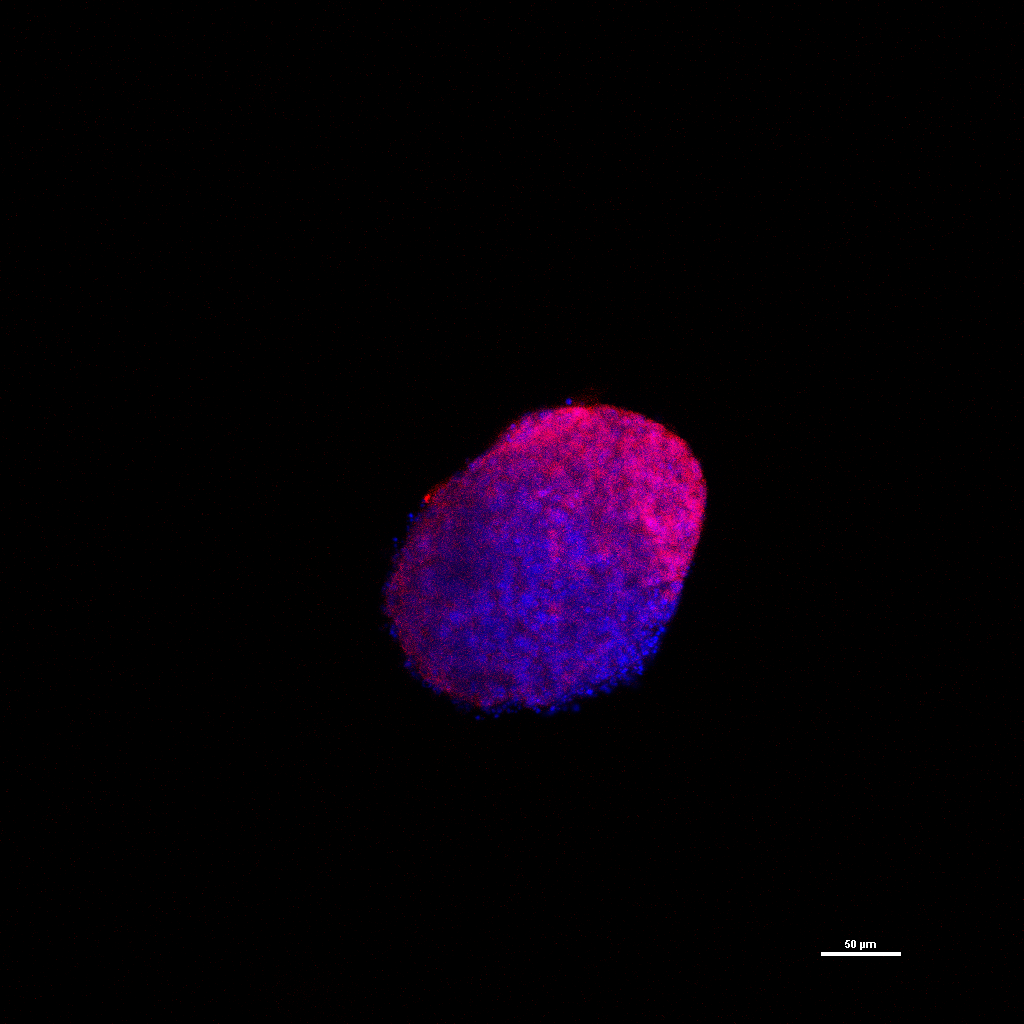

Supplement: Supplementary file 6 — Source data Fig. 4 [file 44318_2025_558_MOESM6_ESM.zip › Figure 4/panel 4B/KD-2_Cdx2_3uM/seq9900_seq9900_RGB.tif]

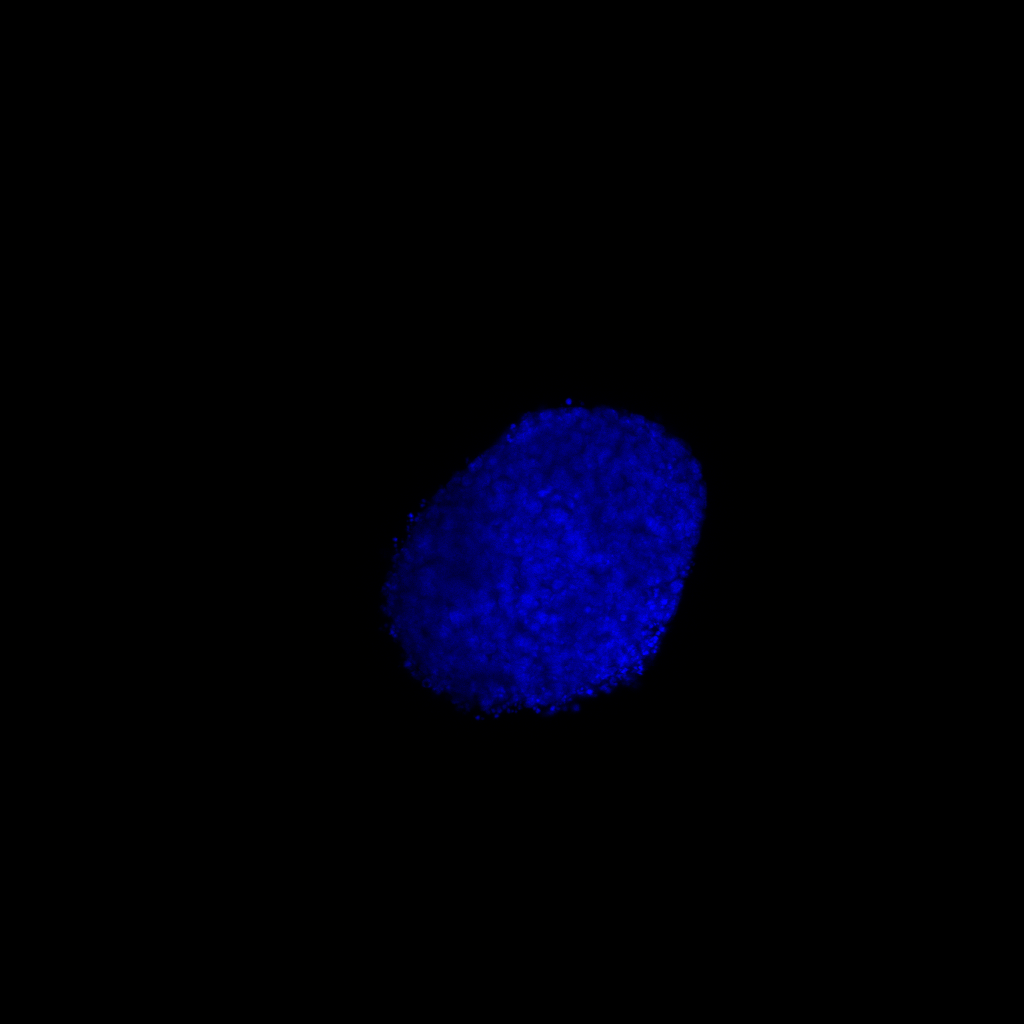

Supplement: Supplementary file 6 — Source data Fig. 4 [file 44318_2025_558_MOESM6_ESM.zip › Figure 4/panel 4B/KD-2_Cdx2_3uM/seq9900_seq9900_RGB_DAPI.tif]

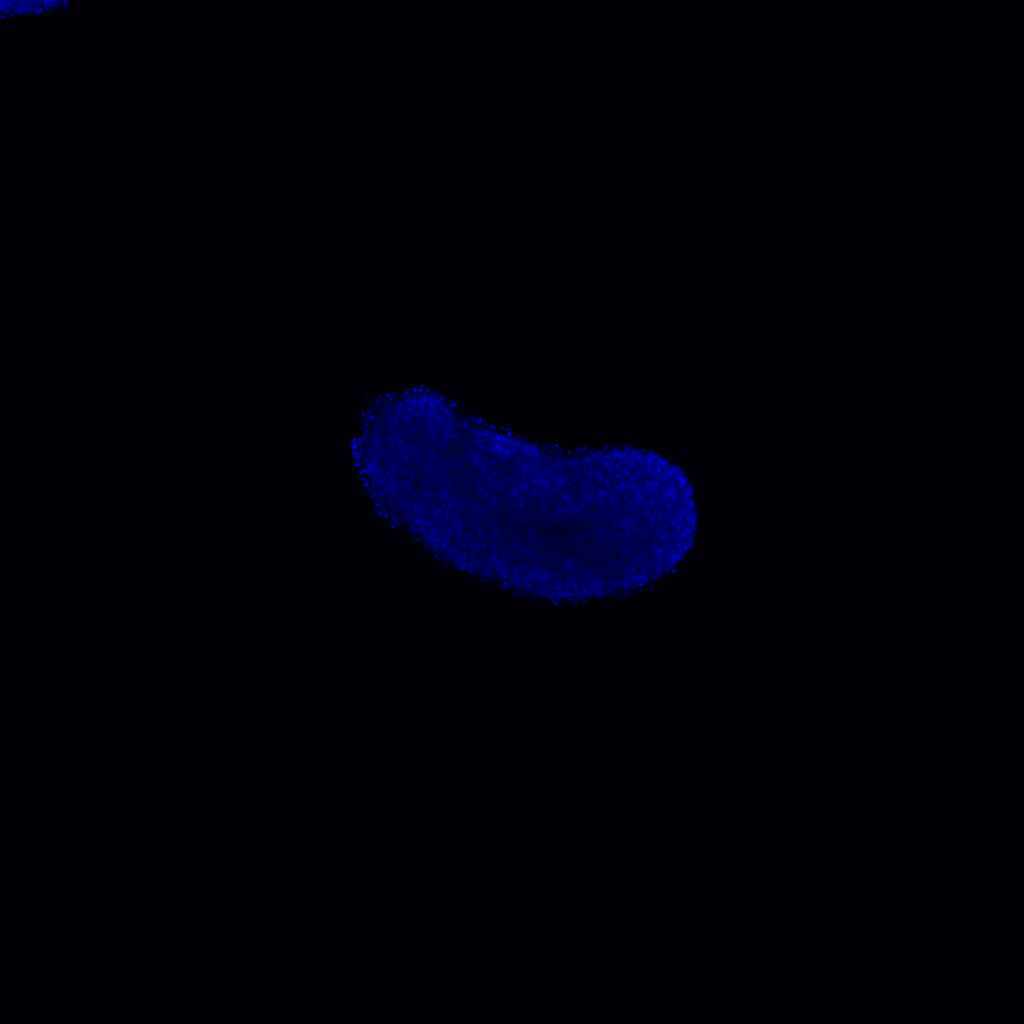

Supplement: Supplementary file 6 — Source data Fig. 4 [file 44318_2025_558_MOESM6_ESM.zip › Figure 4/panel 4B/NT_Bra_3uM/fila51864_RGB_DAPI.tif]

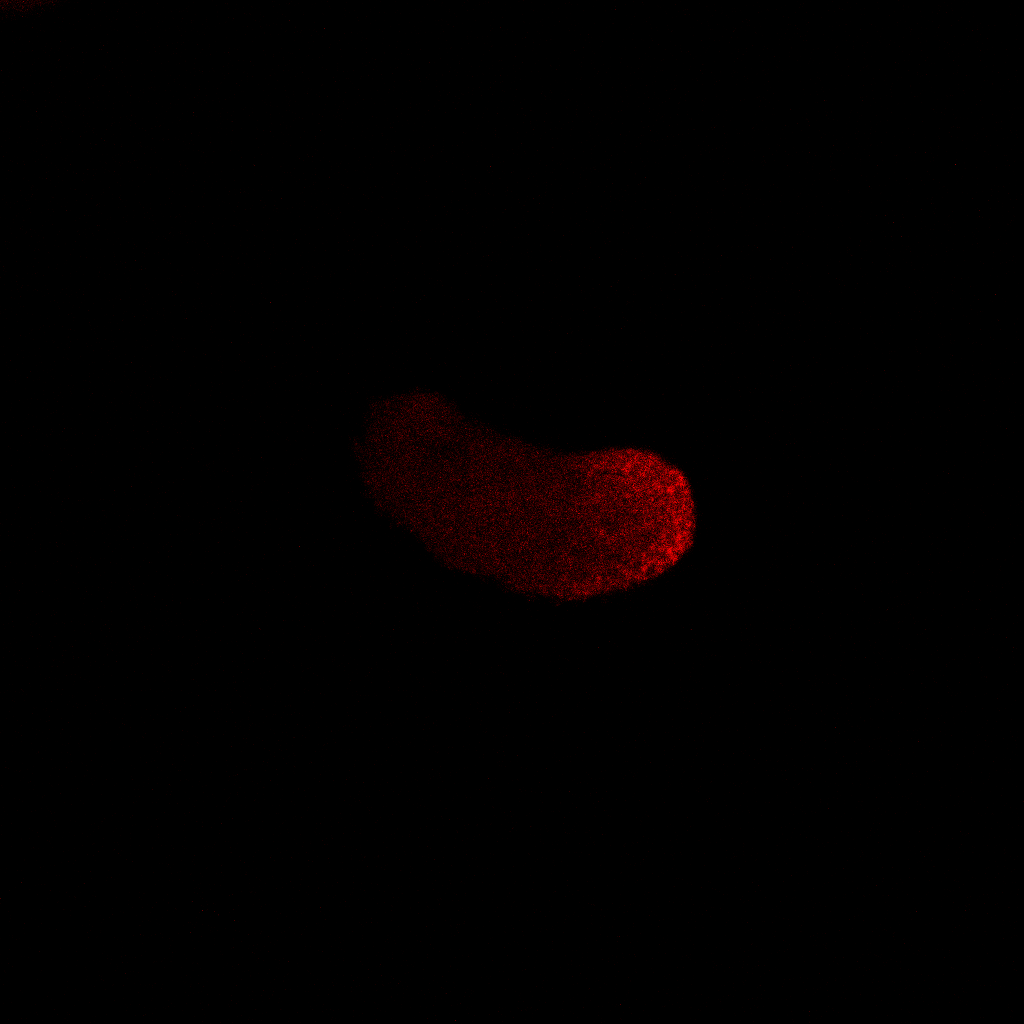

Supplement: Supplementary file 6 — Source data Fig. 4 [file 44318_2025_558_MOESM6_ESM.zip › Figure 4/panel 4B/NT_Bra_3uM/fila51864_RGB_Alexa Fluor 594 cadaverine_H2O.tif]

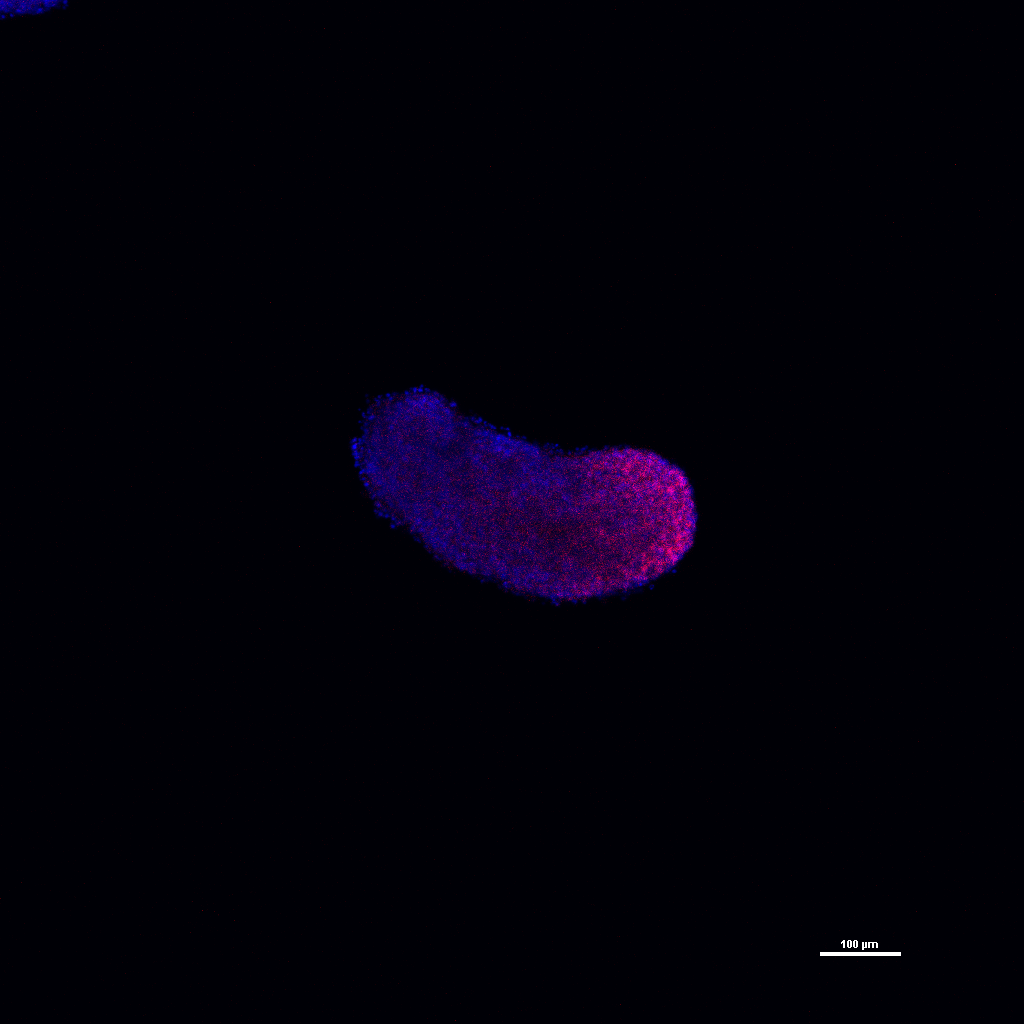

Supplement: Supplementary file 6 — Source data Fig. 4 [file 44318_2025_558_MOESM6_ESM.zip › Figure 4/panel 4B/NT_Bra_3uM/fila51864_RGB.tif]

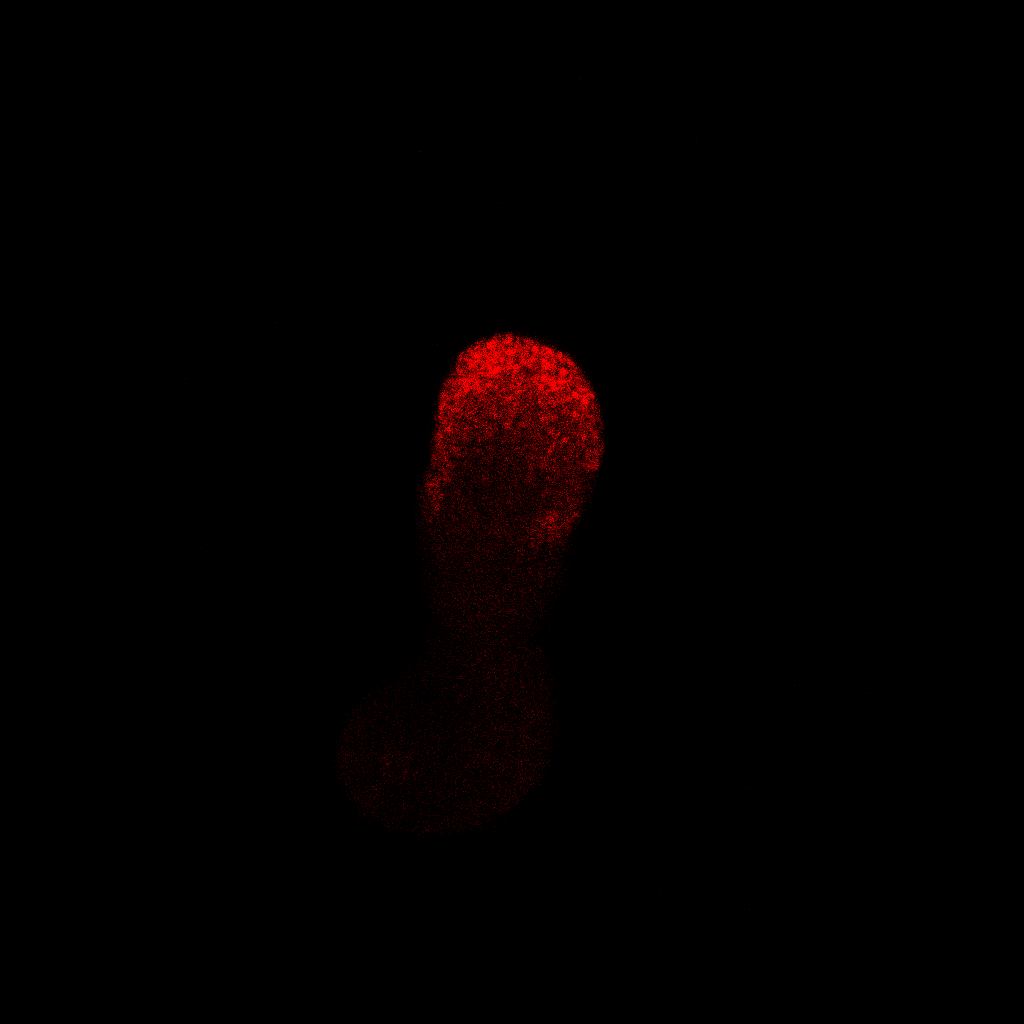

Supplement: Supplementary file 6 — Source data Fig. 4 [file 44318_2025_558_MOESM6_ESM.zip › Figure 4/panel 4B/KD-1_Bra_5uM/fila51874_RGB_Alexa Fluor 594 cadaverine_H2O.tif]

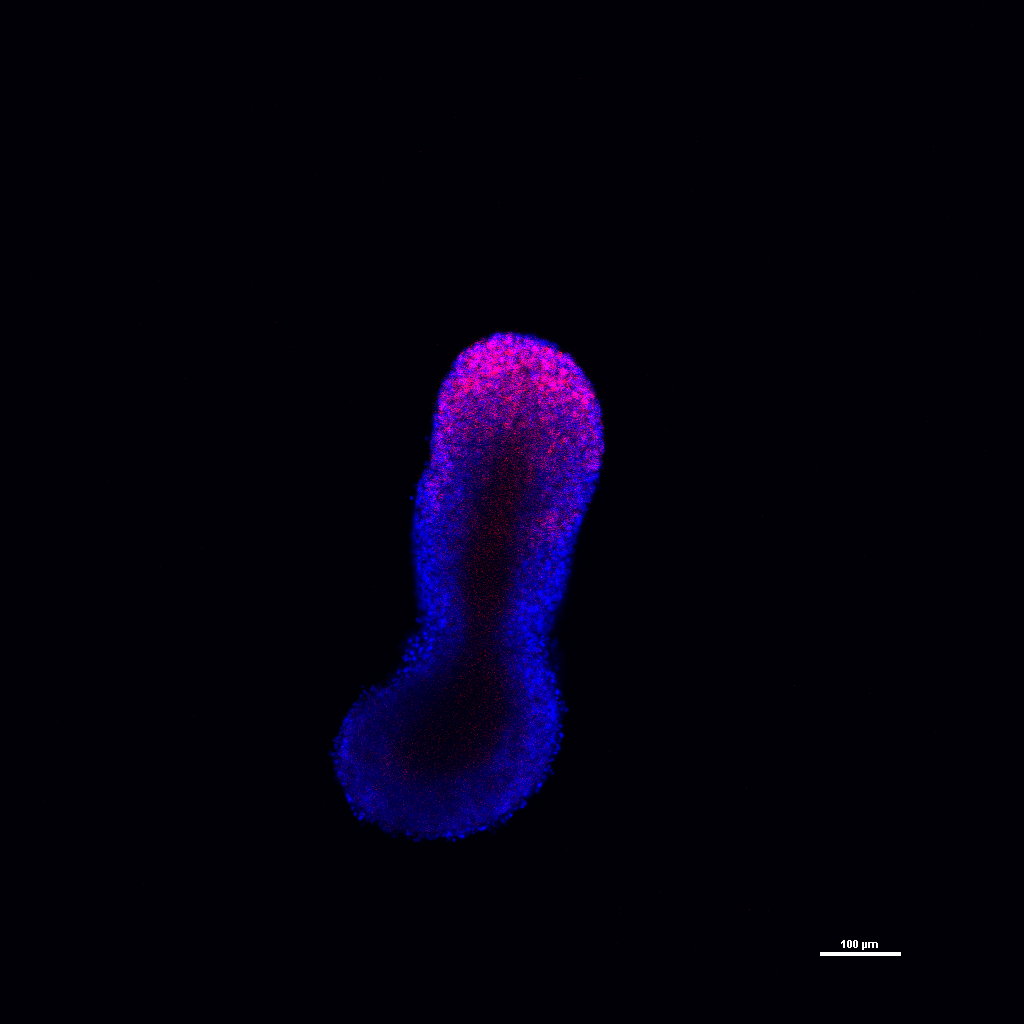

Supplement: Supplementary file 6 — Source data Fig. 4 [file 44318_2025_558_MOESM6_ESM.zip › Figure 4/panel 4B/KD-1_Bra_5uM/fila51874_RGB.tif]

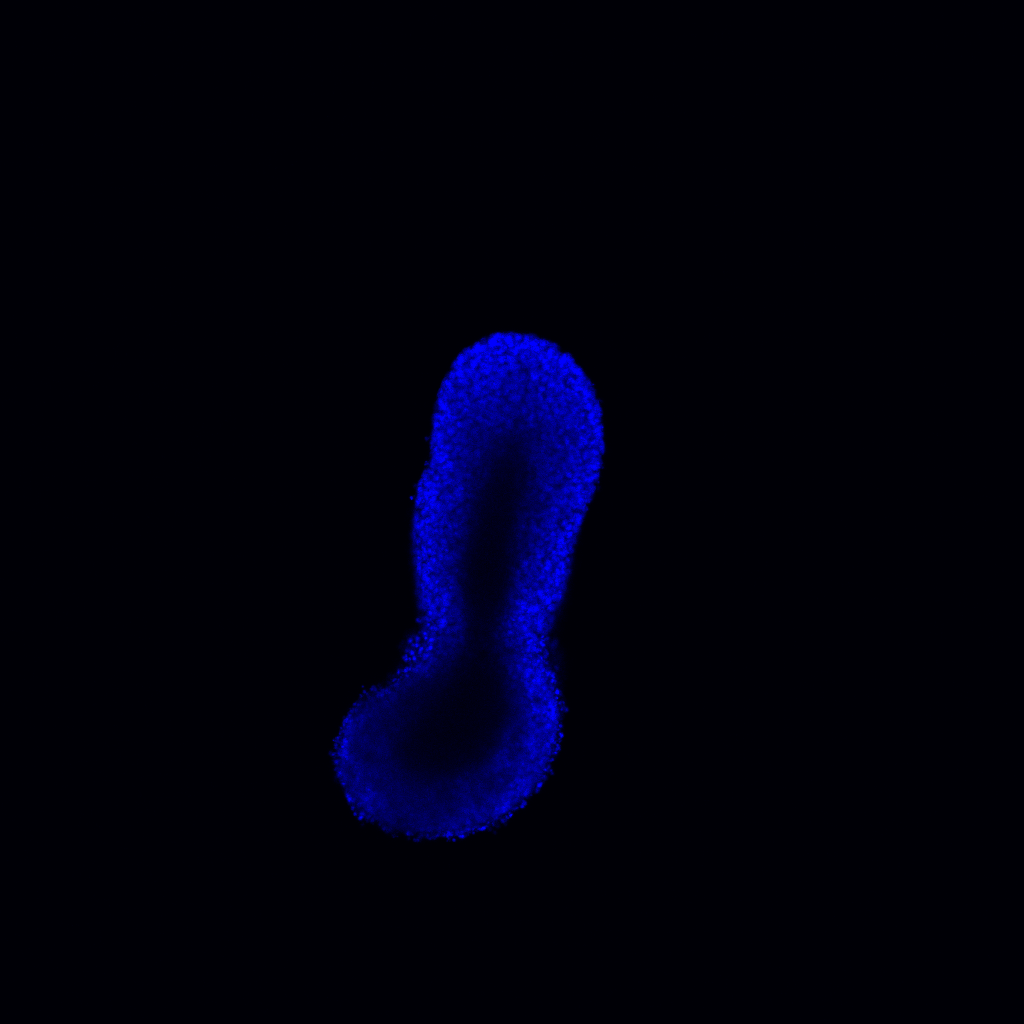

Supplement: Supplementary file 6 — Source data Fig. 4 [file 44318_2025_558_MOESM6_ESM.zip › Figure 4/panel 4B/KD-1_Bra_5uM/fila51874_RGB_DAPI.tif]

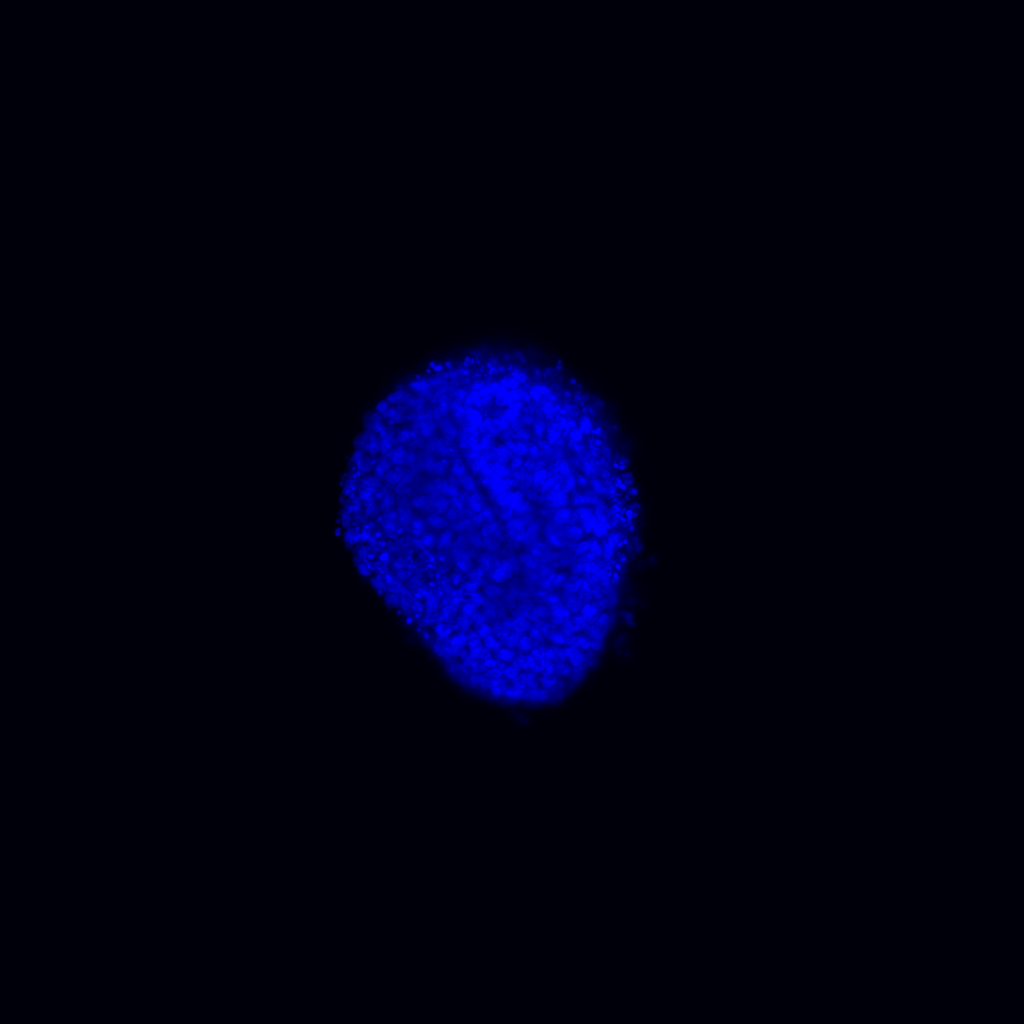

Supplement: Supplementary file 6 — Source data Fig. 4 [file 44318_2025_558_MOESM6_ESM.zip › Figure 4/panel 4B/KD-1_Oct4_6uM/seq9161_seq9161_RGB_DAPI.tif]

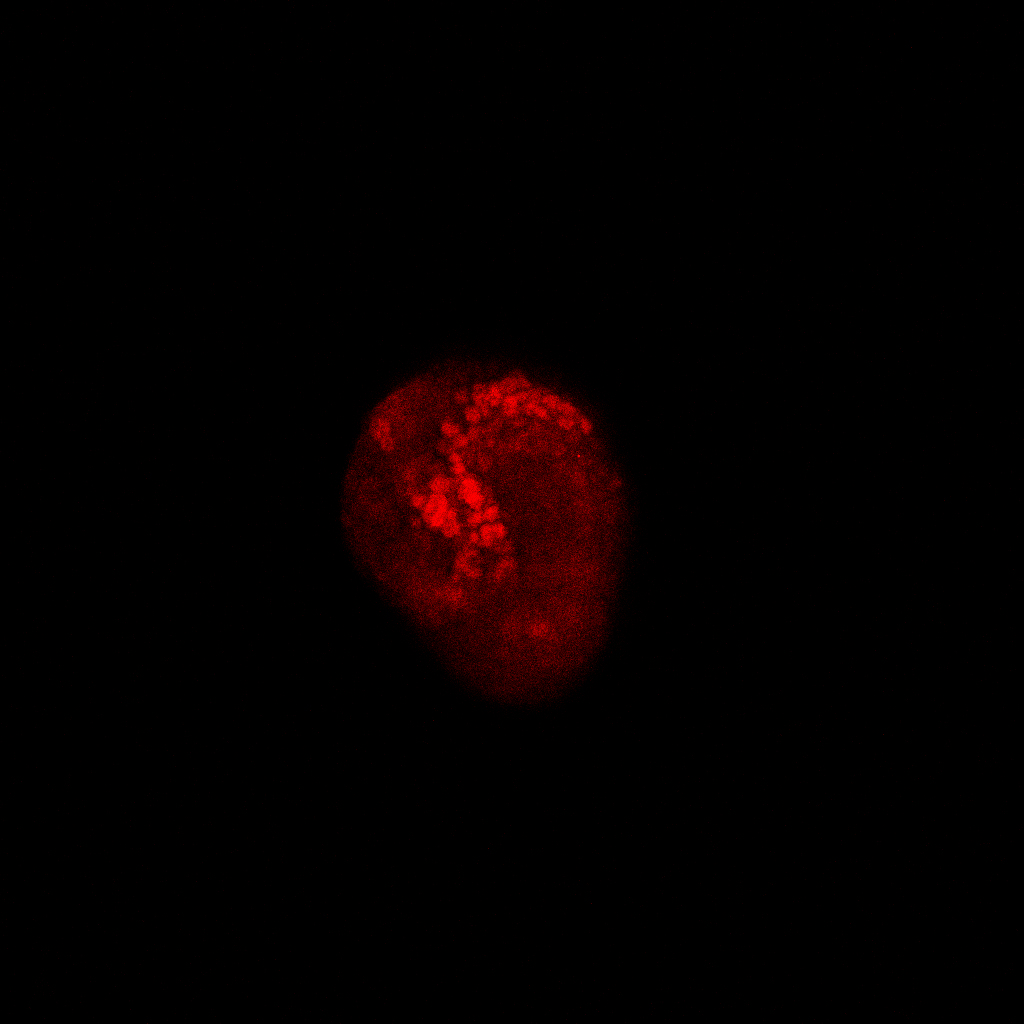

Supplement: Supplementary file 6 — Source data Fig. 4 [file 44318_2025_558_MOESM6_ESM.zip › Figure 4/panel 4B/KD-1_Oct4_6uM/seq9161_seq9161_RGB_Texas Red.tif]

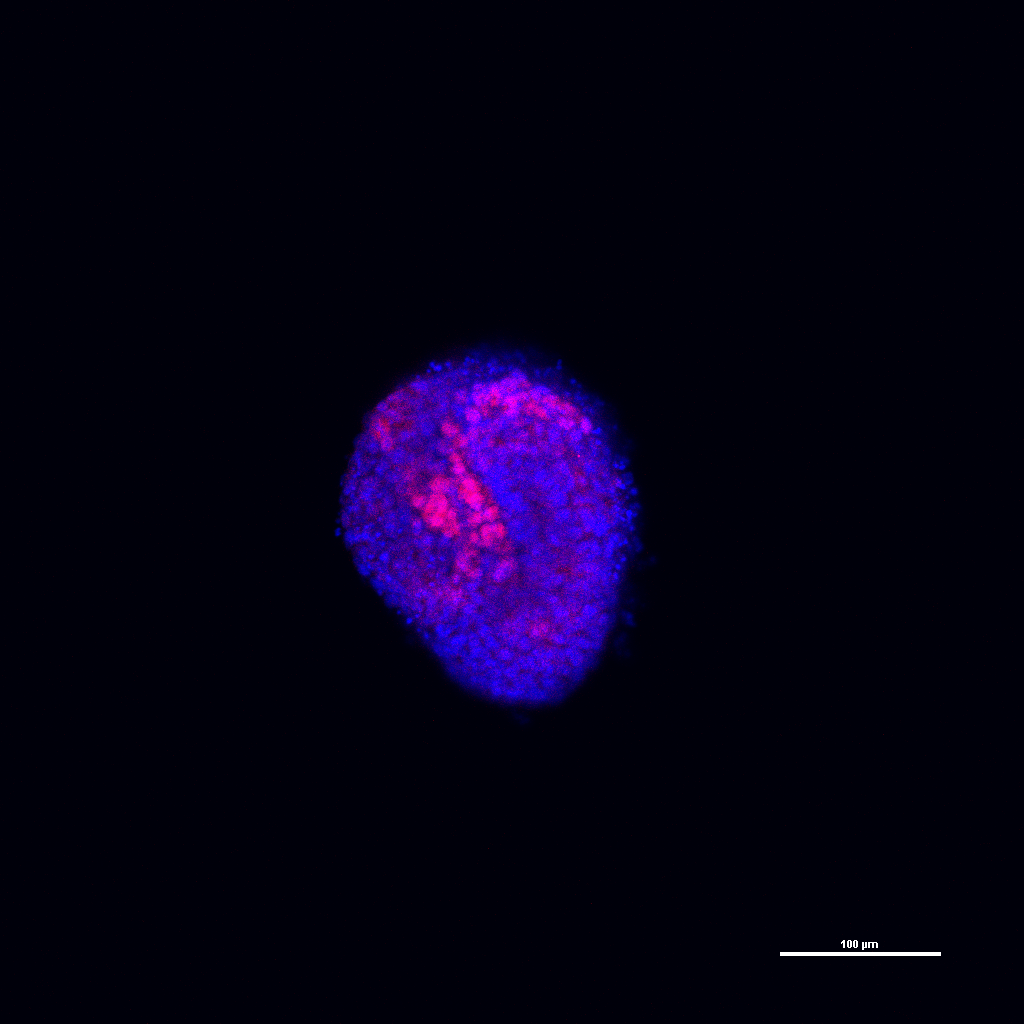

Supplement: Supplementary file 6 — Source data Fig. 4 [file 44318_2025_558_MOESM6_ESM.zip › Figure 4/panel 4B/KD-1_Oct4_6uM/seq9161_seq9161_RGB.tif]

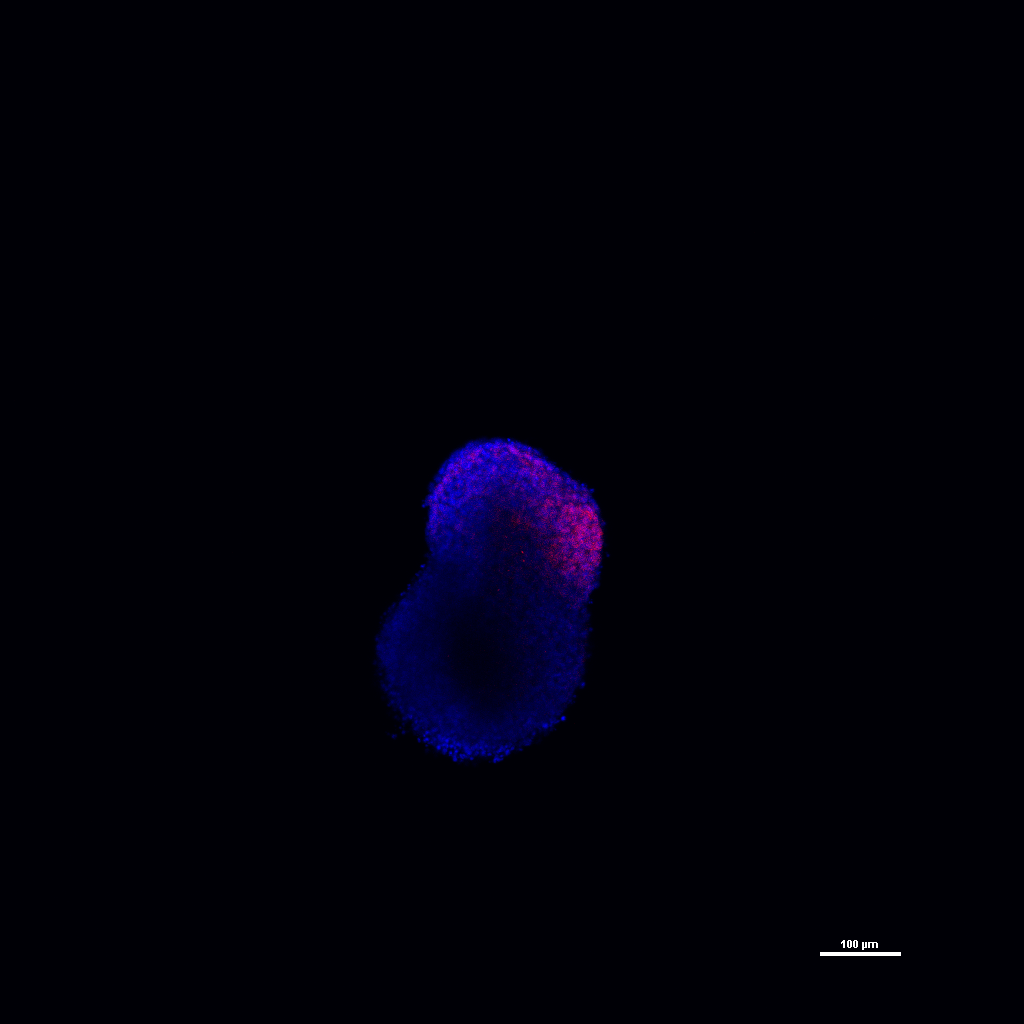

Supplement: Supplementary file 6 — Source data Fig. 4 [file 44318_2025_558_MOESM6_ESM.zip › Figure 4/panel 4B/NT_Nanog_5uM/fila51911_RGB.tif]

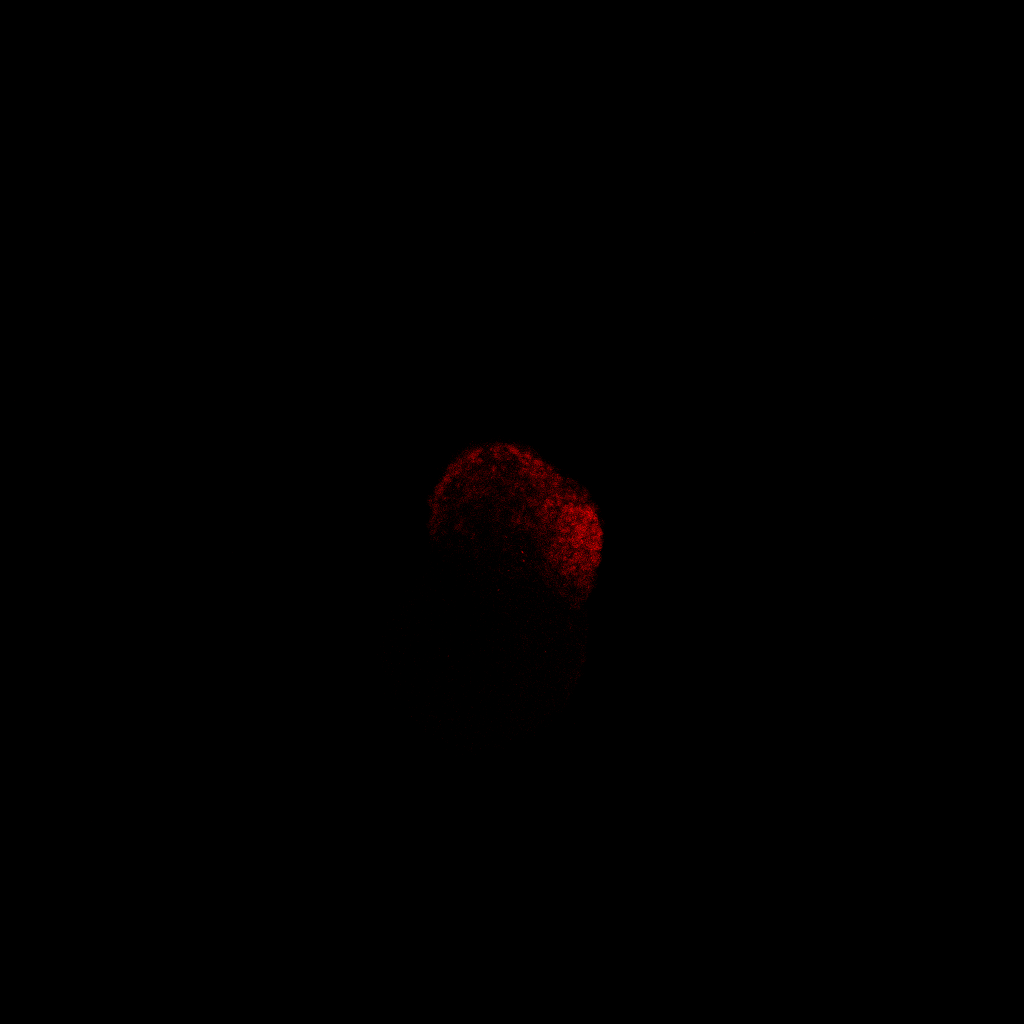

Supplement: Supplementary file 6 — Source data Fig. 4 [file 44318_2025_558_MOESM6_ESM.zip › Figure 4/panel 4B/NT_Nanog_5uM/fila51911_RGB_Alexa Fluor 594 cadaverine_H2O.tif]

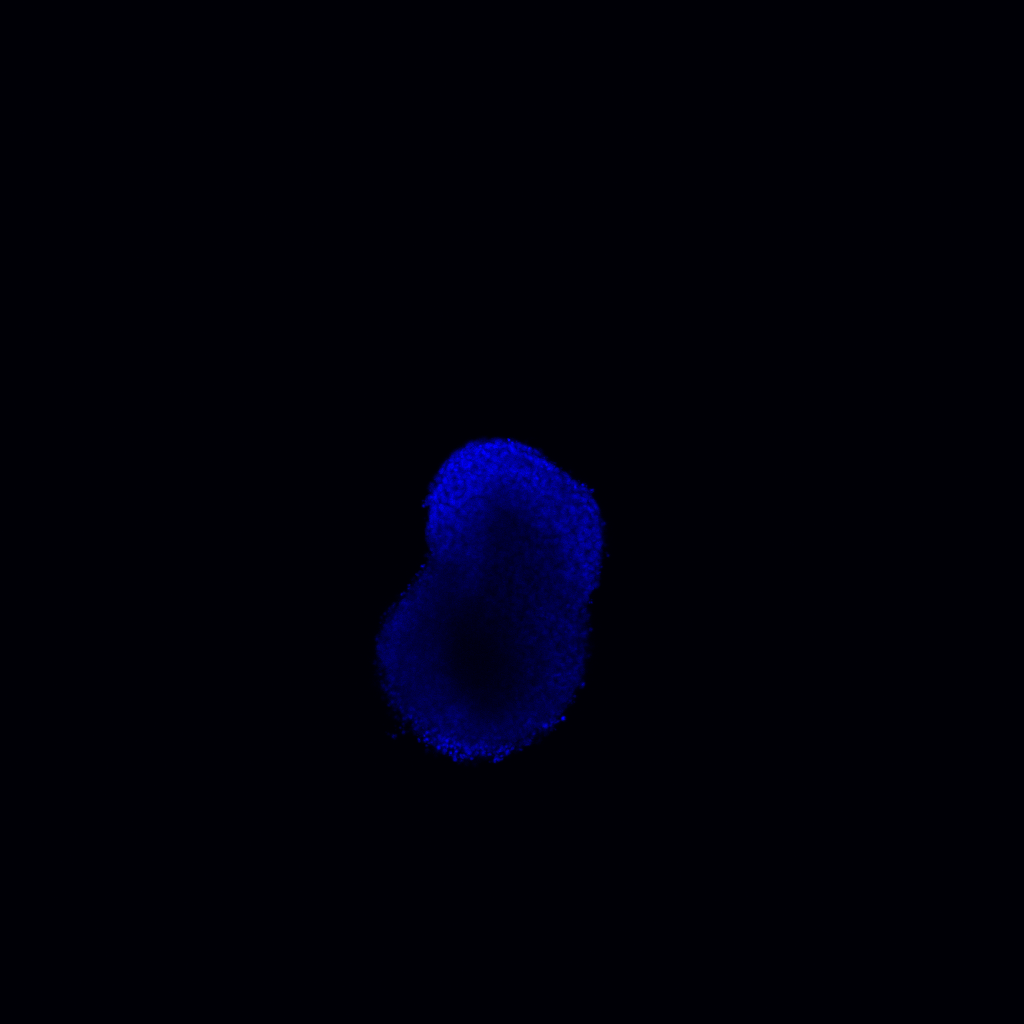

Supplement: Supplementary file 6 — Source data Fig. 4 [file 44318_2025_558_MOESM6_ESM.zip › Figure 4/panel 4B/NT_Nanog_5uM/fila51911_RGB_DAPI.tif]

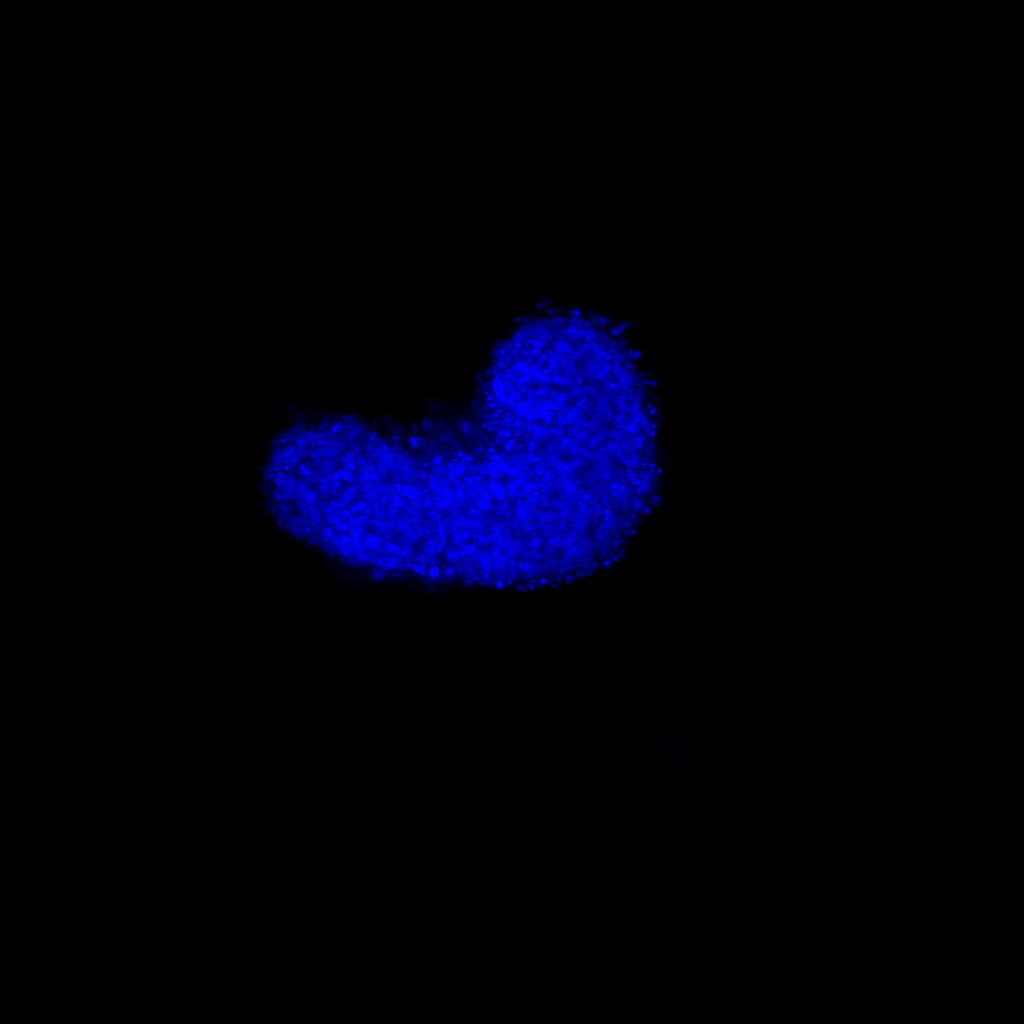

Supplement: Supplementary file 6 — Source data Fig. 4 [file 44318_2025_558_MOESM6_ESM.zip › Figure 4/panel 4B/KD-1_Cdx2_5uM/seq9891_seq9891_RGB_DAPI.tif]

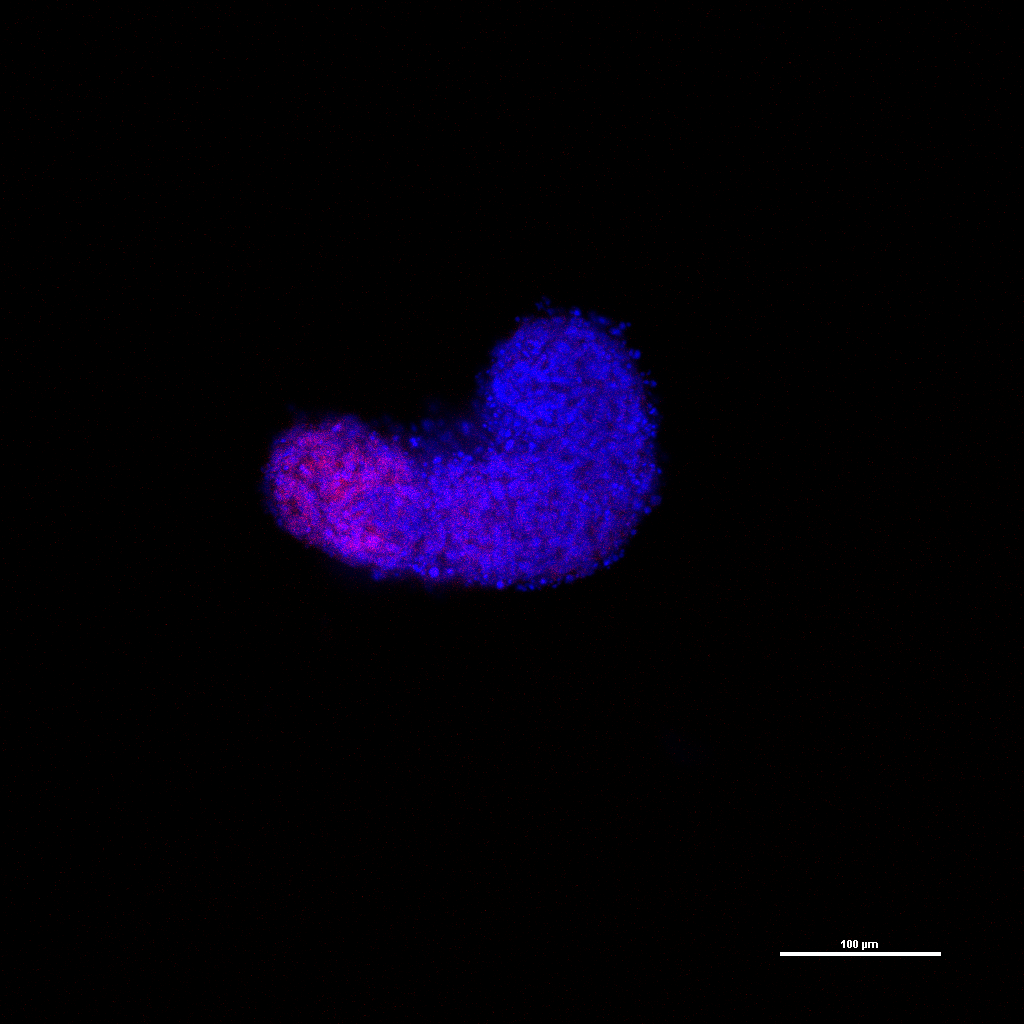

Supplement: Supplementary file 6 — Source data Fig. 4 [file 44318_2025_558_MOESM6_ESM.zip › Figure 4/panel 4B/KD-1_Cdx2_5uM/seq9891_seq9891_RGB.tif]

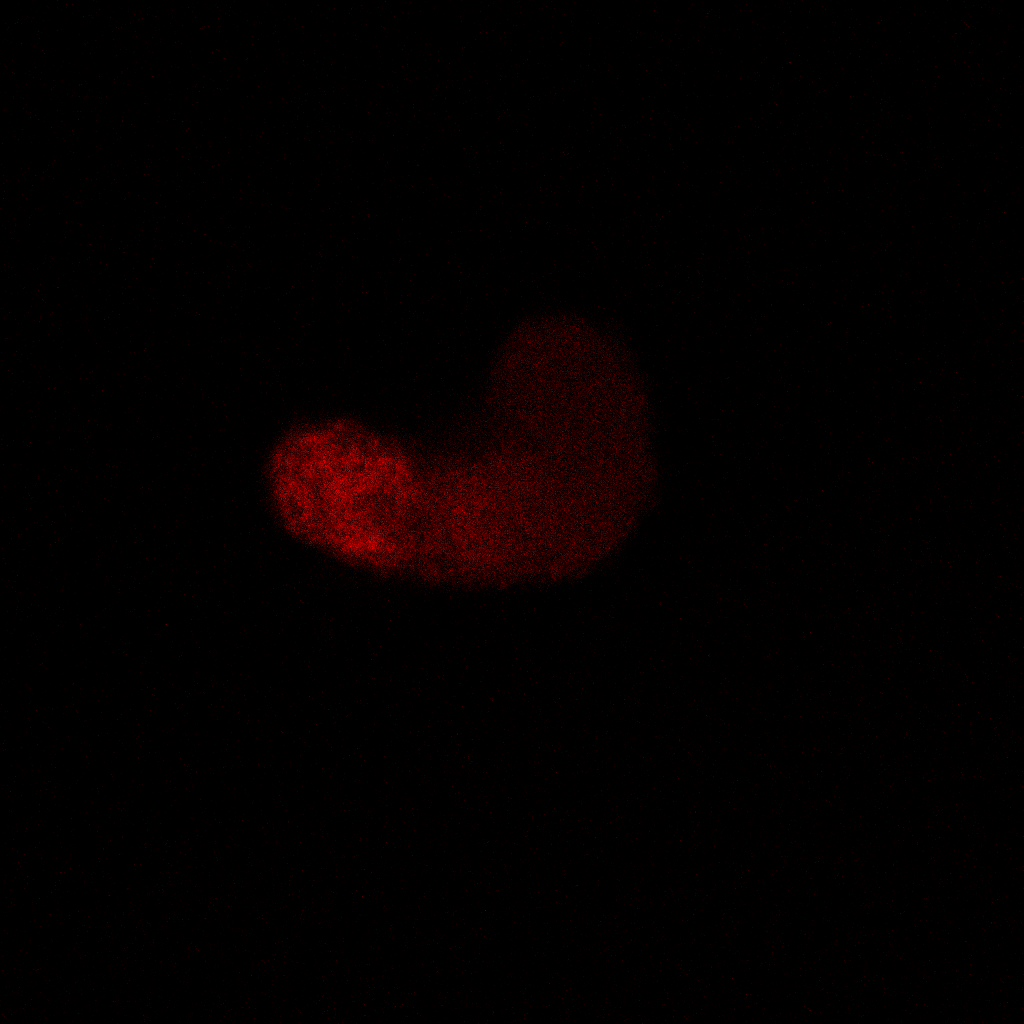

Supplement: Supplementary file 6 — Source data Fig. 4 [file 44318_2025_558_MOESM6_ESM.zip › Figure 4/panel 4B/KD-1_Cdx2_5uM/seq9891_seq9891_RGB_TRITC.tif]

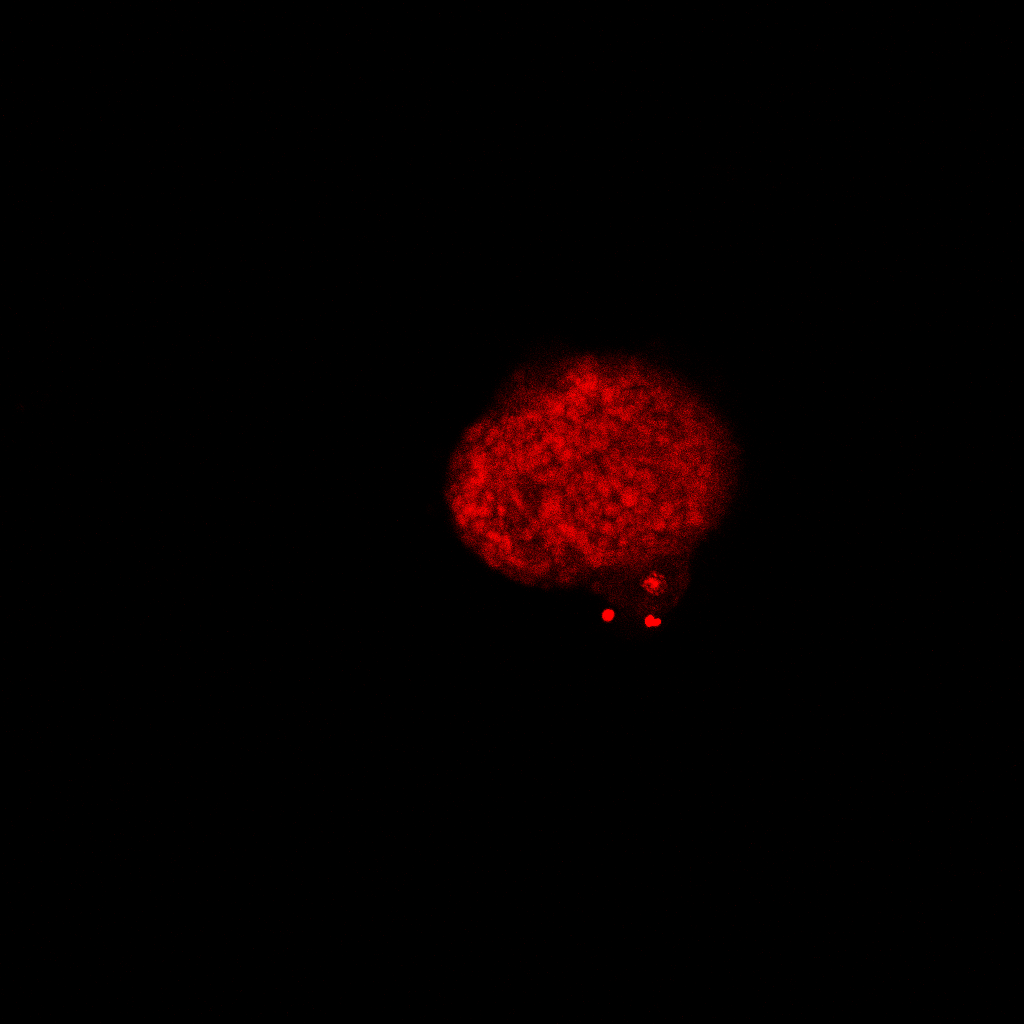

Supplement: Supplementary file 6 — Source data Fig. 4 [file 44318_2025_558_MOESM6_ESM.zip › Figure 4/panel 4B/KD-1_Nanog_6uM/seq9190_seq9190_RGB_Texas Red.tif]

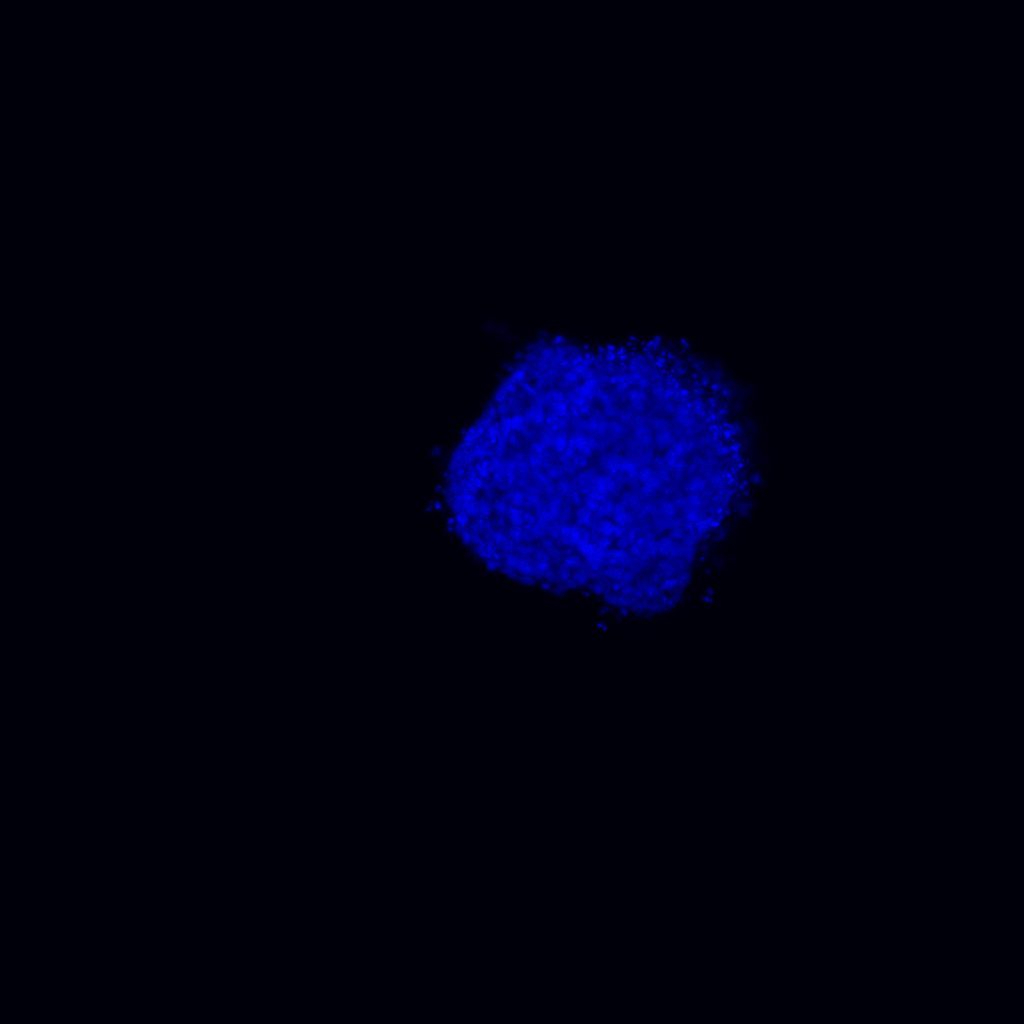

Supplement: Supplementary file 6 — Source data Fig. 4 [file 44318_2025_558_MOESM6_ESM.zip › Figure 4/panel 4B/KD-1_Nanog_6uM/seq9190_seq9190_RGB_DAPI.tif]

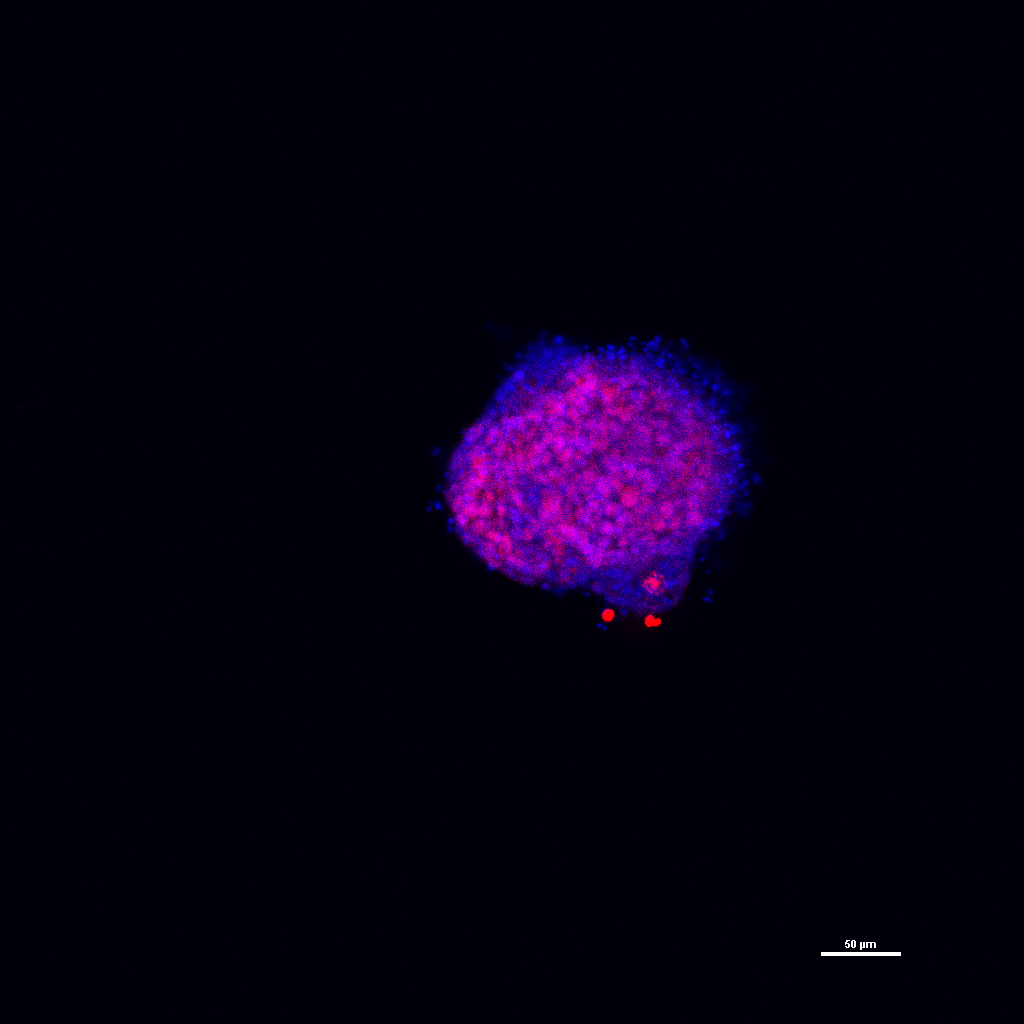

Supplement: Supplementary file 6 — Source data Fig. 4 [file 44318_2025_558_MOESM6_ESM.zip › Figure 4/panel 4B/KD-1_Nanog_6uM/seq9190_seq9190_RGB.tif]

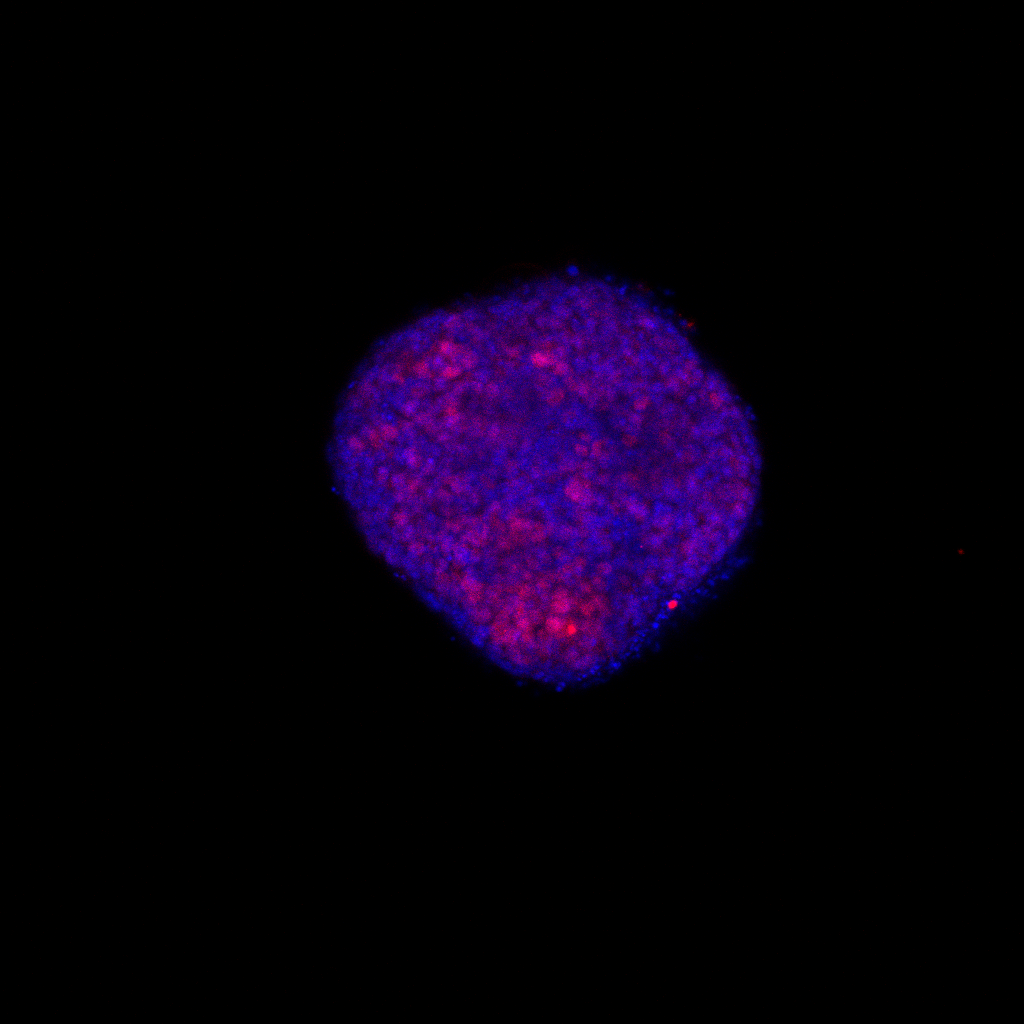

Supplement: Supplementary file 6 — Source data Fig. 4 [file 44318_2025_558_MOESM6_ESM.zip › Figure 4/panel 4B/KD-2_Nanog_3uM/seq8693.tif]
