# Supplementary figures and images for: Non-cell-autonomous control of mouse gastruloid development by the ultra-conserved lncRNA T-UCstem1 (part 4 of 6)
Source: EMBO J. 2025 Oct 31;44(24):7620–48. doi: 10.1038/s44318-025-00558-2 (PMC12706062; doi:10.1038/s44318-025-00558-2)

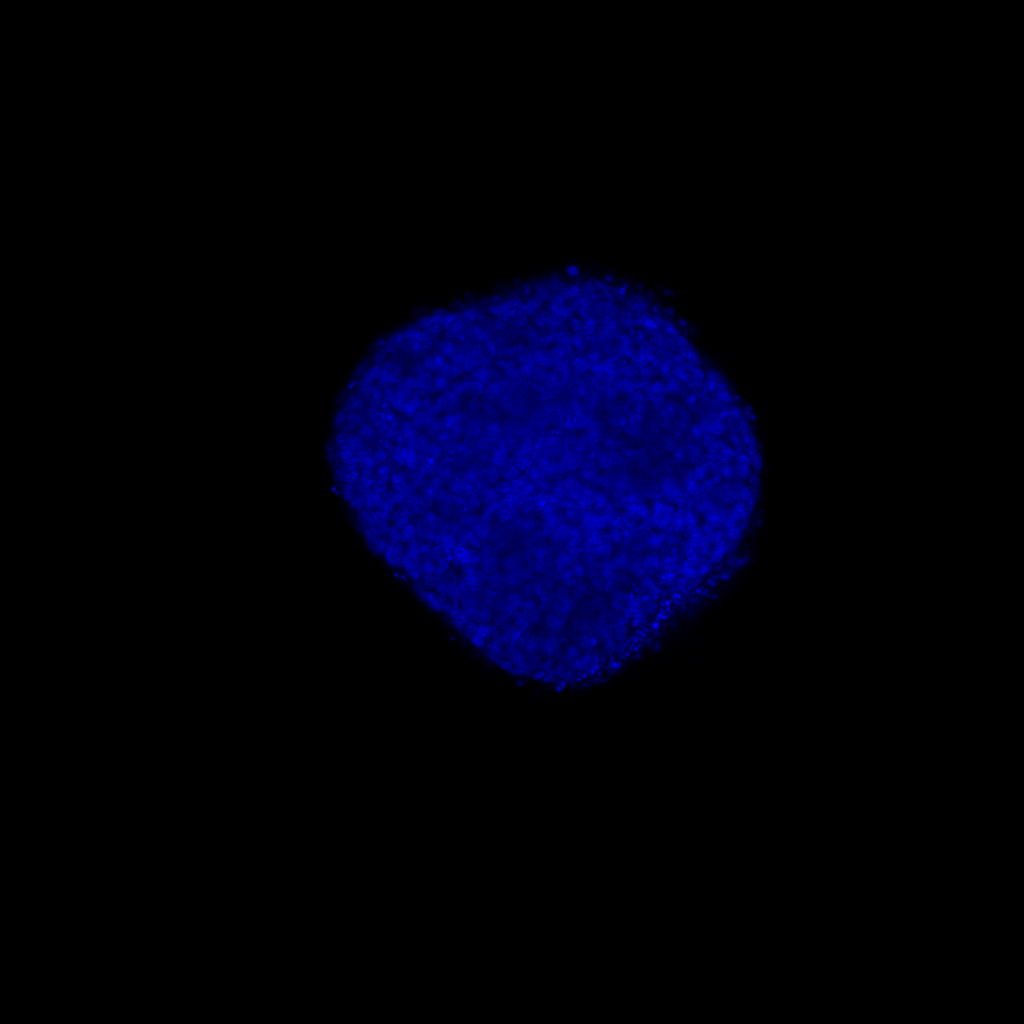

Supplement: Supplementary file 6 — Source data Fig. 4 [file 44318_2025_558_MOESM6_ESM.zip › Figure 4/panel 4B/KD-2_Nanog_3uM/seq8693c1.tif]

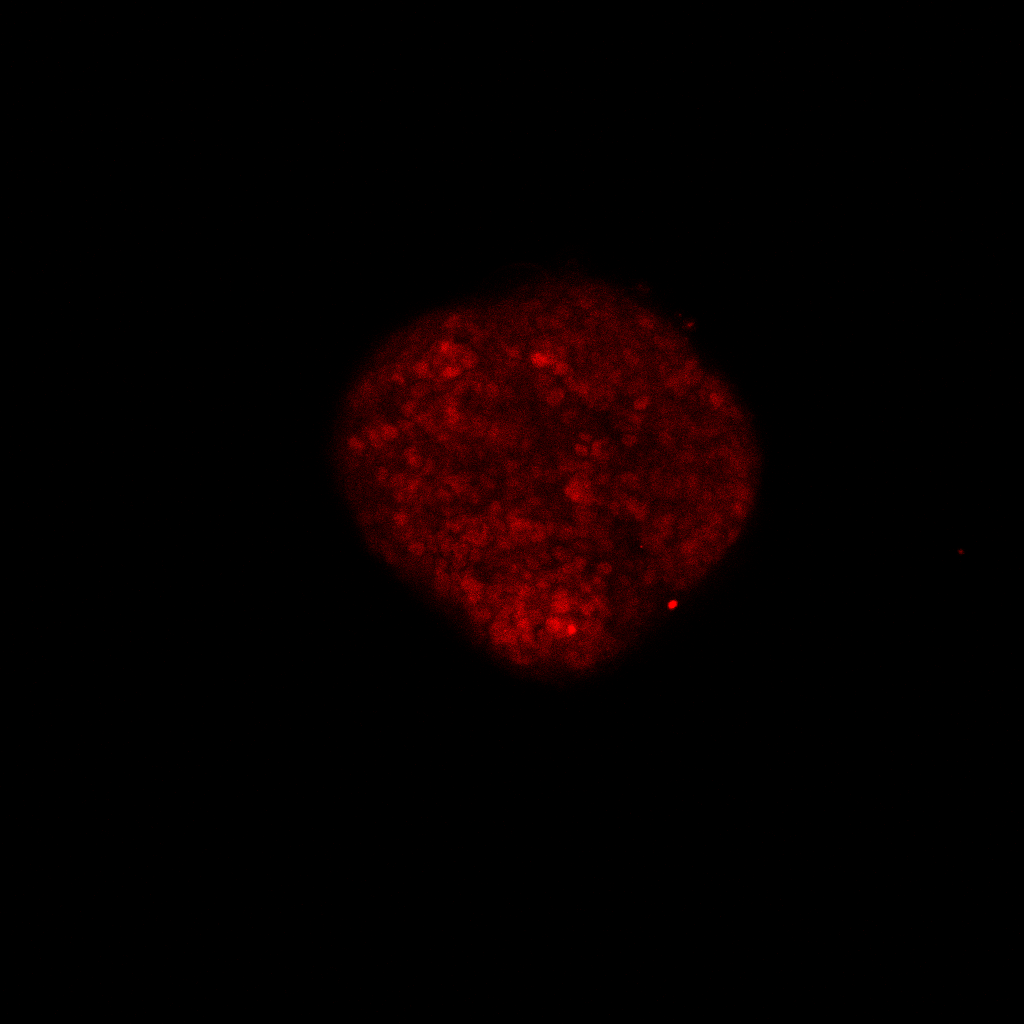

Supplement: Supplementary file 6 — Source data Fig. 4 [file 44318_2025_558_MOESM6_ESM.zip › Figure 4/panel 4B/KD-2_Nanog_3uM/seq8693c2.tif]

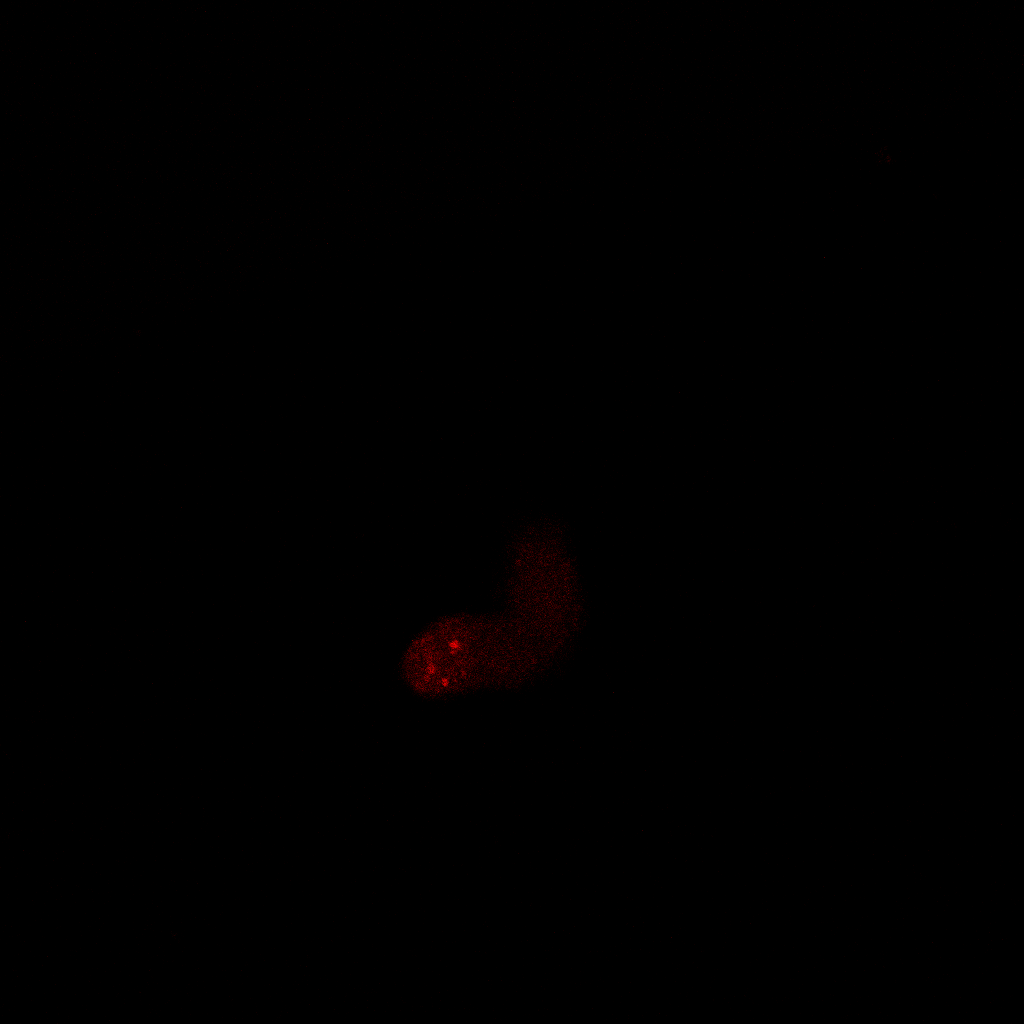

Supplement: Supplementary file 6 — Source data Fig. 4 [file 44318_2025_558_MOESM6_ESM.zip › Figure 4/panel 4B/NT_Bra_6uM/fila51890_RGB_Alexa Fluor 594 cadaverine_H2O.tif]

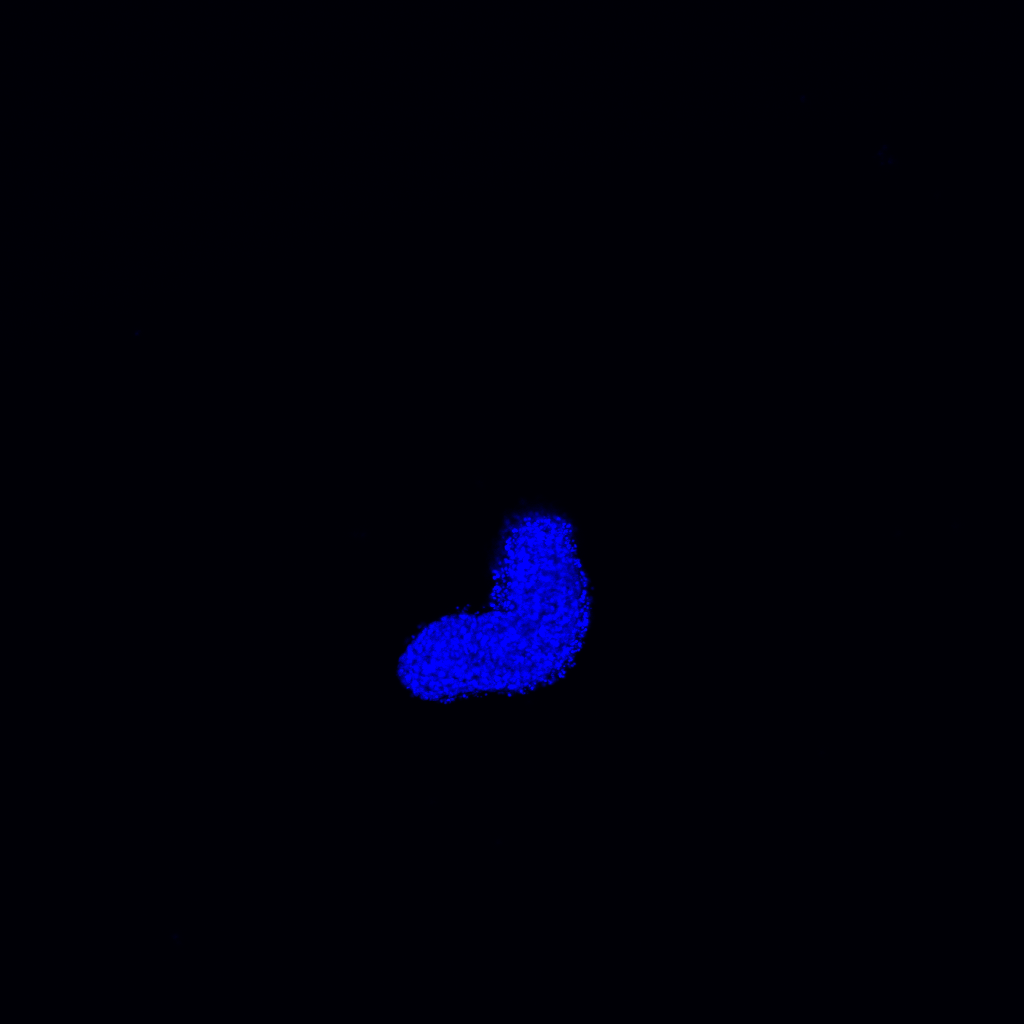

Supplement: Supplementary file 6 — Source data Fig. 4 [file 44318_2025_558_MOESM6_ESM.zip › Figure 4/panel 4B/NT_Bra_6uM/fila51890_RGB_DAPI.tif]

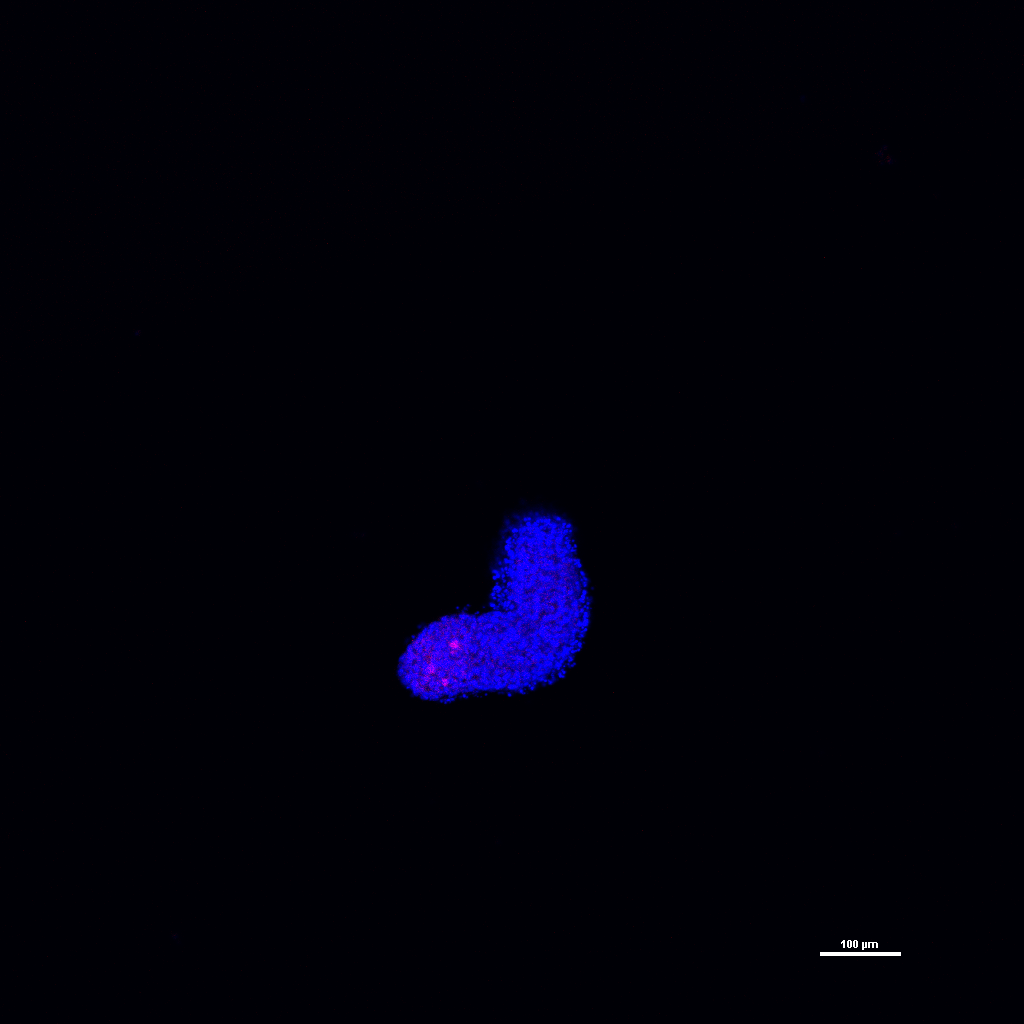

Supplement: Supplementary file 6 — Source data Fig. 4 [file 44318_2025_558_MOESM6_ESM.zip › Figure 4/panel 4B/NT_Bra_6uM/fila51890_RGB.tif]

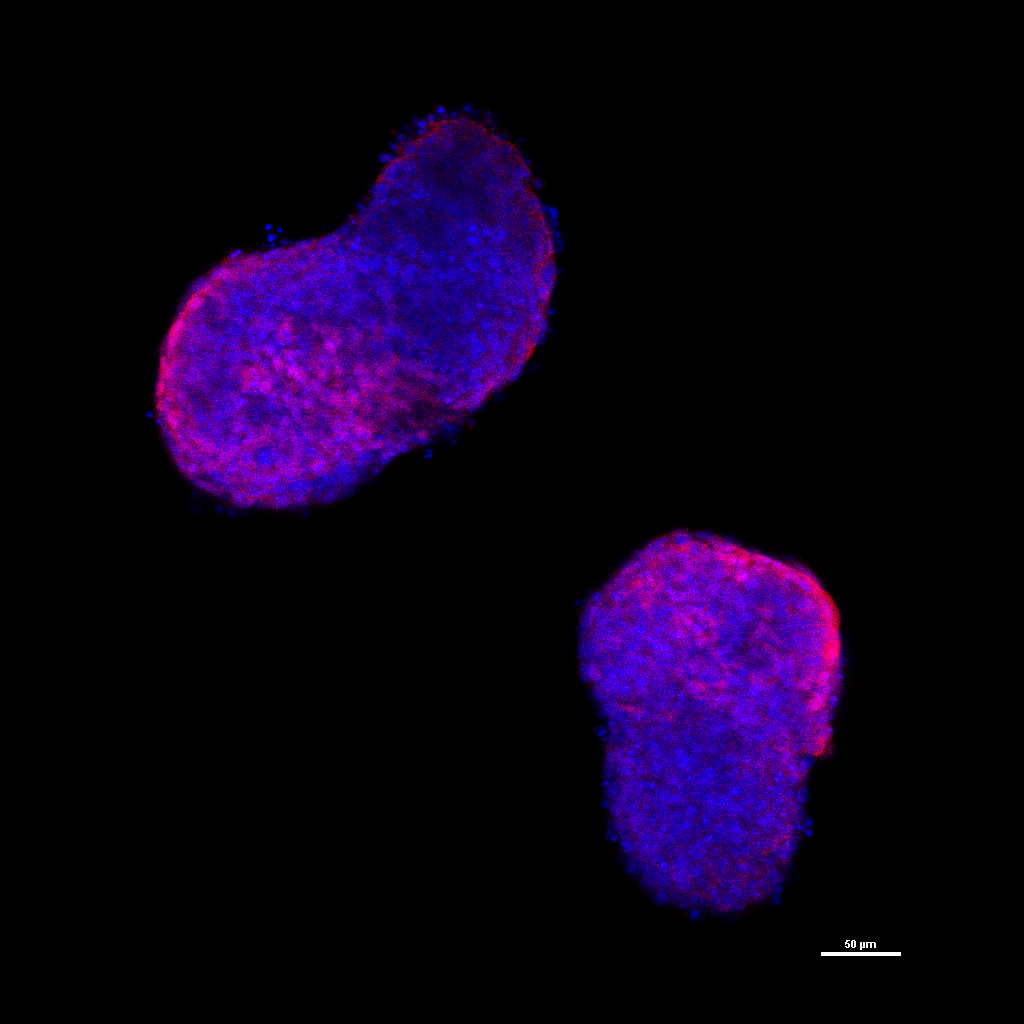

Supplement: Supplementary file 6 — Source data Fig. 4 [file 44318_2025_558_MOESM6_ESM.zip › Figure 4/panel 4B/KD-2_Cdx2_6uM/seq9904_seq9904_RGB.tif]

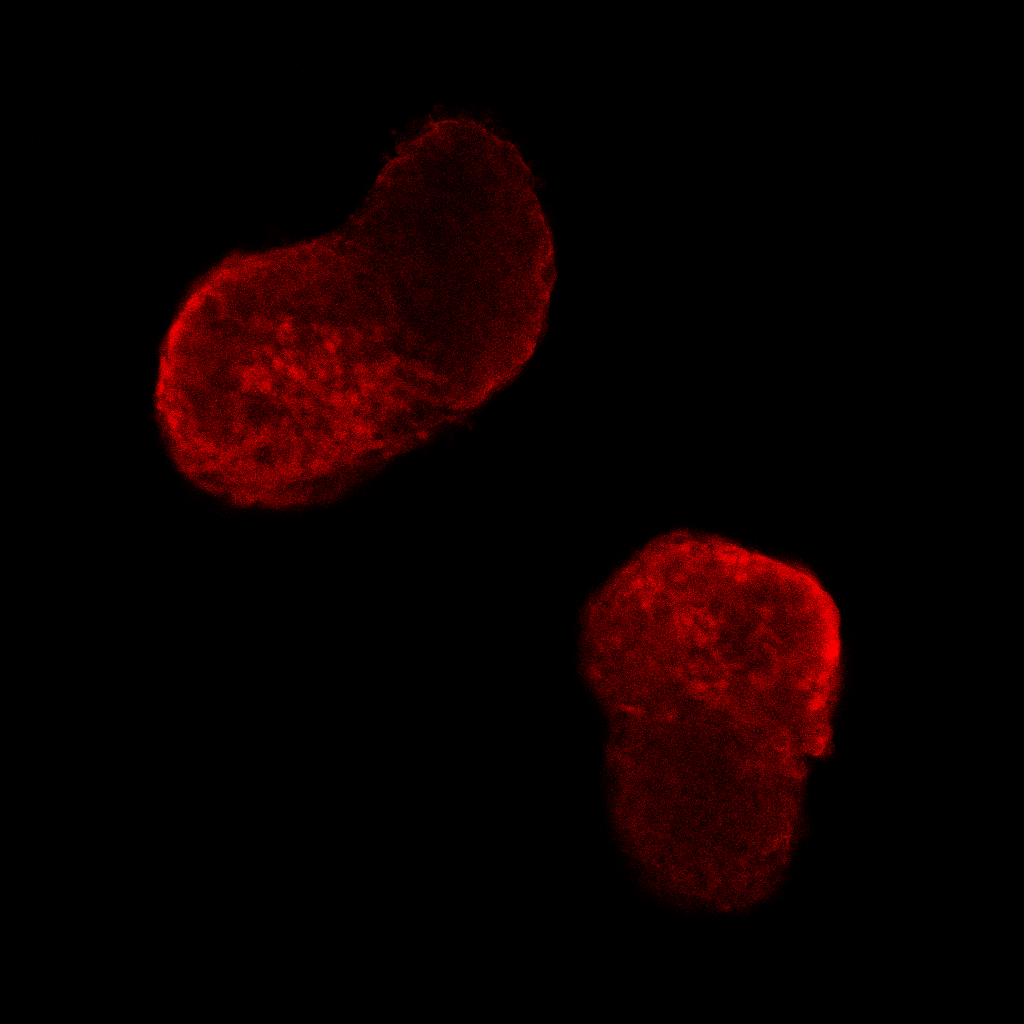

Supplement: Supplementary file 6 — Source data Fig. 4 [file 44318_2025_558_MOESM6_ESM.zip › Figure 4/panel 4B/KD-2_Cdx2_6uM/seq9904_seq9904_RGB_TRITC.tif]

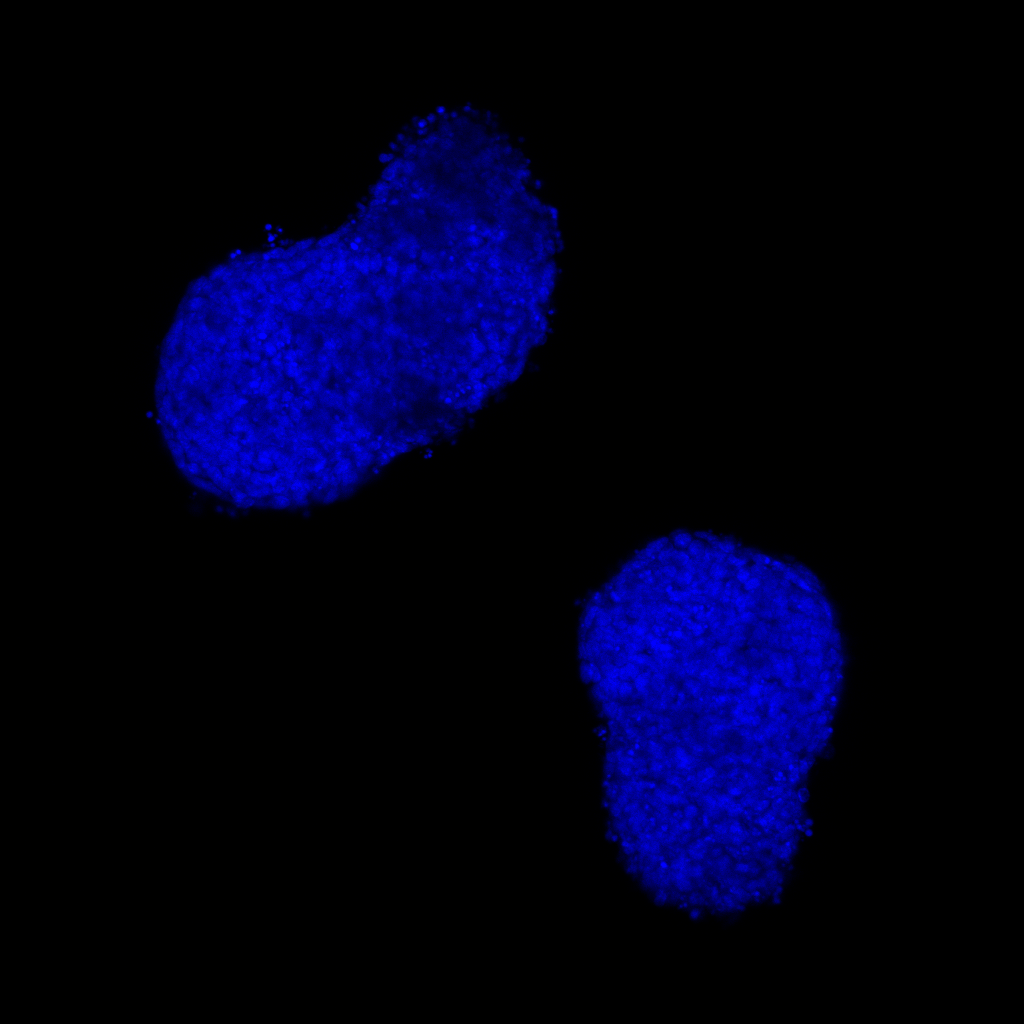

Supplement: Supplementary file 6 — Source data Fig. 4 [file 44318_2025_558_MOESM6_ESM.zip › Figure 4/panel 4B/KD-2_Cdx2_6uM/seq9904_seq9904_RGB_DAPI.tif]

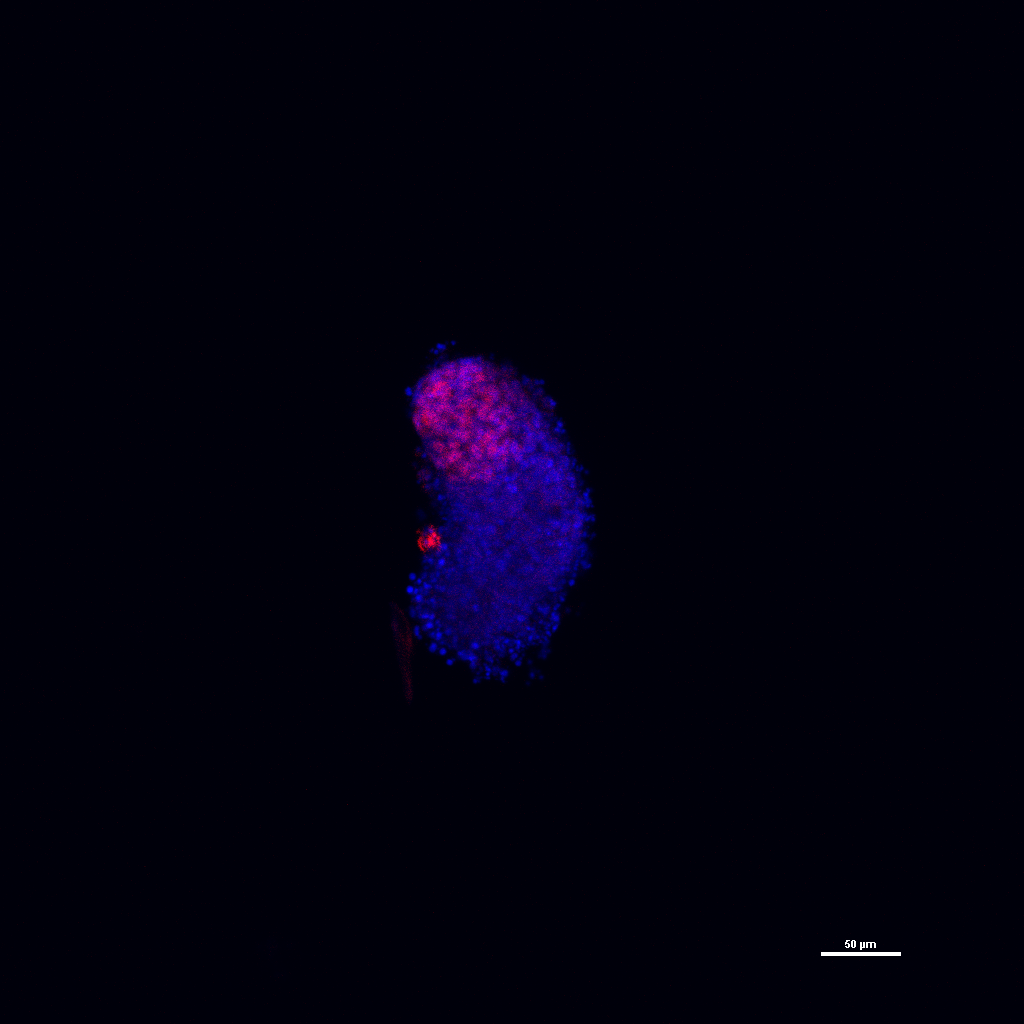

Supplement: Supplementary file 6 — Source data Fig. 4 [file 44318_2025_558_MOESM6_ESM.zip › Figure 4/panel 4B/NT_Cdx2_5uM/seq9177_seq9177_RGB.tif]

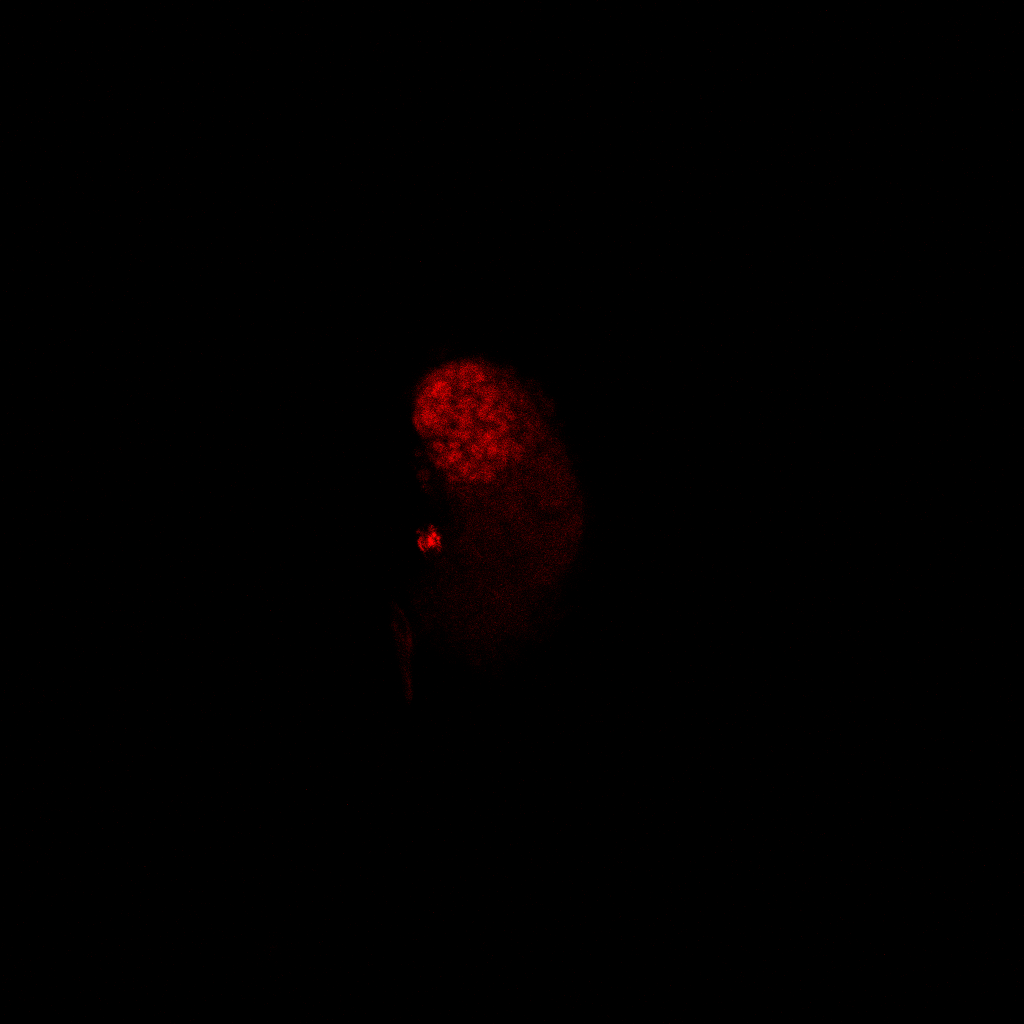

Supplement: Supplementary file 6 — Source data Fig. 4 [file 44318_2025_558_MOESM6_ESM.zip › Figure 4/panel 4B/NT_Cdx2_5uM/seq9177_seq9177_RGB_Texas Red.tif]

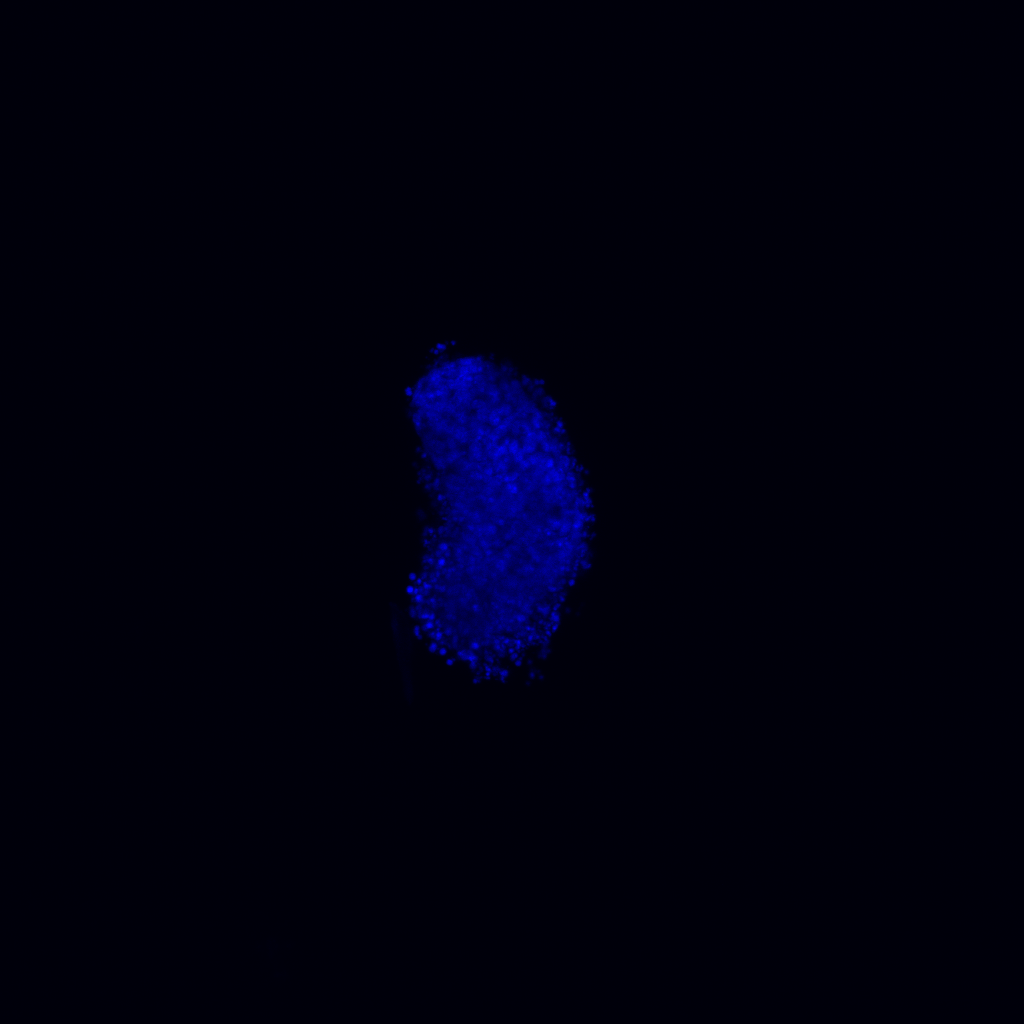

Supplement: Supplementary file 6 — Source data Fig. 4 [file 44318_2025_558_MOESM6_ESM.zip › Figure 4/panel 4B/NT_Cdx2_5uM/seq9177_seq9177_RGB_DAPI.tif]

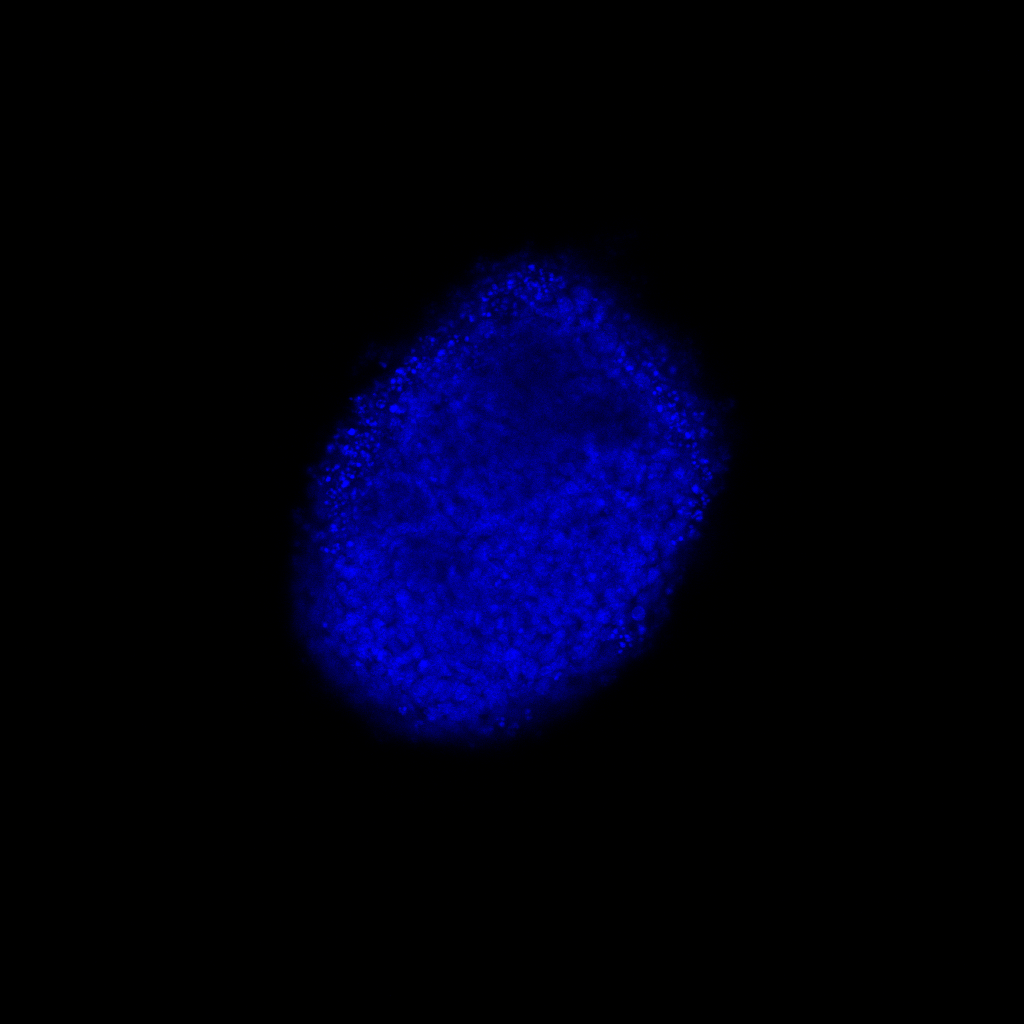

Supplement: Supplementary file 6 — Source data Fig. 4 [file 44318_2025_558_MOESM6_ESM.zip › Figure 4/panel 4B/KD-2_Bra_3uM/seq8687c1.tif]

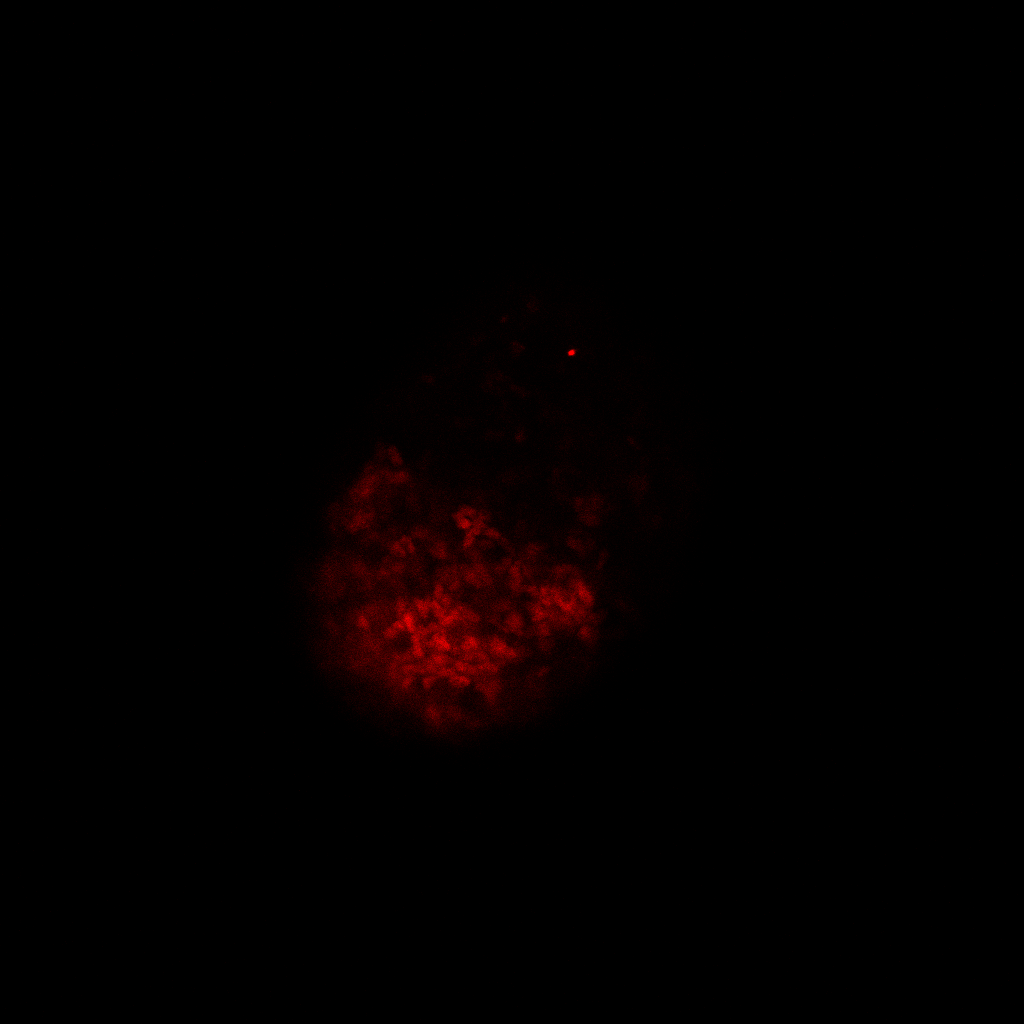

Supplement: Supplementary file 6 — Source data Fig. 4 [file 44318_2025_558_MOESM6_ESM.zip › Figure 4/panel 4B/KD-2_Bra_3uM/seq8687c2.tif]

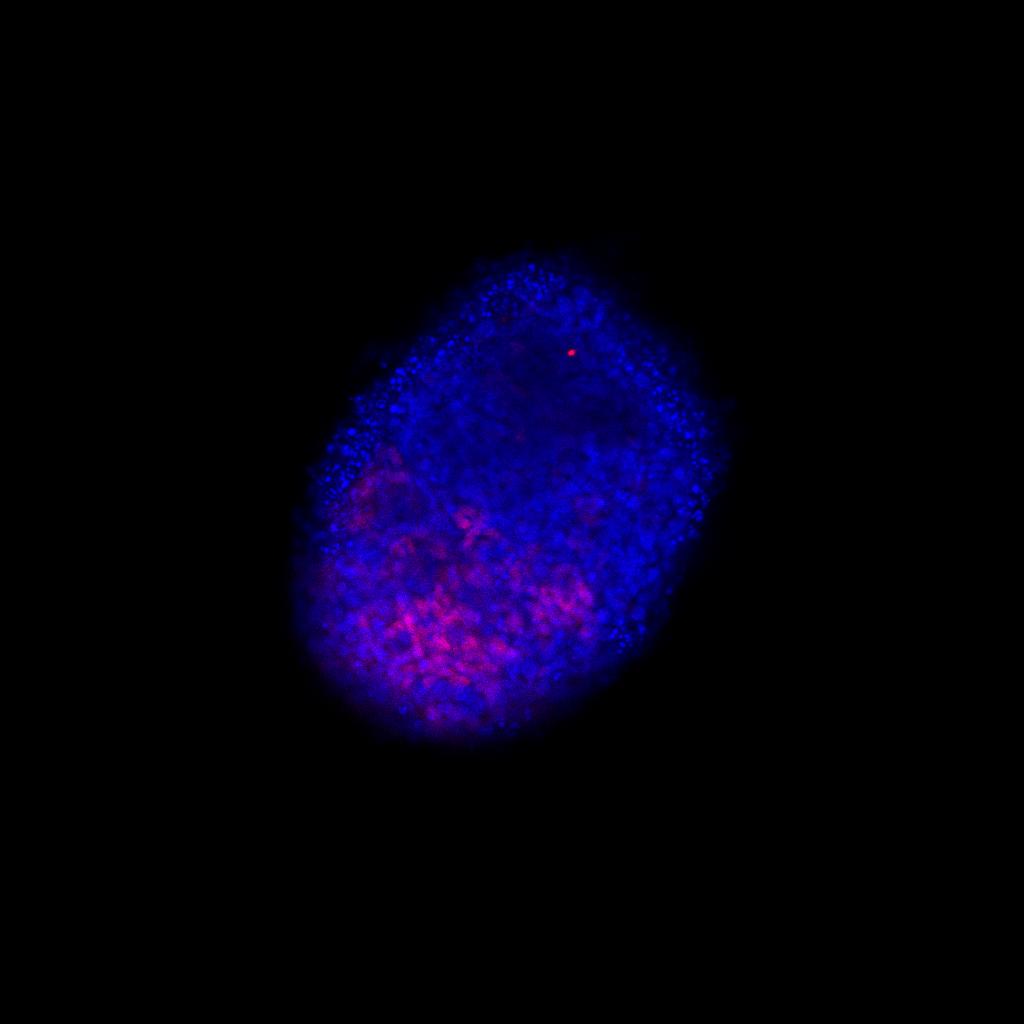

Supplement: Supplementary file 6 — Source data Fig. 4 [file 44318_2025_558_MOESM6_ESM.zip › Figure 4/panel 4B/KD-2_Bra_3uM/seq8687.tif]

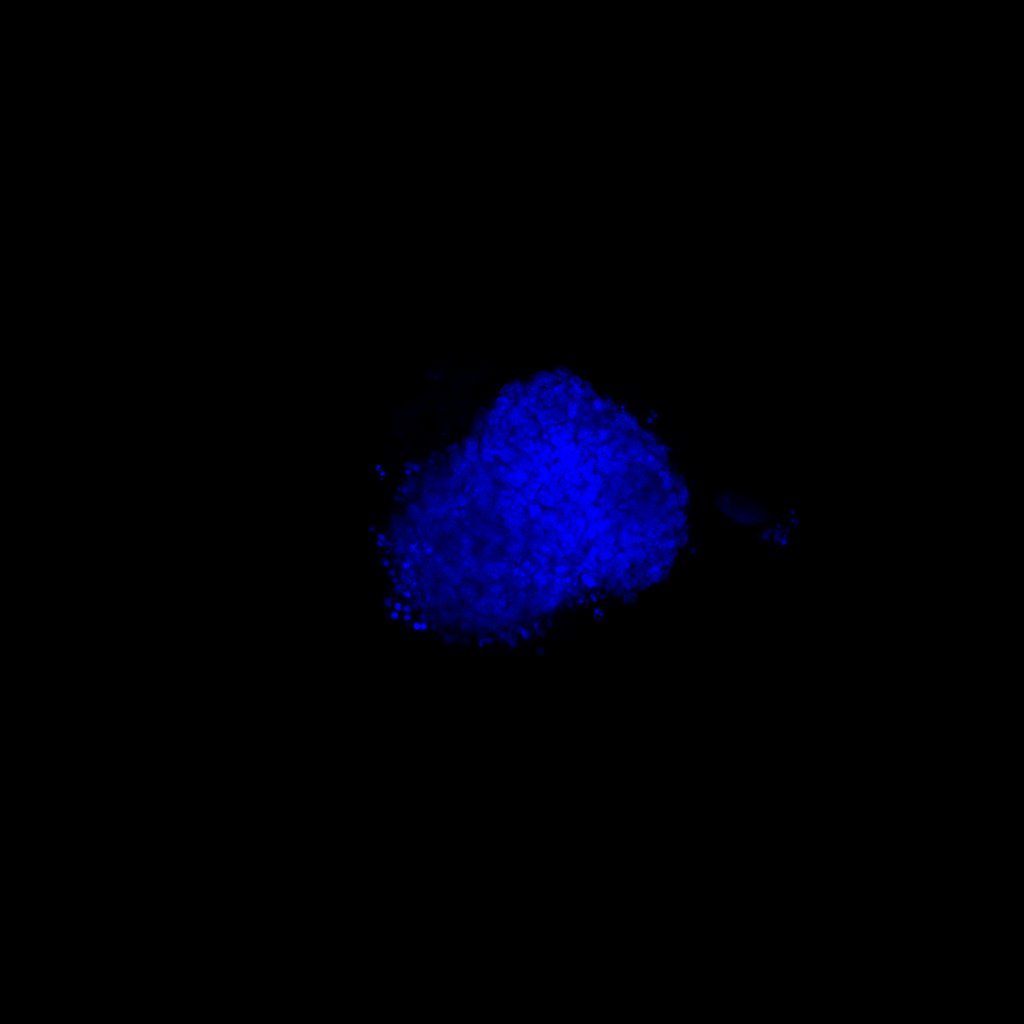

Supplement: Supplementary file 6 — Source data Fig. 4 [file 44318_2025_558_MOESM6_ESM.zip › Figure 4/panel 4B/KD-2_Oct4_5uM/seq9870_seq9870_RGB_DAPI.tif]

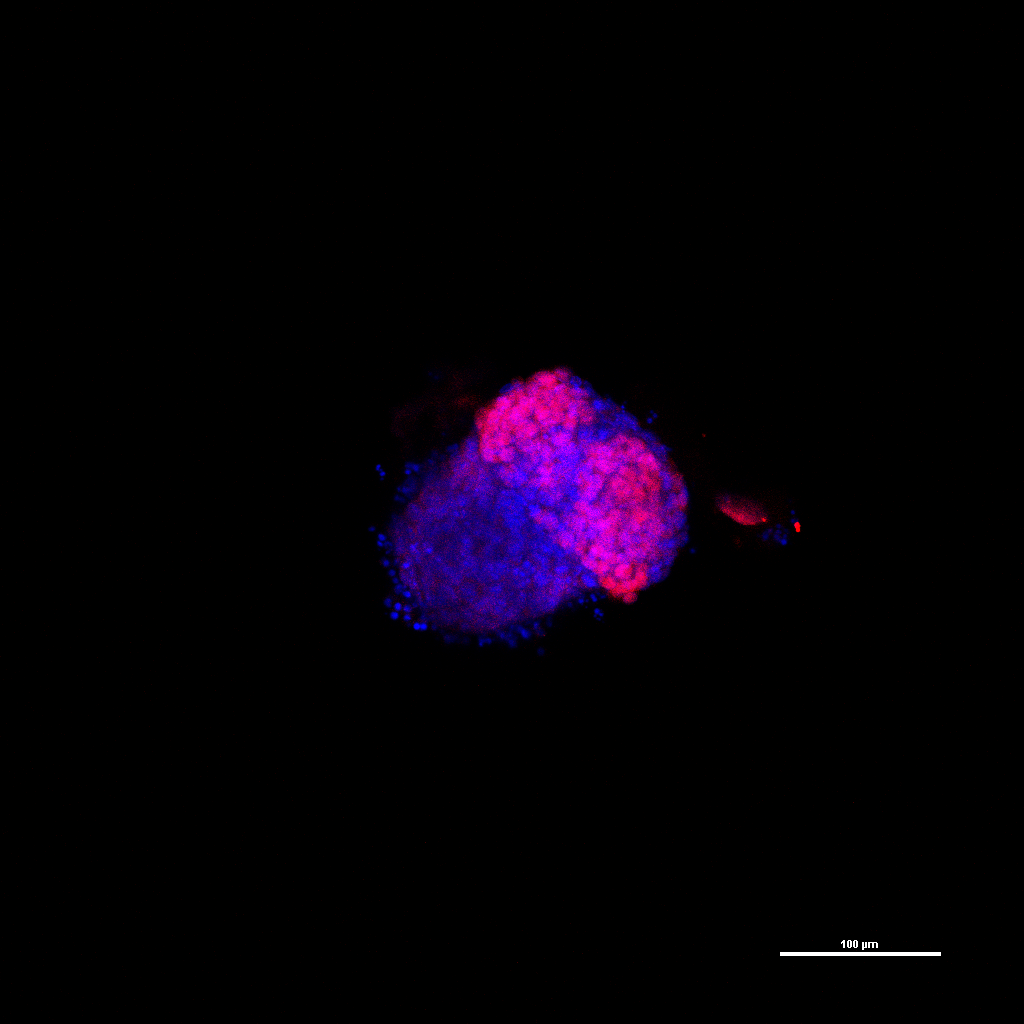

Supplement: Supplementary file 6 — Source data Fig. 4 [file 44318_2025_558_MOESM6_ESM.zip › Figure 4/panel 4B/KD-2_Oct4_5uM/seq9870_seq9870_RGB.tif]

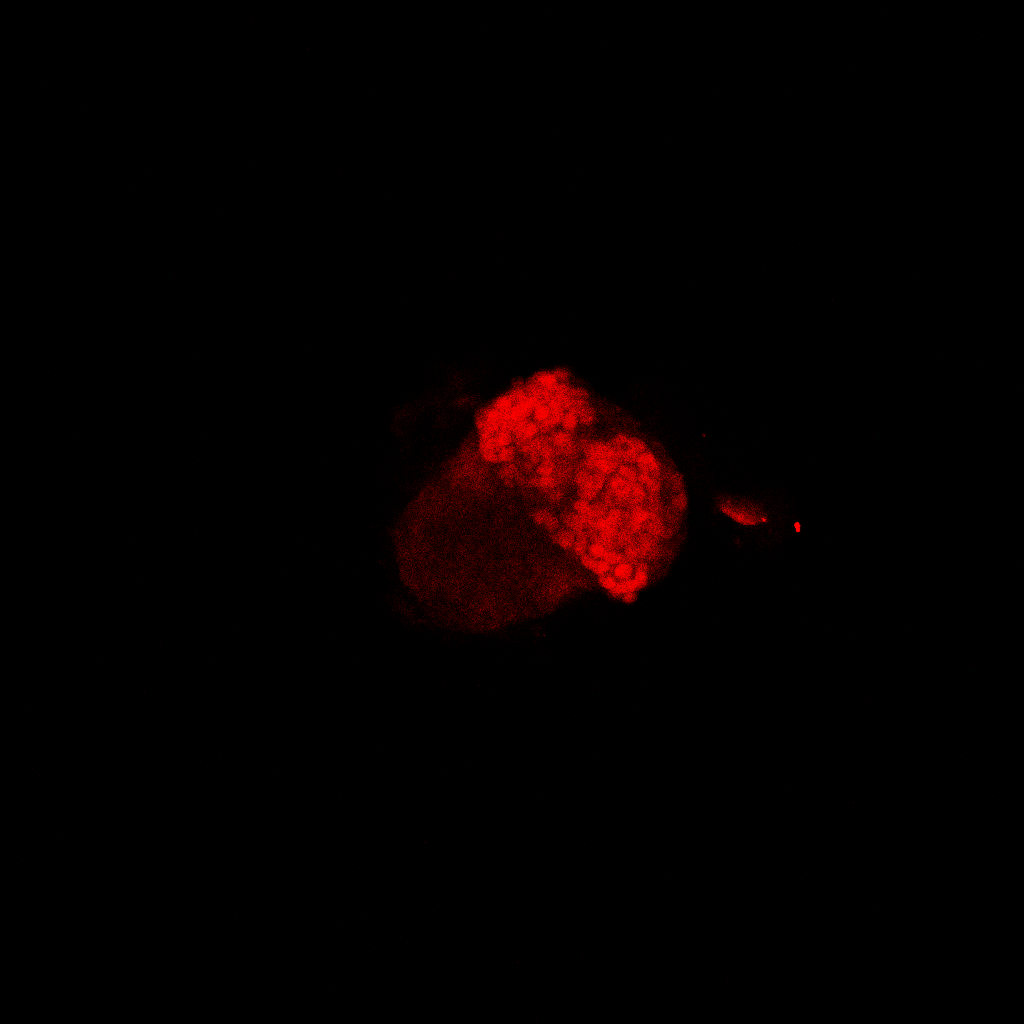

Supplement: Supplementary file 6 — Source data Fig. 4 [file 44318_2025_558_MOESM6_ESM.zip › Figure 4/panel 4B/KD-2_Oct4_5uM/seq9870_seq9870_RGB_TRITC.tif]

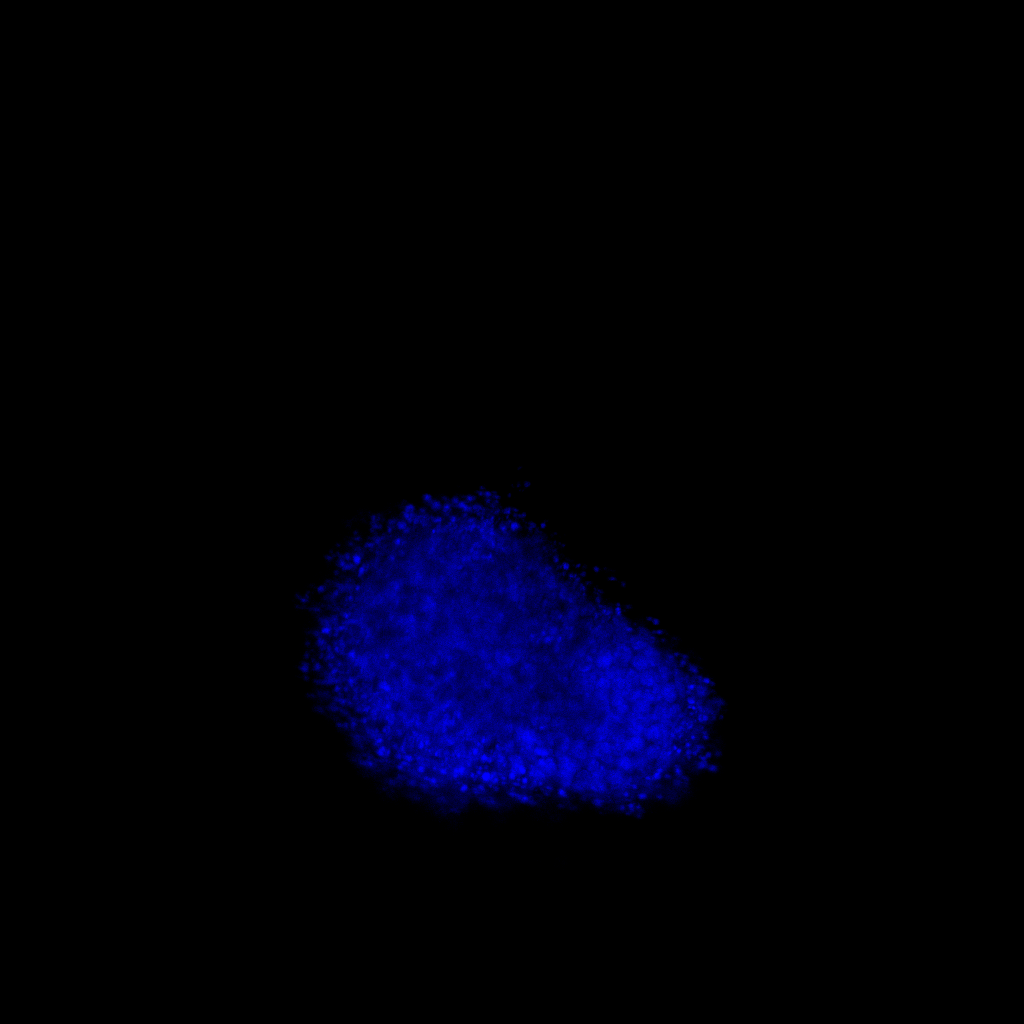

Supplement: Supplementary file 6 — Source data Fig. 4 [file 44318_2025_558_MOESM6_ESM.zip › Figure 4/panel 4B/NT_Oct4_6uM/seq9850_seq9850_RGB_DAPI.tif]

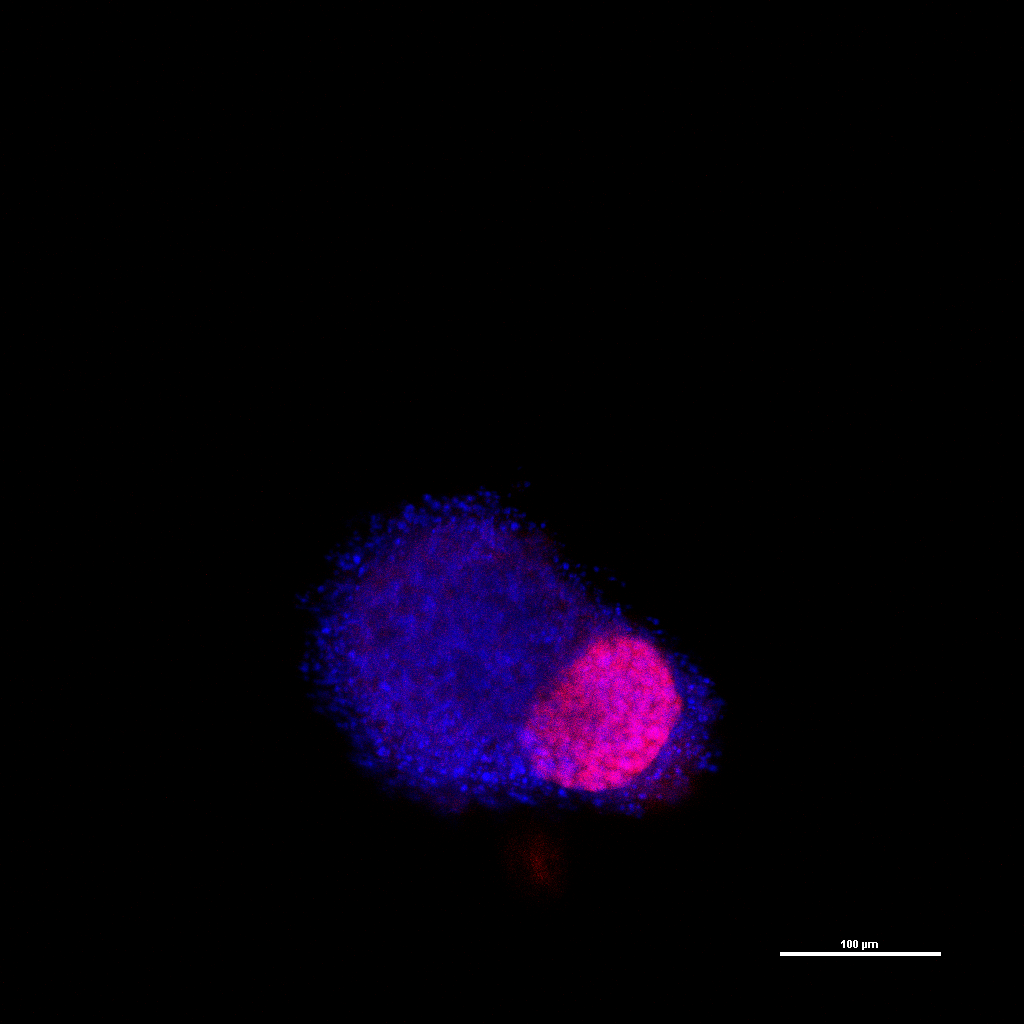

Supplement: Supplementary file 6 — Source data Fig. 4 [file 44318_2025_558_MOESM6_ESM.zip › Figure 4/panel 4B/NT_Oct4_6uM/seq9850_seq9850_RGB.tif]

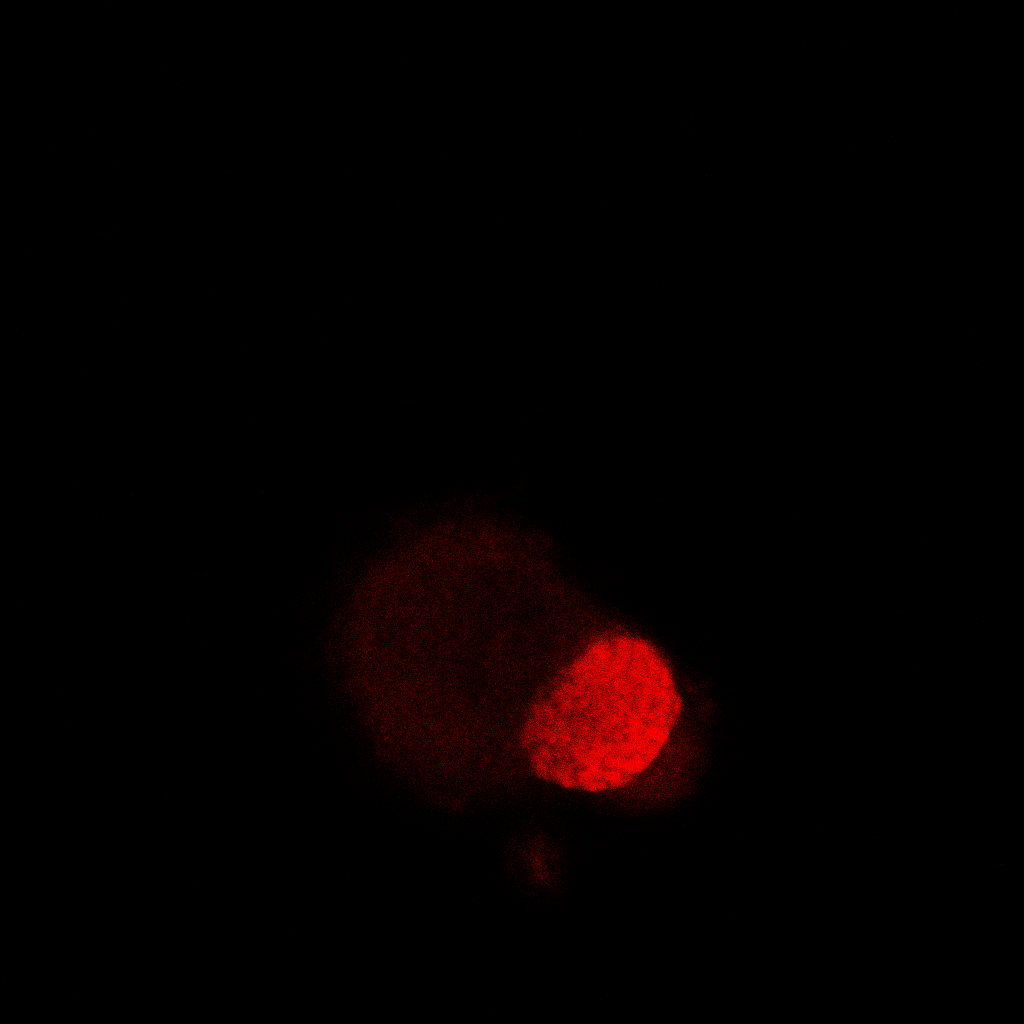

Supplement: Supplementary file 6 — Source data Fig. 4 [file 44318_2025_558_MOESM6_ESM.zip › Figure 4/panel 4B/NT_Oct4_6uM/seq9850_seq9850_RGB_TRITC.tif]

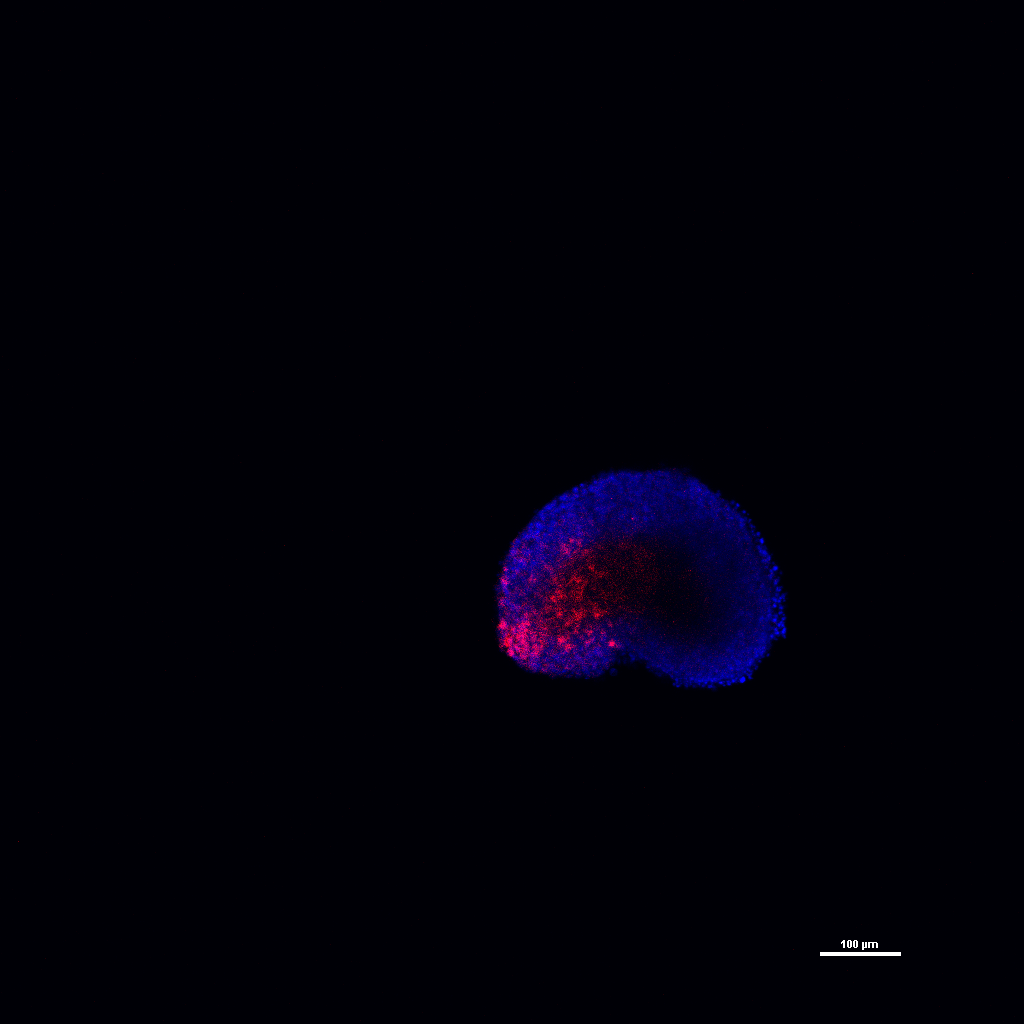

Supplement: Supplementary file 6 — Source data Fig. 4 [file 44318_2025_558_MOESM6_ESM.zip › Figure 4/panel 4B/KD-2_Nanog_6uM/fila51867_RGB.tif]

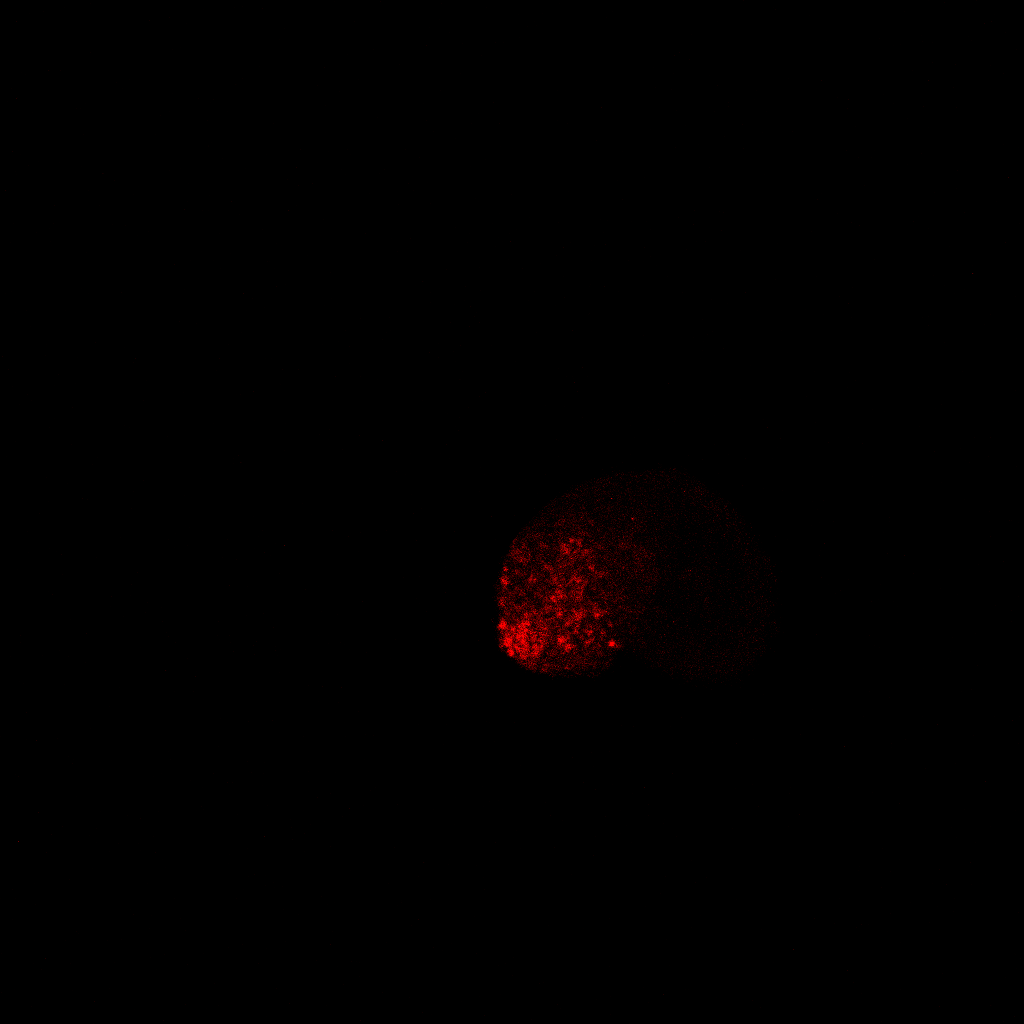

Supplement: Supplementary file 6 — Source data Fig. 4 [file 44318_2025_558_MOESM6_ESM.zip › Figure 4/panel 4B/KD-2_Nanog_6uM/fila51867_RGB_Alexa Fluor 594 cadaverine_H2O.tif]

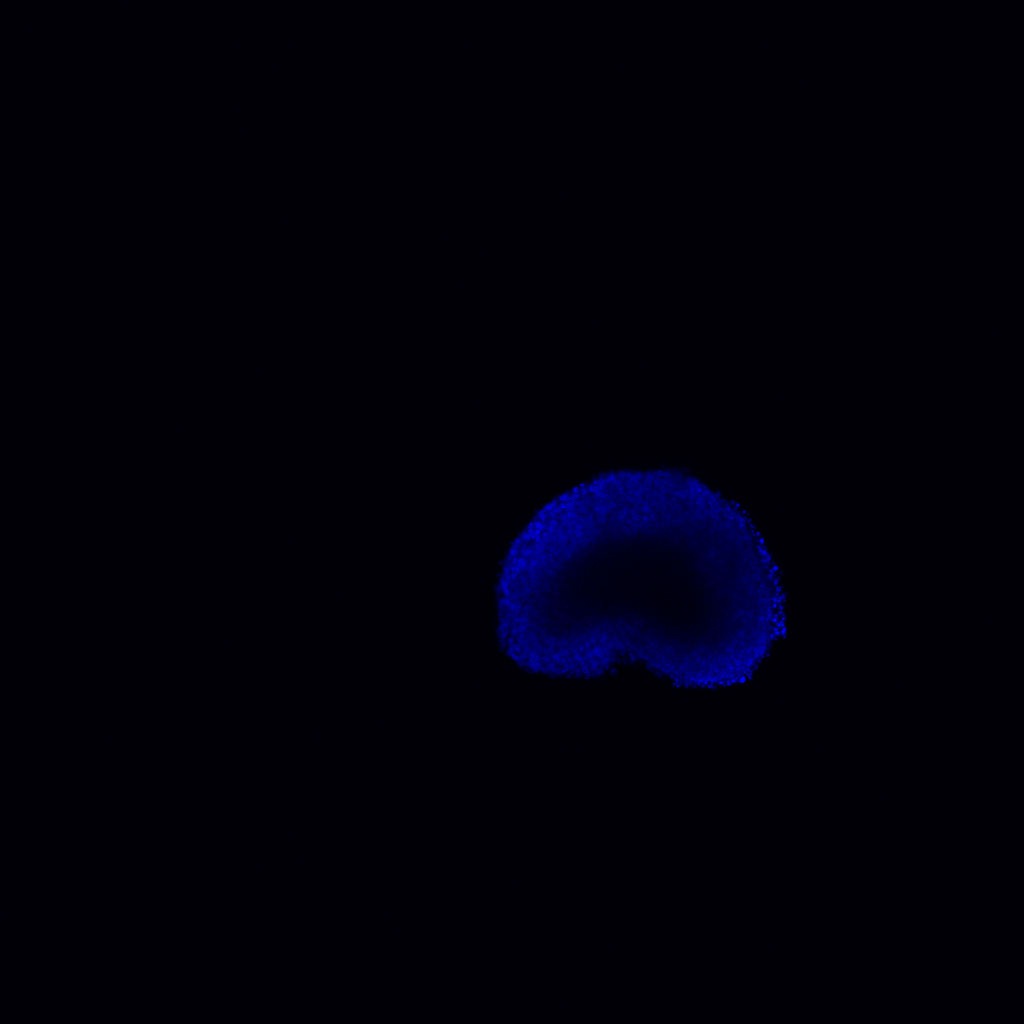

Supplement: Supplementary file 6 — Source data Fig. 4 [file 44318_2025_558_MOESM6_ESM.zip › Figure 4/panel 4B/KD-2_Nanog_6uM/fila51867_RGB_DAPI.tif]

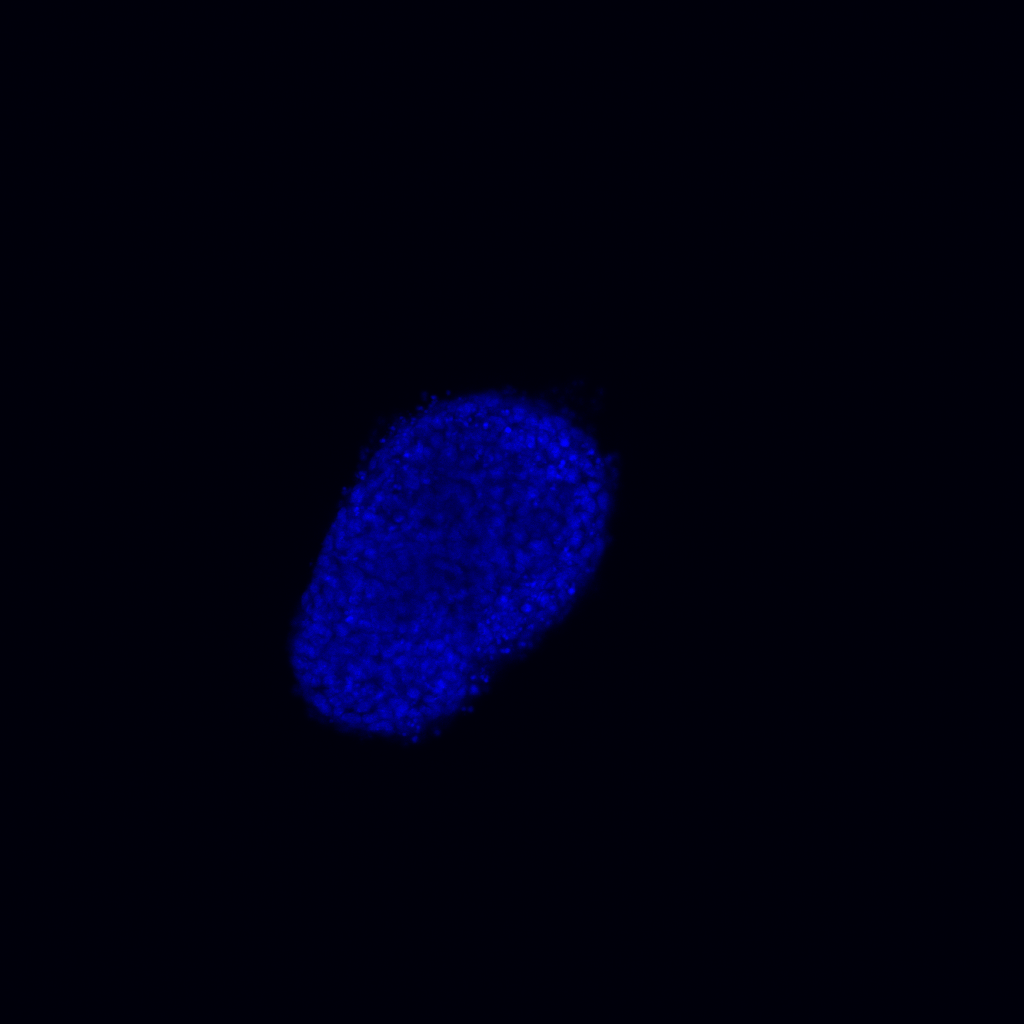

Supplement: Supplementary file 6 — Source data Fig. 4 [file 44318_2025_558_MOESM6_ESM.zip › Figure 4/panel 4B/KD-1_Nanog_3uM/seq9146_seq9146_RGB_DAPI.tif]

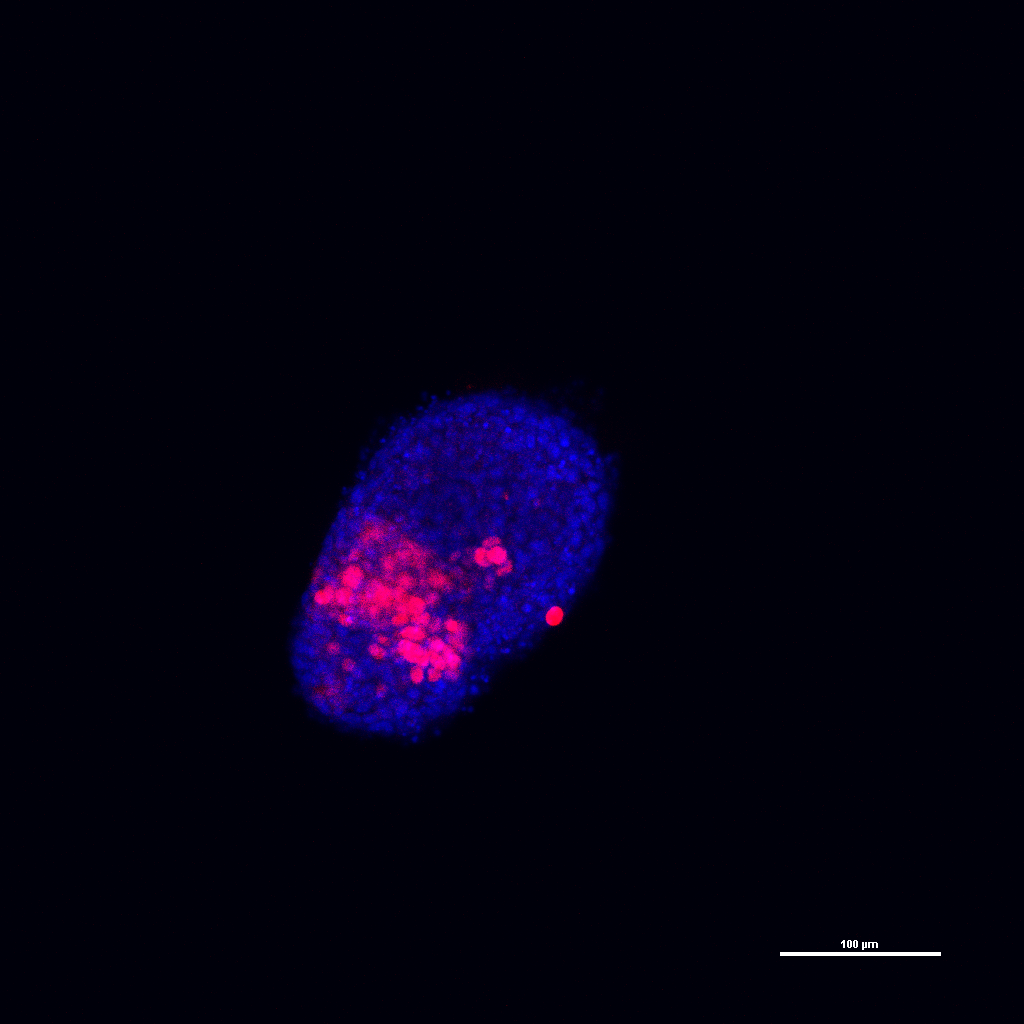

Supplement: Supplementary file 6 — Source data Fig. 4 [file 44318_2025_558_MOESM6_ESM.zip › Figure 4/panel 4B/KD-1_Nanog_3uM/seq9146_seq9146_RGB.tif]

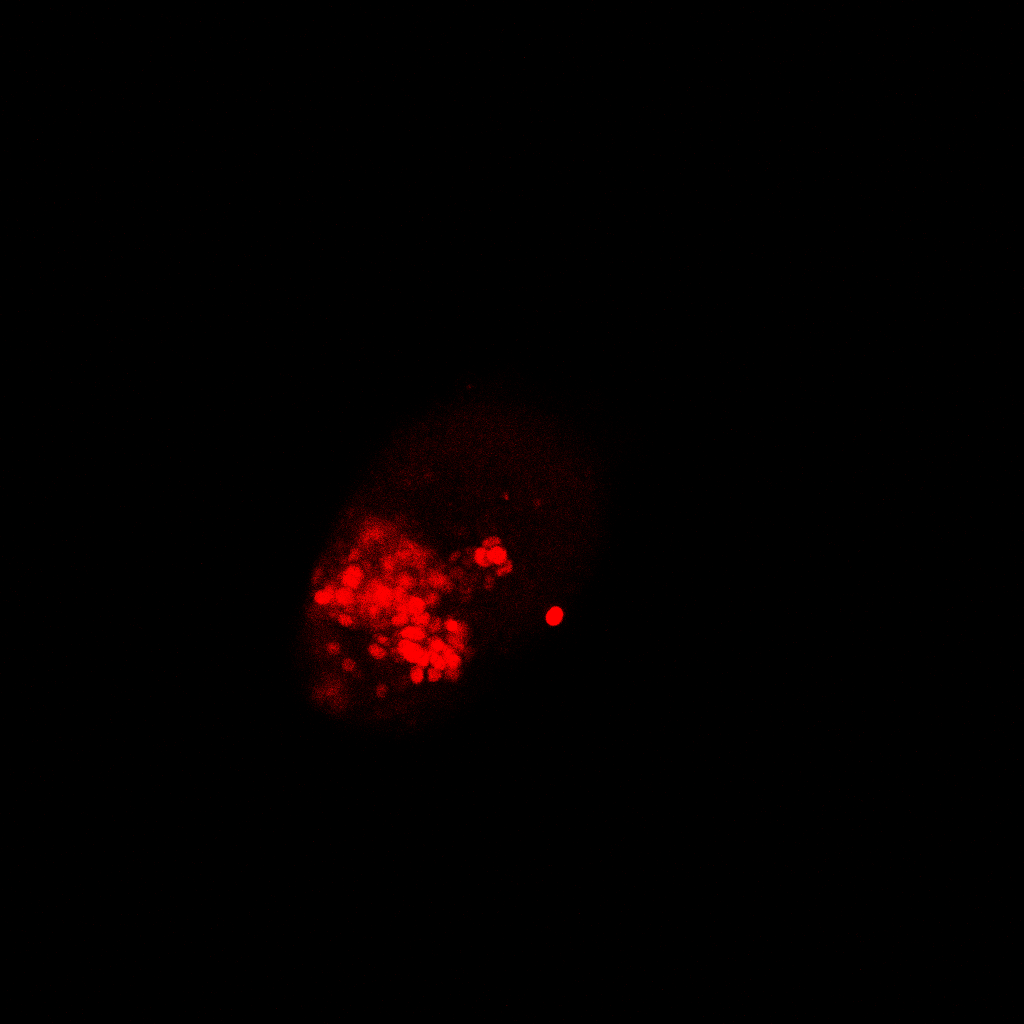

Supplement: Supplementary file 6 — Source data Fig. 4 [file 44318_2025_558_MOESM6_ESM.zip › Figure 4/panel 4B/KD-1_Nanog_3uM/seq9146_seq9146_RGB_Texas Red.tif]

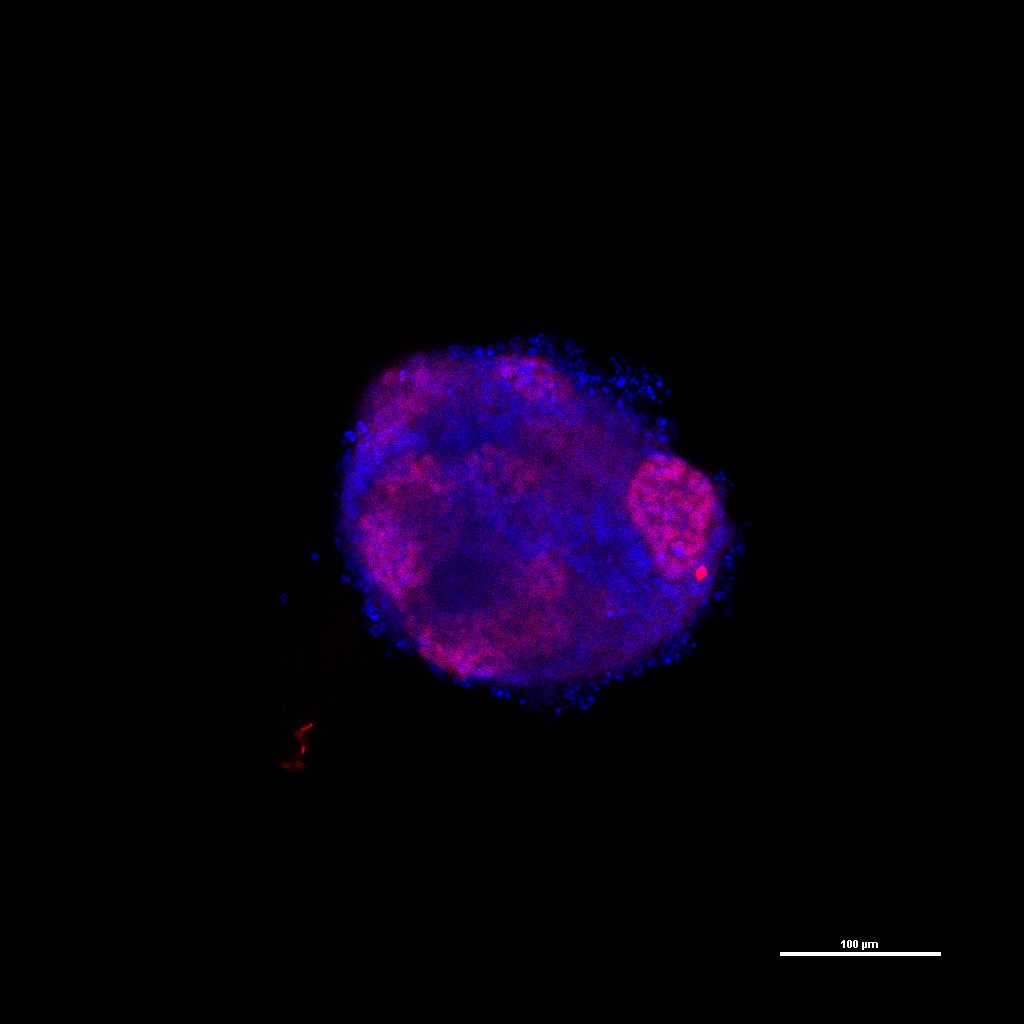

Supplement: Supplementary file 6 — Source data Fig. 4 [file 44318_2025_558_MOESM6_ESM.zip › Figure 4/panel 4B/KD-1_Oct4_3uM/seq9856_seq9856_RGB.tif]

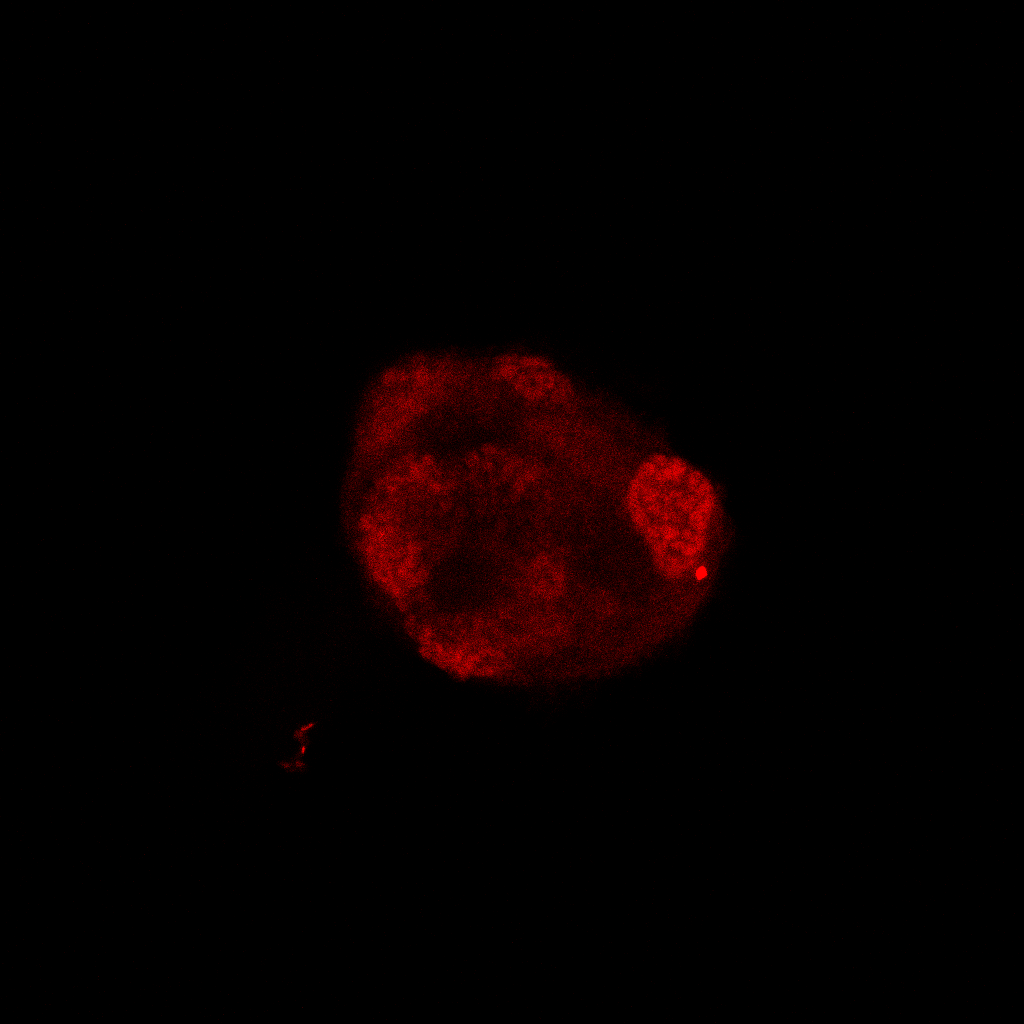

Supplement: Supplementary file 6 — Source data Fig. 4 [file 44318_2025_558_MOESM6_ESM.zip › Figure 4/panel 4B/KD-1_Oct4_3uM/seq9856_seq9856_RGB_TRITC.tif]

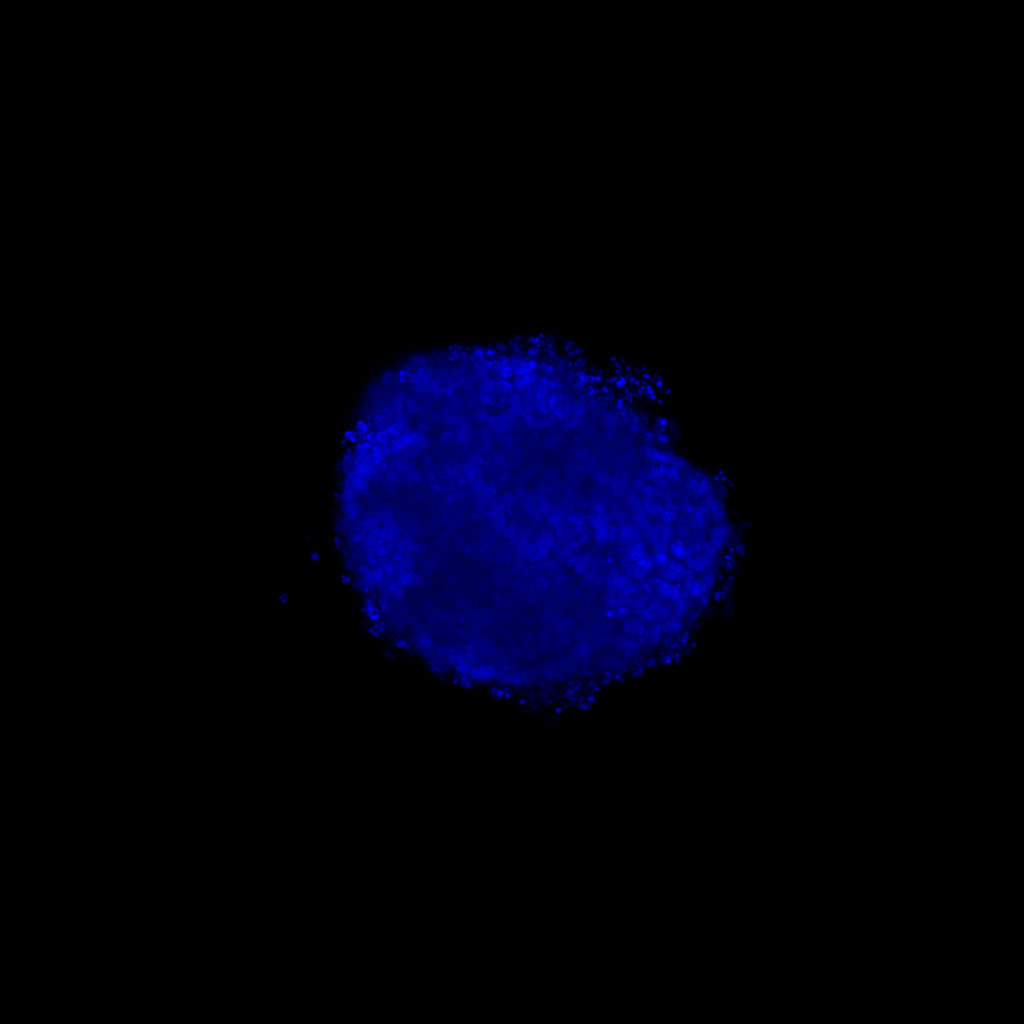

Supplement: Supplementary file 6 — Source data Fig. 4 [file 44318_2025_558_MOESM6_ESM.zip › Figure 4/panel 4B/KD-1_Oct4_3uM/seq9856_seq9856_RGB_DAPI.tif]

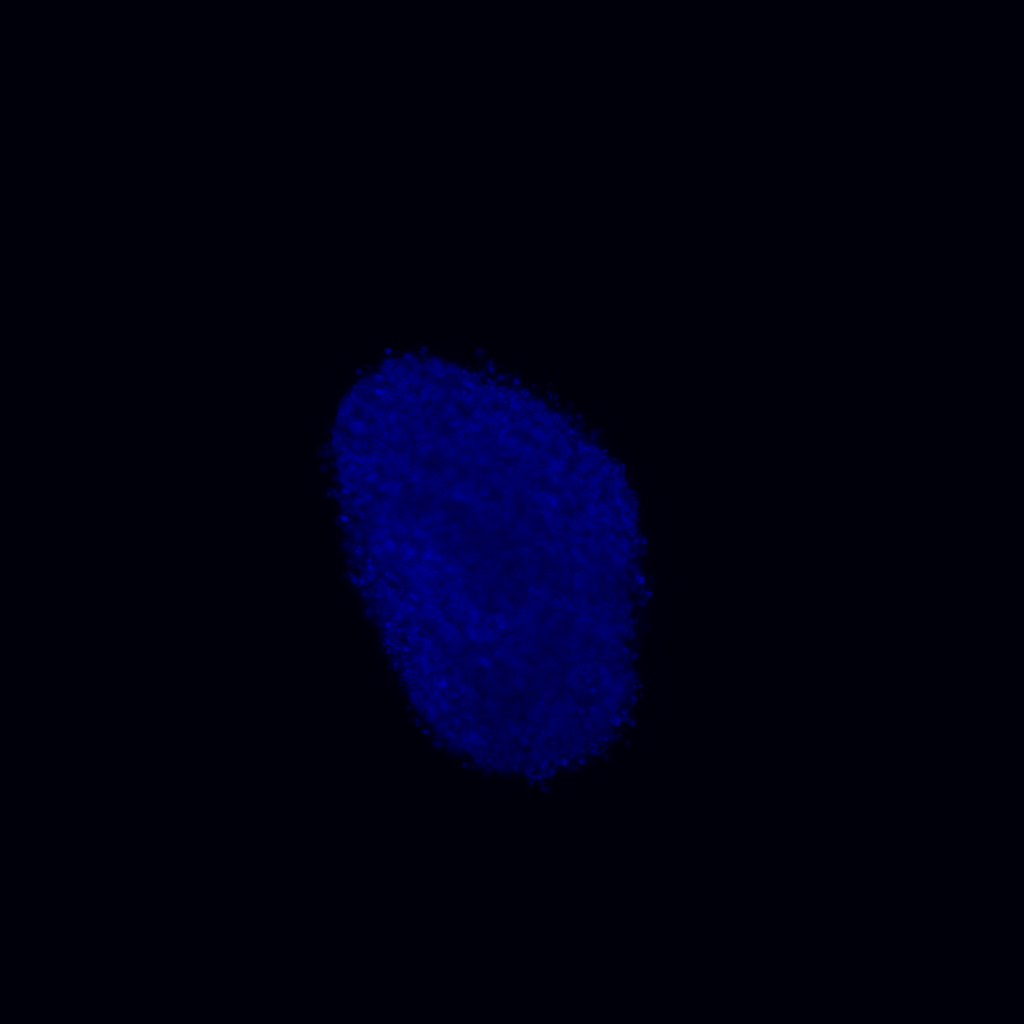

Supplement: Supplementary file 6 — Source data Fig. 4 [file 44318_2025_558_MOESM6_ESM.zip › Figure 4/panel 4B/KD-1_Bra_3uM/seq9144_seq9144_RGB_DAPI.tif]

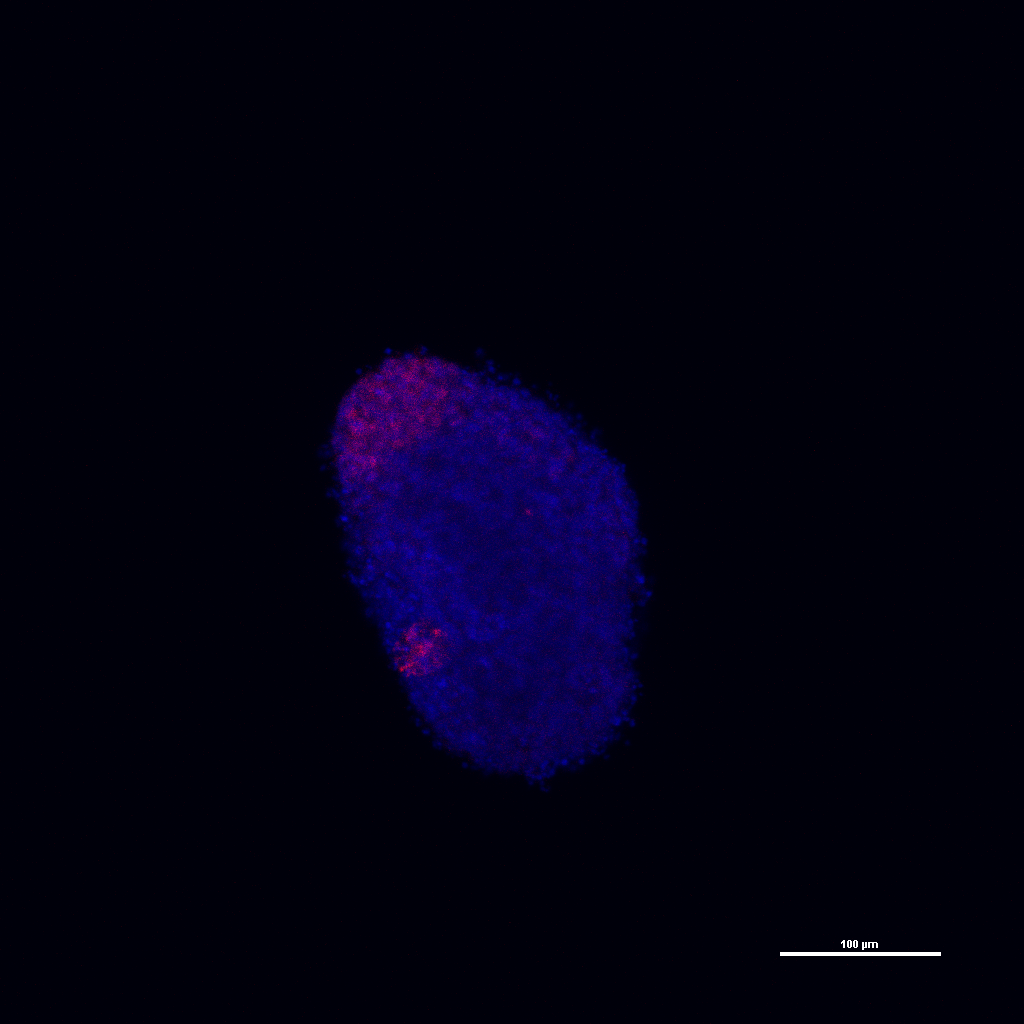

Supplement: Supplementary file 6 — Source data Fig. 4 [file 44318_2025_558_MOESM6_ESM.zip › Figure 4/panel 4B/KD-1_Bra_3uM/seq9144_seq9144_RGB.tif]

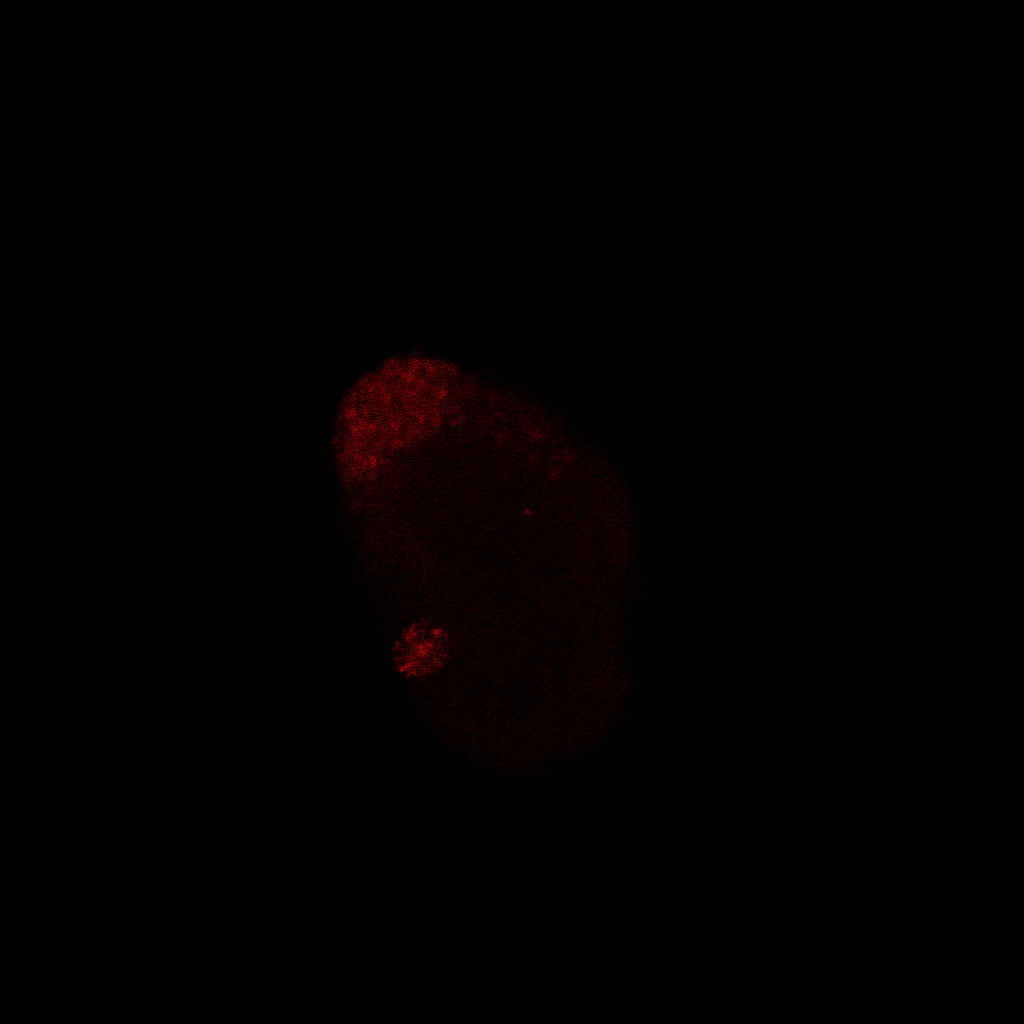

Supplement: Supplementary file 6 — Source data Fig. 4 [file 44318_2025_558_MOESM6_ESM.zip › Figure 4/panel 4B/KD-1_Bra_3uM/seq9144_seq9144_RGB_Texas Red.tif]

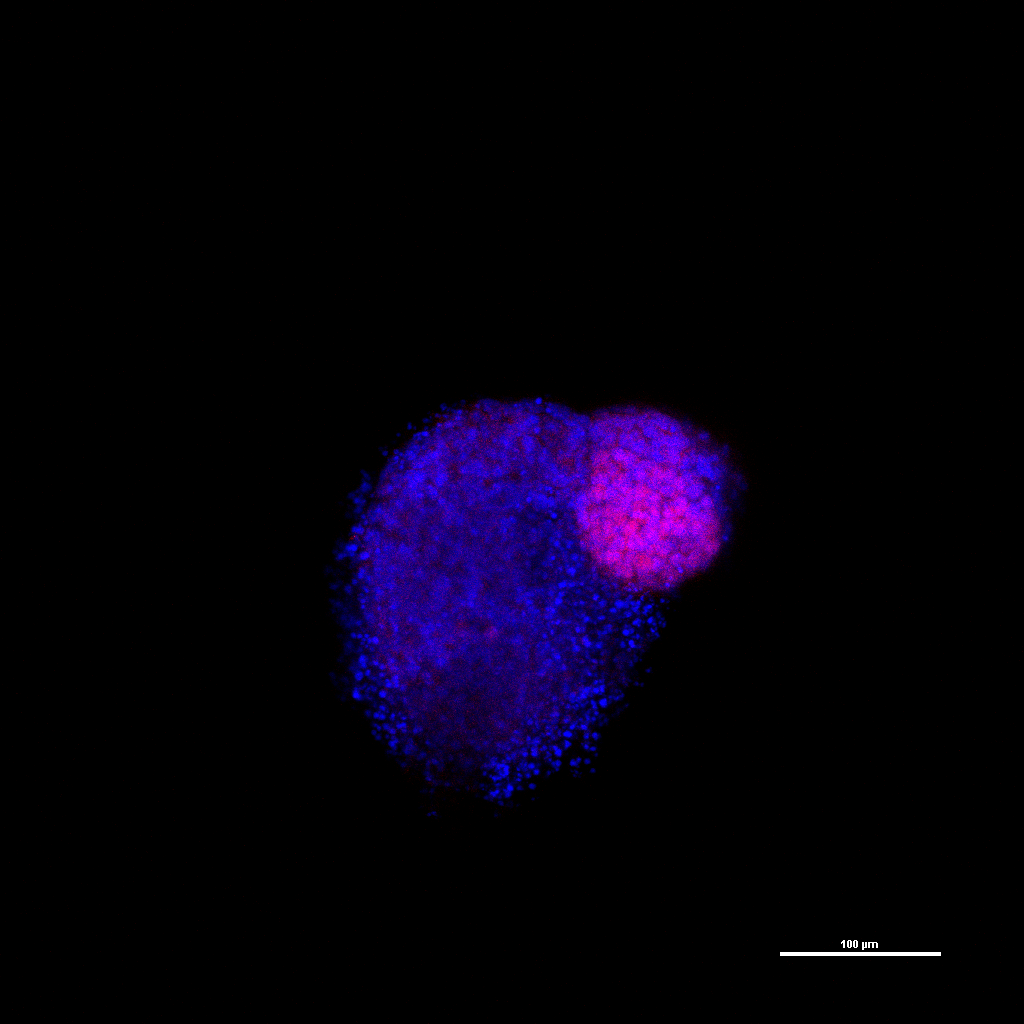

Supplement: Supplementary file 6 — Source data Fig. 4 [file 44318_2025_558_MOESM6_ESM.zip › Figure 4/panel 4B/NT_Oct4_5uM/seq9846_seq9846_RGB.tif]

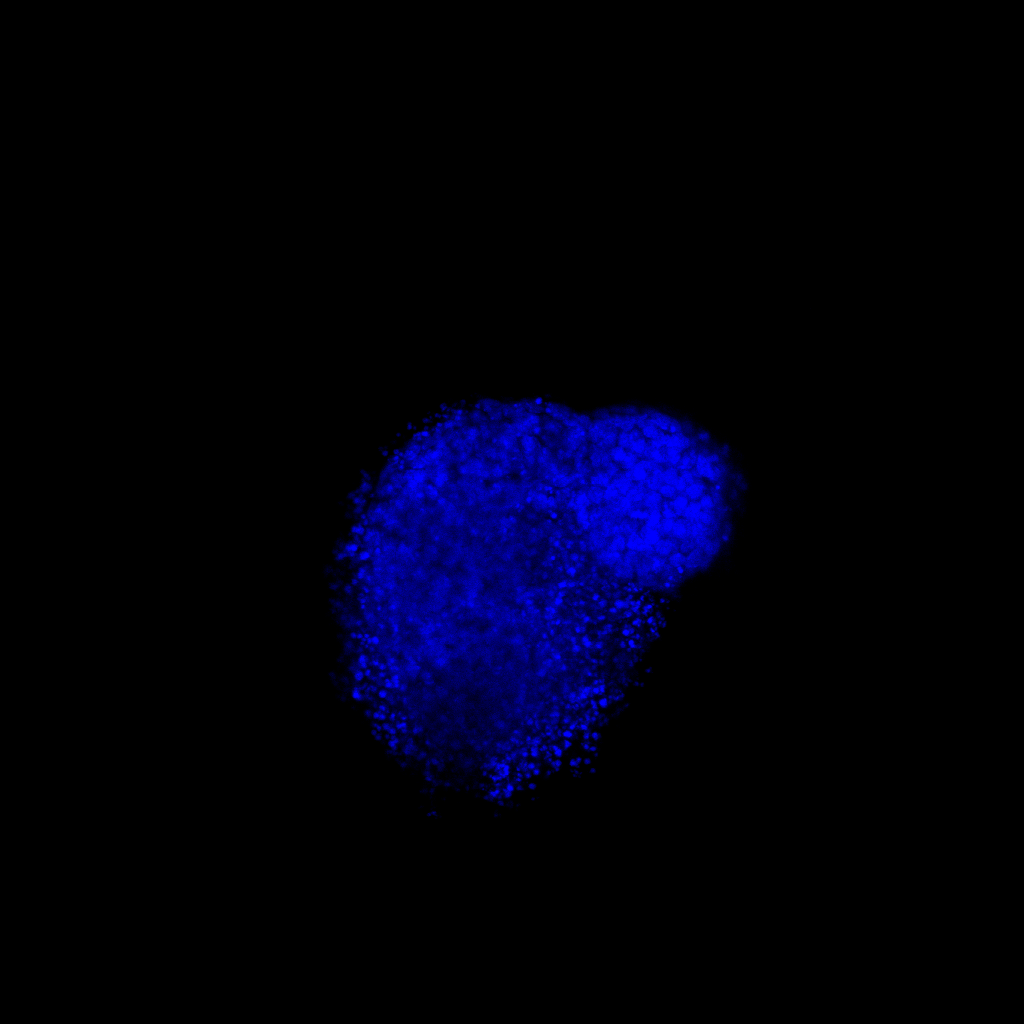

Supplement: Supplementary file 6 — Source data Fig. 4 [file 44318_2025_558_MOESM6_ESM.zip › Figure 4/panel 4B/NT_Oct4_5uM/seq9846_seq9846_RGB_DAPI.tif]

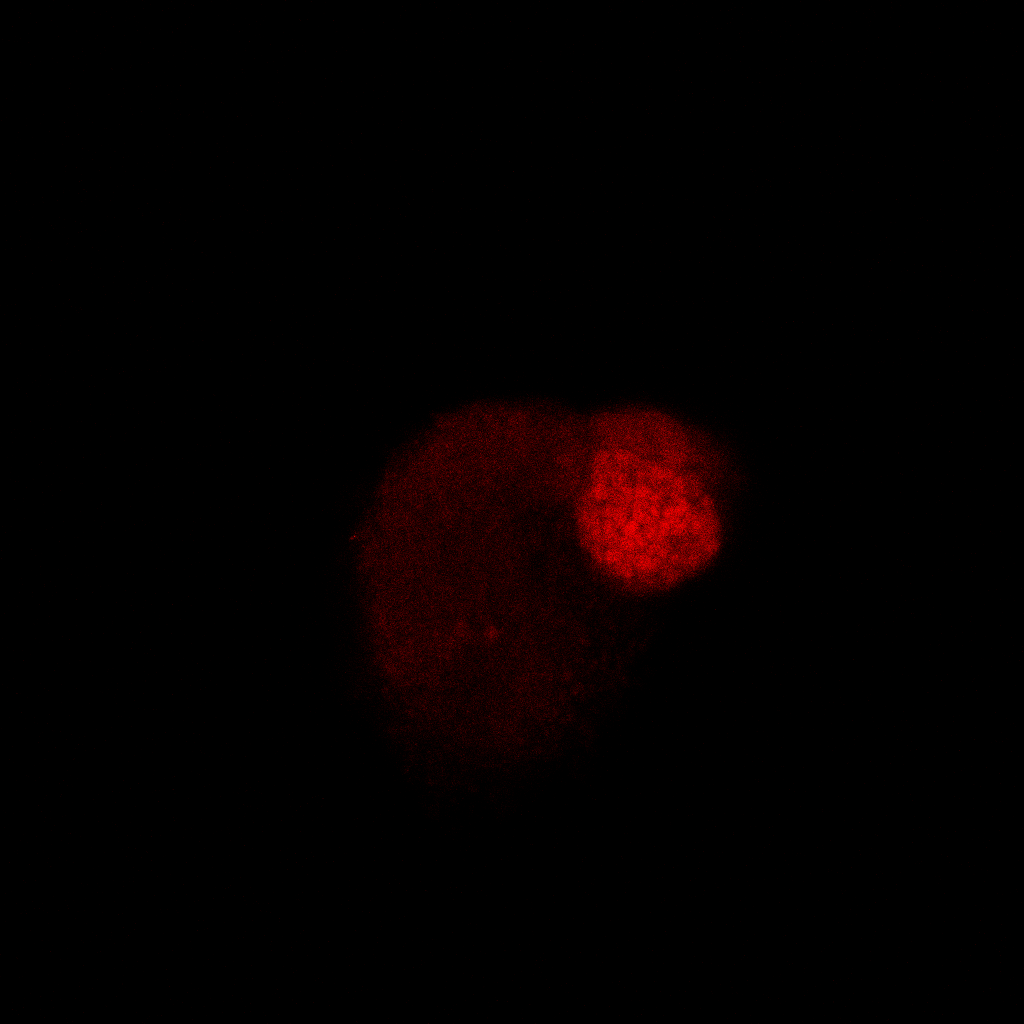

Supplement: Supplementary file 6 — Source data Fig. 4 [file 44318_2025_558_MOESM6_ESM.zip › Figure 4/panel 4B/NT_Oct4_5uM/seq9846_seq9846_RGB_TRITC.tif]

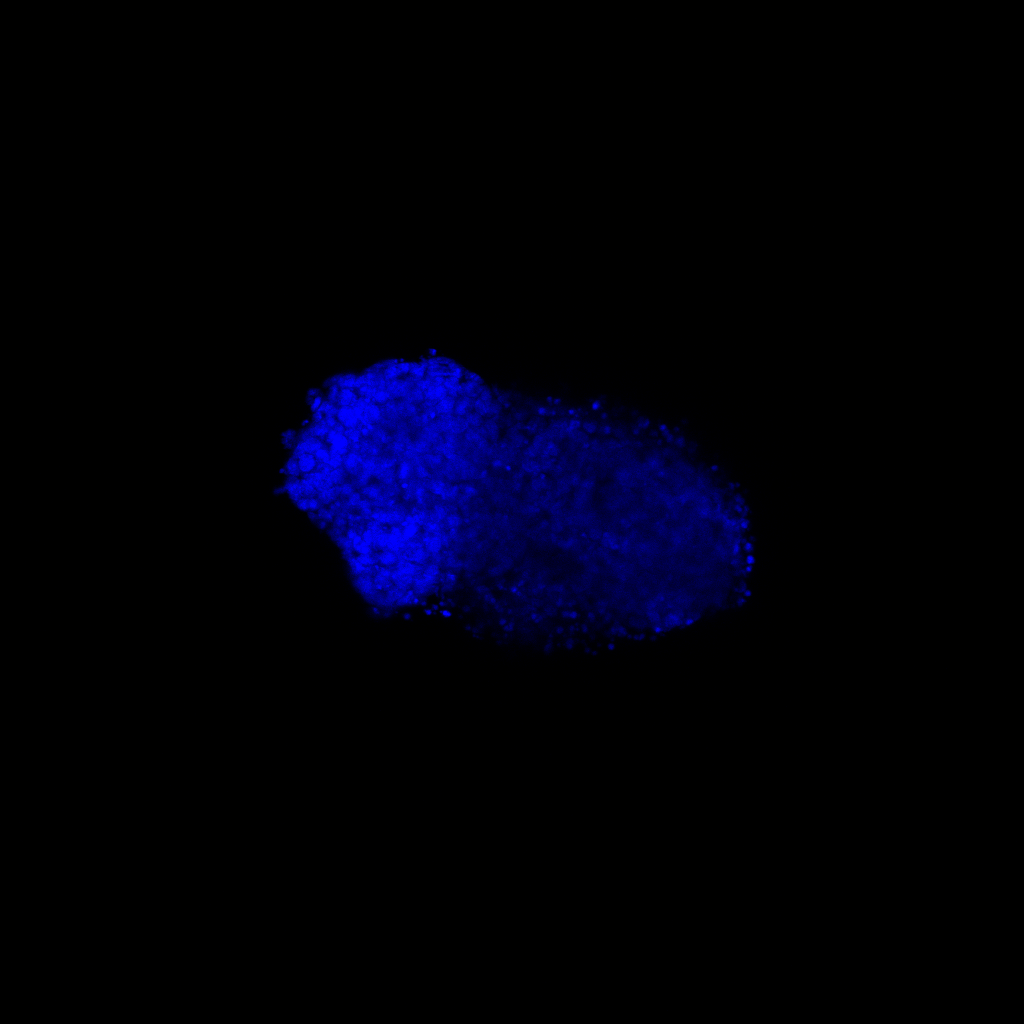

Supplement: Supplementary file 6 — Source data Fig. 4 [file 44318_2025_558_MOESM6_ESM.zip › Figure 4/panel 4B/KD-2_Oct4_6uM/seq9871_seq9871_RGB_DAPI.tif]

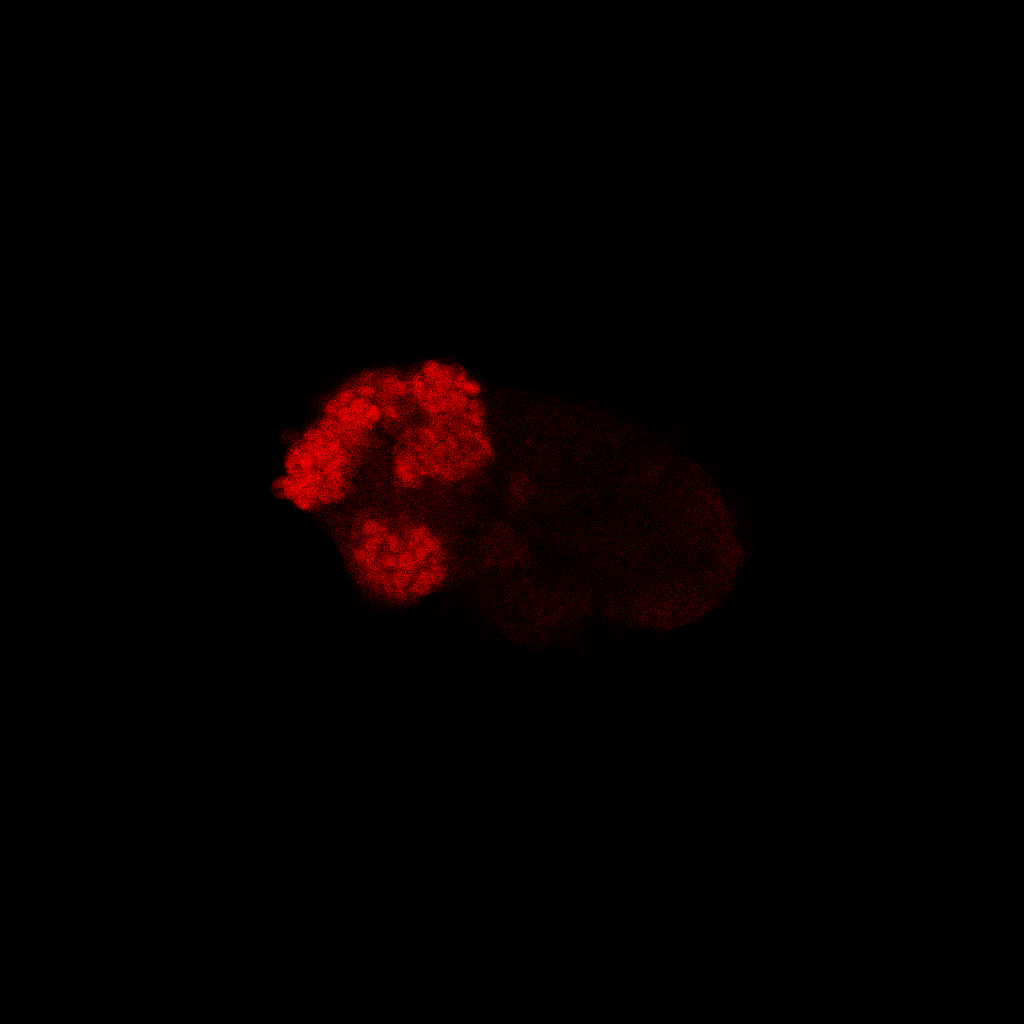

Supplement: Supplementary file 6 — Source data Fig. 4 [file 44318_2025_558_MOESM6_ESM.zip › Figure 4/panel 4B/KD-2_Oct4_6uM/seq9871_seq9871_RGB_TRITC.tif]

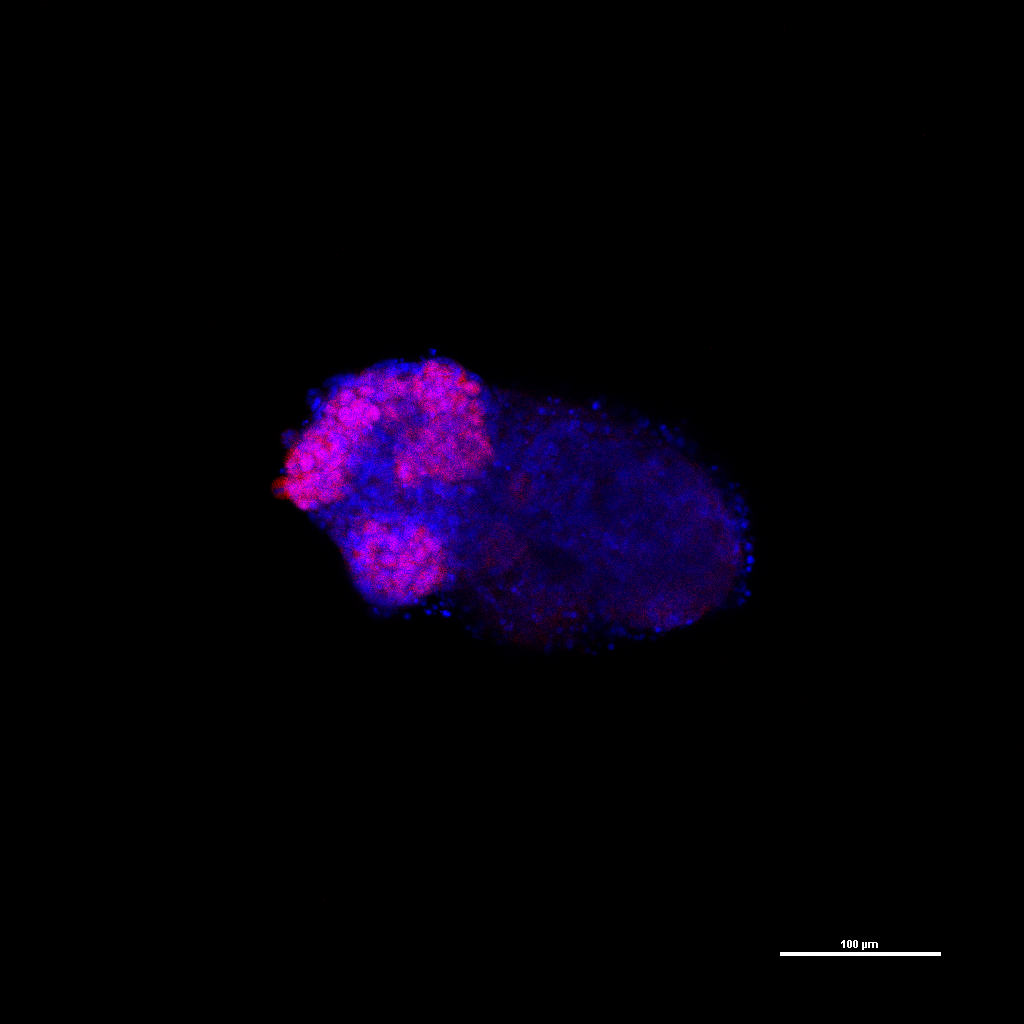

Supplement: Supplementary file 6 — Source data Fig. 4 [file 44318_2025_558_MOESM6_ESM.zip › Figure 4/panel 4B/KD-2_Oct4_6uM/seq9871_seq9871_RGB.tif]

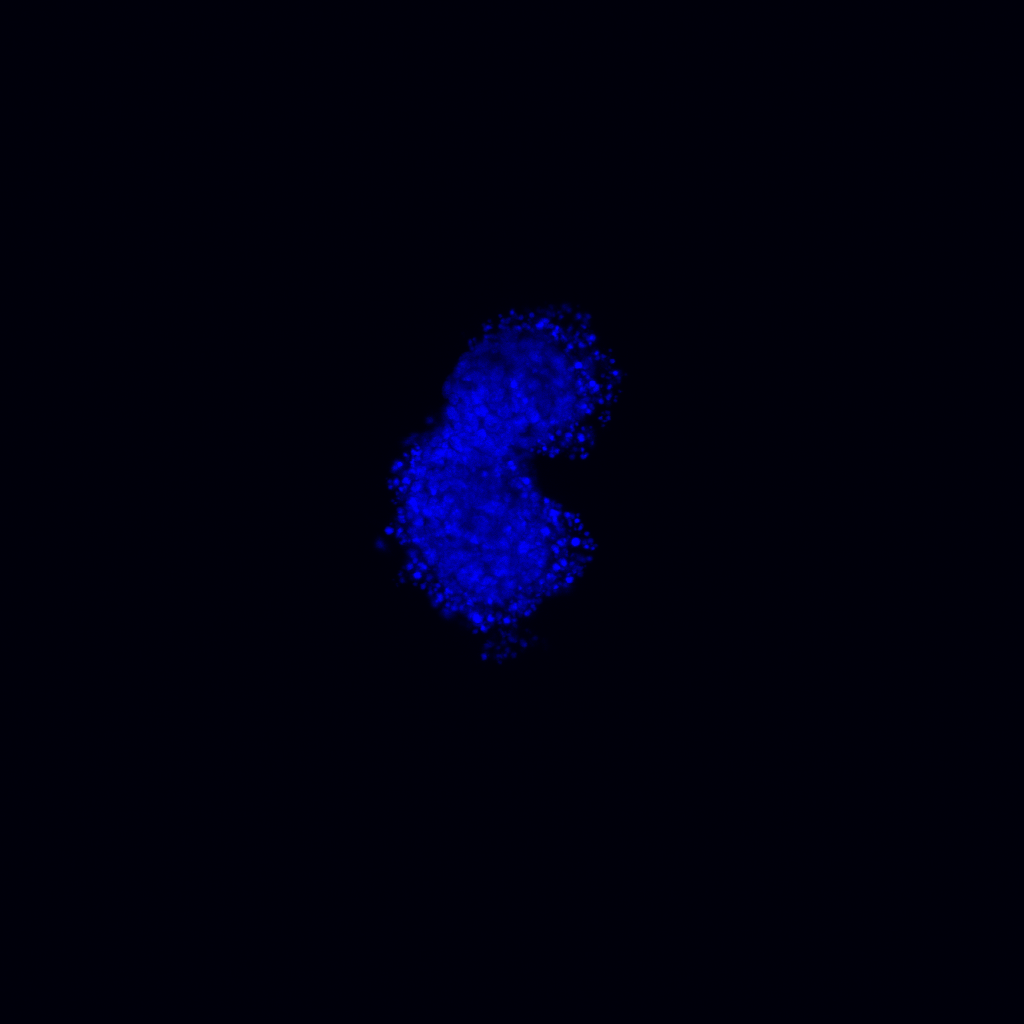

Supplement: Supplementary file 6 — Source data Fig. 4 [file 44318_2025_558_MOESM6_ESM.zip › Figure 4/panel 4B/NT_Cdx2_6uM/seq9180_seq9180_RGB_DAPI.tif]

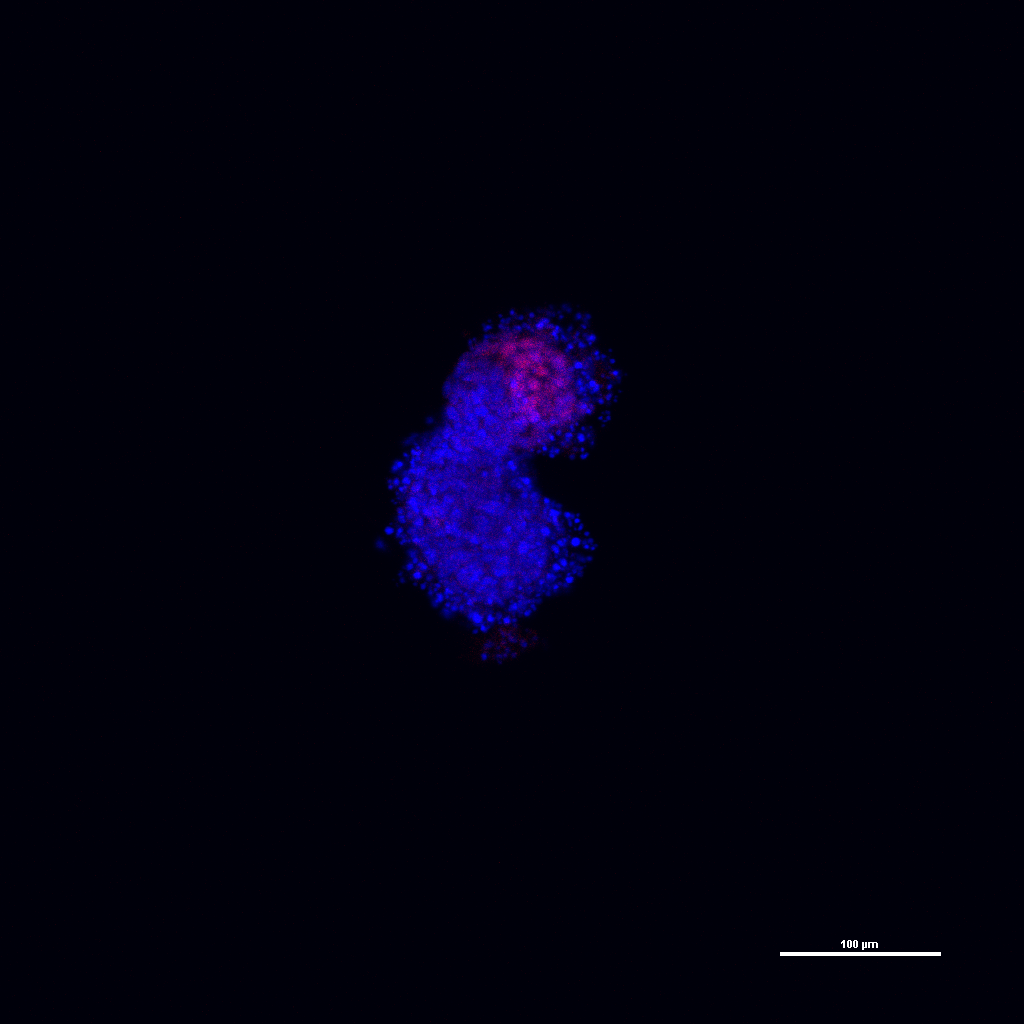

Supplement: Supplementary file 6 — Source data Fig. 4 [file 44318_2025_558_MOESM6_ESM.zip › Figure 4/panel 4B/NT_Cdx2_6uM/seq9180_seq9180_RGB.tif]

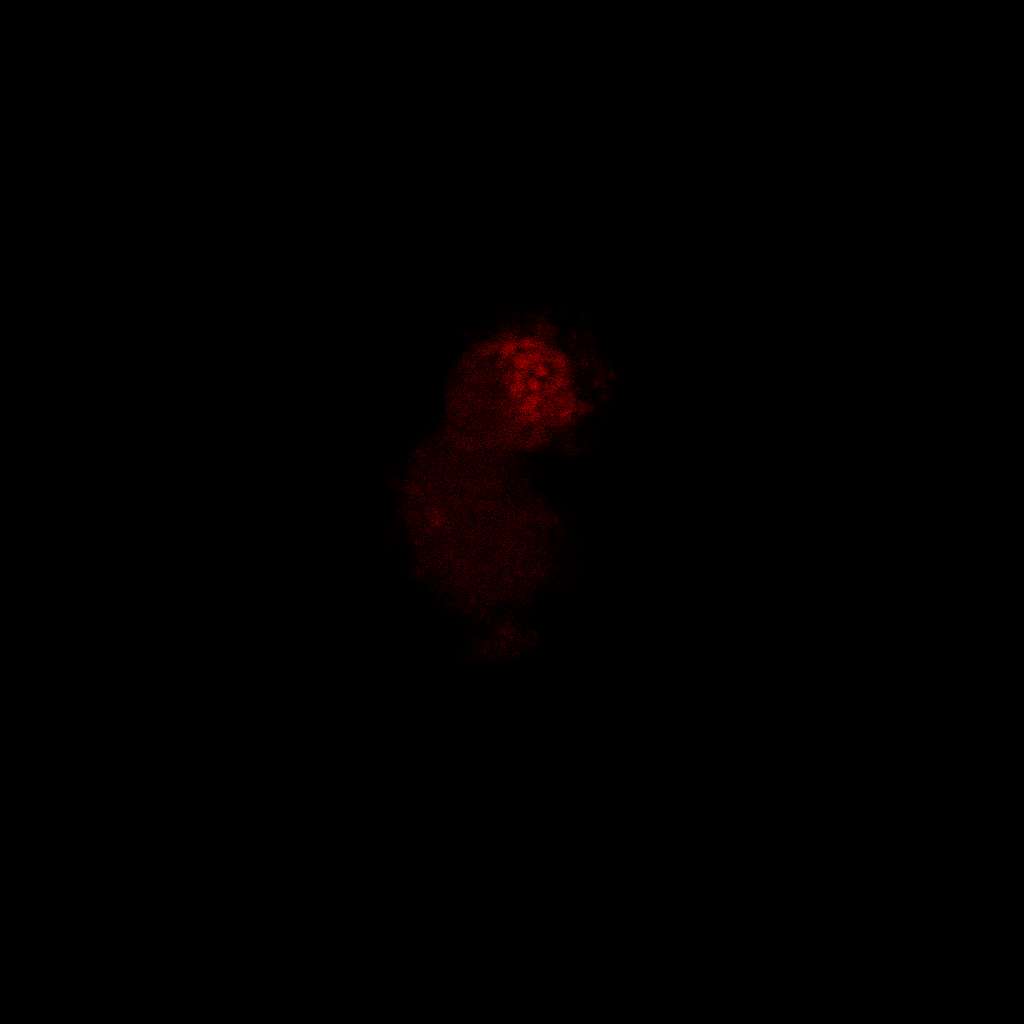

Supplement: Supplementary file 6 — Source data Fig. 4 [file 44318_2025_558_MOESM6_ESM.zip › Figure 4/panel 4B/NT_Cdx2_6uM/seq9180_seq9180_RGB_Texas Red.tif]

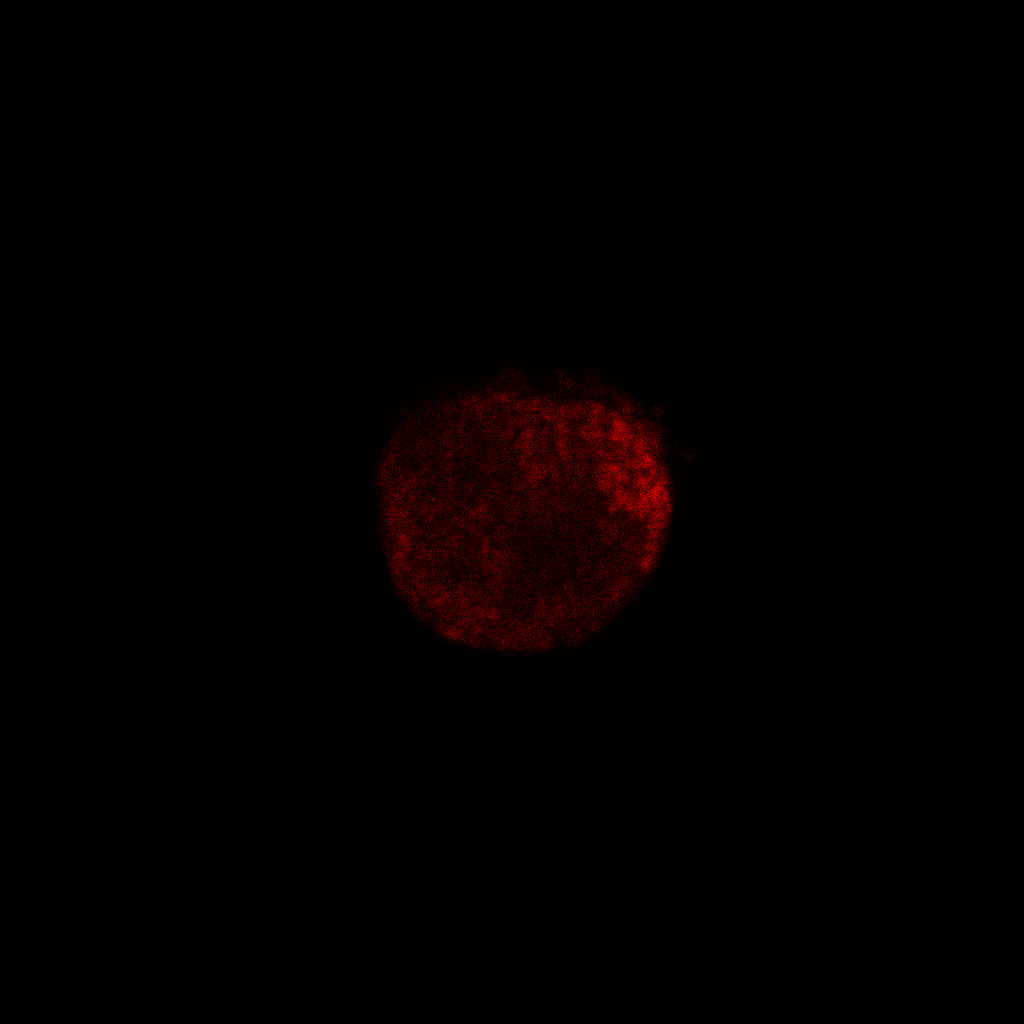

Supplement: Supplementary file 6 — Source data Fig. 4 [file 44318_2025_558_MOESM6_ESM.zip › Figure 4/panel 4B/KD-2_Cdx2_5uM/seq9195_seq9195_RGB_Texas Red.tif]

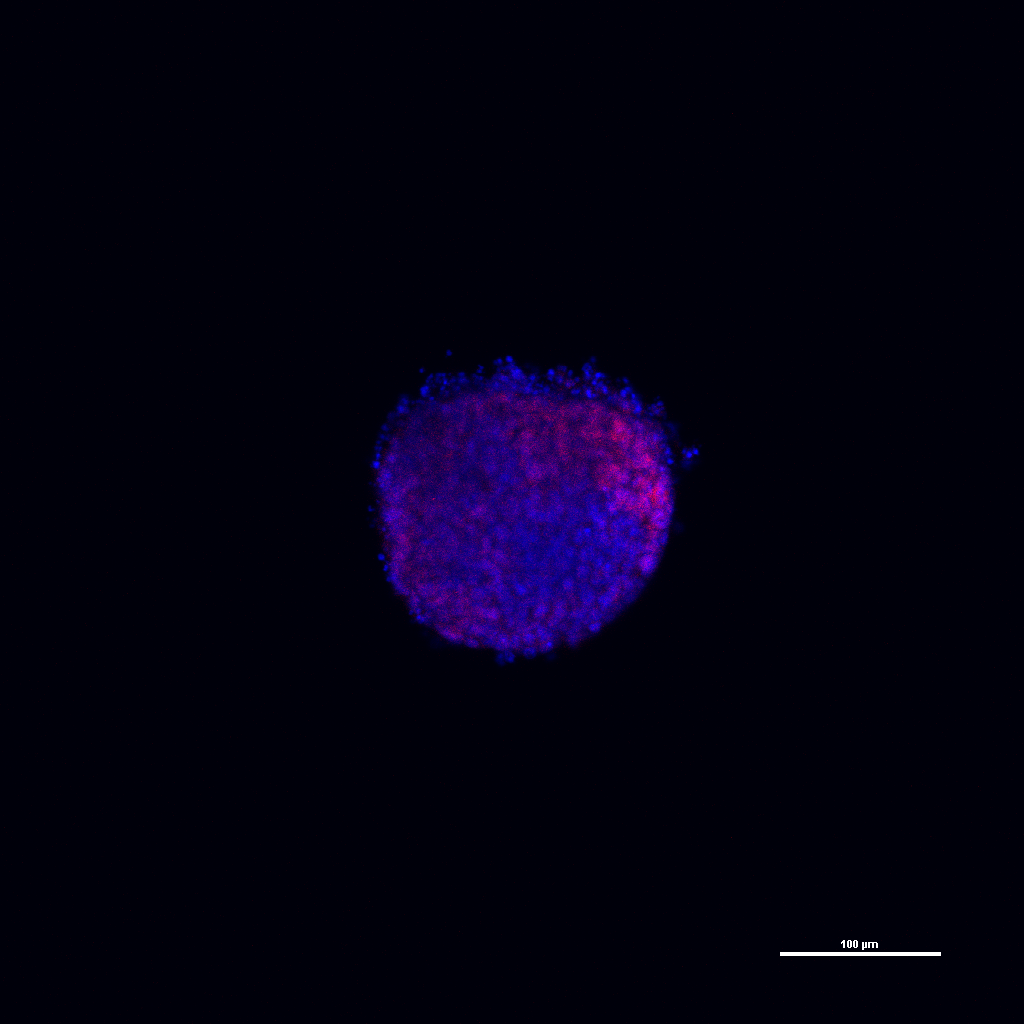

Supplement: Supplementary file 6 — Source data Fig. 4 [file 44318_2025_558_MOESM6_ESM.zip › Figure 4/panel 4B/KD-2_Cdx2_5uM/seq9195_seq9195_RGB.tif]

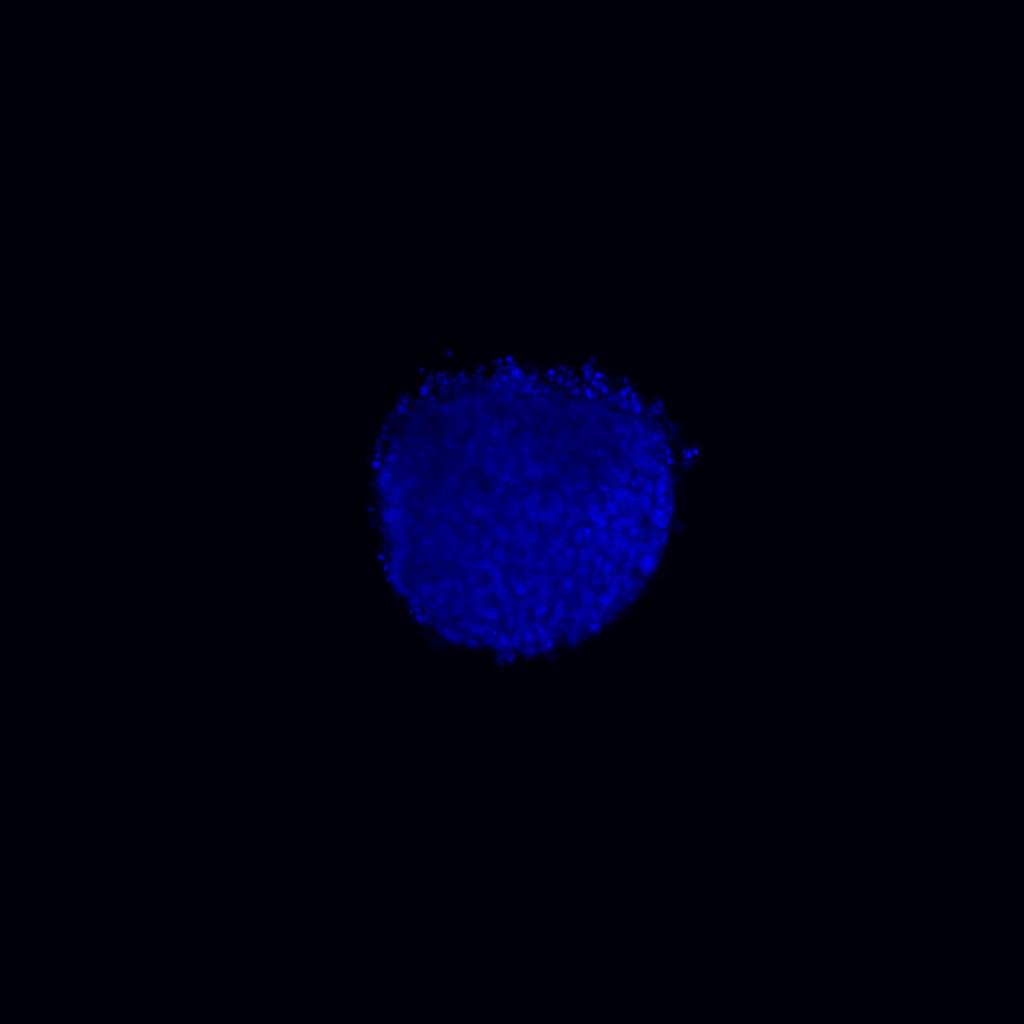

Supplement: Supplementary file 6 — Source data Fig. 4 [file 44318_2025_558_MOESM6_ESM.zip › Figure 4/panel 4B/KD-2_Cdx2_5uM/seq9195_seq9195_RGB_DAPI.tif]

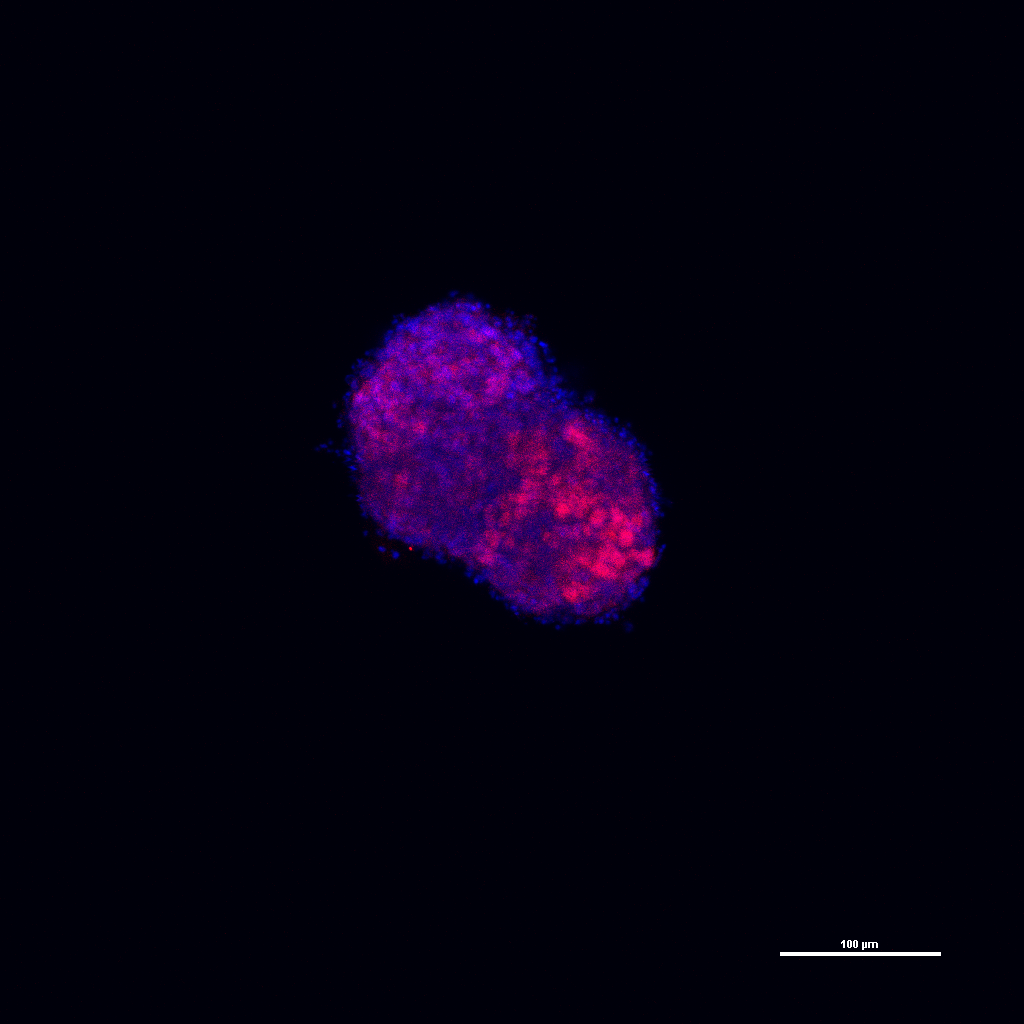

Supplement: Supplementary file 6 — Source data Fig. 4 [file 44318_2025_558_MOESM6_ESM.zip › Figure 4/panel 4B/KD-2_Nanog_5uM/seq9153_seq9153_RGB.tif]

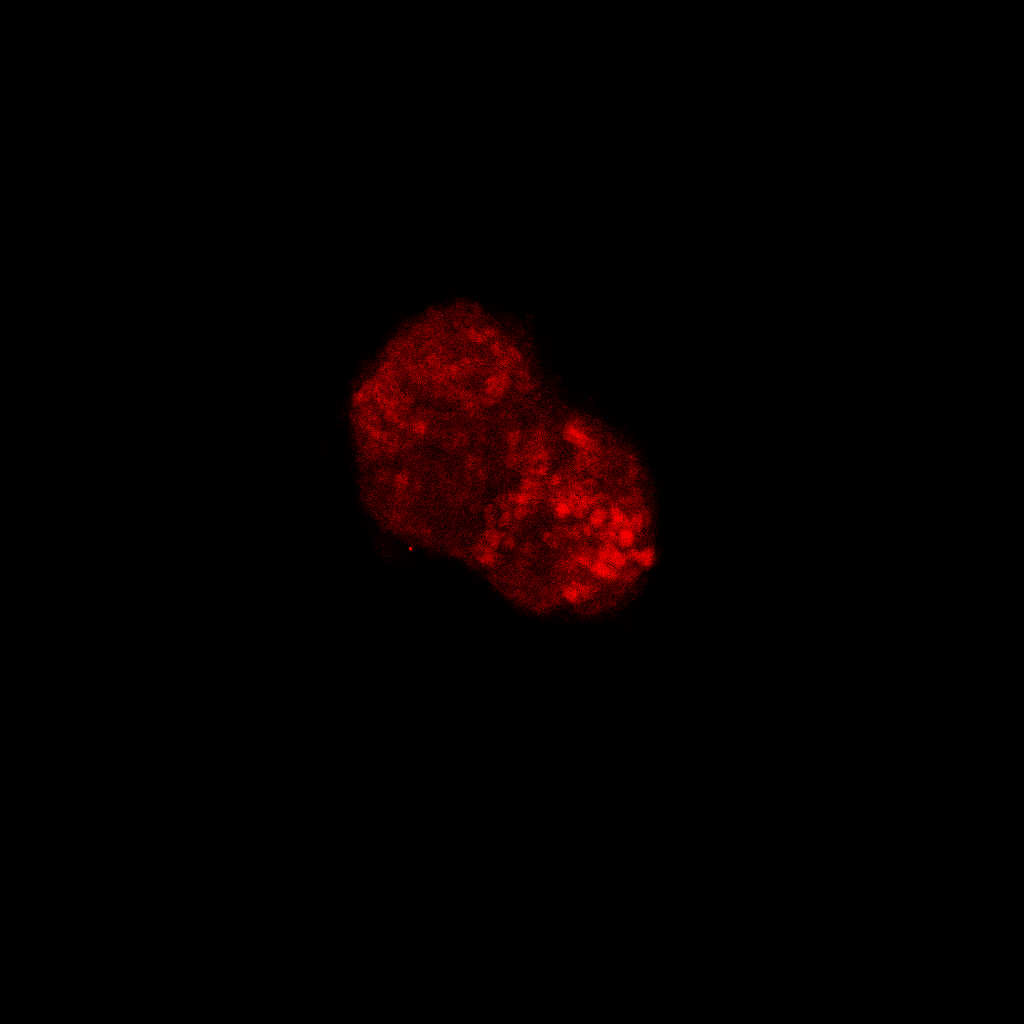

Supplement: Supplementary file 6 — Source data Fig. 4 [file 44318_2025_558_MOESM6_ESM.zip › Figure 4/panel 4B/KD-2_Nanog_5uM/seq9153_seq9153_RGB_Texas Red.tif]

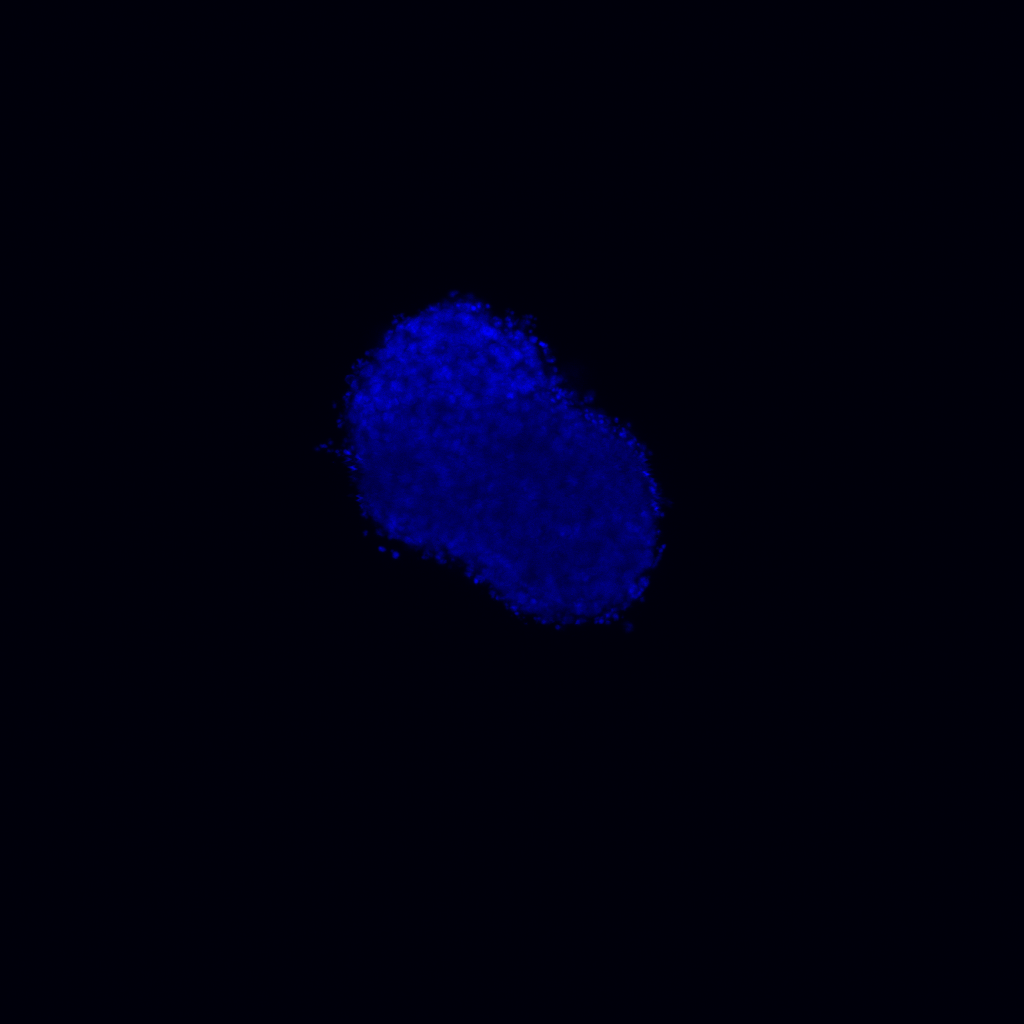

Supplement: Supplementary file 6 — Source data Fig. 4 [file 44318_2025_558_MOESM6_ESM.zip › Figure 4/panel 4B/KD-2_Nanog_5uM/seq9153_seq9153_RGB_DAPI.tif]

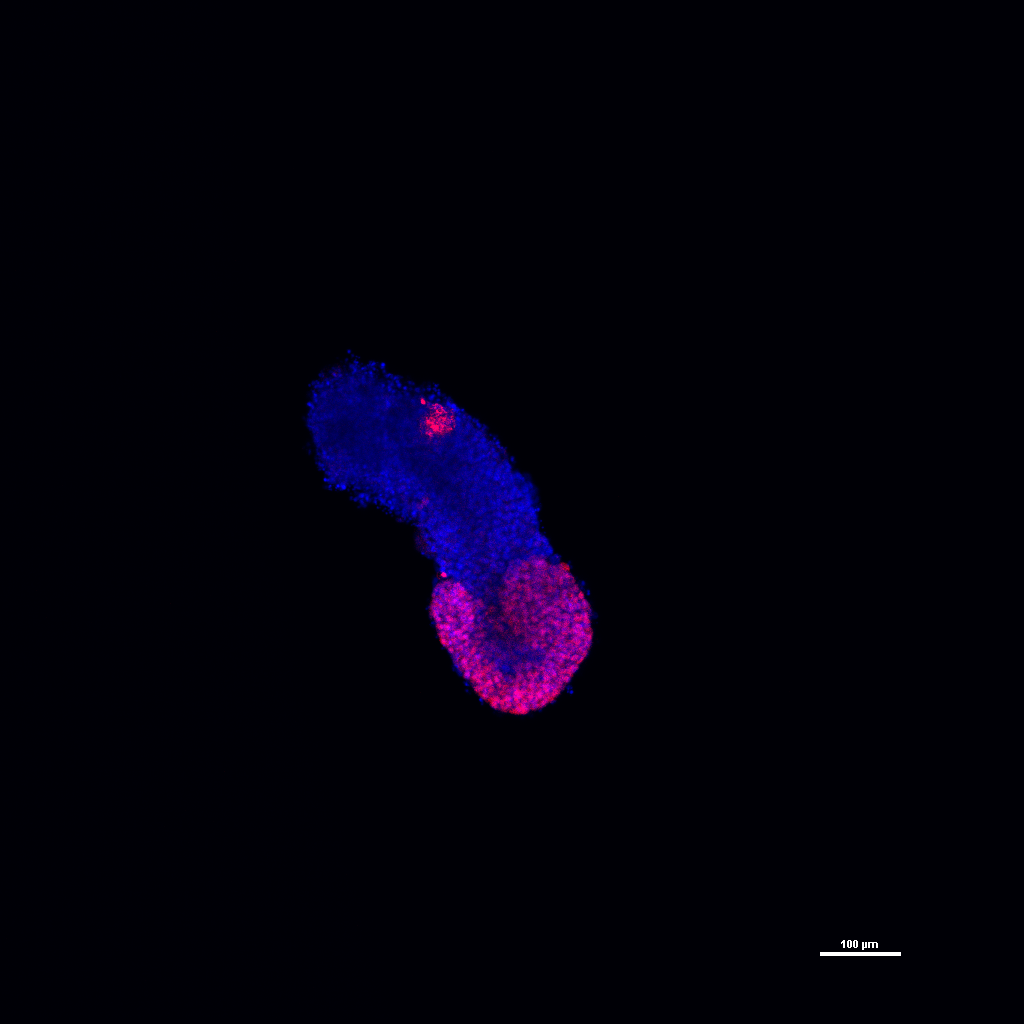

Supplement: Supplementary file 6 — Source data Fig. 4 [file 44318_2025_558_MOESM6_ESM.zip › Figure 4/panel 4B/NT_Nanog_3uM/fila51897_RGB.tif]

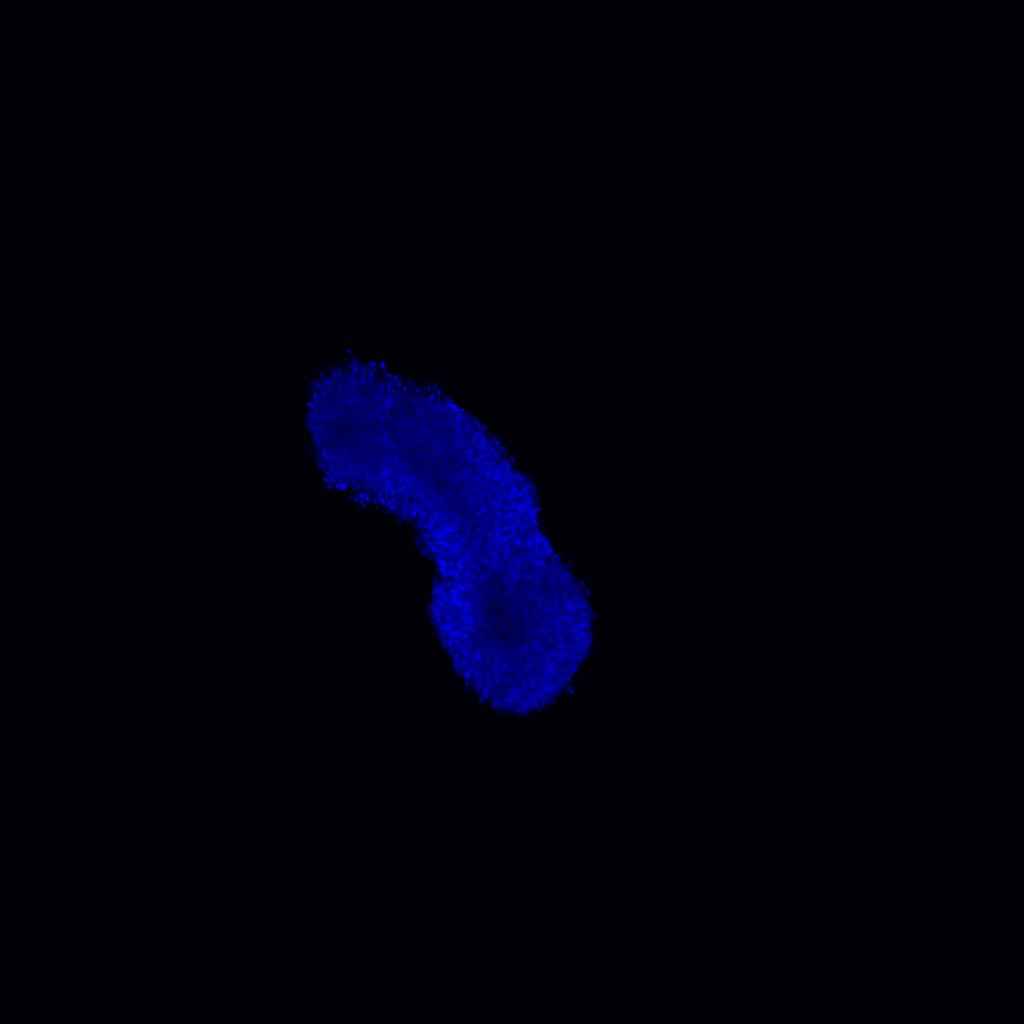

Supplement: Supplementary file 6 — Source data Fig. 4 [file 44318_2025_558_MOESM6_ESM.zip › Figure 4/panel 4B/NT_Nanog_3uM/fila51897_RGB_DAPI.tif]

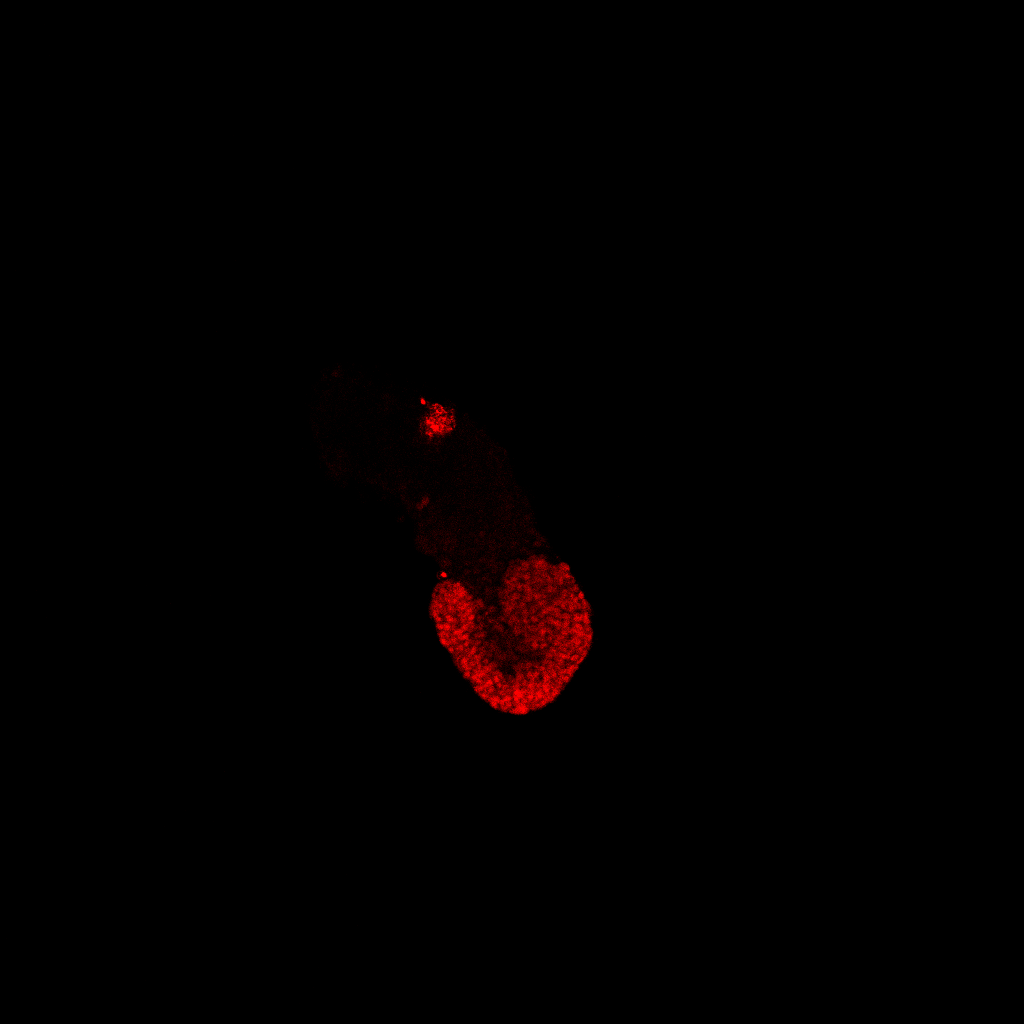

Supplement: Supplementary file 6 — Source data Fig. 4 [file 44318_2025_558_MOESM6_ESM.zip › Figure 4/panel 4B/NT_Nanog_3uM/fila51897_RGB_Alexa Fluor 594 cadaverine_H2O.tif]

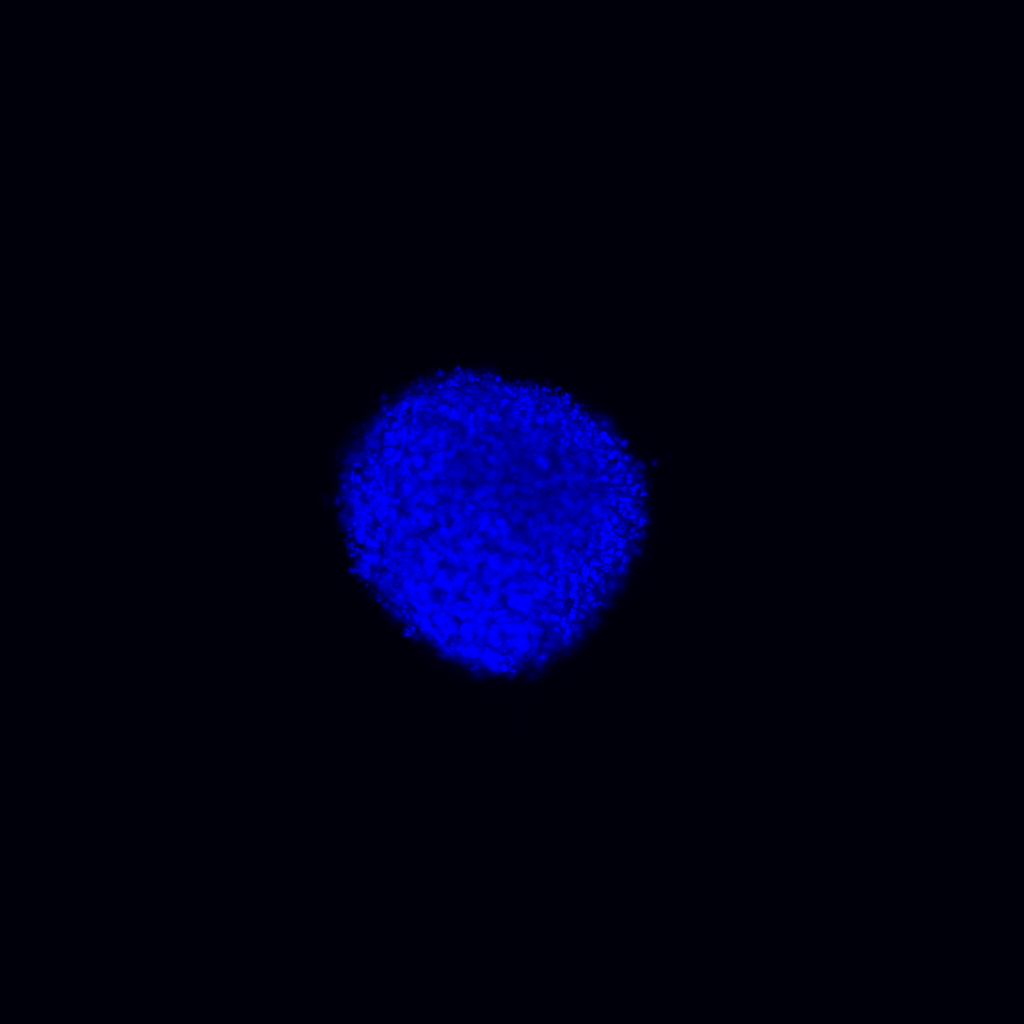

Supplement: Supplementary file 6 — Source data Fig. 4 [file 44318_2025_558_MOESM6_ESM.zip › Figure 4/panel 4B/KD-1_Cdx2_3uM/seq9181_seq9181_RGB_DAPI.tif]

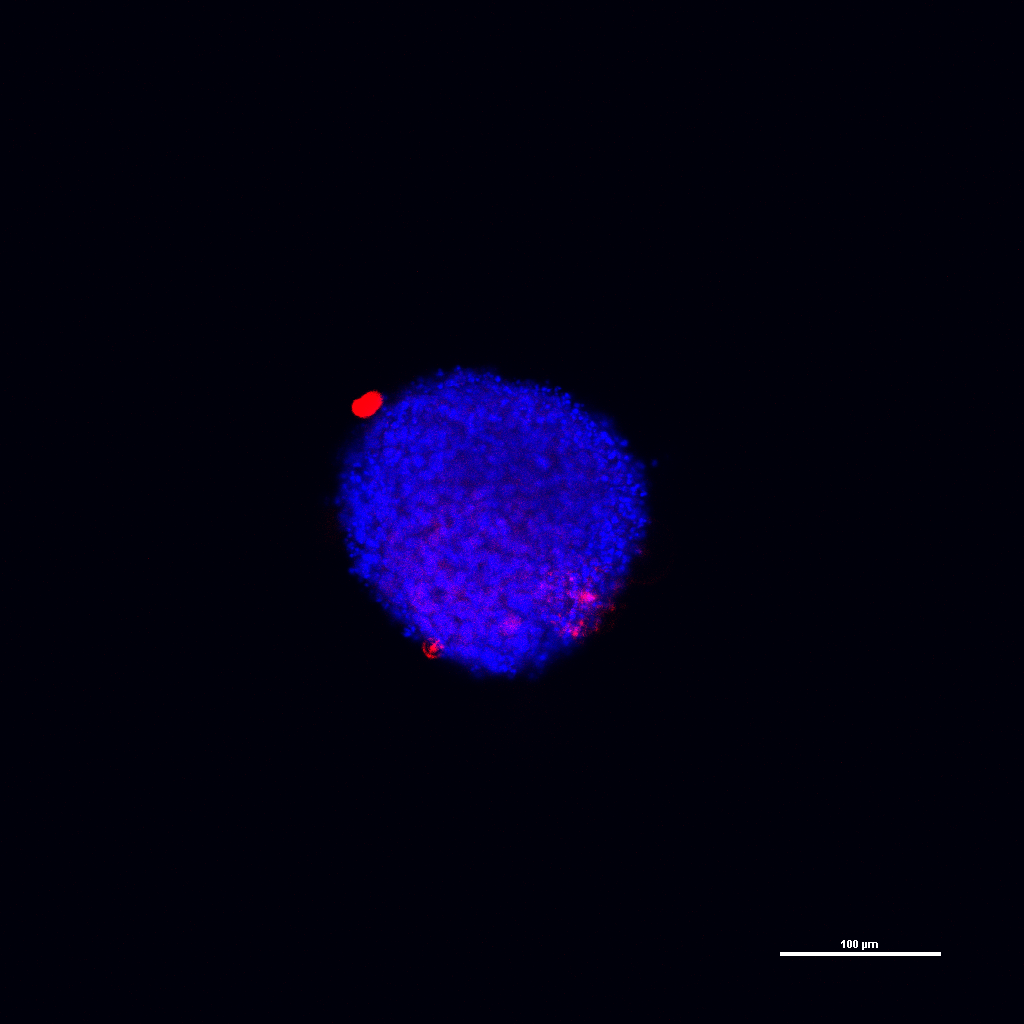

Supplement: Supplementary file 6 — Source data Fig. 4 [file 44318_2025_558_MOESM6_ESM.zip › Figure 4/panel 4B/KD-1_Cdx2_3uM/seq9181_seq9181_RGB.tif]

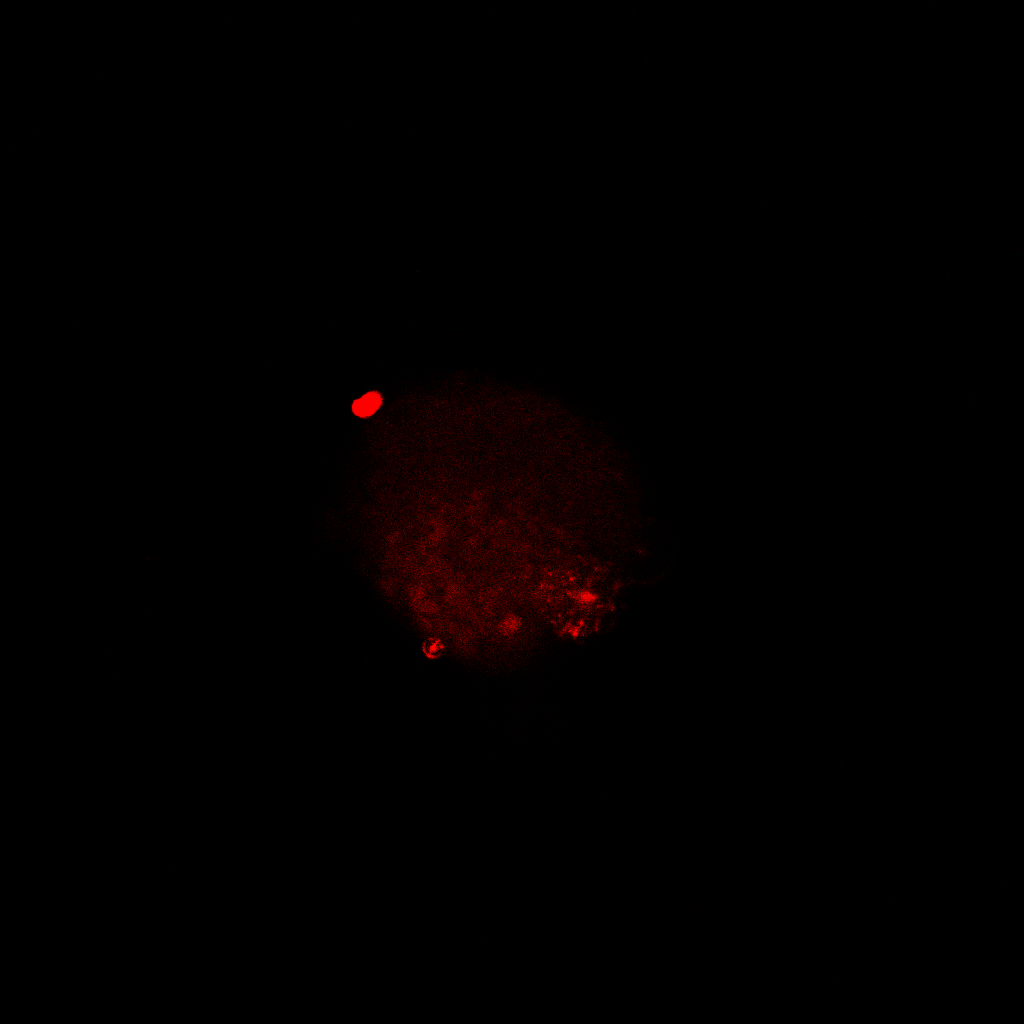

Supplement: Supplementary file 6 — Source data Fig. 4 [file 44318_2025_558_MOESM6_ESM.zip › Figure 4/panel 4B/KD-1_Cdx2_3uM/seq9181_seq9181_RGB_Texas Red.tif]

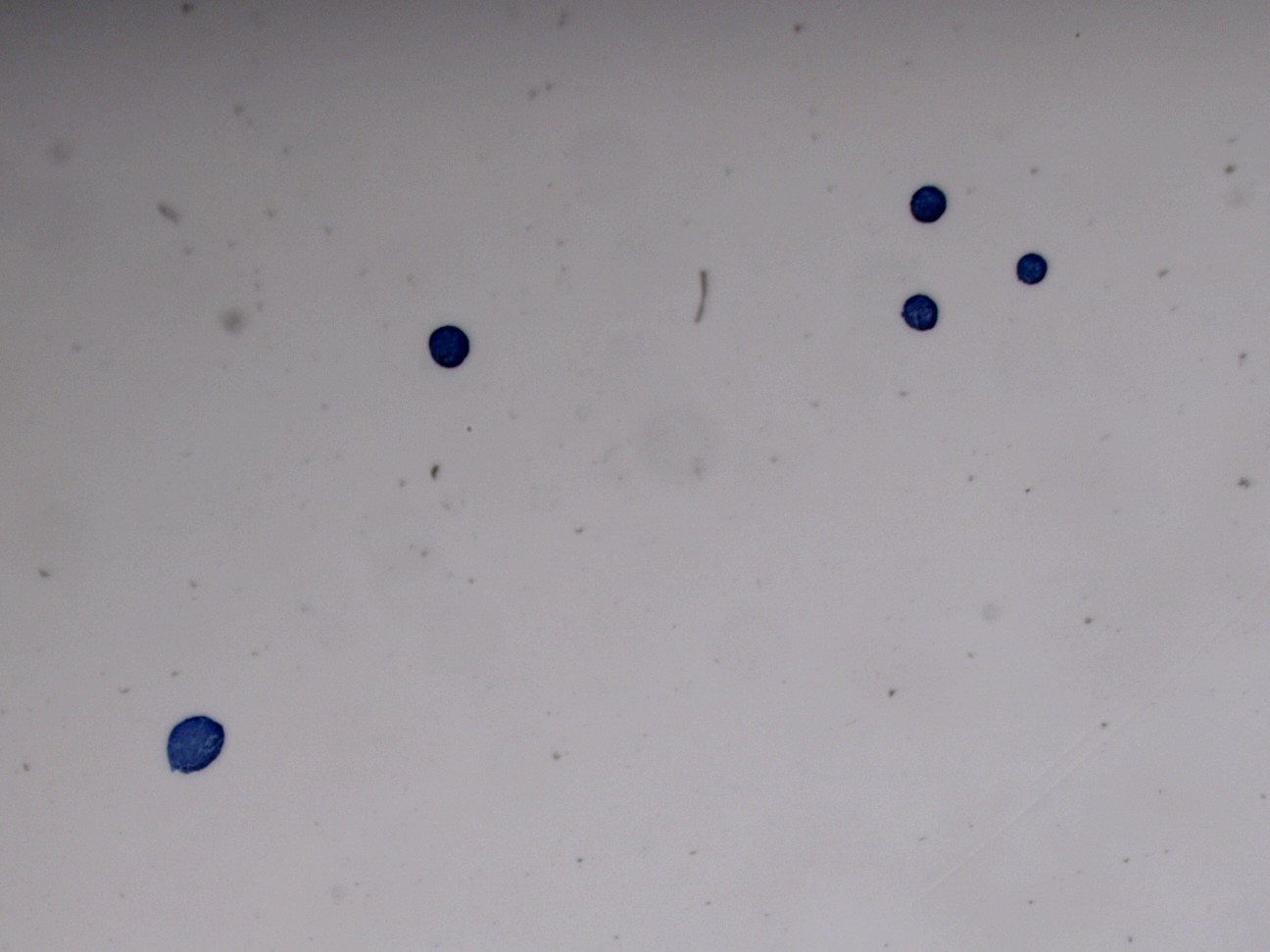

Supplement: Supplementary file 8 — Source data Fig. 6 [file 44318_2025_558_MOESM8_ESM.zip › Figure 6/panel 6B/KD-1_AP_5uM.tiff]

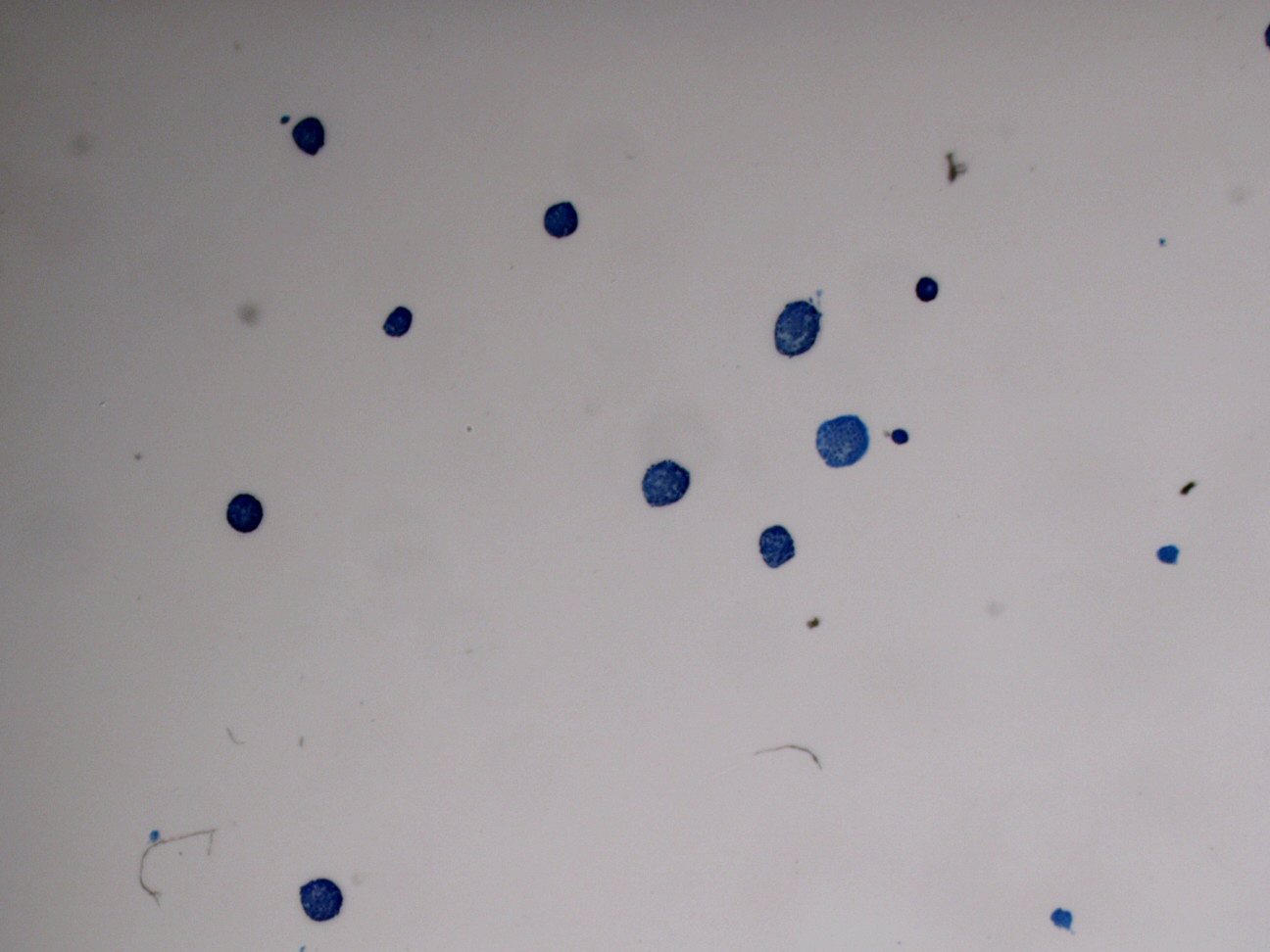

Supplement: Supplementary file 8 — Source data Fig. 6 [file 44318_2025_558_MOESM8_ESM.zip › Figure 6/panel 6B/KD-2_AP_3uM.tiff]

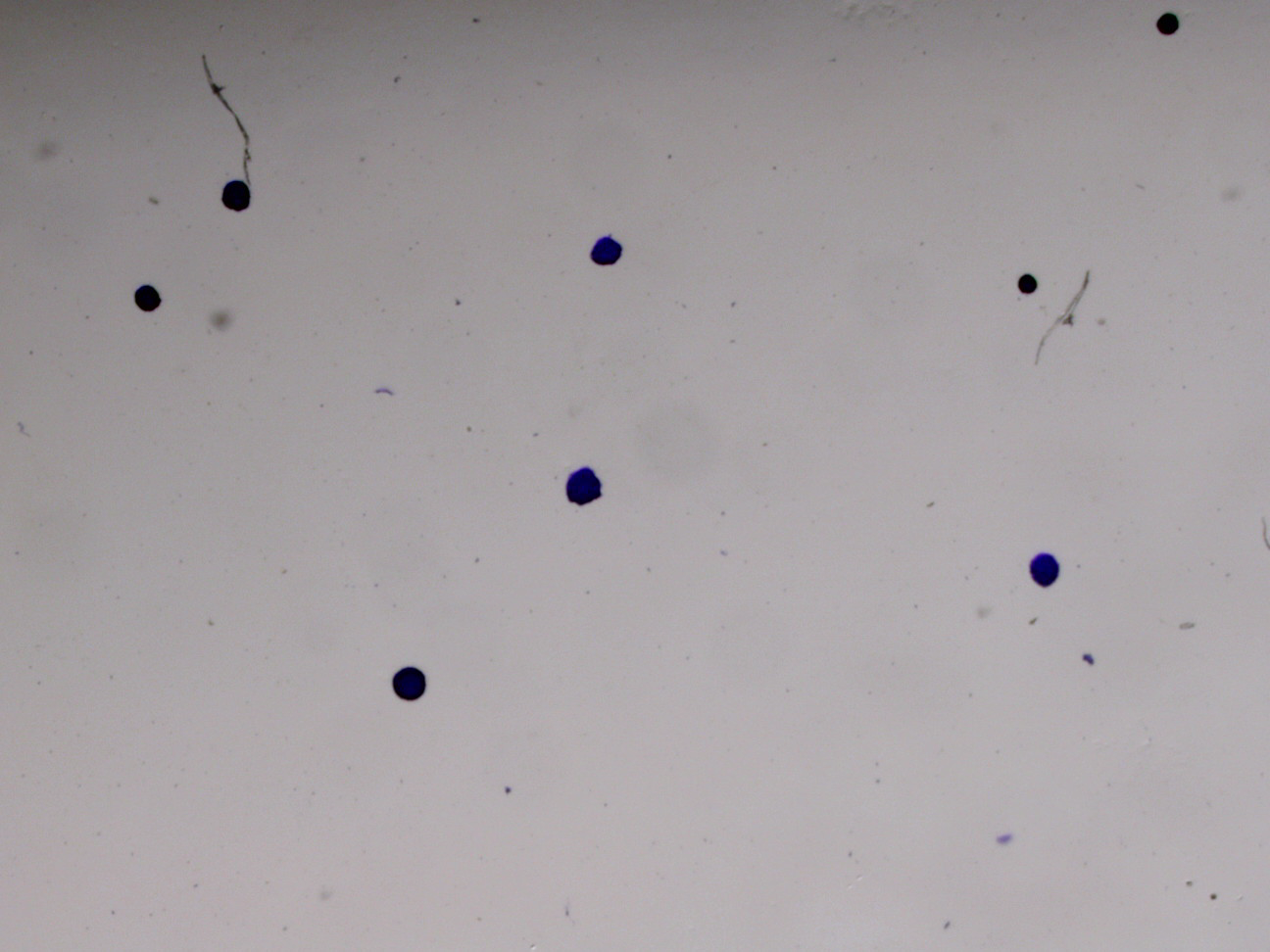

Supplement: Supplementary file 8 — Source data Fig. 6 [file 44318_2025_558_MOESM8_ESM.zip › Figure 6/panel 6B/KD-1_colony_5uM.tiff]

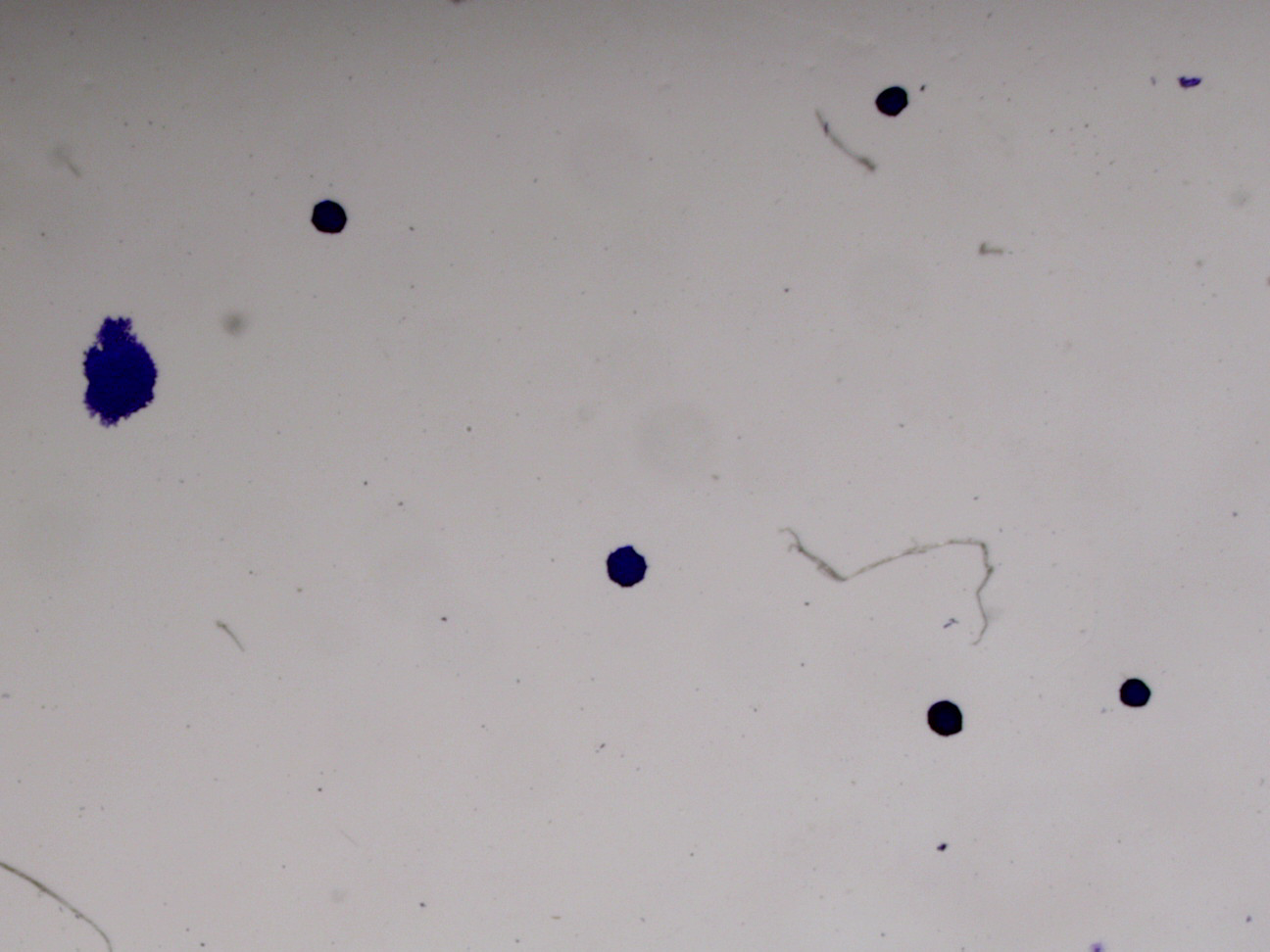

Supplement: Supplementary file 8 — Source data Fig. 6 [file 44318_2025_558_MOESM8_ESM.zip › Figure 6/panel 6B/KD-2_colony_6uM.tiff]

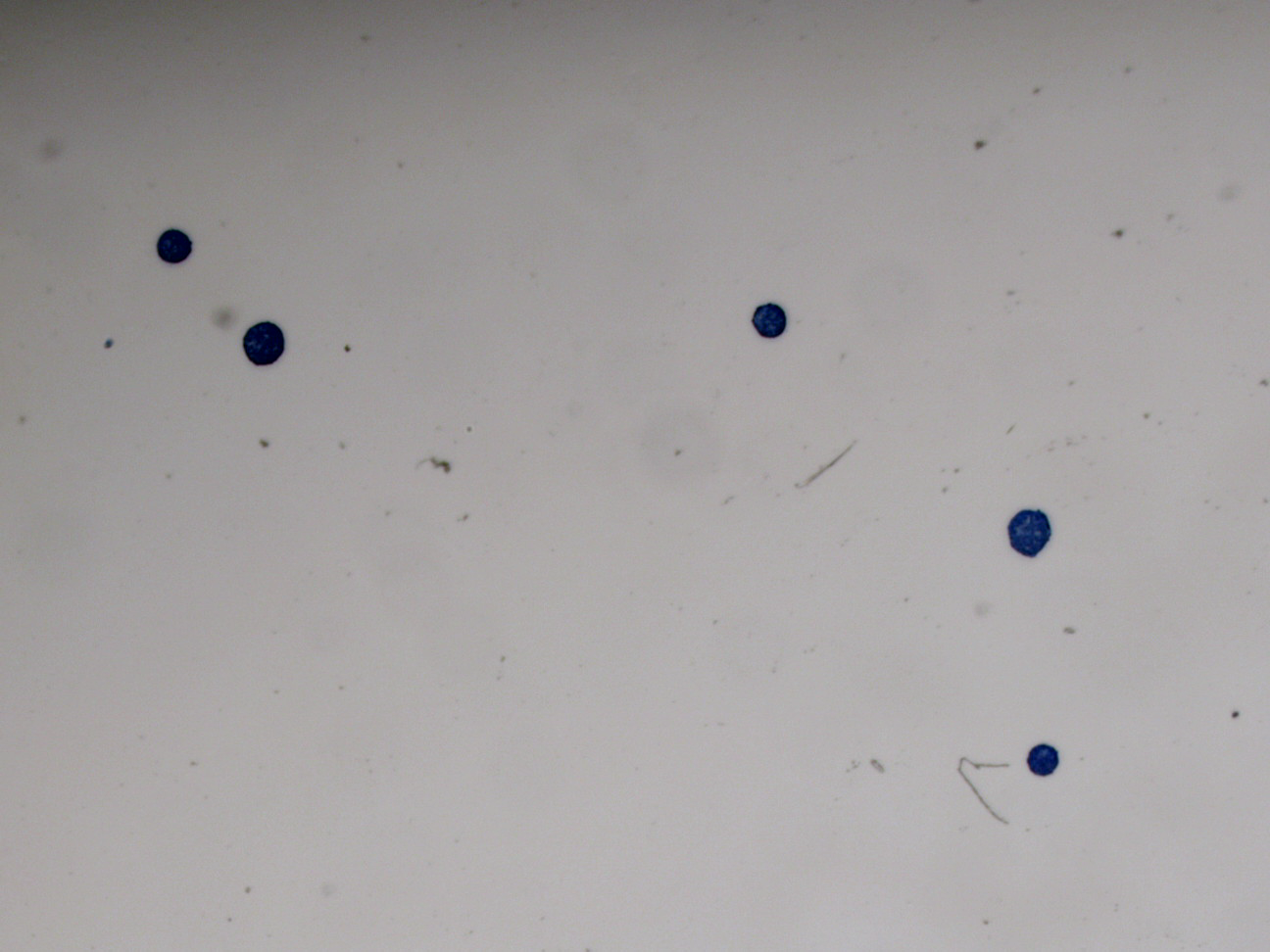

Supplement: Supplementary file 8 — Source data Fig. 6 [file 44318_2025_558_MOESM8_ESM.zip › Figure 6/panel 6B/NT_AP_3uM.tiff]

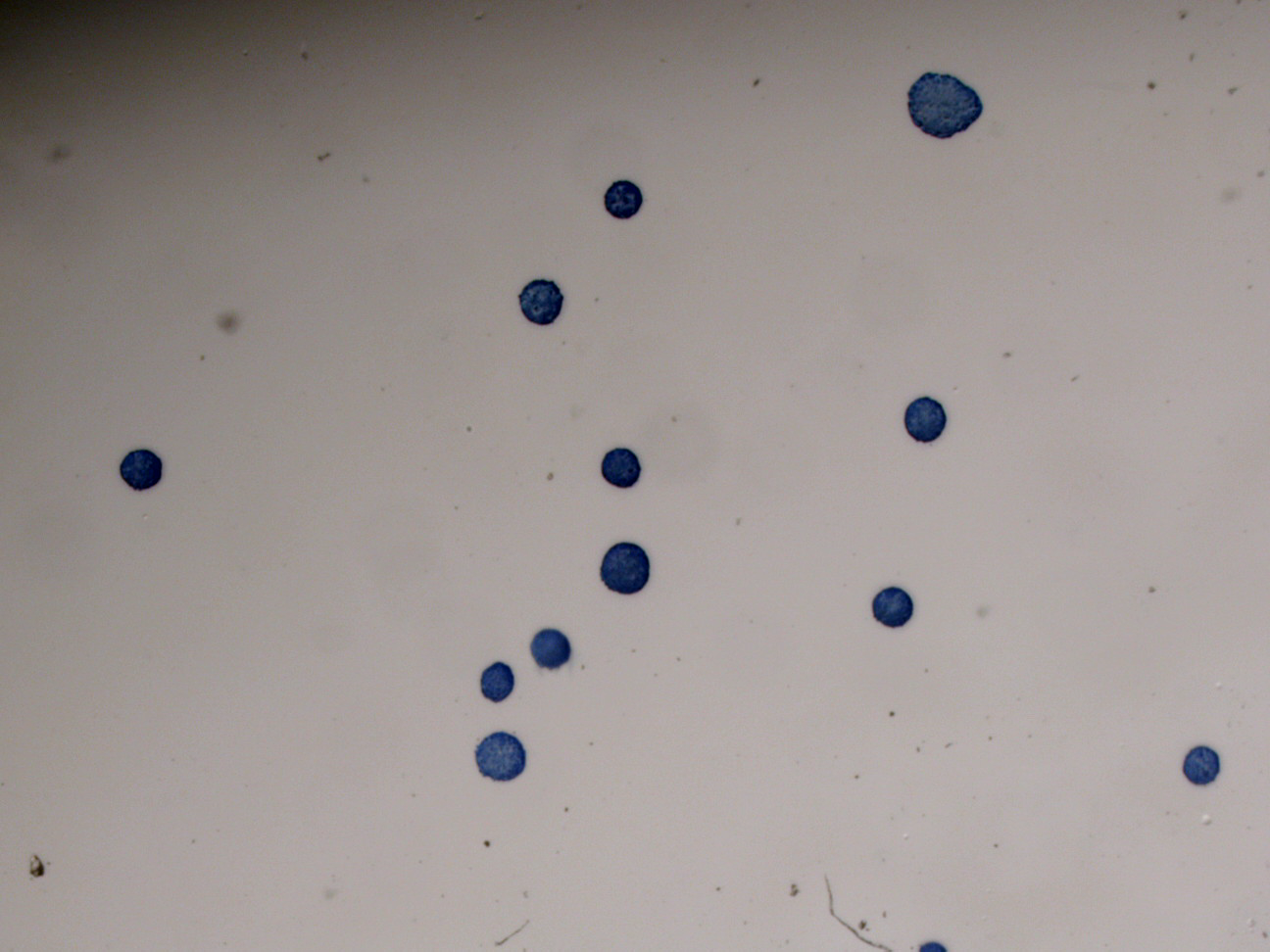

Supplement: Supplementary file 8 — Source data Fig. 6 [file 44318_2025_558_MOESM8_ESM.zip › Figure 6/panel 6B/KD-1_AP_3uM.tiff]

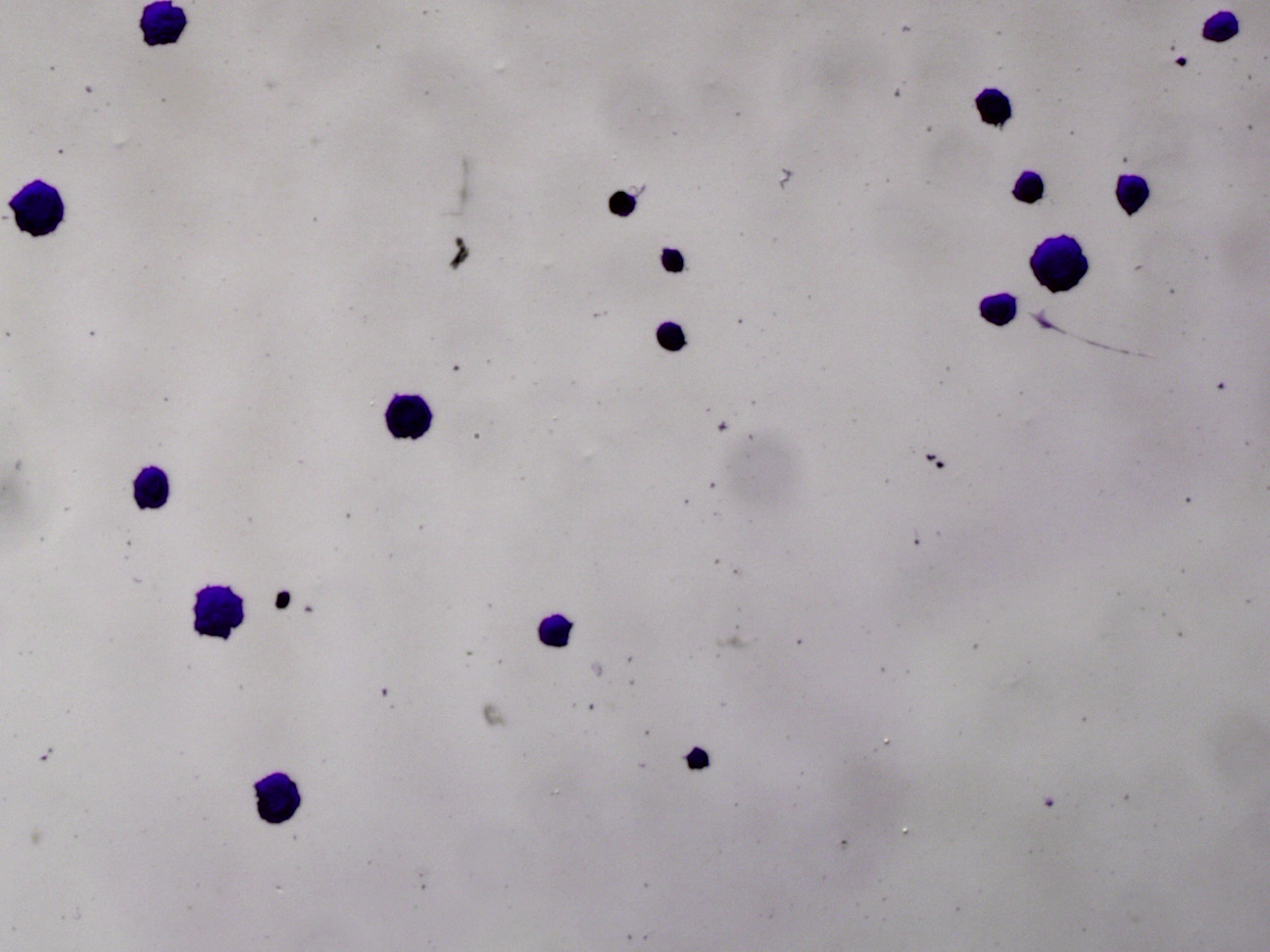

Supplement: Supplementary file 8 — Source data Fig. 6 [file 44318_2025_558_MOESM8_ESM.zip › Figure 6/panel 6B/KD-1_colony_3uM.tiff]

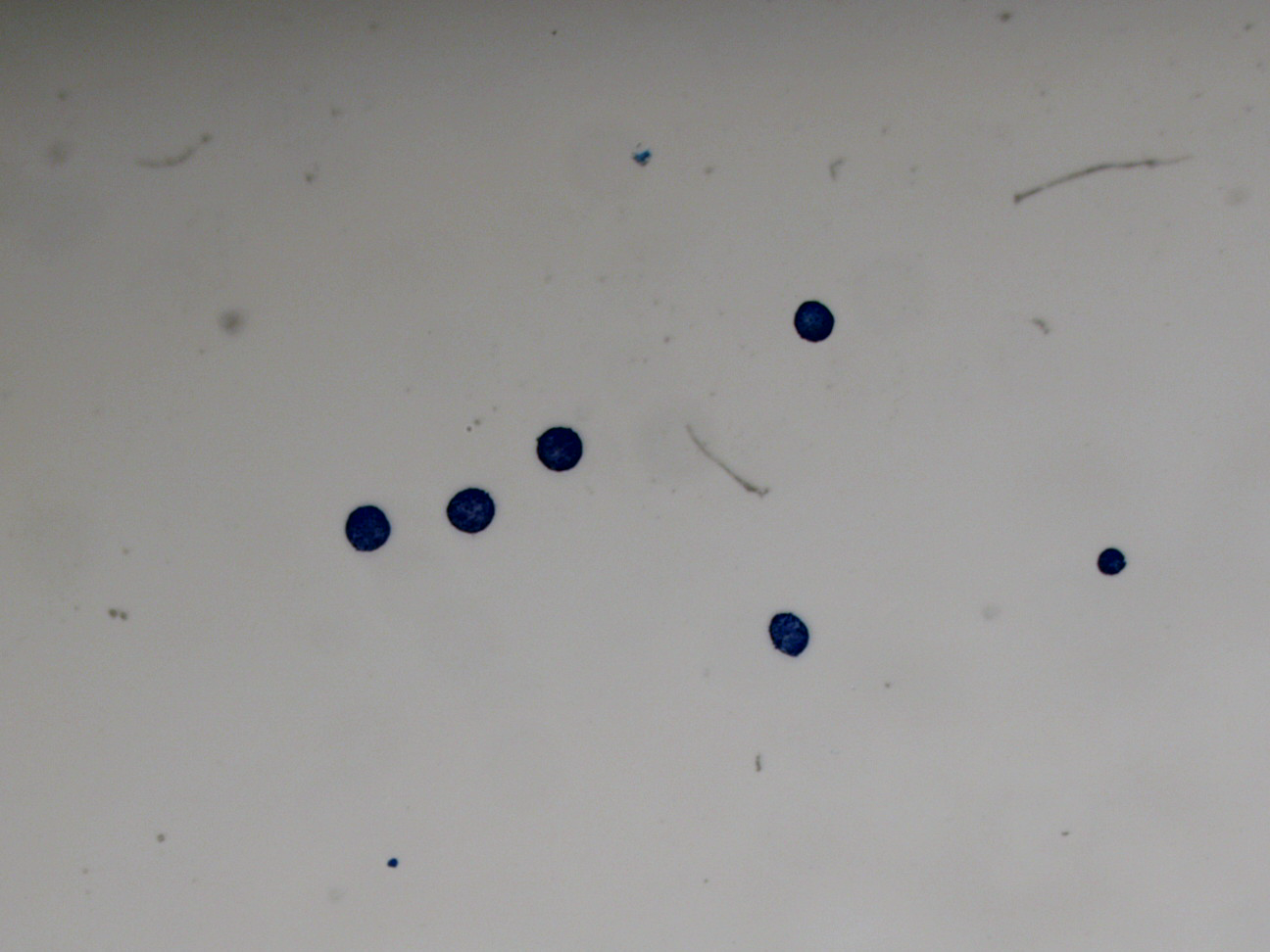

Supplement: Supplementary file 8 — Source data Fig. 6 [file 44318_2025_558_MOESM8_ESM.zip › Figure 6/panel 6B/KD-2_AP_6uM.tiff]

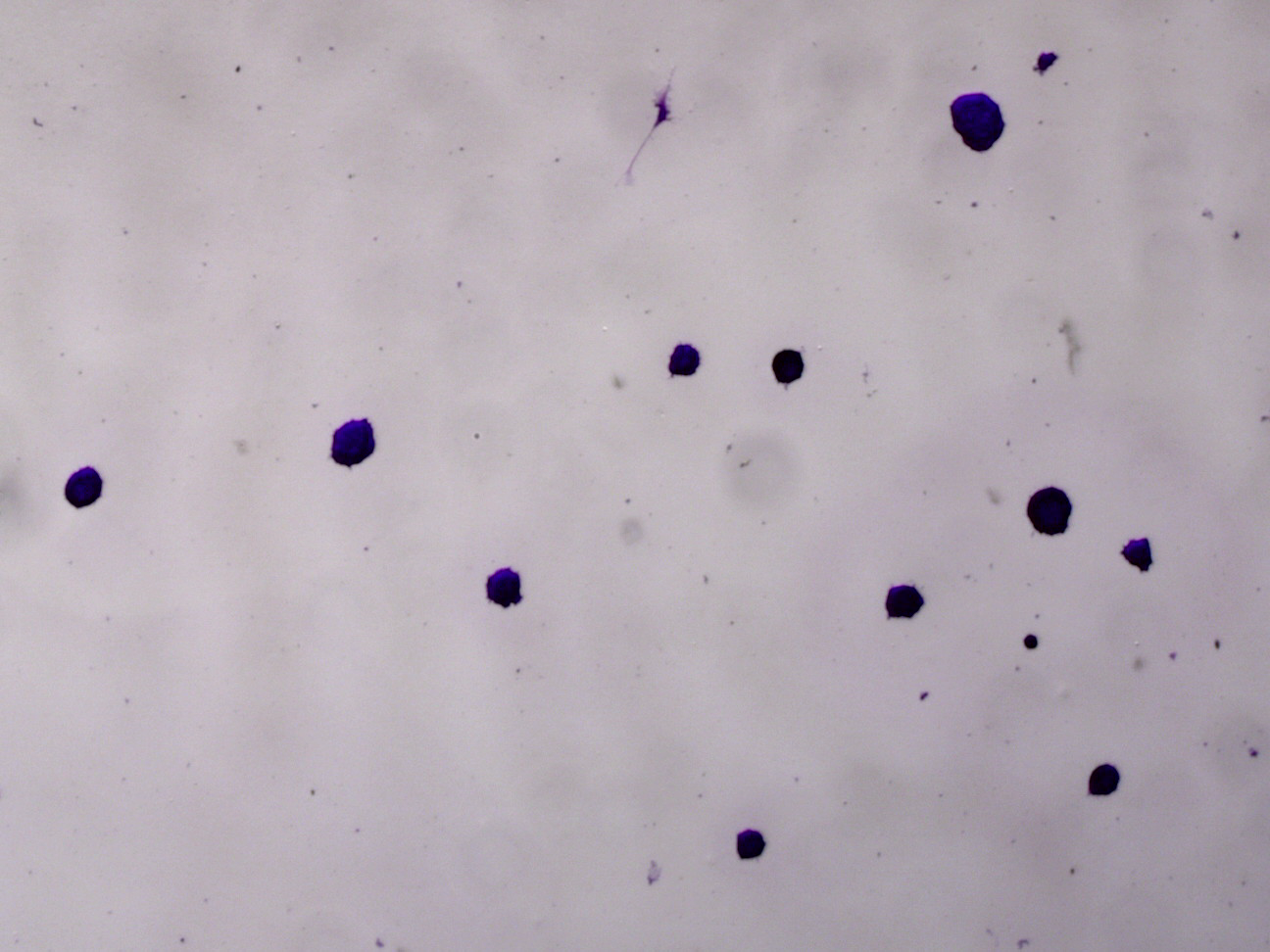

Supplement: Supplementary file 8 — Source data Fig. 6 [file 44318_2025_558_MOESM8_ESM.zip › Figure 6/panel 6B/KD-2_colony_3uM.tiff]

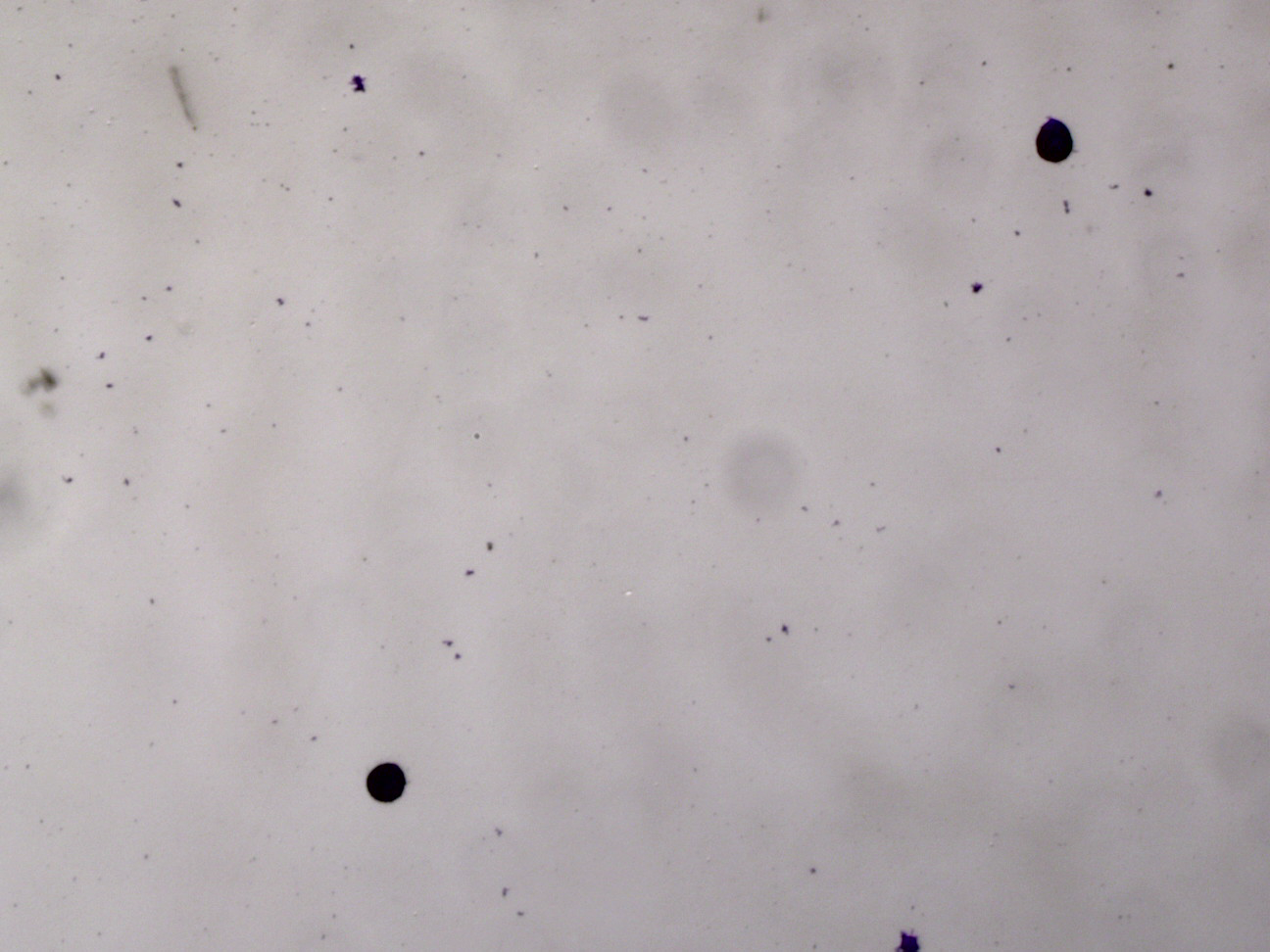

Supplement: Supplementary file 8 — Source data Fig. 6 [file 44318_2025_558_MOESM8_ESM.zip › Figure 6/panel 6B/NT_colony_3uM.tiff]

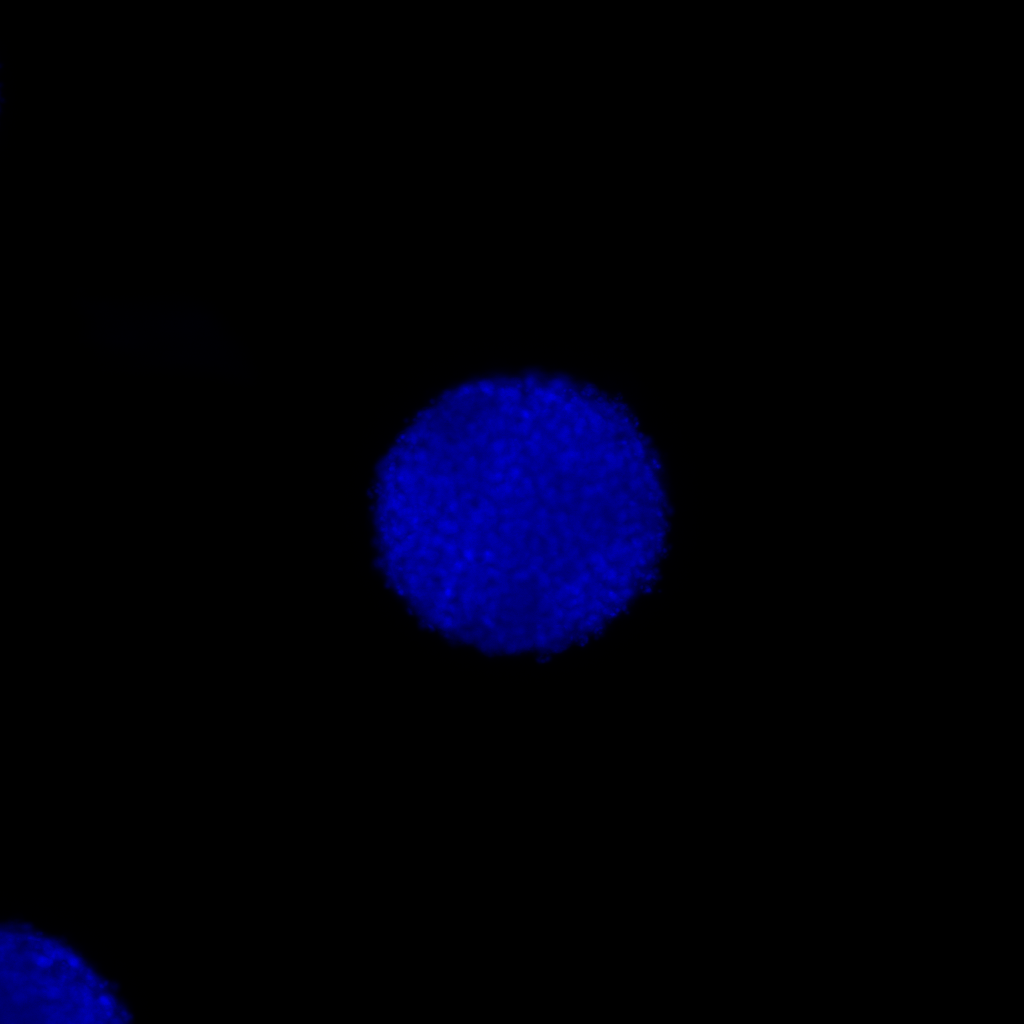

Supplement: Supplementary file 8 — Source data Fig. 6 [file 44318_2025_558_MOESM8_ESM.zip › Figure 6/panel 6C/KD-1_Ki67_3uM/seq11032_seq11032_RGB_DAPI.tif]

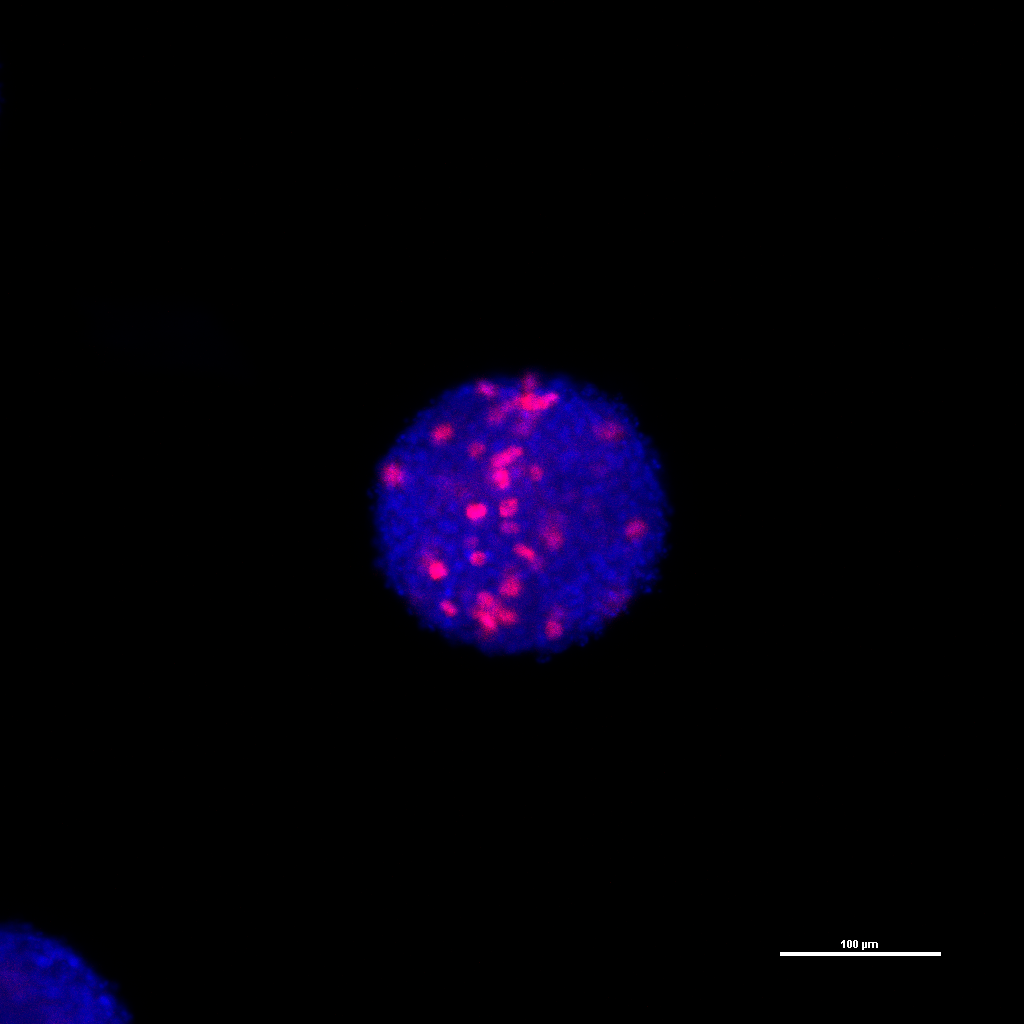

Supplement: Supplementary file 8 — Source data Fig. 6 [file 44318_2025_558_MOESM8_ESM.zip › Figure 6/panel 6C/KD-1_Ki67_3uM/seq11032_seq11032_RGB.tif]

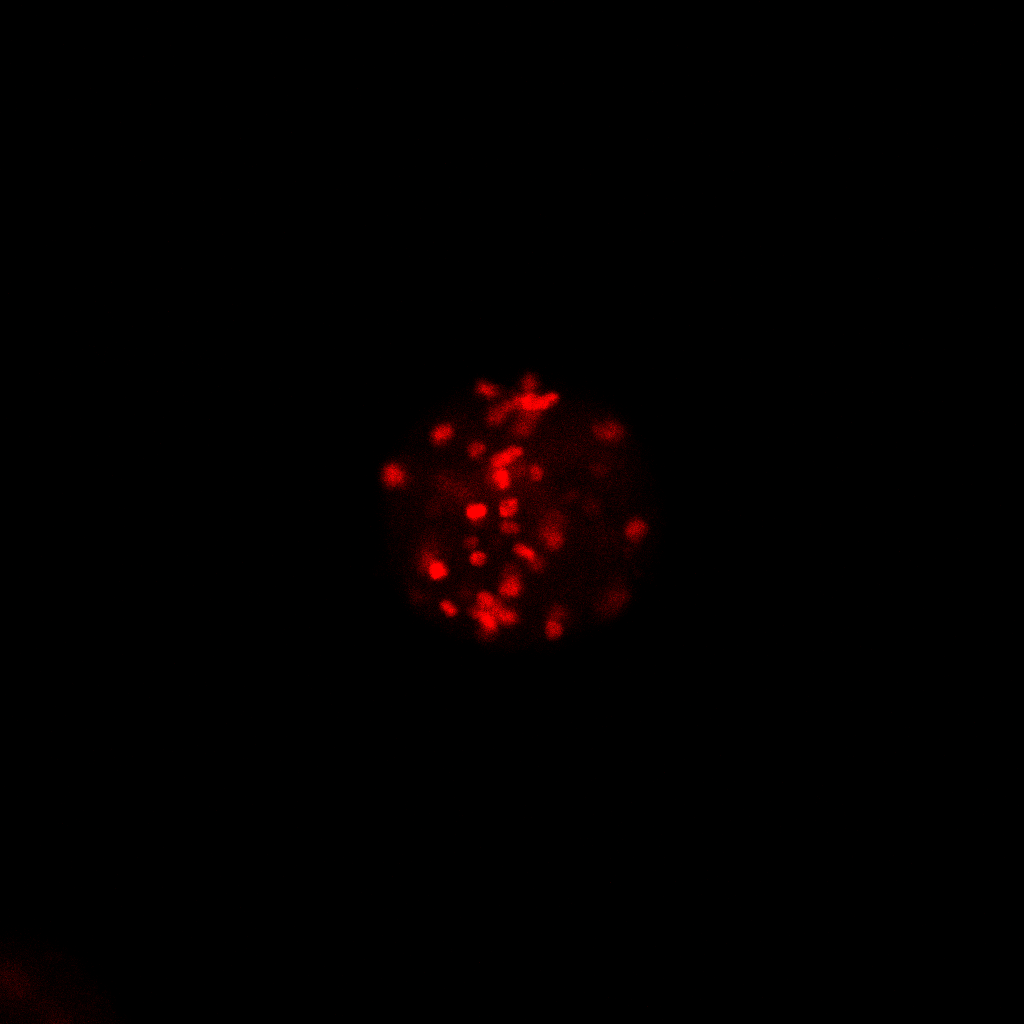

Supplement: Supplementary file 8 — Source data Fig. 6 [file 44318_2025_558_MOESM8_ESM.zip › Figure 6/panel 6C/KD-1_Ki67_3uM/seq11032_seq11032_RGB_TRITC.tif]

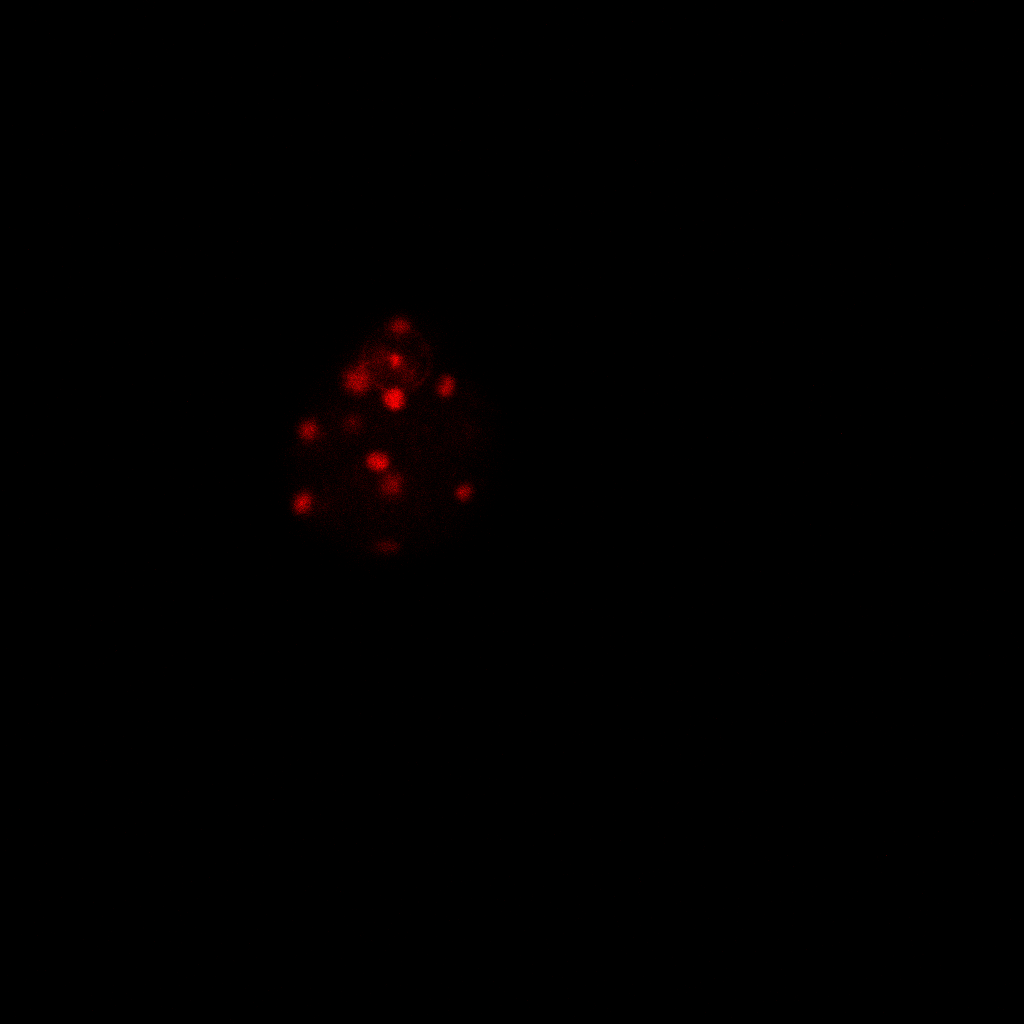

Supplement: Supplementary file 8 — Source data Fig. 6 [file 44318_2025_558_MOESM8_ESM.zip › Figure 6/panel 6C/NT_Ki67/seq11030_seq11030_RGB_TRITC.tif]

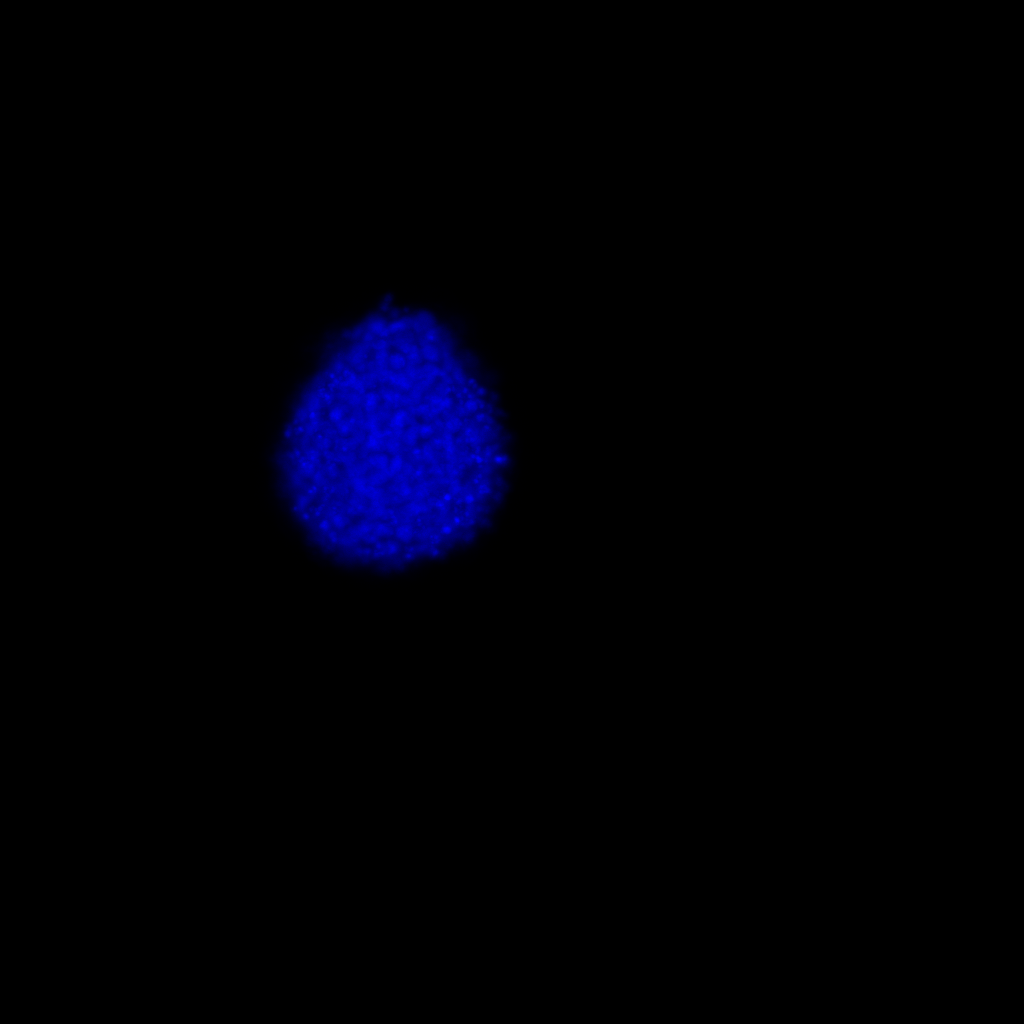

Supplement: Supplementary file 8 — Source data Fig. 6 [file 44318_2025_558_MOESM8_ESM.zip › Figure 6/panel 6C/NT_Ki67/seq11030_seq11030_RGB_DAPI.tif]

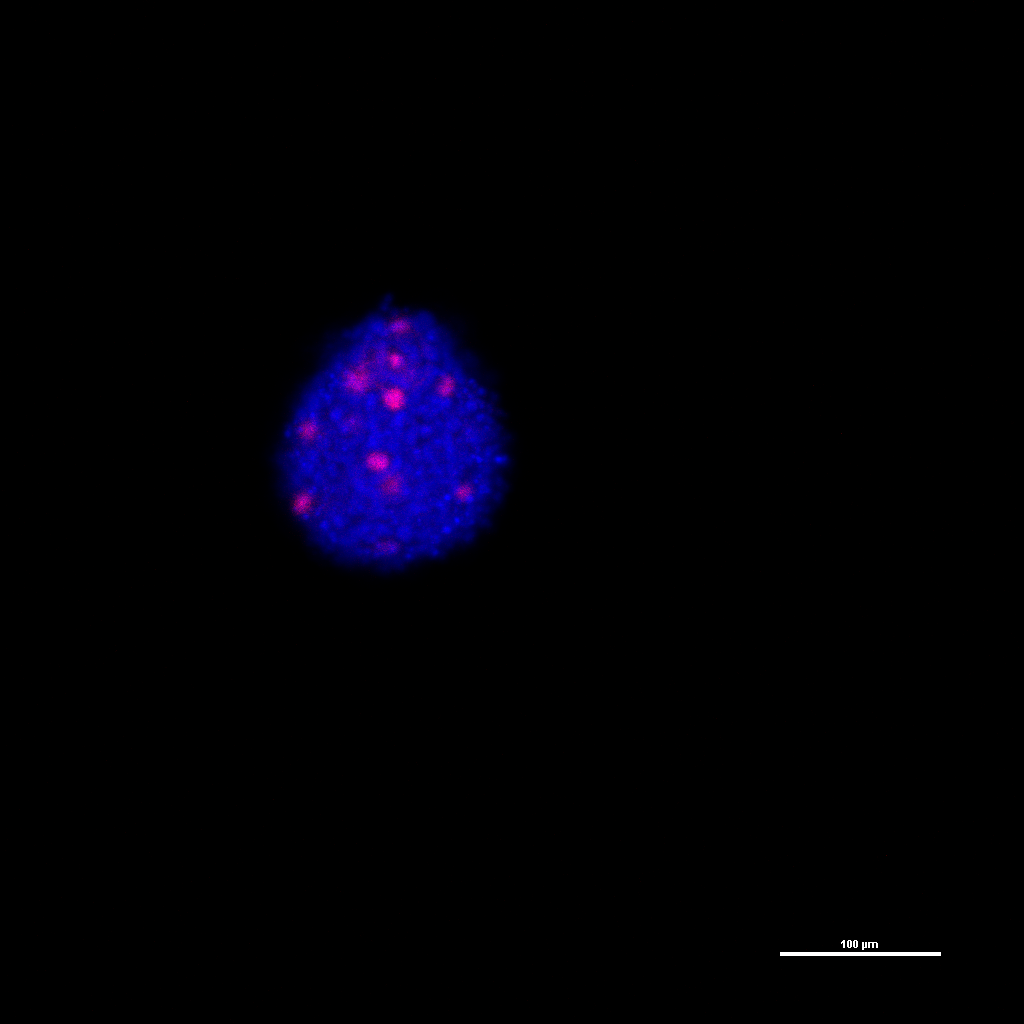

Supplement: Supplementary file 8 — Source data Fig. 6 [file 44318_2025_558_MOESM8_ESM.zip › Figure 6/panel 6C/NT_Ki67/seq11030_seq11030_RGB.tif]

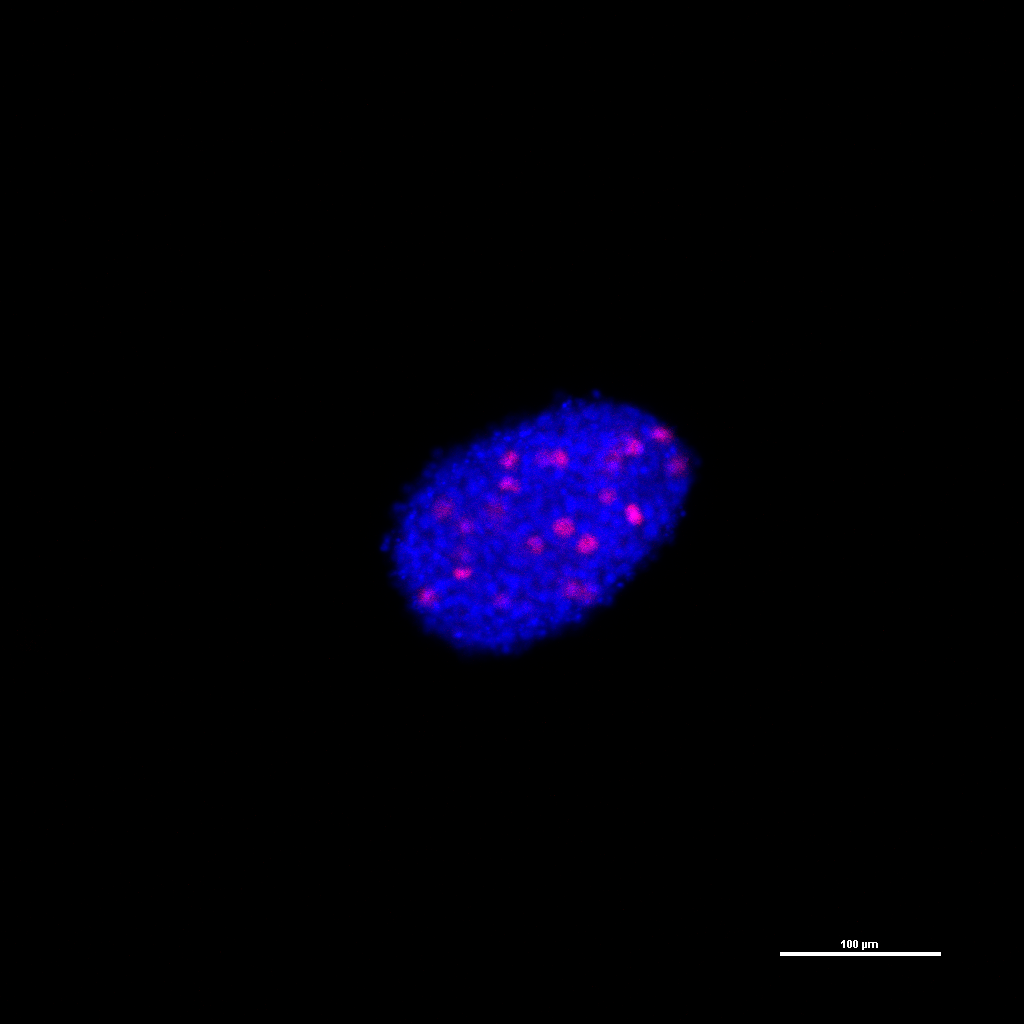

Supplement: Supplementary file 8 — Source data Fig. 6 [file 44318_2025_558_MOESM8_ESM.zip › Figure 6/panel 6C/KD-2_Ki67_6uM/seq11043_seq11043_RGB.tif]

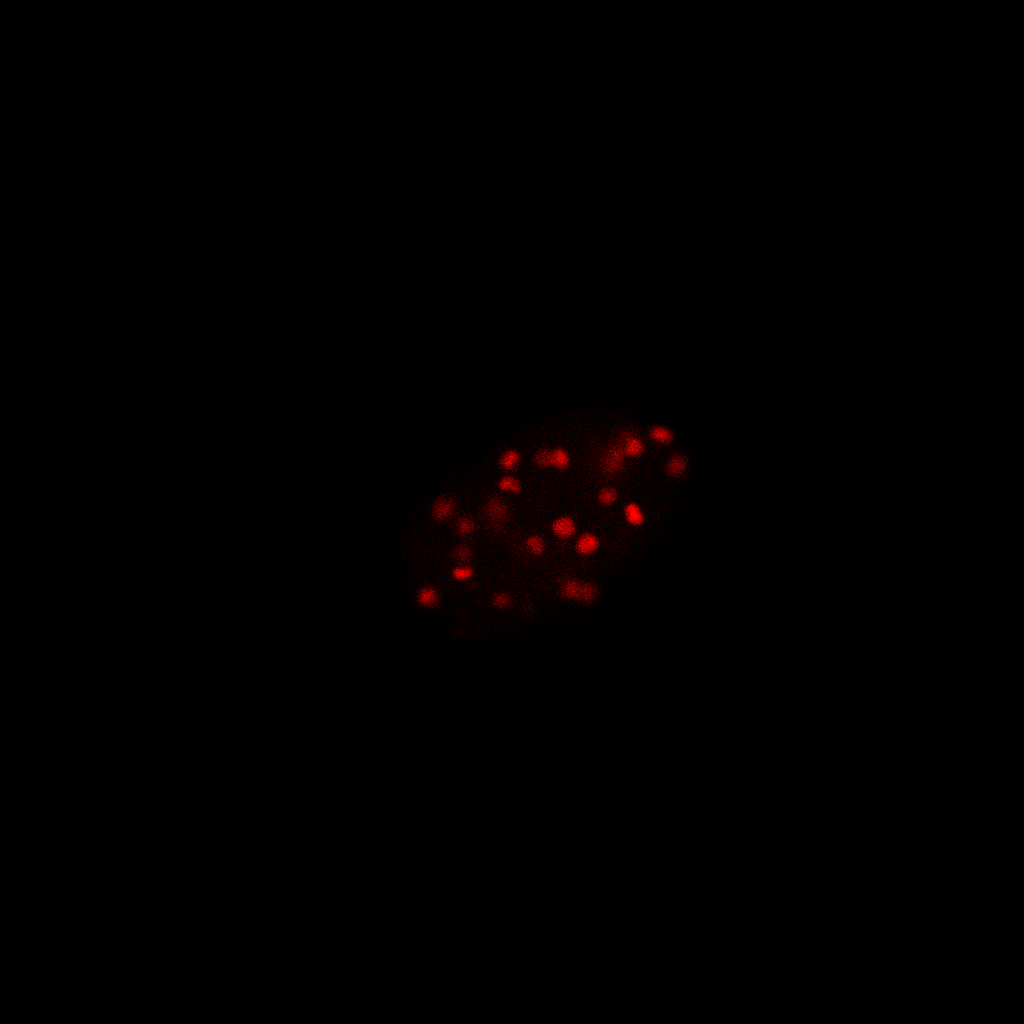

Supplement: Supplementary file 8 — Source data Fig. 6 [file 44318_2025_558_MOESM8_ESM.zip › Figure 6/panel 6C/KD-2_Ki67_6uM/seq11043_seq11043_RGB_TRITC.tif]

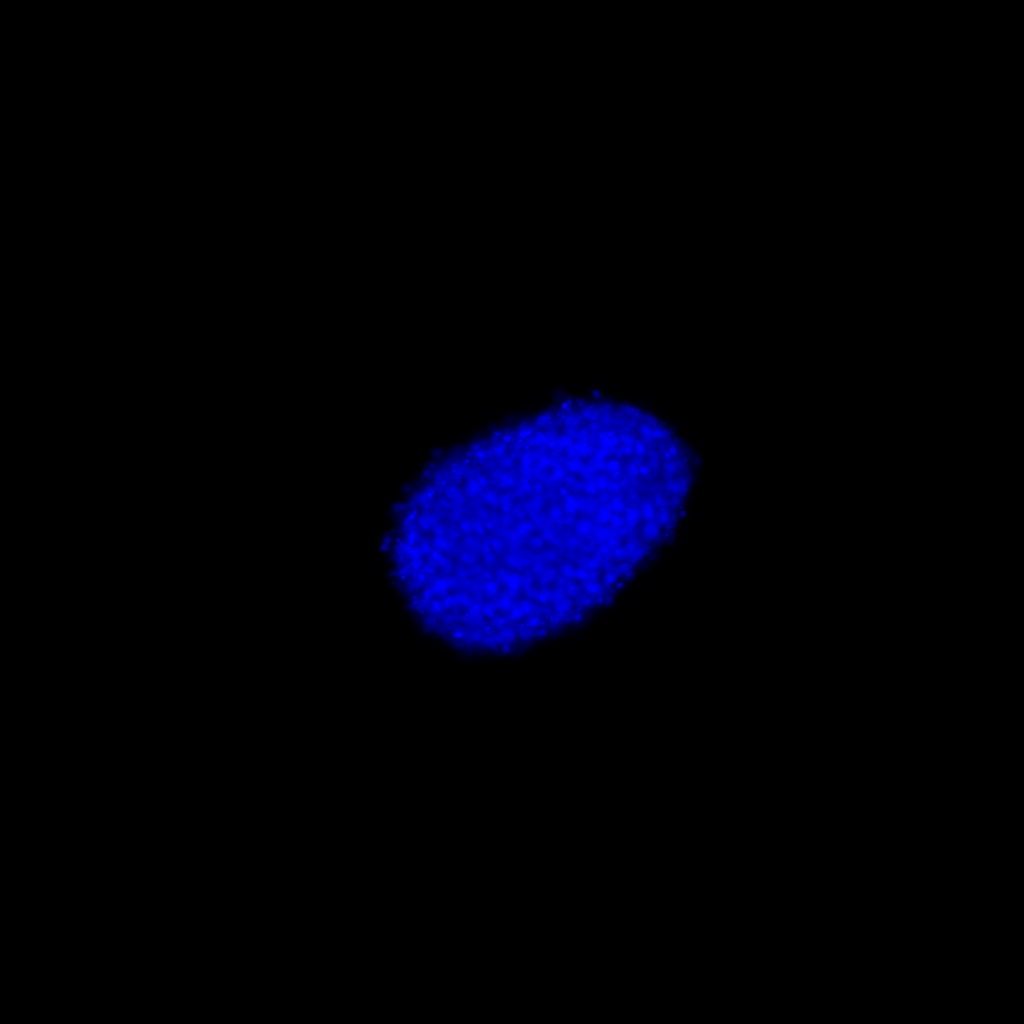

Supplement: Supplementary file 8 — Source data Fig. 6 [file 44318_2025_558_MOESM8_ESM.zip › Figure 6/panel 6C/KD-2_Ki67_6uM/seq11043_seq11043_RGB_DAPI.tif]

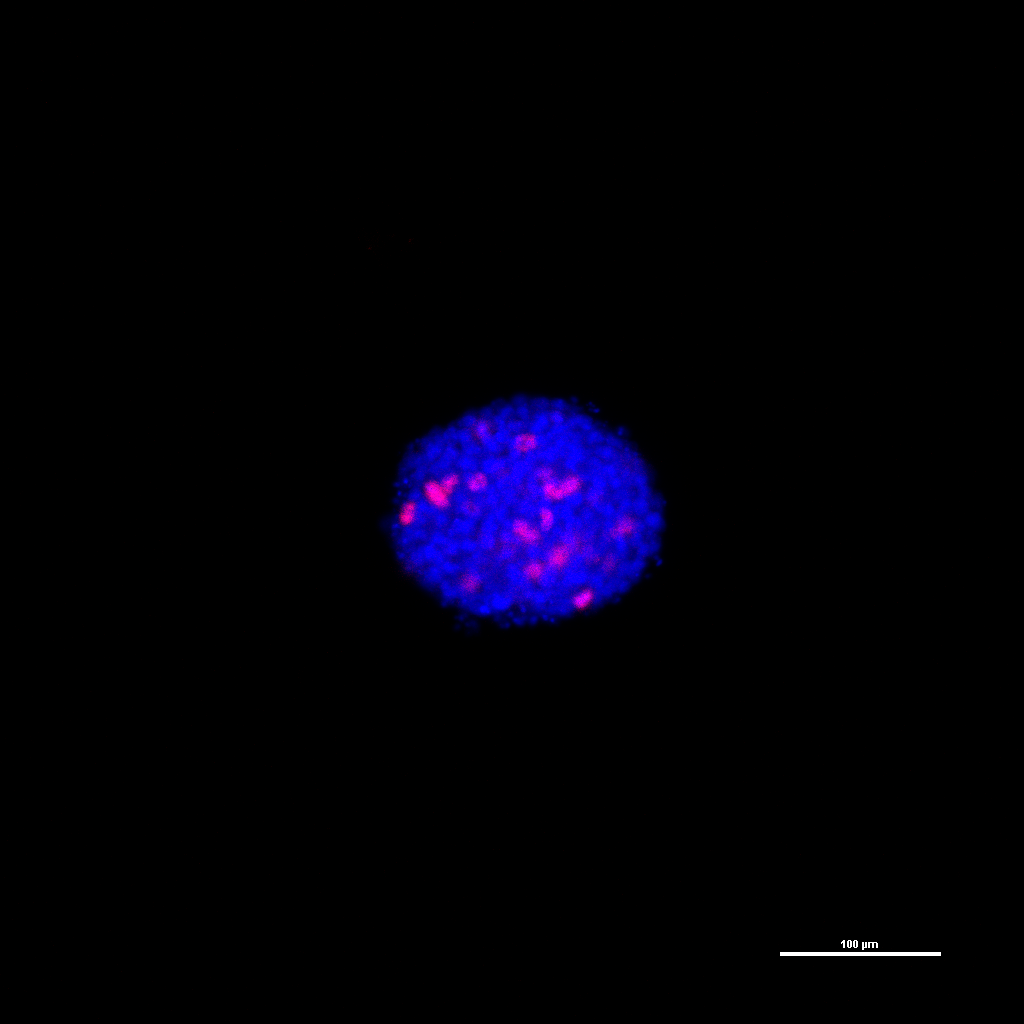

Supplement: Supplementary file 8 — Source data Fig. 6 [file 44318_2025_558_MOESM8_ESM.zip › Figure 6/panel 6C/KD-1_Ki67_5uM/seq11039_seq11039_RGB.tif]

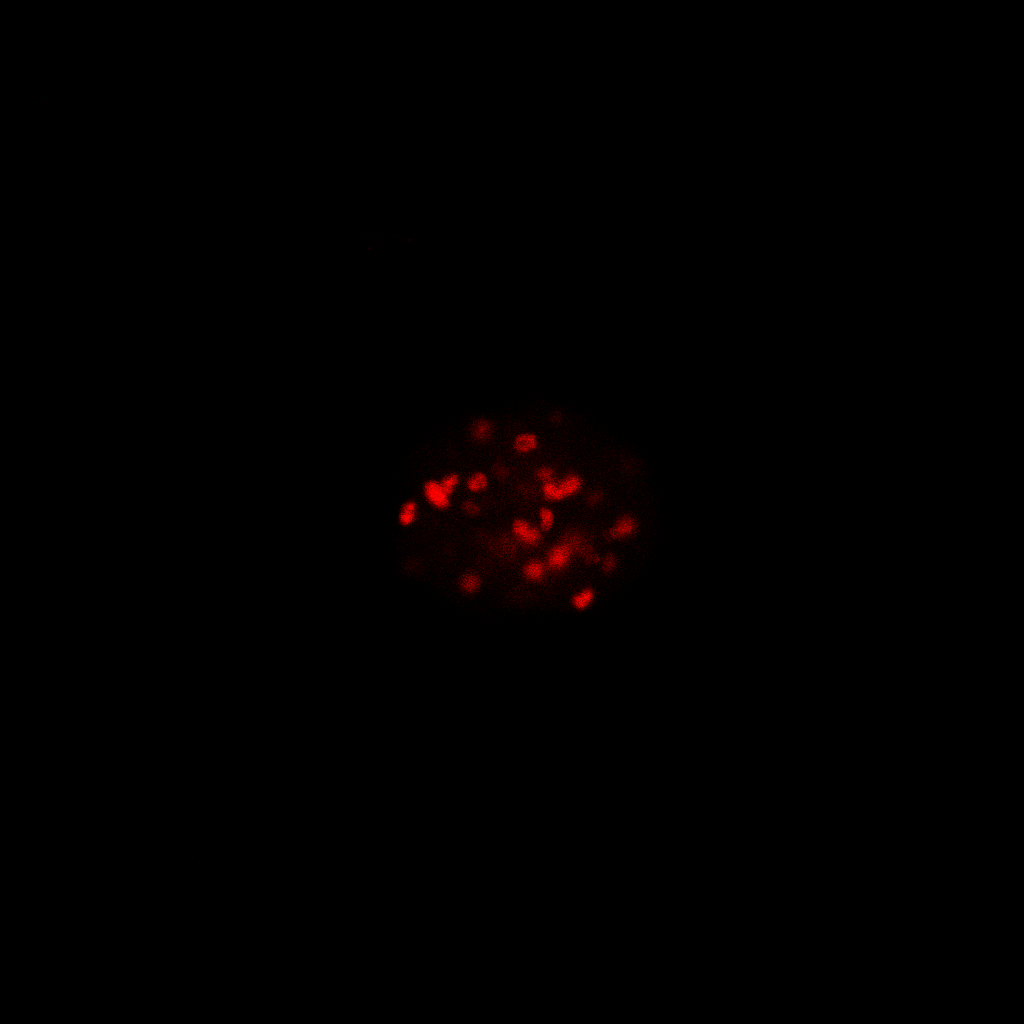

Supplement: Supplementary file 8 — Source data Fig. 6 [file 44318_2025_558_MOESM8_ESM.zip › Figure 6/panel 6C/KD-1_Ki67_5uM/seq11039_seq11039_RGB_TRITC.tif]

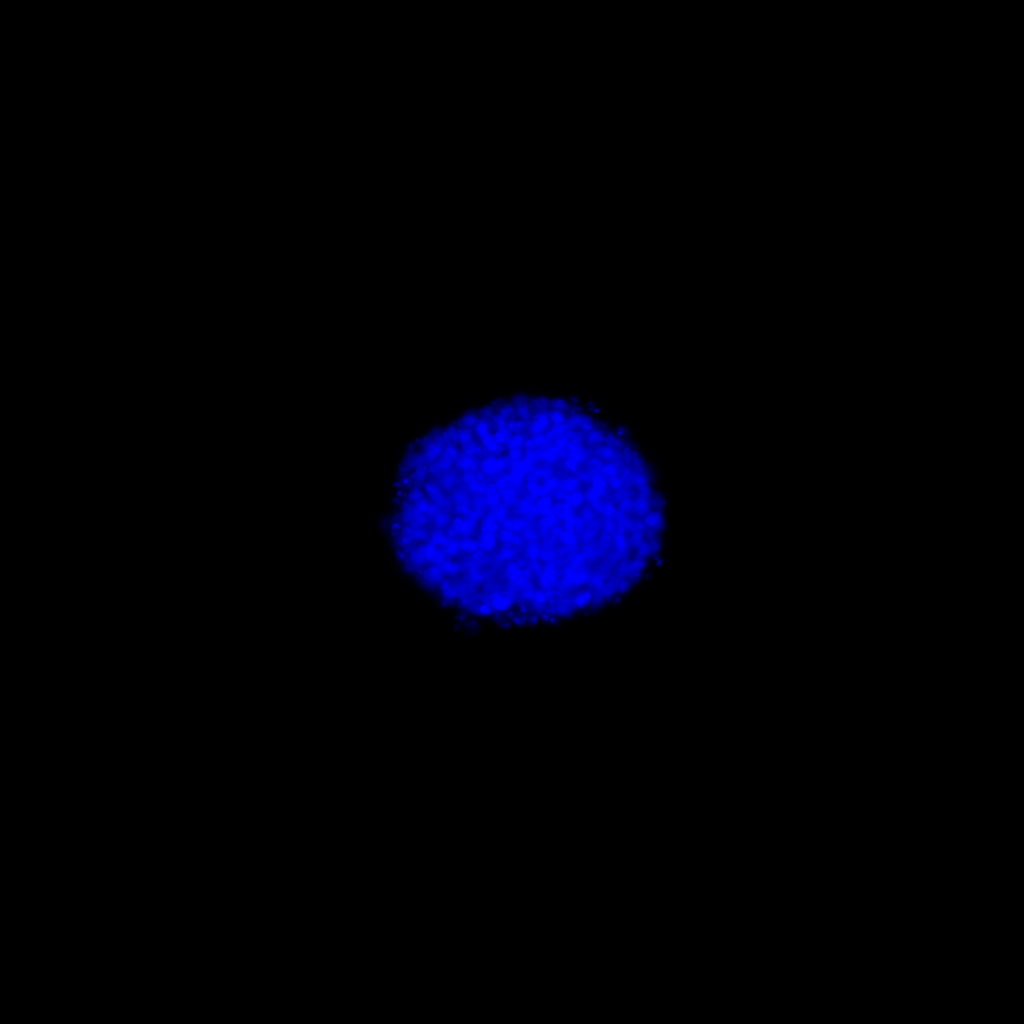

Supplement: Supplementary file 8 — Source data Fig. 6 [file 44318_2025_558_MOESM8_ESM.zip › Figure 6/panel 6C/KD-1_Ki67_5uM/seq11039_seq11039_RGB_DAPI.tif]

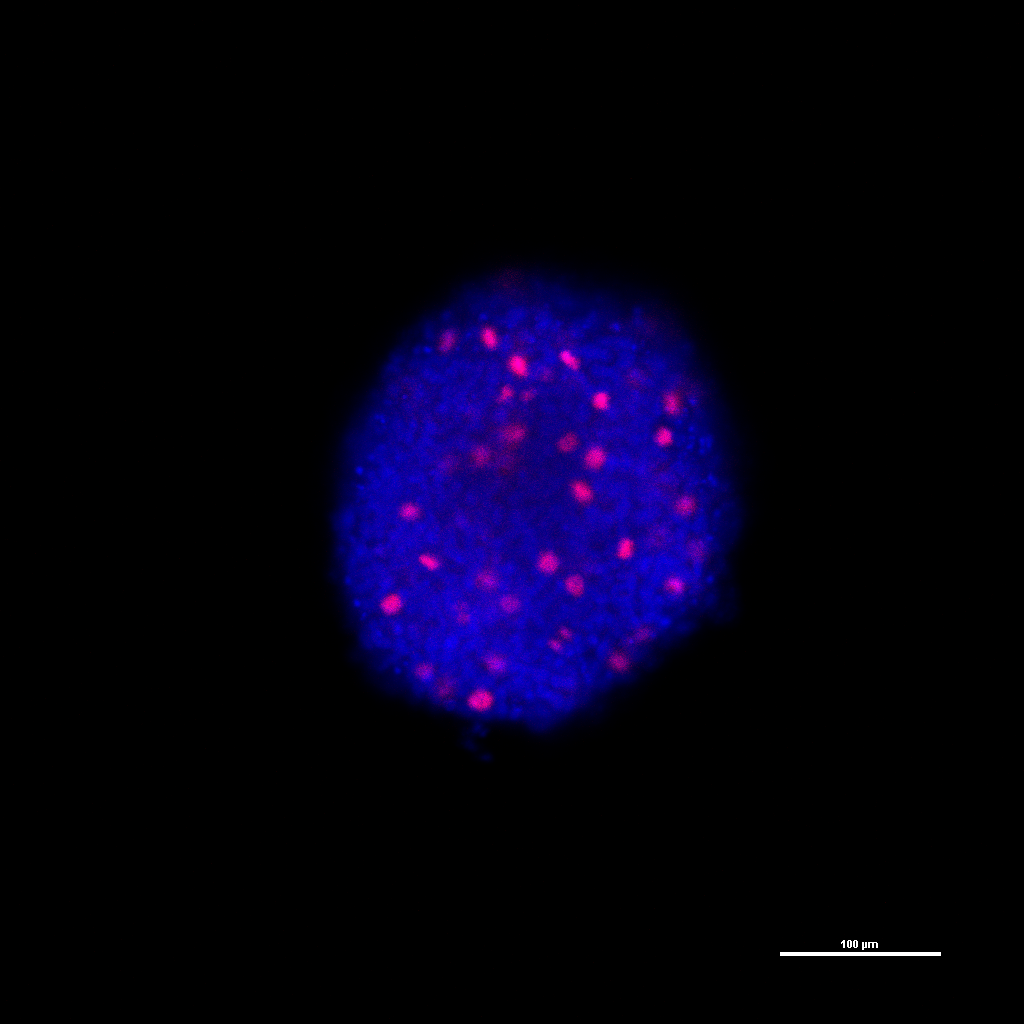

Supplement: Supplementary file 8 — Source data Fig. 6 [file 44318_2025_558_MOESM8_ESM.zip › Figure 6/panel 6C/KD-2_Ki67_3uM/seq11038_seq11038_RGB.tif]

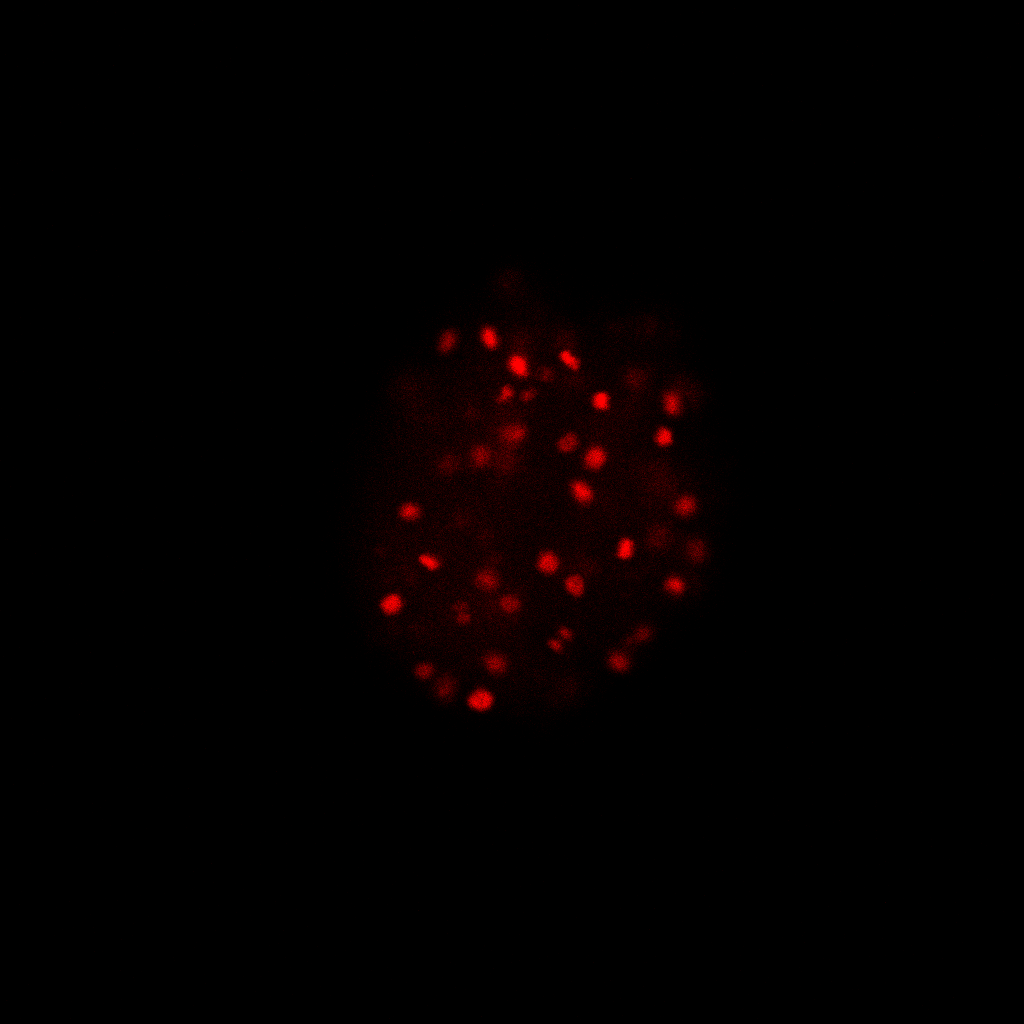

Supplement: Supplementary file 8 — Source data Fig. 6 [file 44318_2025_558_MOESM8_ESM.zip › Figure 6/panel 6C/KD-2_Ki67_3uM/seq11038_seq11038_RGB_TRITC.tif]

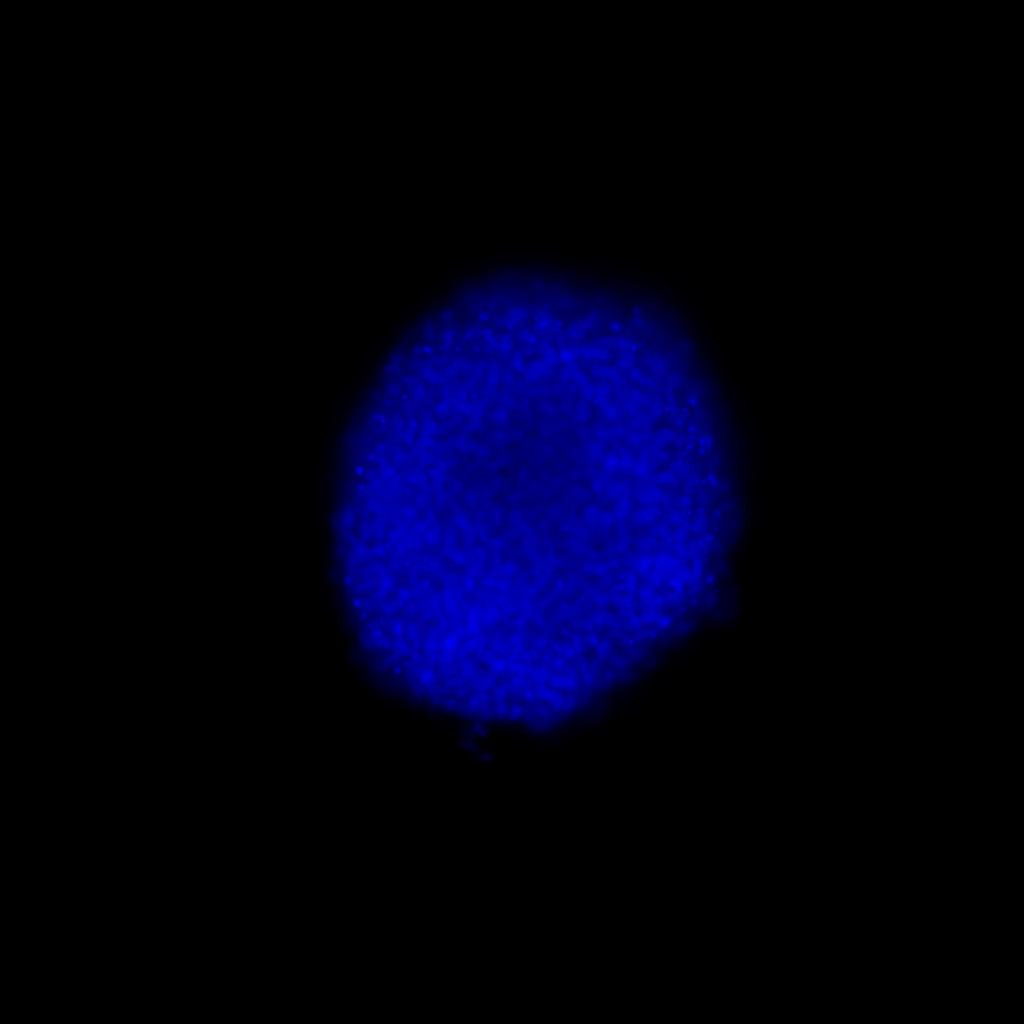

Supplement: Supplementary file 8 — Source data Fig. 6 [file 44318_2025_558_MOESM8_ESM.zip › Figure 6/panel 6C/KD-2_Ki67_3uM/seq11038_seq11038_RGB_DAPI.tif]

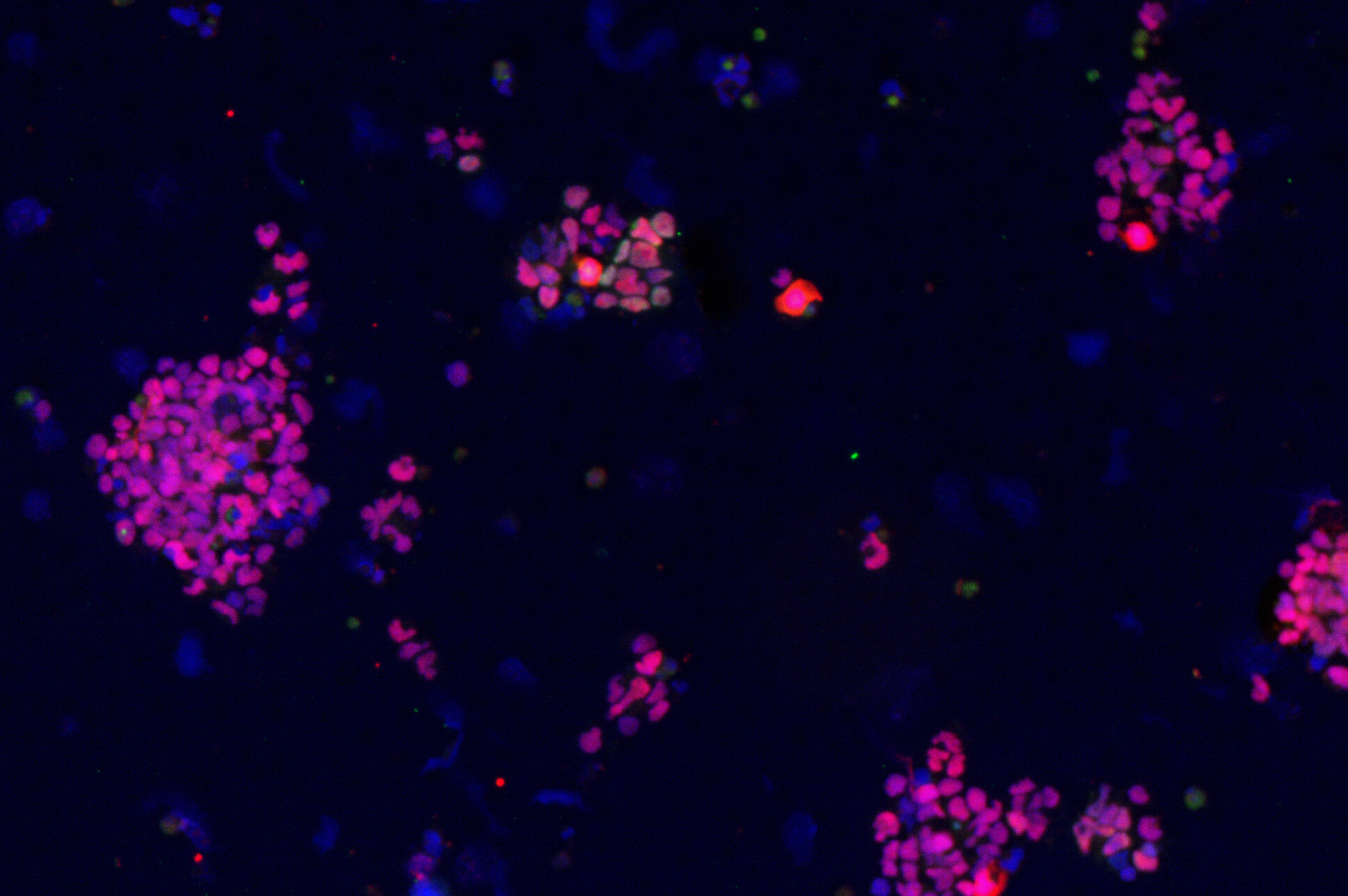

Supplement: Supplementary file 8 — Source data Fig. 6 [file 44318_2025_558_MOESM8_ESM.zip › Figure 6/panel 6D/KD-2/20X016.tif]

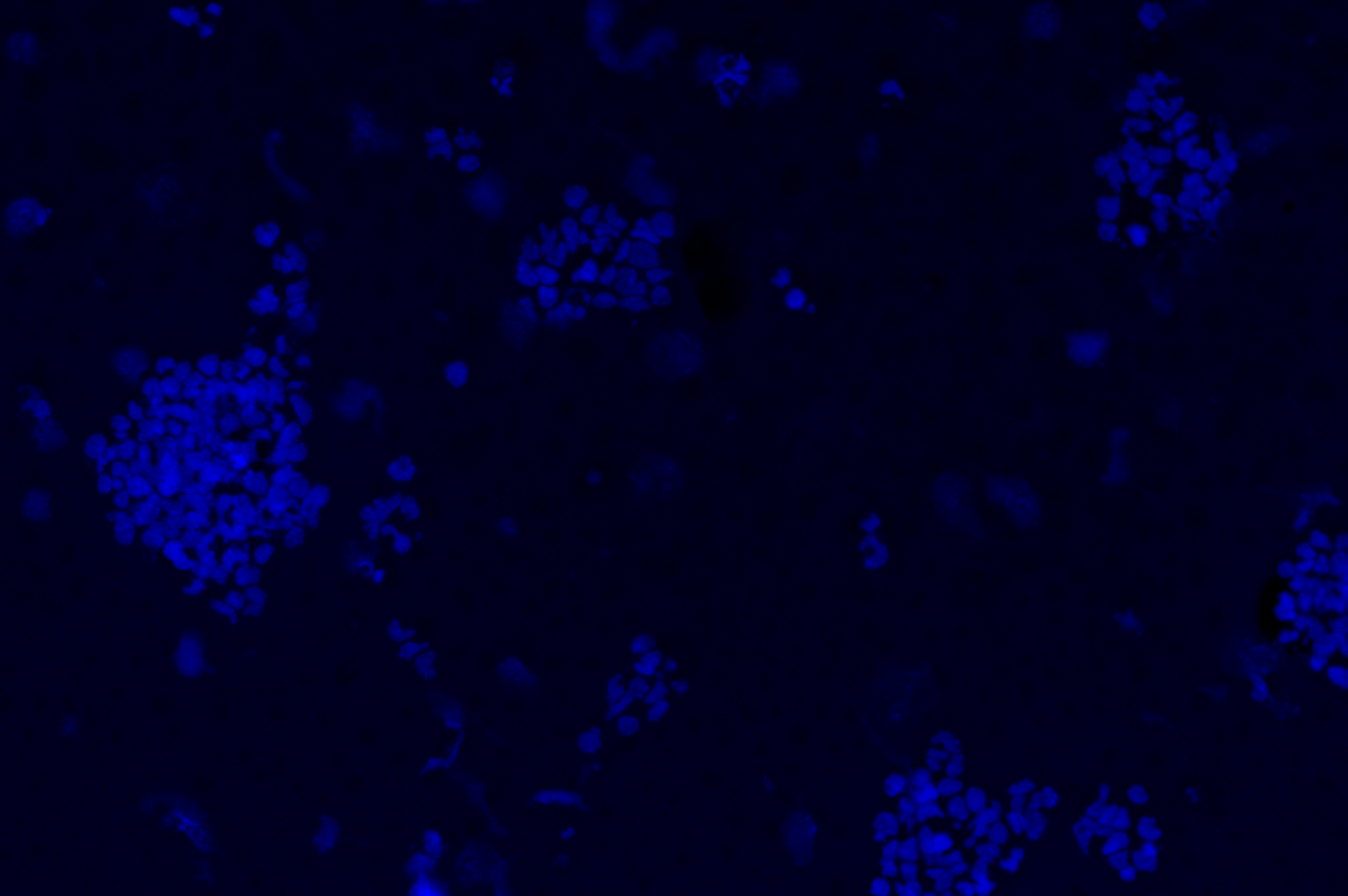

Supplement: Supplementary file 8 — Source data Fig. 6 [file 44318_2025_558_MOESM8_ESM.zip › Figure 6/panel 6D/KD-2/20X016c1.tif]

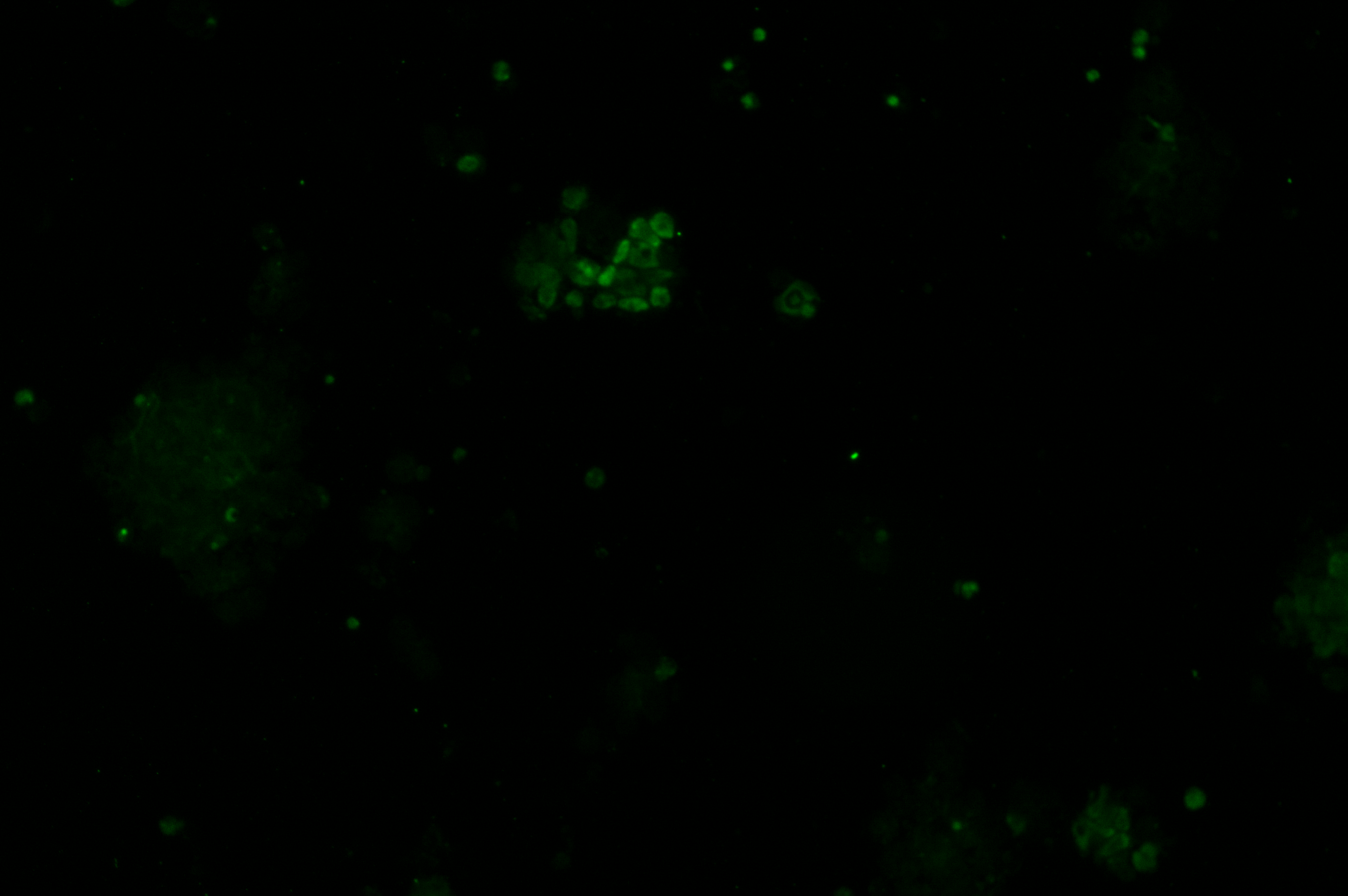

Supplement: Supplementary file 8 — Source data Fig. 6 [file 44318_2025_558_MOESM8_ESM.zip › Figure 6/panel 6D/KD-2/20X016c2.tif]

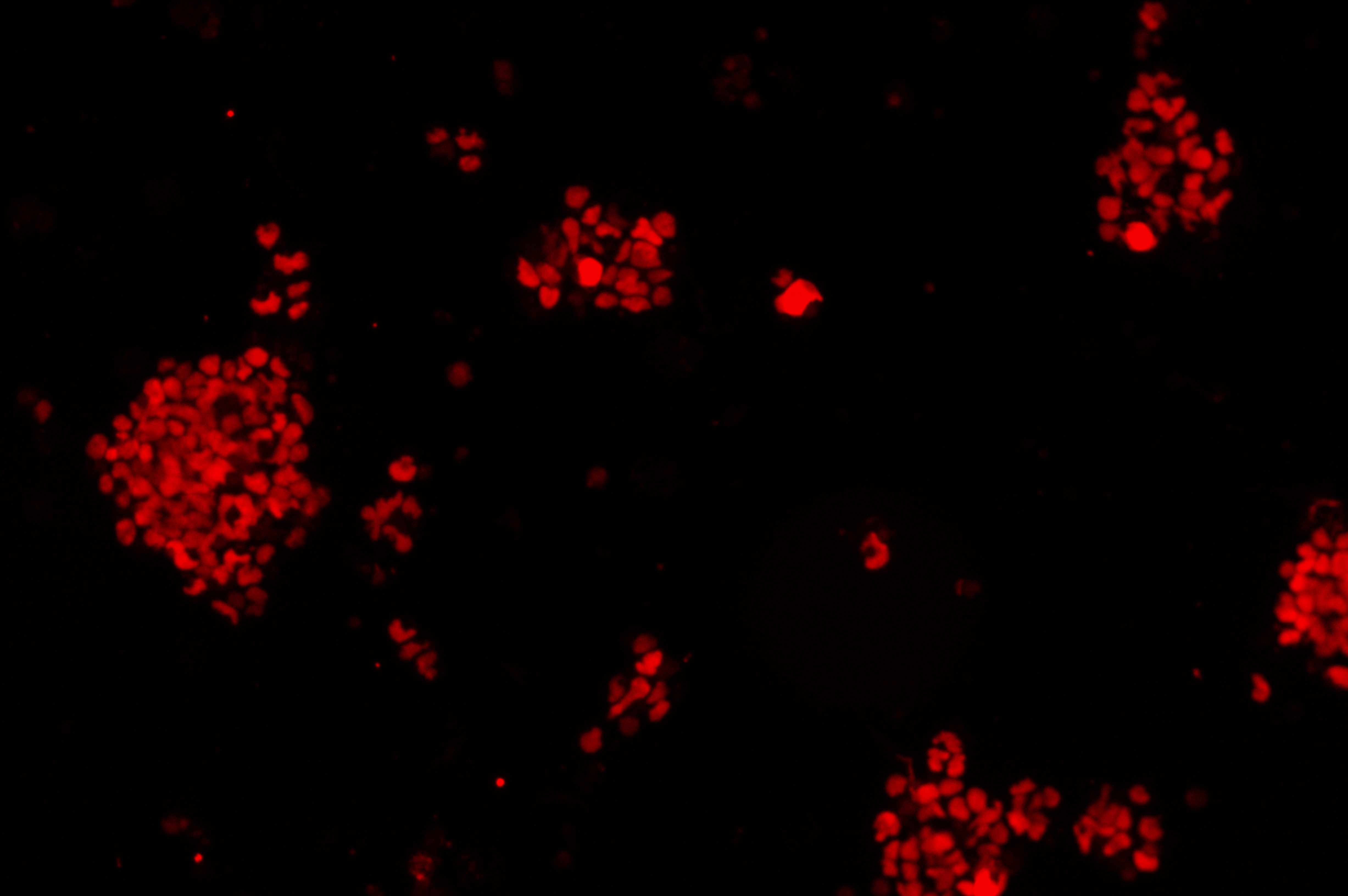

Supplement: Supplementary file 8 — Source data Fig. 6 [file 44318_2025_558_MOESM8_ESM.zip › Figure 6/panel 6D/KD-2/20X016c3.tif]

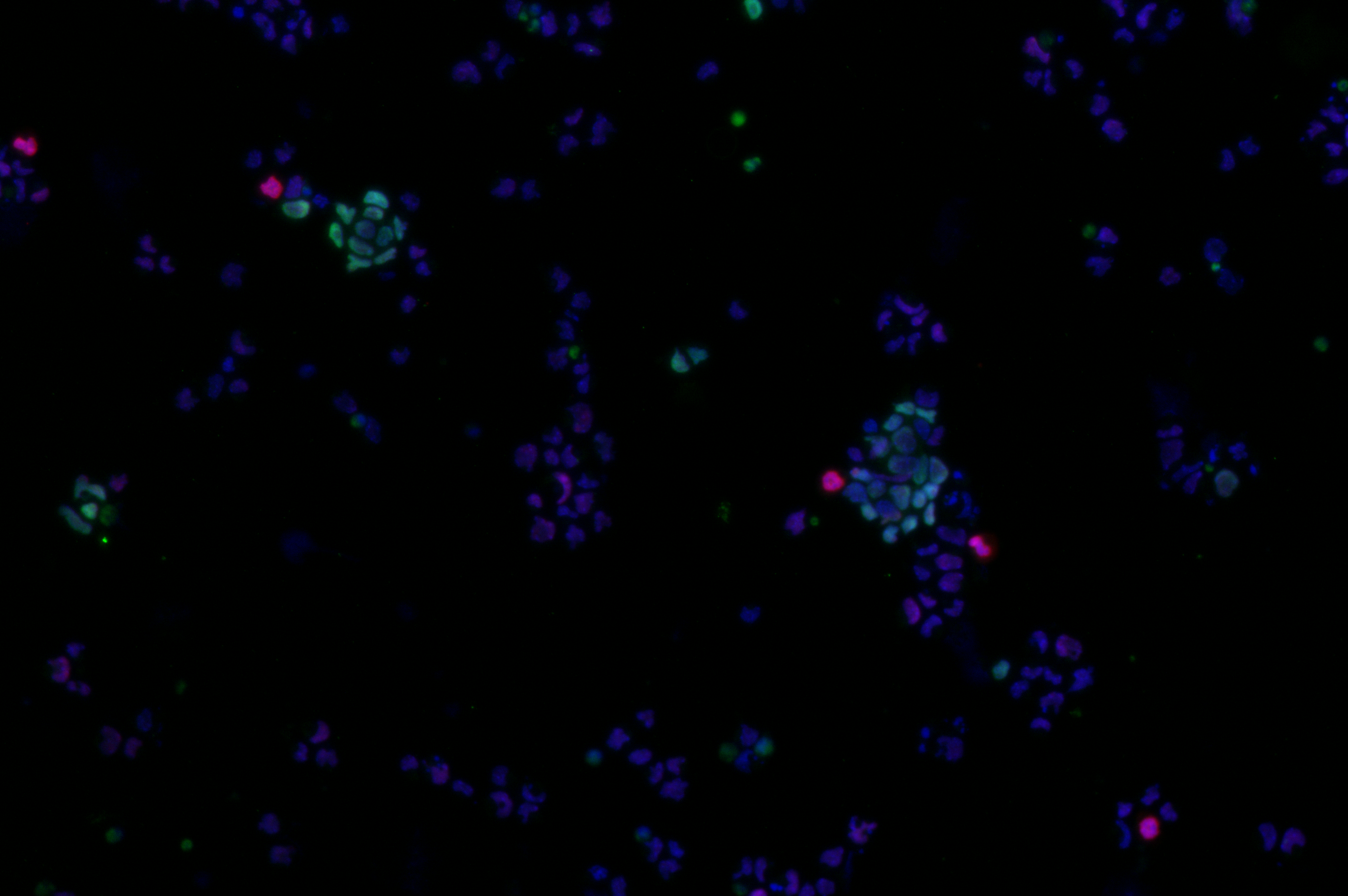

Supplement: Supplementary file 8 — Source data Fig. 6 [file 44318_2025_558_MOESM8_ESM.zip › Figure 6/panel 6D/NT/20X.tif]

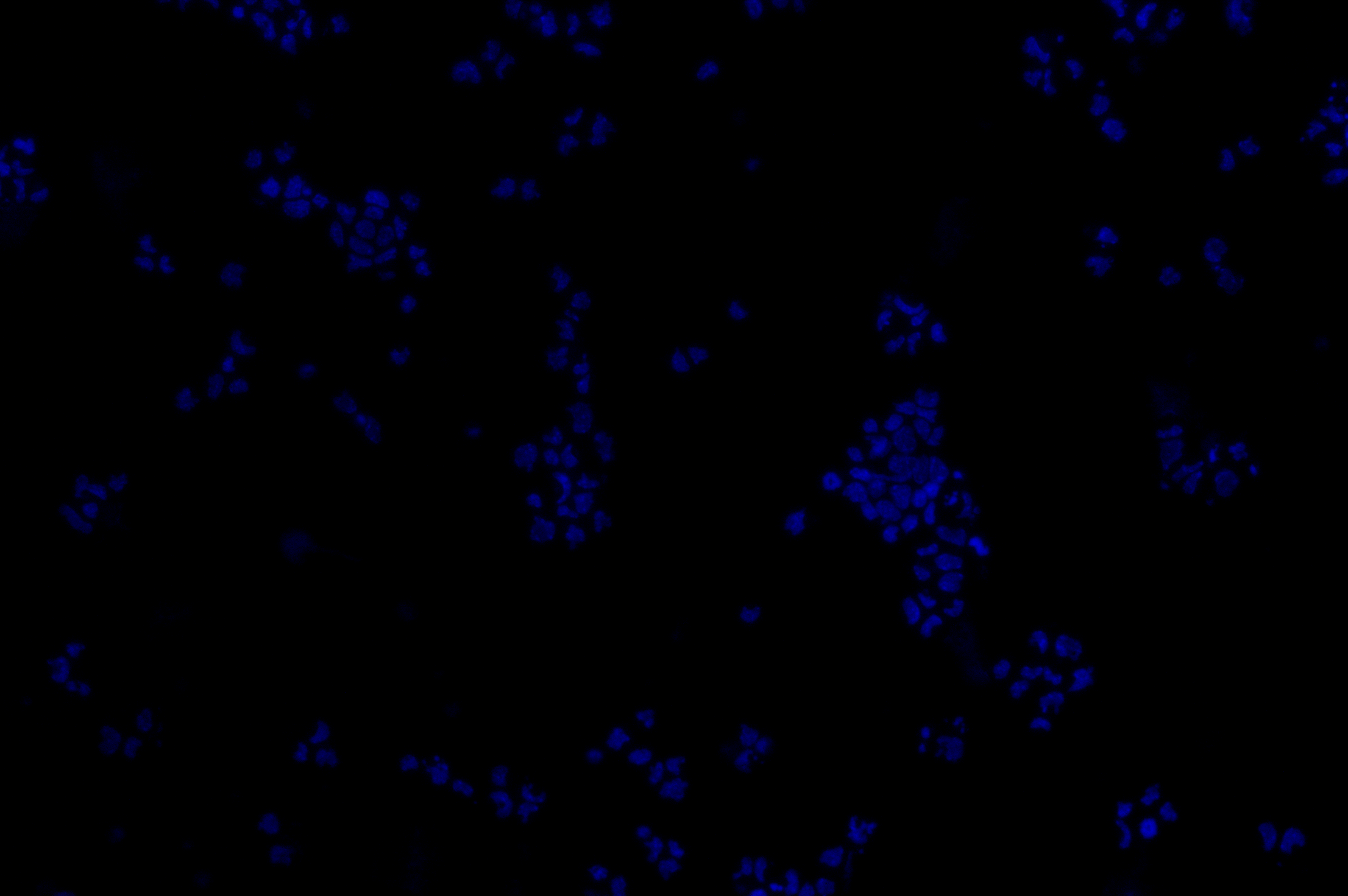

Supplement: Supplementary file 8 — Source data Fig. 6 [file 44318_2025_558_MOESM8_ESM.zip › Figure 6/panel 6D/NT/20Xc1.tif]

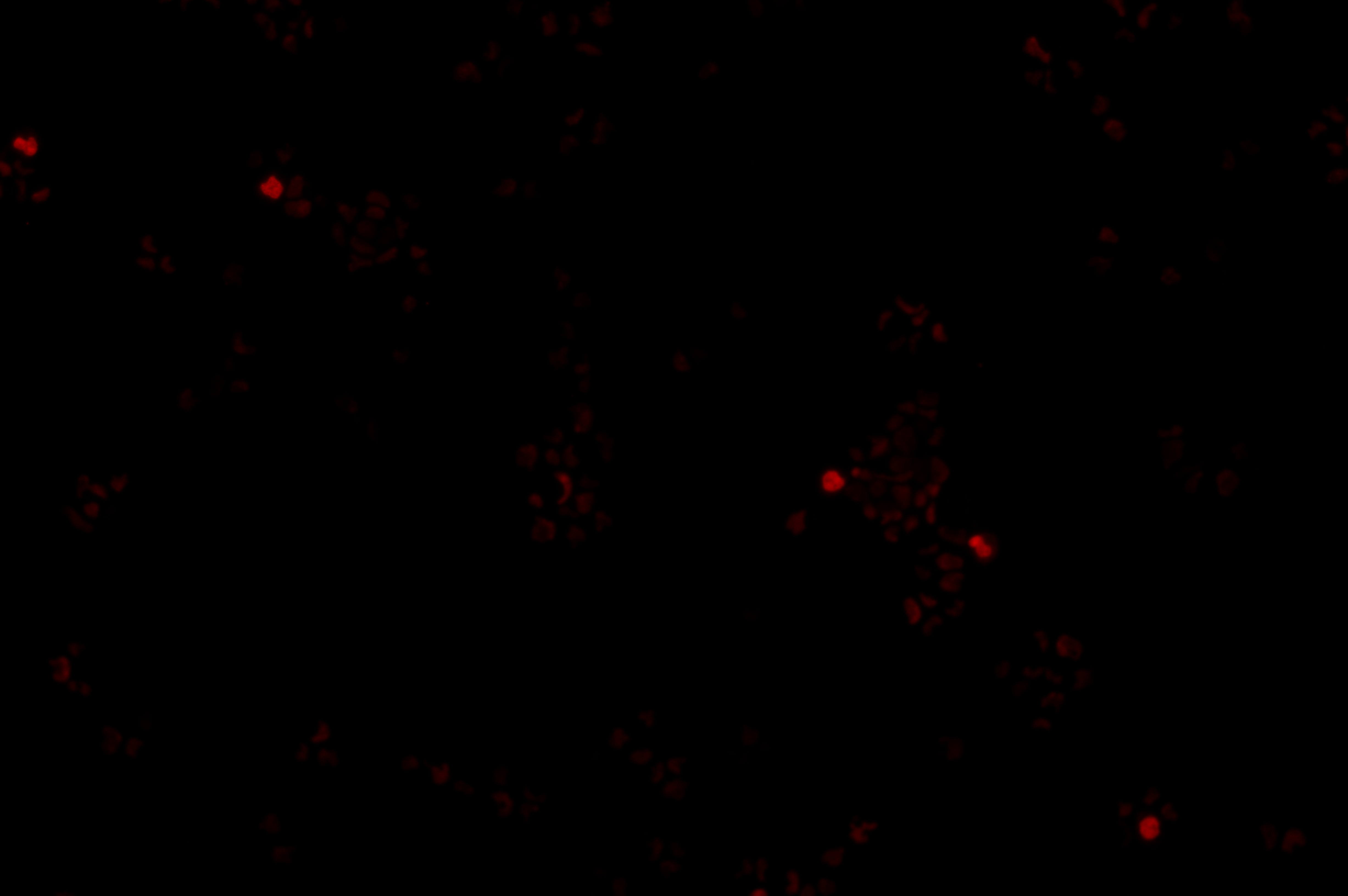

Supplement: Supplementary file 8 — Source data Fig. 6 [file 44318_2025_558_MOESM8_ESM.zip › Figure 6/panel 6D/NT/20Xc3.tif]

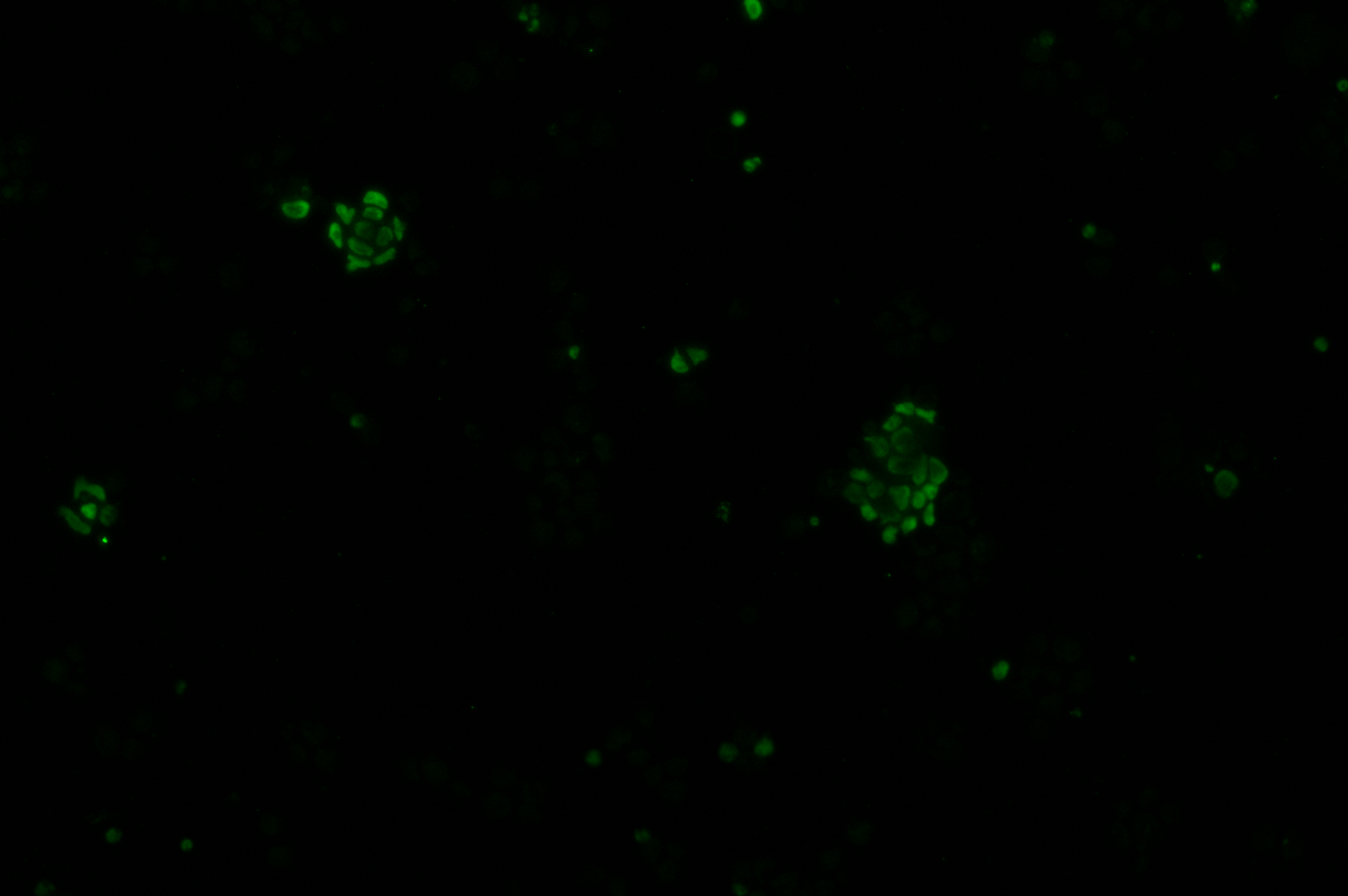

Supplement: Supplementary file 8 — Source data Fig. 6 [file 44318_2025_558_MOESM8_ESM.zip › Figure 6/panel 6D/NT/20Xc2.tif]

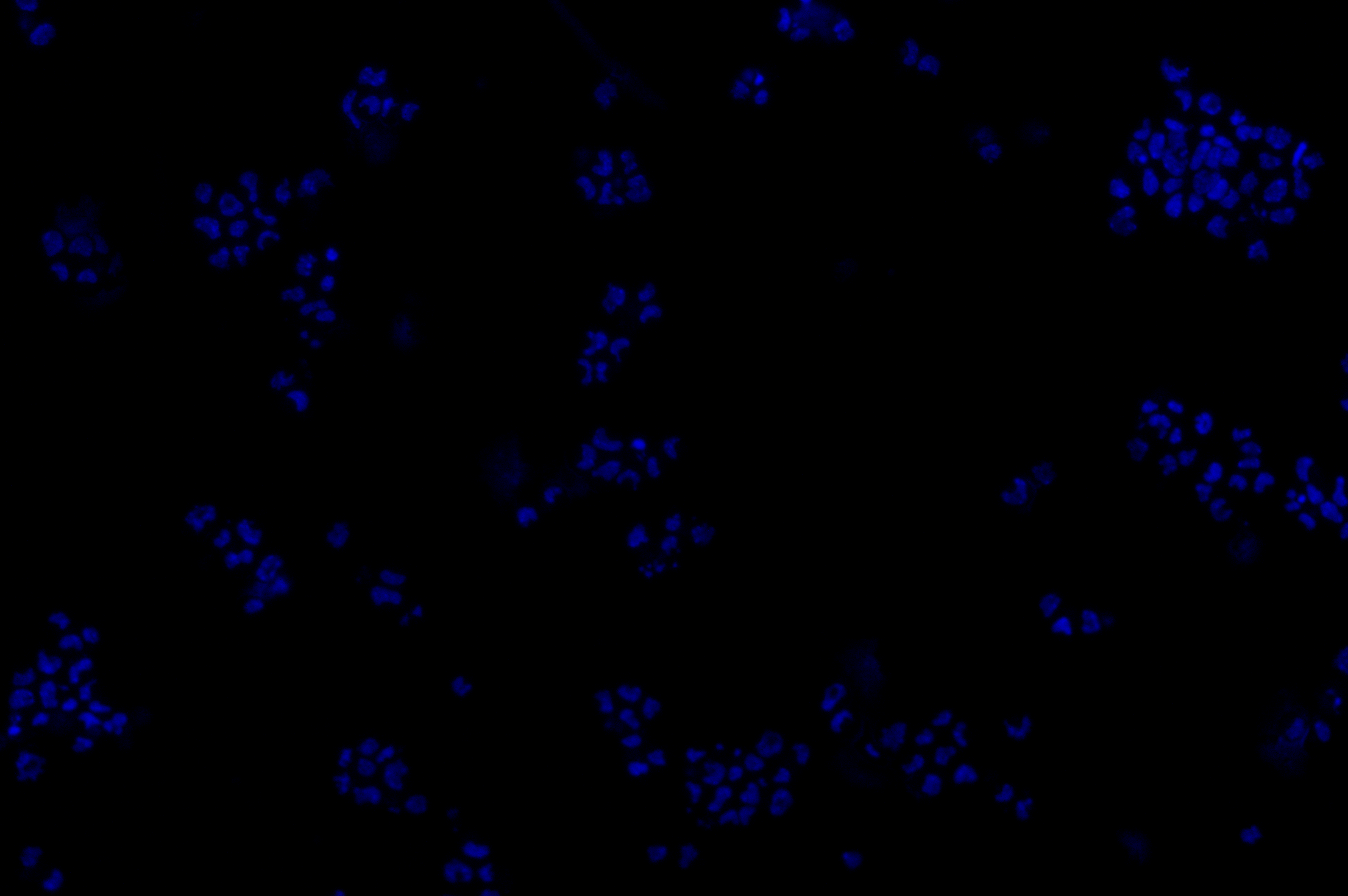

Supplement: Supplementary file 8 — Source data Fig. 6 [file 44318_2025_558_MOESM8_ESM.zip › Figure 6/panel 6D/KD-1/20X004c1.tif]

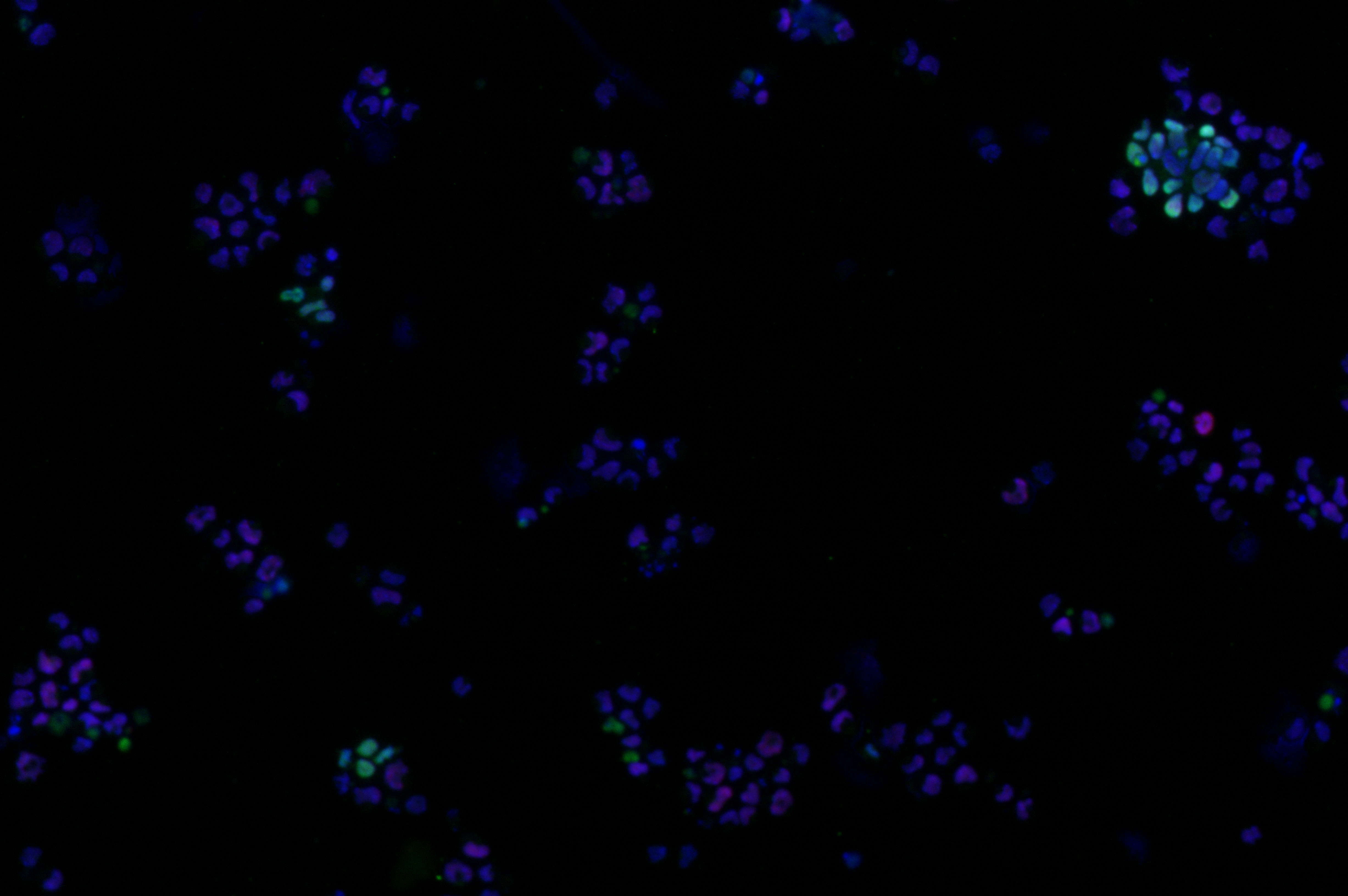

Supplement: Supplementary file 8 — Source data Fig. 6 [file 44318_2025_558_MOESM8_ESM.zip › Figure 6/panel 6D/KD-1/20X004.tif]

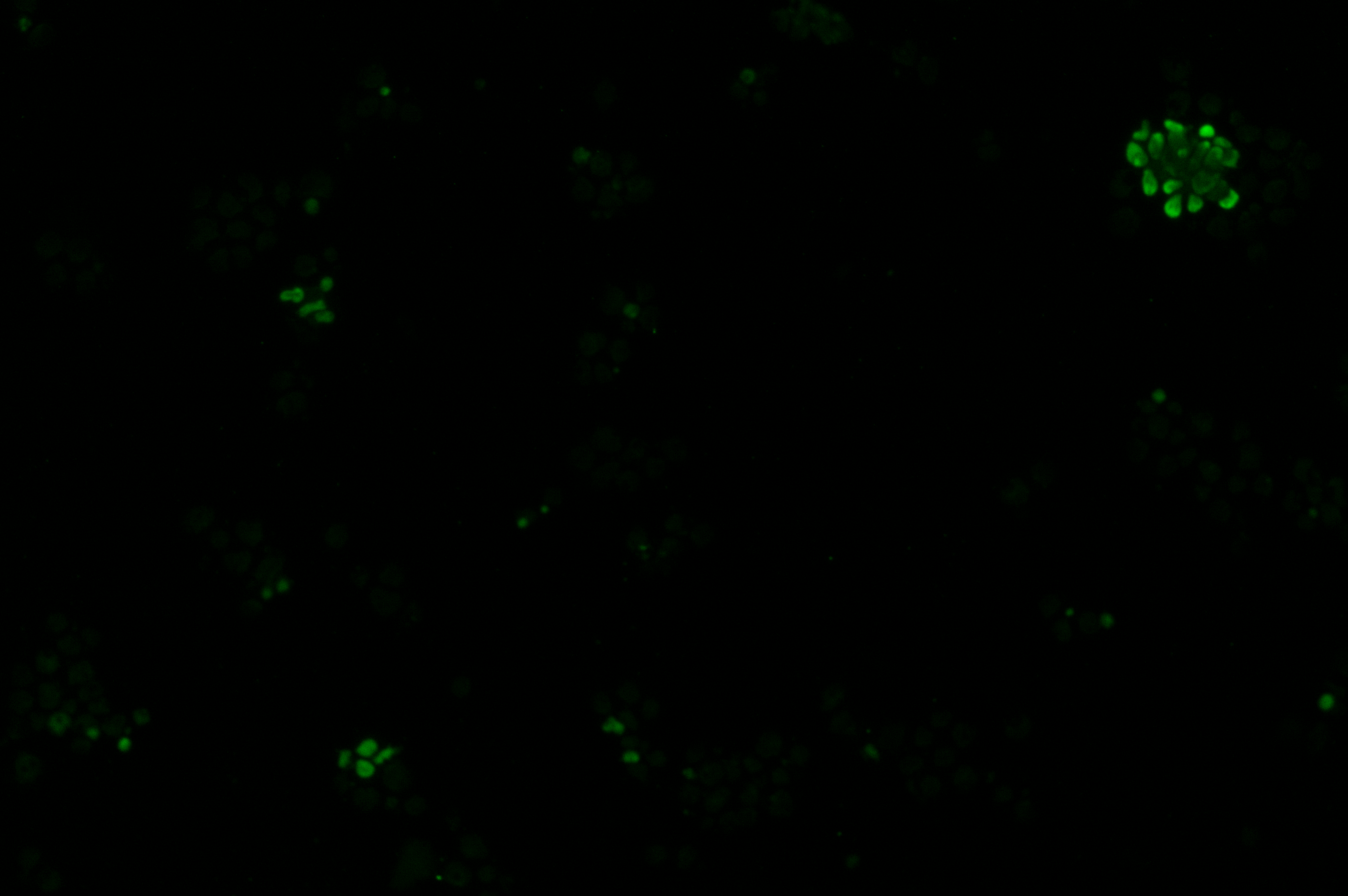

Supplement: Supplementary file 8 — Source data Fig. 6 [file 44318_2025_558_MOESM8_ESM.zip › Figure 6/panel 6D/KD-1/20X004c2.tif]

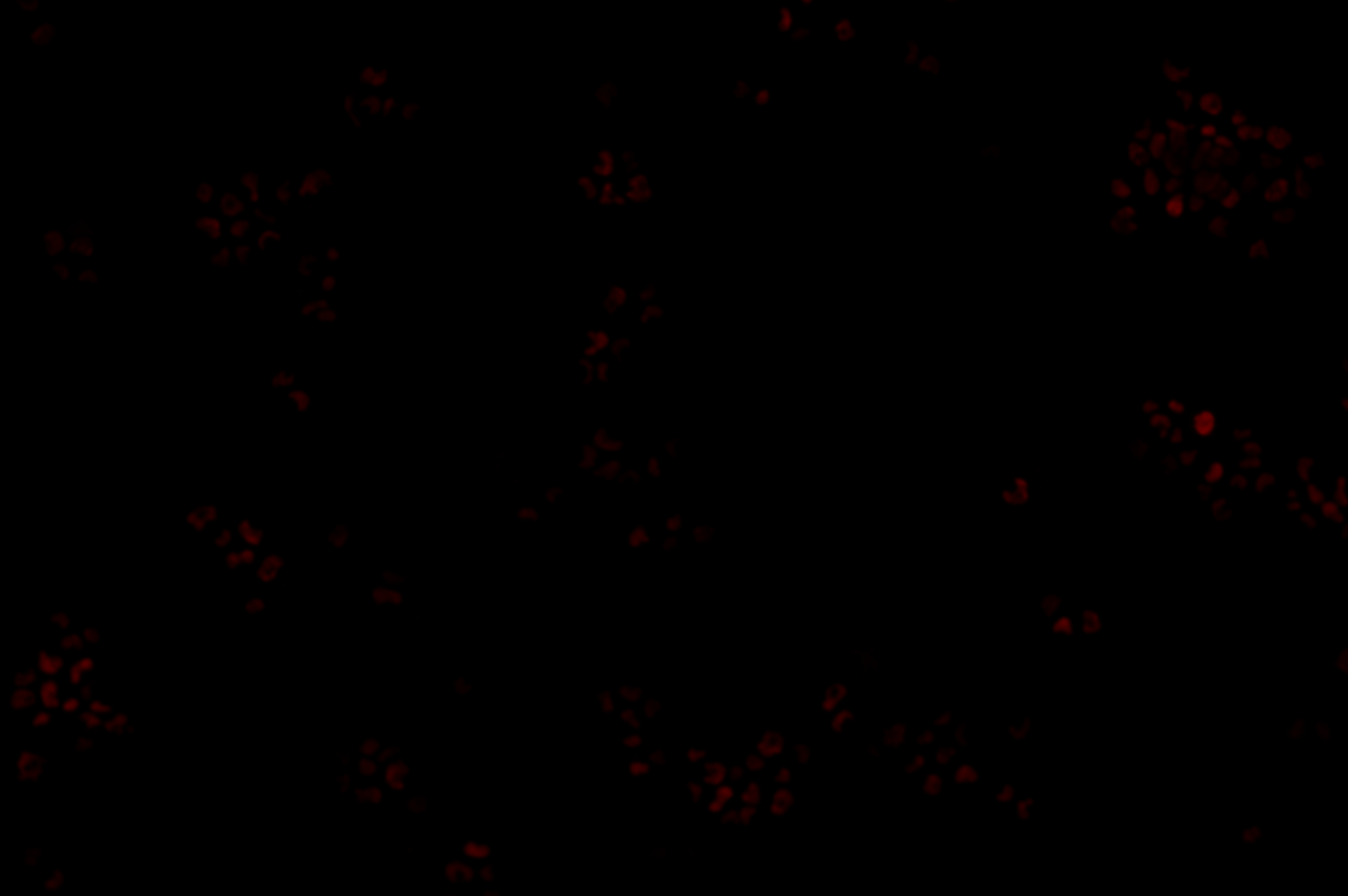

Supplement: Supplementary file 8 — Source data Fig. 6 [file 44318_2025_558_MOESM8_ESM.zip › Figure 6/panel 6D/KD-1/20X004c3.tif]

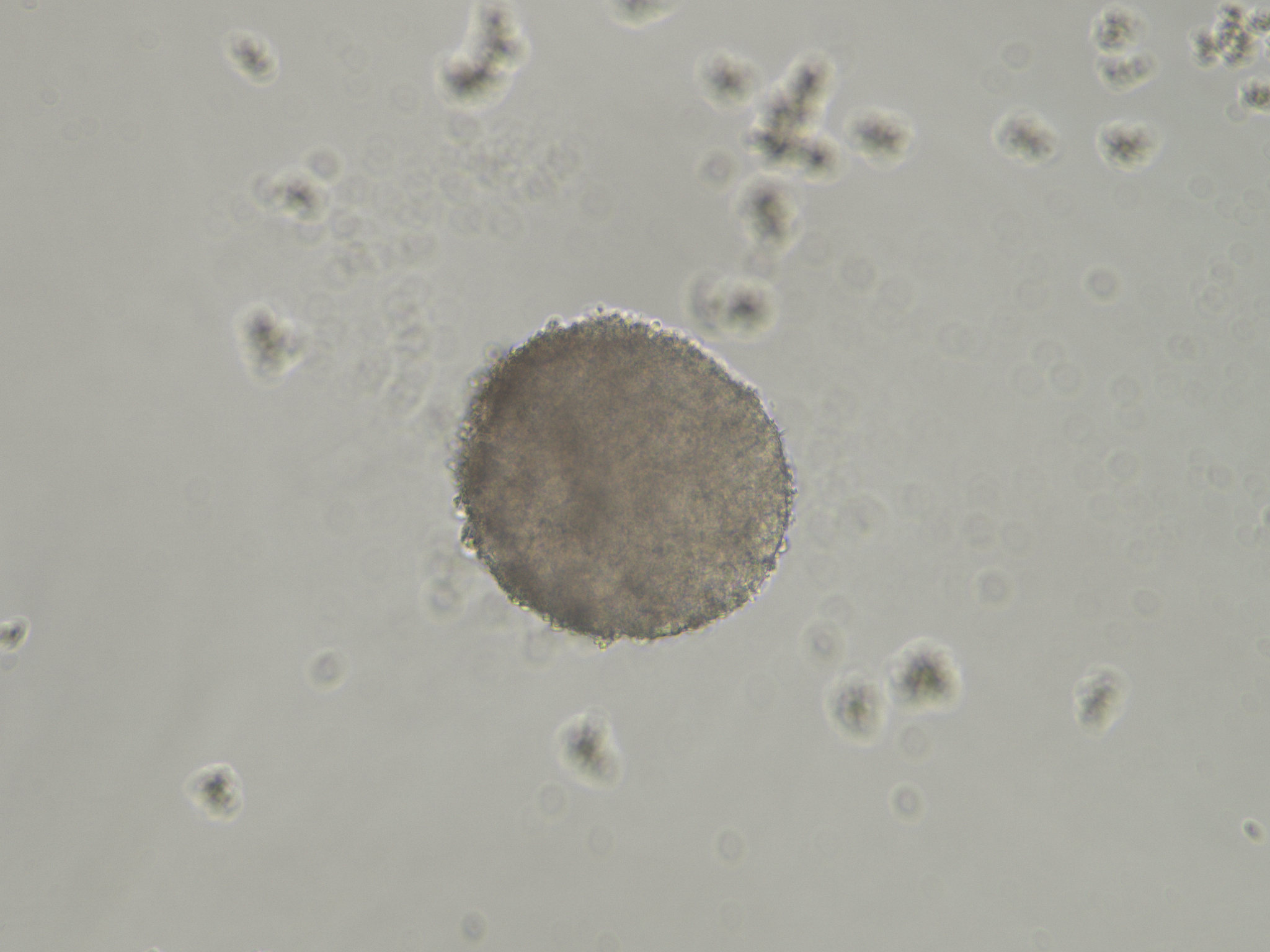

Supplement: Supplementary file 9 — Source data Fig. 7 [file 44318_2025_558_MOESM9_ESM.zip › Figure 7/panel 7B/KD1/KD1_3.tiff]

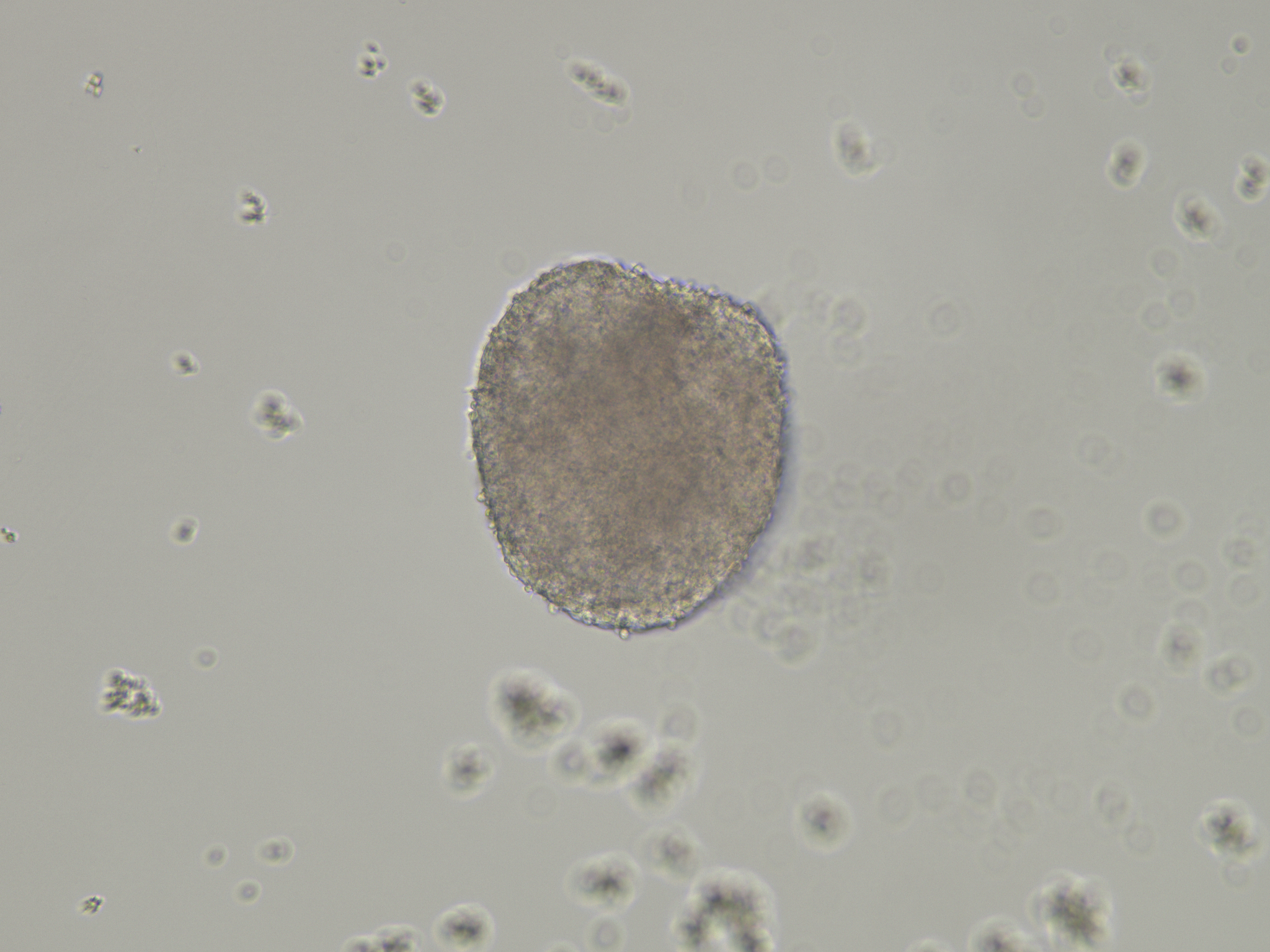

Supplement: Supplementary file 9 — Source data Fig. 7 [file 44318_2025_558_MOESM9_ESM.zip › Figure 7/panel 7B/KD1/KD1_1.tiff]

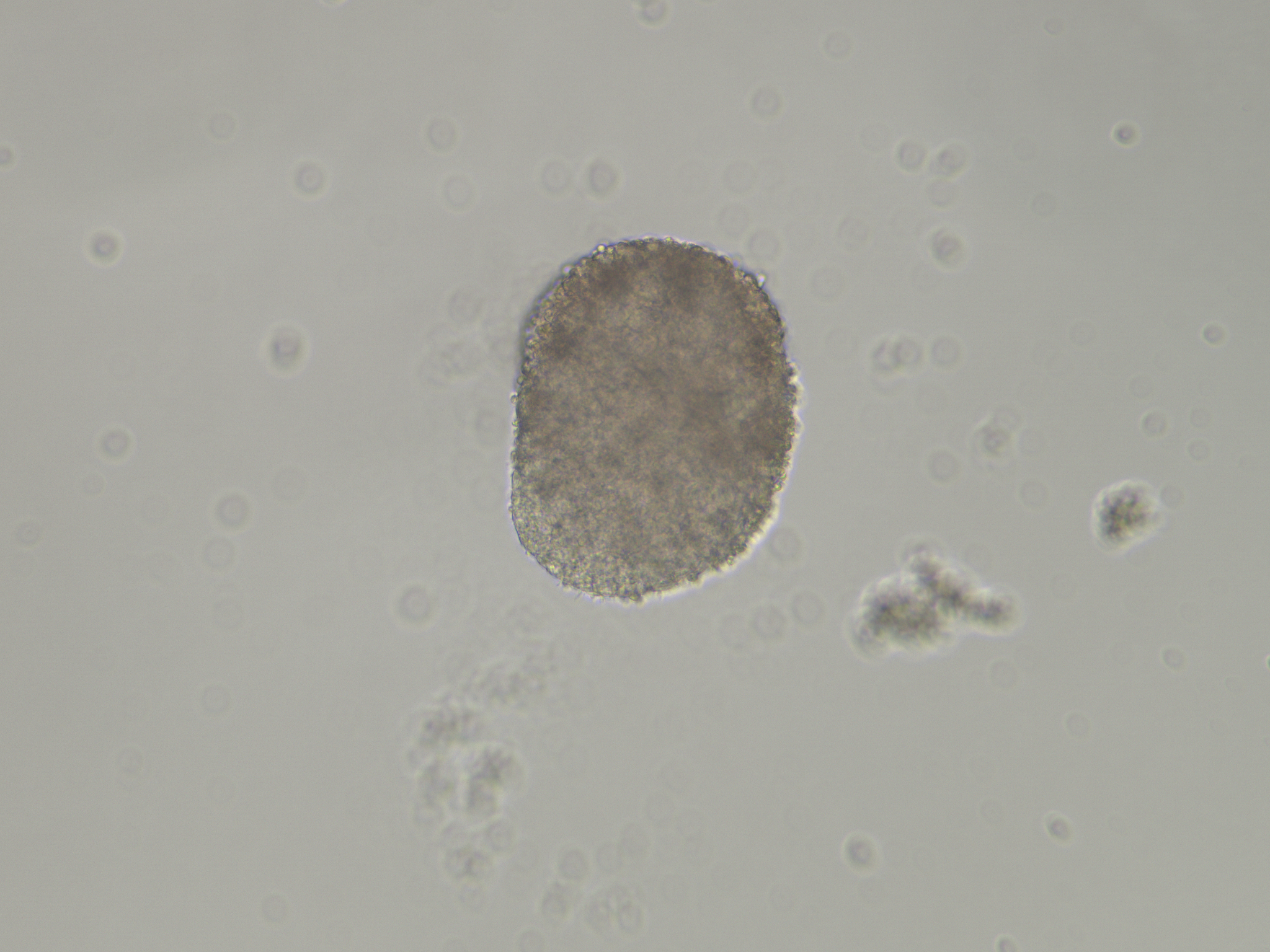

Supplement: Supplementary file 9 — Source data Fig. 7 [file 44318_2025_558_MOESM9_ESM.zip › Figure 7/panel 7B/KD1/KD1-2.tiff]

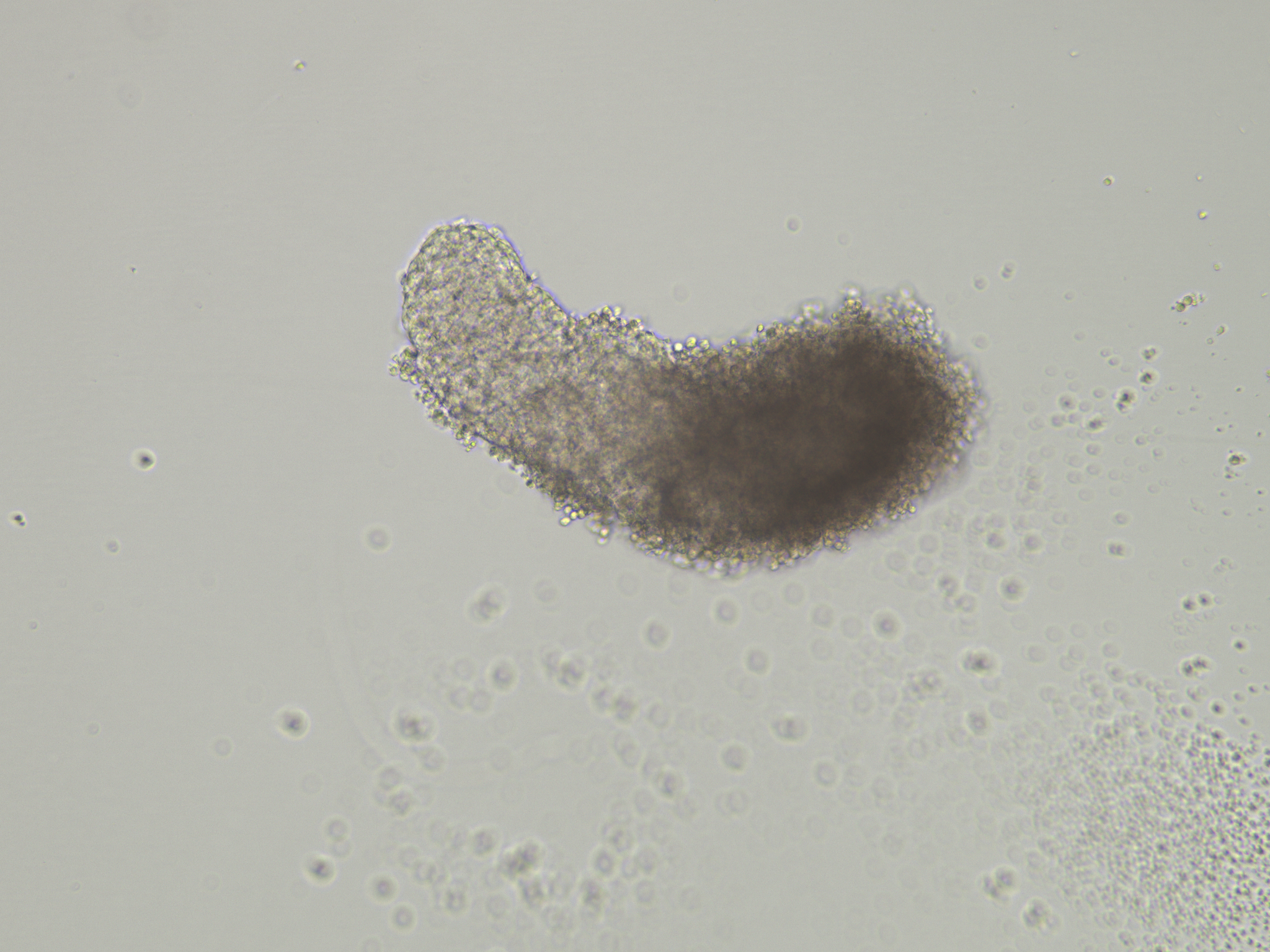

Supplement: Supplementary file 9 — Source data Fig. 7 [file 44318_2025_558_MOESM9_ESM.zip › Figure 7/panel 7B/NT/NT_3.tiff]

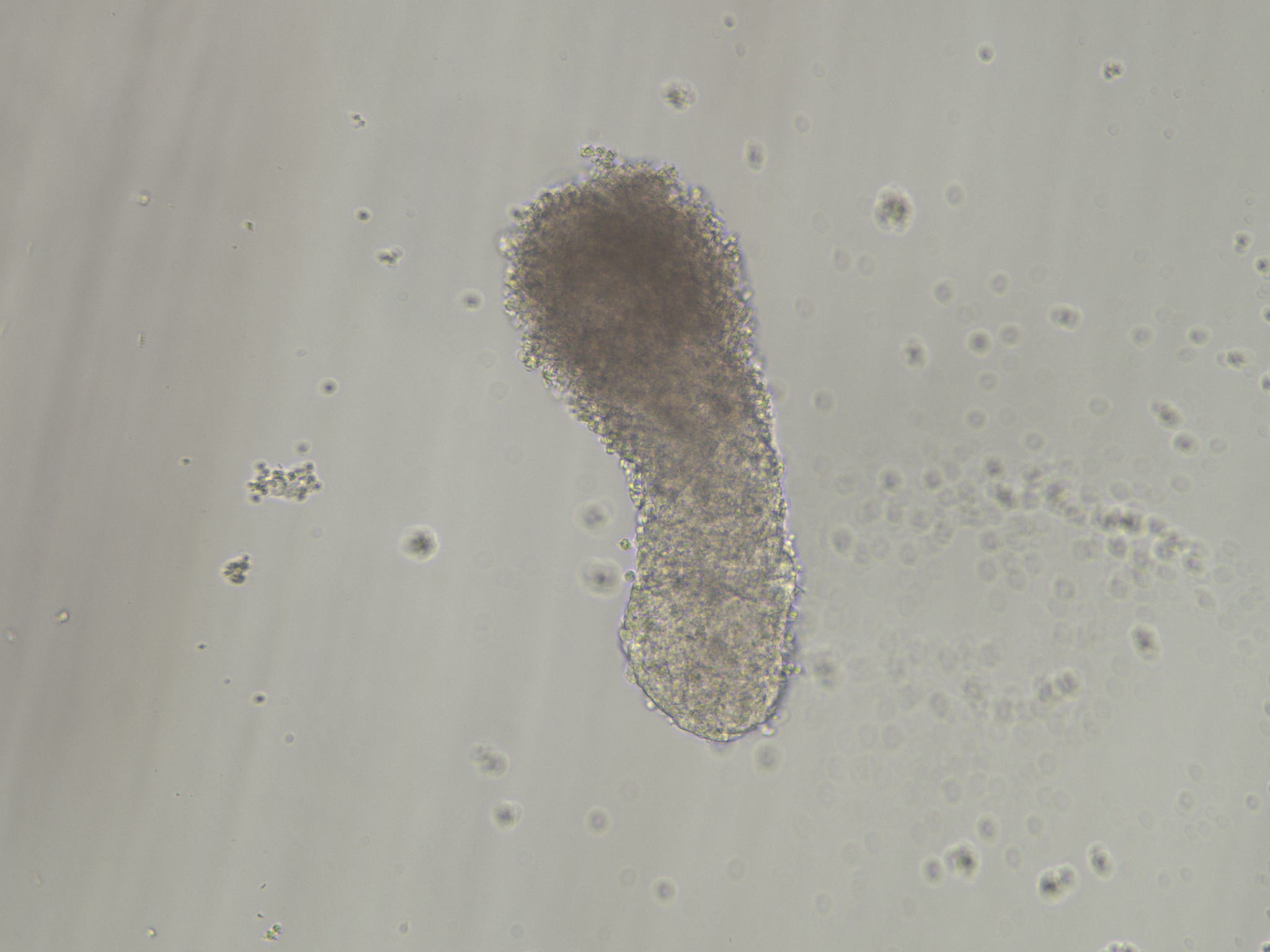

Supplement: Supplementary file 9 — Source data Fig. 7 [file 44318_2025_558_MOESM9_ESM.zip › Figure 7/panel 7B/NT/NT_2.tiff]

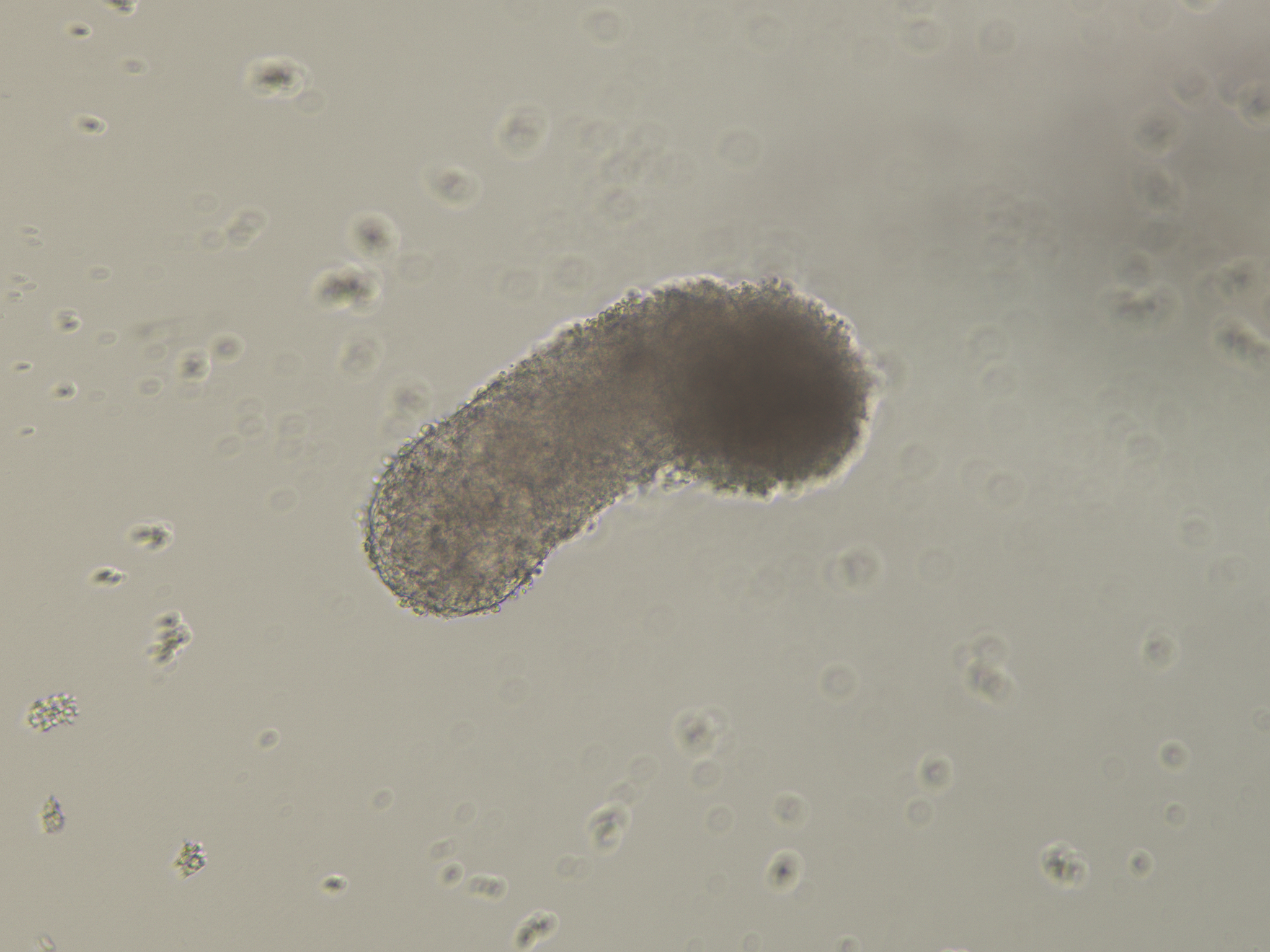

Supplement: Supplementary file 9 — Source data Fig. 7 [file 44318_2025_558_MOESM9_ESM.zip › Figure 7/panel 7B/NT/NT_1.tiff]

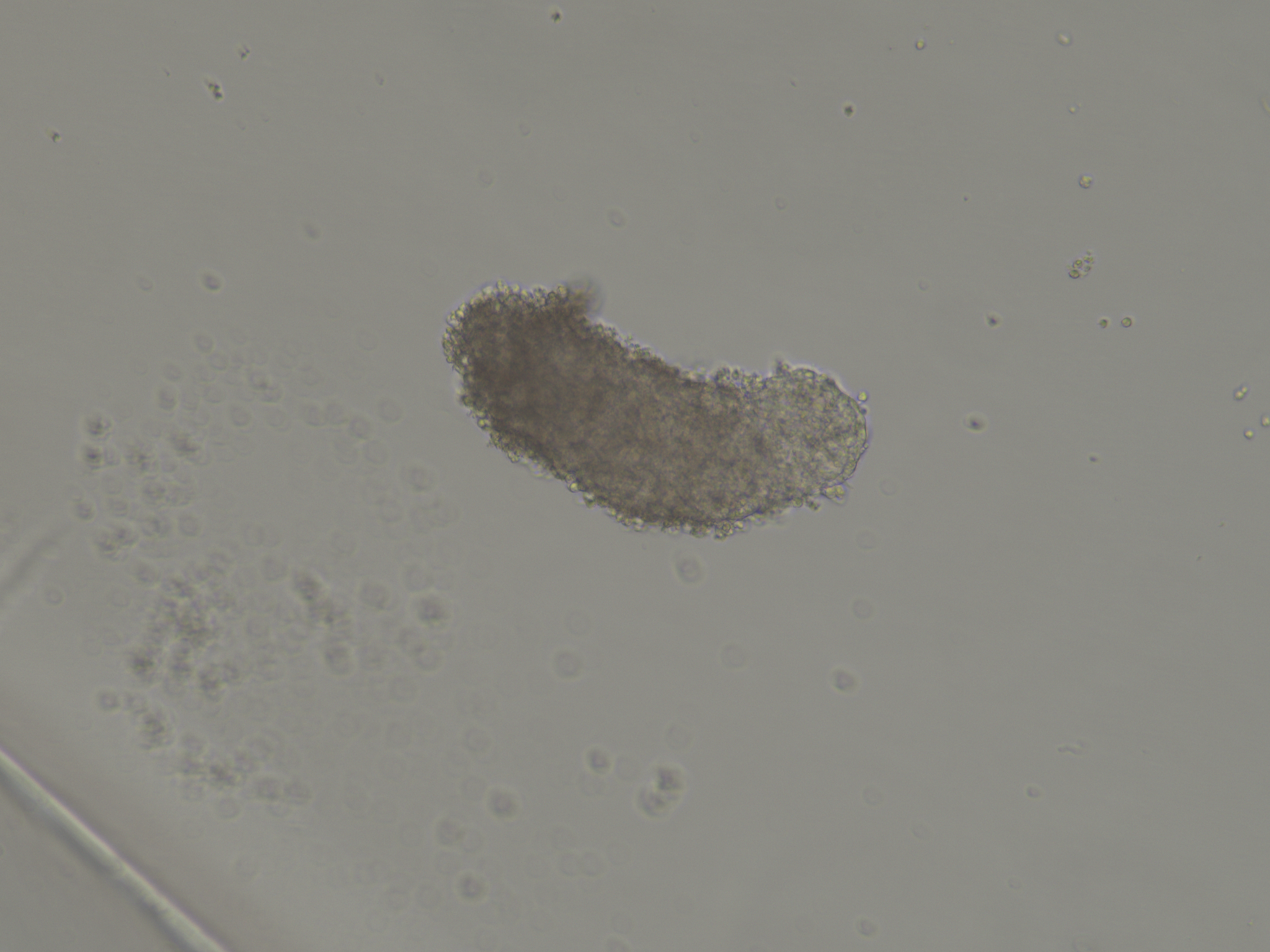

Supplement: Supplementary file 9 — Source data Fig. 7 [file 44318_2025_558_MOESM9_ESM.zip › Figure 7/panel 7C/NT/NT_3.tiff]

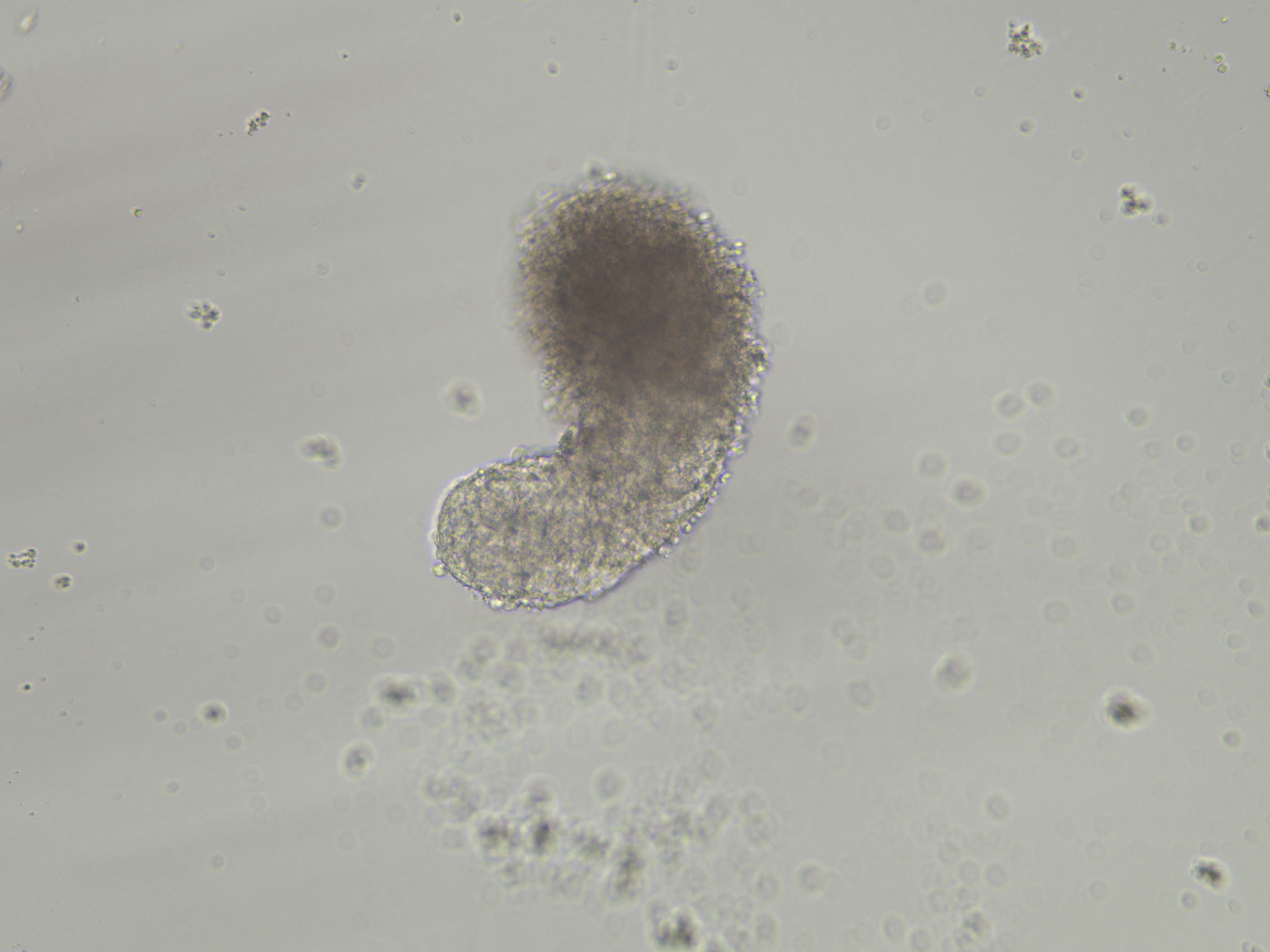

Supplement: Supplementary file 9 — Source data Fig. 7 [file 44318_2025_558_MOESM9_ESM.zip › Figure 7/panel 7C/NT/NT_2.tiff]

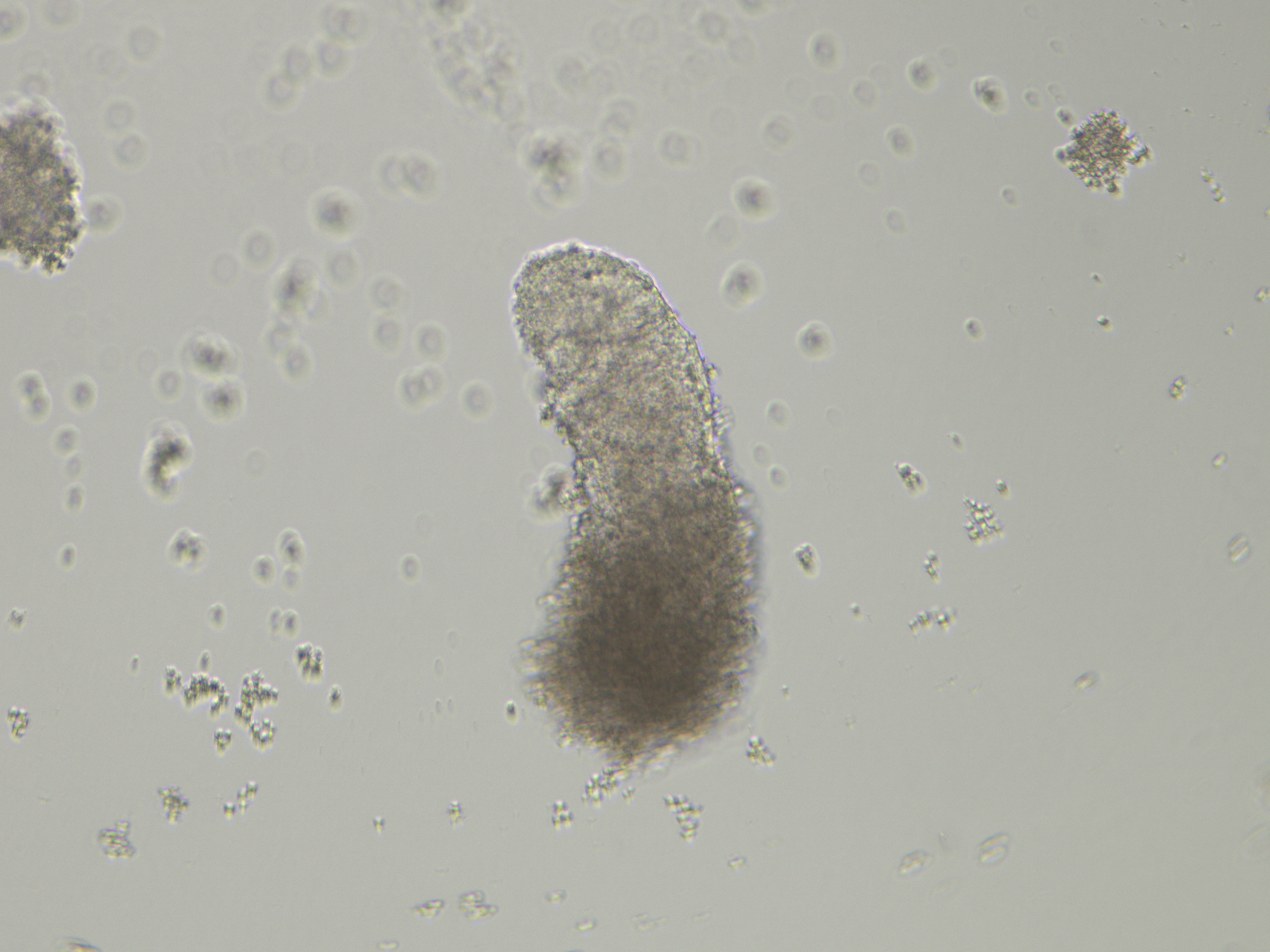

Supplement: Supplementary file 9 — Source data Fig. 7 [file 44318_2025_558_MOESM9_ESM.zip › Figure 7/panel 7C/NT/NT_1.tiff]

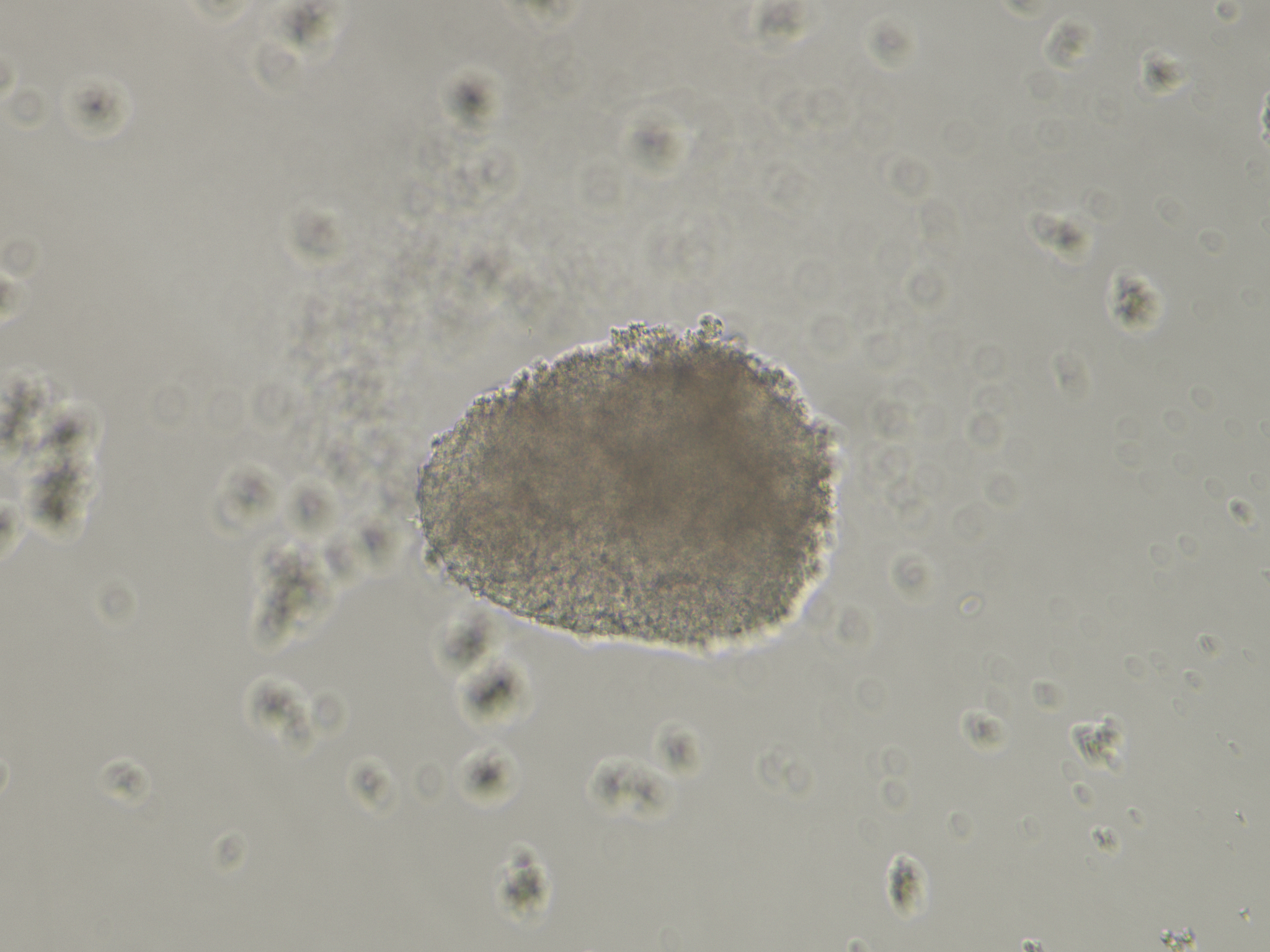

Supplement: Supplementary file 9 — Source data Fig. 7 [file 44318_2025_558_MOESM9_ESM.zip › Figure 7/panel 7C/KD2/KD2_1.tiff]
