# Supplementary figures and images for: Non-cell-autonomous control of mouse gastruloid development by the ultra-conserved lncRNA T-UCstem1 (part 5 of 6)
Source: EMBO J. 2025 Oct 31;44(24):7620–48. doi: 10.1038/s44318-025-00558-2 (PMC12706062; doi:10.1038/s44318-025-00558-2)

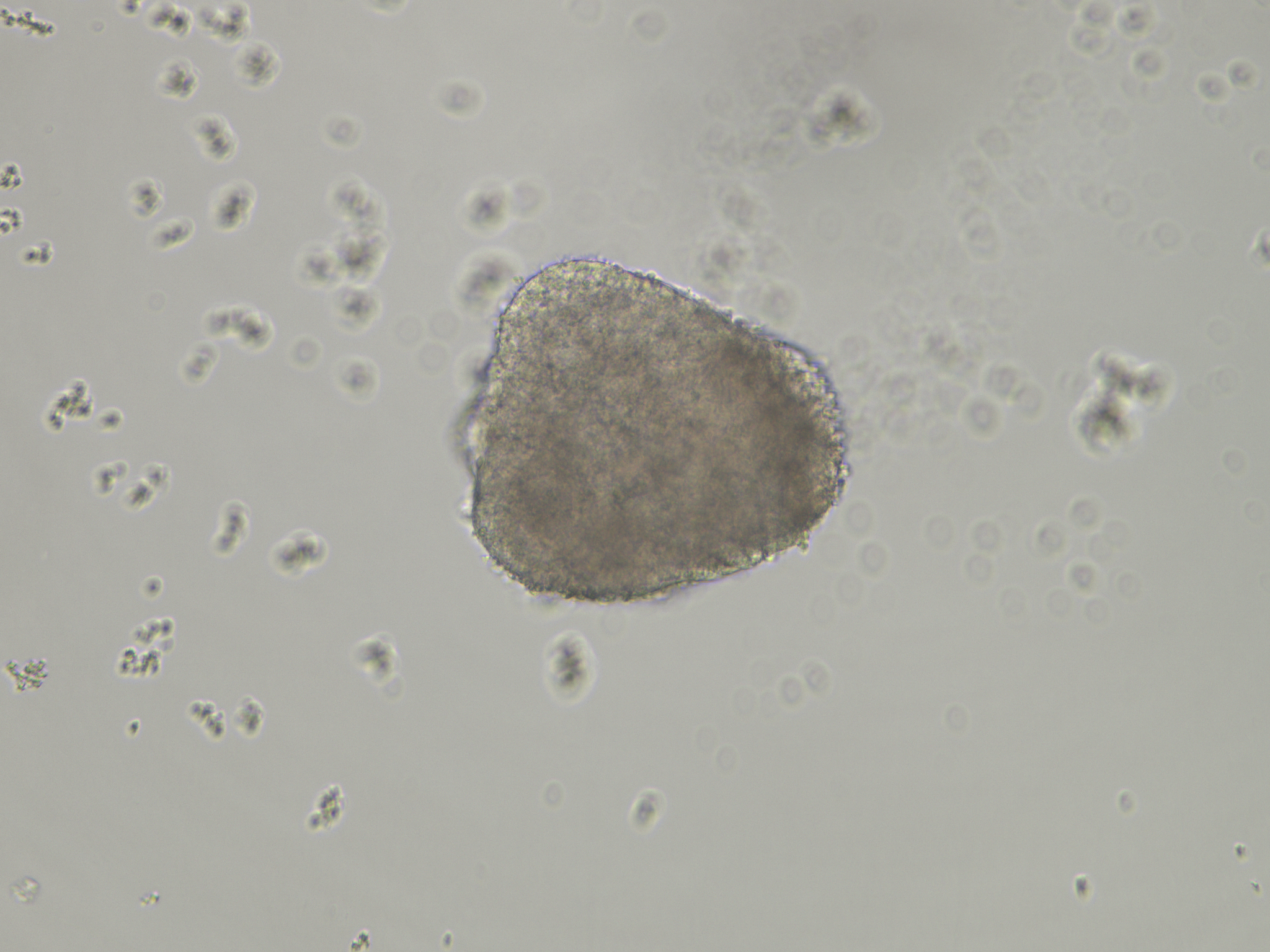

Supplement: Supplementary file 9 — Source data Fig. 7 [file 44318_2025_558_MOESM9_ESM.zip › Figure 7/panel 7C/KD2/KD2_3.tiff]

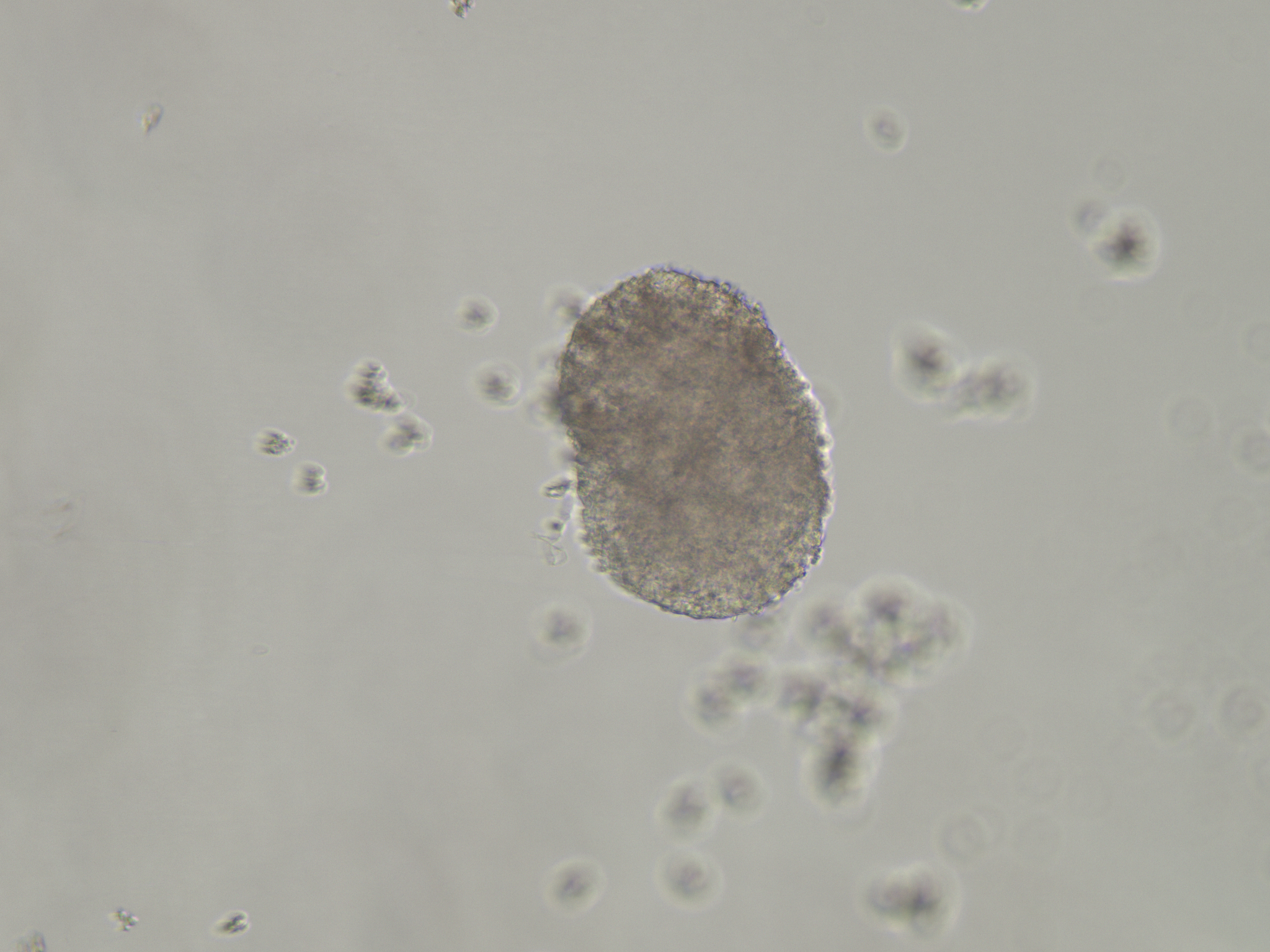

Supplement: Supplementary file 9 — Source data Fig. 7 [file 44318_2025_558_MOESM9_ESM.zip › Figure 7/panel 7C/KD2/KD2_2.tiff]

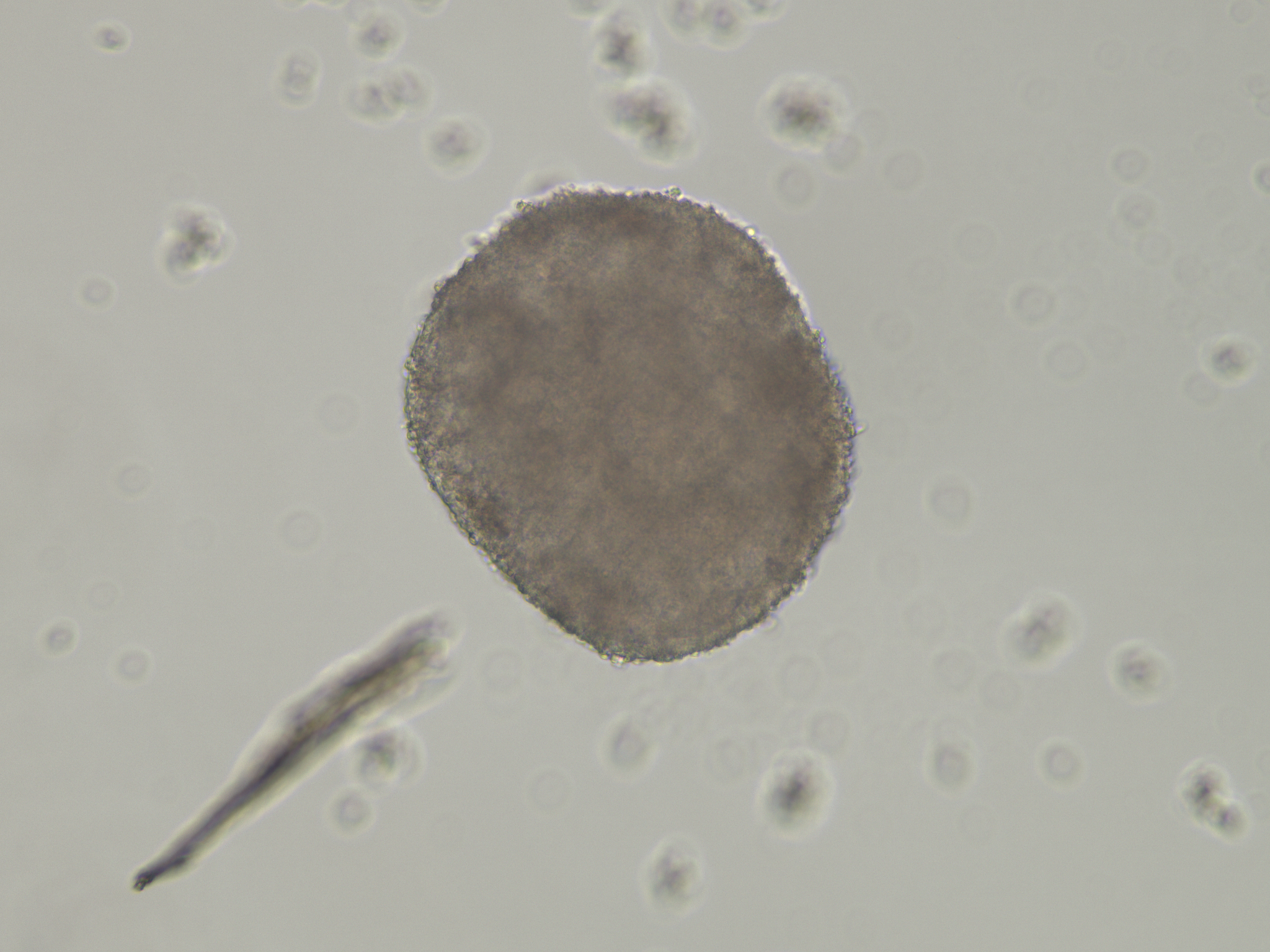

Supplement: Supplementary file 9 — Source data Fig. 7 [file 44318_2025_558_MOESM9_ESM.zip › Figure 7/panel 7B/NT-KD1/Cond 2:3/2:3_1.tiff]

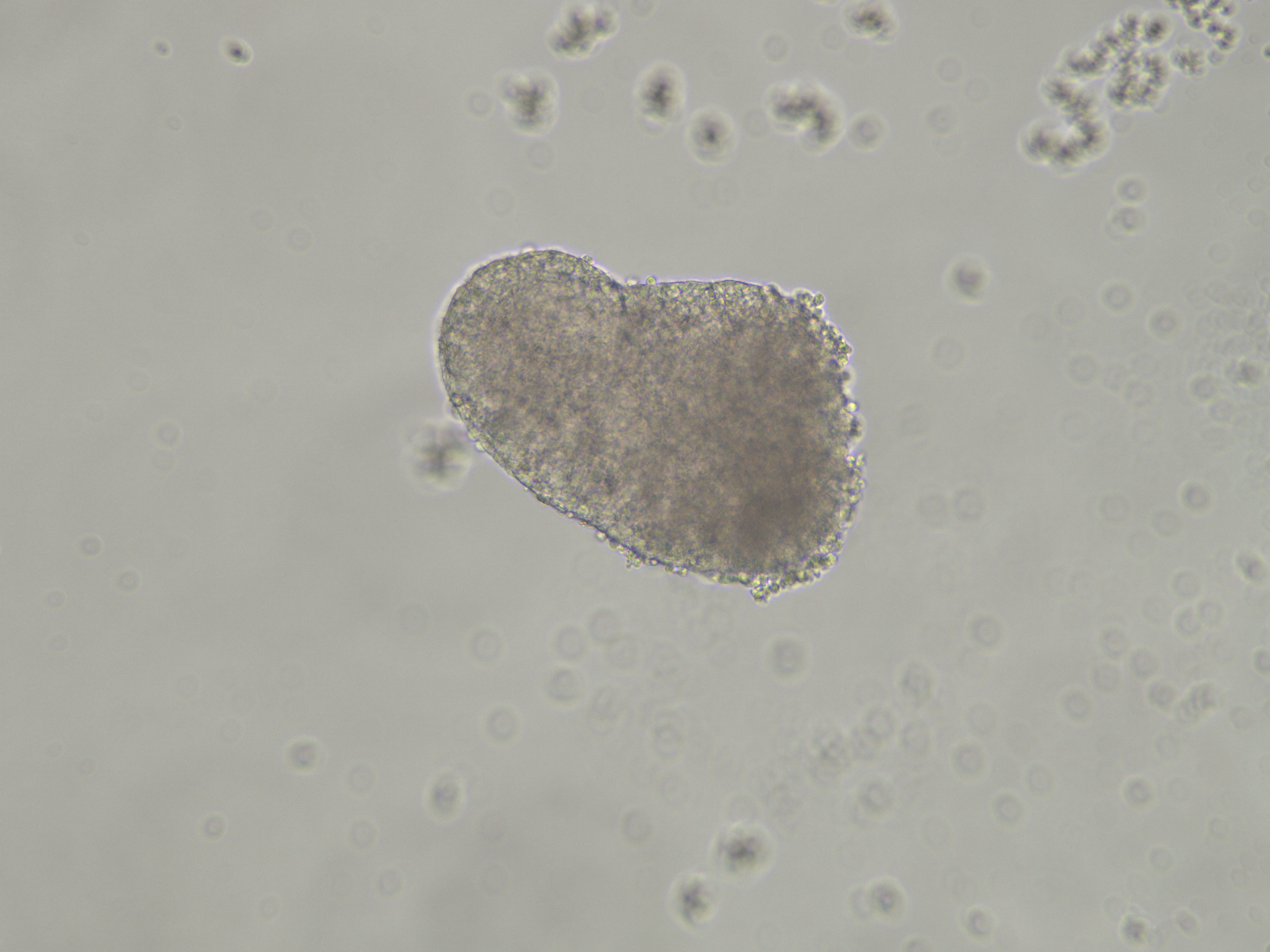

Supplement: Supplementary file 9 — Source data Fig. 7 [file 44318_2025_558_MOESM9_ESM.zip › Figure 7/panel 7B/NT-KD1/Cond 2:3/2:3_2.tiff]

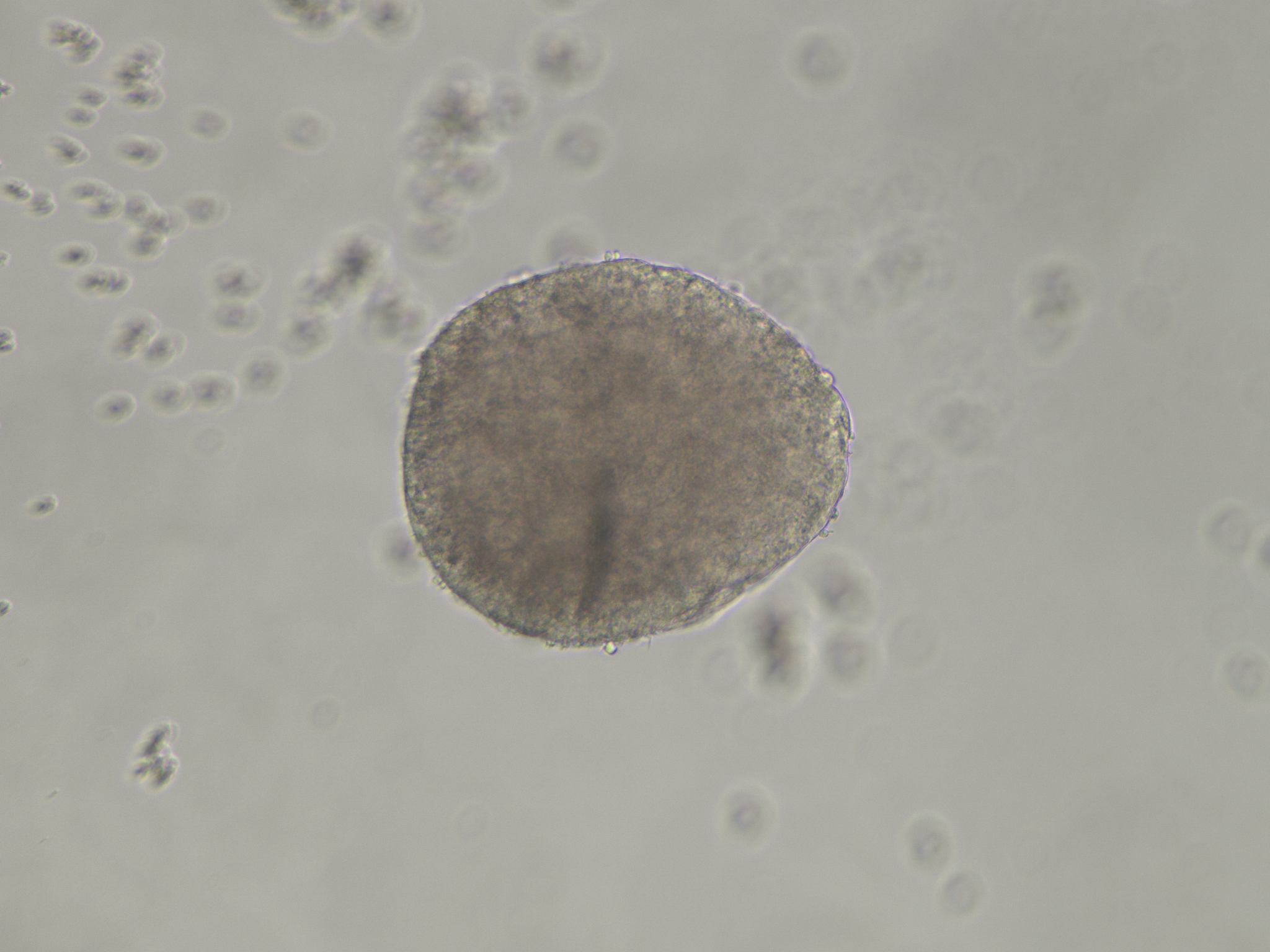

Supplement: Supplementary file 9 — Source data Fig. 7 [file 44318_2025_558_MOESM9_ESM.zip › Figure 7/panel 7B/NT-KD1/Cond 2:3/2:3_3.tiff]

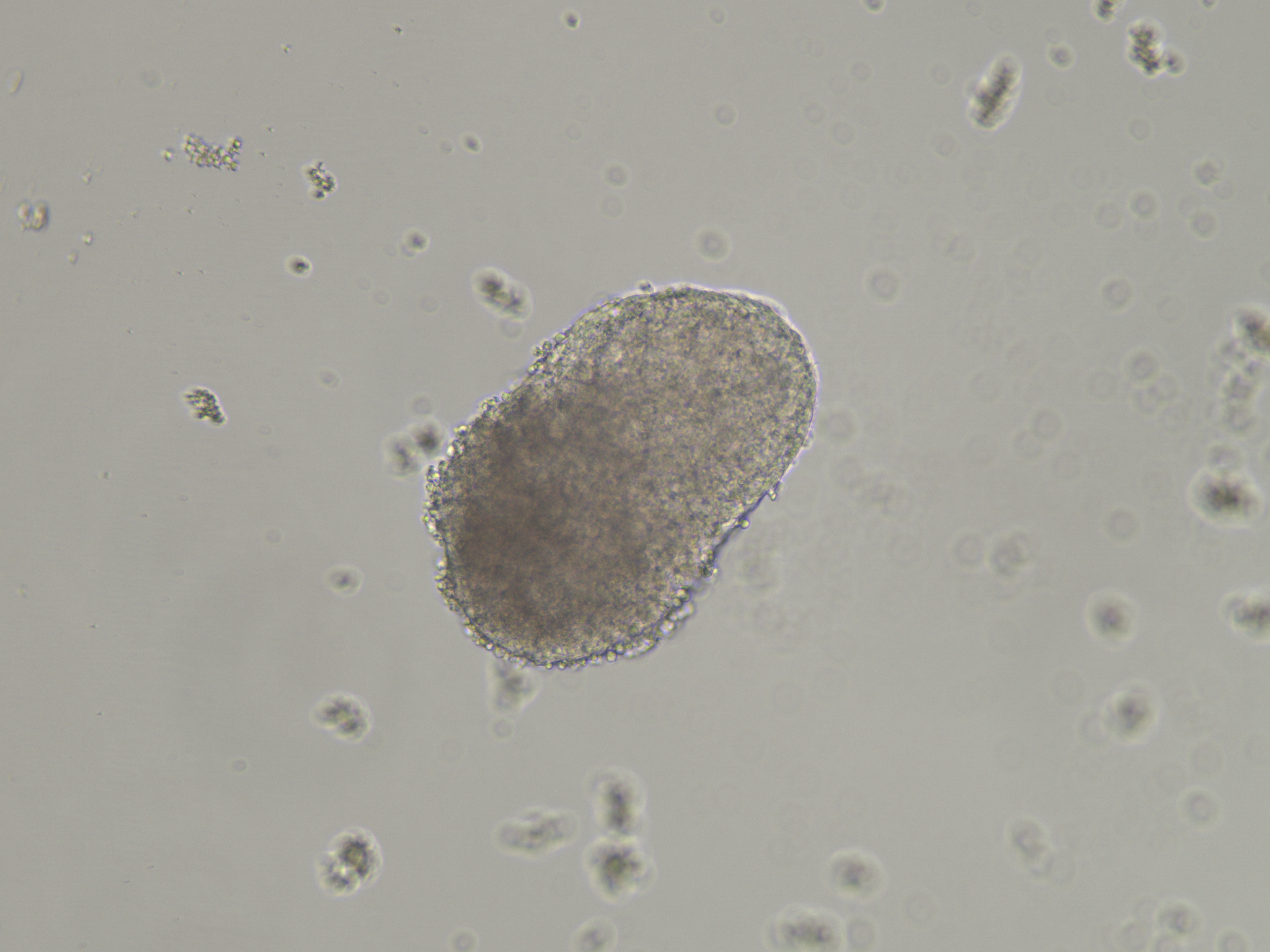

Supplement: Supplementary file 9 — Source data Fig. 7 [file 44318_2025_558_MOESM9_ESM.zip › Figure 7/panel 7B/NT-KD1/Cond 4:1/4:1_1.tiff]

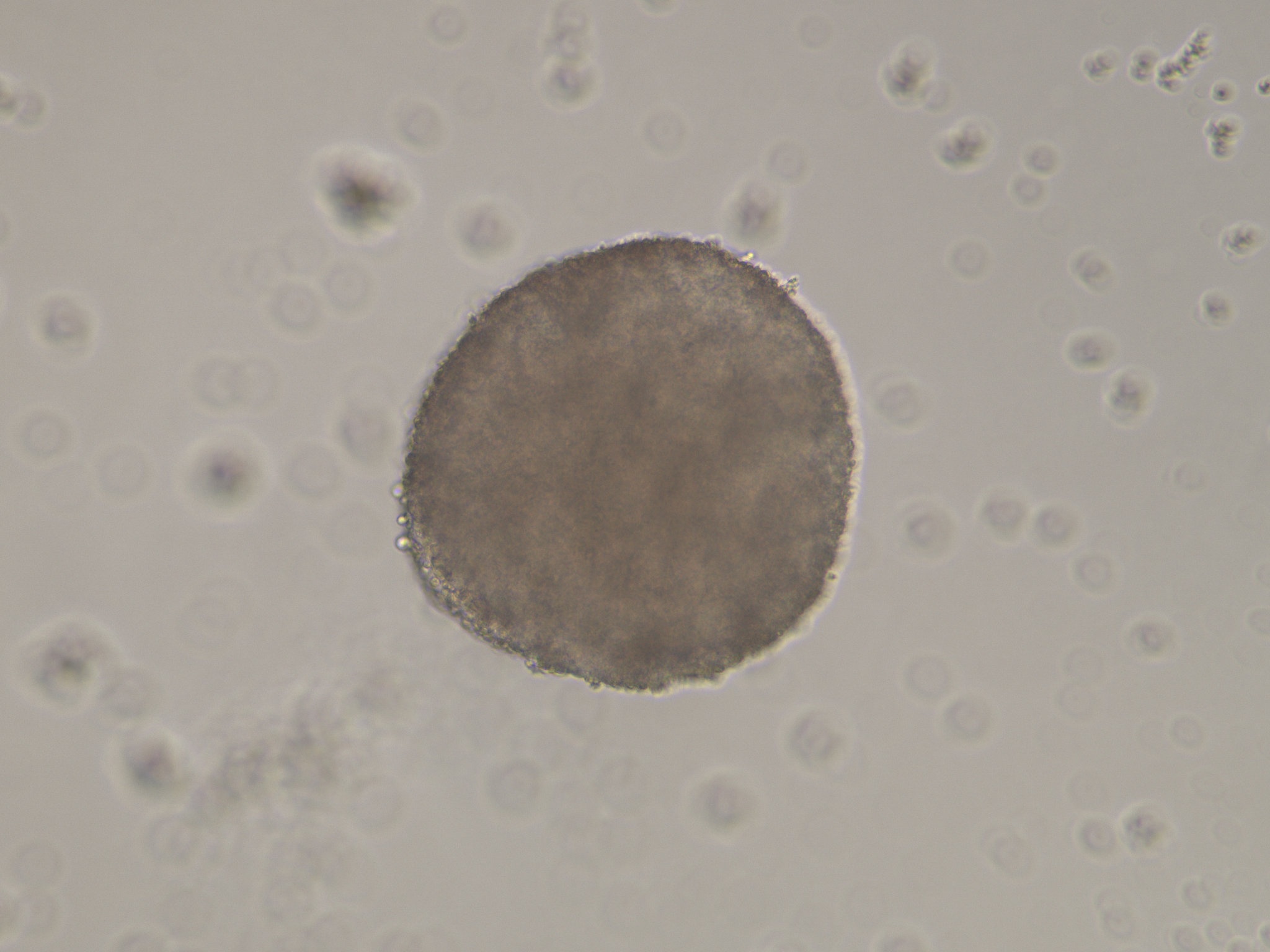

Supplement: Supplementary file 9 — Source data Fig. 7 [file 44318_2025_558_MOESM9_ESM.zip › Figure 7/panel 7B/NT-KD1/Cond 4:1/4:1_2.tiff]

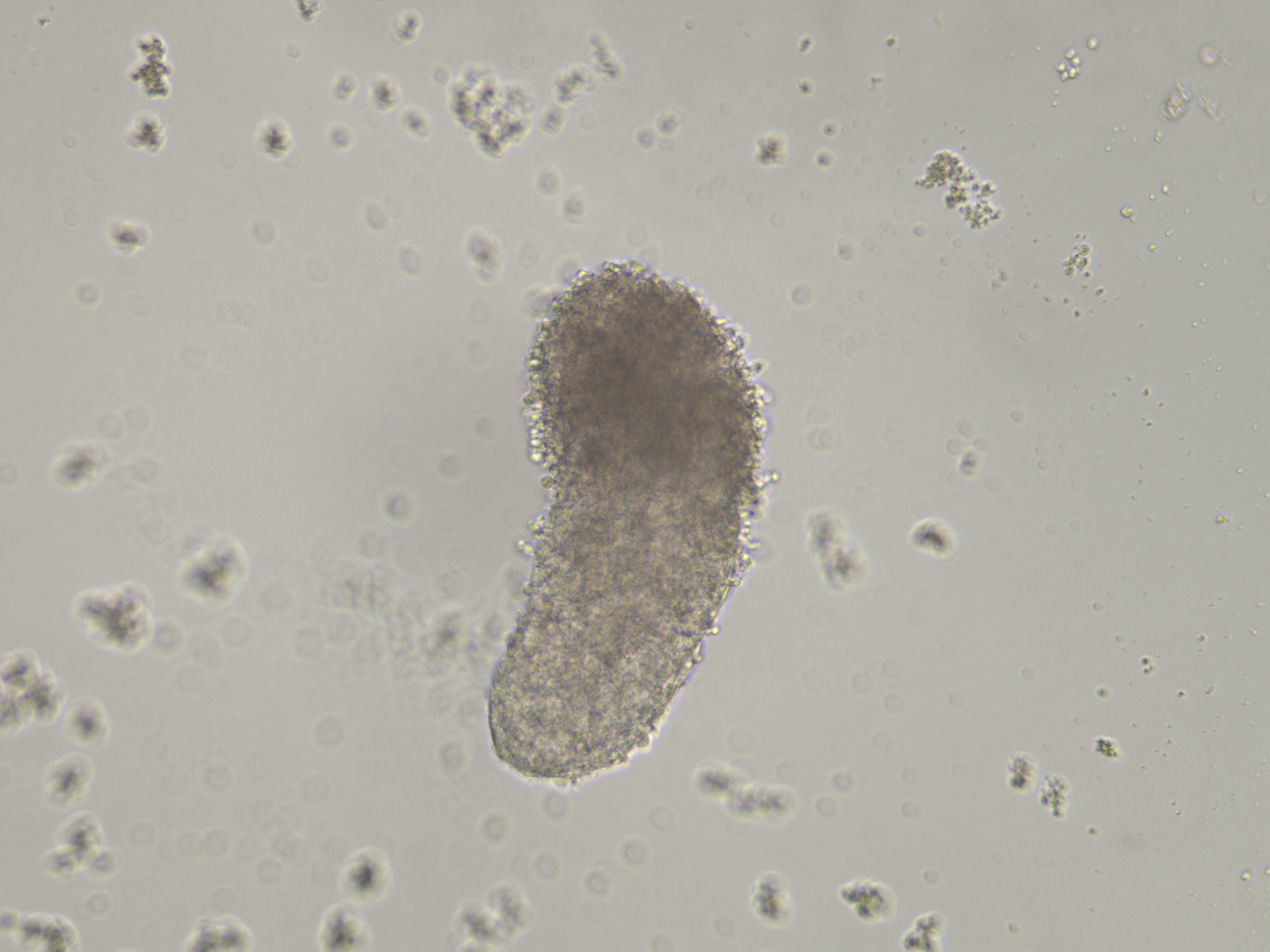

Supplement: Supplementary file 9 — Source data Fig. 7 [file 44318_2025_558_MOESM9_ESM.zip › Figure 7/panel 7B/NT-KD1/Cond 4:1/4:1_3.tiff]

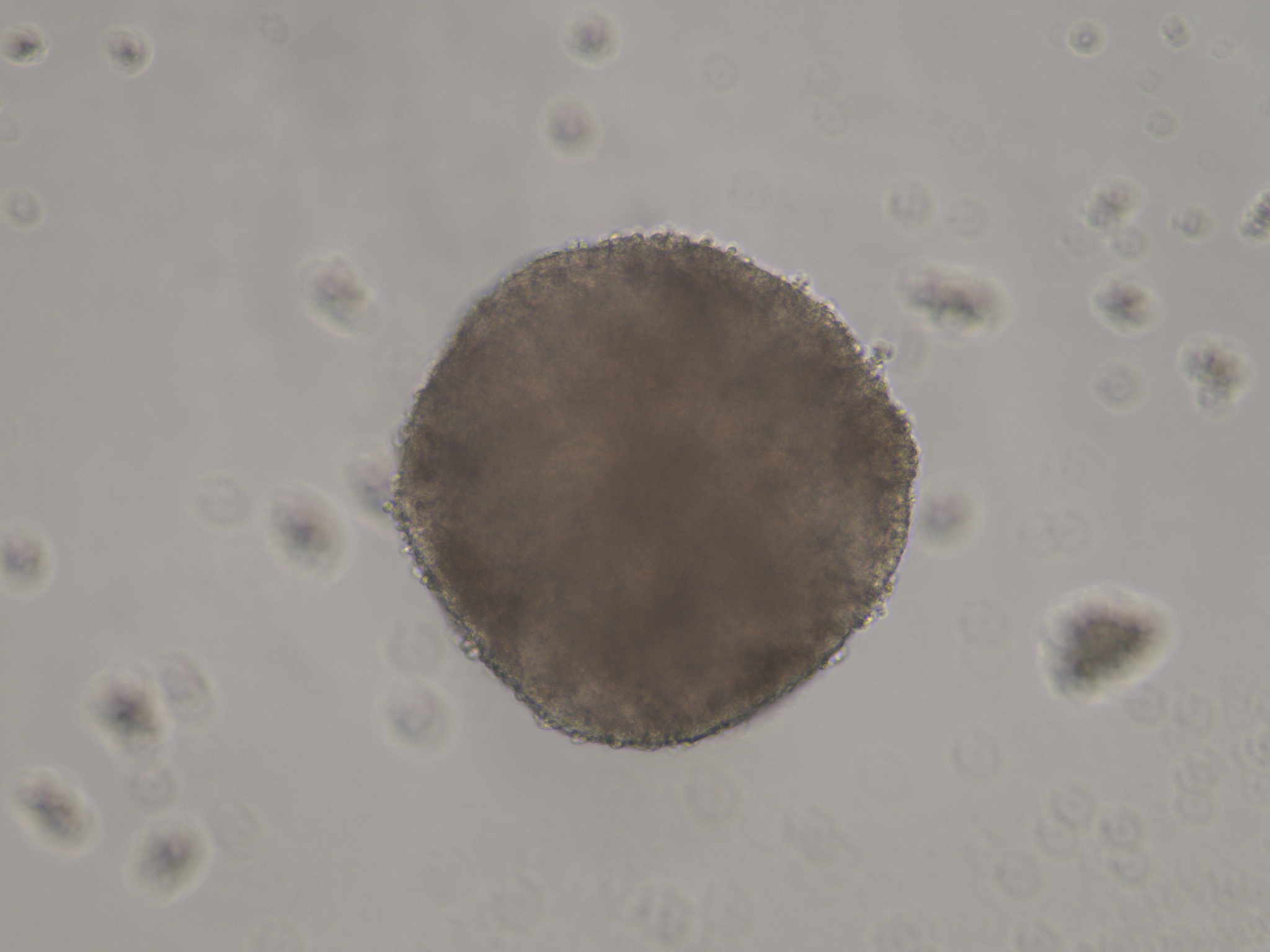

Supplement: Supplementary file 9 — Source data Fig. 7 [file 44318_2025_558_MOESM9_ESM.zip › Figure 7/panel 7B/NT-KD1/Cond 1:4/1:4_1.tiff]

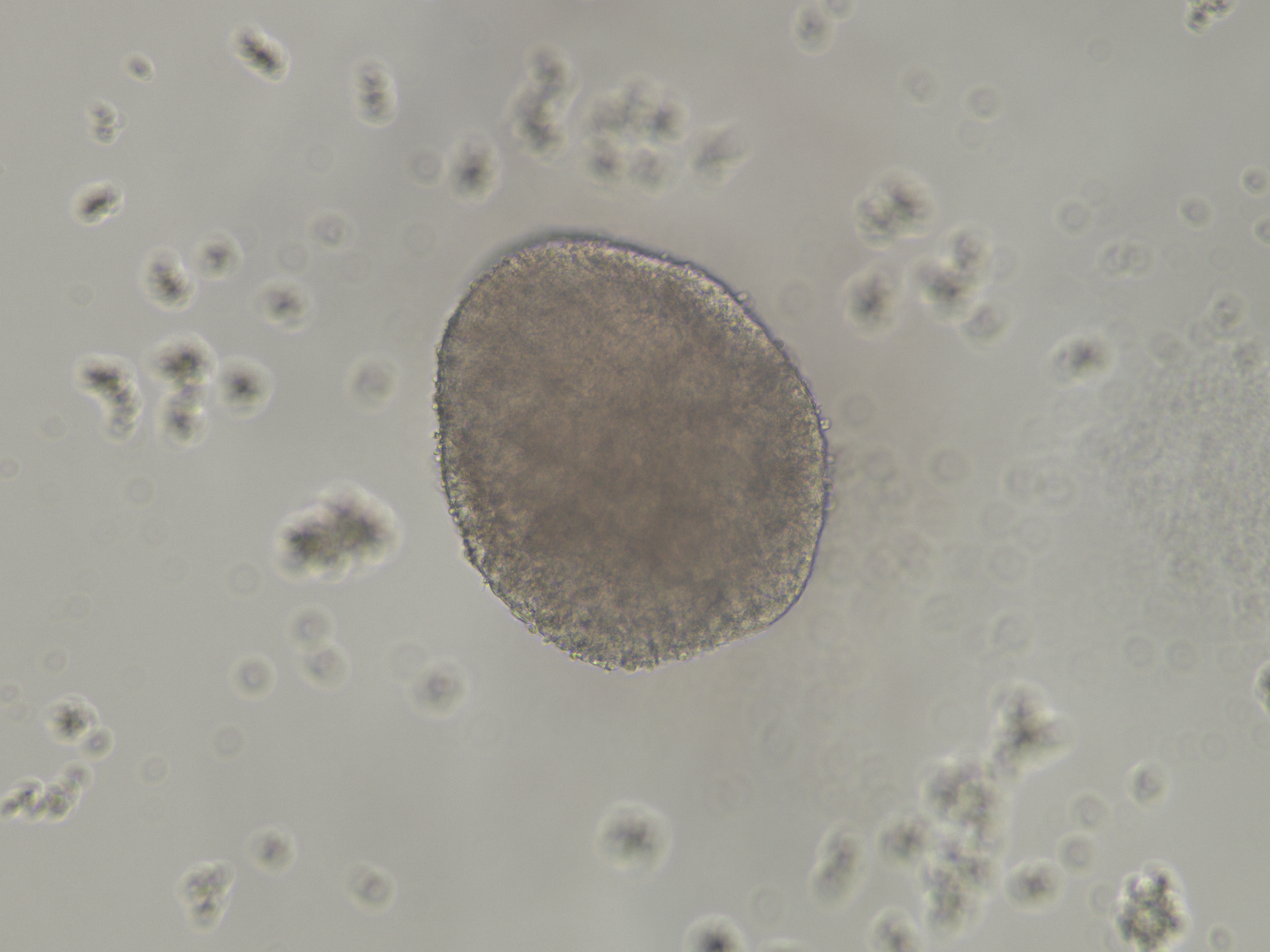

Supplement: Supplementary file 9 — Source data Fig. 7 [file 44318_2025_558_MOESM9_ESM.zip › Figure 7/panel 7B/NT-KD1/Cond 1:4/1:4_3.tiff]

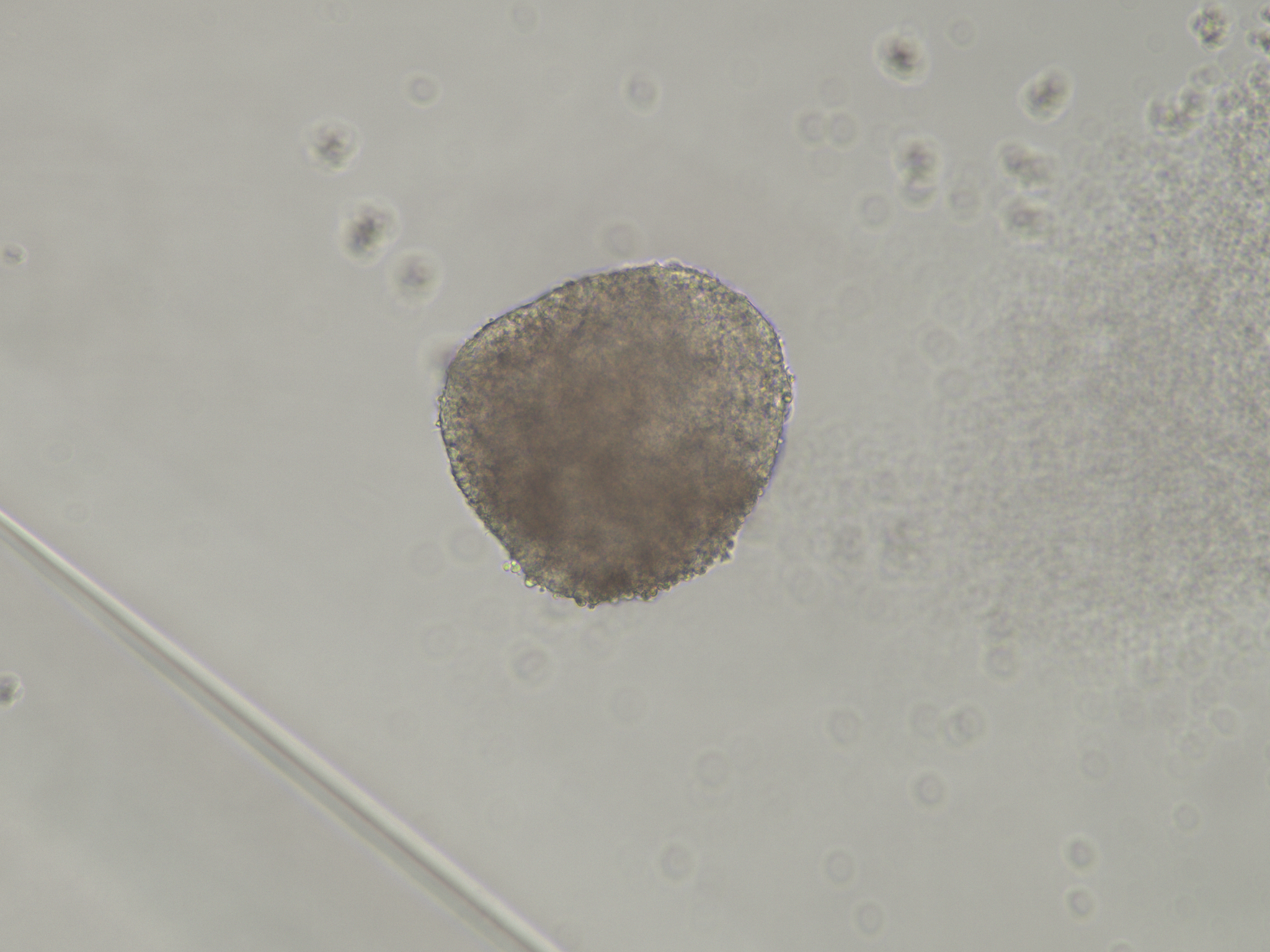

Supplement: Supplementary file 9 — Source data Fig. 7 [file 44318_2025_558_MOESM9_ESM.zip › Figure 7/panel 7B/NT-KD1/Cond 1:4/1:4_2.tiff]

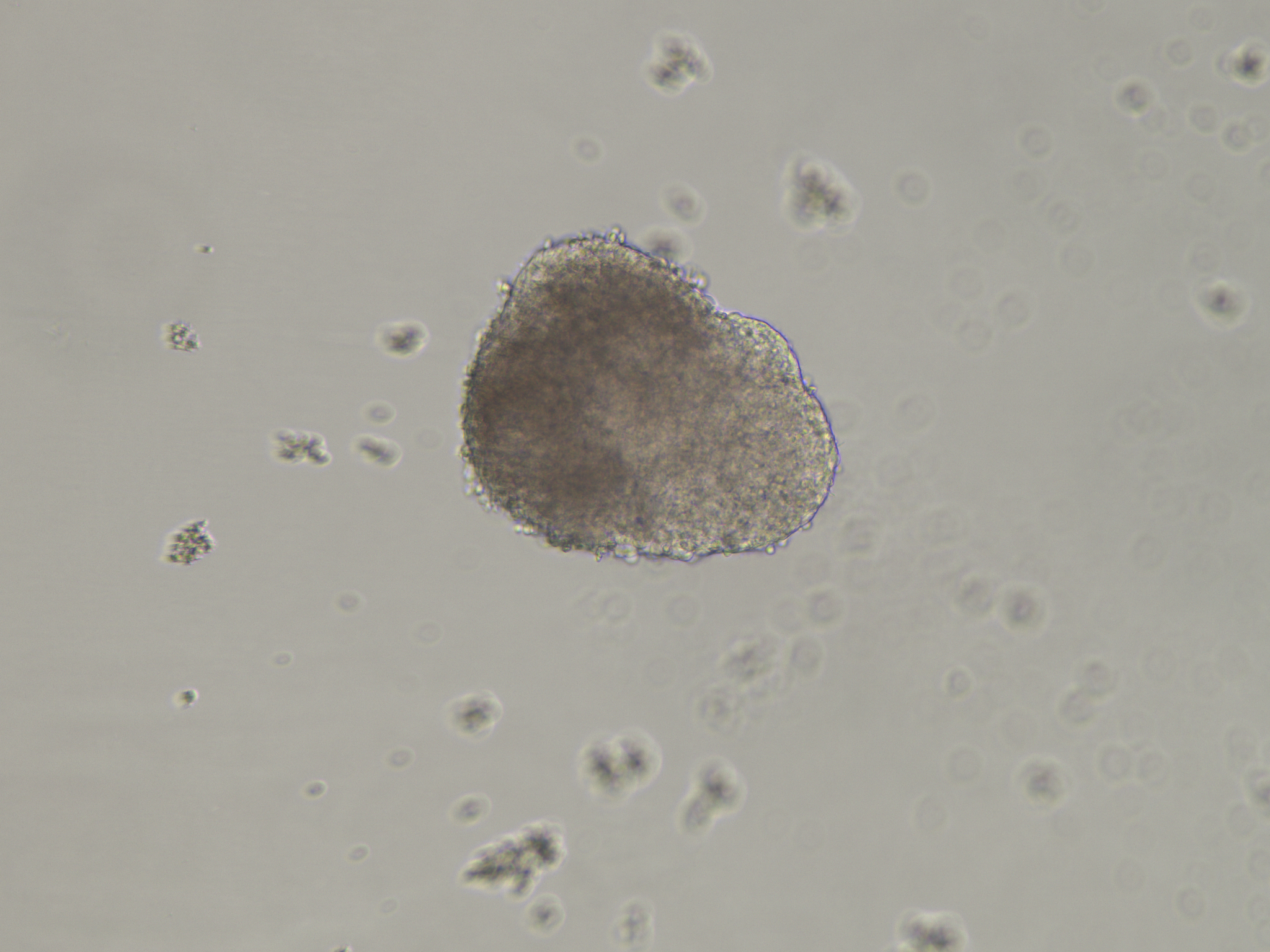

Supplement: Supplementary file 9 — Source data Fig. 7 [file 44318_2025_558_MOESM9_ESM.zip › Figure 7/panel 7B/NT-KD1/Cond 3:2/3:2_2.tiff]

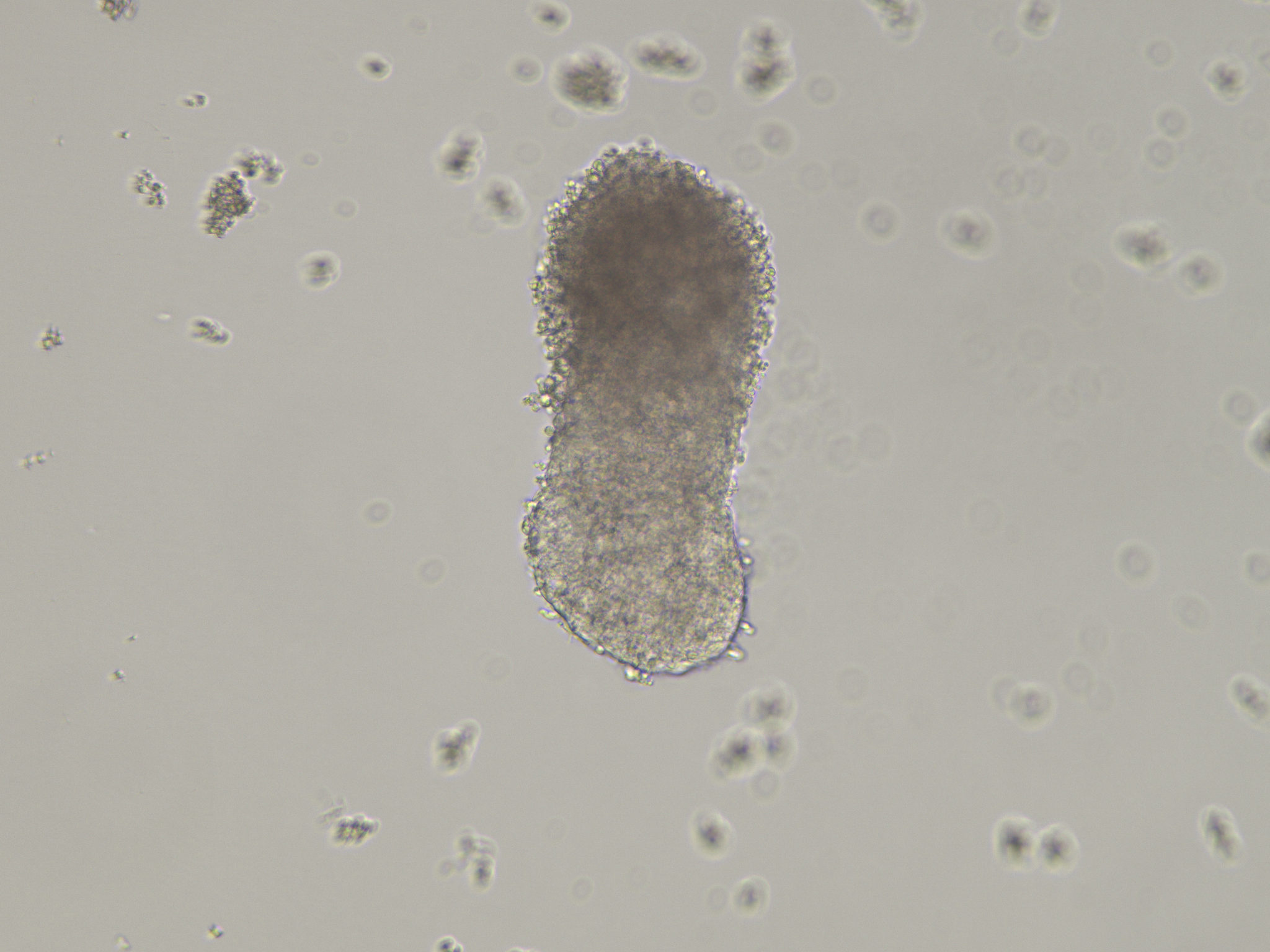

Supplement: Supplementary file 9 — Source data Fig. 7 [file 44318_2025_558_MOESM9_ESM.zip › Figure 7/panel 7B/NT-KD1/Cond 3:2/3:2_3.tiff]

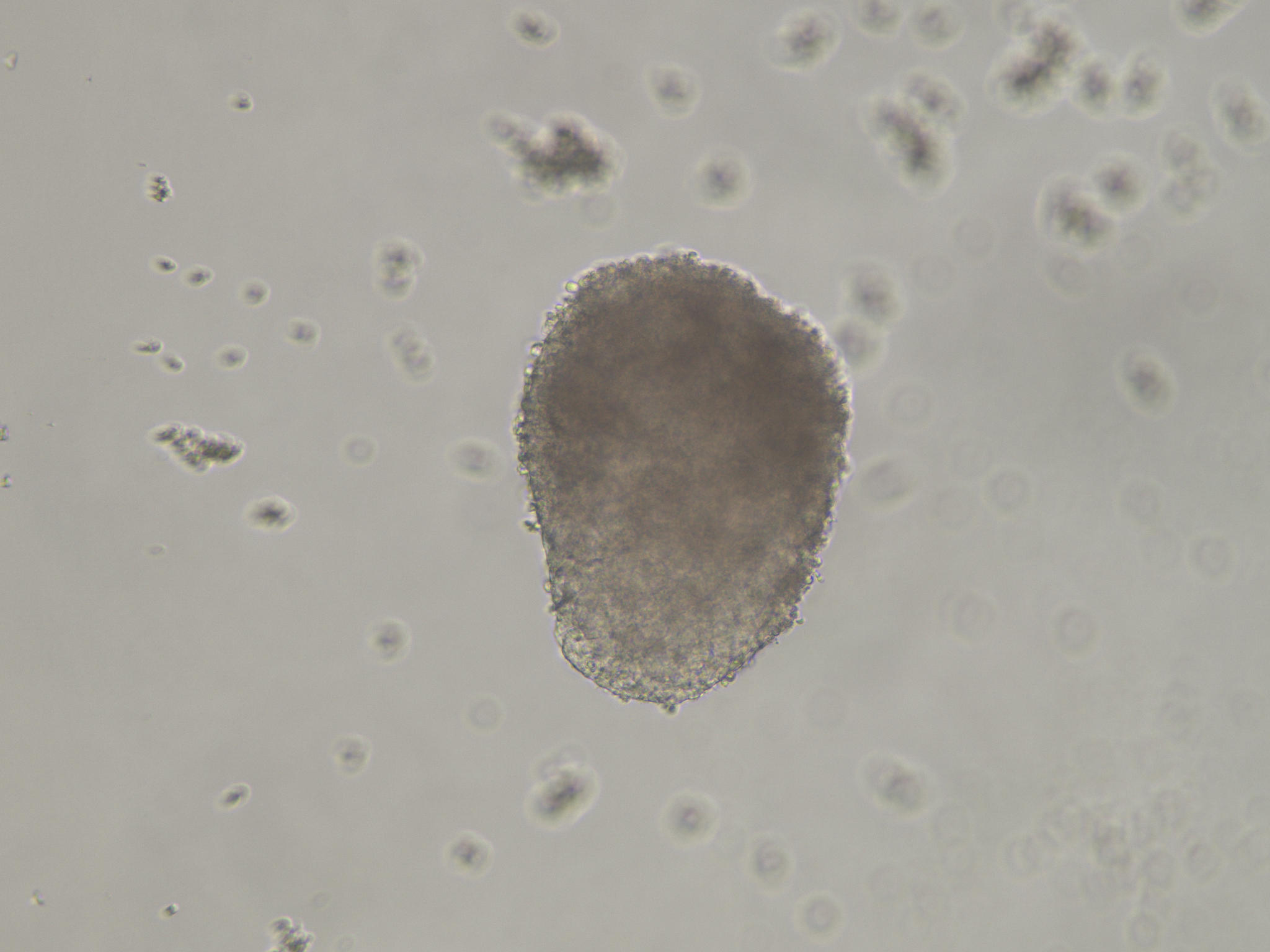

Supplement: Supplementary file 9 — Source data Fig. 7 [file 44318_2025_558_MOESM9_ESM.zip › Figure 7/panel 7B/NT-KD1/Cond 3:2/3:2_1.tiff]

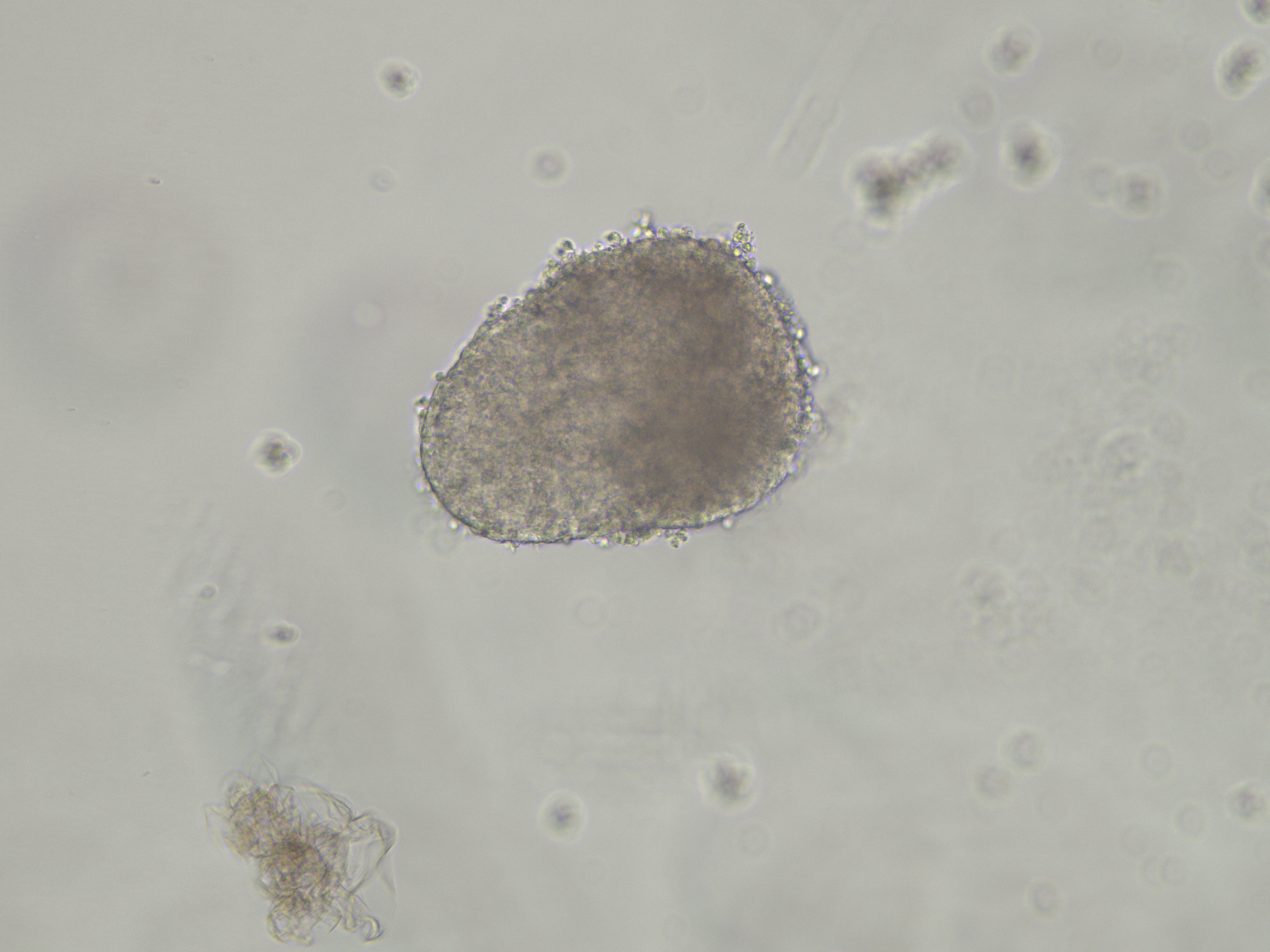

Supplement: Supplementary file 9 — Source data Fig. 7 [file 44318_2025_558_MOESM9_ESM.zip › Figure 7/panel 7B/NT-KD1/Cond 1:1/1:1_2.tiff]

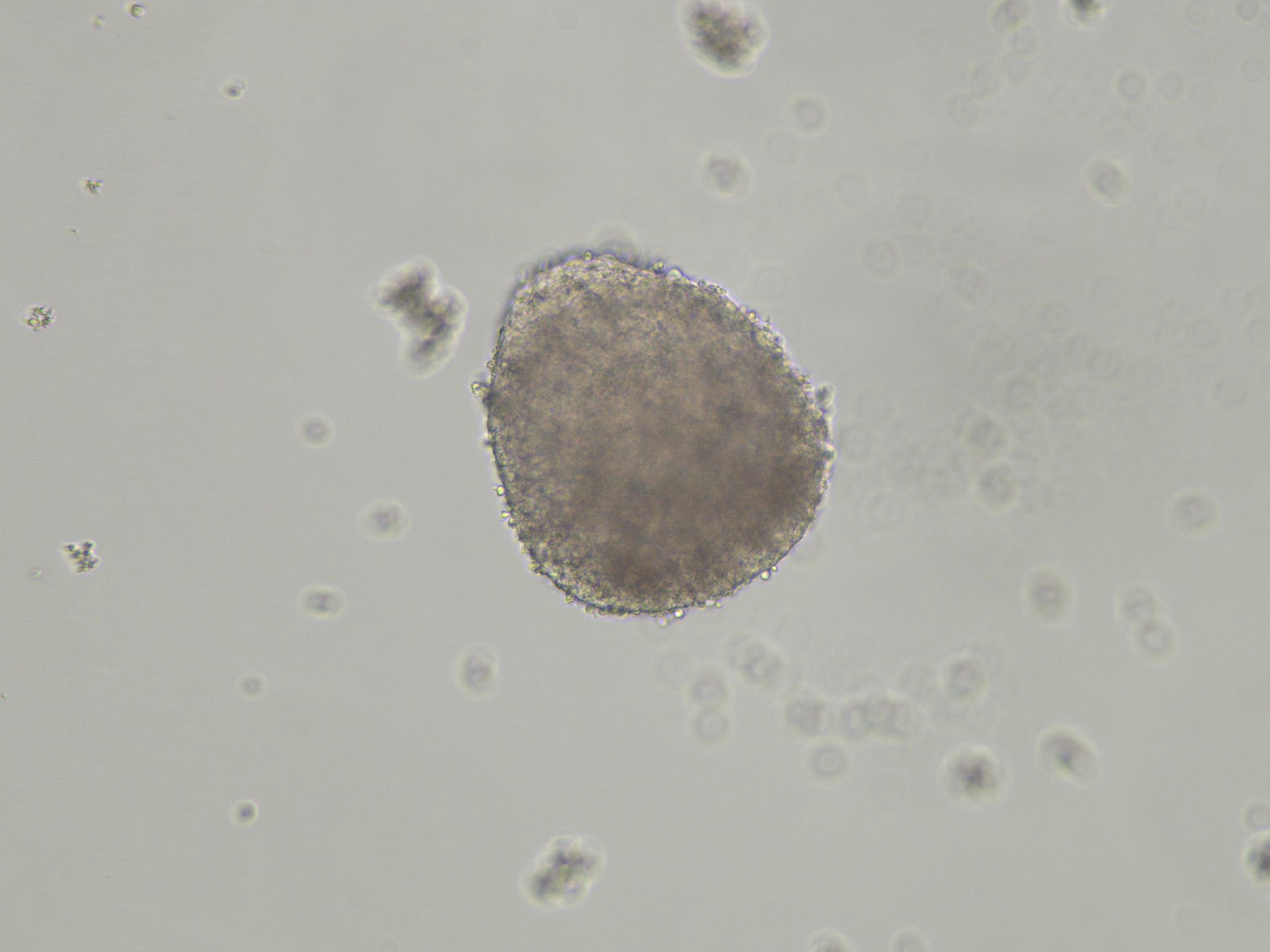

Supplement: Supplementary file 9 — Source data Fig. 7 [file 44318_2025_558_MOESM9_ESM.zip › Figure 7/panel 7B/NT-KD1/Cond 1:1/1:1_3.tiff]

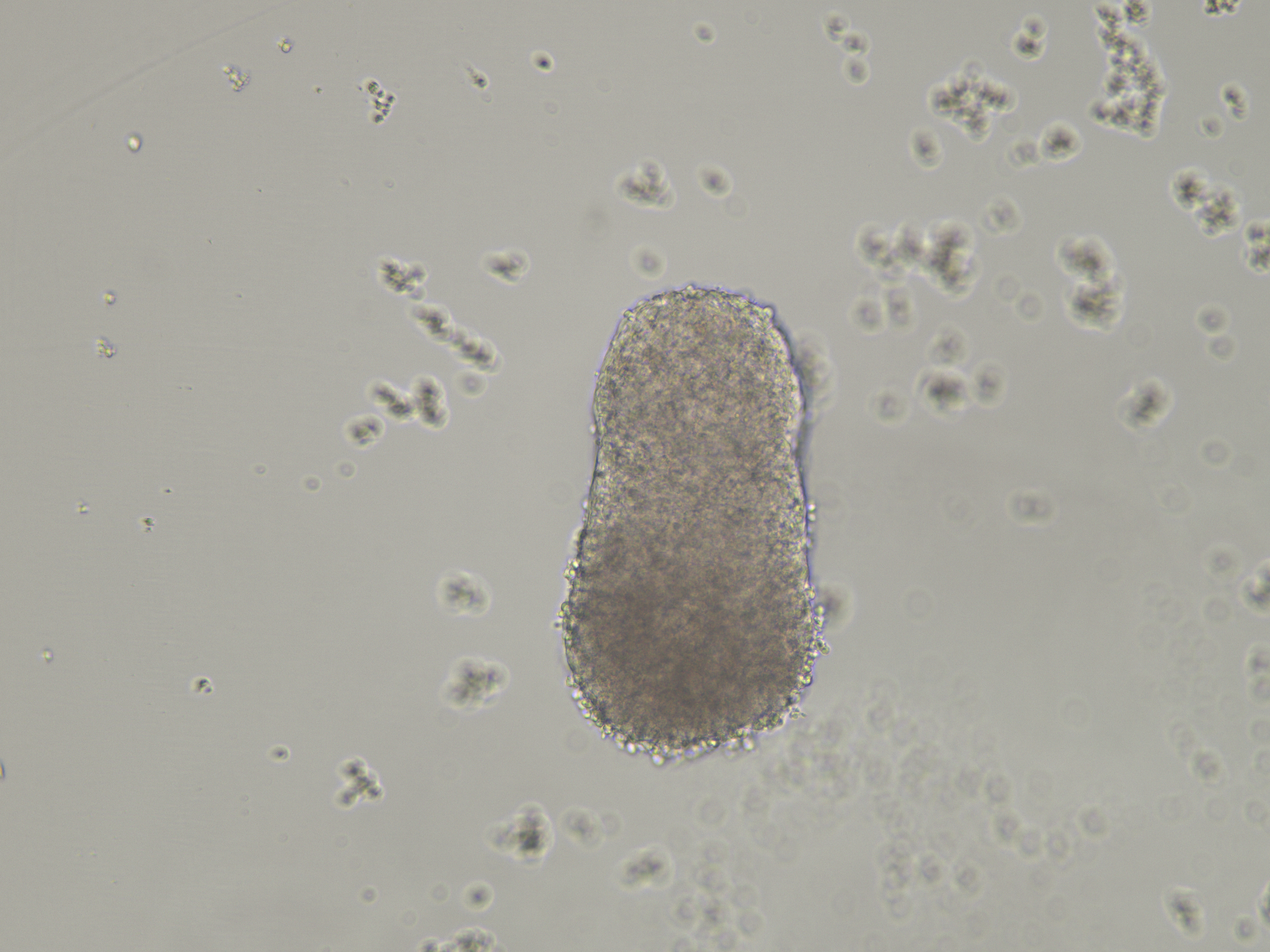

Supplement: Supplementary file 9 — Source data Fig. 7 [file 44318_2025_558_MOESM9_ESM.zip › Figure 7/panel 7B/NT-KD1/Cond 1:1/1:1_1.tiff]

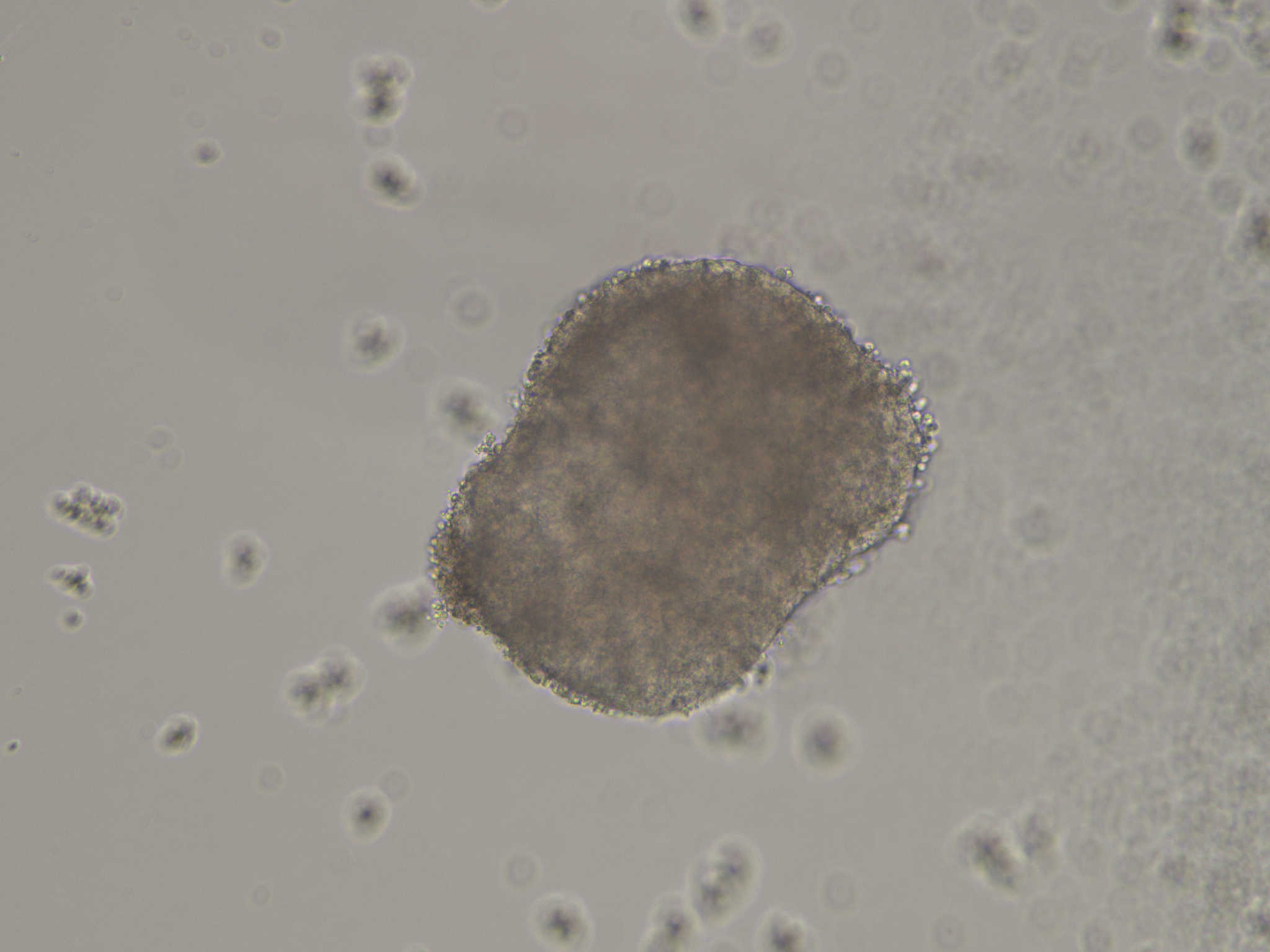

Supplement: Supplementary file 9 — Source data Fig. 7 [file 44318_2025_558_MOESM9_ESM.zip › Figure 7/panel 7C/NT-KD2/Cond 2:3/2:3_1.tiff]

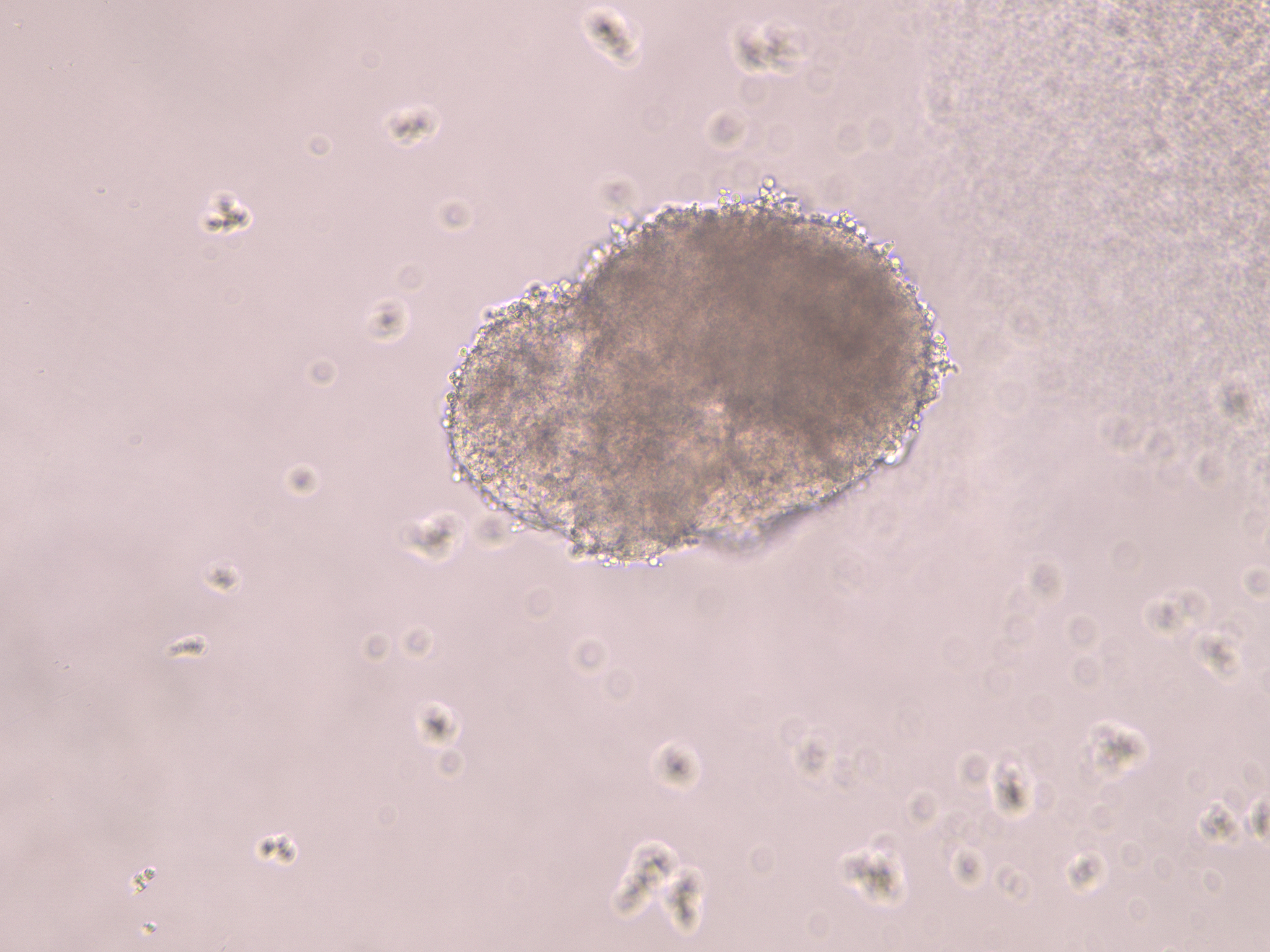

Supplement: Supplementary file 9 — Source data Fig. 7 [file 44318_2025_558_MOESM9_ESM.zip › Figure 7/panel 7C/NT-KD2/Cond 2:3/2:3_2.tiff]

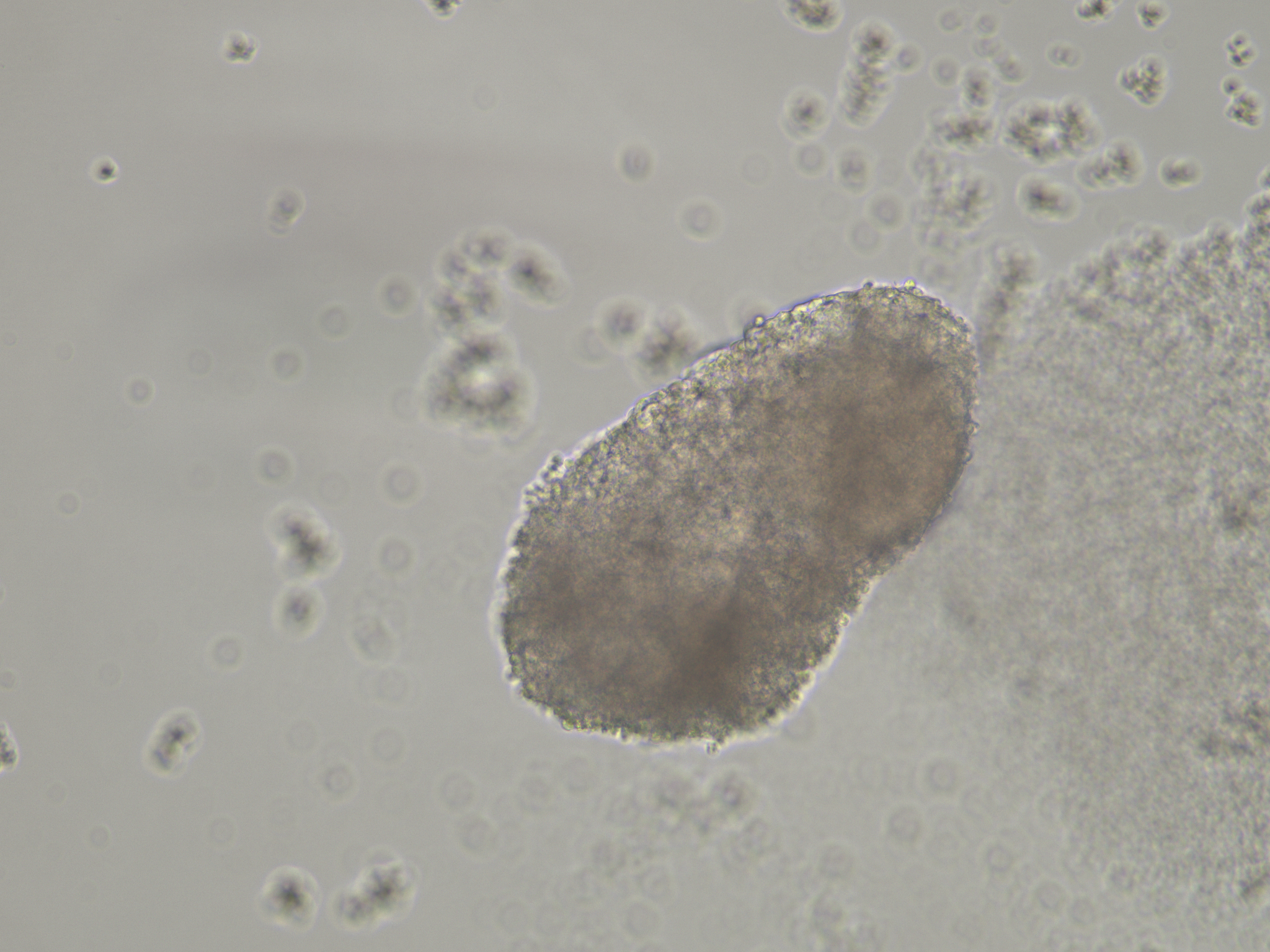

Supplement: Supplementary file 9 — Source data Fig. 7 [file 44318_2025_558_MOESM9_ESM.zip › Figure 7/panel 7C/NT-KD2/Cond 2:3/2:3_3.tiff]

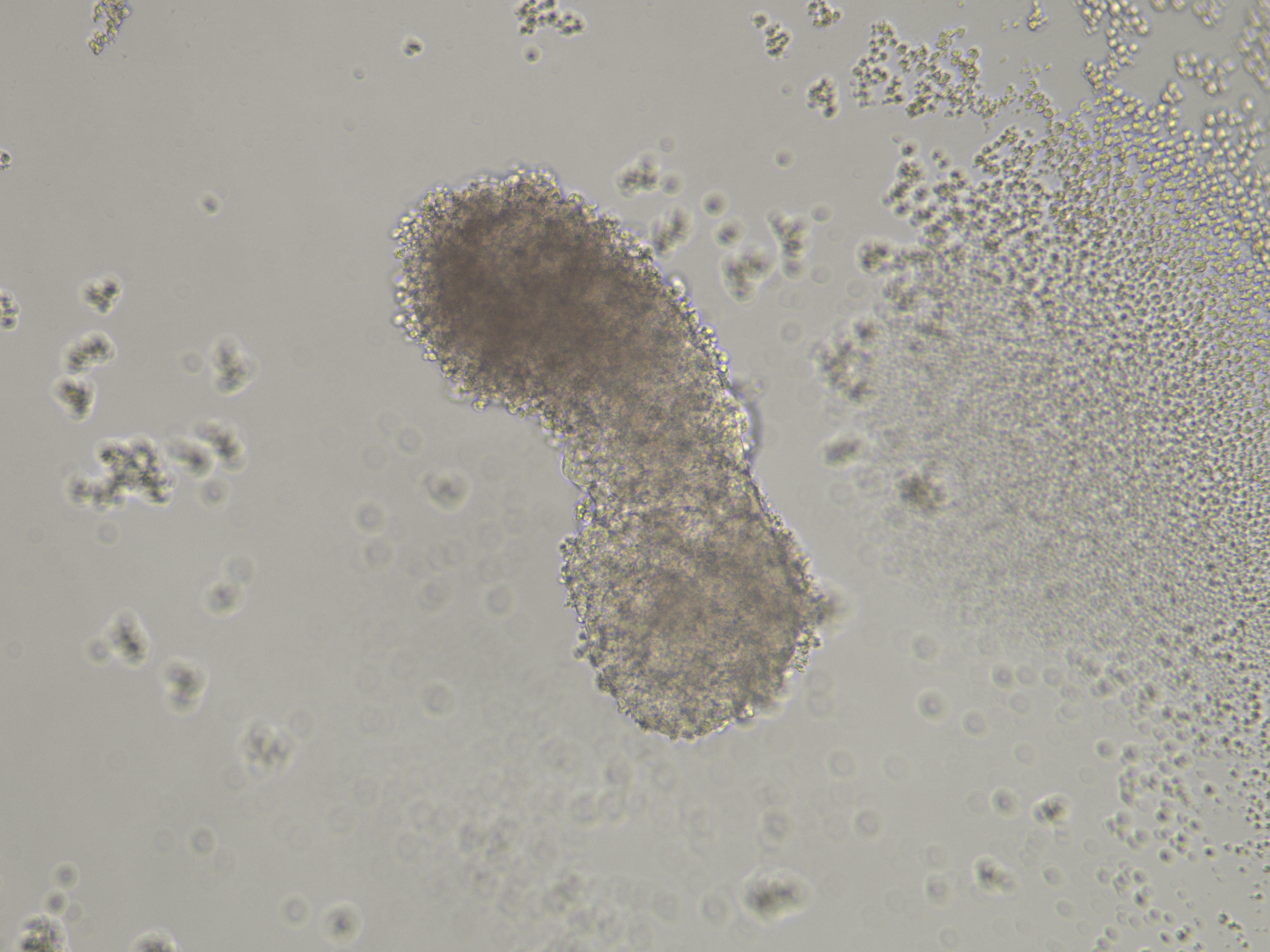

Supplement: Supplementary file 9 — Source data Fig. 7 [file 44318_2025_558_MOESM9_ESM.zip › Figure 7/panel 7C/NT-KD2/Cond 4:1/4:1_1.tiff]

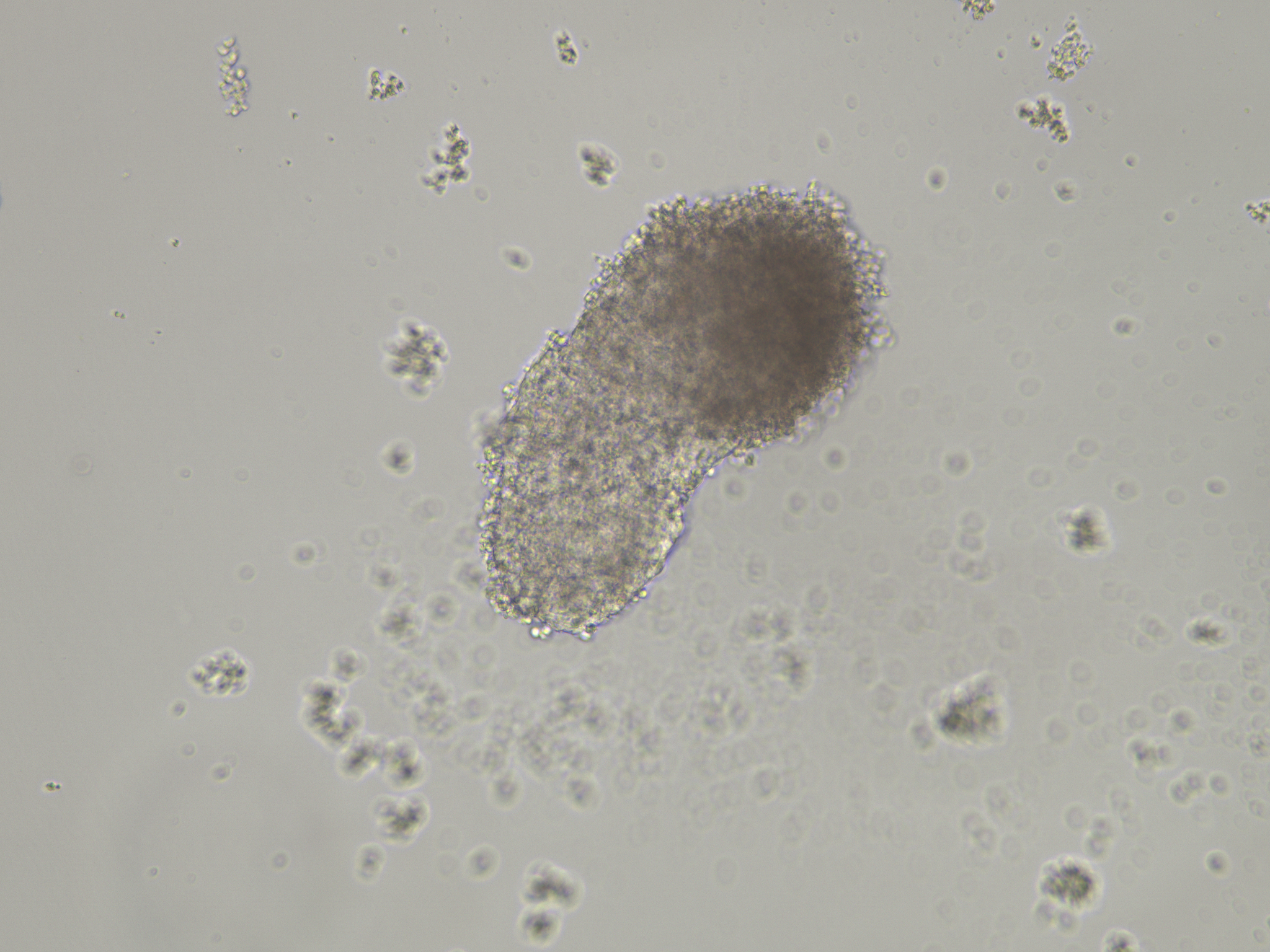

Supplement: Supplementary file 9 — Source data Fig. 7 [file 44318_2025_558_MOESM9_ESM.zip › Figure 7/panel 7C/NT-KD2/Cond 4:1/4:1_2.tiff]

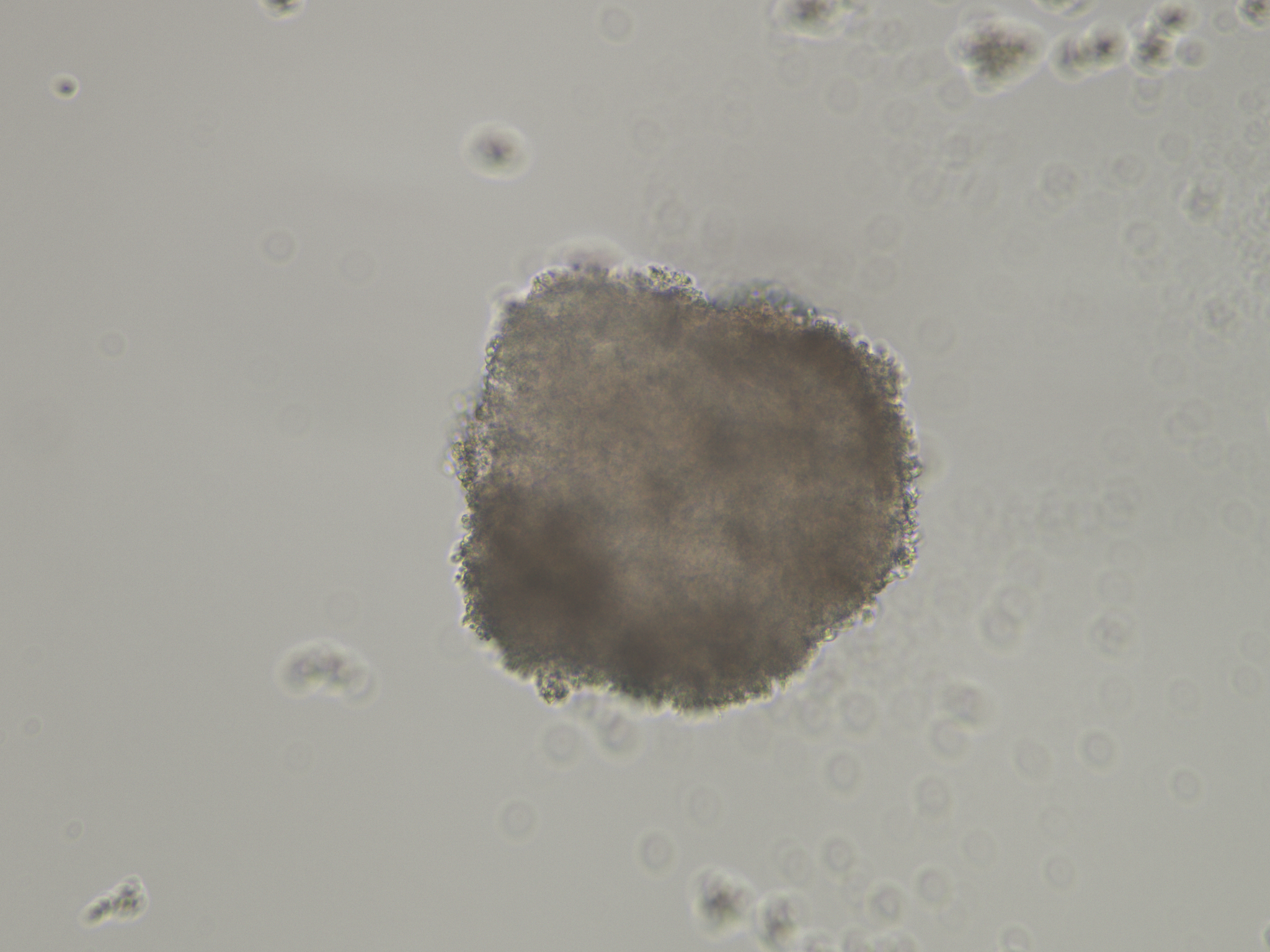

Supplement: Supplementary file 9 — Source data Fig. 7 [file 44318_2025_558_MOESM9_ESM.zip › Figure 7/panel 7C/NT-KD2/Cond 4:1/4:1_3.tiff]

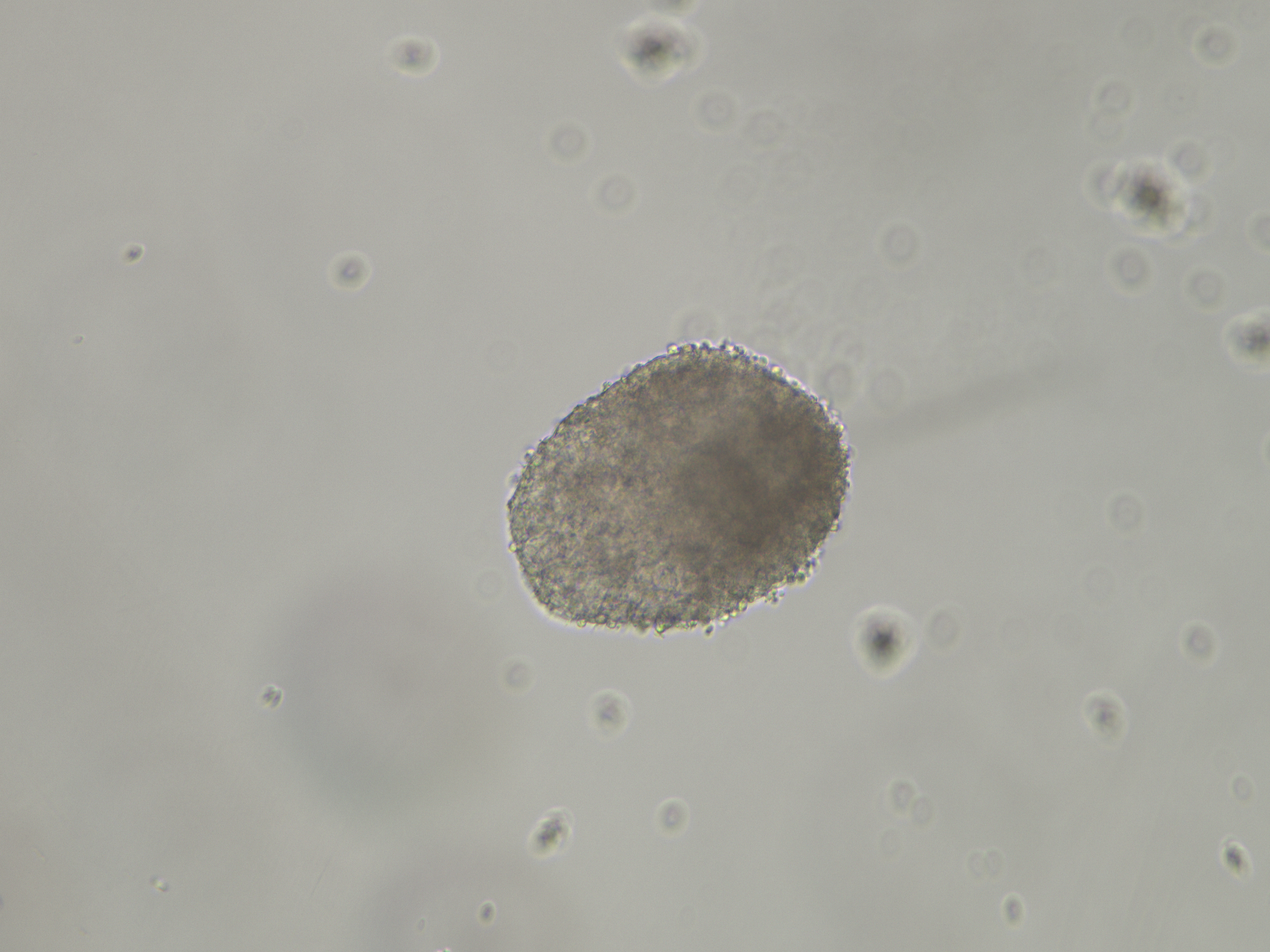

Supplement: Supplementary file 9 — Source data Fig. 7 [file 44318_2025_558_MOESM9_ESM.zip › Figure 7/panel 7C/NT-KD2/Cond 1:4/1:4_1.tiff]

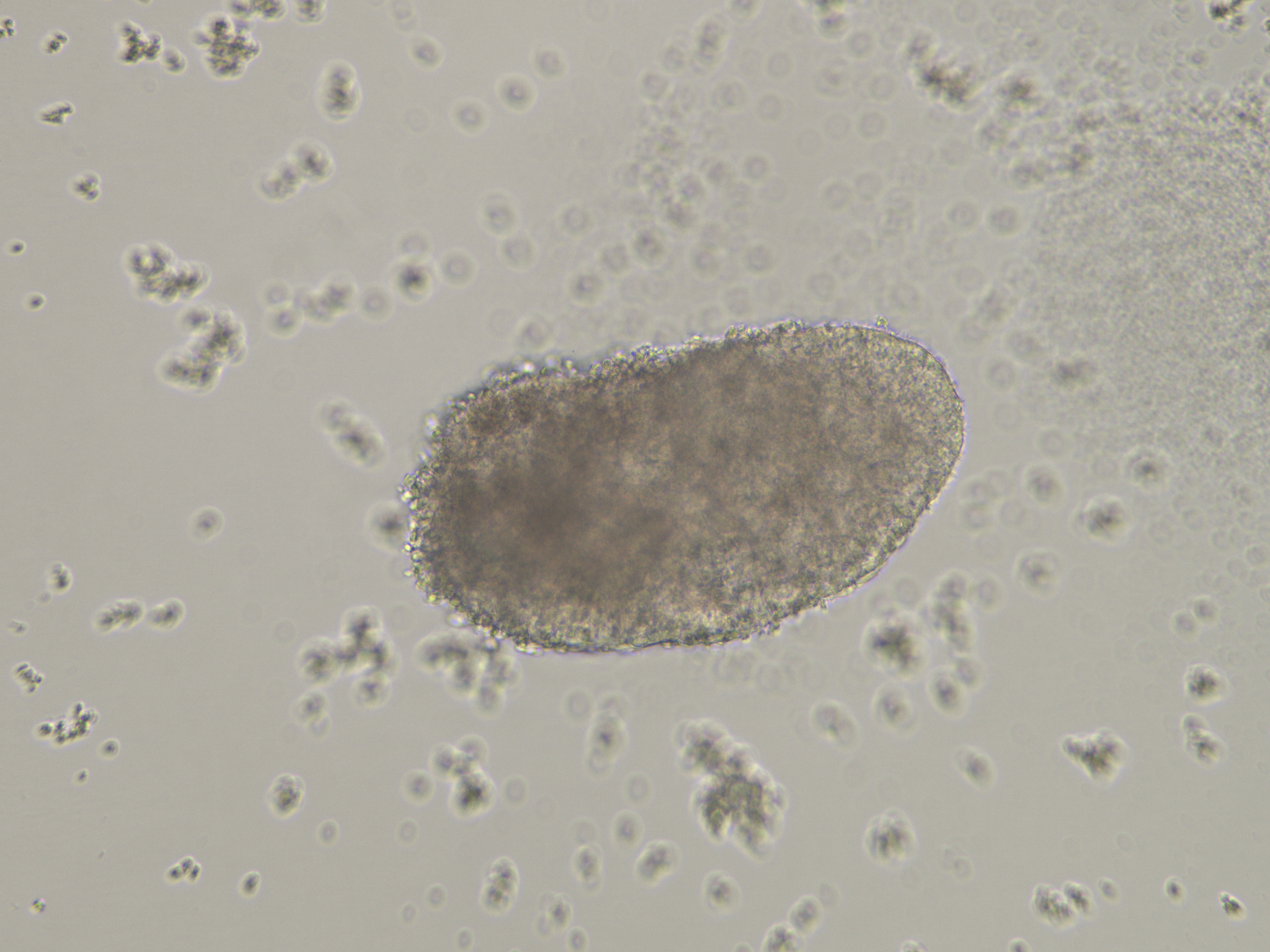

Supplement: Supplementary file 9 — Source data Fig. 7 [file 44318_2025_558_MOESM9_ESM.zip › Figure 7/panel 7C/NT-KD2/Cond 1:4/1:4_3.tiff]

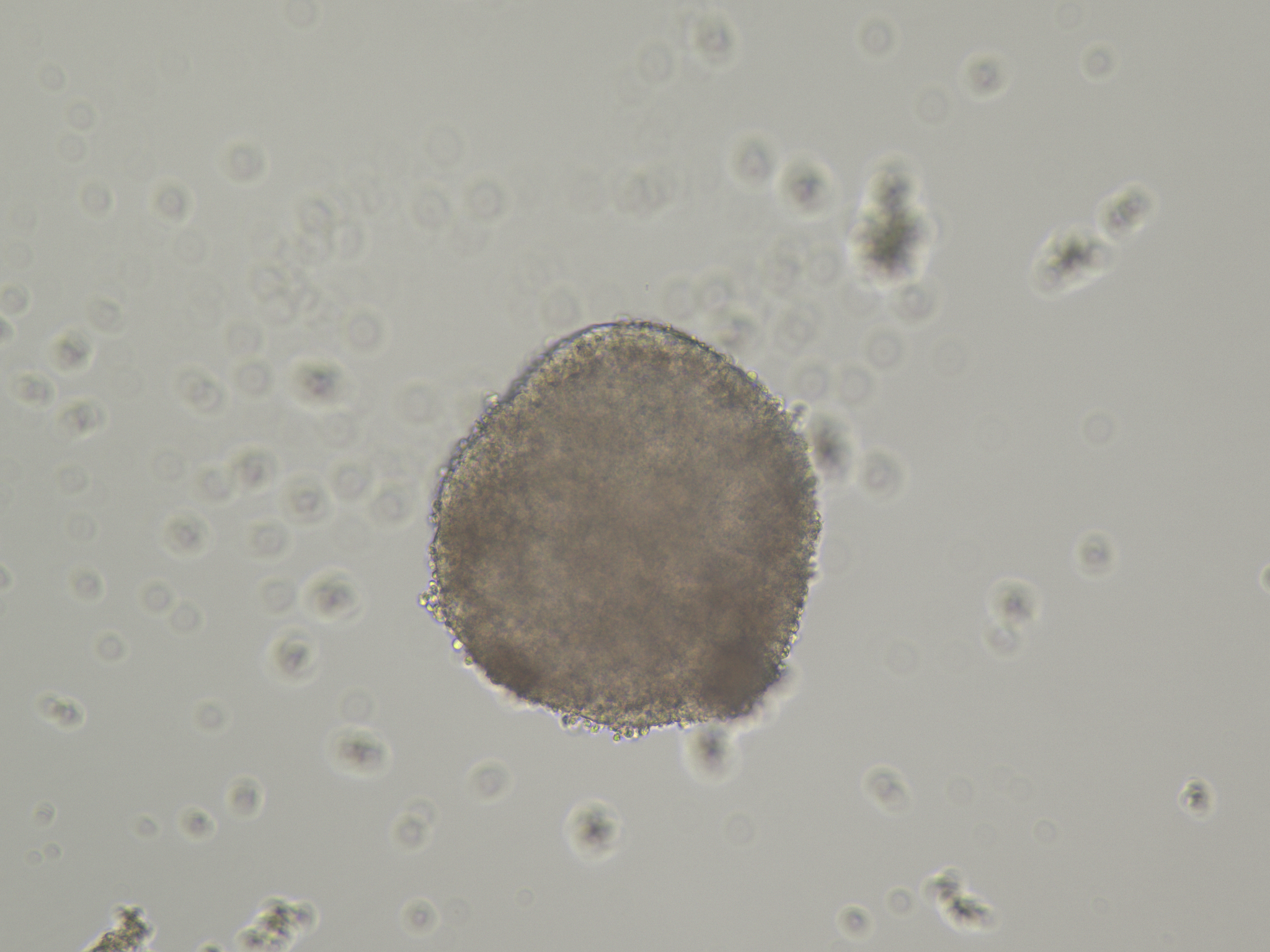

Supplement: Supplementary file 9 — Source data Fig. 7 [file 44318_2025_558_MOESM9_ESM.zip › Figure 7/panel 7C/NT-KD2/Cond 1:4/1:4_2.tiff]

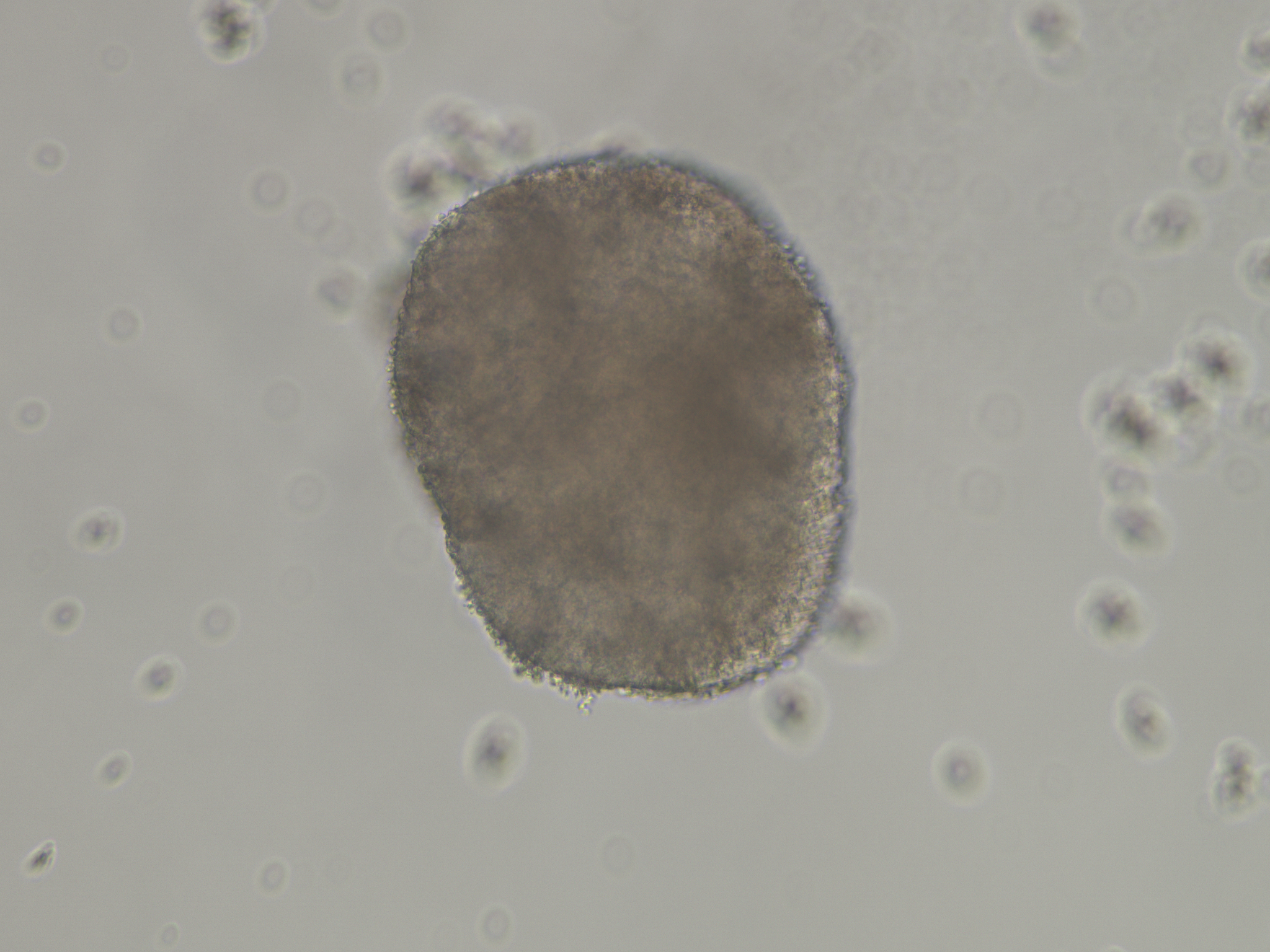

Supplement: Supplementary file 9 — Source data Fig. 7 [file 44318_2025_558_MOESM9_ESM.zip › Figure 7/panel 7C/NT-KD2/Cond 3:2/3:2_2.tiff]

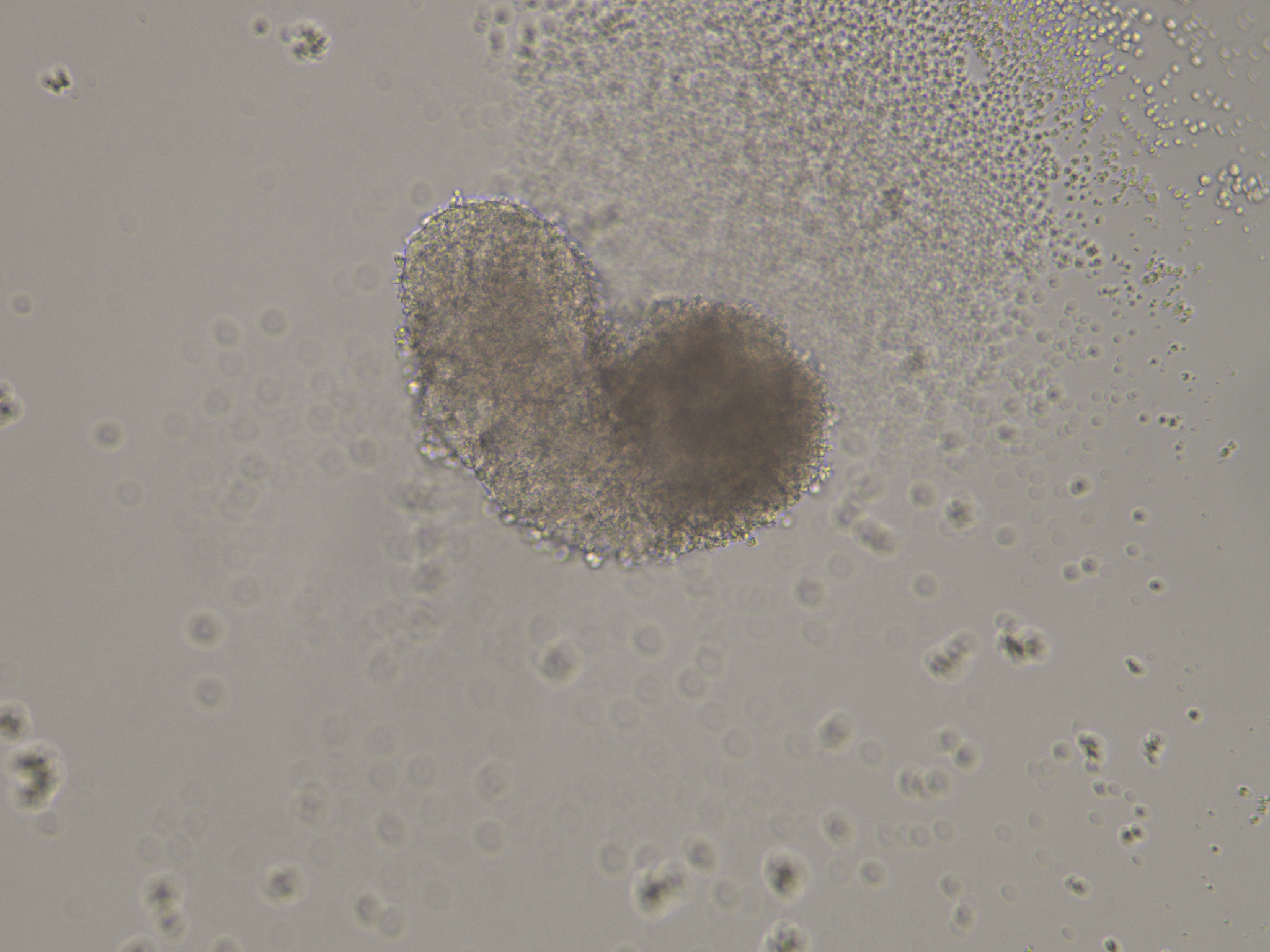

Supplement: Supplementary file 9 — Source data Fig. 7 [file 44318_2025_558_MOESM9_ESM.zip › Figure 7/panel 7C/NT-KD2/Cond 3:2/3:2_3.tiff]

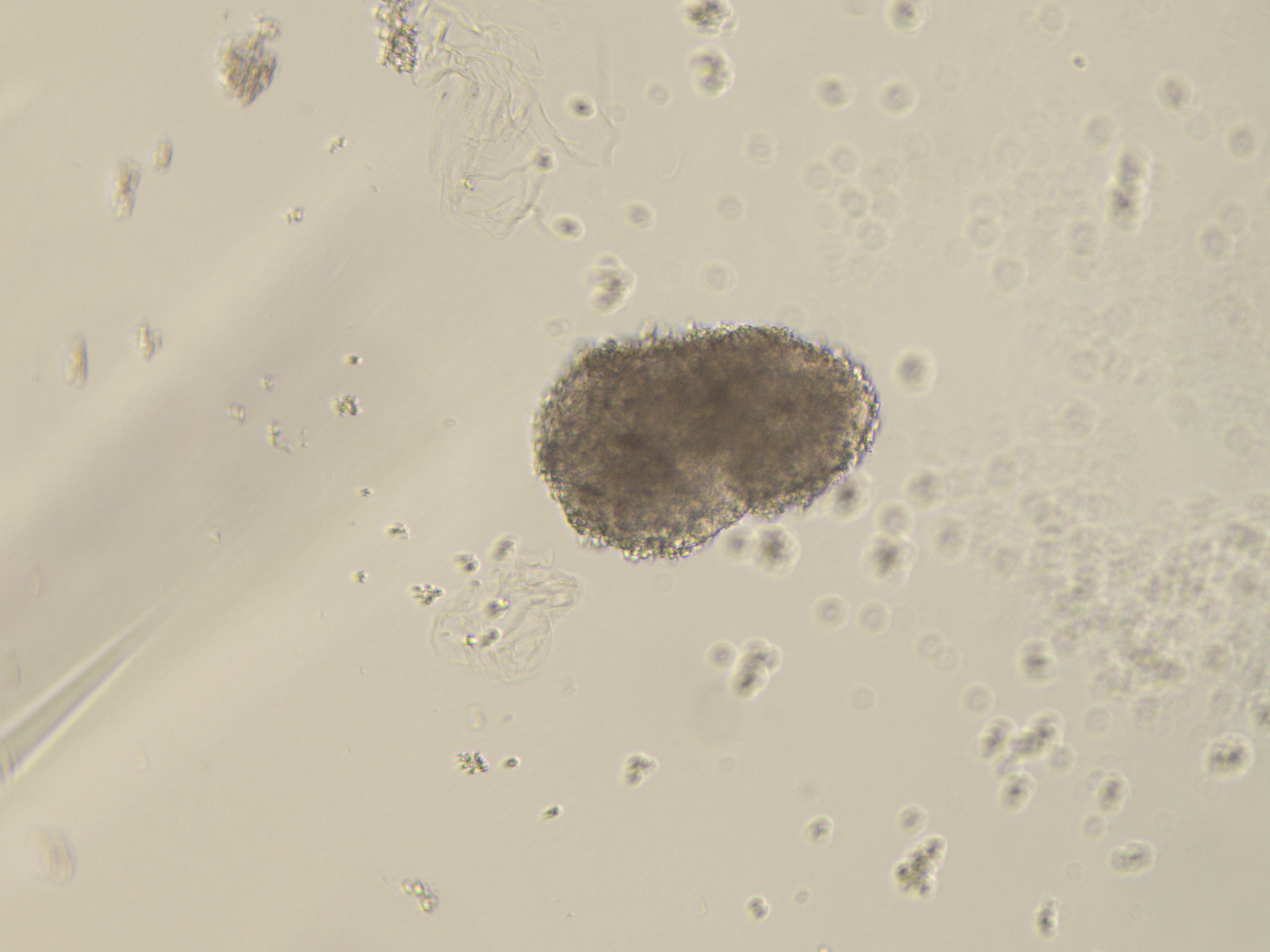

Supplement: Supplementary file 9 — Source data Fig. 7 [file 44318_2025_558_MOESM9_ESM.zip › Figure 7/panel 7C/NT-KD2/Cond 3:2/3:2_1.tiff]

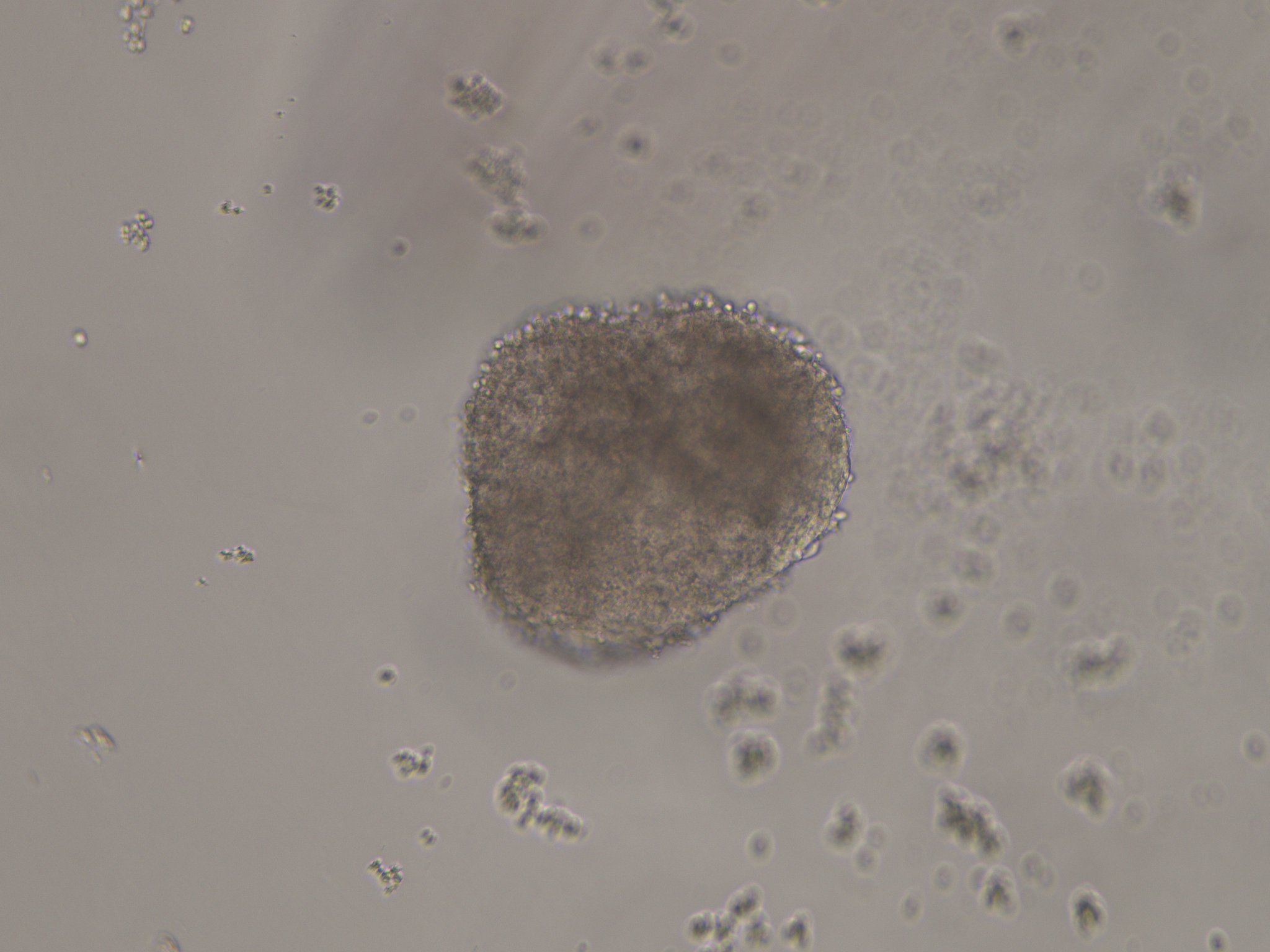

Supplement: Supplementary file 9 — Source data Fig. 7 [file 44318_2025_558_MOESM9_ESM.zip › Figure 7/panel 7C/NT-KD2/Cond 1:1/1:1_2.tiff]

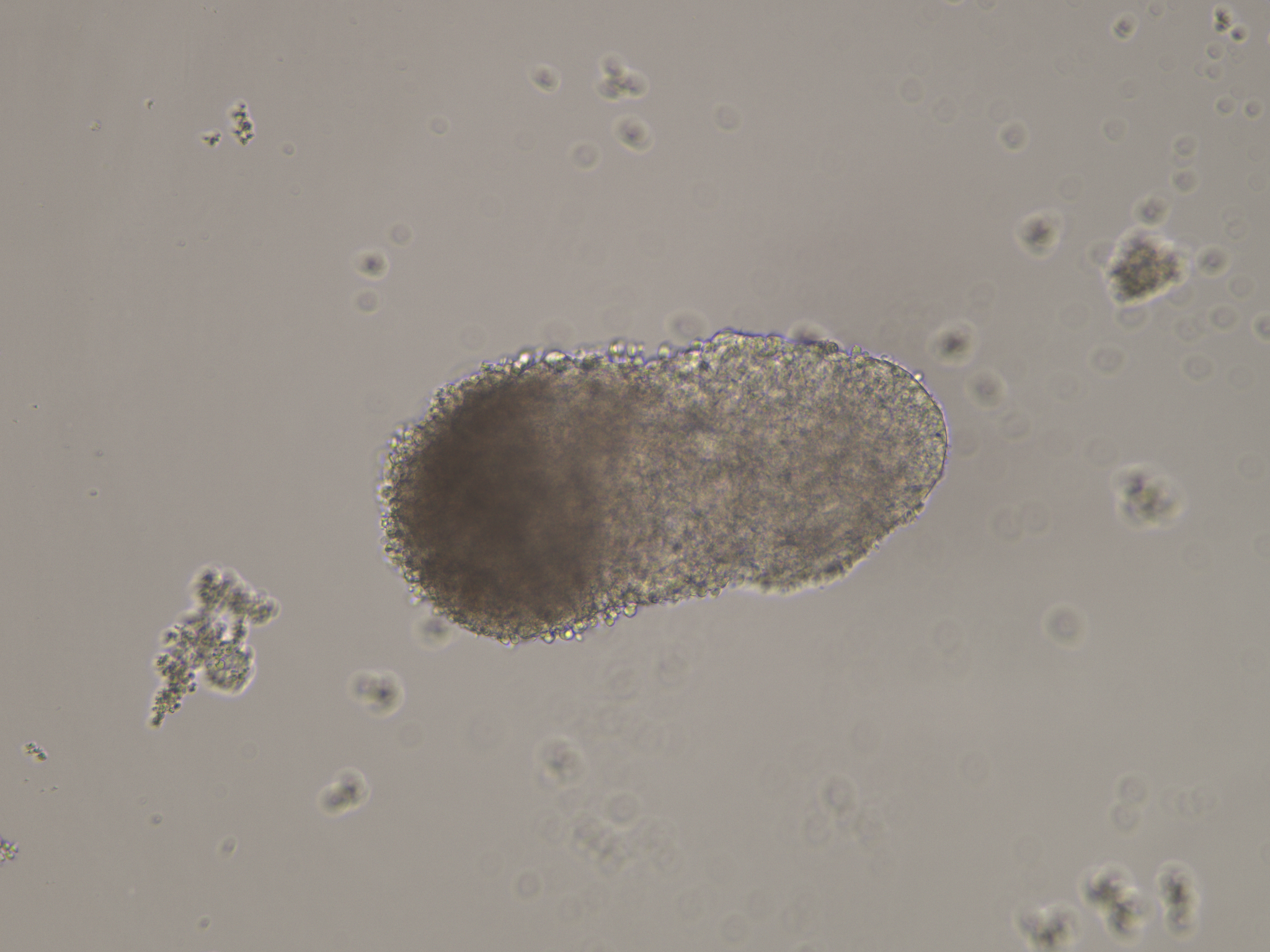

Supplement: Supplementary file 9 — Source data Fig. 7 [file 44318_2025_558_MOESM9_ESM.zip › Figure 7/panel 7C/NT-KD2/Cond 1:1/1:1_3.tiff]

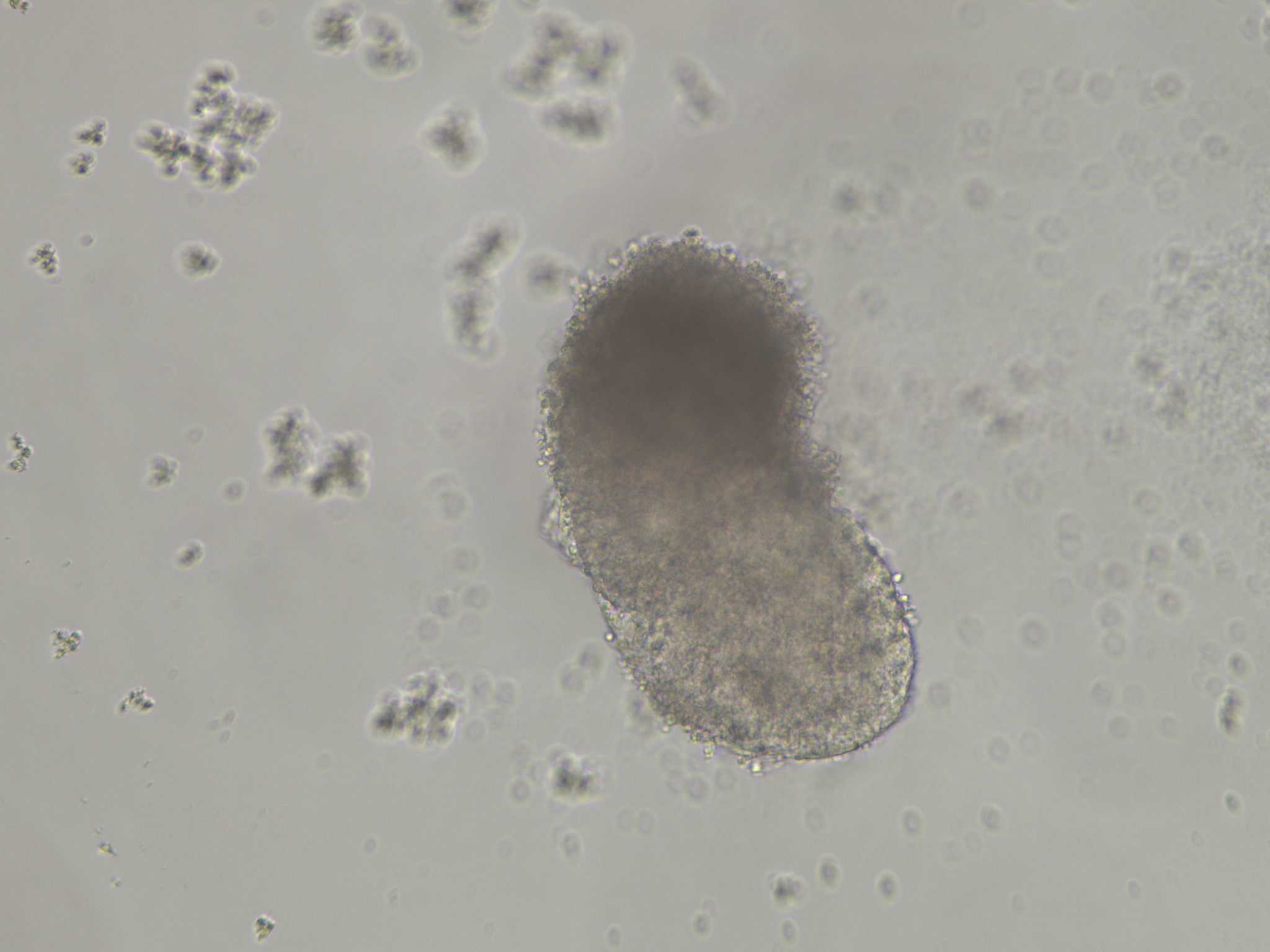

Supplement: Supplementary file 9 — Source data Fig. 7 [file 44318_2025_558_MOESM9_ESM.zip › Figure 7/panel 7C/NT-KD2/Cond 1:1/1:1_1.tiff]

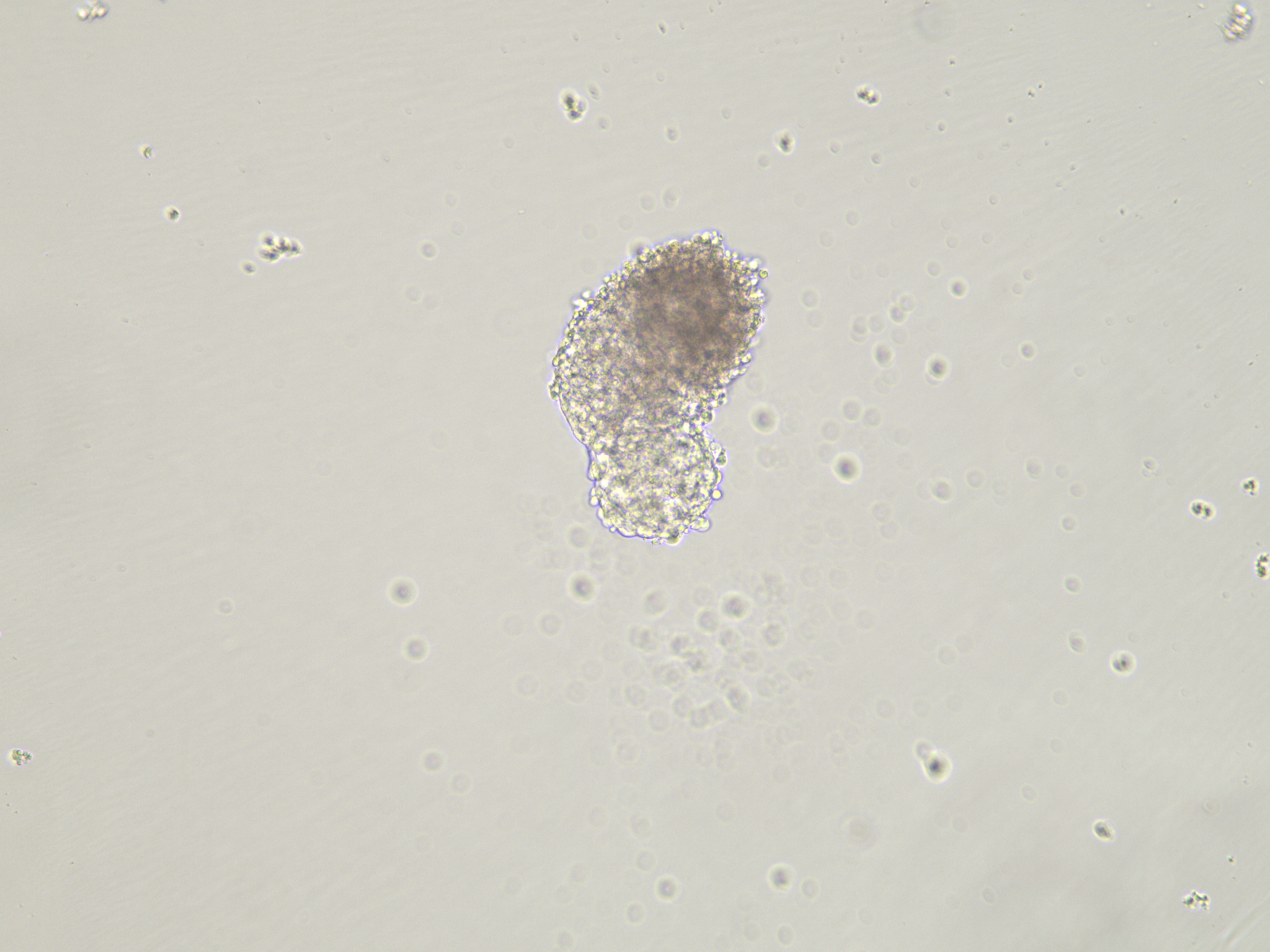

Supplement: Supplementary file 10 — Source data Fig. 8 [file 44318_2025_558_MOESM10_ESM.zip › Figure 8/panel 8B/NT_2.tiff]

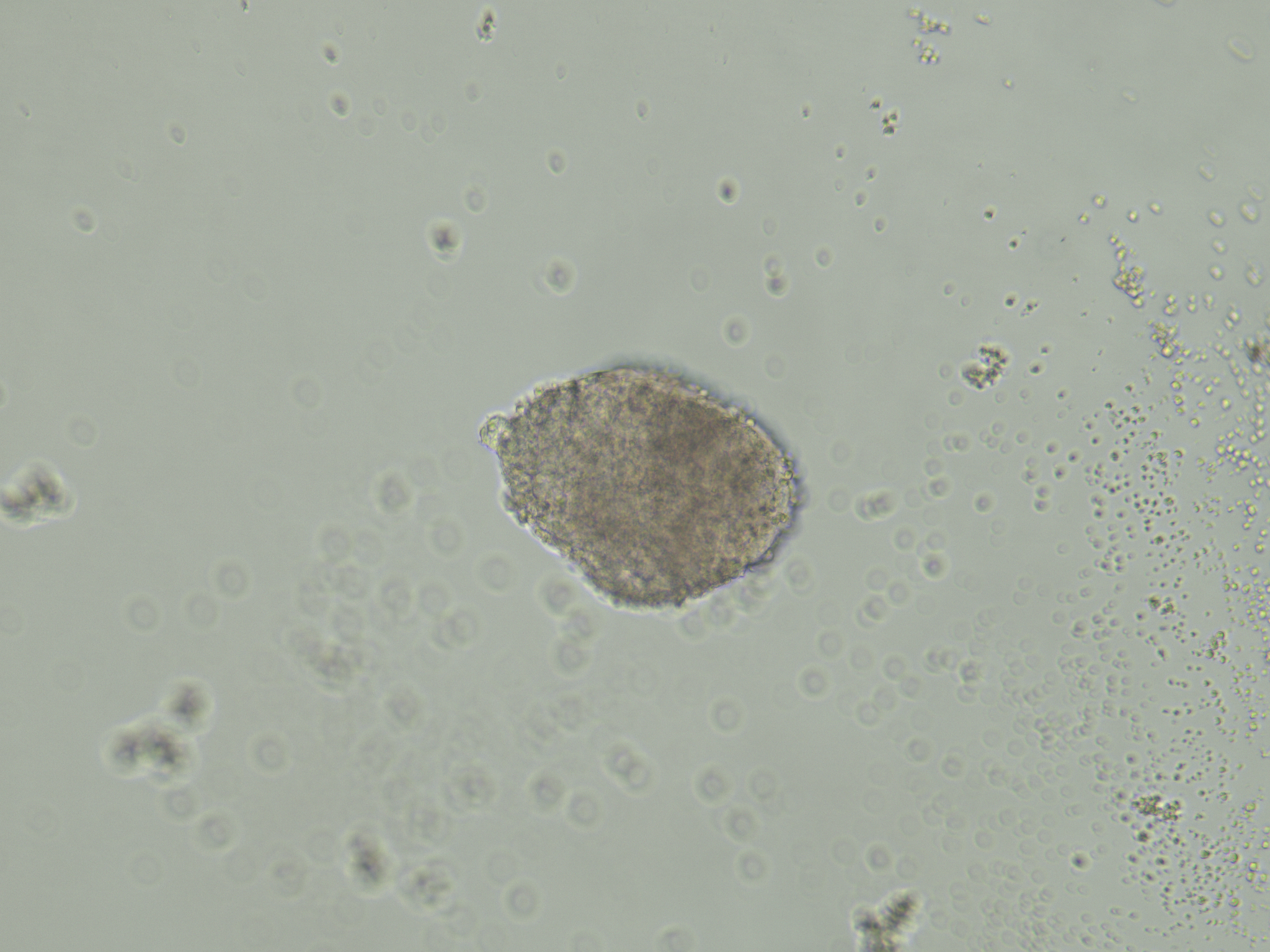

Supplement: Supplementary file 10 — Source data Fig. 8 [file 44318_2025_558_MOESM10_ESM.zip › Figure 8/panel 8B/NT+DKK-1_1.tiff]

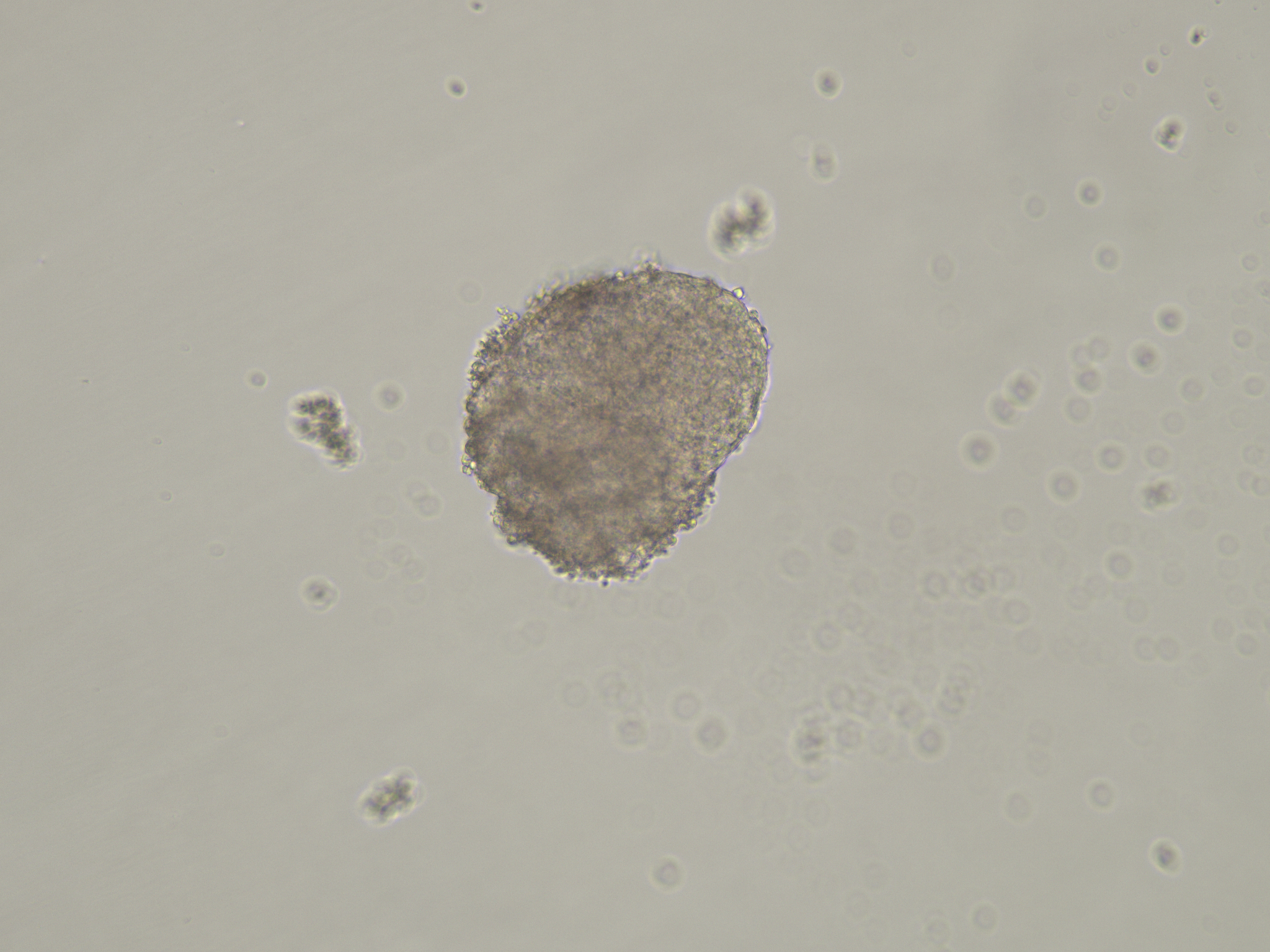

Supplement: Supplementary file 10 — Source data Fig. 8 [file 44318_2025_558_MOESM10_ESM.zip › Figure 8/panel 8B/KD1.tiff]

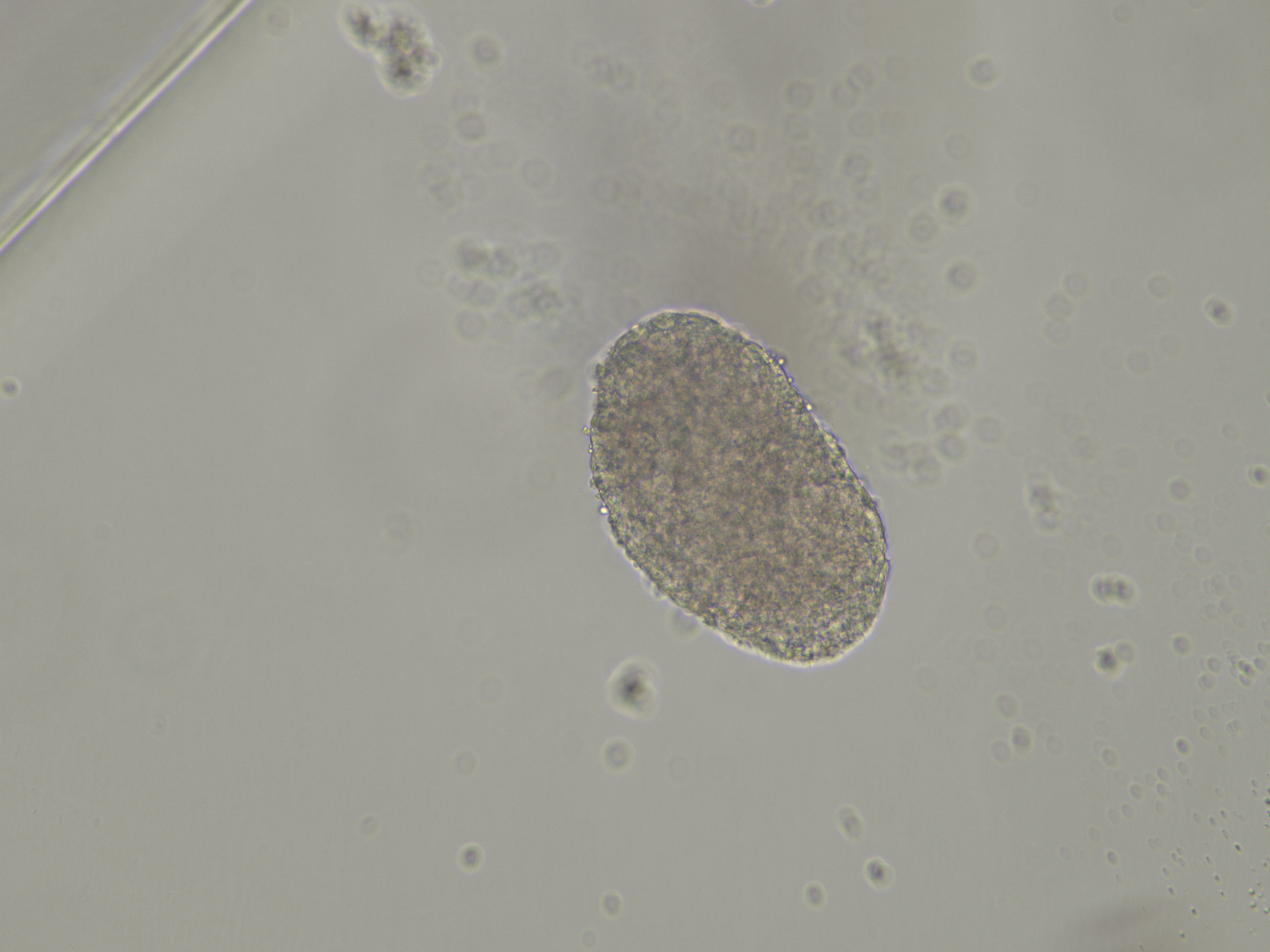

Supplement: Supplementary file 10 — Source data Fig. 8 [file 44318_2025_558_MOESM10_ESM.zip › Figure 8/panel 8B/NT+DKK1_2.tiff]

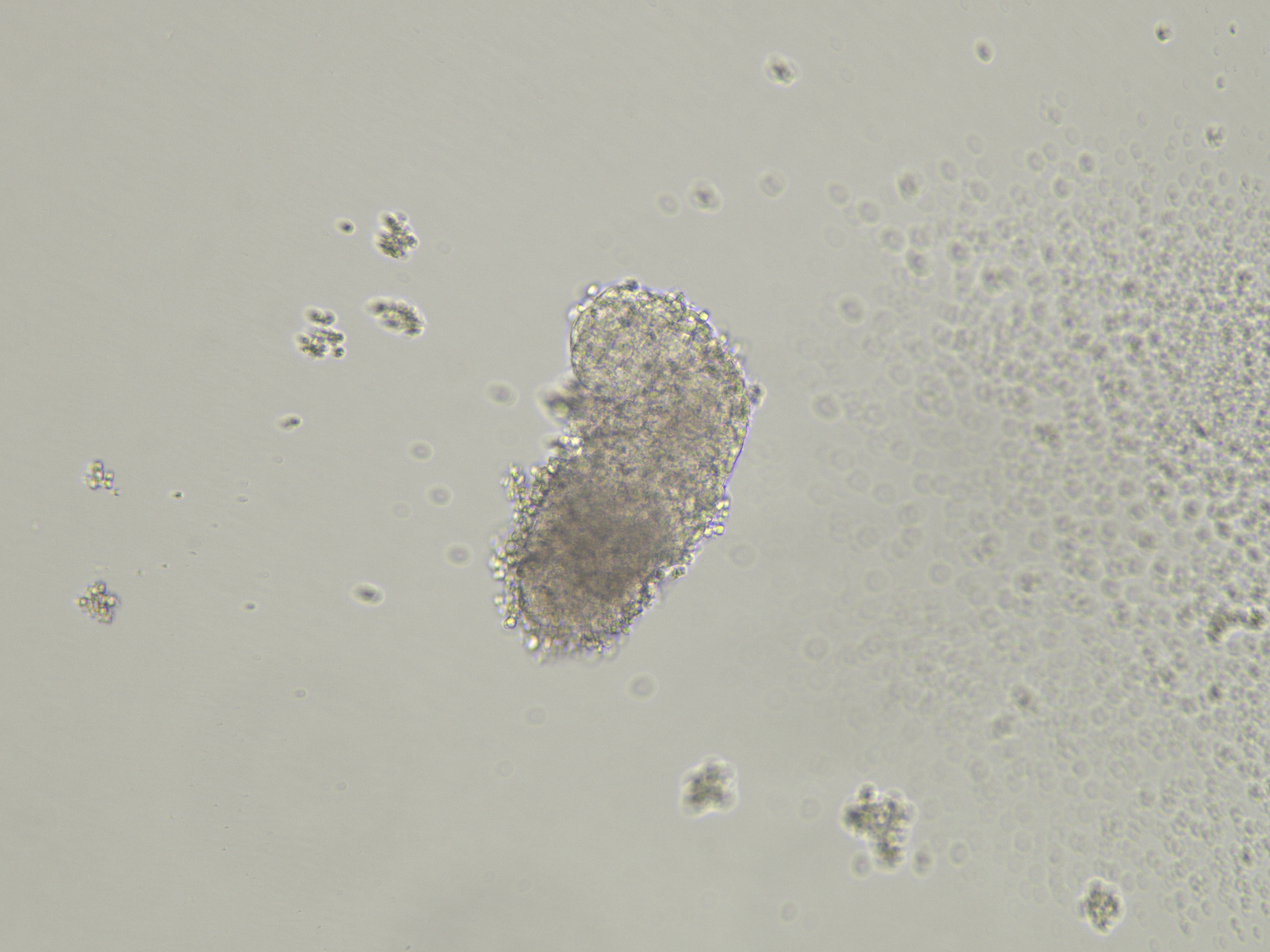

Supplement: Supplementary file 10 — Source data Fig. 8 [file 44318_2025_558_MOESM10_ESM.zip › Figure 8/panel 8B/NT_1.tiff]

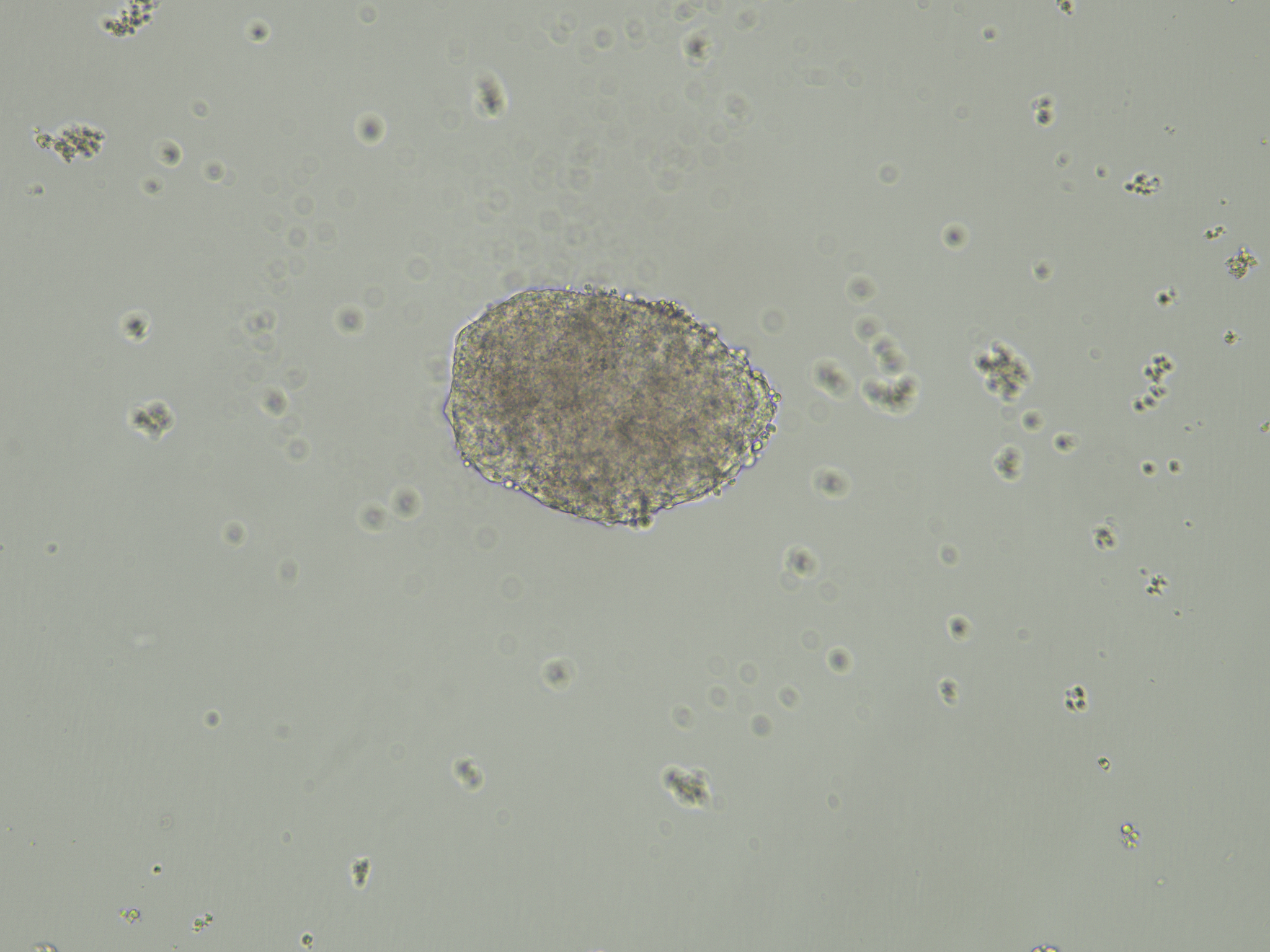

Supplement: Supplementary file 10 — Source data Fig. 8 [file 44318_2025_558_MOESM10_ESM.zip › Figure 8/panel 8B/KD-1_2.tiff]

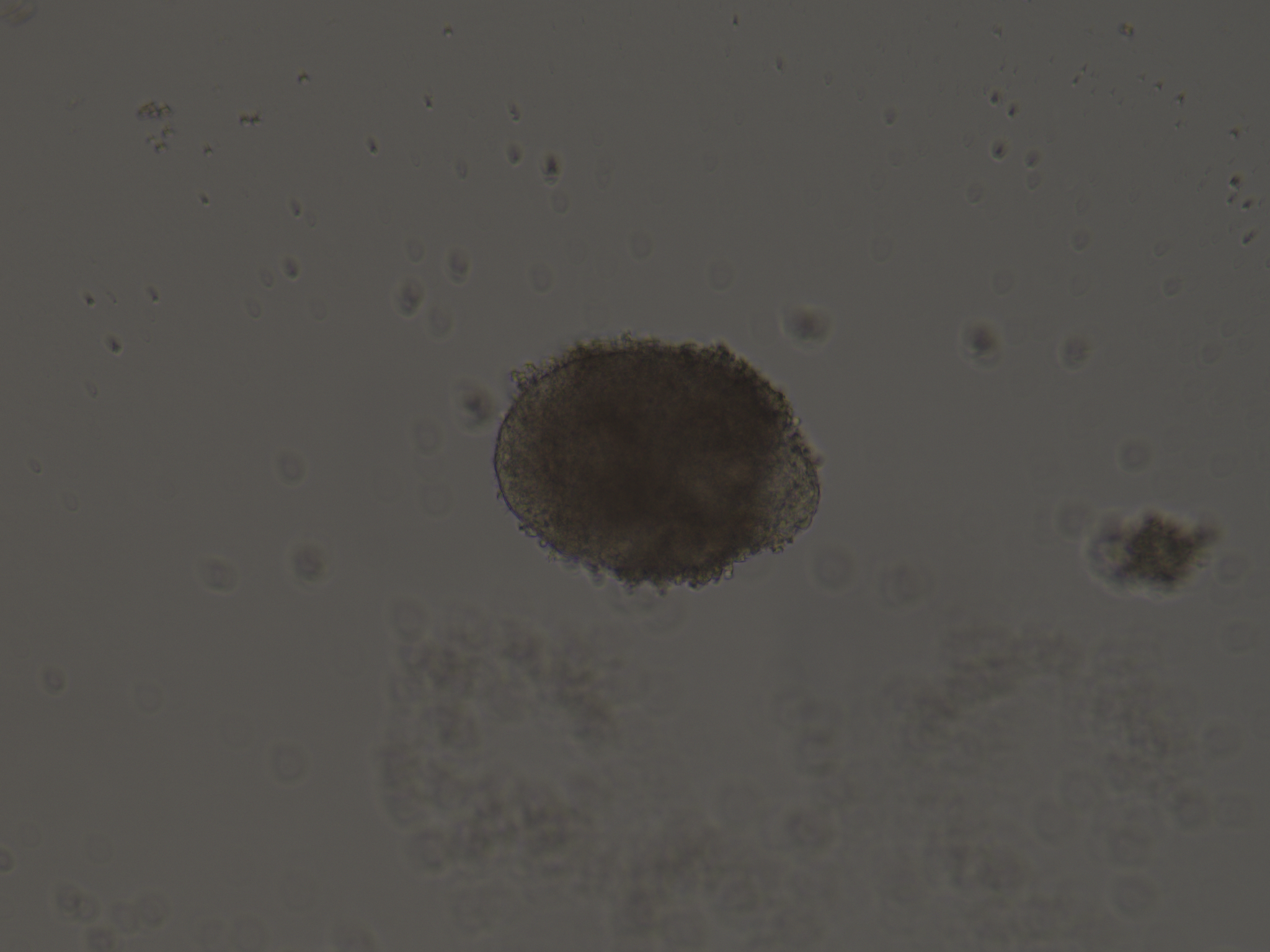

Supplement: Supplementary file 10 — Source data Fig. 8 [file 44318_2025_558_MOESM10_ESM.zip › Figure 8/panel 8F/KD-2.tiff]

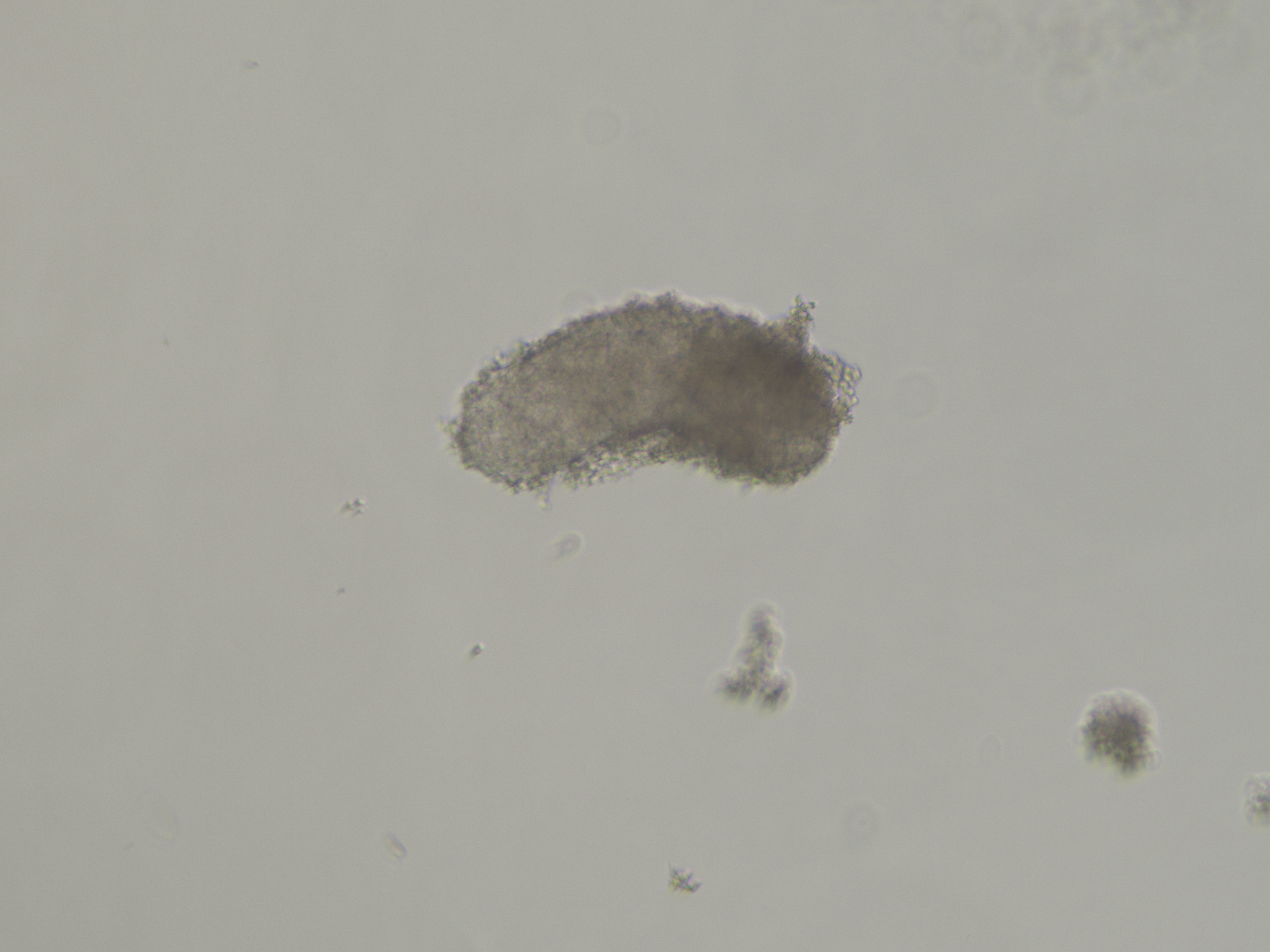

Supplement: Supplementary file 10 — Source data Fig. 8 [file 44318_2025_558_MOESM10_ESM.zip › Figure 8/panel 8F/KD-1_0.20uM.tiff]

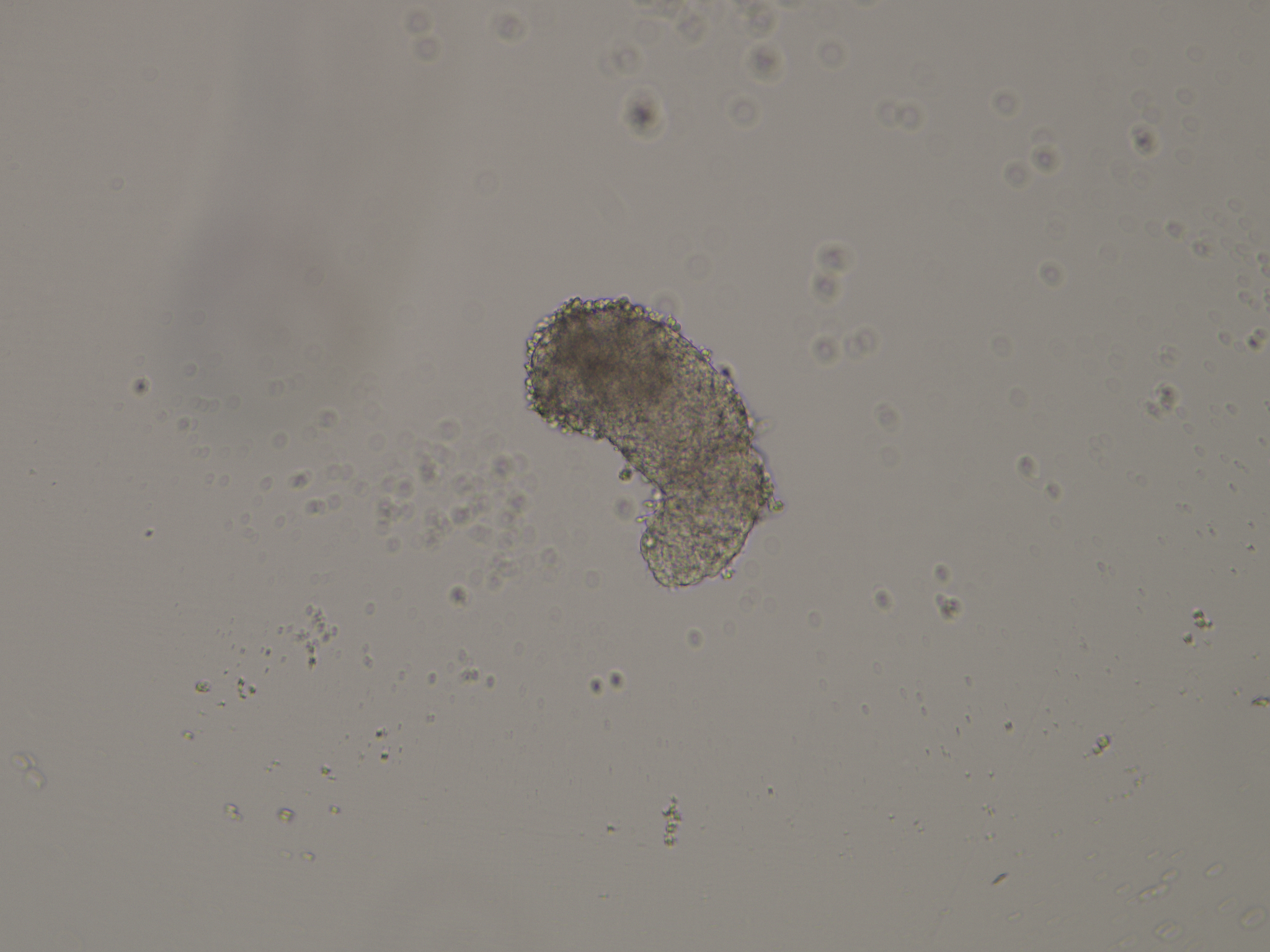

Supplement: Supplementary file 10 — Source data Fig. 8 [file 44318_2025_558_MOESM10_ESM.zip › Figure 8/panel 8F/NT_2.tiff]

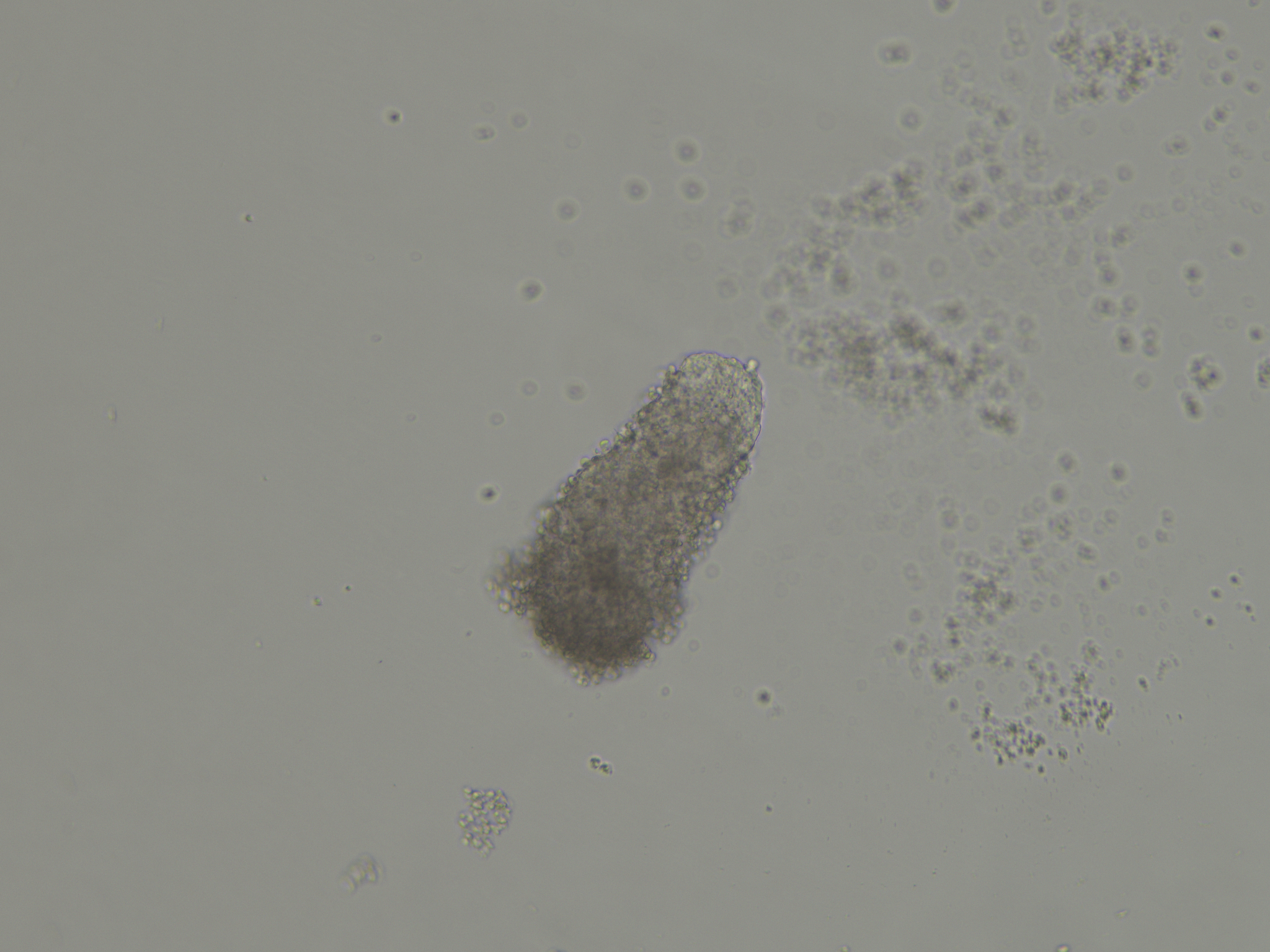

Supplement: Supplementary file 10 — Source data Fig. 8 [file 44318_2025_558_MOESM10_ESM.zip › Figure 8/panel 8F/KD-2_0.20uM.tiff]

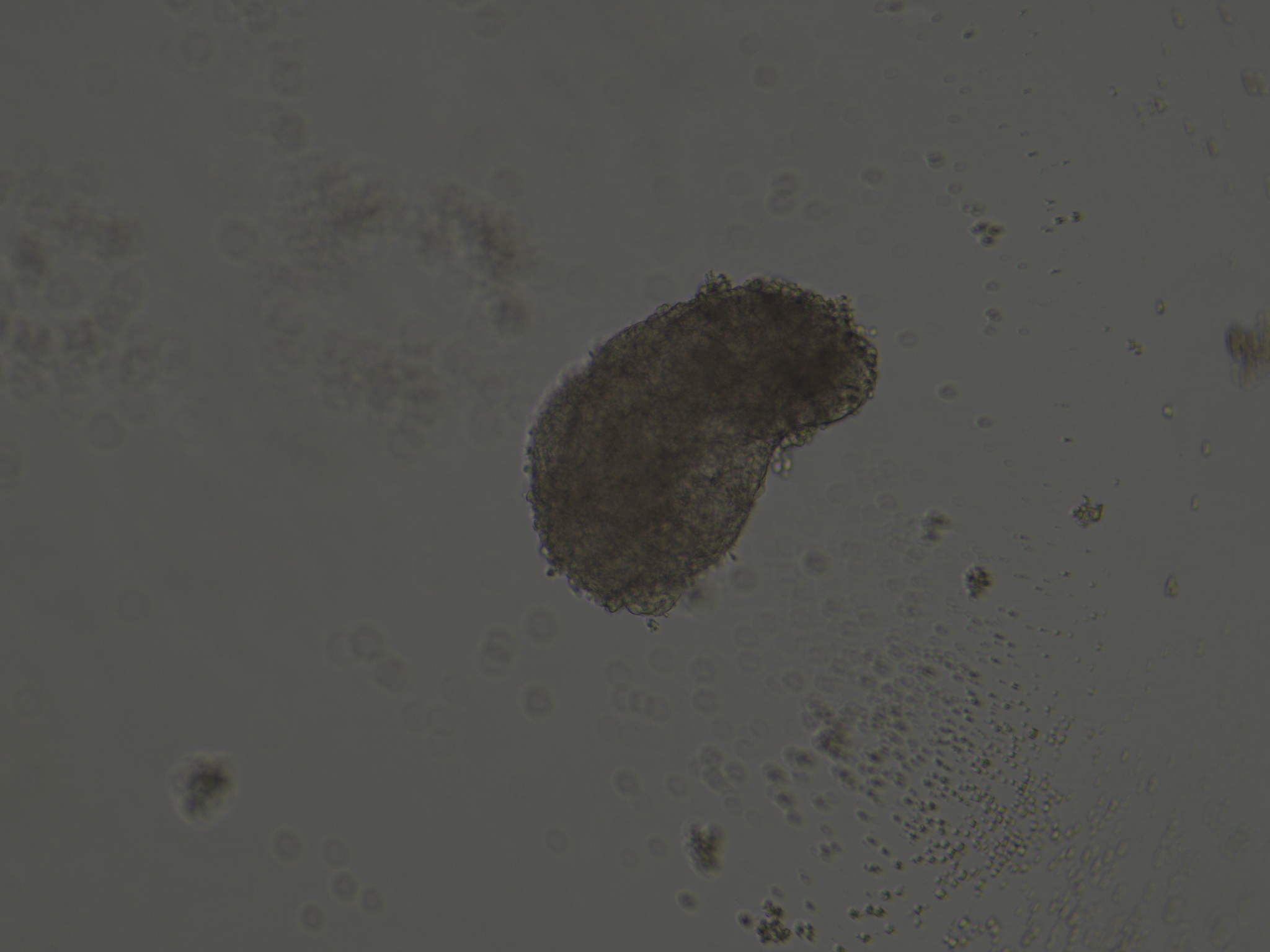

Supplement: Supplementary file 10 — Source data Fig. 8 [file 44318_2025_558_MOESM10_ESM.zip › Figure 8/panel 8F/KD-1_0.1uM.tiff]

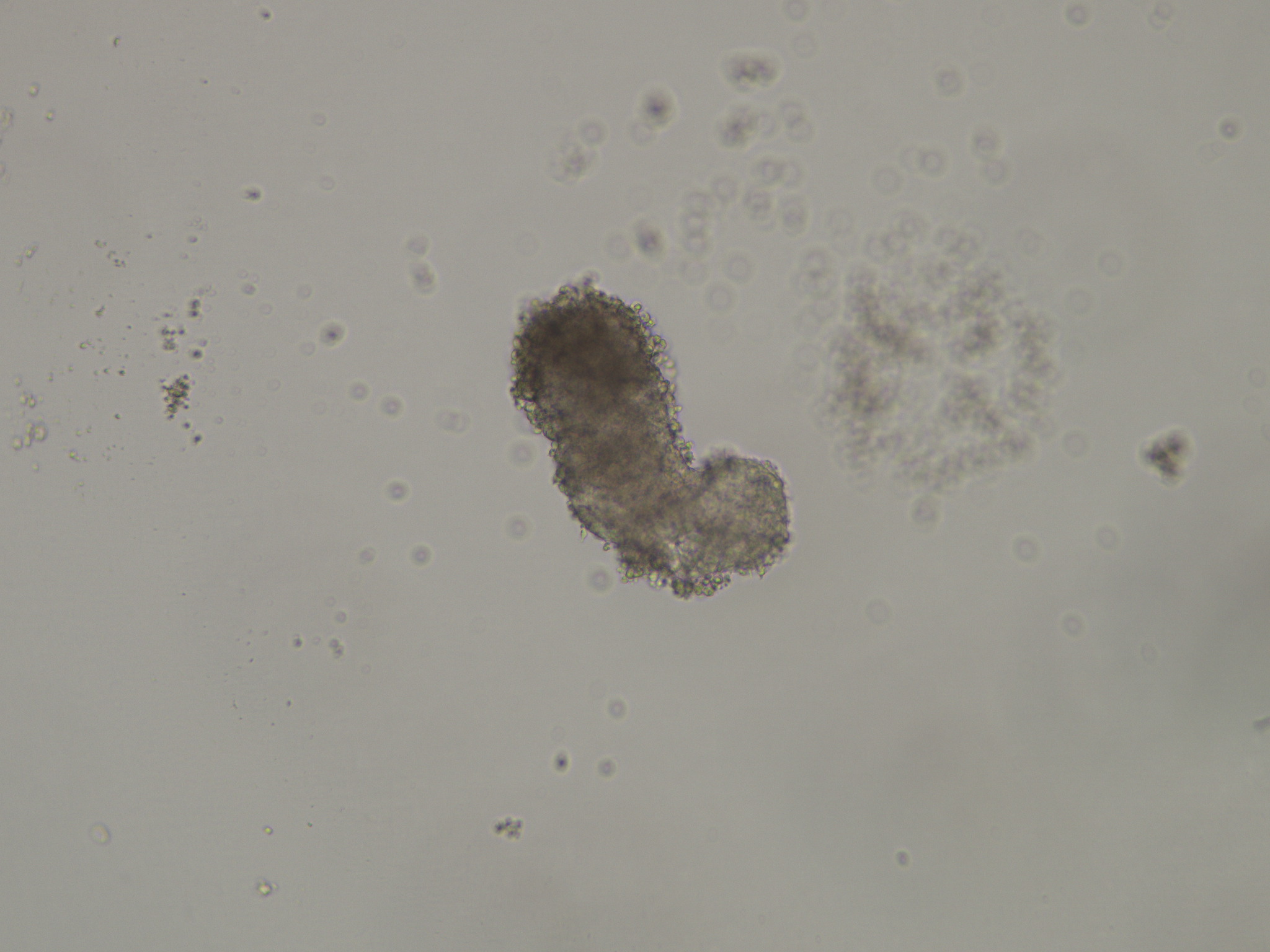

Supplement: Supplementary file 10 — Source data Fig. 8 [file 44318_2025_558_MOESM10_ESM.zip › Figure 8/panel 8F/NT_1.tiff]

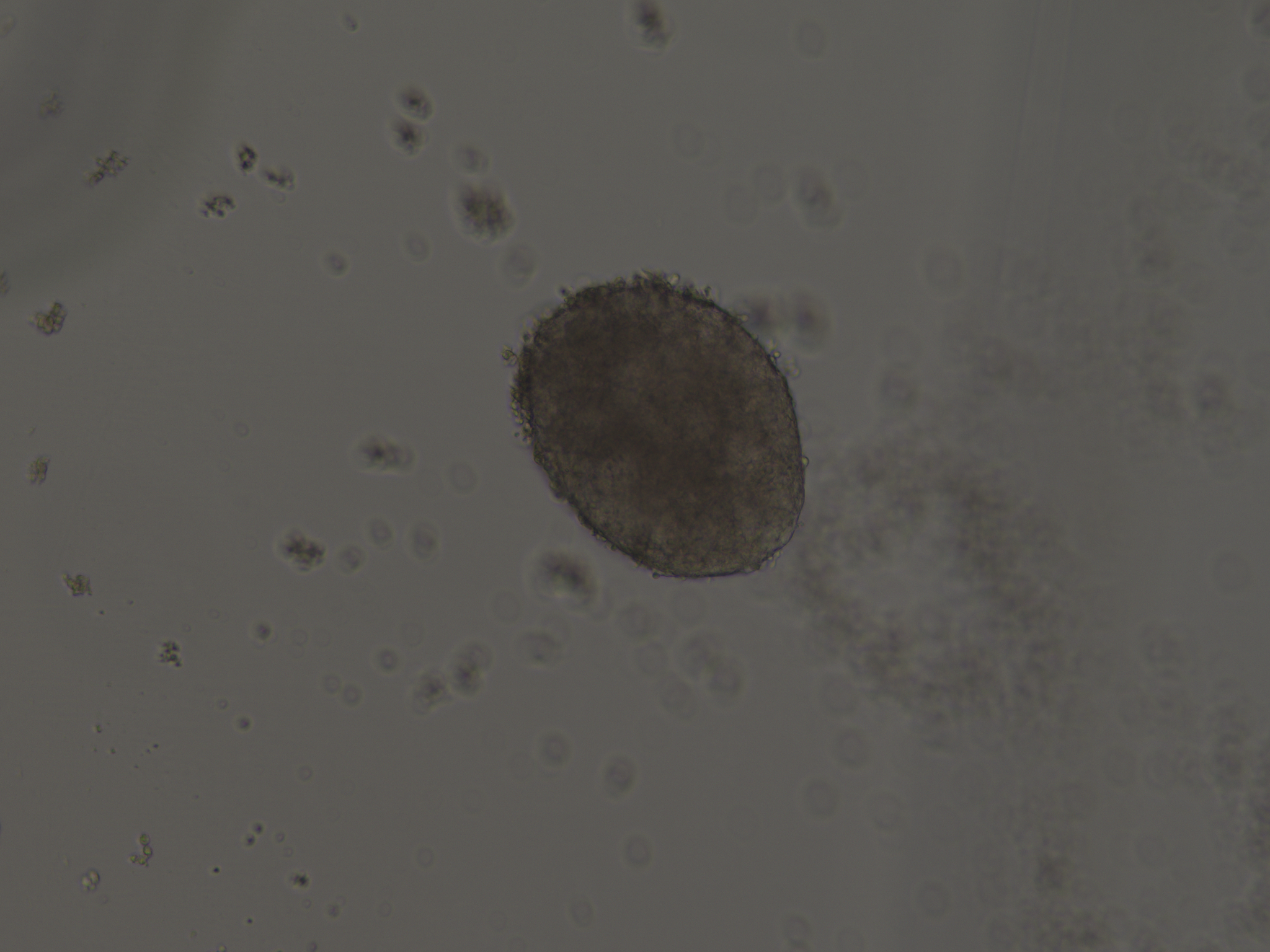

Supplement: Supplementary file 10 — Source data Fig. 8 [file 44318_2025_558_MOESM10_ESM.zip › Figure 8/panel 8F/KD-1.tiff]

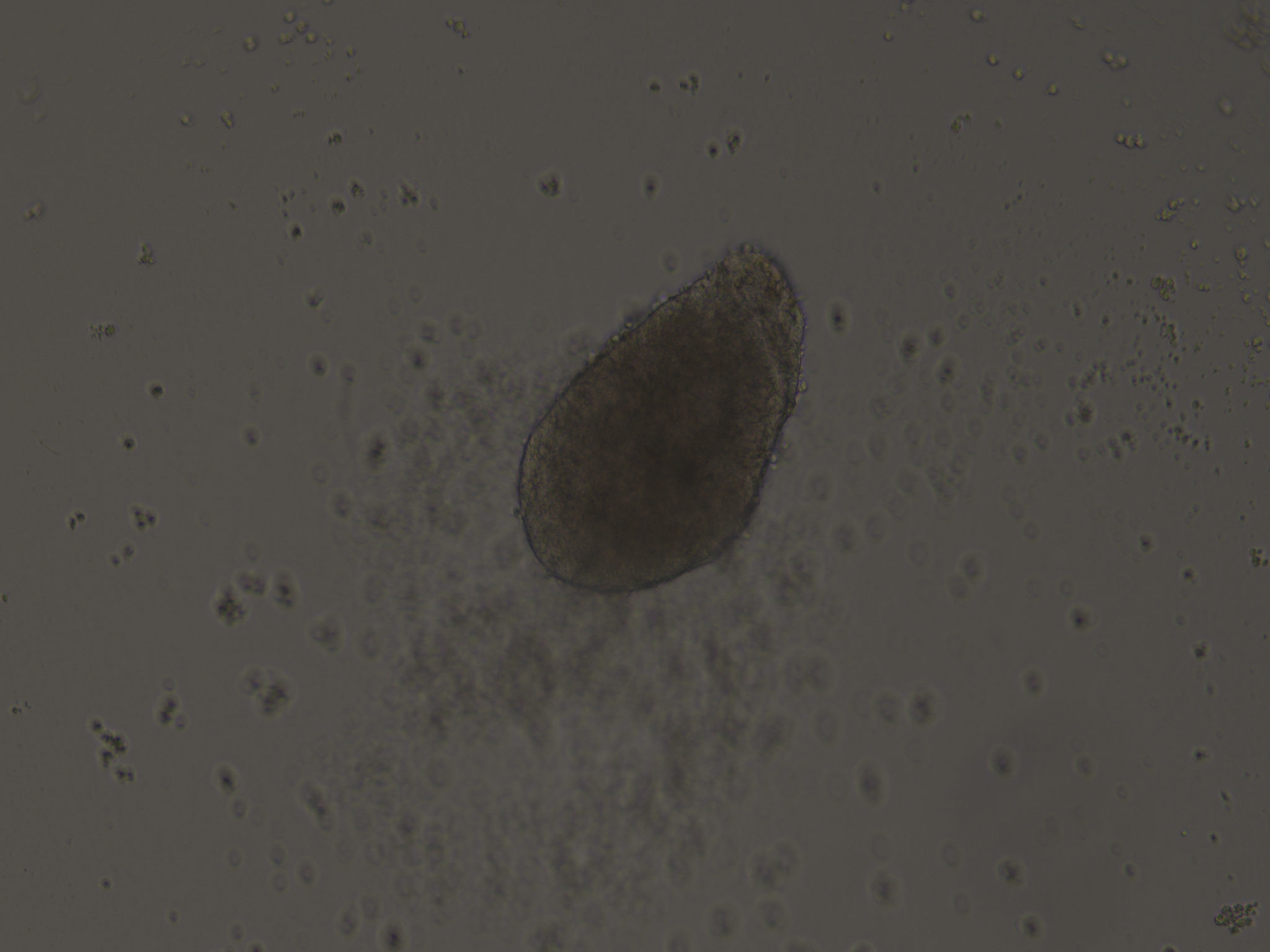

Supplement: Supplementary file 10 — Source data Fig. 8 [file 44318_2025_558_MOESM10_ESM.zip › Figure 8/panel 8F/KD-2_0.1uM.tiff]

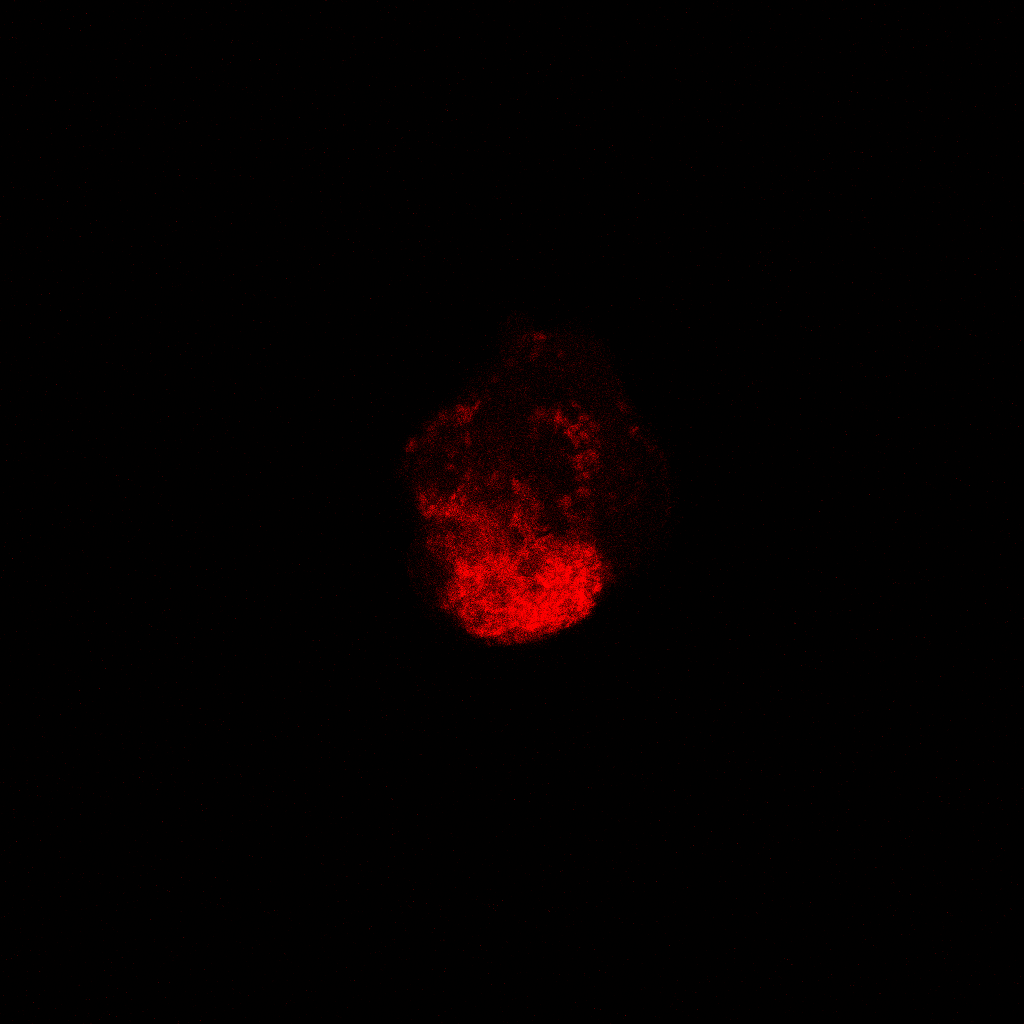

Supplement: Supplementary file 10 — Source data Fig. 8 [file 44318_2025_558_MOESM10_ESM.zip › Figure 8/panel 8C/NT+DKK1_Bra/image0097_image0097_RGB_Texas Red.tif]

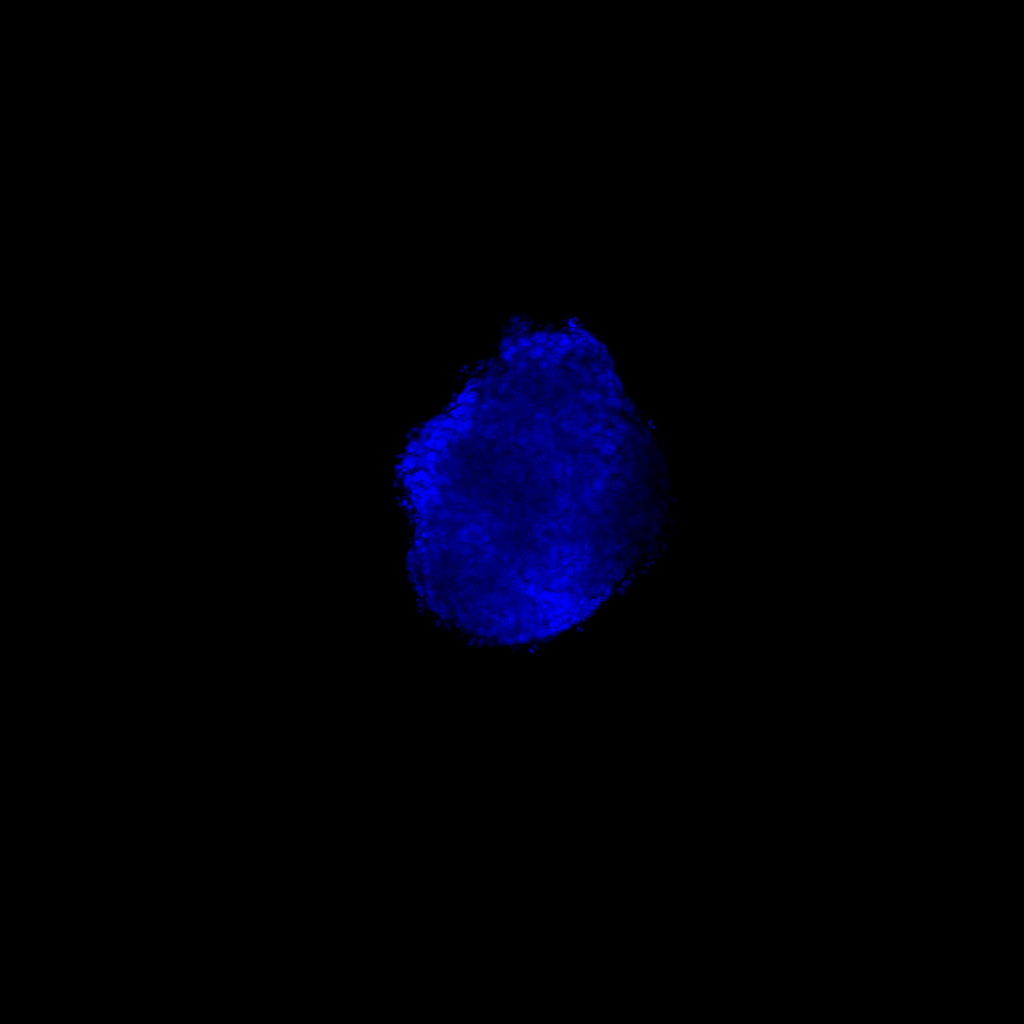

Supplement: Supplementary file 10 — Source data Fig. 8 [file 44318_2025_558_MOESM10_ESM.zip › Figure 8/panel 8C/NT+DKK1_Bra/image0097_image0097_RGB_DAPI.tif]

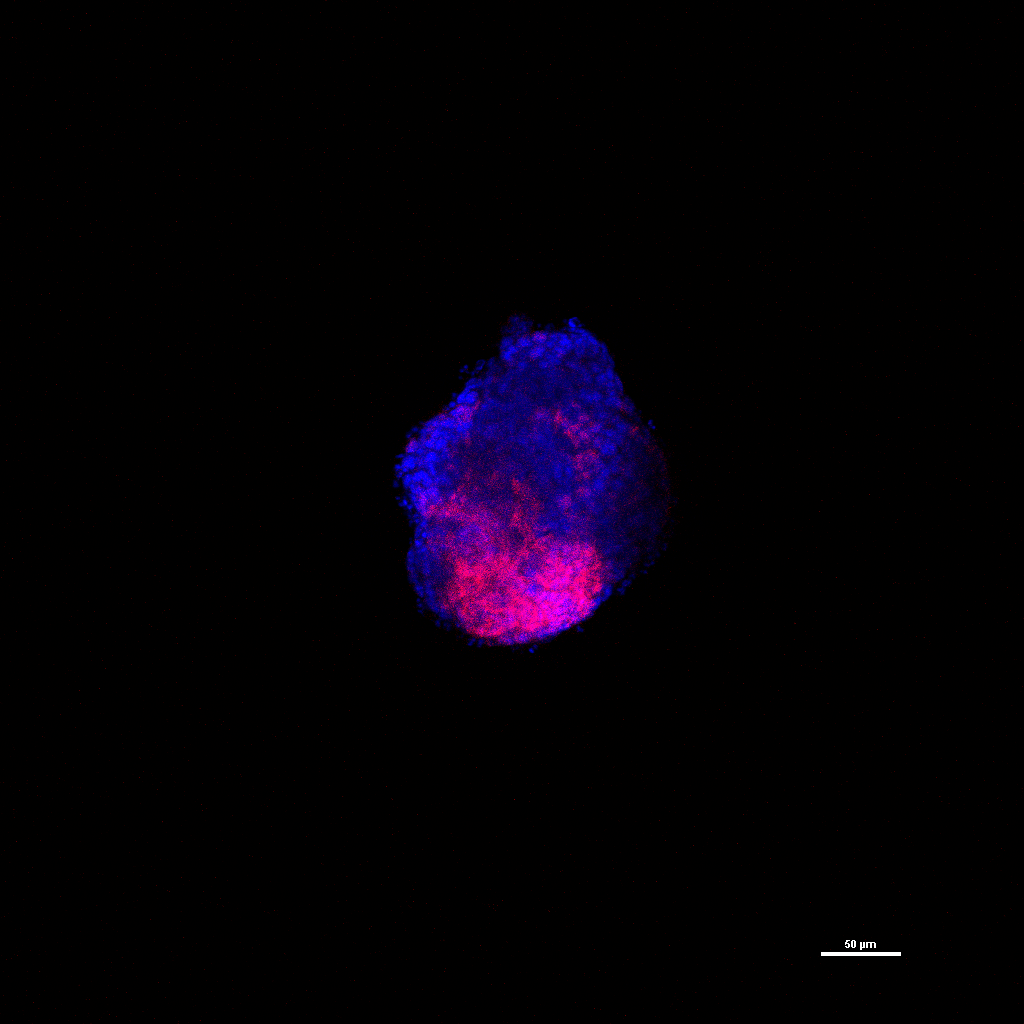

Supplement: Supplementary file 10 — Source data Fig. 8 [file 44318_2025_558_MOESM10_ESM.zip › Figure 8/panel 8C/NT+DKK1_Bra/image0097_image0097_RGB.tif]

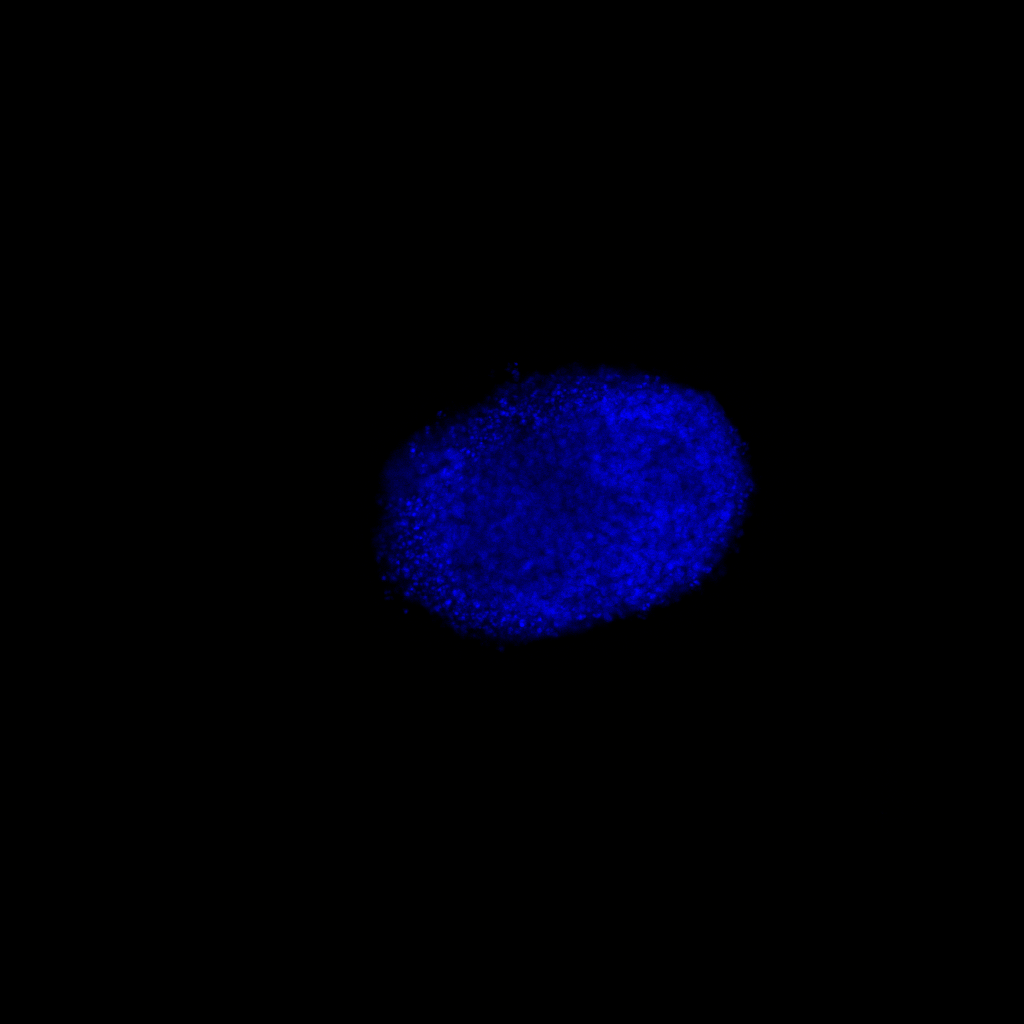

Supplement: Supplementary file 10 — Source data Fig. 8 [file 44318_2025_558_MOESM10_ESM.zip › Figure 8/panel 8C/KD1_Bra/image0145_image0145_RGB_DAPI.tif]

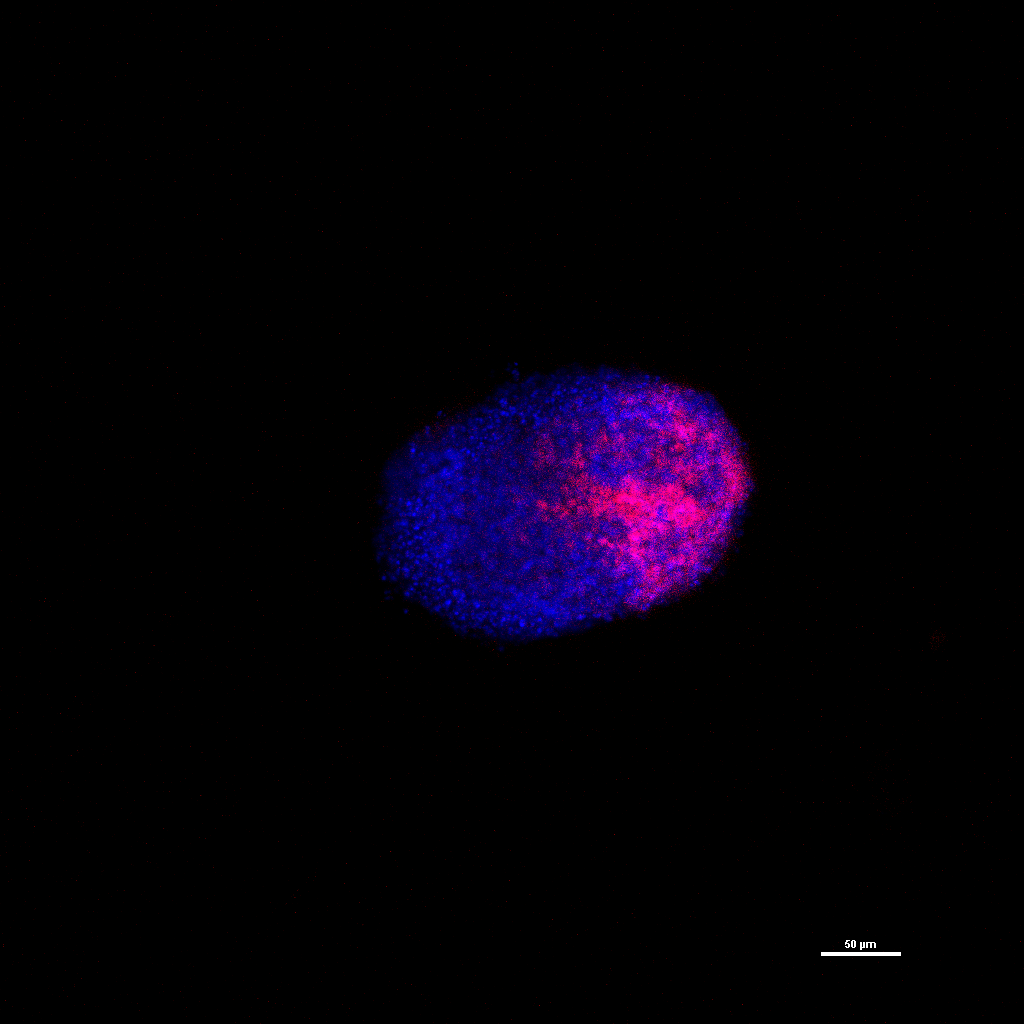

Supplement: Supplementary file 10 — Source data Fig. 8 [file 44318_2025_558_MOESM10_ESM.zip › Figure 8/panel 8C/KD1_Bra/image0145_image0145_RGB.tif]

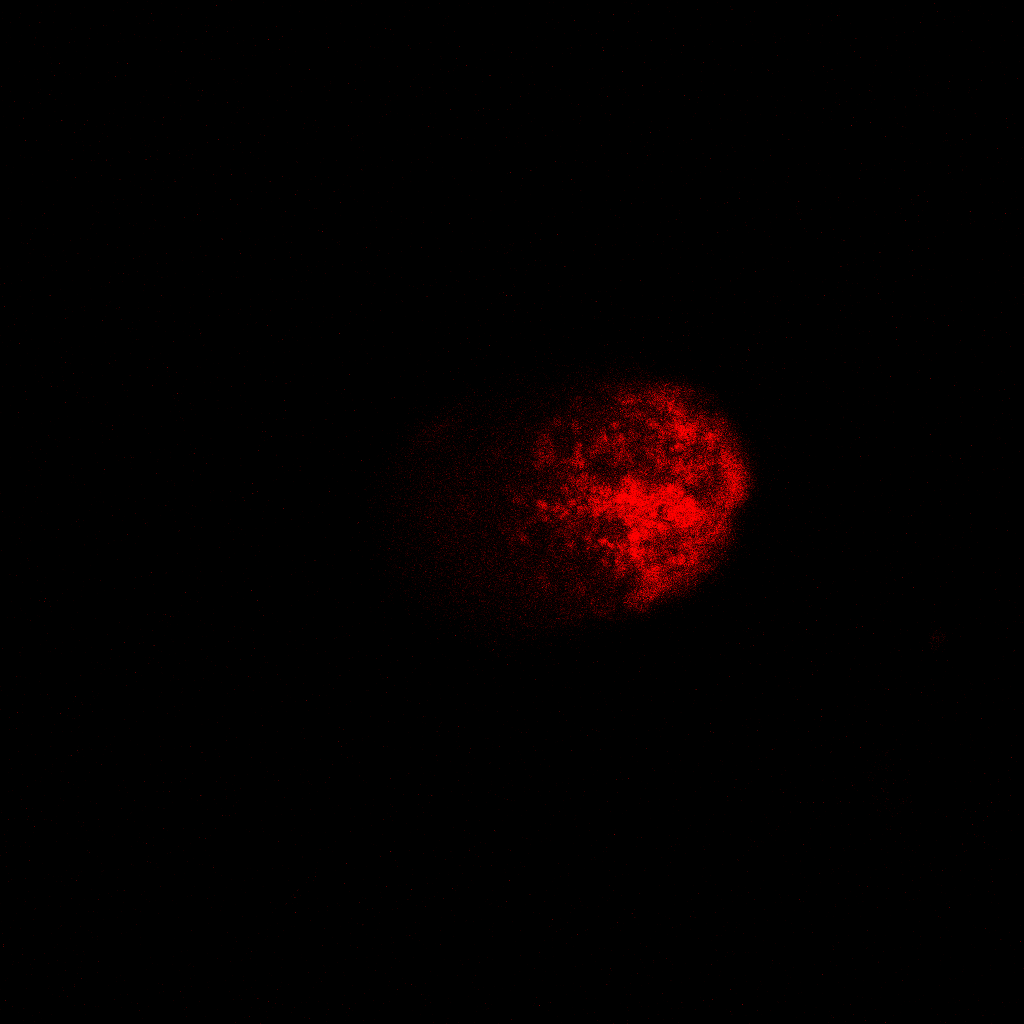

Supplement: Supplementary file 10 — Source data Fig. 8 [file 44318_2025_558_MOESM10_ESM.zip › Figure 8/panel 8C/KD1_Bra/image0145_image0145_RGB_Texas Red.tif]

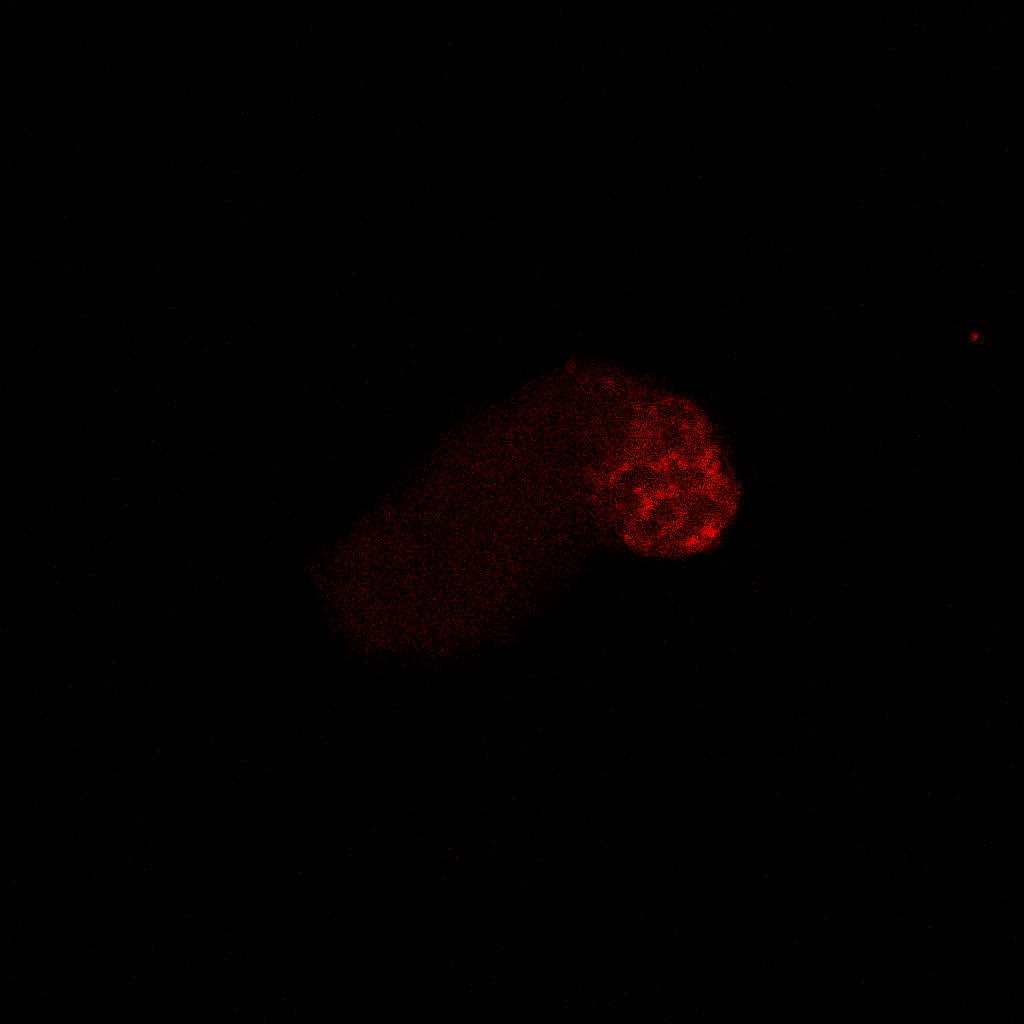

Supplement: Supplementary file 10 — Source data Fig. 8 [file 44318_2025_558_MOESM10_ESM.zip › Figure 8/panel 8C/NT_Bra/image0093_image0093_RGB_Texas Red.tif]

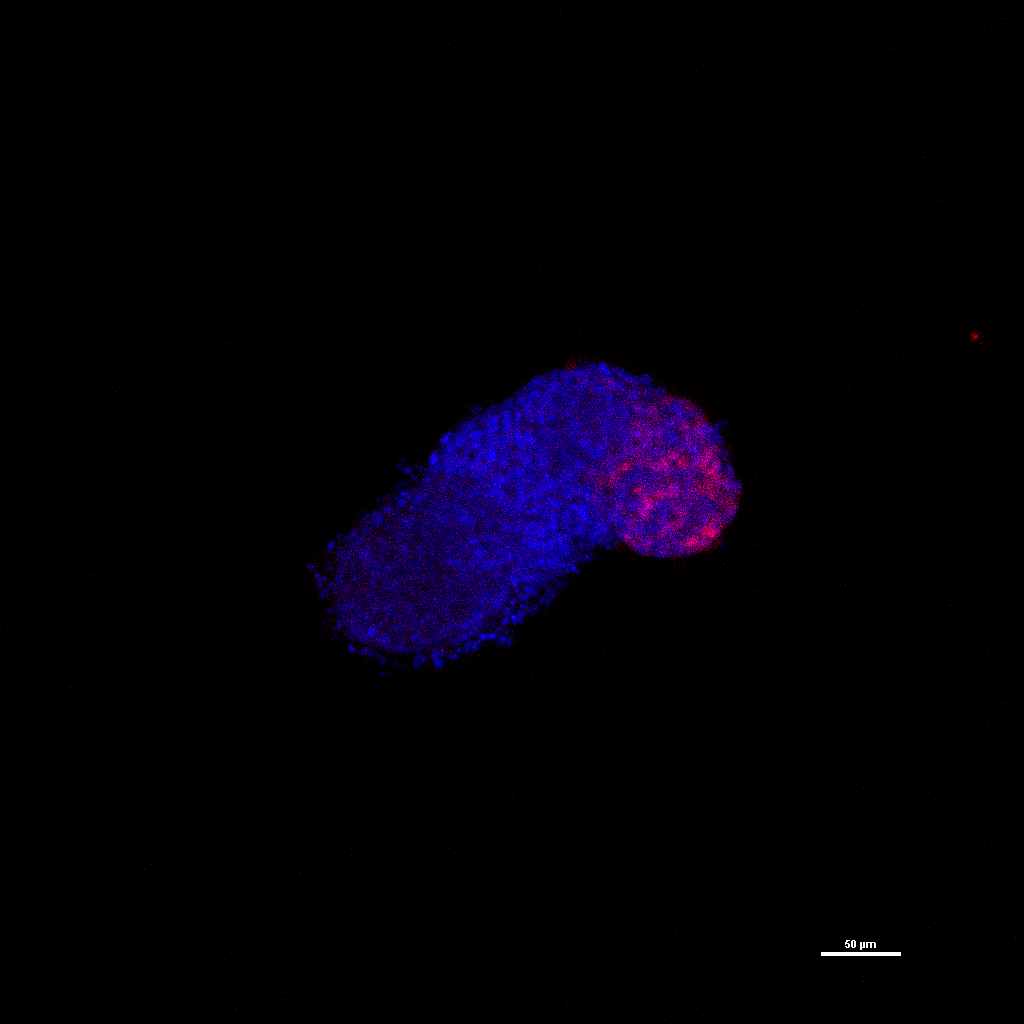

Supplement: Supplementary file 10 — Source data Fig. 8 [file 44318_2025_558_MOESM10_ESM.zip › Figure 8/panel 8C/NT_Bra/image0093_image0093_RGB.tif]

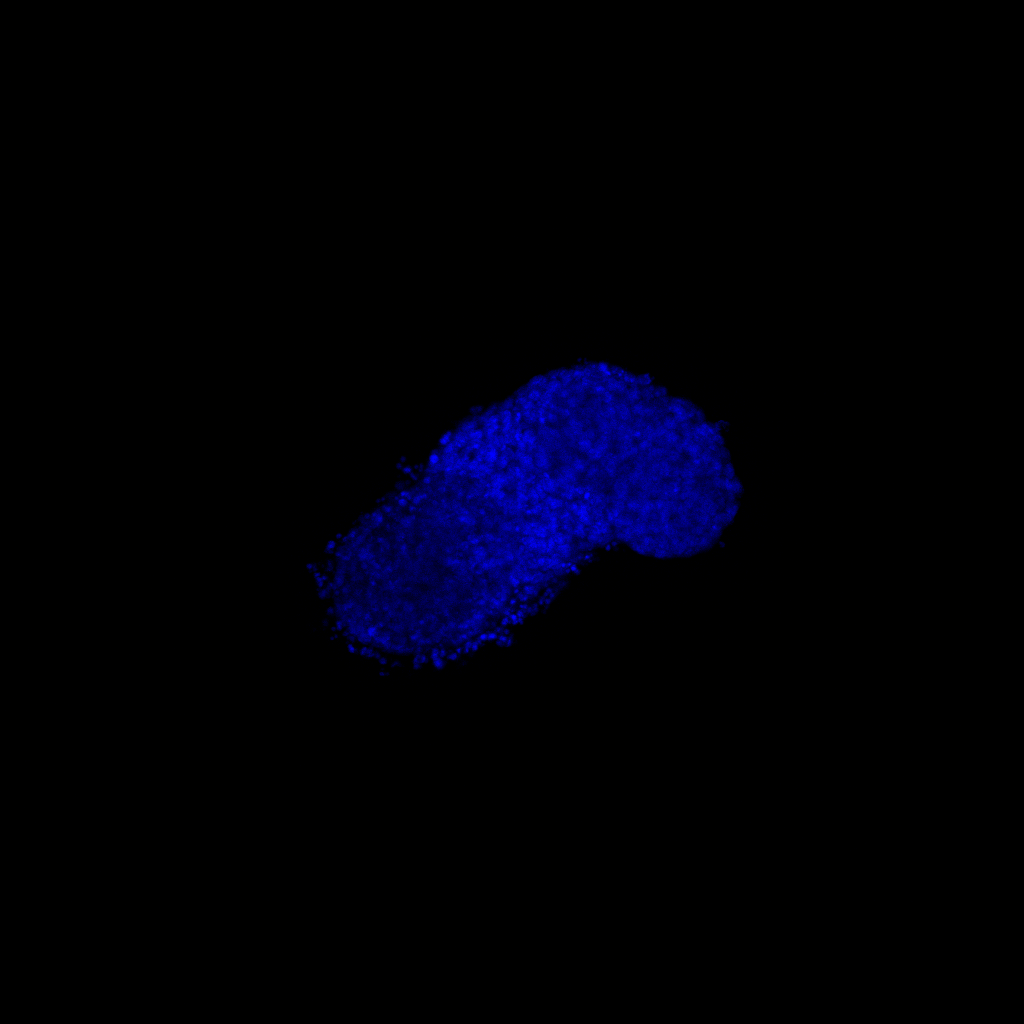

Supplement: Supplementary file 10 — Source data Fig. 8 [file 44318_2025_558_MOESM10_ESM.zip › Figure 8/panel 8C/NT_Bra/image0093_image0093_RGB_DAPI.tif]

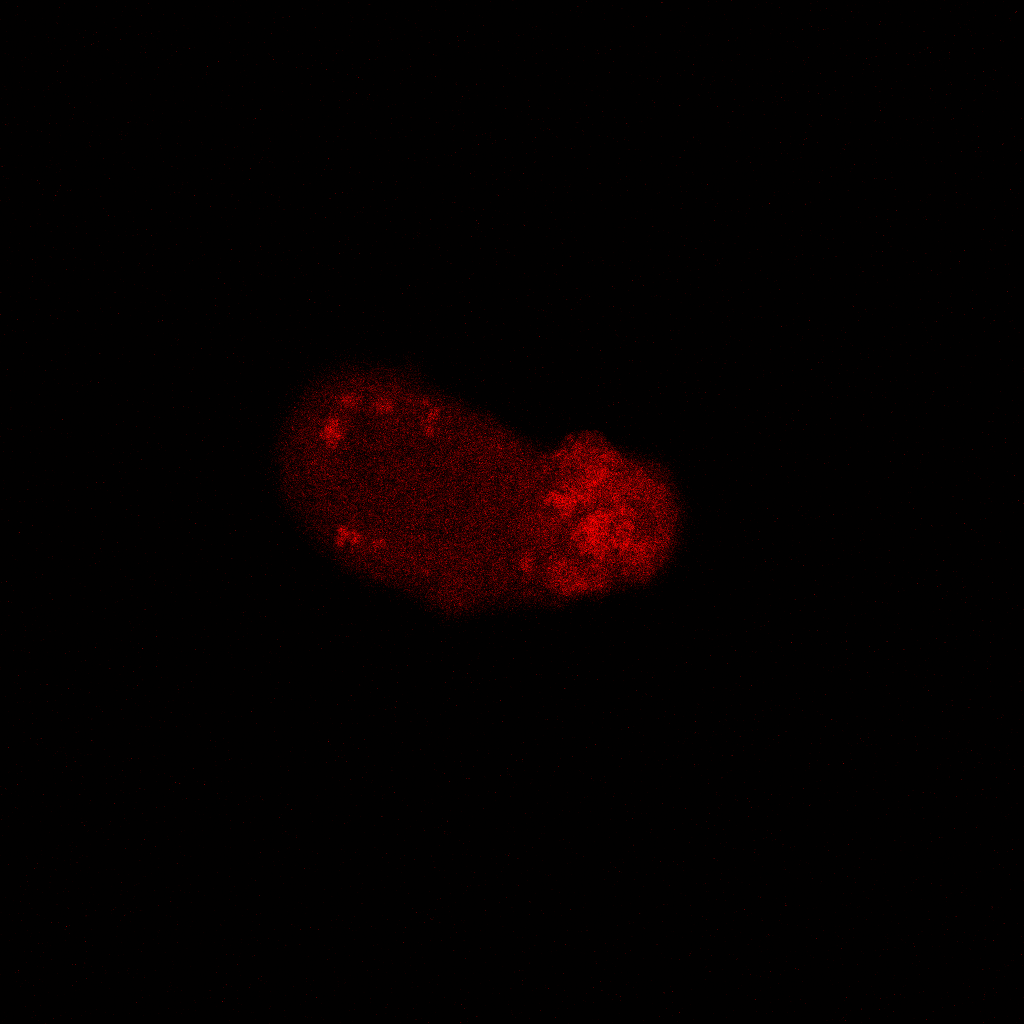

Supplement: Supplementary file 10 — Source data Fig. 8 [file 44318_2025_558_MOESM10_ESM.zip › Figure 8/panel 8C/NT_Oct4/image0083_image0083_RGB_Texas Red.tif]

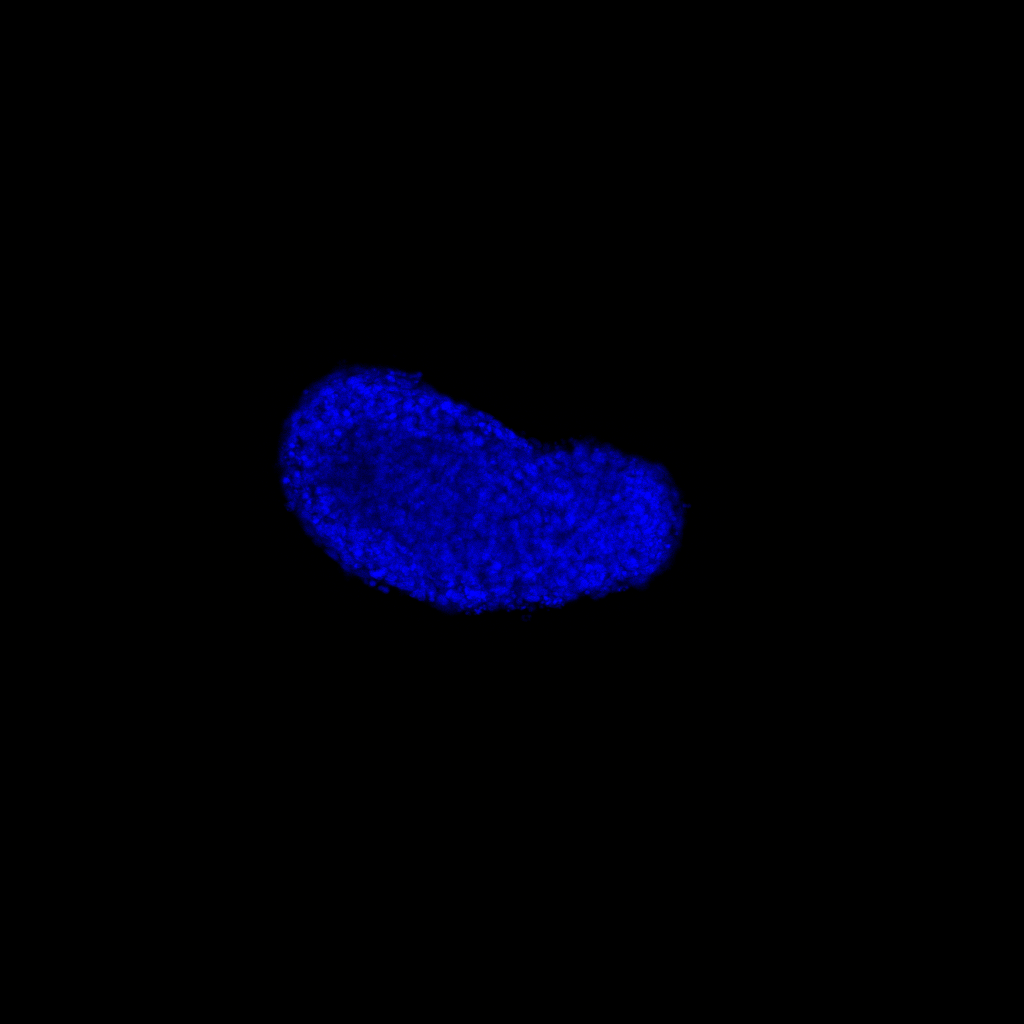

Supplement: Supplementary file 10 — Source data Fig. 8 [file 44318_2025_558_MOESM10_ESM.zip › Figure 8/panel 8C/NT_Oct4/image0083_image0083_RGB_DAPI.tif]

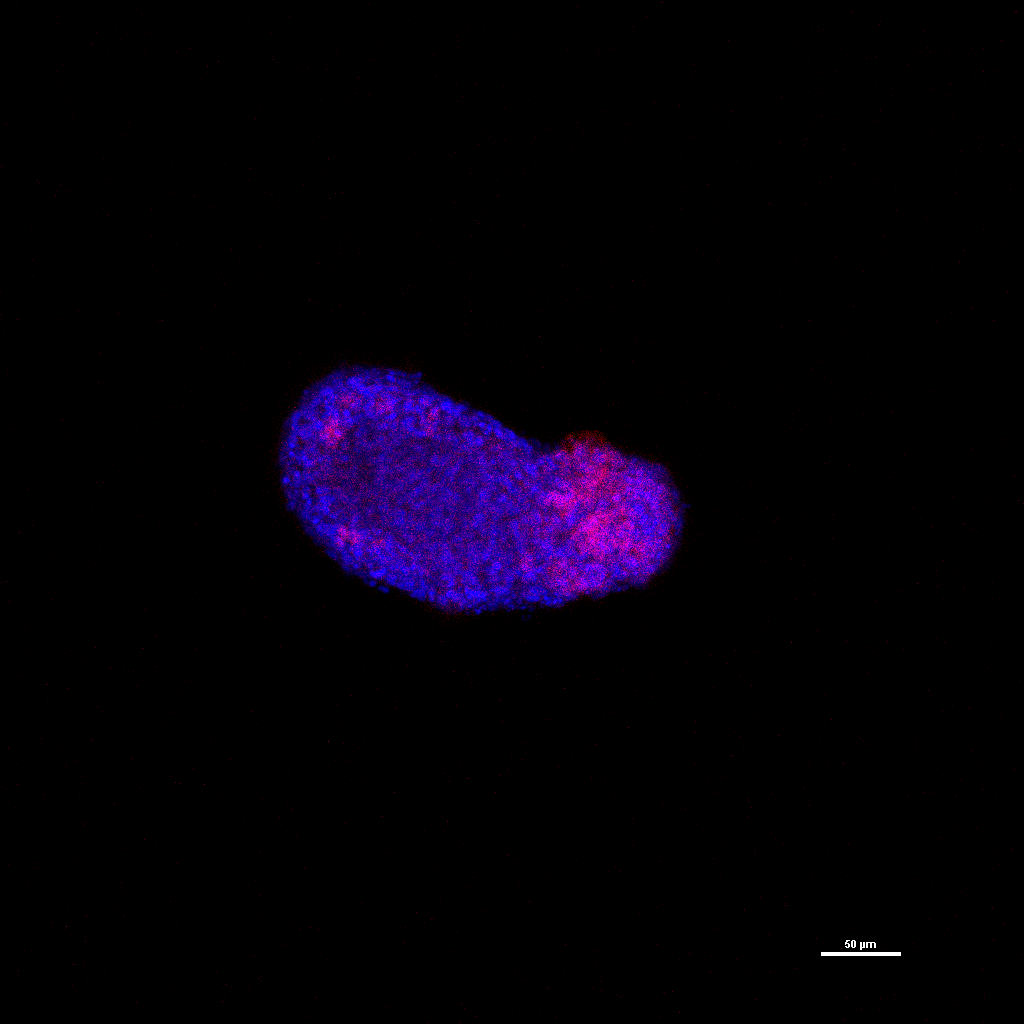

Supplement: Supplementary file 10 — Source data Fig. 8 [file 44318_2025_558_MOESM10_ESM.zip › Figure 8/panel 8C/NT_Oct4/image0083_image0083_RGB.tif]

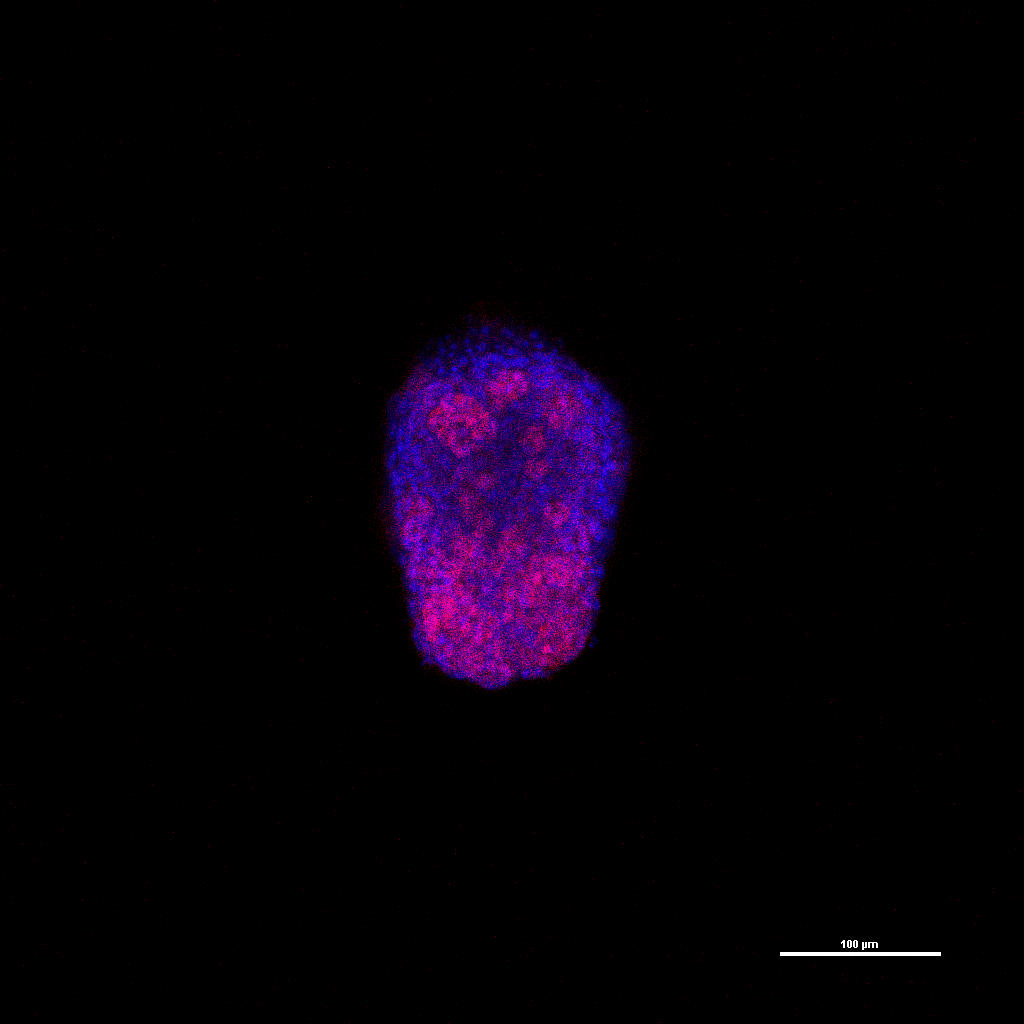

Supplement: Supplementary file 10 — Source data Fig. 8 [file 44318_2025_558_MOESM10_ESM.zip › Figure 8/panel 8C/KD1_Oct4/image0085_image0085_RGB.tif]

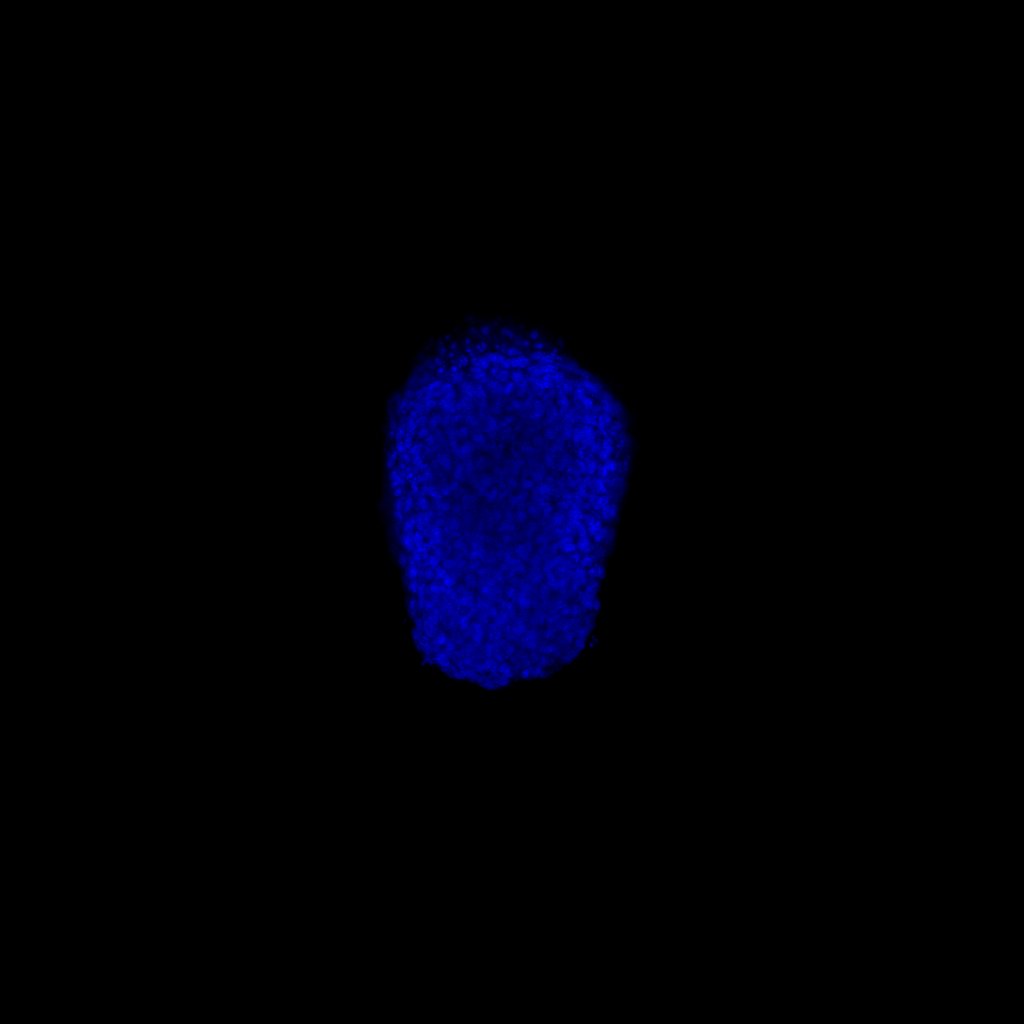

Supplement: Supplementary file 10 — Source data Fig. 8 [file 44318_2025_558_MOESM10_ESM.zip › Figure 8/panel 8C/KD1_Oct4/image0085_image0085_RGB_DAPI.tif]

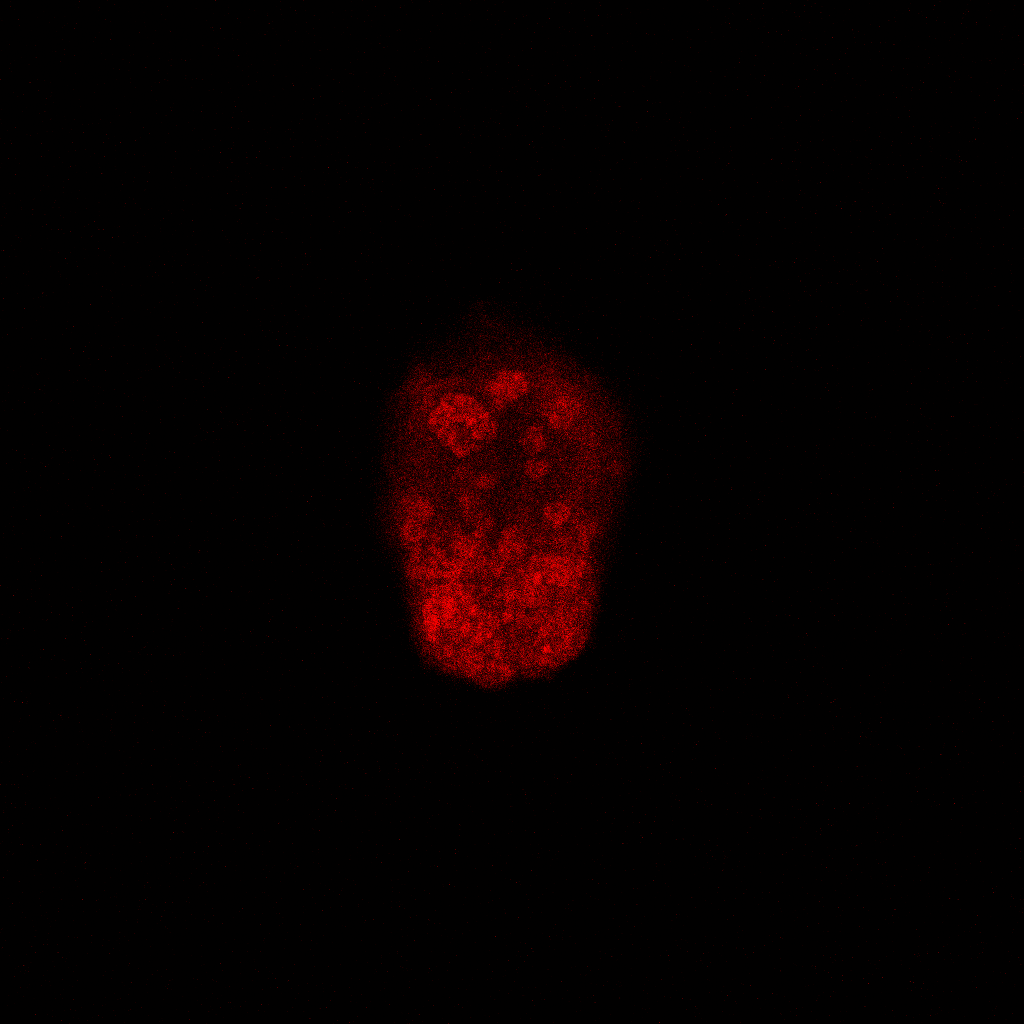

Supplement: Supplementary file 10 — Source data Fig. 8 [file 44318_2025_558_MOESM10_ESM.zip › Figure 8/panel 8C/KD1_Oct4/image0085_image0085_RGB_Texas Red.tif]

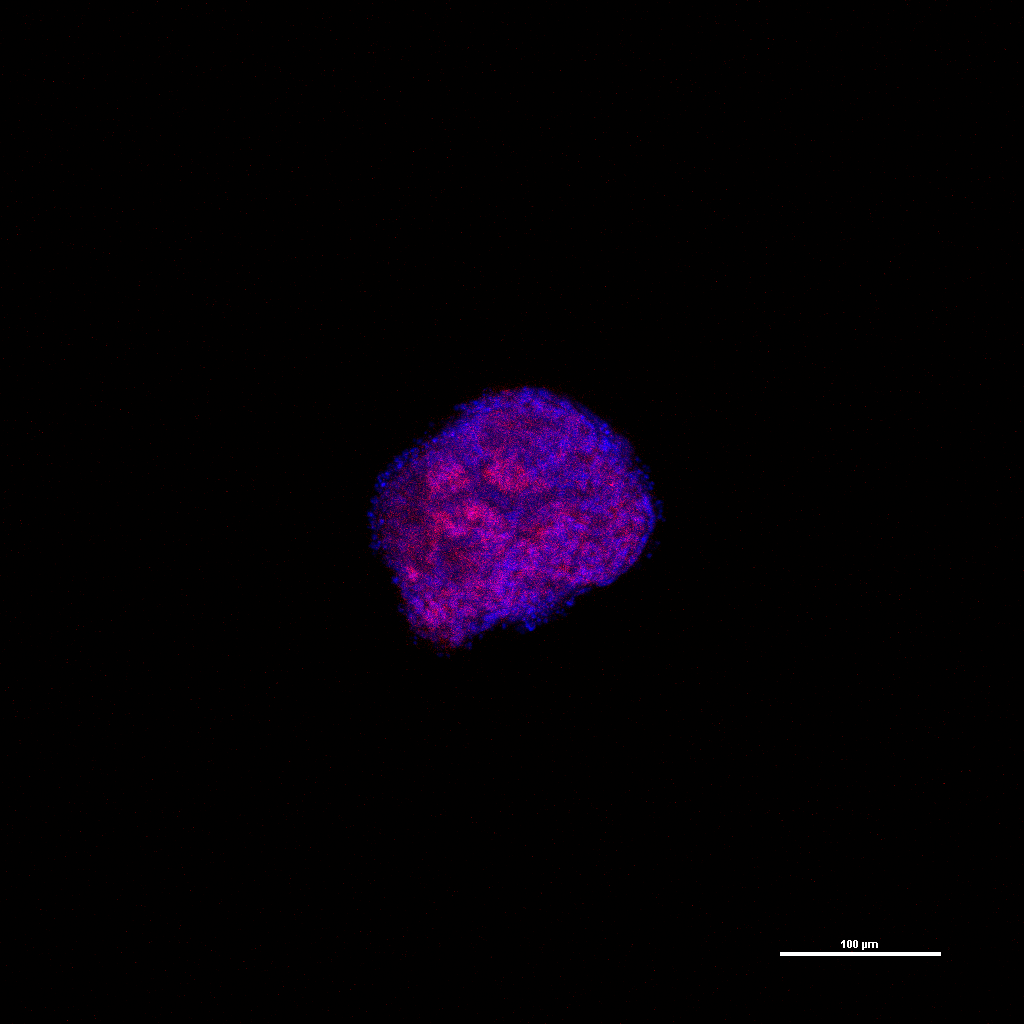

Supplement: Supplementary file 10 — Source data Fig. 8 [file 44318_2025_558_MOESM10_ESM.zip › Figure 8/panel 8C/NT+DKK1_Oct4/image0088_image0088_RGB.tif]

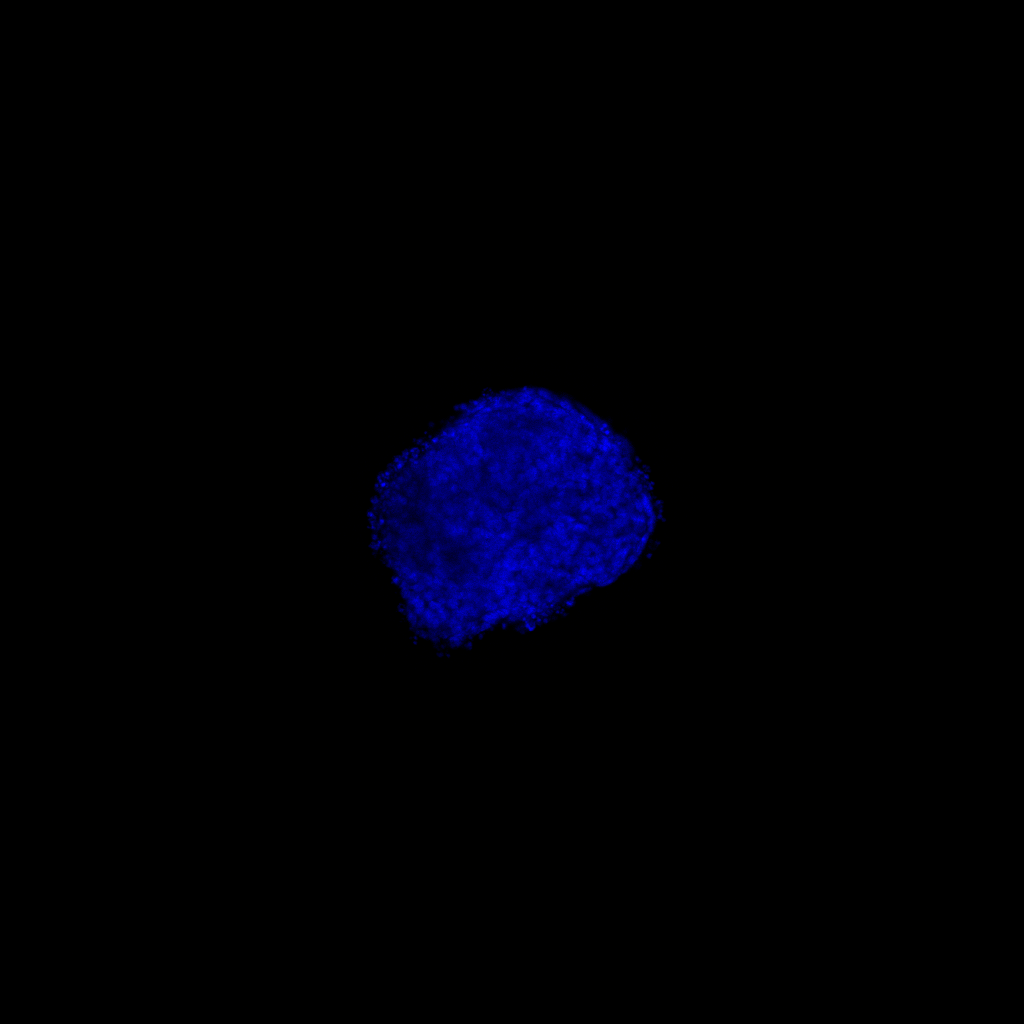

Supplement: Supplementary file 10 — Source data Fig. 8 [file 44318_2025_558_MOESM10_ESM.zip › Figure 8/panel 8C/NT+DKK1_Oct4/image0088_image0088_RGB_DAPI.tif]

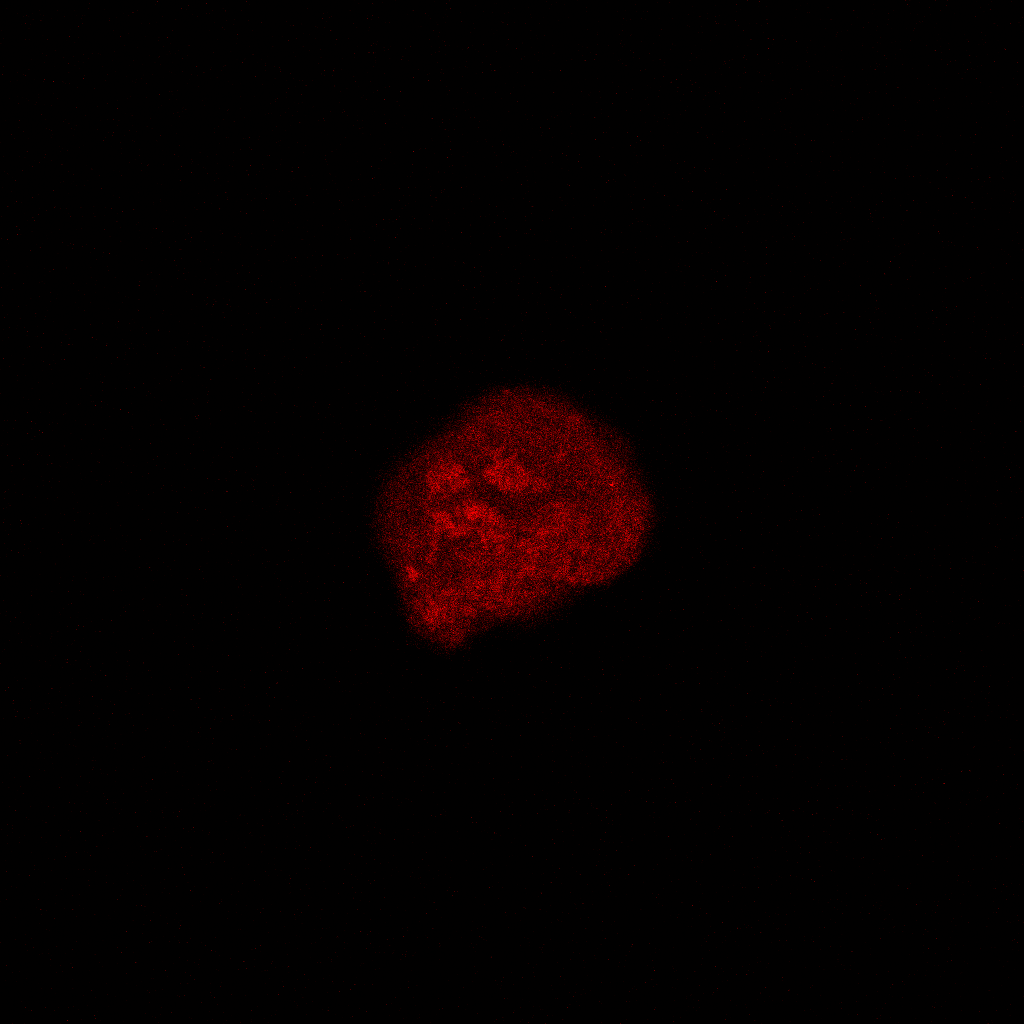

Supplement: Supplementary file 10 — Source data Fig. 8 [file 44318_2025_558_MOESM10_ESM.zip › Figure 8/panel 8C/NT+DKK1_Oct4/image0088_image0088_RGB_Texas Red.tif]

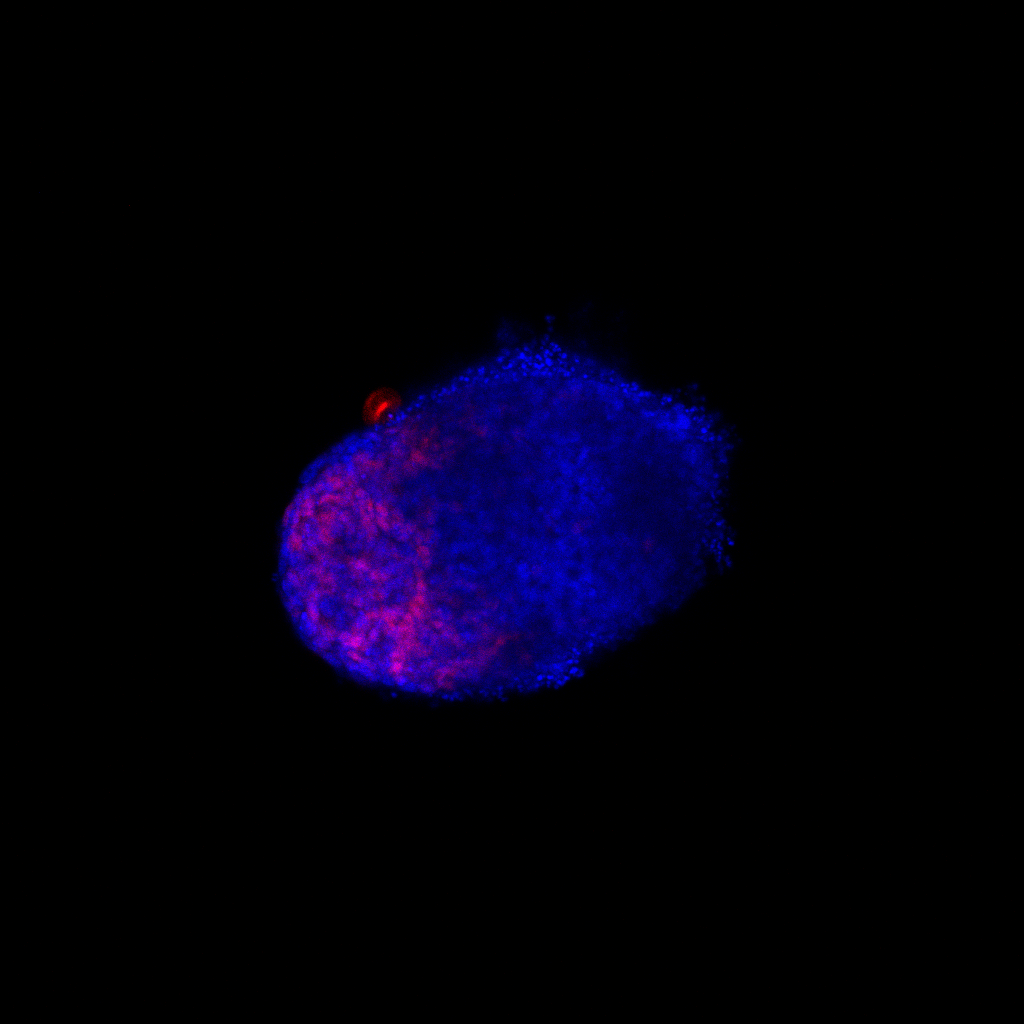

Supplement: Supplementary file 10 — Source data Fig. 8 [file 44318_2025_558_MOESM10_ESM.zip › Figure 8/panel 8G/KD-1_Bra/seq8685.tif]

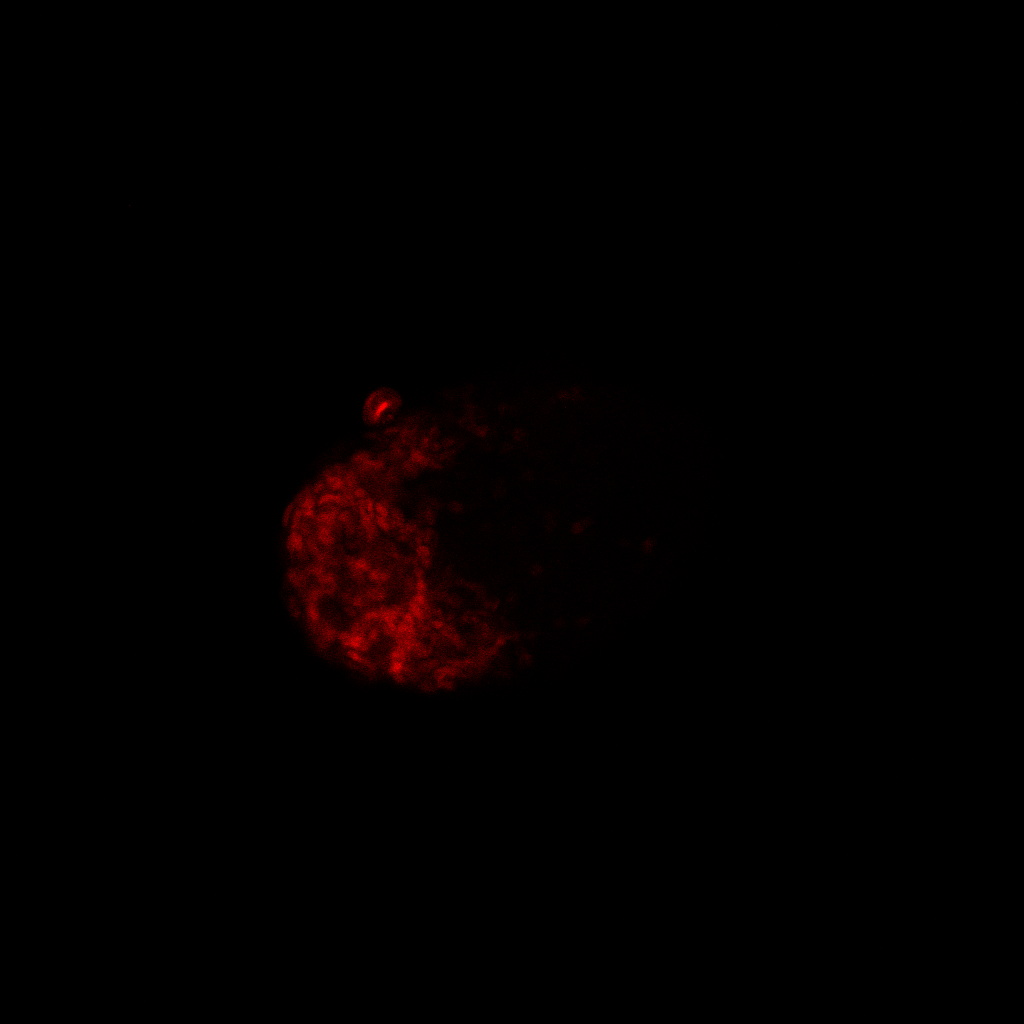

Supplement: Supplementary file 10 — Source data Fig. 8 [file 44318_2025_558_MOESM10_ESM.zip › Figure 8/panel 8G/KD-1_Bra/seq8685c2.tif]

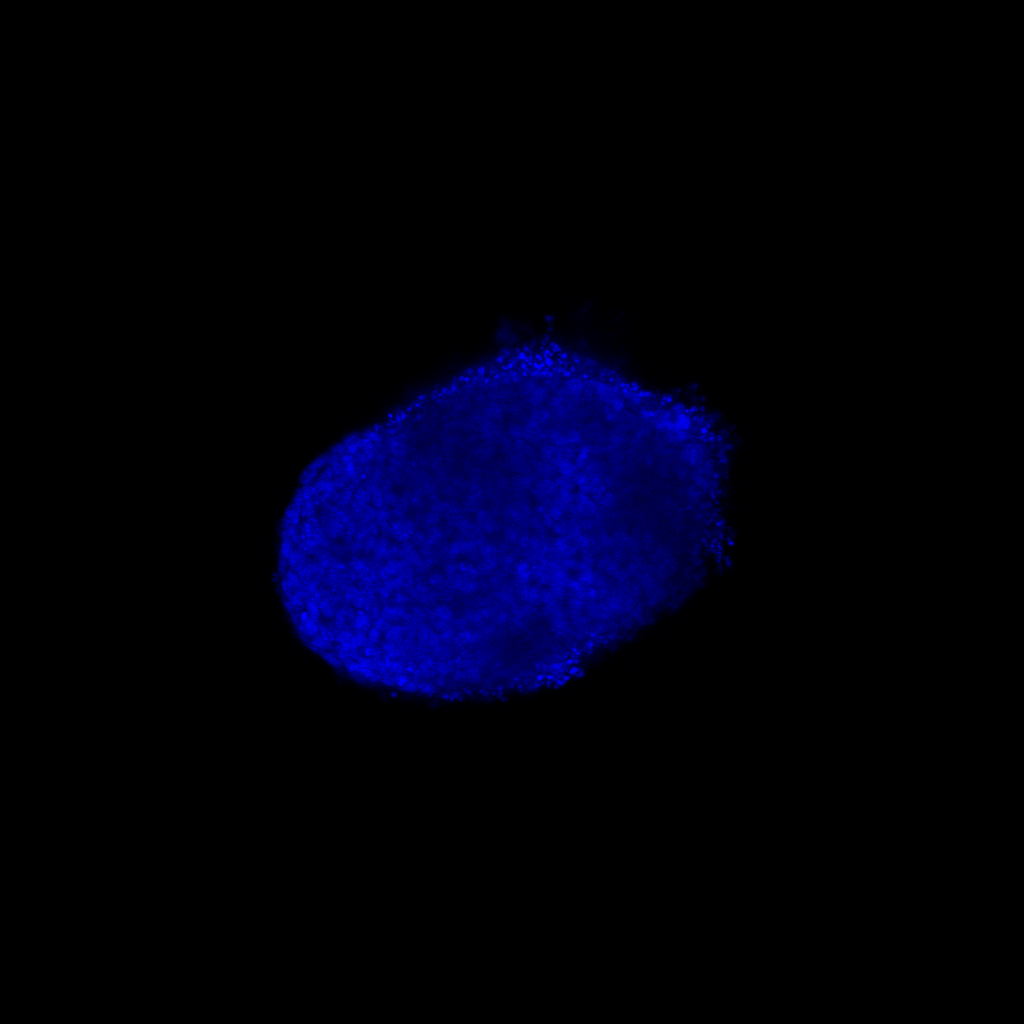

Supplement: Supplementary file 10 — Source data Fig. 8 [file 44318_2025_558_MOESM10_ESM.zip › Figure 8/panel 8G/KD-1_Bra/seq8685c1.tif]

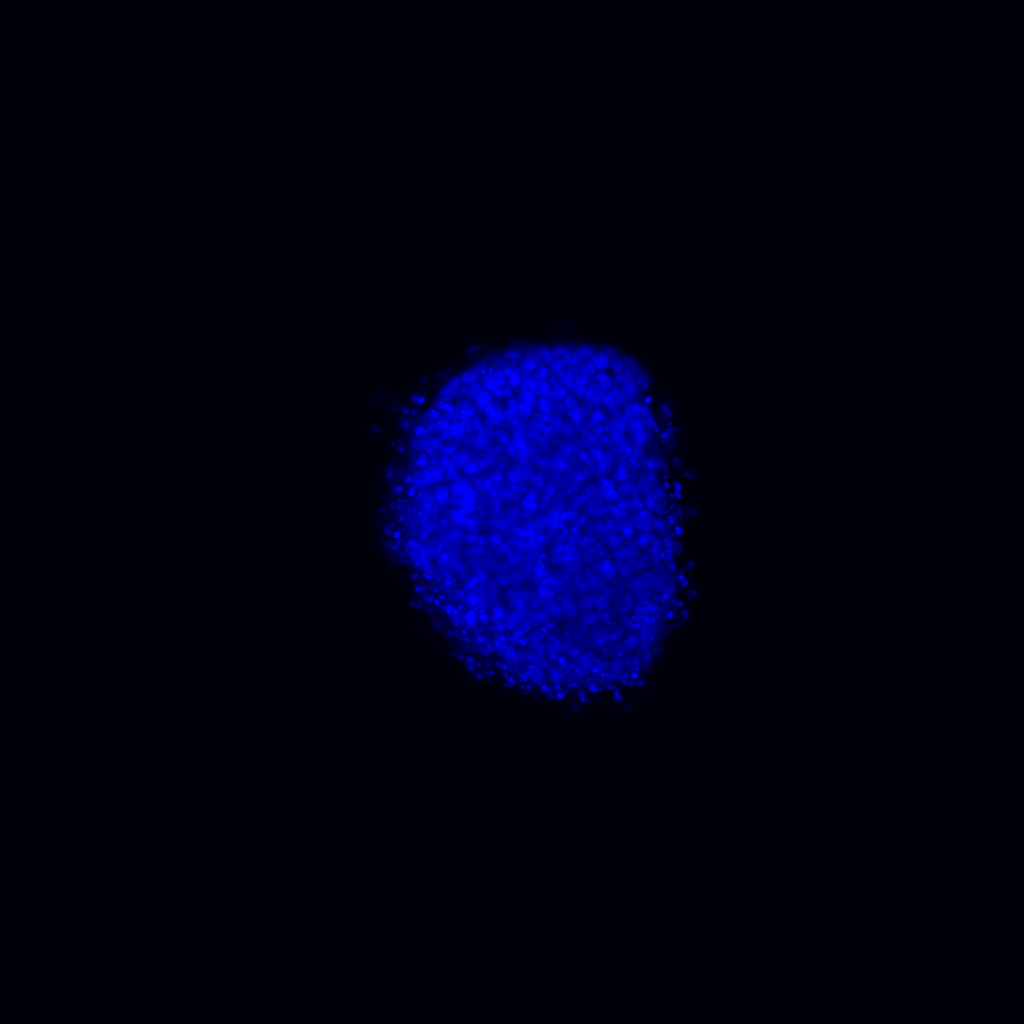

Supplement: Supplementary file 10 — Source data Fig. 8 [file 44318_2025_558_MOESM10_ESM.zip › Figure 8/panel 8G/KD-1_Nanog/seq9160_seq9160_RGB_DAPI.tif]

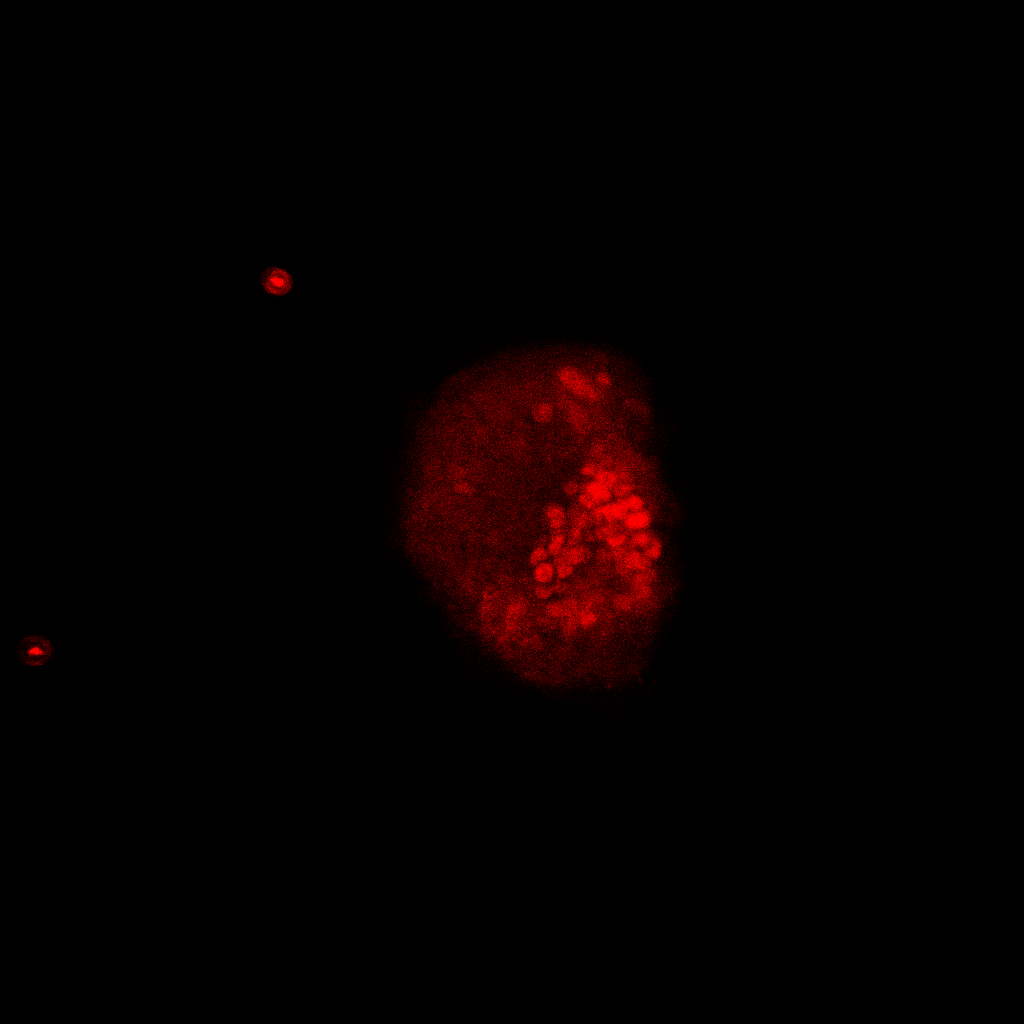

Supplement: Supplementary file 10 — Source data Fig. 8 [file 44318_2025_558_MOESM10_ESM.zip › Figure 8/panel 8G/KD-1_Nanog/seq9160_seq9160_RGB_Texas Red.tif]

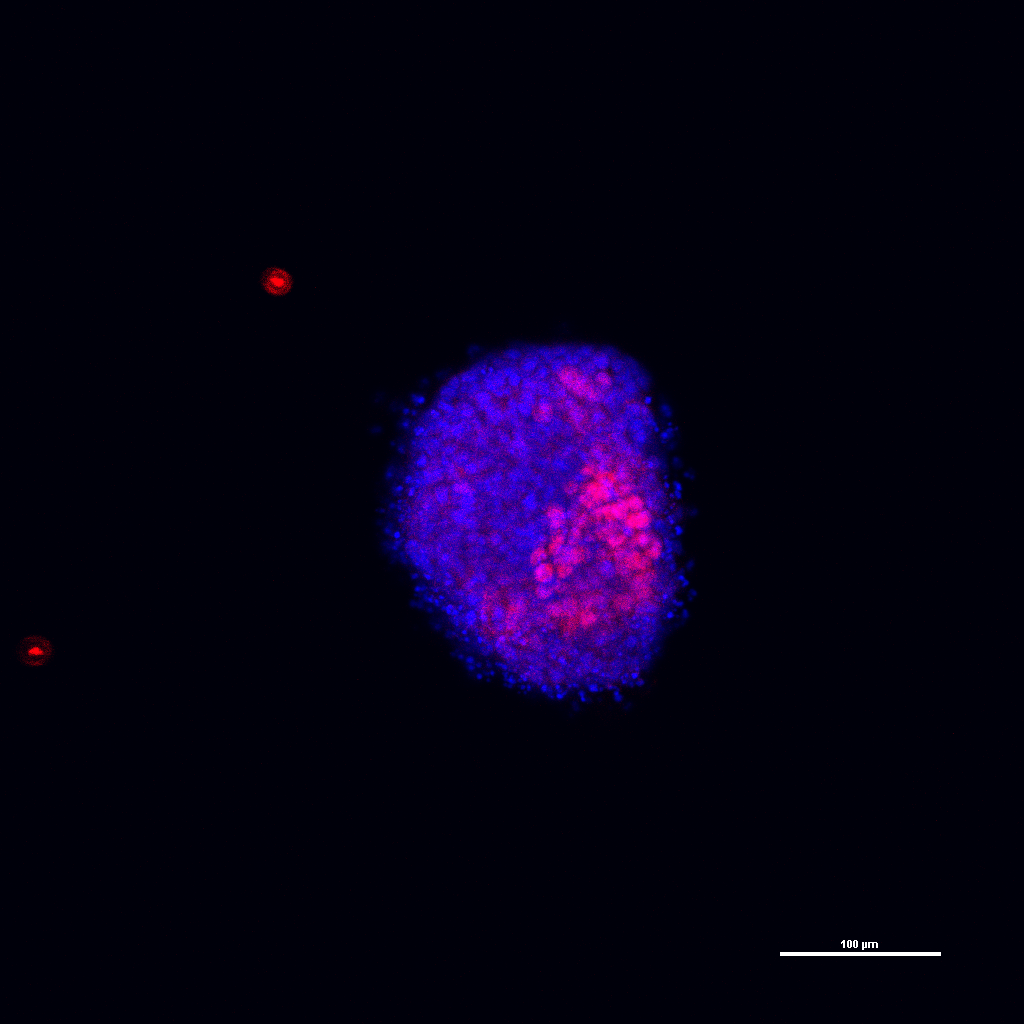

Supplement: Supplementary file 10 — Source data Fig. 8 [file 44318_2025_558_MOESM10_ESM.zip › Figure 8/panel 8G/KD-1_Nanog/seq9160_seq9160_RGB.tif]

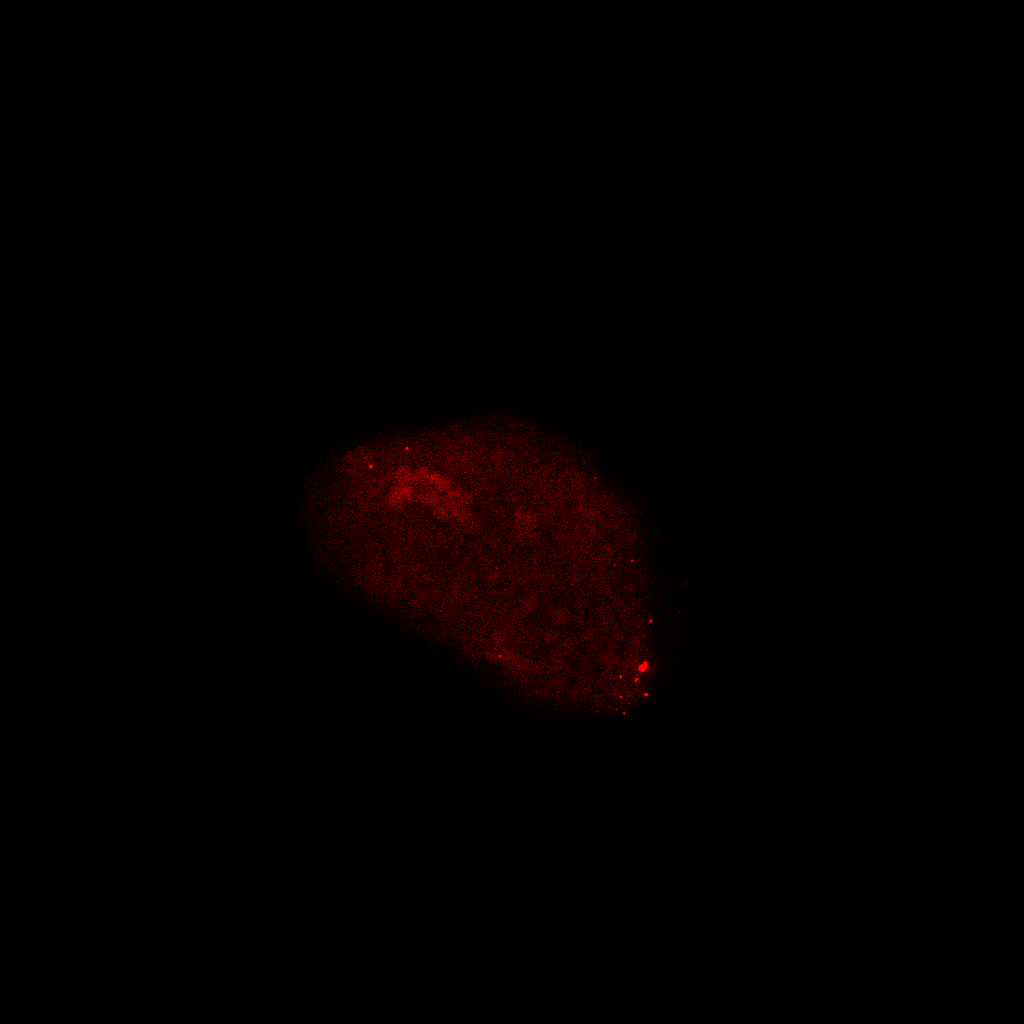

Supplement: Supplementary file 10 — Source data Fig. 8 [file 44318_2025_558_MOESM10_ESM.zip › Figure 8/panel 8G/KD-2_Bra/seq8978_seq8978_RGB_Texas Red.tif]

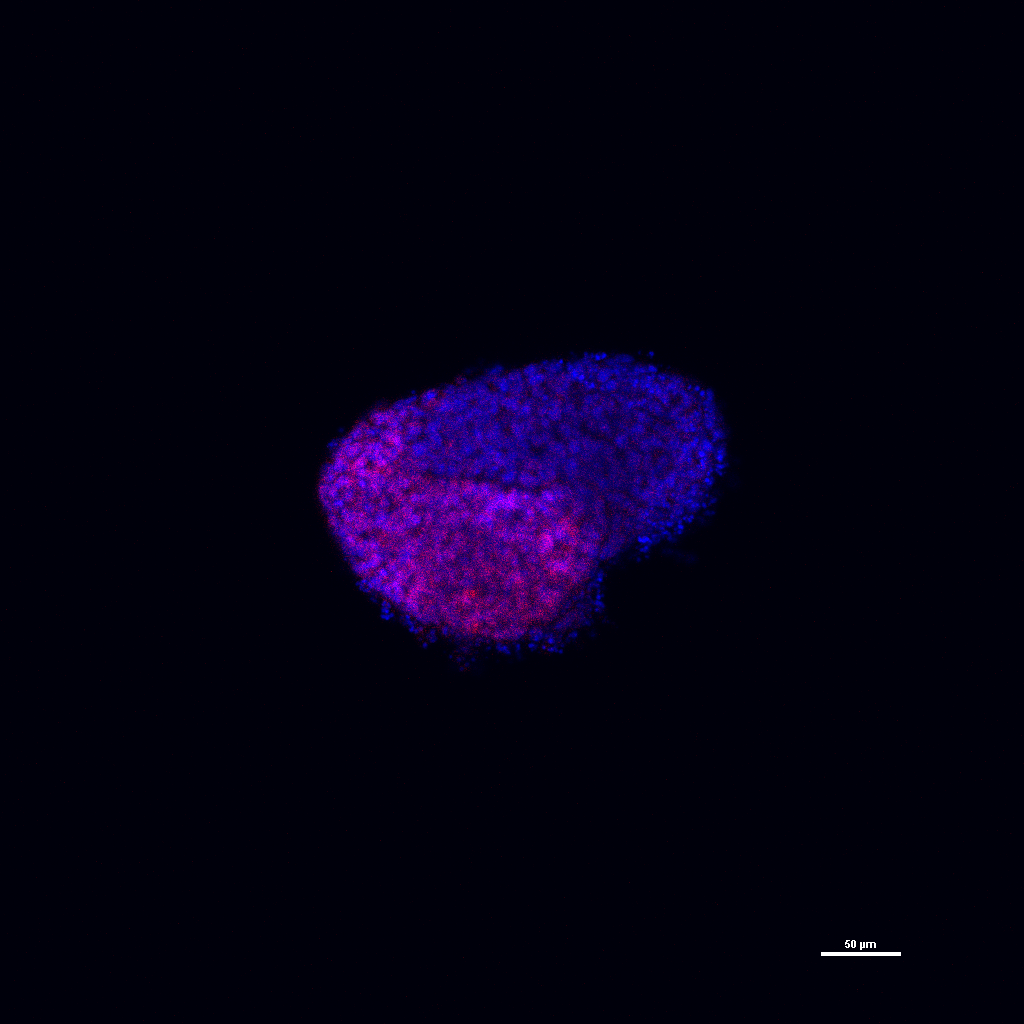

Supplement: Supplementary file 10 — Source data Fig. 8 [file 44318_2025_558_MOESM10_ESM.zip › Figure 8/panel 8G/KD-2_Bra/seq8970_seq8970_RGB.tif]

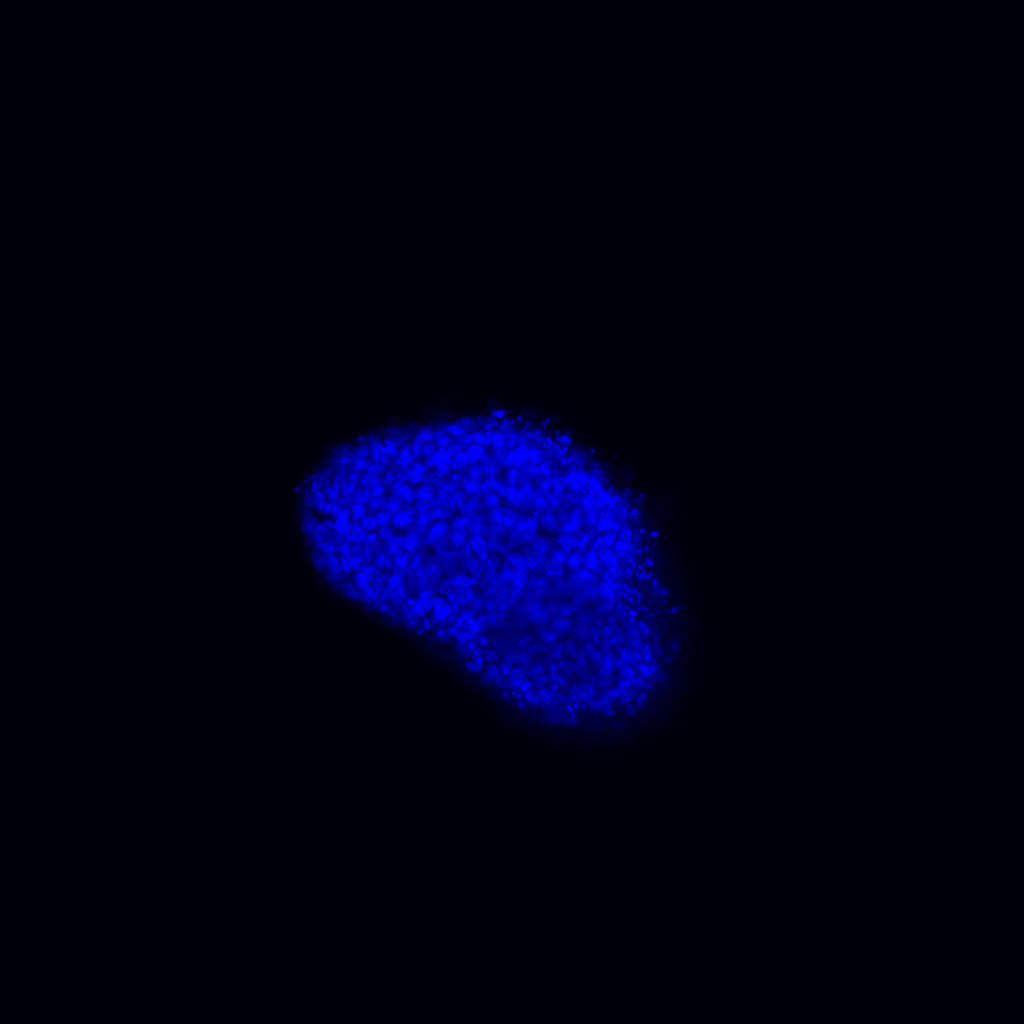

Supplement: Supplementary file 10 — Source data Fig. 8 [file 44318_2025_558_MOESM10_ESM.zip › Figure 8/panel 8G/KD-2_Bra/seq8978_seq8978_RGB_DAPI.tif]

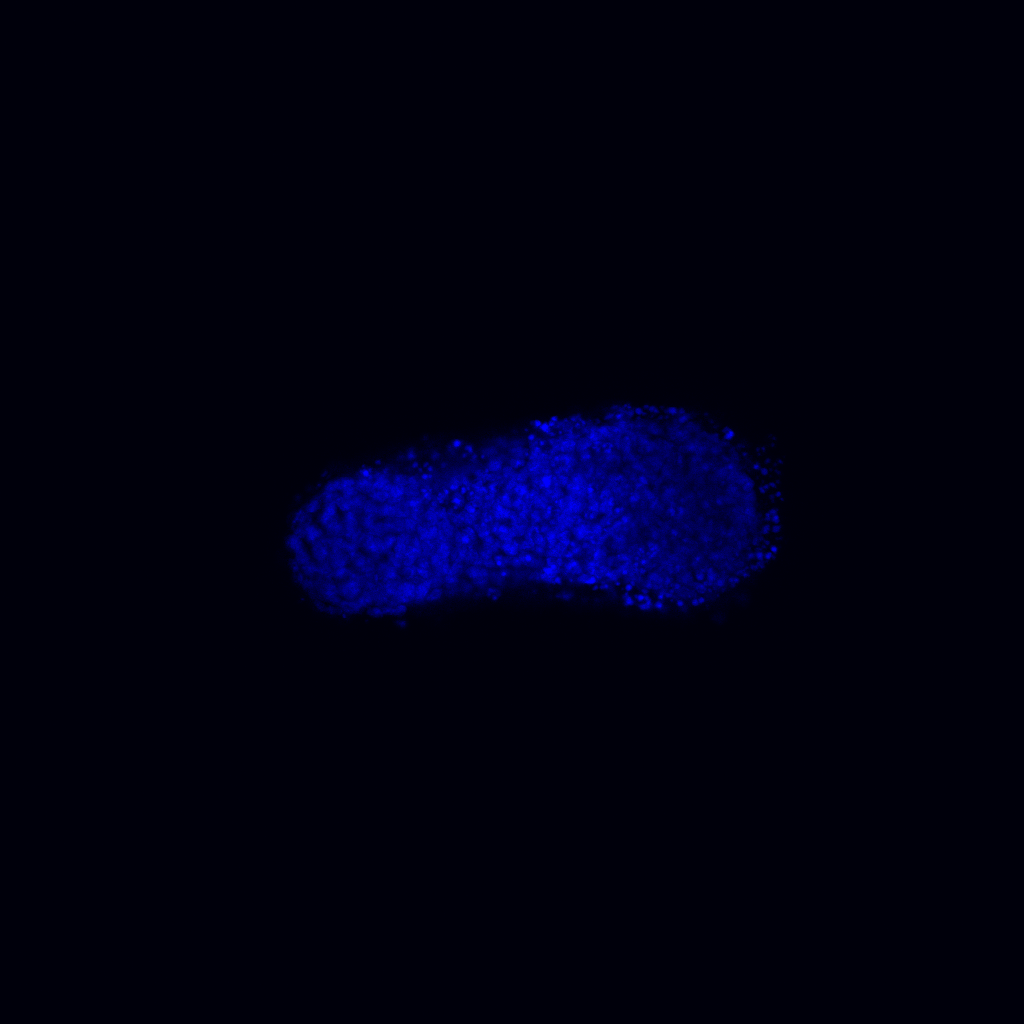

Supplement: Supplementary file 10 — Source data Fig. 8 [file 44318_2025_558_MOESM10_ESM.zip › Figure 8/panel 8G/NT_Nanog/seq8959_seq8959_RGB_DAPI.tif]

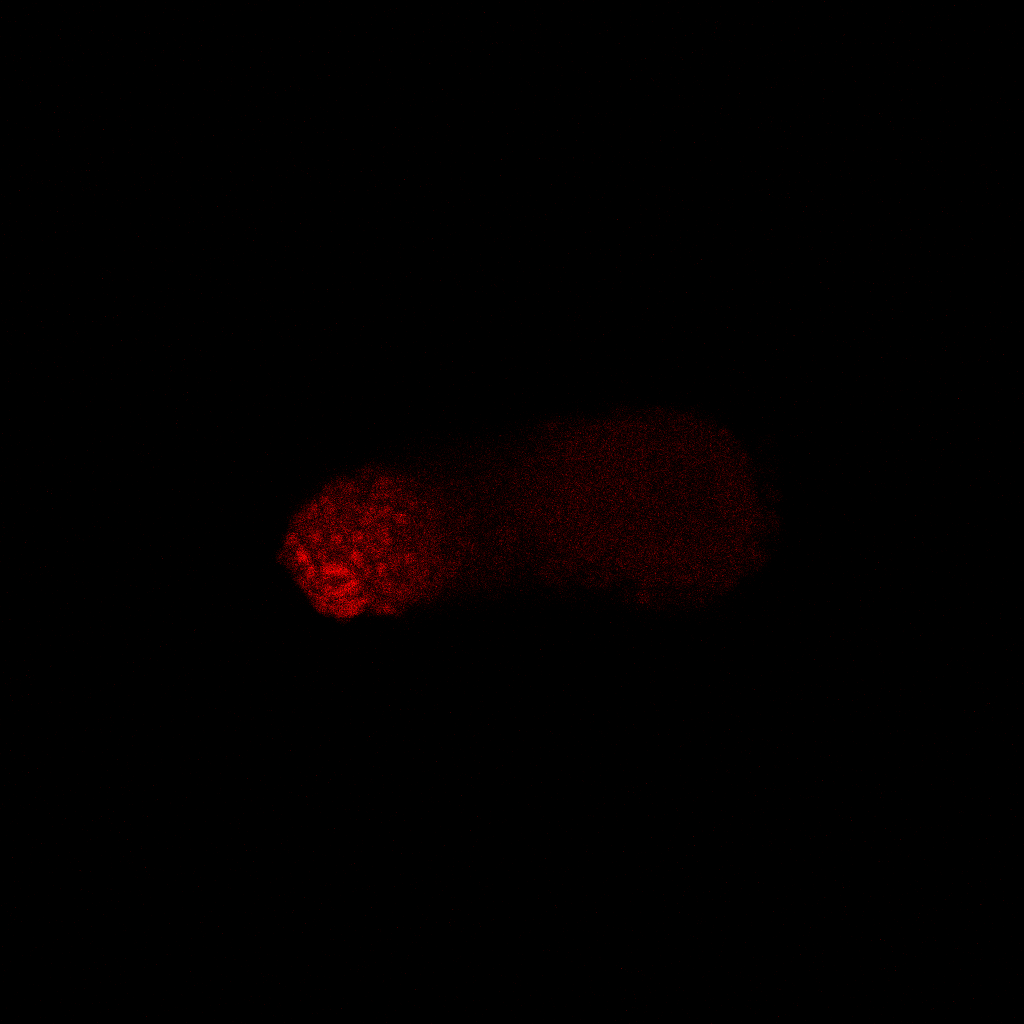

Supplement: Supplementary file 10 — Source data Fig. 8 [file 44318_2025_558_MOESM10_ESM.zip › Figure 8/panel 8G/NT_Nanog/seq8959_seq8959_RGB_Texas Red.tif]

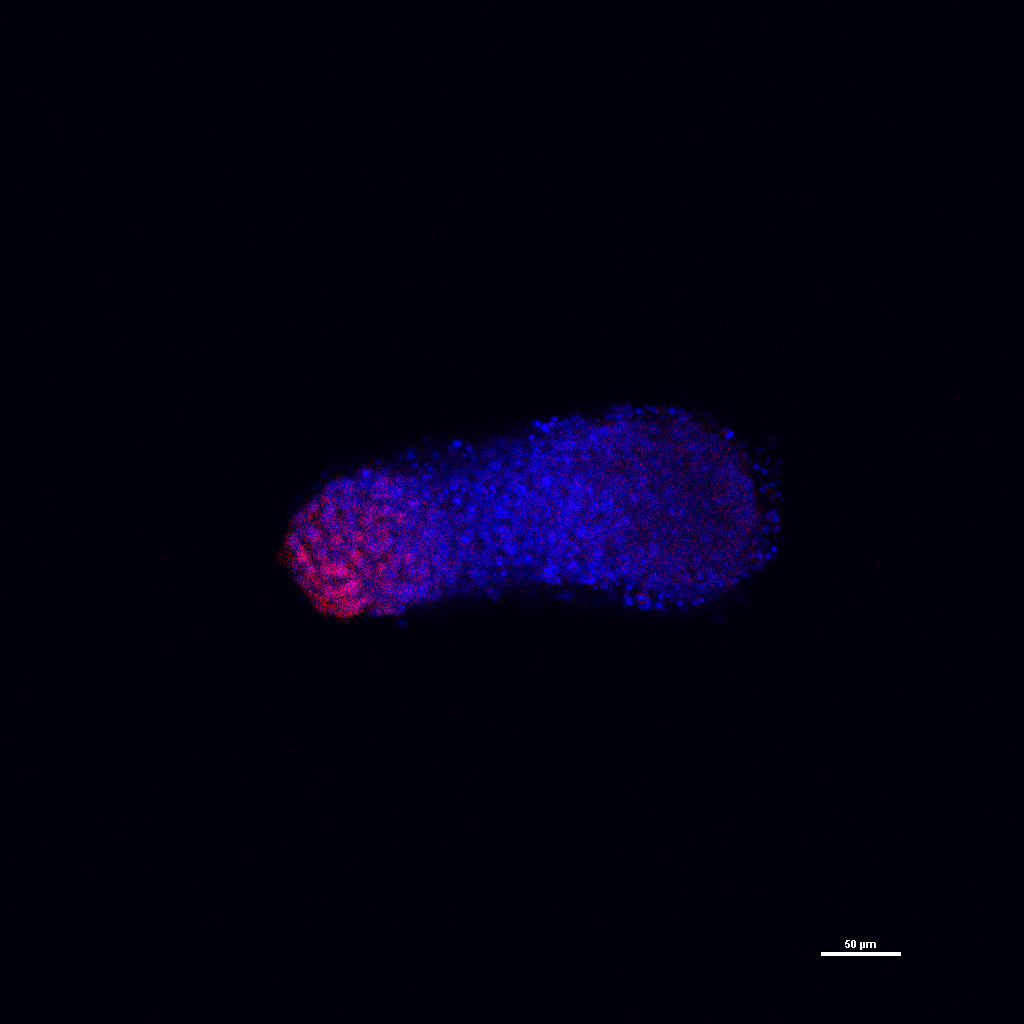

Supplement: Supplementary file 10 — Source data Fig. 8 [file 44318_2025_558_MOESM10_ESM.zip › Figure 8/panel 8G/NT_Nanog/seq8959_seq8959_RGB.tif]

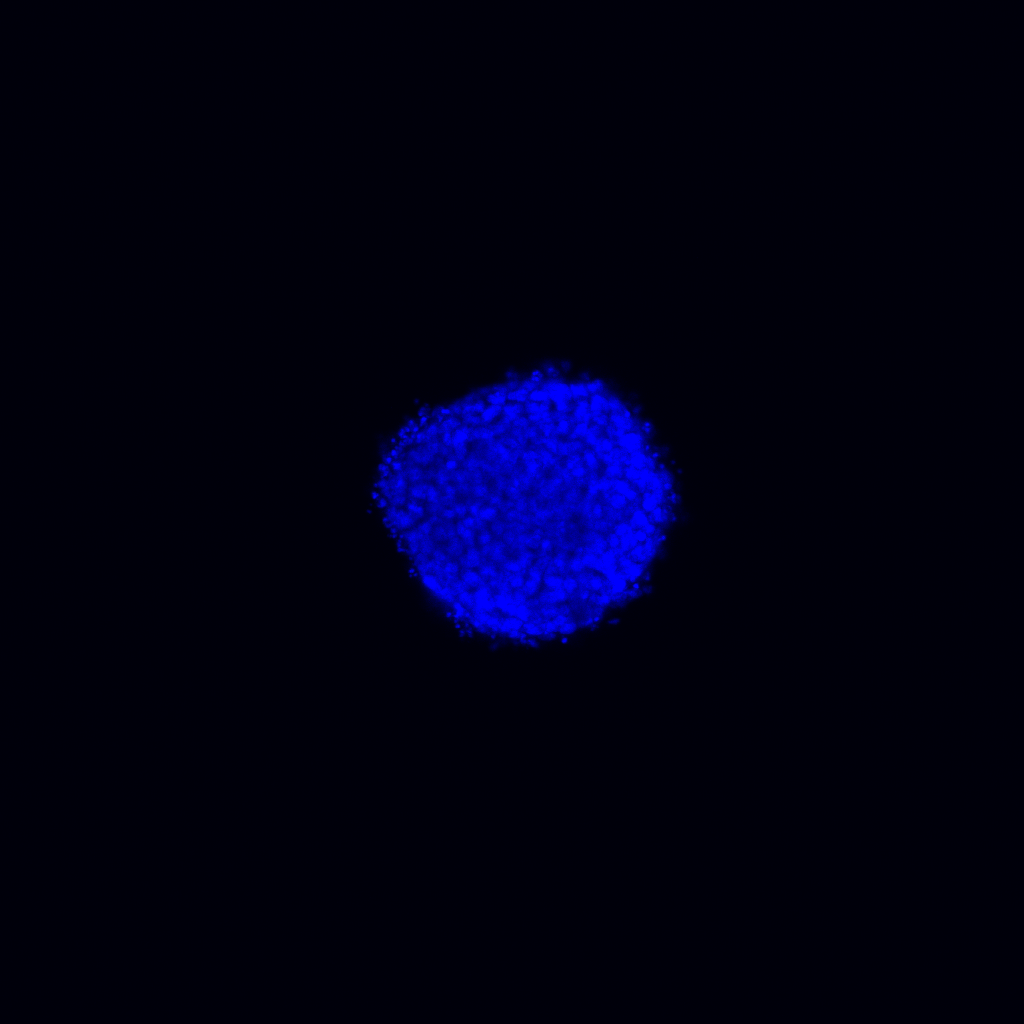

Supplement: Supplementary file 10 — Source data Fig. 8 [file 44318_2025_558_MOESM10_ESM.zip › Figure 8/panel 8G/KD-2_Oct4/seq9149_seq9149_RGB_DAPI.tif]

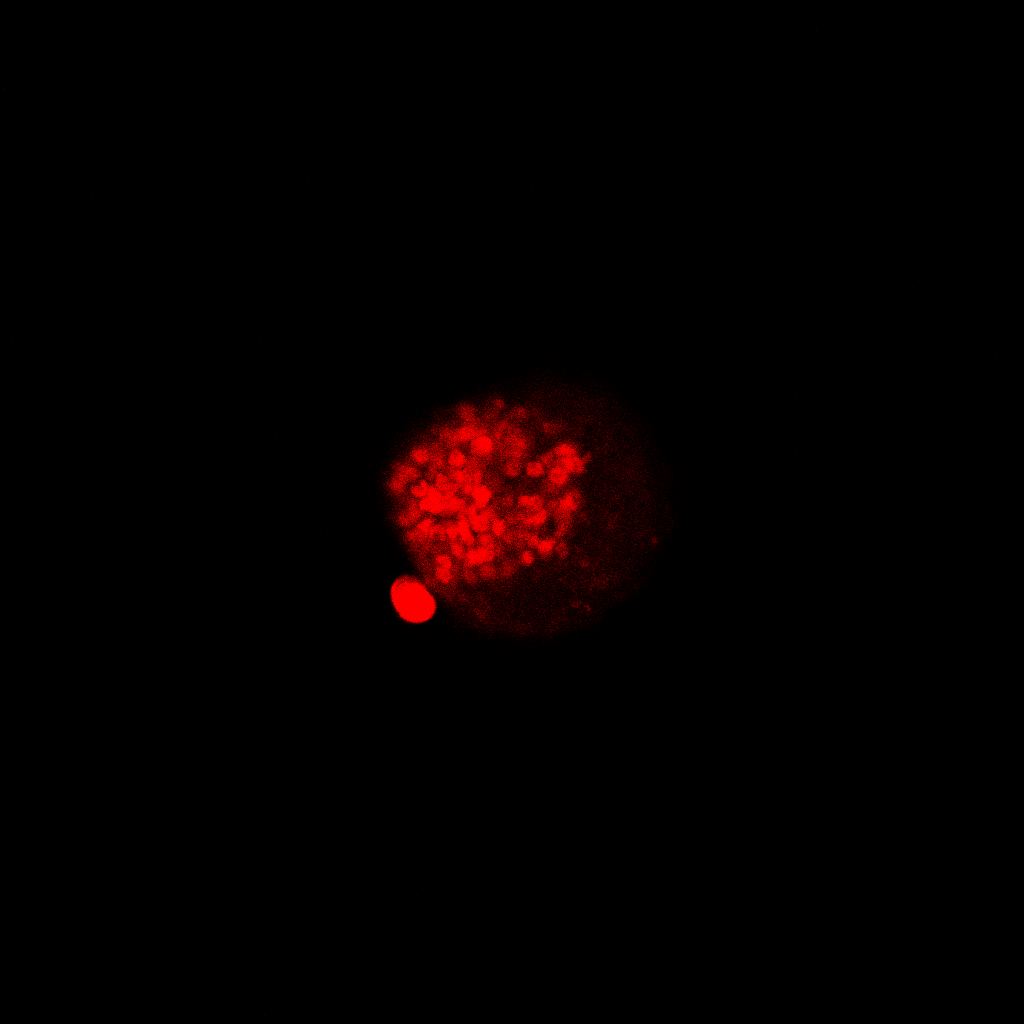

Supplement: Supplementary file 10 — Source data Fig. 8 [file 44318_2025_558_MOESM10_ESM.zip › Figure 8/panel 8G/KD-2_Oct4/seq9149_seq9149_RGB_Texas Red.tif]

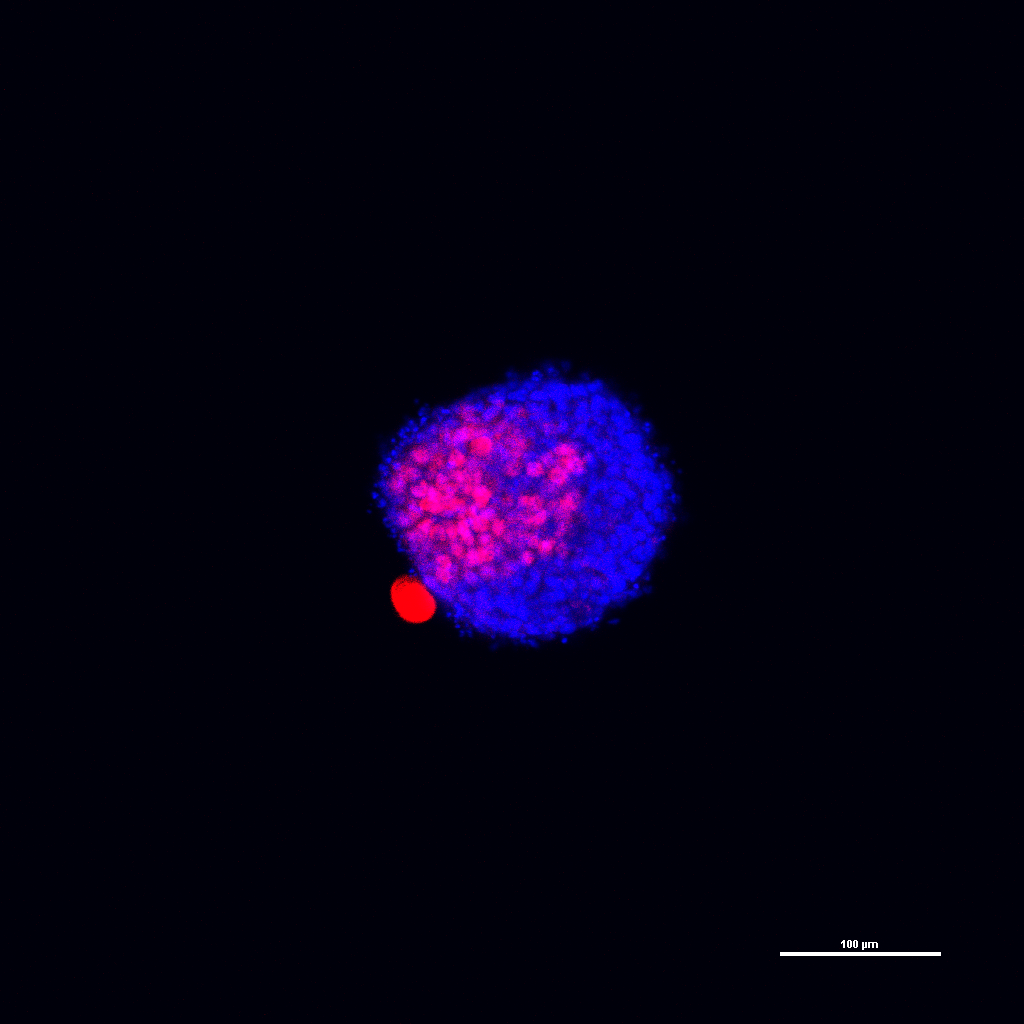

Supplement: Supplementary file 10 — Source data Fig. 8 [file 44318_2025_558_MOESM10_ESM.zip › Figure 8/panel 8G/KD-2_Oct4/seq9149_seq9149_RGB.tif]

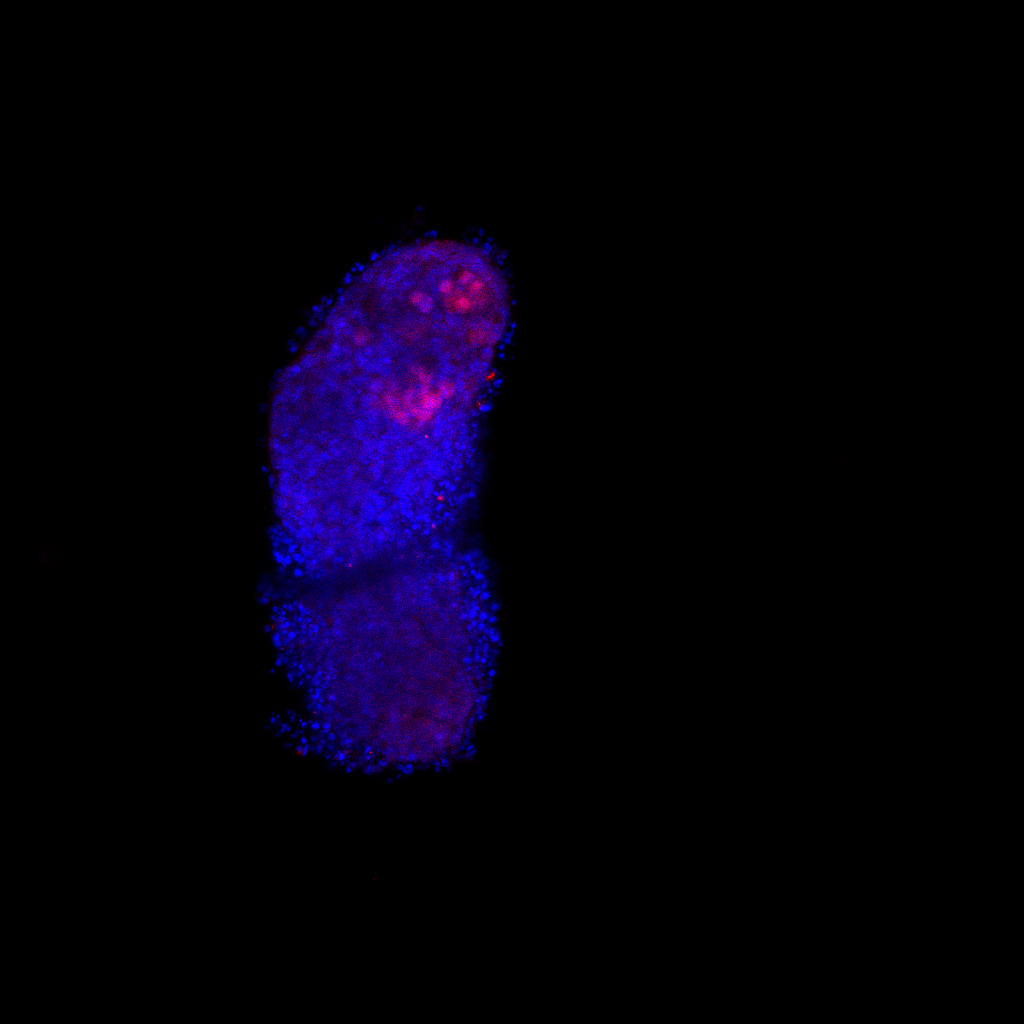

Supplement: Supplementary file 10 — Source data Fig. 8 [file 44318_2025_558_MOESM10_ESM.zip › Figure 8/panel 8G/NT_Bra/seq8694.tif]

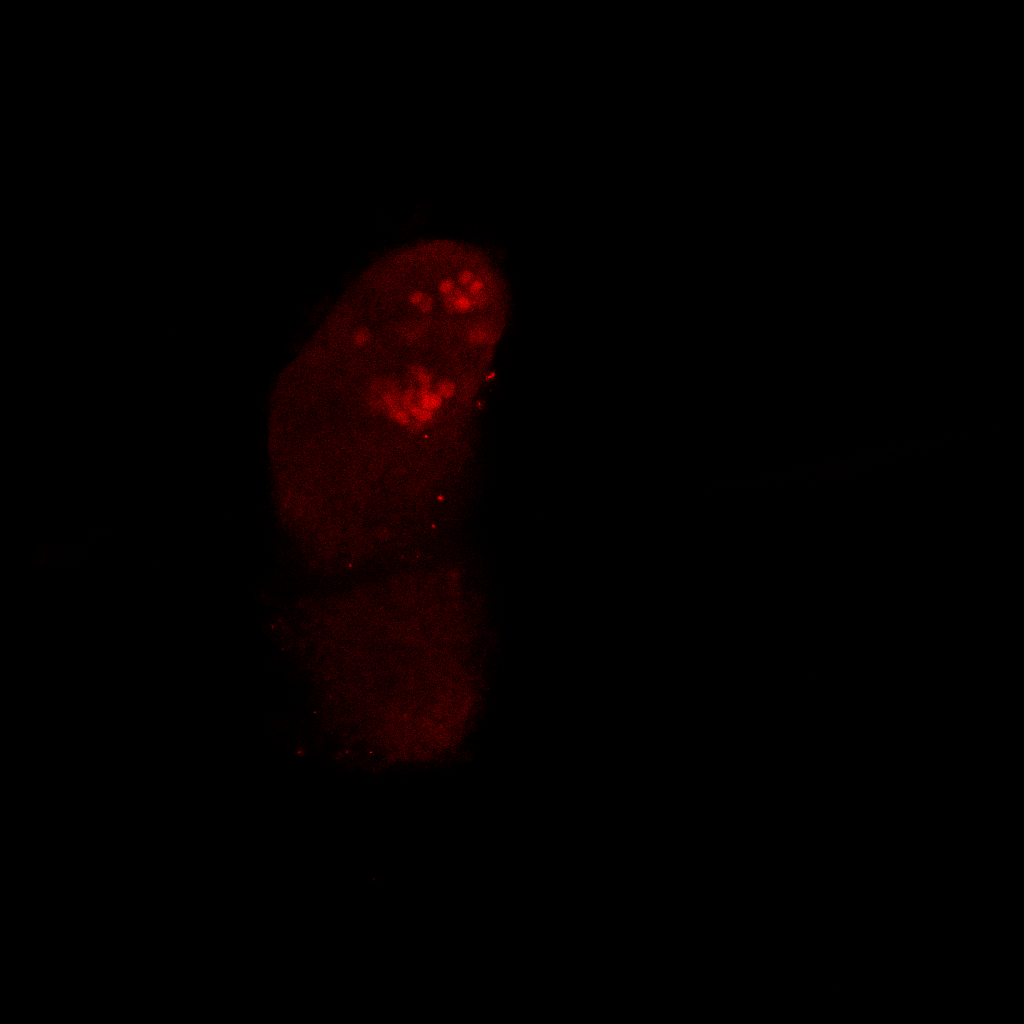

Supplement: Supplementary file 10 — Source data Fig. 8 [file 44318_2025_558_MOESM10_ESM.zip › Figure 8/panel 8G/NT_Bra/seq8694c2.tif]

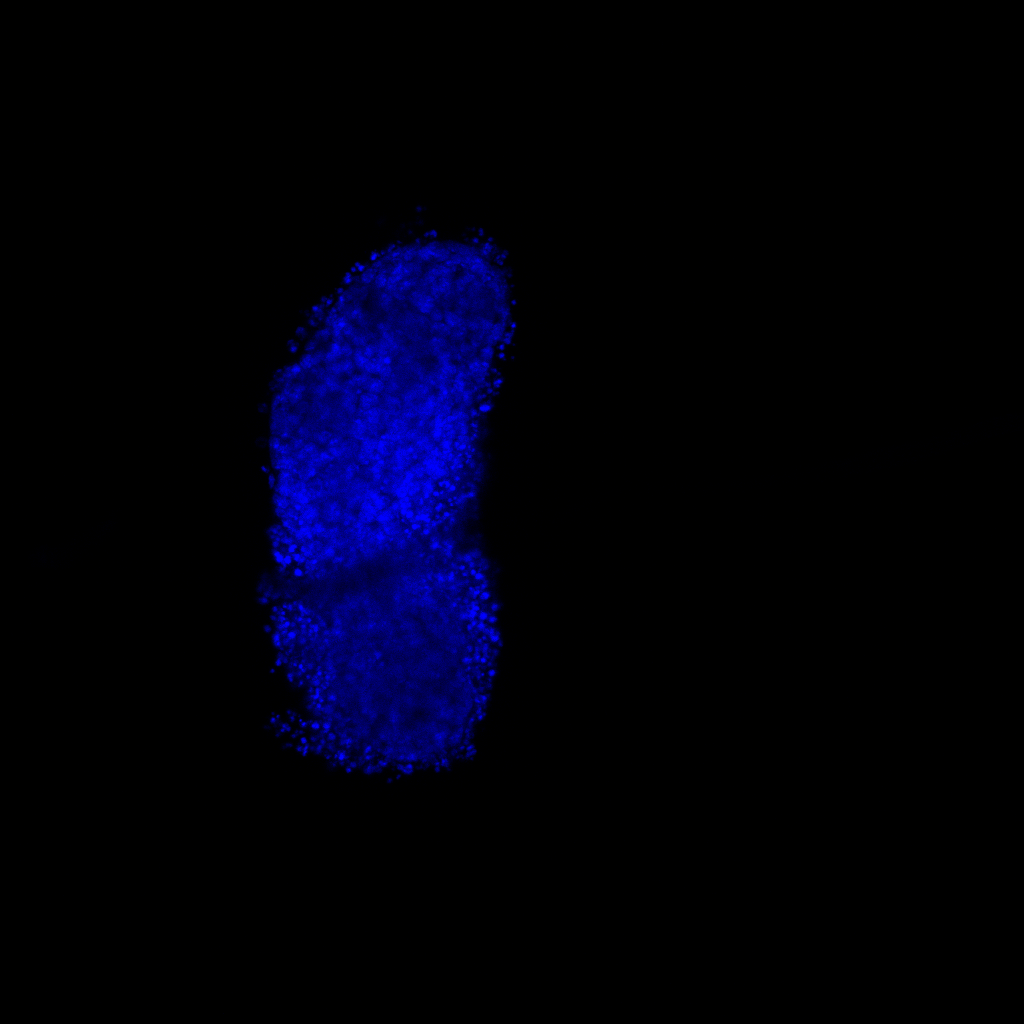

Supplement: Supplementary file 10 — Source data Fig. 8 [file 44318_2025_558_MOESM10_ESM.zip › Figure 8/panel 8G/NT_Bra/seq8694c1.tif]

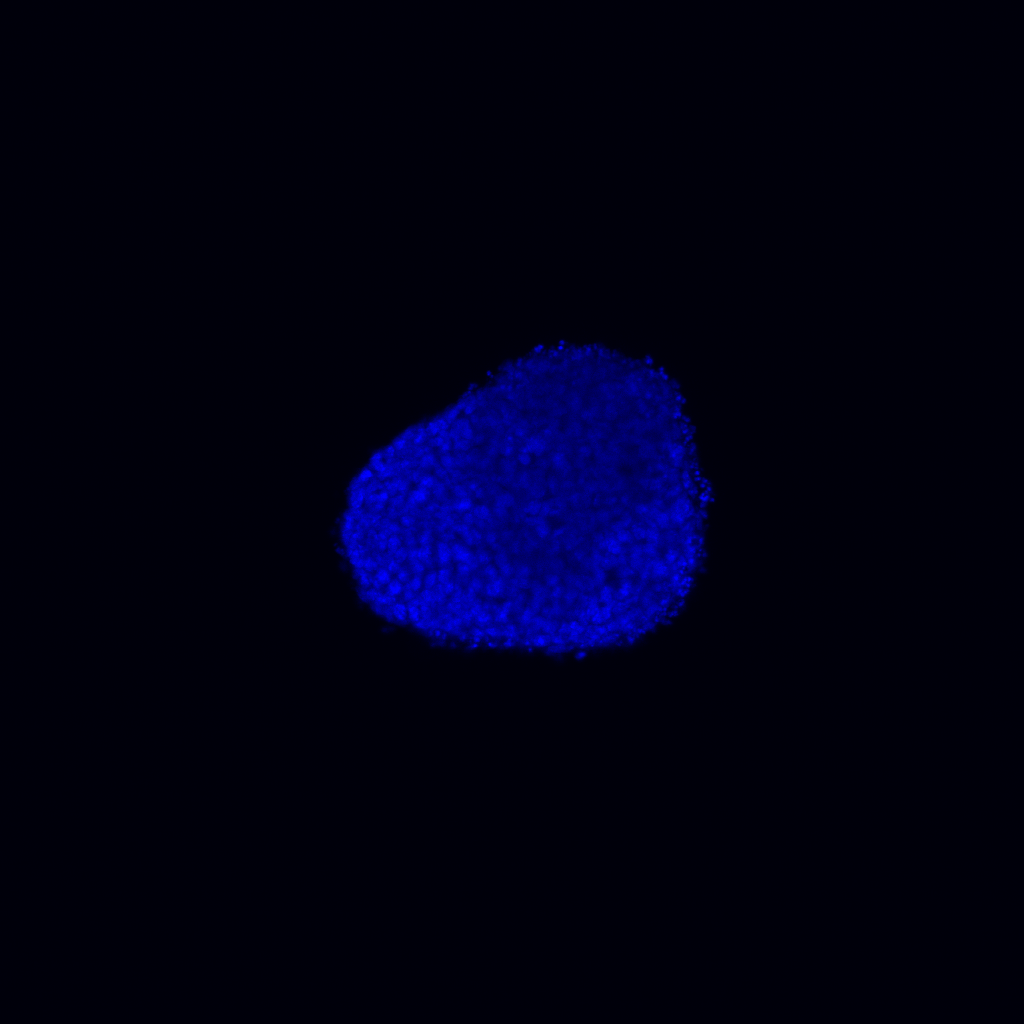

Supplement: Supplementary file 10 — Source data Fig. 8 [file 44318_2025_558_MOESM10_ESM.zip › Figure 8/panel 8G/KD-2_Nanog/seq9154_seq9154_RGB_DAPI.tif]

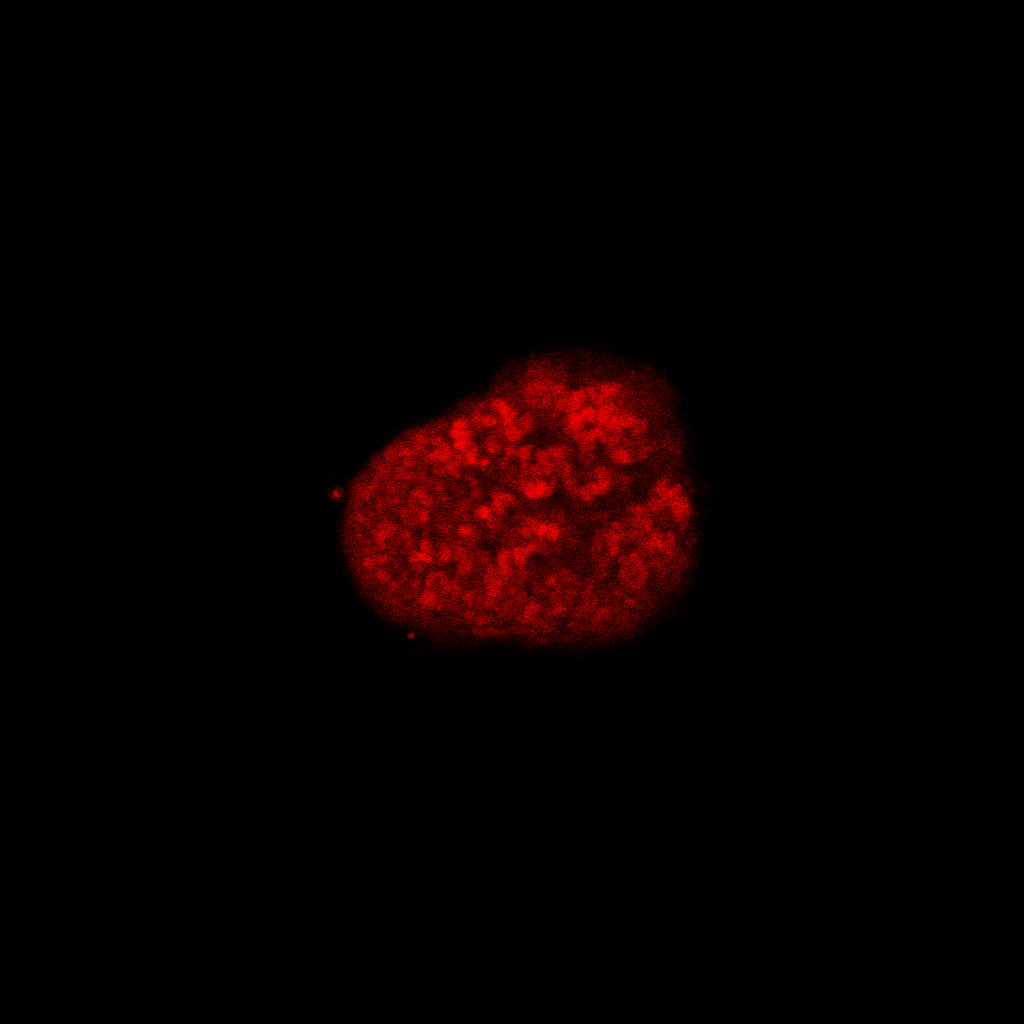

Supplement: Supplementary file 10 — Source data Fig. 8 [file 44318_2025_558_MOESM10_ESM.zip › Figure 8/panel 8G/KD-2_Nanog/seq9154_seq9154_RGB_Texas Red.tif]

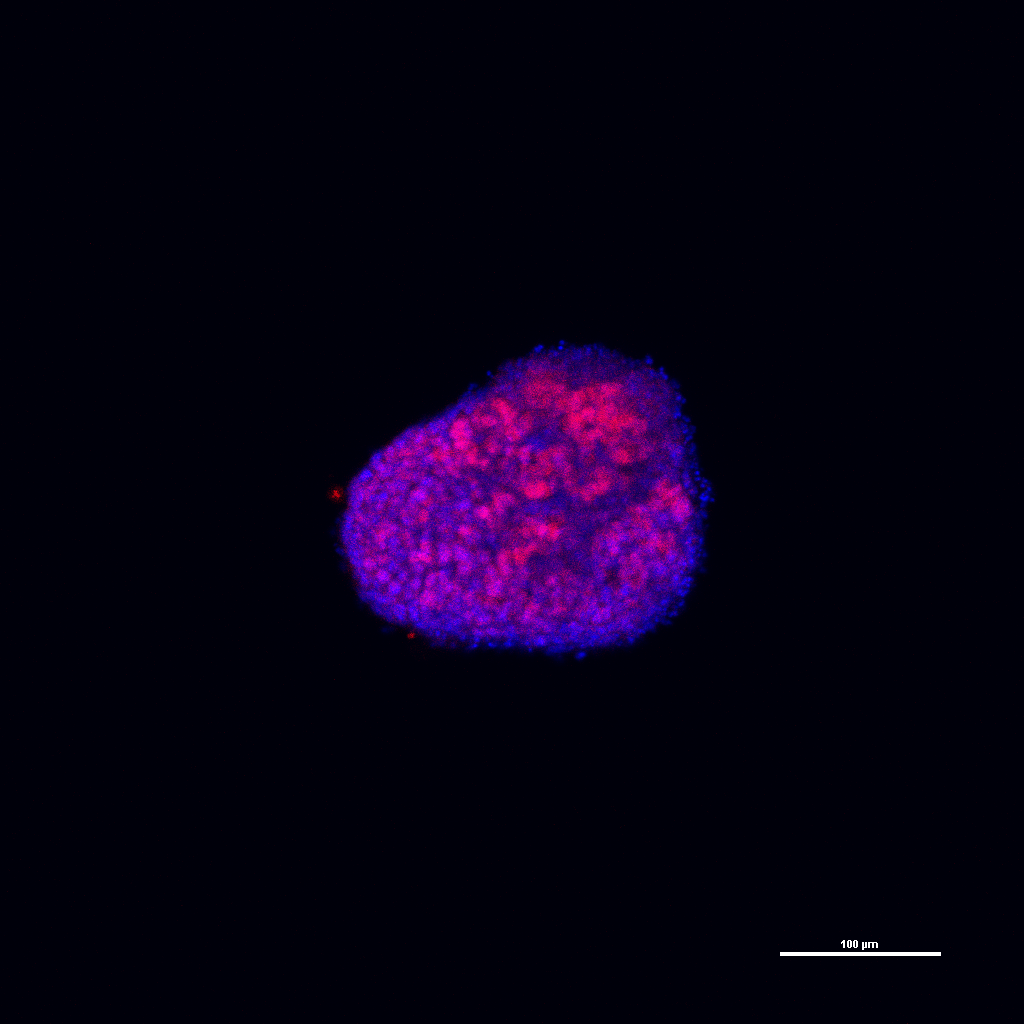

Supplement: Supplementary file 10 — Source data Fig. 8 [file 44318_2025_558_MOESM10_ESM.zip › Figure 8/panel 8G/KD-2_Nanog/seq9154_seq9154_RGB.tif]

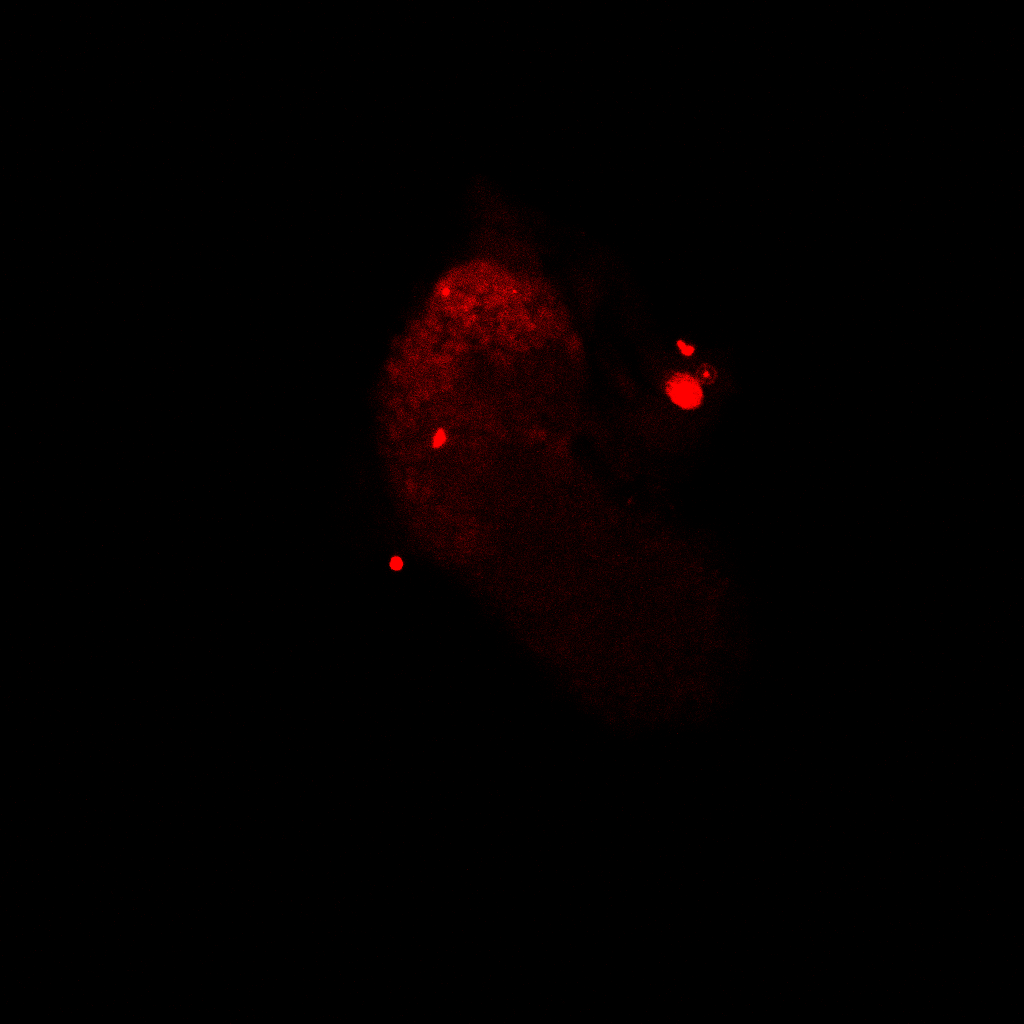

Supplement: Supplementary file 10 — Source data Fig. 8 [file 44318_2025_558_MOESM10_ESM.zip › Figure 8/panel 8G/KD-1+WAY_Oct4/seq9184_seq9184_RGB_Texas Red.tif]

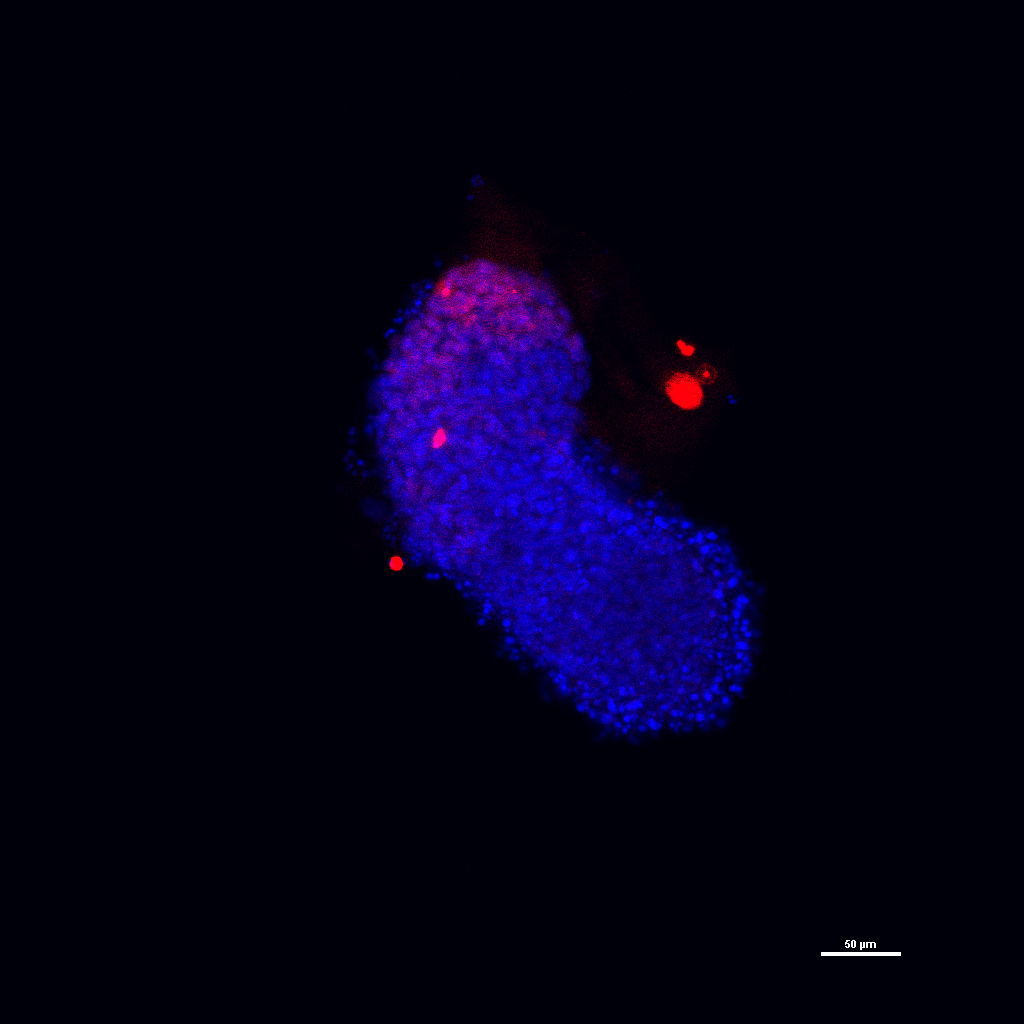

Supplement: Supplementary file 10 — Source data Fig. 8 [file 44318_2025_558_MOESM10_ESM.zip › Figure 8/panel 8G/KD-1+WAY_Oct4/seq9184_seq9184_RGB.tif]

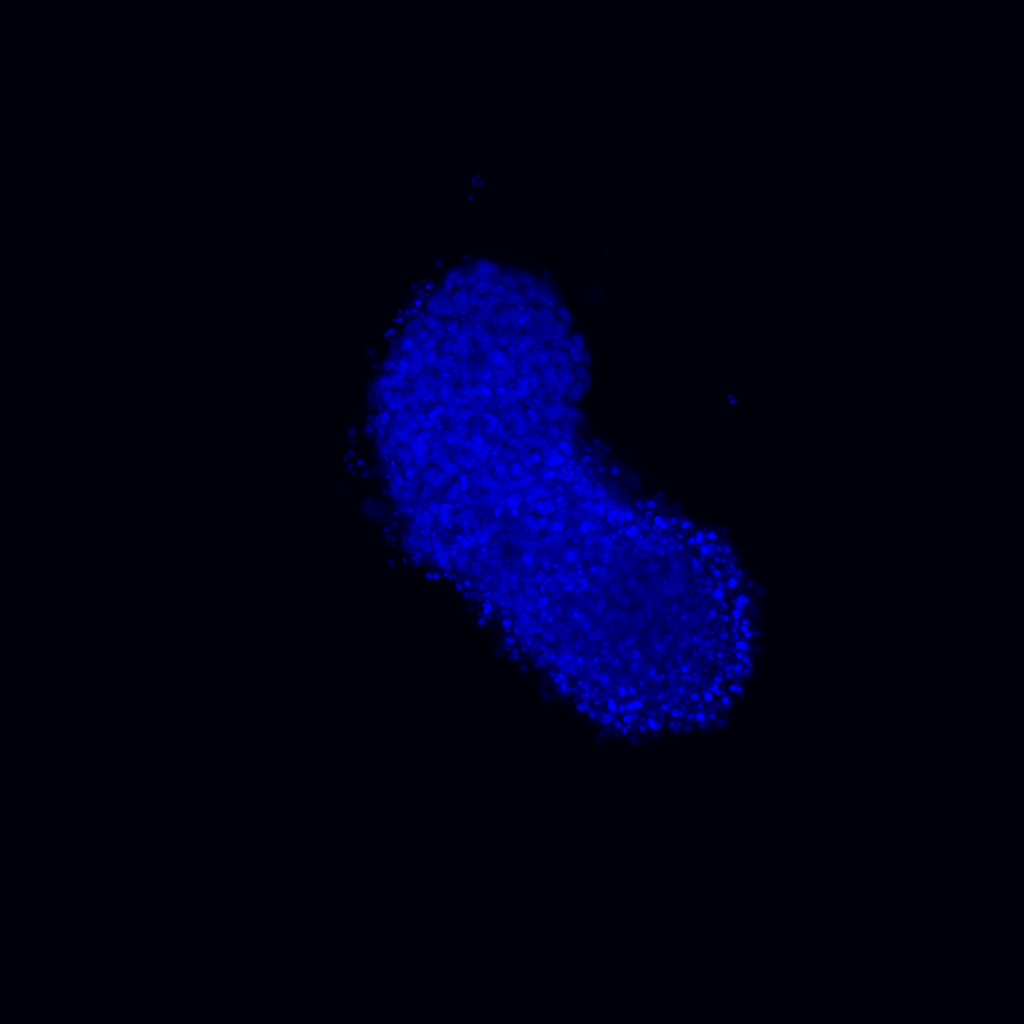

Supplement: Supplementary file 10 — Source data Fig. 8 [file 44318_2025_558_MOESM10_ESM.zip › Figure 8/panel 8G/KD-1+WAY_Oct4/seq9184_seq9184_RGB_DAPI.tif]

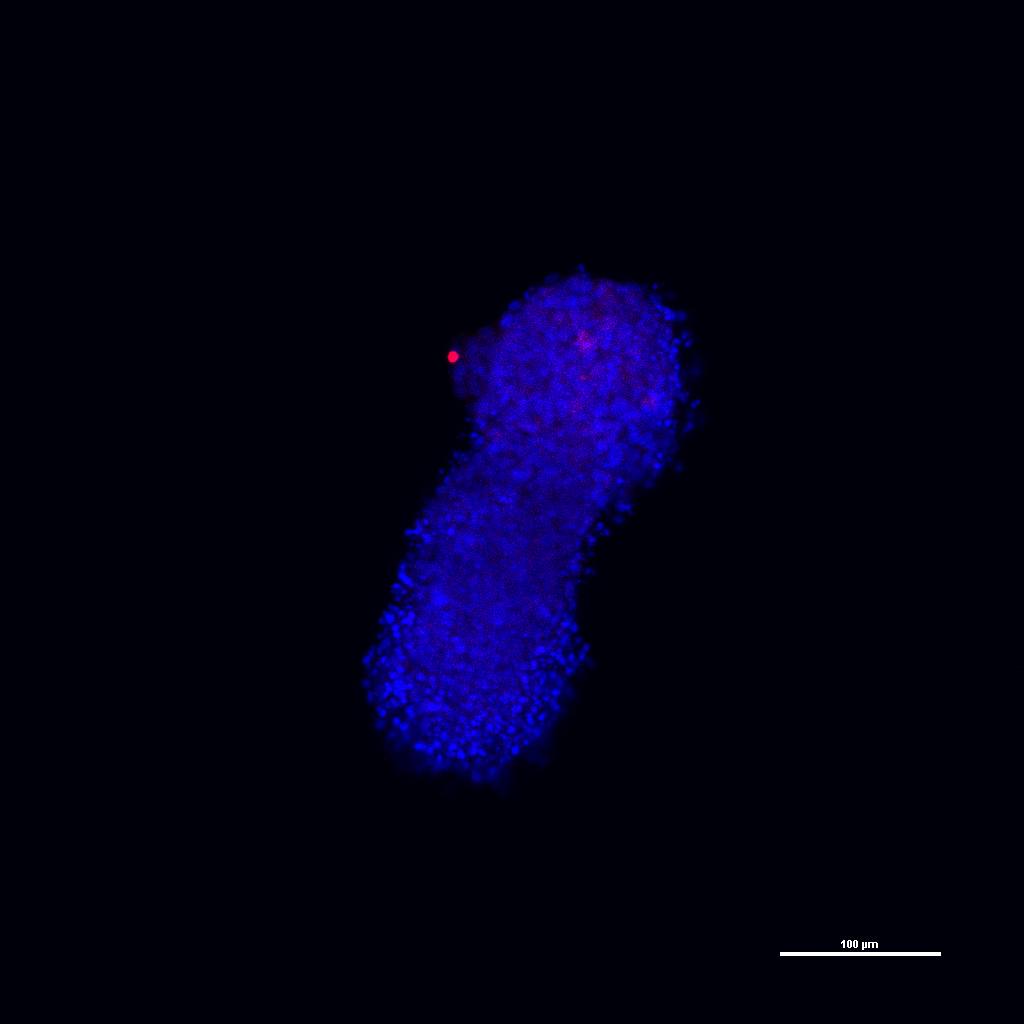

Supplement: Supplementary file 10 — Source data Fig. 8 [file 44318_2025_558_MOESM10_ESM.zip › Figure 8/panel 8G/NT_Oct4/seq9142_seq9142_RGB.tif]

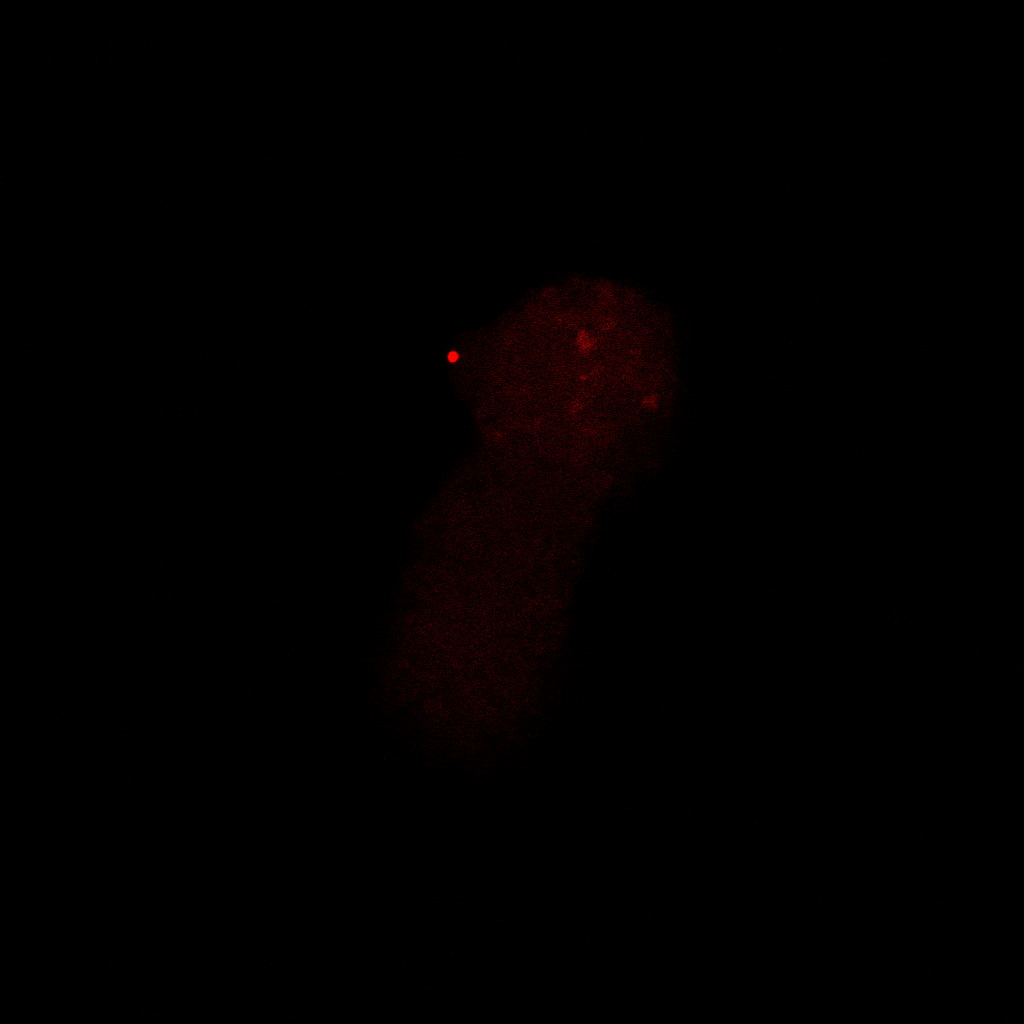

Supplement: Supplementary file 10 — Source data Fig. 8 [file 44318_2025_558_MOESM10_ESM.zip › Figure 8/panel 8G/NT_Oct4/seq9142_seq9142_RGB_Texas Red.tif]

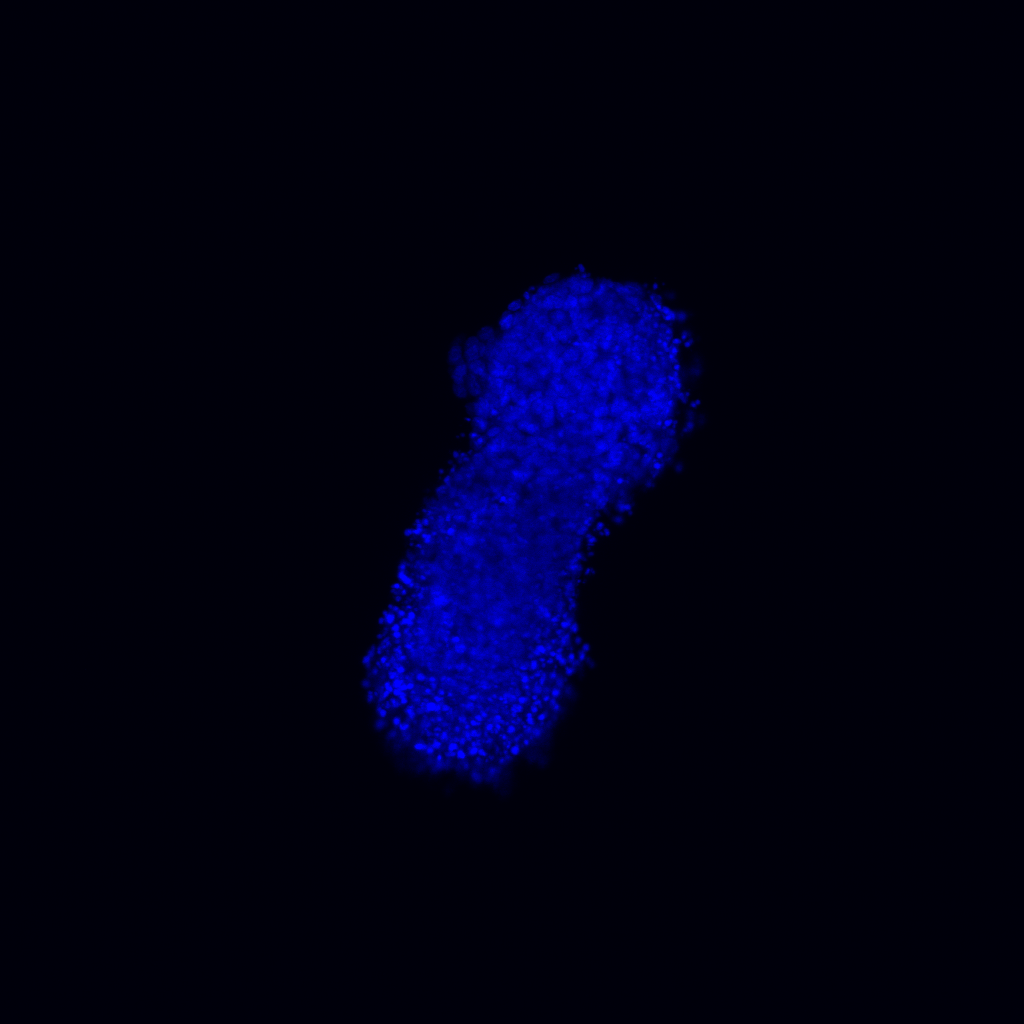

Supplement: Supplementary file 10 — Source data Fig. 8 [file 44318_2025_558_MOESM10_ESM.zip › Figure 8/panel 8G/NT_Oct4/seq9142_seq9142_RGB_DAPI.tif]

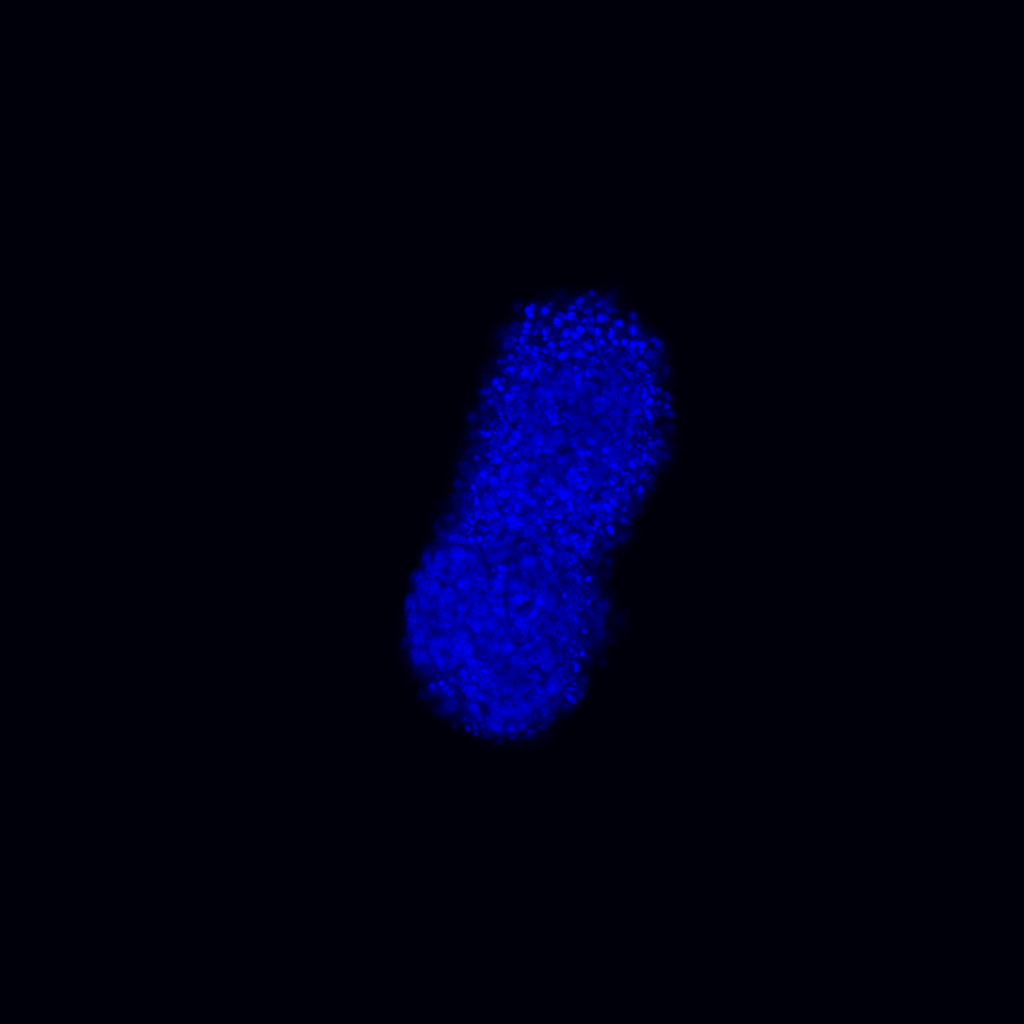

Supplement: Supplementary file 10 — Source data Fig. 8 [file 44318_2025_558_MOESM10_ESM.zip › Figure 8/panel 8G/KD-2+WAY_Nanog/seq9183_seq9183_RGB_DAPI.tif]

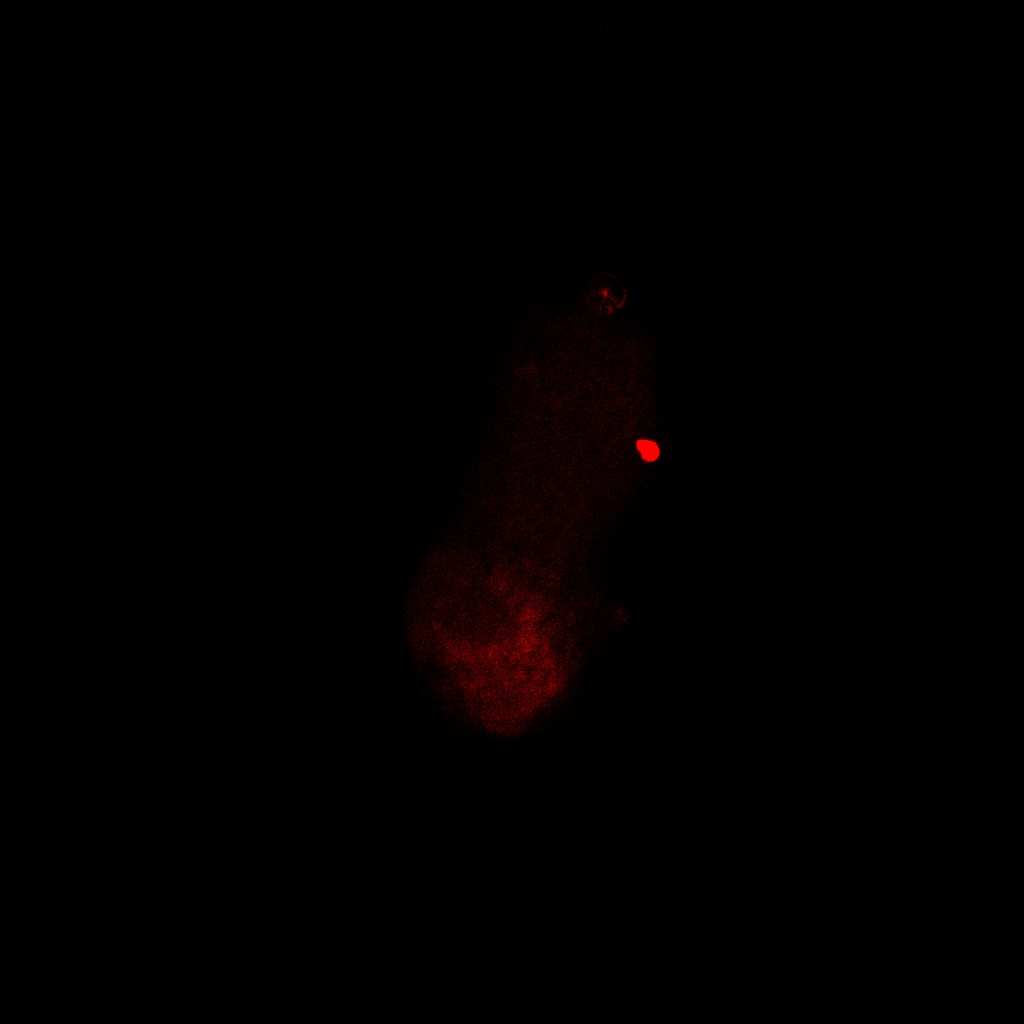

Supplement: Supplementary file 10 — Source data Fig. 8 [file 44318_2025_558_MOESM10_ESM.zip › Figure 8/panel 8G/KD-2+WAY_Nanog/seq9183_seq9183_RGB_Texas Red.tif]

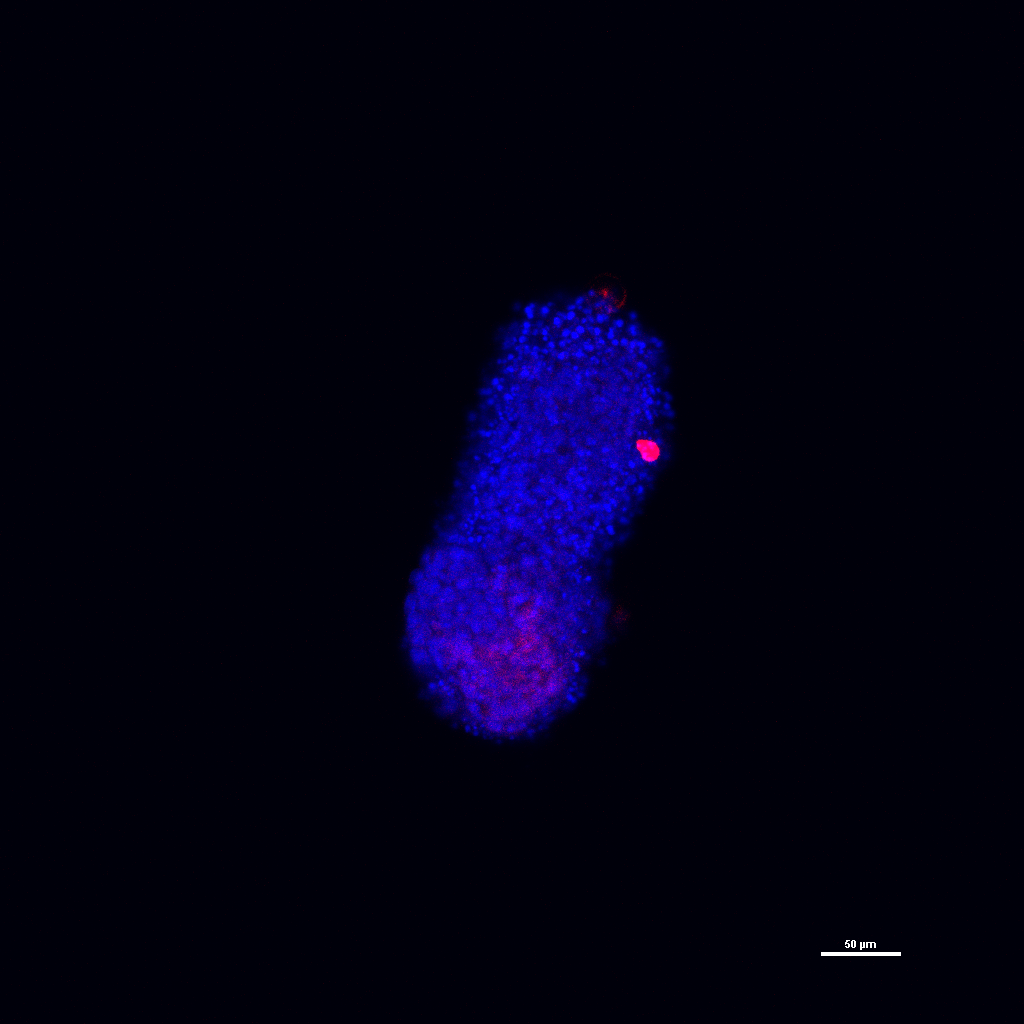

Supplement: Supplementary file 10 — Source data Fig. 8 [file 44318_2025_558_MOESM10_ESM.zip › Figure 8/panel 8G/KD-2+WAY_Nanog/seq9183_seq9183_RGB.tif]

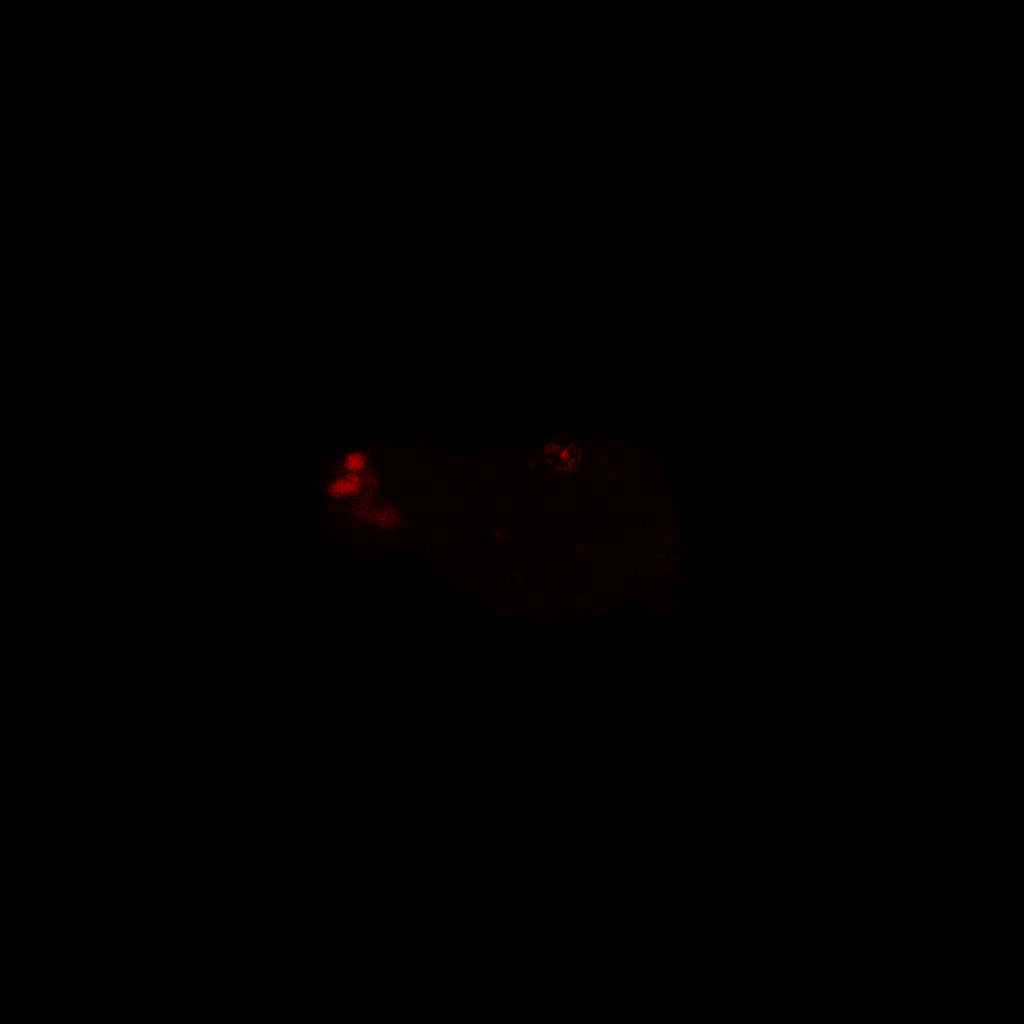

Supplement: Supplementary file 10 — Source data Fig. 8 [file 44318_2025_558_MOESM10_ESM.zip › Figure 8/panel 8G/KD-1+WAY_Nanog/seq8797_seq8797_RGB_Texas Red.tif]

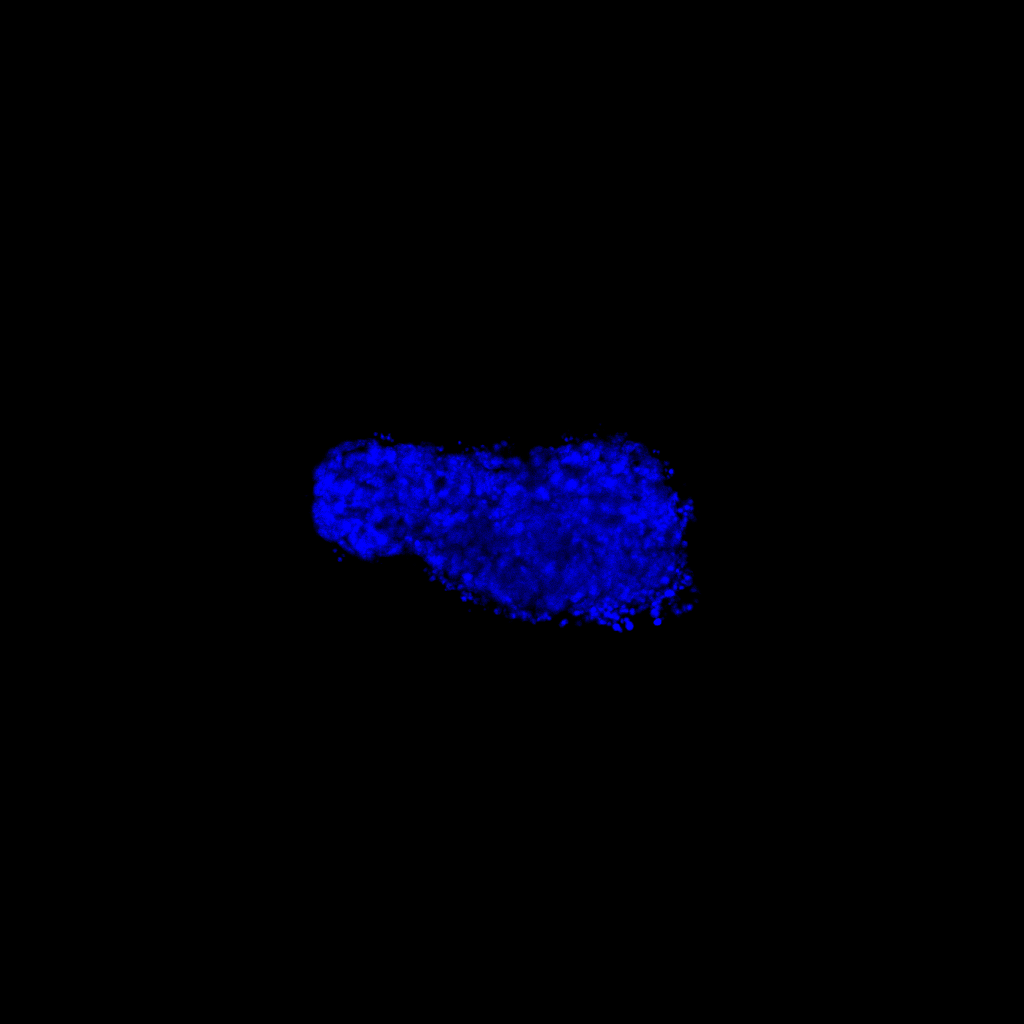

Supplement: Supplementary file 10 — Source data Fig. 8 [file 44318_2025_558_MOESM10_ESM.zip › Figure 8/panel 8G/KD-1+WAY_Nanog/seq8797_seq8797_RGB_DAPI.tif]

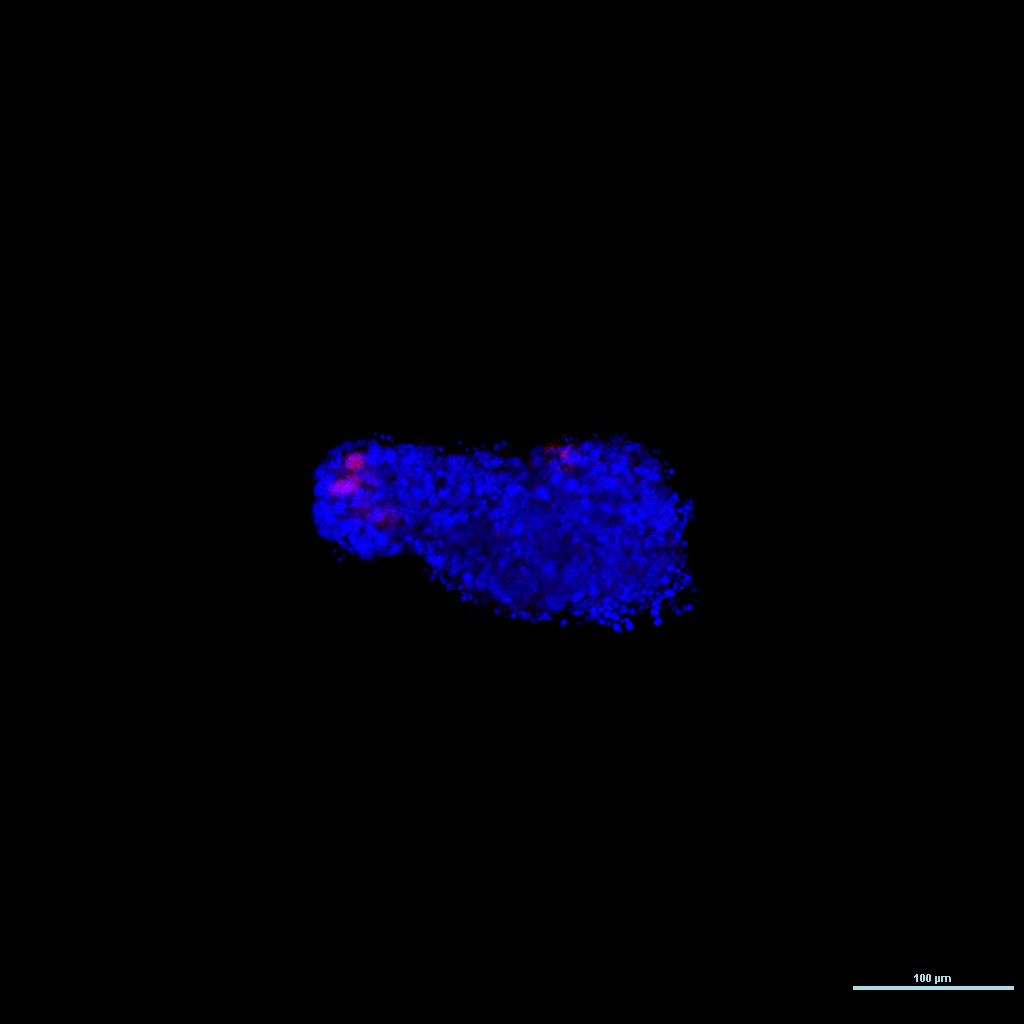

Supplement: Supplementary file 10 — Source data Fig. 8 [file 44318_2025_558_MOESM10_ESM.zip › Figure 8/panel 8G/KD-1+WAY_Nanog/seq8797_seq8797_RGB.tif]

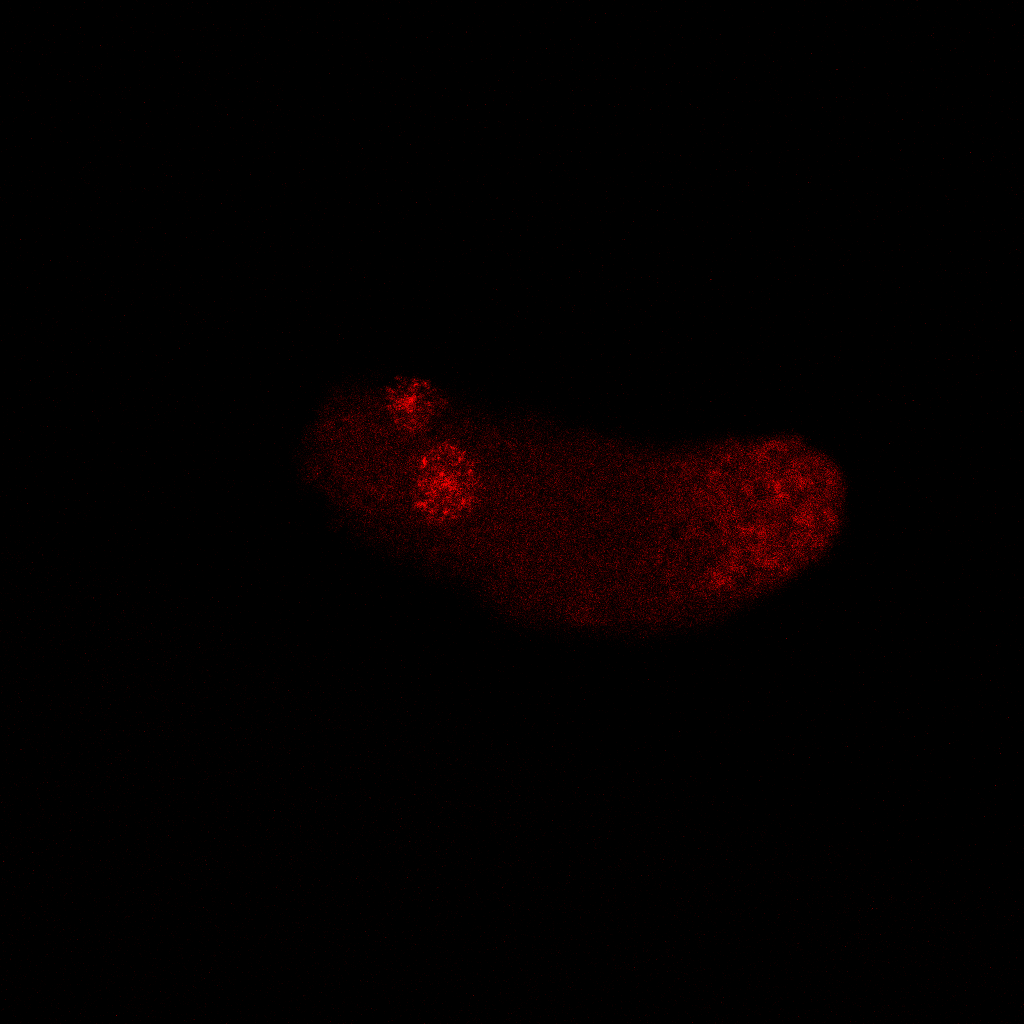

Supplement: Supplementary file 10 — Source data Fig. 8 [file 44318_2025_558_MOESM10_ESM.zip › Figure 8/panel 8G/KD-1+WAY_Bra/seq8960_seq8960_RGB_Texas Red.tif]

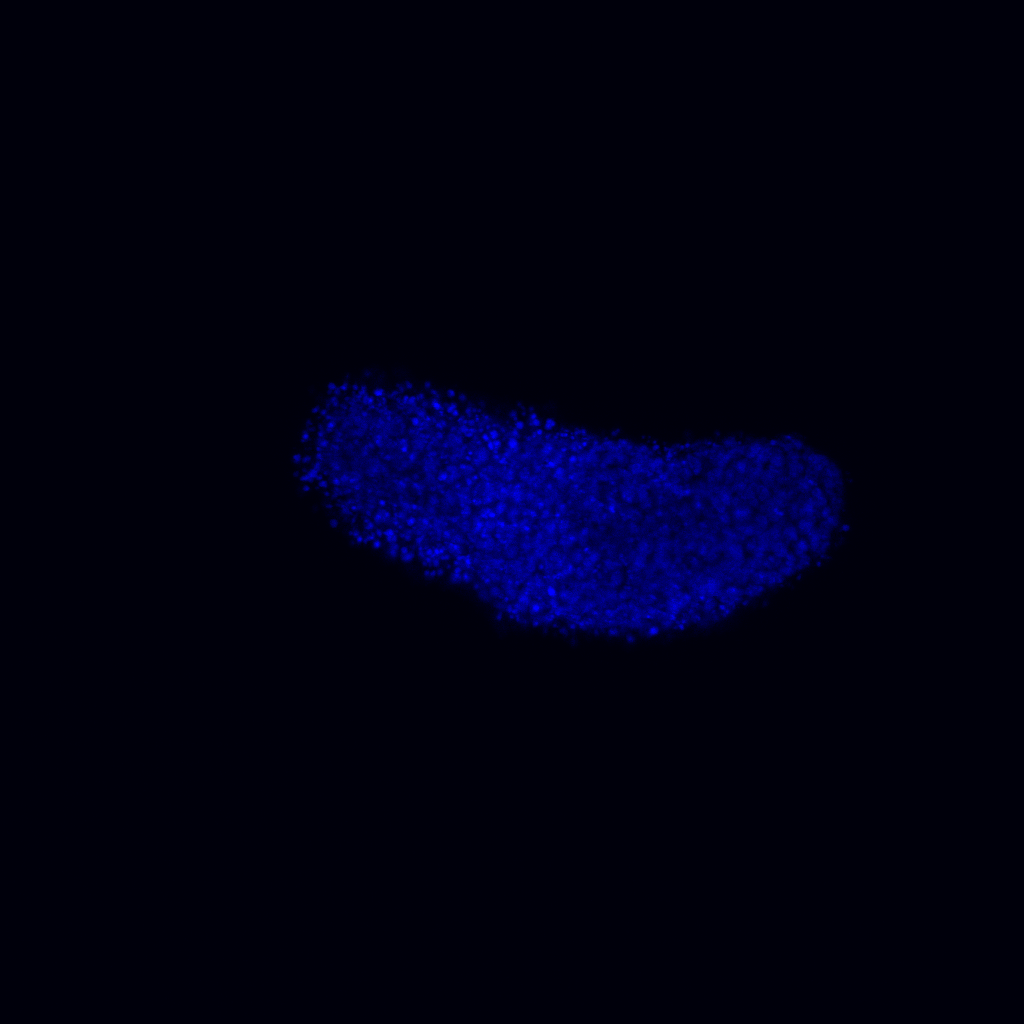

Supplement: Supplementary file 10 — Source data Fig. 8 [file 44318_2025_558_MOESM10_ESM.zip › Figure 8/panel 8G/KD-1+WAY_Bra/seq8960_seq8960_RGB_DAPI.tif]

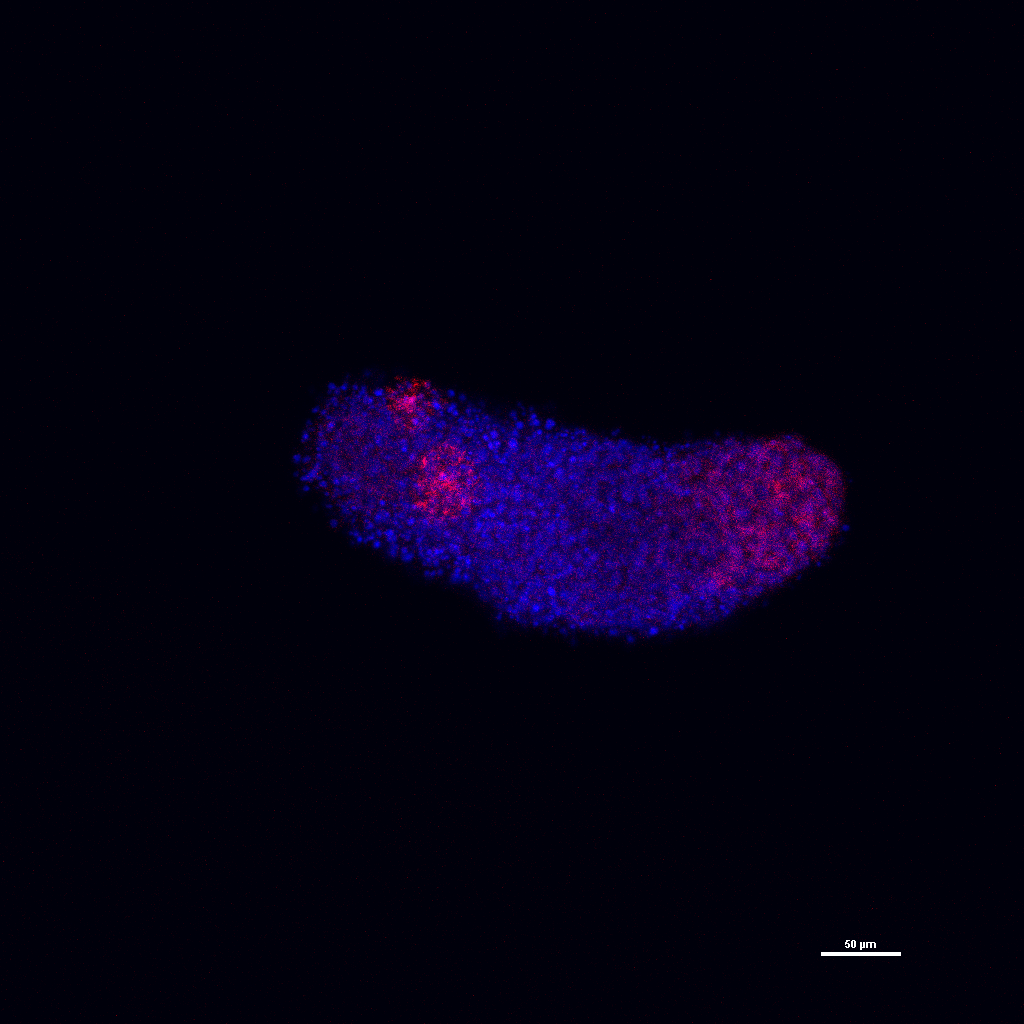

Supplement: Supplementary file 10 — Source data Fig. 8 [file 44318_2025_558_MOESM10_ESM.zip › Figure 8/panel 8G/KD-1+WAY_Bra/seq8960_seq8960_RGB.tif]
