# Supplementary figures and images for: Non-cell-autonomous control of mouse gastruloid development by the ultra-conserved lncRNA T-UCstem1 (part 6 of 6)
Source: EMBO J. 2025 Oct 31;44(24):7620–48. doi: 10.1038/s44318-025-00558-2 (PMC12706062; doi:10.1038/s44318-025-00558-2)

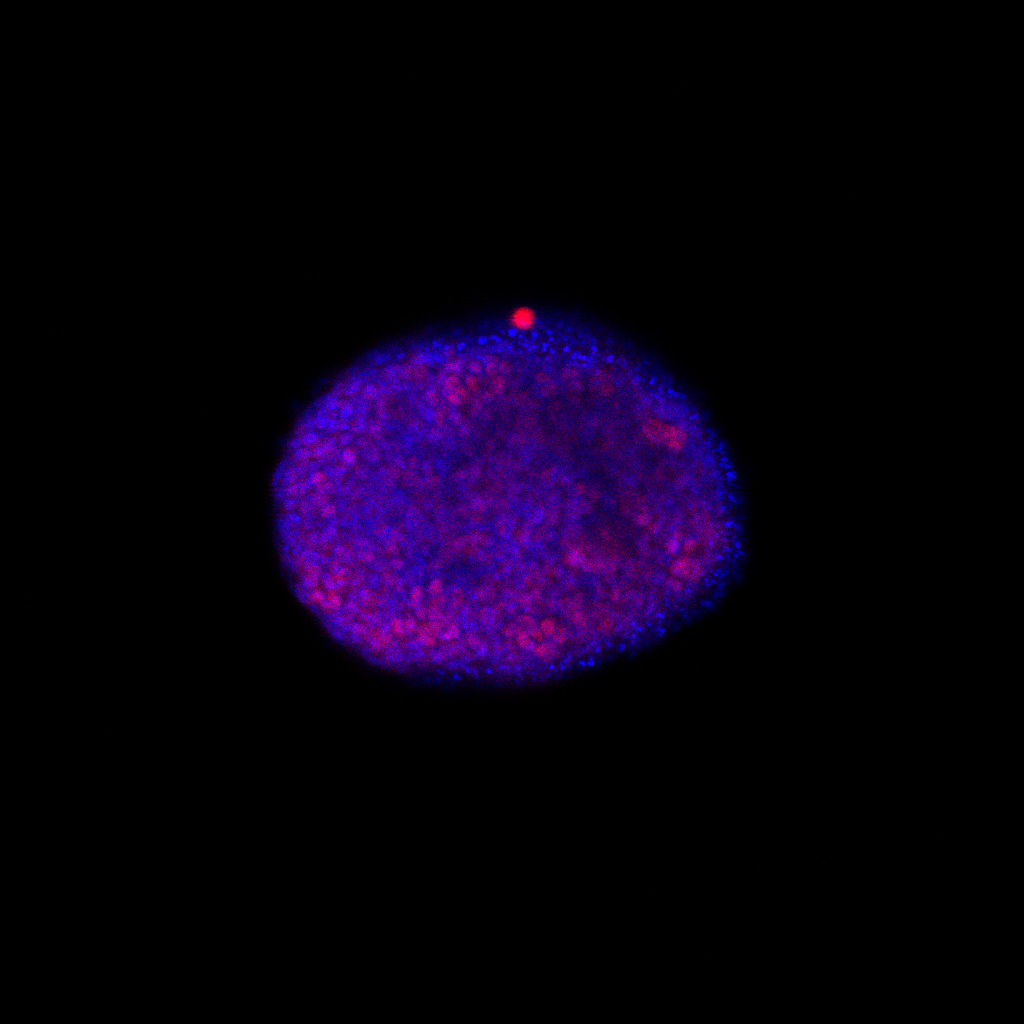

Supplement: Supplementary file 10 — Source data Fig. 8 [file 44318_2025_558_MOESM10_ESM.zip › Figure 8/panel 8G/KD-1_Oct4/seq8692.tif]

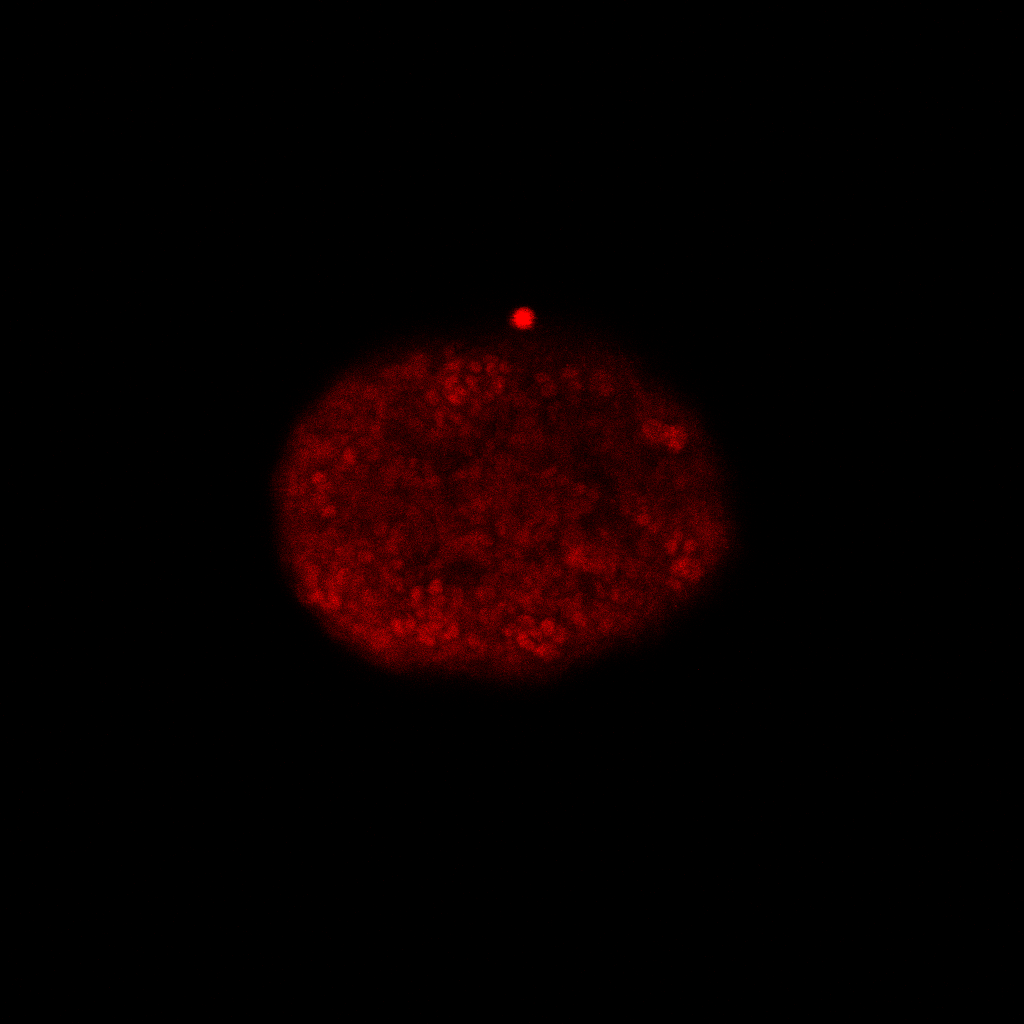

Supplement: Supplementary file 10 — Source data Fig. 8 [file 44318_2025_558_MOESM10_ESM.zip › Figure 8/panel 8G/KD-1_Oct4/seq8692c2.tif]

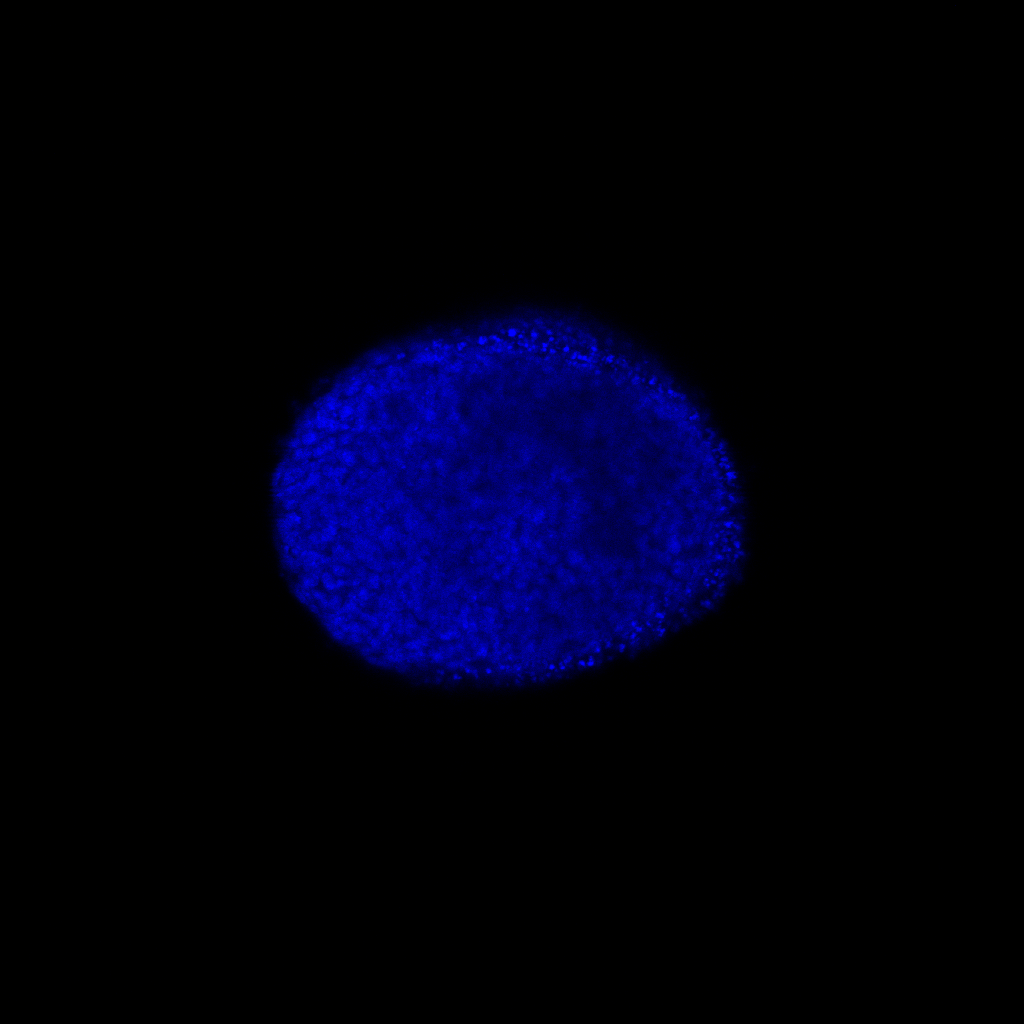

Supplement: Supplementary file 10 — Source data Fig. 8 [file 44318_2025_558_MOESM10_ESM.zip › Figure 8/panel 8G/KD-1_Oct4/seq8692c1.tif]

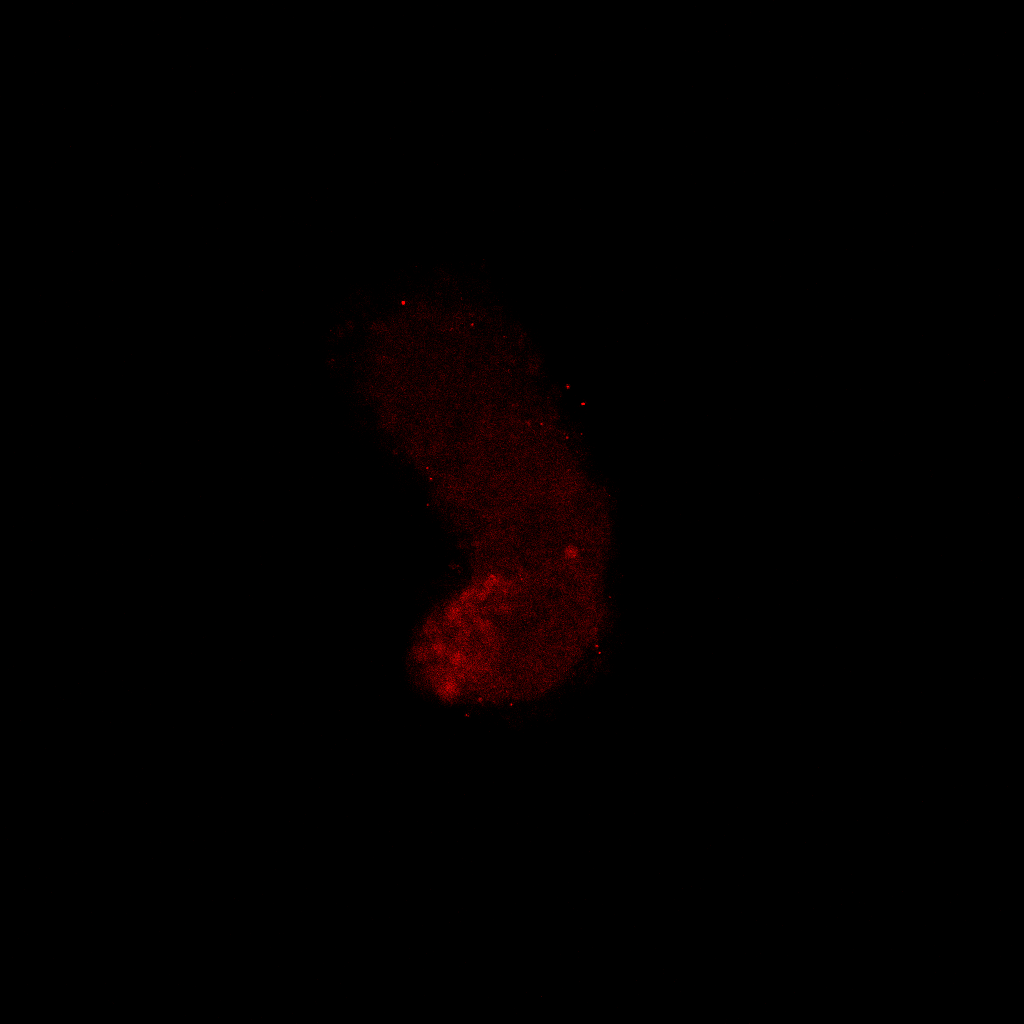

Supplement: Supplementary file 10 — Source data Fig. 8 [file 44318_2025_558_MOESM10_ESM.zip › Figure 8/panel 8G/KD-2+WAY_Oct4/seq8974_seq8974_RGB_Texas Red.tif]

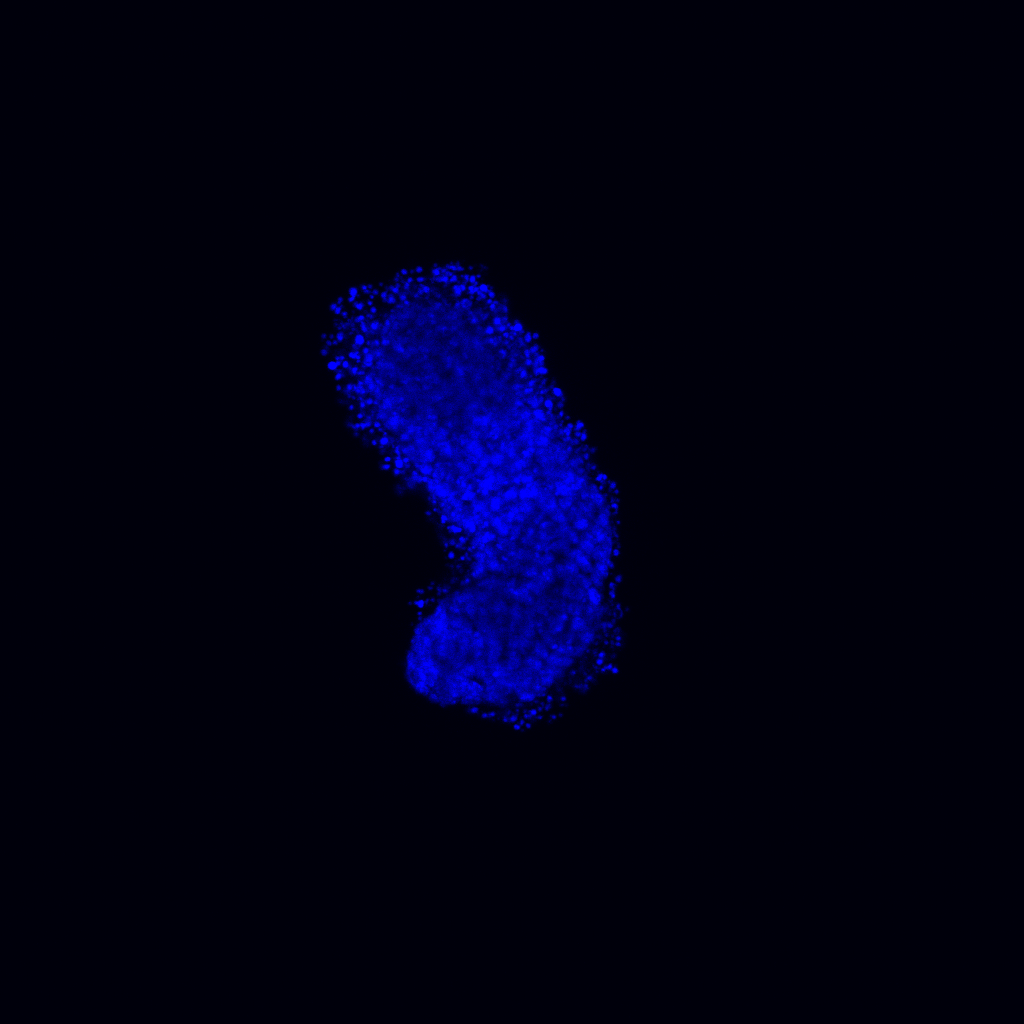

Supplement: Supplementary file 10 — Source data Fig. 8 [file 44318_2025_558_MOESM10_ESM.zip › Figure 8/panel 8G/KD-2+WAY_Oct4/seq8974_seq8974_RGB_DAPI.tif]

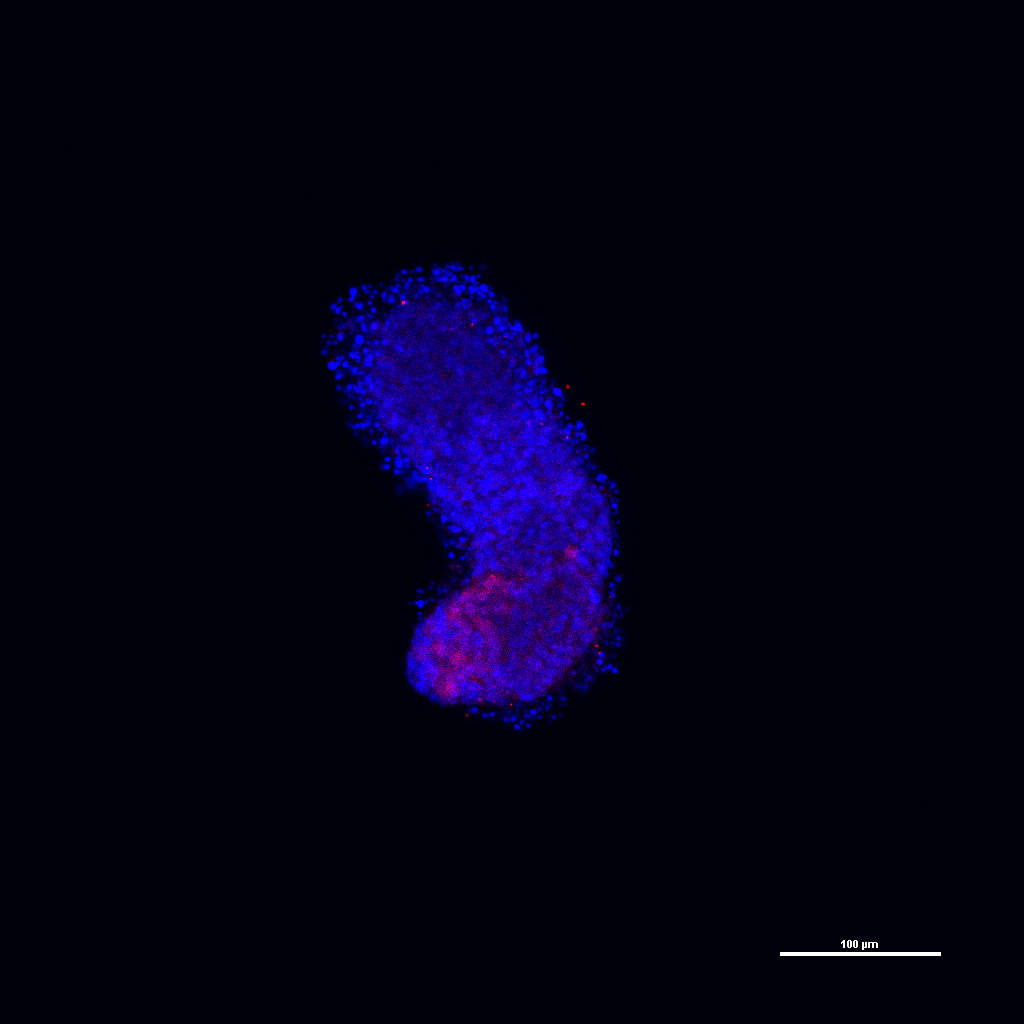

Supplement: Supplementary file 10 — Source data Fig. 8 [file 44318_2025_558_MOESM10_ESM.zip › Figure 8/panel 8G/KD-2+WAY_Oct4/seq8974_seq8974_RGB.tif]

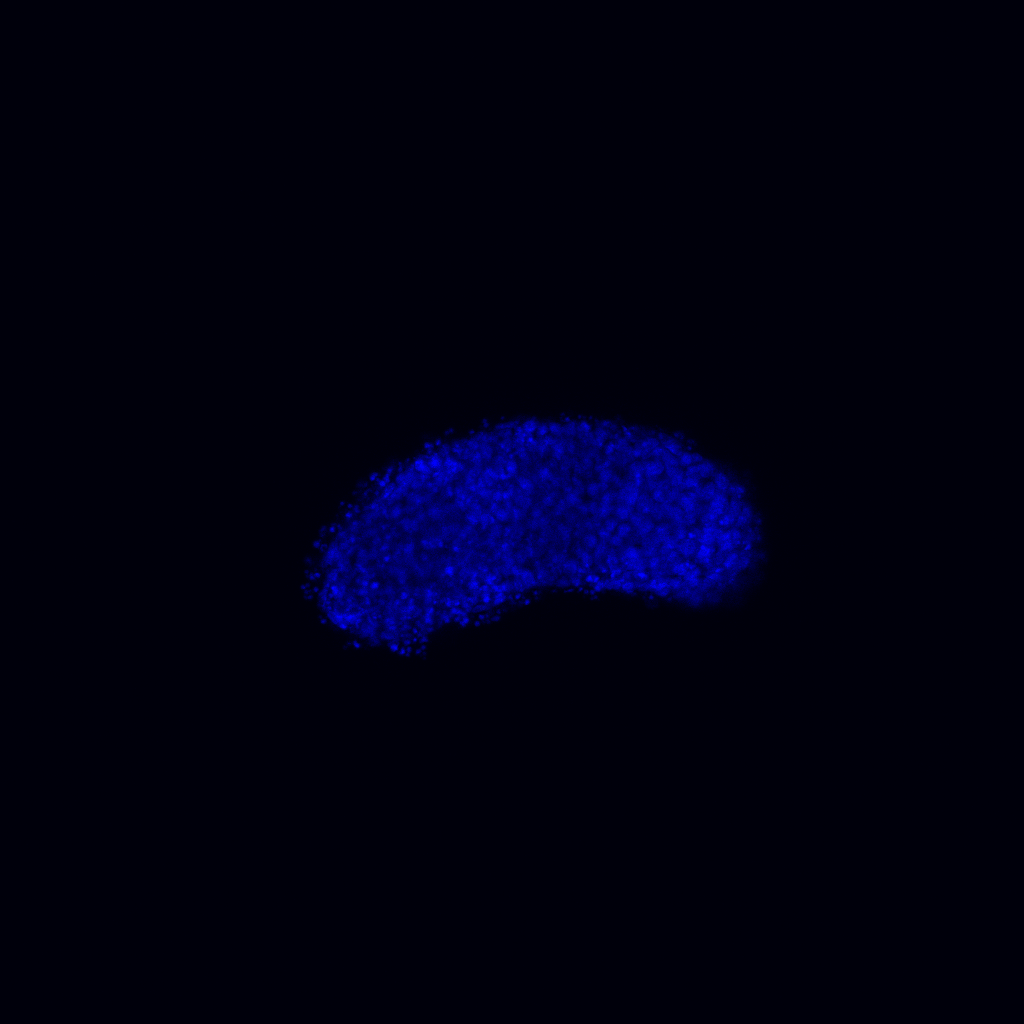

Supplement: Supplementary file 10 — Source data Fig. 8 [file 44318_2025_558_MOESM10_ESM.zip › Figure 8/panel 8G/KD-2+WAY_Bra/seq8967_seq8967_RGB_DAPI.tif]

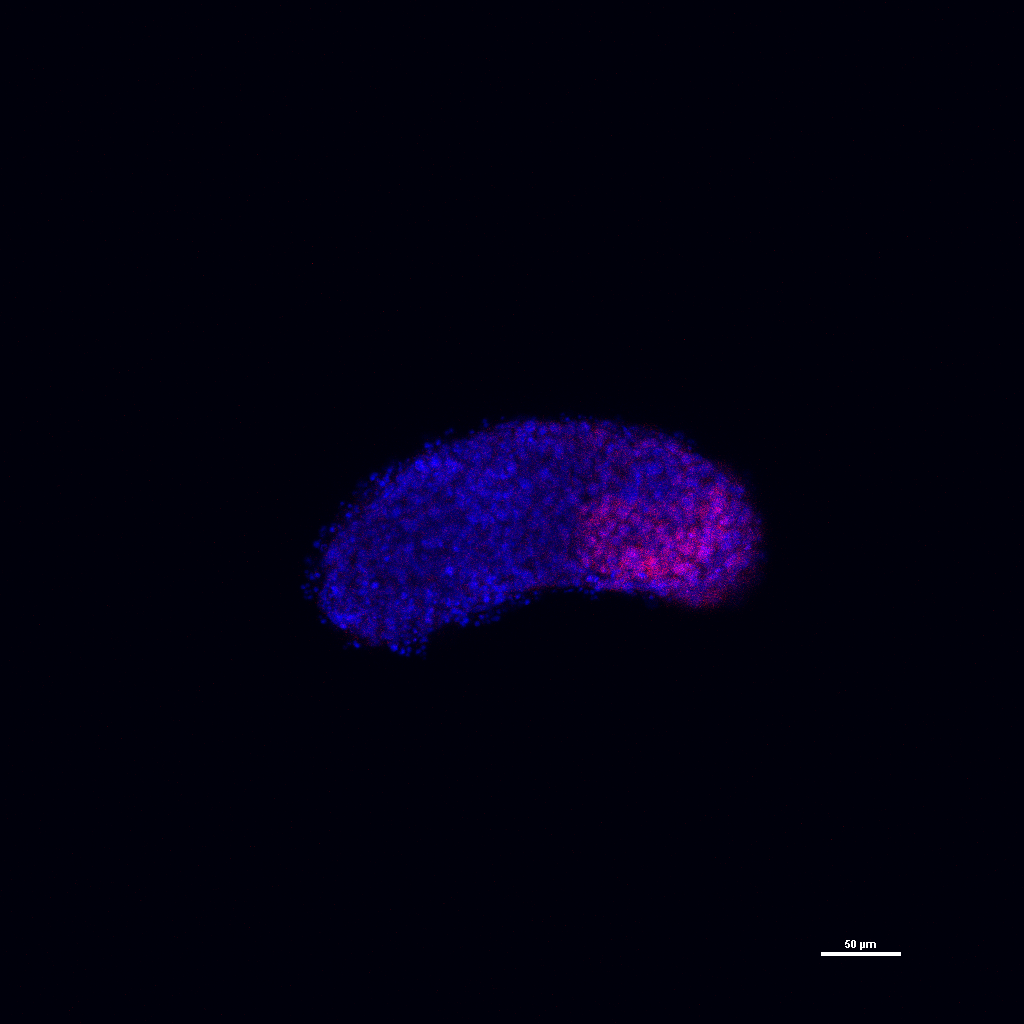

Supplement: Supplementary file 10 — Source data Fig. 8 [file 44318_2025_558_MOESM10_ESM.zip › Figure 8/panel 8G/KD-2+WAY_Bra/seq8967_seq8967_RGB.tif]

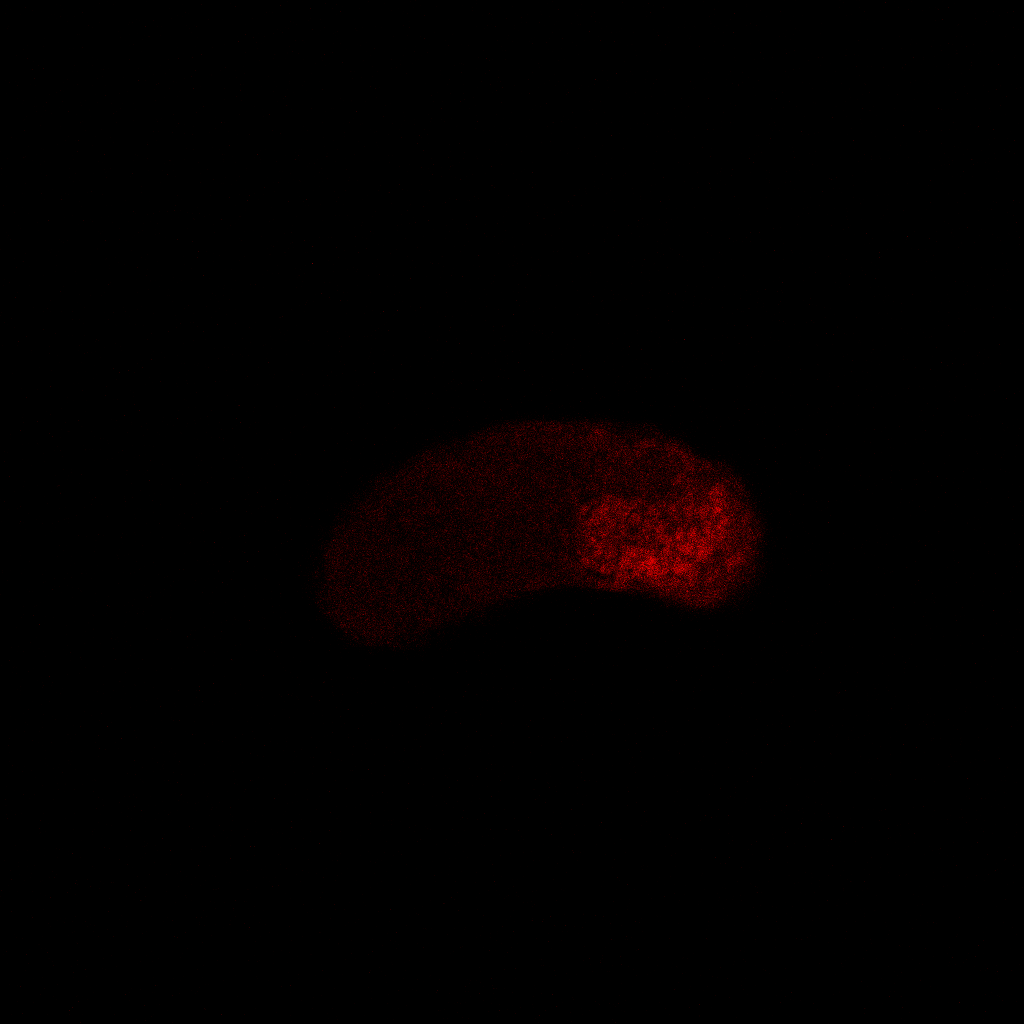

Supplement: Supplementary file 10 — Source data Fig. 8 [file 44318_2025_558_MOESM10_ESM.zip › Figure 8/panel 8G/KD-2+WAY_Bra/seq8967_seq8967_RGB_Texas Red.tif]

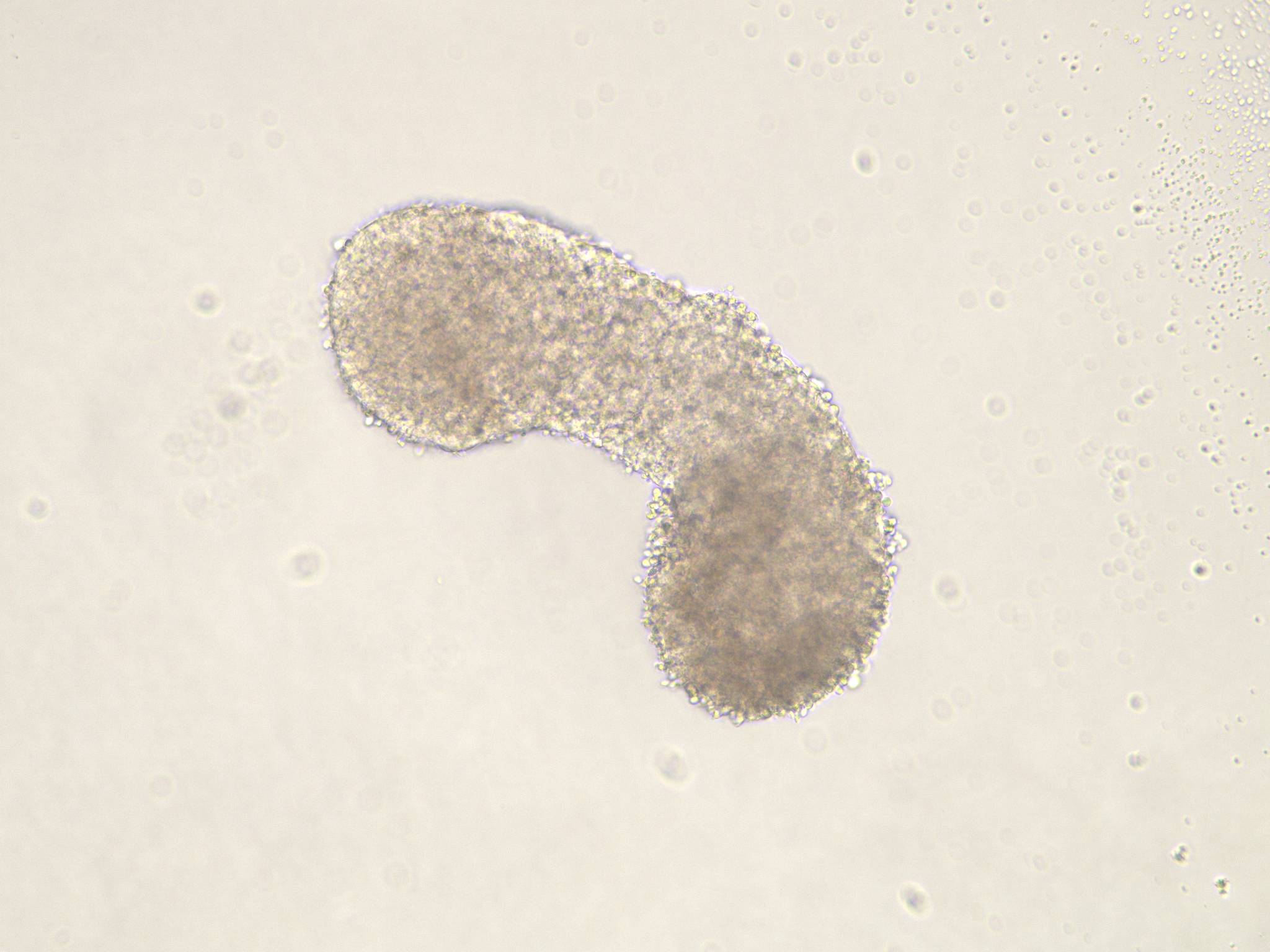

Supplement: Supplementary file 11 — Source data Fig. 9 [file 44318_2025_558_MOESM11_ESM.zip › Figure 9/panel 9B/KD-2 UC_1.tiff]

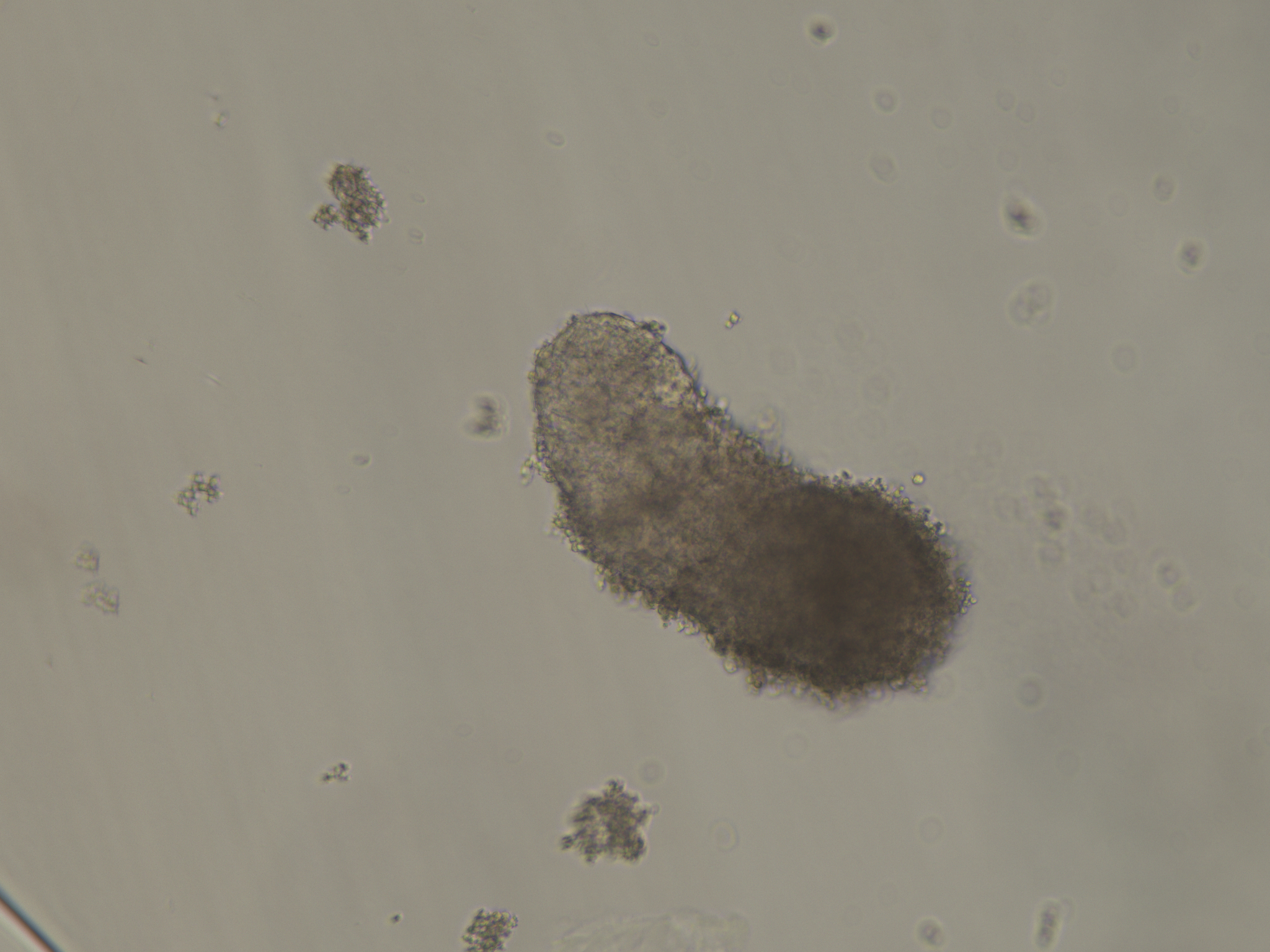

Supplement: Supplementary file 11 — Source data Fig. 9 [file 44318_2025_558_MOESM11_ESM.zip › Figure 9/panel 9B/NT_3.tiff]

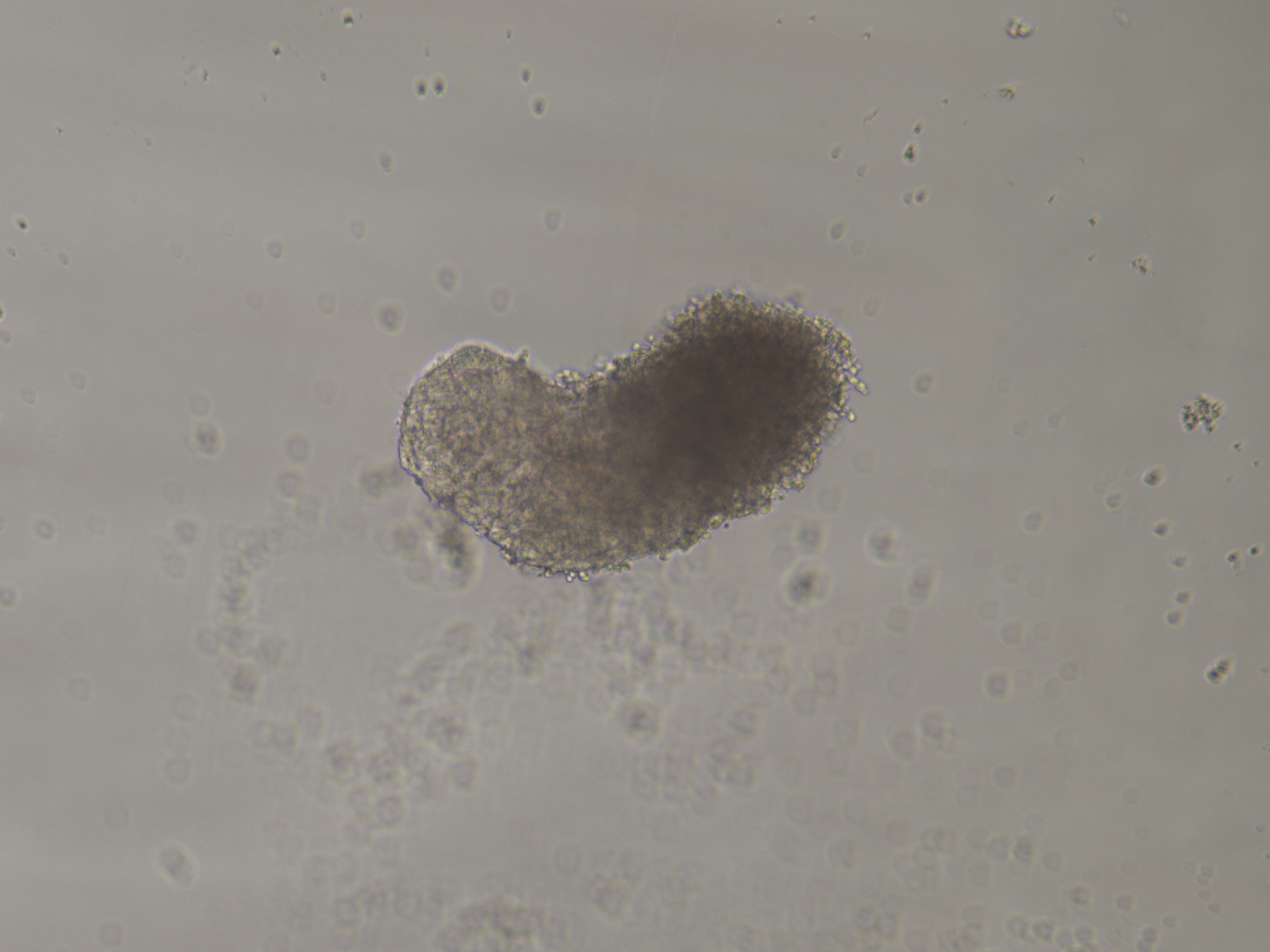

Supplement: Supplementary file 11 — Source data Fig. 9 [file 44318_2025_558_MOESM11_ESM.zip › Figure 9/panel 9B/KD-1 UC_3.tiff]

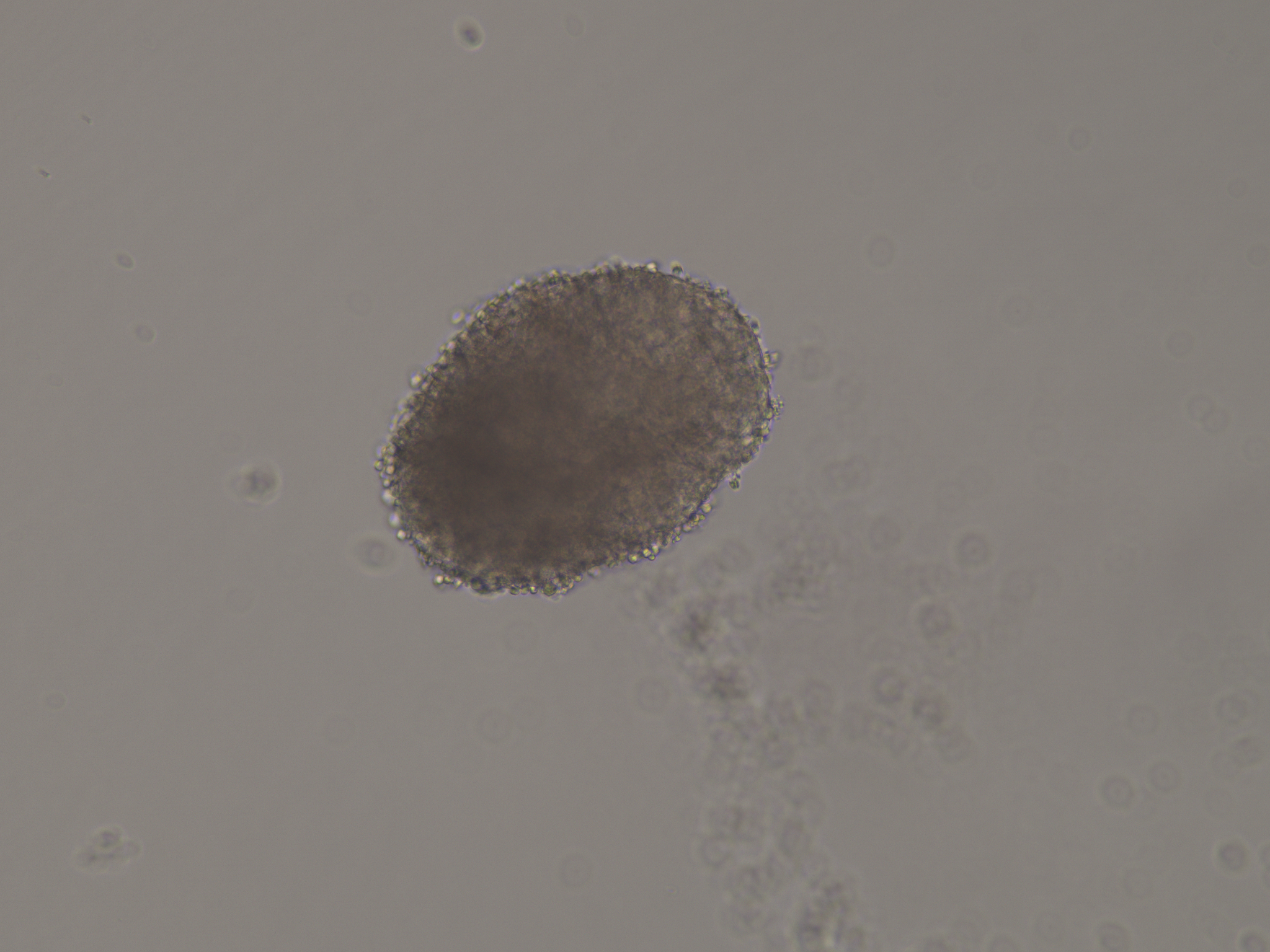

Supplement: Supplementary file 11 — Source data Fig. 9 [file 44318_2025_558_MOESM11_ESM.zip › Figure 9/panel 9B/KD-1_1.tiff]

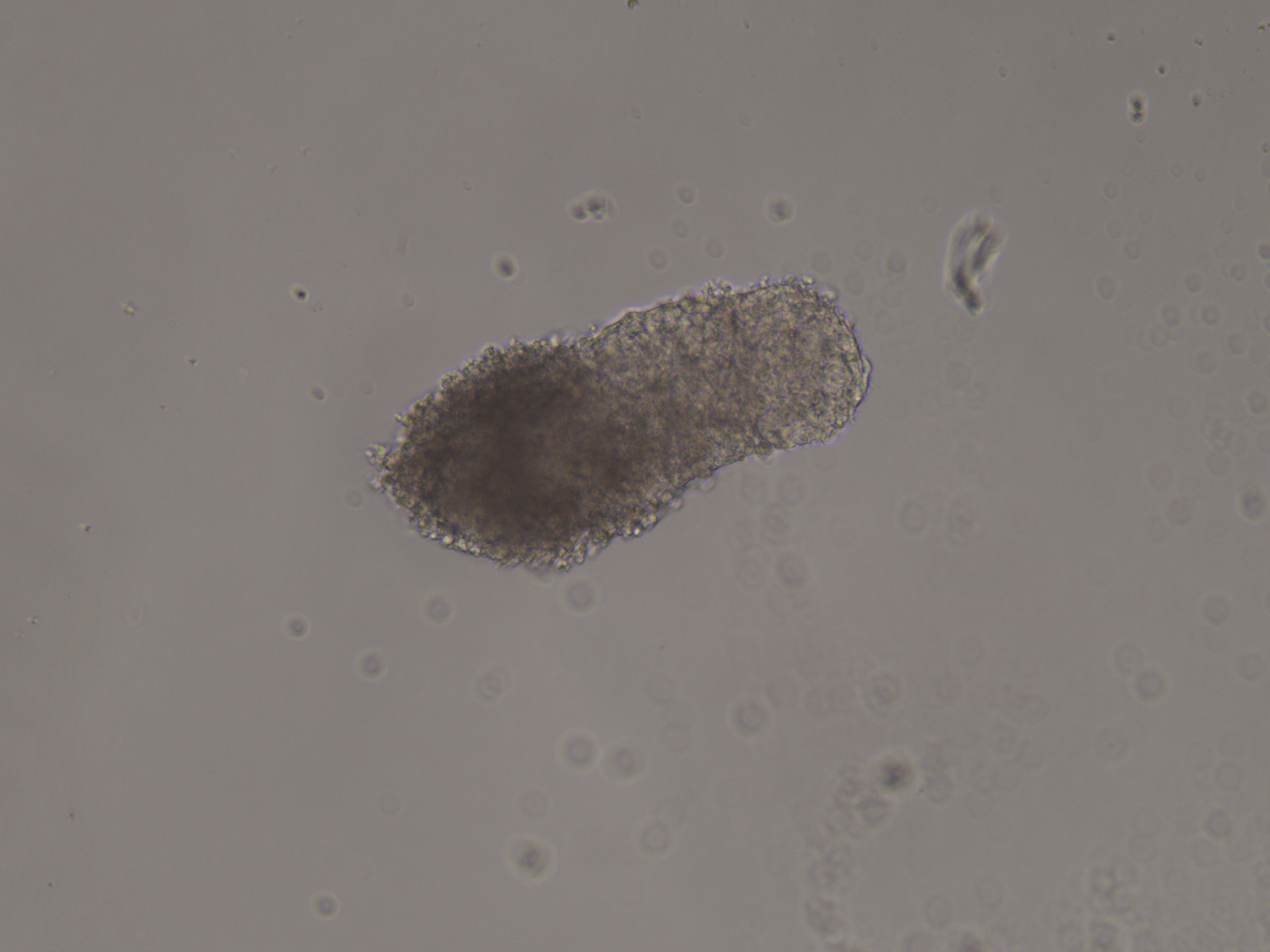

Supplement: Supplementary file 11 — Source data Fig. 9 [file 44318_2025_558_MOESM11_ESM.zip › Figure 9/panel 9B/KD-1 UC_2.tiff]

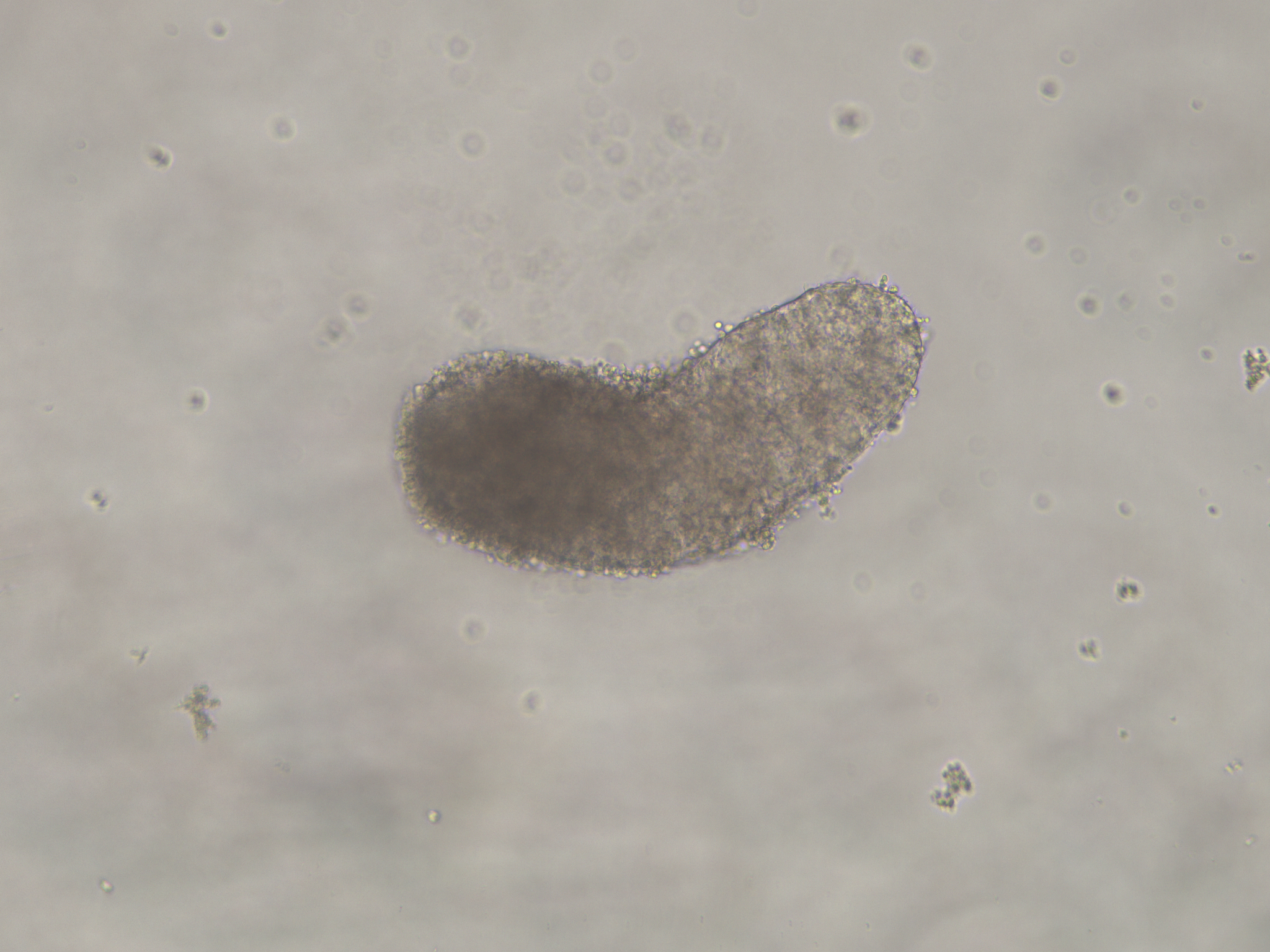

Supplement: Supplementary file 11 — Source data Fig. 9 [file 44318_2025_558_MOESM11_ESM.zip › Figure 9/panel 9B/NT_2.tiff]

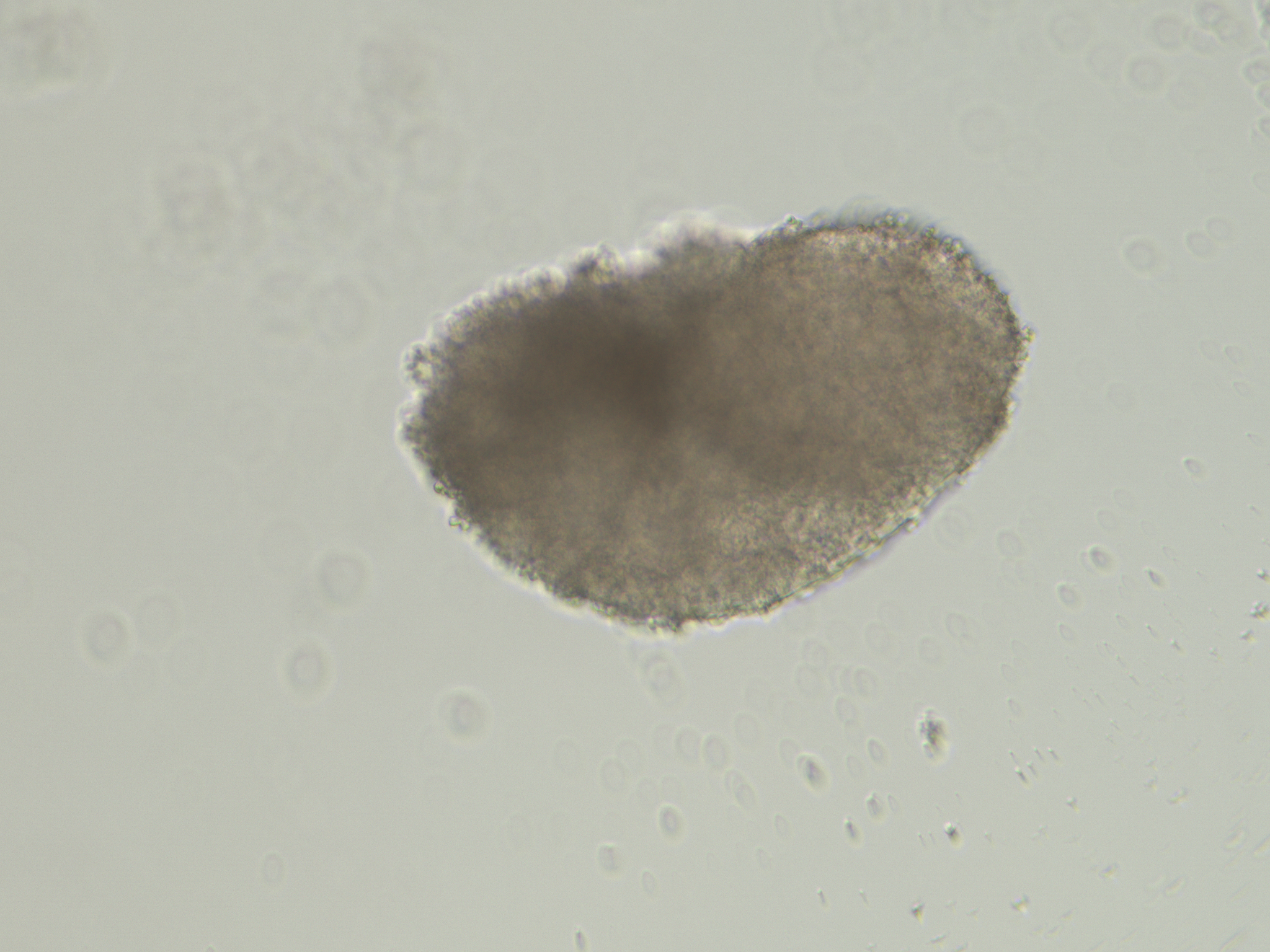

Supplement: Supplementary file 11 — Source data Fig. 9 [file 44318_2025_558_MOESM11_ESM.zip › Figure 9/panel 9B/KD-2_2.tiff]

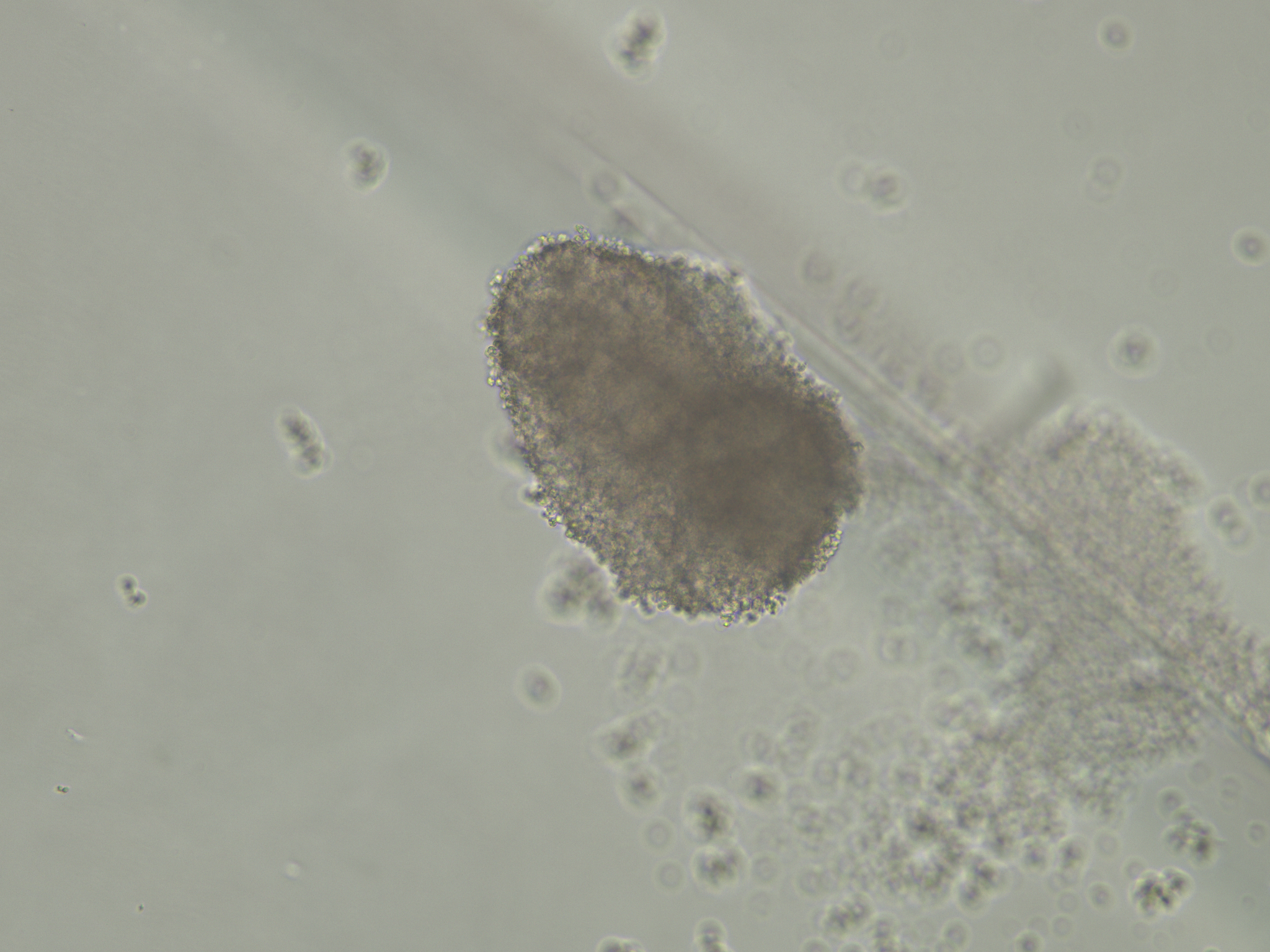

Supplement: Supplementary file 11 — Source data Fig. 9 [file 44318_2025_558_MOESM11_ESM.zip › Figure 9/panel 9B/KD-2 GFP_3.tiff]

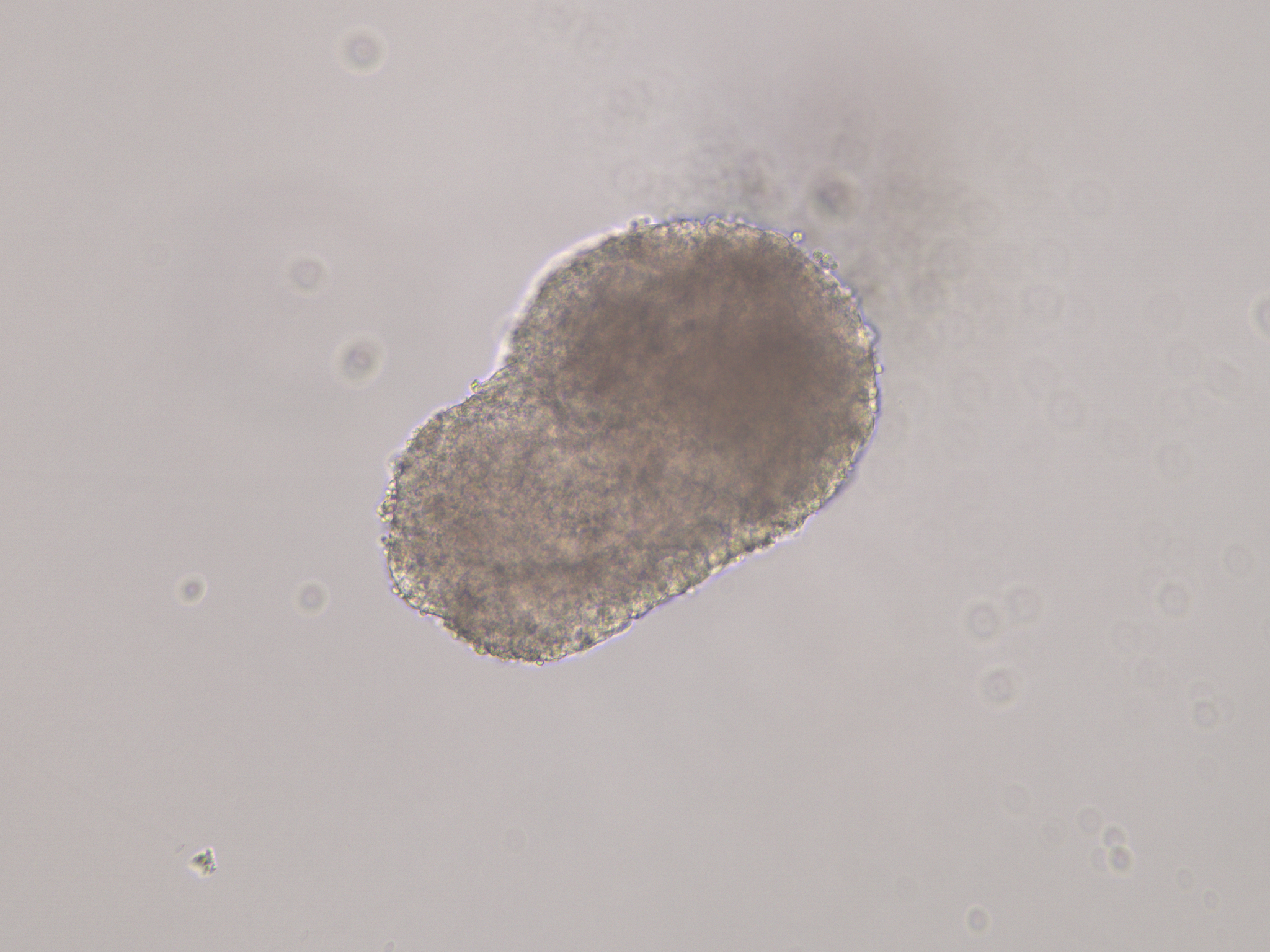

Supplement: Supplementary file 11 — Source data Fig. 9 [file 44318_2025_558_MOESM11_ESM.zip › Figure 9/panel 9B/KD-2 GFP_2.tiff]

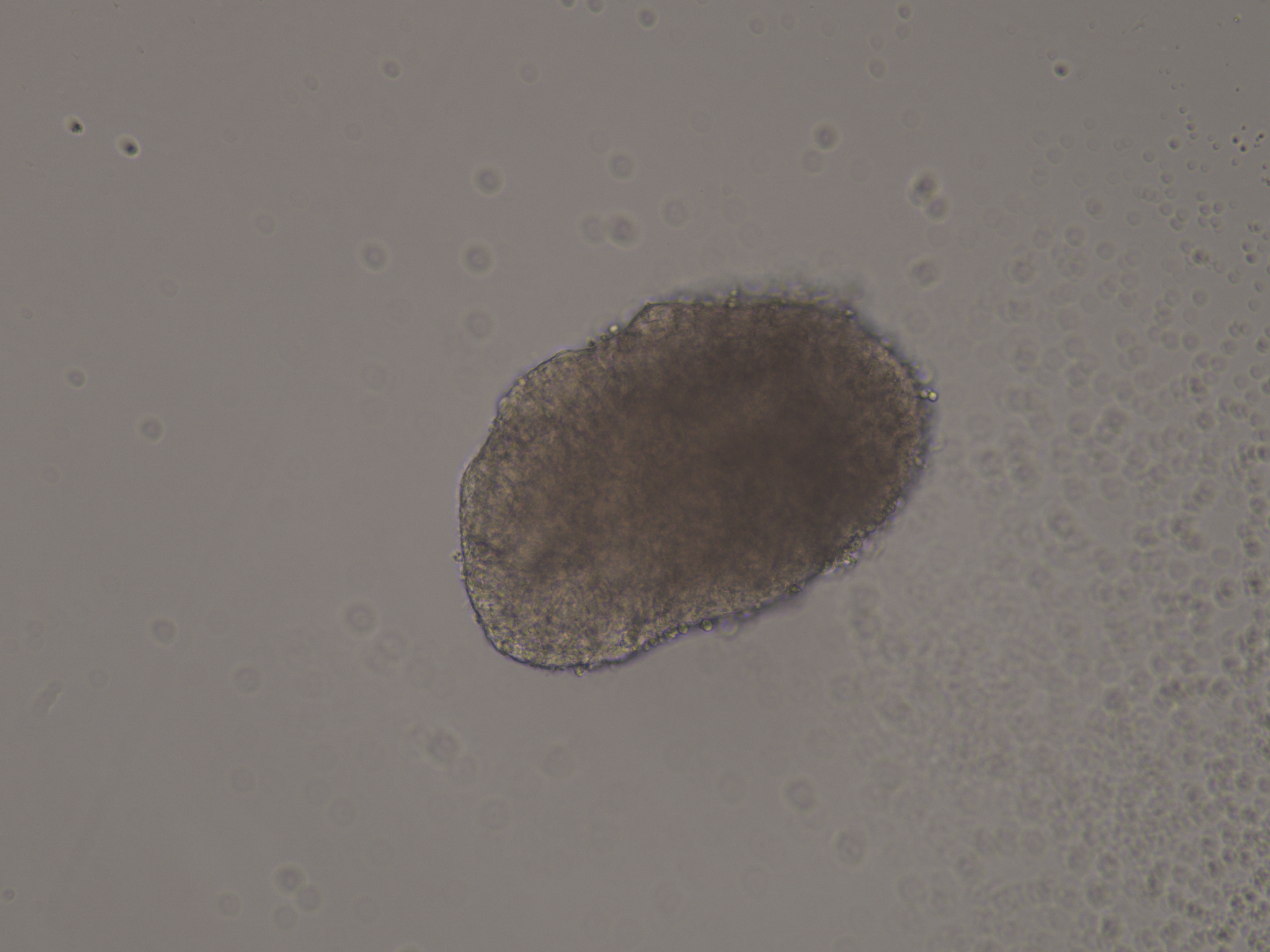

Supplement: Supplementary file 11 — Source data Fig. 9 [file 44318_2025_558_MOESM11_ESM.zip › Figure 9/panel 9B/KD-1 GFP_1.tiff]

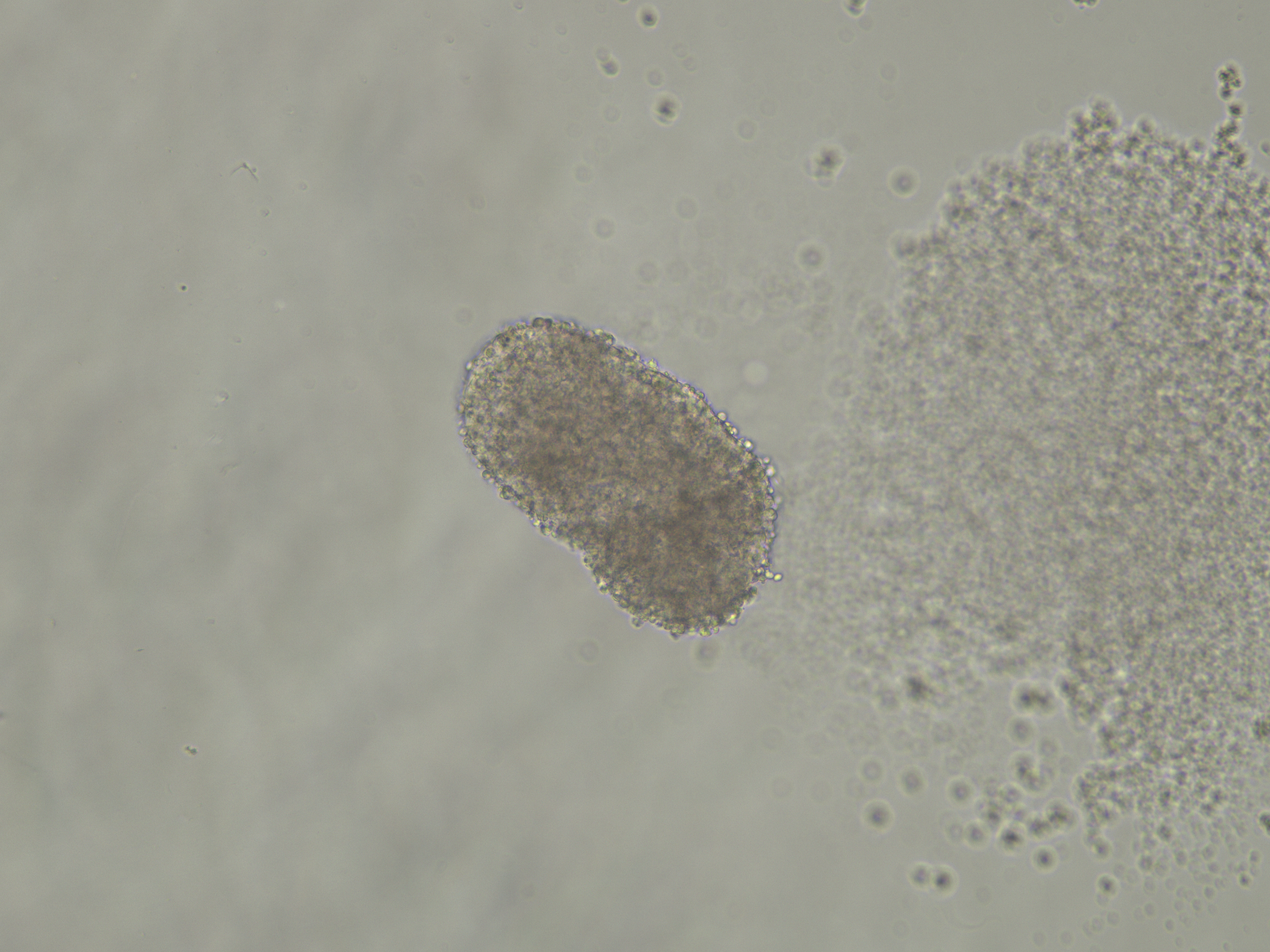

Supplement: Supplementary file 11 — Source data Fig. 9 [file 44318_2025_558_MOESM11_ESM.zip › Figure 9/panel 9B/KD-2_3.tiff]

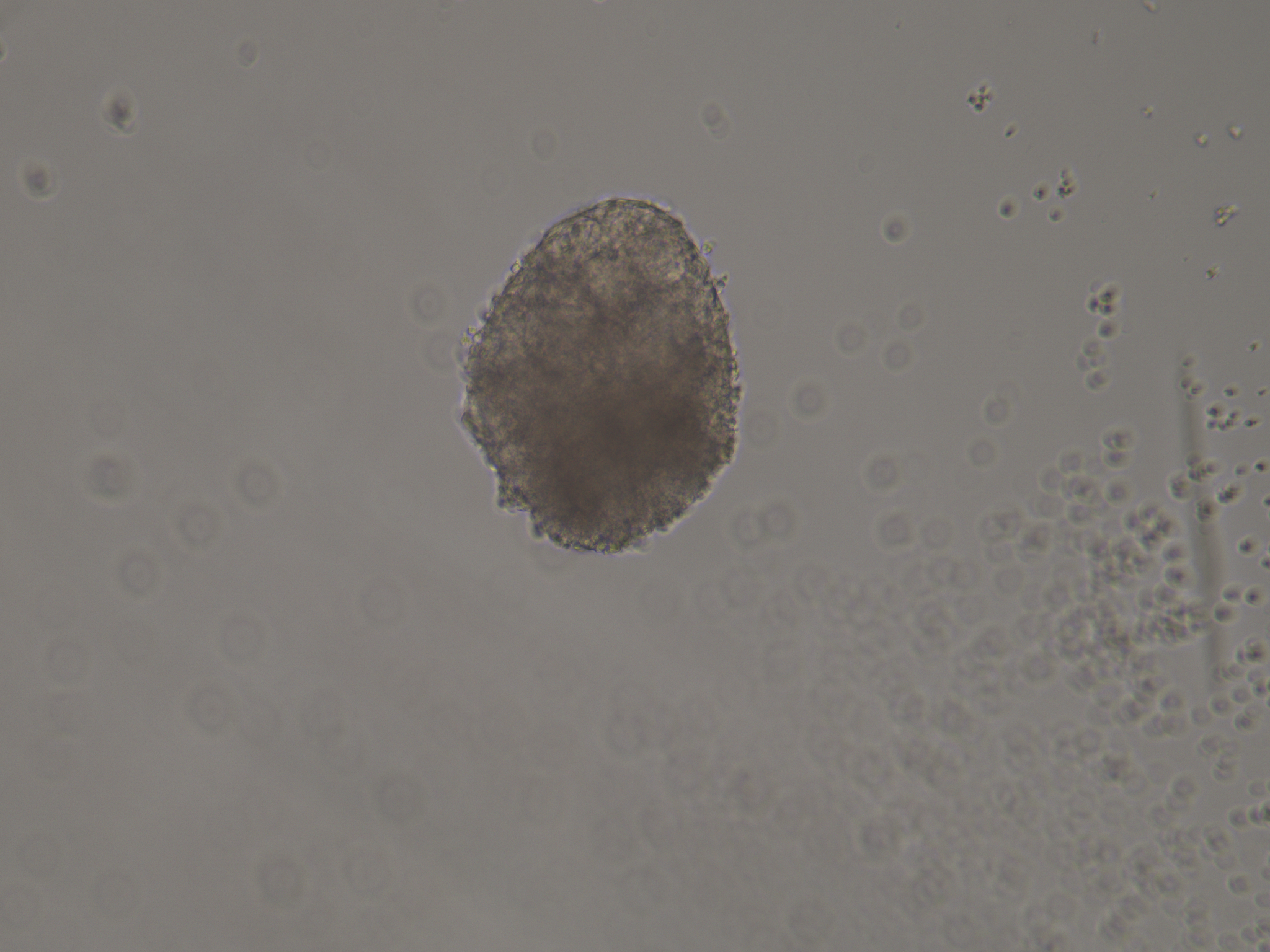

Supplement: Supplementary file 11 — Source data Fig. 9 [file 44318_2025_558_MOESM11_ESM.zip › Figure 9/panel 9B/KD-1 GFP_2.tiff]

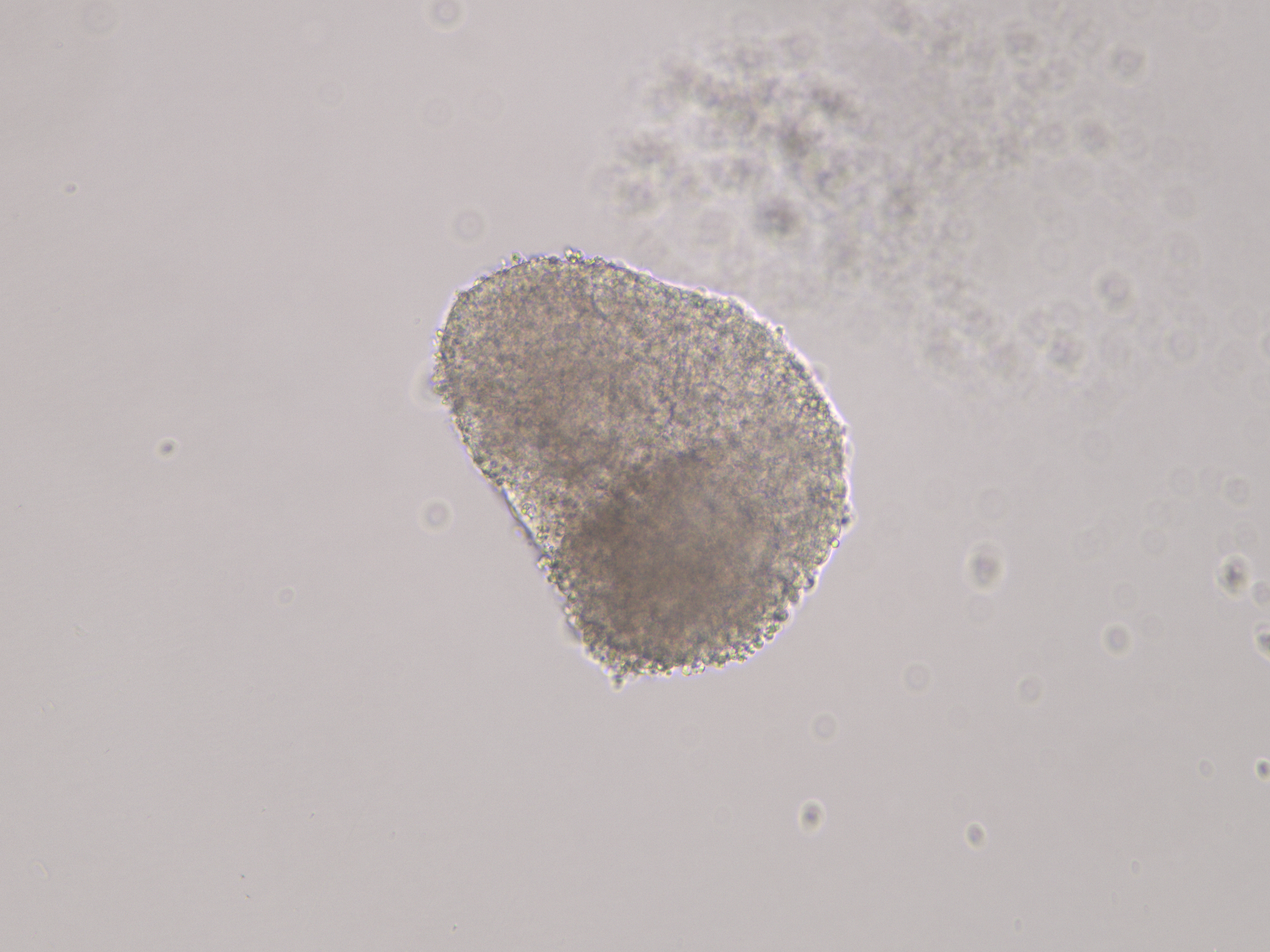

Supplement: Supplementary file 11 — Source data Fig. 9 [file 44318_2025_558_MOESM11_ESM.zip › Figure 9/panel 9B/KD-2 GFP_1.tiff]

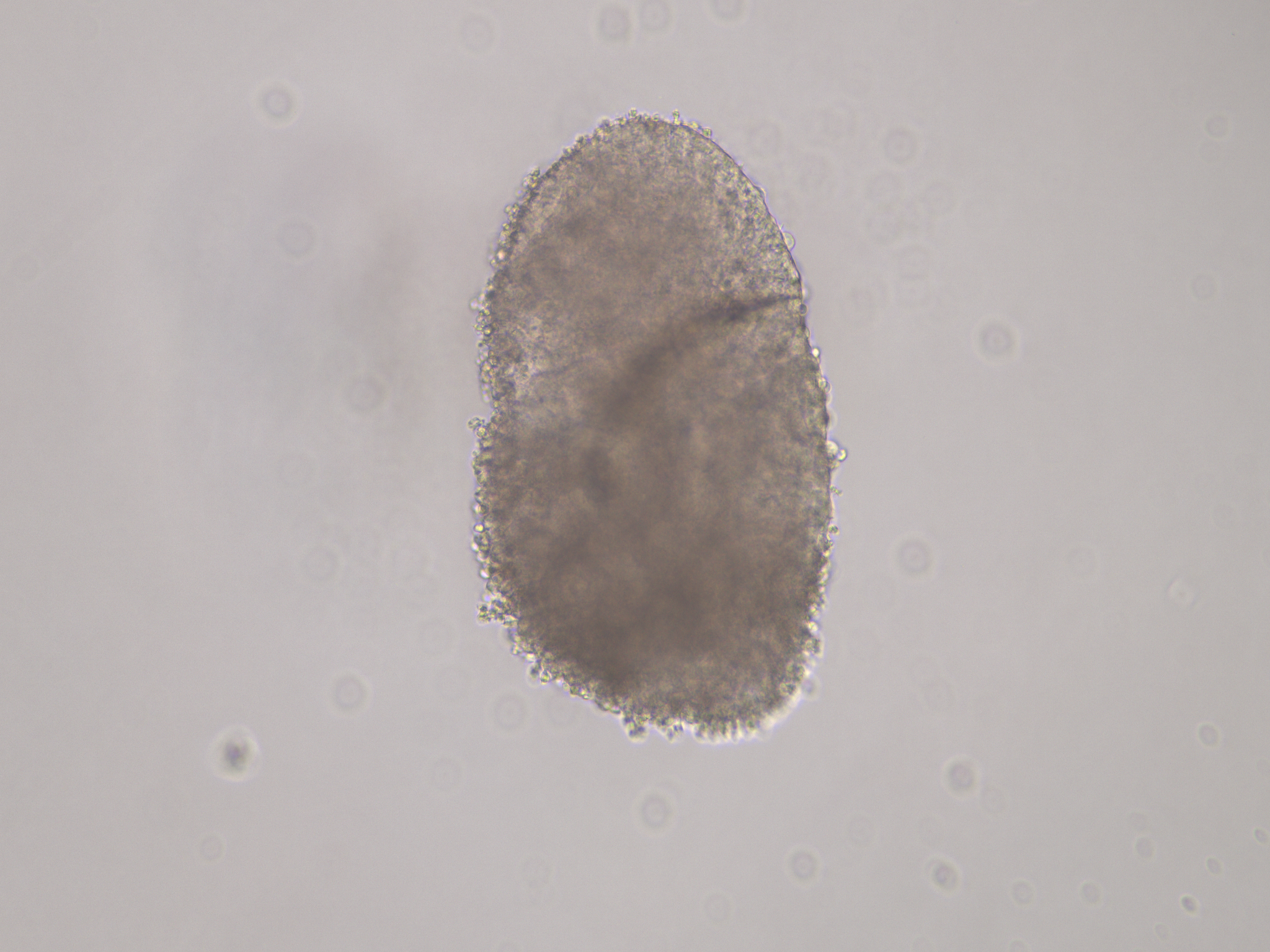

Supplement: Supplementary file 11 — Source data Fig. 9 [file 44318_2025_558_MOESM11_ESM.zip › Figure 9/panel 9B/KD-2_1.tiff]

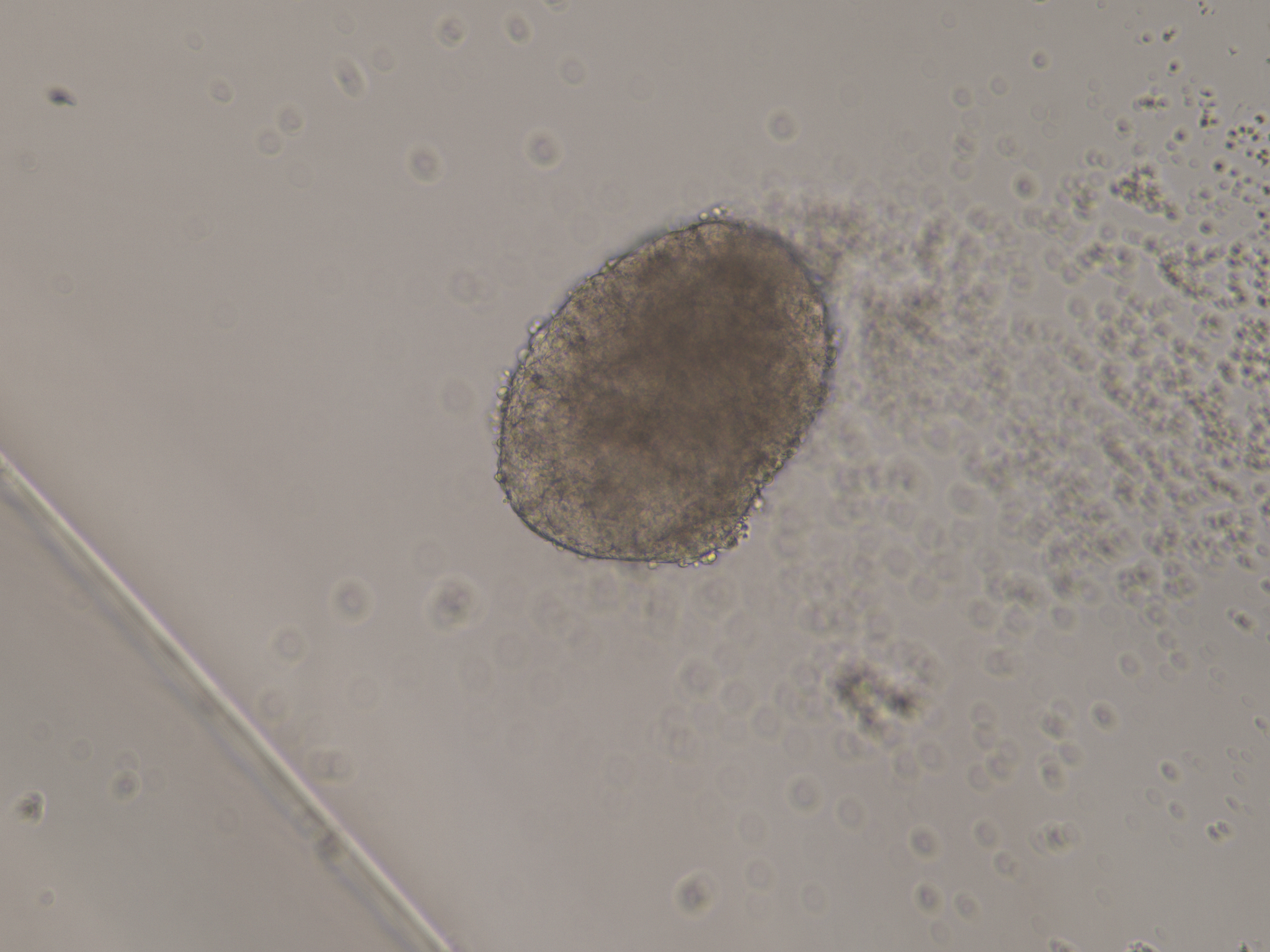

Supplement: Supplementary file 11 — Source data Fig. 9 [file 44318_2025_558_MOESM11_ESM.zip › Figure 9/panel 9B/KD-1 GFP_3.tiff]

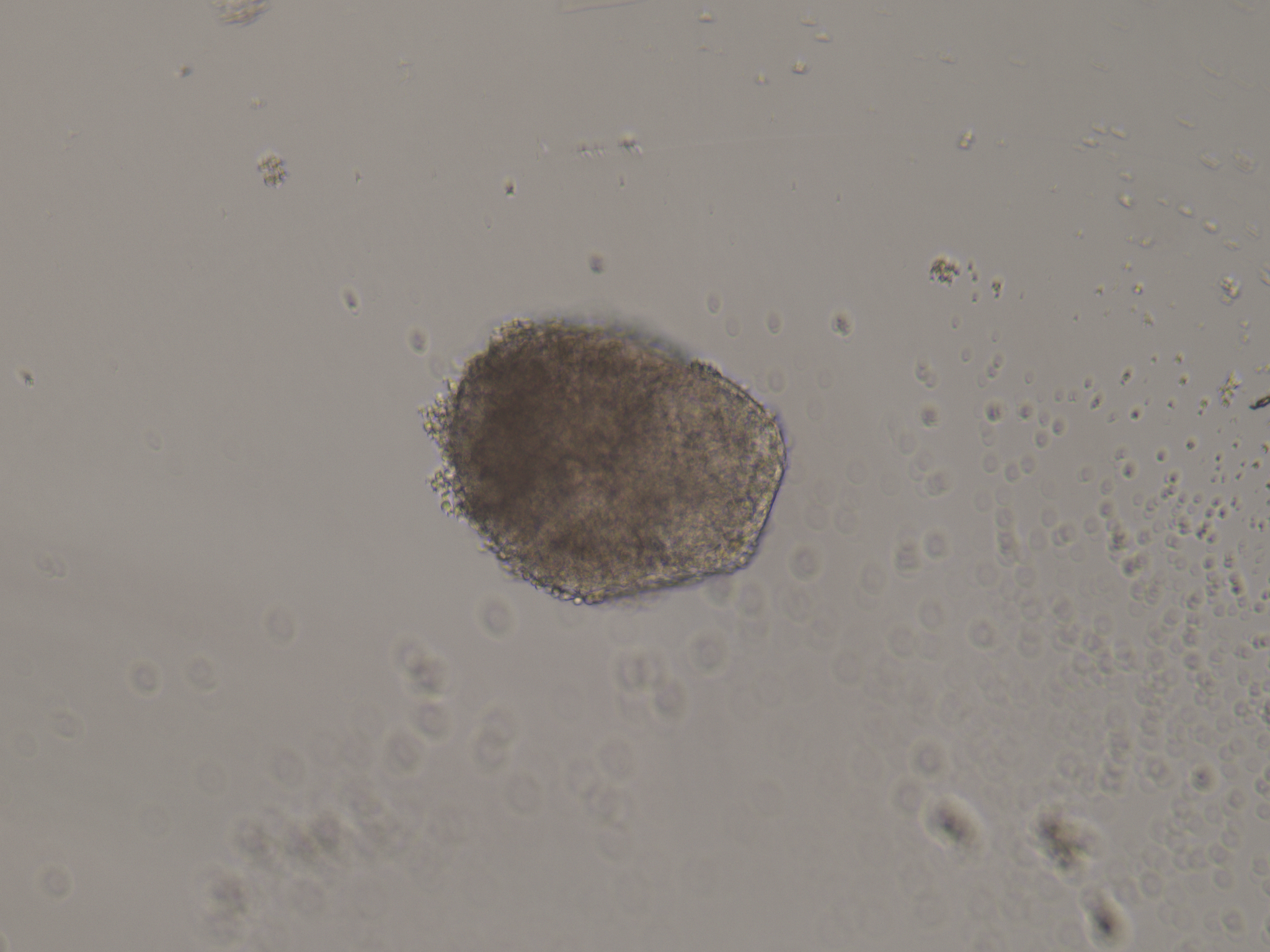

Supplement: Supplementary file 11 — Source data Fig. 9 [file 44318_2025_558_MOESM11_ESM.zip › Figure 9/panel 9B/KD-1_3.tiff]

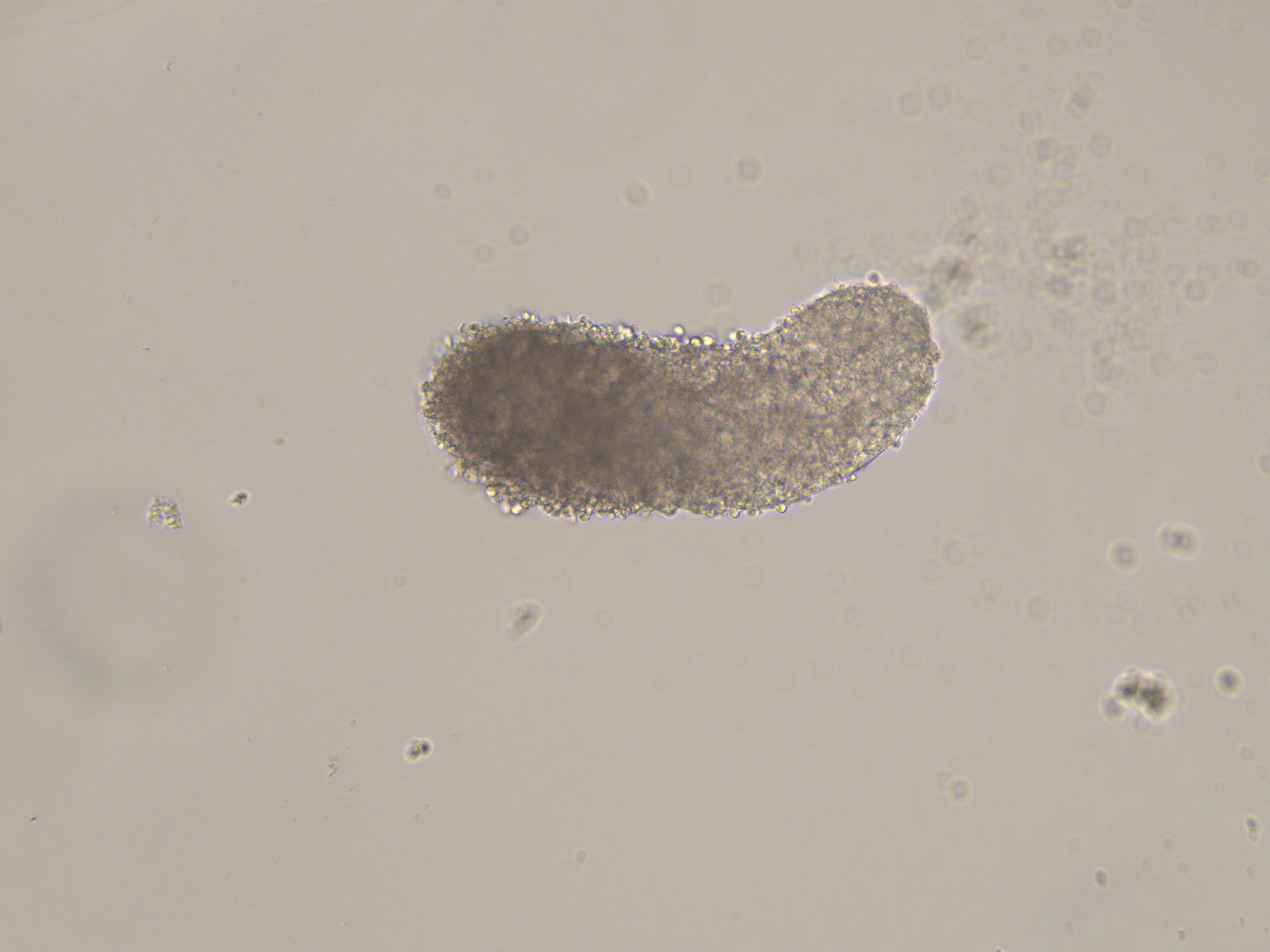

Supplement: Supplementary file 11 — Source data Fig. 9 [file 44318_2025_558_MOESM11_ESM.zip › Figure 9/panel 9B/KD-1 UC_1.tiff]

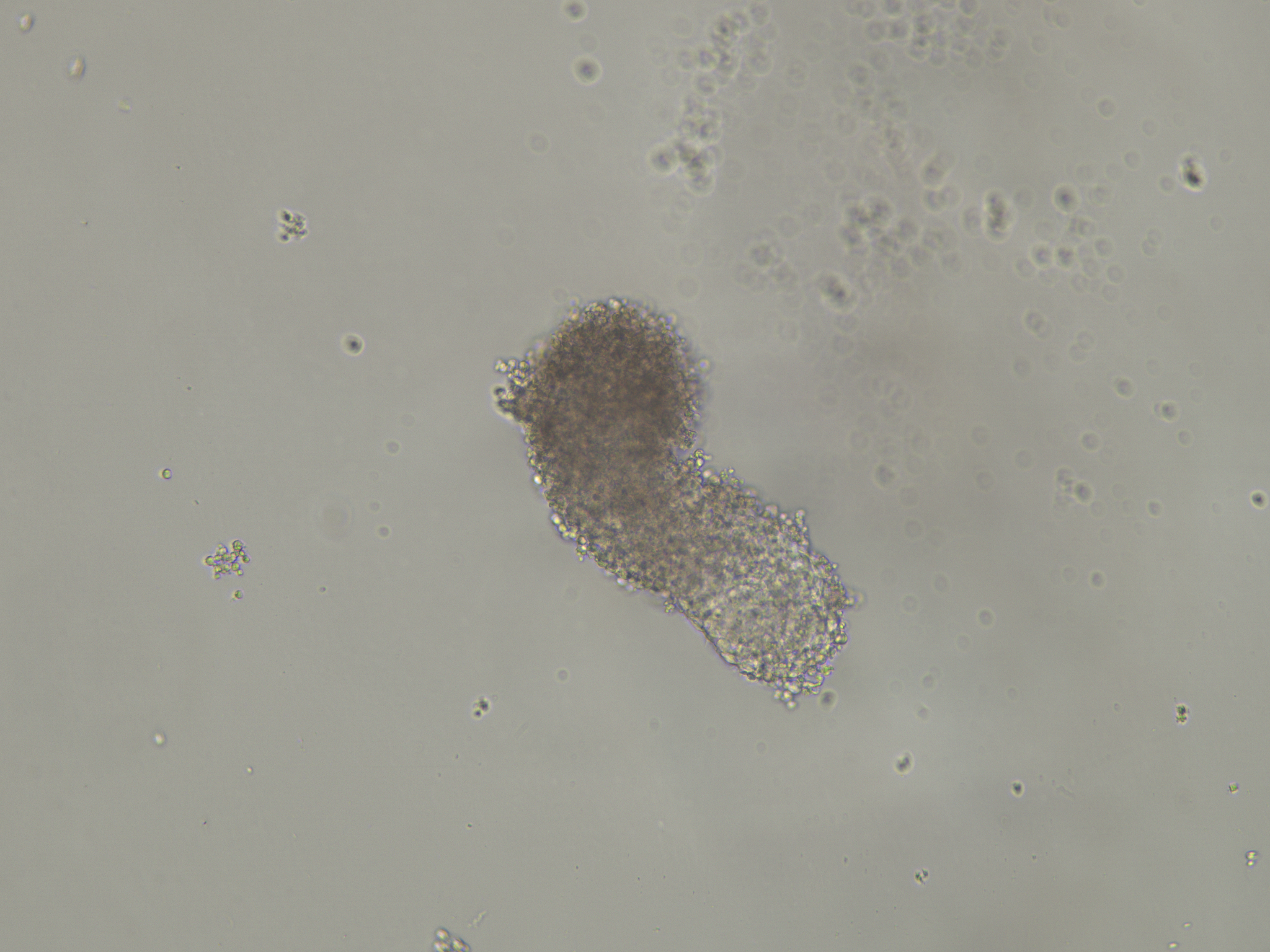

Supplement: Supplementary file 11 — Source data Fig. 9 [file 44318_2025_558_MOESM11_ESM.zip › Figure 9/panel 9B/NT_1.tiff]

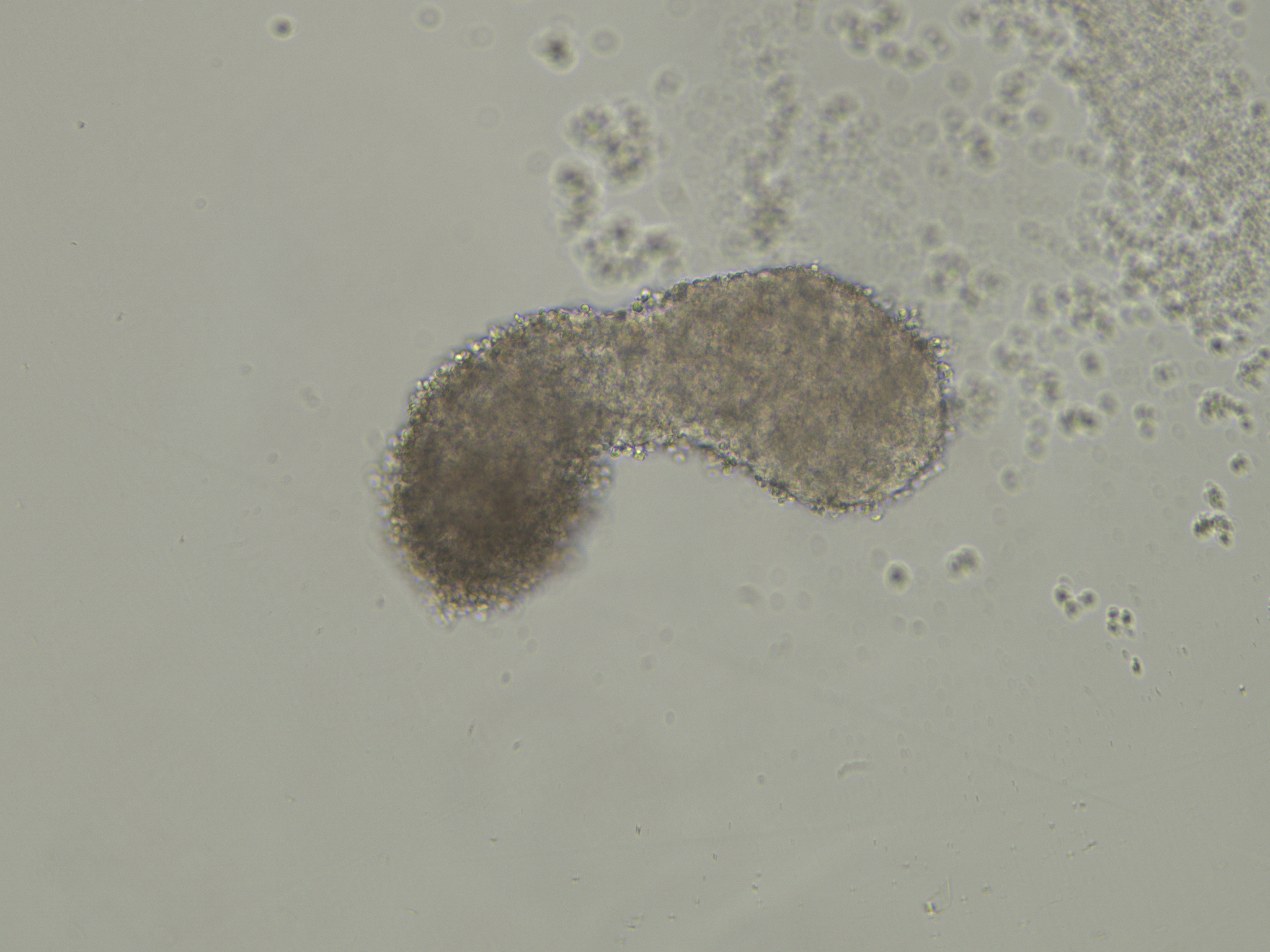

Supplement: Supplementary file 11 — Source data Fig. 9 [file 44318_2025_558_MOESM11_ESM.zip › Figure 9/panel 9B/KD-2 UC_3.tiff]

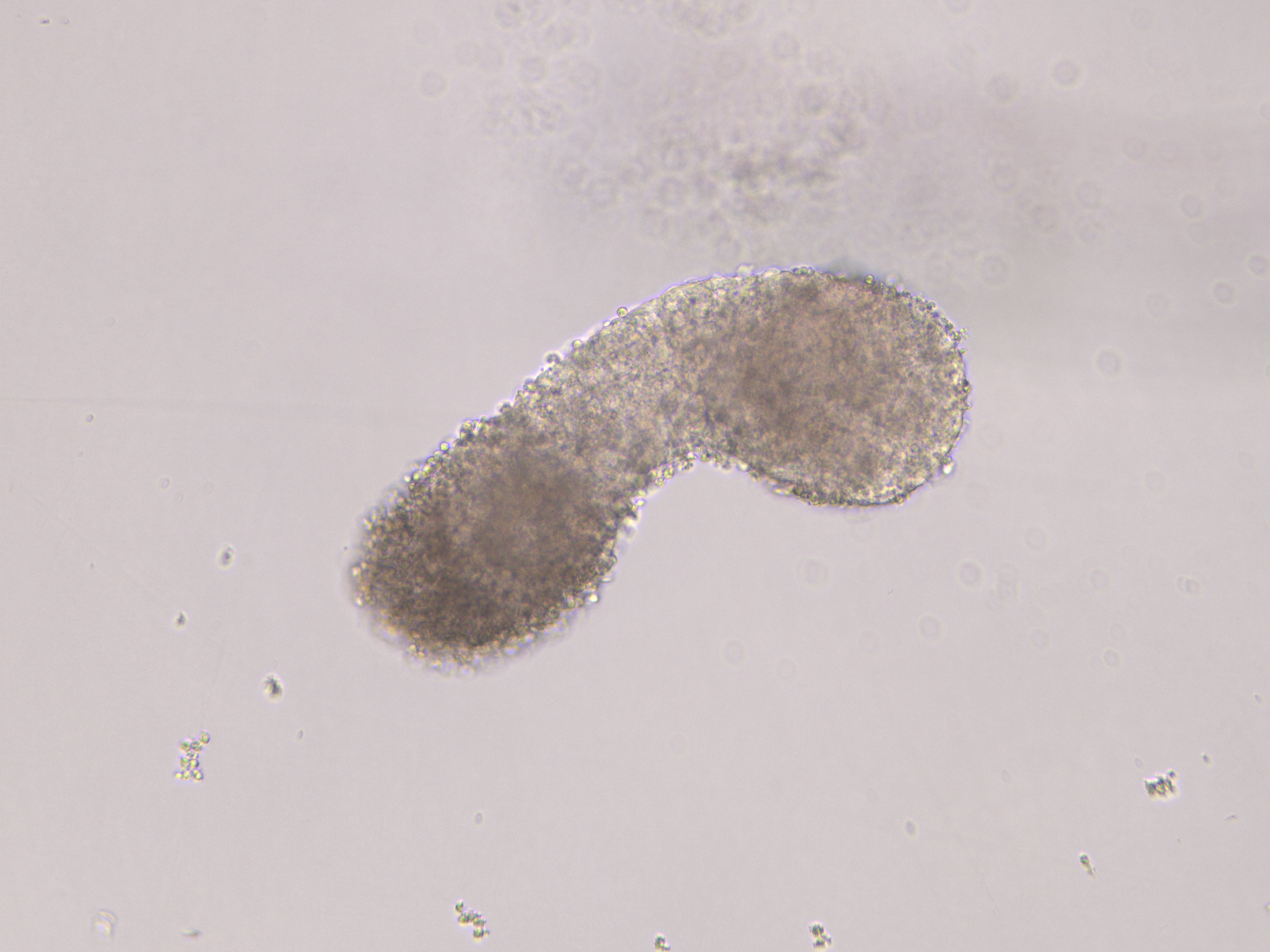

Supplement: Supplementary file 11 — Source data Fig. 9 [file 44318_2025_558_MOESM11_ESM.zip › Figure 9/panel 9B/KD-2 UC_2.tiff]

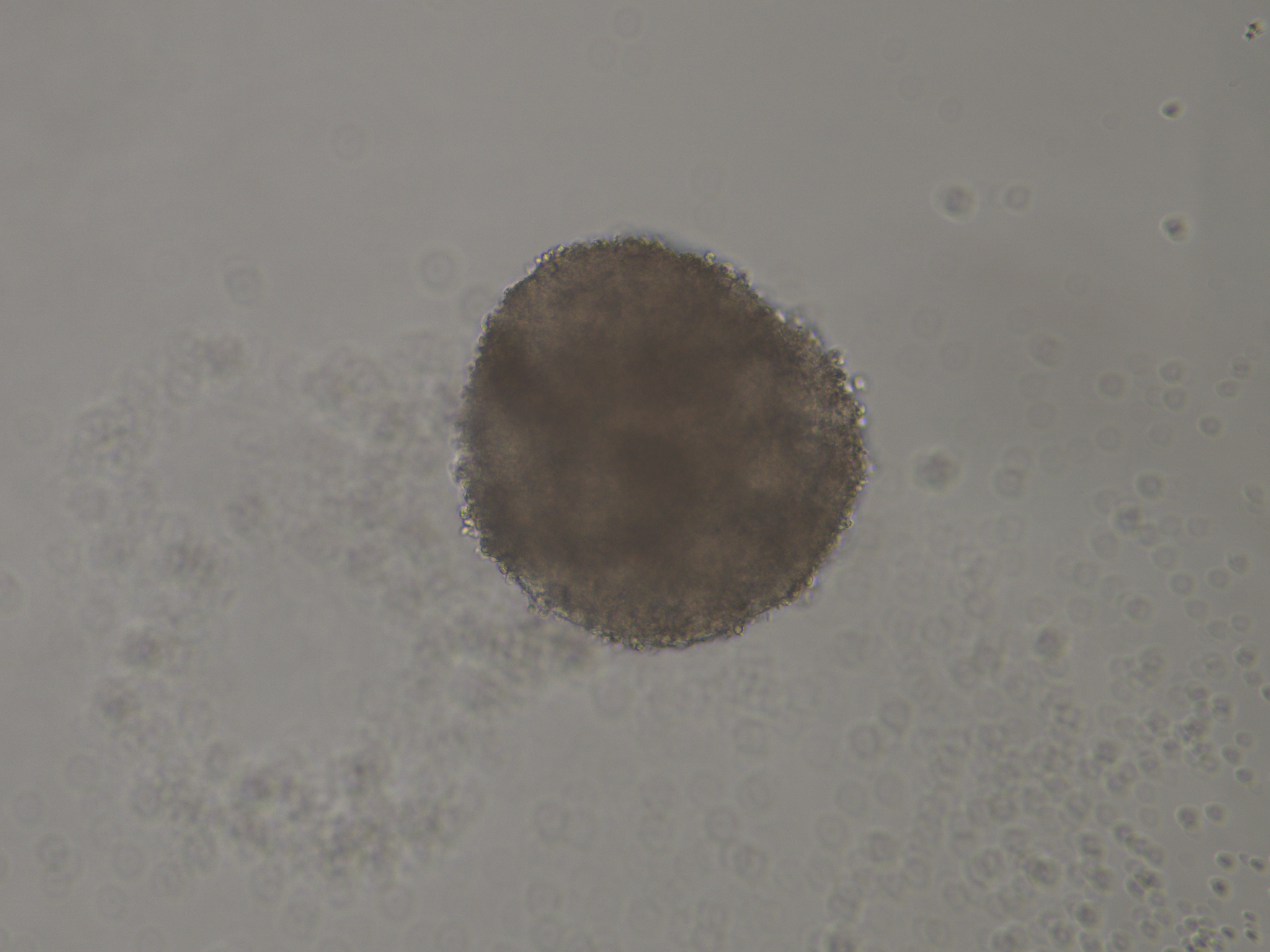

Supplement: Supplementary file 11 — Source data Fig. 9 [file 44318_2025_558_MOESM11_ESM.zip › Figure 9/panel 9B/KD-1_2.tiff]

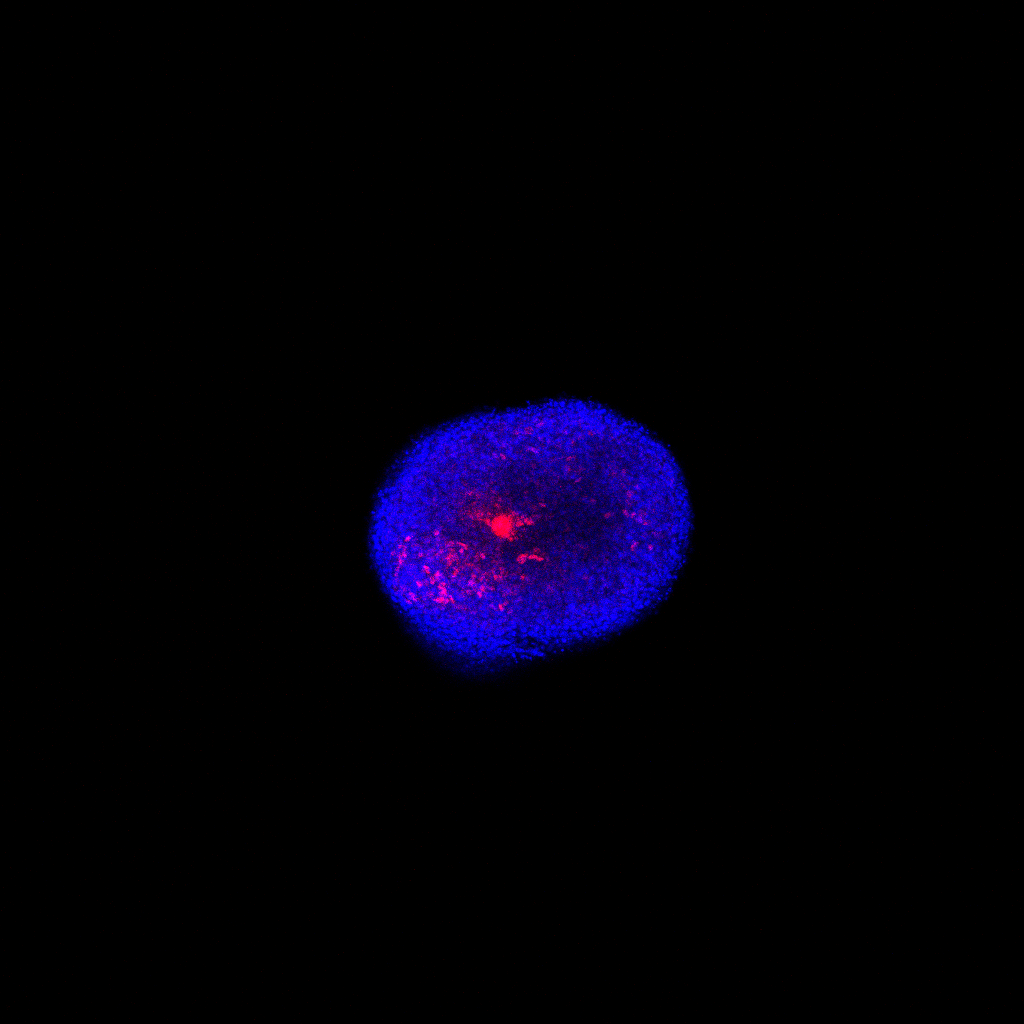

Supplement: Supplementary file 11 — Source data Fig. 9 [file 44318_2025_558_MOESM11_ESM.zip › Figure 9/panel 9D/KD-1_Bra/New-01-Image Export-03_c1-2.tif]

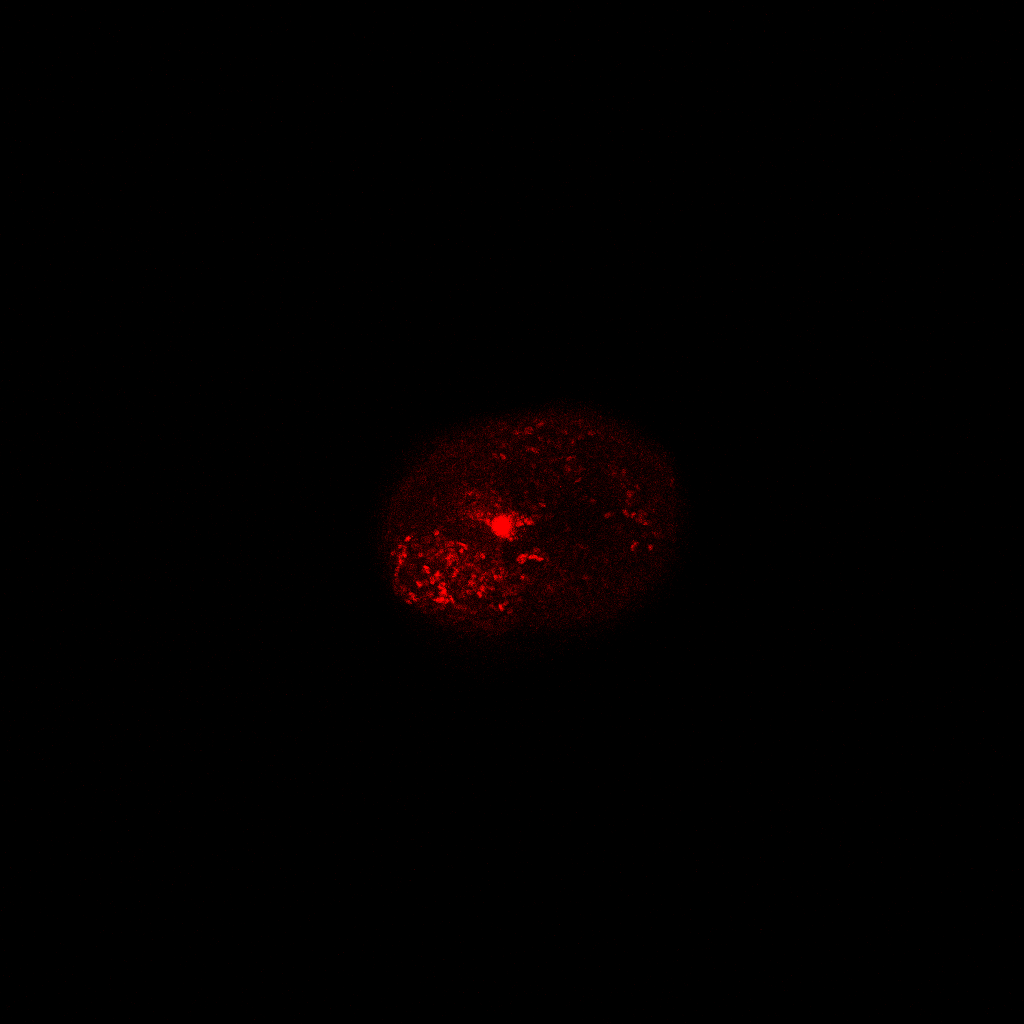

Supplement: Supplementary file 11 — Source data Fig. 9 [file 44318_2025_558_MOESM11_ESM.zip › Figure 9/panel 9D/KD-1_Bra/New-01-Image Export-03_c2.tif]

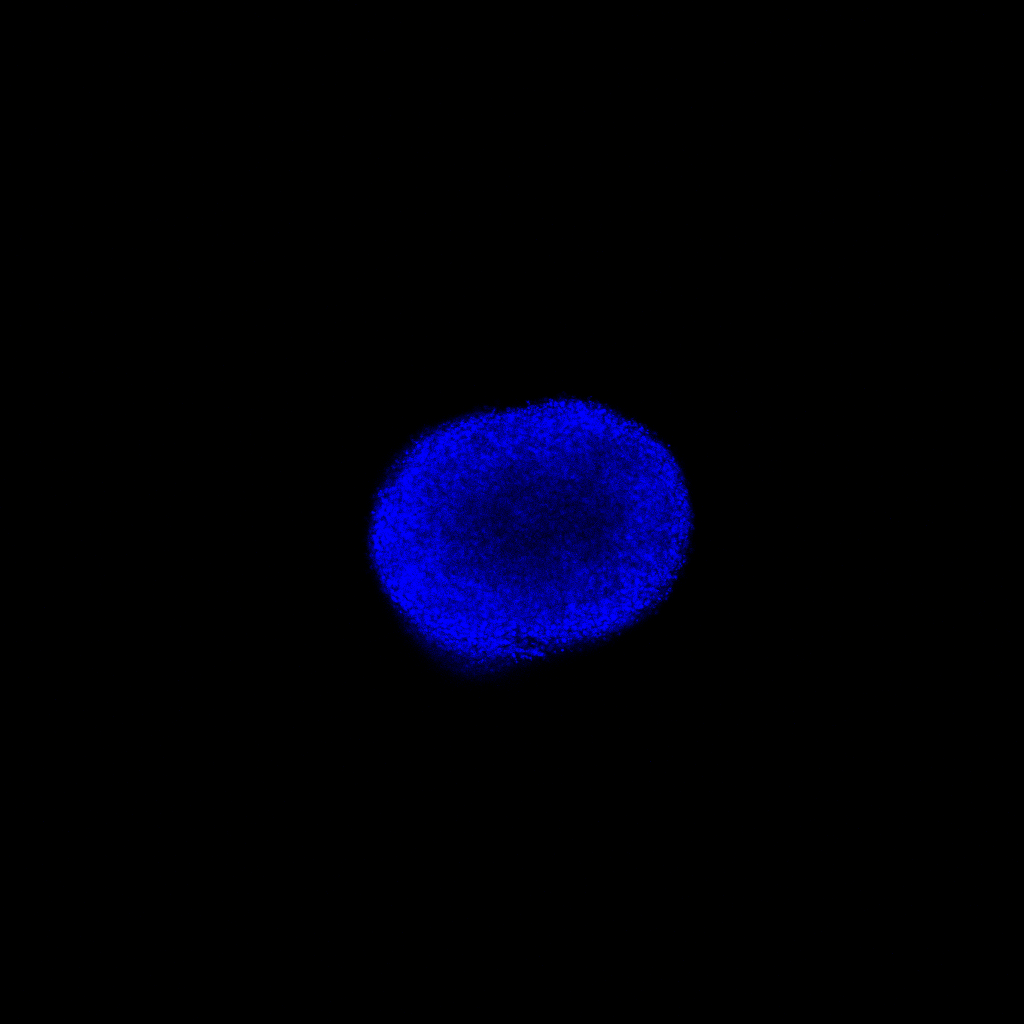

Supplement: Supplementary file 11 — Source data Fig. 9 [file 44318_2025_558_MOESM11_ESM.zip › Figure 9/panel 9D/KD-1_Bra/New-01-Image Export-03_c1.tif]

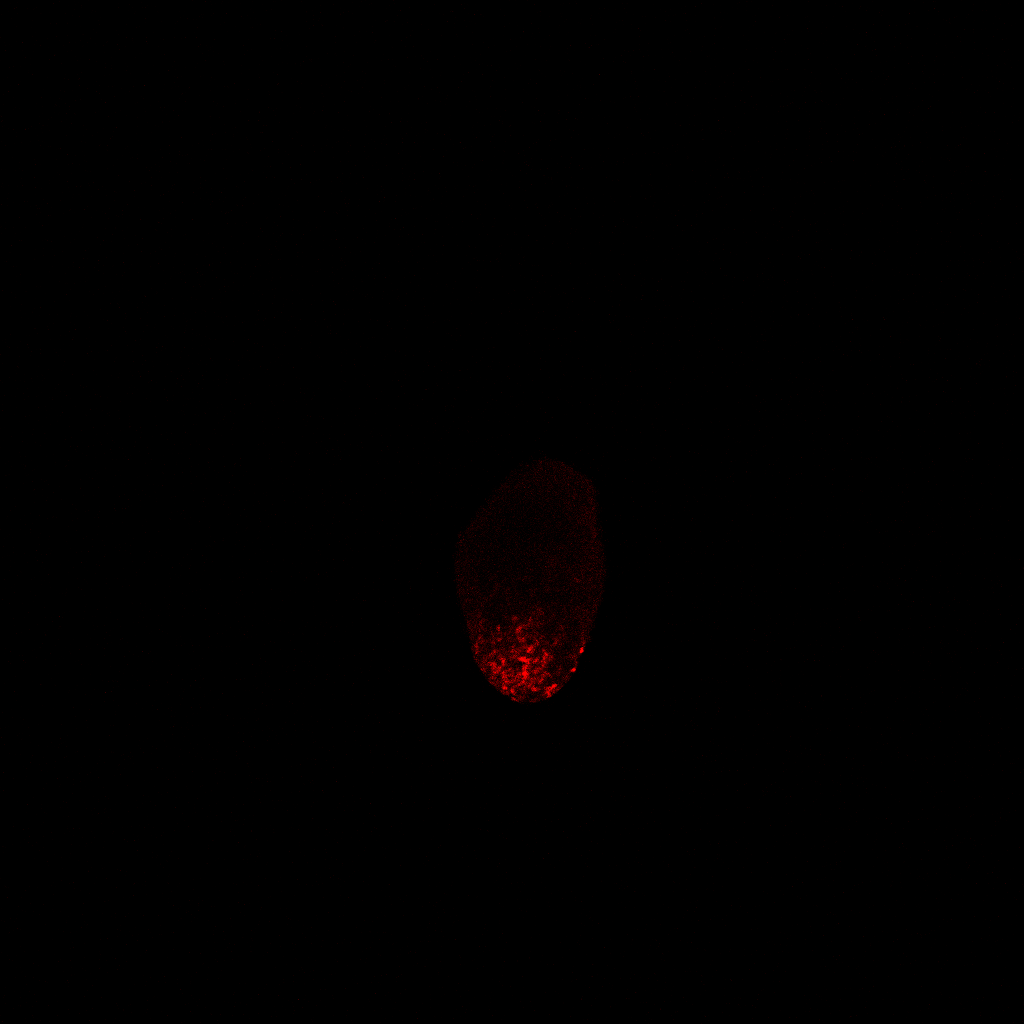

Supplement: Supplementary file 11 — Source data Fig. 9 [file 44318_2025_558_MOESM11_ESM.zip › Figure 9/panel 9D/KD-2_Bra/New-02-Image Export-09_c2.tif]

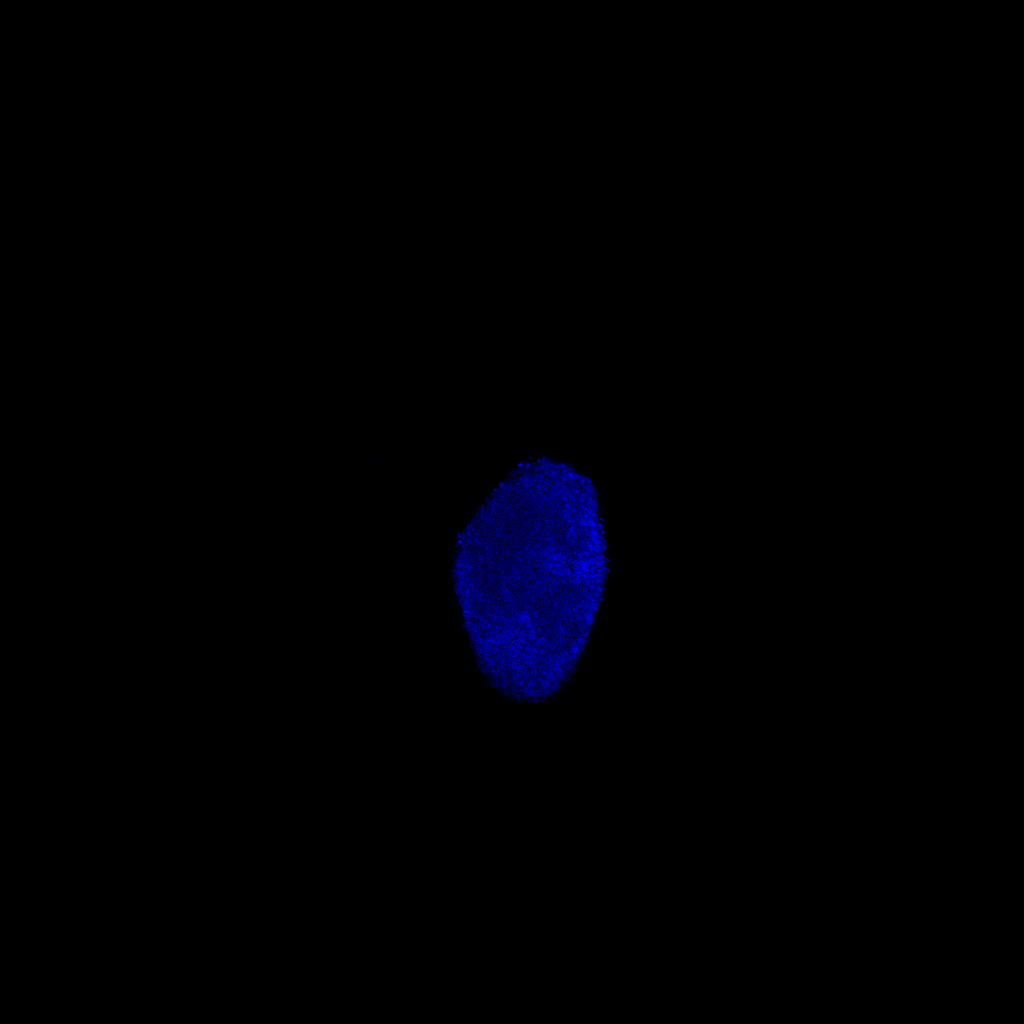

Supplement: Supplementary file 11 — Source data Fig. 9 [file 44318_2025_558_MOESM11_ESM.zip › Figure 9/panel 9D/KD-2_Bra/New-02-Image Export-09_c1.tif]

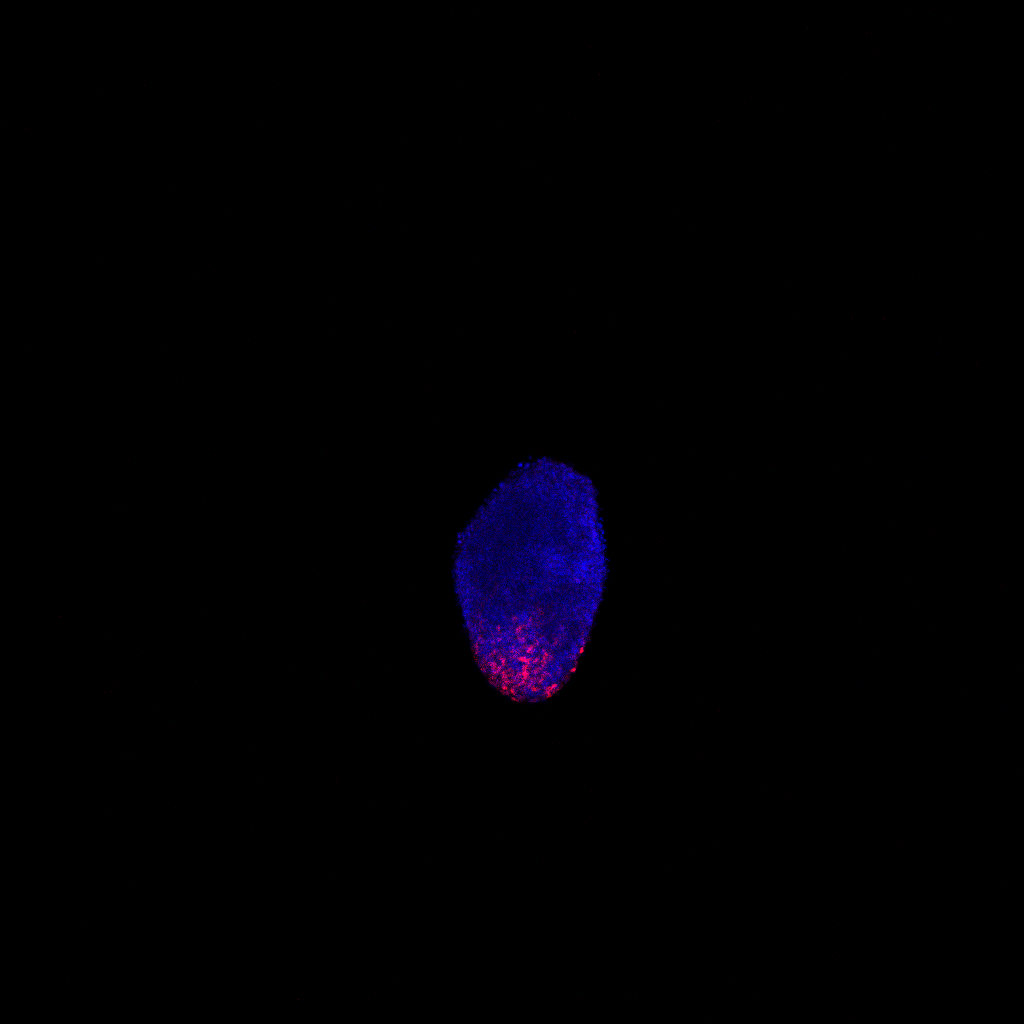

Supplement: Supplementary file 11 — Source data Fig. 9 [file 44318_2025_558_MOESM11_ESM.zip › Figure 9/panel 9D/KD-2_Bra/New-02-Image Export-09_c1-2.tif]

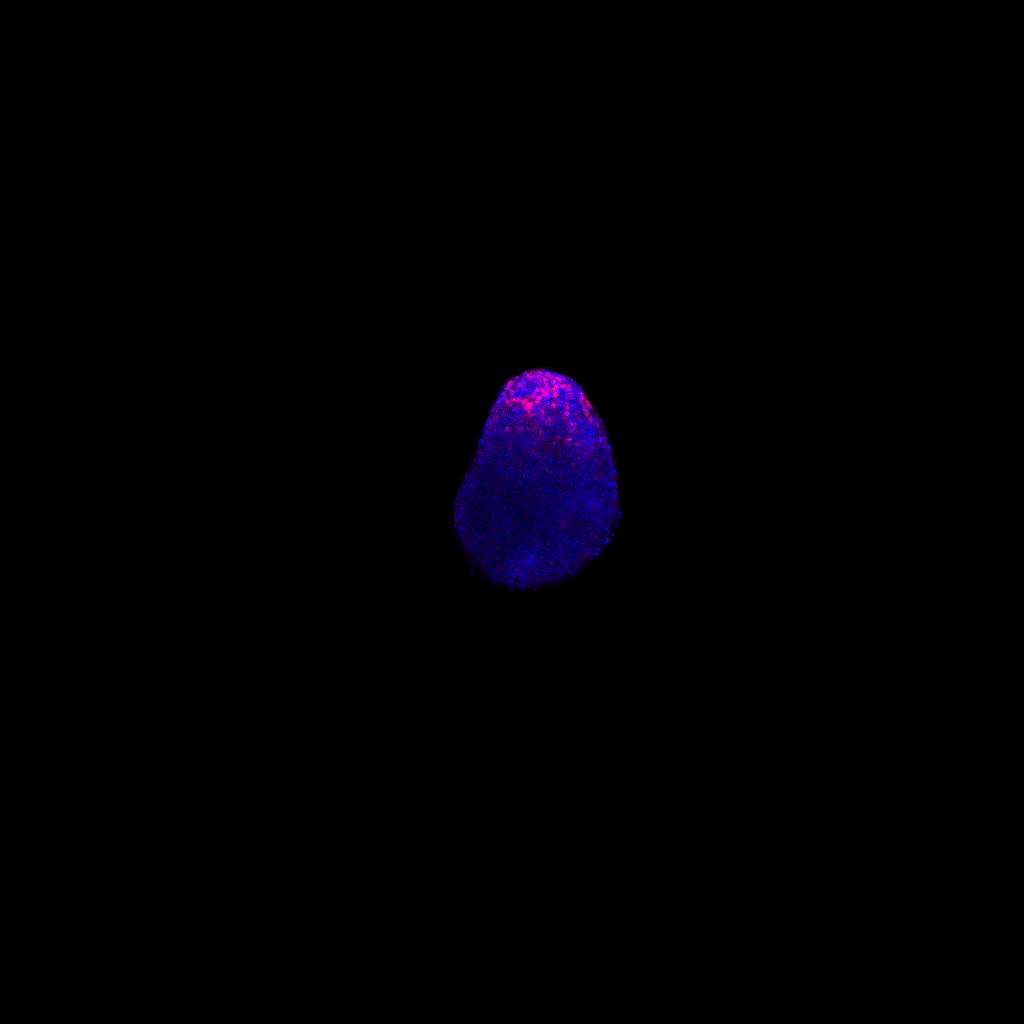

Supplement: Supplementary file 11 — Source data Fig. 9 [file 44318_2025_558_MOESM11_ESM.zip › Figure 9/panel 9D/KD-2 GFP_Bra/New-01-Image Export-11_c1-2.tif]

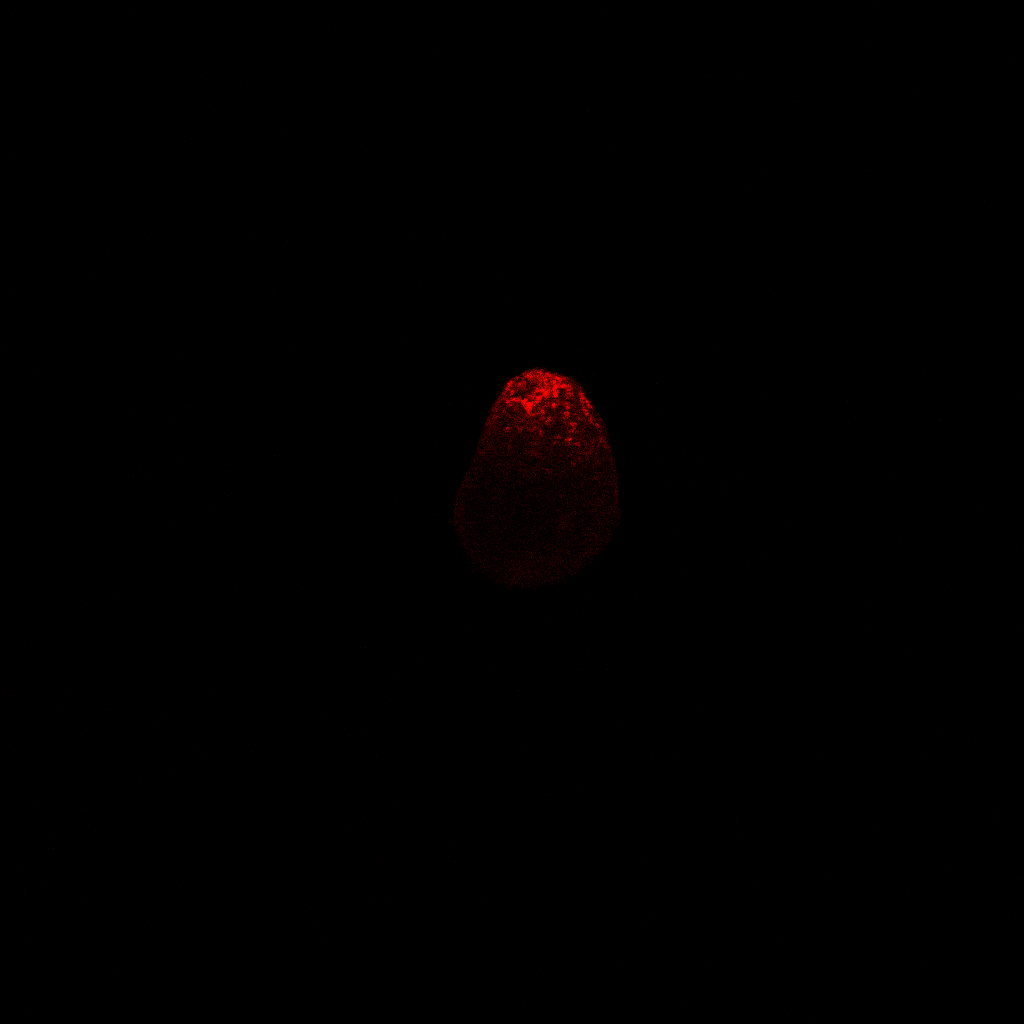

Supplement: Supplementary file 11 — Source data Fig. 9 [file 44318_2025_558_MOESM11_ESM.zip › Figure 9/panel 9D/KD-2 GFP_Bra/New-01-Image Export-11_c2.tif]

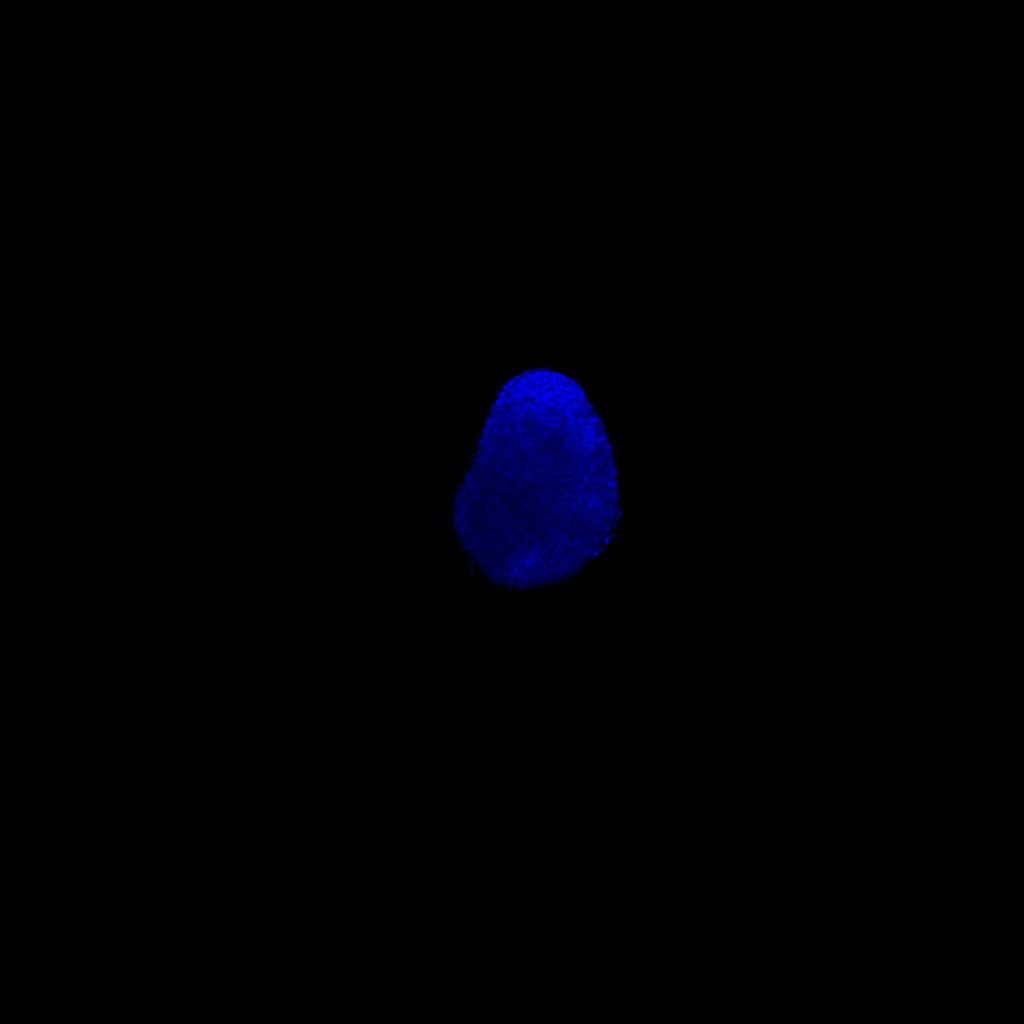

Supplement: Supplementary file 11 — Source data Fig. 9 [file 44318_2025_558_MOESM11_ESM.zip › Figure 9/panel 9D/KD-2 GFP_Bra/New-01-Image Export-11_c1.tif]

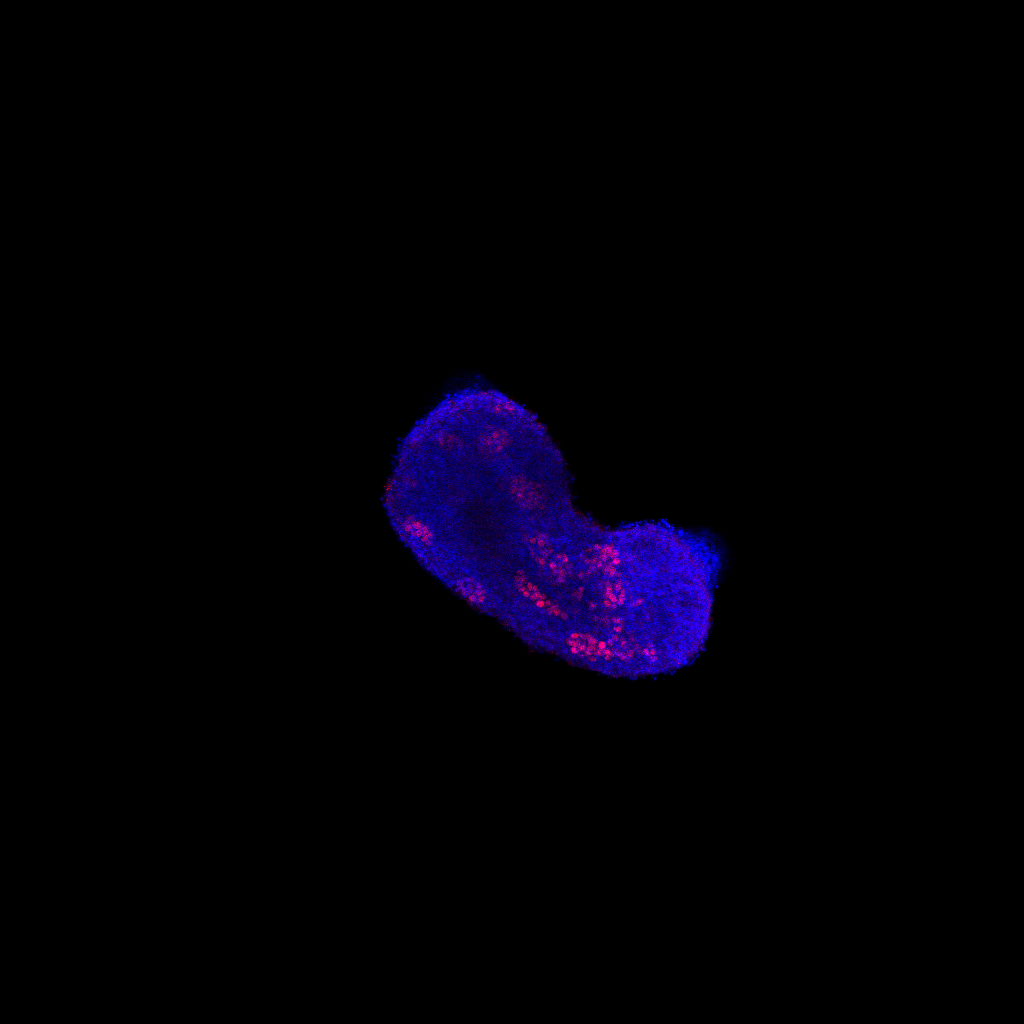

Supplement: Supplementary file 11 — Source data Fig. 9 [file 44318_2025_558_MOESM11_ESM.zip › Figure 9/panel 9D/NT_Oct4_1/New-03-Image Export-28_c1-2.tif]

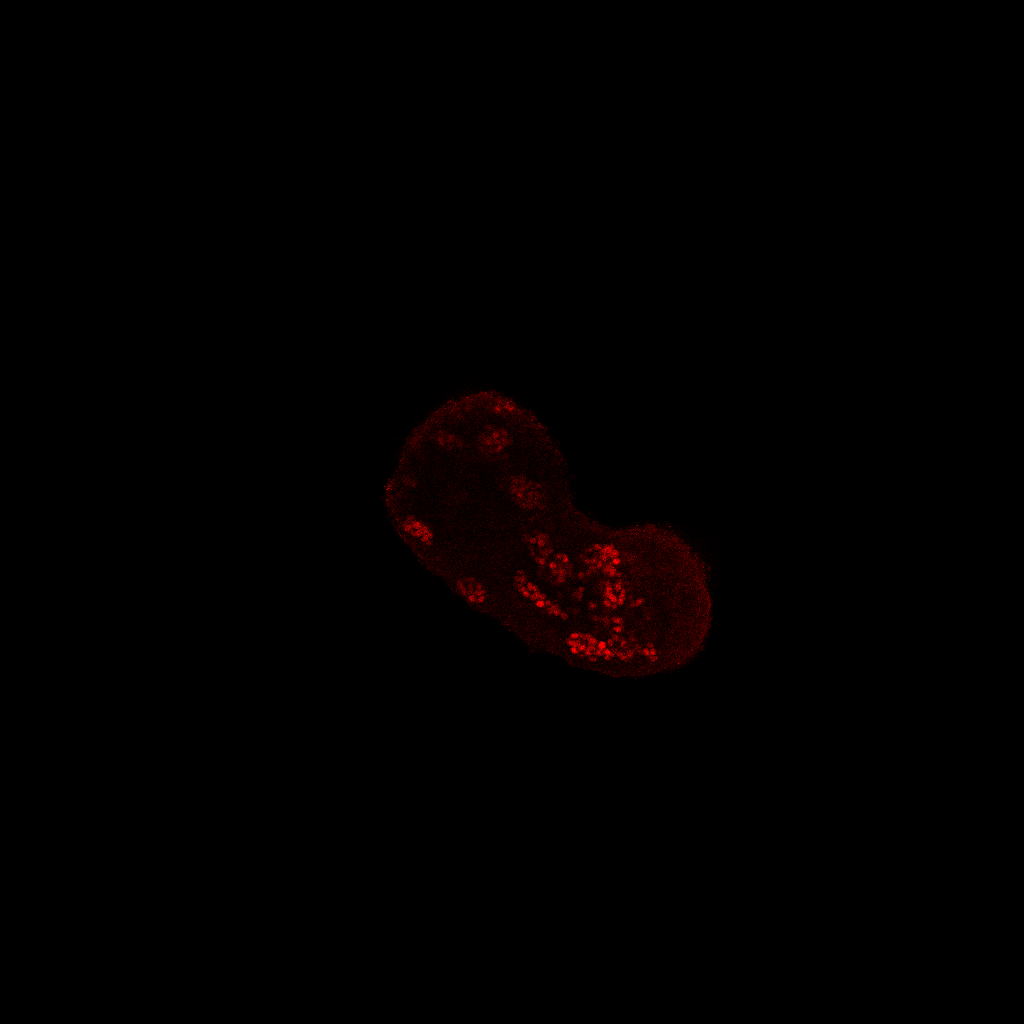

Supplement: Supplementary file 11 — Source data Fig. 9 [file 44318_2025_558_MOESM11_ESM.zip › Figure 9/panel 9D/NT_Oct4_1/New-03-Image Export-28_c2.tif]

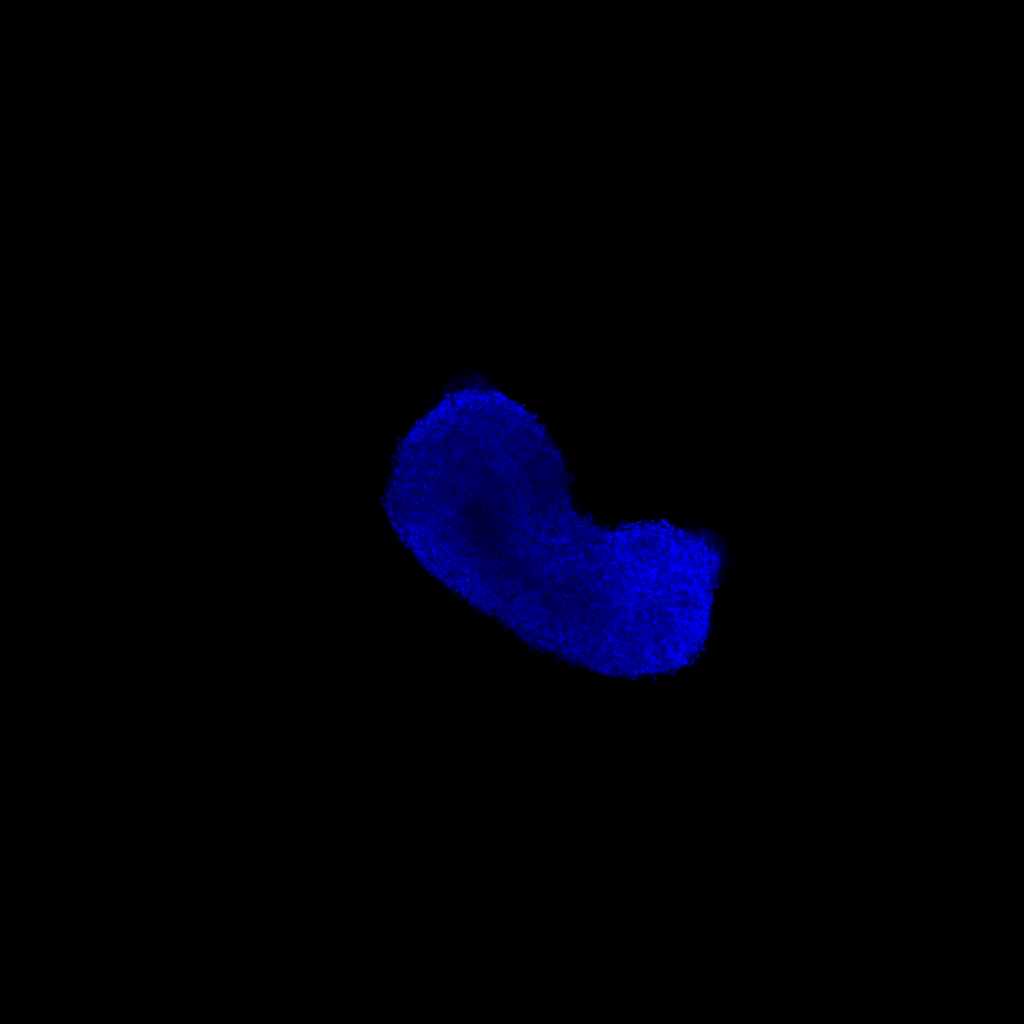

Supplement: Supplementary file 11 — Source data Fig. 9 [file 44318_2025_558_MOESM11_ESM.zip › Figure 9/panel 9D/NT_Oct4_1/New-03-Image Export-28_c1.tif]

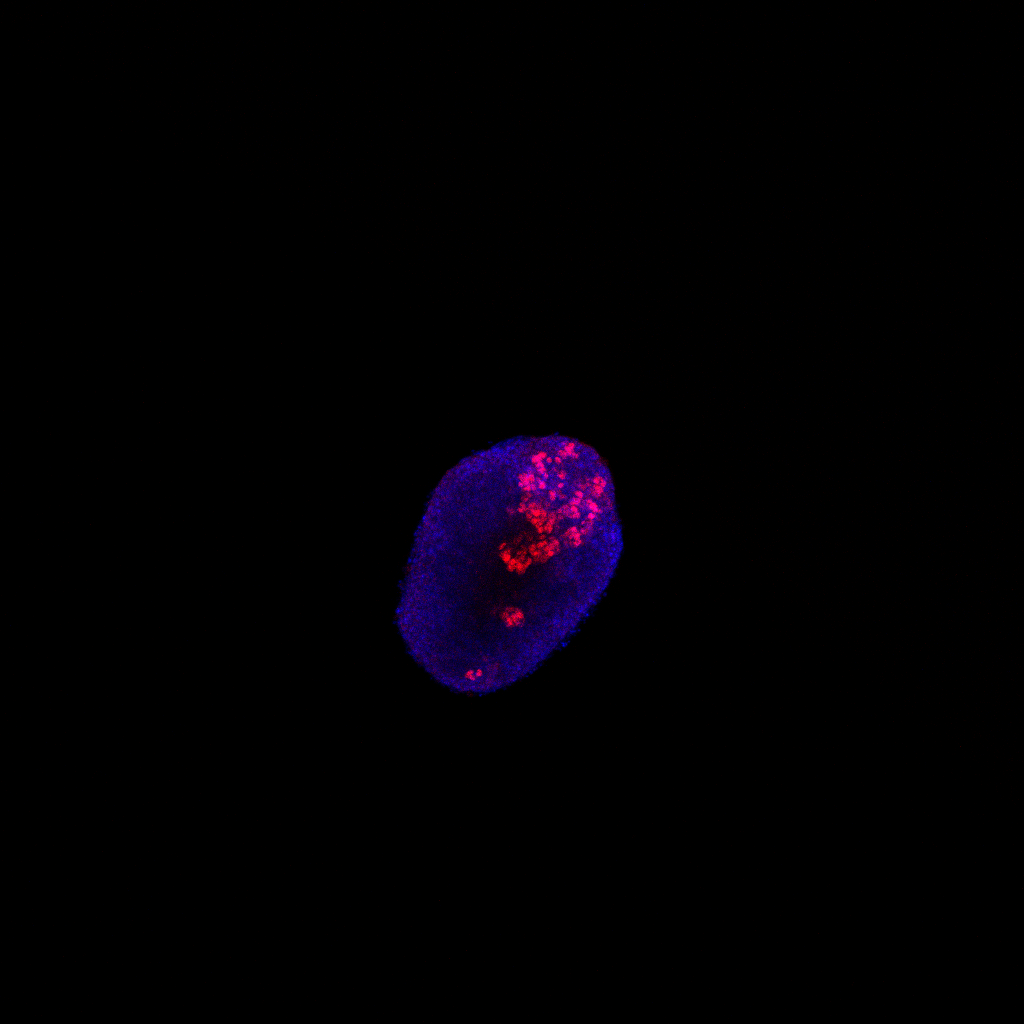

Supplement: Supplementary file 11 — Source data Fig. 9 [file 44318_2025_558_MOESM11_ESM.zip › Figure 9/panel 9D/KD-2_Oct4/New-01-Image Export-18_c1-2.tif]

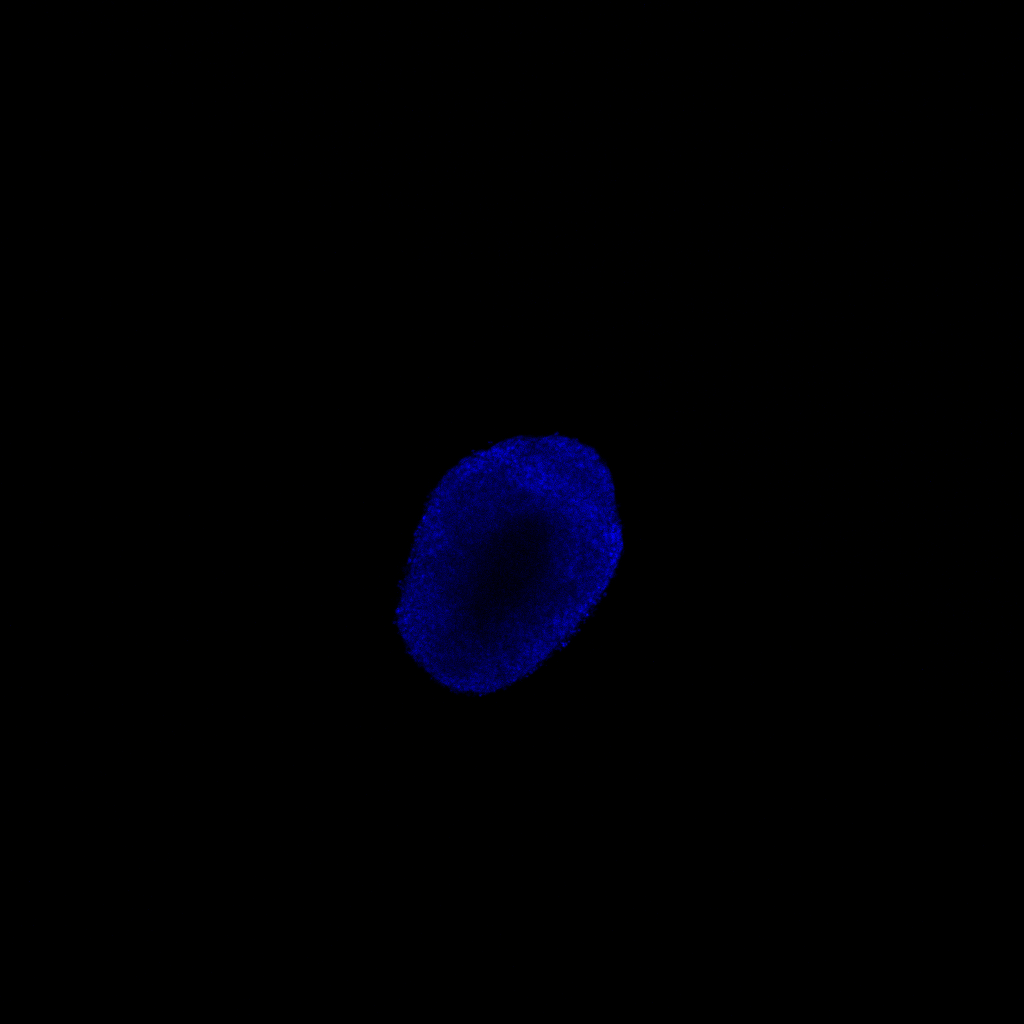

Supplement: Supplementary file 11 — Source data Fig. 9 [file 44318_2025_558_MOESM11_ESM.zip › Figure 9/panel 9D/KD-2_Oct4/New-01-Image Export-18_c1.tif]

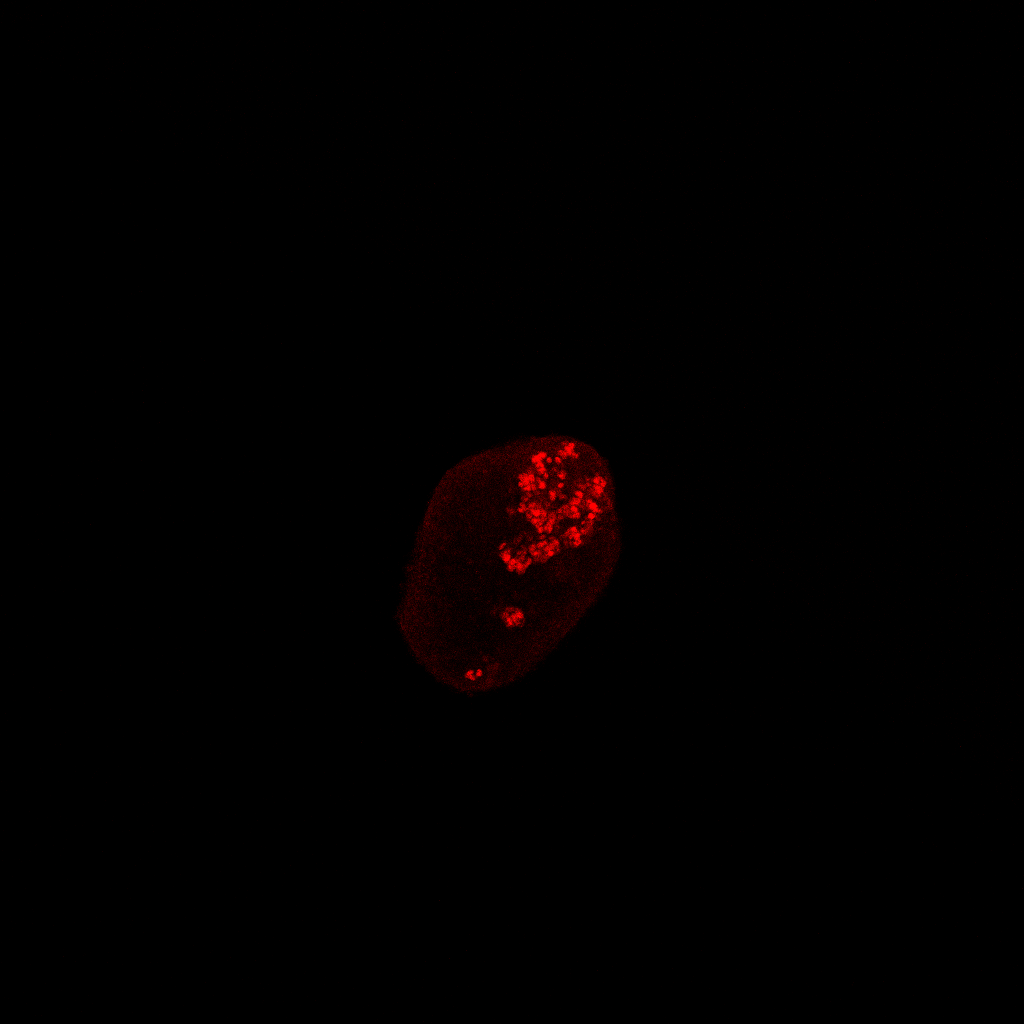

Supplement: Supplementary file 11 — Source data Fig. 9 [file 44318_2025_558_MOESM11_ESM.zip › Figure 9/panel 9D/KD-2_Oct4/New-01-Image Export-18_c2.tif]

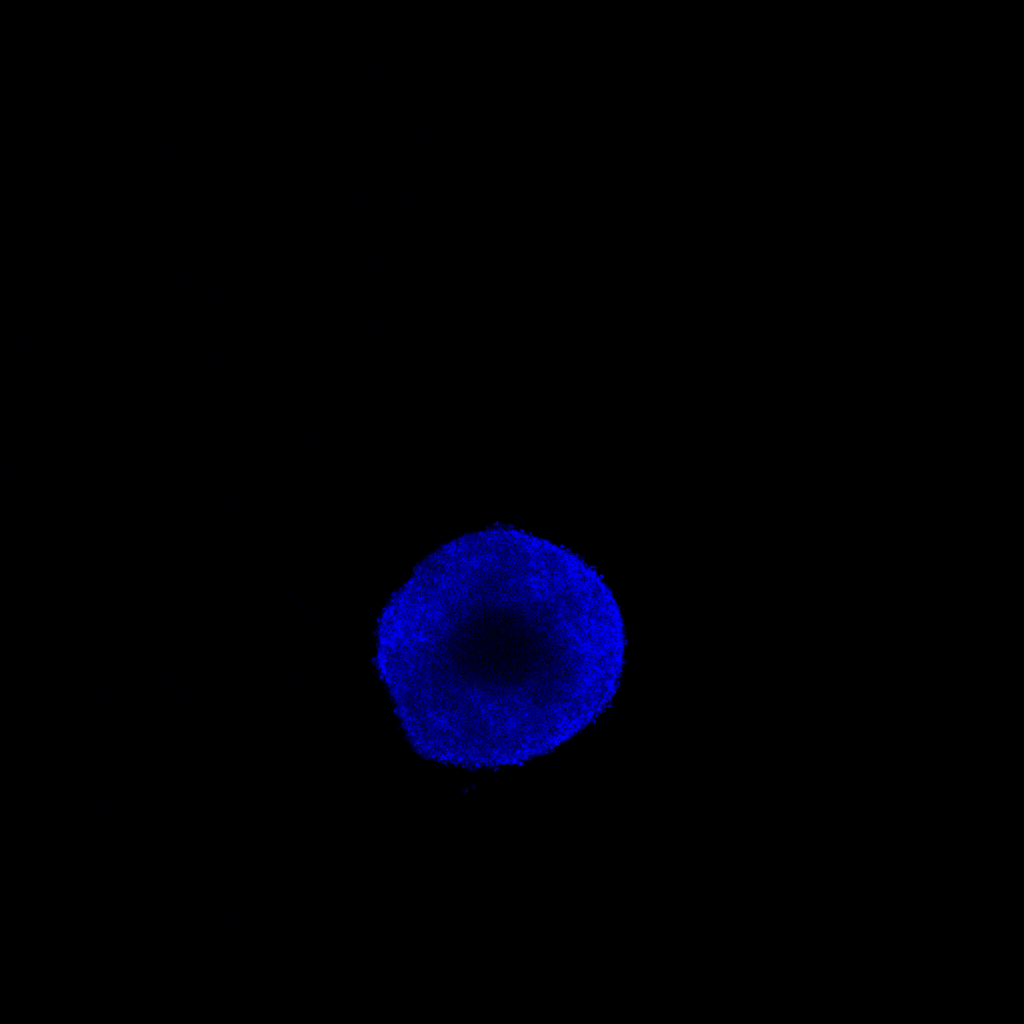

Supplement: Supplementary file 11 — Source data Fig. 9 [file 44318_2025_558_MOESM11_ESM.zip › Figure 9/panel 9D/KD-2 GFP_Oct4/New-03-Image Export-34_c1.tif]

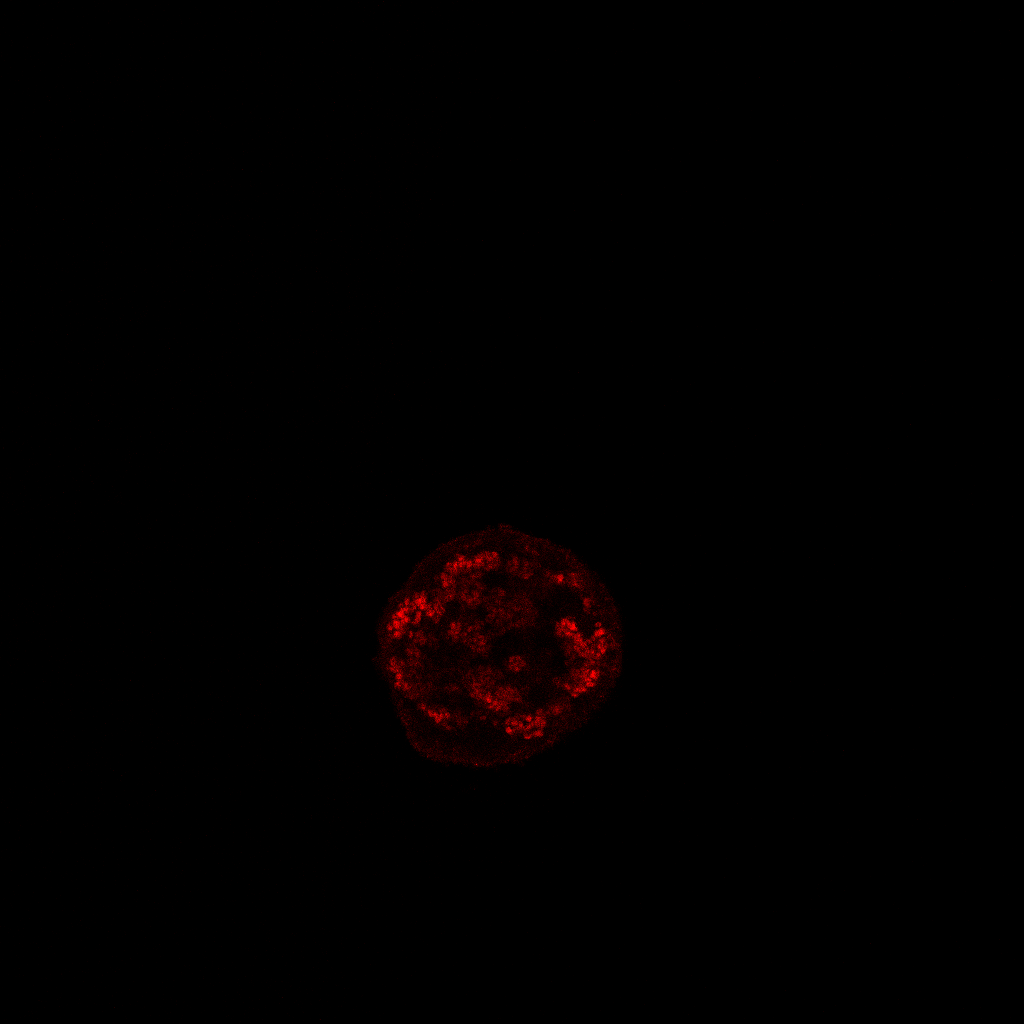

Supplement: Supplementary file 11 — Source data Fig. 9 [file 44318_2025_558_MOESM11_ESM.zip › Figure 9/panel 9D/KD-2 GFP_Oct4/New-03-Image Export-34_c2.tif]

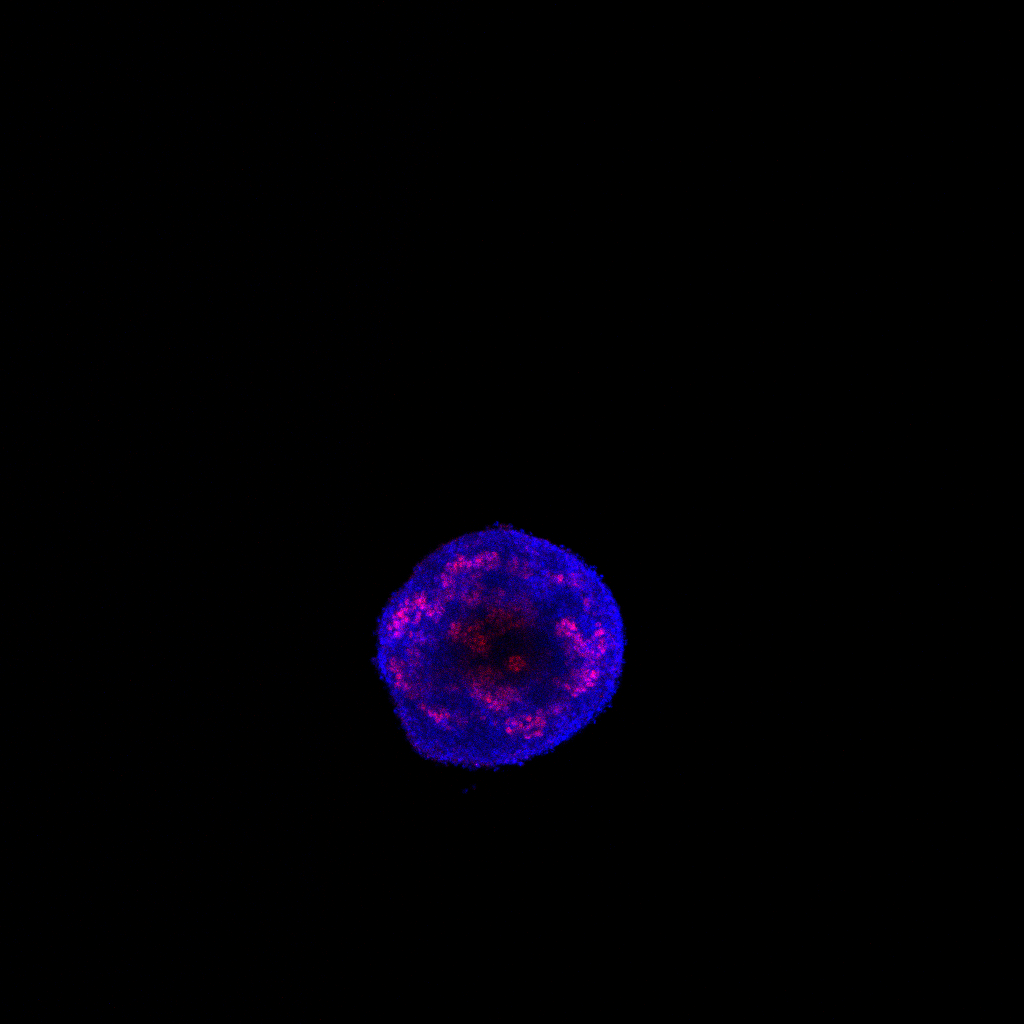

Supplement: Supplementary file 11 — Source data Fig. 9 [file 44318_2025_558_MOESM11_ESM.zip › Figure 9/panel 9D/KD-2 GFP_Oct4/New-03-Image Export-34_c1-2.tif]

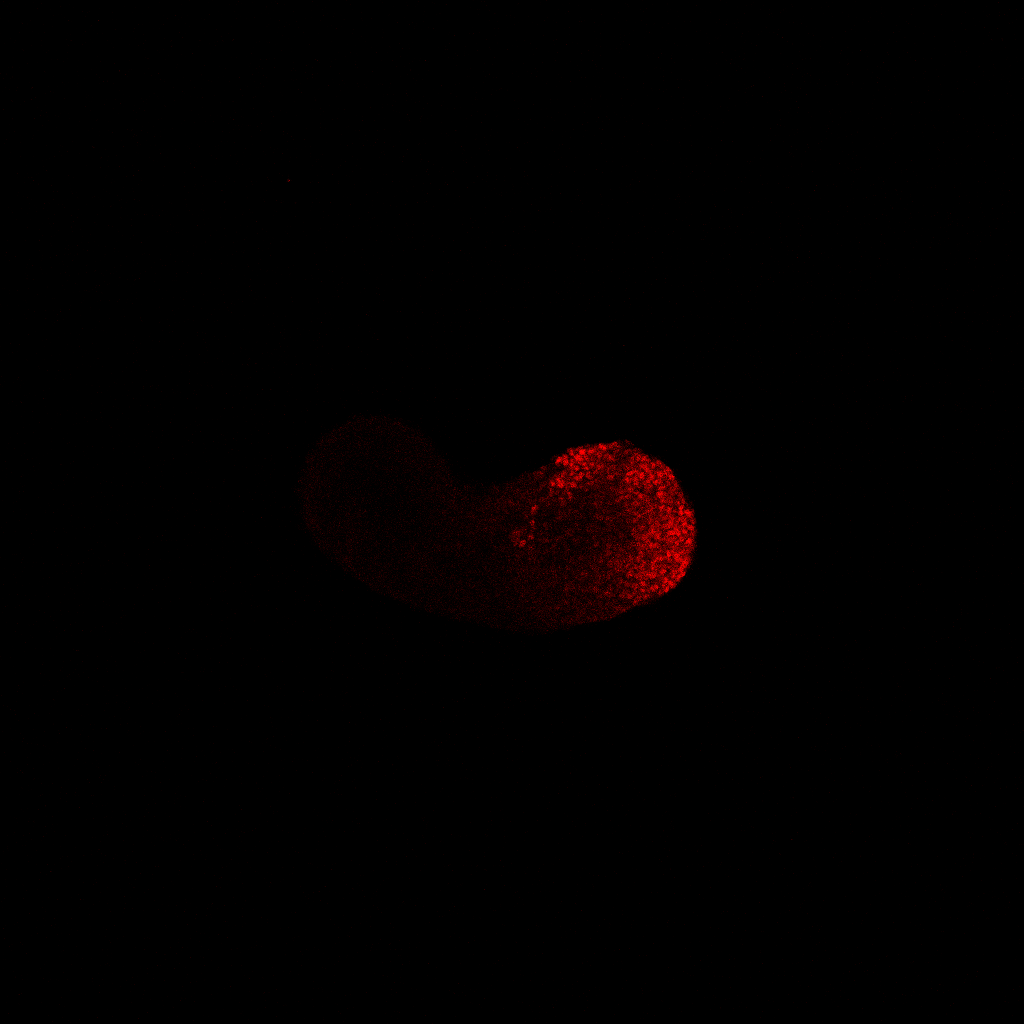

Supplement: Supplementary file 11 — Source data Fig. 9 [file 44318_2025_558_MOESM11_ESM.zip › Figure 9/panel 9D/KD-2 UC_Bra/New-02-Image Export-14_c2.tif]

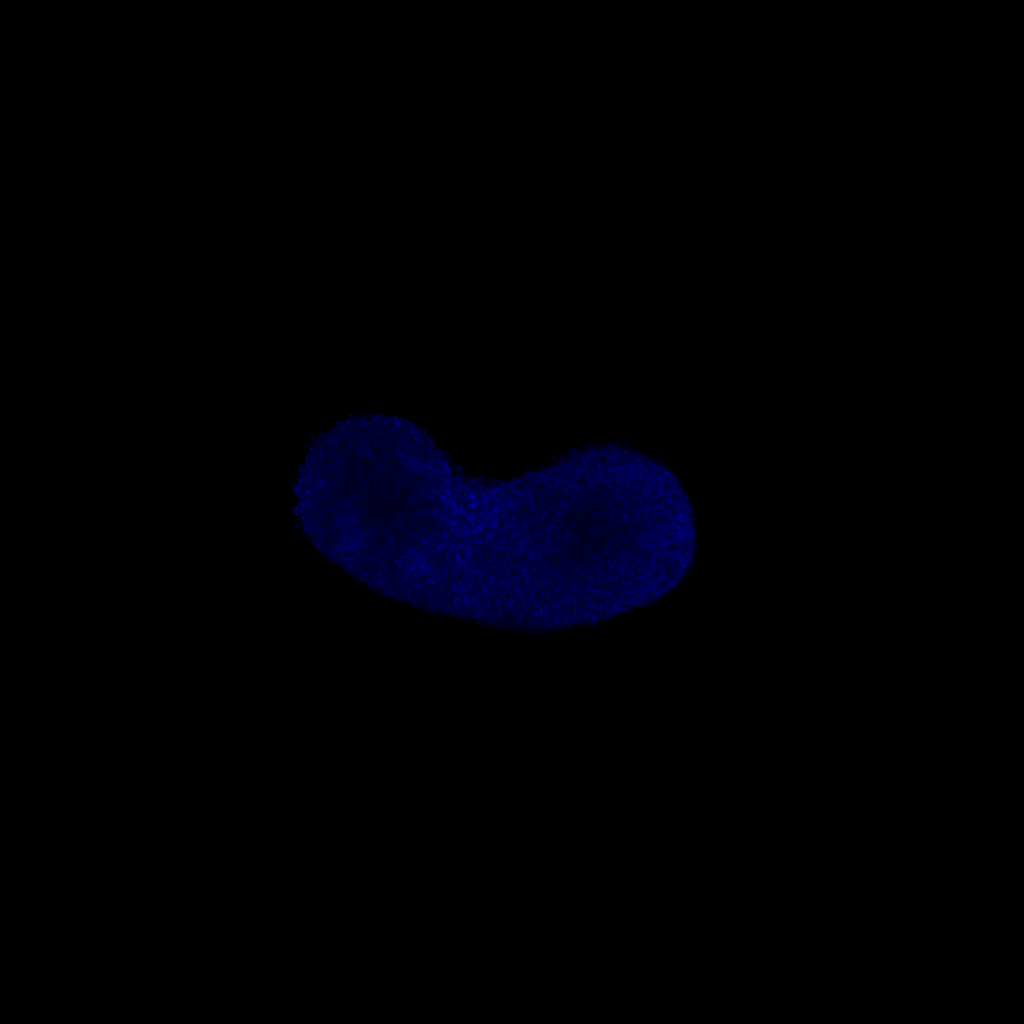

Supplement: Supplementary file 11 — Source data Fig. 9 [file 44318_2025_558_MOESM11_ESM.zip › Figure 9/panel 9D/KD-2 UC_Bra/New-02-Image Export-14_c1.tif]

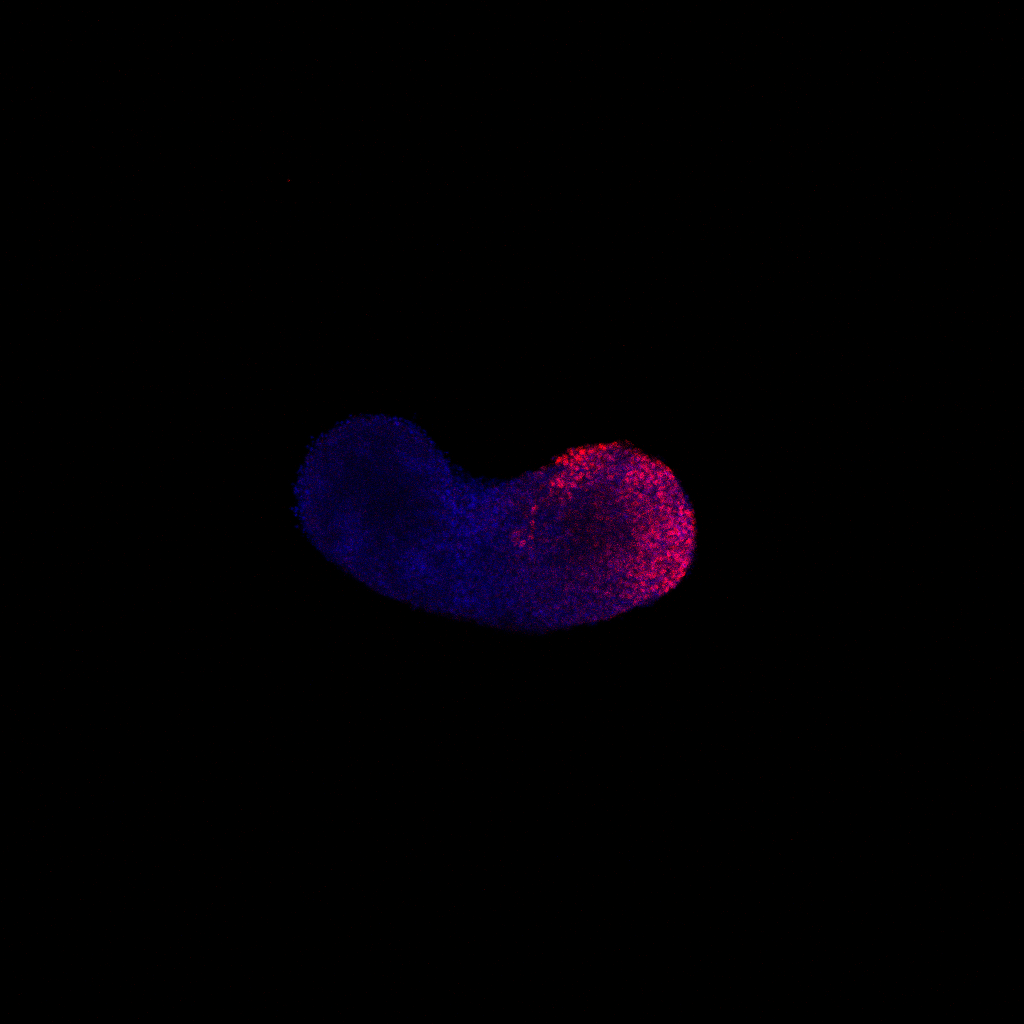

Supplement: Supplementary file 11 — Source data Fig. 9 [file 44318_2025_558_MOESM11_ESM.zip › Figure 9/panel 9D/KD-2 UC_Bra/New-02-Image Export-14_c1-2.tif]

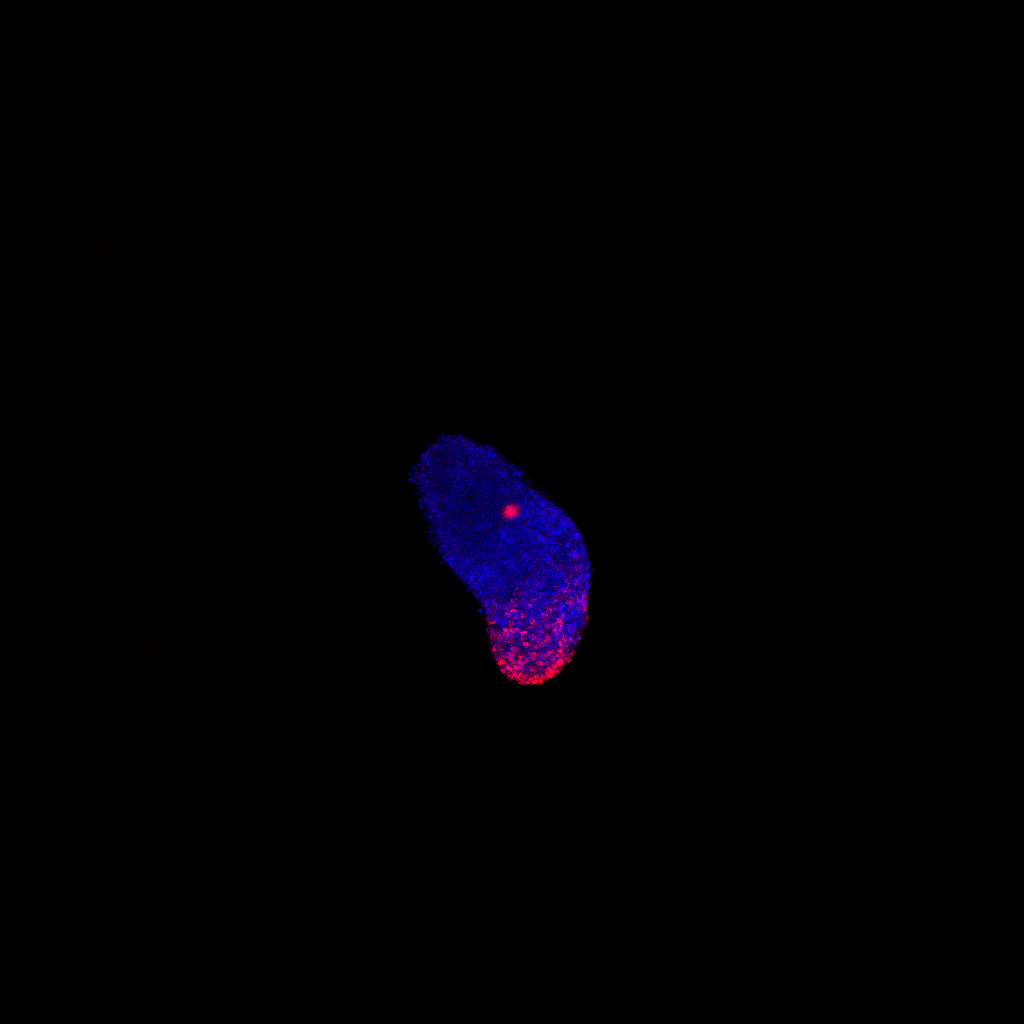

Supplement: Supplementary file 11 — Source data Fig. 9 [file 44318_2025_558_MOESM11_ESM.zip › Figure 9/panel 9D/NT_Bra_2/New-01-Image Export-05_c1-2.tif]

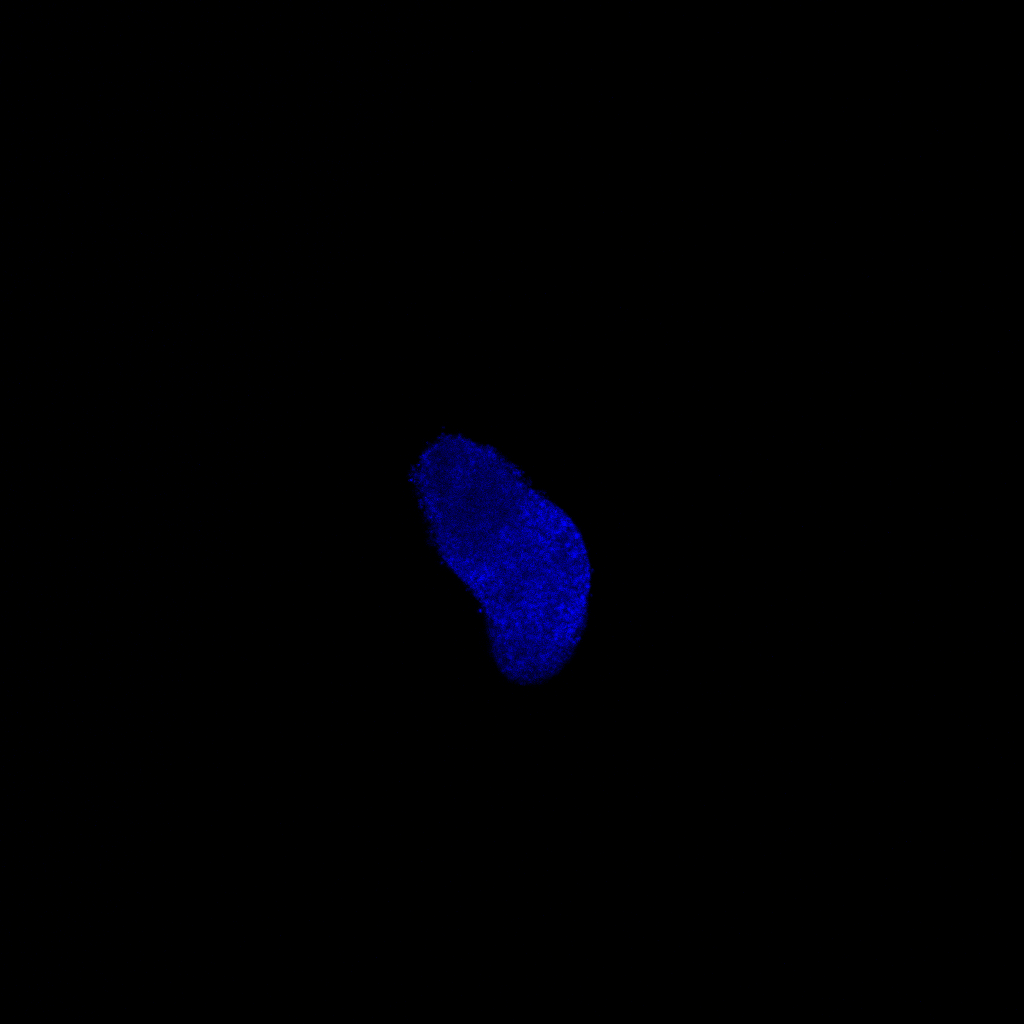

Supplement: Supplementary file 11 — Source data Fig. 9 [file 44318_2025_558_MOESM11_ESM.zip › Figure 9/panel 9D/NT_Bra_2/New-01-Image Export-05_c1.tif]

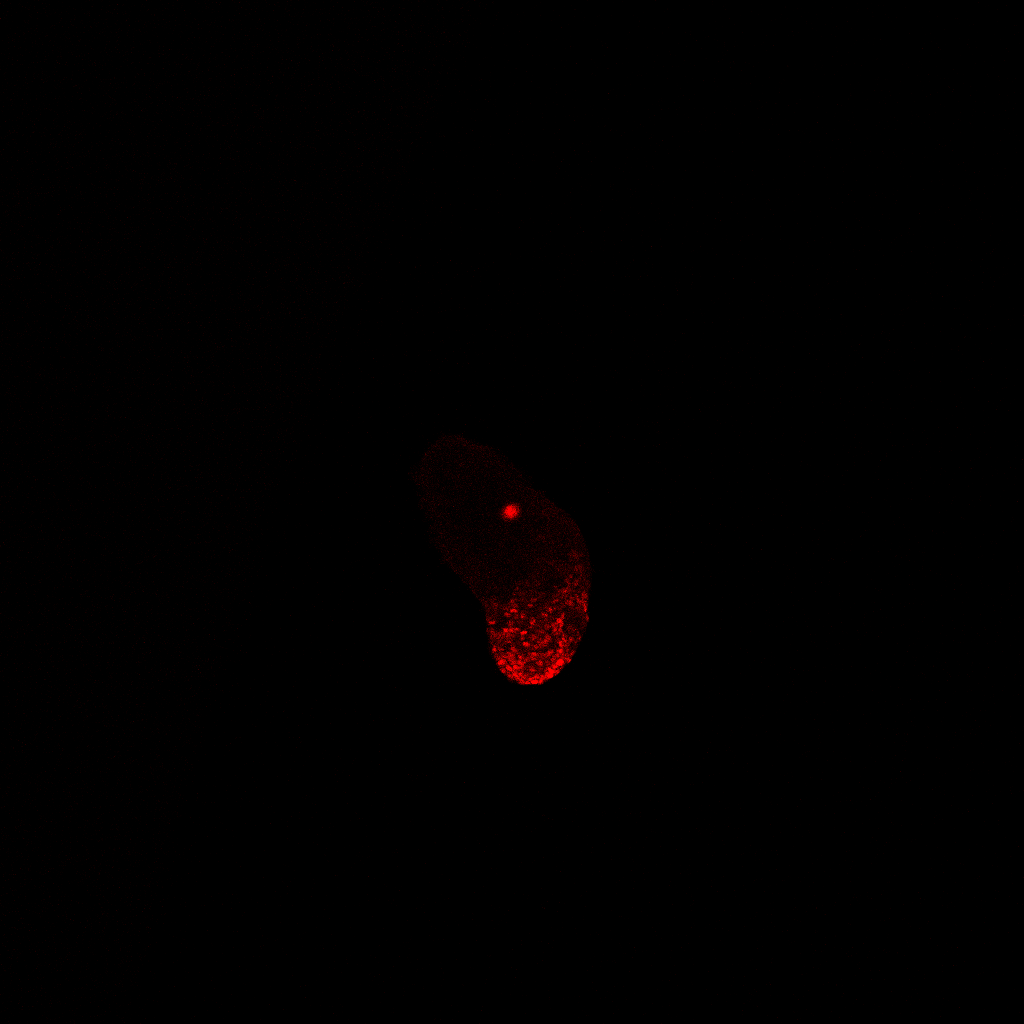

Supplement: Supplementary file 11 — Source data Fig. 9 [file 44318_2025_558_MOESM11_ESM.zip › Figure 9/panel 9D/NT_Bra_2/New-01-Image Export-05_c2.tif]

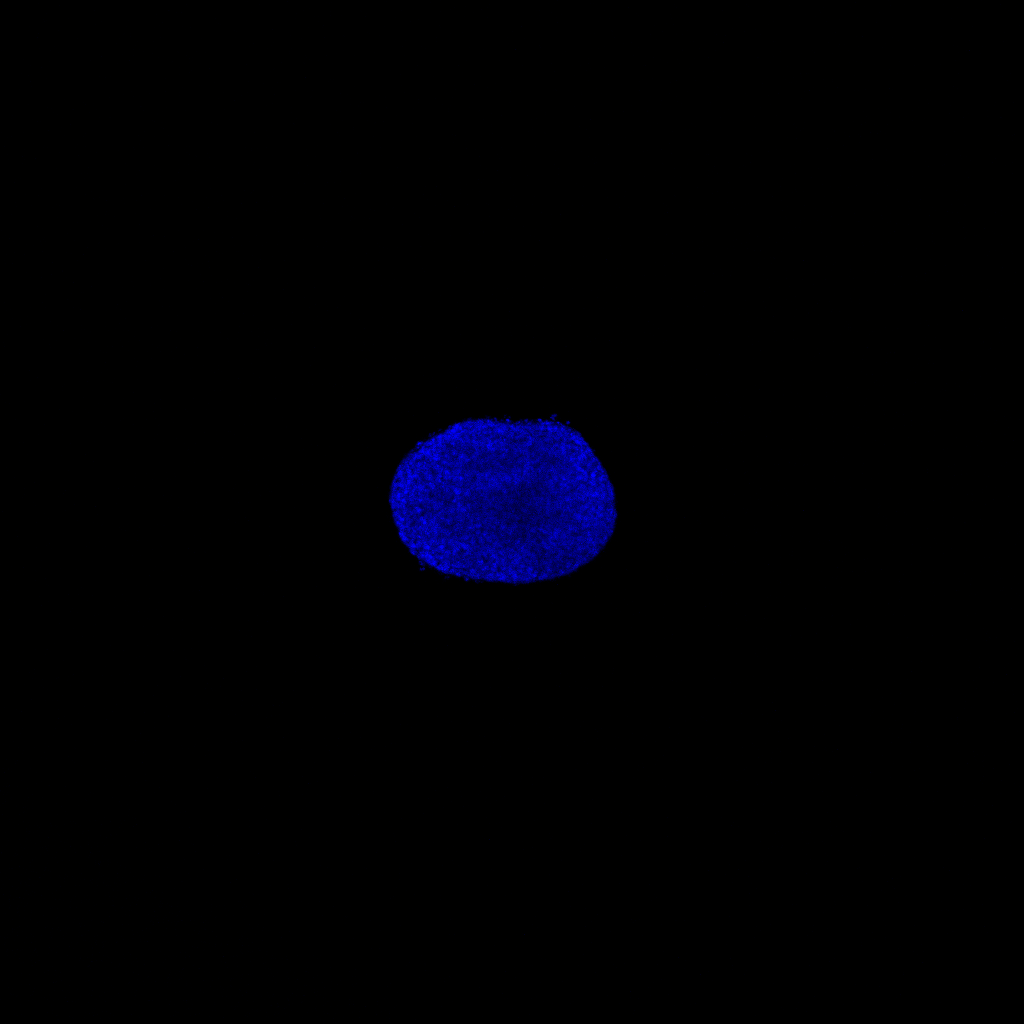

Supplement: Supplementary file 11 — Source data Fig. 9 [file 44318_2025_558_MOESM11_ESM.zip › Figure 9/panel 9D/KD-1 GFP_Bra/New-02-Image Export-04_c1.tif]

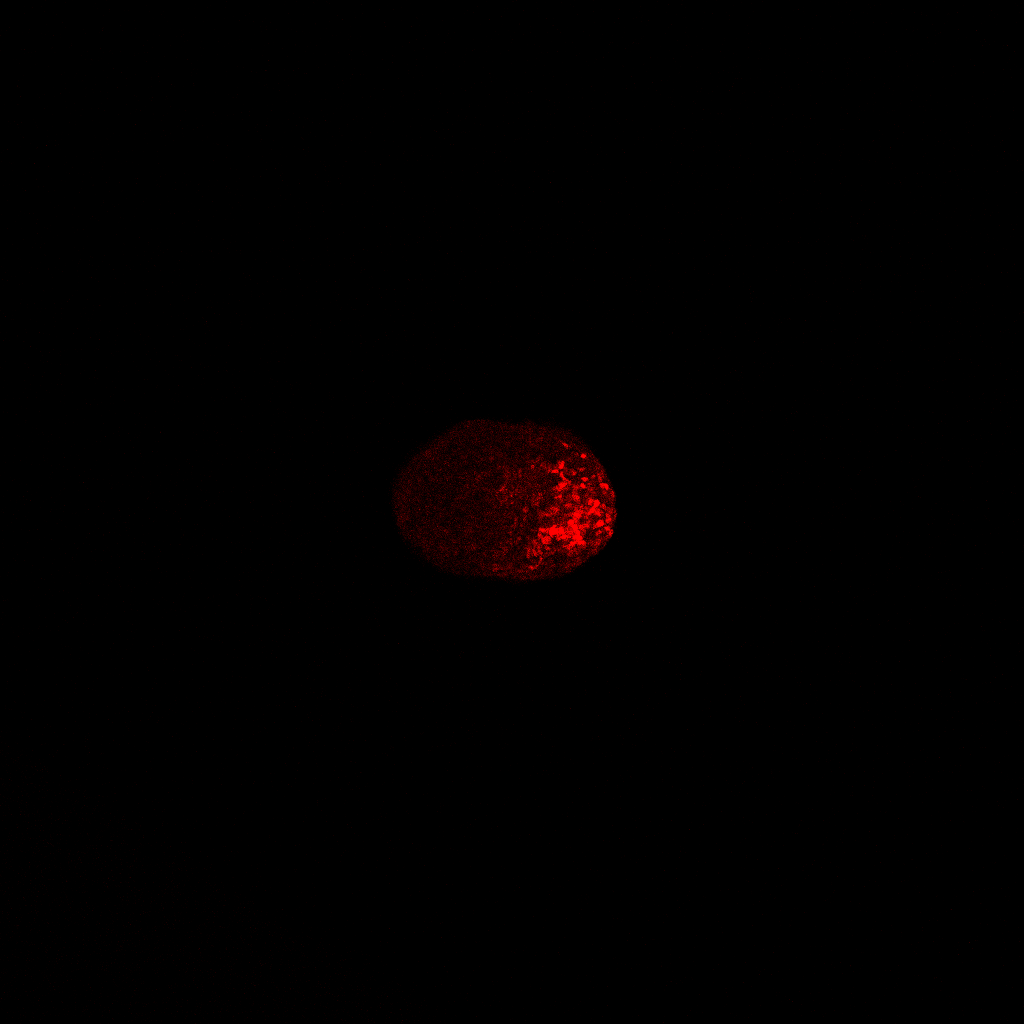

Supplement: Supplementary file 11 — Source data Fig. 9 [file 44318_2025_558_MOESM11_ESM.zip › Figure 9/panel 9D/KD-1 GFP_Bra/New-02-Image Export-04_c2.tif]

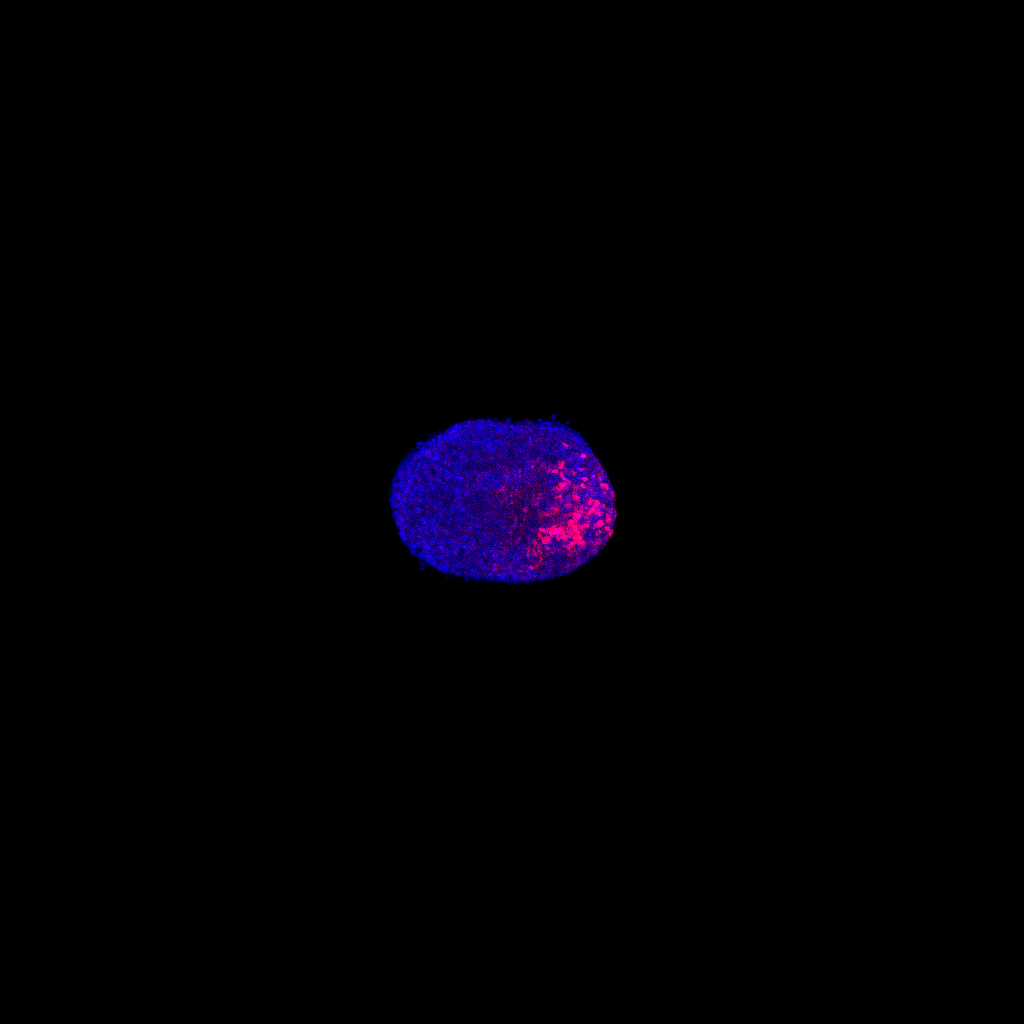

Supplement: Supplementary file 11 — Source data Fig. 9 [file 44318_2025_558_MOESM11_ESM.zip › Figure 9/panel 9D/KD-1 GFP_Bra/New-02-Image Export-04_c1-2.tif]

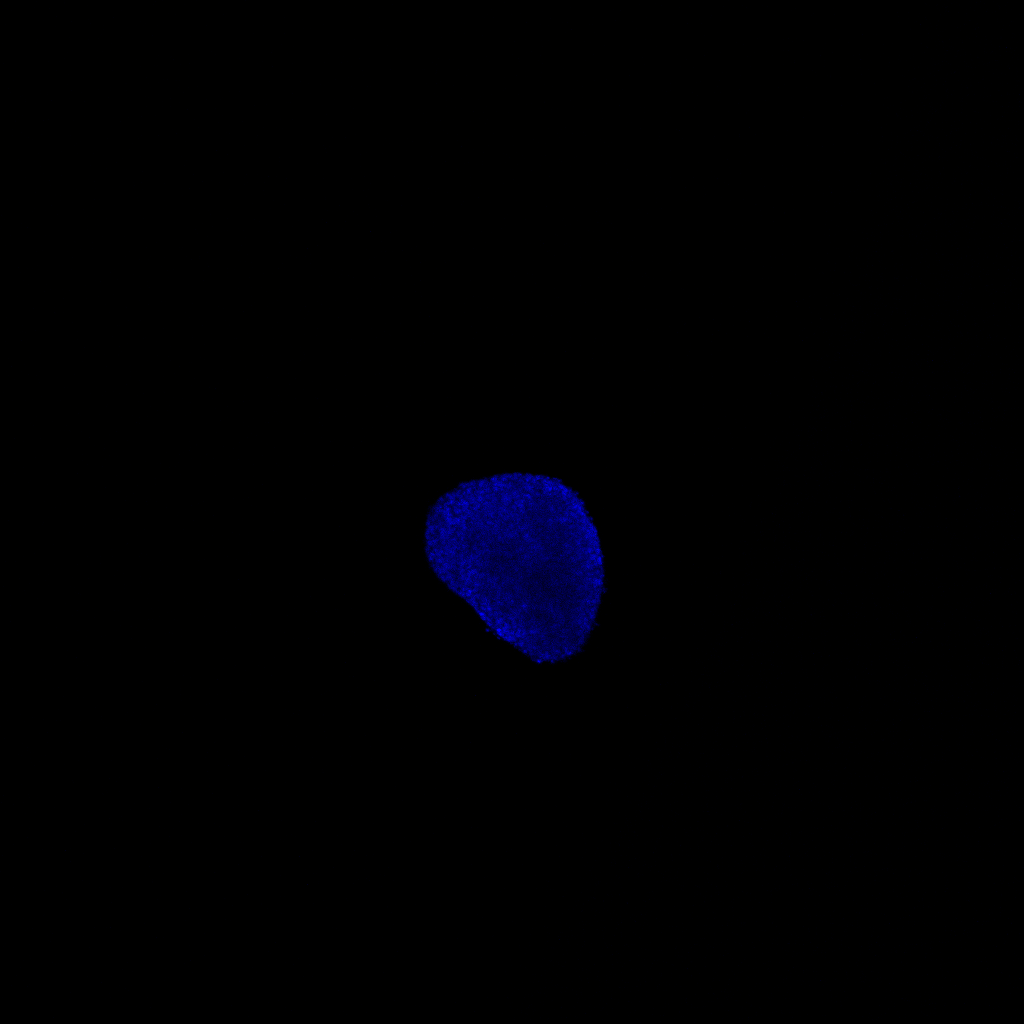

Supplement: Supplementary file 11 — Source data Fig. 9 [file 44318_2025_558_MOESM11_ESM.zip › Figure 9/panel 9D/KD-1 GFP_Oct4/New-02-Image Export-31_c1.tif]

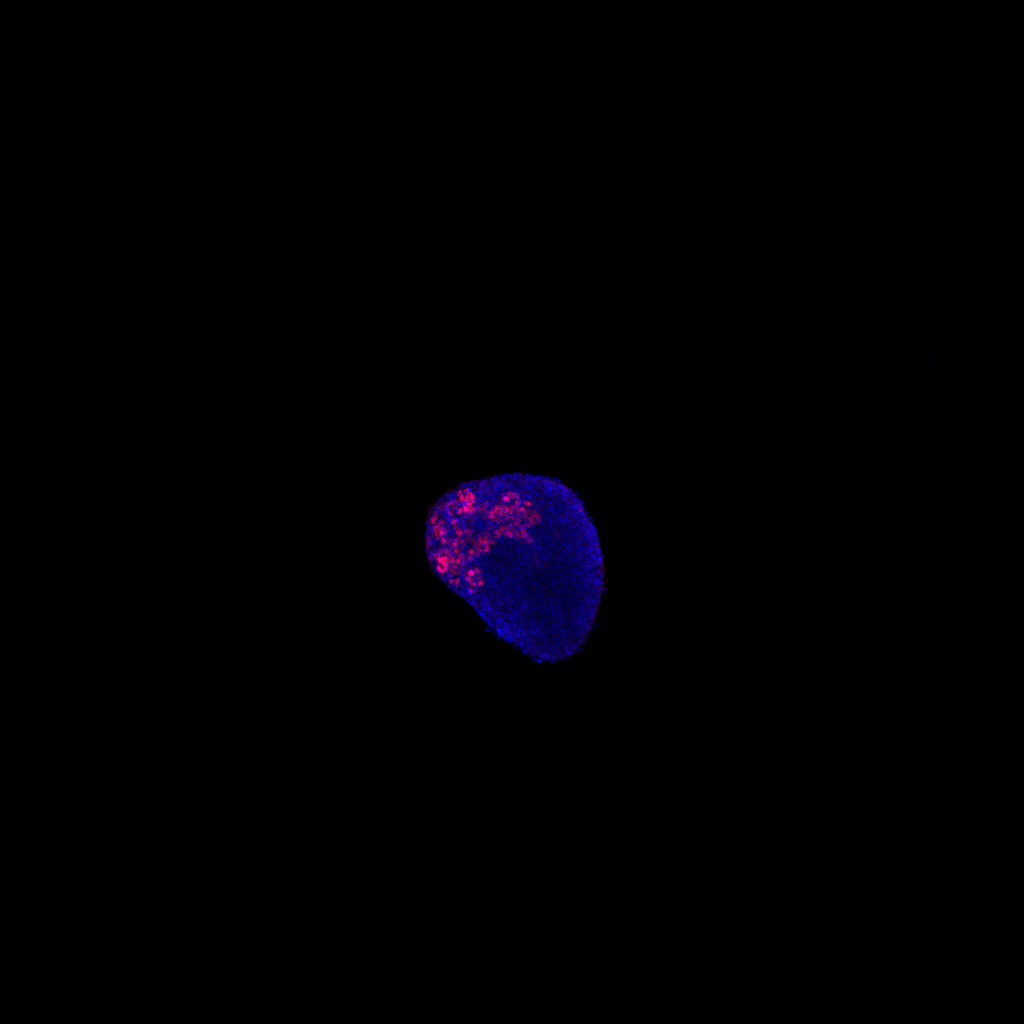

Supplement: Supplementary file 11 — Source data Fig. 9 [file 44318_2025_558_MOESM11_ESM.zip › Figure 9/panel 9D/KD-1 GFP_Oct4/New-02-Image Export-31_c1-2.tif]

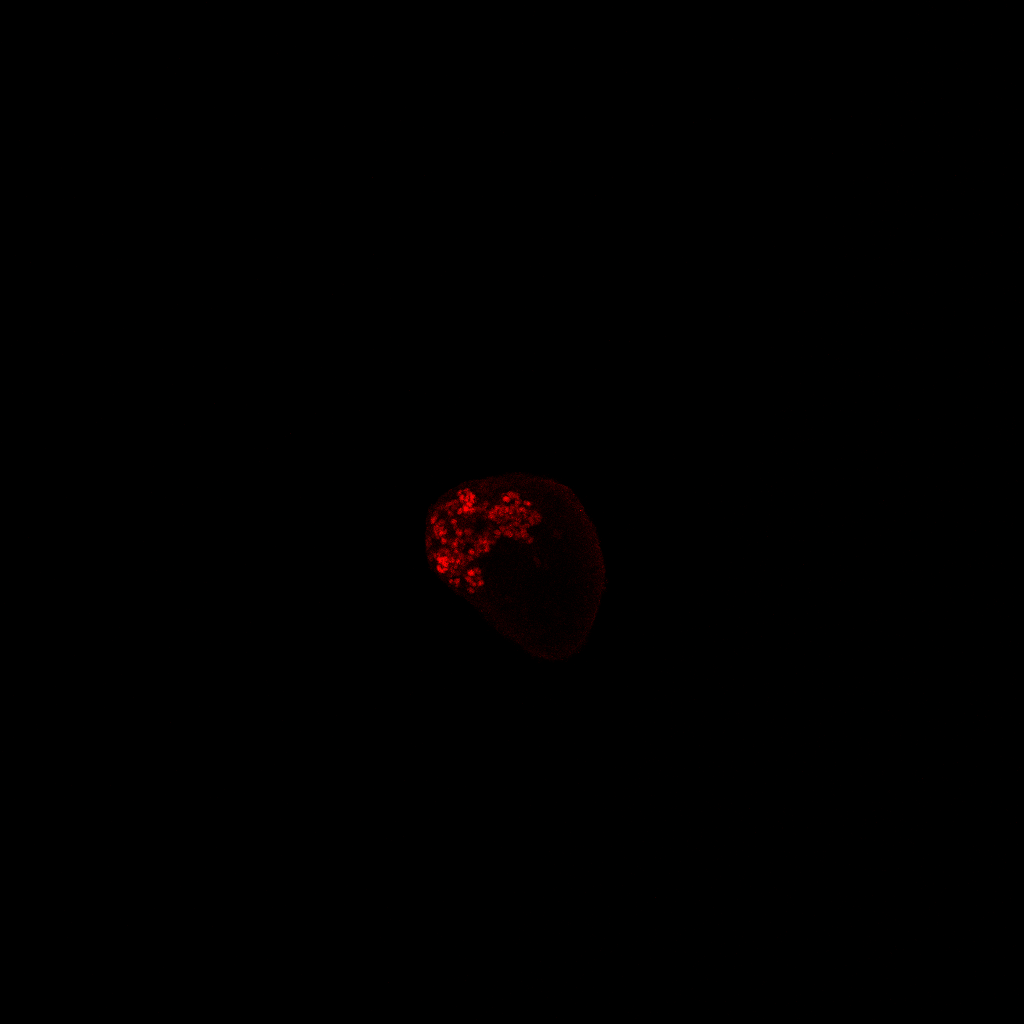

Supplement: Supplementary file 11 — Source data Fig. 9 [file 44318_2025_558_MOESM11_ESM.zip › Figure 9/panel 9D/KD-1 GFP_Oct4/New-02-Image Export-31_c2.tif]

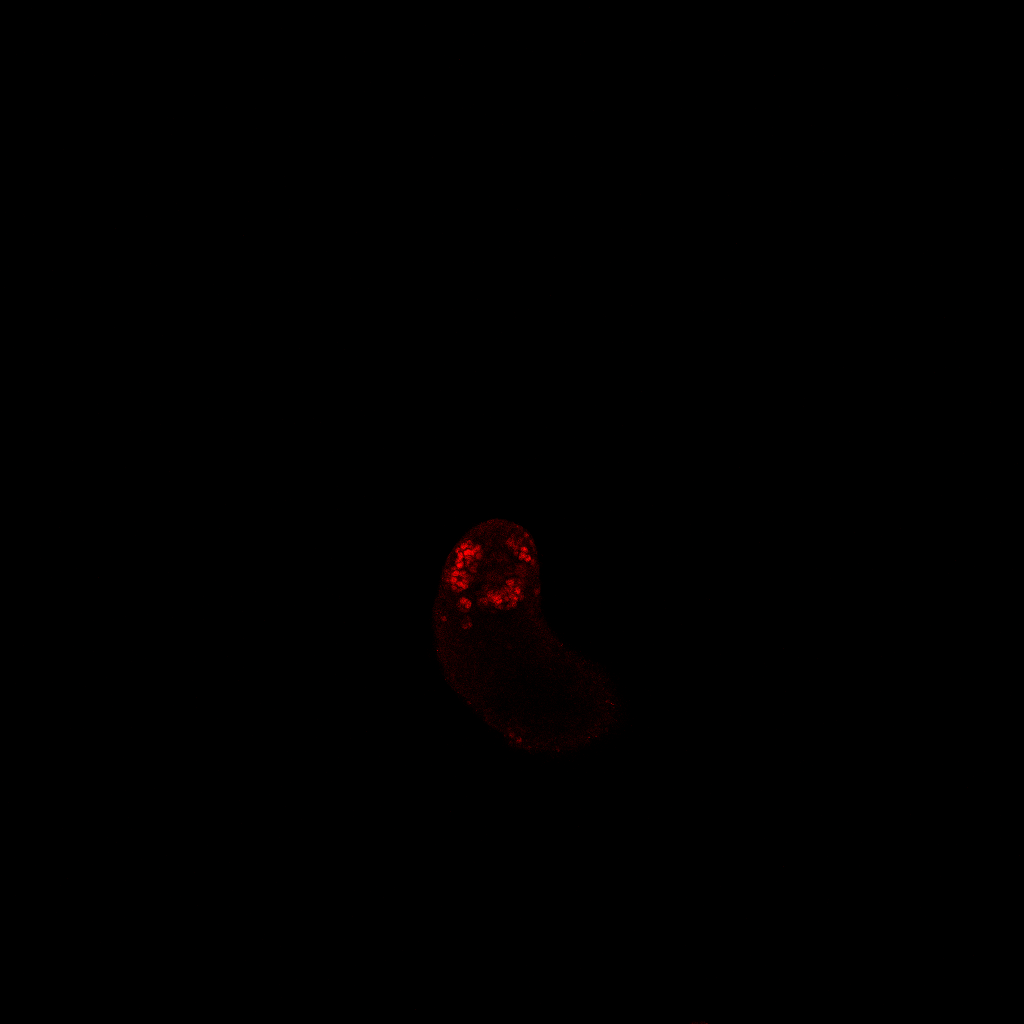

Supplement: Supplementary file 11 — Source data Fig. 9 [file 44318_2025_558_MOESM11_ESM.zip › Figure 9/panel 9D/KD-1 UC_Bra/New-02-Image Export-06_c2.tif]

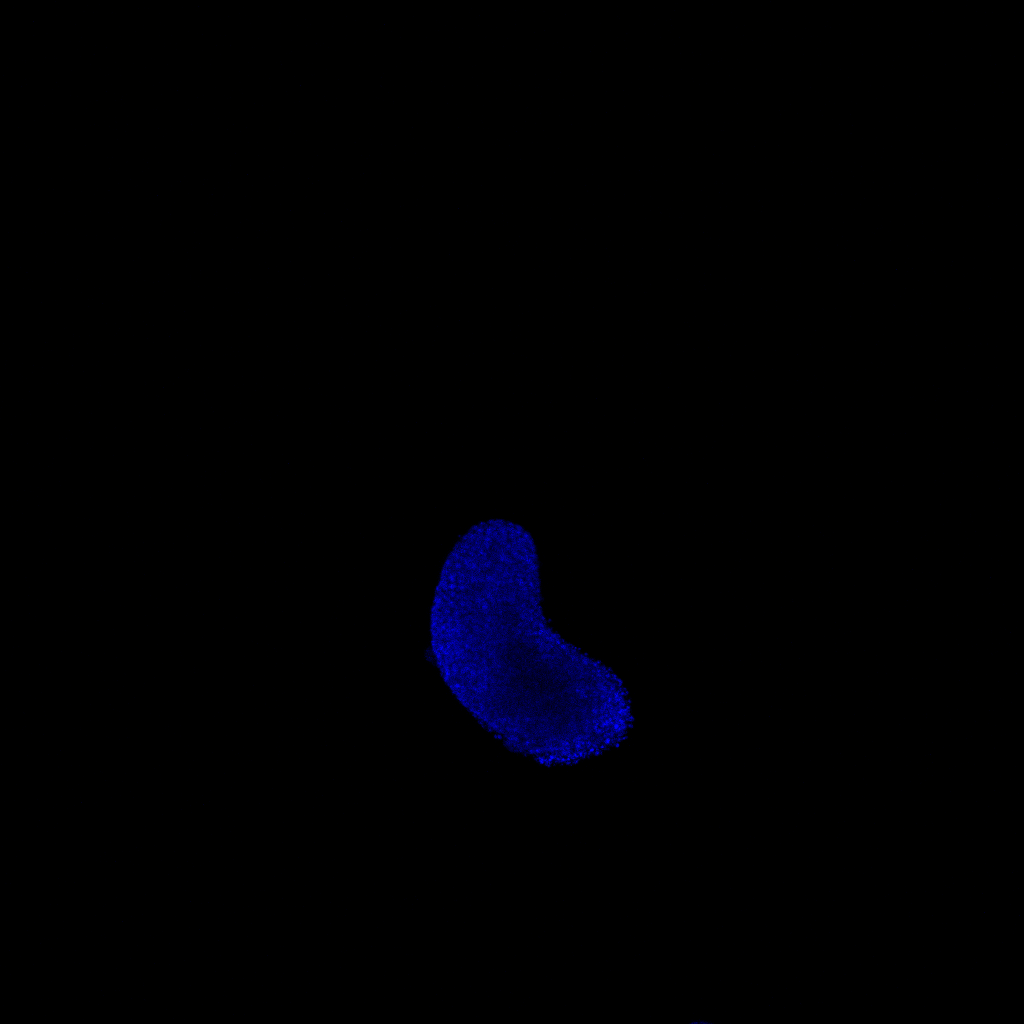

Supplement: Supplementary file 11 — Source data Fig. 9 [file 44318_2025_558_MOESM11_ESM.zip › Figure 9/panel 9D/KD-1 UC_Bra/New-02-Image Export-06_c1.tif]

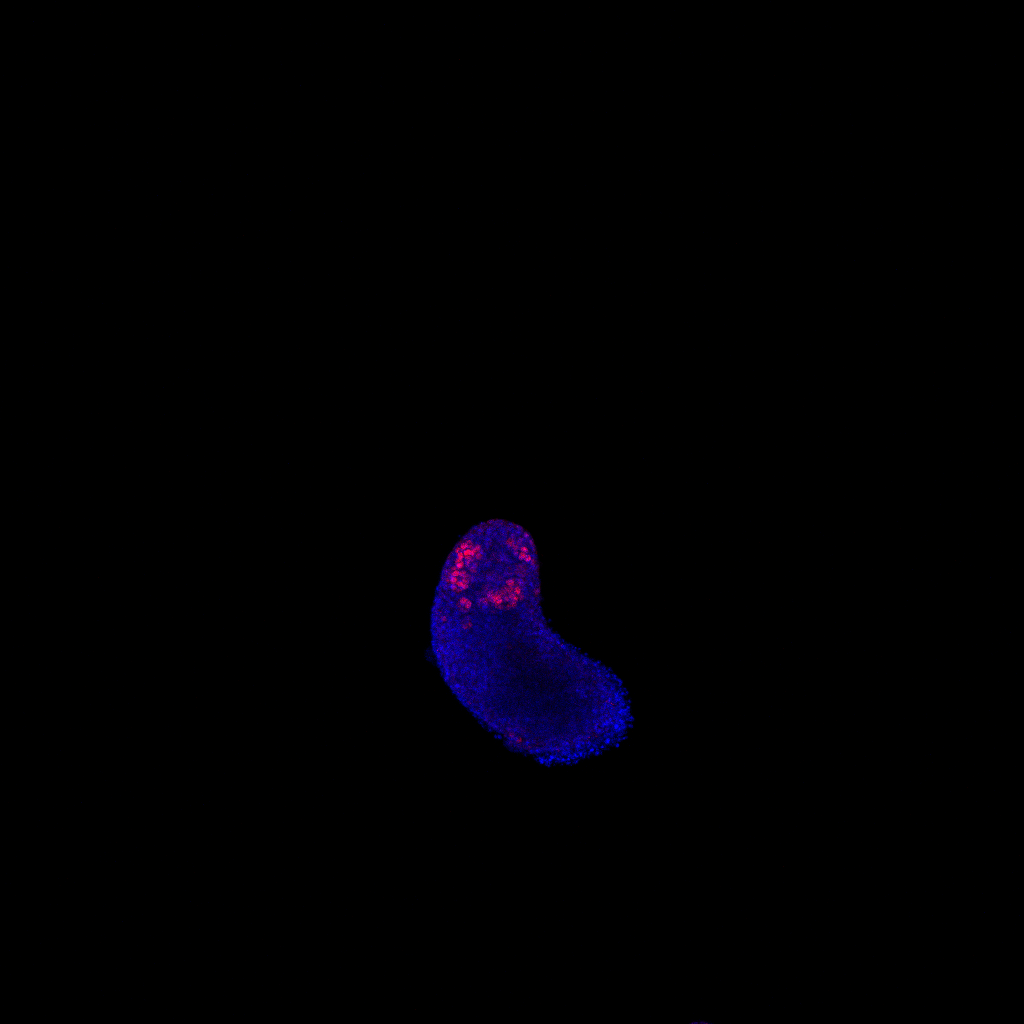

Supplement: Supplementary file 11 — Source data Fig. 9 [file 44318_2025_558_MOESM11_ESM.zip › Figure 9/panel 9D/KD-1 UC_Bra/New-02-Image Export-06_c1-2.tif]

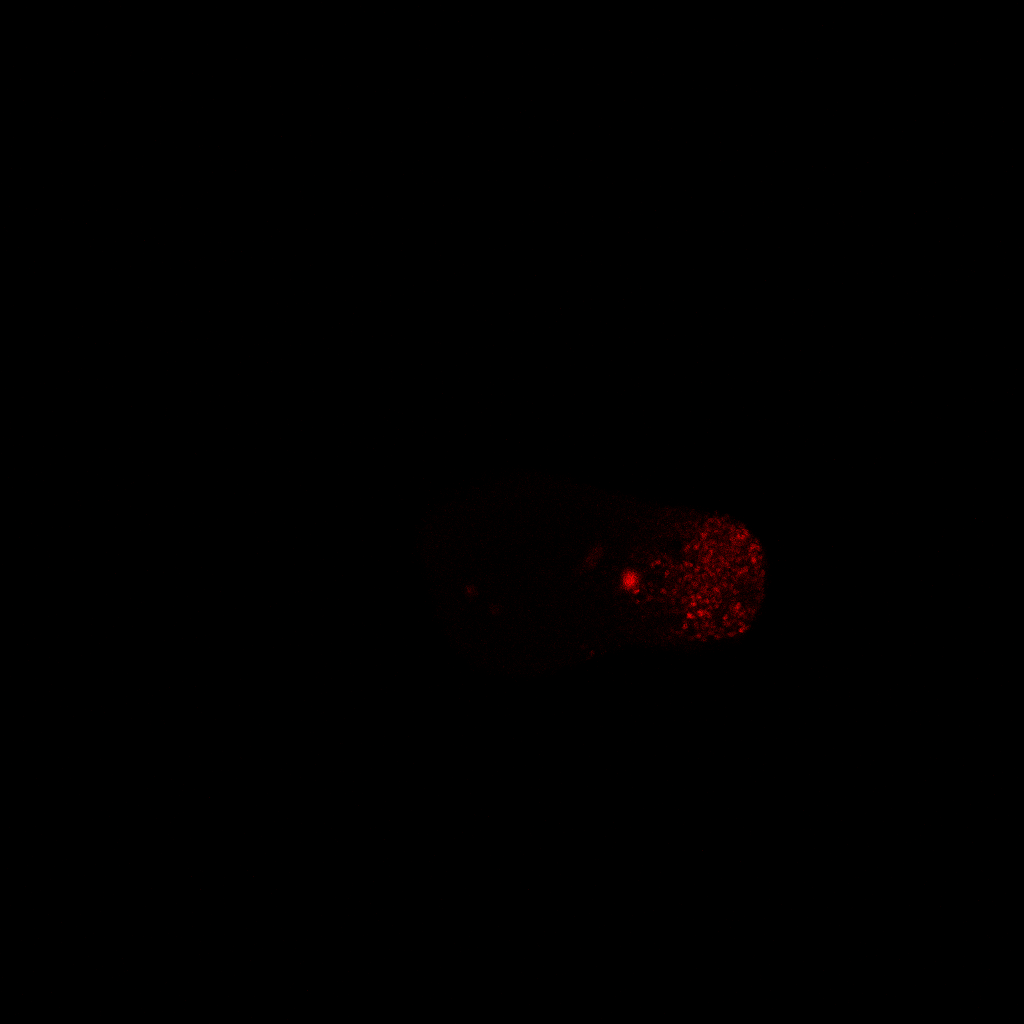

Supplement: Supplementary file 11 — Source data Fig. 9 [file 44318_2025_558_MOESM11_ESM.zip › Figure 9/panel 9D/NT_Oct4_2/New-01-Image Export-16_c2.tif]

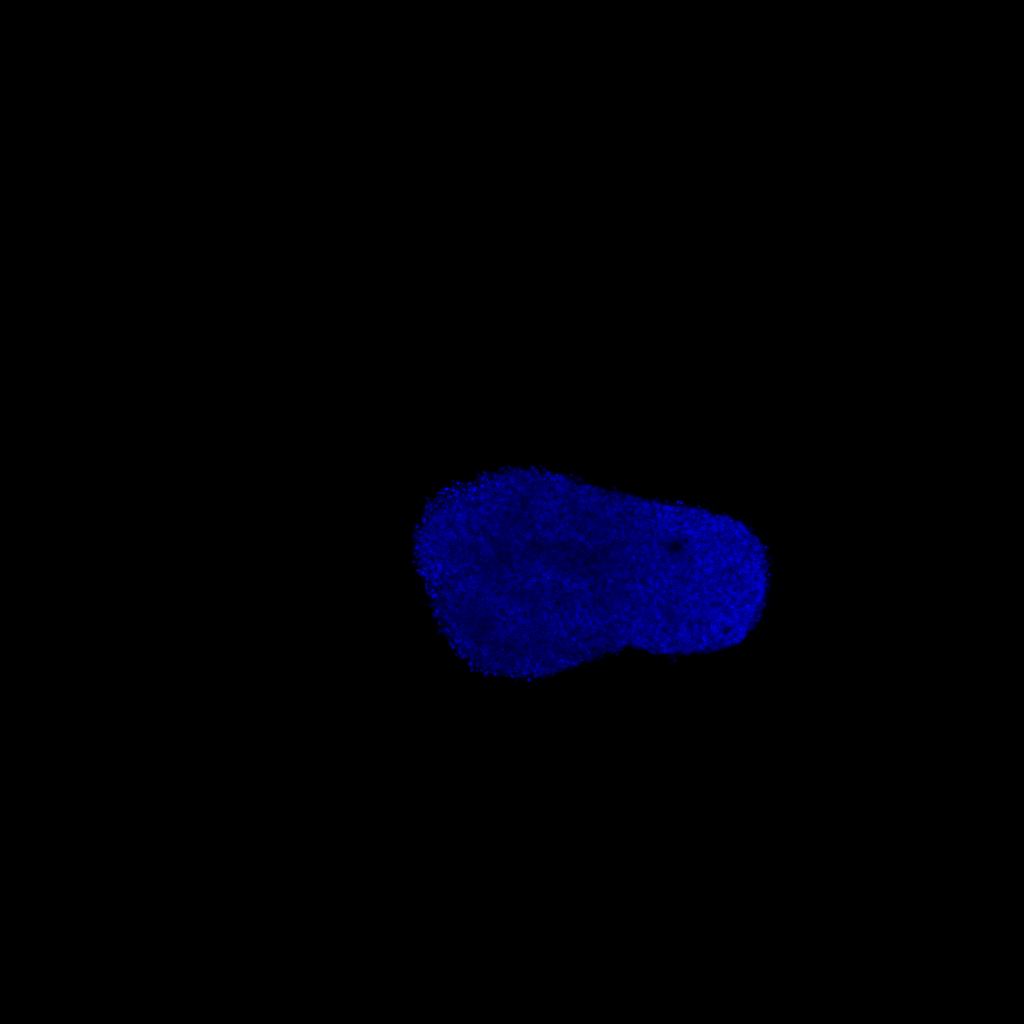

Supplement: Supplementary file 11 — Source data Fig. 9 [file 44318_2025_558_MOESM11_ESM.zip › Figure 9/panel 9D/NT_Oct4_2/New-01-Image Export-16_c1.tif]

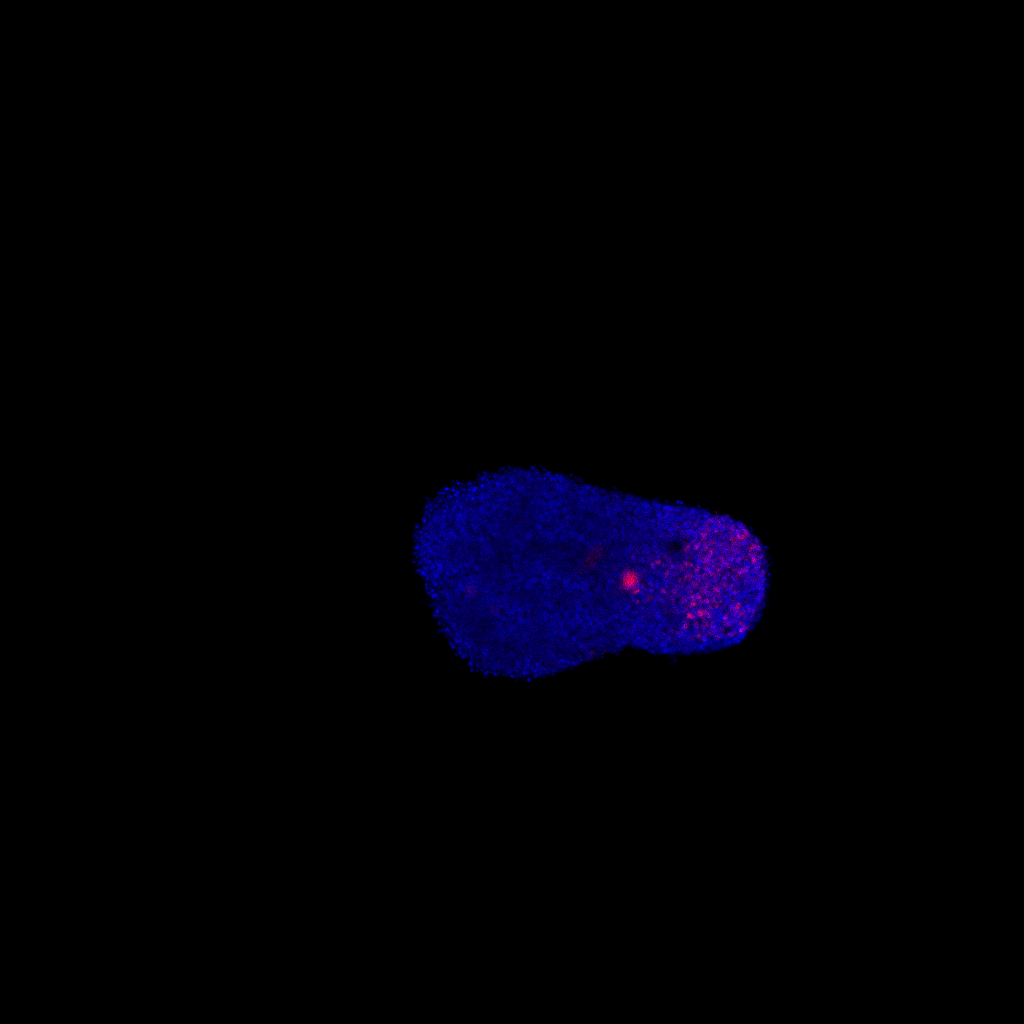

Supplement: Supplementary file 11 — Source data Fig. 9 [file 44318_2025_558_MOESM11_ESM.zip › Figure 9/panel 9D/NT_Oct4_2/New-01-Image Export-16_c1-2.tif]

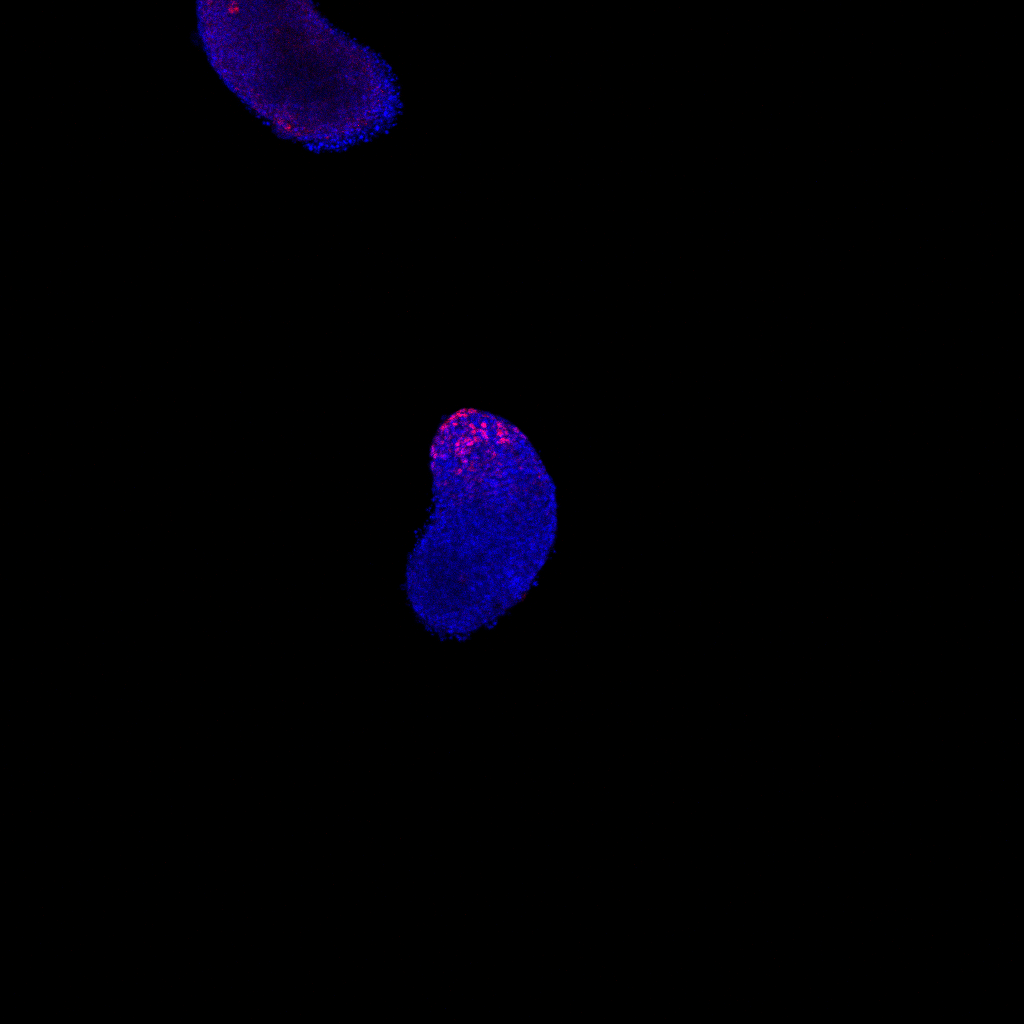

Supplement: Supplementary file 11 — Source data Fig. 9 [file 44318_2025_558_MOESM11_ESM.zip › Figure 9/panel 9D/NT_Bra_1/New-03-Image Export-07_c1-2.tif]

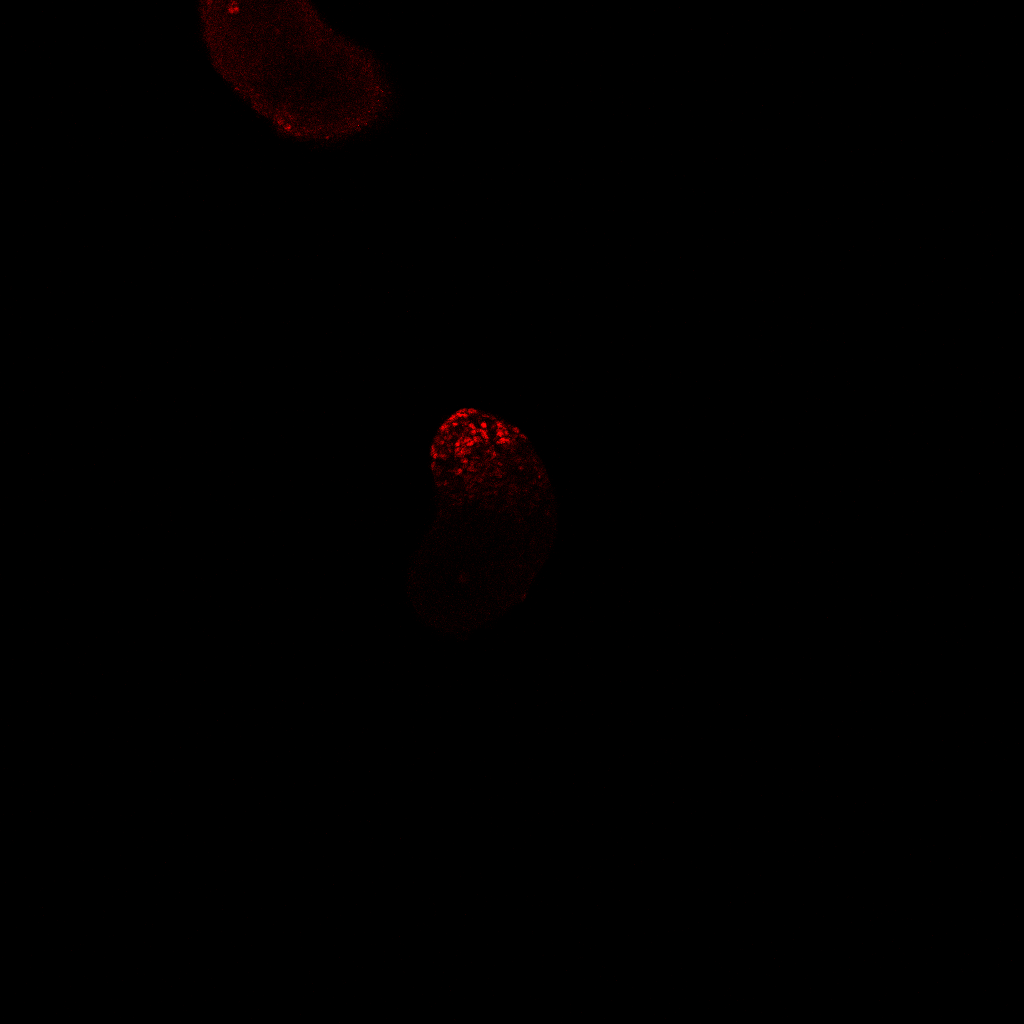

Supplement: Supplementary file 11 — Source data Fig. 9 [file 44318_2025_558_MOESM11_ESM.zip › Figure 9/panel 9D/NT_Bra_1/New-03-Image Export-07_c2.tif]

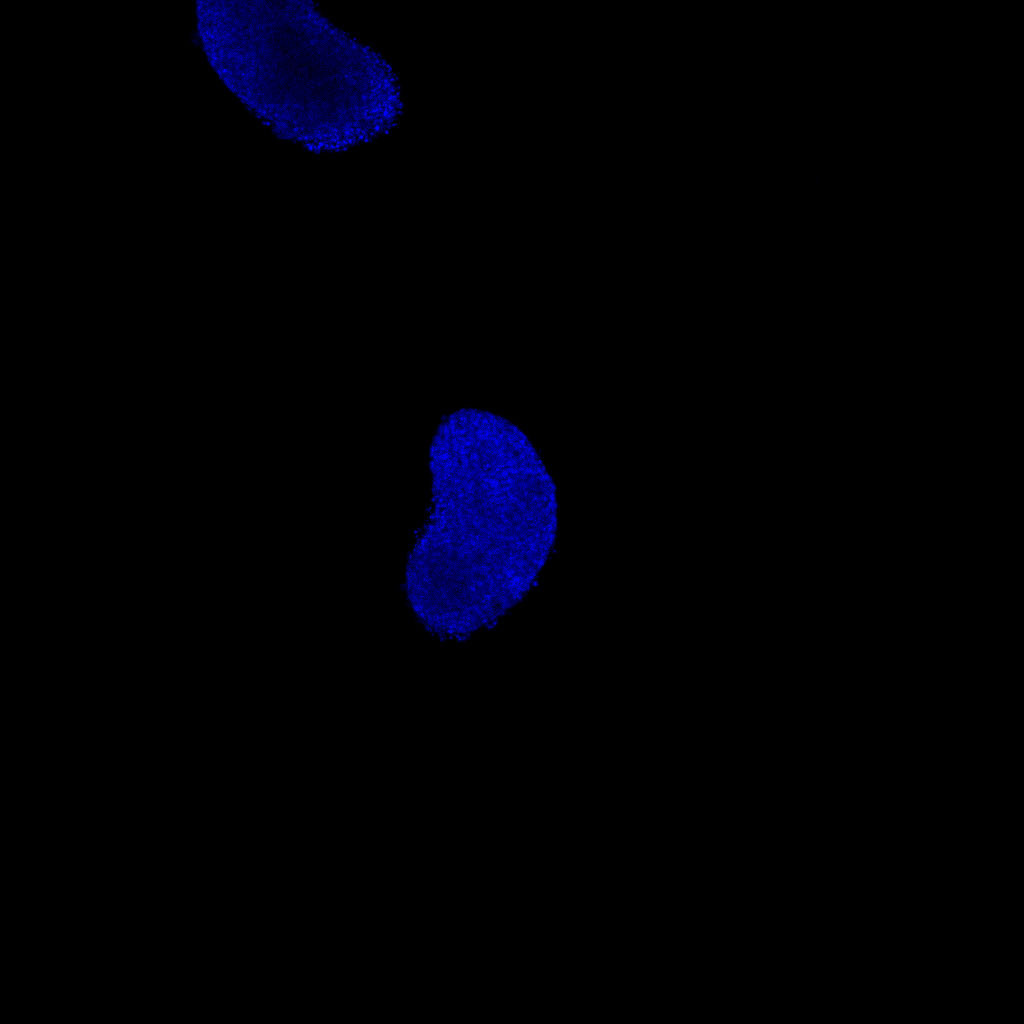

Supplement: Supplementary file 11 — Source data Fig. 9 [file 44318_2025_558_MOESM11_ESM.zip › Figure 9/panel 9D/NT_Bra_1/New-03-Image Export-07_c1.tif]

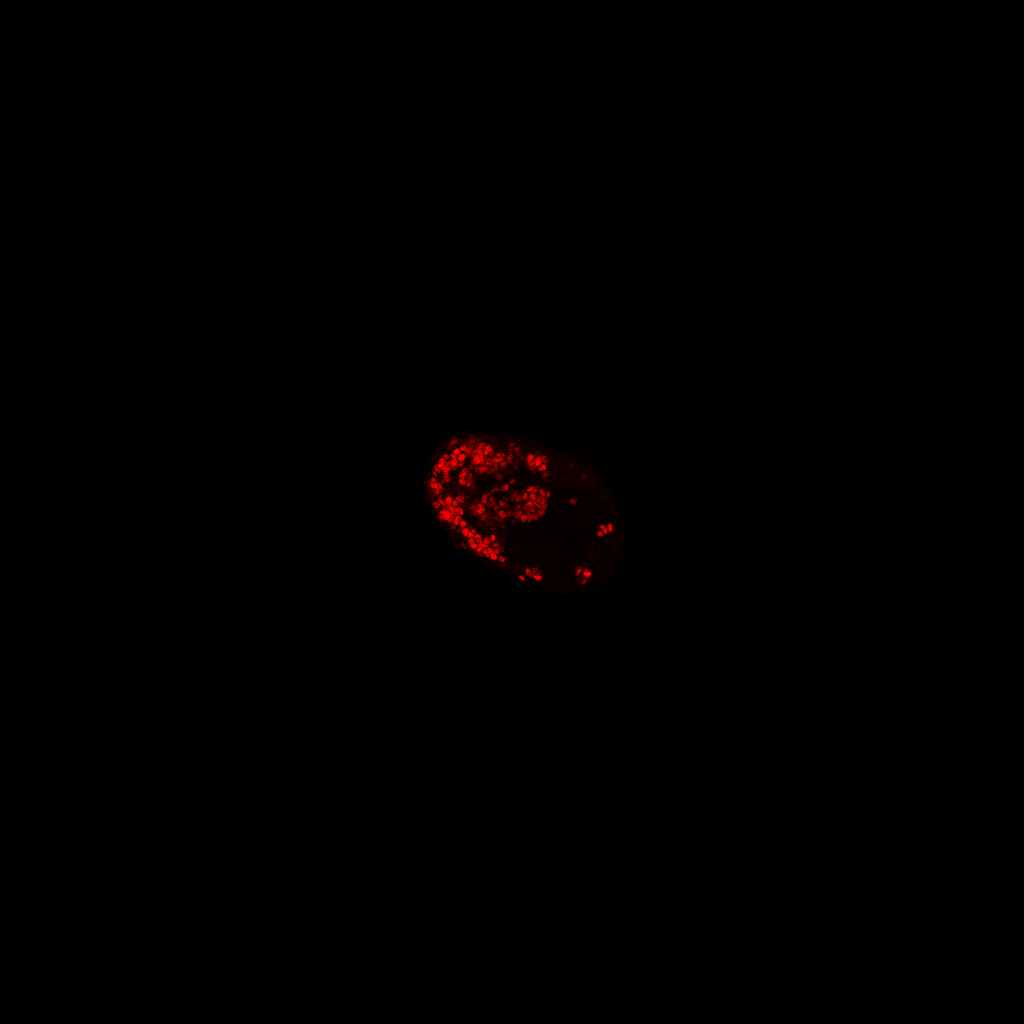

Supplement: Supplementary file 11 — Source data Fig. 9 [file 44318_2025_558_MOESM11_ESM.zip › Figure 9/panel 9D/KD-1_Oct4/New-03-Image Export-20_c2.tif]

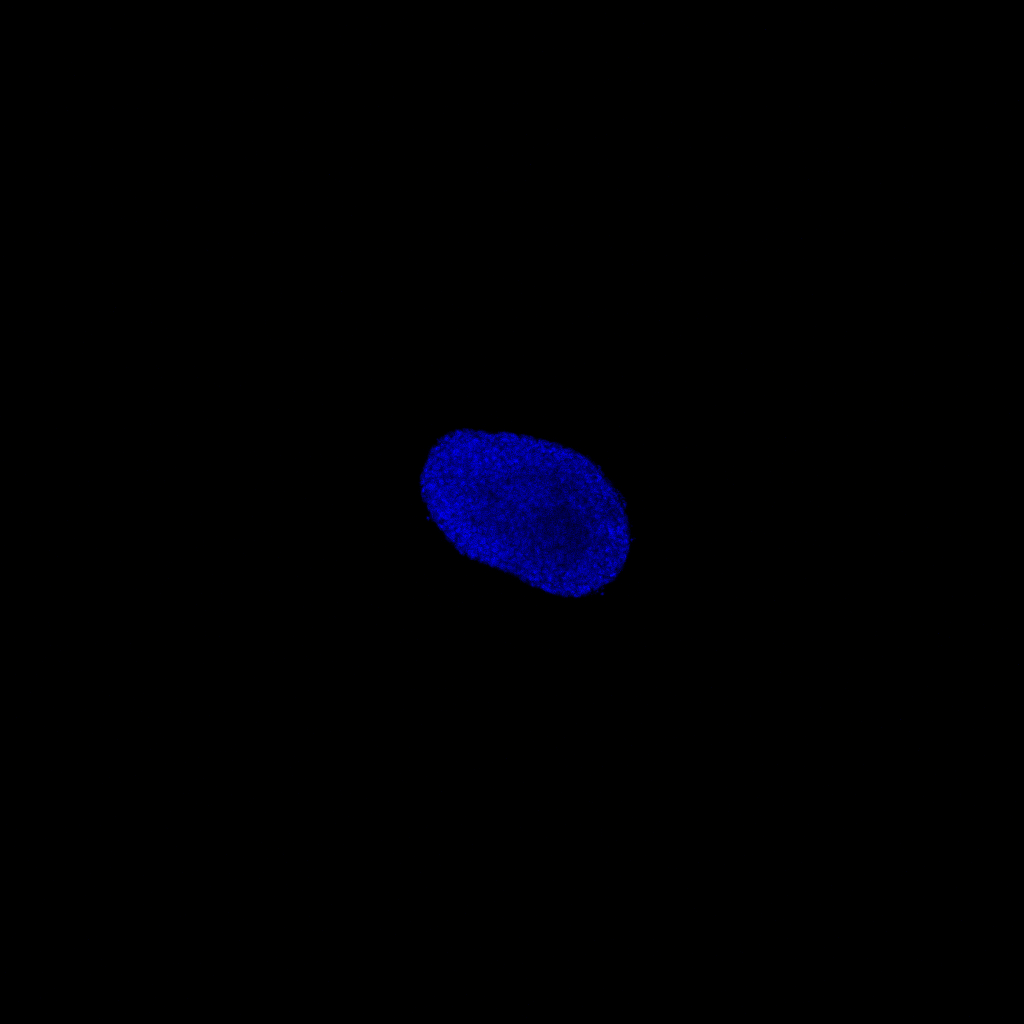

Supplement: Supplementary file 11 — Source data Fig. 9 [file 44318_2025_558_MOESM11_ESM.zip › Figure 9/panel 9D/KD-1_Oct4/New-03-Image Export-20_c1.tif]

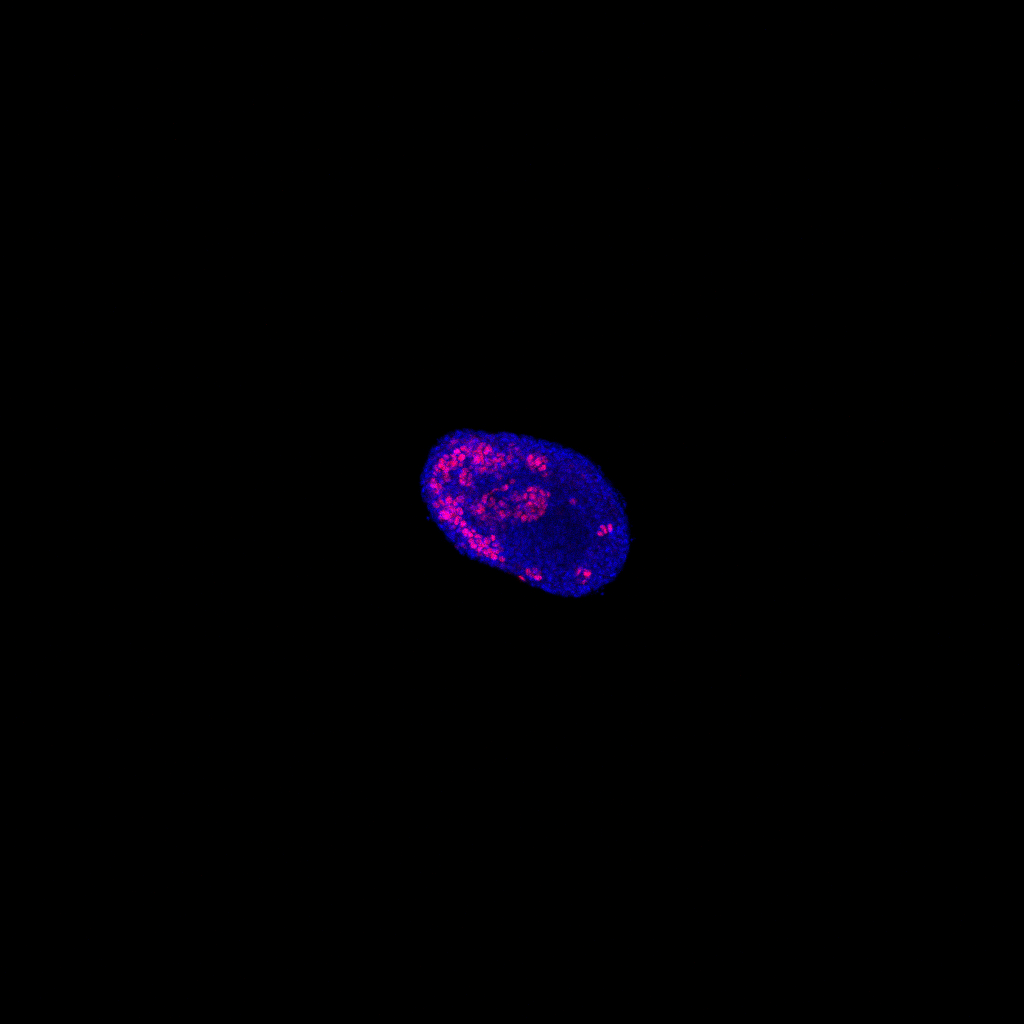

Supplement: Supplementary file 11 — Source data Fig. 9 [file 44318_2025_558_MOESM11_ESM.zip › Figure 9/panel 9D/KD-1_Oct4/New-03-Image Export-20_c1-2.tif]

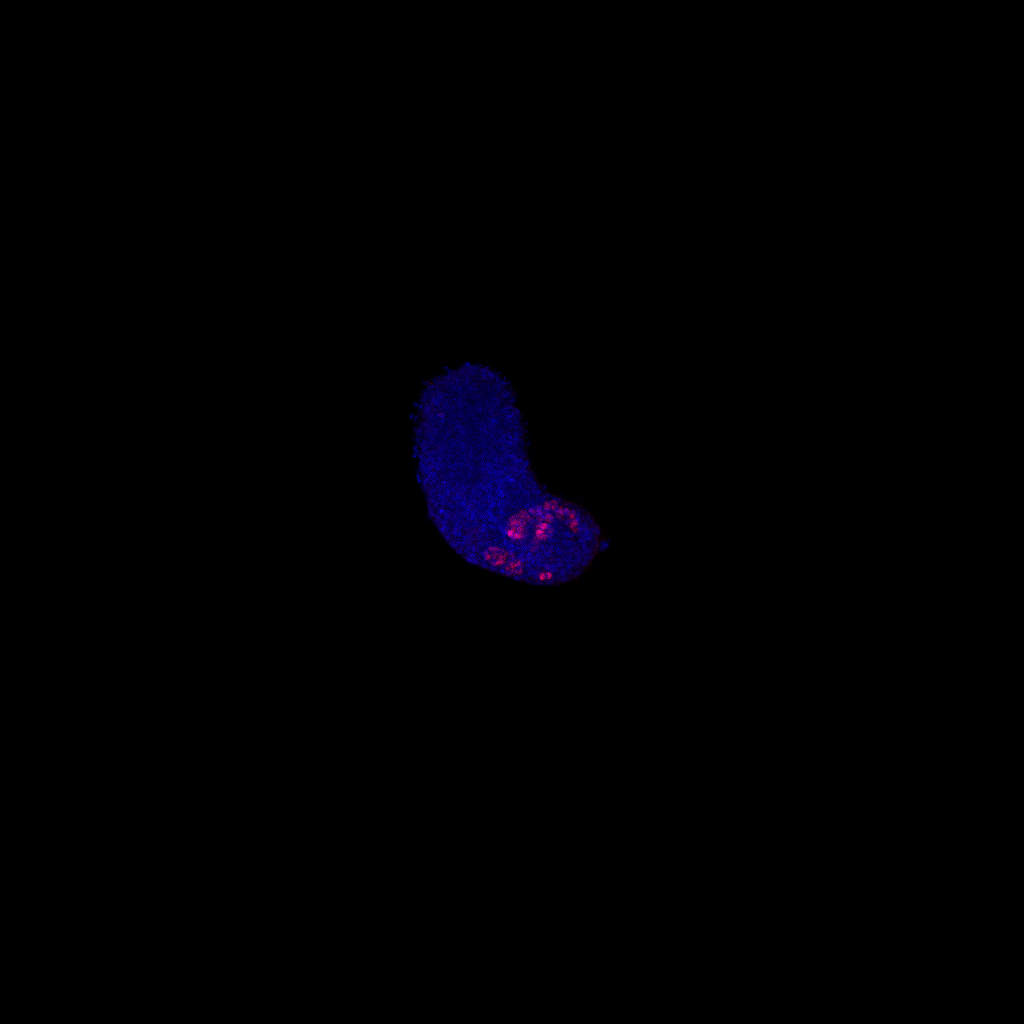

Supplement: Supplementary file 11 — Source data Fig. 9 [file 44318_2025_558_MOESM11_ESM.zip › Figure 9/panel 9D/KD-1 UC _Oct4/New-01-Image Export-21_c1-2.tif]

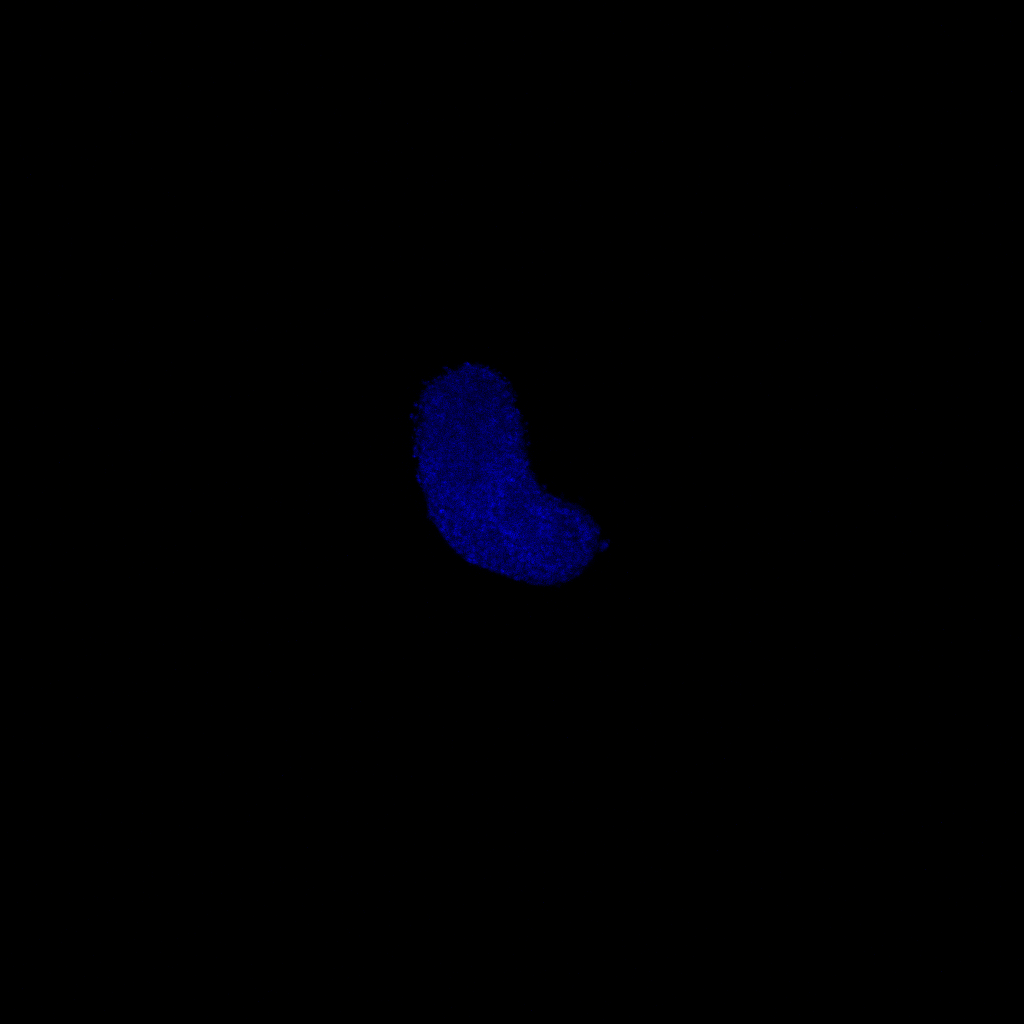

Supplement: Supplementary file 11 — Source data Fig. 9 [file 44318_2025_558_MOESM11_ESM.zip › Figure 9/panel 9D/KD-1 UC _Oct4/New-01-Image Export-21_c1.tif]

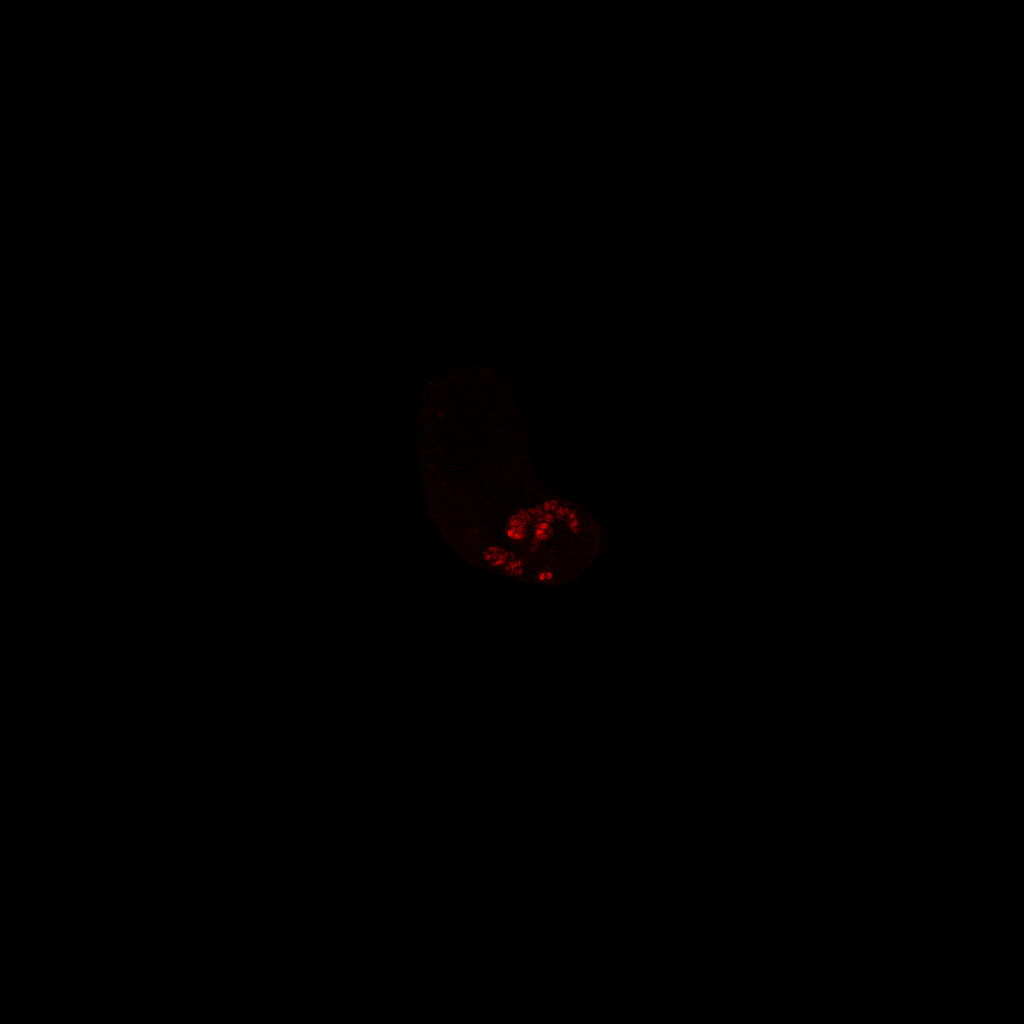

Supplement: Supplementary file 11 — Source data Fig. 9 [file 44318_2025_558_MOESM11_ESM.zip › Figure 9/panel 9D/KD-1 UC _Oct4/New-01-Image Export-21_c2.tif]

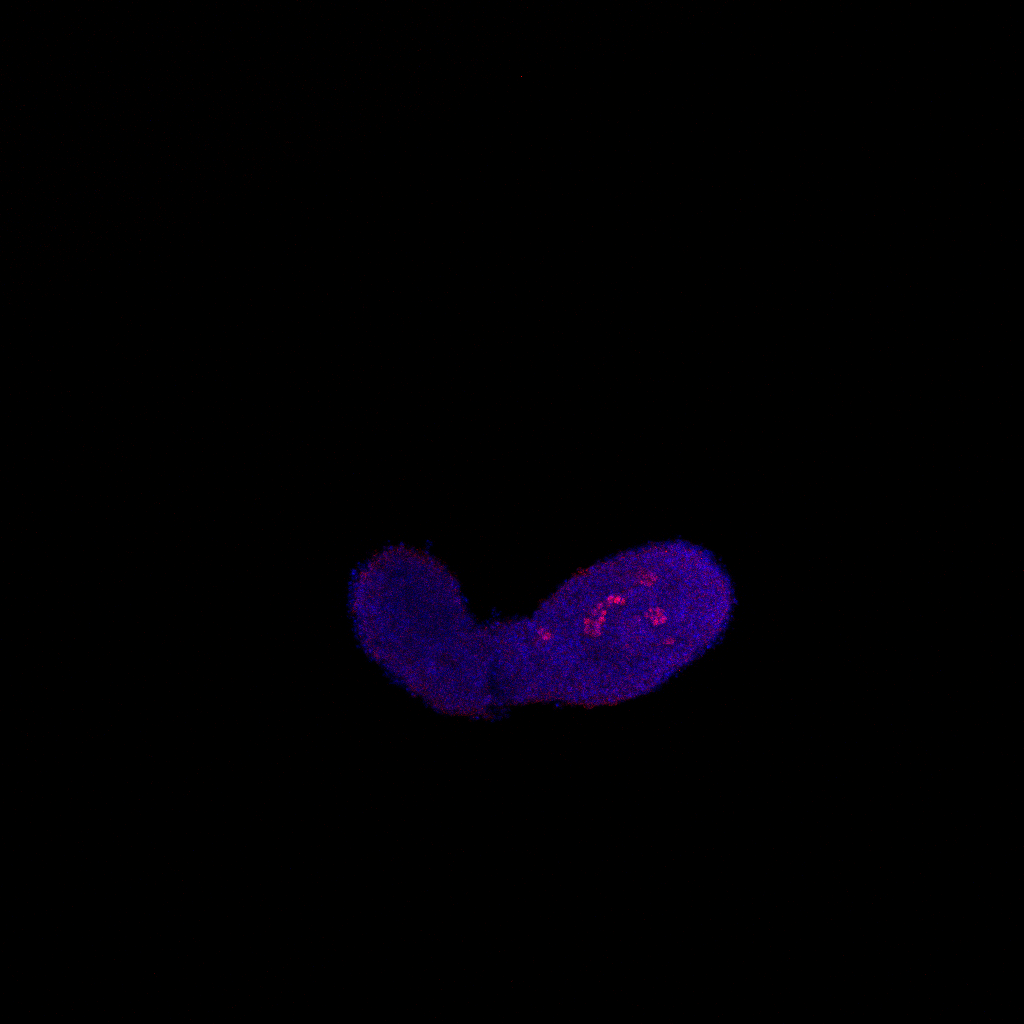

Supplement: Supplementary file 11 — Source data Fig. 9 [file 44318_2025_558_MOESM11_ESM.zip › Figure 9/panel 9D/KD-2 UC_Oct4/New-03-Image Export-23_c1-2.tif]

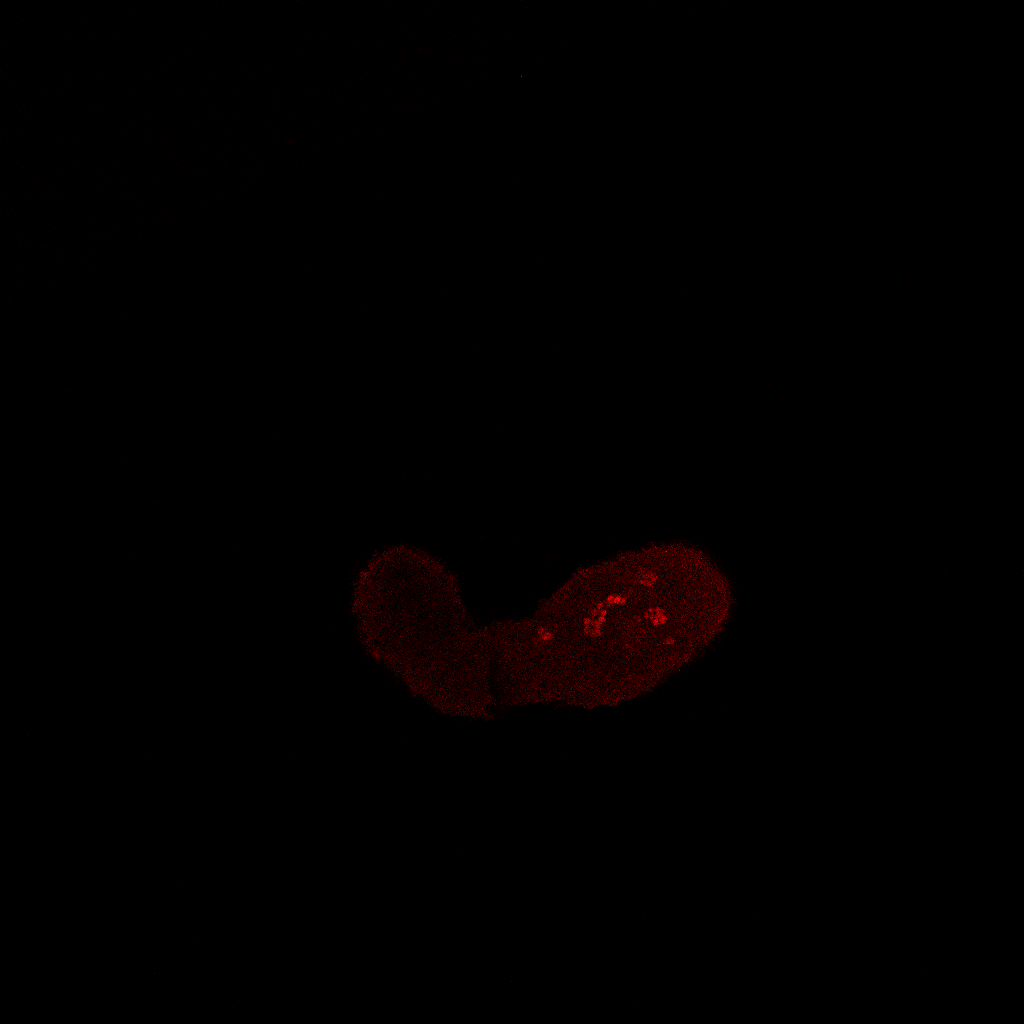

Supplement: Supplementary file 11 — Source data Fig. 9 [file 44318_2025_558_MOESM11_ESM.zip › Figure 9/panel 9D/KD-2 UC_Oct4/New-03-Image Export-23_c2.tif]

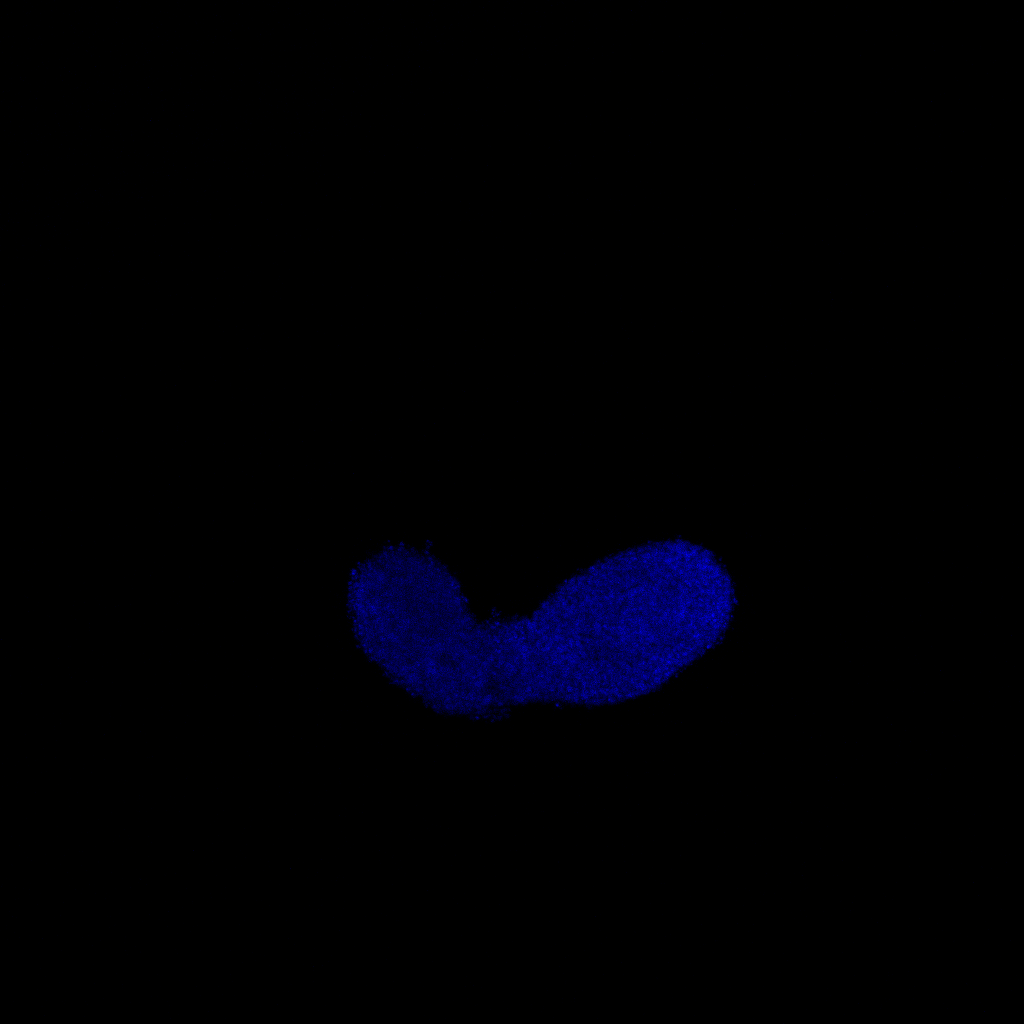

Supplement: Supplementary file 11 — Source data Fig. 9 [file 44318_2025_558_MOESM11_ESM.zip › Figure 9/panel 9D/KD-2 UC_Oct4/New-03-Image Export-23_c1.tif]
