# Supplementary material for: Non-cell-autonomous control of mouse gastruloid development by the ultra-conserved lncRNA T-UCstem1
Source: EMBO J. 2025 Oct 31;44(24):7620–48. doi: 10.1038/s44318-025-00558-2 (PMC12706062; doi:10.1038/s44318-025-00558-2)
Supplement: Supplementary file 13 — Expanded View Figures [file 44318_2025_558_MOESM13_ESM.pdf]

Expanded View Figures

Figure EV1. ***T-UCstem1* conservation across species and shape heterogeneity showed by *T-UCstem1* KD aggregates.**

(**A**) In red NM_1138112 is *T-UCstem1*, while uc.170 is the ultra-conserved sequence by Bejerano et al, 2004 *Cons 35 verts* is the phyloP plot, with sites predicted to be conserved are assigned positive scores (shown in blue), while sites predicted to be fast evolving are assigned negative scores (shown in red). Multiz Alignments of 35 Vertebrates depicts pairwise alignments of some species to the mouse genome indicating alignment quality. (**B**) The figure showing the heterogeneity of *T-UCstem1* KD gastruloids compared to Control (NT). Scale bar, 150 µm ($n = 4$ independent experiments; 30 gastruloids/condition).

© The Author(s)

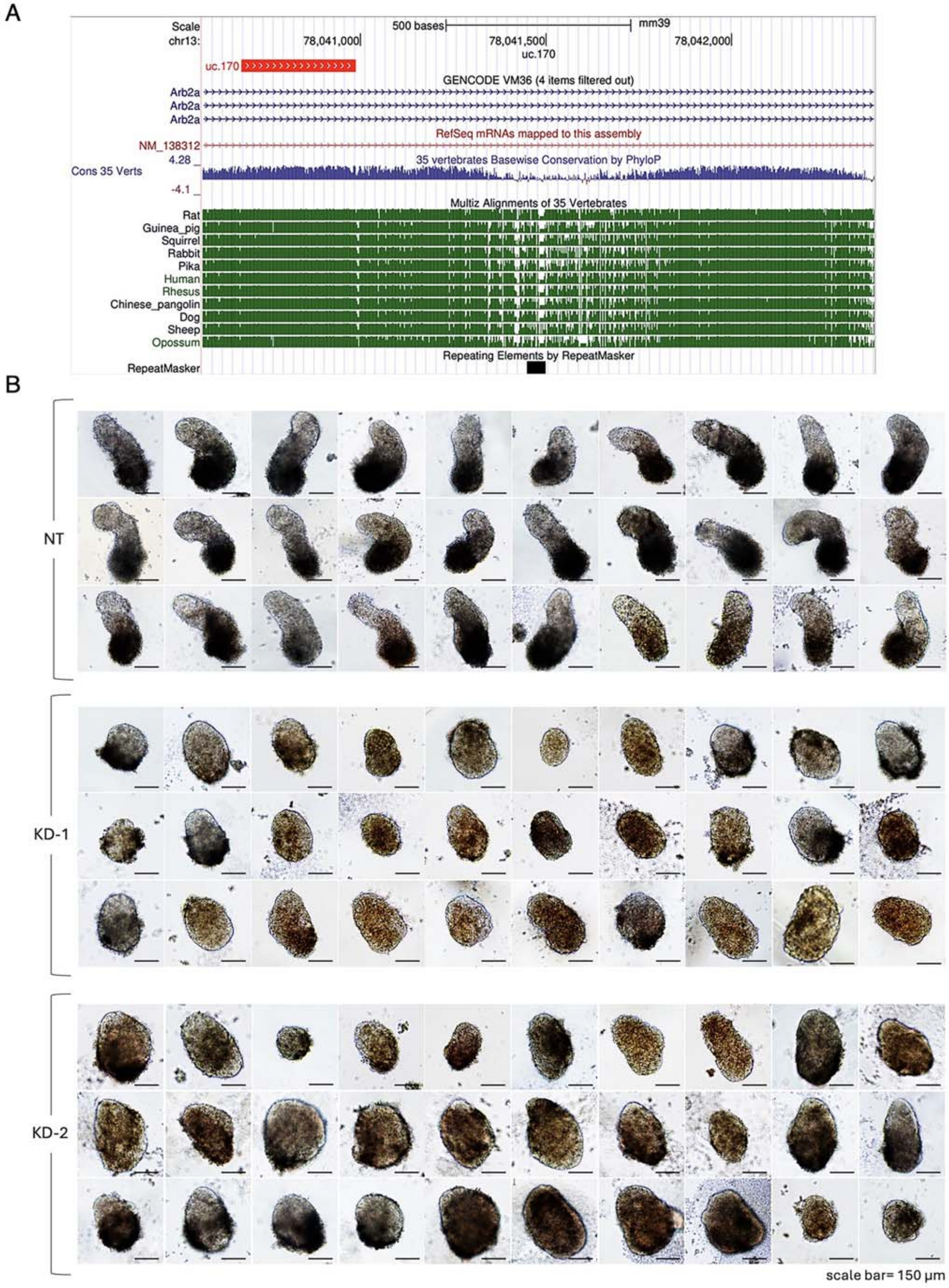
A
Scale
chr13:
500 bases
mm39
78,041,000
78,041,500
78,042,000
uc.170
uc.170
GENCODE VM36 (4 items filtered out)
Arb2a
Arb2a
Arb2a
RefSeq mRNAs mapped to this assembly
NM_138312
4.28
35 vertebrates Basewise Conservation by PhyloP
Cons 35 Verts
-4.1
Multiz Alignments of 35 Vertebrates
Rat
Guinea_pig
Squirrel
Rabbit
Pika
Human
Rhesus
Chinese_pangolin
Dog
Sheep
Opossum
Repeating Elements by RepeatMasker
RepeatMasker
B
NT
KD-1
KD-2
scale bar= 150 μm

 © The Author(s)

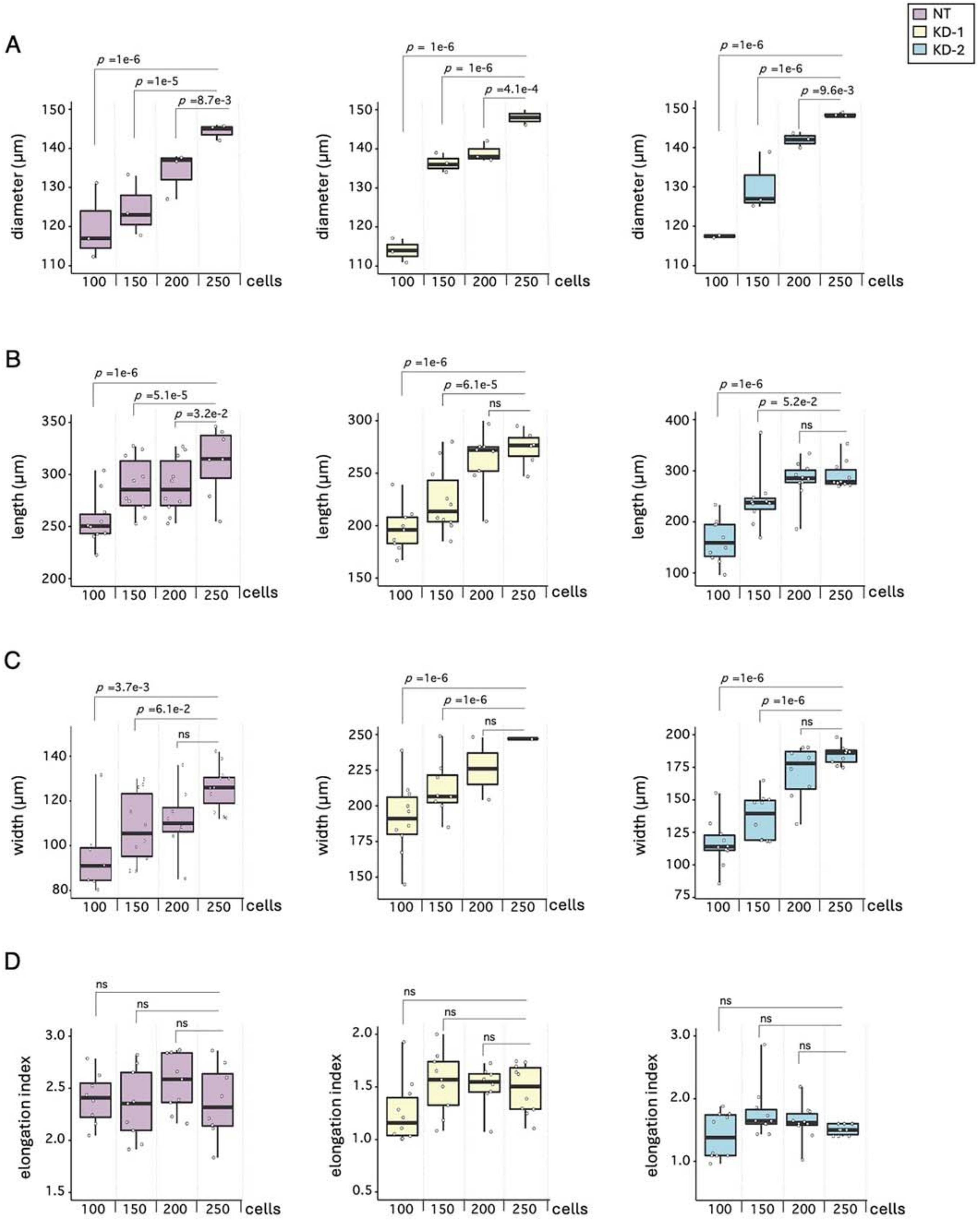
NT
KD-1
KD-2
A
p =1e-6
p =1e-5
p =8.7e-3
diameter (µm)
100 150 200 250 cells
p = 1e-6
p = 1e-6
p =4.1e-4
p =1e-6
p =1e-6
p =9.6e-3
B
p =1e-6
p =5.1e-5
p =3.2e-2
length (µm)
p =1e-6
p =6.1e-5
ns
p =1e-6
p = 5.2e-2
ns
C
p =3.7e-3
p =6.1e-2
ns
width (µm)
p =1e-6
p =1e-6
ns
p =1e-6
p =1e-6
ns
D
ns
ns
ns
elongation index

Figure EV2. *T-UCstem1* KD effect is independent on the initial cell number.

(**A**) Boxplot diagrams of the aggregate diameter at 48 h of NT (left), KD-1 (middle) and KD-2 (right). Boxplots display the minimum, first quartile, median, third quartile, and maximum. Statistical significance was assessed by one-way ANOVA with Tukey's multiple comparison test. *P* values of ≤0.05 were considered statistically significant. (**B–D**) Boxplot diagrams of the gastruloids length (**B**), width (**C**) and elongation index (**D**) at 120 h of NT (left), *T-UCstem1* KD-1 (middle) and *T-UCstem1* KD-2 (right). Data are shown as mean ± SD ($n = 2$ independent experiments; 10 gastruloids/condition). Boxplots display the minimum, first quartile, median, third quartile, and maximum. Statistical significance was assessed by one-way ANOVA with Tukey's multiple comparison test. *P* values of ≤0.05 were considered statistically significant.

 © The Author(s)

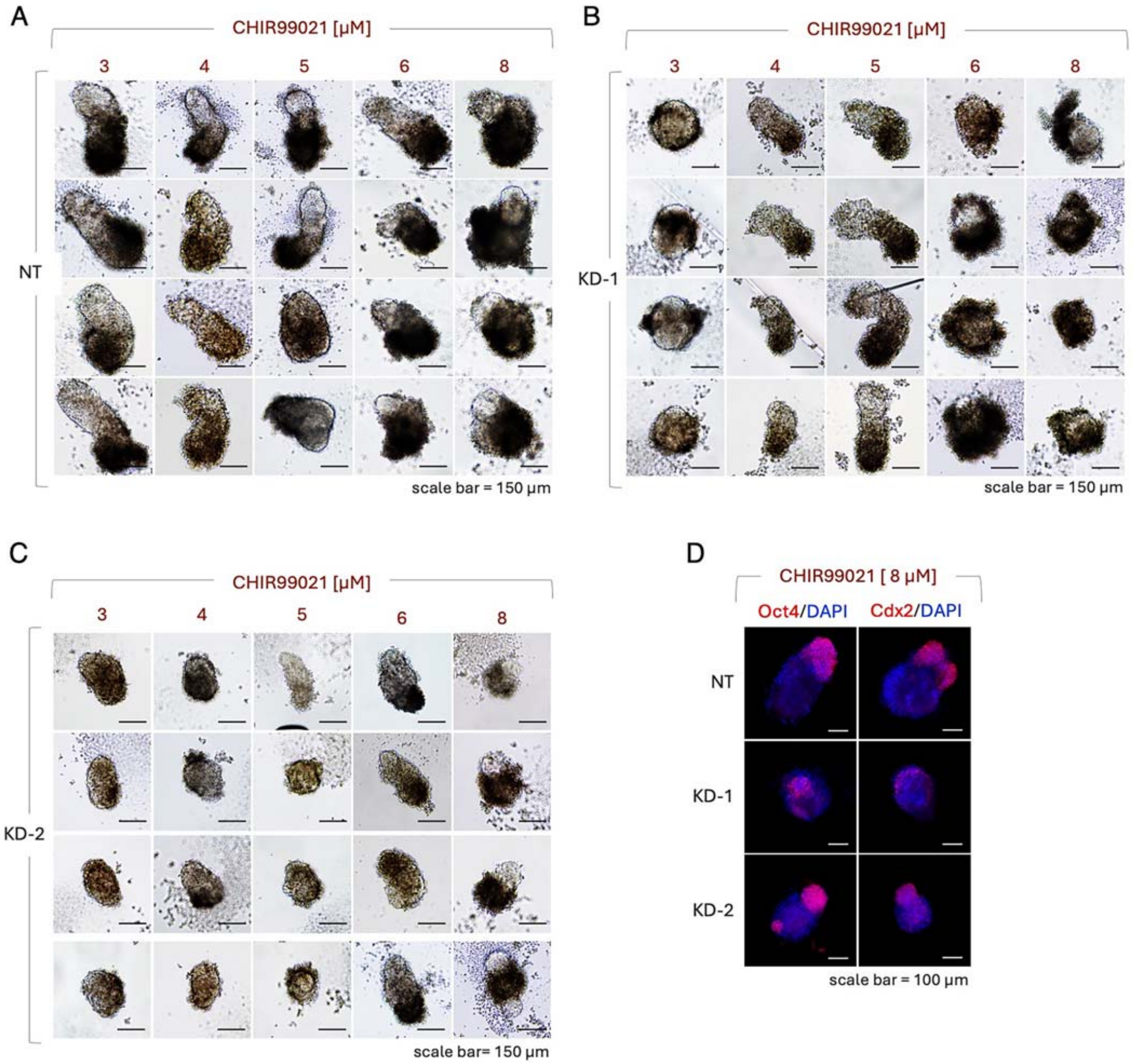

Figure EV3. Dose-dependent effect of CHIR99021 on gastruloid development.

(**A–C**) Representative brightfield images of Control (NT) (**A**), KD-1 (**B**), KD-2 (**C**) gastruloids treated with increasing concentration of CHIR99021 (3–8 µM). Scale bar, 150 µm. (**D**) Representative confocal images of Oct4 (pluripotency marker) and Cdx2 (differentiation marker) in gastruloids treated with 8 µM of CHIR99021. Nuclei were counterstained with DAPI (blue). Scale bar, 100 µm. All images are representative of 4 individual gastruloids.

© The Author(s)

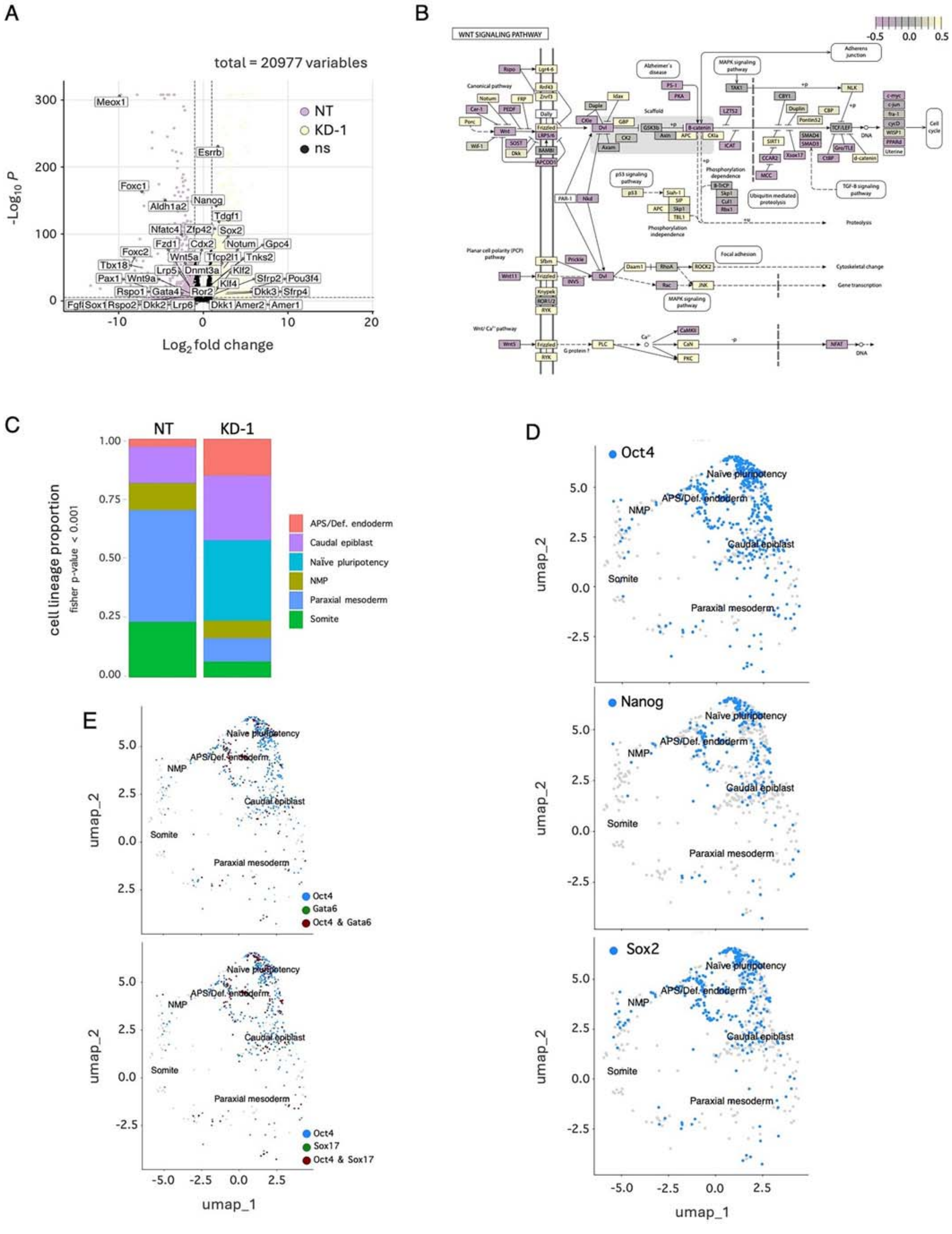
A
total = 20977 variables
NT
KD-1
ns
Meox1
Esrrb
Foxc1
Aldh1a2
Nanog
Tdgf1
Nfatc4
Zfp42
Sox2
Fzd1
Cdx2
Notum
Gpc4
Foxc2
Wnt5a
Tfcp2l1
Tnks2
Tbx18
Lrp5
Dnmt3a
Klf2
Pax1
Wnt9a
Sfrp2
Pou3f4
Klf4
Rspo1
Gata4
Ror2
Dkk3
Sfrp4
Fgfi
Sox1
Rspo2
Dkk2
Lrp6
Dkk1
Amer2
Amer1
$-Log_{10}$ P
Log_2 fold change
B
WNT SIGNALING PATHWAY
Canonical pathway
Planar cell polarity (PCP) pathway
Wnt/Ca2+ pathway
Cytoskeletal change
Gene transcription
Proteolysis
Cell cycle
C
NT
KD-1
cell lineage proportion
fisher p-value < 0.001
APS/Def. endoderm
Caudal epiblast
Naïve pluripotency
NMP
Paraxial mesoderm
Somite
D
Oct4
Nanog
Sox2
Naïve pluripotency
APS/Def. endoderm
NMP
Caudal epiblast
Somite
Paraxial mesoderm
umap_1
umap_2
E
Oct4
Gata6
Oct4 & Gata6
Sox17
Oct4 & Sox17

 © The Author(s)

Figure EV4. Diverse cellular composition in *T-UCstem1* KD gastruloids versus the Control.

(**A**) Volcano plot of differentially expressed genes between *T-UCstem1* KD-1 and NT gastruloids. Differential expression analysis was performed using DESeq2 (Wald test; $n = 3$/group). Genes with adjusted $p < 0.05$ and $|\log_2$ fold change$| > 1$ are color-coded. (**B**) Reference map of the WNT Signaling pathway from the KEGG pathway database; annotated features are coloured based on differential expression between KD and Control (NT). (**C**) Stacked barplots of the cell lineage proportion in *T-UCstem1* KD-1 and NT gastruloids. (**D**) UMAP plot highlighting expression of *Oct4*, *Nanog* and *Sox2* in *T-UCstem1* KD-1 gastruloids. (**E**) UMAP plot highlighting expression of *Oct4*, *Gata6* and *Sox17* in *T-UCstem1* KD-1 gastruloids.

© The Author(s)

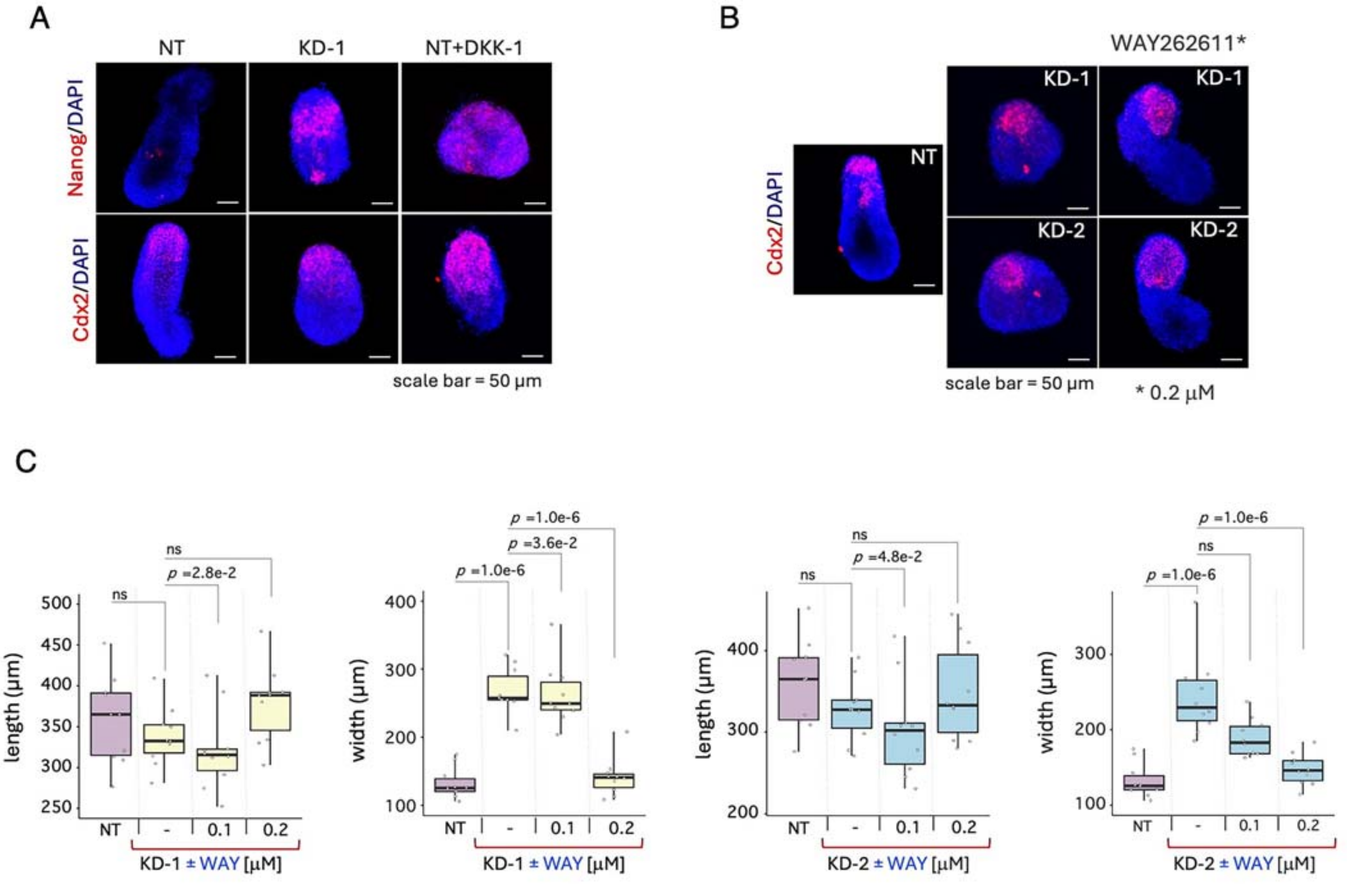

Figure EV5. DKK-1 blocks the correct gastruloid development.

(**A**) Representative confocal images of Nanog (pluripotency marker) and Cdx2 (differentiation marker) in gastruloids ± DKK-1 recombinant protein. Nuclei were counterstained with DAPI (blue). Scale bar, 100 µm. All images are representative of 4 individual gastruloids. (**B**) Representative confocal images of Cdx2 (differentiation marker) in gastruloids NT and *T-UCstem1* KD ± WAY262611 at 0.2 µM. Nuclei were counterstained with DAPI (blue). Scale bar, 50 µm. All images are representative of 4 individual gastruloids. (**C**) Boxplot diagrams of gastruloids length (left), width (right) ± WAY262611 at 120 h. Data are shown as mean ± SD ($n = 3$ independent experiments, 10 gastruloids/condition). Boxplots display the minimum, first quartile, median, third quartile, and maximum. Statistical significance was assessed by one-way ANOVA with Tukey's multiple comparison test. *P* values of ≤0.05 were considered statistically significant.

 © The Author(s)

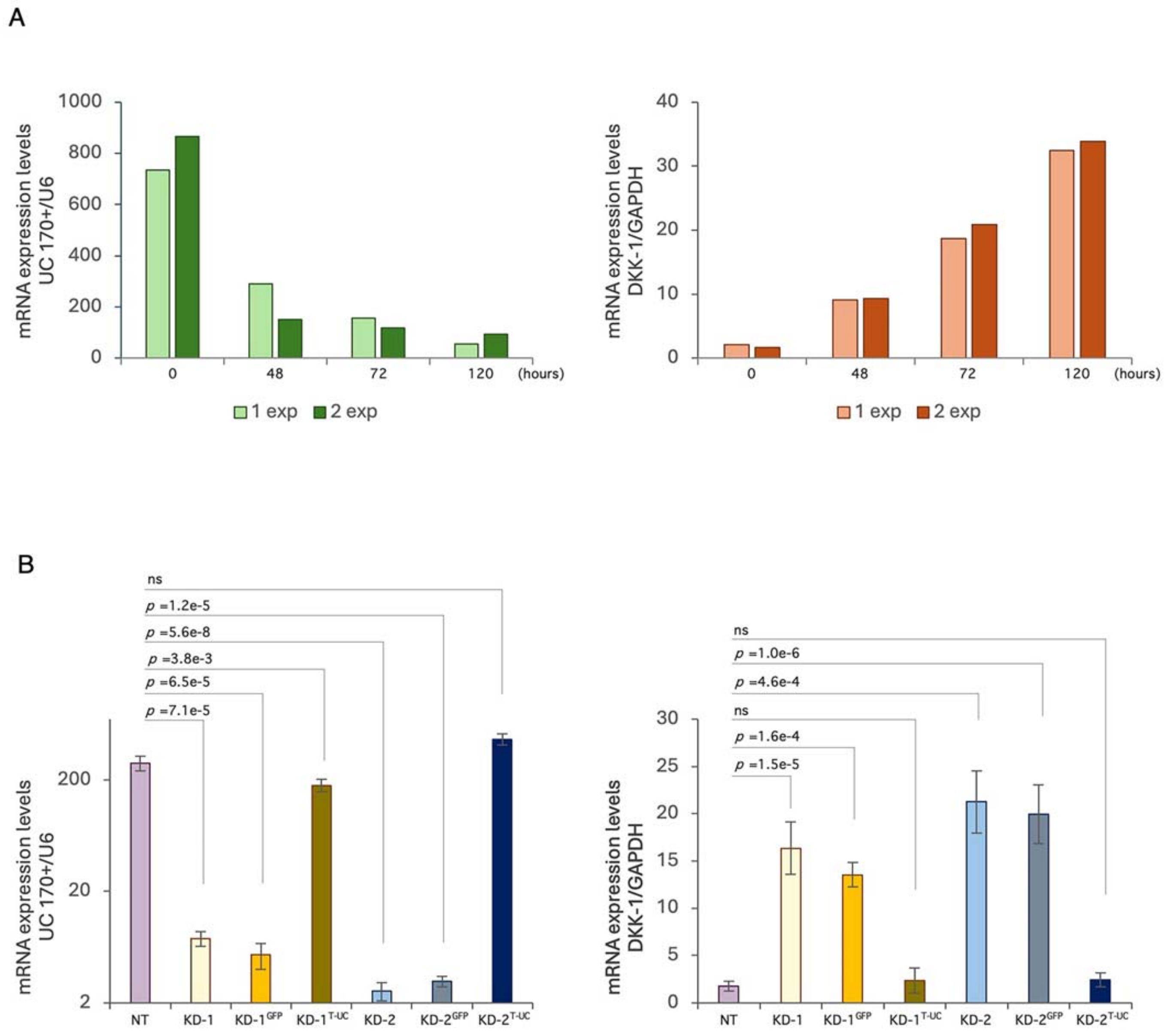

Figure EV6. Expression analysis of *T-UCstem1* and DKK-1 in mESCs and during gastruloid development.

(**A**) qRT–PCR analysis of *T-UCstem1* (left) and *DKK-1* (right) expression levels at different stages of gastruloid development in control (NT) conditions. *T-UCstem1* levels were normalized to *U6*, and *DKK-1* levels to *Gapdh*. Individual values of two biological replicates are shown (Exp 1 and 2). (**B**) qRT–PCR analysis of *T-UCstem1* (left) and *DKK-1* (right) expression levels in control (NT), *T-UCstem1* knockdown (KD), KD^{GFP}, and $KD^{T\text{-}UC}$ mESCs. *T-UCstem1* expression was normalized to *U6*, and *DKK-1* expression to *Gapdh* ($n = 2$ independent experiments). Data are shown as mean ± SD ($n = 3$ independent experiments). Statistical significance was assessed by one-way ANOVA with Tukey's multiple comparison test. *P* values of ≤0.05 were considered statistically significant. Source data are available online for this figure.

© The Author(s)
